# Genome sequencing of *Rhinorhipus* Lawrence exposes an early branch of the Coleoptera

**DOI:** 10.1186/s12983-018-0262-0

**Published:** 2018-05-02

**Authors:** Dominik Kusy, Michal Motyka, Carmelo Andujar, Matej Bocek, Michal Masek, Katerina Sklenarova, Filip Kokas, Milada Bocakova, Alfried P. Vogler, Ladislav Bocak

**Affiliations:** 1 Laboratory of Molecular Systematics, Department of Zoology, Faculty of Science, Palacky University, 17. listopadu 50, 771 46 Olomouc, Czech Republic; 2 Grupo de Ecología y Evolución en Islas, Instituto de Productos Naturales y Agrobiología (IPNA-CSIC), 38206 San Cristóbal de la Laguna, Spain; 3 CRH – Department of Molecular Biology, Faculty of Science, Palacky University, Šlechtitelů 241/27, 783 71 Olomouc-Holice, Czech Republic; 4 Department of Life Science, Natural History Museum, Cromwell Road, London, SW7 5BD UK; 5 Department of Life Science, Silwood Park Campus, Imperial College London Ascot, London, SL5 7BD UK

**Keywords:** Molecular phylogeny, Phylotranscriptomics, Elateriformia, Rhinorhipidae, Triassic, New superfamily

## Abstract

**Background:**

Rhinorhipidae Lawrence, 1988 is an enigmatic beetle family represented by a single species, *Rhinorhipus tamborinensis* Lawrence, 1988, from Australia, with poorly established affinities near the superfamily Elateroidea (click beetles, soldier beetles and fireflies) or the more inclusive series (infraorder) Elateriformia. Its evolutionary position may inform the basal relationships of the suborder Polyphaga, the largest clade of Coleoptera.

**Results:**

We analyzed four densely sampled DNA datasets of major coleopteran lineages for mitogenomes, rRNA genes and single copy nuclear genes. Additionally, genome sequencing was used for incorporation of *R. tamborinensis* into a set of 4220 orthologs for 24 terminals representing 12 polyphagan superfamilies. Topologies differed to various degrees, but all consistently refute the proposed placement of Rhinorhipidae in Elateroidea and instead indicate either sister relationships with other Elateriformia, frequently together with Nosodendridae, another divergent small family hitherto placed in Derodontoidea, or in an isolated position among the deepest lineages of Polyphaga. The phylogenomic analyses recovered *Rhinorhipus* in a sister position to all other Elateriformia composed of five superfamilies. Therefore, we erect the new superfamily Rhinorhipoidea Lawrence, 1988, **stat. Nov.**, with the type-family Rhinorhipidae. The origins of the Rhinorhipidae were dated to the Upper Triassic/Lower Jurassic at the very early phase of polyphagan diversification.

**Conclusions:**

Thus, Rhinorhipidae adds another example to several recently recognized ancient relict lineages which are interspersed within contemporaneous hugely species-rich lineages of Coleoptera.

**Electronic supplementary material:**

The online version of this article (10.1186/s12983-018-0262-0) contains supplementary material, which is available to authorized users.

## Background

**Fig. 1:**
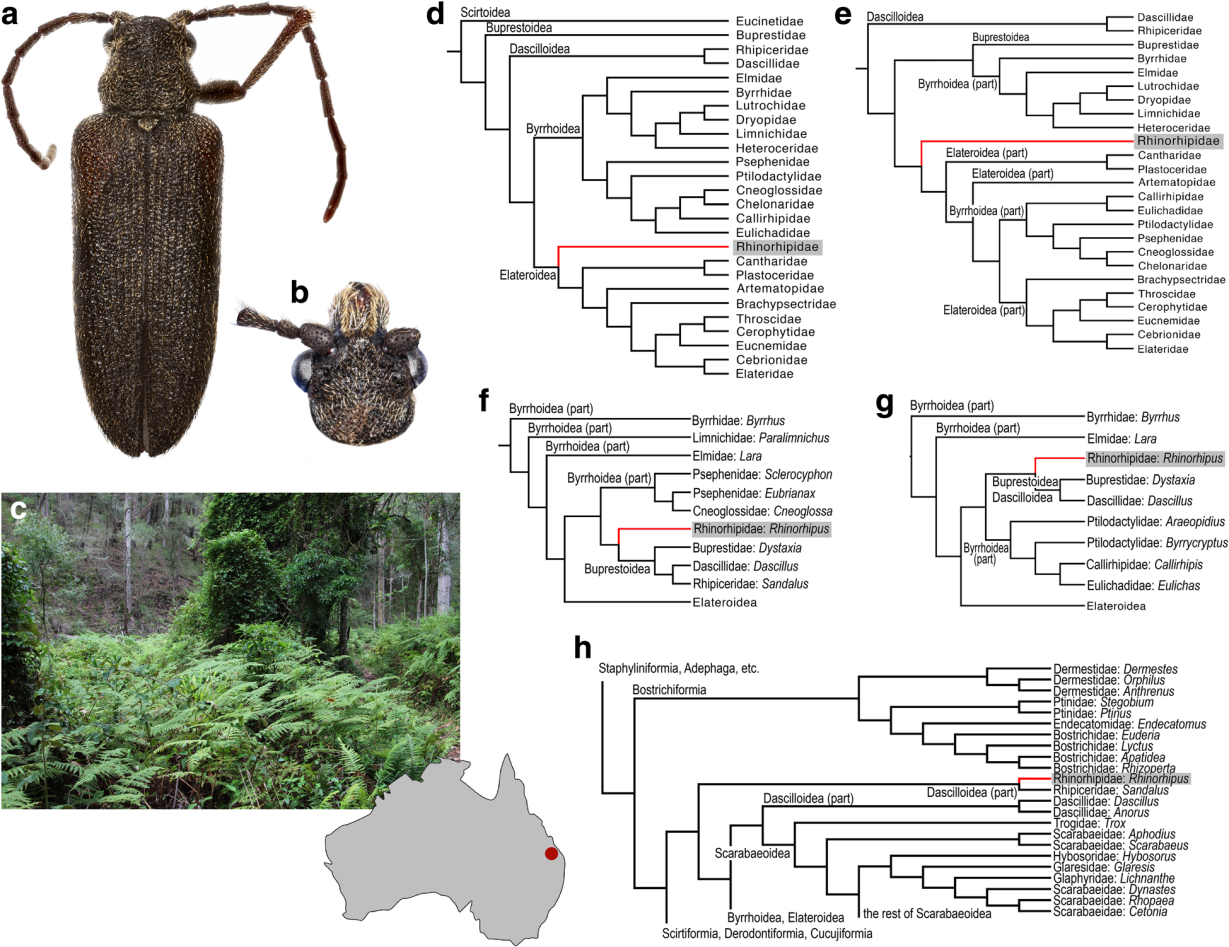
*Rhinorhipus tamborinensis* Lawrence: (**a**) – General appearance; (**b**) – Head; (**c**) – Collecting site. Morphology-based phylogenetic hypotheses: (**d)** – Lawrence (1988), all characters; (**e)** – ditto, adult characters; (**f**) – Lawrence et al. (1995), all characters; (**g**) – ditto, adult characters; (**h**) – Lawrence et al. (2011)

The Coleoptera are the epitome of high species diversity on Earth, but it has long been recognized that richness differs greatly among lineages, e.g. among the four suborders, which range in species numbers from about a combined 120 in Archostemata and Myxophaga, to well over 340,000 species in Polyphaga [1]. With improving molecular and paleontological data, these differences in clade size can be placed in an explicitly temporal context [2–4] and have already contributed to a better understanding of the evolution of Coleoptera. For example, several ‘small’ families (Scirtidae, Clambidae, Eucinetidae, Decliniidae and Derodontidae) previously linked to the series (infraorders) Elateriformia and Derodontoidea were found to be the sister groups to all other ‘core’ Polyphaga. These ‘ancestral five’ [2] families include the family Decliniidae created for a single species that was discovered only in the second half of the last century [5, 6]. Other recent discoveries also represent new families, such as the Iberobaeniidae, Meruidae and Aspidytidae [7–9] known to include only a single or a few closely related species within an isolated lineage. In other cases, species poor lineages such as Derodontidae, Nosodendridae and Jacobsoniidae are well known taxonomically, but there has been great uncertainty over their position that is only gradually resolved with molecular data [3, 4]. Equally, the phylogenetic placement is highly problematic for a single species, *Rhinorhipus tamborinensis* Lawrence, 1988 from Queensland, Australia, which has been discovered some 50 years ago and assigned to the monospecific family Rhinorhipidae [10]. *Rhinorhipus* exhibits various aberrant morphological characters producing ambiguous phylogenetic signal and widely different positions depending on the study, albeit always showing affinities to Elateriformia [6, 10] (Fig. 1a–h). In the most recent classifications *Rhinorhipus* is placed in Elateriformia *incertae sedis* [11] or in Elateroidea [3, 12–15], while an accurate placement is difficult given the uncertainty about the basal relationships of Elateriformia and its closest relatives generally.

Rhinorhipidae is among only eleven out of nearly 200 beetle families for which molecular data have been unavailable in previous studies [1–4]. *Rhinorhipus* has been found in several localities in Queensland in the 1970′s, even in high numbers in some places, but since then the species has not been seen again despite intensive search efforts (G. Monteith and H. Escalona, pers. comm.). Recently, our expedition to Queensland yielded a fresh specimen suitable for DNA isolation and integration with existing molecular phylogenetic analyses. Extensive DNA datasets have been produced in the last decade for the phylogenetic analysis of the Coleoptera [2–4, 16, 17] and Elateriformia [18–20], and we here incorporate *Rhinorhipus* sequences into rRNA, mitogenomic and nuclear protein coding genes (PCGs) datasets. Additionally, we obtained shotgun genome data of *Rhinorhipus tamborinensis* for a phylogenomic analysis using several thousand orthologs available for a growing number of coleopteran lineages [21–29].

The classification of polyphagan beetles employs a hierarchy of series (infraorders) and superfamilies, into which most families can be firmly placed [12–15]. The taxonomic limits of these higher-level groups are increasingly well defined by large-scale molecular studies [1–4]. However, the backbone of the phylogeny specifying the relationships among these lineages emerging in various morphological and molecular analyses still differs in substantive ways, while support is generally low and the presented trees in some cases were constrained to illustrate the most meaningful relationships [1–4, 13, 17]. Equally there is disagreement about the dating of the Coleoptera tree, due to uncertainty about the placement of age-calibrated fossils [2–4, 30]. Resolution of basal relationships in Coleoptera will be improved with the greater completeness of taxon sampling of deep lineages, but this is hampered if DNA-grade specimens are difficult to obtain, which particularly affects the inclusion of species-poor, rare relict lineages. The resulting poor taxon sampling may also be responsible for discrepancies with morphological studies that are less constrained by the availability of specimens [14]. Placement of these lineages is particularly important for studies of diversification which frequently are based on comparisons of species numbers between sister lineages or rate shifts within a tree [31, 32]. Therefore, the aim of this study is to determine the phylogenetic position and taxonomic status of the enigmatic *Rhinorhipus*, as part of the effort to establish the deep divergences of Coleoptera and an increasingly complete sampling of the major lineages constituting the earliest branches. Additionally, by dating the origin of this unique lineage relative to other lineages representing the early beetle evolution, we obtain a clearer picture of the arrangement of species rich and poor lineages making up the great diversity of Coleoptera.

## Results

### Phylogenetic relationships

**Fig. 2:**
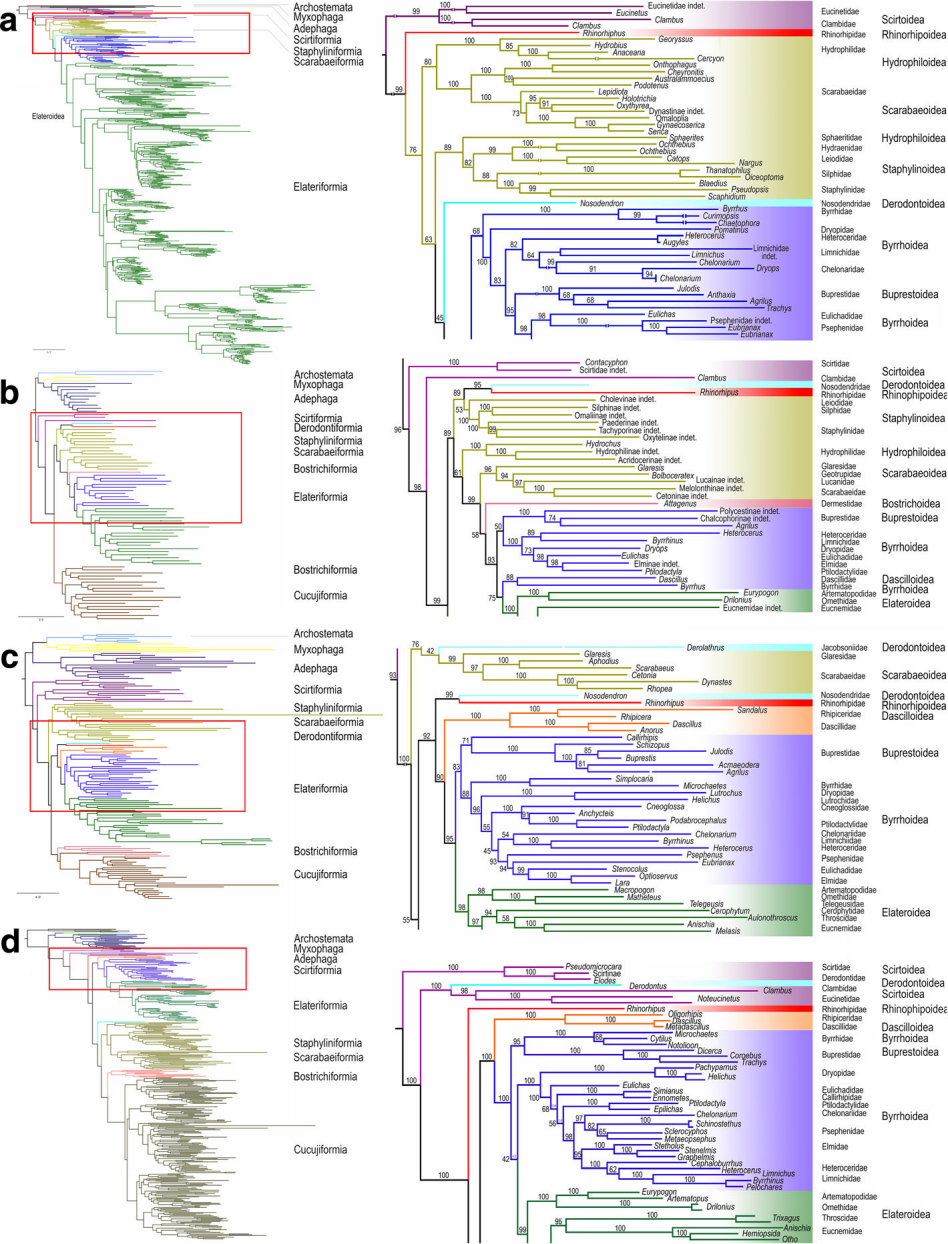
Portions of phylogenetic trees obtained from the four ML analyses of (**a**) the four-gene dataset (Elateriformia and selected outgroups only), (**b**) mitogenomes, (**c**) eight-gene dataset, and (**d**) the 66-gene dataset. The full trees are shown in Additional file 1: Figures S1-S4. The Bayesian tree recovered from the same dataset is shown in Additional file 1: Figure S4

The relationships of *Rhinorhipus* were first investigated using the extensive 4-gene 564-taxa rRNA and mtDNA dataset of Elateriformia. The ML analysis recovered *Rhinorhipus* as the next lineage after the origin of Scirtidae, Derodontidae and Clambidae, i.e., among the deepest splits of Polyphaga and external to Elateriformia (bootstrap values, BS 99%; Fig. 2a, Additional file 1: Figure S1). If *Rhinorhipus* belongs to Elateroidea, the closest relatives should have been identified by the analysis of such densely sampled dataset.

Further datasets covered all major beetle lineages to investigate the relationships of *Rhinorhipus* and the polyphagan series. Using the mitogenome 15-gene 83-taxa dataset of broad representation of Coleoptera, the ML analyses of deep relationships within Polyphaga recovered *Rhinorhipus* as a sister to *Nosodendron* (BS 95%) and both of them combined as a sister to the superfamily Staphylinoidea (BS 89%, Fig. 2b, Additional file 1: Figure S2) or to a grade of paraphyletic Staphyliniformia of the arrangement (Staphylinoidea (Hydrophiloidea (Scarabaeoidea (Elateriformia)))). The poor recovery of the Staphyliniformia is typical for mitogenome data [17]. The analysis was included to show that the mitogenomic phylogeny does not support the placement of Rhinorhipidae in Elateroidea.

Deep relationships within Polyphaga according to the eight-gene 139-taxa nuclear dataset [3] recovered *Rhinorhipus* as a sister to *Nosodendron* in the ML analysis (BS 99%), but here the clade was in the sister position to Elateriformia (BS 92%; Fig. 2c). The BI analysis recovered *Rhinorhipus* as a separate deeply branching lineage, as sister to Polyphaga minus Scirtiformia and Derodontiformia (i.e., the core Polyphaga) with high support (BS 100%), although the core Polyphaga clade was supported only with BS 53% (Additional file 1: Figure S4). *Nosodendron* was recovered as sister to Bostrichiformia + Cucujiformia (BS 53%, Additional file 1: Figure S4).

When included in the 66-gene 376-taxa dataset [4], both ML analyses of the amino acids (using iQ-TREE and RAxML) recovered *Rhinorhipus* as an independent deeply rooted lineage in the sister position to core Polyphaga (Fig. 2d, BS 100%) and *Nosodendron* was recovered as a sister to Bostrichiformia + Cucujiformia (Additional file 1: Figure S5), in agreement with the position obtained by Zhang et al. [4]. *Rhinorhipus* was recovered as a sister to *Nosodendron* in the ML analysis of the nucleotide dataset using RAxML (BS 100%) and they combined were the sister to Elateriformia (BS 94%; Additional file 1: Figure S7). High BS values were recovered for alternative relationships in the 66-gene analyses (Additional file 1: Figures S5–S7).

**Fig. 3:**
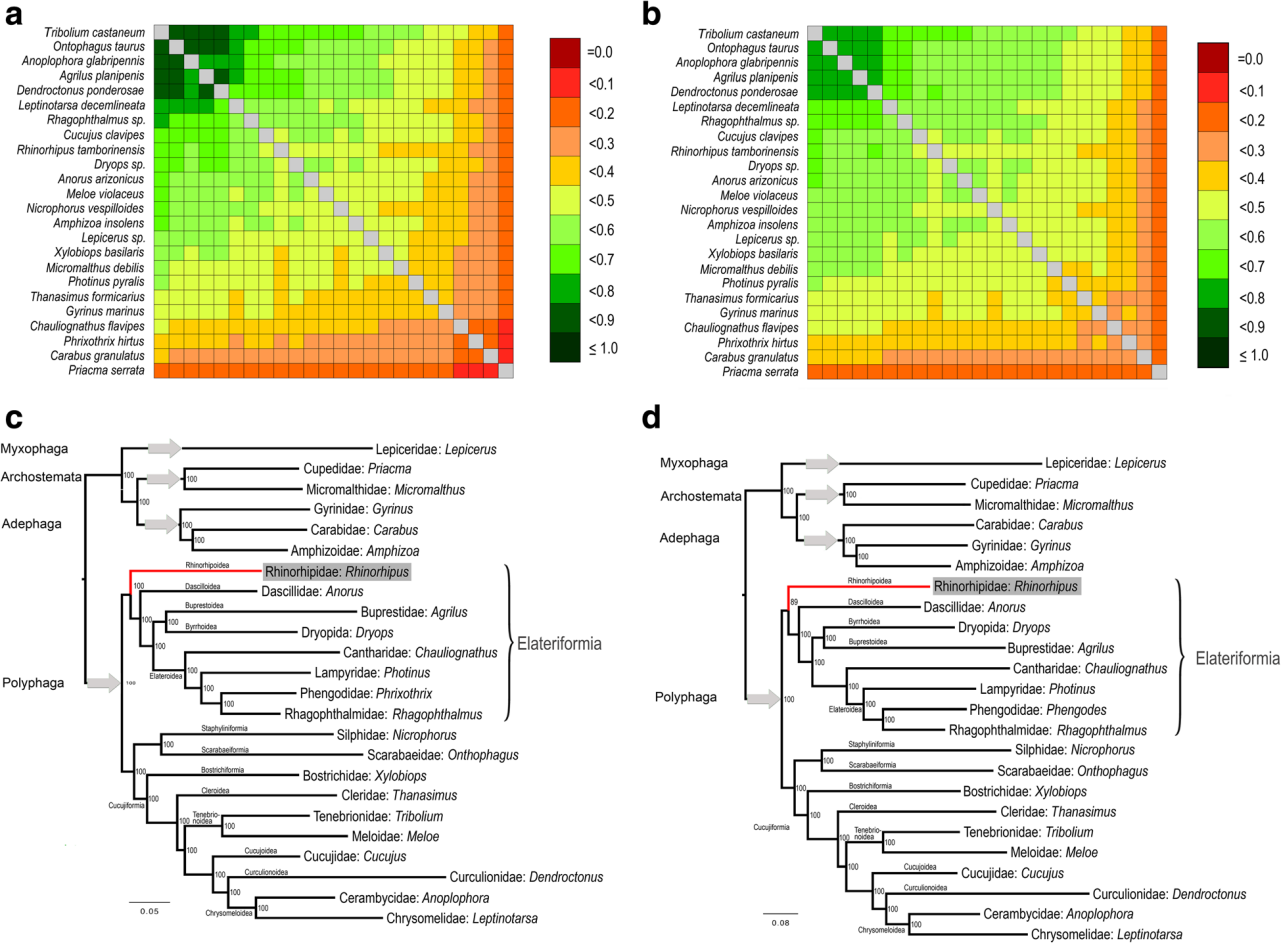
Comparison of completeness of assembled transcriptomes for the phylogenetic analysis: (**a**) – Amino-acid dataset, 4220 orthologs, (**b**) – nucleotide dataset, 4220 orthologs. Phylotranscriptomic trees: (**c**) – Maximum likelihood tree obtained from the analysis of the nucleotide dataset without the 3rd codon position (supermatrix 2 described in Methods), (**d**) – Maximum likelihood tree obtained from the analysis of the amino-acid dataset, without outliers, and alignment processed with Aliscore (supermatrix 3 in Methods)

**Fig. 4:**
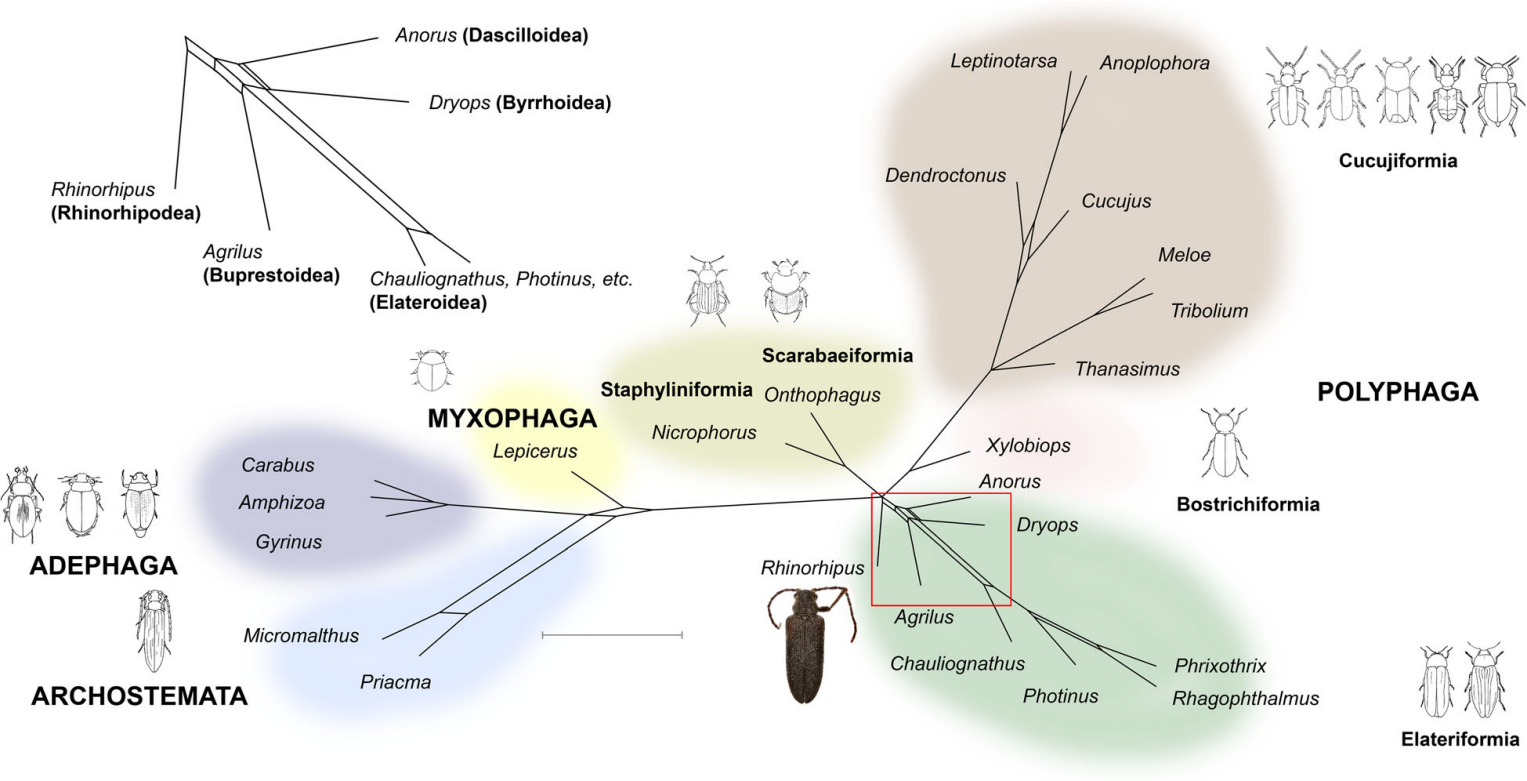
Network obtained from the separate maximum likelihood analyses of all orthologs

Newly generated shotgun genomic sequencing data provided high coverage of protein-coding regions at a sequencing depth of approximately 60×, which was used to create an ortholog set of 4220 genes from 23 publicly available transcriptome and genome data of Coleoptera. The ortholog representation for *Rhinorhipus* was among the most complete of the taxa included in the matrix, exceeding most of the transcriptome data and just short of the few fully assembled genome sequences, as visualized in Fig. 3a and b. The ML analyses of nucleotide and amino acid data produced very similar topologies (Fig. 3c and d), including supermatrix 1 that represented data without any alignment filtering, and supermatrix 4 that contained only 943 mostly highly conservative orthologs present in all taxa. *Rhinorhipus* was regularly recovered in a sister relationship with all other Elateriformia (BS 100%, 89% and 92% in the analyses of the supermatrices 2, 3 and 4, respectively, Fig. 3c and d). A network was constructed from 4203 trees which also showed the monophyly of *Rhinorhipus* + Elateriformia, although not unequivocally, as was evident from some net-like structure indicating a minority of contradicting topologies (Fig. 4).

### Dating the tree

**Fig. 5:**
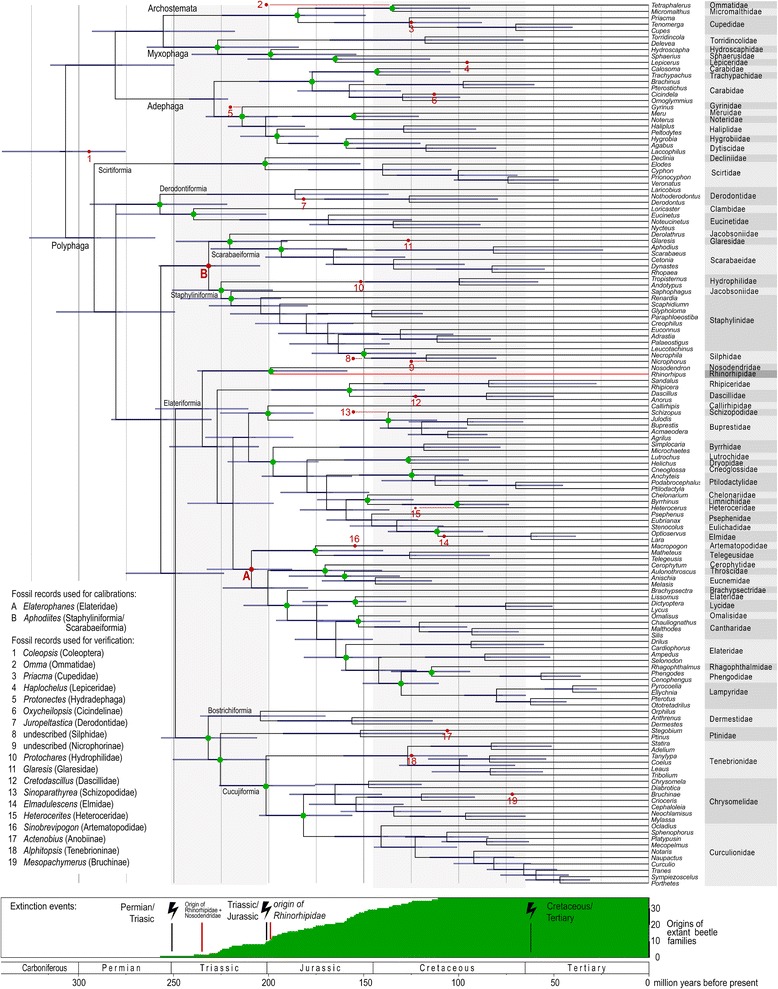
Dated phylogenetic tree of insect relationships inferred from the Bayesian analysis of eight-gene dataset using maximum likelihood constrained topology, two calibration points (**a**), (**b**) and verified by mapping of nineteen fossil records reported by Toussaint et al. (2016). The bottom diagram shows accumulation of the number of extant beetle families (red dots on the tree). Time line relates the tree to extinction events and geologic periods. Red bars designate the origins of Rhinorhipidae + Nosodendridae and/or Rhinorhipidae

The origin of *Rhinorhipus* was dated on the 8-gene dataset, using topologies obtained with the BI (Fig. 5) and ML (Additional file 1: Figure S11) topologies which reflected two alternative placements of *Rhinorhipus*, either as sister to Elateriformia (in conjunction with the Nosodendridae in this case) or as a sister to the core Polyphaga, respectively. Dating was conducted on these fixed topologies with selected fossil ages using BEAST (see Material and Methods). The analysis using the ML topology placed the origin of the *Rhinorhipus* + *Nosodendron* clade to 235.0 mya (95% CI = 210.9–259.7) and the split between these two families at 199.2 million years ago (mya; 95% CI = 158.7–237.6) (Additional file 1: Figure S11). The alternative analysis using the BI topology sets the origin of *Rhinorhipus* to the Lower Triassic to 243.0 mya (95% CI = 220.2–268.8; Additional file 1: Figure S5). The dating analyses with the mitochondrial dataset using ML and BI topologies resulted in an earlier origin of the deepest beetle lineages (Additional file 1: Figures S9 and S10), but in contrast with such deep estimates, the splits between *Rhinorhipus* + *Nosodendron* are inferred either younger (154 mya, 95% CI =121.0–201.0) or similar to the analyses using the 8-gene dataset (215.0 mya, 95% CI = 150.1–274.0).

## Discussion

The study draws on existing datasets that differ in taxon breadth and gene coverage. A summary of the position of *Rhinorhipus* in the various analyses is provided in Table 1. The most extensive taxon coverage for Elateriformia (Fig. 2a), to which *Rhinorhipus* is affiliated in the current classification, clearly demonstrated its position outside of this group, and neither did we find a close affinity to other infraorders based on the three datasets covering all Polyphaga. We regularly observe two alternative positions, either as sister to all core Polyphaga or as sister to Elateriformia (Table 1, Figs. 2, 3, 4 and 5, Additional file 1: Table S1–S11). These placements are complicated by the affinity with Nosodendridae, representing another orphan lineage of the Coleoptera, which was placed in equally deeply branching positions either as sister to Elateriformia (with *Rhinorhipus*) or as sister to Bostrichiformia + Cucujiformia (without *Rhinorhipus*). The presence of two highly morphologically and genetically divergent taxa in an otherwise densely sampled tree likely leads to long-branch attraction of such isolated taxa. Phylogenomic analyses of variously assembled transcriptomic datasets regularly recovered *Rhinorhipus* as sister to all currently defined elateriform superfamilies (Fig. 3c, d and Fig. 4), which represents the preferred hypothesis and which is broadly supported by all of the other datasets (Table 1). These genomic topologies had high support at every node regardless of applied filtering and coding, and apparently profited from the greater information content of a large gene set, but the analyses were limited in taxon sampling (Table 1, Fig. 3c, d and Fig. 4). *Rhinorhipus* (possibly together with *Nosodendron*) thus represents a morphologically and genetically highly disparate beetle lineage that pre-dates most of the large beetle lineages such as Staphyliniformia, Bostrichiformia and Cucujiformia. (Fig. 5, Additional file 1: Figures S9–S11). Thus, *Rhinorhipus* cannot be placed in Elateroidea [10, 15]. Given morphology-based affinities of *Rhinorhipus* with Elateriformia ([6, 10, 12] Additional file 1: Text), morphological divergence compared with Nosodendridae [10, 14] and the results of transcriptome analysis (Figs. 3 and 4), we retain Rhinorhipidae in Elateriformia and erect a monotypic superfamily Rhinorhipoidea Lawrence, 1988, **stat. Nov.** in this series (type-family monotypic Rhinorhipidae Lawrence, 1988).

**Table 1:** The overview of current phylogenetic analyses (PCG – nuclear protein coding genes; AA – amino acid dataset; nucl. – nucleotide dataset; ML – maximum likelihood; BI – Bayesian Inference; iQ – iQ-TREE; StaphF, ScarF, ElatF – the series Staphyliniformia, Scarabaeiformia, Elateriformia; core Polyphaga – all polyphagan series except Scirtiformia and Derodontidae)

Dataset	# of taxa	# of genes	Figs.	Analyses	Topology
rRNA, mtDNA	563	4	2A, S1	ML (RaxML)	*Rhinorhipus*(core Polyphaga)
mitogenomes	82	15	2B, S2	ML (RaxML)	(*Rhin.* + *Nosod.*)(StaphF(ScarF(ElatF)))
mitogenomes	82	15	S9	BI (PhyloBayes)	((*Rhin.* + *Nosod.*)StaphF)(ScarF(ElatF))
rRNA+PCG nucl.	139	8	2C, S3	ML (iQ)	(*Rhinorhipus* + *Nosodendron*)(ElatF)
rRNA+PCG nucl.	139	8	S4	BI (PhyloBayes)	*Rhinorhipus*(core Polyphaga)
PCG AA	372	66	2D, S5	ML (RAxML)	*Rhinorhipus*(core Polyphaga)
PCG AA	372	66	S6	ML (iQ)	*Rhinorhipus*(core Polyphaga)
PCG nucl.	372	66	S7	ML (RAxML)	*Rhinorhipus* + *Nosodendron*(ElatF)
PCG AA (#1)	24	4220	–	ML (iQ, no filtering)	*Rhinorhipus*(Elateriformia)
PCG nucl. (#2)	24	4220	3C	ML (iQ,1st + 2nd)	*Rhinorhipus*(Elateriformia)
PCG AA (#3)	24	4220	3D	ML (iQ, Aliscore)	*Rhinorhipus*(Elateriformia)
PCG AA (#4)	24	943	–	ML (iQ, Aliscore)	*Rhinorhipus*(Elateriformia)

The relationships with *Nosodendron* needs further data to be robustly supported. *Nosodendron* was unavailable for transcriptomic analysis and only some analyses indicate its sister relationships with *Rhinorhipus* (Fig. 2b, c but not Fig. 2a–d). Nosodendridae was placed in Bostrichiformia [11] or Derodontiformia [14] and its relationships to Elateriformia has never been inferred from morphology. Even if further analyses support the relationships of these taxa, their morphological disparity and inferred ancient origins fully support the superfamily rank for each of these unique lineages [14, 15]. Lawrence [10] listed the following characters of *Rhinorhipus* which falsify the relationships with Bostrichoidea including *Nosodendron*: procoxae conical with fully exposed but immovable trochantins; prosternal process fitting into cavity on mesosternum; metendosternite with well developed ventro-lateral processes; wing with elongate radial cell and serricorn folding type; first three ventrites connate; Malphigian tubules free (cited from [10]). The relationships of *Rhinorhipus* and *Sandalus* as a representative of Dascilloidea which are another ancient lineage of Elateriformia (Fig. 3c, d and Fig. 4) is supported by: the relatively long occipital region, distinctly raised antennal sockets, strongly and abruptly declined fronto-clypeal region without a sharp carina, long mandible, strongly projecting mesocoxae, setose metatrochantin, long anterior process of metendosternite, well developed empodium with three or more setae. However, many of these characters occur commonly in numerous distantly related beetle lineages and cannot be considered as synapomorphies of *Sandalus* and *Rhinorhipus*.

The morphological distinctiveness of *Rhinorhipus*, respectively Rhinorhipoidea, is demonstrated by the combination of the following characters: the head is hypognathous, with long temporal regions, without transverse occipital ridge or epicranial suture; the cranium has a short, median occipital endocarina and raised antennal insertions; the fronto-clypeal region is strongly declined and does not have a fronto-clypeal suture; the clypeus is long and narrow; the corporotentorium is very broad; the oral cavity is blocked by hairs; labrum is membranous, highly reduced, mandibles are long and have a setose dorsal cavity at the base; maxillae are highly reduced, membranous, and setose. The prothorax has a pronotum without lateral carinae, is anteriorly constricted and has a pair of elongate, vertical cavities; procoxae are a slender and conical, trochantins are completely visible, the promeso-thoracic interlocking mechanism is weakly developed. The mesothorax has a moderately developed mesosternal cavity reaching to the middle of the sternum and a pair of well developed procoxal housings on the mesepisterna; metasternum has only a moderately short median suture. The metendosternite has very long, curved lateral arms, an anterior process with a foramen at its base and a pair of expanded, ear-like, ventro-lateral processes. Each elytron has 12 more or less complete rows of deep punctures. The legs have the enlarged and mesally produced metatrochanters, apically expanded hind tibiae, the simple tarsal segments, without any pads, brushes, or membranous lobes, the well developed empodium with two or three setae, and the pectinate tarsal claws. *Rhinorhipus* has six Malphigian tubes. The detailed information on morphological characters supporting further contradicting morphology-based relationships is given in the Additional file 1: Text.

**Fig. 6:**
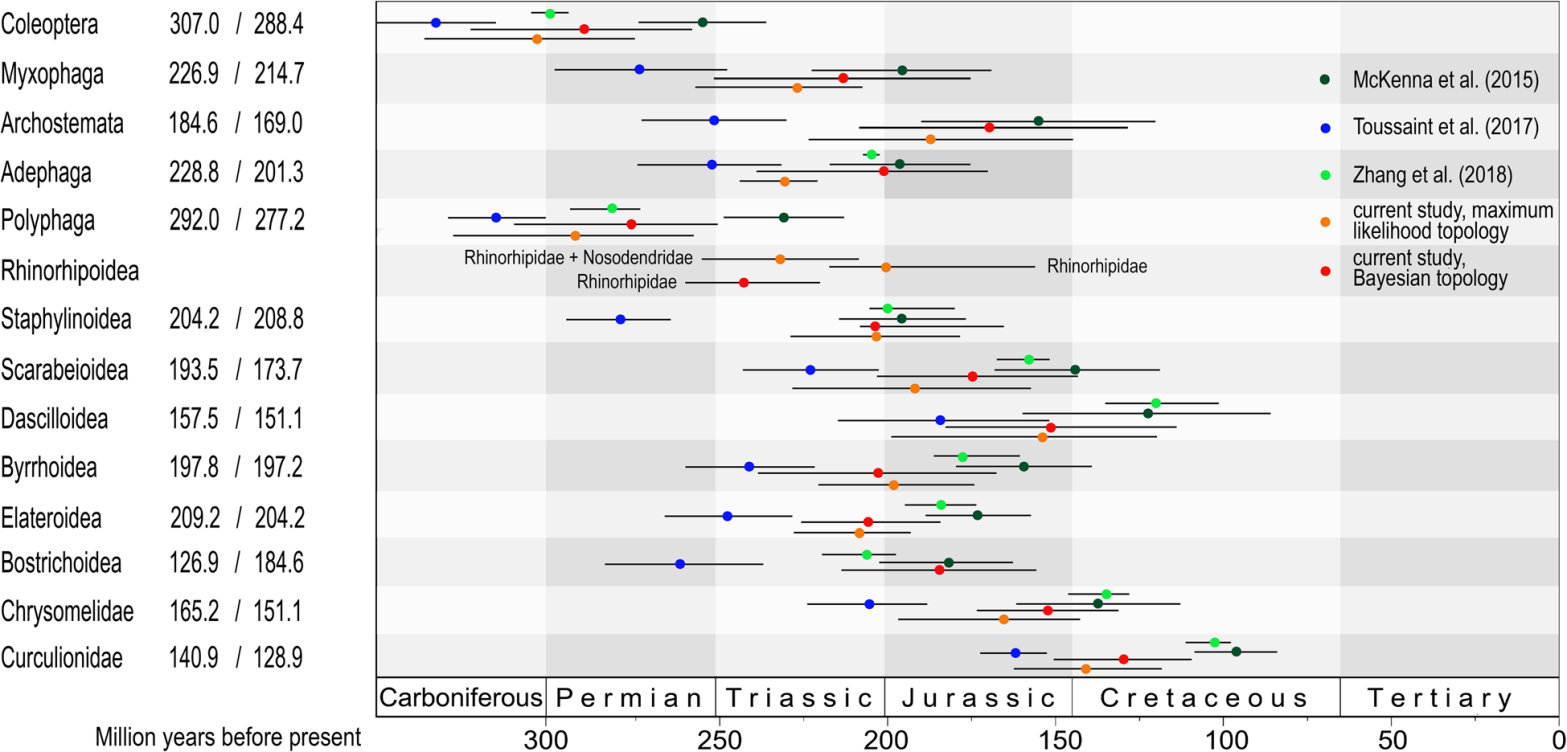
The comparison of the recovered crown ages for selected beetle lineages

We consider the age estimates of the Rhinorhipidae reliable and consistent with previous dating studies of beetles (Fig. 6), even if the exact phylogenetic position remains to be confirmed. Several age estimates of the Coleoptera have been presented recently, and the current analysis arrives at intermediate values mostly in line with the latest estimate of Zhang et al. [4], which is also in good agreement with Hunt et al.’s [2] estimate from three markers. Our age estimates are supported by fossil studies across the wider arthropods, e.g. the crown Coleoptera + Strepsiptera fossil *Adiphlebia* dated at 306.9 Mya, which then becomes the maximum (stem) age of Coleoptera [33], and also fits well with the beetle fossil record [30, 34, 35]. We verified the major branching events by mapping 19 fossils used as calibration points by Touissant et al. [30] (Fig. 5, Additional file 1: Figure S11) and found that fossil ages were in almost all cases within or younger than the 95% CI age intervals obtained for the representative nodes (Fig. 5, except *Omma*). Our estimations used the same eight-gene dataset of McKenna et al. [3] that produced the youngest estimate, but used differences in calibration points (see Methods). In addition, the principal differences are that we only used a subset of terminals from the original studies and did not mask alignment variable regions, which added some 3000 positions compared to the filtered dataset [3, 30]. We also used slightly different algorithms and did not implement any constraints to the tree searches that were implemented in McKenna et al. [3], and thus obtained slightly different topologies (Additional file 1: Figures S3 and S4). A robust estimate of deepest diversification events in the Coleoptera remains elusive due to the uncertainty in identity and age of some fossils [3, 30], conflicting topologies (Fig. 2, Additional file 1: Figures S1–S11) [3, 4] and poorly understood effects of filtering of datasets applied in some studies. Nevertheless, most studies find the origins of the oldest extant, dominantly non-phytophagous beetle families in the latest Permian to Triassic, with further diversification after the Triassic/Jurassic extinction event (Fig. 5, Additional file 1: S11). Rhinorhipidae belongs among these oldest families along with Scirtidae, Eucinetidae, Clambidae, Decliniidae, Derodontidae, and Nosodendridae and the ancestors of the hyperdiverse clades such as Staphyliniformia, Scarabaeiformia, and Cucujiformia.

Multi-gene rRNA and mtDNA phylogenies have become dominant in Coleoptera phylogenetics in the last two decades [e.g., 1, 2, 19, 20], and only the recent datasets of McKenna et al. [3] and Zhang et al. [4] analyzed multiple protein coding nuclear markers. All of these datasets, including the most recent of nearly 100 genes, produced topologies sensitive to the data treatment and choice of phylogenetic algorithm, which affected especially the placement of ancient lineages represented by species poor orphan lineages such as *Nosodendron* and *Rhinorhipus* (Table 1, Figs. 2 and 5, Additional file 1: Figures S1–S11) [1–4]. These data also run the risk of using non-homologous regions; e.g. at least a quarter of all genes used by Zhang et al. [4] which originally consisted of 95 loci were affected by apparent paralogy as judged against the existing reference genomes of Coleoptera (see Additional file 1: Table S5) for excluded loci, and even the loci in McKenna et al. [3] are affected by this problem, which required to make choices about which locus to select as an ortholog. In contrast, the use of shotgun Illumina sequencing produced several thousand one-to-one orthologs that can be recovered consistently from all available Coleoptera genomes and transcriptomes. Shotgun sequencing produced a nearly complete set of these orthologs for *Rhinorhipus* at an approximately 60× coverage. Bioinformatics pipelines [36, 37] that assemble these short reads against reference sequences can readily create thousands of gene sequences from genomic DNA, without the need for isolating mRNA, which is usually more difficult to obtain. Thus, greater taxon sampling is possible with relatively little effort and we can expect a stabilization of the remaining critical relationships among basal branches of the Coleoptera once more data area available.

The monotypic *Rhinorhipus* now identified as sister lineage to Elateriformia, or possibly even to the core Polyphaga, represents another example of a species-poor relict beetle lineage, such as Jurodidae and Crowsoniellidae in Archostemata, Aspidytidae and Meruidae in Adephaga, and Decliniidae in Polyphaga. It is perplexing why these lineages persist among others that are extremely species rich. Given its origin at the time where the continents were interconnected, and the habitat and presumed life style of *Rhinorhipus*, there is no obvious ecological or morphological trait that diminishes their chance of dispersal, diversification and species survival. Nevertheless, *Rhinorhipus* has a limited distributional range in Australia which harbors many relict lineages and is apparently rare in nature.

With the long-standing mystery about the great variation in species richness of Coleoptera in mind [38], these lineages are important for establishing the basal relationships and reconstruction of the dominant ecological role of earliest lineages in the Late Permian and Triassic ecosystems. Already, the realization of the ‘ancestral five’ in the earliest full tree of Coleoptera and the recognition of the core Polyphaga [2] had a major impact on the understanding of beetle relationships and dating. Placing the remaining small groups, including the enigmatic Rhinorhipidae, will be of equal importance for accurate evolutionary relationships and ecological characterization of the oldest beetle lineages. The growing phylogenetic evidence repeatedly indicates the presence of a much higher number of independent ancient beetle lineages than expected just two decades ago [2–4]. These families frequently live in soil, where they depend on organic moist detritus and the presence of molds and fungi and importantly, all these lineages apparently missed opportunities exploited by other lineages, such as the diversification of the angiosperms. They survived harsh conditions following the Permian extinction event [39], but were not able to diversify at the same pace as competing lineages in the changing world of the late Jurassic and Cretaceous. Thus, Rhinorhipidae stands out as a unique Australian witness of the early phase of beetle diversification in the early Mesozoic when the first extant beetles families evolved.

## Methods

### Material, DNA extraction, and sequencing

A single adult specimens of *R. tamborinensis* was collected in the Main Range National Park, Goomburra Section (27°59.0’S 152°21.4′E) on Nov. 28., 2014. DNA was extracted using the DNeasy kit (Qiagene Inc.). The dsDNA concentration was measured using a Qubit 2.0 Fluorometer (Life Technologies Corp., Carlsbad, CA). The voucher specimen was deposited in the collection of Department of Zoology, Palacky University, Olomouc.

The *SSU* and *LSU* rRNA and *cox1* and *rrnL* mtDNA fragments were obtained using the Sanger method and procedures reported earlier [18] (GenBank accession numbers for rRNA fragments AB123456–78). The complete mitochondrial genome was sequenced using the mitochondrial metagenomics approach [40, 41]. The extracted raw DNA was pooled in equimolar concentration with DNA from 20 other Coleoptera from distant lineages not connected to the current study. A TruSeq DNA library was constructed with the pooled DNA and sequenced using the Illumina MiSeq platform (Illumina Inc., San Diego, CA) (2 × 300 bp paired-end sequencing; TruSeq library with 800–950 bp insert size) in 45% of an Illumina flow cell. The Illumina output was processed and assembled in three independent assemblers, followed by super-assembly and circularization in Geneious 7.1.9 (Biomatters Ltd., Auckland, New Zealand) as described in [42]. The mitogenome of *R. tamborinensis* was identified by a Blast match to a *cox1* sequence obtained from the same specimen by PCR-Sanger sequencing. The mitochondrial genome was annotated using gene predictions with MITOS [43] and manually refined in Geneious (GenBank Accession Number KT825140).

Total genomic DNA of *R. tamborinensis* was shotgun sequenced with Illumina X Ten platform (Illumina Inc., San Diego, CA) for 2 × 150 bp paired-end reads. The sequencing service was provided by Novogene Co., Ltd. (Beijing, China). Raw paired-end reads were filtered using the Novogene pipeline. The filtering steps included the removal of read pairs if either one read contains adapter contamination; if more than 10% of bases are uncertain in either one read; or if the proportion of low quality bases is over 50% in either one read. The quality of reads was visualized with FastQC. The genomic data were deposited in GenBank (Accession Number PRJNA448980).

### Construction of data matrices

Various sequences for *R. tamborinensis* were incorporated in existing datasets. In all cases, protein coding genes (PCGs) were individually aligned using the “Translation Align” option with the FFT-NS-i-× 2 algorithm of MAFFT 7.2 [44], and ribosomal genes *rrnS* and *rrnL* were aligned using the Q-INS-I algorithm in MAFFT 7.2 [45]. The GBlocks masking method for alignment variable regions was not applied, unlike in some of the original data sets. The following matrices were produced:

(1) A four-gene dataset of Elateriformia composed of nuclear complete *SSU* and partial *LSU* rRNA and mitochondrial *cox1* and *rrnL* genes was assembled from earlier published data [9, 18–20] (Additional file 1: Table S1). The dataset contained 564 terminals and 4966 homologous positions.

(2) Mitochondrial genomes were retrieved from GenBank to represent all principal beetle lineages (Additional file 1: Table S2). The 15 mitochondrial genes were extracted using Geneious 8.0.5. Individual gene alignments were trimmed and concatenated for a final dataset of 82 taxa (including *R. tamborinensis)* and 15 genes with 12,940 homologous positions.

(3) An eight-gene nuclear dataset (*SSU* rRNA, *LSU* rRNA, wingless, alpha-spectrin; arginine kinase, phosphoenolpyruvate carboxykinase, carbamoyl-phosphate synthase domain, elongation factor-1α) was assembled for 139 taxa selected from a set of 367 taxa reported by McKenna et al. [3]. The homologous reads for *R. tamborinensis* were extracted from shotgun reads using the HybPiper 1.2 pipeline [36] and mapped to reference genes in Geneious 7.1.9. The dataset contained 52 taxa from the series Elateriformia and extensive representation of all other polyphagan series and beetle suborders (Additional file 1: Table S3). Intron regions were manually removed and the exons were concatenated for each gene alignment. All sequences were trimmed to contain only complete codons triplets and alignments were concatenated using FASconCAT-G 1.02 (https://www.zfmk.de/en/research/research-centres-and-groups/fasconcat) to produce a super-matrix consisting of 10,999 aligned positions. The partition scheme and selected models are reported in Additional file 1: Table S4.

(4) A 66-gene 376-taxa matrix was assembled from data of Zhang et al. [4] (Additional file 1: Table S4). The homologous genes for *R. tamborinensis* were extracted from shotgun genome sequencing as described above. The final dataset excluded 28 genes of Zhang et al. [4] which were found to contain up to 21 copies in at least some beetle genomes and thus could not homologized confidently (Additional file 1: Table S5). Exons were extracted and concatenated as described above to produce a supermatrix of 56,340 aligned positions.

### Genomic dataset

Transcriptomes (available on Oct. 6, 2017, Additional file 1: Table S4) were downloaded from the NCBI Transcriptome Shotgun Assembly (TSA) database. Additionally, transcripts of *Rhagophthalmus* sp., *Chauliognathus flavipes* and *Phrixothrix hirtus* were downloaded from the SRA archive and assembled using SOAPdenovo-Trans-31mer 1.04 [46]. Lantern and body transcripts of *P. hirtus* were merged into a single dataset (Additional file 1: Table S4) [47]. Raw data were first examined in FastQC (http://www.bioinformatics.babraham.ac.uk/projects/fastqc) to screen them for irregularities. Removal of low-quality reads and TruSeq adaptor sequences (Illumina Inc., San Diego, CA) was performed with Trimmomatic-0.36 [48].

The ortholog set was obtained by searching the OrthoDB 9.1 database [49] for one-to-one orthologs among Coleoptera in available genome sequences of *Agrilus planipennis*, *Anoplophora glabripennis*, *Dendroctonus ponderosae*, *Leptinotarsa decemlineata*, *Onthophagus taurus*, and *Tribolium castaneum*. OrthoDB 9.1 specified 4225 protein coding single copy genes for the above species and the Coleoptera reference node. We used Orthograph 0.6.1 [50] to search the above transcriptomes for the corresponding sequences. Default settings were used except for extend-orf = 1 and orf-overlap-minimum = 0.5. We searched whole genome data of *Rhinorhipus* with HybPiper 1.2 pipeline [36] for each of the target orthologs using blastx of the Blast 2.6.0+ software package [51] with --evalue 1e-5 to search. Reads were then separated into directories by gene and assembled with SPAdes 3.10.1 [52]. The resulting contigs were processed with Exonerate 2.2.0 [53] with the protein2genome model using sequences from reference species with the best cumulative blast score to recover the exon sequences. We then merged the acquired exons of *R. tamborinensis* at both amino acid and nucleotide level with the corresponding ortholog groups. We removed terminal stop codons and masked internal stop codons at the translational level and nucleotide levels using the perl script summarize_orthograph_results.pl [50].

Alignments from each orthology group were checked for the presence of outlier sequences using BLOSUM62 distance scores [54] and following the methods reported by Misof et al. [55]. We used Pal2Nal [56] to generate multiple sequence alignments of nucleotides corresponding to amino acids and Aliscore 2.0 [57, 58] to identify random similarity within alignments which were removed using Alicut 2.3 (https://github.com/mptrsen/scripts/blob/master/ALICUT_V2.3.pl).

The matrix of all 4220 orthologs and 9,994,362 homologous positions was assembled and derived datasets were used for tree construction. Supermatrix 1 – at amino acid level without any alignment masking (2,308,506 positions); Supermatrix 2 - at nucleotide level, 1st + 2nd codon positions only, outliers filtered out (6,636,362 positions); Supermatrix 3 – at amino acid level after masking with Aliscore (2,100,404 positions); and Supermatrix 4 – using a subset of Supermatrix 3 containing only ortholog alignments with representation of all taxa and after the alignment masking with Aliscore (943 orthologs, 378,949 positions). AliStat 1.3 (https://github.com/thomaskf/AliStat) was used to generate distributions of missing data in the supermatrices.

### Phylogenetic analyses

IQ-TREE 1.5.5 [59] and RaxML [60] were used to calculate maximum likelihood (ML) trees using the IQ-TREE web server [61], with partitions identified by the ModelFinder tool of IQ-TREE using the Bayesian Information Criterion [62, 63]. The partitions, models and parameters are listed in Additional file 1: Tables S10–S14. The ultrafast bootstrap option was used with 1000 bootstrap iterations [64]. The iQ analyses were run with the -spp parameter allowing each partition to have its own evolutionary rate. Bayesian inference (BI) was conducted with PhyloBayes [65] on the CIPRES web portal [66] using two independent chains under a GTRCAT model. Analyses were run checking for convergence every 1800 s excluding the first 500 cycles, and the runs were stopped when the *maxdiff* value was lower than 0.1. A consensus tree was obtained discarding the first 10,000 cycles as a burn-in fraction and taking 1 tree every 10 cycles for the remaining (*bpcomp* options *-× 10,000 10 –c 0*).

Gene tree incongruence was tested for Supermatrices 3 (all gene trees) and 4 (gene trees with representation of all taxa) by visualizations of the dominant bipartitions among individual loci based on the individual IQ-TREE ML gene topologies by constructing supernetworks using the SuperQ method implemented in Spectre selecting the ‘balanced’ edge-weight with ‘JOptimizer’ optimization function, and applying no filter [67, 68]. This methodology decomposes all gene trees into quartets to build supernetworks where edge lengths correspond to quartet frequencies. Resulting supernetworks were visualized in SplitsTree 4.14.6 [69].

### Bayesian dating analyses

Dating analyses were calibrated using fossils relevant to the origin of Dytiscoidea and Geadephaga, Elateriformia and Scarabaeiformia [30, 70, 71]. The earliest fossils of Elateroidea date back to the Hettangian and Sinemurian deposits (190.8–201 mya; Elateridae, *Elaterophanes*; [72]); the fossil of Scarabaeiformia to the Jurassic Formation of Switzerland (196.5–201.6 mya; *Aphodiites* [73]; and the fossil of Dytiscoidea to the Hassberge formation in Germany (221.5 mya; *Protonectes germanicus* [74]. We applied a truncated normal distribution allowing a soft tail to the past as recommended for fossil calibration [75] with a minimum age hard bound at 190.8 mya and a 95% range of 192.4–239.8 mya (mean = 190.8 mya stdev = 25.0 mya) as a prior for the node representing the split of Elateroidea from other Elateriformia; a minimum age hard bound at 196 mya and a 95% range of 197.6–245 mya (mean = 196 mya stdev = 25.0 mya) for the split between Scarabaeoidea and its sister taxon; and a minimum age hard bound at 221.5 mya and a 95% range of 223.1–270.5 mya (mean = 221.5 mya stdev = 25.0 mya) for the origin of Adephaga.

Divergence times at nodes were estimated with BEAST 1.8.4 [76] on the fixed topologies from ML and BI analyses, applying the best-fit substitution model and partition scheme as estimated in PartitionFinder 2 [77]. For the molecular clock settings, the dataset was partitioned in 3 partitions (PCGs, *rrnL* and *rrnS* for mitochondrial genomes; rRNA genes, 1st + 2nd and 3rd codon positions of PCGs for the 8-gene dataset) applying an uncorrelated lognormal clock to each partition and a Yule speciation prior. Analyses were run twice in parallel with 50 million generations sampling one tree every 5000 generations. Consensus trees were estimated with TREEANNOTATOR [76] combining both runs and discarding the 50% initial trees as burn-in after checking the ESS of the tree likelihood and ensuring that values had reached a plateau in TRACER 1.6 (http://beast.bio.ed.ac.uk/Tracer). The inferred diversification events were verified against the set of 19 fossils listed by Toussaint et al. [30]. No prior was set for the root age.

## Additional file

Additional file 1: Text the morphology-based classifications of Rhinorhipidae. **Table S1.** The list of taxa included in the *LSU* rRNA, *SSU* rRNA, *rrnL,* and *cox1* mitochondrial DNA dataset with GenBank accession and voucher ID numbers. **Table S2.** The list of taxa included in the mitogenomic analysis with GenBank accession numbers. **Table S3.** The list of taxa included in the *LSU* rRNA, *SSU* rRNA, and six nuclear protein coding genes. **Table S4.** The list of taxa included in the 65-gene dataset. **Table S5.** The list of markers in the 95-gene dataset with information on multi-copy genes. **Table S6.** The list of taxa included in the phylotranscriptomic dataset and the number of sequences available for each taxon. **Table S7.** Overview of official gene sets of six reference species used for transcript ortholog assessment, including the source, version and number of genes. **Table S8.** Gene descriptions for the 4220 ortholog groups (OGs) as present in. OrthoDB 9.1. Each OG contains one gene of each of the 6 reference species. **Table S9.** Success of transcript assignment to ortholog groups (OGs) of *Rhinorhipus,* published beetles transcriptomes and genomes. **Table S10.** The models and partition selections recovered with ModelFinder for the maximum likelihood analysis of the *LSU* rRNA, *SSU* rRNA, *rrnL* mtDNA, and *cox1* mtDNA dataset. **Table S11.** Identification of the best partition scheme and models for the mitochondrial DNA dataset. **Table S12.** The *LSU* rRNA, *SSU* rRNA, and six nuclear protein coding genes dataset: characteristics, partition scheme and models of DNA evolution. **Table S13.** The transcriptomic supermatrix 3: partition scheme and models of DNA evolution (amino acid dataset, 4220 orthologs). **Table S14.** The transcriptomic supermatrix 4: partition scheme and models of DNA evolution (amino acid dataset, 943 orthologs). **Figure S1.** Maximum likelihood tree for *Rhinorhipus*, 517 Elateriformia and 46 outgroups recovered from the *LSU* rRNA, *SSU* rRNA, *rrnL* mtDNA and *cox1* mtDNA dataset. **Figure S2.** Maximum likelihood tree for 83 species of beetles recovered from 15 mitochondrial genes**. Figure S3.** Maximum likelihood tree for 139 species of beetles recovered from the. *LSU* rRNA, *SSU* rRNA and six nuclear protein coding genes. **Figure S4.** Bayesian tree for 139 species of beetles recovered from the LSU rRNA, *SSU* rRNA and six nuclear protein coding genes. **Figure S5.** Maximum likelihood (RaxML) tree for 372 species of beetles and for outgroups recovered from the 66-gene amino acid dataset. **Figure S6.** Maximum likelihood (iQ) tree for 372 species of beetles and for outgroups recovered from the 66-gene amino acid dataset. **Figure S7.** Maximum likelihood (iQ) tree for 372 species of beetles and for outgroups recovered from the 66-gene nucleotide dataset. **Figure S8.** Tree network obtained from the separate maximum likelihood analyses of 968 orthologs 590. **Figure S9.** Dated phylogenetic tree of beetle relationships inferred from the Bayesian analysis of mitogenomic dataset using maximum likelihood topology. **Figure S10.** Dated phylogenetic tree of beetle relationships inferred from the Bayesian analysis of mitogenomic dataset using Bayesian topology. **Figure S11.** Dated phylogenetic tree of beetle relationships inferred from the Bayesian analysis of eight-gene dataset using constrained Bayesian topology and two calibration points (A, B) and verified by mapping of nineteen fossil records reported by Toussaint et al. (2016). The bottom diagram shows accumulation of the number of extant beetle families (red dots on the tree). Time line relates the tree to extinction events and geologic periods. Red bars designate the origin of Rhinorhipidae. (PDF 30160 kb)
