# Supplementary material for: Genome sequencing of Rhinorhipus Lawrence exposes an early branch of the Coleoptera
Source: Front Zool. 2018 May 2;15:21. doi: 10.1186/s12983-018-0262-0 (PMC5930637; doi:10.1186/s12983-018-0262-0)
Supplement: Supplementary file 1 — Text the morphology-based classifications of Rhinorhipidae. Table S1. The list of taxa included in the LSU rRNA, SSU rRNA, rrnL, and cox1 mitochondrial DNA dataset with GenBank accession and voucher ID numbers. Table S2. The list of taxa included in the mitogenomic analysis with GenBank accession numbers. Table S3. The list of taxa included in the LSU rRNA, SSU rRNA, and six nuclear protein coding genes. Table S4. The list of taxa included in the 65-gene dataset. Table S5. The list of markers in the 95-gene dataset with information on multi-copy genes. Table S6. The list of taxa included in the phylotranscriptomic dataset and the number of sequences available for each taxon. Table S7. Overview of official gene sets of six reference species used for transcript ortholog assessment, including the source, version and number of genes. Table S8. Gene descriptions for the 4220 ortholog groups (OGs) as present in. OrthoDB 9.1. Each OG contains one gene of each of the 6 reference species. Table S9. Success of transcript assignment to ortholog groups (OGs) of Rhinorhipus, published beetles transcriptomes and genomes. Table S10. The models and partition selections recovered with ModelFinder for the maximum likelihood analysis of the LSU rRNA, SSU rRNA, rrnL mtDNA, and cox1 mtDNA dataset. Table S11. Identification of the best partition scheme and models for the mitochondrial DNA dataset. Table S12. The LSU rRNA, SSU rRNA, and six nuclear protein coding genes dataset: characteristics, partition scheme and models of DNA evolution. Table S13. The transcriptomic supermatrix 3: partition scheme and models of DNA evolution (amino acid dataset, 4220 orthologs). Table S14. The transcriptomic supermatrix 4: partition scheme and models of DNA evolution (amino acid dataset, 943 orthologs). Figure S1. Maximum likelihood tree for Rhinorhipus, 517 Elateriformia and 46 outgroups recovered from the LSU rRNA, SSU rRNA, rrnL mtDNA and cox1 mtDNA dataset. Figure S2. Maximum likelihood tree for 83 [file 12983_2018_262_MOESM1_ESM.pdf]

Genome sequencing of *Rhinorhipus* exposes an early branch of the Coleoptera

Dominik Kusy, Michal Motyka, Carmelo Andujar, Matej Bocek, Michal Masek, Katerina Sklenarova, Filip Kokas, Milada Bocakova, Alfried P. Vogler, Ladislav Bocak

The list of Supplementary Material

Supplementary Text. The morphology-based classifications of Rhinorhipidae 3
Supplementary Table S1. The list of taxa included in the *LSU* rRNA, *SSU* rRNA, *rrnL,* and *cox1* mitochondrial DNA dataset with GenBank accession and voucher ID numbers 7
Supplementary Table S2. The list of taxa included in the mitogenomic analysis with GenBank accession numbers 23
Supplementary Table S3. The list of taxa included in the *LSU* rRNA, *SSU* rRNA, and six nuclear protein coding genes 25
Supplementary Table S4. The list of taxa included in the 65-gene dataset....................................... 30
Supplementary Table S5. The list of markers in the 95-gene dataset with information on multi-copy genes 74
Supplementary Table S6. The list of taxa included in the phylotranscriptomic dataset and the number of sequences available for each taxon 77
Supplementary Table S7. Overview of official gene sets of six reference species used for transcript ortholog assessment, including the source, version and number of genes 79
Supplementary Table S8. Gene descriptions for the 4220 ortholog groups (OGs) as present in OrthoDB 9.1. Each OG contains one gene of each of the 6 reference species 81
Supplementary Table S9. Success of transcript assignment to ortholog groups (OGs) of *Rhinorhipus*, published beetles transcriptomes and genomes 462
Supplementary Table S10. The models and partition selections recovered with ModelFinder for the maximum likelihood analysis of the *LSU* rRNA, *SSU* rRNA, *rrnL* mtDNA, and *cox1* mtDNA dataset 463
Supplementary Table S11. Identification of the best partition scheme and models for the mitochondrial DNA dataset 464
Supplementary Table S12. The *LSU* rRNA, *SSU* rRNA, and six nuclear protein coding genes dataset: characteristics, partition scheme and models of DNA evolution 471
Supplementary Table S13. The transcriptomic supermatrix 3: partition scheme and models of DNA evolution (amino acid dataset, 4220 orthologs) 472
Supplementary Table S14. The transcriptomic supermatrix 4: partition scheme and models of DNA evolution (amino acid dataset, 943 orthologs) 559
Supplementary Figure S1. Maximum likelihood tree for *Rhinorhipus*, 517 Elateriformia and 46 outgroups recovered from the *LSU* rRNA, *SSU* rRNA, *rrnL* mtDNA and *cox1* mtDNA dataset 579
Supplementary Figure S2. Maximum likelihood tree for 83 species of beetles recovered from 15 mitochondrial genes 583
Supplementary Figure S3. Maximum likelihood tree for 139 species of beetles recovered from the *LSU* rRNA, *SSU* rRNA and six nuclear protein coding genes 584
Supplementary Figure S4. Bayesian tree for 139 species of beetles recovered from the LSU rRNA, *SSU* rRNA and six nuclear protein coding genes 585
Supplementary Figure S5. Maximum likelihood (RaxML) tree for 372 species of beetles and for

outgroups recovered from the 66-gene amino acid dataset 586
Supplementary Figure S6. Maximum likelihood (iQ) tree for 372 species of beetles and for outgroups recovered from the 66-gene amino acid dataset 588
Supplementary Figure S7. Maximum likelihood (iQ) tree for 372 species of beetles and for outgroups recovered from the 66-gene nucleotide dataset 590
Supplementary Figure S8. Tree network obtained from the separate maximum likelihood analyses of 968 orthologs 590
Supplementary Figure S9. Dated phylogenetic tree of beetle relationships inferred from the Bayesian analysis of mitogenomic dataset using maximum likelihood topology 593
Supplementary Figure S10. Dated phylogenetic tree of beetle relationships inferred from the Bayesian analysis of mitogenomic dataset using Bayesian topology 594
Supplementary Figure S11. Dated phylogenetic tree of beetle relationships inferred from the Bayesian analysis of eight-gene dataset using constrained Bayesian topology and two calibration points (A, B) and verified by mapping of nineteen fossil records reported by Toussaint *et al.* (2016). The bottom diagram shows accumulation of the number of extant beetle families (red dots on the tree). Time line relates the tree to extinction events and geologic periods. Red bars designate the origin of Rhinorhipidae 595

Supplementary Text.

The morphology-based classifications of Rhinorhipidae

Relationships of Rhinorhipoidea and earlier defined superfamilies of Coleoptera

The Rhinorhipidae is probably the only beetle family for which have been proposed numerous contradicting superfamily-level positions in a series of short-living highly contradicting morphological analyses based exclusively on the adult morphology. The first morphology-based analysis by Lawrence (1988) suggested the position of Rhinorhipidae as a sister of Elateroidea, but no synapomorphy was identified to support these relationships and *Rhinorhipus* and Elateroidea differs in many characters: e.g., *Rhinorhipus* has six and Elateroidea only four Malphigian tubes (Lawrence 1988). Plastoceridae and Cantharoidea were identified as close relatives (Fig 1D) with similarly dubious support, but the definition of shared morphological characters is currently impossible as Cantharoidea has already been accepted as a polyphyletic assemblage (Bocakova *et al.* 2007, Hunt *et al.* 2007, Timmermans *et al*. 2010, 2016, Kundrata *et al.* 2014, Bocak *et al.* 2014, McKenna *et al.* 2015, Zhang *et al.* 2018) and *Plastocerus* has recently been transferred to Elateridae when morphological characters indicating its relationships to soft-bodied elateroids were identified as homoplastic (Bocak *et al.* 2018). Mostly the absence of derived characters placed *Rhinorhipus* at the base of the Elateroidea (Lawrence 1988). Similarly, a few variable or plesiomorphic features supported the relationships of *Rhinorhipus* and some byrrhoid families in alternative analyses by Lawrence (1988) (Fig 1E). Due to the uncertain morphological phylogenetic signal, absence of the derived characters and an absent support for alternative topologies, Lawrence (1988) placed *Rhinorhipus* at the base of Elateroidea. The subsequent analysis of the slightly modified dataset by Lawrence *et al*. (1995) found Rhinorhipidae in a distant position from Elateroidea, mostly as a part of a clade comprising Rhipiceridae, Dascillidae and Buprestidae (Figs. 1F; Lawrence et al. 1995). The sister clade of this assemblage was either formed by Psephenidae + Cneoglossidae or by Ptilodactylidae + Callirhipidae + Eulichadidae. Neither this position obtained strong support by clear morphological synapomorphies and generally, the analysis did not support the monophyly of most superfamilies, which have been in the classification of Elateriformia (Lawrence & Newton 1995, Lawrence *et al*. 2011, Bouchard *et al*. 2011, Leschen *et al*. 2010, Beutel & Leschen 2016, etc.).

Relationships of Rhinorhipidae and a part of Dascilloidea, namely Rhipiceridae, was suggested by the latest morphology-based analysis by Lawrence *et al*. (2011). The clade consisting of *Rhinorhipus* and *Sandalus* (Rhipiceridae, Dascilloidea part) was recovered as a sister to the clade ((Dascilloidea part+ Scarabaeiformia), (Byrrhoidea + Buprestoidea + Elateroidea)). The relationships of *Rhinorhipus* with *Sandalus* was supported by a relatively long occipital region, distinctly raised antennal sockets, strongly and abruptly declined fronto-clypeal region without a sharp carina, long mandibles, strongly projecting mesocoxae, setose metatrochantin, long anterior process of metendosternite, and well developed empodium with three setae. Many of these characters, e.g. antennal sockets, the shape of the fronto-clypeal region, long mandibles, long anterior processes of the metendosternite, commonly occur in numerous distantly related beetle lineages. The

topology with paraphyletic Staphyliniformia and close relationships of Dascilloidea, Scarabaeoidea and Elateriformia is highly unlikely.

Lawrence et al. (1988) identified also a number of characters in which *Rhinorhipus* differs from Byrrhoidea, Buprestoidea and Elateroidea: the procoxae are not or slightly projecting below prosternum, paired procoxal rests on anterior edge of mesoventrite are moderately to strongly inclined, the apex of mesoventral process is undivided and broadly rounded, the apex of wedge cell in the hind wing is obliquely truncate. To sum up, all morphological analyses did not provide a clear phylogenetic signal to recover relationships of this morphologically unique beetles lineage.

The morphological relationships of *Nosodendron* and *Rhinorhipus* has never been proposed. Lawrence (1988) listed the following characters which exclude possible relationships of *Rhinorhipus* and Bostrichoidea including *Nosodendron*: (1) procoxae conical with fully exposed but immovable trochantins; (2) prosternal process fitting into cavity on mesosternum; (3) metendosternite with well developed ventro-lateral processes; (4) wing with elongate radial cell and serricorn (rather than bostrichiform) folding type; (5) first three ventrites connate; (6) aedeagus with parameres articulating with phallobase and not joined basally to penis (somewhat similar in Nosodendridae); (7) Malphigian tubules free (the characters are cited from Lawrence 1988).

The sometimes suggested morphology-based placement of Nosodendridae in Derodontoidea opens the question if *Rhinorhipus* and *Derodontus* might be related. Based on multiple analyses of various molecular datasets, the relationships seem highly improbable. All molecular phylogenies recovered Derodontidae among the earliest splits of Polyphaga along with Scirtidae, Decliniidae, Clambidae, and Eucinetidae (Hunt *et al.* 2007, Bocakova *et al*. 2007, Timmermans et al. 2010, 2016, Kundrata *et al*. 2014, Bocak *et al*. 2014, McKenna *et al*. 2015, Zhang *et al*. 2018). Additionally, Lawrence (1988) listed distinguishing characters as follows: Derodontidae: (1) antennae serrate; (2) procoxal cavities widely open internally and externally; (3) protrochantin exposed and adpressed to sternum; (4) prosternal process fitting into cavity on mesosternum; (5) transverse metasternal suture absent; (6) metendosternite with well developed ventrolateral processes; (7) hindwing with distinct radial cell and serricorn folding type; (8) first three ventrites connate (citation from Lawrence 1988). The comparison of Derodontoidea as whole in the sense of Lawrence *et al.* (2011) and *Rhinorhipus* is complicated by the fact that Derodontoidea were not identified as a monophylum by any recent molecular study (Hunt et al. (2007), Bocak et al. (2014), McKenna et al. (2015), Zhang et al. (2011) and the current analysis. Nosodendridae was recovered in various position either close to Elateriformia or close to the clade Bostrichiformia + Cucujiformia. Jacobsoniidae, when present in the analysis, have never been found in relationships with any bostrichiform family. The complicated comparison of morphology of Rhinorhipidae with the morphology of numerous beetle clades is beyond the scope of the study focused on the whole genome sequencing.

Morphological uniqueness of Rhinorhipoidea

Rhinorhipus, respectively Rhinorhipoidea, is a morphologically very distinct lineage and was defined by Lawrence (1988) with a number of morphological characters.

The head is hypognathous, with ventrally directed mouthparts, long temporal

regions, without transverse occipital ridge or epicranial suture; the cranium has a short, median occipital endocarina and raised antennal insertions; the fronto-clypeal region is strongly declined and does not have a fronto-clypeal suture; the clypeus is long and narrow; antennae filiform with sensory elements beginning on segments 4–11; the corporotentorium is very broad. The oral cavity is blocked by hairs. Labrum is membranous, highly reduced, mostly concealed beneath the clypeus, mandibles are long, slender, only slightly curved, without a mola and prostheca and have a setose dorsal cavity at the base; maxillae are highly reduced, membranous, and setose.

The prothorax of has a relatively large pronotum, without lateral carinae, with an anterior constriction a pair of elongate, vertical cavities; procoxae are a slender and conical with an exposed articular region trochantins are completely visible, the promeso-thoracic interlocking mechanism is only weakly developed. The mesothorax has a moderately developed mesosternal cavity reaching to the middle of the sternum and a pair of well developed procoxal housings on the mesepisterna; mesocoxal cavities are relatively narrowly open; metasternum has a moderately short median suture, the transverse suture is absent. The metendosternite has very long, curved lateral arms, an anterior process with a foramen at its base and a pair of expanded, ear-like, ventro-lateral processes. Each elytron has 12 more or less complete rows of deep punctures, a suture is deflected near the apex, the epipleura are represented by a thickening only.

The legs have the enlarged and mesally produced metatrochanters, apically expanded hind tibiae, the simple tarsal segments, without any pads, brushes, or membranous lobes, the well developed empodium with two or three setae, and the pectinate tarsal claws.

References

Beutel RG, Leschen RAB. Coleoptera, Beetles; Volume 1: Morphology and Systematics (Archostemata, Adephaga, Myxophaga, Polyphaga partim). 2nd ed. In: Kristensen NP, Beutel RG, editors. Handbook of Zoology, Arthropoda: Insecta. Berlin and Boston: Walter de Gruyter GmbH & Co. KG; 2016.

Bocak L, Barton C, Crampton-Platt A, Chesters D, Ahrens D, Vogler AP. Building the Coleoptera tree-of-life for >8000 species: composition of public DNA data and fit with Linnaean classification. Syst Entomol. 2014;39:97–110; doi:10.1111/syen.12037.

Bocak L, Motyka M, Bocek M, Bocakova M. Incomplete metamorphosis: the phylogenetic classification *Plastocerus* (Coleoptera: Elateridae). PLoS One. 2018;13(3):e0194026; doi:10.1371/journal.pone.0194026

Bocakova M, Bocak L, Hunt T, Teravainen M, Vogler AP. Molecular phylogenetics of Elateriformia (Coleoptera): evolution of bioluminescence and neoteny. Cladistics. 2007;23:477–496; doi:10.1111/j.1096-0031.2007.00164.x.

Bouchard P, Bousquet Y, Davies AE, Alonso-Zarazaga MA, Lawrence JF, Lyal CHC, et al. Family-group names in Coleoptera (Insecta). ZooKeys. 2011;88:1–972; doi:10.3897/zookeys.88.807.

Hunt T, Bergsten J, Levkanicova Z, Papadopoulou A, John OS, Wild R, et al. A comprehensive phylogeny of beetles reveals the evolutionary origins of a superradiation. Science. 2007;318:1913–1916.

Kundrata R, Bocakova M, Bocak L. The comprehensive phylogeny of the superfamily Elateroidea (Coleoptera: Elateriformia). Mol Phylogenet Evol. 2014;76:162–171; doi:10.1016/j.ympev.2014.03.012.

Lawrence JF. Rhinorhipidae, a new beetle family from Australia, with comments on the phylogeny of the Elateriformia. Invertebr Taxon. 1988;2:1–53.

Lawrence JF, Newton AF. Families and subfamilies of Coleoptera (with selected genera, notes, references and data on family-group names). In: Pakaluk J, Ślipiński SA, editors. Biology, Phylogeny, and Classification of Coleoptera: Papers Celebrating the 80th Birthday of Roy A. Crowson. Warszawa: Muzeum i Instytut Zoologii PAN; 1995. p. 779–1006.

Lawrence JF, Nikitsky NB, Kirejtshuk AB. Phylogenetic position of Decliniidae (Coleoptera: Scirtoidea) and comments on the classification of Elateriformia (sensu lato). In Pakaluk J, Ślipiński SA, editors. Biology, Phylogeny, and Classification of Coleoptera: Papers Celebrating the 80th Birthday of Roy A. Crowson. Warszawa: Muzeum i Instytut Zoologii PAN; 1995. p. 373–410.

Lawrence JF, Ślipiński SA, Seago AE, Thayer MK, Newton AF, Marvaldi AE. Phylogeny of the Coleoptera based on morphological characters of adults and larvae. Annal Zool. 2011;61:1–217; doi:10.3161/000345411X576725.
McKenna DD, Wild AL, Kanda K, Bellamy CL, Beutel RG, Caterino MS, et al. The beetle tree of life reveals that Coleoptera survived end-Permian mass extinction to diversify during the Cretaceous terrestrial revolution. Syst Entomol. 2015;40:835–880; doi:10.1111/syen.12132.

Leschen RAB, Beutel RG, Lawrence JF. Handbook of Zoology, Arthropoda: Insecta. Coleoptera, Beetles; Volume 2: Morphology and Systematics (Elateroidea, Bostrichiformia, Cucujiformia partim). In: Kristensen NP, Beutel RG, editors. Handbook of Zoology, Arthropoda: Insecta. Berlin and New York: Walter de Gruyter GmbH & Co. KG; 2010.

Timmermans MJTN, Dodsworth S, Culverwell CL, Bocak L, Ahrens D, Littlewood DTJ, *et al.* Why barcode? High-throughput multiplex sequencing of mitochondrial genomes for molecular systematics. Nucleic Acids Res. 2010;38(21):e197; doi:10.1093/nar/gkq807.

Timmermans MJTN, Barton C, Haran J, Ahrens D, Ollikainen A, Culverwell L, *et al.* Family-Level Sampling of Mitochondrial Genomes in Coleoptera: Compositional Heterogeneity and Phylogenetics. Genome Biol Evol. 2016;8:161–175; doi:10.1093/gbe/evv241.

Zhang SQ, Che LH, Li Y, Dan L, Pang H, Ślipiński A, Zhang P. Evolutionary history of Coleoptera revealed by extensive sampling of genes and species. Nat Commun. 2018;205:2041–1723; doi:10.1038/s41467-017-02644-4.

Supplementary Table S1. The list of taxa included in the *LSU*, *SSU* rRNA, *rrnL*, and *cox1* mitochondrial DNA dataset with GenBank accession and voucher ID numbers.

Suborder/Series Superfamily/Family	Subfamily	Genus/Species	*SSU* rRNA	*LSU* rRNA	mitochondrial DNA *rrnL*	*cox1*	Voucher ID
**ADEPHAGA**							
Carabidae	Trechinae	*Bembidion* spp.	JN170213	GQ503347	JF778804	GU454796	chimaera
**ARCHOSTEMATA**							
Cupedidae		*Priacma serrata*/ *Prolixocupes lobiceps*	EU797411	GU591995	EU734895	EU839762	chimaera
**MYXOPHAGA**							
Hydroscaphidae		*Hydroscapha granulum* /*natans*	AF012525	-	NC012144	NC012144	chimaera
Sphaeriusidae		*Sphaerius* sp./ *Lepicerus inaequalis*	EU797414	GU591993	NC011322	NC011322	chimaera
**POLYPHAGA**							
**SCIRTIFORMIA**							
SCIRTOIDEA							
Clambidae		*Clambus pubescens*	EF362951	-	AM884186	DQ155704	BMNH673260
Clambidae		gen. sp.	KF625497	KF626105	KF625804	KF625196	UPOL001320
Decliniidae		*Declinia versicolor*	AY745556	AJ862791	-	-	BMNH693609
Eucinetidae		*Eucinetus haemorrhoidalis*	KF625496	KF626097	KF625806	KF625198	UPOL001319
Eucinetidae		*Eucinetus* sp.	AF427609	DQ198697	-	DQ198541	BMNH679351
Scirtidae		*Cyphon hilaris*	AF201419	DQ198698	DQ198620	DQ198542	BMNH679123
Scirtidae		*Cyphon* sp.	KF625501	KF626101	KF625809	KF625202	UPOLRK0161
Scirtidae		*Prionocyphon sexmaculatus*	KF625516	KF626117	KF625822	KF625216	UPOLRK0170
Scirtidae		*Scirtes hemisphericus*	AF451937	DQ198699	-	-	BMNH679275
Scirtidae		*Scirtes* sp.	KF625500	KF626100	KF625808	KF625201	UPOLRK0160
Scirtidae		gen. sp.	KF625505	KF626106	KF625813	KF625206	UPOL001321
Scirtidae		gen. sp.	KF625498	KF626098	KF625807	KF625199	UPOLRK0147
Scirtidae		gen. sp.	KF625499	KF626099	-	KF625200	UPOLRK0159
Scirtidae		gen. sp.	KF625502	KF626102	KF625810	KF625203	UPOLRK0162
Scirtidae		gen. sp.	KF625504	KF626104	KF625812	KF625205	UPOLRK0165
Scirtidae		gen. sp.	KF625512	KF626113	KF625819	KF625212	UPOLRK0166
Scirtidae		gen. sp.	KF625513	KF626114	-	KF625213	UPOLRK0167

**STAPHYLINIFORMIA**							
HYDROPHILOIDEA							
Leiodidae	Cholevinae	*Catops picipes*	AJ810734	-	FM209287	FM209288	BMNH290666
Leiodidae	Cholevinae	*Nargus velox*	AJ810735	-	GU356766	HQ164624	BMNH833811
Hydrophilidae	Georissinae	*Georissus crenulatus*	AY745584	DQ202637	DQ202580	DQ221983	BMNH679200
Hydrophilidae	Sphaeridiinae	*Cercyon ustulatus*	AM287129	-	AM287071	AM287093	346758
Hydrophilidae	Chaetarthriinae	*Anacaena globulus*	AM287125	-	AM287064	AM287086	346697
Hydrophilidae	Hydrophilinae	*Hydrobius fuscipes*	AJ810720	-	AM287070	AM287092	290645
Sphaeritidae		*Sphaerites glabratus*	AJ810728	DQ202650	AM287077	DQ222001	BMNH679280
STAPHYLINOIDEA							
Hydraenidae	Ochthebiinae	*Ochthebius melanescens*	AJ810732	AJ810767	-	HE970900	MNCNAI0344
Hydraenidae	Ochthebiinae	*Ochthebius minimus*	AJ810731	DQ202624	FM209291	FM209292	BMNH679120
Silphidae	Silphinae	*Oiceoptoma thoracicum*	AJ810736	AB285581	AB285549	AB606436	290669
Silphidae	Silphinae	*Thanatophilus rugosus*	EF213790	EF213811	AB285546	AB606434	BMNH673311
Staphylinidae	Oxytelinae	*Bledius femoralis*	AY745627	DQ202681	DQ202608	DQ222015	BMNH679369
Staphylinidae	Pseudopsinae	*Pseudopsis sulcata*	AY745630	DQ202651	DQ202587	DQ221990	BMNH679246
Staphylinidae	Scaphidiinae	*Scaphidium quadrimaculatum*	AY745631	DQ202643	DQ202582	DQ221985	BMNH679234
**SCARABAEIFORMIA**							
SCARABAEOIDEA							
Scarabaeidae	Scarabaeinae	*Cheironitis hoplosternus*	AY821528	AY131781	AY131597	AY131940	BMNH679878
Scarabaeidae	Scarabaeinae	*Onthophagus crinitis*	AY821535	AY131759	AY131574	AY131924	BMNH679858
Scarabaeidae	Aphodiinae	*Australammoecius occidentalis*	EF487639	AY132457	EF487822	EF656781	BMNH703639
Scarabaeidae	Aphodiinae	*Podotenus storeyi*	EF487648	AY132494	EF487788	AY132432	BMNH703575
Scarabaeidae	Melolonthinae	*Lepidiota stradbrokensis*	EF487696	EU084209	EF487881	EF487763	BMNH671319
Scarabaeidae	Dynastinae	Dynastinae gen. sp.	EF487663	AY132488	EF487817	AY132397	BMNH703635
Scarabaeidae	Cetoniinae	*Oxythyrea cinctella*	EF487653	EU084149	EF487962	EF487733	BMNH678461
Scarabaeidae	Melolonthinae	*Holotrichia seticollis*	EF487687	DQ524596	DQ680877	DQ524528	BMNH677874
Scarabaeidae	Sericinae	*Omaloplia nigromarginata*	EF487705	EU084255	EF487791	EF487770	BMNH747063
Scarabaeidae	Sericinae	*Serica brunnea*	EF487712	EU084263	EF487872	EF487776	BMNH703005
Scarabaeidae	Sericinae	*Gynaecoserica variipennis*	EF487683	EU084189	EF487968	EF487752	BMNH678396
**DERODONTIFORMIA**							
DERODONTOIDEA							
Nosodendridae		*Nosodendron fasciculare*/	AY748114	KP419543	KX087322	KX087322	chimaera

		californicum					
**ELATERIFORMIA**							
BUPRESTOIDEA							
Buprestidae	Agrilinae	*Agrilus* sp.	AF451934	DQ198701	DQ198622	DQ198544	UPOL001047
Buprestidae	Buprestinae	*Anthaxia hungarica*	DQ100484	DQ198702	DQ198623	DQ198545	UPOL000M24
Buprestidae	Julodinae	Julodinae gen. sp.	AF451935	DQ198703	DQ198624	DQ198546	BMNH679324
Buprestidae	Agrilinae	*Trachys minutus*	AF451936	DQ198704	-	DQ198547	BMNH679281
BYRRHOIDEA							
Byrrhidae	Byrrhinae	*Byrrhus pilula*	AF427604	DQ198705	DQ198625	DQ198548	BMNH679172
Byrrhidae	Syncalyptinae	*Chaetophora spinosa*	AF451929	DQ198706	-	-	BMNH679203
Byrrhidae	Syncalyptinae	*Curimopsis setigera*	AF451930	DQ198707	-	-	BMNH679204
Callirhipidae		*Horatocera nipponica*	KF625510	KF626111	-	-	UPOL001248
Callirhipidae		gen. sp.	KF625511	KF626112	KF625818	KF625211	UPOL001249
Callirhipidae		gen. sp.	DQ100490	DQ198726	DQ198637	DQ198560	UPOL000M23
Chelonariidae		gen. sp.	KF625508	KF626109	KF625816	KF625209	UPOL001323
Chelonariidae		gen. sp.	KF625509	KF626110	KF625817	KF625210	UPOL001324
Chelonariidae		gen. sp.	DQ100488	DQ198724	DQ198635	DQ198558	UPOL000M06
Dryopidae		*Dryops algiricus*	AF451926	-	AJ862734	-	BMNH693620
Dryopidae		*Pomatinus substriatus*	AF451924	DQ198708	DQ198626	DQ198549	BMNH693616
Elmidae	Elminae	*Elmis maugetti*	AF451916	DQ198709	-	-	BMNH693612
Elmidae	Elminae	*Homalosolus hospitalis*	AF451921	DQ198710	-	-	BMNH693626
Elmidae	Elminae	*Limnius volckmari*	AF451914	DQ198712	DQ198627	DQ198550	BMNH679263
Elmidae	Elminae	*Macronychus quadrituberculatus*	AF451920	DQ198713	EF209458	EF209578	BMNH693611
Elmidae	Elminae	*Oulimnius rivularis*	AF451913	DQ198714	DQ198628	DQ198551	BMNH679264
Elmidae	Elminae	*Limnius perrisi*	AF451915	DQ198711	AJ862736	AJ862800	BMNH693613
Elmidae	Elminae	*Stenelmis canaliculata*	AF451919	DQ198716	-	-	BMNH693614
Elmidae	Larainae	*Potamophilus acuminatus*	AF451911	-	EF209464	EF209584	FC_E07
Elmidae	Larainae	*Potamodytes* sp.	AF451912	DQ198715	DQ198629	DQ198552	BMNH679360
Eulichadidae	Eulichadinae	*Eulichas* sp.	DQ100489	DQ198725	DQ198636	DQ198559	UPOL000M22
Heteroceridae	Heterocerinae	*Augyles maritimus*	AF451927	DQ198717	-	-	BMNH693618
Heteroceridae	Heterocerinae	*Heterocerus* sp.	AF451928	DQ198718	DQ198630	DQ198553	UPOL001048
Limnichidae	Limnichinae	*Limnichus pygmaeus*	AF451923	DQ198719	DQ198631	DQ198554	BMNH679196
Limnichidae	Limnichinae	gen. sp.	KF625495	KF626096	KF625805	KF625197	UPOL001318
Psephenidae		gen. sp.	KF625503	KF626103	KF625811	KF625204	UPOLRK0163
Psephenidae	Eubrianacinae	*Eubrianax edwardsi*	AF451933	DQ198720	-	-	BMNH679347
Psephenidae	Eubrianacinae	*Eubrianax* sp.	DQ100485	DQ198721	DQ198632	DQ198555	UPOL000M33
Psephenidae		gen. sp.	KF625514	KF626115	KF625820	KF625214	UPOLRK0168
Psephenidae		gen. sp.	KF625515	KF626116	KF625821	KF625215	UPOLRK0169

Ptilodactylidae	Cladotominae	*Paralichas pectinatus*	DQ100486	DQ198722	DQ198633	DQ198556	UPOL000M41
Ptilodactylidae	Ptilodactylinae	*Ptilodactyla serricornis*	AF451932	DQ198723	DQ198634	DQ198557	BMNH693606
Ptilodactylidae		gen. sp.	KF625517	KF626118	KF625823	KF625222	UPOLRK0131
Ptilodactylidae		gen. sp.	KF625518	KF626119	KF625824	KF625217	UPOLRK0149
Ptilodactylidae		gen. sp.	KF625519	KF626120	-	KF625218	UPOLRK0150
Ptilodactylidae		gen. sp.	KF625520	KF626121	KF625825	KF625219	UPOLRK0151
Ptilodactylidae		gen. sp.	KF625521	KF626122	KF625826	KF625220	UPOLRK0152
Ptilodactylidae		gen. sp.	KF625522	KF626123	KF625827	KF625221	UPOLRK0153
ELATEROIDEA							
Artematopodidae	Artematopodinae	*Eurypogon brevipennis*	KF294763	KF294769	KF294757	KF294776	UPOL001335
Artematopodidae	Artematopodinae	*Eurypogon japonicus*	KF294761	KF294767	KF294755	KF294774	UPOLRK0091
Artematopodidae	Artematopodinae	*Eurypogon hisamatsui*	KF294762	KF294768	KF294756	KF294775	UPOLRK0128
Cantharidae	Cantharinae	*Cantharis rufa*	-	DQ198767	DQ198684	DQ198607	BMNH703089
Cantharidae	Cantharinae	*Metacantharis haemorrhoidalis*	DQ100526	DQ198768	DQ198685	DQ198608	UPOL000M14
Cantharidae	Cantharinae	*Rhagonycha nigriceps*	DQ100527	DQ198769	DQ198686	DQ198609	UPOL000M15
Cantharidae	Cantharinae	*Rhagonycha lignosa*	AF451939	DQ198770	DQ198687	DQ198610	BMNH679176
Cantharidae	Cantharinae	Cantharinae gen. sp.	KF625614	KF626216	KF625906	KF625313	UPOL001300
Cantharidae	Cantharinae	*Athemus* sp.	KF625615	KF626217	KF625907	KF625314	UPOL001301
Cantharidae	Cantharinae	*Themus* sp.	KF625616	KF626218	KF625908	KF625315	UPOL001302
Cantharidae	Cantharinae	*Prothemus* sp.	KF625617	KF62621	9KF625909	KF625316	UPOL001303
Cantharidae	Cantharinae	*Habronychus* sp.	KF625625	KF626227	KF625917	KF625324	UPOL001311
Cantharidae	Cantharinae	*Cratosilis sicula*	KF625587	KF626189	KF625882	KF625287	UPOLRK0094
Cantharidae	Cantharinae	*Athemellus insulsus*	KF625620	KF626222	KF625912	KF625319	UPOL001306
Cantharidae	Cantharinae	*Cantharis rustica*	AF451940	-	EU301849	DQ156062	BMNH676948
Cantharidae	Cantharinae	*Podabrus temporalis*	KF625621	KF626223	KF625913	KF625320	UPOL001307
Cantharidae	Cantharinae	*Rhagonycha* sp.	KF625628	KF626230	KF625920	KF625327	UPOL001314
Cantharidae	Cantharinae	Cantharinae gen. sp.	KF625629	KF626231	KF625921	KF625328	UPOL001315
Cantharidae	Cantharinae	*Cantharis* sp.	KF625631	KF626233	KF625923	KF625330	UPOL001317
Cantharidae	Cantharinae	*Lycocerus* sp.	KF625591	KF626193	KF625886	KF625291	UPOLRK0176
Cantharidae	Chauliognathinae	*Chauliognathus opacus*	HM156710	HM156702	FJ613418	FJ613418	BT0055
Cantharidae	Chauliognathinae	*Ichthyurus* sp.	DQ100531	DQ198774	DQ198691	DQ198614	UPOL000M12
Cantharidae	Chauliognathinae	*Chauliognathus* sp.	KF625600	KF626202	KF625892	KF625300	UPOL001250
Cantharidae	Chauliognathinae	*Ichthyurus* sp.	KF625605	KF626207	KF625897	KF625304	UPOL001291
Cantharidae	Chauliognathinae	Chauliognathinae gen. sp.	KF625612	KF626214	KF625904	KF625311	UPOL001298
Cantharidae	Chauliognathinae	*Chauliognathus* sp	KF625613	KF626215	KF625905	KF625312	UPOL001299
Cantharidae	Chauliognathinae	*Microichthyurus* sp.	KF625619	KF626221	KF625911	KF625318	UPOL001305
Cantharidae	Chauliognathinae	*Trypherus mutilatus*	KF625622	KF626224	KF625914	KF625321	UPOL001308
Cantharidae	Chauliognathinae	Chauliognathinae gen. sp.	KF625623	KF626225	KF625915	KF625322	UPOL001309

Cantharidae	Chauliognathinae	Chauliognathinae gen. sp.	KF625634	KF626236	KF625926	KF625333	UPOL001393
Cantharidae	Chauliognathinae	Chauliognathinae gen. sp.	KF625635	KF626237	KF625927	KF625334	UPOL001394
Cantharidae	Chauliognathinae	Chauliognathinae gen. sp.	KF625589	KF626191	KF625884	KF625289	UPOLRK0155
Cantharidae	Chauliognathinae	Chauliognathinae gen. sp.	KF625592	KF626194	-	KF625292	UPOLRK0177
Cantharidae	Chauliognathinae	Chauliognathinae gen. sp.	KF625594	KF626196	KF625888	KF625294	UPOLRK0179
Cantharidae	Chauliognathinae	Chauliognathinae gen. sp.	KF625599	KF626201	-	KF625299	UPOLRK0200
Cantharidae	Malthininae	*Malthodes* sp.	DQ100532	DQ198776	DQ198693	DQ198616	UPOL000M20
Cantharidae	Malthininae	Malthininae gen. sp.	DQ100533	DQ198777	DQ198694	DQ198617	UPOL000M21
Cantharidae	Malthininae	*Inmalthodes* sp.	DQ100534	DQ198778	DQ198695	DQ198618	UPOL000M27
Cantharidae	Malthininae	Malthininae gen. sp.	KF625602	KF626204	KF625894	KF625302	UPOL001288
Cantharidae	Malthininae	*Inmalthodes* sp.	KF625603	KF626205	KF625895	KF625303	UPOL001289
Cantharidae	Malthininae	*Inmalthodes* sp.	KF625604	KF626206	KF625896	-	UPOL001290
Cantharidae	Malthininae	Malthininae gen. sp.	KF625624	KF626226	KF625916	KF625323	UPOL001310
Cantharidae	Malthininae	Malthininae gen. sp.	KF625626	KF626228	KF625918	KF625325	UPOL001312
Cantharidae	Malthininae	*Malthinus* sp.	KF625627	KF626229	KF625919	KF625326	UPOL001313
Cantharidae	Malthininae	Malthininae gen. sp.	KF625630	KF626232	KF625922	KF625329	UPOL001316
Cantharidae	Malthininae	Malthininae gen. sp.	KF625590	KF626192	KF625885	KF625290	UPOLRK0157
Cantharidae	Malthininae	Malthininae gen. sp.	KF625593	KF626195	KF625887	KF625293	UPOLRK0178
Cantharidae	Malthininae	Malthininae gen. sp.	KF625595	KF626197	KF625889	KF625295	UPOLRK0180
Cantharidae	Malthininae	*Malthinus* sp.	KF625596	KF626198	KF625890	KF625296	UPOLRK0181
Cantharidae	Silinae	*Laemoglyptus* sp.	DQ100528	DQ198771	DQ198688	DQ198611	UPOL000M10
Cantharidae	Silinae	*Asiosilis* sp.	DQ100530	DQ198773	DQ198690	DQ198613	UPOL000M13
Cantharidae	Silinae	*Laemoglyptus* sp.	KF625601	KF626203	KF625893	KF625301	UPOL001287
Cantharidae	Silinae	gen. sp.	KF625606	KF626208	KF625898	KF625305	UPOL001292
Cantharidae	Silinae	gen. sp.	KF625607	KF626209	KF625899	KF625306	UPOL001293
Cantharidae	Silinae	gen. sp.	KF625608	KF626210	KF625900	KF625307	UPOL001294
Cantharidae	Silinae	gen. sp.	KF625609	KF626211	KF625901	KF625308	UPOL001295
Cantharidae	Silinae	gen. sp.	KF625610	KF626212	KF625902	KF625309	UPOL001296
Cantharidae	Silinae	gen. sp.	KF625588	KF626190	KF625883	KF625288	UPOLRK0154
Cantharidae	Silinae	gen. sp.	KF625597	KF626199	-	KF625297	UPOLRK0182
Cantharidae		gen. sp.	KF625611	KF626213	KF625903	KF625310	UPOL001297
Cantharidae	Cantharinae	*Lycocerus* sp.	DQ100529	DQ198772	DQ198689	DQ198612	UPOL000M11
Cantharidae	Cantharinae	*Micropodabrus* sp.	KF625618	KF626220	KF625910	KF625317	UPOL001304
Cantharidae		gen. sp.	KF625632	KF626234	KF625924	KF625331	UPOL001391
Cantharidae		gen. sp.	KF625633	KF626235	KF625925	KF625332	UPOL001392
Cantharidae		gen. sp.	KF625598	KF626200	KF625891	KF625298	UPOLRK0199
Cerophytidae		*Cerophytum elateroides*	KF625714	KF626302	KF626002	KF625407	UPOLRK0129
Elateridae	Agrypninae	*Adelocera* sp.	HQ333794	HQ333889	KF626035	HQ333976	UPOLRK0041
Elateridae	Agrypninae	*Adelocera* sp.	HQ333815	HQ333910	KF626033	KF625439	UPOLRK0062

Elateridae	Agrypninae	*Lacon* sp.	HQ333789	HQ333884	KF626036	HQ333971	UPOLRK0036
Elateridae	Agrypninae	*Pyrophorus* sp.	HQ333753	HQ333848	HQ333672	KF625435	UPOL001423
Elateridae	Agrypninae	*Tetrigus cyprius*	KF625744	KF626327	KF626032	KF625438	UPOLRK0228
Elateridae	Agrypninae	*Cryptalaus* sp.	HQ333781	HQ333876	KF626034	-	UPOLRK0028
Elateridae	Agrypninae	*Pyrophorus* sp.	KF625742	KF626325	KF626030	KF625436	UPOLRK0213
Elateridae	Agrypninae	*Chalcolepidius* sp.	KF625743	KF626326	KF626031	KF625437	UPOLRK0219
Elateridae	Agrypninae	*Drilus concolor*	HQ333827	KF626322	HQ333734	HQ334007	UPOLRK0074
Elateridae	Agrypninae	*Malacogaster passerinii*	KF625741	KF626321	KF626029	KF625432	UPOLRK0369
Elateridae	Agrypninae	*Selasia* sp.	KF625739	KF626323	KF626027	KF625433	UPOLRK0158
Elateridae	Agrypninae	*Selasia* sp.	KF625740	KF626324	KF626028	KF625434	UPOLRK0172
Elateridae	Agrypninae	Anaissini gen. sp.	KF625761	KF626341	KF626055	KF625452	UPOLRK0301
Elateridae	Agrypninae	*Agrypnus murinus*	AF451943	DQ198735	DQ198645	DQ198567	UPOL001049
Elateridae	Agrypninae	*Agrypnus* sp.	HQ333757	HQ333852	HQ333676	HQ333941	UPOLRK0004
Elateridae	Agrypninae	*Agrypnus* sp.	HQ333783	HQ333878	HQ333697	HQ333965	UPOLRK0030
Elateridae	Agrypninae	*Agrypnus* sp.	HQ333810	HQ333905	HQ333719	HQ333991	UPOLRK0057
Elateridae	Agrypninae	*Agrypnus* sp.	HQ333820	HQ333915	HQ333727	HQ334000	UPOLRK0067
Elateridae	Agrypninae	*Adelocera* sp.	HQ333778	HQ333873	HQ333694	HQ333961	UPOLRK0025
Elateridae	Agrypninae	*Adelocera* sp.	HQ333806	HQ333901	HQ333715	HQ333987	UPOLRK0053
Elateridae	Agrypninae	*Adelocera* sp.	HQ333817	HQ333912	HQ333724	HQ333997	UPOLRK0064
Elateridae	Agrypninae	*Adelocera* sp.	HQ333818	HQ333913	HQ333725	HQ333998	UPOLRK0065
Elateridae	Agrypninae	*Conoderus* sp.	HQ333746	HQ333841	HQ333665	HQ333931	UPOL001416
Elateridae	Agrypninae	*Conoderus* sp	HQ333747	HQ333842	HQ333666	HQ333932	UPOL001417
Elateridae	Agrypninae	*Drasterius bimaculatus*	HQ333793	HQ333888	HQ333704	HQ333975	UPOLRK0040
Elateridae	Agrypninae	*Drasterius* sp.	HQ333816	HQ333911	HQ333723	HQ333996	UPOLRK0063
Elateridae	Agrypninae	Agrypninae gen. sp.	HQ333798	HQ333893	HQ333708	HQ333980	UPOLRK0045
Elateridae	Agrypninae	*Platycrepidius* sp.	HQ333748	HQ333843	HQ333667	HQ333933	UPOL001418
Elateridae	Agrypninae	*Pyrophorus* sp.	HQ333751	HQ333846	HQ333670	HQ333936	UPOL001421
Elateridae	Agrypninae	*Chalcolepidius* sp.	HQ333752	HQ333847	HQ333671	HQ333937	UPOL001422
Elateridae	Agrypninae	*Cryptalaus* sp.	HQ333768	HQ333863	HQ333685	HQ333951	UPOLRK0015
Elateridae	Agrypninae	*Cryptalaus* sp.	HQ333834	HQ333926	HQ333740	HQ334014	UPOLRK0082
Elateridae	Agrypninae	*Selasia* sp.	HQ333824	HQ333919	HQ333731	HQ334004	UPOLRK0071
Elateridae	Agrypninae	*Drilus flavescens*	DQ100501	DQ198748	DQ198657	DQ198579	UPOL001046
Elateridae	Agrypninae	*Drilus* sp.	HQ333826	HQ333921	HQ333733	HQ334006	UPOLRK0073
Elateridae	Agrypninae	*Drilus* sp.	-	-	HQ333739	HQ334013	UPOLRK0081
Elateridae	Agrypninae	*Drilus mauritanicus*	HQ333837	HQ333928	HQ333743	HQ334016	UPOLRK0085
Elateridae	Cardiophorinae	*Cardiophorus erichsoni*	HQ333790	HQ333885	HQ333701	HQ333972	UPOLRK0037
Elateridae	Cardiophorinae	Cardiophorinae gen. sp.	HQ333823	HQ333918	HQ333730	HQ334003	UPOLRK0070
Elateridae	Cardiophorinae	Cardiophorinae gen. sp.	AF451942	DQ198739	DQ198649	DQ198571	BMNH679341
Elateridae	Cardiophorinae	*Dicronychus rubripes*	HQ333764	HQ333859	KF626023	HQ333947	UPOLRK0011

Elateridae	Cardiophorinae	*Dicronychus cinereus*	HQ333776	HQ333871	KF626024	HQ333959	UPOLRK0023
Elateridae	Cardiophorinae	Cardiophorinae gen. sp.	HQ333784	HQ333879	KF626025	HQ333966	UPOLRK0031
Elateridae	Cardiophorinae	Cardiophorinae gen. sp.	HQ333788	HQ333883	KF626026	HQ333970	UPOLRK0035
Elateridae	Dendrometrinae	*Athous vittatus*	HQ333755	HQ333850	HQ333674	HQ333939	UPOLRK0002
Elateridae	Dendrometrinae	*Pheletes quercus*	HQ333775	HQ333870	HQ333692	HQ333958	UPOLRK0022
Elateridae	Dendrometrinae	*Cidnopus pilosus*	HQ333792	HQ333887	HQ333703	HQ333974	UPOLRK0039
Elateridae	Dendrometrinae	*Anostirus purpureus*	HQ333761	HQ333856	HQ333679	HQ333945	UPOLRK0008
Elateridae	Dendrometrinae	*Neopristilophus serrifer*	HQ333765	HQ333860	HQ333682	HQ333948	UPOLRK0012
Elateridae	Dendrometrinae	*Selatosomus latus*	HQ333774	HQ333869	HQ333691	HQ333957	UPOLRK0021
Elateridae	Dendrometrinae	Dendrometrinae gen. sp.	HQ333787	HQ333882	HQ333700	HQ333969	UPOLRK0034
Elateridae	Dendrometrinae	*Platiana* sp.	HQ333782	HQ333877	HQ333696	HQ333964	UPOLRK0029
Elateridae	Dendrometrinae	*Hypolithus* sp.	HQ333795	HQ333890	HQ333705	HQ333977	UPOLRK0042
Elateridae	Dendrometrinae	*Semiotus* sp.	HQ333799	HQ333894	HQ333709	HQ333981	UPOLRK0046
Elateridae	Dendrometrinae	*Oxynopterus* sp.	HQ333800	HQ333895	HQ333710	HQ333982	UPOLRK0047
Elateridae	Dendrometrinae	*Aplotarsus incanus*	-	DQ198737	DQ198647	DQ198569	BMNH703088
Elateridae	Dendrometrinae	*Athous haemorrhoidalis*	AF451944	DQ198738	DQ198648	DQ198570	BMNH679174
Elateridae	Dendrometrinae	*Denticollis linearis*	DQ100498	DQ198741	DQ198651	DQ198573	UPOL000M25
Elateridae	Dendrometrinae	*Panspaeus guttatus*	DQ100499	DQ198742	DQ198652	DQ198574	BMNH703090
Elateridae	Dendrometrinae	*Stenagostus rhombeus*	AF451945	DQ198744	DQ198653	DQ198576	BMNH679144
Elateridae	Dendrometrinae	*Denticollis* sp.	HQ333759	HQ333854	KF626041	HQ333943	UPOLRK0006
Elateridae	Dendrometrinae	*Nothodes parvulus*	HQ333763	HQ333858	HQ333681	KF625443	UPOLRK0010
Elateridae	Dendrometrinae	Dendrometrinae gen. sp.	KF625751	KF626338	KF626053	KF625451	UPOLRK0251
Elateridae	Dendrometrinae	*Hemicrepidius hirtus*	KF625754	KF626340	KF626054	KF625448	UPOLRK0297
Elateridae		gen. sp.	KF625762	KF626346	KF626057	-	UPOL001237
Elateridae	Elaterinae	*Octinodes* sp.	HQ333749	HQ333844	HQ333668	HQ333934	UPOL001419
Elateridae	Elaterinae	*Elater* sp.	HQ333766	HQ333861	HQ333683	HQ333949	UPOLRK0013
Elateridae	Elaterinae	*Tomicephalus* sp.	HQ333750	HQ333845	HQ333669	HQ333935	UPOL001420
Elateridae	Elaterinae	gen. sp.	HQ333773	HQ333868	HQ333690	HQ333956	UPOLRK0020
Elateridae	Elaterinae	gen. sp.	HQ333819	HQ333914	HQ333726	HQ333999	UPOLRK0066
Elateridae	Elaterinae	*Ludioschema* sp.	HQ333777	HQ333872	HQ333693	HQ333960	UPOLRK0024
Elateridae	Elaterinae	*Ludioschema* sp.	HQ333808	HQ333903	HQ333717	HQ333989	UPOLRK0055
Elateridae	Elaterinae	*Anoplischius* sp.	HQ333745	HQ333840	HQ333664	HQ333930	UPOL001415
Elateridae	Elaterinae	*Ampedus* sp.	HQ333758	HQ333853	HQ333677	HQ333942	UPOLRK0005
Elateridae	Elaterinae	*Ampedus sanguinolentus*	HQ333760	HQ333855	HQ333678	HQ333944	UPOLRK0007
Elateridae	Elaterinae	*Ampedus rufipennis*	HQ333762	HQ333857	HQ333680	HQ333946	UPOLRK0009
Elateridae	Elaterinae	*Ampedus* sp.	HQ333771	HQ333866	HQ333688	HQ333954	UPOLRK0018
Elateridae	Elaterinae	*Ampedus sinuatus*	HQ333791	HQ333886	HQ333702	HQ333973	UPOLRK0038
Elateridae	Elaterinae	*Ampedus* sp.	HQ333822	HQ333917	HQ333729	HQ334002	UPOLRK0069
Elateridae	Elaterinae	*Ampedus balteatus*	AF427605	DQ198736	DQ198646	DQ198568	BMNH679173

Elateridae	Elaterinae	*Agriotes acuminatus*	HQ333756	HQ333851	HQ333675	HQ333940	UPOLRK0003
Elateridae	Elaterinae	*Agriotes ustulatus*	HQ333786	HQ333881	HQ333699	HQ333968	UPOLRK0033
Elateridae	Elaterinae	*Agriotes lineatus*	-	DQ198733	DQ198643	DQ198566	BMNH703086
Elateridae	Elaterinae	*Cebrio* sp.	DQ100497	DQ198740	DQ198650	DQ198572	BMNH679376
Elateridae	Elaterinae	gen. sp.	HQ333767	HQ333862	HQ333684	HQ333950	UPOLRK0014
Elateridae	Elaterinae	gen. sp.	HQ333769	HQ333864	HQ333686	HQ333952	UPOLRK0016
Elateridae	Elaterinae	gen. sp.	HQ333770	HQ333865	HQ333687	HQ333953	UPOLRK0017
Elateridae	Elaterinae	gen. sp.	HQ333801	HQ333896	HQ333711	HQ333983	UPOLRK0048
Elateridae	Elaterinae	gen. sp.	HQ333812	HQ333907	HQ333720	HQ333993	UPOLRK0059
Elateridae	Elaterinae	*Melanotus villosus*	HQ333754	HQ333849	HQ333673	HQ333938	UPOLRK0001
Elateridae	Elaterinae	*Priopus ornatus*	HQ333785	HQ333880	HQ333698	HQ333967	UPOLRK0032
Elateridae	Elaterinae	*Priopus humeralis*	HQ333821	HQ333916	HQ333728	HQ334001	UPOLRK0068
Elateridae	Elaterinae	*Anchastus* sp.	HQ333804	HQ333899	HQ333714	HQ333986	UPOLRK0051
Elateridae	Elaterinae	*Anchastus* sp.	HQ333809	HQ333904	HQ333718	HQ333990	UPOLRK0056
Elateridae	Elaterinae	*Anchastus* sp.	HQ333813	HQ333908	HQ333721	HQ333994	UPOLRK0060
Elateridae	Elaterinae	*Anchastus* sp.	HQ333814	HQ333909	HQ333722	HQ333995	UPOLRK0061
Elateridae	Elaterinae	*Mulsanteus* sp.	HQ333744	HQ333839	KF626037	HQ333929	UPOL001414
Elateridae	Elaterinae	*Agriotes obscurus*	HQ333805	HQ333900	KF626039	KF625441	UPOLRK0052
Elateridae	Elaterinae	Adrastini gen. sp.	HQ333780	HQ333875	KF626038	HQ333963	UPOLRK0027
Elateridae	Elaterinae	*Cebrio* sp.	KF625745	KF626329	KF626040	KF625440	UPOLRK0142
Elateridae	Elaterinae	*Octinodes* sp.	KF625746	KF626328	-	KF625442	UPOLRK0306
Elateridae	Elaterinae	gen. sp.	KF625752	KF626337	KF626052	KF625449	UPOLRK0203
Elateridae	Elaterinae	Physorhinini gen. sp.	KF625758	KF626334	KF626056	KF625453	UPOLRK0310
Elateridae	Elaterinae	gen. sp.	KF625755	KF626339	KF626046	KF625455	UPOLRK0400
Elateridae	Elaterinae	gen. sp.	KF625757	KF626343	KF626048	KF625457	UPOLRK0404
Elateridae	Elaterinae	gen. sp.	KF625759	KF626344	KF626049	KF625458	UPOLRK0406
Elateridae	Elaterinae	Agriotini gen. sp.	KF625763	KF626345	-	KF625459	UPOL001413
Elateridae	Hemiopinae	gen. sp.	KF625756	KF626342	KF626047	KF625456	UPOLRK0403
Elateridae	Lissominae	*Drapetes mordelloides*	HQ333828	HQ333922	HQ333735	HQ334008	UPOLRK0075
Elateridae	Lissominae	gen. sp.	KF625747	KF626330	KF626042	KF625444	UPOLRK0334
Elateridae	Lissominae	gen. sp.	KF625748	KF626331	KF626043	KF625445	UPOLRK0335
Elateridae	Lissominae	gen. sp.	KF625749	KF626332	KF626044	KF625446	UPOLRK0353
Elateridae	Lissominae	gen. sp.	KF625750	KF626333	KF626045	KF625447	UPOLRK0354
Elateridae	Lissominae	gen. sp.	KF625760	KF626336	KF626051	KF625450	UPOLRK0174
Elateridae	Morostomatinae	*Diplophoenicus* sp.	KF625753	KF626335	KF626050	KF625454	UPOLRK0145
Elateridae	Negastriinae	*Zorochros* sp.	HQ333796	HQ333891	HQ333706	HQ333978	UPOLRK0043
Elateridae	Negastriinae	*Quasimus* sp.	HQ333802	HQ333897	HQ333712	HQ333984	UPOLRK0049
Elateridae	Negastriinae	*Quasimus* sp.	HQ333803	HQ333898	HQ333713	HQ333985	UPOLRK0050
Elateridae	Negastriinae	gen. sp.	HQ333811	HQ333906	-	HQ333992	UPOLRK0058

Elateridae	Thylacosterninae	*Balgus* sp.	DQ100500	DQ198746	DQ198655	-	BMNH669202
Elateridae	Thylacosterninae	*Pterotarsus bimaculatus*	-	DQ198743	-	DQ198575	BMNH703093
Eucnemidae	Anischiinae	*Anischia kuscheli*	KF625545	KF626145	KF625845	KF625246	UPOLRK0119
Eucnemidae	Anischiinae	*Anischia bicolor*	KF625546	KF626146	KF625846	KF625247	UPOLRK0120
Eucnemidae	Eucneminae	*Idiotarsus* sp.	DQ100493	DQ198730	DQ198641	DQ198563	BMNH703097
Eucnemidae	Macraulacinae	gen. sp.	KF625578	KF626178	KF625873	KF625276	UPOL001233
Eucnemidae	Macraulacinae	gen. sp.	KF625553	KF626152	KF625852	KF625253	UPOLRK0126
Eucnemidae	Macraulacinae	gen. sp.	KF625580	KF626180	KF625875	KF625278	UPOL001235
Eucnemidae	Macraulacinae	gen. sp.	KF625583	KF626184	KF625879	KF625282	UPOL001330
Eucnemidae	Macraulacinae	*Nematodes* sp.	DQ100495	DQ198731	DQ198642	DQ198564	BMNH703107
Eucnemidae	Macraulacinae	*Fornax* sp.	DQ100492	DQ198729	DQ198640	DQ198562	BMNH703106
Eucnemidae	Melasinae	*Entomophthalmus americanus*	DQ100491	DQ198727	DQ198638	-	BMNH703104
Eucnemidae	Melasinae	*Protofarsus* sp.	DQ100496	DQ198732	-	DQ198565	BMNH703095
Eucnemidae	Melasinae	*Arrhipis* sp.	-	DQ198745	DQ198654	DQ198577	BMNH703101
Eucnemidae	Melasinae	*Microrhagus* sp.	KF625569	KF626169	KF625866	KF625270	UPOL001223
Eucnemidae	Melasinae	*Microrhagus pygmaeus*	KF625570	KF626170	KF625867	KF625271	UPOL001224
Eucnemidae	Melasinae	*Isorhipis marmottani*	-	KF626183	KF625878	KF625281	UPOL001329
Eucnemidae	Melasinae	*Melasis buprestoides*	KF625558	KF626158	KF625858	KF625259	UPOLRK0344
Eucnemidae		gen. sp.	HQ333807	HQ333902	HQ333716	HQ333988	UPOLRK0054
Eucnemidae		gen. sp.	HQ333829	HQ333923	HQ333736	HQ334009	UPOLRK0076
Eucnemidae		gen. sp.	HQ333830	HQ333924	HQ333737	HQ334010	UPOLRK0077
Eucnemidae		gen. sp.	HQ333831	HQ333925	KF625843	KF625245	UPOLRK0078
Eucnemidae		gen. sp.	KF625571	KF626171	KF625868	KF625272	UPOL001225
Eucnemidae		gen. sp.	KF625572	KF626172	KF625869	KF625273	UPOL001226
Eucnemidae		gen. sp.	KF625573	KF626173	KF625870	KF625274	UPOL001227
Eucnemidae		gen. sp.	KF625574	KF626174	-	-	UPOL001228
Eucnemidae		gen. sp.	KF625575	KF626175	KF625871	-	UPOL001229
Eucnemidae		gen. sp.	KF625576	KF626176	KF625872	KF625275	UPOL001230
Eucnemidae		gen. sp.	KF625577	KF626177	-	-	UPOL001231
Eucnemidae		gen. sp.	KF625579	KF626179	KF625874	KF625277	UPOL001234
Eucnemidae		gen. sp.	KF625581	KF626181	KF625876	KF625279	UPOL001236
Eucnemidae		gen. sp.	KF625582	KF626182	KF625877	KF625280	UPOL001328
Eucnemidae		gen. sp.	KF625584	KF626185	-	KF625283	UPOL001331
Eucnemidae		gen. sp.	KF625585	KF626186	KF625880	KF625284	UPOL001332
Eucnemidae		gen. sp.	KF625586	KF626187	KF625881	KF625285	UPOL001333
Eucnemidae		gen. sp.	-	KF626188	-	KF625286	UPOL001334
Eucnemidae		gen. sp.	KF625547	KF626147	KF625847	KF625248	UPOLRK0121
Eucnemidae		gen. sp.	KF625548	KF626148	KF625848	KF625249	UPOLRK0122
Eucnemidae		gen. sp.	KF625549	KF626149	KF625849	KF625250	UPOLRK0123

Eucnemidae		gen. sp.	KF625550	KF626150	KF625850	KF625251	UPOLRK0124
Eucnemidae		gen. sp.	KF625552	KF626151	KF625851	KF625252	UPOLRK0125
Eucnemidae		gen. sp.	KF625551	KF626153	KF625853	KF625254	UPOLRK0171
Eucnemidae		gen. sp.	KF625554	KF626154	KF625854	KF625255	UPOLRK0303
Eucnemidae		gen. sp.	KF625555	KF626155	KF625855	KF625256	UPOLRK0340
Eucnemidae		gen. sp.	KF625556	KF626156	KF625856	KF625257	UPOLRK0341
Eucnemidae		gen. sp.	KF625557	KF626157	KF625857	KF625258	UPOLRK0343
Eucnemidae		gen. sp.	KF625559	KF626159	KF625859	KF625260	UPOLRK0345
Eucnemidae		gen. sp.	KF625560	KF626160	KF625860	KF625261	UPOLRK0346
Eucnemidae		gen. sp.	KF625561	KF626161	KF625861	KF625262	UPOLRK0347
Eucnemidae		gen. sp.	KF625562	KF626162	-	KF625263	UPOLRK0348
Eucnemidae		gen. sp.	KF625563	KF626163	-	KF625264	UPOLRK0349
Eucnemidae		gen. sp.	KF625564	KF626164	KF625862	KF625265	UPOLRK0350
Eucnemidae		gen. sp.	KF625565	KF626165	KF625863	KF625266	UPOLRK0351
Eucnemidae		gen. sp.	KF625566	KF626166	KF625864	KF625267	UPOLRK0355
Eucnemidae		gen. sp.	KF625567	KF626167	-	KF625268	UPOLRK0356
Eucnemidae		gen. sp.	KF625568	KF626168	KF625865	KF625269	UPOLRK0357
Throscidae		*Trixagus meybohmi*	KF294766	KF294772	KF294760	KF294779	UPOLRK0139
Throscidae		*Trixagus dermestoides*	AF451950	DQ198747	DQ198656	DQ198578	BMNH679235
Throscidae		gen. sp.	KF625543	KF626143	KF625842	KF625243	UPOL001326
Throscidae		gen. sp.	KF625544	KF626144	-	KF625244	UPOL001327
Throscidae		gen. sp.	KF625532	KF626133	-	KF625232	UPOLRK0137
Throscidae		gen. sp.	KF625533	KF626134	KF625836	KF625233	UPOLRK0138
Throscidae		gen. sp.	KF625534	KF626135	-	KF625234	UPOLRK0140
Throscidae		gen. sp.	KF625535	KF626136	KF625837	KF625235	UPOLRK0141
Throscidae		gen. sp.	KF625536	KF626137	KF625838	KF625236	UPOLRK0175
Throscidae		gen. sp.	KF625537	KF626138	-	KF625237	UPOLRK0330
Throscidae		gen. sp.	KF625538	-	-	KF625238	UPOLRK0331
Throscidae		gen. sp.	KF625539	KF626139	KF625839	KF625239	UPOLRK0333
Throscidae		gen. sp.	KF625540	KF626140	-	KF625240	UPOLRK0336
Throscidae		gen. sp.	KF625541	KF626141	KF625840	KF625241	UPOLRK0337
Throscidae		gen. sp.	KF625542	KF626142	KF625841	KF625242	UPOLRK0338
Throscidae		gen. sp.	HQ333838	-	KF625844	HQ334017	UPOLRK0086
Iberobaeniidae		*Iberobaenia minuta*	KT339296	KT339297	-	KT339298	UPOLRK0790
Lampyridae	Lampyrinae	gen. sp.	DQ100509	DQ198759	DQ198667	DQ198590	UPOL000M19
Lampyridae	Lampyrinae	gen. sp.	DQ100510	-	DQ198668	DQ198591	UPOL000M04
Lampyridae	Lampyrinae	*Vesta* sp.	DQ100511	DQ198760	DQ198669	DQ198592	UPOL000M17
Lampyridae	Cyphonocerinae	*Cyphonocerus ruficollis*	DQ100512	-	DQ198670	DQ198593	UPOL000191
Lampyridae	Luciolinae	*Curtos* sp.	DQ100513	DQ198761	DQ198671	DQ198594	UPOL000M16

Lampyridae	Luciolinae	gen. sp.	DQ100514	DQ198762	DQ198672	DQ198595	UPOL000M03
Lampyridae	Luciolinae	*Bourgeoisia* sp.	DQ100515	-	DQ198673	DQ198596	UPOL000M07
Lampyridae	Luciolinae	gen. sp.	DQ100516	-	DQ198674	DQ198597	UPOL000M18
Lampyridae	Ototretinae	gen. sp.	DQ100518	-	DQ198676	DQ198599	UPOL000M38
Lampyridae	Ototretinae	*Flabellotreta obscuricollis*	DQ100519	-	DQ198677	DQ198600	UPOL000M05
Lampyridae	Ototretinae	*Flabellotreta* sp.	DQ100520	DQ198763	DQ198678	DQ198601	UPOL000M34
Lampyridae	Ototretinae	gen. sp.	DQ100521	DQ198764	DQ198679	DQ198602	UPOL000M37
Lampyridae	Ototretinae	*Drilaster borneensis*	DQ100522	-	DQ198680	DQ198603	UPOL000M39
Lampyridae	Ototretinae	gen. sp.	DQ100523	-	DQ198681	DQ198604	UPOL000M32
Lampyridae	Ototretinae	*Lamellipalpus pacholatkoi*	KF625664	KF626254	KF625955	KF625363	UPOLRK0379
Lampyridae	Ototretinae	gen. sp.	DQ100524	-	DQ198682	DQ198605	UPOL000156
Lampyridae		gen. sp.	KF625636	-	KF625928	KF625335	UPOLRK0087
Lampyridae		gen. sp.	KF625637	KF626238	-	KF625336	UPOLRK0089
Lampyridae		gen. sp.	KF625638	KF626239	KF625929	KF625337	UPOLRK0093
Lampyridae		gen. sp.	KF625639	-	KF625930	KF625338	UPOLRK0095
Lampyridae		gen. sp.	KF625640	KF626240	KF625931	KF625339	UPOLRK0096
Lampyridae		gen. sp.	KF625641	KF626241	KF625932	KF625340	UPOLRK0097
Lampyridae		gen. sp.	KF625642	-	KF625933	KF625341	UPOLRK0098
Lampyridae		gen. sp.	KF625643	KF626242	KF625934	KF625342	UPOLRK0099
Lampyridae		gen. sp.	KF625644	KF626243	KF625935	KF625343	UPOLRK0101
Lampyridae		gen. sp.	KF625645	-	KF625936	KF625344	UPOLRK0102
Lampyridae		gen. sp.	KF625646	-	KF625937	KF625345	UPOLRK0103
Lampyridae		gen. sp.	KF625647	-	KF625938	KF625346	UPOLRK0104
Lampyridae		gen. sp.	KF625648	KF626244	KF625939	KF625347	UPOLRK0106
Lampyridae		gen. sp.	KF625649	KF626245	KF625940	KF625348	UPOLRK0107
Lampyridae		gen. sp.	KF625650	KF626246	KF625941	KF625349	UPOLRK0108
Lampyridae		gen. sp.	KF625651	-	KF625942	KF625350	UPOLRK0109
Lampyridae		gen. sp.	KF625652	KF626247	KF625943	KF625351	UPOLRK0110
Lampyridae		gen. sp.	KF625653	KF626248	KF625944	KF625352	UPOLRK0111
Lampyridae		gen. sp.	KF625654	-	KF625945	KF625353	UPOLRK0112
Lampyridae		gen. sp.	KF625655	-	KF625946	KF625354	UPOLRK0113
Lampyridae		gen. sp.	KF625656	KF626249	KF625947	KF625355	UPOLRK0118
Lampyridae		gen. sp.	KF625657	KF626250	KF625948	KF625356	UPOLRK0173
Lampyridae		gen. sp.	KF625658	-	KF625949	KF625357	UPOLRK0372
Lampyridae		gen. sp.	KF625659	-	KF625950	KF625358	UPOLRK0373
Lampyridae		gen. sp.	KF625660	KF626251	KF625951	KF625359	UPOLRK0374
Lampyridae		gen. sp.	KF625661	KF626252	KF625952	KF625360	UPOLRK0375
Lampyridae		gen. sp.	KF625662	-	KF625953	KF625361	UPOLRK0376
Lampyridae		gen. sp.	KF625663	KF626253	KF625954	KF625362	UPOLRK0378

Lampyridae		gen. sp.	KF625665	KF626255	KF625956	KF625364	UPOLRK0380
Lampyridae		gen. sp.	KF625666	KF626256	KF625957	KF625365	UPOLRK0381
Lampyridae		gen. sp.	KF625667	-	KF625958	KF625366	UPOLRK0382
Lampyridae		gen. sp.	KF625668	KF626257	KF625959	KF625367	UPOLRK0383
Lampyridae		gen. sp.	KF625669	KF626258	KF625960	KF625368	UPOLRK0384
Lampyridae		gen. sp.	KF625670	KF626259	KF625961	KF625369	UPOLRK0385
Lampyridae		gen. sp.	KF625671	KF626260	KF625962	KF625370	UPOLRK0386
Lampyridae		gen. sp.	KF625672	KF626261	KF625963	KF625371	UPOLRK0387
Lampyridae		gen. sp.	KF625673	-	KF625964	KF625372	UPOLRK0388
Lampyridae		gen. sp.	KF625674	-	KF625965	KF625373	UPOLRK0389
Lampyridae		gen. sp.	KF625675	KF626262	KF625966	KF625374	UPOLRK0390
Lampyridae		gen. sp.	KF625676	KF626263	KF625967	KF625375	UPOLRK0391
Lampyridae		gen. sp.	KF625677	KF626264	KF625968	KF625376	UPOLRK0392
Lampyridae		gen. sp.	KF625678	KF626265	KF625969	KF625377	UPOLRK0393
Lampyridae		gen. sp.	KF625679	KF626266	KF625970	KF625378	UPOLRK0394
Lampyridae		gen. sp.	KF625680	KF626267	KF625971	KF625379	UPOLRK0395
Lampyridae		gen. sp.	KF625681	KF626268	KF625972	KF625380	UPOLRK0396
Lampyridae		gen. sp.	KF625682	-	KF625973	KF625381	UPOLRK0397
Lampyridae		gen. sp.	KF625683	KF626269	KF625974	KF625382	UPOLRK0398
Lampyridae		gen. sp.	KF625684	KF626270	-	KF625383	UPOLRK0399
Lycidae	Libnetinae	*Libnetis* sp.	DQ181038	DQ181112	DQ180964	DQ181186	UPOL000L02
Lycidae	Libnetinae	*Libnetis* sp.	DQ181104	DQ181178	DQ181030	DQ181252	UPOL001002
Lycidae	Libnetinae	*Libnetis* sp.	DQ181105	DQ181179	DQ181031	DQ181253	UPOL001008
Lycidae	Libnetinae	*Libnetis granicollis*	DQ181107	DQ181181	DQ181033	DQ181255	UPOL001012
Lycidae	Dictyopterinae	*Lycoprogenthes* sp.	DQ181070	DQ181144	DQ180996	DQ181218	UPOL000358
Lycidae	Dictyopterinae	*Lycoprogenthes* sp.	DQ181095	DQ181169	DQ181021	DQ181243	UPOL000801
Lycidae	Dictyopterinae	*Lycoprogenthes* sp.	DQ181096	DQ181170	DQ181022	DQ181244	UPOL000802
Lycidae	Dictyopterinae	*Taphes brevicollis*	DQ181098	DQ181172	DQ181024	DQ181246	UPOL000812
Lycidae	Dictyopterinae	*Dictyoptera elegans*	DQ181073	DQ181147	DQ180999	DQ181221	UPOL000570
Lycidae	Dictyopterinae	*Dictyoptera speciosa*	DQ181074	DQ181148	DQ181000	DQ181222	UPOL000571
Lycidae	Dictyopterinae	*Benibotarus nigripennis*	DQ181075	DQ181149	DQ181001	DQ181223	UPOL000572
Lycidae	Dictyopterinae	*Benibotarus spinicoxis*	DQ181076	DQ181150	DQ181002	DQ181224	UPOL000573
Lycidae	Dictyopterinae	*Pyropterus nigroruber*	DQ181077	DQ181151	DQ181003	DQ181225	UPOL000574
Lycidae	Dictyopterinae	*Dictyoptera* sp.	KF625686	KF626272	KF625976	KF625385	UPOL001275
Lycidae	Dictyopterinae	*Dictyoptera aurora*	KF625687	KF626273	KF625977	KF625386	UPOL001276
Lycidae	Dictyopterinae	*Pyropterus nigroruber*	KF625688	KF626274	KF625978	KF625387	UPOL001277
Lycidae	Dictyopterinae	Dictyopterini gen. sp.	KF625689	KF626275	KF625979	KF625388	UPOL001278
Lycidae	Dictyopterinae	Dictyopterini gen. sp.	KF625690	KF626276	KF625980	KF625389	UPOL001280
Lycidae	Dictyopterinae	Dictyopterini gen. sp.	KF625691	KF626277	KF625981	KF625390	UPOL001282

Lycidae	Dictyopterinae	Dictyopterini gen. sp.	KF625692	KF626278	KF625982	KF625391	UPOL001283
Lycidae	Dictyopterinae	*Lopheros* sp.	KF625693	KF626279	-	-	UPOL001284
Lycidae	Dictyopterinae	*Benibotarus taygetanus*	KF625694	KF626280	KF625983	KF625392	UPOL001285
Lycidae	Dictyopterinae	*Benibotarus* sp.	KF625698	KF626284	KF625987	KF625396	UPOL001367
Lycidae	Dictyopterinae	*Helcophorus* sp.	KF625699	KF626285	KF625988	KF625397	UPOL001369
Lycidae	Lyropaeinae	*Alyculus kurbatovi*	DQ181072	DQ181146	DQ180998	DQ181220	UPOL000543
Lycidae	Lyropaeinae	*Lyropaeus* sp.	DQ181042	DQ181116	DQ180968	DQ181190	UPOL000L11
Lycidae	Lyropaeinae	*Lyropaeus* sp.	DQ181087	DQ181161	DQ181013	DQ181235	UPOL000584
Lycidae	Lyropaeinae	*Lyropaeus* sp.	DQ181088	DQ181162	DQ181014	DQ181236	UPOL000585
Lycidae	Lyropaeinae	*Antennolycus constrictus*	DQ181051	DQ181125	DQ180977	DQ181199	UPOL000L22
Lycidae	Lyropaeinae	*Microlyropaeus dembickyi*	DQ181071	DQ181145	DQ180997	DQ181219	UPOL000542
Lycidae	Lyropaeinae	*Platerodrilus* sp.	DQ181037	DQ181111	DQ180963	DQ181185	UPOL000L01
Lycidae	Lyropaeinae	*Platerodrilus* sp.	DQ181091	DQ181165	DQ181017	DQ181239	UPOL000588
Lycidae	Lyropaeinae	Platerodrilini gen. sp.	DQ181089	DQ181163	DQ181015	DQ181237	UPOL000586
Lycidae	Lyropaeinae	Platerodrilini gen. sp.	DQ181090	DQ181164	DQ181016	DQ181238	UPOL000587
Lycidae	Lyropaeinae	*Macrolibnetis* sp.	DQ181050	DQ181124	DQ180976	DQ181198	UPOL000L21
Lycidae	Lyropaeinae	*Pendola* sp.	DQ181058	DQ181132	DQ180984	DQ181206	UPOL000M45
Lycidae	Lyropaeinae	*Horakiella emasensis*	DQ181110	DQ181184	DQ181036	DQ181258	UPOL001043
Lycidae	Lyropaeinae	Platerodrilini gen. sp.	KF625700	KF626286	KF625989	KF625398	UPOL001371
Lycidae	Lyropaeinae	Platerodrilini gen. sp.	KF625701	KF626287	KF625990	KF625399	UPOL001372
Lycidae	Lyropaeinae	Platerodrilini gen. sp.	KF625702	KF626288	KF625991	KF625400	UPOL001373
Lycidae	Lyropaeinae	Platerodrilini gen. sp.	KF625703	KF626289	KF625992	KF625401	UPOL001374
Lycidae	Lyropaeinae	Platerodrilini gen. sp.	KF625704	KF626290	KF625993	KF625402	UPOL001376
Lycidae	Lyropaeinae	Platerodrilini gen. sp.	KF625705	KF626291	KF625994	-	UPOL001377
Lycidae	Lyropaeinae	Platerodrilini gen. sp.	KF625706	KF626292	KF625995	-	UPOL001378
Lycidae	Lyropaeinae	Platerodrilini gen. sp.	KF625707	KF626293	KF625996	KF625403	UPOL001379
Lycidae	Lyropaeinae	Platerodrilini gen. sp.	KF625708	KF626294	KF625997	KF625404	UPOL001380
Lycidae	Lyropaeinae	Platerodrilini gen. sp.	KF625709	KF626295	KF625998	-	UPOL001382
Lycidae	Lyropaeinae	Platerodrilini gen. sp.	KF625710	KF626296	-	-	UPOL001384
Lycidae	Lyropaeinae	Platerodrilini gen. sp.	KF625711	KF626297	KF625999	KF625405	UPOL001385
Lycidae	Lyropaeinae	Platerodrilini gen. sp.	KF625712	KF626298	KF626000	-	UPOL001387
Lycidae	Lyropaeinae	Platerodrilini gen. sp.	KF625713	KF626299	KF626001	KF625406	UPOL001388
Lycidae	Ateliinae	*Dilophotes* sp.	DQ181066	DQ181140	DQ180992	DQ181214	UPOL000244
Lycidae	Ateliinae	*Scarelus* sp.	DQ181046	DQ181120	DQ180972	DQ181194	UPOL000L15
Lycidae	Ateliinae	*Scarelus* sp.	DQ181085	DQ181159	DQ181011	DQ181233	UPOL000582
Lycidae	Ateliinae	*Scarelus* sp.	DQ181086	DQ181160	DQ181012	DQ181234	UPOL000583
Lycidae	Lycinae	*Dihammatus* sp.	DQ181043	DQ181117	DQ180969	DQ181191	UPOL000L12
Lycidae	Lycinae	*Dihammatus* sp.	DQ181103	DQ181177	DQ181029	DQ181251	UPOL001001
Lycidae	Lycinae	*Dihammatus* sp.	DQ181106	DQ181180	DQ181032	DQ181254	UPOL001009

Lycidae	Lycinae	*Dihammatus* sp.	DQ181108	DQ181182	DQ181034	DQ181256	UPOL001017
Lycidae	Lycinae	*Eurrhacus* sp	DQ181056	DQ181130	DQ180982	DQ181204	UPOL000M43
Lycidae	Lycinae	*Conderis signicollis*	DQ181062	DQ181136	DQ180988	DQ181210	UPOL000194
Lycidae	Lycinae	*Conderis rufohumeralis*	DQ181084	DQ181158	DQ181010	DQ181232	UPOL000581
Lycidae	Lycinae	*Conderis* sp.	DQ350139	DQ350138	DQ350141	DQ350140	UPOL000M42
Lycidae	Lycinae	*Plateros* sp.	DQ181044	DQ181118	DQ180970	DQ181192	UPOL000L13
Lycidae	Lycinae	*Plateros* sp.	DQ181059	DQ181133	DQ180985	DQ181207	UPOL000031
Lycidae	Lycinae	*Plateros* sp.	DQ181065	DQ181139	DQ180991	DQ181213	UPOL000243
Lycidae	Lycinae	*Plateros* sp.	DQ181067	DQ181141	DQ180993	DQ181215	UPOL000303
Lycidae	Lycinae	*Plateros* sp.	DQ181109	DQ181183	DQ181035	DQ181257	UPOL001031
Lycidae	Lycinae	*Macrolycus* sp.	DQ181049	DQ181123	DQ180975	DQ181197	UPOL000L18
Lycidae	Lycinae	*Macrolycus* sp.	DQ181102	DQ181176	DQ181028	DQ181250	UPOL000828
Lycidae	Lycinae	*Thonalmus sinuaticostis*	DQ181093	DQ181167	DQ181019	DQ181241	UPOL000594
Lycidae	Lycinae	*Thonalmus hubbardi*	DQ181094	DQ181168	DQ181020	DQ181242	UPOL000595
Lycidae	Lycinae	*Lyponia nigrohumeralis*	DQ181048	DQ181122	DQ180974	DQ181196	UPOL000L17
Lycidae	Lycinae	*Lyponia delicatula*	DQ181099	DQ181173	DQ181025	DQ181247	UPOL000815
Lycidae	Lycinae	*Lyponia* sp.	DQ181100	DQ181174	DQ181026	DQ181248	UPOL000816
Lycidae	Lycinae	*Lyponia quadricollis*	DQ181101	DQ181175	DQ181027	DQ181249	UPOL000817
Lycidae	Lycinae	*Lycus* sp.	DQ181039	DQ181113	DQ180965	DQ181187	UPOL000L03
Lycidae	Lycinae	*Lycostomus* sp.	DQ181055	DQ181129	DQ180981	DQ181203	UPOL000L27
Lycidae	Lycinae	*Calopteron* sp.	DQ181053	DQ181127	DQ180979	DQ181201	UPOL000L25
Lycidae	Lycinae	*Idiopteron biplagiatum*	DQ181057	DQ181131	DQ180983	DQ181205	UPOL000M44
Lycidae	Lycinae	Lycinae gen. sp.	DQ181092	DQ181166	DQ181018	DQ181240	UPOL000592
Lycidae	Lycinae	*Metapteronsp* sp.	AF451946	DQ198757	-	DQ198588	BMNH679218
Lycidae	Lycinae	*Cautires* sp.	DQ181045	DQ181119	DQ180971	DQ181193	UPOL000L14
Lycidae	Lycinae	*Cautires* sp.	DQ181041	DQ181115	DQ180967	DQ181189	UPOL000L06
Lycidae	Lycinae	*Metriorrhynchus lineatus*	DQ181040	DQ181114	DQ180966	DQ181188	UPOL000L05
Lycidae	Lycinae	*Microtrichalus* sp.	DQ181052	DQ181126	DQ180978	DQ181200	UPOL000L23
Lycidae	Lycinae	*Leptotrichalus* sp.	DQ181064	DQ181138	DQ180990	DQ181212	UPOL000208
Lycidae	Lycinae	*Calochromus* sp.	DQ181047	DQ181121	DQ180973	DQ181195	UPOL000L16
Lycidae	Lycinae	*Calochromus* sp.	DQ181060	DQ181134	DQ180986	DQ181208	UPOL000033
Lycidae	Lycinae	*Calochromus* sp.	DQ181068	DQ181142	DQ180994	DQ181216	UPOL000347
Lycidae	Lycinae	*Platycis minutus*	DQ181069	DQ181143	DQ180995	DQ181217	UPOL000348
Lycidae	Lycinae	*Platycis nasutus*	DQ181079	DQ181153	DQ181005	DQ181227	UPOL000576
Lycidae	Lycinae	*Konoplatycis otome*	DQ181078	DQ181152	DQ181004	DQ181226	UPOL000575
Lycidae	Lycinae	*Lopheros* sp.	DQ181080	DQ181154	DQ181006	DQ181228	UPOL000577
Lycidae	Lycinae	*Lopheros* sp.	DQ181081	DQ181155	DQ181007	DQ181229	UPOL000578
Lycidae	Lycinae	*Eropterus nothus*	DQ181082	DQ181156	DQ181008	DQ181230	UPOL000579
Lycidae	Lycinae	*Eropterus* sp.	DQ181083	DQ181157	DQ181009	DQ181231	UPOL000580

Lycidae	Lycinae	*Flagrax* sp.	DQ181054	DQ181128	DQ180980	DQ181202	UPOL000L26
Lycidae	Lycinae	*Plateros* sp.	KF625685	KF626271	KF625975	KF625384	UPOLRK0377
Lycidae	Lycinae	*Platycis cosnardi*	KF625695	KF626281	KF625984	KF625393	UPOL001286
Lycidae	Lycinae	*Platycis* sp.	KF625696	KF626282	KF625985	KF625394	UPOL001365
Lycidae	Lycinae	*Platycis* sp.	KF625697	KF626283	KF625986	KF625395	UPOL001366
Omalisidae	Omalisinae	*Omalisus sanguinipennis*	HQ333835	-	HQ333741	-	UPOLRK0083
Omalisidae	Omalisinae	*Omalisus fontisbellaquei*	AF451948	DQ198749	DQ198658	DQ198580	UPOL000377
Omalisidae	Omalisinae	*Phaeopterus unicolor*	-	-	-	KJ909286	UPOLRK0092
Omalisidae	Thilmaninae	*Paradrilus opacus*	KJ909284	KJ909285	-	KJ909287	UPOLRK0626
Omalisidae	Euanominae	*Pseudeuanoma* sp.	HQ333832	KF626300	HQ333738	HQ334011	UPOLRK0079
Omalisidae	Euanominae	*Pseudeuanoma* sp.	HQ333833	KF626301	-	HQ334012	UPOLRK0080
Omethidae	Driloniinae	*Drilonius striatulus*	KF625527	KF626128	KF625830	KF625227	UPOL001272
Omethidae	Driloniinae	*Drilonius* sp.	DQ100502	DQ198750	DQ198659	DQ198581	UPOL000M26
Omethidae	Driloniinae	*Drilonius* sp.	KF294764	KF294770	KF294758	KF294777	UPOL001273
Omethidae	Driloniinae	*Drilonius* sp.	KF294765	KF294771	KF294759	KF294778	UPOLRK0134
Omethidae	Driloniinae	*Drilonius* sp.	KF625528	KF626129	KF625831	KF625228	UPOL001274
Omethidae	Driloniinae	*Drilonius* sp.	KF625523	KF626124	KF625832	KF625223	UPOLRK0132
Omethidae	Driloniinae	*Drilonius* sp.	KF625524	KF626125	KF625833	KF625224	UPOLRK0135
Omethidae	Driloniinae	*Drilonius* sp.	KF625525	KF626126	KF625834	KF625225	UPOLRK0136
Omethidae	Driloniinae	*Drilonius* sp.	KF625526	KF626127	KF625835	KF625226	UPOLRK0362
Omethidae	Matheteinae	*Ginglymocladus* sp.	KF625530	KF626131	KF625829	KF625230	UPOL001341
Omethidae	Omethinae	*Troglomethes leechi*	KF625529	KF626130	KF625828	KF625229	UPOL001340
Omethidae	Telegeusinae	*Telegeusis nubifer*	DQ100503	DQ198751	DQ198660	DQ198582	UPOL000321
Omethidae	Telegeusinae	gen. sp.	KF625531	KF626132	-	KF625231	UPOLRK0360
Omethidae	Telegeusinae	gen. sp.	-	KF626318	-	KF625425	UPOL001345
Phengodidae	Phengodinae	*Phengodes* sp.	DQ100504	DQ198752	DQ198661	DQ198583	UPOL000M29
Phengodidae	Phengodinae	*Phengodes* sp.	KF625725	KF626312	KF626011	KF625418	UPOL001238
Phengodidae	Phengodinae	*Phengodes* sp.	KF625726	KF626313	KF626012	KF625419	UPOL001241
Phengodidae	Phengodinae	*Phengodes* sp.	KF625723	KF626310	KF626009	KF625416	UPOLRK0366
Phengodidae		gen. sp.	KF625727	KF626314	KF626013	KF625420	UPOL001243
Phengodidae		gen. sp.	KF625728	KF626315	KF626014	KF625421	UPOL001245
Phengodidae		gen. sp.	KF625729	KF626316	KF626015	KF625422	UPOL001246
Phengodidae		gen. sp.	KF625730	KF626317	KF626016	KF625423	UPOL001247
Phengodidae		gen. sp.	KF625731	-	KF626017	KF625424	UPOL001343
Phengodidae		gen. sp.	KF625732	KF626319	-	KF625426	UPOL001346
Phengodidae		gen. sp.	KF625733	-	KF626018	KF625427	UPOL001348
Phengodidae		gen. sp.	KF625734	-	-	KF625428	UPOL001349
Phengodidae		gen. sp.	KF625735	-	KF626019	KF625429	UPOL001350
Phengodidae		gen. sp.	KF625736	KF626320	KF626020	KF625430	UPOL001351

Phengodidae	gen. sp.	KF625737	-	KF626021	-	UPOL001353
Phengodidae	gen. sp.	KF625738	-	KF626022	KF625431	UPOL001354
Phengodidae	gen. sp.	KF625719	KF626306	KF626007	KF625412	UPOLRK0361
Phengodidae	gen. sp.	KF625720	KF626307	-	KF625413	UPOLRK0363
Phengodidae	gen. sp.	KF625721	KF626308	KF626008	KF625414	UPOLRK0364
Phengodidae	gen. sp.	KF625722	KF626309	-	KF625415	UPOLRK0365
Phengodidae	gen. sp.	KF625724	KF626311	KF626010	KF625417	UPOLRK0368
Rhagophthalmidae	*Bicladodrilus* sp.	DQ100507	DQ198755	DQ198664	DQ198586	UPOL000M35
Rhagophthalmidae	*Mimoochotyra* sp.	DQ100505	DQ198753	DQ198662	DQ198584	UPOL000M30
Rhagophthalmidae	*Rhagophthalmus ohbai*	AB298864	-	NC_010964	NC_01096	471225
Rhagophthalmidae	*Rhagophthalmus* sp.	DQ100508	DQ198756	DQ198665	DQ198587	UPOL000155
Rhagophthalmidae	gen. sp.	KF625717	KF626305	KF626005	KF625410	UPOL001359
Rhagophthalmidae	gen. sp.	KF625718	-	KF626006	KF625411	UPOL001363
Rhagophthalmidae	gen. sp.	KF625715	KF626303	KF626003	KF625408	UPOLRK0088
Rhagophthalmidae	gen. sp.	KF625716	KF626304	KF626004	KF625409	UPOLRK0370
Rhagophthalmidae	gen. sp.	DQ100506	DQ198754	DQ198663	DQ198585	UPOL000M31

				Genus species			
				Tetraphalerus bruchi		6	
				Priacma serrata	6		
				Hydroscapha granulum	66		
				Sphaerius			
				Trachypachus			
			holmbergi	*Brachinus crepitans*	6		
				Abax parallelepipedus	6		
				Scarites buparius		6	
				Acilius	6	6	
				Hygrobia hermanni	6	6	6
				Noterus	6	6	
				Aspidytes niobe			
				Macrogyrus oblongus		66	
				Haliplus	6		
				Contacyphon			
				Clambus *Scirtidae*			
						6	
				Nosodendron fasciculare			6
				Rhinorhipus tamborinensis	6		66
				Chrysochroa fulgidissima	6		
				Agrilus		6	
				Acmaeodera		6	
				Dascillus		66	
				cervinus Byrrhus		6	
				Dryops	6	6	
						6	
				Eulichas		6	
				Heterocerus fenestratus			
				Byrrhinus		6	
				Ptilodactyla		6	
				Eurypogon		6	
				Chauliognathus opacus	6		
				Drilus flavescens			
				Pyrophorus divergens		6	
				Teslasena femoralis			
				Athous	6	6	
				Eucnemidae			
				Iberobaenia		6	
				Pyrocoelia rufa			
				Aquatica leii	66	6	6
				Lycus dentipes			
				Platerodrilus			

				Genus species		
				Platerodrilus	6	6
				Omalisus fontisbellaquei		
				Drilonius striatulus		
				Brasilocerus		
				Phrixothrix hirtus		
				Rhagophthalmus ohbai	6	
				Trixagus		
				Hydrochus	6	
				Amphiops globus	6	
				Helochares	6	6
				Necrophila americana	6	6
				Astenus lyonessius	6 6	
				Dacrila fallax	6	6
				Scaphidium quadrimaculatum	6	
				Olophrum piceum	6	6 6
				Bolboceratex	6	
				Glaresis		66
				Nargus velox	66	
				Dorcus	6	6
				Cheirotonus jansoni		
				Osmoderma opicum	6	
				Attagenus hottentotus		
				Epicauta aptera		6
				Mordella atrata		
				Scraptia		
				Tenebrio molitor	6	
				Cucujus clavipes	6	6
					6 6 6	6
				Aspidiphorus	6	
				Dastarcus helophoroides		
				Cerylon histeroides		
				Propylea japonica	66	
				Trichodes sinae	6	6
				Chaetosoma scaritides		
				Curculio davidi		6
				Agasicles hygrophila		
				Acanthoscelides obtectus	6	6
				Gastrolina thoracica	6	6
				Massicus raddei	6	
				Spiniphilus spinicornis		

Supplementary Table S3. The list of taxa included in the *LSU* rRNA, *SSU* rRNA, and six nuclear protein coding genes.

Suborder<br>Series<br>Superfamily<br>Family	Subfamily	Species	Gene:<br>18S rRNA	28S rRNA	EF1-a	CAD	AK	wingless	PEPCK	AS	DNA Voucher
**ADEPHAGA**											
Carabidae	Brachininae	*Brachinus* sp.	KP419023	KP419376	KP677641	KP812769	KP812194	KP813354	KP813093	KP812490	BT0086
Carabidae	Carabinae	*Calosoma scrutator*	KP419030	KP419383	KP677645	EU677530	EU681831	EU677661	KP813099	-	BT0076
Carabidae	Cicindelinae	*Cicindela sexguttata*	KP419048	KP419402	KP677659	KP812795	KP812218	KP813379	-	KP812511	DDM0004
Carabidae	Harpalinae	*Pterostichus melanarius*	KP419252	KP419605	KP677819	KP812985	-	KP813551	KP813255	KP812665	BT0063
Dytiscidae	Agabinae	*Agabus* sp.	KP418992	KP419343	KP677613	KP812739	KP812163	KP813322	KP813068	KP812466	BT0154
Dytiscidae	Laccophilinae	*Laccophilus pictus*	KP419139	KP419495	KP677731	KP812883	KP812298	KP813456	KP813177	KP812582	BT0073
Gyrinidae	Gyrininae	*Gyrinus* sp.	KP419114	KP419468	KP677712	KP812859	KP812274	KP813435	KP813158	KP812563	DNA1586
Haliplidae		*Haliplus* sp.	KP419115	KP419469	KP677713	KP812860	KP812275	KP813436	-	KP812564	BT0159
Haliplidae	Peltodytinae	*Peltodytes* sp.	KP419221	KP419578	-	KP812957	KP812365	KP813526	KP813235	KP812643	BT0096, BT0065
Hygrobiidae		*Hygrobia* sp.	KP419129	KP419484	KP677723	KP812874	KP812288	KP813447	KP813170	KP812577	BT0156
Meruidae		*Meru phyllisae*	KP419165	-	KP677753	KP812908	KP812319	KP813481	-	KP812602	BT0145
Noteridae	Noterinae	*Noterus clavicornis*	KP419190	KP419546	KP677774	KP812928	KP812339	-	KP813212	KP812621	BT0124
Rhysodidae		*Omoglymmius hamatus*	KP419200	KP419557	KP677779	KP812939	KP812350	KP813509	KP813219	KP812629	BT0067
Trachypachidae		*Trachypachus holmbergi*	KP419320	KP419675	KP677873	KP813049	KP812450	KP813611	KP813306	KP812720	BT0132
**ARCHOSTEMATA**											
Cupedidae	Cupedinae	*Cupes capitatus*	KP419064	KP419418	KP677674	KP812810	KP812232	KP813392	KP813124	-	BT0098
Cupedidae	Cupedinae	*Tenomerga cinerea*	EU797417	KP419665	KP677864	KP813040	KP812440	KP813601	KP813297	-	BT0022
Cupedidae	Priacminae	*Priacma serrata*	EU797411	KP419595	AGRH: C27039	EU677524	EU677504	EU677656	AGRH: C3016	EU677477	BT0018, Genome
Micromalthidae		*Micromalthus debilis*	KP419171	KP419526	KP677758	KP812914	KP812325	-	KP813201	KP812607	BT0112
Ommatidae	Tetraphalerinae	*Tetraphalerus bruchi*	KP419314	KP419668	KP677867	KP813043	KP812443	KP813604	KP813300	-	BT0099, BT0025
**MYXOPHAGA**											
Hydroscaphidae		*Hydroscapha natans*	KP419128	KP419483	KP677722	KP812873	KP812287	KP813446	KP813169	-	BT0092
Lepiceridae		*Lepicerus* sp.	KP419146	KP419503	KP677738	KP812891	-	KP813464	KP813182	KP812590	BT0106
Sphaerusidae		*Sphaerius* sp.	KP419294	KP419646	KP677849	KP813023	-	KP813586	KP813282	KP812698	BT0075
Torridincolidae	Deleveinae	*Delevea* sp.	KP419074	KP419427	KP677682	-	KP812240	KP813400	KP813129	KP812533	BT0142
Torridincolidae	Torridincolinae	*Torridincola* sp.	KP419318	KP419672	KP677871	KP813047	KP812447	KP813608	KP813304	KP812718	BT0146
**POLYPHAGA**											

**Scirtiformia**											
Scirtoidea											
Clambidae	Clambinae	*Loricaster rotundus*	-	KP419508	-	KP812895	KP812308	KP813468	KP813186	-	MSC1308
Decliniidae		*Declinia relicta*	KP419073	KP419426	KP677681	KP812818	-	KP813399	KP813128	KP812532	BT0152
Eucinetidae		*Eucinetus* sp.	KP419099	KP419453	KP677701	KP812845	-	KP813423	KP813148	KP812554	DDM0816, BT0058, BT0117
Eucinetidae		*Noteucinetus* sp.	KP419191	KP419547	-	KP812929	KP812340	KP813499	KP813213	KP812622	BT0108
Eucinetidae		*Nycteus infumatus*	KP419194	KP419551	KP677775	KP812933	KP812343	KP813503	KP813216	KP812625	MSC1286
Scirtidae	Scirtinae	*Veronatus* sp.	KP419331	KP419686	-	KP813059	KP812457	-	KP813312	KP812727	DDM0660
Scirtidae	Scirtinae	*Cyphon* sp.	KP419069	KP419422	KP677678	KP812815	KP812237	KP813396	KP813126	KP812529	BT0013
Scirtidae	Scirtinae	*Elodes apicalis*	KP419091	KP419445	KP677695	KP812837	KP812254	KP813416	KP813142	KP812548	BT0121
Scirtidae	Scirtinae	*Prionocyphon* sp.	KP419243	KP419599	-	KP812977	KP812383	KP813544	-	-	BT0126
**Derodontiformia**											
Derodontoidea											
Derodontidae	Derodontinae	*Derodontus* sp.	KP419078	KP419431	KP677685	KP812822	-	KP813403	KP813131	KP812535	BT0162
Derodontidae	Laricobiinae	*Laricobius nigrinus*	KP419143	KP419500	KP677735	KP812888	KP812303	KP813461	KP813179	KP812587	DDM0620, DDM0248
Derodontidae	Laricobiinae	*Nothoderodontus* sp.	-	KP419548	-	KP812930	KP812341	KP813500	KP813214	KP812623	DDM0152
Jacobsoniidae		*Derolathrus* sp.	KP419079	KP419432	-	KP812823	KP812154	KP813315	KP813132	KP812536	DDM0611, BT0184"
Jacobsoniidae		*Saphophagus minutus*	KP419278	KP419631	-	KP813009	KP812410	-	KP813271	-	DDM0250
Nosodendridae		*N. unicolor, N. californica*	KP419187	KP419543	KP677771	KP812926	KP812338	KP813497	KP813210	KP812619	MSC1292
**Elateriformia**											
Rhinorhipoidea											
Rhinorhipidae		*Rhinorhipus tamborinensis*	AB123456	AB123456	AB123456	AB123456	AB123456	AB123456	AB123456	AB123456	A00672
Buprestoidea											
Buprestidae	Agrilinae	*Agrilus* sp.	AJ810746	KP419345	KP677615	KP812741	KP812165	KP813324	KP813070	KP812468	DDM0390
Buprestidae	Buprestinae	*Buprestis adjecta*	KP418979	KP419378	-	KP812773	-	-	-	-	BUP0046
Buprestidae	Julodinae	Julodis sulcicollis	KP418981	KP419493	KP677729	KP812881	KP812296	KP813454	-	-	BT0178
Buprestidae	Polycestinae	*Acmaeodera* sp.	KP418977	KP419338	KP677610	KP812734	KP812159	KP813318	-	KP812462	DDM0391
Schizopodidae	Schizopodinae	*Schizopus laetus*	KP419282	KP419635	-	KP813011	KP812412	KP813575	-	KP812688	BUP0272
Dascilloidea											
Dascillidae	Dascillinae	*Dascillus* sp.	KP419072	KP419425	KP677680	KP812817	KP812239	KP813398	KP813127	KP812531	DDM0608
Dascillidae	Karumiinae	*Anorus piceus*	KP419003	KP419355	KP677625	KP812749	KP812175	KP813335	KP813078	KP812476	MSC1281, BT0127
Rhipiceridae	Rhipicerinae	*Sandalus* sp.	KP419277	KP419630	-	KP813008	KP812409	KP813572	KP813270	KP812686	BTO110
Rhipiceridae	Rhipicerinae	*Rhipicera* sp.	KP419264	KP419617	-	-	-	-	-	-	DDM2261
Byrrhoidea											
Byrrhidae	Byrrhinae	*Simplocaria semistriata*	KP419288	KP419640	KP677845	KP813017	KP812418	KP813581	-	KP812693	BT0017
Byrrhidae	Syncalyptinae	*Microchaetes* sp.	KP419170	KP419525	KP677757	KP812913	KP812324	KP813486	KP813200	KP812606	DDM0784
Callirhipidae		*Callirhipis* sp.	KP419028	KP419381	-	KP812775	KP812200	KP813359	KP813097	KP812495	CO129, DDM1305
Chelonariidae		*Chelonarium* sp.	KP419045	KP419399	KP677656	KP812792	-	KP813376	KP813111	KP812510	DDM0617

Cneoglossidae		*Cneoglossa* sp.	KP419053	KP419407	KP677663	KP812799	KP812223	KP813382	KP813116	KP812515	DDM1299
Dryopidae		*Helichus* sp.	KP419117	KP419471	KP677715	KP812862	-	KP813438	KP813160	KP812566	BT0051
Elmidae	Elminae	*Optioservus* sp.	KP419202	KP419558	KP677781	KP812941	KP812352	KP813511	KP813221	KP812630	DDM0238
Elmidae	Larainae	*Lara avara*	KP419142	KP419499	KP677734	KP812887	KP812302	KP813460	-	KP812586	MSC1288
Eulichadidae		*Stenocolus scutellaris*	KP419302	KP419654	-	KP813030	KP812430	KP813592	KP813288	KP812705	DDM1010
Heteroceridae	Heterocerinae	*Heterocerus fenestratus*	KP419120	KP419475	KP677719	KP812866	KP812280	KP813441	KP813163	KP812570	BT0115, BT0116
Limnichidae	Limnichinae	*Byrrhinus* sp.	KP419026	KP419379	KP677643	-	KP812198	KP813357	KP813096	KP812494	DDM0393
Lutrochidae		*Lutrochus arizonicus*	KP419152	KP419510	KP677742	KP812897	KP812310	KP813470	KP813188	KP812594	DDM0233
Psephenidae	Eubrianacinae	*Eubrianax edwardsi*	KP419098	KP419452	-	KP812844	KP812260	KP813422	KP813147	KP812553	BT0034
Psephenidae	Psepheninae	*Psephenus minckleyi*	KP419251	-	KP677818	KP812984	KP812389	KP813550	KP813254	KP812664	DDM0228
Ptilodactylidae	Anchytarsinae	*Anchyteis velutina*	KP418999	KP419351	KP677621	-	KP812171	KP813331	-	-	DDM0809
Ptilodactylidae	Ptilodactylinae	*Podabrocephalus* sp.	KP419237	KP419593	KP677808	KP812972	KP812378	KP813540	KP813244	-	MSC1355
Ptilodactylidae	Ptilodactylinae	*Ptilodactyla* sp.	KP419254	KP419607	KP677821	KP812987	KP812391	-	KP813256	KP812667	DDM0396
Elateroidea											
Artematopidae	Artematopodinae	*Macrop. testaceipennis*	KP419156	KP419513	KP677745	KP812900	KP812313	KP813473	KP813191	KP812595	MSC1296
Brachypsectridae		*Brachypsectra fulva*	KP419025	-	-	KP812771	KP812196	KP813355	KP813095	KP812492	DDM1028
Cantharidae	Chauliognathinae	*C. opacus*	KP419044	KP419398	KP677655	KP812791	KP812216	KP813375	KP813110	KP812509	BT0055
Cantharidae	Malthininae	*Malthodes* sp.	KP419157	KP419514	KP677746	KP812901	KP812314	KP813474	KP813192	KP812596	DDM0621
Cantharidae	Silinae	*Silis* sp.	KP419287	KP419639	KP677844	KP813016	KP812417	KP813580	KP813277	KP812692	MSC1278
Cerophytidae		*Cerophytum* sp.	KP419040	KP419394	-	KP812788	KP812213	KP813372	-	KP812507	BT0107
Elateridae	Agrypninae	*Drilus* sp.	KP419086	KP419440	KP677691	KP812832	-	KP813411	KP813139	KP812544	DDM0403
Elateridae	Cebrioninae	*Selonodon floridensis*	KP419285	KP419637	KP677842	KP813014	KP812415	KP813578	KP813275	KP812690	DDM0602
Elateridae	Elaterinae	*Ampedus* sp.	KP418996	KP419348	KP677618	KP812745	KP812169	KP813328	KP813074	KP812472	DDM0618
Elateridae	Elaterinae	*Cardiophorus* sp.	KP419033	KP419386	-	KP812780	KP812205	KP813364	KP813102	KP812499	CO050
Elateridae	Lissominae	*Lissomus* sp.	KP419149	KP419506	-	KP812893	-	KP813466	KP813184	KP812592	DDM0615
Eucnemidae	Anischiinae	*Anischia bicolor*	KP419001	KP419353	KP677623	KP812747	KP812173	KP813333	KP813076	KP812474	DDM0659
Eucnemidae	Malasinae	*Melasis buprestoides*	KP419161	KP419517	KP677749	KP812904	-	-	KP813194	KP812599	DDM0401
Lampyridae	Lampyrinae	*Ellychnia californica*	KP419090	KP419444	-	KP812836	KP812253	KP813415	KP813141	KP812547	BT0033
Lampyridae	Lampyrinae	*Pyrocoelia* sp.	KP419258	KP419611	KP677824	KP812991	-	KP813556	KP813259	KP812670	CO428, BT0094
Lampyridae	Ototretinae	*Ototretadrilus* sp.	KP419207	KP419564	KP677785	-	-	-	-	-	DDM0399
Lampyridae	Photurinae	*Pterotus obscuripennis*	KP419253	KP419606	KP677820	KP812986	KP812390	KP813552	-	KP812666	MSC1287
Lycidae	Dictyopterinae	*Dictyoptera* sp.	KP419081	KP419434	KP677686	KP812825	KP812245	KP813405	KP813133	KP812538	DDM0001
Lycidae	Lycinae	*Lycus* sp.	KP419154	KP419512	KP677744	KP812899	KP812312	KP813472	KP813190	-	CO253
Omalisidae	Omalisinae	*Omalisus fontisbellaquei*	KP419199	KP419556	KP677778	KP812938	KP812349	KP813508	KP813218	KP812628	DDM0408
Omethidae	Matheteinae	*Matheteus theveneti*	KP419158	KP419515	KP677747	KP812902	KP812315	KP813475	KP813193	KP812597	DDM0948
Phengodidae	Mastinocerinae	*Cenophengus debilis*	KP419037	KP419390	KP677650	KP812784	KP812209	KP813368	KP813105	KP812503	DDM0613
Phengodidae	Phengodinae	*Phengodes* sp.	KP419227	KP419584	KP677799	KP812962	KP812370	KP813531	KP813240	KP812648	DDM0008, BT0030
Rhagophthalmidae		*Rhagophthalmus ohbai*	KP419262	KP419615	KP677827	KP812995	KP812398	KP813560	-	KP812673	CO409
Telegeusidae		*Telegeusis* sp.	DQ100503	KP419663	KP677862	KP813038	KP812438	KP813599	KP813295	-	BT0095, BT0023
Throscidae		*Aulonothroscus* sp.	KP419017	KP419370	KP677637	KP812763	KP812188	KP813348	KP813089	KP812486	DDM0394

**Staphyliniformia**											
Hydrophiloidea											
Hydrophilidae	Hydrophilinae	*Tropisternus* sp.	KP419325	KP419680	KP677876	KP813054	KP812453	KP813615	KP813309	KP812723	DDM0011, CO166
Hydrophilidae	Sphaeridiinae	*Andotypus ashworthi*	KP419000	KP419352	KP677622	-	KP812172	KP813332	-	-	DDM0032
Staphylinoidea											
Silphidae	Nicrophorinae	*Nicrophorus tomentosus*	KP419186	KP419542	KP677770	KP812925	KP812337	KP813496	KP813209	KP812618	DDM0054
Silphidae	Silphinae	*Necrophila americana*	KP419182	KP419537	KP677767	KP812922	KP812334	KP813493	KP813207	KP812615	DDM0046
Staphylinidae	Glypholomatinae	*Glypholoma pecki*	KP419112	KP419466	KP677711	KP812857	KP812272	KP813433	KP813156	-	DDM0056
Staphylinidae	Omaliinae	*Paraphloeost. gayndahensis*	KP419215	KP419572	-	KP812953	KP812361	KP813522	KP813231	KP812639	DDM0780
Staphylinidae	Osoriinae	*Renardia cf. nigrella*	KP419260	KP419613	KP677826	KP812993	KP812396	KP813558	KP813261	-	DDM0058
Staphylinidae	Scaphidiinae	*Scaphidium* sp.	KP419280	KP419633	KP677840	KP813010	KP812411	KP813574	KP813273	KP812687	DDM0298
Staphylinidae	Scydmaeninae	*Adrastia* sp.	KP418991	KP419342	-	KP812738	-	KP813321	KP813067	KP812465	DDM0781
Staphylinidae	Scydmaeninae	*Euconnus* sp.	KP419100	KP419454	KP677702	KP812846	-	KP813424	KP813149	-	DDM0049
Staphylinidae	Scydmaeninae	*Palaeostigus bifoveolatus*	KP419210	KP419567	KP677788	KP812949	KP812358	KP813518	KP813227	KP812635	DDM0047
Staphylinidae	Staphylininae	*Creophilus maxillosus*	KP419060	KP419414	KP677670	KP812806	KP812228	KP813388	KP813121	KP812521	DDM0013
Staphylinidae	Tachyporinae	*Leucotachinus luteonitens*	KP419147	KP419504	KP677739	KP812892	KP812306	KP813465	KP813183	KP812591	DDM0057
**Scarabaeiformia**											
Scarabaeoidea											
Scarabaeidae	Aphodiinae	*Aphodius fimetarius*	KP419009	KP419361	KP677630	KP812754	KP812180	-	-	-	MP312
Scarabaeidae	Cetoniinae	*Cetonia aurata*	KP419041	KP419395	-	-	KP812214	-	-	-	S551
Scarabaeidae	Dynastinae	*Dynastes granti*	KP419087	KP419441	KP677692	KP812833	EU677503	KP813412	EU677599	EU677479	BT0056, BT0057, S791
Scarabaeidae	Melolonthinae	*Rhop. magnicornis*, R. sp.	KP419267	KP419620	KP677831	KP812999	KP812402	-	KP813266	KP812677	S359, CO133
Scarabaeidae	Scarabaeinae	*Scarabaeus deludens*	KP419281	KP419634	-	-	-	-	-	-	FO42
Glaresidae		*Glaresis ecostata*	KP419110	KP419464	KP677709	KP812855	-	-	-	-	S7
**Bostrichiformia**											
Bostrichoidea											
Dermestidae	Dermestinae	*Dermestes* sp.	KP419077	KP419430	KP677684	KP812821	KP812243	KP813402	KP813130	KP812534	BT0061
Dermestidae	Megatominae	*Anthrenus ?lepidus*	KP419005	KP419357	KP677626	KP812751	KP812177	KP813337	KP813080	KP812478	BT0123, DDM0374
Dermestidae	Orphilinae	*Orphilus subnitidus*	KP419204	KP419560	KP677783	KP812943	KP812354	KP813513	KP813223	KP812632	MSC1282
Ptinidae	Anobiinae	*Stegobium panaceum*	KP419300	KP419652	KP677854	KP813028	KP812428	KP813590	-	KP812703	DDM0808
Ptinidae	Ptininae	*Ptinus* sp.	KP419255	KP419608	-	KP812988	KP812392	KP813553	-	-	MSC1285
**Cucujiformia**											
Chrysomeloidea											
Chrysomelidae	Cryptocephalinae	*Mylassa rubronotata*	KP419177	KP419532	KP677762	-	-	-	-	-	DDM0388
Chrysomelidae	Cryptocephalinae	*Neochlamisus bebbiana*	KP419184	KP419540	KP677769	KP812924	KP812336	KP813495	-	KP812617	DDM0227
Chrysomelidae	Bruchinae	*Bruchus* spp.	KP419329	KP419684	AY997389	KP812772	KP812197	KP813356	KP813311	KP812493	DDM1310 , DDM0804

Chrysomelidae	Cassidinae	*Cephaloleia belti*	KP419038	KP419391	KP677651	KP812785	KP812210	KP813369	-	KP812504	DDM0041
Chrysomelidae	Chrysomelinae	*Chrysomela vigipunctata*	KP419047	KP419401	KP677658	KP812794	KP812217	KP813378	-	-	DDM0040
Chrysomelidae	Criocerinae	*Crioceris duodecimpunctata*	KP419061	KP419415	KP677671	KP812807	KP812229	KP813389	KP813122	KP812522	DDM0039
Chrysomelidae	Galerucinae	*Diabrotica limitata*	KP419080	KP419433	-	KP812824	KP812244	KP813404	-	KP812537	DDM0801
Tenebrionoidea											
Tenebrionidae	Lagriinae	*Adelium* sp.	KP418989	KP419340	KP677611	KP812736	KP812161	KP813319	KP813065	KP812463	CO135
Tenebrionidae	Lagriinae	*Statira gagatina*	KP419299	KP419651	KP677853	KP813027	KP812427	KP813589	KP813286	KP812702	DDM0943
Tenebrionidae	Pimeliinae	*Coelus ciliatus*	KP419054	KP419408	KP677664	KP812800	KP812224	KP813383	KP813117	KP812516	MSC0597
Tenebrionidae	Tenebrioninae	*Leaus tasmanicus*	KP419144	KP419501	KP677736	KP812889	KP812304	KP813462	KP813180	KP812588	DDM0791
Tenebrionidae	Tenebrioninae	*Tribolium castaneum*	KP419322	KP419677	KP677875	KP813051	XM966707	NM1114350	XM8201535	XM8203131	BT0053, BT0052, Genome
Tenebrionidae	Zolodininae	*Tanylypa morio*	KP419309	KP419661	-	KP813037	KP812437	KP813598	KP813294	KP812711	DDM0793
Curculionoidea											
Curculionidae	Brachycerinae	*Ocladius* sp.	KP419196	KP419553	KP677777	KP812935	KP812346	KP813506	-	KP812626	DDM0537
Curculionidae	Brachycerinae	*Notaris* sp.	KP419189	KP419545	KP677773	-	-	-	-	-	GenBank
Curculionidae	Curculioninae	*Curculio* spp.	KP419065	FJ867675	FJ867861	KP812811	KP812233	KP813393	-	KP812525	DDM0044, DDM0444, DDM0045
Curculionidae	Dryophthorinae	*Sphenophorus brunnipennis*	KP419296	KP419648	KP677850	KP813024	KP812424	-	KP813283	KP812699	DDM0022
Curculionidae	Entiminae	*Naupactus* sp.	KP419179	KP419534	KP677764	-	KP812331	-	-	KP812612	DDM0006
Curculionidae	Molytinae	*Porthetes* sp.	KP419239	HQ883577	HQ883737	HQ883805	HQ883895	-	EU310663	-	DDM0060
Curculionidae	Molytinae	*Sympiezoscelus spencei*	KP419304	KP419656	KP677857	KP813032	KP812432	KP813593	-	KP812707	DDM0112
Curculionidae	Molytinae	*Tranes lyteroides*	KP419321	KP419676	KP677874	KP813050	-	KP813612	-	KP812721	DDM0064
Curculionidae	Platypodinae	*Platypus incompertus*	KP419234	KP419591	KP677805	KP812969	KP812375	KP813538	-	KP812653	DDM0506
Curculionidae	Platypodinae	*Mecopelmus zetecki*	HQ883574	HQ883735	HQ883802	HQ883892	-	-	-	-	KP419160

Species	Ace	alpha-Spec	alphaCOP	AP47	Art1	beta'Cop	BOP1	Bx42	CadN	CAD	calypso	CaP60A	Cdk7	CG11652	CG3999	zip
**Outgroups**																
Neochauliodesorientalis		MG610067	MG609792	MG610376		MG610995			MG612655	MG612344	MG612959	MG613261	MG613607	MG613930	MG614614	MG636843
Ascalohybrissubjacens	MG609513	MG610066	MG609791	MG610375	MG610703		MG611236	MG612087	MG612654	MG612343	MG612958	MG613260		MG613929	MG614613	MG636842
Dichochrysaformosana			MG609790	MG610374	MG610702				MG612653	MG612342	MG612957	MG613259		MG613928	MG614612	MG636841
Myrmeleonsp.		MG610065	MG609789	MG610373	MG610701			MG612086	MG612652	MG612341	MG612956	MG613258			MG614611	MG636840
**Adephaga / Carabidae**																
Dischissussp.		MG610077		MG610388	MG610713			MG612092	MG612665	MG612353	MG612966	MG613271		MG613942		MG636855
Carabussp.	MG609579	MG610141	MG609855	MG610459	MG610788	MG611057	MG611304	MG612144	MG612735	MG612426	MG613029	MG613345	MG613695	MG614017	MG614695	MG636917
Cicindelachinensis	MG609580	MG610142	MG609856	MG610460	MG610789		MG611305		MG612736	MG612427	MG613030	MG613346		MG614018	MG614696	MG636918
Omoglymmiussp.	MG609586	MG610157	MG609867	MG610476	MG610804		MG611316	MG612151	MG612749	MG612441	MG613045	MG613362		MG614035		MG636930
Clivinasp.			MG609903	MG610517	MG610844	MG611088			MG612790	MG612478	MG613082	MG613402		MG614077		MG636968
Pheropsophussp.		MG610193	MG609904	MG610518	MG610845		MG611353		MG612791	MG612479	MG613083	MG613403		MG614078		MG636969
Lebiacoelestis			MG609905	MG610520	MG610847			MG612182	MG612793	MG612481	MG613085	MG613405		MG614080		MG636971
Pentagonicaruficollis	MG609618	MG610195		MG610521	MG610848			MG612183	MG612794	MG612482	MG613086	MG613406	MG613743	MG614081		MG636972
Elaphrussp.	MG609639	MG610216		MG610547	MG610875		MG611378	MG612202	MG612815	MG612504		MG613433		MG614109		MG636995
gen.sp.	MG609641	MG610218	MG609930	MG610549	MG610877						MG613108	MG613435		MG614110	MG614771	
**Dytiscidae**																
Agabussp.				MG610468	MG610796				MG612741	MG612433	MG613037	MG613353	MG613700	MG614026		MG636923
Laccophilusdifficilis	MG609584	MG610149	MG609863	MG610469	MG610797	MG611062	MG611310	MG612149	MG612742	MG612434	MG613038	MG613354		MG614027		MG636924
Eretesgriseus		MG610150		MG610470	MG610798				MG612743	MG612435	MG613039	MG613355		MG614028		MG636925
Rhantussuturalis		MG610151	MG609864	MG610471	MG610799				MG612744	MG612436	MG613040	MG613356		MG614029		MG636926
Hyphydrusdetectus		MG610152		MG610472	MG610800		MG611311		MG612745	MG612437	MG613041	MG613357		MG614030		
**Gyrinidae**																
Orectochilussp.		MG610095	MG609815	MG610407	MG610733	MG611020	MG611260		MG612685	MG612372		MG613291	MG613638			MG636872
**Haliplidae**																
Peltodytessp.			MG609868	MG610477	MG610805		MG611317	MG612152	MG612750	MG612442	MG613046	MG613363		MG614036		MG636931
**Noteridae**																
gen.sp.	MG609418	MG609971	MG609684			MG610912		MG612005	MG612553	MG612244	MG612864	MG613153	MG613487	MG613818	MG614500	MG636749
Canthydrussp.		MG610153	MG609865	MG610473		MG611063	MG611312				MG613042	MG613358		MG614031	MG614703	
**Archostemata/ Cupedidae**																
Tenomergasp.		MG610155			MG610802		MG611314	MG612150	MG612747	MG612439	MG613044	MG613360		MG614033	MG614704	MG636928
**Myxophaga / Torridincolidae**																
Satoniusschoenmanni		MG610154		MG610474	MG610801	MG611064	MG611313		MG612746	MG612438	MG613043	MG613359		MG614032		MG636927
**Bostrichoidea /Bostrichidae**																
Lyctussp.	MG609438		MG609709	MG610291	MG610618		MG611160	MG612026	MG612579	MG612268	MG612879	MG613178	MG613515	MG613842	MG614525	MG636771
Polycaonsp.				MG610402	MG610728	MG611016			MG612680	MG612368		MG613286	MG613633	MG613957	MG614637	MG636868
**Dermestidae**																
Dermestessp.	MG609453	MG610002	MG609725	MG610307	MG610635	MG610940	MG611170	MG612036	MG612593	MG612279	MG612895	MG613190	MG613534	MG613858	MG614541	MG636782
Evorineasp.		MG610003		MG610308	MG610636		MG611171	MG612037	MG612594	MG612280	MG612896	MG613191	MG613535	MG613859	MG614542	
Orphinussp.	MG609593	MG610164	MG609874	MG610483	MG610811	MG611070	MG611324	MG612157	MG612757	MG612449	MG613051	MG613370	MG613708	MG614043	MG614712	MG636938
**Ptinidae**																
Ptinussp.			MG609688	MG610269	MG610599	MG610916	MG611142			MG612248	MG612868		MG613492		MG614504	
Dorcatomasp.		MG609975		MG610270			MG611143	MG612009	MG612558	MG612249			MG613493		MG614505	MG636753
Ptinussp.			MG609876	MG610485	MG610813	MG611072			MG612759	MG612451			MG613710		MG614714	
Hedobiasp.	MG609633		MG609922	MG610540	MG610869	MG611106			MG612810			MG613425	MG613765	MG614101	MG614763	MG636990
**Buprestoidea / Buprestidae**																
Coroebussp.	MG609522	MG610080	MG609802	MG610393	MG610717	MG611005	MG611248	MG612096	MG612670	MG612357	MG612969	MG613276	MG613621		MG614626	

Dicercasp.	MG609567	MG610130		MG610443	MG610773		MG611290		MG612719	MG612412	MG613018	MG613331	MG613678		MG614680	MG636903
Trachyssp.	MG609568	MG610131	MG609843	MG610444	MG610774	MG611046	MG611291	MG612135	MG612720	MG612413	MG613019	MG613332	MG613679	MG614002	MG614681	MG636904
**Byrrhoidea / Byrrhidae**																
Notolioonsp.	MG609392		MG609659	MG610239	MG610571	MG610892			MG612526	MG612220	MG612838	MG613127	MG613459	MG613793	MG614473	MG636726
Microchaetessp.	MG609442	MG609994	MG609713	MG610295	MG610623	MG610933	MG611162	MG612028	MG612582	MG612272	MG612884	MG613182	MG613521	MG613847	MG614530	MG636774
Cytilussp.				MG610419	MG610746	MG611030	MG611271		MG612697	MG612386			MG613653	MG613976	MG614654	
**Callirhipidae**																
Ennometessp.	MG609393	MG609950		MG610240	MG610572	MG610893	MG611119		MG612527	MG612221		MG613128	MG613460		MG614474	MG636727
Simianussp.	MG609585	MG610156	MG609866	MG610475	MG610803	MG611065	MG611315		MG612748	MG612440		MG613361	MG613701	MG614034	MG614705	MG636929
**Dryopidae**																
Helichussp.	MG609401	MG609956	MG609667	MG610247	MG610581				MG612534		MG612846		MG613469		MG614482	
Pachyparnussp.	MG609616		MG609902	MG610516	MG610843		MG611352		MG612789		MG613081		MG613741	MG614076	MG614746	MG636967
Helichussp.	MG609627	MG610204	MG609915	MG610533	MG610861				MG612804	MG612491			MG613757	MG614094	MG614756	MG636983
**Elmidae**																
Graphelmissp.	MG609456	MG610006	MG609728	MG610311	MG610639	MG610943	MG611174		MG612597	MG612283		MG613194	MG613538	MG613862	MG614545	
Stetholussp.	MG609457	MG610007			MG610640	MG610944			MG612598	MG612284	MG612899	MG613195	MG613539	MG613863	MG614546	MG636784
Stenelmissp.	MG609537	MG610096		MG610408		MG611021	MG611261		MG612686	MG612373		MG613292	MG613639	MG613962		
**Eulichadidae**																
Eulichassp.	MG609527	MG610085	MG609806	MG610397	MG610722	MG611010	MG611251		MG612675	MG612362	MG612973	MG613281	MG613627	MG613951	MG614631	MG636862
**Heteroceridae**																
Heterocerussp.	MG609573		MG609850	MG610451	MG610781		MG611298	MG612139	MG612728	MG612420		MG613338	MG613687	MG614010	MG614688	MG636910
**Chelonariidae**																
Chelonariumsp.	MG609395	MG609951	MG609662	MG610242	MG610575	MG610894		MG611983	MG612528	MG612222	MG612840	MG613130	MG613463	MG613796	MG614476	MG636728
**Limnichidae**																
Byrrhinussp.	MG609469	MG610020	MG609742	MG610329	MG610654	MG610958	MG611189	MG612046	MG612609	MG612294	MG612913	MG613210	MG613556	MG613881	MG614563	MG636800
Pelocharessp.	MG609541	MG610099	MG609818	MG610412	MG610736	MG611024	MG611264	MG612113	MG612690	MG612377	MG612984	MG613296	MG613643	MG613966	MG614643	MG636875
Cephalobyrrhussp.	MG609566	MG610129	MG609842	MG610442	MG610772			MG612134	MG612718	MG612411	MG613017	MG613330	MG613677	MG614001	MG614679	MG636902
Limnichussp.	MG609640	MG610217	MG609929	MG610548	MG610876		MG611379	MG612203	MG612816	MG612505	MG613107	MG613434			MG614770	MG636996
**Psephenidae**																
Sclerocyphonsp.			MG609687	MG610268									MG613491	MG613822		
Schinostethussp.	MG609548	MG610107	MG609825	MG610418	MG610745	MG611029		MG612121	MG612696	MG612385	MG612994	MG613306	MG613652	MG613975	MG614653	MG636883
Schinostethussp.	MG609564	MG610127	MG609840	MG610440	MG610770	MG611045		MG612133	MG612717	MG612409	MG613016	MG613328	MG613675	MG613999	MG614677	MG636901
Mataeopsephussp.	MG609565	MG610128	MG609841	MG610441	MG610771		MG611289			MG612410		MG613329	MG613676	MG614000	MG614678	
**Ptilodactylidae**																
Ptilodactylasp.	MG609519	MG610078	MG609799	MG610390	MG610714	MG611002	MG611245		MG612667	MG612354	MG612967	MG613273	MG613618	MG613943	MG614623	MG636856
Epilichassp.	MG609563			MG610439	MG610769	MG611044	MG611288		MG612716	MG612408	MG613015	MG613327	MG613674	MG613998	MG614676	MG636900
**Cleroidea /Acanthocnemidae**																
Acanthocnemusnigricans	MG609433	MG609985	MG609701	MG610283	MG610611	MG610925	MG611154	MG612020	MG612572	MG612261		MG613170	MG613507	MG613834	MG614517	MG636764
**Biphyllidae**																
Althaesiasp.		MG609948	MG609657	MG610237	MG610568	MG610891			MG612524	MG612218	MG612836	MG613125	MG613456	MG613790		
Biphyllussp.	MG609437		MG609707	MG610289	MG610616	MG610929	MG611158		MG612577	MG612267	MG612878	MG613176	MG613513	MG613840	MG614523	MG636769
**Byturidae**																
Haematoidessp.	MG609538	MG610097	MG609816	MG610409	MG610734	MG611022	MG611262	MG612110	MG612687	MG612374	MG612982	MG613293	MG613640	MG613963		MG636873
**Cleridae**																
Tenerussp.				MG610301									MG613528			
Necrobiasp.	MG609449		MG609720	MG610302	MG610630	MG610937	MG611165		MG612589	MG612276		MG613186	MG613529	MG613853		
Xenorthriussp.		MG610076		MG610387		MG611000	MG611243					MG613270	MG613617	MG613941		MG636854
Cladiscussp.	MG609622	MG610200	MG609910	MG610529	MG610856	MG611095	MG611361	MG612188	MG612799	MG612487		MG613414	MG613752	MG614089		MG636979
Stenocallimerussp.	MG609623	MG610201	MG609911	MG610530	MG610857	MG611096	MG611362		MG612800			MG613415	MG613753	MG614090		

Allochotessp.	MG609624		MG609912	MG610531	MG610858	MG611097	MG611363	MG612189	MG612801	MG612488	MG613093	MG613416	MG613754	MG614091		MG636980
**Melyridae**																
Dasytess.l.sp.	MG609472		MG609745	MG610332	MG610657	MG610960	MG611192	MG612048	MG612612	MG612297	MG612916	MG613213	MG613559	MG613883	MG614566	MG636802
Dicranolaiusbellulus	MG609473	MG610023	MG609746	MG610333	MG610658	MG610961	MG611193			MG612298	MG612917	MG613214	MG613560	MG613884	MG614567	
Carphurussp.	MG609474	MG610024	MG609747	MG610334	MG610659	MG610962	MG611194		MG612613	MG612299	MG612918	MG613215	MG613561	MG613885	MG614568	MG636803
gen.sp.	MG609515		MG609793	MG610379	MG610706		MG611239	MG612089	MG612658	MG612347			MG613610	MG613933		MG636846
Carphurussp.	MG609594	MG610165	MG609875	MG610484	MG610812	MG611071	MG611325	MG612158	MG612758	MG612450	MG613052	MG613371	MG613709	MG614044	MG614713	MG636939
**Prionoceridae**																
Idgiasp.	MG609539		MG609817	MG610410	MG610735	MG611023	MG611263	MG612111	MG612688	MG612375		MG613294	MG613641	MG613964		MG636874
**Thanerocleridae**																
Isoclerussp.	MG609428		MG609696	MG610278	MG610607		MG611150	MG612016	MG612567	MG612256		MG613165	MG613502	MG613829		
**Trogossitidae**																
Larinotusumblicatus	MG609431		MG609699	MG610281	MG610609	MG610923	MG611152	MG612018	MG612570	MG612259		MG613168	MG613504	MG613832		MG636762
Rentonellumsp.																
Ancyronasp.		MG610061		MG610367	MG610695		MG611230	MG612081		MG612336		MG613252	MG613601	MG613922		
Parapeltisaustralicum			MG609784	MG610368	MG610696	MG610992	MG611231	MG612082	MG612648	MG612337	MG612953	MG613253	MG613602	MG613923	MG614607	MG636838
Leperinasp.		MG610062	MG609785	MG610369	MG610697		MG611232	MG612083	MG612649	MG612338		MG613254	MG613603	MG613924		
Thymalussp.	MG609526	MG610084				MG611009		MG612101	MG612674	MG612361		MG613280	MG613626	MG613950		MG636861
**Coccinelloidea /Anamorphidae**																
Papuellasp.		MG610008	MG609729	MG610312	MG610641	MG610945	MG611175				MG612900	MG613196	MG613540	MG613864	MG614547	MG636785
**Bothrideridae**																
Deretaphrussp.			MG609711	MG610292							MG612881		MG613517			
Ascetoderessp.	MG609441	MG609993		MG610293	MG610621		MG611161			MG612271	MG612883	MG613181	MG613519	MG613845	MG614528	MG636773
**Cerylonidae**																
Philothermussp.			MG609661	MG610241	MG610574						MG612839		MG613462	MG613795		
Ostomopsissp.	MG609445	MG609996	MG609716	MG610297	MG610626	MG610934		MG612029	MG612585		MG612887		MG613524	MG613850	MG614533	
**Coccinellidae**																
Chnootribasp.				MG610227	MG610557				MG612514		MG612825	MG613115	MG613444	MG613780		
Harmoniaoctomaculata		MG609939		MG610228	MG610558			MG611975	MG612515	MG612210	MG612826	MG613116	MG613445	MG613781	MG614465	MG636717
Exochomussp.			MG609650	MG610229	MG610559				MG612516		MG612827		MG613446			
Rhizobiussp.		MG609940		MG610230	MG610560		MG611113	MG611976	MG612517	MG612211	MG612828	MG613117	MG613447	MG613782		MG636718
Ortaliasp.	MG609386	MG609941		MG610231	MG610561	MG610886	MG611114		MG612518	MG612212	MG612829	MG613118	MG613448	MG613783		MG636719
Microfreudeasp.			MG609651	MG610232	MG610562								MG613449	MG613784		
Sasajiscymnussp.	MG609387	MG609942		MG610233	MG610563	MG610887	MG611115	MG611977	MG612519	MG612213	MG612830	MG613119	MG613450	MG613785	MG614466	MG636720
Stethorussp.	MG609388	MG609943	MG609652	MG610234	MG610564	MG610888	MG611116	MG611978	MG612520	MG612214	MG612831	MG613120	MG613451	MG613786	MG614467	MG636721
**Corylophidae**																
Sericoderussp.	MG609397	MG609953		MG610244	MG610577	MG610896	MG611121	MG611985	MG612530	MG612224	MG612842	MG613132	MG613465	MG613798	MG614478	MG636730
Priamimasp.	MG609450	MG609999	MG609721	MG610303	MG610631	MG610938	MG611166				MG612891	MG613187	MG613530	MG613854	MG614537	MG636778
Periptyctussp.	MG609451		MG609722	MG610304	MG610632		MG611167	MG612033	MG612590		MG612892		MG613531	MG613855	MG614538	MG636779
Orthoperussp.	MG609637		MG609927		MG610872	MG611110	MG611375	MG612200	MG612813	MG612501	MG613104	MG613430		MG614106	MG614768	MG636993
**Discolomatidae**																
Aphanocephalussp.	MG609400	MG609955	MG609666		MG610580	MG610899	MG611124	MG611988	MG612533	MG612227	MG612845		MG613468	MG613801	MG614481	MG636733
**Endomychidae**																
Cyclotomasp.	MG609402		MG609668	MG610248	MG610582	MG610900		MG611989	MG612535		MG612847	MG613135	MG613470	MG613802	MG614483	MG636734
Holoparamecussp.			MG609730	MG610313	MG610642		MG611176				MG612901	MG613197	MG613541	MG613865	MG614548	
Encymonimmaculatus	MG609458		MG609731	MG610314	MG610643				MG612599			MG613198	MG613542	MG613866	MG614549	
Stenotarsussp.	MG609459			MG610315	MG610644	MG610946	MG611177		MG612600		MG612902	MG613199	MG613543	MG613867	MG614550	MG636786
Sinocymbachussp.				MG610381	MG610708				MG612660	MG612349		MG613265	MG613612	MG613935	MG614617	MG636848
**Euxestidae**																

Hypodacnellasp.	MG609394		MG609660		MG610573							MG613129	MG613461	MG613794	MG614475	
**Latridiidae**																
Enicmussp.		MG609963			MG610590	MG610906	MG611130		MG612542	MG612235	MG612854	MG613142	MG613477	MG613808	MG614490	
Corticariasp.			MG609675	MG610255					MG612543		MG612855	MG613143	MG613478	MG613809		MG636741
Melanophthalmasp.		MG610019	MG609741	MG610328		MG610957			MG612608			MG613209	MG613555	MG613880		MG636799
**Murmidiidae**																
Murmidiusovalis	MG609444		MG609715		MG610625				MG612584	MG612274	MG612886		MG613523	MG613849	MG614532	MG636775
**Teredidae**																
Xylariophilussp.					MG610570								MG613458	MG613792		
Xylariophilussp.	MG609439	MG609991	MG609710		MG610619	MG610931		MG612027	MG612580	MG612269	MG612880	MG613179	MG613516	MG613843	MG614526	MG636772
Teredolaemussp.	MG609440	MG609992	MG609712		MG610620	MG610932			MG612581	MG612270	MG612882	MG613180	MG613518	MG613844	MG614527	
**Cucujoidea/Alexiidae**																
Sphaerosomasp.		MG609945	MG609654		MG610565				MG612521		MG612833	MG613122	MG613453	MG613787	MG614469	MG636722
**Boganiidae**																
Paracucujusrostratus	MG609391	MG609949	MG609658	MG610238	MG610569		MG611118	MG611982	MG612525	MG612219	MG612837	MG613126	MG613457	MG613791	MG614472	MG636725
**Cryptophagidae**																
Micrambinasp.	MG609398		MG609664	MG610245	MG610578	MG610897	MG611122	MG611986	MG612531	MG612225	MG612843	MG613133	MG613466	MG613799	MG614479	MG636731
Cureliussp.	MG609625	MG610202	MG609913	MG610532	MG610859	MG611098	MG611364		MG612802	MG612489	MG613094	MG613417	MG613755	MG614092	MG614754	MG636981
**Cucujidae**																
Platisussp.	MG609399	MG609954	MG609665	MG610246	MG610579	MG610898	MG611123	MG611987	MG612532	MG612226	MG612844	MG613134	MG613467	MG613800	MG614480	MG636732
Pediacusmajor	MG609452	MG610000	MG609723	MG610305	MG610633	MG610939	MG611168	MG612034	MG612591	MG612277	MG612893	MG613188	MG613532	MG613856	MG614539	MG636780
**Cybocephalidae**																
Cybocephalussp.		MG610001	MG609724	MG610306	MG610634		MG611169	MG612035	MG612592	MG612278	MG612894	MG613189	MG613533	MG613857	MG614540	MG636781
**Erotylidae**																
Thallissp.	MG609460	MG610009	MG609732	MG610316	MG610645	MG610947	MG611178	MG612039		MG612285	MG612903	MG613200	MG613544	MG613868	MG614551	MG636787
Anadastussp.	MG609461	MG610010	MG609733	MG610317	MG610646	MG610948		MG612040		MG612286	MG612904		MG613545	MG613869	MG614552	MG636788
Episcaphulasp.	MG609462	MG610011		MG610318	MG610647	MG610949	MG611179	MG612041			MG612905		MG613546	MG613870	MG614553	MG636789
Cryptophilussp.	MG609463	MG610012	MG609734	MG610319	MG610648	MG610950	MG611180		MG612601	MG612287	MG612906	MG613201	MG613547	MG613871	MG614554	MG636790
Episcaphasp.	MG609525	MG610083		MG610396	MG610721	MG611008		MG612100			MG612972		MG613625	MG613949	MG614630	
Tetraphalasp.	MG609530	MG610088	MG609809		MG610725	MG611013	MG611253	MG612103		MG612365	MG612976		MG613630	MG613954	MG614634	MG636865
**Helotidae**																
Neohelotasp.	MG609516	MG610070	MG609794	MG610380	MG610707	MG610998	MG611240	MG612090	MG612659	MG612348	MG612962	MG613264	MG613611	MG613934	MG614616	MG636847
**Hobartiidae**																
Hydnobioidessp.		MG610016	MG609737	MG610323	MG610651		MG611184	MG612043	MG612605	MG612291	MG612909	MG613204	MG613551	MG613875	MG614558	MG636794
**Kateretidae**																
Notobrachypterussp.	MG609408		MG609674		MG610589			MG611995	MG612541	MG612234	MG612853	MG613141	MG613476	MG613807	MG614489	MG636740
**Laemophloeidae**																
Laemophloeussp.	MG609468	MG610018	MG609740	MG610327	MG610653	MG610956	MG611188	MG612045	MG612607	MG612293	MG612912	MG613208	MG613554	MG613879	MG614562	MG636798
Cryptolestessp.	MG609626	MG610203	MG609914		MG610860	MG611099	MG611365	MG612190	MG612803	MG612490	MG613095	MG613418	MG613756	MG614093	MG614755	MG636982
**Monotomidae**																
Mimemodessp.	MG609413	MG609966	MG609679	MG610260	MG610592	MG610908	MG611135	MG612000	MG612548	MG612240	MG612860	MG613148		MG613813	MG614495	MG636745
Monotomopsissp.	MG609475	MG610025	MG609748	MG610335	MG610660	MG610963	MG611195	MG612049	MG612614	MG612300	MG612919	MG613216	MG613562	MG613886	MG614569	MG636804
Rhizophagussp.	MG609476	MG610026	MG609749	MG610336	MG610661	MG610964	MG611196	MG612050	MG612615	MG612301	MG612920	MG613217	MG613563	MG613887	MG614570	MG636805
Thionesp.	MG609635	MG610212	MG609925	MG610543	MG610870	MG611108	MG611373	MG612198	MG612811	MG612499	MG613102	MG613428	MG613768	MG614104	MG614766	MG636991
**Myraboliidae**																
Myraboliasp.	MG609416	MG609969	MG609682	MG610263	MG610594	MG610910	MG611137	MG612003	MG612551	MG612242	MG612862	MG613151	MG613485	MG613816	MG614498	MG636747
**Nitidulidae**																
Brachypeplussp.		MG610028	MG609750	MG610338	MG610663	MG610965	MG611197	MG612052	MG612616	MG612303	MG612921	MG613219	MG613565	MG613889	MG614572	MG636807
Pallodessp.	MG609477	MG610029	MG609751		MG610664	MG610966	MG611198		MG612617	MG612304	MG612922		MG613566	MG613890	MG614573	MG636808

Urophorussp.		MG610109	MG609827		MG610749	MG611032	MG611273		MG612699	MG612389		MG613308	MG613656	MG613979	MG614657	MG636886
Carpophilussp.		MG610205	MG609916	MG610534	MG610862	MG611100	MG611366	MG612191	MG612805	MG612492		MG613419	MG613758	MG614095	MG614757	MG636984
Glischrochilusparvipustulatus	MG609628	MG610206	MG609917		MG610863	MG611101	MG611367	MG612192	MG612806	MG612493	MG613096	MG613420	MG613759	MG614096	MG614758	MG636985
**Passandridae**																
Passandrasp.	MG609480	MG610031	MG609753	MG610340	MG610667	MG610968	MG611201	MG612054	MG612620	MG612307	MG612924	MG613222	MG613569	MG613893	MG614576	MG636811
**Phalacridae**																
gen.sp.	MG609419	MG609972	MG609685	MG610265	MG610596	MG610913	MG611139	MG612006	MG612554	MG612245	MG612865	MG613154	MG613488	MG613819	MG614501	MG636750
Phalacrinussp.	MG609481	MG610032	MG609754	MG610341	MG610668	MG610969	MG611202	MG612055	MG612621	MG612308	MG612925	MG613223	MG613570	MG613894	MG614577	MG636812
Olibrussp.	MG609587	MG610158	MG609869	MG610478	MG610806	MG611066	MG611318	MG612153	MG612751	MG612443	MG613047	MG613364	MG613702	MG614037	MG614706	MG636932
**Phloeostichidae**																
Hymaeasp.	MG609482	MG610033	MG609755	MG610342	MG610669	MG610970	MG611203	MG612056	MG612622	MG612309	MG612926	MG613224	MG613571	MG613895	MG614578	MG636813
Rhopalobrachiumcrowsoni	MG609483	MG610034	MG609756	MG610343	MG610670	MG610971	MG611204	MG612057	MG612623	MG612310	MG612927	MG613225	MG613572	MG613896	MG614579	MG636814
**Propalticidae**																
Propalticussp.		MG609974	MG609686	MG610267	MG610598	MG610915	MG611141	MG612008	MG612556	MG612247	MG612867	MG613156	MG613490	MG613821	MG614503	MG636752
**Protocucujidae**																
Ericmodescostatus	MG609484	MG610035	MG609757	MG610344	MG610671	MG610972	MG611205	MG612058	MG612624	MG612311	MG612928	MG613226	MG613573	MG613897	MG614580	MG636815
**Silvanidae**																
Uleiotasp.	MG609426	MG609981	MG609694	MG610276	MG610605	MG610921	MG611149	MG612014	MG612564	MG612254		MG613162	MG613499	MG613827	MG614511	MG636758
Cryptamorphasp.	MG609499	MG610048	MG609771				MG611217		MG612638		MG612940	MG613239	MG613588	MG613910	MG614593	MG636829
Silvanoprusscuticollis		MG610049	MG609772			MG610980	MG611218					MG613240			MG614594	MG636830
Psammoecussp.	MG609575	MG610136	MG609852	MG610454	MG610783	MG611054	MG611300		MG612730	MG612422	MG613025	MG613340	MG613690	MG614012	MG614690	MG636912
Psammoecussp.	MG609588	MG610159	MG609870		MG610807		MG611319	MG612154	MG612752	MG612444	MG613048	MG613365	MG613703	MG614038	MG614707	MG636933
**Sphindidae**																
Aspidiphorussp.	MG609427	MG609982		MG610277					MG612565	MG612255	MG612873	MG613163	MG613500	MG613828	MG614512	MG636759
**Curculionoidea/Anthribidae**																
Acorynussp.		MG610072	MG609795	MG610383	MG610710				MG612662	MG612351	MG612963	MG613266	MG613613	MG613937	MG614619	MG636850
Ozotomerussp.			MG609819	MG610413	MG610737					MG612378	MG612985	MG613297	MG613644	MG613967	MG614644	MG636876
Xylinadasp.				MG610420	MG610747					MG612387	MG612995		MG613654	MG613977	MG614655	MG636884
Peribathyssp.			MG609857	MG610461	MG610790					MG612428	MG613031	MG613347	MG613696	MG614019	MG614697	MG636919
**Attelabidae**																
Phymatapoderussp.	MG609547				MG610744		MG611270	MG612120			MG612993	MG613305	MG613651	MG613974	MG614652	
Involvulussp.				MG610435	MG610764			MG612131		MG612404	MG613011	MG613323	MG613670	MG613993		MG636896
Byctiscussp.		MG610124		MG610436	MG610766		MG611285		MG612714	MG612406	MG613012	MG613324	MG613671	MG613995	MG614673	MG636897
Paratrachelophorussp.				MG610528	MG610855		MG611360	MG612187		MG612486	MG613092	MG613413	MG613751		MG614753	MG636978
**Belidae**																
Rhinotiasp.		MG609990	MG609706	MG610288	MG610615			MG612025		MG612266	MG612877	MG613175	MG613512	MG613839	MG614522	MG636768
**Brentidae**																
Apions.l.sp.				MG610294	MG610622								MG613520	MG613846	MG614529	
Baryrhynchussp.		MG610100			MG610738		MG611265	MG612114	MG612691	MG612379	MG612986	MG613298	MG613645	MG613968	MG614645	MG636877
Apions.l.sp.					MG610765					MG612405				MG613994	MG614672	
Cylassp.				MG610535	MG610864						MG613097		MG613760			
**Curculionidae**																
Episomussp.		MG610074	MG609797	MG610385				MG612091			MG612965	MG613268	MG613615	MG613939	MG614621	MG636852
gen.sp.		MG610101		MG610414	MG610739			MG612115		MG612380	MG612987	MG613299			MG614646	MG636878
Xylosandrussp.	MG609544	MG610104	MG609822	MG610416	MG610742	MG611027	MG611268	MG612118			MG612990	MG613302	MG613648	MG613971	MG614649	MG636881
Curculiosp.		MG610199		MG610527	MG610854	MG611094	MG611358				MG613090	MG613411	MG613749	MG614087	MG614751	MG636976
Peribleptussp.			MG609909				MG611359	MG612186			MG613091	MG613412	MG613750	MG614088	MG614752	MG636977
**Nemonychidae**																
AragomaceruniformisKuschel		MG610027		MG610337	MG610662			MG612051		MG612302		MG613218	MG613564	MG613888	MG614571	MG636806

**Dasciloidea/ Dascillidae**																
Dascillussp.	MG609529	MG610087	MG609808	MG610399	MG610724	MG611012			MG612677	MG612364	MG612975	MG613283	MG613629	MG613953	MG614633	MG636864
Metallidascillussp.	MG609629	MG610207	MG609918	MG610536	MG610865	MG611102	MG611368	MG612193		MG612494	MG613098	MG613421	MG613761	MG614097	MG614759	MG636986
**Rhipiceridae**																
Oligorhipisbifossata	MG609423	MG609978	MG609691	MG610273	MG610602	MG610918	MG611146	MG612011	MG612561	MG612251	MG612870	MG613159	MG613496		MG614508	MG636755
**Derodontoidea /Derodontidae**																
Derodontussp.	MG609454	MG610004	MG609726	MG610309	MG610637	MG610941	MG611172		MG612595	MG612281	MG612897	MG613192	MG613536	MG613860	MG614543	MG636783
Derolathrussp.		MG609962	MG609673	MG610254	MG610588	MG610905				MG612233	MG612852	MG613140	MG613475		MG614488	MG636739
**Jacobsoniidae**																
Derolathrussp.			MG609739	MG610326	MG610652	MG610955	MG611187			MG612292	MG612911	MG613207	MG613553	MG613878	MG614561	MG636797
**Nosodendridae**																
Nosodendronsp.	MG609417	MG609970	MG609683	MG610264	MG610595	MG610911	MG611138	MG612004	MG612552	MG612243	MG612863	MG613152	MG613486	MG613817	MG614499	MG636748
**Elateroidea/Artematopodidae**																
Artematopussp.	MG609390	MG609947	MG609656	MG610236	MG610567	MG610890		MG611981	MG612523	MG612217	MG612835	MG613124	MG613455	MG613789	MG614471	MG636724
Eurypogonsp.	MG609631	MG610209	MG609920	MG610538	MG610867	MG611104	MG611370	MG612195	MG612808	MG612496	MG613100	MG613423	MG613763	MG614099	MG614761	MG636988
**Cantharidae**																
Heteromastixsp.	MG609443	MG609995	MG609714	MG610296	MG610624		MG611163		MG612583	MG612273	MG612885	MG613183	MG613522	MG613848	MG614531	
Themussp.		MG610068		MG610377	MG610704	MG610996	MG611238		MG612656	MG612345	MG612960	MG613262	MG613608	MG613931	MG614615	MG636844
Themussp.	MG609554	MG610115	MG609831	MG610427	MG610755	MG611038	MG611278	MG612125	MG612705	MG612395	MG613002	MG613314	MG613661	MG613985	MG614663	MG636890
Prothemussp.	MG609555	MG610116	MG609832	MG610428	MG610756		MG611279	MG612126	MG612706	MG612396	MG613003	MG613315	MG613662	MG613986	MG614664	
Ichthyurusbourgeoisi	MG609556	MG610117	MG609833		MG610757	MG611039		MG612127	MG612707	MG612397	MG613004	MG613316	MG613663		MG614665	MG636891
Laemoglyptussp.	MG609604	MG610181	MG609890	MG610503	MG610830	MG611080	MG611339		MG612776	MG612465	MG613070	MG613389	MG613729	MG614064	MG614733	MG636958
Malthinussp.	MG609605	MG610182	MG609891	MG610504	MG610831	MG611081	MG611340	MG612169	MG612777	MG612466	MG613071	MG613390	MG613730	MG614065	MG614734	MG636959
Lycocerussp.		MG610183	MG609892	MG610505	MG610832	MG611082	MG611341	MG612170	MG612778	MG612467	MG613072	MG613391	MG613731	MG614066	MG614735	MG636960
Fissocantharissp.	MG609606	MG610184	MG609893	MG610506	MG610833		MG611342	MG612171	MG612779	MG612468	MG613073	MG613392	MG613732	MG614067	MG614736	MG636961
**Elateridae**																
Osslimussp.	MG609455	MG610005	MG609727	MG610310	MG610638	MG610942	MG611173	MG612038	MG612596	MG612282	MG612898	MG613193	MG613537	MG613861	MG614544	
Hemicrepidiussp.	MG609520		MG609800	MG610391	MG610715	MG611003	MG611246	MG612094	MG612668	MG612355		MG613274	MG613619	MG613944	MG614624	MG636857
Pectocerasp.	MG609609		MG609896	MG610509	MG610836	MG611085	MG611345	MG612174	MG612782	MG612471		MG613395	MG613735	MG614069	MG614739	
Agrypnussp.	MG609610	MG610187	MG609897	MG610510	MG610837		MG611346	MG612175	MG612783	MG612472	MG613076	MG613396	MG613736	MG614070	MG614740	
Ampedussp.	MG609611	MG610188		MG610511	MG610838		MG611347	MG612176	MG612784	MG612473	MG613077	MG613397	MG613737	MG614071	MG614741	
Denticollissp.	MG609612	MG610189	MG609898	MG610512	MG610839	MG611086	MG611348	MG612177	MG612785	MG612474	MG613078	MG613398	MG613738	MG614072	MG614742	MG636964
Peniasp.	MG609613	MG610190	MG609899	MG610513	MG610840	MG611087	MG611349	MG612178	MG612786	MG612475	MG613079	MG613399	MG613739	MG614073	MG614743	MG636965
gen.sp.	MG609614	MG610191	MG609900	MG610514	MG610841		MG611350	MG612179	MG612787	MG612476	MG613080	MG613400	MG613740	MG614074	MG614744	MG636966
Denticollissp.	MG609615	MG610192	MG609901	MG610515	MG610842		MG611351	MG612180	MG612788	MG612477		MG613401		MG614075	MG614745	
Cardiotarsussp.		MG610214		MG610545	MG610873		MG611376			MG612502	MG613105	MG613431	MG613770	MG614107		
Melanotussp.	MG609638	MG610215	MG609928	MG610546	MG610874	MG611111	MG611377	MG612201	MG612814	MG612503	MG613106	MG613432	MG613771	MG614108	MG614769	MG636994
Cebriosp.	MG609643	MG610220	MG609932	MG610551	MG610879		MG611381	MG612205	MG612818	MG612507	MG613110	MG613437	MG613773	MG614112	MG614773	
**Eucnemidae**																
Anischiasp.	MG609404	MG609958	MG609670	MG610250	MG610584	MG610902	MG611126	MG611991	MG612537	MG612229	MG612848	MG613137	MG613472	MG613804	MG614485	MG636736
Hemiopsidasp.	MG609465	MG610014		MG610321		MG610951	MG611182		MG612603	MG612289		MG613202	MG613549	MG613873	MG614556	MG636792
Othosp.	MG609535	MG610093		MG610405	MG610731	MG611018	MG611258	MG612108	MG612683	MG612370	MG612981	MG613289	MG613636	MG613960	MG614640	
Pyrocoeliasp.	MG609521	MG610079	MG609801	MG610392	MG610716	MG611004	MG611247	MG612095	MG612669	MG612356	MG612968	MG613275	MG613620	MG613945	MG614625	
Vestasp.		MG610111		MG610423	MG610751	MG611034	MG611274		MG612701	MG612391	MG612998	MG613310	MG613657	MG613981	MG614659	
Pristolycussp.	MG609551	MG610112	MG609829	MG610424	MG610752	MG611035	MG611275		MG612702	MG612392	MG612999	MG613311	MG613658	MG613982	MG614660	MG636888
Luciolasp.	MG609552	MG610113	MG609830	MG610425	MG610753	MG611036	MG611276	MG612123	MG612703	MG612393	MG613000	MG613312	MG613659	MG613983	MG614661	MG636889
Cyphonocerussp.	MG609553	MG610114		MG610426	MG610754	MG611037	MG611277	MG612124	MG612704	MG612394	MG613001	MG613313	MG613660	MG613984	MG614662	
Drilastersp.	MG609607	MG610185	MG609894	MG610507	MG610834	MG611083	MG611343	MG612172	MG612780	MG612469	MG613074	MG613393	MG613733	MG614068	MG614737	MG636962
Diaphanessp.	MG609608	MG610186	MG609895	MG610508	MG610835	MG611084	MG611344	MG612173	MG612781	MG612470	MG613075	MG613394	MG613734		MG614738	MG636963

Gorhamiasp.	MG609642	MG610219	MG609931	MG610550	MG610878	MG611112	MG611380	MG612204	MG612817	MG612506	MG613109	MG613436	MG613772	MG614111	MG614772	MG636997
**Lycidae**																
Porrostomasp.	MG609470	MG610021	MG609743	MG610330	MG610655		MG611190		MG612610	MG612295	MG612914	MG613211	MG613557	MG613882	MG614564	
Benibotarussp.	MG609644	MG610221	MG609933	MG610552	MG610880		MG611382		MG612819	MG612508	MG613111	MG613438	MG613774	MG614113	MG614774	
Dilophotessp.	MG609645	MG610222	MG609934	MG610553	MG610881		MG611383	MG612206	MG612820	MG612509	MG613112	MG613439	MG613775	MG614114	MG614775	
Platerodrilussp.	MG609646	MG610223	MG609935		MG610882		MG611384		MG612821	MG612510		MG613440	MG613776	MG614115	MG614776	
Libnetissp.	MG609647	MG610224	MG609936	MG610554	MG610883		MG611385	MG612207	MG612822	MG612511	MG613113	MG613441	MG613777	MG614116	MG614777	
Macrolycussichuanensis	MG609648	MG610225	MG609937	MG610555	MG610884		MG611386	MG612208	MG612823	MG612512	MG613114	MG613442	MG613778	MG614117	MG614778	
Lycostomussp.	MG609649	MG610226	MG609938	MG610556	MG610885		MG611387	MG612209	MG612824	MG612513		MG613443	MG613779	MG614118	MG614779	
**Omethidae**																
Driloniussp.	MG609590	MG610161	MG609872	MG610480	MG610808	MG611068	MG611321	MG612155	MG612754	MG612446		MG613367	MG613705	MG614040	MG614709	MG636935
Driloniussp.	MG609592	MG610163	MG609873	MG610482	MG610810	MG611069	MG611323	MG612156	MG612756	MG612448		MG613369	MG613707	MG614042	MG614711	MG636937
**Phengodidae**																
Stenophrixothrixsp.	MG609420	MG609973		MG610266	MG610597	MG610914	MG611140	MG612007	MG612555	MG612246	MG612866	MG613155	MG613489	MG613820	MG614502	MG636751
**Rhagophthalmidae**																
Rhagophthalmussp.	MG609422	MG609977	MG609690	MG610272	MG610601		MG611145		MG612560		MG612869	MG613158	MG613495	MG613824	MG614507	
Rhagophthalmussp.	MG609487	MG610038	MG609760	MG610347	MG610674		MG611208		MG612627		MG612930		MG613576	MG613900	MG614583	
gen.sp.	MG609591	MG610162		MG610481	MG610809		MG611322		MG612755	MG612447	MG613050	MG613368	MG613706	MG614041	MG614710	MG636936
**Throscidae**																
Trixagussp.	MG609429		MG609697	MG610279					MG612568	MG612257		MG613166		MG613830	MG614514	
Trixagussp.	MG609589	MG610160	MG609871	MG610479		MG611067	MG611320		MG612753	MG612445	MG613049	MG613366	MG613704	MG614039	MG614708	MG636934
**Histeridae**																
Platysomasp.	MG609405	MG609959	MG609671	MG610251	MG610585	MG610903	MG611127	MG611992	MG612538	MG612230	MG612849	MG613138			MG614486	MG636737
Saprinussp.	MG609406	MG609960	MG609672	MG610252	MG610586	MG610904	MG611128	MG611993	MG612539	MG612231	MG612850	MG613139	MG613473	MG613805	MG614487	MG636738
**Hydrophiloidea/ Hydrophilidae**																
Sternolophusimmarginatus				MG610324		MG610953	MG611185		MG612606		MG612910	MG613205		MG613876	MG614559	MG636795
Helocharessp.	MG609467	MG610017	MG609738	MG610325		MG610954	MG611186	MG612044				MG613206	MG613552	MG613877	MG614560	MG636796
Oocyclussp.				MG610389		MG611001	MG611244	MG612093	MG612666			MG613272			MG614622	
Hydrophilusacuminatus		MG610143	MG609858	MG610462	MG610791	MG611058		MG612145	MG612737		MG613032	MG613348		MG614020	MG614698	
Anacaenapui		MG610145		MG610464	MG610793	MG611059	MG611306		MG612739	MG612430		MG613349		MG614022	MG614699	
Georissussp.	MG609582	MG610146	MG609860	MG610465			MG611307		MG612740	MG612431	MG613034	MG613350		MG614023	MG614700	
Sphaeridiumquinquemaculatum		MG610147	MG609861	MG610466	MG610794	MG611060	MG611308	MG612147			MG613035	MG613351	MG613698	MG614024	MG614701	MG636921
Berosusspinosus															MG614732	
**Chrysomeloidea/ Cerambycidae**																
Strangaliafortunei	MG609557	MG610118	MG609834	MG610429	MG610758		MG611280	MG612128	MG612708	MG612398	MG613005	MG613317	MG613664	MG613987	MG614666	MG636892
Obereasp.	MG609558	MG610119	MG609835	MG610430	MG610759	MG611040			MG612709	MG612399	MG613006	MG613318	MG613665	MG613988	MG614667	MG636893
Spondylisbuprestoides	MG609559	MG610120		MG610431	MG610760		MG611281		MG612710	MG612400	MG613007	MG613319	MG613666	MG613989	MG614668	MG636894
Xylotrechussp.	MG609560	MG610121	MG609836	MG610432	MG610761		MG611282		MG612711	MG612401	MG613008	MG613320	MG613667	MG613990	MG614669	
Dorystheneshydropicus		MG610179	MG609888	MG610502	MG610828						MG613068		MG613727	MG614062	MG614730	MG636956
**Chrysomelidae**																
Cassidasp.		MG610071		MG610382	MG610709				MG612661	MG612350				MG613936	MG614618	MG636849
Chrysomelapopuli			MG609803		MG610718			MG612097	MG612671	MG612358		MG613277	MG613622	MG613946	MG614627	MG636858
Chlamisussp.		MG610125	MG609838	MG610437	MG610767	MG611042	MG611286				MG613013	MG613325	MG613672	MG613996	MG614674	MG636898
Sagrafulgidajanthina	MG609596	MG610167	MG609878	MG610487	MG610814				MG612761	MG612452	MG613054	MG613373	MG613712	MG614046	MG614716	MG636941
Liliocerissp.		MG610168		MG610488	MG610815				MG612762	MG612453	MG613055	MG613374	MG613713	MG614047	MG614717	MG636942
Dactylispasp.		MG610169		MG610489	MG610816				MG612763	MG612454			MG613714	MG614048	MG614718	MG636943
Anisoderasp.		MG610170		MG610490	MG610817		MG611327	MG612160	MG612764	MG612455		MG613375	MG613715	MG614049	MG614719	MG636944
Sominellasp.			MG609879	MG610491	MG610818		MG611328		MG612765	MG612456	MG613056	MG613376	MG613716	MG614050	MG614720	MG636945
Trichochryseasp.				MG610492			MG611329	MG612161	MG612766		MG613057	MG613377	MG613717	MG614051	MG614721	MG636946

gen.sp.	MG609597	MG610171	MG609880		MG610819	MG611074	MG611330	MG612162	MG612767	MG612457	MG613058	MG613378	MG613718	MG614052	MG614722	MG636947
Alticasp.		MG610172		MG610493	MG610820	MG611075	MG611331		MG612768	MG612458	MG613059	MG613379		MG614053	MG614723	MG636948
Oomorphoidessp.		MG610173	MG609881	MG610494	MG610821	MG611076	MG611332		MG612769	MG612459	MG613060	MG613380	MG613719	MG614054	MG614724	MG636949
Cryptocephalussp.		MG610174	MG609882	MG610495			MG611333				MG613061	MG613381	MG613720	MG614055	MG614725	MG636950
Bruchidiussp.			MG609883	MG610496	MG610822		MG611334	MG612163			MG613062	MG613382	MG613721	MG614056	MG614726	MG636951
**Megalopodidae**																
Temnaspissp.	MG609532	MG610090	MG609811	MG610401	MG610727	MG611015	MG611255	MG612105	MG612679	MG612367	MG612978	MG613285	MG613632	MG613956	MG614636	MG636867
**Lymexyloidea/Lymexylidae**																
Melittommasp.	MG609412		MG609678	MG610259			MG611134	MG611999	MG612547	MG612239	MG612859	MG613147	MG613482	MG613812	MG614494	
Atractocerussp.				MG610453							MG613024		MG613689			
**Rhinorhipoidea/Rhinorhipidae**																
Rhinorhipus tamborensis	AB123456	AB123457	AB123458	AB123459	AB123460	AB123461	AB123462	AB123463	AB123464	AB123465	AB123466	AB123467	AB123468	AB123469	AB123470	AB123520
**Scarabaeoidea/Geotrupidae**																
Australobolbussp.	MG609492		MG609764	MG610351	MG610678	MG610977	MG611212	MG612063	MG612631	MG612317	MG612934	MG613233	MG613581	MG613904	MG614587	MG636822
Geotrupessp.		MG610138	MG609853	MG610456	MG610785		MG611302	MG612142	MG612732	MG612423	MG613027	MG613342	MG613692	MG614014	MG614692	MG636914
**Glaphyridae**																
Amphicomasp.	MG609518	MG610075	MG609798	MG610386	MG610712		MG611242		MG612664	MG612352		MG613269	MG613616	MG613940		MG636853
**Hybosoridae**																
Liparochrussp.	MG609407	MG609961		MG610253	MG610587		MG611129	MG611994	MG612540	MG612232	MG612851		MG613474	MG613806		
Cyphopisthessp.	MG609493	MG610042	MG609765	MG610352	MG610679				MG612632	MG612318			MG613582		MG614588	MG636823
**Lucanidae**																
Phalacrognathusmuelleri	MG609577	MG610139		MG610457	MG610786	MG611056	MG611303		MG612733	MG612424	MG613028	MG613343	MG613693	MG614015	MG614693	MG636915
Cyclommatusmetallifer	MG609578	MG610140	MG609854	MG610458	MG610787			MG612143	MG612734	MG612425		MG613344	MG613694	MG614016	MG614694	MG636916
Aegussp.	MG609601	MG610177	MG609886	MG610500	MG610826		MG611337	MG612166	MG612773	MG612462	MG613066	MG613386	MG613725	MG614060	MG614729	MG636954
**Passalidae**																
Ceracupessp.	MG609550	MG610110	MG609828	MG610422	MG610750	MG611033			MG612700	MG612390	MG612997	MG613309		MG613980	MG614658	MG636887
Aceraiussp.	MG609576	MG610137		MG610455	MG610784	MG611055	MG611301	MG612141	MG612731		MG613026	MG613341	MG613691	MG614013	MG614691	MG636913
**Scarabaeidae**																
Carneodonsp.	MG609494	MG610043	MG609766	MG610353	MG610680	MG610978	MG611213	MG612064	MG612633	MG612319	MG612935	MG613234	MG613583	MG613905		MG636824
Heteronyxsp.	MG609495	MG610044	MG609767	MG610354			MG611214	MG612065	MG612634	MG612320	MG612936	MG613235	MG613584	MG613906	MG614589	MG636825
Anomalaantiqua	MG609496	MG610045	MG609768	MG610355	MG610681	MG610979	MG611215	MG612066	MG612635	MG612321	MG612937	MG613236	MG613585	MG613907	MG614590	MG636826
Onthophagusgazella	MG609497	MG610046	MG609769	MG610356	MG610682		MG611216	MG612067	MG612636	MG612322	MG612938	MG613237	MG613586	MG613908	MG614591	MG636827
Rhyparussp.		MG610069		MG610378	MG610705	MG610997			MG612657	MG612346	MG612961	MG613263	MG613609	MG613932		MG636845
Protaetiaaerata	MG609581	MG610144	MG609859	MG610463	MG610792			MG612146	MG612738	MG612429	MG613033		MG613697	MG614021		MG636920
Mimelasp.	MG609598	MG610175	MG609884	MG610497	MG610823	MG611077	MG611335	MG612164	MG612770	MG612460	MG613063	MG613383	MG613722	MG614057	MG614727	
Coprissp.	MG609599	MG610176		MG610498	MG610824	MG611078	MG611336	MG612165	MG612771	MG612461	MG613064	MG613384	MG613723	MG614058	MG614728	MG636952
Ectinohopliasp.	MG609600		MG609885	MG610499	MG610825				MG612772		MG613065	MG613385	MG613724	MG614059		MG636953
Dasyvalgussp.	MG609602	MG610178	MG609887	MG610501	MG610827			MG612167	MG612774	MG612463	MG613067	MG613387	MG613726	MG614061		MG636955
Cheirotonusjansoni	MG609603	MG610180	MG609889		MG610829	MG611079	MG611338	MG612168	MG612775	MG612464	MG613069	MG613388	MG613728	MG614063	MG614731	MG636957
Allomyrinadichotoma	MG609617	MG610194		MG610519	MG610846	MG611089	MG611354	MG612181	MG612792	MG612480	MG613084	MG613404	MG613742	MG614079		MG636970
**Trogidae**																
Omorgussp.	MG609430	MG609983	MG609698	MG610280	MG610608	MG610922	MG611151	MG612017	MG612569	MG612258		MG613167	MG613503	MG613831	MG614515	MG636761
Troxsp.	MG609536	MG610094	MG609814	MG610406	MG610732	MG611019	MG611259	MG612109	MG612684	MG612371		MG613290	MG613637	MG613961	MG614641	MG636871
**Scirtoidea/Clambidae**																
Clambussp.	MG609448		MG609719	MG610300	MG610629	MG610936		MG612032	MG612588		MG612890	MG613185	MG613527		MG614536	MG636777
**Eucinetidae**																
Noteucinetussp.	MG609403	MG609957	MG609669	MG610249	MG610583	MG610901	MG611125	MG611990	MG612536	MG612228		MG613136	MG613471	MG613803	MG614484	MG636735
Noteucinetussp.	MG609464	MG610013	MG609735	MG610320	MG610649		MG611181		MG612602	MG612288	MG612907		MG613548	MG613872	MG614555	MG636791
**Scirtidae**																

Pseudomicrocarasp.	MG609498	MG610047	MG609770	MG610357	MG610683			MG612068	MG612637	MG612323	MG612939	MG613238	MG613587	MG613909	MG614592	MG636828
gen.sp.	MG609540	MG610098		MG610411				MG612112	MG612689	MG612376	MG612983	MG613295	MG613642	MG613965	MG614642	
Elodessp.	MG609595	MG610166	MG609877	MG610486		MG611073	MG611326	MG612159	MG612760		MG613053	MG613372	MG613711	MG614045	MG614715	MG636940
**Staphylinoidea/Agyrtidae**																
Pterolomaforsstromii	MG609630	MG610208	MG609919	MG610537	MG610866	MG611103	MG611369	MG612194	MG612807	MG612495	MG613099	MG613422	MG613762	MG614098	MG614760	MG636987
**Hydraenidae**																
Hydraenasp.	MG609583	MG610148	MG609862	MG610467	MG610795	MG611061	MG611309	MG612148		MG612432	MG613036	MG613352	MG613699	MG614025	MG614702	MG636922
**Leiodidae**																
Agyrtodessp.	MG609409	MG609964	MG609676	MG610256	MG610591		MG611131	MG611996	MG612544	MG612236	MG612856	MG613144	MG613479	MG613810	MG614491	MG636742
gen.sp.	MG609410			MG610257			MG611132	MG611997	MG612545	MG612237	MG612857	MG613145	MG613480		MG614492	MG636743
Agathidiumsp.	MG609411	MG609965	MG609677	MG610258		MG610907	MG611133	MG611998	MG612546	MG612238	MG612858	MG613146	MG613481	MG613811	MG614493	MG636744
**Ptiliidae**																
gen.sp.									MG612557							
**Silphidae**																
Nicrophorusnepalensis	MG609524	MG610082	MG609805	MG610395	MG610720	MG611007	MG611250	MG612099	MG612673	MG612360	MG612971	MG613279	MG613624	MG613948	MG614629	MG636860
Necrodeslittoralis	MG609562	MG610126	MG609839	MG610438	MG610768	MG611043	MG611287	MG612132	MG612715	MG612407	MG613014	MG613326	MG613673	MG613997	MG614675	MG636899
**Staphylinidae**																
Scaphidiumsp.			MG609695		MG610606			MG612015	MG612566			MG613164	MG613501		MG614513	MG636760
gen.sp.				MG610358				MG612069	MG612639	MG612324	MG612941		MG613589	MG613911	MG614595	
Staphylinussp.	MG609517	MG610073	MG609796	MG610384	MG610711	MG610999	MG611241		MG612663		MG612964	MG613267	MG613614	MG613938	MG614620	MG636851
Megalopaederussp.	MG609531	MG610089	MG609810	MG610400	MG610726	MG611014	MG611254	MG612104	MG612678	MG612366	MG612977	MG613284	MG613631	MG613955	MG614635	MG636866
Apateticasp.	MG609569		MG609844	MG610445	MG610775	MG611047	MG611292		MG612721	MG612414	MG613020	MG613333	MG613680	MG614003	MG614682	
Priochirussp.	MG609570	MG610132	MG609845	MG610446	MG610776	MG611048	MG611293	MG612136	MG612722	MG612415		MG613334	MG613681	MG614004	MG614683	MG636905
Osoriussp.	MG609571		MG609846	MG610447	MG610777	MG611049	MG611294	MG612137	MG612723	MG612416	MG613021	MG613335	MG613682	MG614005	MG614684	MG636906
Tachinussp.		MG610133	MG609847	MG610448	MG610778	MG611050	MG611295		MG612724	MG612417		MG613336	MG613683	MG614006	MG614685	MG636907
Scaphidiumsp.			MG609848		MG610779		MG611296	MG612138	MG612725	MG612418	MG613022		MG613684	MG614007	MG614686	MG636908
Tachinussp.	MG609572	MG610134	MG609849	MG610449	MG610780	MG611051	MG611297		MG612726	MG612419		MG613337	MG613685	MG614008	MG614687	MG636909
Centrophthalmussp.				MG610450		MG611052			MG612727				MG613686	MG614009		
gen.sp.				MG610522	MG610849								MG613744	MG614082		
Dianoussp.	MG609619	MG610196	MG609906	MG610523	MG610850	MG611090	MG611355		MG612795	MG612483		MG613407	MG613745	MG614083	MG614747	
gen.sp.	MG609620			MG610524	MG610851	MG611091			MG612796		MG613087	MG613408	MG613746	MG614084	MG614748	MG636973
**Tenebrionoidea/Aderidae**																
gen.sp.		MG609944	MG609653	MG610235				MG611979		MG612215	MG612832	MG613121	MG613452		MG614468	
gen.sp.	MG609634	MG610211	MG609924	MG610542		MG611107	MG611372	MG612197		MG612498		MG613427	MG613767	MG614103	MG614765	
**Anthicidae**																
Lemodessp.	MG609389	MG609946	MG609655		MG610566	MG610889	MG611117	MG611980	MG612522	MG612216	MG612834	MG613123	MG613454	MG613788	MG614470	MG636723
Anthicuss.l.sp.		MG609986	MG609702	MG610284	MG610612	MG610926		MG612021	MG612573	MG612262		MG613171	MG613508	MG613835	MG614518	
Trichanancasp.	MG609434	MG609987	MG609703	MG610285	MG610613	MG610927	MG611155	MG612022	MG612574	MG612263	MG612875	MG613172	MG613509	MG613836	MG614519	MG636765
Macratriasp.	MG609435	MG609988	MG609704	MG610286			MG611156	MG612023	MG612575	MG612264		MG613173	MG613510	MG613837	MG614520	MG636766
Macratriasp.	MG609546	MG610106	MG609824	MG610417		MG611028	MG611269	MG612119	MG612695	MG612384	MG612992	MG613304	MG613650	MG613973	MG614651	MG636882
**Archeocrypticidae**																
Wattianusqueenslandicus	MG609436	MG609989	MG609705	MG610287	MG610614	MG610928	MG611157	MG612024	MG612576	MG612265	MG612876	MG613174	MG613511	MG613838	MG614521	MG636767
**Boridae**																
Synercticussp.			MG609708	MG610290	MG610617	MG610930	MG611159		MG612578			MG613177	MG613514	MG613841	MG614524	MG636770
**Ciidae**																
Cissp.	MG609396	MG609952	MG609663	MG610243	MG610576	MG610895	MG611120	MG611984	MG612529	MG612223	MG612841	MG613131	MG613464	MG613797	MG614477	MG636729
Cissp.	MG609446	MG609997	MG609717	MG610298	MG610627	MG610935	MG611164	MG612030	MG612586		MG612888		MG613525	MG613851	MG614534	
Australocissp.	MG609447	MG609998	MG609718	MG610299	MG610628			MG612031	MG612587	MG612275	MG612889	MG613184	MG613526	MG613852	MG614535	MG636776
**Tenebrionoidea/IncertaeSedis**																

Rhizoniumantiquum	MG609466	MG610015	MG609736	MG610322	MG610650	MG610952	MG611183	MG612042	MG612604	MG612290	MG612908	MG613203	MG613550	MG613874	MG614557	MG636793
**Ischaliidae**																
Ischaliasp.	MG609545	MG610105	MG609823		MG610743				MG612694	MG612383	MG612991	MG613303	MG613649	MG613972	MG614650	
**Melandryidae**																
Dircaeomorphasp.	MG609549	MG610108	MG609826	MG610421	MG610748	MG611031	MG611272	MG612122	MG612698	MG612388	MG612996	MG613307	MG613655	MG613978	MG614656	MG636885
gen.sp.	MG609632	MG610210	MG609921	MG610539	MG610868	MG611105	MG611371	MG612196	MG612809	MG612497	MG613101	MG613424	MG613764	MG614100	MG614762	MG636989
**Meloidae**																
Zonitiss.l.sp.	MG609471	MG610022	MG609744	MG610331	MG610656	MG610959	MG611191	MG612047	MG612611	MG612296	MG612915	MG613212	MG613558		MG614565	MG636801
Epicautasp.	MG609574	MG610135	MG609851	MG610452	MG610782	MG611053	MG611299	MG612140	MG612729	MG612421	MG613023	MG613339	MG613688	MG614011	MG614689	MG636911
**Mordellidae**																
Hoshihananomiasp.	MG609534	MG610092	MG609813	MG610404	MG610730		MG611257	MG612107	MG612682		MG612980	MG613288	MG613635	MG613959	MG614639	MG636870
**Mycetophagidae**																
Nototriphyllussp.	MG609414	MG609967	MG609680	MG610261	MG610593	MG610909	MG611136	MG612001	MG612549	MG612241	MG612861	MG613149	MG613483	MG613814	MG614496	MG636746
Mycetophagussp.	MG609543	MG610103	MG609821	MG610415	MG610741	MG611026	MG611267	MG612117	MG612693	MG612382	MG612989	MG613301	MG613647	MG613970	MG614648	MG636880
**Mycteridae**																
Trichosalpingussp.	MG609415	MG609968	MG609681	MG610262				MG612002	MG612550			MG613150	MG613484	MG613815	MG614497	
**Oedemeridae**																
Thelyphassasp.	MG609478			MG610339	MG610665		MG611199		MG612618	MG612305		MG613220	MG613567	MG613891	MG614574	MG636809
Pseudolycussp.	MG609479	MG610030	MG609752		MG610666	MG610967	MG611200	MG612053	MG612619	MG612306	MG612923	MG613221	MG613568	MG613892	MG614575	MG636810
Ditylussp.	MG609533	MG610091	MG609812	MG610403	MG610729	MG611017	MG611256	MG612106	MG612681	MG612369	MG612979	MG613287	MG613634	MG613958	MG614638	MG636869
Pyrochroidae																
Morpholycussp.	MG609421	MG609976	MG609689	MG610271	MG610600	MG610917	MG611144	MG612010	MG612559	MG612250		MG613157	MG613494	MG613823	MG614506	MG636754
MorpholycusmonilicornisLea	MG609485	MG610036	MG609758	MG610345	MG610672		MG611206	MG612059	MG612625	MG612312	MG612929	MG613227	MG613574	MG613898	MG614581	MG636816
Pseudopyrochroasp.	MG609523	MG610081	MG609804	MG610394	MG610719	MG611006	MG611249	MG612098	MG612672	MG612359	MG612970	MG613278	MG613623	MG613947	MG614628	MG636859
Eupyrochroainsignita	MG609528	MG610086	MG609807	MG610398	MG610723	MG611011	MG611252	MG612102	MG612676	MG612363	MG612974	MG613282	MG613628	MG613952	MG614632	MG636863
**Pythidae**																
Anaplopussp.	MG609486	MG610037	MG609759	MG610346	MG610673	MG610973	MG611207		MG612626	MG612313		MG613228	MG613575	MG613899	MG614582	MG636817
**Ripiphoridae**																
Trigonoderasp.	MG609424	MG609979	MG609692	MG610274	MG610603	MG610919	MG611147	MG612012	MG612562	MG612252	MG612871	MG613160	MG613497	MG613825	MG614509	MG636756
Rhipidioidessp.	MG609488											MG613229	MG613577			MG636818
**Salpingidae**																
OrphanotrophiumpallidipennisLea	MG609425	MG609980	MG609693	MG610275	MG610604	MG610920	MG611148	MG612013	MG612563	MG612253	MG612872	MG613161	MG613498	MG613826	MG614510	MG636757
Euryplatusdimidiatus	MG609489	MG610039	MG609761	MG610348	MG610675	MG610974	MG611209	MG612060	MG612628	MG612314	MG612931	MG613230	MG613578	MG613901	MG614584	MG636819
Orphanotrophiumsp.	MG609490	MG610040	MG609762	MG610349	MG610676	MG610975	MG611210	MG612061	MG612629	MG612315	MG612932	MG613231	MG613579	MG613902	MG614585	MG636820
Ocholissasp.	MG609491	MG610041	MG609763	MG610350	MG610677	MG610976	MG611211	MG612062	MG612630	MG612316	MG612933	MG613232	MG613580	MG613903	MG614586	MG636821
**Scraptiidae**																
Scraptiasp.			MG609923	MG610541								MG613426	MG613766	MG614102	MG614764	
**Tenebrionidae**																
Amarygmussp.	MG609500	MG610050	MG609773	MG610359	MG610684	MG610981	MG611219	MG612070	MG612640	MG612325	MG612942	MG613241	MG613590	MG613912	MG614596	MG636831
Cyphaleussp.	MG609501	MG610051	MG609774	MG610360	MG610685	MG610982	MG611220	MG612071	MG612641	MG612326	MG612943	MG613242	MG613591	MG613913	MG614597	MG636832
Adeliumsp.	MG609502	MG610052	MG609775		MG610686	MG610983	MG611221	MG612072		MG612327	MG612944	MG613243	MG613592	MG613914	MG614598	MG636833
Cillibussp.	MG609503	MG610053	MG609776	MG610361	MG610687	MG610984	MG611222	MG612073	MG612642	MG612328	MG612945	MG613244	MG613593	MG613915	MG614599	
Palorussp.		MG610054	MG609777	MG610362	MG610688	MG610985	MG611223	MG612074	MG612643	MG612329	MG612946	MG613245	MG613594	MG613916	MG614600	
Derispiasp.	MG609504	MG610055	MG609778	MG610363	MG610689	MG610986	MG611224	MG612075	MG612644	MG612330	MG612947	MG613246	MG613595	MG613917	MG614601	
Cossyphussp.	MG609505	MG610056	MG609779	MG610364	MG610690	MG610987	MG611225	MG612076	MG612645	MG612331	MG612948	MG613247	MG613596	MG613918	MG614602	MG636834
Tanychilussp.	MG609506	MG610057	MG609780		MG610691	MG610988	MG611226	MG612077	MG612646	MG612332	MG612949	MG613248	MG613597		MG614603	MG636835
Platydemasp.	MG609507	MG610058	MG609781	MG610365	MG610692	MG610989	MG611227	MG612078	MG612647	MG612333	MG612950	MG613249	MG613598	MG613919	MG614604	MG636836
Tyrtaeussp.	MG609508	MG610059	MG609782	MG610366	MG610693	MG610990	MG611228	MG612079		MG612334	MG612951	MG613250	MG613599	MG613920	MG614605	MG636837
Ecnolagriasp.	MG609509	MG610060	MG609783		MG610694	MG610991	MG611229	MG612080		MG612335	MG612952	MG613251	MG613600	MG613921	MG614606	

Chlorophilasp.	MG609542	MG610102	MG609820		MG610740	MG611025	MG611266	MG612116	MG612692	MG612381	MG612988	MG613300	MG613646	MG613969	MG614647	MG636879
Derispiasp.	MG609561	MG610122	MG609837	MG610433	MG610762		MG611283	MG612129	MG612712	MG612402	MG613009	MG613321	MG613668	MG613991	MG614670	
Cteniopinussp.		MG610123		MG610434	MG610763	MG611041	MG611284	MG612130	MG612713	MG612403	MG613010	MG613322	MG613669	MG613992	MG614671	MG636895
Strongyliumsp.	MG609621	MG610197	MG609907	MG610525	MG610852	MG611092	MG611356	MG612184	MG612797	MG612484	MG613088	MG613409	MG613747	MG614085	MG614749	MG636974
Cryphaeussp.		MG610198	MG609908	MG610526	MG610853	MG611093	MG611357	MG612185	MG612798	MG612485	MG613089	MG613410	MG613748	MG614086	MG614750	MG636975
Trictenotomasp.	MG609636	MG610213	MG609926	MG610544	MG610871	MG611109	MG611374	MG612199	MG612812	MG612500	MG613103	MG613429	MG613769	MG614105	MG614767	MG636992
**Ulodidae**																
Ulodessp.	MG609510	MG610063	MG609786	MG610370	MG610698	MG610993	MG611233	MG612084	MG612650	MG612339		MG613255	MG613604	MG613925	MG614608	
Meryxsp.	MG609511	MG610064	MG609787	MG610371	MG610699	MG610994	MG611234	MG612085	MG612651	MG612340	MG612954	MG613256	MG613605	MG613926	MG614609	MG636839
**Zopheridae**																
Monommasp.													MG613505			
Bitomasp.	MG609432	MG609984	MG609700	MG610282	MG610610	MG610924	MG611153	MG612019	MG612571	MG612260	MG612874	MG613169	MG613506	MG613833	MG614516	MG636763
Zopherosisgeorgei	MG609512		MG609788	MG610372	MG610700		MG611235				MG612955	MG613257	MG613606	MG613927	MG614610	

	CG4933	CG6512	CG7288	CG8545	Crc	dbo	Dhc98D	dnc	DopR2	dyl	Elp3	Emb	FBXO11	fz2	Gapdh2	Hem	Hmgs
**Outgroup**																	
Neochauliodesorientalis	MG615245	MG615832		MG617001		MG618149			MG619248	MG619588		MG620163		MG620755		MG621621	MG621972
Ascalohybrissubjacens	MG615244	MG615831	MG616106	MG617000	MG617864	MG618148	MG618413		MG619247	MG619587	MG619888	MG620162	MG620449	MG620754	MG621040	MG621620	MG621971
Dichochrysaformosana	MG615243	MG615830		MG616999	MG617863	MG618147			MG619246	MG619586	MG619887	MG620161	MG620448	MG620753	MG621039	MG621619	MG621970
Myrmeleonsp.	MG615242	MG615829	MG616105	MG616998	MG617862	MG618146	MG618412		MG619245	MG619585	MG619886	MG620160	MG620447	MG620752	MG621038	MG621618	MG621969
**Adephaga / Carabidae**																	
Dischissussp.	MG615255	MG615841	MG616114		MG617873	MG618158	MG618424	MG618931	MG619259	MG619599	MG619899	MG620174	MG620460	MG620766	MG621049	MG621633	MG621984
Carabussp.	MG615332	MG615898	MG616176	MG617070	MG617948	MG618203	MG618495	MG619000	MG619332	MG619672	MG619956	MG620238	MG620525	MG620829	MG621110	MG621710	MG622064
Cicindelachinensis	MG615333		MG616177	MG617071		MG618204	MG618496	MG619001	MG619333	MG619673		MG620239	MG620526	MG620830	MG621111	MG621711	MG622065
Omoglymmiussp.	MG615349		MG616190	MG617086	MG617959		MG618509	MG619015	MG619349	MG619689	MG619969	MG620253	MG620542	MG620844	MG621126	MG621728	MG622082
Clivinasp.	MG615394	MG615949	MG616226	MG617116			MG618548	MG619051	MG619392	MG619731		MG620290	MG620578	MG620883	MG621161	MG621771	MG622123
Pheropsophussp.	MG615395	MG615950	MG616227	MG617117	MG617993	MG618245	MG618549	MG619052	MG619393	MG619732		MG620291	MG620579	MG620884	MG621162	MG621772	MG622124
Lebiacoelestis	MG615397	MG615952	MG616229	MG617119				MG619054	MG619395	MG619734	MG620003	MG620292	MG620581			MG621774	MG622126
Pentagonicaruficollis	MG615398	MG615953		MG617120	MG617995		MG618551	MG619055	MG619396	MG619735	MG620004	MG620293	MG620582	MG620886	MG621164	MG621775	MG622127
Elaphrussp.	MG615425	MG615973		MG617146	MG618018		MG618574	MG619080	MG619423	MG619762	MG620025	MG620316	MG620604	MG620914	MG621186	MG621801	MG622156
gen.sp.	MG615427	MG615974	MG616245	MG617148		MG618267	MG618576	MG619081	MG619425	MG619764	MG620027	MG620318	MG620605		MG621188		MG622158
**Dytiscidae**																	
Agabussp.	MG615341	MG615906		MG617078		MG618210		MG619008	MG619340	MG619680		MG620245	MG620533	MG620837	MG621118	MG621719	MG622073
Laccophilusdifficilis	MG615342	MG615907	MG616185	MG617079	MG617955	MG618211	MG618504	MG619009	MG619341	MG619681	MG619964		MG620534	MG620838	MG621119	MG621720	MG622074
Eretesgriseus	MG615343					MG618212		MG619010	MG619342	MG619682		MG620246	MG620535	MG620839	MG621120	MG621721	MG622075
Rhantussuturalis	MG615344	MG615908	MG616186	MG617080		MG618213		MG619011	MG619343	MG619683	MG619965	MG620247	MG620536	MG620840	MG621121	MG621722	MG622076
Hyphydrusdetectus	MG615345			MG617081		MG618214			MG619344	MG619684	MG619966	MG620248	MG620537	MG620841	MG621122	MG621723	MG622077
**Gyrinidae**																	
Orectochilussp.	MG615275	MG615858		MG617027	MG617893			MG618950	MG619281	MG619621	MG619915		MG620475		MG621068		MG622006
**Haliplidae**																	
Peltodytessp.	MG615350	MG615913	MG616191		MG617960	MG618218	MG618510	MG619016	MG619350	MG619690	MG619970	MG620254	MG620543	MG620845	MG621127	MG621729	MG622083
**Noteridae**																	
gen.sp.	MG615127		MG616014	MG616888	MG617752		MG618311	MG618844	MG619133	MG619475	MG619802	MG620065	MG620355		MG620957	MG621508	MG621852
Canthydrussp.		MG615909	MG616187	MG617082	MG617956	MG618215	MG618505	MG619012	MG619345	MG619685	MG619967	MG620249	MG620538		MG621123	MG621724	MG622078
**Archostemata/ Cupedidae**																	
Tenomergasp.	MG615347	MG615911	MG616189	MG617084		MG618217	MG618507		MG619347	MG619687		MG620251	MG620540			MG621726	MG622080
**Myxophaga / Torridincolidae**																	
Satoniusschoenmanni	MG615346	MG615910	MG616188	MG617083	MG617957	MG618216	MG618506	MG619013	MG619346	MG619686	MG619968	MG620250	MG620539	MG620842	MG621124	MG621725	MG622079
**Bostrichoidea /Bostrichidae**																	
Lyctussp.	MG615154	MG615759	MG616030	MG616914	MG617777	MG618081	MG618334		MG619160	MG619501	MG619822	MG620085		MG620664		MG621533	MG621879
Polycaonsp.	MG615271		MG616125	MG617022	MG617888		MG618438		MG619276	MG619616	MG619910			MG620778		MG621650	MG622001
**Dermestidae**																	
Dermestessp.	MG615169	MG615770		MG616929	MG617794	MG618092	MG618348	MG618872	MG619176	MG619516		MG620096	MG620388	MG620679	MG620987	MG621547	MG621897
Evorineasp.	MG615170	MG615771	MG616043	MG616930	MG617795	MG618093	MG618349		MG619177			MG620097	MG620389	MG620680		MG621548	MG621898
Orphinussp.	MG615357	MG615920	MG616197	MG617093	MG617967	MG618222	MG618517	MG619022	MG619357	MG619697	MG619974	MG620260	MG620549	MG620851	MG621133	MG621736	MG622090
**Ptinidae**																	
Ptinussp.						MG618063	MG618315	MG618849		MG619479		MG620067		MG620648	MG620961	MG621512	MG621857
Dorcatomasp.	MG615131	MG615741		MG616894	MG617758	MG618064	MG618316		MG619138	MG619480	MG619806		MG620360	MG620649	MG620962	MG621513	MG621858
Ptinussp.	MG615359			MG617095				MG619024	MG619359	MG619699		MG620262		MG620853		MG621738	MG622092
Hedobiasp.	MG615417	MG615969				MG618260		MG619073	MG619416	MG619755		MG620311		MG620906		MG621795	MG622149
**Buprestoidea / Buprestidae**																	
Coroebussp.	MG615260		MG616117	MG617011	MG617878		MG618428	MG618936	MG619264	MG619604		MG620179	MG620465	MG620771	MG621054	MG621638	MG621989

Dicercasp.	MG615315			MG617058	MG617932	MG618195	MG618480	MG618987	MG619318	MG619657	MG619943	MG620225	MG620510	MG620815		MG621696	MG622048
Trachyssp.	MG615316	MG615885	MG616166		MG617933		MG618481	MG618988		MG619658	MG619944	MG620226	MG620511	MG620816	MG621100	MG621697	MG622049
**Byrrhoidea / Byrrhidae**																	
Notolioonsp.	MG615098			MG616865	MG617724	MG618040	MG618283	MG618825	MG619105	MG619447		MG620042	MG620335	MG620624		MG621482	MG621824
Microchaetessp.	MG615158		MG616035	MG616918	MG617782	MG618084	MG618338	MG618865	MG619164	MG619505	MG619825	MG620089	MG620380	MG620668	MG620978	MG621537	MG621885
Cytilussp.	MG615289			MG617038	MG617907		MG618454	MG618964	MG619294	MG619633		MG620203	MG620488	MG620791		MG621670	MG622021
**Callirhipidae**																	
Ennometessp.	MG615099	MG615715			MG617725		MG618284	MG618826	MG619106	MG619448		MG620043	MG620336	MG620625	MG620935	MG621483	MG621825
Simianussp.	MG615348	MG615912		MG617085	MG617958		MG618508	MG619014	MG619348	MG619688		MG620252	MG620541	MG620843	MG621125	MG621727	MG622081
**Dryopidae**																	
Helichussp.	MG615108	MG615723		MG616874	MG617733		MG618292		MG619114	MG619456		MG620050	MG620343		MG620942	MG621492	MG621834
Pachyparnussp.	MG615393	MG615948		MG617115	MG617992		MG618547		MG619391	MG619730		MG620289	MG620577	MG620882		MG621770	MG622122
Helichussp.	MG615409	MG615962		MG617132			MG618560	MG619066	MG619409	MG619748		MG620304	MG620593	MG620899	MG621173	MG621788	MG622141
**Elmidae**																	
Graphelmissp.	MG615173	MG615774	MG616046	MG616933	MG617798		MG618352	MG618875	MG619180	MG619519		MG620100	MG620392	MG620683	MG620990	MG621551	MG621901
Stetholussp.	MG615174		MG616047	MG616934	MG617799		MG618353	MG618876	MG619181	MG619520	MG619835	MG620101	MG620393	MG620684		MG621552	MG621902
Stenelmissp.	MG615276			MG617028	MG617894		MG618442	MG618951	MG619282	MG619622			MG620476			MG621655	MG622007
**Eulichadidae**																	
Eulichassp.	MG615265	MG615849	MG616121	MG617016	MG617882		MG618433	MG618940	MG619270	MG619610		MG620184	MG620469	MG620774		MG621644	MG621995
**Heteroceridae**																	
Heterocerussp.	MG615324	MG615890	MG616171	MG617064	MG617940	MG618198	MG618489	MG618993	MG619325	MG619665	MG619950	MG620231	MG620517	MG620823	MG621104	MG621703	MG622056
**Chelonariidae**																	
Chelonariumsp.	MG615102	MG615717		MG616868	MG617727		MG618286		MG619109	MG619451		MG620045	MG620339	MG620628		MG621486	MG621828
**Limnichidae**																	
Byrrhinussp.	MG615192	MG615788	MG616059	MG616949	MG617815	MG618105	MG618371	MG618887	MG619198	MG619537	MG619847	MG620115	MG620407	MG620702	MG621002	MG621569	MG621920
Pelocharessp.	MG615279	MG615861	MG616131	MG617032	MG617898	MG618174	MG618444	MG618955	MG619286		MG619918	MG620195	MG620480	MG620783	MG621072	MG621659	MG622011
Cephalobyrrhussp.	MG615314	MG615884	MG616165	MG617057	MG617931	MG618194	MG618479	MG618986	MG619317	MG619656	MG619942	MG620224	MG620509	MG620814	MG621099	MG621695	MG622047
Limnichussp.	MG615426			MG617147	MG618019	MG618266	MG618575		MG619424	MG619763	MG620026	MG620317		MG620915	MG621187		MG622157
**Psephenidae**																	
Sclerocyphonsp.	MG615130			MG616892	MG617756	MG618062		MG618848	MG619137					MG620647			MG621856
Schinostethussp.	MG615288	MG615866	MG616141	MG617037	MG617906	MG618178	MG618453	MG618963	MG619293	MG619632	MG619926	MG620202	MG620487	MG620790	MG621081	MG621669	MG622020
Schinostethussp.	MG615312	MG615882	MG616163	MG617055	MG617929	MG618193	MG618477	MG618984	MG619315	MG619655	MG619940	MG620222	MG620507	MG620812	MG621098	MG621693	MG622045
Mataeopsephussp.	MG615313	MG615883	MG616164	MG617056	MG617930		MG618478	MG618985	MG619316		MG619941	MG620223	MG620508	MG620813		MG621694	MG622046
**Ptilodactylidae**																	
Ptilodactylasp.	MG615257	MG615843	MG616115	MG617008	MG617875	MG618160	MG618425	MG618933	MG619261	MG619601	MG619900	MG620176	MG620462	MG620768	MG621051	MG621635	MG621986
Epilichassp.	MG615311	MG615881	MG616162	MG617054	MG617928	MG618192	MG618476	MG618983	MG619314	MG619654		MG620221	MG620506	MG620811		MG621692	MG622044
**Cleroidea /Acanthocnemidae**																	
Acanthocnemusnigricans	MG615147	MG615751	MG616026	MG616907	MG617770	MG618075			MG619153	MG619493	MG619817	MG620079	MG620371	MG620656		MG621526	MG621872
**Biphyllidae**																	
Althaesiasp.	MG615095	MG615713	MG615991	MG616862	MG617721	MG618038		MG618823	MG619102	MG619445		MG620040	MG620333		MG620933	MG621480	MG621821
Biphyllussp.		MG615757		MG616912	MG617775	MG618079	MG618332	MG618862	MG619158	MG619499	MG619821	MG620084	MG620375	MG620662		MG621532	MG621877
**Byturidae**																	
Haematoidessp.		MG615859	MG616129	MG617029	MG617895		MG618443	MG618952	MG619283	MG619623	MG619916	MG620192	MG620477		MG621069	MG621656	MG622008
**Cleridae**																	
Tenerussp.	MG615164														MG620982		MG621892
Necrobiasp.	MG615165			MG616925	MG617789			MG618869	MG619171	MG619511		MG620094	MG620386	MG620674	MG620983	MG621542	MG621893
Xenorthriussp.	MG615254			MG617006	MG617872				MG619258	MG619598	MG619898	MG620173	MG620459	MG620765		MG621632	MG621983
Cladiscussp.	MG615404			MG617127					MG619404	MG619743		MG620299	MG620588	MG620894		MG621783	MG622136
Stenocallimerussp.	MG615405			MG617128		MG618252		MG619062	MG619405	MG619744		MG620300	MG620589	MG620895		MG621784	MG622137

Allochotessp.	MG615406			MG617129	MG618003			MG619063	MG619406	MG619745	MG620011	MG620301	MG620590	MG620896	MG621170	MG621785	MG622138
**Melyridae**																	
Dasytess.l.sp.	MG615195			MG616952	MG617818	MG618108				MG619540	MG619849	MG620117	MG620410	MG620705	MG621005	MG621572	MG621923
Dicranolaiusbellulus	MG615196	MG615791	MG616061	MG616953		MG618109		MG618890	MG619201	MG619541	MG619850	MG620118	MG620411	MG620706	MG621006	MG621573	MG621924
Carphurussp.	MG615197		MG616062	MG616954	MG617819	MG618110		MG618891	MG619202	MG619542	MG619851	MG620119	MG620412	MG620707	MG621007	MG621574	MG621925
gen.sp.	MG615247			MG617002	MG617867	MG618152	MG618416		MG619251		MG619892	MG620166	MG620453		MG621044	MG621624	MG621975
Carphurussp.	MG615358	MG615921	MG616198	MG617094		MG618223	MG618518	MG619023	MG619358	MG619698	MG619975	MG620261	MG620550	MG620852	MG621134	MG621737	MG622091
**Prionoceridae**																	
Idgiasp.	MG615277			MG617030	MG617896			MG618953	MG619284	MG619624		MG620193	MG620478		MG621070	MG621657	MG622009
**Thanerocleridae**																	
Isoclerussp.	MG615140			MG616902					MG619147	MG619488	MG619813					MG621521	MG621867
**Trogossitidae**																	
Larinotusumblicatus	MG615143			MG616905	MG617768	MG618072			MG619150			MG620076	MG620369	MG620655	MG620970	MG621524	MG621869
Rentonellumsp.	MG615144		MG616024						MG619151	MG619491		MG620077					MG621870
Ancyronasp.				MG616994						MG619579	MG619882	MG620154	MG620443	MG620746	MG621034	MG621612	MG621964
Parapeltisaustralicum	MG615237			MG616995	MG617858	MG618143			MG619240	MG619580		MG620155		MG620747		MG621613	MG621965
Leperinasp.	MG615238			MG616996					MG619241	MG619581		MG620156		MG620748		MG621614	MG621966
Thymalussp.	MG615264			MG617015		MG618165			MG619269	MG619609	MG619905		MG620468			MG621643	MG621994
**Coccinelloidea /Anamorphidae**																	
Papuellasp.	MG615175	MG615775	MG616048	MG616935	MG617800	MG618096	MG618354		MG619182	MG619521	MG619836	MG620102	MG620394	MG620685	MG620991	MG621553	MG621903
**Bothrideridae**																	
Deretaphrussp.			MG616032		MG617779												MG621881
Ascetoderessp.	MG615157	MG615762	MG616033	MG616917	MG617781		MG618337	MG618864	MG619163	MG619504	MG619824	MG620088	MG620379	MG620667	MG620977	MG621536	MG621883
**Cerylonidae**																	
Philothermussp.	MG615101	MG615716	MG615994	MG616867					MG619108	MG619450		MG620044	MG620338	MG620627	MG620937	MG621485	MG621827
Ostomopsissp.		MG615764	MG616037	MG616921	MG617785		MG618341		MG619167		MG619826		MG620382	MG620671	MG620979	MG621539	MG621888
**Coccinellidae**																	
Chnootribasp.	MG615083	MG615703	MG615982		MG617711	MG618028	MG618271		MG619090	MG619434	MG619773	MG620032	MG620322	MG620614	MG620923	MG621469	MG621810
Harmoniaoctomaculata	MG615084	MG615704	MG615983	MG616853	MG617712	MG618029	MG618272		MG619091	MG619435	MG619774	MG620033	MG620323	MG620615	MG620924	MG621470	MG621811
Exochomussp.	MG615085	MG615705	MG615984			MG618030	MG618273		MG619092	MG619436	MG619775		MG620324	MG620616		MG621471	MG621812
Rhizobiussp.	MG615086	MG615706	MG615985		MG617713	MG618031	MG618274		MG619093	MG619437	MG619776	MG620034	MG620325	MG620617	MG620925	MG621472	MG621813
Ortaliasp.	MG615087	MG615707	MG615986	MG616854	MG617714	MG618032	MG618275	MG618819	MG619094	MG619438	MG619777	MG620035	MG620326		MG620926	MG621473	MG621814
Microfreudeasp.	MG615088			MG616855		MG618033			MG619095		MG619778						
Sasajiscymnussp.	MG615089	MG615708	MG615987	MG616856	MG617715	MG618034	MG618276		MG619096	MG619439	MG619779	MG620036	MG620327	MG620618	MG620927	MG621474	MG621815
Stethorussp.	MG615090	MG615709	MG615988	MG616857	MG617716	MG618035	MG618277		MG619097	MG619440	MG619780	MG620037	MG620328	MG620619	MG620928	MG621475	MG621816
**Corylophidae**																	
Sericoderussp.	MG615104	MG615719	MG615996	MG616870	MG617729	MG618042	MG618288	MG618828	MG619111	MG619453	MG619787	MG620047	MG620341	MG620629	MG620939	MG621488	MG621830
Priamimasp.		MG615767	MG616040	MG616926	MG617790	MG618089	MG618345		MG619172	MG619512	MG619830			MG620675	MG620984	MG621543	MG621894
Periptyctussp.	MG615166			MG616927	MG617791	MG618090			MG619173	MG619513	MG619831			MG620676		MG621544	MG621895
Orthoperussp.	MG615422	MG615971	MG616243	MG617143	MG618015	MG618263	MG618571	MG619077	MG619420	MG619760	MG620022	MG620314	MG620601	MG620911	MG621183	MG621799	MG622153
**Discolomatidae**																	
Aphanocephalussp.	MG615107	MG615722	MG615999	MG616873	MG617732	MG618045	MG618291				MG619789	MG620049		MG620631		MG621491	MG621833
**Endomychidae**																	
Cyclotomasp.	MG615109	MG615724	MG616000	MG616875	MG617734		MG618293	MG618831	MG619115	MG619457	MG619790	MG620051		MG620632	MG620943	MG621493	MG621835
Holoparamecussp.	MG615176		MG616049	MG616936	MG617801	MG618097	MG618355		MG619183	MG619522	MG619837	MG620103	MG620395	MG620686	MG620992	MG621554	MG621904
Encymonimmaculatus	MG615177	MG615776		MG616937	MG617802		MG618356		MG619184	MG619523		MG620104	MG620396	MG620687		MG621555	MG621905
Stenotarsussp.	MG615178	MG615777			MG617803	MG618098	MG618357	MG618877	MG619185	MG619524			MG620397	MG620688		MG621556	MG621906
Sinocymbachussp.	MG615249	MG615835			MG617869	MG618154	MG618418		MG619253	MG619592	MG619894	MG620168	MG620455	MG620759		MG621626	MG621977
**Euxestidae**																	

Hypodacnellasp.	MG615100		MG615993	MG616866	MG617726		MG618285		MG619107	MG619449			MG620337	MG620626	MG620936	MG621484	MG621826
**Latridiidae**																	
Enicmussp.	MG615117			MG616881	MG617741	MG618051	MG618301		MG619123	MG619465	MG619795	MG620058			MG620949	MG621499	MG621843
Corticariasp.	MG615118	MG615729			MG617742		MG618302	MG618838						MG620638			MG621844
Melanophthalmasp.	MG615191	MG615787		MG616948	MG617814		MG618370			MG619536	MG619846			MG620701			MG621919
**Murmidiidae**																	
Murmidiusovalis	MG615160			MG616920	MG617784	MG618085	MG618340		MG619166	MG619507		MG620090		MG620670			MG621887
**Teredidae**																	
Xylariophilussp.	MG615097			MG616864	MG617723				MG619104					MG620623			MG621823
Xylariophilussp.	MG615155	MG615760	MG616031	MG616915	MG617778	MG618082	MG618335		MG619161	MG619502	MG619823	MG620086	MG620377	MG620665		MG621534	MG621880
Teredolaemussp.	MG615156	MG615761		MG616916	MG617780		MG618336		MG619162	MG619503		MG620087	MG620378	MG620666		MG621535	MG621882
**Cucujoidea/Alexiidae**																	
Sphaerosomasp.	MG615092	MG615710	MG615989	MG616859	MG617718	MG618036	MG618279		MG619099	MG619442	MG619782		MG620330	MG620620	MG620930	MG621477	MG621818
**Boganiidae**																	
Paracucujusrostratus	MG615096	MG615714	MG615992	MG616863	MG617722	MG618039	MG618282	MG618824	MG619103	MG619446	MG619785	MG620041	MG620334	MG620622	MG620934	MG621481	MG621822
**Cryptophagidae**																	
Micrambinasp.	MG615105	MG615720	MG615997	MG616871	MG617730	MG618043	MG618289	MG618829	MG619112	MG619454	MG619788	MG620048	MG620342	MG620630	MG620940	MG621489	MG621831
Cureliussp.	MG615407	MG615960	MG616235	MG617130	MG618004	MG618253	MG618558	MG619064	MG619407	MG619746	MG620012	MG620302	MG620591	MG620897	MG621171	MG621786	MG622139
**Cucujidae**																	
Platisussp.	MG615106	MG615721	MG615998	MG616872	MG617731	MG618044	MG618290	MG618830	MG619113	MG619455					MG620941	MG621490	MG621832
Pediacusmajor	MG615167	MG615768	MG616041	MG616928	MG617792	MG618091	MG618346	MG618870	MG619174	MG619514	MG619832			MG620677	MG620985	MG621545	
**Cybocephalidae**																	
Cybocephalussp.	MG615168	MG615769	MG616042		MG617793		MG618347	MG618871	MG619175	MG619515	MG619833	MG620095	MG620387	MG620678	MG620986	MG621546	MG621896
**Erotylidae**																	
Thallissp.	MG615179	MG615778		MG616938	MG617804		MG618358	MG618878	MG619186	MG619525	MG619838	MG620105		MG620689	MG620993	MG621557	MG621907
Anadastussp.	MG615180	MG615779		MG616939		MG618099	MG618359	MG618879	MG619187	MG619526		MG620106		MG620690		MG621558	MG621908
Episcaphulasp.	MG615181	MG615780	MG616050	MG616940	MG617805		MG618360	MG618880	MG619188	MG619527		MG620107		MG620691		MG621559	MG621909
Cryptophilussp.	MG615182	MG615781	MG616051	MG616941		MG618100	MG618361	MG618881	MG619189	MG619528	MG619839	MG620108	MG620398	MG620692	MG620994	MG621560	MG621910
Episcaphasp.	MG615263	MG615848		MG617014	MG617881		MG618432	MG618939	MG619268	MG619608	MG619904	MG620183		MG620773	MG621058	MG621642	MG621993
Tetraphalasp.	MG615268	MG615852		MG617019	MG617885	MG618168	MG618436	MG618943	MG619273	MG619613	MG619907	MG620187		MG620776	MG621061	MG621647	MG621998
**Helotidae**																	
Neohelotasp.	MG615248	MG615834	MG616110	MG617003	MG617868	MG618153	MG618417	MG618925	MG619252	MG619591	MG619893	MG620167	MG620454	MG620758	MG621045	MG621625	MG621976
**Hobartiidae**																	
Hydnobioidessp.	MG615186	MG615783	MG616055	MG616944	MG617809	MG618103	MG618365		MG619193	MG619532	MG619842	MG620111	MG620402	MG620696	MG620997	MG621564	MG621914
**Kateretidae**																	
Notobrachypterussp.	MG615116	MG615728		MG616880		MG618050	MG618300	MG618837	MG619122	MG619464		MG620057			MG620948	MG621498	MG621842
**Laemophloeidae**																	
Laemophloeussp.	MG615190	MG615786	MG616058	MG616947	MG617813	MG618104	MG618369	MG618886	MG619197	MG619535	MG619845	MG620114	MG620406	MG620700	MG621001	MG621568	MG621918
Cryptolestessp.	MG615408	MG615961	MG616236	MG617131	MG618005	MG618254	MG618559	MG619065	MG619408	MG619747	MG620013	MG620303	MG620592	MG620898	MG621172	MG621787	MG622140
**Monotomidae**																	
Mimemodessp.	MG615122	MG615733	MG616009	MG616884	MG617747	MG618054	MG618306	MG618840	MG619128	MG619470	MG619799	MG620061	MG620352		MG620953	MG621503	
Monotomopsissp.	MG615198	MG615792	MG616063	MG616955	MG617820	MG618111	MG618373	MG618892	MG619203	MG619543	MG619852	MG620120	MG620413	MG620708		MG621575	MG621926
Rhizophagussp.	MG615199	MG615793	MG616064	MG616956	MG617821	MG618112	MG618374	MG618893	MG619204	MG619544	MG619853	MG620121	MG620414	MG620709	MG621008	MG621576	MG621927
Thionesp.	MG615420	MG615970	MG616241	MG617141	MG618013	MG618261	MG618569	MG619075	MG619418	MG619758	MG620020	MG620312	MG620599	MG620909	MG621181	MG621797	MG622151
**Myraboliidae**																	
Myraboliasp.	MG615125	MG615736	MG616012	MG616886	MG617750	MG618057	MG618309	MG618842	MG619131	MG619473		MG620063		MG620643	MG620956	MG621506	MG621850
**Nitidulidae**																	
Brachypeplussp.	MG615201	MG615795	MG616066	MG616958	MG617822	MG618113	MG618376	MG618895	MG619205	MG619546	MG619854	MG620123	MG620415	MG620711	MG621009	MG621578	MG621929
Pallodessp.	MG615202		MG616067	MG616959	MG617823	MG618114	MG618377		MG619206	MG619547	MG619855	MG620124	MG620416	MG620712		MG621579	MG621930

Urophorussp.	MG615292	MG615869	MG616144		MG617909	MG618180	MG618457	MG618966	MG619296	MG619636		MG620205		MG620794	MG621083	MG621673	MG622024
Carpophilussp.	MG615410	MG615963	MG616237	MG617133	MG618006	MG618255	MG618561	MG619067	MG619410	MG619749		MG620305		MG620900	MG621174	MG621789	MG622142
Glischrochilusparvipustulatus	MG615411	MG615964	MG616238	MG617134		MG618256	MG618562	MG619068	MG619411	MG619750	MG620014	MG620306		MG620901	MG621175	MG621790	MG622143
**Passandridae**																	
Passandrasp.	MG615205		MG616070	MG616961	MG617826	MG618115	MG618380	MG618898	MG619209	MG619550		MG620125	MG620417	MG620715		MG621582	MG621933
**Phalacridae**																	
gen.sp.	MG615128	MG615738		MG616889	MG617753	MG618059	MG618312	MG618845	MG619134	MG619476	MG619803		MG620356	MG620645	MG620958	MG621509	MG621853
Phalacrinussp.	MG615206	MG615797	MG616071	MG616962	MG617827	MG618116	MG618381	MG618899	MG619210	MG619551	MG619857		MG620418	MG620716	MG621011	MG621583	MG621934
Olibrussp.	MG615351	MG615914	MG616192	MG617087	MG617961	MG618219	MG618511	MG619017	MG619351	MG619691	MG619971		MG620544		MG621128	MG621730	MG622084
**Phloeostichidae**																	
Hymaeasp.	MG615207	MG615798	MG616072	MG616963	MG617828	MG618117	MG618382	MG618900	MG619211	MG619552	MG619858	MG620126	MG620419	MG620717		MG621584	MG621935
Rhopalobrachiumcrowsoni	MG615208	MG615799	MG616073	MG616964	MG617829	MG618118	MG618383	MG618901	MG619212	MG619553	MG619859	MG620127		MG620718	MG621012	MG621585	MG621936
**Propalticidae**																	
Propalticussp.		MG615740	MG616016	MG616891	MG617755	MG618061	MG618314	MG618847	MG619136	MG619478	MG619805		MG620358		MG620960	MG621511	MG621855
**Protocucujidae**																	
Ericmodescostatus	MG615209	MG615800	MG616074	MG616965	MG617830		MG618384	MG618902	MG619213		MG619860	MG620128		MG620719		MG621586	MG621937
**Silvanidae**																	
Uleiotasp.	MG615137	MG615746		MG616900	MG617764			MG618855	MG619144	MG619486				MG620653	MG620967	MG621519	MG621864
Cryptamorphasp.	MG615224	MG615815		MG616980	MG617844	MG618131	MG618397		MG619227	MG619567	MG619872			MG620733		MG621600	MG621951
Silvanoprusscuticollis		MG615816	MG616089	MG616981	MG617845	MG618132		MG618915				MG620141				MG621601	
Psammoecussp.	MG615327	MG615893	MG616173	MG617066	MG617943	MG618200	MG618490	MG618995	MG619327	MG619667	MG619952	MG620233	MG620520		MG621106	MG621705	MG622059
Psammoecussp.	MG615352	MG615915	MG616193	MG617088	MG617962	MG618220	MG618512	MG619018	MG619352	MG619692	MG619972	MG620255	MG620545	MG620846	MG621129	MG621731	MG622085
**Sphindidae**																	
Aspidiphorussp.	MG615138			MG616901	MG617765	MG618070	MG618322		MG619145		MG619812	MG620072	MG620366		MG620968		MG621865
**Curculionoidea/Anthribidae**																	
Acorynussp.	MG615251	MG615837	MG616111				MG618420	MG618927		MG619594	MG619895			MG620761		MG621628	MG621979
Ozotomerussp.	MG615280	MG615862	MG616132				MG618445	MG618956			MG619919					MG621660	MG622012
Xylinadasp.	MG615290	MG615867	MG616142				MG618455			MG619634				MG620792		MG621671	MG622022
Peribathyssp.	MG615334	MG615899	MG616178	MG617072			MG618497	MG619002		MG619674	MG619957		MG620527	MG620831	MG621112	MG621712	MG622066
**Attelabidae**																	
Phymatapoderussp.	MG615287		MG616140		MG617905			MG618962		MG619631			MG620486		MG621080	MG621668	
Involvulussp.	MG615306		MG616158		MG617924	MG618188	MG618471	MG618979		MG619651	MG619936	MG620217	MG620503	MG620806		MG621688	MG622039
Byctiscussp.	MG615308		MG616159	MG617052	MG617925	MG618189	MG618473		MG619311	MG619652	MG619938	MG620218	MG620504	MG620808	MG621095	MG621689	MG622041
Paratrachelophorussp.	MG615403	MG615959	MG616234	MG617126	MG618002	MG618251		MG619061		MG619742	MG620010			MG620893	MG621169	MG621782	MG622135
**Belidae**																	
Rhinotiasp.	MG615152	MG615756				MG618078	MG618331			MG619498	MG619820	MG620083		MG620661	MG620976	MG621531	MG621876
**Brentidae**																	
Apions.l.sp.			MG616034			MG618083											MG621884
Baryrhynchussp.	MG615281		MG616133				MG618446	MG618957		MG619626	MG619920	MG620196		MG620784	MG621073	MG621661	MG622013
Apions.l.sp.	MG615307						MG618472	MG618980	MG619310		MG619937			MG620807			MG622040
Cylassp.	MG615412																MG622144
**Curculionidae**																	
Episomussp.		MG615839				MG618156	MG618422	MG618929	MG619256	MG619596	MG619897	MG620171	MG620457	MG620763	MG621048	MG621630	MG621981
gen.sp.	MG615282		MG616134		MG617899	MG618175	MG618447		MG619287			MG620197	MG620481	MG620785	MG621074	MG621662	MG622014
Xylosandrussp.		MG615864	MG616137		MG617902	MG618177	MG618450	MG618960	MG619290	MG619629	MG619923	MG620200		MG620787	MG621077	MG621665	MG622017
Curculiosp.			MG616232		MG618001	MG618249	MG618556	MG619059	MG619402	MG619740		MG620297		MG620891	MG621167	MG621780	MG622133
Peribleptussp.			MG616233			MG618250	MG618557	MG619060	MG619403	MG619741	MG620009	MG620298		MG620892	MG621168	MG621781	MG622134
**Nemonychidae**																	
AragomaceruniformisKuschel	MG615200	MG615794	MG616065	MG616957			MG618375	MG618894		MG619545		MG620122		MG620710		MG621577	MG621928

**Dasciloidea/ Dascillidae**																	
Dascillussp.	MG615267	MG615851		MG617018	MG617884	MG618167	MG618435	MG618942	MG619272	MG619612		MG620186	MG620471		MG621060	MG621646	MG621997
Metallidascillussp.	MG615413	MG615965	MG616239	MG617135	MG618007	MG618257	MG618563	MG619069	MG619412	MG619751	MG620015	MG620307	MG620594	MG620902	MG621176	MG621791	MG622145
**Rhipiceridae**																	
Oligorhipisbifossata	MG615134	MG615743		MG616897	MG617761	MG618067	MG618319	MG618852	MG619141	MG619483	MG619809	MG620070	MG620363	MG620652	MG620964	MG621516	MG621861
**Derodontoidea /Derodontidae**																	
Derodontussp.	MG615171	MG615772	MG616044	MG616931	MG617796	MG618094	MG618350	MG618873	MG619178	MG619517		MG620098	MG620390	MG620681	MG620988	MG621549	MG621899
Derolathrussp.	MG615115		MG616006		MG617740	MG618049	MG618299	MG618836	MG619121	MG619463	MG619794		MG620347	MG620637	MG620947	MG621497	MG621841
**Jacobsoniidae**																	
Derolathrussp.	MG615189		MG616057		MG617812		MG618368		MG619196		MG619844		MG620405	MG620699	MG621000	MG621567	MG621917
**Nosodendridae**																	
Nosodendronsp.	MG615126	MG615737	MG616013	MG616887	MG617751	MG618058	MG618310	MG618843	MG619132	MG619474	MG619801	MG620064	MG620354	MG620644		MG621507	MG621851
**Elateroidea/Artematopodidae**																	
Artematopussp.	MG615094	MG615712		MG616861	MG617720		MG618281	MG618822	MG619101	MG619444	MG619784	MG620039	MG620332	MG620621	MG620932	MG621479	MG621820
Eurypogonsp.	MG615415	MG615967	MG616240	MG617137	MG618009		MG618565	MG619071	MG619414	MG619753	MG620017	MG620309	MG620596	MG620904		MG621793	MG622147
**Cantharidae**																	
Heteromastixsp.	MG615159	MG615763	MG616036	MG616919	MG617783		MG618339	MG618866	MG619165	MG619506			MG620381	MG620669		MG621538	MG621886
Themussp.	MG615246	MG615833	MG616108		MG617865	MG618150	MG618414	MG618924	MG619249	MG619589	MG619890	MG620164	MG620451	MG620756	MG621042	MG621622	MG621973
Themussp.	MG615297		MG616149	MG617044	MG617915	MG618185			MG619302	MG619642	MG619930	MG620210	MG620495	MG620799	MG621089	MG621679	MG622030
Prothemussp.	MG615298	MG615874	MG616150	MG617045	MG617916		MG618463	MG618972	MG619303	MG619643	MG619931		MG620496	MG620800	MG621090	MG621680	MG622031
Ichthyurusbourgeoisi	MG615299	MG615875	MG616151		MG617917		MG618464		MG619304	MG619644	MG619932		MG620497	MG620801	MG621091	MG621681	MG622032
Laemoglyptussp.	MG615380		MG616213		MG617979		MG618534	MG619039	MG619378	MG619717	MG619991	MG620280	MG620564	MG620869	MG621148	MG621757	MG622110
Malthinussp.	MG615381	MG615936	MG616214	MG617104	MG617980	MG618236	MG618535	MG619040	MG619379	MG619718	MG619992	MG620281	MG620565	MG620870	MG621149	MG621758	MG622111
Lycocerussp.	MG615382	MG615937	MG616215	MG617105	MG617981		MG618536		MG619380	MG619719	MG619993	MG620282	MG620566	MG620871	MG621150	MG621759	MG622112
Fissocantharissp.	MG615383	MG615938	MG616216	MG617106	MG617982		MG618537	MG619041	MG619381	MG619720	MG619994		MG620567	MG620872	MG621151	MG621760	MG622113
**Elateridae**																	
Osslimussp.	MG615172	MG615773	MG616045	MG616932	MG617797	MG618095	MG618351	MG618874	MG619179	MG619518	MG619834	MG620099	MG620391	MG620682	MG620989	MG621550	MG621900
Hemicrepidiussp.	MG615258		MG616116	MG617009	MG617876	MG618161	MG618426	MG618934	MG619262	MG619602	MG619901	MG620177	MG620463	MG620769	MG621052	MG621636	MG621987
Pectocerasp.	MG615386	MG615941	MG616219	MG617109	MG617985		MG618540	MG619044	MG619384	MG619723	MG619997	MG620285	MG620570	MG620875	MG621154	MG621763	MG622116
Agrypnussp.	MG615387	MG615942	MG616220	MG617110	MG617986	MG618239	MG618541	MG619045	MG619385	MG619724			MG620571	MG620876	MG621155	MG621764	
Ampedussp.	MG615388	MG615943	MG616221	MG617111	MG617987	MG618240	MG618542	MG619046	MG619386	MG619725	MG619998		MG620572	MG620877	MG621156	MG621765	MG622117
Denticollissp.	MG615389	MG615944	MG616222	MG617112	MG617988	MG618241	MG618543	MG619047	MG619387	MG619726	MG619999	MG620286	MG620573	MG620878	MG621157	MG621766	MG622118
Peniasp.	MG615390	MG615945	MG616223		MG617989	MG618242	MG618544	MG619048	MG619388	MG619727	MG620000	MG620287	MG620574	MG620879	MG621158	MG621767	MG622119
gen.sp.	MG615391	MG615946	MG616224	MG617113	MG617990	MG618243	MG618545	MG619049	MG619389	MG619728	MG620001		MG620575	MG620880	MG621159	MG621768	MG622120
Denticollissp.	MG615392	MG615947	MG616225	MG617114	MG617991	MG618244	MG618546	MG619050	MG619390	MG619729		MG620288	MG620576	MG620881	MG621160	MG621769	MG622121
Cardiotarsussp.	MG615423			MG617144	MG618016	MG618264	MG618572	MG619078	MG619421		MG620023		MG620602	MG620912	MG621184		MG622154
Melanotussp.	MG615424	MG615972	MG616244	MG617145	MG618017	MG618265	MG618573	MG619079	MG619422	MG619761	MG620024	MG620315	MG620603	MG620913	MG621185	MG621800	MG622155
Cebriosp.	MG615429	MG615976	MG616247	MG617150	MG618021	MG618269	MG618578	MG619083	MG619427	MG619766	MG620029	MG620320	MG620607	MG620917	MG621190	MG621803	MG622160
**Eucnemidae**																	
Anischiasp.	MG615111		MG616002	MG616877	MG617736	MG618046	MG618295	MG618832	MG619117	MG619459	MG619792	MG620053	MG620345	MG620633	MG620945	MG621494	MG621837
Hemiopsidasp.	MG615184		MG616053		MG617807		MG618363	MG618883	MG619191	MG619530		MG620109	MG620400	MG620694	MG620995	MG621562	MG621912
Othosp.	MG615273	MG615856	MG616128	MG617025	MG617891	MG618172	MG618440	MG618948	MG619279	MG619619	MG619913	MG620190	MG620473	MG620780	MG621066	MG621653	MG622004
Pyrocoeliasp.	MG615259	MG615844		MG617010	MG617877	MG618162	MG618427	MG618935	MG619263	MG619603		MG620178	MG620464	MG620770	MG621053	MG621637	MG621988
Vestasp.			MG616145		MG617911		MG618459	MG618968	MG619298	MG619638		MG620206	MG620491	MG620795	MG621085	MG621675	MG622026
Pristolycussp.	MG615294	MG615871	MG616146	MG617041	MG617912	MG618182	MG618460	MG618969	MG619299	MG619639	MG619929	MG620207	MG620492	MG620796	MG621086	MG621676	MG622027
Luciolasp.	MG615295	MG615872	MG616147	MG617042	MG617913	MG618183	MG618461	MG618970	MG619300	MG619640		MG620208	MG620493	MG620797	MG621087	MG621677	MG622028
Cyphonocerussp.	MG615296	MG615873	MG616148	MG617043	MG617914	MG618184	MG618462	MG618971	MG619301	MG619641		MG620209	MG620494	MG620798	MG621088	MG621678	MG622029
Drilastersp.	MG615384	MG615939	MG616217	MG617107	MG617983	MG618237	MG618538	MG619042	MG619382	MG619721	MG619995	MG620283	MG620568	MG620873	MG621152	MG621761	MG622114
Diaphanessp.	MG615385	MG615940	MG616218	MG617108	MG617984	MG618238	MG618539	MG619043	MG619383	MG619722	MG619996	MG620284	MG620569	MG620874	MG621153	MG621762	MG622115

Gorhamiasp.	MG615428	MG615975	MG616246	MG617149	MG618020	MG618268	MG618577	MG619082	MG619426	MG619765	MG620028	MG620319	MG620606	MG620916	MG621189	MG621802	MG622159
**Lycidae**																	
Porrostomasp.	MG615193	MG615789		MG616950	MG617816	MG618106	MG618372	MG618888	MG619199	MG619538			MG620408	MG620703	MG621003	MG621570	MG621921
Benibotarussp.	MG615430	MG615977		MG617151	MG618022		MG618579	MG619084	MG619428	MG619767			MG620608	MG620918	MG621191	MG621804	MG622161
Dilophotessp.	MG615431		MG616248	MG617152	MG618023		MG618580	MG619085	MG619429	MG619768	MG620030		MG620609		MG621192	MG621805	MG622162
Platerodrilussp.	MG615432	MG615978	MG616249	MG617153	MG618024		MG618581	MG619086	MG619430	MG619769			MG620610	MG620919	MG621193	MG621806	MG622163
Libnetissp.	MG615433	MG615979		MG617154	MG618025	MG618270	MG618582	MG619087	MG619431	MG619770		MG620321	MG620611	MG620920	MG621194	MG621807	MG622164
Macrolycussichuanensis	MG615434	MG615980	MG616250	MG617155	MG618026		MG618583	MG619088	MG619432	MG619771	MG620031		MG620612	MG620921	MG621195	MG621808	MG622165
Lycostomussp.	MG615435	MG615981		MG617156	MG618027		MG618584	MG619089	MG619433	MG619772			MG620613	MG620922	MG621196	MG621809	
**Omethidae**																	
Driloniussp.	MG615354	MG615917	MG616195	MG617090	MG617964		MG618514	MG619019	MG619354	MG619694		MG620257	MG620546	MG620848	MG621131	MG621733	MG622087
Driloniussp.	MG615356	MG615919		MG617092	MG617966		MG618516	MG619021	MG619356	MG619696		MG620259	MG620548	MG620850	MG621132	MG621735	MG622089
**Phengodidae**																	
Stenophrixothrixsp.	MG615129	MG615739	MG616015	MG616890	MG617754	MG618060	MG618313	MG618846	MG619135	MG619477	MG619804	MG620066	MG620357	MG620646	MG620959	MG621510	MG621854
**Rhagophthalmidae**																	
Rhagophthalmussp.	MG615133		MG616019	MG616896	MG617760	MG618066	MG618318	MG618851	MG619140	MG619482	MG619808	MG620069	MG620362	MG620651	MG620963	MG621515	MG621860
Rhagophthalmussp.	MG615212	MG615803	MG616077	MG616968	MG617833	MG618121	MG618387	MG618904	MG619216	MG619556	MG619862	MG620130	MG620421	MG620722	MG621014	MG621589	MG621940
gen.sp.	MG615355	MG615918	MG616196	MG617091	MG617965	MG618221	MG618515	MG619020	MG619355	MG619695		MG620258	MG620547	MG620849		MG621734	MG622088
**Throscidae**																	
Trixagussp.	MG615141	MG615748	MG616023	MG616903			MG618324	MG618856	MG619148	MG619489	MG619814	MG620074				MG621522	
Trixagussp.	MG615353	MG615916	MG616194	MG617089	MG617963		MG618513		MG619353	MG619693	MG619973	MG620256		MG620847	MG621130	MG621732	MG622086
**Histeridae**																	
Platysomasp.	MG615112	MG615726	MG616003	MG616878	MG617737	MG618047	MG618296	MG618833	MG619118	MG619460		MG620054	MG620346	MG620634		MG621495	MG621838
Saprinussp.	MG615113	MG615727	MG616004	MG616879	MG617738	MG618048	MG618297	MG618834	MG619119	MG619461	MG619793	MG620055		MG620635	MG620946	MG621496	MG621839
**Hydrophiloidea/ Hydrophilidae**																	
Sternolophusimmarginatus	MG615187	MG615784		MG616945	MG617810		MG618366	MG618884	MG619194	MG619533		MG620112	MG620403	MG620697	MG620998	MG621565	MG621915
Helocharessp.	MG615188	MG615785	MG616056	MG616946	MG617811		MG618367	MG618885	MG619195	MG619534	MG619843	MG620113	MG620404	MG620698	MG620999	MG621566	MG621916
Oocyclussp.	MG615256	MG615842		MG617007	MG617874	MG618159		MG618932	MG619260	MG619600		MG620175	MG620461	MG620767	MG621050	MG621634	MG621985
Hydrophilusacuminatus	MG615335	MG615900	MG616179	MG617073	MG617949		MG618498		MG619334	MG619675	MG619958	MG620240	MG620528	MG620832		MG621713	MG622067
Anacaenapui	MG615337	MG615902	MG616181		MG617951	MG618206	MG618500	MG619004	MG619336	MG619676	MG619960	MG620241	MG620530	MG620834	MG621114	MG621715	MG622069
Georissussp.	MG615338	MG615903	MG616182	MG617075	MG617952	MG618207	MG618501	MG619005	MG619337	MG619677	MG619961	MG620242	MG620531	MG620835	MG621115	MG621716	MG622070
Sphaeridiumquinquemaculatum	MG615339	MG615904	MG616183	MG617076	MG617953	MG618208	MG618502	MG619006	MG619338	MG619678	MG619962	MG620243	MG620532		MG621116	MG621717	MG622071
Berosusspinosus	MG615379								MG619377		MG619990		MG620563				
**Chrysomeloidea/ Cerambycidae**																	
Strangaliafortunei	MG615300	MG615876	MG616152	MG617046	MG617918		MG618465	MG618973	MG619305	MG619645		MG620211	MG620498			MG621682	MG622033
Obereasp.	MG615301	MG615877	MG616153	MG617047	MG617919	MG618186	MG618466	MG618974	MG619306	MG619646	MG619933	MG620212	MG620499	MG620802	MG621092	MG621683	MG622034
Spondylisbuprestoides	MG615302	MG615878	MG616154	MG617048	MG617920		MG618467	MG618975	MG619307	MG619647		MG620213	MG620500	MG620803	MG621093	MG621684	MG622035
Xylotrechussp.	MG615303		MG616155	MG617049	MG617921		MG618468	MG618976	MG619308	MG619648	MG619934	MG620214				MG621685	MG622036
Dorystheneshydropicus	MG615377		MG616212	MG617103				MG619037	MG619375								MG622108
**Chrysomelidae**																	
Cassidasp.	MG615250	MG615836					MG618419	MG618926	MG619254	MG619593		MG620169		MG620760	MG621046	MG621627	MG621978
Chrysomelapopuli		MG615845	MG616118				MG618429	MG618937	MG619265	MG619605		MG620180			MG621055	MG621639	MG621990
Chlamisussp.	MG615309	MG615879	MG616160		MG617926	MG618190	MG618474	MG618981	MG619312		MG619939	MG620219		MG620809	MG621096	MG621690	MG622042
Sagrafulgidajanthina	MG615361	MG615923	MG616200		MG617969		MG618520		MG619361	MG619701	MG619977	MG620264	MG620552	MG620855	MG621136	MG621740	MG622094
Liliocerissp.	MG615362	MG615924	MG616201			MG618225	MG618521		MG619362	MG619702		MG620265	MG620553	MG620856	MG621137	MG621741	MG622095
Dactylispasp.	MG615363	MG615925					MG618522	MG619026	MG619363	MG619703	MG619978	MG620266		MG620857	MG621138	MG621742	MG622096
Anisoderasp.	MG615364	MG615926					MG618523	MG619027	MG619364	MG619704	MG619979	MG620267		MG620858	MG621139	MG621743	MG622097
Sominellasp.	MG615365	MG615927	MG616202	MG617097	MG617970	MG618226	MG618524			MG619705	MG619980	MG620268		MG620859		MG621744	MG622098
Trichochryseasp.	MG615366					MG618227	MG618525	MG619028	MG619365	MG619706	MG619981	MG620269			MG621140	MG621745	MG622099

gen.sp.	MG615367	MG615928	MG616203		MG617971	MG618228	MG618526	MG619029	MG619366	MG619707	MG619982	MG620270	MG620554	MG620860	MG621141	MG621746	
Alticasp.	MG615368		MG616204				MG618527	MG619030	MG619367	MG619708	MG619983	MG620271	MG620555	MG620861	MG621142	MG621747	MG622100
Oomorphoidessp.	MG615369	MG615929	MG616205			MG618229	MG618528	MG619031	MG619368	MG619709				MG620862		MG621748	MG622101
Cryptocephalussp.	MG615370	MG615930	MG616206		MG617972	MG618230			MG619369	MG619710	MG619984	MG620272	MG620556	MG620863		MG621749	MG622102
Bruchidiussp.	MG615371		MG616207			MG618231					MG619985	MG620273			MG621143	MG621750	
**Megalopodidae**																	
Temnaspissp.	MG615270	MG615853	MG616124	MG617021	MG617887	MG618169	MG618437	MG618945	MG619275	MG619615	MG619909	MG620189			MG621063	MG621649	MG622000
**Lymexyloidea/Lymexylidae**																	
Melittommasp.	MG615121	MG615732			MG617746			MG618839	MG619127	MG619469			MG620351	MG620641	MG620952	MG621502	MG621847
Atractocerussp.	MG615326	MG615892			MG617942								MG620519				MG622058
**Rhinorhipoidea/Rhinorhipidae**																	
Rhinorhipus tamborensis	AB123471	AB123472	AB123473	AB123474	AB123475	AB123476	AB123477	AB123478	AB123479	AB123480	AB123481	AB123482	AB123483	AB123484	AB123485	AB123486	AB123487
**Scarabaeoidea/Geotrupidae**																	
Australobolbussp.	MG615217	MG615808	MG616082	MG616973	MG617838	MG618125	MG618392	MG618909	MG619220	MG619561	MG619867	MG620135	MG620426	MG620726	MG621018	MG621594	MG621944
Geotrupessp.	MG615329	MG615895	MG616175	MG617068	MG617945		MG618492	MG618997	MG619329	MG619669	MG619953	MG620235	MG620522	MG620826	MG621107	MG621707	MG622061
**Glaphyridae**																	
Amphicomasp.	MG615253	MG615840	MG616113	MG617005	MG617871	MG618157	MG618423	MG618930	MG619257	MG619597		MG620172	MG620458	MG620764		MG621631	MG621982
**Hybosoridae**																	
Liparochrussp.	MG615114		MG616005		MG617739		MG618298	MG618835	MG619120	MG619462		MG620056		MG620636			MG621840
Cyphopisthessp.	MG615218	MG615809	MG616083	MG616974					MG619221					MG620727			MG621945
**Lucanidae**																	
Phalacrognathusmuelleri	MG615330	MG615896			MG617946	MG618202	MG618493	MG618998	MG619330	MG619670	MG619954	MG620236	MG620523	MG620827	MG621108	MG621708	MG622062
Cyclommatusmetallifer	MG615331	MG615897		MG617069	MG617947		MG618494	MG618999	MG619331	MG619671	MG619955	MG620237	MG620524	MG620828	MG621109	MG621709	MG622063
Aegussp.	MG615375	MG615933	MG616210	MG617101	MG617976	MG618234	MG618531	MG619035	MG619373	MG619714	MG619988	MG620277	MG620560	MG620867	MG621146	MG621754	MG622106
**Passalidae**																	
Ceracupessp.	MG615293	MG615870		MG617040	MG617910	MG618181	MG618458	MG618967	MG619297	MG619637	MG619928		MG620490		MG621084	MG621674	MG622025
Aceraiussp.	MG615328	MG615894	MG616174	MG617067	MG617944	MG618201	MG618491	MG618996	MG619328	MG619668		MG620234	MG620521	MG620825		MG621706	MG622060
**Scarabaeidae**																	
Carneodonsp.	MG615219	MG615810	MG616084	MG616975	MG617839	MG618126	MG618393	MG618910	MG619222	MG619562	MG619868	MG620136	MG620427	MG620728	MG621019	MG621595	MG621946
Heteronyxsp.	MG615220	MG615811	MG616085	MG616976	MG617840	MG618127	MG618394	MG618911	MG619223	MG619563		MG620137	MG620428	MG620729	MG621020	MG621596	MG621947
Anomalaantiqua	MG615221	MG615812	MG616086	MG616977	MG617841	MG618128	MG618395	MG618912	MG619224	MG619564	MG619869	MG620138	MG620429	MG620730	MG621021	MG621597	MG621948
Onthophagusgazella	MG615222	MG615813	MG616087	MG616978	MG617842	MG618129	MG618396	MG618913	MG619225	MG619565	MG619870	MG620139	MG620430	MG620731	MG621022	MG621598	MG621949
Rhyparussp.			MG616109		MG617866	MG618151	MG618415		MG619250	MG619590	MG619891	MG620165	MG620452	MG620757	MG621043	MG621623	MG621974
Protaetiaaerata	MG615336	MG615901	MG616180	MG617074	MG617950	MG618205	MG618499	MG619003	MG619335		MG619959		MG620529	MG620833	MG621113	MG621714	MG622068
Mimelasp.	MG615372	MG615931	MG616208	MG617098	MG617973	MG618232	MG618529	MG619032	MG619370	MG619711	MG619986	MG620274	MG620557	MG620864	MG621144	MG621751	MG622103
Coprissp.	MG615373	MG615932	MG616209	MG617099	MG617974		MG618530	MG619033	MG619371	MG619712	MG619987	MG620275	MG620558	MG620865	MG621145	MG621752	MG622104
Ectinohopliasp.	MG615374			MG617100	MG617975	MG618233		MG619034	MG619372	MG619713		MG620276	MG620559	MG620866		MG621753	MG622105
Dasyvalgussp.	MG615376	MG615934	MG616211	MG617102	MG617977	MG618235	MG618532	MG619036	MG619374	MG619715	MG619989	MG620278	MG620561	MG620868		MG621755	MG622107
Cheirotonusjansoni	MG615378	MG615935			MG617978		MG618533	MG619038	MG619376	MG619716		MG620279	MG620562		MG621147	MG621756	MG622109
Allomyrinadichotoma	MG615396	MG615951	MG616228	MG617118	MG617994		MG618550	MG619053	MG619394	MG619733	MG620002		MG620580	MG620885	MG621163	MG621773	MG622125
**Trogidae**																	
Omorgussp.	MG615142	MG615749		MG616904	MG617767	MG618071	MG618325	MG618857	MG619149	MG619490	MG619815	MG620075	MG620368	MG620654	MG620969	MG621523	MG621868
Troxsp.	MG615274	MG615857		MG617026	MG617892	MG618173	MG618441	MG618949	MG619280	MG619620	MG619914	MG620191	MG620474	MG620781	MG621067	MG621654	MG622005
**Scirtoidea/Clambidae**																	
Clambussp.	MG615163	MG615766	MG616039	MG616924	MG617788	MG618088	MG618344		MG619170	MG619510	MG619829	MG620093	MG620385	MG620673	MG620981		MG621891
**Eucinetidae**																	
Noteucinetussp.	MG615110	MG615725	MG616001	MG616876	MG617735		MG618294		MG619116	MG619458	MG619791	MG620052	MG620344		MG620944		MG621836
Noteucinetussp.	MG615183		MG616052	MG616942	MG617806	MG618101	MG618362	MG618882	MG619190	MG619529	MG619840		MG620399	MG620693		MG621561	MG621911
**Scirtidae**																	

Pseudomicrocarasp.	MG615223	MG615814	MG616088	MG616979	MG617843	MG618130		MG618914	MG619226	MG619566	MG619871	MG620140	MG620431	MG620732	MG621023	MG621599	MG621950
gen.sp.	MG615278	MG615860	MG616130	MG617031	MG617897			MG618954	MG619285	MG619625	MG619917	MG620194	MG620479	MG620782	MG621071	MG621658	MG622010
Elodessp.	MG615360	MG615922	MG616199	MG617096	MG617968	MG618224	MG618519	MG619025	MG619360	MG619700	MG619976	MG620263	MG620551	MG620854	MG621135	MG621739	MG622093
**Staphylinoidea/Agyrtidae**																	
Pterolomaforsstromii	MG615414	MG615966		MG617136	MG618008	MG618258	MG618564	MG619070	MG619413	MG619752	MG620016	MG620308	MG620595	MG620903	MG621177	MG621792	MG622146
**Hydraenidae**																	
Hydraenasp.	MG615340	MG615905	MG616184	MG617077	MG617954	MG618209	MG618503	MG619007	MG619339	MG619679	MG619963	MG620244		MG620836	MG621117	MG621718	MG622072
**Leiodidae**																	
Agyrtodessp.		MG615730	MG616007	MG616882	MG617743	MG618052	MG618303		MG619124	MG619466	MG619796	MG620059	MG620348	MG620639		MG621500	MG621845
gen.sp.	MG615119			MG616883	MG617744		MG618304		MG619125	MG619467	MG619797		MG620349	MG620640	MG620950		MG621846
Agathidiumsp.	MG615120	MG615731	MG616008		MG617745	MG618053	MG618305		MG619126	MG619468	MG619798	MG620060	MG620350		MG620951	MG621501	
**Ptiliidae**																	
gen.sp.			MG616017	MG616893	MG617757								MG620359				
**Silphidae**																	
Nicrophorusnepalensis	MG615262	MG615847	MG616120	MG617013	MG617880	MG618164	MG618431		MG619267	MG619607	MG619903	MG620182	MG620467		MG621057	MG621641	MG621992
Necrodeslittoralis	MG615310	MG615880	MG616161	MG617053	MG617927	MG618191	MG618475	MG618982	MG619313	MG619653		MG620220	MG620505	MG620810	MG621097	MG621691	MG622043
**Staphylinidae**																	
Scaphidiumsp.	MG615139	MG615747	MG616022		MG617766		MG618323		MG619146	MG619487		MG620073	MG620367			MG621520	MG621866
gen.sp.	MG615225	MG615817	MG616090	MG616982	MG617846				MG619228		MG619873	MG620142	MG620432	MG620734	MG621024		MG621952
Staphylinussp.	MG615252	MG615838	MG616112	MG617004	MG617870	MG618155	MG618421	MG618928	MG619255	MG619595	MG619896	MG620170	MG620456	MG620762	MG621047	MG621629	MG621980
Megalopaederussp.	MG615269		MG616123	MG617020	MG617886			MG618944	MG619274	MG619614	MG619908	MG620188	MG620472	MG620777	MG621062	MG621648	MG621999
Apateticasp.	MG615317	MG615886	MG616167	MG617059	MG617934	MG618196	MG618482		MG619319	MG619659		MG620227	MG620512	MG620817	MG621101	MG621698	MG622050
Priochirussp.	MG615318		MG616168		MG617935	MG618197	MG618483	MG618989	MG619320	MG619660	MG619945	MG620228	MG620513	MG620818			MG622051
Osoriussp.	MG615319	MG615887		MG617060	MG617936		MG618484	MG618990	MG619321	MG619661	MG619946		MG620514	MG620819	MG621102	MG621699	MG622052
Tachinussp.	MG615320		MG616169	MG617061	MG617937		MG618485	MG618991	MG619322	MG619662	MG619947		MG620515	MG620820		MG621700	MG622053
Scaphidiumsp.	MG615321	MG615888		MG617062	MG617938		MG618486		MG619323	MG619663	MG619948	MG620229			MG621103	MG621701	MG622054
Tachinussp.	MG615322	MG615889	MG616170	MG617063	MG617939		MG618487	MG618992	MG619324	MG619664	MG619949		MG620516	MG620821		MG621702	MG622055
Centrophthalmussp.	MG615323						MG618488					MG620230		MG620822			
gen.sp.	MG615399	MG615954		MG617121	MG617996			MG619056	MG619397				MG620583				MG622128
Dianoussp.	MG615400	MG615955		MG617122	MG617997	MG618246	MG618552	MG619057	MG619398	MG619736	MG620005	MG620294	MG620584	MG620887		MG621776	MG622129
gen.sp.	MG615401	MG615956		MG617123	MG617998		MG618553	MG619058	MG619399	MG619737	MG620006		MG620585	MG620888		MG621777	MG622130
**Tenebrionoidea/Aderidae**																	
gen.sp.	MG615091			MG616858	MG617717		MG618278	MG618820	MG619098	MG619441	MG619781		MG620329		MG620929	MG621476	MG621817
gen.sp.	MG615419			MG617140	MG618012		MG618568	MG619074	MG619417	MG619757	MG620019		MG620598	MG620908	MG621180		MG622150
**Anthicidae**																	
Lemodessp.	MG615093	MG615711	MG615990	MG616860	MG617719	MG618037	MG618280	MG618821	MG619100	MG619443	MG619783	MG620038	MG620331		MG620931	MG621478	MG621819
Anthicuss.l.sp.	MG615148	MG615752		MG616908	MG617771		MG618327	MG618858	MG619154	MG619494				MG620657	MG620972	MG621527	
Trichanancasp.	MG615149	MG615753	MG616027	MG616909	MG617772	MG618076	MG618328	MG618859	MG619155	MG619495	MG619818	MG620080	MG620372	MG620658	MG620973	MG621528	MG621873
Macratriasp.	MG615150	MG615754	MG616028	MG616910	MG617773		MG618329	MG618860	MG619156	MG619496		MG620081	MG620373	MG620659	MG620974	MG621529	MG621874
Macratriasp.	MG615286	MG615865	MG616139	MG617036	MG617904		MG618452	MG618961	MG619292	MG619630	MG619925	MG620201	MG620485	MG620789	MG621079	MG621667	MG622019
**Archeocrypticidae**																	
Wattianusqueenslandicus	MG615151	MG615755		MG616911	MG617774	MG618077	MG618330	MG618861	MG619157	MG619497	MG619819	MG620082	MG620374	MG620660	MG620975	MG621530	MG621875
**Boridae**																	
Synercticussp.	MG615153	MG615758	MG616029	MG616913	MG617776	MG618080	MG618333	MG618863	MG619159	MG619500			MG620376	MG620663			MG621878
**Ciidae**																	
Cissp.	MG615103	MG615718	MG615995	MG616869	MG617728	MG618041	MG618287	MG618827	MG619110	MG619452	MG619786	MG620046	MG620340		MG620938	MG621487	MG621829
Cissp.	MG615161			MG616922	MG617786	MG618086	MG618342	MG618867	MG619168	MG619508	MG619827	MG620091	MG620383			MG621540	MG621889
Australocissp.	MG615162	MG615765	MG616038	MG616923	MG617787	MG618087	MG618343	MG618868	MG619169	MG619509	MG619828	MG620092	MG620384	MG620672	MG620980	MG621541	MG621890
**Tenebrionoidea/IncertaeSedis**																	

Rhizoniumantiquum	MG615185	MG615782	MG616054	MG616943	MG617808	MG618102	MG618364		MG619192	MG619531	MG619841	MG620110	MG620401	MG620695	MG620996	MG621563	MG621913
**Ischaliidae**																	
Ischaliasp.	MG615285		MG616138	MG617035	MG617903		MG618451		MG619291		MG619924		MG620484	MG620788	MG621078	MG621666	MG622018
**Melandryidae**																	
Dircaeomorphasp.	MG615291	MG615868	MG616143	MG617039	MG617908	MG618179	MG618456	MG618965	MG619295	MG619635	MG619927	MG620204	MG620489	MG620793	MG621082	MG621672	MG622023
gen.sp.	MG615416	MG615968		MG617138	MG618010	MG618259	MG618566	MG619072	MG619415	MG619754	MG620018	MG620310	MG620597	MG620905	MG621178	MG621794	MG622148
**Meloidae**																	
Zonitiss.l.sp.	MG615194	MG615790	MG616060	MG616951	MG617817	MG618107		MG618889	MG619200	MG619539	MG619848	MG620116	MG620409	MG620704	MG621004	MG621571	MG621922
Epicautasp.	MG615325	MG615891	MG616172	MG617065	MG617941	MG618199		MG618994	MG619326	MG619666	MG619951	MG620232	MG620518	MG620824	MG621105	MG621704	MG622057
**Mordellidae**																	
Hoshihananomiasp.		MG615855	MG616127	MG617024	MG617890	MG618171		MG618947	MG619278	MG619618	MG619912			MG620779	MG621065	MG621652	MG622003
**Mycetophagidae**																	
Nototriphyllussp.	MG615123	MG615734	MG616010	MG616885	MG617748	MG618055	MG618307	MG618841	MG619129	MG619471	MG619800	MG620062	MG620353		MG620954	MG621504	MG621848
Mycetophagussp.	MG615284	MG615863	MG616136	MG617034	MG617901	MG618176	MG618449	MG618959	MG619289	MG619628	MG619922	MG620199	MG620483		MG621076	MG621664	MG622016
**Mycteridae**																	
Trichosalpingussp.	MG615124	MG615735	MG616011		MG617749	MG618056	MG618308		MG619130	MG619472				MG620642	MG620955	MG621505	MG621849
**Oedemeridae**																	
Thelyphassasp.	MG615203		MG616068	MG616960	MG617824		MG618378	MG618896	MG619207	MG619548				MG620713		MG621580	MG621931
Pseudolycussp.	MG615204	MG615796	MG616069		MG617825		MG618379	MG618897	MG619208	MG619549	MG619856			MG620714	MG621010	MG621581	MG621932
Ditylussp.	MG615272	MG615854	MG616126	MG617023	MG617889	MG618170	MG618439	MG618946	MG619277	MG619617	MG619911				MG621064	MG621651	MG622002
Pyrochroidae																	
Morpholycussp.	MG615132	MG615742	MG616018	MG616895	MG617759	MG618065	MG618317	MG618850	MG619139	MG619481	MG619807	MG620068	MG620361	MG620650		MG621514	MG621859
MorpholycusmonilicornisLea	MG615210	MG615801	MG616075	MG616966	MG617831	MG618119	MG618385	MG618903	MG619214	MG619554	MG619861	MG620129	MG620420	MG620720	MG621013	MG621587	MG621938
Pseudopyrochroasp.	MG615261	MG615846	MG616119	MG617012	MG617879	MG618163	MG618430	MG618938	MG619266	MG619606	MG619902	MG620181	MG620466	MG620772	MG621056	MG621640	MG621991
Eupyrochroainsignita	MG615266	MG615850	MG616122	MG617017	MG617883	MG618166	MG618434	MG618941	MG619271	MG619611	MG619906	MG620185	MG620470	MG620775	MG621059	MG621645	MG621996
**Pythidae**																	
Anaplopussp.	MG615211	MG615802	MG616076	MG616967	MG617832	MG618120	MG618386		MG619215	MG619555				MG620721		MG621588	MG621939
**Ripiphoridae**																	
Trigonoderasp.	MG615135	MG615744	MG616020	MG616898	MG617762	MG618068	MG618320	MG618853	MG619142	MG619484	MG619810	MG620071	MG620364		MG620965	MG621517	MG621862
Rhipidioidessp.	MG615213	MG615804	MG616078	MG616969	MG617834		MG618388	MG618905		MG619557	MG619863	MG620131	MG620422			MG621590	
**Salpingidae**																	
OrphanotrophiumpallidipennisLea	MG615136	MG615745	MG616021	MG616899	MG617763	MG618069	MG618321	MG618854	MG619143	MG619485	MG619811		MG620365		MG620966	MG621518	MG621863
Euryplatusdimidiatus	MG615214	MG615805	MG616079	MG616970	MG617835	MG618122	MG618389	MG618906	MG619217	MG619558	MG619864	MG620132	MG620423	MG620723	MG621015	MG621591	MG621941
Orphanotrophiumsp.	MG615215	MG615806	MG616080	MG616971	MG617836	MG618123	MG618390	MG618907	MG619218	MG619559	MG619865	MG620133	MG620424	MG620724	MG621016	MG621592	MG621942
Ocholissasp.	MG615216	MG615807	MG616081	MG616972	MG617837	MG618124	MG618391	MG618908	MG619219	MG619560	MG619866	MG620134	MG620425	MG620725	MG621017	MG621593	MG621943
**Scraptiidae**																	
Scraptiasp.	MG615418			MG617139	MG618011		MG618567			MG619756				MG620907	MG621179	MG621796	
**Tenebrionidae**																	
Amarygmussp.	MG615226		MG616091	MG616983	MG617847	MG618133	MG618398	MG618916	MG619229	MG619568	MG619874	MG620143	MG620433	MG620735	MG621025	MG621602	MG621953
Cyphaleussp.	MG615227	MG615818	MG616092	MG616984	MG617848	MG618134	MG618399		MG619230	MG619569		MG620144	MG620434	MG620736			MG621954
Adeliumsp.	MG615228	MG615819	MG616093	MG616985	MG617849	MG618135	MG618400	MG618917	MG619231	MG619570	MG619875	MG620145	MG620435	MG620737	MG621026	MG621603	MG621955
Cillibussp.	MG615229	MG615820	MG616094	MG616986	MG617850	MG618136	MG618401		MG619232	MG619571		MG620146	MG620436	MG620738	MG621027	MG621604	MG621956
Palorussp.	MG615230		MG616095	MG616987	MG617851	MG618137	MG618402	MG618918	MG619233	MG619572	MG619876	MG620147		MG620739	MG621028	MG621605	MG621957
Derispiasp.	MG615231	MG615821	MG616096	MG616988	MG617852		MG618403	MG618919	MG619234	MG619573	MG619877	MG620148	MG620437	MG620740	MG621029	MG621606	MG621958
Cossyphussp.	MG615232	MG615822	MG616097	MG616989	MG617853	MG618138	MG618404		MG619235	MG619574		MG620149	MG620438	MG620741	MG621030	MG621607	MG621959
Tanychilussp.	MG615233	MG615823	MG616098	MG616990	MG617854	MG618139	MG618405	MG618920	MG619236	MG619575	MG619878	MG620150	MG620439	MG620742		MG621608	MG621960
Platydemasp.	MG615234		MG616099	MG616991	MG617855	MG618140	MG618406		MG619237	MG619576	MG619879	MG620151	MG620440	MG620743	MG621031	MG621609	MG621961
Tyrtaeussp.	MG615235	MG615824	MG616100	MG616992	MG617856	MG618141	MG618407	MG618921	MG619238	MG619577	MG619880	MG620152	MG620441	MG620744	MG621032	MG621610	MG621962
Ecnolagriasp.	MG615236	MG615825	MG616101	MG616993	MG617857	MG618142	MG618408		MG619239	MG619578	MG619881	MG620153	MG620442	MG620745	MG621033	MG621611	MG621963

Chlorophilasp.	MG615283		MG616135	MG617033	MG617900		MG618448	MG618958	MG619288	MG619627	MG619921	MG620198	MG620482	MG620786	MG621075	MG621663	MG622015
Derispiasp.	MG615304		MG616156	MG617050	MG617922		MG618469	MG618977		MG619649		MG620215	MG620501	MG620804		MG621686	MG622037
Cteniopinussp.	MG615305		MG616157	MG617051	MG617923	MG618187	MG618470	MG618978	MG619309	MG619650	MG619935	MG620216	MG620502	MG620805	MG621094	MG621687	MG622038
Strongyliumsp.	MG615402	MG615957	MG616230	MG617124	MG617999	MG618247	MG618554		MG619400	MG619738	MG620007	MG620295	MG620586	MG620889	MG621165	MG621778	MG622131
Cryphaeussp.		MG615958	MG616231	MG617125	MG618000	MG618248	MG618555		MG619401	MG619739	MG620008	MG620296	MG620587	MG620890	MG621166	MG621779	MG622132
Trictenotomasp.	MG615421		MG616242	MG617142	MG618014	MG618262	MG618570	MG619076	MG619419	MG619759	MG620021	MG620313	MG620600	MG620910	MG621182	MG621798	MG622152
**Ulodidae**																	
Ulodessp.	MG615239	MG615826	MG616102		MG617859	MG618144	MG618409		MG619242	MG619582	MG619883	MG620157	MG620444	MG620749	MG621035	MG621615	
Meryxsp.	MG615240	MG615827	MG616103	MG616997	MG617860	MG618145	MG618410	MG618922	MG619243	MG619583	MG619884	MG620158	MG620445	MG620750	MG621036	MG621616	MG621967
**Zopheridae**																	
Monommasp.	MG615145					MG618073											
Bitomasp.	MG615146	MG615750	MG616025	MG616906	MG617769	MG618074	MG618326		MG619152	MG619492	MG619816	MG620078	MG620370		MG620971	MG621525	MG621871
Zopherosisgeorgei	MG615241	MG615828	MG616104		MG617861		MG618411	MG618923	MG619244	MG619584	MG619885	MG620159	MG620446	MG620751	MG621037	MG621617	MG621968

	Hmgs	Hr38	Hsc70-5	ico	Idh	Int6	IntS11	Lar	Ndae1	nito	nonC	Notch	Nrx-IV	Past1	RnrL	RpII140
**Outgroup**																
Neochauliodesorientalis	MG621972	MG622288		MG623139	MG623436			MG625364	MG626317		MG627779	MG627999	MG628217		MG629633	
Ascalohybrissubjacens	MG621971	MG622287	MG622603	MG623138	MG623435	MG623737	MG614278	MG625363	MG626316		MG627778	MG627998	MG628216		MG629632	MG630271
Dichochrysaformosana	MG621970		MG622602	MG623137	MG623434		MG614277	MG625362	MG626315	MG627509		MG627997	MG628215	MG628491	MG629631	
Myrmeleonsp.	MG621969	MG622286	MG622601	MG623136	MG623433		MG614276	MG625361	MG626314			MG627996	MG628214	MG628490	MG629630	MG630270
**Adephaga / Carabidae**																
Dischissussp.	MG621984	MG622299	MG622614	MG623147	MG623446		MG614290	MG625374	MG626323	MG627521		MG628009		MG628502	MG629642	MG630283
Carabussp.	MG622064	MG622369	MG622683	MG623208	MG623510	MG623817	MG614367	MG625439	MG626385	MG627595	MG627842	MG628055	MG628266	MG628579	MG629707	MG630352
Cicindelachinensis	MG622065	MG622370		MG623209	MG623511		MG614368	MG625440	MG626386		MG627843	MG628056	MG628267	MG628580	MG629708	MG630353
Omoglymmiussp.	MG622082	MG622387	MG622698			MG623826	MG614385	MG625452	MG626400		MG627854		MG628275	MG628595		
Clivinasp.	MG622123	MG622423	MG622736	MG623258	MG623552	MG623867	MG614426	MG625486	MG626433				MG628304	MG628639	MG629754	MG630405
Pheropsophussp.	MG622124	MG622424	MG622737	MG623259	MG623553	MG623868	MG614427	MG625487	MG626434	MG627650	MG627878	MG628098	MG628305	MG628640	MG629755	MG630406
Lebiacoelestis	MG622126	MG622426	MG622739	MG623261	MG623555	MG623870	MG614429	MG625488	MG626436	MG627652	MG627880		MG628306	MG628642	MG629756	
Pentagonicaruficollis	MG622127	MG622427	MG622740	MG623262		MG623871	MG614430	MG625489	MG626437	MG627653	MG627881	MG628099	MG628307	MG628643	MG629757	MG630408
Elaphrussp.	MG622156	MG622449		MG623285		MG623899	MG614456	MG625511	MG626461		MG627904		MG628323	MG628670	MG629778	MG630437
gen.sp.	MG622158	MG622451	MG622762		MG623579		MG614457		MG626463	MG627677				MG628672		MG630438
**Dytiscidae**																
Agabussp.	MG622073	MG622378	MG622690	MG623214	MG623517		MG614376	MG625446	MG626392	MG627602	MG627849	MG628061	MG628270	MG628588	MG629713	MG630361
Laccophilusdifficilis	MG622074	MG622379	MG622691	MG623215	MG623518	MG623822	MG614377		MG626393	MG627603	MG627850	MG628062	MG628271	MG628589	MG629714	MG630362
Eretesgriseus	MG622075	MG622380		MG623216			MG614378	MG625447	MG626394	MG627604		MG628063	MG628272	MG628590	MG629715	
Rhantussuturalis	MG622076	MG622381	MG622692	MG623217	MG623519		MG614379	MG625448	MG626395	MG627605	MG627851	MG628064	MG628273	MG628591	MG629716	MG630363
Hyphydrusdetectus	MG622077	MG622382	MG622693	MG623218	MG623520		MG614380	MG625449	MG626396	MG627606		MG628065		MG628592		
**Gyrinidae**																
Orectochilussp.	MG622006			MG623167	MG623466	MG623764	MG614311	MG625394	MG626342	MG627542			MG628233	MG628522	MG629661	MG630302
**Haliplidae**																
Peltodytessp.	MG622083		MG622699	MG623223		MG623827	MG614386	MG625453	MG626401		MG627855		MG628276	MG628596	MG629721	MG630367
**Noteridae**																
gen.sp.	MG621852	MG622196	MG622493	MG623046	MG623333	MG623626	MG614160	MG625258	MG626211	MG627402			MG628146	MG628370	MG629536	MG630158
Canthydrussp.	MG622078	MG622383	MG622694	MG623219		MG623823	MG614381							MG628593	MG629717	MG630364
**Archostemata/ Cupedidae**																
Tenomergasp.	MG622080	MG622385	MG622696	MG623221	MG623522	MG623824	MG614383		MG626398	MG627608				MG628594	MG629719	
**Myxophaga / Torridincolidae**																
Satoniusschoenmanni	MG622079	MG622384	MG622695	MG623220	MG623521		MG614382	MG625450	MG626397	MG627607	MG627852	MG628066			MG629718	MG630365
**Bostrichoidea /Bostrichidae**																
Lyctussp.	MG621879	MG622219	MG622517	MG623067	MG623355		MG614187	MG625282	MG626235	MG627427	MG627721	MG627931		MG628399	MG629557	MG630186
Polycaonsp.	MG622001	MG622315	MG622630	MG623162		MG623760	MG614306	MG625389		MG627537		MG628019		MG628519	MG629657	MG630298
**Dermestidae**																
Dermestessp.	MG621897	MG622230	MG622532	MG623079	MG623367	MG623666	MG614205	MG625297		MG627444	MG627727	MG627942	MG628172	MG628417	MG629570	MG630200
Evorineasp.	MG621898		MG622533	MG623080	MG623368	MG623667	MG614206	MG625298	MG626252	MG627445		MG627943		MG628418		MG630201
Orphinussp.	MG622090		MG622706	MG623230	MG623530	MG623833	MG614392	MG625459	MG626407	MG627615	MG627860		MG628282	MG628603	MG629726	MG630373
**Ptinidae**																
Ptinussp.	MG621857			MG623050		MG623631	MG614166		MG626217	MG627406				MG628376		MG630163
Dorcatomasp.	MG621858	MG622202	MG622497		MG623337	MG623632		MG625262		MG627407				MG628377	MG629540	MG630164
Ptinussp.	MG622092			MG623232		MG623835	MG614394	MG625461		MG627617		MG628073		MG628605		
Hedobiasp.	MG622149	MG622445		MG623279		MG623891	MG614448		MG626454	MG627672	MG627899	MG628115		MG628664		MG630429
**Buprestoidea / Buprestidae**																
Coroebussp.	MG621989	MG622304		MG623152	MG623451	MG623749	MG614295		MG626327	MG627526	MG627789	MG628013	MG628223	MG628507	MG629647	

Dicercasp.	MG622048	MG622355	MG622669	MG623194		MG623802	MG614351	MG625428	MG626371			MG628044		MG628563	MG629695	MG630338
Trachyssp.	MG622049	MG622356	MG622670	MG623195	MG623496	MG623803	MG614352		MG626372		MG627828	MG628045	MG628258	MG628564	MG629696	MG630339
**Byrrhoidea / Byrrhidae**																
Notolioonsp.	MG621824	MG622174	MG622468			MG623597		MG625236	MG626184	MG627375				MG628346		MG630130
Microchaetessp.	MG621885		MG622521	MG623071		MG623654	MG614193	MG625286	MG626241	MG627432		MG627935	MG628164	MG628405	MG629561	MG630189
Cytilussp.	MG622021	MG622332	MG622646		MG623476	MG623779	MG614325	MG625407	MG626353	MG627556		MG628027		MG628537	MG629674	
**Callirhipidae**																
Ennometessp.	MG621825	MG622175	MG622469	MG623024	MG623307	MG623598	MG614133	MG625237	MG626185	MG627376	MG627690		MG628131		MG629511	MG630131
Simianussp.	MG622081	MG622386	MG622697	MG623222	MG623523	MG623825	MG614384	MG625451	MG626399	MG627609	MG627853		MG628274		MG629720	MG630366
**Dryopidae**																
Helichussp.	MG621834	MG622181	MG622476		MG623314	MG623607	MG614141	MG625243		MG627385				MG628353		MG630138
Pachyparnussp.	MG622122		MG622735	MG623257	MG623551	MG623866	MG614425	MG625485		MG627649		MG628097	MG628303	MG628638		MG630404
Helichussp.	MG622141		MG622748	MG623273	MG623566	MG623884		MG625500	MG626447	MG627665	MG627892	MG628109		MG628656		MG630422
**Elmidae**																
Graphelmissp.	MG621901	MG622232	MG622536	MG623083	MG623371	MG623670	MG614209	MG625301	MG626255	MG627446	MG627730		MG628175	MG628421	MG629573	MG630204
Stetholussp.	MG621902	MG622233	MG622537	MG623084	MG623372	MG623671	MG614210	MG625302	MG626256	MG627447	MG627731	MG627946		MG628422	MG629574	MG630205
Stenelmissp.	MG622007	MG622320	MG622635		MG623467		MG614312	MG625395	MG626343	MG627543				MG628523		
**Eulichadidae**																
Eulichassp.	MG621995	MG622310	MG622624	MG623157	MG623456	MG623754	MG614300	MG625384	MG626332	MG627532	MG627793		MG628225	MG628513	MG629651	MG630292
**Heteroceridae**																
Heterocerussp.	MG622056	MG622364	MG622676	MG623201	MG623503	MG623810	MG614360	MG625432	MG626377	MG627588	MG627835		MG628263	MG628571	MG629700	MG630346
**Chelonariidae**																
Chelonariumsp.	MG621828	MG622176	MG622470	MG623025	MG623309	MG623601	MG614135		MG626188	MG627379	MG627691		MG628132	MG628348	MG629514	MG630132
**Limnichidae**																
Byrrhinussp.	MG621920	MG622247	MG622555	MG623094	MG623389	MG623689	MG614227	MG625319	MG626271	MG627462	MG627738	MG627961	MG628182	MG628440	MG629588	MG630221
Pelocharessp.	MG622011	MG622324	MG622639	MG623170	MG623471	MG623768	MG614316	MG625398	MG626346	MG627546	MG627804		MG628235	MG628527	MG629665	MG630306
Cephalobyrrhussp.	MG622047	MG622354	MG622668	MG623193	MG623495	MG623801	MG614350	MG625427	MG626370	MG627581	MG627827	MG628043	MG628257	MG628562	MG629694	MG630337
Limnichussp.	MG622157	MG622450	MG622761		MG623578	MG623900		MG625512	MG626462			MG628119		MG628671	MG629779	
**Psephenidae**																
Sclerocyphonsp.	MG621856	MG622200			MG623336	MG623630	MG614164		MG626215					MG628374		
Schinostethussp.	MG622020	MG622331	MG622645	MG623173	MG623475	MG623778	MG614324	MG625406	MG626352	MG627555	MG627810		MG628240	MG628536	MG629673	MG630315
Schinostethussp.	MG622045	MG622352	MG622666	MG623191	MG623493	MG623799	MG614348	MG625425	MG626368	MG627579	MG627825		MG628255	MG628560	MG629692	MG630335
Mataeopsephussp.	MG622046	MG622353	MG622667	MG623192	MG623494	MG623800	MG614349	MG625426	MG626369	MG627580	MG627826		MG628256	MG628561	MG629693	MG630336
**Ptilodactylidae**																
Ptilodactylasp.	MG621986	MG622301	MG622616	MG623149	MG623448	MG623746	MG614292	MG625376	MG626325	MG627523	MG627786		MG628220	MG628504	MG629644	MG630284
Epilichassp.	MG622044	MG622351	MG622665	MG623190	MG623492	MG623798	MG614347		MG626367	MG627578	MG627824	MG628042	MG628254	MG628559	MG629691	
**Cleroidea /Acanthocnemidae**																
Acanthocnemusnigricans	MG621872	MG622213	MG622510	MG623061	MG623349	MG623643	MG614179	MG625276	MG626228	MG627420	MG627716	MG627924	MG628157	MG628391	MG629550	MG630178
**Biphyllidae**																
Althaesiasp.	MG621821	MG622173	MG622466	MG623022	MG623305	MG623594	MG614130	MG625234	MG626181	MG627374			MG628129	MG628343	MG629509	MG630128
Biphyllussp.	MG621877	MG622218	MG622515	MG623065	MG623353	MG623648	MG614185	MG625281	MG626233	MG627425	MG627719	MG627929	MG628162	MG628397	MG629555	MG630184
**Byturidae**																
Haematoidessp.	MG622008	MG622321	MG622636	MG623168	MG623468	MG623765	MG614313	MG625396	MG626344	MG627544	MG627802	MG628021	MG628234	MG628524	MG629662	MG630303
**Cleridae**																
Tenerussp.	MG621892						MG614200							MG628411		
Necrobiasp.	MG621893	MG622226	MG622527	MG623076	MG623362	MG623661	MG614201	MG625293	MG626247	MG627439		MG627937		MG628412	MG629566	MG630195
Xenorthriussp.	MG621983	MG622298	MG622613	MG623146	MG623445	MG623744	MG614289	MG625373		MG627520	MG627784			MG628501		MG630282
Cladiscussp.	MG622136	MG622434	MG622744	MG623269	MG623561	MG623879	MG614438	MG625496	MG626443	MG627660	MG627887	MG628105		MG628651	MG629762	MG630417
Stenocallimerussp.	MG622137	MG622435		MG623270	MG623562	MG623880	MG614439			MG627661	MG627888	MG628106		MG628652	MG629763	MG630418

Allochotessp.	MG622138	MG622436	MG622745	MG623271	MG623563	MG623881	MG614440	MG625497	MG626444	MG627662	MG627889	MG628107		MG628653	MG629764	MG630419
**Melyridae**																
Dasytess.l.sp.	MG621923	MG622250		MG623097	MG623392	MG623692	MG614230	MG625322	MG626274	MG627465	MG627739			MG628443		MG630224
Dicranolaiusbellulus	MG621924	MG622251	MG622558	MG623098	MG623393	MG623693	MG614231	MG625323	MG626275	MG627466	MG627740	MG627964		MG628444	MG629591	MG630225
Carphurussp.	MG621925	MG622252	MG622559	MG623099	MG623394	MG623694	MG614232	MG625324	MG626276	MG627467	MG627741	MG627965	MG628185	MG628445	MG629592	MG630226
gen.sp.	MG621975	MG622291	MG622607	MG623142	MG623439	MG623740	MG614282	MG625367	MG626319	MG627512	MG627781	MG628002		MG628494	MG629636	MG630275
Carphurussp.	MG622091	MG622394	MG622707	MG623231	MG623531	MG623834	MG614393	MG625460	MG626408	MG627616	MG627861	MG628072	MG628283	MG628604	MG629727	MG630374
**Prionoceridae**																
Idgiasp.	MG622009	MG622322	MG622637	MG623169	MG623469	MG623766	MG614314	MG625397	MG626345	MG627545		MG628022		MG628525	MG629663	MG630304
**Thanerocleridae**																
Isoclerussp.	MG621867	MG622209	MG622505	MG623058	MG623345			MG625271		MG627415	MG627713			MG628385		MG630173
**Trogossitidae**																
Larinotusumblicatus	MG621869	MG622211	MG622508	MG623060	MG623347	MG623641	MG614176	MG625274	MG626226	MG627418	MG627714			MG628388	MG629548	MG630176
Rentonellumsp.	MG621870						MG614177							MG628389		
Ancyronasp.	MG621964	MG622280		MG623131	MG623427	MG623731			MG626308	MG627503	MG627773	MG627991		MG628484		MG630265
Parapeltisaustralicum	MG621965	MG622281	MG622597	MG623132	MG623428	MG623732	MG614271	MG625357	MG626309	MG627504	MG627774	MG627992		MG628485		MG630266
Leperinasp.	MG621966	MG622282		MG623133	MG623429	MG623733	MG614272		MG626310	MG627505	MG627775	MG627993		MG628486	MG629627	
Thymalussp.	MG621994	MG622309	MG622623	MG623156	MG623455	MG623753	MG614299	MG625383	MG626331	MG627531	MG627792			MG628512	MG629650	MG630291
**Coccinelloidea /Anamorphidae**																
Papuellasp.	MG621903		MG622538	MG623085	MG623373	MG623672	MG614211	MG625303		MG627448		MG627947		MG628423	MG629575	MG630206
**Bothrideridae**																
Deretaphrussp.	MG621881					MG623651	MG614189		MG626237					MG628401		
Ascetoderessp.	MG621883		MG622520	MG623070	MG623358	MG623653	MG614191	MG625285	MG626239	MG627430		MG627934		MG628403	MG629560	
**Cerylonidae**																
Philothermussp.	MG621827				MG623308	MG623600			MG626187	MG627378					MG629513	
Ostomopsissp.	MG621888		MG622523		MG623359	MG623657	MG614196	MG625289		MG627435			MG628166	MG628407	MG629562	MG630191
**Coccinellidae**																
Chnootribasp.	MG621810	MG622166			MG623294		MG614119	MG625224	MG626174	MG627364		MG627906		MG628332	MG629498	MG630117
Harmoniaoctomaculata	MG621811		MG622458	MG623015	MG623295		MG614120	MG625225	MG626175	MG627365		MG627907		MG628333	MG629499	MG630118
Exochomussp.	MG621812		MG622459		MG623296		MG614121			MG627366				MG628334	MG629500	MG630119
Rhizobiussp.	MG621813				MG623297		MG614122	MG625226		MG627367		MG627908		MG628335	MG629501	MG630120
Ortaliasp.	MG621814	MG622167	MG622460	MG623016	MG623298	MG623588	MG614123	MG625227		MG627368	MG627686	MG627909		MG628336	MG629502	MG630121
Microfreudeasp.				MG623017			MG614124							MG628337		
Sasajiscymnussp.	MG621815	MG622168	MG622461	MG623018	MG623299	MG623589	MG614125	MG625228	MG626176	MG627369				MG628338	MG629503	MG630122
Stethorussp.	MG621816	MG622169	MG622462		MG623300	MG623590	MG614126	MG625229	MG626177	MG627370		MG627910	MG628124		MG629504	MG630123
**Corylophidae**																
Sericoderussp.	MG621830	MG622178	MG622472	MG623027	MG623311	MG623603	MG614137	MG625239	MG626190	MG627381	MG627693	MG627912		MG628350	MG629516	MG630134
Priamimasp.	MG621894	MG622227	MG622528	MG623077	MG623363	MG623662	MG614202	MG625294	MG626248	MG627440	MG627726	MG627938	MG628170	MG628413	MG629567	MG630196
Periptyctussp.	MG621895	MG622228	MG622529		MG623364	MG623663	MG614203	MG625295	MG626249	MG627441		MG627939		MG628414		MG630197
Orthoperussp.	MG622153		MG622758	MG623282	MG623576	MG623896	MG614453	MG625509	MG626458	MG627674			MG628321	MG628668	MG629776	MG630434
**Discolomatidae**																
Aphanocephalussp.	MG621833	MG622180	MG622475	MG623030		MG623606	MG614140	MG625242	MG626193	MG627384			MG628134		MG629519	MG630137
**Endomychidae**																
Cyclotomasp.	MG621835		MG622477		MG623315	MG623608	MG614142	MG625244	MG626194	MG627386				MG628354	MG629520	MG630139
Holoparamecussp.	MG621904	MG622234	MG622539		MG623374	MG623673	MG614212	MG625304	MG626257	MG627449		MG627948		MG628424		MG630207
Encymonimmaculatus	MG621905	MG622235	MG622540	MG623086	MG623375	MG623674	MG614213	MG625305	MG626258	MG627450		MG627949		MG628425	MG629576	MG630208
Stenotarsussp.	MG621906		MG622541		MG623376	MG623675	MG614214	MG625306		MG627451				MG628426	MG629577	MG630209
Sinocymbachussp.	MG621977		MG622609	MG623143	MG623441		MG614284	MG625369	MG626321	MG627514		MG628003		MG628495	MG629638	MG630277
**Euxestidae**																

Hypodacnellasp.	MG621826					MG623599	MG614134		MG626186	MG627377				MG628347	MG629512	
**Latridiidae**																
Enicmussp.	MG621843		MG622484	MG623037	MG623323	MG623616	MG614150	MG625250	MG626201	MG627393		MG627916				MG630147
Corticariasp.	MG621844	MG622187			MG623324		MG614151	MG625251	MG626202					MG628361	MG629526	MG630148
Melanophthalmasp.	MG621919	MG622246	MG622554		MG623388	MG623688	MG614226	MG625318	MG626270	MG627461		MG627960		MG628439	MG629587	MG630220
**Murmidiidae**																
Murmidiusovalis	MG621887	MG622223		MG623073		MG623656	MG614195	MG625288	MG626243	MG627434	MG627723					
**Teredidae**																
Xylariophilussp.	MG621823					MG623596	MG614132		MG626183					MG628345		
Xylariophilussp.	MG621880	MG622220	MG622518	MG623068	MG623356	MG623650	MG614188	MG625283	MG626236	MG627428		MG627932		MG628400	MG629558	MG630187
Teredolaemussp.	MG621882	MG622221	MG622519	MG623069	MG623357	MG623652	MG614190	MG625284	MG626238	MG627429		MG627933	MG628163	MG628402	MG629559	
**Cucujoidea/Alexiidae**																
Sphaerosomasp.	MG621818	MG622170		MG623019	MG623302		MG614127	MG625231	MG626178	MG627371		MG627911	MG628126	MG628340	MG629506	MG630125
**Boganiidae**																
Paracucujusrostratus	MG621822		MG622467	MG623023	MG623306	MG623595	MG614131	MG625235	MG626182		MG627689		MG628130	MG628344	MG629510	MG630129
**Cryptophagidae**																
Micrambinasp.	MG621831	MG622179	MG622473	MG623028	MG623312	MG623604	MG614138	MG625240	MG626191	MG627382	MG627694	MG627913		MG628351	MG629517	MG630135
Cureliussp.	MG622139	MG622437	MG622746	MG623272	MG623564	MG623882	MG614441	MG625498	MG626445	MG627663	MG627890		MG628312	MG628654	MG629765	MG630420
**Cucujidae**																
Platisussp.	MG621832		MG622474	MG623029	MG623313	MG623605	MG614139	MG625241	MG626192	MG627383				MG628352	MG629518	MG630136
Pediacusmajor		MG622229	MG622530	MG623078	MG623365	MG623664	MG614204		MG626250	MG627442		MG627940	MG628171	MG628415	MG629568	MG630198
**Cybocephalidae**																
Cybocephalussp.	MG621896		MG622531		MG623366	MG623665		MG625296	MG626251	MG627443		MG627941		MG628416	MG629569	MG630199
**Erotylidae**																
Thallissp.	MG621907	MG622236	MG622542		MG623377	MG623676	MG614215	MG625307	MG626259	MG627452		MG627950		MG628427		MG630210
Anadastussp.	MG621908	MG622237	MG622543		MG623378	MG623677	MG614216	MG625308	MG626260	MG627453		MG627951		MG628428	MG629578	MG630211
Episcaphulasp.	MG621909	MG622238	MG622544	MG623087	MG623379	MG623678	MG614217	MG625309	MG626261	MG627454			MG628176	MG628429	MG629579	MG630212
Cryptophilussp.	MG621910	MG622239	MG622545	MG623088	MG623380	MG623679	MG614218	MG625310	MG626262		MG627732	MG627952	MG628177	MG628430	MG629580	MG630213
Episcaphasp.	MG621993	MG622308	MG622622	MG623155	MG623454	MG623752	MG614298	MG625382	MG626330	MG627530		MG628014		MG628511		MG630290
Tetraphalasp.	MG621998	MG622313	MG622627	MG623160	MG623459	MG623757	MG614303	MG625387	MG626335		MG627796	MG628017		MG628516	MG629654	MG630295
**Helotidae**																
Neohelotasp.	MG621976	MG622292	MG622608		MG623440	MG623741	MG614283	MG625368	MG626320	MG627513	MG627782				MG629637	MG630276
**Hobartiidae**																
Hydnobioidessp.	MG621914	MG622242	MG622549	MG623091	MG623383	MG623683	MG614221	MG625314	MG626266			MG627956		MG628434	MG629583	MG630216
**Kateretidae**																
Notobrachypterussp.	MG621842	MG622186		MG623036	MG623322	MG623615	MG614149		MG626200	MG627392	MG627700			MG628360	MG629525	MG630146
**Laemophloeidae**																
Laemophloeussp.	MG621918	MG622245	MG622553	MG623093	MG623387	MG623687	MG614225	MG625317	MG626269	MG627460	MG627737	MG627959	MG628181	MG628438	MG629586	MG630219
Cryptolestessp.	MG622140	MG622438	MG622747		MG623565	MG623883	MG614442	MG625499	MG626446	MG627664	MG627891	MG628108	MG628313	MG628655	MG629766	MG630421
**Monotomidae**																
Mimemodessp.		MG622192	MG622488	MG623042		MG623621	MG614156	MG625254	MG626206	MG627397			MG628142	MG628365	MG629531	MG630153
Monotomopsissp.	MG621926	MG622253	MG622560	MG623100	MG623395	MG623695	MG614233	MG625325	MG626277	MG627468	MG627742	MG627966	MG628186	MG628446	MG629593	MG630227
Rhizophagussp.	MG621927	MG622254	MG622561	MG623101		MG623696	MG614234	MG625326	MG626278	MG627469		MG627967	MG628187	MG628447	MG629594	
Thionesp.	MG622151	MG622447	MG622757	MG623281	MG623574	MG623894	MG614451	MG625507	MG626457	MG627673	MG627901	MG628117	MG628320	MG628667	MG629774	MG630432
**Myraboliidae**																
Myraboliasp.	MG621850	MG622194	MG622491	MG623044	MG623331	MG623624	MG614158	MG625256	MG626209	MG627400	MG627705			MG628368	MG629534	MG630156
**Nitidulidae**																
Brachypeplussp.	MG621929	MG622256	MG622563	MG623102	MG623396	MG623697	MG614236	MG625328	MG626280	MG627471	MG627743		MG628189	MG628449	MG629595	MG630229
Pallodessp.	MG621930	MG622257		MG623103	MG623397		MG614237	MG625329	MG626281	MG627472	MG627744	MG627969	MG628190	MG628450	MG629596	MG630230

Urophorussp.	MG622024	MG622334	MG622648		MG623478	MG623781	MG614328	MG625409	MG626355	MG627559		MG628029		MG628540	MG629676	MG630318
Carpophilussp.	MG622142	MG622439	MG622749	MG623274	MG623567	MG623885	MG614443	MG625501	MG626448	MG627666	MG627893	MG628110	MG628314	MG628657	MG629767	MG630423
Glischrochilusparvipustulatus	MG622143	MG622440	MG622750	MG623275	MG623568	MG623886	MG614444	MG625502	MG626449	MG627667	MG627894		MG628315	MG628658	MG629768	MG630424
**Passandridae**																
Passandrasp.	MG621933		MG622566	MG623104	MG623399	MG623700	MG614240	MG625332	MG626284	MG627475				MG628453	MG629599	MG630233
**Phalacridae**																
gen.sp.	MG621853	MG622197	MG622494	MG623047	MG623334	MG623627	MG614161	MG625259	MG626212	MG627403	MG627706	MG627918	MG628147	MG628371	MG629537	MG630159
Phalacrinussp.	MG621934	MG622259	MG622567	MG623105	MG623400	MG623701	MG614241	MG625333	MG626285	MG627476		MG627972	MG628191	MG628454	MG629600	MG630234
Olibrussp.	MG622084	MG622388	MG622700	MG623224	MG623524	MG623828	MG614387	MG625454	MG626402	MG627610	MG627856	MG628067	MG628277	MG628597		MG630368
**Phloeostichidae**																
Hymaeasp.	MG621935	MG622260	MG622568	MG623106	MG623401	MG623702	MG614242	MG625334	MG626286	MG627477		MG627973	MG628192	MG628455	MG629601	MG630235
Rhopalobrachiumcrowsoni	MG621936	MG622261	MG622569	MG623107	MG623402	MG623703	MG614243	MG625335	MG626287	MG627478	MG627747		MG628193	MG628456	MG629602	MG630236
**Propalticidae**																
Propalticussp.	MG621855	MG622199	MG622496	MG623049	MG623335	MG623629	MG614163	MG625261	MG626214	MG627405	MG627708		MG628149	MG628373	MG629539	MG630161
**Protocucujidae**																
Ericmodescostatus	MG621937	MG622262	MG622570		MG623403	MG623704	MG614244	MG625336	MG626288	MG627479	MG627748	MG627974		MG628457	MG629603	MG630237
**Silvanidae**																
Uleiotasp.	MG621864	MG622208	MG622503	MG623056	MG623343	MG623638	MG614172	MG625268	MG626222	MG627413				MG628383	MG629545	MG630170
Cryptamorphasp.	MG621951	MG622274	MG622583	MG623119		MG623717	MG614259		MG626300	MG627490		MG627983		MG628471		MG630252
Silvanoprusscuticollis			MG622584	MG623120	MG623417	MG623718	MG614260			MG627491					MG629614	MG630253
Psammoecussp.	MG622059		MG622678	MG623203	MG623505	MG623812	MG614363	MG625434	MG626380	MG627590	MG627837	MG628052	MG628264	MG628574	MG629702	MG630348
Psammoecussp.	MG622085	MG622389	MG622701	MG623225	MG623525		MG614388		MG626403	MG627611		MG628068		MG628598		MG630369
**Sphindidae**																
Aspidiphorussp.	MG621865			MG623057		MG623639	MG614173	MG625269	MG626223				MG628155			MG630171
**Curculionoidea/Anthribidae**																
Acorynussp.	MG621979	MG622294					MG614286			MG627516		MG628005		MG628497	MG629640	MG630279
Ozotomerussp.	MG622012					MG623769	MG614317			MG627547				MG628528	MG629666	MG630307
Xylinadasp.	MG622022						MG614326			MG627557		MG628028		MG628538		MG630316
Peribathyssp.	MG622066	MG622371					MG614369			MG627596				MG628581		MG630354
**Attelabidae**																
Phymatapoderussp.		MG622330	MG622644		MG623474	MG623777	MG614323	MG625405	MG626351	MG627554		MG628026	MG628239	MG628535	MG629672	MG630314
Involvulussp.	MG622039	MG622348	MG622661	MG623186	MG623489	MG623794	MG614343	MG625421	MG626365	MG627574			MG628251	MG628555		MG630330
Byctiscussp.	MG622041	MG622349	MG622662	MG623187		MG623795	MG614344	MG625422	MG626366	MG627575	MG627822	MG628041		MG628556	MG629688	MG630332
Paratrachelophorussp.	MG622135	MG622433		MG623268	MG623560	MG623878		MG625495	MG626442	MG627659	MG627886	MG628104		MG628650		MG630416
**Belidae**																
Rhinotiasp.	MG621876	MG622217		MG623064		MG623647	MG614184		MG626232	MG627424			MG628161	MG628396		MG630183
**Brentidae**																
Apions.l.sp.	MG621884						MG614192		MG626240	MG627431				MG628404		MG630188
Baryrhynchussp.	MG622013		MG622640			MG623770		MG625399		MG627548				MG628529	MG629667	MG630308
Apions.l.sp.	MG622040															MG630331
Cylassp.	MG622144													MG628659		
**Curculionidae**																
Episomussp.	MG621981	MG622296			MG623443		MG614288			MG627518		MG628007		MG628499		MG630281
gen.sp.	MG622014	MG622325	MG622641			MG623771		MG625400		MG627549				MG628530		MG630309
Xylosandrussp.	MG622017	MG622328		MG623171		MG623774	MG614320		MG626349	MG627552	MG627807	MG628025	MG628237	MG628533	MG629670	MG630312
Curculiosp.	MG622133	MG622431		MG623267		MG623877	MG614436			MG627657		MG628102		MG628648		MG630414
Peribleptussp.	MG622134	MG622432					MG614437	MG625494		MG627658		MG628103		MG628649		MG630415
**Nemonychidae**																
AragomaceruniformisKuschel	MG621928	MG622255	MG622562				MG614235	MG625327	MG626279	MG627470		MG627968	MG628188	MG628448		MG630228

**Dasciloidea/ Dascillidae**																
Dascillussp.	MG621997	MG622312	MG622626	MG623159	MG623458	MG623756	MG614302	MG625386	MG626334	MG627534	MG627795	MG628016	MG628227	MG628515	MG629653	MG630294
Metallidascillussp.	MG622145	MG622441	MG622751	MG623276	MG623569	MG623887	MG614445	MG625503	MG626450	MG627668	MG627895	MG628111	MG628316	MG628660	MG629769	MG630425
**Rhipiceridae**																
Oligorhipisbifossata	MG621861	MG622205	MG622500	MG623053	MG623340	MG623635	MG614169	MG625265	MG626220	MG627410	MG627710	MG627921	MG628152	MG628380	MG629542	MG630167
**Derodontoidea /Derodontidae**																
Derodontussp.	MG621899	MG622231	MG622534	MG623081	MG623369	MG623668	MG614207	MG625299	MG626253		MG627728	MG627944	MG628173	MG628419	MG629571	MG630202
Derolathrussp.	MG621841		MG622483		MG623321	MG623614	MG614148		MG626199	MG627391				MG628359		MG630145
**Jacobsoniidae**																
Derolathrussp.	MG621917		MG622552		MG623386	MG623686	MG614224		MG626268	MG627459				MG628437		
**Nosodendridae**																
Nosodendronsp.	MG621851	MG622195	MG622492	MG623045	MG623332	MG623625	MG614159	MG625257	MG626210	MG627401			MG628145	MG628369	MG629535	MG630157
**Elateroidea/Artematopodidae**																
Artematopussp.	MG621820	MG622172	MG622465	MG623021	MG623304	MG623593	MG614129	MG625233	MG626180	MG627373	MG627688		MG628128	MG628342	MG629508	MG630127
Eurypogonsp.	MG622147	MG622443	MG622753	MG623277	MG623570	MG623889	MG614446	MG625504	MG626452	MG627670	MG627897	MG628113	MG628318	MG628662	MG629771	MG630427
**Cantharidae**																
Heteromastixsp.	MG621886	MG622222	MG622522	MG623072		MG623655	MG614194	MG625287	MG626242	MG627433	MG627722	MG627936	MG628165	MG628406		MG630190
Themussp.	MG621973	MG622289	MG622605	MG623140	MG623437	MG623739	MG614280	MG625365		MG627510	MG627780	MG628000		MG628493	MG629634	MG630273
Themussp.	MG622030	MG622339	MG622654	MG623179	MG623483	MG623786	MG614334	MG625415	MG626357	MG627565		MG628033	MG628246	MG628546	MG629679	MG630322
Prothemussp.	MG622031	MG622340	MG622655	MG623180		MG623787	MG614335	MG625416	MG626358	MG627566	MG627814	MG628034	MG628247	MG628547	MG629680	MG630323
Ichthyurusbourgeoisi	MG622032	MG622341		MG623181	MG623484	MG623788	MG614336		MG626359	MG627567	MG627815	MG628035	MG628248	MG628548	MG629681	MG630324
Laemoglyptussp.	MG622110	MG622410	MG622722	MG623244	MG623541	MG623853	MG614412	MG625473	MG626422	MG627636	MG627869		MG628290	MG628625	MG629742	MG630391
Malthinussp.	MG622111	MG622411	MG622723	MG623245	MG623542	MG623854	MG614413	MG625474	MG626423	MG627637	MG627870	MG628089	MG628291	MG628626	MG629743	MG630392
Lycocerussp.	MG622112	MG622412	MG622724	MG623246		MG623855	MG614414	MG625475	MG626424	MG627638	MG627871		MG628292	MG628627	MG629744	MG630393
Fissocantharissp.	MG622113	MG622413	MG622725	MG623247	MG623543	MG623856	MG614415	MG625476	MG626425	MG627639	MG627872		MG628293	MG628628	MG629745	MG630394
**Elateridae**																
Osslimussp.	MG621900		MG622535	MG623082	MG623370	MG623669	MG614208	MG625300	MG626254		MG627729	MG627945	MG628174	MG628420	MG629572	MG630203
Hemicrepidiussp.	MG621987	MG622302	MG622617	MG623150	MG623449	MG623747	MG614293	MG625377	MG626326	MG627524	MG627787	MG628011	MG628221	MG628505	MG629645	MG630285
Pectocerasp.	MG622116	MG622416	MG622728	MG623250	MG623546	MG623859	MG614418	MG625479	MG626426	MG627642		MG628091	MG628296	MG628631	MG629748	MG630397
Agrypnussp.		MG622417	MG622729	MG623251		MG623860	MG614419	MG625480	MG626427	MG627643	MG627875	MG628092	MG628297	MG628632		MG630398
Ampedussp.	MG622117	MG622418	MG622730	MG623252		MG623861	MG614420	MG625481	MG626428	MG627644		MG628093	MG628298	MG628633	MG629749	MG630399
Denticollissp.	MG622118	MG622419	MG622731	MG623253	MG623547	MG623862	MG614421	MG625482	MG626429	MG627645	MG627876	MG628094	MG628299	MG628634	MG629750	MG630400
Peniasp.	MG622119	MG622420	MG622732	MG623254	MG623548	MG623863	MG614422	MG625483	MG626430	MG627646	MG627877	MG628095	MG628300	MG628635	MG629751	MG630401
gen.sp.	MG622120	MG622421	MG622733	MG623255	MG623549	MG623864	MG614423		MG626431	MG627647			MG628301	MG628636	MG629752	MG630402
Denticollissp.	MG622121	MG622422	MG622734	MG623256	MG623550	MG623865	MG614424	MG625484	MG626432	MG627648		MG628096	MG628302	MG628637	MG629753	MG630403
Cardiotarsussp.	MG622154		MG622759	MG623283		MG623897	MG614454		MG626459	MG627675						MG630435
Melanotussp.	MG622155	MG622448	MG622760	MG623284	MG623577	MG623898	MG614455	MG625510	MG626460	MG627676	MG627903	MG628118	MG628322	MG628669	MG629777	MG630436
Cebriosp.	MG622160		MG622764	MG623287	MG623581	MG623902	MG614459	MG625514	MG626464	MG627679			MG628325	MG628674		MG630440
**Eucnemidae**																
Anischiasp.	MG621837		MG622479	MG623032	MG623317	MG623610	MG614144	MG625246	MG626195		MG627696		MG628136	MG628355	MG629522	MG630141
Hemiopsidasp.	MG621912		MG622547	MG623089		MG623681	MG614219	MG625312	MG626264	MG627455	MG627733	MG627954	MG628178	MG628432		
Othosp.	MG622004	MG622318	MG622633	MG623165	MG623464	MG623762	MG614309	MG625392	MG626340	MG627540	MG627800		MG628231			MG630301
Pyrocoeliasp.	MG621988	MG622303	MG622618	MG623151	MG623450	MG623748	MG614294	MG625378		MG627525	MG627788	MG628012	MG628222	MG628506	MG629646	MG630286
Vestasp.	MG622026	MG622336	MG622650	MG623175	MG623479		MG614330	MG625411		MG627561			MG628242	MG628542		MG630319
Pristolycussp.	MG622027	MG622337	MG622651	MG623176	MG623480	MG623783	MG614331	MG625412		MG627562	MG627812	MG628030	MG628243	MG628543		MG630320
Luciolasp.	MG622028	MG622338	MG622652	MG623177	MG623481	MG623784	MG614332	MG625413		MG627563	MG627813	MG628031	MG628244	MG628544		MG630321
Cyphonocerussp.	MG622029		MG622653	MG623178	MG623482	MG623785	MG614333	MG625414		MG627564		MG628032	MG628245	MG628545	MG629678	
Drilastersp.	MG622114	MG622414	MG622726	MG623248	MG623544	MG623857	MG614416	MG625477		MG627640	MG627873		MG628294	MG628629	MG629746	MG630395
Diaphanessp.	MG622115	MG622415	MG622727	MG623249	MG623545	MG623858	MG614417	MG625478		MG627641	MG627874	MG628090	MG628295	MG628630	MG629747	MG630396

Gorhamiasp.	MG622159	MG622452	MG622763	MG623286	MG623580	MG623901	MG614458	MG625513		MG627678	MG627905		MG628324	MG628673	MG629780	MG630439
**Lycidae**																
Porrostomasp.	MG621921	MG622248	MG622556	MG623095	MG623390	MG623690	MG614228	MG625320	MG626272	MG627463		MG627962	MG628183	MG628441	MG629589	MG630222
Benibotarussp.	MG622161	MG622453	MG622765	MG623288	MG623582	MG623903		MG625515	MG626465	MG627680		MG628120	MG628326	MG628675		MG630441
Dilophotessp.	MG622162		MG622766	MG623289	MG623583	MG623904	MG614460	MG625516	MG626466	MG627681		MG628121	MG628327	MG628676	MG629781	MG630442
Platerodrilussp.	MG622163	MG622454	MG622767	MG623290	MG623584	MG623905	MG614461	MG625517	MG626467	MG627682			MG628328	MG628677		
Libnetissp.	MG622164	MG622455	MG622768	MG623291	MG623585	MG623906	MG614462	MG625518	MG626468	MG627683		MG628122	MG628329	MG628678	MG629782	MG630443
Macrolycussichuanensis	MG622165	MG622456	MG622769	MG623292	MG623586	MG623907	MG614463	MG625519	MG626469	MG627684			MG628330	MG628679	MG629783	MG630444
Lycostomussp.		MG622457	MG622770	MG623293	MG623587	MG623908	MG614464	MG625520	MG626470	MG627685		MG628123	MG628331	MG628680		MG630445
**Omethidae**																
Driloniussp.	MG622087	MG622391	MG622703	MG623227	MG623527	MG623830	MG614389	MG625456	MG626404	MG627613	MG627857		MG628279	MG628600	MG629723	MG630371
Driloniussp.	MG622089	MG622393	MG622705	MG623229	MG623529	MG623832	MG614391	MG625458	MG626406		MG627859	MG628071	MG628281	MG628602	MG629725	
**Phengodidae**																
Stenophrixothrixsp.	MG621854	MG622198	MG622495	MG623048		MG623628	MG614162	MG625260	MG626213	MG627404	MG627707	MG627919	MG628148	MG628372	MG629538	MG630160
**Rhagophthalmidae**																
Rhagophthalmussp.	MG621860	MG622204	MG622499	MG623052	MG623339	MG623634	MG614168	MG625264	MG626219	MG627409			MG628151	MG628379		MG630166
Rhagophthalmussp.	MG621940	MG622265	MG622573	MG623109	MG623406	MG623707	MG614247	MG625339	MG626290	MG627482				MG628460		MG630240
gen.sp.	MG622088	MG622392	MG622704	MG623228	MG623528	MG623831	MG614390	MG625457	MG626405	MG627614	MG627858	MG628070	MG628280	MG628601	MG629724	MG630372
**Throscidae**																
Trixagussp.			MG622506					MG625272		MG627416				MG628386		MG630174
Trixagussp.	MG622086	MG622390	MG622702	MG623226	MG623526	MG623829		MG625455		MG627612		MG628069	MG628278	MG628599	MG629722	MG630370
**Histeridae**																
Platysomasp.	MG621838	MG622183	MG622480	MG623033	MG623318	MG623611	MG614145	MG625247	MG626196	MG627388	MG627697	MG627915	MG628137	MG628356	MG629523	MG630142
Saprinussp.	MG621839	MG622184	MG622481	MG623034	MG623319	MG623612	MG614146	MG625248	MG626197	MG627389	MG627698		MG628138	MG628357	MG629524	MG630143
**Hydrophiloidea/ Hydrophilidae**																
Sternolophusimmarginatus	MG621915	MG622243	MG622550	MG623092	MG623384	MG623684	MG614222	MG625315	MG626267	MG627457	MG627735	MG627957		MG628435	MG629584	MG630217
Helocharessp.	MG621916	MG622244	MG622551		MG623385	MG623685	MG614223	MG625316		MG627458	MG627736	MG627958	MG628180	MG628436	MG629585	MG630218
Oocyclussp.	MG621985	MG622300	MG622615	MG623148	MG623447	MG623745	MG614291	MG625375	MG626324	MG627522	MG627785	MG628010		MG628503	MG629643	
Hydrophilusacuminatus	MG622067	MG622372	MG622684	MG623210			MG614370	MG625441	MG626387	MG627597	MG627844	MG628057		MG628582		MG630355
Anacaenapui	MG622069	MG622374	MG622686	MG623212	MG623513		MG614372	MG625442	MG626389	MG627599	MG627845	MG628059		MG628584	MG629709	MG630357
Georissussp.	MG622070	MG622375	MG622687		MG623514	MG623819	MG614373	MG625443	MG626390	MG627600	MG627846	MG628060	MG628268	MG628585	MG629710	MG630358
Sphaeridiumquinquemaculatum	MG622071	MG622376	MG622688	MG623213	MG623515	MG623820	MG614374	MG625444			MG627847		MG628269	MG628586	MG629711	MG630359
Berosusspinosus								MG625472						MG628624		
**Chrysomeloidea/ Cerambycidae**																
Strangaliafortunei	MG622033	MG622342	MG622656			MG623789	MG614337	MG625417	MG626360	MG627568	MG627816			MG628549	MG629682	MG630325
Obereasp.	MG622034	MG622343	MG622657			MG623790	MG614338	MG625418	MG626361	MG627569	MG627817	MG628036	MG628249	MG628550	MG629683	MG630326
Spondylisbuprestoides	MG622035	MG622344	MG622658	MG623182	MG623485	MG623791	MG614339	MG625419	MG626362	MG627570	MG627818	MG628037		MG628551	MG629684	MG630327
Xylotrechussp.	MG622036	MG622345		MG623183	MG623486	MG623792	MG614340	MG625420		MG627571	MG627819	MG628038		MG628552	MG629685	MG630328
Dorysthenes hydropicus	MG622108					MG623851	MG614410							MG628622		
**Chrysomelidae**																
Cassidasp.	MG621978	MG622293	MG622610				MG614285	MG625370		MG627515		MG628004		MG628496	MG629639	MG630278
Chrysomelapopuli	MG621990	MG622305	MG622619					MG625379		MG627527				MG628508		MG630287
Chlamisussp.	MG622042		MG622663	MG623188	MG623490	MG623796	MG614345	MG625423		MG627576			MG628252	MG628557	MG629689	MG630333
Sagrafulgidajanthina	MG622094	MG622396	MG622709	MG623234		MG623837	MG614396	MG625463	MG626410	MG627619		MG628075	MG628285	MG628607	MG629729	
Liliocerissp.	MG622095	MG622397	MG622710					MG625464	MG626411	MG627620		MG628076		MG628608	MG629730	MG630376
Dactylispasp.	MG622096	MG622398	MG622711				MG614397			MG627621		MG628077		MG628609	MG629731	MG630377
Anisoderasp.	MG622097	MG622399				MG623838	MG614398			MG627622		MG628078		MG628610	MG629732	MG630378
Sominellasp.	MG622098	MG622400	MG622712		MG623533	MG623839	MG614399	MG625465	MG626412	MG627623		MG628079		MG628611		MG630379
Trichochryseasp.	MG622099					MG623840	MG614400	MG625466		MG627624					MG629733	MG630380

gen.sp.		MG622401	MG622713	MG623235		MG623841	MG614401		MG626413	MG627625		MG628080	MG628286	MG628612	MG629734	MG630381
Alticasp.	MG622100	MG622402		MG623236		MG623842	MG614402		MG626414	MG627626		MG628081		MG628613	MG629735	MG630382
Oomorphoidessp.	MG622101	MG622403				MG623843	MG614403	MG625467		MG627627		MG628082		MG628614	MG629736	MG630383
Cryptocephalussp.	MG622102		MG622714	MG623237	MG623534	MG623844		MG625468	MG626415	MG627628	MG627863	MG628083		MG628615	MG629737	MG630384
Bruchidiussp.			MG622715			MG623845	MG614404			MG627629		MG628084		MG628616		MG630385
**Megalopodidae**																
Temnaspissp.	MG622000	MG622314	MG622629		MG623461	MG623759	MG614305	MG625388	MG626337	MG627536	MG627797		MG628228	MG628518	MG629656	MG630297
**Lymexyloidea/Lymexylidae**																
Melittommasp.	MG621847	MG622191	MG622487	MG623041	MG623328	MG623620	MG614155	MG625253	MG626205	MG627396			MG628141		MG629530	MG630152
Atractocerussp.	MG622058						MG614362		MG626379					MG628573		
**Rhinorhipoidea/Rhinorhipidae**																
Rhinorhipus tamborensis	AB123487	AB123488	AB123489	AB123490	AB123491	AB123492	AB123493	AB123494	AB123495	AB123496	AB123497	AB123498	AB123499	AB123500	AB123501	AB123502
**Scarabaeoidea/Geotrupidae**																
Australobolbussp.	MG621944	MG622267	MG622578	MG623113	MG623411	MG623711	MG614252	MG625343	MG626295	MG627485	MG627755		MG628199	MG628464	MG629609	MG630245
Geotrupessp.	MG622061	MG622367	MG622680	MG623205	MG623507	MG623814	MG614364	MG625436	MG626382	MG627592	MG627839	MG628053		MG628576	MG629704	MG630350
**Glaphyridae**																
Amphicomasp.	MG621982	MG622297	MG622612	MG623145	MG623444	MG623743		MG625372	MG626322	MG627519		MG628008	MG628219	MG628500	MG629641	
**Hybosoridae**																
Liparochrussp.	MG621840	MG622185	MG622482	MG623035	MG623320	MG623613	MG614147	MG625249	MG626198	MG627390	MG627699		MG628139	MG628358		MG630144
Cyphopisthessp.	MG621945	MG622268					MG614253	MG625344						MG628465		MG630246
**Lucanidae**																
Phalacrognathusmuelleri	MG622062		MG622681	MG623206	MG623508	MG623815	MG614365	MG625437	MG626383	MG627593	MG627840			MG628577	MG629705	MG630351
Cyclommatusmetallifer	MG622063	MG622368	MG622682	MG623207	MG623509	MG623816	MG614366	MG625438	MG626384	MG627594	MG627841	MG628054		MG628578	MG629706	
Aegussp.	MG622106	MG622407	MG622719	MG623241	MG623538	MG623849	MG614408	MG625469	MG626419	MG627633	MG627866	MG628086	MG628289	MG628620	MG629740	MG630389
**Passalidae**																
Ceracupessp.	MG622025	MG622335	MG622649			MG623782	MG614329	MG625410	MG626356	MG627560				MG628541	MG629677	
Aceraiussp.	MG622060	MG622366	MG622679	MG623204	MG623506	MG623813		MG625435	MG626381	MG627591	MG627838		MG628265	MG628575	MG629703	MG630349
**Scarabaeidae**																
Carneodonsp.	MG621946	MG622269	MG622579	MG623114	MG623412	MG623712	MG614254		MG626296	MG627486	MG627756		MG628200	MG628466		MG630247
Heteronyxsp.	MG621947	MG622270	MG622580	MG623115	MG623413	MG623713	MG614255			MG627487	MG627757	MG627980		MG628467	MG629610	MG630248
Anomalaantiqua	MG621948	MG622271	MG622581	MG623116	MG623414	MG623714	MG614256	MG625345	MG626297	MG627488	MG627758	MG627981	MG628201	MG628468	MG629611	MG630249
Onthophagusgazella	MG621949	MG622272		MG623117	MG623415	MG623715	MG614257	MG625346	MG626298	MG627489	MG627759			MG628469	MG629612	MG630250
Rhyparussp.	MG621974	MG622290	MG622606	MG623141	MG623438		MG614281	MG625366	MG626318	MG627511		MG628001			MG629635	MG630274
Protaetiaaerata	MG622068	MG622373	MG622685	MG623211	MG623512	MG623818	MG614371		MG626388	MG627598		MG628058		MG628583		MG630356
Mimelasp.	MG622103	MG622404	MG622716	MG623238	MG623535	MG623846	MG614405		MG626416	MG627630	MG627864	MG628085	MG628287	MG628617	MG629738	MG630386
Coprissp.	MG622104	MG622405	MG622717	MG623239	MG623536	MG623847	MG614406		MG626417	MG627631	MG627865		MG628288	MG628618	MG629739	MG630387
Ectinohopliasp.	MG622105	MG622406	MG622718	MG623240	MG623537	MG623848	MG614407		MG626418	MG627632				MG628619		MG630388
Dasyvalgussp.	MG622107	MG622408	MG622720	MG623242	MG623539	MG623850	MG614409	MG625470	MG626420	MG627634	MG627867	MG628087		MG628621		MG630390
Cheirotonusjansoni	MG622109	MG622409	MG622721	MG623243	MG623540	MG623852	MG614411	MG625471	MG626421	MG627635	MG627868	MG628088		MG628623	MG629741	
Allomyrinadichotoma	MG622125	MG622425	MG622738	MG623260	MG623554	MG623869	MG614428		MG626435	MG627651	MG627879			MG628641		MG630407
**Trogidae**																
Omorgussp.	MG621868	MG622210	MG622507	MG623059	MG623346	MG623640	MG614175	MG625273	MG626225	MG627417			MG628156	MG628387	MG629547	MG630175
Troxsp.	MG622005	MG622319	MG622634	MG623166	MG623465	MG623763	MG614310	MG625393	MG626341	MG627541	MG627801		MG628232	MG628521	MG629660	
**Scirtoidea/Clambidae**																
Clambussp.	MG621891	MG622225	MG622526	MG623075	MG623361	MG623660	MG614199	MG625292	MG626246	MG627438	MG627725		MG628169	MG628410	MG629565	MG630194
**Eucinetidae**																
Noteucinetussp.	MG621836	MG622182	MG622478	MG623031	MG623316	MG623609	MG614143	MG625245		MG627387	MG627695	MG627914	MG628135		MG629521	MG630140
Noteucinetussp.	MG621911	MG622240	MG622546		MG623381	MG623680		MG625311	MG626263			MG627953		MG628431	MG629581	MG630214
**Scirtidae**																

Pseudomicrocarasp.	MG621950	MG622273	MG622582	MG623118	MG623416	MG623716	MG614258	MG625347	MG626299		MG627760	MG627982	MG628202	MG628470	MG629613	MG630251
gen.sp.	MG622010	MG622323	MG622638		MG623470	MG623767	MG614315				MG627803	MG628023		MG628526	MG629664	MG630305
Elodessp.	MG622093	MG622395	MG622708	MG623233	MG623532	MG623836	MG614395	MG625462	MG626409	MG627618	MG627862	MG628074	MG628284	MG628606	MG629728	MG630375
**Staphylinoidea/Agyrtidae**																
Pterolomaforsstromii	MG622146	MG622442	MG622752			MG623888			MG626451	MG627669	MG627896	MG628112	MG628317	MG628661	MG629770	MG630426
**Hydraenidae**																
Hydraenasp.	MG622072	MG622377	MG622689		MG623516	MG623821	MG614375	MG625445	MG626391	MG627601	MG627848			MG628587	MG629712	MG630360
**Leiodidae**																
Agyrtodessp.	MG621845	MG622188	MG622485	MG623038	MG623325	MG623617	MG614152	MG625252	MG626203	MG627394	MG627701	MG627917		MG628362	MG629527	MG630149
gen.sp.	MG621846	MG622189		MG623039	MG623326	MG623618	MG614153				MG627702			MG628363	MG629528	MG630150
Agathidiumsp.		MG622190	MG622486	MG623040	MG623327	MG623619	MG614154		MG626204	MG627395	MG627703		MG628140	MG628364	MG629529	MG630151
**Ptiliidae**																
gen.sp.		MG622201					MG614165		MG626216					MG628375		MG630162
**Silphidae**																
Nicrophorusnepalensis	MG621992	MG622307	MG622621	MG623154	MG623453	MG623751	MG614297	MG625381	MG626329	MG627529	MG627791		MG628224	MG628510	MG629649	MG630289
Necrodeslittoralis	MG622043	MG622350	MG622664	MG623189	MG623491	MG623797	MG614346	MG625424		MG627577	MG627823		MG628253	MG628558	MG629690	MG630334
**Staphylinidae**																
Scaphidiumsp.	MG621866		MG622504		MG623344		MG614174	MG625270	MG626224	MG627414		MG627923		MG628384	MG629546	MG630172
gen.sp.	MG621952		MG622585	MG623121	MG623418	MG623719	MG614261			MG627492	MG627761	MG627984		MG628472	MG629615	MG630254
Staphylinussp.	MG621980	MG622295	MG622611	MG623144	MG623442	MG623742	MG614287	MG625371		MG627517	MG627783	MG628006	MG628218	MG628498		MG630280
Megalopaederussp.	MG621999		MG622628	MG623161	MG623460	MG623758	MG614304		MG626336	MG627535		MG628018		MG628517	MG629655	MG630296
Apateticasp.	MG622050	MG622357		MG623196	MG623497	MG623804	MG614353	MG625429	MG626373	MG627582	MG627829	MG628046	MG628259	MG628565	MG629697	MG630340
Priochirussp.	MG622051	MG622358	MG622671	MG623197	MG623498	MG623805	MG614354		MG626374	MG627583	MG627830	MG628047	MG628260	MG628566	MG629698	MG630341
Osoriussp.	MG622052	MG622359	MG622672	MG623198	MG623499	MG623806	MG614355			MG627584	MG627831	MG628048		MG628567	MG629699	MG630342
Tachinussp.	MG622053	MG622360	MG622673	MG623199	MG623500	MG623807	MG614356	MG625430	MG626375	MG627585	MG627832	MG628049	MG628261	MG628568		MG630343
Scaphidiumsp.	MG622054	MG622361	MG622674		MG623501	MG623808	MG614357			MG627586	MG627833					MG630344
Tachinussp.	MG622055	MG622362	MG622675	MG623200	MG623502	MG623809	MG614358	MG625431	MG626376	MG627587	MG627834	MG628050	MG628262	MG628569		MG630345
Centrophthalmussp.		MG622363					MG614359							MG628570		
gen.sp.	MG622128		MG622741			MG623872	MG614431							MG628644		MG630409
Dianoussp.	MG622129	MG622428		MG623263	MG623556	MG623873	MG614432	MG625490	MG626438	MG627654	MG627882	MG628100	MG628308	MG628645	MG629758	MG630410
gen.sp.	MG622130	MG622429		MG623264	MG623557	MG623874	MG614433	MG625491	MG626439	MG627655	MG627883		MG628309	MG628646	MG629759	MG630411
**Tenebrionoidea/Aderidae**																
gen.sp.	MG621817		MG622463		MG623301	MG623591		MG625230					MG628125	MG628339	MG629505	MG630124
gen.sp.	MG622150	MG622446	MG622756		MG623573	MG623893	MG614450		MG626456		MG627900	MG628116		MG628666	MG629773	MG630431
**Anthicidae**																
Lemodessp.	MG621819	MG622171	MG622464	MG623020	MG623303	MG623592	MG614128	MG625232	MG626179	MG627372	MG627687		MG628127	MG628341	MG629507	MG630126
Anthicuss.l.sp.		MG622214	MG622511		MG623350		MG614180	MG625277	MG626229	MG627421		MG627925	MG628158	MG628392	MG629551	MG630179
Trichanancasp.	MG621873	MG622215	MG622512	MG623062	MG623351	MG623644	MG614181	MG625278	MG626230	MG627422	MG627717	MG627926	MG628159	MG628393	MG629552	MG630180
Macratriasp.	MG621874		MG622513			MG623645	MG614182	MG625279				MG627927		MG628394	MG629553	MG630181
Macratriasp.	MG622019		MG622643	MG623172		MG623776	MG614322	MG625404		MG627553	MG627809		MG628238	MG628534	MG629671	MG630313
**Archeocrypticidae**																
Wattianusqueenslandicus	MG621875	MG622216	MG622514	MG623063	MG623352	MG623646	MG614183	MG625280	MG626231	MG627423	MG627718	MG627928	MG628160	MG628395	MG629554	MG630182
**Boridae**																
Synercticussp.	MG621878		MG622516	MG623066	MG623354	MG623649	MG614186		MG626234	MG627426	MG627720	MG627930		MG628398	MG629556	MG630185
**Ciidae**																
Cissp.	MG621829	MG622177	MG622471	MG623026	MG623310	MG623602	MG614136	MG625238	MG626189	MG627380	MG627692		MG628133	MG628349	MG629515	MG630133
Cissp.	MG621889		MG622524			MG623658	MG614197	MG625290	MG626244	MG627436			MG628167	MG628408	MG629563	MG630192
Australocissp.	MG621890	MG622224	MG622525	MG623074	MG623360	MG623659	MG614198	MG625291	MG626245	MG627437	MG627724		MG628168	MG628409	MG629564	MG630193
**Tenebrionoidea/IncertaeSedis**																

Rhizoniumantiquum	MG621913	MG622241	MG622548	MG623090	MG623382	MG623682	MG614220	MG625313	MG626265	MG627456	MG627734	MG627955	MG628179	MG628433	MG629582	MG630215
**Ischaliidae**																
Ischaliasp.	MG622018	MG622329				MG623775	MG614321	MG625403	MG626350		MG627808					
**Melandryidae**																
Dircaeomorphasp.	MG622023	MG622333	MG622647	MG623174	MG623477	MG623780	MG614327	MG625408	MG626354	MG627558	MG627811		MG628241	MG628539	MG629675	MG630317
gen.sp.	MG622148	MG622444	MG622754	MG623278	MG623571	MG623890	MG614447	MG625505	MG626453	MG627671	MG627898	MG628114	MG628319	MG628663	MG629772	MG630428
**Meloidae**																
Zonitiss.l.sp.	MG621922	MG622249	MG622557	MG623096	MG623391	MG623691	MG614229	MG625321	MG626273	MG627464		MG627963	MG628184	MG628442	MG629590	MG630223
Epicautasp.	MG622057	MG622365	MG622677	MG623202	MG623504	MG623811	MG614361	MG625433	MG626378	MG627589	MG627836	MG628051		MG628572	MG629701	MG630347
**Mordellidae**																
Hoshihananomiasp.	MG622003	MG622317	MG622632	MG623164	MG623463		MG614308	MG625391	MG626339	MG627539	MG627799	MG628020	MG628230		MG629659	MG630300
**Mycetophagidae**																
Nototriphyllussp.	MG621848	MG622193	MG622489	MG623043	MG623329	MG623622	MG614157	MG625255	MG626207	MG627398	MG627704		MG628143	MG628366	MG629532	MG630154
Mycetophagussp.	MG622016	MG622327	MG622642		MG623473	MG623773	MG614319	MG625402	MG626348	MG627551	MG627806		MG628236	MG628532	MG629669	MG630311
**Mycteridae**																
Trichosalpingussp.	MG621849		MG622490		MG623330	MG623623			MG626208	MG627399			MG628144	MG628367	MG629533	MG630155
**Oedemeridae**																
Thelyphassasp.	MG621931		MG622564			MG623698	MG614238	MG625330	MG626282	MG627473	MG627745	MG627970		MG628451	MG629597	MG630231
Pseudolycussp.	MG621932	MG622258	MG622565		MG623398	MG623699	MG614239	MG625331	MG626283	MG627474	MG627746	MG627971		MG628452	MG629598	MG630232
Ditylussp.	MG622002	MG622316	MG622631	MG623163	MG623462	MG623761	MG614307	MG625390	MG626338	MG627538	MG627798		MG628229	MG628520	MG629658	MG630299
Pyrochroidae																
Morpholycussp.	MG621859	MG622203	MG622498	MG623051	MG623338	MG623633	MG614167	MG625263	MG626218	MG627408	MG627709	MG627920	MG628150	MG628378	MG629541	MG630165
MorpholycusmonilicornisLea	MG621938	MG622263	MG622571	MG623108	MG623404	MG623705	MG614245	MG625337	MG626289	MG627480	MG627749	MG627975	MG628194	MG628458	MG629604	MG630238
Pseudopyrochroasp.	MG621991	MG622306	MG622620	MG623153	MG623452	MG623750	MG614296	MG625380	MG626328	MG627528	MG627790			MG628509	MG629648	MG630288
Eupyrochroainsignita	MG621996	MG622311	MG622625	MG623158	MG623457	MG623755	MG614301	MG625385	MG626333	MG627533	MG627794	MG628015	MG628226	MG628514	MG629652	MG630293
**Pythidae**																
Anaplopussp.	MG621939	MG622264	MG622572		MG623405	MG623706	MG614246	MG625338		MG627481	MG627750	MG627976	MG628195	MG628459	MG629605	MG630239
**Ripiphoridae**																
Trigonoderasp.	MG621862	MG622206	MG622501	MG623054	MG623341	MG623636	MG614170	MG625266		MG627411	MG627711	MG627922	MG628153	MG628381	MG629543	MG630168
Rhipidioidessp.		MG622266	MG622574		MG623407		MG614248	MG625340	MG626291		MG627751	MG627977		MG628461		MG630241
**Salpingidae**																
OrphanotrophiumpallidipennisLea	MG621863	MG622207	MG622502	MG623055	MG623342	MG623637	MG614171	MG625267	MG626221	MG627412	MG627712		MG628154	MG628382	MG629544	MG630169
Euryplatusdimidiatus	MG621941		MG622575	MG623110	MG623408	MG623708	MG614249	MG625341	MG626292		MG627752		MG628196		MG629606	MG630242
Orphanotrophiumsp.	MG621942		MG622576	MG623111	MG623409	MG623709	MG614250	MG625342	MG626293	MG627483	MG627753	MG627978	MG628197	MG628462	MG629607	MG630243
Ocholissasp.	MG621943		MG622577	MG623112	MG623410	MG623710	MG614251		MG626294	MG627484	MG627754	MG627979	MG628198	MG628463	MG629608	MG630244
**Scraptiidae**																
Scraptiasp.			MG622755	MG623280	MG623572	MG623892	MG614449	MG625506	MG626455					MG628665		MG630430
**Tenebrionidae**																
Amarygmussp.	MG621953	MG622275	MG622586	MG623122	MG623419	MG623720		MG625348		MG627493	MG627762		MG628203	MG628473	MG629616	MG630255
Cyphaleussp.	MG621954		MG622587			MG623721	MG614262	MG625349	MG626301	MG627494	MG627763		MG628204	MG628474	MG629617	MG630256
Adeliumsp.	MG621955	MG622276	MG622588	MG623123		MG623722	MG614263	MG625350	MG626302	MG627495	MG627764	MG627985	MG628205	MG628475	MG629618	MG630257
Cillibussp.	MG621956		MG622589	MG623124	MG623420	MG623723	MG614264		MG626303	MG627496	MG627765	MG627986	MG628206	MG628476	MG629619	MG630258
Palorussp.	MG621957		MG622590	MG623125	MG623421	MG623724		MG625351	MG626304	MG627497	MG627766	MG627987		MG628477	MG629620	MG630259
Derispiasp.	MG621958	MG622277	MG622591	MG623126	MG623422	MG623725	MG614265	MG625352	MG626305	MG627498	MG627767	MG627988	MG628207	MG628478	MG629621	MG630260
Cossyphussp.	MG621959	MG622278	MG622592		MG623423	MG623726	MG614266		MG626306	MG627499	MG627768		MG628208	MG628479	MG629622	MG630261
Tanychilussp.	MG621960		MG622593	MG623127	MG623424	MG623727	MG614267	MG625353		MG627500	MG627769		MG628209	MG628480	MG629623	
Platydemasp.	MG621961	MG622279	MG622594	MG623128	MG623425	MG623728	MG614268	MG625354		MG627501	MG627770	MG627989	MG628210	MG628481	MG629624	MG630262
Tyrtaeussp.	MG621962		MG622595	MG623129	MG623426	MG623729	MG614269	MG625355			MG627771			MG628482	MG629625	MG630263
Ecnolagriasp.	MG621963		MG622596	MG623130		MG623730	MG614270	MG625356	MG626307	MG627502	MG627772	MG627990		MG628483	MG629626	MG630264

Chlorophilasp.	MG622015	MG622326			MG623472	MG623772	MG614318	MG625401	MG626347	MG627550	MG627805	MG628024		MG628531	MG629668	MG630310
Derispiasp.	MG622037	MG622346	MG622659	MG623184	MG623487	MG623793	MG614341		MG626363	MG627572	MG627820	MG628039	MG628250	MG628553	MG629686	
Cteniopinussp.	MG622038	MG622347	MG622660	MG623185	MG623488		MG614342		MG626364	MG627573	MG627821	MG628040		MG628554	MG629687	MG630329
Strongyliumsp.	MG622131	MG622430	MG622742	MG623265	MG623558	MG623875	MG614434	MG625492	MG626440	MG627656	MG627884	MG628101	MG628310	MG628647	MG629760	MG630412
Cryphaeussp.	MG622132		MG622743	MG623266	MG623559	MG623876	MG614435	MG625493	MG626441		MG627885		MG628311		MG629761	MG630413
Trictenotomasp.	MG622152				MG623575	MG623895	MG614452	MG625508			MG627902				MG629775	MG630433
**Ulodidae**																
Ulodessp.		MG622283	MG622598	MG623134	MG623430	MG623734	MG614273	MG625358	MG626311	MG627506	MG627776		MG628211	MG628487	MG629628	MG630267
Meryxsp.	MG621967	MG622284	MG622599	MG623135	MG623431	MG623735	MG614274	MG625359	MG626312	MG627507	MG627777	MG627994	MG628212	MG628488	MG629629	MG630268
**Zopheridae**																
Monommasp.																
Bitomasp.	MG621871	MG622212	MG622509		MG623348	MG623642	MG614178	MG625275	MG626227	MG627419	MG627715			MG628390	MG629549	MG630177
Zopherosisgeorgei	MG621968	MG622285	MG622600		MG623432	MG623736	MG614275	MG625360	MG626313	MG627508		MG627995	MG628213	MG628489		MG630269

	Rpn1	Rpn3	sec71	Shal	Shot	SIPA1L1	Spt6	Su(H)	Sur-8	sxc	Taf2	TfIIEalpha	Top2	Trpml	Vacht	VGAT	wls
**Outgroup**																	
Neochauliodesorientalis	MG630864	MG631176		MG632395	MG632743	MG629141	MG633034	MG633286	MG633606			MG634420		MG614921		MG635997	
Ascalohybrissubjacens	MG630863	MG631175	MG632098	MG632394	MG632742	MG629140	MG633033	MG633285	MG633605	MG633904	MG634156	MG634419	MG635049	MG614920	MG635667	MG635996	
Dichochrysaformosana	MG630862	MG631174	MG632097	MG632393	MG632741	MG629139	MG633032	MG633284		MG633903	MG634155	MG634418	MG635048	MG614919		MG635995	
Myrmeleonsp.	MG630861	MG631173	MG632096		MG632740	MG629138	MG633031			MG633902	MG634154	MG634417	MG635047	MG614918	MG635666		
**Adephaga / Carabidae**																	
Dischissussp.	MG630874	MG631187	MG632109	MG632407	MG632754			MG633298	MG633616	MG633913	MG634160	MG634430	MG635059	MG614928	MG635678	MG636008	
Carabussp.	MG630938	MG631266	MG632168	MG632480	MG632828	MG629202	MG633089	MG633369	MG633686	MG633978	MG634200	MG634505	MG635118	MG614996	MG635746	MG636086	MG636632
Cicindelachinensis	MG630939	MG631267	MG632169	MG632481	MG632829	MG629203		MG633370	MG633687	MG633979	MG634201		MG635119	MG614997	MG635747	MG636087	
Omoglymmiussp.		MG631282	MG632181	MG632498	MG632845		MG633099	MG633383	MG633703	MG633995	MG634210				MG635760	MG636102	
Clivinasp.	MG630986	MG631325	MG632208	MG632537	MG632885	MG629242	MG633124	MG633421	MG633737	MG634034		MG634556	MG635161	MG615049	MG635800	MG636140	MG636681
Pheropsophussp.		MG631326	MG632209	MG632538	MG632886		MG633125	MG633422	MG633738	MG634035		MG634557	MG635162	MG615050	MG635801	MG636141	
Lebiacoelestis		MG631328	MG632210	MG632540	MG632887			MG633424	MG633740	MG634037	MG634235	MG634559	MG635164	MG615052	MG635803	MG636143	
Pentagonicaruficollis	MG630988	MG631329	MG632211	MG632541	MG632888		MG633127	MG633425	MG633741	MG634038	MG634236	MG634560	MG635165	MG615053	MG635804	MG636144	MG636683
Elaphrussp.	MG631008	MG631357	MG632234	MG632569	MG632915	MG629261	MG633145	MG633450	MG633766	MG634060	MG634251	MG634590	MG635188	MG615074	MG635831	MG636172	
gen.sp.		MG631358	MG632236	MG632571	MG632917	MG629263	MG633147	MG633451	MG633767		MG634253		MG635190	MG615075	MG635833	MG636174	
**Dytiscidae**																	
Agabussp.		MG631274	MG632174	MG632489	MG632837			MG633375	MG633694	MG633986		MG634512	MG635127	MG615001	MG635753	MG636095	
Laccophilusdifficilis		MG631275	MG632175	MG632490	MG632838	MG629209	MG633095	MG633376	MG633695	MG633987	MG634205	MG634513	MG635128	MG615002	MG635754	MG636096	MG636638
Eretesgriseus	MG630944	MG631276	MG632176	MG632491	MG632839			MG633377	MG633696	MG633988				MG615003	MG635755	MG636097	
Rhantussuturalis	MG630945	MG631277	MG632177	MG632492	MG632840	MG629210		MG633378	MG633697	MG633989	MG634206		MG635129	MG615004	MG635756	MG636098	
Hyphydrusdetectus	MG630946	MG631278		MG632493	MG632841			MG633379	MG633698	MG633990			MG635130	MG615005		MG636099	
**Gyrinidae**																	
Orectochilussp.	MG630892	MG631209	MG632124		MG632774			MG633317	MG633634	MG633933	MG634173	MG634449		MG614949		MG636029	
**Haliplidae**																	
Peltodytessp.	MG630950	MG631283	MG632182	MG632499	MG632846	MG629214		MG633384	MG633704	MG633996	MG634211	MG634517	MG635135	MG615010	MG635761	MG636103	
**Noteridae**																	
gen.sp.	MG630764	MG631055		MG632279	MG632621	MG629045	MG632950	MG633186	MG633498	MG633808	MG634091		MG634947	MG614819	MG635549		MG636433
Canthydrussp.	MG630947	MG631279		MG632494	MG632842	MG629211	MG633096	MG633380	MG633699	MG633991	MG634207	MG634514	MG635131	MG615006	MG635757		MG636639
**Archostemata/ Cupedidae**																	
Tenomergasp.	MG630949		MG632179	MG632496		MG629213	MG633098		MG633701	MG633993	MG634209		MG635133	MG615008		MG636100	
**Myxophaga / Torridincolidae**																	
Satoniusschoenmanni	MG630948	MG631280	MG632178	MG632495	MG632843	MG629212	MG633097	MG633381	MG633700	MG633992	MG634208	MG634515	MG635132	MG615007	MG635758		MG636640
**Bostrichoidea /Bostrichidae**																	
Lyctussp.	MG630786	MG631084	MG632034	MG632303	MG632650	MG629067		MG633207	MG633525	MG633830		MG634330		MG614842	MG635575	MG635908	MG636459
Polycaonsp.		MG631204	MG632120	MG632423	MG632771		MG633051	MG633312	MG633630	MG633929		MG634445	MG635072	MG614945		MG636024	MG636572
**Dermestidae**																	
Dermestessp.	MG630800	MG631101	MG632045	MG632320	MG632667	MG629078	MG632979	MG633220	MG633540	MG633843		MG634347	MG634985	MG614855	MG635591	MG635923	MG636476
Evorineasp.	MG630801	MG631102		MG632321	MG632668	MG629079	MG632980	MG633221	MG633541	MG633844		MG634348	MG634986	MG614856	MG635592	MG635924	MG636477
Orphinussp.	MG630956	MG631290	MG632187	MG632504	MG632853	MG629218	MG633103	MG633390	MG633711	MG634003	MG634214	MG634522	MG635142	MG615017	MG635768	MG636109	MG636648
**Ptinidae**																	
Ptinussp.	MG630767	MG631060	MG632013	MG632283	MG632627		MG632954		MG633502			MG634305	MG634951	MG614823		MG635886	MG636437
Dorcatomasp.	MG630768	MG631061	MG632014	MG632284	MG632628	MG629049	MG632955	MG633190	MG633503		MG634095	MG634306	MG634952	MG614824	MG635553	MG635887	MG636438
Ptinussp.	MG630958	MG631292		MG632506	MG632855	MG629220						MG634524	MG635143	MG615019		MG636111	MG636650
Hedobiasp.	MG631002	MG631349		MG632562	MG632908			MG633443	MG633759		MG634245	MG634582	MG635183	MG615069	MG635823	MG636164	MG636703
**Buprestoidea / Buprestidae**																	
Coroebussp.	MG630878	MG631192	MG632111	MG632412	MG632759	MG629150	MG633043	MG633302	MG633621	MG633918		MG634435	MG635061	MG614933	MG635683	MG636012	MG636560

Dicercasp.		MG631251	MG632154	MG632467	MG632814	MG629191	MG633078	MG633355	MG633672	MG633966		MG634489		MG614981		MG636069	MG636616
Trachyssp.		MG631252	MG632155	MG632468	MG632815	MG629192	MG633079	MG633356		MG633967	MG634193	MG634490	MG635109	MG614982	MG635732	MG636070	MG636617
**Byrrhoidea / Byrrhidae**																	
Notolioonsp.	MG630742	MG631026	MG631989	MG632253	MG632592	MG629026	MG632932		MG633469				MG634925	MG614791	MG635522	MG635855	MG636406
Microchaetessp.	MG630791	MG631089	MG632039	MG632308	MG632654	MG629070	MG632972	MG633209	MG633529	MG633834	MG634106	MG634335	MG634974		MG635579	MG635912	MG636464
Cytilussp.	MG630905	MG631224		MG632441	MG632790	MG629170		MG633332	MG633648	MG633945		MG634464	MG635088	MG614961	MG635709	MG636043	MG636590
**Callirhipidae**																	
Ennometessp.		MG631027	MG631990	MG632254	MG632593				MG633470	MG633787		MG634273	MG634926	MG614792	MG635523	MG635856	MG636407
Simianussp.		MG631281	MG632180	MG632497	MG632844			MG633382	MG633702	MG633994		MG634516	MG635134	MG615009	MG635759	MG636101	MG636641
**Dryopidae**																	
Helichussp.		MG631036	MG631998	MG632261	MG632602				MG633479	MG633795		MG634282	MG634934	MG614800	MG635532	MG635863	MG636415
Pachyparnussp.	MG630985	MG631324	MG632207	MG632536	MG632884				MG633736	MG634033		MG634555	MG635160	MG615048		MG636139	MG636680
Helichussp.		MG631342	MG632223	MG632555	MG632901			MG633436	MG633752	MG634047		MG634574	MG635176	MG615062	MG635816	MG636157	MG636696
**Elmidae**																	
Graphelmissp.		MG631105	MG632048	MG632324	MG632671	MG629082	MG632982	MG633224	MG633544	MG633847	MG634111	MG634351	MG634989	MG614859	MG635595	MG635927	MG636480
Stetholussp.	MG630804	MG631106	MG632049	MG632325	MG632672		MG632983	MG633225	MG633545	MG633848		MG634352	MG634990	MG614860	MG635596	MG635928	MG636481
Stenelmissp.	MG630893	MG631210	MG632125	MG632427	MG632775			MG633318	MG633635	MG633934	MG634174	MG634450			MG635698	MG636030	MG636576
**Eulichadidae**																	
Eulichassp.		MG631198	MG632116	MG632418	MG632765	MG629153		MG633306	MG633625	MG633923		MG634439	MG635067	MG614939	MG635688	MG636018	MG636566
**Heteroceridae**																	
Heterocerussp.	MG630933	MG631260	MG632162	MG632473	MG632822	MG629195	MG633083	MG633363	MG633679	MG633971		MG634498	MG635111	MG614989	MG635739	MG636078	MG636625
**Chelonariidae**																	
Chelonariumsp.		MG631030	MG631992	MG632256	MG632596			MG633164	MG633473	MG633789		MG634276	MG634928	MG614794	MG635526	MG635857	MG636409
**Limnichidae**																	
Byrrhinussp.	MG630817	MG631123	MG632061	MG632343	MG632689	MG629096	MG632992	MG633240	MG633561	MG633861	MG634117	MG634369	MG635001	MG614873	MG635614	MG635946	MG636496
Pelocharessp.	MG630896	MG631214	MG632129	MG632431	MG632779	MG629160	MG633056	MG633322	MG633639		MG634177	MG634454	MG635080	MG614953	MG635702	MG636034	MG636580
Cephalobyrrhussp.	MG630927	MG631250	MG632153	MG632466	MG632813	MG629190		MG633354	MG633671	MG633965	MG634192	MG634488	MG635108	MG614980	MG635731	MG636068	MG636615
Limnichussp.	MG631009		MG632235	MG632570	MG632916	MG629262	MG633146				MG634252		MG635189		MG635832	MG636173	MG636709
**Psephenidae**																	
Sclerocyphonsp.		MG631059			MG632625							MG634304				MG635884	
Schinostethussp.	MG630904	MG631223	MG632133	MG632440	MG632789	MG629169	MG633064	MG633331	MG633647	MG633944		MG634463	MG635087	MG614960	MG635708	MG636042	MG636589
Schinostethussp.	MG630925	MG631248	MG632151	MG632464	MG632811	MG629188	MG633077	MG633352	MG633669	MG633963		MG634486	MG635106	MG614978	MG635729	MG636066	MG636613
Mataeopsephussp.	MG630926	MG631249	MG632152	MG632465	MG632812	MG629189		MG633353	MG633670	MG633964		MG634487	MG635107	MG614979	MG635730	MG636067	MG636614
**Ptilodactylidae**																	
Ptilodactylasp.	MG630875	MG631189	MG632110	MG632409	MG632756	MG629147	MG633041	MG633300	MG633618	MG633915	MG634161	MG634432	MG635060	MG614930	MG635680	MG636009	MG636557
Epilichassp.	MG630924	MG631247	MG632150	MG632463	MG632810	MG629187		MG633351	MG633668	MG633962		MG634485	MG635105	MG614977	MG635728	MG636065	MG636612
**Cleroidea /Acanthocnemidae**																	
Acanthocnemusnigricans	MG630779	MG631076	MG632026	MG632295	MG632642	MG629061			MG633518	MG633823		MG634322	MG634966	MG614835	MG635567	MG635900	MG636451
**Biphyllidae**																	
Althaesiasp.		MG631023	MG631987	MG632251	MG632589			MG633161	MG633467	MG633785		MG634270	MG634923	MG614789	MG635519	MG635852	MG636404
Biphyllussp.		MG631082	MG632032	MG632301	MG632648			MG633205	MG633523	MG633828	MG634104	MG634328	MG634970	MG614840	MG635573	MG635906	MG636457
**Byturidae**																	
Haematoidessp.	MG630894	MG631211	MG632126	MG632428	MG632776		MG633054	MG633319	MG633636	MG633935	MG634175	MG634451	MG635077	MG614950	MG635699	MG636031	MG636577
**Cleridae**																	
Tenerussp.					MG632661							MG634341					
Necrobiasp.		MG631096	MG632042	MG632315	MG632662	MG629075			MG633536	MG633840		MG634342	MG634980	MG614851	MG635586	MG635919	MG636471
Xenorthriussp.	MG630873	MG631186		MG632406	MG632753			MG633297	MG633615	MG633912		MG634429	MG635058	MG614927	MG635677	MG636007	MG636556
Cladiscussp.		MG631337	MG632218	MG632550	MG632896				MG633747	MG634042		MG634569	MG635171	MG615059	MG635811	MG636152	MG636692
Stenocallimerussp.		MG631338	MG632219	MG632551	MG632897	MG629246		MG633432	MG633748	MG634043	MG634240	MG634570	MG635172	MG615060	MG635812	MG636153	

Allochotessp.		MG631339	MG632220	MG632552	MG632898	MG629247		MG633433	MG633749	MG634044		MG634571	MG635173		MG635813	MG636154	MG636693
**Melyridae**																	
Dasytess.l.sp.		MG631126		MG632345	MG632692			MG633243		MG633863			MG635003	MG614874	MG635617	MG635949	MG636498
Dicranolaiusbellulus	MG630819	MG631127		MG632346	MG632693	MG629098	MG632994	MG633244	MG633564	MG633864		MG634372	MG635004	MG614875	MG635618	MG635950	MG636499
Carphurussp.	MG630820	MG631128	MG632063	MG632347	MG632694		MG632995	MG633245	MG633565	MG633865	MG634120	MG634373		MG614876	MG635619	MG635951	MG636500
gen.sp.		MG631179	MG632102	MG632398	MG632746			MG633289	MG633609	MG633907	MG634158	MG634421	MG635052	MG614923	MG635671	MG636000	MG636548
Carphurussp.	MG630957	MG631291	MG632188	MG632505	MG632854	MG629219	MG633104	MG633391	MG633712	MG634004	MG634215	MG634523		MG615018	MG635769	MG636110	MG636649
**Prionoceridae**																	
Idgiasp.	MG630895	MG631212	MG632127	MG632429	MG632777			MG633320	MG633637	MG633936		MG634452	MG635078	MG614951	MG635700	MG636032	MG636578
**Thanerocleridae**																	
Isoclerussp.		MG631070	MG632022	MG632290	MG632636	MG629057	MG632963		MG633512	MG633819		MG634315	MG634961	MG614829	MG635562	MG635896	MG636447
**Trogossitidae**																	
Larinotusumblicatus		MG631073	MG632024	MG632292	MG632638	MG629059			MG633515	MG633821		MG634318	MG634964	MG614832	MG635565	MG635898	MG636449
Rentonellumsp.		MG631074		MG632293	MG632639				MG633516			MG634319		MG614833			
Ancyronasp.		MG631167	MG632090	MG632387	MG632734				MG633600	MG633896		MG634411		MG614912	MG635660	MG635991	MG636539
Parapeltisaustralicum	MG630857	MG631168	MG632091	MG632388	MG632735	MG629134	MG633027	MG633279	MG633601	MG633897		MG634412	MG635042	MG614913	MG635661		MG636540
Leperinasp.		MG631169	MG632092	MG632389	MG632736			MG633280	MG633602	MG633898		MG634413	MG635043	MG614914	MG635662	MG635992	MG636541
Thymalussp.	MG630883	MG631197	MG632115	MG632417	MG632764				MG633624	MG633922		MG634438	MG635066	MG614938	MG635687	MG636017	MG636565
**Coccinelloidea /Anamorphidae**																	
Papuellasp.	MG630805	MG631107	MG632050	MG632326	MG632673	MG629083		MG633226	MG633546			MG634353	MG634991	MG614861	MG635597	MG635929	
**Bothrideridae**																	
Deretaphrussp.												MG634332					
Ascetoderessp.	MG630789	MG631087	MG632037	MG632306	MG632653	MG629069	MG632971		MG633528	MG633833		MG634334	MG634973	MG614845	MG635578		MG636462
**Cerylonidae**																	
Philothermussp.		MG631029	MG631991	MG632255	MG632595			MG633163	MG633472			MG634275			MG635525		MG636408
Ostomopsissp.	MG630793	MG631092	MG632040	MG632311	MG632657	MG629071		MG633212	MG633532			MG634338	MG634976	MG614847	MG635582	MG635915	MG636467
**Coccinellidae**																	
Chnootribasp.	MG630731	MG631012		MG632240	MG632578			MG633151	MG633457	MG633776				MG614780	MG635512	MG635842	MG636394
Harmoniaoctomaculata	MG630732	MG631013		MG632241		MG629017				MG633777						MG635843	MG636395
Exochomussp.	MG630733	MG631014		MG632242	MG632579				MG633458			MG634260					MG636396
Rhizobiussp.		MG631015		MG632243	MG632580	MG629018	MG632925	MG633152	MG633459	MG633778		MG634261		MG614781		MG635844	MG636397
Ortaliasp.	MG630734	MG631016	MG631980	MG632244	MG632581	MG629019	MG632926	MG633153	MG633460	MG633779		MG634262	MG634918	MG614782		MG635845	MG636398
Microfreudeasp.		MG631017		MG632245	MG632582			MG633154			MG634069	MG634263				MG635846	
Sasajiscymnussp.	MG630735	MG631018	MG631981	MG632246	MG632583	MG629020	MG632927	MG633155	MG633461	MG633780	MG634070	MG634264		MG614783	MG635513	MG635847	
Stethorussp.	MG630736	MG631019	MG631982	MG632247	MG632584	MG629021	MG632928	MG633156	MG633462	MG633781		MG634265	MG634919	MG614784	MG635514		MG636399
**Corylophidae**																	
Sericoderussp.	MG630745	MG631032	MG631994	MG632258	MG632598	MG629027	MG632934	MG633166	MG633475	MG633791	MG634076	MG634278	MG634930	MG614796	MG635528	MG635859	MG636411
Priamimasp.	MG630797	MG631097	MG632043	MG632316	MG632663	MG629076		MG633216	MG633537	MG633841		MG634343	MG634981	MG614852	MG635587	MG635920	MG636472
Periptyctussp.		MG631098		MG632317	MG632664			MG633217	MG633538			MG634344	MG634982	MG614853	MG635588		MG636473
Orthoperussp.	MG631006	MG631354	MG632232		MG632912	MG629258		MG633448	MG633763	MG634057	MG634249	MG634587	MG635187	MG615073	MG635828	MG636169	
**Discolomatidae**																	
Aphanocephalussp.	MG630748	MG631035	MG631997		MG632601	MG629029	MG632937	MG633169	MG633478	MG633794	MG634079	MG634281	MG634933	MG614799	MG635531	MG635862	MG636414
**Endomychidae**																	
Cyclotomasp.	MG630749	MG631037	MG631999	MG632262	MG632603	MG629030	MG632938	MG633170	MG633480	MG633796		MG634283	MG634935	MG614801	MG635533	MG635864	MG636416
Holoparamecussp.	MG630806	MG631108	MG632051	MG632327		MG629084	MG632984	MG633227	MG633547			MG634354			MG635598	MG635930	MG636482
Encymonimmaculatus	MG630807	MG631109	MG632052	MG632328	MG632674	MG629085		MG633228	MG633548	MG633849		MG634355	MG634992	MG614862	MG635599	MG635931	MG636483
Stenotarsussp.		MG631110	MG632053	MG632329	MG632675	MG629086				MG633850		MG634356	MG634993	MG614863	MG635600	MG635932	MG636484
Sinocymbachussp.	MG630868	MG631181	MG632104	MG632400	MG632748	MG629145		MG633291	MG633611	MG633909		MG634423	MG635054		MG635673	MG636002	MG636550
**Euxestidae**																	

Hypodacnellasp.	MG630743	MG631028			MG632594				MG633471	MG633788		MG634274	MG634927	MG614793	MG635524		
**Latridiidae**																	
Enicmussp.	MG630755	MG631045		MG632269		MG629036		MG633177	MG633488		MG634084	MG634291		MG614809		MG635871	MG636423
Corticariasp.				MG632270	MG632611	MG629037						MG634292	MG634941		MG635539	MG635872	
Melanophthalmasp.			MG632060	MG632342	MG632688			MG633239	MG633560			MG634368	MG635000		MG635613	MG635945	MG636495
**Murmidiidae**																	
Murmidiusovalis		MG631091		MG632310	MG632656			MG633211	MG633531	MG633836		MG634337	MG634975	MG614846	MG635581	MG635914	MG636466
**Teredidae**																	
Xylariophilussp.		MG631025			MG632591							MG634272			MG635521	MG635854	
Xylariophilussp.	MG630787	MG631085	MG632035	MG632304	MG632651				MG633526	MG633831		MG634331	MG634972	MG614843	MG635576	MG635909	MG636460
Teredolaemussp.	MG630788	MG631086	MG632036	MG632305	MG632652	MG629068		MG633208	MG633527	MG633832		MG634333		MG614844	MG635577	MG635910	MG636461
**Cucujoidea/Alexiidae**																	
Sphaerosomasp.	MG630738	MG631020	MG631984	MG632248	MG632586			MG633158	MG633464	MG633782		MG634267		MG614786	MG635516	MG635849	MG636401
**Boganiidae**																	
Paracucujusrostratus	MG630741	MG631024	MG631988	MG632252	MG632590	MG629025	MG632931	MG633162	MG633468	MG633786	MG634074	MG634271	MG634924	MG614790	MG635520	MG635853	MG636405
**Cryptophagidae**																	
Micrambinasp.	MG630746	MG631033	MG631995	MG632259	MG632599	MG629028	MG632935	MG633167	MG633476	MG633792	MG634077	MG634279	MG634931	MG614797	MG635529	MG635860	MG636412
Cureliussp.	MG630995	MG631340	MG632221	MG632553	MG632899	MG629248	MG633134	MG633434	MG633750	MG634045		MG634572	MG635174	MG615061	MG635814	MG636155	MG636694
**Cucujidae**																	
Platisussp.	MG630747	MG631034	MG631996	MG632260	MG632600		MG632936	MG633168	MG633477	MG633793	MG634078	MG634280	MG634932	MG614798	MG635530	MG635861	MG636413
Pediacusmajor	MG630798	MG631099		MG632318	MG632665		MG632977	MG633218	MG633539		MG634108	MG634345	MG634983	MG614854	MG635589	MG635921	MG636474
**Cybocephalidae**																	
Cybocephalussp.	MG630799	MG631100	MG632044	MG632319	MG632666	MG629077	MG632978	MG633219		MG633842	MG634109	MG634346	MG634984		MG635590	MG635922	MG636475
**Erotylidae**																	
Thallissp.	MG630808	MG631111	MG632054	MG632330	MG632676	MG629087			MG633549	MG633851		MG634357			MG635601	MG635933	MG636485
Anadastussp.	MG630809	MG631112		MG632331	MG632677			MG633229				MG634358		MG614864	MG635602	MG635934	MG636486
Episcaphulasp.	MG630810	MG631113		MG632332	MG632678			MG633230	MG633550	MG633852	MG634112	MG634359		MG614865	MG635603	MG635935	MG636487
Cryptophilussp.	MG630811	MG631114		MG632333	MG632679	MG629088	MG632985	MG633231	MG633551	MG633853	MG634113	MG634360	MG634994	MG614866	MG635604	MG635936	MG636488
Episcaphasp.	MG630882	MG631196		MG632416	MG632763			MG633305		MG633921			MG635065	MG614937		MG636016	MG636564
Tetraphalasp.	MG630886	MG631201		MG632421	MG632768		MG633049	MG633309	MG633628	MG633926	MG634168	MG634442	MG635070	MG614942	MG635691	MG636021	MG636569
**Helotidae**																	
Neohelotasp.	MG630867	MG631180	MG632103	MG632399	MG632747	MG629144	MG633037	MG633290	MG633610	MG633908		MG634422	MG635053	MG614924	MG635672	MG636001	MG636549
**Hobartiidae**																	
Hydnobioidessp.	MG630814	MG631118		MG632337	MG632683	MG629091	MG632988	MG633234	MG633555	MG633857		MG634364		MG614869	MG635608	MG635940	MG636491
**Kateretidae**																	
Notobrachypterussp.	MG630754	MG631044	MG632004	MG632268	MG632610	MG629035		MG633176	MG633487	MG633801		MG634290	MG634940	MG614808	MG635538	MG635870	MG636422
**Laemophloeidae**																	
Laemophloeussp.	MG630816	MG631122	MG632059	MG632341	MG632687	MG629095	MG632991	MG633238	MG633559	MG633860	MG634116	MG634367	MG634999	MG614872	MG635612	MG635944	MG636494
Cryptolestessp.	MG630996	MG631341	MG632222	MG632554	MG632900	MG629249	MG633135	MG633435	MG633751	MG634046		MG634573	MG635175		MG635815	MG636156	MG636695
**Monotomidae**																	
Mimemodessp.	MG630759	MG631050	MG632006	MG632274	MG632616	MG629040	MG632946	MG633181	MG633493	MG633804	MG634087	MG634296		MG614814	MG635544	MG635876	MG636428
Monotomopsissp.	MG630821	MG631129	MG632064	MG632348	MG632695	MG629099	MG632996	MG633246	MG633566	MG633866	MG634121	MG634374	MG635005	MG614877	MG635620	MG635952	MG636501
Rhizophagussp.	MG630822	MG631130	MG632065	MG632349	MG632696	MG629100	MG632997	MG633247	MG633567	MG633867	MG634122	MG634375	MG635006	MG614878	MG635621	MG635953	MG636502
Thionesp.	MG631004	MG631352	MG632230	MG632565	MG632910	MG629257	MG633142	MG633446	MG633761	MG634055	MG634247	MG634585	MG635185	MG615071	MG635826	MG636167	MG636705
**Myraboliidae**																	
Myraboliasp.	MG630762	MG631053	MG632008	MG632277	MG632619	MG629043	MG632948	MG633184	MG633496	MG633806	MG634089	MG634299		MG614817	MG635547	MG635879	MG636431
**Nitidulidae**																	
Brachypeplussp.	MG630824	MG631132	MG632067	MG632351	MG632698	MG629102	MG632999	MG633249	MG633569	MG633868	MG634124	MG634376	MG635008	MG614879	MG635623	MG635954	MG636504
Pallodessp.	MG630825	MG631133		MG632352	MG632699	MG629103	MG633000	MG633250	MG633570	MG633869		MG634377	MG635009	MG614880	MG635624	MG635955	MG636505

Urophorussp.	MG630908	MG631227	MG632135	MG632444	MG632793		MG633067	MG633335		MG633947	MG634184	MG634467	MG635091	MG614963		MG636046	MG636593
Carpophilussp.	MG630997	MG631343	MG632224	MG632556	MG632902	MG629250	MG633136	MG633437	MG633753	MG634048	MG634241	MG634575	MG635177	MG615063	MG635817	MG636158	MG636697
Glischrochilusparvipustulatus	MG630998	MG631344	MG632225	MG632557	MG632903	MG629251	MG633137	MG633438	MG633754	MG634049	MG634242	MG634576	MG635178	MG615064	MG635818	MG636159	MG636698
**Passandridae**																	
Passandrasp.	MG630828	MG631136	MG632069	MG632355	MG632702	MG629106		MG633252	MG633572	MG633870		MG634380		MG614883	MG635627	MG635958	MG636508
**Phalacridae**																	
gen.sp.	MG630765	MG631056	MG632010	MG632280	MG632622	MG629046	MG632951	MG633187	MG633499	MG633809	MG634092	MG634301		MG614820	MG635550	MG635881	MG636434
Phalacrinussp.	MG630829		MG632070	MG632356	MG632703	MG629107	MG633003	MG633253	MG633573	MG633871	MG634127	MG634381	MG635012	MG614884	MG635628	MG635959	MG636509
Olibrussp.	MG630951	MG631284	MG632183	MG632500	MG632847	MG629215	MG633100	MG633385	MG633705	MG633997		MG634518	MG635136	MG615011	MG635762	MG636104	MG636642
**Phloeostichidae**																	
Hymaeasp.	MG630830	MG631137	MG632071	MG632357	MG632704	MG629108	MG633004	MG633254	MG633574	MG633872	MG634128	MG634382	MG635013	MG614885	MG635629	MG635960	MG636510
Rhopalobrachiumcrowsoni	MG630831	MG631138	MG632072	MG632358	MG632705	MG629109		MG633255	MG633575	MG633873	MG634129	MG634383	MG635014	MG614886	MG635630	MG635961	MG636511
**Propalticidae**																	
Propalticussp.	MG630766	MG631058	MG632012	MG632281	MG632624	MG629048	MG632953	MG633189	MG633501	MG633811	MG634094	MG634303	MG634949	MG614822	MG635552	MG635883	MG636436
**Protocucujidae**																	
Ericmodescostatus	MG630832	MG631139	MG632073	MG632359	MG632706	MG629110	MG633005	MG633256	MG633576	MG633874			MG635015	MG614887	MG635631	MG635962	MG636512
**Silvanidae**																	
Uleiotasp.	MG630773	MG631067	MG632020			MG629055	MG632960		MG633509	MG633816		MG634312	MG634958	MG614828	MG635559	MG635893	MG636444
Cryptamorphasp.	MG630843	MG631153		MG632374	MG632721		MG633017				MG634140	MG634397	MG635028		MG635646	MG635977	MG636526
Silvanoprusscuticollis	MG630844	MG631154					MG633018			MG633886	MG634141	MG634398	MG635029	MG614900	MG635647	MG635978	
Psammoecussp.	MG630935	MG631262	MG632164	MG632475		MG629197	MG633085	MG633365	MG633681	MG633973	MG634196	MG634500	MG635113	MG614991	MG635742	MG636081	MG636627
Psammoecussp.	MG630952	MG631285	MG632184	MG632501	MG632848		MG633101	MG633386	MG633706	MG633998	MG634212	MG634519	MG635137	MG615012	MG635763		MG636643
**Sphindidae**																	
Aspidiphorussp.	MG630774	MG631068	MG632021	MG632289	MG632634	MG629056	MG632961	MG633196	MG633510	MG633817		MG634313	MG634959		MG635560	MG635894	MG636445
**Curculionoidea/Anthribidae**																	
Acorynussp.	MG630870	MG631183		MG632402	MG632750		MG633039	MG633293	MG633612			MG634425	MG635055			MG636003	MG636552
Ozotomerussp.	MG630897	MG631215			MG632780	MG629161	MG633057	MG633323	MG633640	MG633938		MG634455				MG636035	MG636581
Xylinadasp.	MG630906	MG631225		MG632442	MG632791		MG633065	MG633333	MG633649			MG634465	MG635089			MG636044	MG636591
Peribathyssp.	MG630940	MG631268		MG632482	MG632830	MG629204	MG633090		MG633688			MG634506	MG635120			MG636088	MG636633
**Attelabidae**																	
Phymatapoderussp.	MG630903	MG631222		MG632439	MG632788	MG629168		MG633330	MG633646		MG634182	MG634462					MG636588
Involvulussp.	MG630920	MG631242	MG632147	MG632458	MG632807	MG629183		MG633347	MG633664	MG633959		MG634480	MG635101			MG636060	MG636607
Byctiscussp.	MG630921	MG631244	MG632148	MG632460	MG632808	MG629184		MG633349	MG633665			MG634482	MG635102	MG614974		MG636062	MG636609
Paratrachelophorussp.	MG630994	MG631336		MG632549	MG632895		MG633133	MG633431	MG633746			MG634568			MG635810	MG636151	MG636691
**Belidae**																	
Rhinotiasp.	MG630784	MG631081	MG632031	MG632300	MG632647		MG632970	MG633204	MG633522			MG634327	MG634969		MG635572	MG635905	MG636456
**Brentidae**																	
Apions.l.sp.	MG630790	MG631088	MG632038	MG632307												MG635911	MG636463
Baryrhynchussp.		MG631216		MG632432	MG632781	MG629162	MG633058	MG633324	MG633641			MG634456	MG635081	MG614954		MG636036	MG636582
Apions.l.sp.		MG631243		MG632459				MG633348				MG634481			MG635725	MG636061	MG636608
Cylassp.												MG634577					
**Curculionidae**																	
Episomussp.	MG630872		MG632107	MG632404	MG632752			MG633295	MG633613			MG634427				MG636005	MG636554
gen.sp.	MG630898			MG632433	MG632782	MG629163				MG633939			MG635082		MG635703		
Xylosandrussp.	MG630901	MG631219	MG632131	MG632436	MG632785	MG629165	MG633061	MG633327	MG633644	MG633942	MG634180	MG634459	MG635085	MG614957		MG636039	MG636585
Curculiosp.	MG630992		MG632216	MG632547			MG633132	MG633429		MG634041	MG634239	MG634566	MG635170	MG615058	MG635809	MG636149	MG636689
Peribleptussp.	MG630993	MG631335	MG632217	MG632548	MG632894			MG633430				MG634567				MG636150	MG636690
**Nemonychidae**																	
AragomaceruniformisKuschel	MG630823	MG631131	MG632066	MG632350	MG632697	MG629101	MG632998	MG633248	MG633568		MG634123		MG635007		MG635622		MG636503

**Dasciloidea/ Dascillidae**																	
Dascillussp.	MG630885	MG631200	MG632118	MG632420	MG632767		MG633048	MG633308	MG633627	MG633925	MG634167	MG634441	MG635069	MG614941	MG635690	MG636020	MG636568
Metallidascillussp.	MG630999	MG631345	MG632226	MG632558	MG632904	MG629252	MG633138	MG633439	MG633755	MG634050		MG634578	MG635179	MG615065	MG635819	MG636160	MG636699
**Rhipiceridae**																	
Oligorhipisbifossata	MG630770	MG631064	MG632017	MG632286	MG632631	MG629052	MG632957	MG633193	MG633506	MG633814	MG634097	MG634309	MG634955		MG635556	MG635890	MG636441
**Derodontoidea /Derodontidae**																	
Derodontussp.	MG630802	MG631103	MG632046	MG632322	MG632669	MG629080		MG633222	MG633542	MG633845	MG634110	MG634349	MG634987	MG614857	MG635593	MG635925	MG636478
Derolathrussp.		MG631043	MG632003	MG632267	MG632609			MG633175	MG633486			MG634289		MG614807		MG635869	MG636421
**Jacobsoniidae**																	
Derolathrussp.		MG631121	MG632058	MG632340	MG632686	MG629094		MG633237	MG633558			MG634366		MG614871	MG635611	MG635943	MG636493
**Nosodendridae**																	
Nosodendronsp.	MG630763	MG631054	MG632009	MG632278	MG632620	MG629044	MG632949	MG633185	MG633497	MG633807	MG634090	MG634300	MG634946	MG614818	MG635548	MG635880	MG636432
**Elateroidea/Artematopodidae**																	
Artematopussp.	MG630740	MG631022	MG631986	MG632250	MG632588	MG629024	MG632930	MG633160	MG633466	MG633784	MG634073	MG634269	MG634922	MG614788	MG635518	MG635851	MG636403
Eurypogonsp.		MG631347	MG632228	MG632560	MG632906	MG629254	MG633140	MG633441	MG633757	MG634052	MG634243	MG634580	MG635181	MG615067	MG635821	MG636162	MG636701
**Cantharidae**																	
Heteromastixsp.	MG630792	MG631090		MG632309	MG632655		MG632973	MG633210	MG633530	MG633835		MG634336			MG635580	MG635913	MG636465
Themussp.	MG630865	MG631177	MG632100	MG632396	MG632744	MG629142	MG633035	MG633287	MG633607	MG633905			MG635051		MG635669	MG635998	MG636546
Themussp.	MG630912	MG631233	MG632139	MG632450	MG632799	MG629176	MG633070	MG633339	MG633655	MG633953	MG634188	MG634472	MG635093		MG635716	MG636051	MG636598
Prothemussp.	MG630913	MG631234	MG632140	MG632451	MG632800	MG629177	MG633071	MG633340	MG633656	MG633954		MG634473	MG635094		MG635717	MG636052	MG636599
Ichthyurusbourgeoisi		MG631235	MG632141	MG632452		MG629178	MG633072	MG633341	MG633657	MG633955	MG634189		MG635095		MG635718	MG636053	MG636600
Laemoglyptussp.	MG630975	MG631311	MG632198	MG632524	MG632872	MG629230	MG633116	MG633409	MG633723	MG634020	MG634226	MG634542	MG635154	MG615036	MG635787	MG636126	MG636667
Malthinussp.	MG630976	MG631312	MG632199	MG632525	MG632873	MG629231	MG633117	MG633410	MG633724	MG634021	MG634227	MG634543	MG635155	MG615037	MG635788	MG636127	MG636668
Lycocerussp.	MG630977	MG631313	MG632200	MG632526	MG632874	MG629232	MG633118	MG633411	MG633725	MG634022	MG634228	MG634544	MG635156		MG635789	MG636128	MG636669
Fissocantharissp.	MG630978	MG631314	MG632201	MG632527	MG632875	MG629233	MG633119	MG633412	MG633726	MG634023	MG634229	MG634545	MG635157	MG615038	MG635790	MG636129	MG636670
**Elateridae**																	
Osslimussp.	MG630803	MG631104	MG632047	MG632323	MG632670	MG629081	MG632981	MG633223	MG633543	MG633846		MG634350	MG634988	MG614858	MG635594	MG635926	MG636479
Hemicrepidiussp.	MG630876	MG631190		MG632410	MG632757	MG629148	MG633042	MG633301	MG633619	MG633916	MG634162	MG634433		MG614931	MG635681	MG636010	MG636558
Pectocerasp.	MG630980	MG631317		MG632529	MG632878	MG629236		MG633415	MG633729	MG634026		MG634548		MG615041	MG635793	MG636132	MG636673
Agrypnussp.	MG630981	MG631318		MG632530	MG632879			MG633416	MG633730	MG634027		MG634549		MG615042	MG635794	MG636133	MG636674
Ampedussp.	MG630982	MG631319	MG632204	MG632531	MG632880	MG629237	MG633121	MG633417	MG633731	MG634028	MG634232	MG634550		MG615043	MG635795	MG636134	MG636675
Denticollissp.	MG630983	MG631320	MG632205	MG632532	MG632881	MG629238	MG633122	MG633418	MG633732	MG634029	MG634233	MG634551		MG615044	MG635796	MG636135	MG636676
Peniasp.	MG630984	MG631321	MG632206	MG632533		MG629239		MG633419	MG633733	MG634030		MG634552	MG635158	MG615045	MG635797	MG636136	MG636677
gen.sp.		MG631322		MG632534	MG632882	MG629240	MG633123	MG633420	MG633734	MG634031		MG634553	MG635159	MG615046	MG635798	MG636137	MG636678
Denticollissp.		MG631323		MG632535	MG632883	MG629241			MG633735	MG634032		MG634554		MG615047	MG635799	MG636138	MG636679
Cardiotarsussp.		MG631355		MG632567	MG632913	MG629259			MG633764	MG634058		MG634588			MG635829	MG636170	MG636707
Melanotussp.	MG631007	MG631356	MG632233	MG632568	MG632914	MG629260	MG633144	MG633449	MG633765	MG634059	MG634250	MG634589			MG635830	MG636171	MG636708
Cebriosp.	MG631010	MG631360	MG632238	MG632572	MG632919	MG629265		MG633452	MG633769	MG634062	MG634255	MG634592		MG615077	MG635835	MG636176	MG636711
**Eucnemidae**																	
Anischiasp.	MG630751	MG631039	MG632000	MG632263	MG632605	MG629031	MG632940	MG633172	MG633482	MG633798	MG634081	MG634285	MG634937	MG614803	MG635535	MG635866	MG636417
Hemiopsidasp.	MG630812	MG631116	MG632056	MG632335	MG632681	MG629089			MG633553	MG633855	MG634114	MG634362	MG634996		MG635606	MG635938	MG636489
Othosp.	MG630891	MG631207	MG632122	MG632425	MG632773			MG633315	MG633632	MG633931	MG634171	MG634447	MG635075	MG614947	MG635696	MG636027	MG636574
Pyrocoeliasp.	MG630877	MG631191		MG632411	MG632758	MG629149			MG633620	MG633917	MG634163	MG634434		MG614932	MG635682	MG636011	MG636559
Vestasp.	MG630909	MG631229		MG632446	MG632795	MG629172				MG633949	MG634185	MG634468			MG635712	MG636047	
Pristolycussp.	MG630910	MG631230	MG632137	MG632447	MG632796	MG629173	MG633068	MG633337	MG633652	MG633950	MG634186	MG634469		MG614965	MG635713	MG636048	MG636595
Luciolasp.	MG630911	MG631231	MG632138	MG632448	MG632797	MG629174	MG633069	MG633338	MG633653	MG633951	MG634187	MG634470		MG614966	MG635714	MG636049	MG636596
Cyphonocerussp.		MG631232		MG632449	MG632798	MG629175			MG633654	MG633952		MG634471		MG614967	MG635715	MG636050	MG636597
Drilastersp.		MG631315	MG632202		MG632876	MG629234		MG633413	MG633727	MG634024	MG634230	MG634546		MG615039	MG635791	MG636130	MG636671
Diaphanessp.	MG630979	MG631316	MG632203	MG632528	MG632877	MG629235	MG633120	MG633414	MG633728	MG634025	MG634231	MG634547		MG615040	MG635792	MG636131	MG636672

Gorhamiasp.		MG631359	MG632237		MG632918	MG629264	MG633148		MG633768	MG634061	MG634254	MG634591		MG615076	MG635834	MG636175	MG636710
**Lycidae**																	
Porrostomasp.		MG631124		MG632344	MG632690			MG633241	MG633562		MG634118	MG634370			MG635615	MG635947	MG636497
Benibotarussp.		MG631361		MG632573	MG632920	MG629266			MG633770	MG634063	MG634256	MG634593		MG615078	MG635836	MG636177	MG636712
Dilophotessp.		MG631362		MG632574	MG632921	MG629267		MG633453	MG633771	MG634064		MG634594		MG615079	MG635837	MG636178	MG636713
Platerodrilussp.		MG631363		MG632575	MG632922				MG633772	MG634065	MG634257	MG634595		MG615080	MG635838	MG636179	MG636714
Libnetissp.	MG631011	MG631364		MG632576	MG632923		MG633149	MG633454	MG633773	MG634066	MG634258	MG634596	MG635191	MG615081	MG635839	MG636180	MG636715
Macrolycussichuanensis		MG631365	MG632239	MG632577	MG632924	MG629268	MG633150	MG633455	MG633774	MG634067	MG634259	MG634597		MG615082	MG635840	MG636181	
Lycostomussp.		MG631366						MG633456	MG633775	MG634068		MG634598			MG635841	MG636182	MG636716
**Omethidae**																	
Driloniussp.	MG630954	MG631287	MG632185	MG632502	MG632850	MG629216		MG633388	MG633708	MG634000			MG635139	MG615014	MG635765	MG636106	MG636645
Driloniussp.	MG630955	MG631289	MG632186	MG632503	MG632852			MG633389	MG633710	MG634002			MG635141	MG615016	MG635767	MG636108	MG636647
**Phengodidae**																	
Stenophrixothrixsp.		MG631057	MG632011		MG632623	MG629047	MG632952	MG633188	MG633500	MG633810	MG634093	MG634302	MG634948	MG614821	MG635551	MG635882	MG636435
**Rhagophthalmidae**																	
Rhagophthalmussp.		MG631063	MG632016		MG632630	MG629051		MG633192	MG633505	MG633813		MG634308	MG634954	MG614826	MG635555	MG635889	MG636440
Rhagophthalmussp.		MG631142		MG632362	MG632709	MG629113		MG633258	MG633579	MG633877		MG634385		MG614890	MG635634	MG635965	MG636515
gen.sp.		MG631288			MG632851	MG629217			MG633709	MG634001	MG634213	MG634521	MG635140	MG615015	MG635766	MG636107	MG636646
**Throscidae**																	
Trixagussp.	MG630776	MG631071			MG632637		MG632964	MG633198	MG633513			MG634316	MG634962	MG614830	MG635563		
Trixagussp.	MG630953	MG631286			MG632849		MG633102	MG633387	MG633707	MG633999		MG634520	MG635138	MG615013	MG635764	MG636105	MG636644
**Histeridae**																	
Platysomasp.	MG630752	MG631040	MG632001	MG632264	MG632606	MG629032	MG632941	MG633173	MG633483	MG633799		MG634286	MG634938	MG614804	MG635536	MG635867	MG636418
Saprinussp.	MG630753	MG631041	MG632002	MG632265	MG632607	MG629033	MG632942	MG633174	MG633484	MG633800	MG634082	MG634287	MG634939	MG614805		MG635868	MG636419
**Hydrophiloidea/ Hydrophilidae**																	
Sternolophusimmarginatus		MG631119		MG632338	MG632684	MG629092	MG632989	MG633235	MG633556	MG633858				MG614870	MG635609	MG635941	MG636492
Helocharessp.	MG630815	MG631120		MG632339	MG632685	MG629093	MG632990	MG633236	MG633557	MG633859		MG634365	MG634998		MG635610	MG635942	
Oocyclussp.		MG631188		MG632408	MG632755			MG633299	MG633617	MG633914		MG634431		MG614929	MG635679		
Hydrophilusacuminatus				MG632483	MG632831		MG633091		MG633689	MG633980	MG634202		MG635121	MG614998	MG635748	MG636089	MG636634
Anacaenapui		MG631270	MG632170	MG632485	MG632833	MG629206	MG633092	MG633371		MG633982	MG634203	MG634508	MG635123		MG635750	MG636091	MG636636
Georissussp.		MG631271	MG632171	MG632486	MG632834			MG633372	MG633691	MG633983	MG634204	MG634509	MG635124	MG614999		MG636092	MG636637
Sphaeridiumquinquemaculatum	MG630942	MG631272	MG632172	MG632487	MG632835	MG629207	MG633093	MG633373	MG633692	MG633984		MG634510	MG635125		MG635751	MG636093	
Berosusspinosus																MG636125	
**Chrysomeloidea/ Cerambycidae**																	
Strangaliafortunei	MG630914	MG631236	MG632142	MG632453	MG632801	MG629179		MG633342	MG633658			MG634474	MG635096	MG614968	MG635719	MG636054	MG636601
Obereasp.	MG630915	MG631237	MG632143	MG632454	MG632802			MG633343	MG633659	MG633956		MG634475	MG635097	MG614969	MG635720	MG636055	MG636602
Spondylisbuprestoides	MG630916	MG631238	MG632144	MG632455	MG632803	MG629180		MG633344	MG633660		MG634190	MG634476		MG614970	MG635721	MG636056	MG636603
Xylotrechussp.	MG630917	MG631239	MG632145	MG632456	MG632804		MG633073	MG633345	MG633661			MG634477	MG635098	MG614971	MG635722	MG636057	MG636604
Dorystheneshydropicus		MG631309						MG633408				MG634540				MG636123	
**Chrysomelidae**																	
Cassidasp.	MG630869	MG631182	MG632105	MG632401	MG632749		MG633038	MG633292		MG633910		MG634424			MG635674		MG636551
Chrysomelapopuli	MG630879	MG631193	MG632112	MG632413	MG632760		MG633044						MG635062	MG614934	MG635684	MG636013	MG636561
Chlamisussp.	MG630922	MG631245		MG632461		MG629185	MG633075		MG633666	MG633960		MG634483	MG635103	MG614975	MG635726	MG636063	MG636610
Sagrafulgidajanthina	MG630960	MG631294	MG632190	MG632508	MG632857	MG629222	MG633106	MG633393		MG634006		MG634526	MG635145	MG615021	MG635771	MG636113	MG636652
Liliocerissp.	MG630961	MG631295		MG632509	MG632858	MG629223		MG633394		MG634007	MG634217	MG634527		MG615022	MG635772		MG636653
Dactylispasp.	MG630962	MG631296	MG632191	MG632510	MG632859	MG629224		MG633395				MG634528		MG615023	MG635773		MG636654
Anisoderasp.	MG630963	MG631297	MG632192	MG632511	MG632860	MG629225	MG633107			MG634008		MG634529		MG615024	MG635774		MG636655
Sominellasp.	MG630964	MG631298		MG632512	MG632861			MG633396	MG633714	MG634009		MG634530		MG615025	MG635775		MG636656
Trichochryseasp.	MG630965	MG631299		MG632513	MG632862		MG633108	MG633397			MG634218	MG634531	MG635146	MG615026	MG635776	MG636114	

gen.sp.	MG630966	MG631300	MG632193	MG632514	MG632863		MG633109	MG633398	MG633715	MG634010		MG634532		MG615027	MG635777	MG636115	MG636657
Alticasp.	MG630967	MG631301		MG632515	MG632864			MG633399		MG634011				MG615028	MG635778		MG636658
Oomorphoidessp.	MG630968	MG631302	MG632194	MG632516	MG632865	MG629226	MG633110	MG633400	MG633716	MG634012		MG634533	MG635147	MG615029	MG635779	MG636116	MG636659
Cryptocephalussp.	MG630969			MG632517			MG633111	MG633401	MG633717			MG634534	MG635148	MG615030	MG635780	MG636117	MG636660
Bruchidiussp.		MG631303					MG633112	MG633402	MG633718	MG634013	MG634219	MG634535		MG615031			MG636661
**Megalopodidae**																	
Temnaspissp.	MG630888	MG631203	MG632119	MG632422	MG632770	MG629156	MG633050	MG633311	MG633629	MG633928	MG634169	MG634444	MG635071	MG614944	MG635693	MG636023	MG636571
**Lymexyloidea/Lymexylidae**																	
Melittommasp.	MG630758	MG631049			MG632615	MG629039	MG632945	MG633180	MG633492	MG633803				MG614813	MG635543	MG635875	MG636427
Atractocerussp.					MG632824										MG635741	MG636080	
**Rhinorhipoidea/Rhinorhipidae**																	
Rhinorhipus tamborensis	AB123503	AB123504	AB123505	AB123506	AB123507	AB123508	AB123509	AB123510	AB123511	AB123512	AB123513	AB123514	AB123515	AB123516	AB123517	AB123518	AB123519
**Scarabaeoidea/Geotrupidae**																	
Australobolbussp.		MG631146	MG632078	MG632367	MG632714		MG633011		MG633583	MG633880	MG634135	MG634390	MG635021	MG614893	MG635639	MG635970	MG636520
Geotrupessp.		MG631263	MG632166	MG632477	MG632826	MG629199	MG633086	MG633367	MG633683	MG633975	MG634198	MG634502	MG635115	MG614993	MG635744	MG636083	MG636629
**Glaphyridae**																	
Amphicomasp.		MG631185	MG632108	MG632405				MG633296	MG633614	MG633911	MG634159	MG634428	MG635057	MG614926	MG635676	MG636006	MG636555
**Hybosoridae**																	
Liparochrussp.		MG631042		MG632266	MG632608	MG629034			MG633485		MG634083	MG634288		MG614806	MG635537		MG636420
Cyphopisthessp.		MG631147	MG632079	MG632368	MG632715			MG633262	MG633584			MG634391	MG635022	MG614894	MG635640	MG635971	
**Lucanidae**																	
Phalacrognathusmuelleri		MG631264		MG632478	MG632827	MG629200	MG633087	MG633368	MG633684	MG633976		MG634503	MG635116	MG614994		MG636084	MG636630
Cyclommatusmetallifer	MG630937	MG631265	MG632167	MG632479		MG629201	MG633088		MG633685	MG633977	MG634199	MG634504	MG635117	MG614995	MG635745	MG636085	MG636631
Aegussp.	MG630972	MG631307	MG632196	MG632521	MG632869	MG629228		MG633406	MG633721	MG634017	MG634223		MG635151	MG615033	MG635784	MG636121	MG636664
**Passalidae**																	
Ceracupessp.		MG631228	MG632136	MG632445	MG632794			MG633336	MG633651	MG633948			MG635092	MG614964	MG635711		MG636594
Aceraiussp.	MG630936		MG632165	MG632476	MG632825	MG629198		MG633366	MG633682	MG633974	MG634197	MG634501	MG635114	MG614992	MG635743	MG636082	MG636628
**Scarabaeidae**																	
Carneodonsp.	MG630838	MG631148		MG632369	MG632716	MG629118	MG633012	MG633263	MG633585	MG633881	MG634136	MG634392	MG635023	MG614895	MG635641	MG635972	MG636521
Heteronyxsp.	MG630839	MG631149		MG632370	MG632717	MG629119	MG633013	MG633264	MG633586	MG633882	MG634137	MG634393	MG635024	MG614896	MG635642	MG635973	MG636522
Anomalaantiqua	MG630840	MG631150		MG632371	MG632718	MG629120	MG633014	MG633265	MG633587	MG633883	MG634138	MG634394	MG635025	MG614897	MG635643	MG635974	MG636523
Onthophagusgazella	MG630841	MG631151	MG632080	MG632372	MG632719		MG633015	MG633266	MG633588	MG633884		MG634395	MG635026	MG614898	MG635644	MG635975	MG636524
Rhyparussp.	MG630866	MG631178	MG632101	MG632397	MG632745	MG629143	MG633036	MG633288	MG633608	MG633906	MG634157			MG614922	MG635670	MG635999	MG636547
Protaetiaaerata	MG630941	MG631269		MG632484	MG632832	MG629205			MG633690	MG633981		MG634507	MG635122		MG635749	MG636090	MG636635
Mimelasp.	MG630970	MG631304		MG632518	MG632866	MG629227	MG633113	MG633403	MG633719	MG634014	MG634220	MG634536	MG635149		MG635781	MG636118	MG636662
Coprissp.		MG631305	MG632195	MG632519	MG632867		MG633114	MG633404	MG633720	MG634015	MG634221	MG634537	MG635150	MG615032	MG635782	MG636119	MG636663
Ectinohopliasp.	MG630971	MG631306		MG632520	MG632868			MG633405		MG634016	MG634222	MG634538			MG635783	MG636120	
Dasyvalgussp.	MG630973	MG631308	MG632197	MG632522	MG632870			MG633407		MG634018	MG634224	MG634539	MG635152	MG615034	MG635785	MG636122	MG636665
Cheirotonusjansoni	MG630974	MG631310		MG632523	MG632871	MG629229	MG633115		MG633722	MG634019	MG634225	MG634541	MG635153	MG615035	MG635786	MG636124	MG636666
Allomyrinadichotoma	MG630987	MG631327		MG632539		MG629243	MG633126	MG633423	MG633739	MG634036	MG634234	MG634558	MG635163	MG615051	MG635802	MG636142	MG636682
**Trogidae**																	
Omorgussp.	MG630777	MG631072	MG632023	MG632291		MG629058	MG632965	MG633199	MG633514	MG633820	MG634100	MG634317	MG634963	MG614831	MG635564	MG635897	MG636448
Troxsp.		MG631208	MG632123	MG632426				MG633316	MG633633	MG633932	MG634172	MG634448	MG635076	MG614948	MG635697	MG636028	MG636575
**Scirtoidea/Clambidae**																	
Clambussp.	MG630796	MG631095	MG632041	MG632314	MG632660	MG629074	MG632976	MG633215	MG633535	MG633839			MG634979	MG614850	MG635585	MG635918	MG636470
**Eucinetidae**																	
Noteucinetussp.	MG630750	MG631038			MG632604		MG632939	MG633171	MG633481	MG633797	MG634080	MG634284	MG634936	MG614802	MG635534	MG635865	
Noteucinetussp.		MG631115	MG632055	MG632334	MG632680		MG632986	MG633232	MG633552	MG633854		MG634361	MG634995	MG614867	MG635605	MG635937	
**Scirtidae**																	

Pseudomicrocarasp.	MG630842	MG631152	MG632081	MG632373	MG632720	MG629121	MG633016	MG633267	MG633589	MG633885	MG634139	MG634396	MG635027	MG614899	MG635645	MG635976	MG636525
gen.sp.		MG631213	MG632128	MG632430	MG632778	MG629159	MG633055	MG633321	MG633638	MG633937	MG634176	MG634453	MG635079	MG614952	MG635701	MG636033	MG636579
Elodessp.	MG630959	MG631293	MG632189	MG632507	MG632856	MG629221	MG633105	MG633392	MG633713	MG634005	MG634216	MG634525	MG635144	MG615020	MG635770	MG636112	MG636651
**Staphylinoidea/Agyrtidae**																	
Pterolomaforsstromii	MG631000	MG631346	MG632227	MG632559	MG632905	MG629253	MG633139	MG633440	MG633756	MG634051		MG634579	MG635180	MG615066	MG635820	MG636161	MG636700
**Hydraenidae**																	
Hydraenasp.	MG630943	MG631273	MG632173	MG632488	MG632836	MG629208	MG633094	MG633374	MG633693	MG633985		MG634511	MG635126	MG615000	MG635752	MG636094	
**Leiodidae**																	
Agyrtodessp.	MG630756	MG631046	MG632005	MG632271	MG632612	MG629038		MG633178	MG633489	MG633802	MG634085	MG634293	MG634942	MG614810	MG635540	MG635873	MG636424
gen.sp.		MG631047		MG632272	MG632613		MG632943		MG633490			MG634294		MG614811	MG635541		MG636425
Agathidiumsp.	MG630757	MG631048		MG632273	MG632614		MG632944	MG633179	MG633491		MG634086	MG634295	MG634943	MG614812	MG635542	MG635874	MG636426
**Ptiliidae**																	
gen.sp.				MG632282	MG632626								MG634950			MG635885	
**Silphidae**																	
Nicrophorusnepalensis	MG630881	MG631195	MG632114	MG632415	MG632762	MG629152	MG633046	MG633304	MG633623	MG633920	MG634165	MG634437	MG635064	MG614936	MG635686	MG636015	MG636563
Necrodeslittoralis	MG630923	MG631246	MG632149	MG632462	MG632809	MG629186	MG633076	MG633350	MG633667	MG633961		MG634484	MG635104	MG614976	MG635727	MG636064	MG636611
**Staphylinidae**																	
Scaphidiumsp.	MG630775	MG631069			MG632635		MG632962	MG633197	MG633511	MG633818		MG634314	MG634960		MG635561	MG635895	MG636446
gen.sp.	MG630845	MG631155		MG632375	MG632722	MG629122	MG633019	MG633268			MG634142	MG634399	MG635030	MG614901	MG635648	MG635979	MG636527
Staphylinussp.	MG630871	MG631184	MG632106	MG632403	MG632751	MG629146	MG633040	MG633294				MG634426	MG635056	MG614925	MG635675	MG636004	MG636553
Megalopaederussp.	MG630887	MG631202			MG632769	MG629155		MG633310		MG633927		MG634443		MG614943	MG635692	MG636022	MG636570
Apateticasp.	MG630928	MG631253	MG632156	MG632469	MG632816			MG633357	MG633673			MG634491	MG635110	MG614983	MG635733	MG636071	MG636618
Priochirussp.	MG630929	MG631254	MG632157	MG632470	MG632817		MG633080	MG633358	MG633674	MG633968	MG634194	MG634492		MG614984	MG635734	MG636072	MG636619
Osoriussp.	MG630930	MG631255	MG632158		MG632818	MG629193	MG633081	MG633359	MG633675	MG633969		MG634493		MG614985	MG635735	MG636073	MG636620
Tachinussp.	MG630931	MG631256	MG632159		MG632819			MG633360	MG633676			MG634494		MG614986	MG635736	MG636074	MG636621
Scaphidiumsp.	MG630932	MG631257	MG632160		MG632820		MG633082	MG633361	MG633677	MG633970		MG634495		MG614987	MG635737	MG636075	MG636622
Tachinussp.		MG631258	MG632161	MG632471	MG632821				MG633678			MG634496		MG614988	MG635738	MG636076	MG636623
Centrophthalmussp.		MG631259		MG632472		MG629194		MG633362				MG634497				MG636077	MG636624
gen.sp.		MG631330		MG632542	MG632889							MG634561					MG636684
Dianoussp.	MG630989	MG631331	MG632212	MG632543	MG632890	MG629244	MG633128	MG633426	MG633742			MG634562	MG635166	MG615054	MG635805	MG636145	MG636685
gen.sp.		MG631332	MG632213	MG632544	MG632891		MG633129	MG633427	MG633743			MG634563	MG635167	MG615055	MG635806	MG636146	MG636686
**Tenebrionoidea/Aderidae**																	
gen.sp.	MG630737		MG631983		MG632585	MG629022		MG633157	MG633463		MG634071	MG634266	MG634920	MG614785	MG635515	MG635848	MG636400
gen.sp.	MG631003	MG631351		MG632564	MG632909	MG629256		MG633445	MG633760		MG634246	MG634584		MG615070	MG635825	MG636166	MG636704
**Anthicidae**																	
Lemodessp.	MG630739	MG631021	MG631985	MG632249	MG632587	MG629023	MG632929	MG633159	MG633465	MG633783	MG634072	MG634268	MG634921	MG614787	MG635517	MG635850	MG636402
Anthicuss.l.sp.	MG630780	MG631077	MG632027	MG632296	MG632643	MG629062	MG632967			MG633824		MG634323		MG614836	MG635568	MG635901	MG636452
Trichanancasp.	MG630781	MG631078	MG632028	MG632297	MG632644	MG629063	MG632968	MG633201	MG633519	MG633825	MG634102	MG634324	MG634967	MG614837	MG635569	MG635902	MG636453
Macratriasp.	MG630782	MG631079	MG632029	MG632298	MG632645	MG629064		MG633202	MG633520	MG633826		MG634325		MG614838	MG635570	MG635903	MG636454
Macratriasp.	MG630902	MG631221	MG632132	MG632438	MG632787	MG629167	MG633063	MG633329	MG633645	MG633943	MG634181	MG634461		MG614959	MG635707	MG636041	MG636587
**Archeocrypticidae**																	
Wattianusqueenslandicus	MG630783	MG631080	MG632030	MG632299	MG632646	MG629065	MG632969	MG633203	MG633521	MG633827	MG634103	MG634326	MG634968	MG614839	MG635571	MG635904	MG636455
**Boridae**																	
Synercticussp.	MG630785	MG631083	MG632033	MG632302	MG632649	MG629066		MG633206	MG633524	MG633829	MG634105	MG634329	MG634971	MG614841	MG635574	MG635907	MG636458
**Ciidae**																	
Cissp.	MG630744	MG631031	MG631993	MG632257	MG632597		MG632933	MG633165	MG633474	MG633790	MG634075	MG634277	MG634929	MG614795	MG635527	MG635858	MG636410
Cissp.	MG630794	MG631093		MG632312	MG632658	MG629072	MG632974	MG633213	MG633533	MG633837		MG634339	MG634977	MG614848	MG635583	MG635916	MG636468
Australocissp.	MG630795	MG631094		MG632313	MG632659	MG629073	MG632975	MG633214	MG633534	MG633838	MG634107	MG634340	MG634978	MG614849	MG635584	MG635917	MG636469
**Tenebrionoidea/IncertaeSedis**																	

Rhizoniumantiquum	MG630813	MG631117	MG632057	MG632336	MG632682	MG629090	MG632987	MG633233	MG633554	MG633856	MG634115	MG634363	MG634997	MG614868	MG635607	MG635939	MG636490
**Ischaliidae**																	
Ischaliasp.		MG631220		MG632437	MG632786	MG629166	MG633062	MG633328				MG634460	MG635086	MG614958	MG635706	MG636040	MG636586
**Melandryidae**																	
Dircaeomorphasp.	MG630907	MG631226	MG632134	MG632443	MG632792	MG629171	MG633066	MG633334	MG633650	MG633946	MG634183	MG634466	MG635090	MG614962	MG635710	MG636045	MG636592
gen.sp.	MG631001	MG631348	MG632229	MG632561	MG632907	MG629255	MG633141	MG633442	MG633758	MG634053	MG634244	MG634581	MG635182	MG615068	MG635822	MG636163	MG636702
**Meloidae**																	
Zonitiss.l.sp.	MG630818	MG631125	MG632062		MG632691	MG629097	MG632993	MG633242	MG633563	MG633862	MG634119	MG634371	MG635002		MG635616	MG635948	
Epicautasp.	MG630934	MG631261	MG632163	MG632474	MG632823	MG629196	MG633084	MG633364	MG633680	MG633972	MG634195	MG634499	MG635112	MG614990	MG635740	MG636079	MG636626
**Mordellidae**																	
Hoshihananomiasp.	MG630890	MG631206				MG629158	MG633053	MG633314					MG635074		MG635695	MG636026	
**Mycetophagidae**																	
Nototriphyllussp.	MG630760	MG631051	MG632007	MG632275	MG632617	MG629041	MG632947	MG633182	MG633494	MG633805	MG634088	MG634297	MG634944	MG614815	MG635545	MG635877	MG636429
Mycetophagussp.	MG630900	MG631218	MG632130	MG632435	MG632784	MG629164	MG633060	MG633326	MG633643	MG633941	MG634179	MG634458	MG635084	MG614956	MG635705	MG636038	MG636584
**Mycteridae**																	
Trichosalpingussp.	MG630761	MG631052		MG632276	MG632618	MG629042		MG633183	MG633495			MG634298	MG634945	MG614816	MG635546	MG635878	MG636430
**Oedemeridae**																	
Thelyphassasp.	MG630826	MG631134		MG632353	MG632700	MG629104	MG633001				MG634125	MG634378	MG635010	MG614881	MG635625	MG635956	MG636506
Pseudolycussp.	MG630827	MG631135	MG632068	MG632354	MG632701	MG629105	MG633002	MG633251	MG633571		MG634126	MG634379	MG635011	MG614882	MG635626	MG635957	MG636507
Ditylussp.	MG630889	MG631205	MG632121	MG632424	MG632772	MG629157	MG633052	MG633313	MG633631	MG633930	MG634170	MG634446	MG635073	MG614946	MG635694	MG636025	MG636573
Pyrochroidae																	
Morpholycussp.	MG630769	MG631062	MG632015	MG632285	MG632629	MG629050	MG632956	MG633191	MG633504	MG633812	MG634096	MG634307	MG634953	MG614825	MG635554	MG635888	MG636439
MorpholycusmonilicornisLea	MG630833	MG631140	MG632074	MG632360	MG632707	MG629111	MG633006	MG633257	MG633577	MG633875	MG634130	MG634384	MG635016	MG614888	MG635632	MG635963	MG636513
Pseudopyrochroasp.	MG630880	MG631194	MG632113	MG632414	MG632761	MG629151	MG633045	MG633303	MG633622	MG633919	MG634164	MG634436	MG635063	MG614935	MG635685	MG636014	MG636562
Eupyrochroainsignita	MG630884	MG631199	MG632117	MG632419	MG632766	MG629154	MG633047	MG633307	MG633626	MG633924	MG634166	MG634440	MG635068	MG614940	MG635689	MG636019	MG636567
**Pythidae**																	
Anaplopussp.	MG630834	MG631141	MG632075	MG632361	MG632708	MG629112			MG633578	MG633876	MG634131		MG635017	MG614889	MG635633	MG635964	MG636514
**Ripiphoridae**																	
Trigonoderasp.	MG630771	MG631065	MG632018	MG632287	MG632632	MG629053	MG632958	MG633194	MG633507	MG633815	MG634098	MG634310	MG634956	MG614827	MG635557	MG635891	MG636442
Rhipidioidessp.				MG632363	MG632710	MG629114	MG633007					MG634386			MG635635	MG635966	MG636516
**Salpingidae**																	
OrphanotrophiumpallidipennisLea	MG630772	MG631066	MG632019	MG632288	MG632633	MG629054	MG632959	MG633195	MG633508		MG634099	MG634311	MG634957		MG635558	MG635892	MG636443
Euryplatusdimidiatus	MG630835	MG631143	MG632076	MG632364	MG632711	MG629115	MG633008	MG633259	MG633580	MG633878	MG634132	MG634387	MG635018	MG614891	MG635636	MG635967	MG636517
Orphanotrophiumsp.	MG630836	MG631144	MG632077	MG632365	MG632712	MG629116	MG633009	MG633260	MG633581		MG634133	MG634388	MG635019		MG635637	MG635968	MG636518
Ocholissasp.	MG630837	MG631145		MG632366	MG632713	MG629117	MG633010	MG633261	MG633582	MG633879	MG634134	MG634389	MG635020	MG614892	MG635638	MG635969	MG636519
**Scraptiidae**																	
Scraptiasp.		MG631350		MG632563				MG633444		MG634054		MG634583	MG635184		MG635824	MG636165	
**Tenebrionidae**																	
Amarygmussp.	MG630846	MG631156	MG632082	MG632376	MG632723	MG629123	MG633020	MG633269		MG633887	MG634143	MG634400	MG635031	MG614902	MG635649	MG635980	MG636528
Cyphaleussp.	MG630847	MG631157		MG632377	MG632724	MG629124	MG633021		MG633590	MG633888	MG634144	MG634401	MG635032	MG614903	MG635650	MG635981	MG636529
Adeliumsp.	MG630848	MG631158	MG632083	MG632378	MG632725	MG629125	MG633022	MG633270	MG633591		MG634145	MG634402	MG635033	MG614904	MG635651	MG635982	MG636530
Cillibussp.	MG630849	MG631159	MG632084	MG632379	MG632726	MG629126		MG633271	MG633592	MG633889	MG634146	MG634403	MG635034	MG614905	MG635652	MG635983	MG636531
Palorussp.	MG630850	MG631160		MG632380	MG632727	MG629127	MG633023	MG633272	MG633593	MG633890	MG634147	MG634404	MG635035	MG614906	MG635653	MG635984	MG636532
Derispiasp.	MG630851	MG631161	MG632085	MG632381	MG632728	MG629128		MG633273	MG633594	MG633891		MG634405	MG635036	MG614907	MG635654	MG635985	MG636533
Cossyphussp.	MG630852	MG631162	MG632086	MG632382	MG632729	MG629129	MG633024	MG633274	MG633595	MG633892	MG634148	MG634406	MG635037	MG614908	MG635655	MG635986	MG636534
Tanychilussp.	MG630853	MG631163	MG632087	MG632383	MG632730	MG629130		MG633275	MG633596	MG633893		MG634407	MG635038		MG635656	MG635987	MG636535
Platydemasp.	MG630854	MG631164	MG632088	MG632384	MG632731	MG629131	MG633025	MG633276	MG633597		MG634149	MG634408	MG635039	MG614909	MG635657	MG635988	MG636536
Tyrtaeussp.	MG630855	MG631165		MG632385	MG632732	MG629132		MG633277	MG633598	MG633894	MG634150	MG634409	MG635040	MG614910	MG635658	MG635989	MG636537
Ecnolagriasp.	MG630856	MG631166	MG632089	MG632386	MG632733	MG629133	MG633026	MG633278	MG633599	MG633895	MG634151	MG634410	MG635041	MG614911	MG635659	MG635990	MG636538

Chlorophilasp.	MG630899	MG631217		MG632434	MG632783		MG633059	MG633325	MG633642	MG633940	MG634178	MG634457	MG635083	MG614955	MG635704	MG636037	MG636583
Derispiasp.	MG630918	MG631240	MG632146		MG632805	MG629181			MG633662	MG633957		MG634478	MG635099	MG614972	MG635723	MG636058	MG636605
Cteniopinussp.	MG630919	MG631241		MG632457	MG632806	MG629182	MG633074	MG633346	MG633663	MG633958	MG634191	MG634479	MG635100	MG614973	MG635724	MG636059	MG636606
Strongyliumsp.	MG630990	MG631333	MG632214	MG632545	MG632892		MG633130	MG633428	MG633744	MG634039	MG634237	MG634564	MG635168	MG615056	MG635807	MG636147	MG636687
Cryphaeussp.	MG630991	MG631334	MG632215	MG632546	MG632893	MG629245	MG633131		MG633745	MG634040	MG634238	MG634565	MG635169	MG615057	MG635808	MG636148	MG636688
Trictenotomasp.	MG631005	MG631353	MG632231	MG632566	MG632911		MG633143	MG633447	MG633762	MG634056	MG634248	MG634586	MG635186	MG615072	MG635827	MG636168	MG636706
**Ulodidae**																	
Ulodessp.	MG630858	MG631170	MG632093	MG632390	MG632737	MG629135	MG633028	MG633281	MG633603	MG633899	MG634152	MG634414	MG635044	MG614915	MG635663	MG635993	MG636542
Meryxsp.	MG630859	MG631171	MG632094	MG632391	MG632738	MG629136	MG633029	MG633282	MG633604	MG633900	MG634153	MG634415	MG635045	MG614916	MG635664	MG635994	MG636543
**Zopheridae**																	
Monommasp.					MG632640							MG634320					
Bitomasp.	MG630778	MG631075	MG632025	MG632294	MG632641	MG629060	MG632966	MG633200	MG633517	MG633822	MG634101	MG634321	MG634965	MG614834	MG635566	MG635899	MG636450
Zopherosisgeorgei	MG630860	MG631172	MG632095	MG632392	MG632739	MG629137	MG633030	MG633283		MG633901		MG634416	MG635046	MG614917	MG635665		MG636544

Supplementary Table S5. The list of markers in the 95-gene dataset with information on multi-copy genes.

Zhang et. al 2018 Genes SQ	*Rhinorhipus* Genes	Otrhology/Paralogy	Occurence in OrthoDB9.1 Species (# of gene copies)
Aats-ile	-	multi-copy	*Onthophagus taurus* (2)
Ace	Ace	single-copy	
alphaCOP	alphaCOP	single-copy	
alpha-Spec	alpha-Spec	single-copy	
AP47	AP47	single-copy	
Art1	Art1	single-copy	
beta'Cop	beta'Cop	single-copy	
BOP1	BOP1	single-copy	
brat	-	multi-copy	*Dendroctonus ponderosae* (2)
Brel	-	single-copy	Missing in *Agrilus planipennis*
Bx42	Bx42	single-copy	
CAD	CAD	single-copy	
CadN	CadN	single-copy	
calypso	calypso	single-copy	
CaP60A	CaP60A	single-copy	
Cdk7	Cdk7	single-copy	
CG11652	CG11652	single-copy	
CG3999	CG3999	single-copy	
CG4933	CG4933	single-copy	
CG6230	-	multi-copy	*Onthophagus taurus* (2)
CG6512	CG6512	single-copy	
CG7288	CG7288	single-copy	
CG7433	-	multi-copy	*Leptinotarsa decemlineata* (4), *Agrilus planipennis* (3), *Anoplophora glabripennis* (3) *Onthophagus taurus* (3), *Dendroctonus ponderosae* (3), *Tribolium castaneum* (3)
CG7470	-	multi-copy	*Agrilus planipennis*, *Anoplophora glabripennis*, *Leptinotarsa decemlineata* (
CG8545	CG8545	single-copy	
CG9518	-	multi-copy	*Agrilus planipennis* (25), *Anoplophora glabripennis* (14), *Leptinotarsa decemlineata* (20), *Onthophagus taurus* (10), *Dendroctonus ponderosae* (16), *Tribolium castaneum* (21)
ck	-	multi-copy	*Agrilus planipennis* (2), *Onthophagus taurus* (2), *Leptinotarsa decemlineata* missing
Crc	Crc	single-copy	
dbo	dbo	single-copy	
Dhc98D	Dhc98D	single-copy	
DIP2	-	multi-copy	*Agrilus planipennis* (2), *Onthophagus taurus* (2), *Leptinotarsa decemlineata* (2)
dnc	dnc	single-copy	
DopR2	DopR2	single-copy	
dyl	dyl	single-copy	
Elp3	Elp3	single-copy	
Emb	Emb	single-copy	
FBXO11	FBXO11	single-copy	
fz2	fz2	single-copy	

Gapdh2	Gapdh2	single-copy	
Glus	-	multi-copy	*Anoplophora glabripennis* (2), *Leptinotarsa decemlineata* (2)
Hem	Hem	single-copy	
Hmgs	Hmgs	single-copy	
Hr38	Hr38	single-copy	
Hsc70-5	Hsc70-5	single-copy	
hts	-	multi-copy	*Dendroctonus ponderosae* (2)
ico	ico	single-copy	
Idh	Idh	single-copy	
Int6	Int6	single-copy	
IntS11	IntS11	single-copy	
Inx2	-	multi-copy	*Dendroctonus ponderosae* (2)
Ip259	-	multi-copy	*Dendroctonus ponderosae* (3)
l(2)37Cb	-	multi-copy	*Dendroctonus ponderosae* (2), *Leptinotarsa decemlineata* (2)
l(3)72Ab	-	multi-copy	*Agrilus planipennis* (2), *Anoplophora glabripennis* (2), *Leptinotarsa decemlineata* (2), *Onthophagus taurus*(2), *Dendroctonus ponderosae* (3), *Tribolium castaneum* (2)
Lar	Lar	single-copy	
Lon	-	multi-copy	*Agrilus planipennis* (2), *Leptinotarsa decemlineata* (2)
mor	-	multi-copy	*Dendroctonus ponderosae* (2)
Mtpalpha	-	multi-copy	*Dendroctonus ponderosae* (2)
Ndae1	Ndae1	single-copy	
nej	-	multi-copy	*Dendroctonus ponderosae* (2)
nero	nero	single-copy	
Nhel	-	single-copy	missing *Tribolium castaneum*
nito	nito	single-copy	
nonC	nonC	single-copy	
Notch	Notch	single-copy	
Nrx-IV	Nrx-IV	single-copy	
Past1	Past1	single-copy	
PlexA	-	multi-copy	*Anoplophora glabripennis* (2)
Rbcn-3A	-	multi-copy	*Leptinotarsa decemlineata* (5)
RnrL	RnrL	single-copy	
rols	-	multi-copy	*Leptinotarsa decemlineata* (3)
RpII140	RpII140	single-copy	
RpII215	-	multi-copy	*Anoplophora glabripennis* (2)
Rpn1	Rpn1	single-copy	
Rpn3	Rpn3	single-copy	
Rpn6	-	multi-copy	*Agrilus planipennis* (2), *Anoplophora glabripennis* (2), *Leptinotarsa decemlineata* (3), *Onthophagus taurus* (2), *Tribolium castaneum* (2)
Sec24	-	multi-copy	*Dendroctonus ponderosae* (2)
sec71	sec71	single-copy	
Shal	Shal	single-copy	
Shot	Shot	single-copy	
SIPA1L1	SIPA1L1	single-copy	
Spt6	Spt6	single-copy	
Su(H)	Su(H)	single-copy	
Sur-8	Sur-8	single-copy	
sxc	sxc	single-copy	

Taf2	Taf2	single-copy	
TfIIEalpha	TfIIEalpha	single-copy	
Tmp	-	multi-copy	*Agrilus planipennis* (5), *Anoplophora glabripennis* (2), *Leptinotarsa decemlineata* (5), *Onthophagus taurus* (3), *Dendroctonus ponderosae* (6), *Tribolium castaneum* (3)
Top2	Top2	single-copy	
Tor	-	multi-copy	*Onthophagus taurus* (3), *Leptinotarsa decemlineata* (3), *Agrilus planipennis* (2)
Trpml	Trpml	single-copy	
Vacht	Vacht	single-copy	
VGAT	VGAT	single-copy	
WDR44	-	multi-copy	*Onthophagus taurus* (2)
wls	wls	single-copy	
zip	zip	single-copy	

Supplementary Table S6. The list of taxa included in the phylotranscriptomic dataset and the number of available for each taxon.

Species	Accession	# of seq.	Ref. taxon	Download	Date	Citations
Lepicerus sp.	PRJNA219569	96025		NCBI, TSA	5.3.2017	1
Micromalthus debilis	PRJNA286555	28718		NCBI, TSA	6.10.2017	2
Rhagophthalmus sp.	PRJNA339505	38989		NCBI, SRA	5.3.2017	3
Cucujus clavipes	PRJNA286510	20731		NCBI, TSA	6.10.2017	2
Dryops sp.	PRJNA286519	30332		NCBI, TSA	6.10.2017	2
Anorus arizonicus	PRJNA286485	22370		NCBI, TSA	6.10.2017	2
Meloe violaceus	PRJNA219578	20011		NCBI, TSA	5.3.2017	1
Nicrophorus vespilloides	PRJNA285436	17019		NCBI, TSA	5.3.2017	4
Amphizoa insolens	PRJNA398088	23404		NCBI, TSA	6.10.2017	5
Xylobiops basilaris	PRJNA286597	16807		NCBI, TSA	6.10.2017	2
Photinus pyralis	PRJNA321737	174087		NCBI, TSA	5.3.2017	6
Thanasimus formicarius	PRJNA286592	36455		NCBI, TSA	6.10.2017	2
Gyrinus marinus	PRJNA219564	23491		NCBI, TSA	5.3.2017	1
Chauliognathus flavipes	PRJNA347807	92143		NCBI, SRA	5.3.2017	7
Phrixothrix hirtus	PRJNA347807	31428		NCBI, SRA	5.3.2017	7
Carabus granulatus	PRJNA181026	55363		NCBI, TSA	5.3.2017	8
Priacma serrata	PRJNA181032	18752		NCBI, TSA	5.3.2017	8
Agrilus planipennis	PRJNA230921	15497	yes	i5K	5.3.2017	9
Anoplophora glabripennis	PRJNA167479	22035	yes	i5K	5.3.2017	10
Dendroctonus ponderosae	PRJNA360270	13088	yes	Ens. Metazoa	5.3.2017	11
Leptinotarsa decemlineata	PRJNA171749	24671	yes	i5K	5.3.2017	9
Ontophagus taurus	PRJNA167478	17483	yes	i5K	5.3.2017	9
Tribolium castaneum	PRJNA12540	16631	yes	iBeetle-Base	5.3.2017	12, 13

References

1 Misof, B. et al.. (2014). Phylogenomics resolves the timing and pattern of insect evolution. Science, 346(6210), 763-767. doi:10.1126/science.1257570

2 Sharkey, C. R. et al. (2017). Overcoming the loss of blue sensitivity through opsin duplication in the largest animal group, beetles. Scientific Reports, 7(1). doi:10.1038/s41598-017-00061-7

3 Wang, K. et al. (2017). Transcriptome sequencing and phylogenetic analysis of four species of luminescent beetles. Scientific Reports, 7(1). doi:10.1038/s41598-017-01835-9

4 Parker, D. J. et al. (2015). Transcriptomes of parents identify parenting strategies and sexual conflict in a subsocial beetle. Nature Communications, 6, 8449. doi:10.1038/ncomms9449

5 Transcriptome of Amphizoa insolens : Unpublished – Pflug,J.M., Maddison,D.R.

6 Fallon et al. (2016). Sulfoluciferin is Biosynthesized by a Specialized Luciferin Sulfotransferase in Fireflies. Biochemistry, 55(24), 3341-3344. doi:10.1021/acs.biochem.6b00402

7 Amaral, D. T. et al. (2017). Transcriptional comparison of the photogenic and non-photogenic tissues of Phrixothrix hirtus (Coleoptera: Phengodidae) and non-luminescent *Chauliognathus flavipes* (Coleoptera: Cantharidae) give insights on the origin of lanterns in railroad worms. Gene Reports, 7, 78-86. doi:10.1016/j.genrep.2017.02.004

8 Peters, R. S. et al. (2014). The evolutionary history of holometabolous insects inferred from transcriptome-based phylogeny and comprehensive morphological data. BMC Evolutionary Biology, 14(1), 52. doi:10.1186/1471-2148-14-52

9 Poelchau, M. et al. (2014). The i5k Workspace@NAL—enabling genomic data access, visualization and curation of arthropod genomes. Nucleic Acids Research, 43(D1). doi:10.1093/nar/gku983

10 McKenna, D. D. et al. (2017) Genome of the Asian longhorned beetle (Anoplophora glabripennis), a globally significant invasive species, reveals key functional and evolutionary innovations at the beetle–plant interface. Genome Biology 2017 17(1), 227. DOI: 10.1186/s13059-016-1088-8

11 Keeling, C. I. et al. (2013). Draft genome of the mountain pine beetle, *Dendroctonus ponderosae* Hopkins, a major forest pest. Genome Biology, 14(3). doi:10.1186/gb-2013-14-3-r27

12 Shelton, J. M. et al. (2015). Tools and pipelines for BioNano data: molecule assembly pipeline and FASTA super scaffolding tool. BMC Genomics, 16(1). doi:10.1186/s12864-015-1911-8

13 Richards, S. et al. (2008). The genome of the model beetle and pest Tribolium castaneum. Nature, 452(7190), 949-955. doi:10.1038/nature06784

Supplementary Table S7. Overview of official gene sets of six reference species used for transcript ortholog assessment, including the source, version and number of genes. URLs for downloading the data are given below. Number of genes correspond with OrthoDB 9.1.

Species	Acronym	Source	Version	Download date	Number of genes	Reference
Agrilus planipennis	APLA	i5K	0.5.3	5.3.2017	15 497	1
Anopl. glabripennis	AGLA	i5K	0.5.3	5.3.2017	22 035	2
Dendr. ponderosae	YQE	Ensembl Metazoa	1.0	5.3.2017	13 088	3
Lept. decemlineata	LDEC	i5K	0.5.3	5.3.2017	24 671	1
Ontophagus taurus	OTAU	i5K	0.5.3	5.3.2017	17 483	1
Tribol. castaneum	TC	iBeetle-Base	5.2	5.3.2017	16 631	4,5

References

1 Poelchau, M. et al. (2014). The i5k Workspace@NAL—enabling genomic data access, visualization and curation of arthropod genomes. Nucleic Acids Research, 43(D1). doi:10.1093/nar/gku983

2 McKenna, D. D. et al. Genome of the Asian longhorned beetle (Anoplophora glabripennis), a globally significant invasive species, reveals key functional and evolutionary innovations at the beetle–plant interface. Genome Biology 2017 17(1), 227. DOI: 10.1186/s13059-016-1088-8

3 Keeling, C. I. et al. (2013). Draft genome of the mountain pine beetle, Dendroctonus ponderosae Hopkins, a major forest pest. Genome Biology, 14(3). doi:10.1186/gb-2013-14-3-r27

4 Shelton, J. M. et al. (2015). Tools and pipelines for BioNano data: molecule assembly pipeline and FASTA super scaffolding tool. BMC Genomics, 16(1). doi:10.1186/s12864-015-1911-8

5 Richards, S. et al. (2008). The genome of the model beetle and pest Tribolium castaneum. Nature, 452(7190), 949-955. doi:10.1038/nature06784

Tribolium castaneum

http://bioinf.uni-greifswald.de/tcas/genes/tcas5_annotation/Tcas5.2_GenBank.corrected_v5.renamed.aa

http://bioinf.uni-greifswald.de/tcas/genes/tcas5_annotation/Tcas5.2_GenBank.corrected_v5.renamed.codingseq

Onthophagus taurus

https://i5k.nal.usda.gov/data/Arthropoda/onttau-%28Onthophagus_taurus%29/Current%20Genome%20Assembly/2.Official%20or%20Primary%20Gene%20Set/BCM_version_0.5.3/consensus_gene_set/OTAU.faa

https://i5k.nal.usda.gov/data/Arthropoda/onttau-%28Onthophagus_taurus%29/Current%20Genome%20Assembly/2.Official%20or%20Primary%20Gene%20Set/BCM_version_0.5.3/consensus_gene_set/OTAU.CDS.fna

Anoplophora glabripennis
https://i5k.nal.usda.gov/data/Arthropoda/anogla-%28Anoplophora_glabripennis%29/Current%20Genome%20Assembly/3.Additional%20Gene%20Sets%20and%20Annotation%20Projects/BCM_version_0.5.3-Primary_Gene_Set/primary_gene_set/AGLA.CDS.fna.gz
https://i5k.nal.usda.gov/data/Arthropoda/anogla-%28Anoplophora_glabripennis%29/Current%20Genome%20Assembly/3.Additional%20Gene%20Sets%20and%20Annotation%20Projects/BCM_version_0.5.3-Primary_Gene_Set/primary_gene_set/AGLA.faa.gz
Agrilus planipennis
https://i5k.nal.usda.gov/data/Arthropoda/agrpla-%28Agrilus_planipennis%29/Current%20Genome%20Assembly/2.Official%20or%20Primary%20Gene%20Set/BCM_version_0.5.3/consensus_gene_set/APLA.faa
https://i5k.nal.usda.gov/data/Arthropoda/agrpla-%28Agrilus_planipennis%29/Current%20Genome%20Assembly/2.Official%20or%20Primary%20Gene%20Set/BCM_version_0.5.3/consensus_gene_set/APLA.CDS.fna
Dendroctonus ponderosae
ftp://ftp.ensemblgenomes.org/pub/metazoa/release-37/fasta/dendroctonus_ponderosae/cds/Dendroctonus_ponderosae.DendPond_male_1.0.cds.all.fa.gz
ftp://ftp.ensemblgenomes.org/pub/metazoa/release-37/fasta/dendroctonus_ponderosae/pep/Dendroctonus_ponderosae.DendPond_male_1.0.pep.all.fa.gz
Leptinotarsa decemlineata
https://i5k.nal.usda.gov/data/Arthropoda/lepdec-%28Leptinotarsa_decemlineata%29/Current%20Genome%20Assembly/2.Official%20or%20Primary%20Gene%20Set/BCM_version_0.5.3-Primary_Gene_Set/primary_gene_set/LDEC.faa.gz
https://i5k.nal.usda.gov/data/Arthropoda/lepdec-%28Leptinotarsa_decemlineata%29/Current%20Genome%20Assembly/2.Official%20or%20Primary%20Gene%20Set/BCM_version_0.5.3-Primary_Gene_Set/primary_gene_set/LDEC.CDS.fna.gz

Supplementary Table S8.

OG ID	Gene ID	Species	Description
EOG09120001	OTAU012964	Onthophagus taurus	Putative uncharacterized protein
EOG09120001	AGLA002212	Anoplophora glabripennis	Putative uncharacterized protein
EOG09120001	APLA003512	Agrilus planipennis	Putative uncharacterized protein
EOG09120001	TC004721	Tribolium castaneum	Putative uncharacterized protein
EOG09120001	LDEC009170	Leptinotarsa decemlineata	Putative uncharacterized protein
EOG09120001	YQE_02432	Dendroctonus ponderosae	Putative uncharacterized protein
EOG09120002	OTAU005778	Onthophagus taurus	Putative uncharacterized protein
EOG09120002	AGLA015499	Anoplophora glabripennis	Putative uncharacterized protein
EOG09120002	APLA010155	Agrilus planipennis	Putative uncharacterized protein
EOG09120002	TC030701	Tribolium castaneum	Putative uncharacterized protein
EOG09120002	LDEC009802	Leptinotarsa decemlineata	Putative uncharacterized protein
EOG09120002	YQE_05866	Dendroctonus ponderosae	Putative uncharacterized protein
EOG09120003	OTAU014439	Onthophagus taurus	calcium ion binding
EOG09120003	AGLA007235	Anoplophora glabripennis	calcium ion binding
EOG09120003	APLA012282	Agrilus planipennis	calcium ion binding
EOG09120003	TC033680	Tribolium castaneum	calcium ion binding
EOG09120003	LDEC009012	Leptinotarsa decemlineata	calcium ion binding
EOG09120003	YQE_08797	Dendroctonus ponderosae	calcium ion binding
EOG09120006	OTAU006802	Onthophagus taurus	Putative uncharacterized protein
EOG09120006	AGLA003416	Anoplophora glabripennis	Putative uncharacterized protein
EOG09120006	APLA002019	Agrilus planipennis	Putative uncharacterized protein
EOG09120006	TC002610	Tribolium castaneum	Putative uncharacterized protein
EOG09120006	LDEC020453	Leptinotarsa decemlineata	Putative uncharacterized protein
EOG09120006	YQE_12305	Dendroctonus ponderosae	Putative uncharacterized protein
EOG09120008	OTAU005795	Onthophagus taurus	nucleoside-triphosphatase activity
EOG09120008	AGLA001513	Anoplophora glabripennis	nucleoside-triphosphatase activity
EOG09120008	APLA003789	Agrilus planipennis	nucleoside-triphosphatase activity
EOG09120008	TC034619	Tribolium castaneum	nucleoside-triphosphatase activity
EOG09120008	LDEC004651	Leptinotarsa decemlineata	nucleoside-triphosphatase activity
EOG09120008	YQE_04556	Dendroctonus ponderosae	nucleoside-triphosphatase activity
EOG0912000A	OTAU003247	Onthophagus taurus	Putative uncharacterized protein
EOG0912000A	AGLA006825	Anoplophora glabripennis	Putative uncharacterized protein
EOG0912000A	APLA014915	Agrilus planipennis	Putative uncharacterized protein
EOG0912000A	TC008640	Tribolium castaneum	Putative uncharacterized protein
EOG0912000A	LDEC018396	Leptinotarsa decemlineata	Putative uncharacterized protein
EOG0912000A	YQE_12637	Dendroctonus ponderosae	Putative uncharacterized protein
EOG0912000C	OTAU011459	Onthophagus taurus	Putative uncharacterized protein
EOG0912000C	AGLA016973	Anoplophora glabripennis	Putative uncharacterized protein
EOG0912000C	APLA005710	Agrilus planipennis	Putative uncharacterized protein
EOG0912000C	TC009719	Tribolium castaneum	Putative uncharacterized protein
EOG0912000C	LDEC002210	Leptinotarsa decemlineata	Putative uncharacterized protein
EOG0912000C	YQE_12461	Dendroctonus ponderosae	Putative uncharacterized protein
EOG0912000F	OTAU005953	Onthophagus taurus	calcium ion binding
EOG0912000F	AGLA019450	Anoplophora glabripennis	calcium ion binding
EOG0912000F	APLA014600	Agrilus planipennis	calcium ion binding
EOG0912000F	TC032760	Tribolium castaneum	calcium ion binding
EOG0912000F	LDEC000172	Leptinotarsa decemlineata	calcium ion binding
EOG0912000F	YQE_04187	Dendroctonus ponderosae	calcium ion binding
EOG0912000K	OTAU010241	Onthophagus taurus	Putative uncharacterized protein
EOG0912000K	AGLA007494	Anoplophora glabripennis	Putative uncharacterized protein
EOG0912000K	APLA008718	Agrilus planipennis	Putative uncharacterized protein
EOG0912000K	TC000076	Tribolium castaneum	Putative uncharacterized protein
EOG0912000K	LDEC018579	Leptinotarsa decemlineata	Putative uncharacterized protein
EOG0912000K	YQE_09854	Dendroctonus ponderosae	Putative uncharacterized protein
EOG0912000O	OTAU008405	Onthophagus taurus	phospholipid binding
EOG0912000O	AGLA001476	Anoplophora glabripennis	phospholipid binding
EOG0912000O	APLA008728	Agrilus planipennis	phospholipid binding
EOG0912000O	TC032479	Tribolium castaneum	phospholipid binding
EOG0912000O	LDEC003299	Leptinotarsa decemlineata	phospholipid binding
EOG0912000O	YQE_05183	Dendroctonus ponderosae	phospholipid binding
EOG0912000P	OTAU014311	Onthophagus taurus	binding
EOG0912000P	AGLA003943	Anoplophora glabripennis	binding
EOG0912000P	APLA014432	Agrilus planipennis	binding

EOG0912000P	TC033331	Tribolium castaneum	binding
EOG0912000P	LDEC012251	Leptinotarsa decemlineata	binding
EOG0912000P	YQE_11668	Dendroctonus ponderosae	binding
EOG0912000T	OTAU005288	Onthophagus taurus	None
EOG0912000T	AGLA000513	Anoplophora glabripennis	None
EOG0912000T	APLA002278	Agrilus planipennis	None
EOG0912000T	TC032815	Tribolium castaneum	None
EOG0912000T	LDEC002378	Leptinotarsa decemlineata	None
EOG0912000T	YQE_11437	Dendroctonus ponderosae	None
EOG0912000V	OTAU001128	Onthophagus taurus	calcium ion binding
EOG0912000V	AGLA013328	Anoplophora glabripennis	calcium ion binding
EOG0912000V	APLA005499	Agrilus planipennis	calcium ion binding
EOG0912000V	TC032189	Tribolium castaneum	calcium ion binding
EOG0912000V	LDEC015706	Leptinotarsa decemlineata	calcium ion binding
EOG0912000V	YQE_01688	Dendroctonus ponderosae	calcium ion binding
EOG0912000X	OTAU003857	Onthophagus taurus	Ubiquitin carboxyl-terminal hydrolase
EOG0912000X	AGLA007993	Anoplophora glabripennis	Ubiquitin carboxyl-terminal hydrolase
EOG0912000X	APLA006948	Agrilus planipennis	Ubiquitin carboxyl-terminal hydrolase
EOG0912000X	TC009817	Tribolium castaneum	Ubiquitin carboxyl-terminal hydrolase
EOG0912000X	LDEC010700	Leptinotarsa decemlineata	Ubiquitin carboxyl-terminal hydrolase
EOG0912000X	YQE_12604	Dendroctonus ponderosae	Ubiquitin carboxyl-terminal hydrolase
EOG09120010	OTAU016531	Onthophagus taurus	Putative uncharacterized protein
EOG09120010	AGLA008094	Anoplophora glabripennis	Putative uncharacterized protein
EOG09120010	APLA007705	Agrilus planipennis	Putative uncharacterized protein
EOG09120010	TC000971	Tribolium castaneum	Putative uncharacterized protein
EOG09120010	LDEC003165	Leptinotarsa decemlineata	Putative uncharacterized protein
EOG09120010	YQE_08475	Dendroctonus ponderosae	Putative uncharacterized protein
EOG09120013	OTAU011009	Onthophagus taurus	binding
EOG09120013	AGLA001130	Anoplophora glabripennis	binding
EOG09120013	APLA005595	Agrilus planipennis	binding
EOG09120013	TC031561	Tribolium castaneum	binding
EOG09120013	LDEC009163	Leptinotarsa decemlineata	binding
EOG09120013	YQE_02454	Dendroctonus ponderosae	binding
EOG09120016	OTAU000647	Onthophagus taurus	Starry night
EOG09120016	AGLA008378	Anoplophora glabripennis	Starry night
EOG09120016	APLA010398	Agrilus planipennis	Starry night
EOG09120016	TC012521	Tribolium castaneum	Starry night
EOG09120016	LDEC021911	Leptinotarsa decemlineata	Starry night
EOG09120016	YQE_12981	Dendroctonus ponderosae	Starry night
EOG09120017	OTAU010843	Onthophagus taurus	Alpha spectrin
EOG09120017	AGLA002967	Anoplophora glabripennis	Alpha spectrin
EOG09120017	APLA001292	Agrilus planipennis	Alpha spectrin
EOG09120017	TC000749	Tribolium castaneum	Alpha spectrin
EOG09120017	LDEC006258	Leptinotarsa decemlineata	Alpha spectrin
EOG09120017	YQE_01583	Dendroctonus ponderosae	Alpha spectrin
EOG09120019	OTAU006765	Onthophagus taurus	DNA binding
EOG09120019	AGLA007017	Anoplophora glabripennis	DNA binding
EOG09120019	APLA009547	Agrilus planipennis	DNA binding
EOG09120019	TC032626	Tribolium castaneum	DNA binding
EOG09120019	LDEC016603	Leptinotarsa decemlineata	DNA binding
EOG09120019	YQE_10224	Dendroctonus ponderosae	DNA binding
EOG0912001A	OTAU003320	Onthophagus taurus	lipid transporter activity
EOG0912001A	AGLA002199	Anoplophora glabripennis	lipid transporter activity
EOG0912001A	APLA003678	Agrilus planipennis	lipid transporter activity
EOG0912001A	TC031518	Tribolium castaneum	lipid transporter activity
EOG0912001A	LDEC001101	Leptinotarsa decemlineata	lipid transporter activity
EOG0912001A	YQE_06611	Dendroctonus ponderosae	lipid transporter activity
EOG0912001B	OTAU010837	Onthophagus taurus	None
EOG0912001B	AGLA016199	Anoplophora glabripennis	None
EOG0912001B	APLA013085	Agrilus planipennis	None
EOG0912001B	TC033411	Tribolium castaneum	None
EOG0912001B	LDEC008000	Leptinotarsa decemlineata	None
EOG0912001B	YQE_09410	Dendroctonus ponderosae	None
EOG0912001D	OTAU013600	Onthophagus taurus	Putative uncharacterized protein
EOG0912001D	AGLA014834	Anoplophora glabripennis	Putative uncharacterized protein
EOG0912001D	APLA002201	Agrilus planipennis	Putative uncharacterized protein
EOG0912001D	TC007861	Tribolium castaneum	Putative uncharacterized protein

EOG0912001D	LDEC004607	Leptinotarsa decemlineata	Putative uncharacterized protein
EOG0912001D	YQE_04641	Dendroctonus ponderosae	Putative uncharacterized protein
EOG0912001GOTAU001334		Onthophagus taurus	Putative uncharacterized protein
EOG0912001GAGLA002345		Anoplophora glabripennis	Putative uncharacterized protein
EOG0912001GAPLA009955		Agrilus planipennis	Putative uncharacterized protein
EOG0912001GTC010767		Tribolium castaneum	Putative uncharacterized protein
EOG0912001GLDEC001063		Leptinotarsa decemlineata	Putative uncharacterized protein
EOG0912001GYQE_09078		Dendroctonus ponderosae	Putative uncharacterized protein
EOG0912001H	OTAU004322	Onthophagus taurus	Putative uncharacterized protein
EOG0912001H	AGLA004535	Anoplophora glabripennis	Putative uncharacterized protein
EOG0912001H	APLA006754	Agrilus planipennis	Putative uncharacterized protein
EOG0912001H	TC005186	Tribolium castaneum	Putative uncharacterized protein
EOG0912001H	LDEC003530	Leptinotarsa decemlineata	Putative uncharacterized protein
EOG0912001H	YQE_09299	Dendroctonus ponderosae	Putative uncharacterized protein
EOG0912001I	OTAU010984	Onthophagus taurus	Putative uncharacterized protein
EOG0912001I	AGLA000106	Anoplophora glabripennis	Putative uncharacterized protein
EOG0912001I	APLA011139	Agrilus planipennis	Putative uncharacterized protein
EOG0912001I	TC000586	Tribolium castaneum	Putative uncharacterized protein
EOG0912001I	LDEC003169	Leptinotarsa decemlineata	Putative uncharacterized protein
EOG0912001I	YQE_07746	Dendroctonus ponderosae	Putative uncharacterized protein
EOG0912001J	OTAU005992	Onthophagus taurus	Putative uncharacterized protein
EOG0912001J	AGLA018213	Anoplophora glabripennis	Putative uncharacterized protein
EOG0912001J	APLA002072	Agrilus planipennis	Putative uncharacterized protein
EOG0912001J	TC011522	Tribolium castaneum	Putative uncharacterized protein
EOG0912001J	LDEC010285	Leptinotarsa decemlineata	Putative uncharacterized protein
EOG0912001J	YQE_09777	Dendroctonus ponderosae	Putative uncharacterized protein
EOG0912001L	OTAU006378	Onthophagus taurus	Putative uncharacterized protein
EOG0912001L	AGLA006496	Anoplophora glabripennis	Putative uncharacterized protein
EOG0912001L	APLA014053	Agrilus planipennis	Putative uncharacterized protein
EOG0912001L	TC008668	Tribolium castaneum	Putative uncharacterized protein
EOG0912001L	LDEC006173	Leptinotarsa decemlineata	Putative uncharacterized protein
EOG0912001L	YQE_10546	Dendroctonus ponderosae	Putative uncharacterized protein
EOG0912001M	OTAU002012	Onthophagus taurus	Putative uncharacterized protein
EOG0912001M	AGLA005110	Anoplophora glabripennis	Putative uncharacterized protein
EOG0912001M	APLA007660	Agrilus planipennis	Putative uncharacterized protein
EOG0912001M	TC012734	Tribolium castaneum	Putative uncharacterized protein
EOG0912001M	LDEC003889	Leptinotarsa decemlineata	Putative uncharacterized protein
EOG0912001M	YQE_12947	Dendroctonus ponderosae	Putative uncharacterized protein
EOG0912001N	OTAU001423	Onthophagus taurus	Putative uncharacterized protein
EOG0912001N	AGLA000343	Anoplophora glabripennis	Putative uncharacterized protein
EOG0912001N	APLA010352	Agrilus planipennis	Putative uncharacterized protein
EOG0912001N	TC004798	Tribolium castaneum	Putative uncharacterized protein
EOG0912001N	LDEC003776	Leptinotarsa decemlineata	Putative uncharacterized protein
EOG0912001N	YQE_03246	Dendroctonus ponderosae	Putative uncharacterized protein
EOG0912001O	OTAU005942	Onthophagus taurus	Putative uncharacterized protein
EOG0912001O	AGLA000440	Anoplophora glabripennis	Putative uncharacterized protein
EOG0912001O	APLA009207	Agrilus planipennis	Putative uncharacterized protein
EOG0912001O	TC007046	Tribolium castaneum	Putative uncharacterized protein
EOG0912001O	LDEC008663	Leptinotarsa decemlineata	Putative uncharacterized protein
EOG0912001O	YQE_02993	Dendroctonus ponderosae	Putative uncharacterized protein
EOG0912001Q	OTAU000052	Onthophagus taurus	Ubiquitin carboxyl-terminal hydrolase
EOG0912001Q	AGLA006408	Anoplophora glabripennis	Ubiquitin carboxyl-terminal hydrolase
EOG0912001Q	APLA009822	Agrilus planipennis	Ubiquitin carboxyl-terminal hydrolase
EOG0912001Q	TC010455	Tribolium castaneum	Ubiquitin carboxyl-terminal hydrolase
EOG0912001Q	LDEC013321	Leptinotarsa decemlineata	Ubiquitin carboxyl-terminal hydrolase
EOG0912001Q	YQE_02083	Dendroctonus ponderosae	Ubiquitin carboxyl-terminal hydrolase
EOG0912001S	OTAU003150	Onthophagus taurus	Putative uncharacterized protein
EOG0912001S	AGLA011146	Anoplophora glabripennis	Putative uncharacterized protein
EOG0912001S	APLA008362	Agrilus planipennis	Putative uncharacterized protein
EOG0912001S	TC014081	Tribolium castaneum	Putative uncharacterized protein
EOG0912001S	LDEC013718	Leptinotarsa decemlineata	Putative uncharacterized protein
EOG0912001S	YQE_08201	Dendroctonus ponderosae	Putative uncharacterized protein
EOG0912001U	OTAU004648	Onthophagus taurus	Putative uncharacterized protein
EOG0912001U	AGLA004922	Anoplophora glabripennis	Putative uncharacterized protein
EOG0912001U	APLA004130	Agrilus planipennis	Putative uncharacterized protein
EOG0912001U	TC008934	Tribolium castaneum	Putative uncharacterized protein
EOG0912001U	LDEC014267	Leptinotarsa decemlineata	Putative uncharacterized protein

EOG0912001U	YQE_03171	Dendroctonus ponderosae	Putative uncharacterized protein
EOG0912001V	OTAU012842	Onthophagus taurus	Putative uncharacterized protein
EOG0912001V	AGLA013702	Anoplophora glabripennis	Putative uncharacterized protein
EOG0912001V	APLA011927	Agrilus planipennis	Putative uncharacterized protein
EOG0912001V	TC004393	Tribolium castaneum	Putative uncharacterized protein
EOG0912001V	LDEC016542	Leptinotarsa decemlineata	Putative uncharacterized protein
EOG0912001V	YQE_07751	Dendroctonus ponderosae	Putative uncharacterized protein
EOG0912001Y	OTAU002314	Onthophagus taurus	Putative uncharacterized protein
EOG0912001Y	AGLA010082	Anoplophora glabripennis	Putative uncharacterized protein
EOG0912001Y	APLA014976	Agrilus planipennis	Putative uncharacterized protein
EOG0912001Y	TC016161	Tribolium castaneum	Putative uncharacterized protein
EOG0912001Y	LDEC004999	Leptinotarsa decemlineata	Putative uncharacterized protein
EOG0912001Y	YQE_01909	Dendroctonus ponderosae	Putative uncharacterized protein
EOG0912001Z	OTAU013159	Onthophagus taurus	Putative uncharacterized protein
EOG0912001Z	AGLA011142	Anoplophora glabripennis	Putative uncharacterized protein
EOG0912001Z	APLA002786	Agrilus planipennis	Putative uncharacterized protein
EOG0912001Z	TC013716	Tribolium castaneum	Putative uncharacterized protein
EOG0912001Z	LDEC013723	Leptinotarsa decemlineata	Putative uncharacterized protein
EOG0912001Z	YQE_08199	Dendroctonus ponderosae	Putative uncharacterized protein
EOG09120021	OTAU008921	Onthophagus taurus	Trithorax
EOG09120021	AGLA000269	Anoplophora glabripennis	Trithorax
EOG09120021	APLA006438	Agrilus planipennis	Trithorax
EOG09120021	TC004768	Tribolium castaneum	Trithorax
EOG09120021	LDEC011089	Leptinotarsa decemlineata	Trithorax
EOG09120021	YQE_09091	Dendroctonus ponderosae	Trithorax
EOG09120022	OTAU003943	Onthophagus taurus	Putative uncharacterized protein
EOG09120022	AGLA006859	Anoplophora glabripennis	Putative uncharacterized protein
EOG09120022	APLA002345	Agrilus planipennis	Putative uncharacterized protein
EOG09120022	TC012392	Tribolium castaneum	Putative uncharacterized protein
EOG09120022	LDEC011866	Leptinotarsa decemlineata	Putative uncharacterized protein
EOG09120022	YQE_02688	Dendroctonus ponderosae	Putative uncharacterized protein
EOG09120025	OTAU012195	Onthophagus taurus	Putative uncharacterized protein
EOG09120025	AGLA016457	Anoplophora glabripennis	Putative uncharacterized protein
EOG09120025	APLA002495	Agrilus planipennis	Putative uncharacterized protein
EOG09120025	TC014275	Tribolium castaneum	Putative uncharacterized protein
EOG09120025	LDEC022232	Leptinotarsa decemlineata	Putative uncharacterized protein
EOG09120025	YQE_06319	Dendroctonus ponderosae	Putative uncharacterized protein
EOG09120026	OTAU012825	Onthophagus taurus	lipid transporter activity
EOG09120026	AGLA003897	Anoplophora glabripennis	lipid transporter activity
EOG09120026	APLA002555	Agrilus planipennis	lipid transporter activity
EOG09120026	TC034740	Tribolium castaneum	lipid transporter activity
EOG09120026	LDEC003616	Leptinotarsa decemlineata	lipid transporter activity
EOG09120026	YQE_02131	Dendroctonus ponderosae	lipid transporter activity
EOG09120027	OTAU005777	Onthophagus taurus	None
EOG09120027	AGLA013043	Anoplophora glabripennis	None
EOG09120027	APLA010156	Agrilus planipennis	None
EOG09120027	TC032674	Tribolium castaneum	None
EOG09120027	LDEC009803	Leptinotarsa decemlineata	None
EOG09120027	YQE_05867	Dendroctonus ponderosae	None
EOG0912002C	OTAU000410	Onthophagus taurus	Putative uncharacterized protein
EOG0912002C	AGLA020403	Anoplophora glabripennis	Putative uncharacterized protein
EOG0912002C	APLA006652	Agrilus planipennis	Putative uncharacterized protein
EOG0912002C	TC003342	Tribolium castaneum	Putative uncharacterized protein
EOG0912002C	LDEC011962	Leptinotarsa decemlineata	Putative uncharacterized protein
EOG0912002C	YQE_03781	Dendroctonus ponderosae	Putative uncharacterized protein
EOG0912002F	OTAU002724	Onthophagus taurus	Putative uncharacterized protein
EOG0912002F	AGLA000385	Anoplophora glabripennis	Putative uncharacterized protein
EOG0912002F	APLA003565	Agrilus planipennis	Putative uncharacterized protein
EOG0912002F	TC004631	Tribolium castaneum	Putative uncharacterized protein
EOG0912002F	LDEC001533	Leptinotarsa decemlineata	Putative uncharacterized protein
EOG0912002F	YQE_06655	Dendroctonus ponderosae	Putative uncharacterized protein
EOG0912002H	OTAU009926	Onthophagus taurus	Putative uncharacterized protein
EOG0912002H	AGLA012421	Anoplophora glabripennis	Putative uncharacterized protein
EOG0912002H	APLA003268	Agrilus planipennis	Putative uncharacterized protein
EOG0912002H	TC012344	Tribolium castaneum	Putative uncharacterized protein
EOG0912002H	LDEC012361	Leptinotarsa decemlineata	Putative uncharacterized protein
EOG0912002H	YQE_08006	Dendroctonus ponderosae	Putative uncharacterized protein

EOG0912002I	OTAU002196	Onthophagus taurus	None
EOG0912002I	AGLA018298	Anoplophora glabripennis	None
EOG0912002I	APLA000129	Agrilus planipennis	None
EOG0912002I	TC031058	Tribolium castaneum	None
EOG0912002I	LDEC007905	Leptinotarsa decemlineata	None
EOG0912002I	YQE_05141	Dendroctonus ponderosae	None
EOG0912002J	OTAU001286	Onthophagus taurus	Plexin B
EOG0912002J	AGLA002196	Anoplophora glabripennis	Plexin B
EOG0912002J	APLA015225	Agrilus planipennis	Plexin B
EOG0912002J	TC004144	Tribolium castaneum	Plexin B
EOG0912002J	LDEC004321	Leptinotarsa decemlineata	Plexin B
EOG0912002J	YQE_09068	Dendroctonus ponderosae	Plexin B
EOG0912002L	OTAU006249	Onthophagus taurus	DNA polymerase
EOG0912002L	AGLA011807	Anoplophora glabripennis	DNA polymerase
EOG0912002L	APLA008283	Agrilus planipennis	DNA polymerase
EOG0912002L	TC009600	Tribolium castaneum	DNA polymerase
EOG0912002L	LDEC007562	Leptinotarsa decemlineata	DNA polymerase
EOG0912002L	YQE_05287	Dendroctonus ponderosae	DNA polymerase
EOG0912002R	OTAU011834	Onthophagus taurus	None
EOG0912002R	AGLA012826	Anoplophora glabripennis	None
EOG0912002R	APLA006139	Agrilus planipennis	None
EOG0912002R	TC031046	Tribolium castaneum	None
EOG0912002R	LDEC005983	Leptinotarsa decemlineata	None
EOG0912002R	YQE_06177	Dendroctonus ponderosae	None
EOG0912002S	OTAU000258	Onthophagus taurus	Putative uncharacterized protein
EOG0912002S	AGLA016395	Anoplophora glabripennis	Putative uncharacterized protein
EOG0912002S	APLA009850	Agrilus planipennis	Putative uncharacterized protein
EOG0912002S	TC013742	Tribolium castaneum	Putative uncharacterized protein
EOG0912002S	LDEC015108	Leptinotarsa decemlineata	Putative uncharacterized protein
EOG0912002S	YQE_08223	Dendroctonus ponderosae	Putative uncharacterized protein
EOG0912002T	OTAU000277	Onthophagus taurus	Putative uncharacterized protein
EOG0912002T	AGLA011399	Anoplophora glabripennis	Putative uncharacterized protein
EOG0912002T	APLA012878	Agrilus planipennis	Putative uncharacterized protein
EOG0912002T	TC013407	Tribolium castaneum	Putative uncharacterized protein
EOG0912002T	LDEC006524	Leptinotarsa decemlineata	Putative uncharacterized protein
EOG0912002T	YQE_07214	Dendroctonus ponderosae	Putative uncharacterized protein
EOG0912002V	OTAU000604	Onthophagus taurus	Putative uncharacterized protein
EOG0912002V	AGLA012419	Anoplophora glabripennis	Putative uncharacterized protein
EOG0912002V	APLA008680	Agrilus planipennis	Putative uncharacterized protein
EOG0912002V	TC012345	Tribolium castaneum	Putative uncharacterized protein
EOG0912002V	LDEC021292	Leptinotarsa decemlineata	Putative uncharacterized protein
EOG0912002V	YQE_08005	Dendroctonus ponderosae	Putative uncharacterized protein
EOG0912002Y	OTAU005733	Onthophagus taurus	Putative uncharacterized protein
EOG0912002Y	AGLA006967	Anoplophora glabripennis	Putative uncharacterized protein
EOG0912002Y	APLA002256	Agrilus planipennis	Putative uncharacterized protein
EOG0912002Y	TC002360	Tribolium castaneum	Putative uncharacterized protein
EOG0912002Y	LDEC006957	Leptinotarsa decemlineata	Putative uncharacterized protein
EOG0912002Y	YQE_05154	Dendroctonus ponderosae	Putative uncharacterized protein
EOG0912002Z	OTAU009820	Onthophagus taurus	Putative uncharacterized protein
EOG0912002Z	AGLA005964	Anoplophora glabripennis	Putative uncharacterized protein
EOG0912002Z	APLA004450	Agrilus planipennis	Putative uncharacterized protein
EOG0912002Z	TC005447	Tribolium castaneum	Putative uncharacterized protein
EOG0912002Z	LDEC011212	Leptinotarsa decemlineata	Putative uncharacterized protein
EOG0912002Z	YQE_02849	Dendroctonus ponderosae	Putative uncharacterized protein
EOG09120030	OTAU005615	Onthophagus taurus	None
EOG09120030	AGLA005748	Anoplophora glabripennis	None
EOG09120030	APLA010178	Agrilus planipennis	None
EOG09120030	TC033567	Tribolium castaneum	None
EOG09120030	LDEC007457	Leptinotarsa decemlineata	None
EOG09120030	YQE_11317	Dendroctonus ponderosae	None
EOG09120031	OTAU008173	Onthophagus taurus	Putative uncharacterized protein
EOG09120031	AGLA019383	Anoplophora glabripennis	Putative uncharacterized protein
EOG09120031	APLA008767	Agrilus planipennis	Putative uncharacterized protein
EOG09120031	TC015014	Tribolium castaneum	Putative uncharacterized protein
EOG09120031	LDEC017401	Leptinotarsa decemlineata	Putative uncharacterized protein
EOG09120031	YQE_10839	Dendroctonus ponderosae	Putative uncharacterized protein
EOG09120033	OTAU006058	Onthophagus taurus	helicase activity

EOG09120033	AGLA001958	Anoplophora glabripennis	helicase activity
EOG09120033	APLA010015	Agrilus planipennis	helicase activity
EOG09120033	TC034734	Tribolium castaneum	helicase activity
EOG09120033	LDEC015640	Leptinotarsa decemlineata	helicase activity
EOG09120033	YQE_07311	Dendroctonus ponderosae	helicase activity
EOG09120034	OTAU009380	Onthophagus taurus	Putative uncharacterized protein
EOG09120034	AGLA005860	Anoplophora glabripennis	Putative uncharacterized protein
EOG09120034	APLA001547	Agrilus planipennis	Putative uncharacterized protein
EOG09120034	TC010195	Tribolium castaneum	Putative uncharacterized protein
EOG09120034	LDEC013264	Leptinotarsa decemlineata	Putative uncharacterized protein
EOG09120034	YQE_11556	Dendroctonus ponderosae	Putative uncharacterized protein
EOG09120036	OTAU005879	Onthophagus taurus	calcium ion binding
EOG09120036	AGLA001785	Anoplophora glabripennis	calcium ion binding
EOG09120036	APLA000833	Agrilus planipennis	calcium ion binding
EOG09120036	TC033193	Tribolium castaneum	calcium ion binding
EOG09120036	LDEC010187	Leptinotarsa decemlineata	calcium ion binding
EOG09120036	YQE_03875	Dendroctonus ponderosae	calcium ion binding
EOG09120039	OTAU013438	Onthophagus taurus	None
EOG09120039	AGLA002002	Anoplophora glabripennis	None
EOG09120039	APLA000742	Agrilus planipennis	None
EOG09120039	TC032643	Tribolium castaneum	None
EOG09120039	LDEC004572	Leptinotarsa decemlineata	None
EOG09120039	YQE_12406	Dendroctonus ponderosae	None
EOG0912003D	OTAU002816	Onthophagus taurus	Putative uncharacterized protein
EOG0912003D	AGLA016795	Anoplophora glabripennis	Putative uncharacterized protein
EOG0912003D	APLA011850	Agrilus planipennis	Putative uncharacterized protein
EOG0912003D	TC004583	Tribolium castaneum	Putative uncharacterized protein
EOG0912003D	LDEC014493	Leptinotarsa decemlineata	Putative uncharacterized protein
EOG0912003D	YQE_04851	Dendroctonus ponderosae	Putative uncharacterized protein
EOG0912003E	OTAU003382	Onthophagus taurus	Domino
EOG0912003E	AGLA006599	Anoplophora glabripennis	Domino
EOG0912003E	APLA013849	Agrilus planipennis	Domino
EOG0912003E	TC012058	Tribolium castaneum	Domino
EOG0912003E	LDEC007663	Leptinotarsa decemlineata	Domino
EOG0912003E	YQE_12826	Dendroctonus ponderosae	Domino
EOG0912003H	OTAU010043	Onthophagus taurus	Putative uncharacterized protein
EOG0912003H	AGLA000485	Anoplophora glabripennis	Putative uncharacterized protein
EOG0912003H	APLA012798	Agrilus planipennis	Putative uncharacterized protein
EOG0912003H	TC007146	Tribolium castaneum	Putative uncharacterized protein
EOG0912003H	LDEC013476	Leptinotarsa decemlineata	Putative uncharacterized protein
EOG0912003H	YQE_03106	Dendroctonus ponderosae	Putative uncharacterized protein
EOG0912003I	OTAU000295	Onthophagus taurus	Midasin
EOG0912003I	AGLA011920	Anoplophora glabripennis	Midasin
EOG0912003I	APLA014458	Agrilus planipennis	Midasin
EOG0912003I	TC012898	Tribolium castaneum	Midasin
EOG0912003I	LDEC017774	Leptinotarsa decemlineata	Midasin
EOG0912003I	YQE_02094	Dendroctonus ponderosae	Midasin
EOG0912003M	OTAU016195	Onthophagus taurus	Putative uncharacterized protein
EOG0912003M	AGLA012291	Anoplophora glabripennis	Putative uncharacterized protein
EOG0912003M	APLA006008	Agrilus planipennis	Putative uncharacterized protein
EOG0912003M	TC009430	Tribolium castaneum	Putative uncharacterized protein
EOG0912003M	LDEC014666	Leptinotarsa decemlineata	Putative uncharacterized protein
EOG0912003M	YQE_12996	Dendroctonus ponderosae	Putative uncharacterized protein
EOG0912003N	OTAU008441	Onthophagus taurus	Megator
EOG0912003N	AGLA013913	Anoplophora glabripennis	Megator
EOG0912003N	APLA006060	Agrilus planipennis	Megator
EOG0912003N	TC002131	Tribolium castaneum	Megator
EOG0912003N	LDEC007909	Leptinotarsa decemlineata	Megator
EOG0912003N	YQE_03776	Dendroctonus ponderosae	Megator
EOG0912003O	OTAU003666	Onthophagus taurus	Putative uncharacterized protein
EOG0912003O	AGLA000682	Anoplophora glabripennis	Putative uncharacterized protein
EOG0912003O	APLA005352	Agrilus planipennis	Putative uncharacterized protein
EOG0912003O	TC007308	Tribolium castaneum	Putative uncharacterized protein
EOG0912003O	LDEC000658	Leptinotarsa decemlineata	Putative uncharacterized protein
EOG0912003O	YQE_03347	Dendroctonus ponderosae	Putative uncharacterized protein
EOG0912003R	OTAU001633	Onthophagus taurus	Putative uncharacterized protein
EOG0912003R	AGLA012526	Anoplophora glabripennis	Putative uncharacterized protein

EOG0912003R	APLA008462	Agrilus planipennis	Putative uncharacterized protein
EOG0912003R	TC006356	Tribolium castaneum	Putative uncharacterized protein
EOG0912003R	LDEC010083	Leptinotarsa decemlineata	Putative uncharacterized protein
EOG0912003R	YQE_12110	Dendroctonus ponderosae	Putative uncharacterized protein
EOG0912003S	OTAU006013	Onthophagus taurus	Putative uncharacterized protein
EOG0912003S	AGLA001455	Anoplophora glabripennis	Putative uncharacterized protein
EOG0912003S	APLA005174	Agrilus planipennis	Putative uncharacterized protein
EOG0912003S	TC006977	Tribolium castaneum	Putative uncharacterized protein
EOG0912003S	LDEC003365	Leptinotarsa decemlineata	Putative uncharacterized protein
EOG0912003S	YQE_11854	Dendroctonus ponderosae	Putative uncharacterized protein
EOG0912003U	OTAU001424	Onthophagus taurus	Putative uncharacterized protein
EOG0912003U	AGLA002369	Anoplophora glabripennis	Putative uncharacterized protein
EOG0912003U	APLA009945	Agrilus planipennis	Putative uncharacterized protein
EOG0912003U	TC004594	Tribolium castaneum	Putative uncharacterized protein
EOG0912003U	LDEC012577	Leptinotarsa decemlineata	Putative uncharacterized protein
EOG0912003U	YQE_02501	Dendroctonus ponderosae	Putative uncharacterized protein
EOG0912003V	OTAU005056	Onthophagus taurus	Putative uncharacterized protein
EOG0912003V	AGLA010670	Anoplophora glabripennis	Putative uncharacterized protein
EOG0912003V	APLA013662	Agrilus planipennis	Putative uncharacterized protein
EOG0912003V	TC006253	Tribolium castaneum	Putative uncharacterized protein
EOG0912003V	LDEC017235	Leptinotarsa decemlineata	Putative uncharacterized protein
EOG0912003V	YQE_12684	Dendroctonus ponderosae	Putative uncharacterized protein
EOG0912003W	OTAU002394	Onthophagus taurus	Putative uncharacterized protein
EOG0912003W	AGLA003392	Anoplophora glabripennis	Putative uncharacterized protein
EOG0912003W	APLA003345	Agrilus planipennis	Putative uncharacterized protein
EOG0912003W	TC002423	Tribolium castaneum	Putative uncharacterized protein
EOG0912003W	LDEC010044	Leptinotarsa decemlineata	Putative uncharacterized protein
EOG0912003W	YQE_07864	Dendroctonus ponderosae	Putative uncharacterized protein
EOG0912003X	OTAU013822	Onthophagus taurus	binding
EOG0912003X	AGLA016655	Anoplophora glabripennis	binding
EOG0912003X	APLA006659	Agrilus planipennis	binding
EOG0912003X	TC031024	Tribolium castaneum	binding
EOG0912003X	LDEC002678	Leptinotarsa decemlineata	binding
EOG0912003X	YQE_02281	Dendroctonus ponderosae	binding
EOG09120040	OTAU009826	Onthophagus taurus	None
EOG09120040	AGLA005961	Anoplophora glabripennis	None
EOG09120040	APLA004447	Agrilus planipennis	None
EOG09120040	TC034166	Tribolium castaneum	None
EOG09120040	LDEC011204	Leptinotarsa decemlineata	None
EOG09120040	YQE_07527	Dendroctonus ponderosae	None
EOG09120041	OTAU005419	Onthophagus taurus	Dicer-1
EOG09120041	AGLA015685	Anoplophora glabripennis	Dicer-1
EOG09120041	APLA010009	Agrilus planipennis	Dicer-1
EOG09120041	TC001750	Tribolium castaneum	Dicer-1
EOG09120041	LDEC001540	Leptinotarsa decemlineata	Dicer-1
EOG09120041	YQE_09128	Dendroctonus ponderosae	Dicer-1
EOG09120043	OTAU003106	Onthophagus taurus	Putative uncharacterized protein
EOG09120043	AGLA016097	Anoplophora glabripennis	Putative uncharacterized protein
EOG09120043	APLA002642	Agrilus planipennis	Putative uncharacterized protein
EOG09120043	TC001242	Tribolium castaneum	Putative uncharacterized protein
EOG09120043	LDEC003869	Leptinotarsa decemlineata	Putative uncharacterized protein
EOG09120043	YQE_04767	Dendroctonus ponderosae	Putative uncharacterized protein
EOG09120044	OTAU000234	Onthophagus taurus	None
EOG09120044	AGLA018947	Anoplophora glabripennis	None
EOG09120044	APLA011073	Agrilus planipennis	None
EOG09120044	TC033077	Tribolium castaneum	None
EOG09120044	LDEC013728	Leptinotarsa decemlineata	None
EOG09120044	YQE_06349	Dendroctonus ponderosae	None
EOG09120046	OTAU009900	Onthophagus taurus	Tyrosine-protein kinase receptor
EOG09120046	AGLA015534	Anoplophora glabripennis	Tyrosine-protein kinase receptor
EOG09120046	APLA002110	Agrilus planipennis	Tyrosine-protein kinase receptor
EOG09120046	TC001239	Tribolium castaneum	Tyrosine-protein kinase receptor
EOG09120046	LDEC008863	Leptinotarsa decemlineata	Tyrosine-protein kinase receptor
EOG09120046	YQE_09577	Dendroctonus ponderosae	Tyrosine-protein kinase receptor
EOG09120047	OTAU000154	Onthophagus taurus	Putative uncharacterized protein
EOG09120047	AGLA006016	Anoplophora glabripennis	Putative uncharacterized protein
EOG09120047	APLA002734	Agrilus planipennis	Putative uncharacterized protein

EOG09120047	TC014634	Tribolium castaneum	Putative uncharacterized protein
EOG09120047	LDEC004105	Leptinotarsa decemlineata	Putative uncharacterized protein
EOG09120047	YQE_03999	Dendroctonus ponderosae	Putative uncharacterized protein
EOG09120048	OTAU004312	Onthophagus taurus	Putative uncharacterized protein
EOG09120048	AGLA002209	Anoplophora glabripennis	Putative uncharacterized protein
EOG09120048	APLA013965	Agrilus planipennis	Putative uncharacterized protein
EOG09120048	TC004719	Tribolium castaneum	Putative uncharacterized protein
EOG09120048	LDEC006822	Leptinotarsa decemlineata	Putative uncharacterized protein
EOG09120048	YQE_09123	Dendroctonus ponderosae	Putative uncharacterized protein
EOG0912004A	OTAU009098	Onthophagus taurus	nucleotide binding
EOG0912004A	AGLA011074	Anoplophora glabripennis	nucleotide binding
EOG0912004A	APLA001193	Agrilus planipennis	nucleotide binding
EOG0912004A	TC034132	Tribolium castaneum	nucleotide binding
EOG0912004A	LDEC020217	Leptinotarsa decemlineata	nucleotide binding
EOG0912004A	YQE_07496	Dendroctonus ponderosae	nucleotide binding
EOG0912004C	OTAU002042	Onthophagus taurus	Putative uncharacterized protein
EOG0912004C	AGLA002600	Anoplophora glabripennis	Putative uncharacterized protein
EOG0912004C	APLA005222	Agrilus planipennis	Putative uncharacterized protein
EOG0912004C	TC008566	Tribolium castaneum	Putative uncharacterized protein
EOG0912004C	LDEC002007	Leptinotarsa decemlineata	Putative uncharacterized protein
EOG0912004C	YQE_01793	Dendroctonus ponderosae	Putative uncharacterized protein
EOG0912004E	OTAU002392	Onthophagus taurus	Putative uncharacterized protein
EOG0912004E	AGLA002025	Anoplophora glabripennis	Putative uncharacterized protein
EOG0912004E	APLA010332	Agrilus planipennis	Putative uncharacterized protein
EOG0912004E	TC006853	Tribolium castaneum	Putative uncharacterized protein
EOG0912004E	LDEC013417	Leptinotarsa decemlineata	Putative uncharacterized protein
EOG0912004E	YQE_02653	Dendroctonus ponderosae	Putative uncharacterized protein
EOG0912004J	OTAU008331	Onthophagus taurus	calcium ion binding
EOG0912004J	AGLA001368	Anoplophora glabripennis	calcium ion binding
EOG0912004J	APLA005028	Agrilus planipennis	calcium ion binding
EOG0912004J	TC032257	Tribolium castaneum	calcium ion binding
EOG0912004J	LDEC007290	Leptinotarsa decemlineata	calcium ion binding
EOG0912004J	YQE_01589	Dendroctonus ponderosae	calcium ion binding
EOG0912004M	OTAU006547	Onthophagus taurus	Putative uncharacterized protein
EOG0912004M	AGLA004649	Anoplophora glabripennis	Putative uncharacterized protein
EOG0912004M	APLA006050	Agrilus planipennis	Putative uncharacterized protein
EOG0912004M	TC002498	Tribolium castaneum	Putative uncharacterized protein
EOG0912004M	LDEC010293	Leptinotarsa decemlineata	Putative uncharacterized protein
EOG0912004M	YQE_09879	Dendroctonus ponderosae	Putative uncharacterized protein
EOG0912004V	OTAU012334	Onthophagus taurus	Putative uncharacterized protein
EOG0912004V	AGLA012377	Anoplophora glabripennis	Putative uncharacterized protein
EOG0912004V	APLA004804	Agrilus planipennis	Putative uncharacterized protein
EOG0912004V	TC014560	Tribolium castaneum	Putative uncharacterized protein
EOG0912004V	LDEC003245	Leptinotarsa decemlineata	Putative uncharacterized protein
EOG0912004V	YQE_07550	Dendroctonus ponderosae	Putative uncharacterized protein
EOG0912004W	OTAU011106	Onthophagus taurus	Putative uncharacterized protein
EOG0912004W	AGLA011577	Anoplophora glabripennis	Putative uncharacterized protein
EOG0912004W	APLA006261	Agrilus planipennis	Putative uncharacterized protein
EOG0912004W	TC000358	Tribolium castaneum	Putative uncharacterized protein
EOG0912004W	LDEC007783	Leptinotarsa decemlineata	Putative uncharacterized protein
EOG0912004W	YQE_03011	Dendroctonus ponderosae	Putative uncharacterized protein
EOG0912004Y	OTAU001504	Onthophagus taurus	Putative uncharacterized protein
EOG0912004Y	AGLA000273	Anoplophora glabripennis	Putative uncharacterized protein
EOG0912004Y	APLA003693	Agrilus planipennis	Putative uncharacterized protein
EOG0912004Y	TC004764	Tribolium castaneum	Putative uncharacterized protein
EOG0912004Y	LDEC001077	Leptinotarsa decemlineata	Putative uncharacterized protein
EOG0912004Y	YQE_06628	Dendroctonus ponderosae	Putative uncharacterized protein
EOG0912004Z	OTAU004374	Onthophagus taurus	Putative uncharacterized protein
EOG0912004Z	AGLA000322	Anoplophora glabripennis	Putative uncharacterized protein
EOG0912004Z	APLA010464	Agrilus planipennis	Putative uncharacterized protein
EOG0912004Z	TC004140	Tribolium castaneum	Putative uncharacterized protein
EOG0912004Z	LDEC020547	Leptinotarsa decemlineata	Putative uncharacterized protein
EOG0912004Z	YQE_09129	Dendroctonus ponderosae	Putative uncharacterized protein
EOG09120051	OTAU002259	Onthophagus taurus	None
EOG09120051	AGLA005541	Anoplophora glabripennis	None
EOG09120051	APLA011787	Agrilus planipennis	None
EOG09120051	TC034249	Tribolium castaneum	None

EOG09120051	LDEC021148	Leptinotarsa decemlineata	None
EOG09120051	YQE_10087	Dendroctonus ponderosae	None
EOG09120052	OTAU000610	Onthophagus taurus	Rutabaga
EOG09120052	AGLA005621	Anoplophora glabripennis	Rutabaga
EOG09120052	APLA007418	Agrilus planipennis	Rutabaga
EOG09120052	TC012082	Tribolium castaneum	Rutabaga
EOG09120052	LDEC015434	Leptinotarsa decemlineata	Rutabaga
EOG09120052	YQE_01975	Dendroctonus ponderosae	Rutabaga
EOG09120053	OTAU001697	Onthophagus taurus	Putative uncharacterized protein
EOG09120053	AGLA007278	Anoplophora glabripennis	Putative uncharacterized protein
EOG09120053	APLA007503	Agrilus planipennis	Putative uncharacterized protein
EOG09120053	TC006498	Tribolium castaneum	Putative uncharacterized protein
EOG09120053	LDEC009691	Leptinotarsa decemlineata	Putative uncharacterized protein
EOG09120053	YQE_06878	Dendroctonus ponderosae	Putative uncharacterized protein
EOG09120054	OTAU005700	Onthophagus taurus	Putative uncharacterized protein
EOG09120054	AGLA000914	Anoplophora glabripennis	Putative uncharacterized protein
EOG09120054	APLA008173	Agrilus planipennis	Putative uncharacterized protein
EOG09120054	TC009935	Tribolium castaneum	Putative uncharacterized protein
EOG09120054	LDEC009343	Leptinotarsa decemlineata	Putative uncharacterized protein
EOG09120054	YQE_05340	Dendroctonus ponderosae	Putative uncharacterized protein
EOG09120055	OTAU008526	Onthophagus taurus	Putative uncharacterized protein
EOG09120055	AGLA019729	Anoplophora glabripennis	Putative uncharacterized protein
EOG09120055	APLA002024	Agrilus planipennis	Putative uncharacterized protein
EOG09120055	TC011551	Tribolium castaneum	Putative uncharacterized protein
EOG09120055	LDEC005911	Leptinotarsa decemlineata	Putative uncharacterized protein
EOG09120055	YQE_05191	Dendroctonus ponderosae	Putative uncharacterized protein
EOG09120058	OTAU003583	Onthophagus taurus	binding
EOG09120058	AGLA006581	Anoplophora glabripennis	binding
EOG09120058	APLA007670	Agrilus planipennis	binding
EOG09120058	TC034312	Tribolium castaneum	binding
EOG09120058	LDEC021146	Leptinotarsa decemlineata	binding
EOG09120058	YQE_10022	Dendroctonus ponderosae	binding
EOG09120059	OTAU001403	Onthophagus taurus	Putative uncharacterized protein
EOG09120059	AGLA002204	Anoplophora glabripennis	Putative uncharacterized protein
EOG09120059	APLA008885	Agrilus planipennis	Putative uncharacterized protein
EOG09120059	TC004715	Tribolium castaneum	Putative uncharacterized protein
EOG09120059	LDEC020322	Leptinotarsa decemlineata	Putative uncharacterized protein
EOG09120059	YQE_09119	Dendroctonus ponderosae	Putative uncharacterized protein
EOG0912005C	OTAU010960	Onthophagus taurus	DNA-directed RNA polymerase
EOG0912005C	AGLA008589	Anoplophora glabripennis	DNA-directed RNA polymerase
EOG0912005C	APLA000113	Agrilus planipennis	DNA-directed RNA polymerase
EOG0912005C	TC010518	Tribolium castaneum	DNA-directed RNA polymerase
EOG0912005C	LDEC002687	Leptinotarsa decemlineata	DNA-directed RNA polymerase
EOG0912005C	YQE_01852	Dendroctonus ponderosae	DNA-directed RNA polymerase
EOG0912005D	OTAU008671	Onthophagus taurus	Putative uncharacterized protein
EOG0912005D	AGLA010673	Anoplophora glabripennis	Putative uncharacterized protein
EOG0912005D	APLA009092	Agrilus planipennis	Putative uncharacterized protein
EOG0912005D	TC005584	Tribolium castaneum	Putative uncharacterized protein
EOG0912005D	LDEC017240	Leptinotarsa decemlineata	Putative uncharacterized protein
EOG0912005D	YQE_05581	Dendroctonus ponderosae	Putative uncharacterized protein
EOG0912005E	OTAU005482	Onthophagus taurus	Putative uncharacterized protein
EOG0912005E	AGLA010970	Anoplophora glabripennis	Putative uncharacterized protein
EOG0912005E	APLA008294	Agrilus planipennis	Putative uncharacterized protein
EOG0912005E	TC013942	Tribolium castaneum	Putative uncharacterized protein
EOG0912005E	LDEC008916	Leptinotarsa decemlineata	Putative uncharacterized protein
EOG0912005E	YQE_07440	Dendroctonus ponderosae	Putative uncharacterized protein
EOG0912005G	OTAU001027	Onthophagus taurus	None
EOG0912005G	AGLA001332	Anoplophora glabripennis	None
EOG0912005G	APLA013031	Agrilus planipennis	None
EOG0912005G	TC031981	Tribolium castaneum	None
EOG0912005G	LDEC008036	Leptinotarsa decemlineata	None
EOG0912005G	YQE_07685	Dendroctonus ponderosae	None
EOG0912005J	OTAU013678	Onthophagus taurus	Putative uncharacterized protein
EOG0912005J	AGLA010091	Anoplophora glabripennis	Putative uncharacterized protein
EOG0912005J	APLA009827	Agrilus planipennis	Putative uncharacterized protein
EOG0912005J	TC010408	Tribolium castaneum	Putative uncharacterized protein
EOG0912005J	LDEC016181	Leptinotarsa decemlineata	Putative uncharacterized protein

EOG0912005J	YQE_02693	Dendroctonus ponderosae	Putative uncharacterized protein
EOG0912005L	OTAU006689	Onthophagus taurus	Putative uncharacterized protein
EOG0912005L	AGLA019279	Anoplophora glabripennis	Putative uncharacterized protein
EOG0912005L	APLA009708	Agrilus planipennis	Putative uncharacterized protein
EOG0912005L	TC003479	Tribolium castaneum	Putative uncharacterized protein
EOG0912005L	LDEC018282	Leptinotarsa decemlineata	Putative uncharacterized protein
EOG0912005L	YQE_02394	Dendroctonus ponderosae	Putative uncharacterized protein
EOG0912005N	OTAU005547	Onthophagus taurus	Putative uncharacterized protein
EOG0912005N	AGLA000489	Anoplophora glabripennis	Putative uncharacterized protein
EOG0912005N	APLA011028	Agrilus planipennis	Putative uncharacterized protein
EOG0912005N	TC008477	Tribolium castaneum	Putative uncharacterized protein
EOG0912005N	LDEC002405	Leptinotarsa decemlineata	Putative uncharacterized protein
EOG0912005N	YQE_02924	Dendroctonus ponderosae	Putative uncharacterized protein
EOG0912005P	OTAU009033	Onthophagus taurus	Putative uncharacterized protein
EOG0912005P	AGLA019970	Anoplophora glabripennis	Putative uncharacterized protein
EOG0912005P	APLA005251	Agrilus planipennis	Putative uncharacterized protein
EOG0912005P	TC001087	Tribolium castaneum	Putative uncharacterized protein
EOG0912005P	LDEC007340	Leptinotarsa decemlineata	Putative uncharacterized protein
EOG0912005P	YQE_09940	Dendroctonus ponderosae	Putative uncharacterized protein
EOG0912005S	OTAU005858	Onthophagus taurus	Putative uncharacterized protein
EOG0912005S	AGLA006264	Anoplophora glabripennis	Putative uncharacterized protein
EOG0912005S	APLA000761	Agrilus planipennis	Putative uncharacterized protein
EOG0912005S	TC003068	Tribolium castaneum	Putative uncharacterized protein
EOG0912005S	LDEC010611	Leptinotarsa decemlineata	Putative uncharacterized protein
EOG0912005S	YQE_01773	Dendroctonus ponderosae	Putative uncharacterized protein
EOG0912005U	OTAU004495	Onthophagus taurus	Putative uncharacterized protein
EOG0912005U	AGLA017525	Anoplophora glabripennis	Putative uncharacterized protein
EOG0912005U	APLA002064	Agrilus planipennis	Putative uncharacterized protein
EOG0912005U	TC002713	Tribolium castaneum	Putative uncharacterized protein
EOG0912005U	LDEC010279	Leptinotarsa decemlineata	Putative uncharacterized protein
EOG0912005U	YQE_10439	Dendroctonus ponderosae	Putative uncharacterized protein
EOG0912005W	OTAU003590	Onthophagus taurus	Putative uncharacterized protein
EOG0912005W	AGLA016604	Anoplophora glabripennis	Putative uncharacterized protein
EOG0912005W	APLA005463	Agrilus planipennis	Putative uncharacterized protein
EOG0912005W	TC003206	Tribolium castaneum	Putative uncharacterized protein
EOG0912005W	LDEC017718	Leptinotarsa decemlineata	Putative uncharacterized protein
EOG0912005W	YQE_11103	Dendroctonus ponderosae	Putative uncharacterized protein
EOG0912005X	OTAU001413	Onthophagus taurus	Putative uncharacterized protein
EOG0912005X	AGLA000363	Anoplophora glabripennis	Putative uncharacterized protein
EOG0912005X	APLA003490	Agrilus planipennis	Putative uncharacterized protein
EOG0912005X	TC013590	Tribolium castaneum	Putative uncharacterized protein
EOG0912005X	LDEC012177	Leptinotarsa decemlineata	Putative uncharacterized protein
EOG0912005X	YQE_09318	Dendroctonus ponderosae	Putative uncharacterized protein
EOG0912005Y	OTAU012375	Onthophagus taurus	Tollo
EOG0912005Y	AGLA006925	Anoplophora glabripennis	Tollo
EOG0912005Y	APLA007040	Agrilus planipennis	Tollo
EOG0912005Y	TC004898	Tribolium castaneum	Tollo
EOG0912005Y	LDEC016275	Leptinotarsa decemlineata	Tollo
EOG0912005Y	YQE_02320	Dendroctonus ponderosae	Tollo
EOG09120060	OTAU014160	Onthophagus taurus	None
EOG09120060	AGLA008213	Anoplophora glabripennis	None
EOG09120060	APLA001934	Agrilus planipennis	None
EOG09120060	TC032094	Tribolium castaneum	None
EOG09120060	LDEC006619	Leptinotarsa decemlineata	None
EOG09120060	YQE_11017	Dendroctonus ponderosae	None
EOG09120061	OTAU000687	Onthophagus taurus	DNA-directed RNA polymerase
EOG09120061	AGLA008126	Anoplophora glabripennis	DNA-directed RNA polymerase
EOG09120061	APLA014956	Agrilus planipennis	DNA-directed RNA polymerase
EOG09120061	TC011771	Tribolium castaneum	DNA-directed RNA polymerase
EOG09120061	LDEC016252	Leptinotarsa decemlineata	DNA-directed RNA polymerase
EOG09120061	YQE_12953	Dendroctonus ponderosae	DNA-directed RNA polymerase
EOG09120062	OTAU016444	Onthophagus taurus	Shattered
EOG09120062	AGLA011676	Anoplophora glabripennis	Shattered
EOG09120062	APLA005017	Agrilus planipennis	Shattered
EOG09120062	TC001072	Tribolium castaneum	Shattered
EOG09120062	LDEC018762	Leptinotarsa decemlineata	Shattered
EOG09120062	YQE_02930	Dendroctonus ponderosae	Shattered

EOG09120063	OTAU013246	Onthophagus taurus	Putative uncharacterized protein
EOG09120063	AGLA001591	Anoplophora glabripennis	Putative uncharacterized protein
EOG09120063	APLA008217	Agrilus planipennis	Putative uncharacterized protein
EOG09120063	TC011037	Tribolium castaneum	Putative uncharacterized protein
EOG09120063	LDEC001633	Leptinotarsa decemlineata	Putative uncharacterized protein
EOG09120063	YQE_11993	Dendroctonus ponderosae	Putative uncharacterized protein
EOG09120065	OTAU002926	Onthophagus taurus	None
EOG09120065	AGLA016176	Anoplophora glabripennis	None
EOG09120065	APLA007528	Agrilus planipennis	None
EOG09120065	TC033858	Tribolium castaneum	None
EOG09120065	LDEC016127	Leptinotarsa decemlineata	None
EOG09120065	YQE_05422	Dendroctonus ponderosae	None
EOG09120067	OTAU003515	Onthophagus taurus	Putative uncharacterized protein
EOG09120067	AGLA020486	Anoplophora glabripennis	Putative uncharacterized protein
EOG09120067	APLA014049	Agrilus planipennis	Putative uncharacterized protein
EOG09120067	TC009803	Tribolium castaneum	Putative uncharacterized protein
EOG09120067	LDEC005804	Leptinotarsa decemlineata	Putative uncharacterized protein
EOG09120067	YQE_08827	Dendroctonus ponderosae	Putative uncharacterized protein
EOG09120069	OTAU002214	Onthophagus taurus	Putative uncharacterized protein
EOG09120069	AGLA002588	Anoplophora glabripennis	Putative uncharacterized protein
EOG09120069	APLA004007	Agrilus planipennis	Putative uncharacterized protein
EOG09120069	TC012120	Tribolium castaneum	Putative uncharacterized protein
EOG09120069	LDEC002014	Leptinotarsa decemlineata	Putative uncharacterized protein
EOG09120069	YQE_06476	Dendroctonus ponderosae	Putative uncharacterized protein
EOG0912006D	OTAU013619	Onthophagus taurus	Putative uncharacterized protein
EOG0912006D	AGLA006318	Anoplophora glabripennis	Putative uncharacterized protein
EOG0912006D	APLA014530	Agrilus planipennis	Putative uncharacterized protein
EOG0912006D	TC009599	Tribolium castaneum	Putative uncharacterized protein
EOG0912006D	LDEC007557	Leptinotarsa decemlineata	Putative uncharacterized protein
EOG0912006D	YQE_11514	Dendroctonus ponderosae	Putative uncharacterized protein
EOG0912006F	OTAU001385	Onthophagus taurus	Putative uncharacterized protein
EOG0912006F	AGLA000340	Anoplophora glabripennis	Putative uncharacterized protein
EOG0912006F	APLA003657	Agrilus planipennis	Putative uncharacterized protein
EOG0912006F	TC010807	Tribolium castaneum	Putative uncharacterized protein
EOG0912006F	LDEC003751	Leptinotarsa decemlineata	Putative uncharacterized protein
EOG0912006F	YQE_09281	Dendroctonus ponderosae	Putative uncharacterized protein
EOG0912006G	OTAU005549	Onthophagus taurus	ATP binding
EOG0912006G	AGLA020361	Anoplophora glabripennis	ATP binding
EOG0912006G	APLA004030	Agrilus planipennis	ATP binding
EOG0912006G	TC033419	Tribolium castaneum	ATP binding
EOG0912006G	LDEC020748	Leptinotarsa decemlineata	ATP binding
EOG0912006G	YQE_08357	Dendroctonus ponderosae	ATP binding
EOG0912006I	OTAU001626	Onthophagus taurus	Putative uncharacterized protein
EOG0912006I	AGLA005938	Anoplophora glabripennis	Putative uncharacterized protein
EOG0912006I	APLA001178	Agrilus planipennis	Putative uncharacterized protein
EOG0912006I	TC006692	Tribolium castaneum	Putative uncharacterized protein
EOG0912006I	LDEC022157	Leptinotarsa decemlineata	Putative uncharacterized protein
EOG0912006I	YQE_05586	Dendroctonus ponderosae	Putative uncharacterized protein
EOG0912006J	OTAU008724	Onthophagus taurus	Putative uncharacterized protein
EOG0912006J	AGLA014942	Anoplophora glabripennis	Putative uncharacterized protein
EOG0912006J	APLA013354	Agrilus planipennis	Putative uncharacterized protein
EOG0912006J	TC002977	Tribolium castaneum	Putative uncharacterized protein
EOG0912006J	LDEC002047	Leptinotarsa decemlineata	Putative uncharacterized protein
EOG0912006J	YQE_10272	Dendroctonus ponderosae	Putative uncharacterized protein
EOG0912006K	OTAU006553	Onthophagus taurus	Putative uncharacterized protein
EOG0912006K	AGLA004231	Anoplophora glabripennis	Putative uncharacterized protein
EOG0912006K	APLA006655	Agrilus planipennis	Putative uncharacterized protein
EOG0912006K	TC003310	Tribolium castaneum	Putative uncharacterized protein
EOG0912006K	LDEC008587	Leptinotarsa decemlineata	Putative uncharacterized protein
EOG0912006K	YQE_12285	Dendroctonus ponderosae	Putative uncharacterized protein
EOG0912006L	OTAU002528	Onthophagus taurus	Putative uncharacterized protein
EOG0912006L	AGLA005371	Anoplophora glabripennis	Putative uncharacterized protein
EOG0912006L	APLA013059	Agrilus planipennis	Putative uncharacterized protein
EOG0912006L	TC016380	Tribolium castaneum	Putative uncharacterized protein
EOG0912006L	LDEC014895	Leptinotarsa decemlineata	Putative uncharacterized protein
EOG0912006L	YQE_12501	Dendroctonus ponderosae	Putative uncharacterized protein
EOG0912006O	OTAU005698	Onthophagus taurus	Putative uncharacterized protein

EOG0912006O	AGLA015140	Anoplophora glabripennis	Putative uncharacterized protein
EOG0912006O	APLA001751	Agrilus planipennis	Putative uncharacterized protein
EOG0912006O	TC009937	Tribolium castaneum	Putative uncharacterized protein
EOG0912006O	LDEC009345	Leptinotarsa decemlineata	Putative uncharacterized protein
EOG0912006O	YQE_05332	Dendroctonus ponderosae	Putative uncharacterized protein
EOG0912006P	OTAU003398	Onthophagus taurus	Putative uncharacterized protein
EOG0912006P	AGLA004256	Anoplophora glabripennis	Putative uncharacterized protein
EOG0912006P	APLA000094	Agrilus planipennis	Putative uncharacterized protein
EOG0912006P	TC003226	Tribolium castaneum	Putative uncharacterized protein
EOG0912006P	LDEC008205	Leptinotarsa decemlineata	Putative uncharacterized protein
EOG0912006P	YQE_07819	Dendroctonus ponderosae	Putative uncharacterized protein
EOG0912006Q	OTAU006287	Onthophagus taurus	Mini spindles
EOG0912006Q	AGLA009513	Anoplophora glabripennis	Mini spindles
EOG0912006Q	APLA010886	Agrilus planipennis	Mini spindles
EOG0912006Q	TC004968	Tribolium castaneum	Mini spindles
EOG0912006Q	LDEC010335	Leptinotarsa decemlineata	Mini spindles
EOG0912006Q	YQE_10323	Dendroctonus ponderosae	Mini spindles
EOG0912006R	OTAU001313	Onthophagus taurus	Putative uncharacterized protein
EOG0912006R	AGLA016156	Anoplophora glabripennis	Putative uncharacterized protein
EOG0912006R	APLA012813	Agrilus planipennis	Putative uncharacterized protein
EOG0912006R	TC016033	Tribolium castaneum	Putative uncharacterized protein
EOG0912006R	LDEC000565	Leptinotarsa decemlineata	Putative uncharacterized protein
EOG0912006R	YQE_04383	Dendroctonus ponderosae	Putative uncharacterized protein
EOG0912006S	OTAU008911	Onthophagus taurus	Neuroglian
EOG0912006S	AGLA004580	Anoplophora glabripennis	Neuroglian
EOG0912006S	APLA009949	Agrilus planipennis	Neuroglian
EOG0912006S	TC001889	Tribolium castaneum	Neuroglian
EOG0912006S	LDEC019714	Leptinotarsa decemlineata	Neuroglian
EOG0912006S	YQE_06683	Dendroctonus ponderosae	Neuroglian
EOG0912006T	OTAU005076	Onthophagus taurus	Putative uncharacterized protein
EOG0912006T	AGLA001650	Anoplophora glabripennis	Putative uncharacterized protein
EOG0912006T	APLA000385	Agrilus planipennis	Putative uncharacterized protein
EOG0912006T	TC005994	Tribolium castaneum	Putative uncharacterized protein
EOG0912006T	LDEC019491	Leptinotarsa decemlineata	Putative uncharacterized protein
EOG0912006T	YQE_12782	Dendroctonus ponderosae	Putative uncharacterized protein
EOG0912006U	OTAU006306	Onthophagus taurus	Toll-7-like protein
EOG0912006U	AGLA006442	Anoplophora glabripennis	Toll-7-like protein
EOG0912006U	APLA004062	Agrilus planipennis	Toll-7-like protein
EOG0912006U	TC004474	Tribolium castaneum	Toll-7-like protein
EOG0912006U	LDEC008836	Leptinotarsa decemlineata	Toll-7-like protein
EOG0912006U	YQE_02782	Dendroctonus ponderosae	Toll-7-like protein
EOG0912006W	OTAU001453	Onthophagus taurus	Putative uncharacterized protein
EOG0912006W	AGLA004515	Anoplophora glabripennis	Putative uncharacterized protein
EOG0912006W	APLA003695	Agrilus planipennis	Putative uncharacterized protein
EOG0912006W	TC010870	Tribolium castaneum	Putative uncharacterized protein
EOG0912006W	LDEC017469	Leptinotarsa decemlineata	Putative uncharacterized protein
EOG0912006W	YQE_02462	Dendroctonus ponderosae	Putative uncharacterized protein
EOG0912006Z	OTAU016469	Onthophagus taurus	Hem
EOG0912006Z	AGLA007437	Anoplophora glabripennis	Hem
EOG0912006Z	APLA005856	Agrilus planipennis	Hem
EOG0912006Z	TC001541	Tribolium castaneum	Hem
EOG0912006Z	LDEC017526	Leptinotarsa decemlineata	Hem
EOG0912006Z	YQE_11145	Dendroctonus ponderosae	Hem
EOG09120070	OTAU000515	Onthophagus taurus	Putative uncharacterized protein
EOG09120070	AGLA014920	Anoplophora glabripennis	Putative uncharacterized protein
EOG09120070	APLA010421	Agrilus planipennis	Putative uncharacterized protein
EOG09120070	TC012834	Tribolium castaneum	Putative uncharacterized protein
EOG09120070	LDEC008247	Leptinotarsa decemlineata	Putative uncharacterized protein
EOG09120070	YQE_08082	Dendroctonus ponderosae	Putative uncharacterized protein
EOG09120072	OTAU013301	Onthophagus taurus	None
EOG09120072	AGLA004353	Anoplophora glabripennis	None
EOG09120072	APLA011730	Agrilus planipennis	None
EOG09120072	TC033046	Tribolium castaneum	None
EOG09120072	LDEC002167	Leptinotarsa decemlineata	None
EOG09120072	YQE_08645	Dendroctonus ponderosae	None
EOG09120073	OTAU005593	Onthophagus taurus	Putative uncharacterized protein
EOG09120073	AGLA009191	Anoplophora glabripennis	Putative uncharacterized protein

EOG09120073	APLA006012	Agrilus planipennis	Putative uncharacterized protein
EOG09120073	TC009376	Tribolium castaneum	Putative uncharacterized protein
EOG09120073	LDEC015726	Leptinotarsa decemlineata	Putative uncharacterized protein
EOG09120073	YQE_06020	Dendroctonus ponderosae	Putative uncharacterized protein
EOG09120076	OTAU005081	Onthophagus taurus	Putative uncharacterized protein
EOG09120076	AGLA007285	Anoplophora glabripennis	Putative uncharacterized protein
EOG09120076	APLA013158	Agrilus planipennis	Putative uncharacterized protein
EOG09120076	TC005830	Tribolium castaneum	Putative uncharacterized protein
EOG09120076	LDEC016710	Leptinotarsa decemlineata	Putative uncharacterized protein
EOG09120076	YQE_09456	Dendroctonus ponderosae	Putative uncharacterized protein
EOG09120078	OTAU009884	Onthophagus taurus	Putative uncharacterized protein
EOG09120078	AGLA002996	Anoplophora glabripennis	Putative uncharacterized protein
EOG09120078	APLA011527	Agrilus planipennis	Putative uncharacterized protein
EOG09120078	TC000192	Tribolium castaneum	Putative uncharacterized protein
EOG09120078	LDEC009654	Leptinotarsa decemlineata	Putative uncharacterized protein
EOG09120078	YQE_08175	Dendroctonus ponderosae	Putative uncharacterized protein
EOG0912007B	OTAU002241	Onthophagus taurus	Putative uncharacterized protein
EOG0912007B	AGLA006603	Anoplophora glabripennis	Putative uncharacterized protein
EOG0912007B	APLA013285	Agrilus planipennis	Putative uncharacterized protein
EOG0912007B	TC012416	Tribolium castaneum	Putative uncharacterized protein
EOG0912007B	LDEC007667	Leptinotarsa decemlineata	Putative uncharacterized protein
EOG0912007B	YQE_11253	Dendroctonus ponderosae	Putative uncharacterized protein
EOG0912007C	OTAU001755	Onthophagus taurus	Putative uncharacterized protein
EOG0912007C	AGLA007335	Anoplophora glabripennis	Putative uncharacterized protein
EOG0912007C	APLA001449	Agrilus planipennis	Putative uncharacterized protein
EOG0912007C	TC005493	Tribolium castaneum	Putative uncharacterized protein
EOG0912007C	LDEC015879	Leptinotarsa decemlineata	Putative uncharacterized protein
EOG0912007C	YQE_10180	Dendroctonus ponderosae	Putative uncharacterized protein
EOG0912007D	OTAU006224	Onthophagus taurus	Putative uncharacterized protein
EOG0912007D	AGLA003663	Anoplophora glabripennis	Putative uncharacterized protein
EOG0912007D	APLA004110	Agrilus planipennis	Putative uncharacterized protein
EOG0912007D	TC003597	Tribolium castaneum	Putative uncharacterized protein
EOG0912007D	LDEC003318	Leptinotarsa decemlineata	Putative uncharacterized protein
EOG0912007D	YQE_05198	Dendroctonus ponderosae	Putative uncharacterized protein
EOG0912007E	OTAU009067	Onthophagus taurus	Putative uncharacterized protein
EOG0912007E	AGLA001772	Anoplophora glabripennis	Putative uncharacterized protein
EOG0912007E	APLA011585	Agrilus planipennis	Putative uncharacterized protein
EOG0912007E	TC013545	Tribolium castaneum	Putative uncharacterized protein
EOG0912007E	LDEC011408	Leptinotarsa decemlineata	Putative uncharacterized protein
EOG0912007E	YQE_04017	Dendroctonus ponderosae	Putative uncharacterized protein
EOG0912007G	OTAU000988	Onthophagus taurus	Putative uncharacterized protein
EOG0912007G	AGLA001318	Anoplophora glabripennis	Putative uncharacterized protein
EOG0912007G	APLA013464	Agrilus planipennis	Putative uncharacterized protein
EOG0912007G	TC004859	Tribolium castaneum	Putative uncharacterized protein
EOG0912007G	LDEC012368	Leptinotarsa decemlineata	Putative uncharacterized protein
EOG0912007G	YQE_02244	Dendroctonus ponderosae	Putative uncharacterized protein
EOG0912007I	OTAU001223	Onthophagus taurus	Putative uncharacterized protein
EOG0912007I	AGLA013517	Anoplophora glabripennis	Putative uncharacterized protein
EOG0912007I	APLA012404	Agrilus planipennis	Putative uncharacterized protein
EOG0912007I	TC001467	Tribolium castaneum	Putative uncharacterized protein
EOG0912007I	LDEC014393	Leptinotarsa decemlineata	Putative uncharacterized protein
EOG0912007I	YQE_03303	Dendroctonus ponderosae	Putative uncharacterized protein
EOG0912007J	OTAU007536	Onthophagus taurus	Tudor
EOG0912007J	AGLA010825	Anoplophora glabripennis	Tudor
EOG0912007J	APLA008360	Agrilus planipennis	Tudor
EOG0912007J	TC003753	Tribolium castaneum	Tudor
EOG0912007J	LDEC012137	Leptinotarsa decemlineata	Tudor
EOG0912007J	YQE_03886	Dendroctonus ponderosae	Tudor
EOG0912007K	OTAU007807	Onthophagus taurus	Putative uncharacterized protein
EOG0912007K	AGLA013210	Anoplophora glabripennis	Putative uncharacterized protein
EOG0912007K	APLA013456	Agrilus planipennis	Putative uncharacterized protein
EOG0912007K	TC007523	Tribolium castaneum	Putative uncharacterized protein
EOG0912007K	LDEC009604	Leptinotarsa decemlineata	Putative uncharacterized protein
EOG0912007K	YQE_02766	Dendroctonus ponderosae	Putative uncharacterized protein
EOG0912007M	OTAU000675	Onthophagus taurus	Putative uncharacterized protein
EOG0912007M	AGLA015995	Anoplophora glabripennis	Putative uncharacterized protein
EOG0912007M	APLA012619	Agrilus planipennis	Putative uncharacterized protein

EOG0912007M	TC012042	Tribolium castaneum	Putative uncharacterized protein
EOG0912007M	LDEC017021	Leptinotarsa decemlineata	Putative uncharacterized protein
EOG0912007M	YQE_07998	Dendroctonus ponderosae	Putative uncharacterized protein
EOG0912007O	OTAU004973	Onthophagus taurus	diacylglycerol binding
EOG0912007O	AGLA000093	Anoplophora glabripennis	diacylglycerol binding
EOG0912007O	APLA012559	Agrilus planipennis	diacylglycerol binding
EOG0912007O	TC031721	Tribolium castaneum	diacylglycerol binding
EOG0912007O	LDEC006616	Leptinotarsa decemlineata	diacylglycerol binding
EOG0912007O	YQE_10724	Dendroctonus ponderosae	diacylglycerol binding
EOG0912007Q	OTAU002310	Onthophagus taurus	Putative uncharacterized protein
EOG0912007Q	AGLA010145	Anoplophora glabripennis	Putative uncharacterized protein
EOG0912007Q	APLA002267	Agrilus planipennis	Putative uncharacterized protein
EOG0912007Q	TC008185	Tribolium castaneum	Putative uncharacterized protein
EOG0912007Q	LDEC012908	Leptinotarsa decemlineata	Putative uncharacterized protein
EOG0912007Q	YQE_02228	Dendroctonus ponderosae	Putative uncharacterized protein
EOG0912007R	OTAU016067	Onthophagus taurus	Putative uncharacterized protein
EOG0912007R	AGLA009862	Anoplophora glabripennis	Putative uncharacterized protein
EOG0912007R	APLA008080	Agrilus planipennis	Putative uncharacterized protein
EOG0912007R	TC014705	Tribolium castaneum	Putative uncharacterized protein
EOG0912007R	LDEC006273	Leptinotarsa decemlineata	Putative uncharacterized protein
EOG0912007R	YQE_08586	Dendroctonus ponderosae	Putative uncharacterized protein
EOG0912007S	OTAU009965	Onthophagus taurus	Putative uncharacterized protein
EOG0912007S	AGLA000834	Anoplophora glabripennis	Putative uncharacterized protein
EOG0912007S	APLA007461	Agrilus planipennis	Putative uncharacterized protein
EOG0912007S	TC008151	Tribolium castaneum	Putative uncharacterized protein
EOG0912007S	LDEC021174	Leptinotarsa decemlineata	Putative uncharacterized protein
EOG0912007S	YQE_04525	Dendroctonus ponderosae	Putative uncharacterized protein
EOG0912007U	OTAU006359	Onthophagus taurus	Structural maintenance of chromosomes protein
EOG0912007U	AGLA009059	Anoplophora glabripennis	Structural maintenance of chromosomes protein
EOG0912007U	APLA003145	Agrilus planipennis	Structural maintenance of chromosomes protein
EOG0912007U	TC011028	Tribolium castaneum	Structural maintenance of chromosomes protein
EOG0912007U	LDEC010434	Leptinotarsa decemlineata	Structural maintenance of chromosomes protein
EOG0912007U	YQE_10945	Dendroctonus ponderosae	Structural maintenance of chromosomes protein
EOG0912007V	OTAU002177	Onthophagus taurus	Putative uncharacterized protein
EOG0912007V	AGLA011379	Anoplophora glabripennis	Putative uncharacterized protein
EOG0912007V	APLA012623	Agrilus planipennis	Putative uncharacterized protein
EOG0912007V	TC012347	Tribolium castaneum	Putative uncharacterized protein
EOG0912007V	LDEC002073	Leptinotarsa decemlineata	Putative uncharacterized protein
EOG0912007V	YQE_08124	Dendroctonus ponderosae	Putative uncharacterized protein
EOG0912007W	OTAU010623	Onthophagus taurus	Bluestreak
EOG0912007W	AGLA018482	Anoplophora glabripennis	Bluestreak
EOG0912007W	APLA011083	Agrilus planipennis	Bluestreak
EOG0912007W	TC014417	Tribolium castaneum	Bluestreak
EOG0912007W	LDEC002594	Leptinotarsa decemlineata	Bluestreak
EOG0912007W	YQE_04864	Dendroctonus ponderosae	Bluestreak
EOG0912007X	OTAU009529	Onthophagus taurus	Putative uncharacterized protein
EOG0912007X	AGLA016066	Anoplophora glabripennis	Putative uncharacterized protein
EOG0912007X	APLA004750	Agrilus planipennis	Putative uncharacterized protein
EOG0912007X	TC008734	Tribolium castaneum	Putative uncharacterized protein
EOG0912007X	LDEC005820	Leptinotarsa decemlineata	Putative uncharacterized protein
EOG0912007X	YQE_11332	Dendroctonus ponderosae	Putative uncharacterized protein
EOG0912007Y	OTAU002084	Onthophagus taurus	Guanylate cyclase
EOG0912007Y	AGLA003296	Anoplophora glabripennis	Guanylate cyclase
EOG0912007Y	APLA001123	Agrilus planipennis	Guanylate cyclase
EOG0912007Y	TC012843	Tribolium castaneum	Guanylate cyclase
EOG0912007Y	LDEC013566	Leptinotarsa decemlineata	Guanylate cyclase
EOG0912007Y	YQE_04355	Dendroctonus ponderosae	Guanylate cyclase
EOG09120080	OTAU001987	Onthophagus taurus	ion channel activity
EOG09120080	AGLA002495	Anoplophora glabripennis	ion channel activity
EOG09120080	APLA001465	Agrilus planipennis	ion channel activity
EOG09120080	TC034186	Tribolium castaneum	ion channel activity
EOG09120080	LDEC005858	Leptinotarsa decemlineata	ion channel activity
EOG09120080	YQE_12005	Dendroctonus ponderosae	ion channel activity
EOG09120081	OTAU007166	Onthophagus taurus	metal ion binding
EOG09120081	AGLA015906	Anoplophora glabripennis	metal ion binding
EOG09120081	APLA000588	Agrilus planipennis	metal ion binding
EOG09120081	TC033414	Tribolium castaneum	metal ion binding

EOG09120081	LDEC009932	Leptinotarsa decemlineata	metal ion binding
EOG09120081	YQE_11711	Dendroctonus ponderosae	metal ion binding
EOG09120086	OTAU010886	Onthophagus taurus	DNA topoisomerase 2
EOG09120086	AGLA001518	Anoplophora glabripennis	DNA topoisomerase 2
EOG09120086	APLA004984	Agrilus planipennis	DNA topoisomerase 2
EOG09120086	TC003811	Tribolium castaneum	DNA topoisomerase 2
EOG09120086	LDEC004658	Leptinotarsa decemlineata	DNA topoisomerase 2
EOG09120086	YQE_06745	Dendroctonus ponderosae	DNA topoisomerase 2
EOG0912008A	OTAU004681	Onthophagus taurus	Putative uncharacterized protein
EOG0912008A	AGLA005527	Anoplophora glabripennis	Putative uncharacterized protein
EOG0912008A	APLA014679	Agrilus planipennis	Putative uncharacterized protein
EOG0912008A	TC012162	Tribolium castaneum	Putative uncharacterized protein
EOG0912008A	LDEC011147	Leptinotarsa decemlineata	Putative uncharacterized protein
EOG0912008A	YQE_05513	Dendroctonus ponderosae	Putative uncharacterized protein
EOG0912008D	OTAU010198	Onthophagus taurus	Putative uncharacterized protein
EOG0912008D	AGLA005482	Anoplophora glabripennis	Putative uncharacterized protein
EOG0912008D	APLA002482	Agrilus planipennis	Putative uncharacterized protein
EOG0912008D	TC014664	Tribolium castaneum	Putative uncharacterized protein
EOG0912008D	LDEC003068	Leptinotarsa decemlineata	Putative uncharacterized protein
EOG0912008D	YQE_04876	Dendroctonus ponderosae	Putative uncharacterized protein
EOG0912008E	OTAU011462	Onthophagus taurus	Putative uncharacterized protein
EOG0912008E	AGLA012345	Anoplophora glabripennis	Putative uncharacterized protein
EOG0912008E	APLA001660	Agrilus planipennis	Putative uncharacterized protein
EOG0912008E	TC010010	Tribolium castaneum	Putative uncharacterized protein
EOG0912008E	LDEC010663	Leptinotarsa decemlineata	Putative uncharacterized protein
EOG0912008E	YQE_05832	Dendroctonus ponderosae	Putative uncharacterized protein
EOG0912008G	OTAU000806	Onthophagus taurus	Putative uncharacterized protein
EOG0912008G	AGLA002441	Anoplophora glabripennis	Putative uncharacterized protein
EOG0912008G	APLA001971	Agrilus planipennis	Putative uncharacterized protein
EOG0912008G	TC011252	Tribolium castaneum	Putative uncharacterized protein
EOG0912008G	LDEC019594	Leptinotarsa decemlineata	Putative uncharacterized protein
EOG0912008G	YQE_08179	Dendroctonus ponderosae	Putative uncharacterized protein
EOG0912008H	OTAU014545	Onthophagus taurus	Putative uncharacterized protein
EOG0912008H	AGLA004514	Anoplophora glabripennis	Putative uncharacterized protein
EOG0912008H	APLA009673	Agrilus planipennis	Putative uncharacterized protein
EOG0912008H	TC013627	Tribolium castaneum	Putative uncharacterized protein
EOG0912008H	LDEC019403	Leptinotarsa decemlineata	Putative uncharacterized protein
EOG0912008H	YQE_02463	Dendroctonus ponderosae	Putative uncharacterized protein
EOG0912008I	OTAU011899	Onthophagus taurus	None
EOG0912008I	AGLA008723	Anoplophora glabripennis	None
EOG0912008I	APLA001667	Agrilus planipennis	None
EOG0912008I	TC033811	Tribolium castaneum	None
EOG0912008I	LDEC021303	Leptinotarsa decemlineata	None
EOG0912008I	YQE_05734	Dendroctonus ponderosae	None
EOG0912008J	OTAU014356	Onthophagus taurus	Putative uncharacterized protein
EOG0912008J	AGLA008223	Anoplophora glabripennis	Putative uncharacterized protein
EOG0912008J	APLA012530	Agrilus planipennis	Putative uncharacterized protein
EOG0912008J	TC007848	Tribolium castaneum	Putative uncharacterized protein
EOG0912008J	LDEC003658	Leptinotarsa decemlineata	Putative uncharacterized protein
EOG0912008J	YQE_06853	Dendroctonus ponderosae	Putative uncharacterized protein
EOG0912008K	OTAU001227	Onthophagus taurus	Putative uncharacterized protein
EOG0912008K	AGLA002170	Anoplophora glabripennis	Putative uncharacterized protein
EOG0912008K	APLA013633	Agrilus planipennis	Putative uncharacterized protein
EOG0912008K	TC010805	Tribolium castaneum	Putative uncharacterized protein
EOG0912008K	LDEC001517	Leptinotarsa decemlineata	Putative uncharacterized protein
EOG0912008K	YQE_02473	Dendroctonus ponderosae	Putative uncharacterized protein
EOG0912008L	OTAU002904	Onthophagus taurus	acid phosphatase activity
EOG0912008L	AGLA005848	Anoplophora glabripennis	acid phosphatase activity
EOG0912008L	APLA001574	Agrilus planipennis	acid phosphatase activity
EOG0912008L	TC033834	Tribolium castaneum	acid phosphatase activity
EOG0912008L	LDEC011900	Leptinotarsa decemlineata	acid phosphatase activity
EOG0912008L	YQE_05262	Dendroctonus ponderosae	acid phosphatase activity
EOG0912008N	OTAU011801	Onthophagus taurus	Putative uncharacterized protein
EOG0912008N	AGLA001665	Anoplophora glabripennis	Putative uncharacterized protein
EOG0912008N	APLA000489	Agrilus planipennis	Putative uncharacterized protein
EOG0912008N	TC006063	Tribolium castaneum	Putative uncharacterized protein
EOG0912008N	LDEC011195	Leptinotarsa decemlineata	Putative uncharacterized protein

EOG0912008N	YQE_12771	Dendroctonus ponderosae	Putative uncharacterized protein
EOG0912008P	OTAU007058	Onthophagus taurus	Peroxidasin
EOG0912008P	AGLA009149	Anoplophora glabripennis	Peroxidasin
EOG0912008P	APLA014196	Agrilus planipennis	Peroxidasin
EOG0912008P	TC001556	Tribolium castaneum	Peroxidasin
EOG0912008P	LDEC023107	Leptinotarsa decemlineata	Peroxidasin
EOG0912008P	YQE_10222	Dendroctonus ponderosae	Peroxidasin
EOG0912008R	OTAU011258	Onthophagus taurus	Putative uncharacterized protein
EOG0912008R	AGLA001528	Anoplophora glabripennis	Putative uncharacterized protein
EOG0912008R	APLA000705	Agrilus planipennis	Putative uncharacterized protein
EOG0912008R	TC003942	Tribolium castaneum	Putative uncharacterized protein
EOG0912008R	LDEC011115	Leptinotarsa decemlineata	Putative uncharacterized protein
EOG0912008R	YQE_03712	Dendroctonus ponderosae	Putative uncharacterized protein
EOG0912008T	OTAU009713	Onthophagus taurus	hydrolase activity
EOG0912008T	AGLA005859	Anoplophora glabripennis	hydrolase activity
EOG0912008T	APLA001638	Agrilus planipennis	hydrolase activity
EOG0912008T	TC033838	Tribolium castaneum	hydrolase activity
EOG0912008T	LDEC013268	Leptinotarsa decemlineata	hydrolase activity
EOG0912008T	YQE_11557	Dendroctonus ponderosae	hydrolase activity
EOG0912008W	OTAU007194	Onthophagus taurus	Ubiquitin carboxyl-terminal hydrolase
EOG0912008W	AGLA003954	Anoplophora glabripennis	Ubiquitin carboxyl-terminal hydrolase
EOG0912008W	APLA002584	Agrilus planipennis	Ubiquitin carboxyl-terminal hydrolase
EOG0912008W	TC033410	Tribolium castaneum	Ubiquitin carboxyl-terminal hydrolase
EOG0912008W	LDEC019674	Leptinotarsa decemlineata	Ubiquitin carboxyl-terminal hydrolase
EOG0912008W	YQE_11715	Dendroctonus ponderosae	Ubiquitin carboxyl-terminal hydrolase
EOG09120090	OTAU003627	Onthophagus taurus	calcium ion binding
EOG09120090	AGLA011235	Anoplophora glabripennis	calcium ion binding
EOG09120090	APLA001485	Agrilus planipennis	calcium ion binding
EOG09120090	TC032394	Tribolium castaneum	calcium ion binding
EOG09120090	LDEC003269	Leptinotarsa decemlineata	calcium ion binding
EOG09120090	YQE_12338	Dendroctonus ponderosae	calcium ion binding
EOG09120093	OTAU007777	Onthophagus taurus	Putative uncharacterized protein
EOG09120093	AGLA016003	Anoplophora glabripennis	Putative uncharacterized protein
EOG09120093	APLA006963	Agrilus planipennis	Putative uncharacterized protein
EOG09120093	TC009974	Tribolium castaneum	Putative uncharacterized protein
EOG09120093	LDEC008731	Leptinotarsa decemlineata	Putative uncharacterized protein
EOG09120093	YQE_04067	Dendroctonus ponderosae	Putative uncharacterized protein
EOG09120094	OTAU001456	Onthophagus taurus	Putative uncharacterized protein
EOG09120094	AGLA000306	Anoplophora glabripennis	Putative uncharacterized protein
EOG09120094	APLA010274	Agrilus planipennis	Putative uncharacterized protein
EOG09120094	TC004637	Tribolium castaneum	Putative uncharacterized protein
EOG09120094	LDEC003870	Leptinotarsa decemlineata	Putative uncharacterized protein
EOG09120094	YQE_06668	Dendroctonus ponderosae	Putative uncharacterized protein
EOG09120096	OTAU011763	Onthophagus taurus	Putative uncharacterized protein
EOG09120096	AGLA002818	Anoplophora glabripennis	Putative uncharacterized protein
EOG09120096	APLA005701	Agrilus planipennis	Putative uncharacterized protein
EOG09120096	TC009783	Tribolium castaneum	Putative uncharacterized protein
EOG09120096	LDEC009976	Leptinotarsa decemlineata	Putative uncharacterized protein
EOG09120096	YQE_02193	Dendroctonus ponderosae	Putative uncharacterized protein
EOG09120097	OTAU000588	Onthophagus taurus	Putative uncharacterized protein
EOG09120097	AGLA007751	Anoplophora glabripennis	Putative uncharacterized protein
EOG09120097	APLA009371	Agrilus planipennis	Putative uncharacterized protein
EOG09120097	TC003916	Tribolium castaneum	Putative uncharacterized protein
EOG09120097	LDEC004009	Leptinotarsa decemlineata	Putative uncharacterized protein
EOG09120097	YQE_12672	Dendroctonus ponderosae	Putative uncharacterized protein
EOG09120098	OTAU008811	Onthophagus taurus	galactosyltransferase activity
EOG09120098	AGLA002766	Anoplophora glabripennis	galactosyltransferase activity
EOG09120098	APLA014921	Agrilus planipennis	galactosyltransferase activity
EOG09120098	TC033521	Tribolium castaneum	galactosyltransferase activity
EOG09120098	LDEC016619	Leptinotarsa decemlineata	galactosyltransferase activity
EOG09120098	YQE_12467	Dendroctonus ponderosae	galactosyltransferase activity
EOG0912009A	OTAU011036	Onthophagus taurus	Stromalin
EOG0912009A	AGLA006384	Anoplophora glabripennis	Stromalin
EOG0912009A	APLA008284	Agrilus planipennis	Stromalin
EOG0912009A	TC014340	Tribolium castaneum	Stromalin
EOG0912009A	LDEC011741	Leptinotarsa decemlineata	Stromalin
EOG0912009A	YQE_04038	Dendroctonus ponderosae	Stromalin

EOG0912009B	OTAU001235	Onthophagus taurus	serine-type endopeptidase activity
EOG0912009B	AGLA000315	Anoplophora glabripennis	serine-type endopeptidase activity
EOG0912009B	APLA009482	Agrilus planipennis	serine-type endopeptidase activity
EOG0912009B	TC034512	Tribolium castaneum	serine-type endopeptidase activity
EOG0912009B	LDEC022911	Leptinotarsa decemlineata	serine-type endopeptidase activity
EOG0912009B	YQE_09064	Dendroctonus ponderosae	serine-type endopeptidase activity
EOG0912009C	OTAU000902	Onthophagus taurus	Brahma
EOG0912009C	AGLA005638	Anoplophora glabripennis	Brahma
EOG0912009C	APLA008243	Agrilus planipennis	Brahma
EOG0912009C	TC011073	Tribolium castaneum	Brahma
EOG0912009C	LDEC007520	Leptinotarsa decemlineata	Brahma
EOG0912009C	YQE_02801	Dendroctonus ponderosae	Brahma
EOG0912009D	OTAU007464	Onthophagus taurus	None
EOG0912009D	AGLA013471	Anoplophora glabripennis	None
EOG0912009D	APLA000558	Agrilus planipennis	None
EOG0912009D	TC034726	Tribolium castaneum	None
EOG0912009D	LDEC007937	Leptinotarsa decemlineata	None
EOG0912009D	YQE_09388	Dendroctonus ponderosae	None
EOG0912009G	OTAU002731	Onthophagus taurus	None
EOG0912009G	AGLA000213	Anoplophora glabripennis	None
EOG0912009G	APLA010012	Agrilus planipennis	None
EOG0912009G	TC031494	Tribolium castaneum	None
EOG0912009G	LDEC004511	Leptinotarsa decemlineata	None
EOG0912009G	YQE_09086	Dendroctonus ponderosae	None
EOG0912009I	OTAU010713	Onthophagus taurus	Coronin
EOG0912009I	AGLA010036	Anoplophora glabripennis	Coronin
EOG0912009I	APLA010299	Agrilus planipennis	Coronin
EOG0912009I	TC008045	Tribolium castaneum	Coronin
EOG0912009I	LDEC000715	Leptinotarsa decemlineata	Coronin
EOG0912009I	YQE_08702	Dendroctonus ponderosae	Coronin
EOG0912009J	OTAU008049	Onthophagus taurus	Putative uncharacterized protein
EOG0912009J	AGLA014889	Anoplophora glabripennis	Putative uncharacterized protein
EOG0912009J	APLA001565	Agrilus planipennis	Putative uncharacterized protein
EOG0912009J	TC009943	Tribolium castaneum	Putative uncharacterized protein
EOG0912009J	LDEC011845	Leptinotarsa decemlineata	Putative uncharacterized protein
EOG0912009J	YQE_03499	Dendroctonus ponderosae	Putative uncharacterized protein
EOG0912009Q	OTAU003579	Onthophagus taurus	Putative uncharacterized protein
EOG0912009Q	AGLA005676	Anoplophora glabripennis	Putative uncharacterized protein
EOG0912009Q	APLA002386	Agrilus planipennis	Putative uncharacterized protein
EOG0912009Q	TC011774	Tribolium castaneum	Putative uncharacterized protein
EOG0912009Q	LDEC021393	Leptinotarsa decemlineata	Putative uncharacterized protein
EOG0912009Q	YQE_03442	Dendroctonus ponderosae	Putative uncharacterized protein
EOG0912009R	OTAU010794	Onthophagus taurus	Putative uncharacterized protein
EOG0912009R	AGLA009182	Anoplophora glabripennis	Putative uncharacterized protein
EOG0912009R	APLA013215	Agrilus planipennis	Putative uncharacterized protein
EOG0912009R	TC009560	Tribolium castaneum	Putative uncharacterized protein
EOG0912009R	LDEC015716	Leptinotarsa decemlineata	Putative uncharacterized protein
EOG0912009R	YQE_06036	Dendroctonus ponderosae	Putative uncharacterized protein
EOG0912009S	OTAU003702	Onthophagus taurus	Putative uncharacterized protein
EOG0912009S	AGLA003742	Anoplophora glabripennis	Putative uncharacterized protein
EOG0912009S	APLA006605	Agrilus planipennis	Putative uncharacterized protein
EOG0912009S	TC007813	Tribolium castaneum	Putative uncharacterized protein
EOG0912009S	LDEC011496	Leptinotarsa decemlineata	Putative uncharacterized protein
EOG0912009S	YQE_01499	Dendroctonus ponderosae	Putative uncharacterized protein
EOG0912009T	OTAU009473	Onthophagus taurus	Putative uncharacterized protein
EOG0912009T	AGLA002514	Anoplophora glabripennis	Putative uncharacterized protein
EOG0912009T	APLA000699	Agrilus planipennis	Putative uncharacterized protein
EOG0912009T	TC006019	Tribolium castaneum	Putative uncharacterized protein
EOG0912009T	LDEC003620	Leptinotarsa decemlineata	Putative uncharacterized protein
EOG0912009T	YQE_06530	Dendroctonus ponderosae	Putative uncharacterized protein
EOG0912009U	OTAU013032	Onthophagus taurus	Putative uncharacterized protein
EOG0912009U	AGLA003323	Anoplophora glabripennis	Putative uncharacterized protein
EOG0912009U	APLA001878	Agrilus planipennis	Putative uncharacterized protein
EOG0912009U	TC015096	Tribolium castaneum	Putative uncharacterized protein
EOG0912009U	LDEC007893	Leptinotarsa decemlineata	Putative uncharacterized protein
EOG0912009U	YQE_09418	Dendroctonus ponderosae	Putative uncharacterized protein
EOG0912009Y	OTAU010503	Onthophagus taurus	Putative uncharacterized protein

EOG0912009Y	AGLA014909	Anoplophora glabripennis	Putative uncharacterized protein
EOG0912009Y	APLA000183	Agrilus planipennis	Putative uncharacterized protein
EOG0912009Y	TC003397	Tribolium castaneum	Putative uncharacterized protein
EOG0912009Y	LDEC005918	Leptinotarsa decemlineata	Putative uncharacterized protein
EOG0912009Y	YQE_10868	Dendroctonus ponderosae	Putative uncharacterized protein
EOG091200A0	OTAU001442	Onthophagus taurus	Putative uncharacterized protein
EOG091200A0	AGLA001142	Anoplophora glabripennis	Putative uncharacterized protein
EOG091200A0	APLA001445	Agrilus planipennis	Putative uncharacterized protein
EOG091200A0	TC004712	Tribolium castaneum	Putative uncharacterized protein
EOG091200A0	LDEC004524	Leptinotarsa decemlineata	Putative uncharacterized protein
EOG091200A0	YQE_02479	Dendroctonus ponderosae	Putative uncharacterized protein
EOG091200A2	OTAU006865	Onthophagus taurus	Putative uncharacterized protein
EOG091200A2	AGLA011723	Anoplophora glabripennis	Putative uncharacterized protein
EOG091200A2	APLA000831	Agrilus planipennis	Putative uncharacterized protein
EOG091200A2	TC013052	Tribolium castaneum	Putative uncharacterized protein
EOG091200A2	LDEC017506	Leptinotarsa decemlineata	Putative uncharacterized protein
EOG091200A2	YQE_07227	Dendroctonus ponderosae	Putative uncharacterized protein
EOG091200A4	OTAU014737	Onthophagus taurus	Putative uncharacterized protein
EOG091200A4	AGLA014991	Anoplophora glabripennis	Putative uncharacterized protein
EOG091200A4	APLA014160	Agrilus planipennis	Putative uncharacterized protein
EOG091200A4	TC006051	Tribolium castaneum	Putative uncharacterized protein
EOG091200A4	LDEC015310	Leptinotarsa decemlineata	Putative uncharacterized protein
EOG091200A4	YQE_11718	Dendroctonus ponderosae	Putative uncharacterized protein
EOG091200A5	OTAU005959	Onthophagus taurus	Putative uncharacterized protein
EOG091200A5	AGLA009531	Anoplophora glabripennis	Putative uncharacterized protein
EOG091200A5	APLA010292	Agrilus planipennis	Putative uncharacterized protein
EOG091200A5	TC007529	Tribolium castaneum	Putative uncharacterized protein
EOG091200A5	LDEC021947	Leptinotarsa decemlineata	Putative uncharacterized protein
EOG091200A5	YQE_08968	Dendroctonus ponderosae	Putative uncharacterized protein
EOG091200A6	OTAU001851	Onthophagus taurus	Putative uncharacterized protein
EOG091200A6	AGLA007297	Anoplophora glabripennis	Putative uncharacterized protein
EOG091200A6	APLA009758	Agrilus planipennis	Putative uncharacterized protein
EOG091200A6	TC006548	Tribolium castaneum	Putative uncharacterized protein
EOG091200A6	LDEC010129	Leptinotarsa decemlineata	Putative uncharacterized protein
EOG091200A6	YQE_12453	Dendroctonus ponderosae	Putative uncharacterized protein
EOG091200A7	OTAU003370	Onthophagus taurus	Putative uncharacterized protein
EOG091200A7	AGLA007743	Anoplophora glabripennis	Putative uncharacterized protein
EOG091200A7	APLA011370	Agrilus planipennis	Putative uncharacterized protein
EOG091200A7	TC003911	Tribolium castaneum	Putative uncharacterized protein
EOG091200A7	LDEC004021	Leptinotarsa decemlineata	Putative uncharacterized protein
EOG091200A7	YQE_09875	Dendroctonus ponderosae	Putative uncharacterized protein
EOG091200A8	OTAU007594	Onthophagus taurus	Putative uncharacterized protein
EOG091200A8	AGLA007516	Anoplophora glabripennis	Putative uncharacterized protein
EOG091200A8	APLA000141	Agrilus planipennis	Putative uncharacterized protein
EOG091200A8	TC003212	Tribolium castaneum	Putative uncharacterized protein
EOG091200A8	LDEC013846	Leptinotarsa decemlineata	Putative uncharacterized protein
EOG091200A8	YQE_07812	Dendroctonus ponderosae	Putative uncharacterized protein
EOG091200A9	OTAU014238	Onthophagus taurus	Putative uncharacterized protein
EOG091200A9	AGLA015002	Anoplophora glabripennis	Putative uncharacterized protein
EOG091200A9	APLA010424	Agrilus planipennis	Putative uncharacterized protein
EOG091200A9	TC012433	Tribolium castaneum	Putative uncharacterized protein
EOG091200A9	LDEC011812	Leptinotarsa decemlineata	Putative uncharacterized protein
EOG091200A9	YQE_08502	Dendroctonus ponderosae	Putative uncharacterized protein
EOG091200AA	OTAU009703	Onthophagus taurus	Putative uncharacterized protein
EOG091200AA	AGLA000790	Anoplophora glabripennis	Putative uncharacterized protein
EOG091200AA	APLA006584	Agrilus planipennis	Putative uncharacterized protein
EOG091200AA	TC007248	Tribolium castaneum	Putative uncharacterized protein
EOG091200AA	LDEC004393	Leptinotarsa decemlineata	Putative uncharacterized protein
EOG091200AA	YQE_07650	Dendroctonus ponderosae	Putative uncharacterized protein
EOG091200AB	OTAU001280	Onthophagus taurus	Putative uncharacterized protein
EOG091200AB	AGLA004526	Anoplophora glabripennis	Putative uncharacterized protein
EOG091200AB	APLA008115	Agrilus planipennis	Putative uncharacterized protein
EOG091200AB	TC010798	Tribolium castaneum	Putative uncharacterized protein
EOG091200AB	LDEC001524	Leptinotarsa decemlineata	Putative uncharacterized protein
EOG091200AB	YQE_09113	Dendroctonus ponderosae	Putative uncharacterized protein
EOG091200AC	OTAU000519	Onthophagus taurus	Putative uncharacterized protein
EOG091200AC	AGLA019247	Anoplophora glabripennis	Putative uncharacterized protein

EOG091200AC	APLA012467	Agrilus planipennis	Putative uncharacterized protein
EOG091200AC	TC012671	Tribolium castaneum	Putative uncharacterized protein
EOG091200AC	LDEC000483	Leptinotarsa decemlineata	Putative uncharacterized protein
EOG091200AC	YQE_02891	Dendroctonus ponderosae	Putative uncharacterized protein
EOG091200AD	OTAU013430	Onthophagus taurus	Putative uncharacterized protein
EOG091200AD	AGLA016911	Anoplophora glabripennis	Putative uncharacterized protein
EOG091200AD	APLA013591	Agrilus planipennis	Putative uncharacterized protein
EOG091200AD	TC002791	Tribolium castaneum	Putative uncharacterized protein
EOG091200AD	LDEC000223	Leptinotarsa decemlineata	Putative uncharacterized protein
EOG091200AD	YQE_11092	Dendroctonus ponderosae	Putative uncharacterized protein
EOG091200AE	OTAU005125	Onthophagus taurus	helicase activity
EOG091200AE	AGLA006473	Anoplophora glabripennis	helicase activity
EOG091200AE	APLA011386	Agrilus planipennis	helicase activity
EOG091200AE	TC032628	Tribolium castaneum	helicase activity
EOG091200AE	LDEC020274	Leptinotarsa decemlineata	helicase activity
EOG091200AE	YQE_12785	Dendroctonus ponderosae	helicase activity
EOG091200AF	OTAU001618	Onthophagus taurus	None
EOG091200AF	AGLA002488	Anoplophora glabripennis	None
EOG091200AF	APLA005467	Agrilus planipennis	None
EOG091200AF	TC034180	Tribolium castaneum	None
EOG091200AF	LDEC018125	Leptinotarsa decemlineata	None
EOG091200AF	YQE_07512	Dendroctonus ponderosae	None
EOG091200AG	OTAU005152	Onthophagus taurus	Putative uncharacterized protein
EOG091200AG	AGLA007484	Anoplophora glabripennis	Putative uncharacterized protein
EOG091200AG	APLA012550	Agrilus planipennis	Putative uncharacterized protein
EOG091200AG	TC008407	Tribolium castaneum	Putative uncharacterized protein
EOG091200AG	LDEC006484	Leptinotarsa decemlineata	Putative uncharacterized protein
EOG091200AG	YQE_04170	Dendroctonus ponderosae	Putative uncharacterized protein
EOG091200AH	OTAU002964	Onthophagus taurus	ligase activity
EOG091200AH	AGLA003856	Anoplophora glabripennis	ligase activity
EOG091200AH	APLA008833	Agrilus planipennis	ligase activity
EOG091200AH	TC033636	Tribolium castaneum	ligase activity
EOG091200AH	LDEC020034	Leptinotarsa decemlineata	ligase activity
EOG091200AH	YQE_06011	Dendroctonus ponderosae	ligase activity
EOG091200AO	OTAU014493	Onthophagus taurus	Histone H2A
EOG091200AO	AGLA003908	Anoplophora glabripennis	Histone H2A
EOG091200AO	APLA014712	Agrilus planipennis	Histone H2A
EOG091200AO	TC015124	Tribolium castaneum	Histone H2A
EOG091200AO	LDEC002346	Leptinotarsa decemlineata	Histone H2A
EOG091200AO	YQE_06523	Dendroctonus ponderosae	Histone H2A
EOG091200AQ	OTAU006845	Onthophagus taurus	Putative uncharacterized protein
EOG091200AQ	AGLA016805	Anoplophora glabripennis	Putative uncharacterized protein
EOG091200AQ	APLA003091	Agrilus planipennis	Putative uncharacterized protein
EOG091200AQ	TC030624	Tribolium castaneum	Putative uncharacterized protein
EOG091200AQ	LDEC014486	Leptinotarsa decemlineata	Putative uncharacterized protein
EOG091200AQ	YQE_07184	Dendroctonus ponderosae	Putative uncharacterized protein
EOG091200AR	OTAU008964	Onthophagus taurus	Putative uncharacterized protein
EOG091200AR	AGLA006778	Anoplophora glabripennis	Putative uncharacterized protein
EOG091200AR	APLA014442	Agrilus planipennis	Putative uncharacterized protein
EOG091200AR	TC004220	Tribolium castaneum	Putative uncharacterized protein
EOG091200AR	LDEC011544	Leptinotarsa decemlineata	Putative uncharacterized protein
EOG091200AR	YQE_05906	Dendroctonus ponderosae	Putative uncharacterized protein
EOG091200AS	OTAU004339	Onthophagus taurus	Putative uncharacterized protein
EOG091200AS	AGLA013525	Anoplophora glabripennis	Putative uncharacterized protein
EOG091200AS	APLA014539	Agrilus planipennis	Putative uncharacterized protein
EOG091200AS	TC013593	Tribolium castaneum	Putative uncharacterized protein
EOG091200AS	LDEC001014	Leptinotarsa decemlineata	Putative uncharacterized protein
EOG091200AS	YQE_09303	Dendroctonus ponderosae	Putative uncharacterized protein
EOG091200AV	OTAU011125	Onthophagus taurus	Putative uncharacterized protein
EOG091200AV	AGLA017323	Anoplophora glabripennis	Putative uncharacterized protein
EOG091200AV	APLA004867	Agrilus planipennis	Putative uncharacterized protein
EOG091200AV	TC013456	Tribolium castaneum	Putative uncharacterized protein
EOG091200AV	LDEC003462	Leptinotarsa decemlineata	Putative uncharacterized protein
EOG091200AV	YQE_08991	Dendroctonus ponderosae	Putative uncharacterized protein
EOG091200AX	OTAU002137	Onthophagus taurus	Putative uncharacterized protein
EOG091200AX	AGLA020047	Anoplophora glabripennis	Putative uncharacterized protein
EOG091200AX	APLA000286	Agrilus planipennis	Putative uncharacterized protein

EOG091200AX	TC003560	Tribolium castaneum	Putative uncharacterized protein
EOG091200AX	LDEC019723	Leptinotarsa decemlineata	Putative uncharacterized protein
EOG091200AX	YQE_07839	Dendroctonus ponderosae	Putative uncharacterized protein
EOG091200B1	OTAU001209	Onthophagus taurus	Putative uncharacterized protein
EOG091200B1	AGLA000084	Anoplophora glabripennis	Putative uncharacterized protein
EOG091200B1	APLA007698	Agrilus planipennis	Putative uncharacterized protein
EOG091200B1	TC002074	Tribolium castaneum	Putative uncharacterized protein
EOG091200B1	LDEC014176	Leptinotarsa decemlineata	Putative uncharacterized protein
EOG091200B1	YQE_10324	Dendroctonus ponderosae	Putative uncharacterized protein
EOG091200B4	OTAU000148	Onthophagus taurus	Putative uncharacterized protein
EOG091200B4	AGLA012790	Anoplophora glabripennis	Putative uncharacterized protein
EOG091200B4	APLA008533	Agrilus planipennis	Putative uncharacterized protein
EOG091200B4	TC014794	Tribolium castaneum	Putative uncharacterized protein
EOG091200B4	LDEC016468	Leptinotarsa decemlineata	Putative uncharacterized protein
EOG091200B4	YQE_03602	Dendroctonus ponderosae	Putative uncharacterized protein
EOG091200B5	OTAU006619	Onthophagus taurus	ligase activity
EOG091200B5	AGLA014915	Anoplophora glabripennis	ligase activity
EOG091200B5	APLA000168	Agrilus planipennis	ligase activity
EOG091200B5	TC031473	Tribolium castaneum	ligase activity
EOG091200B5	LDEC005909	Leptinotarsa decemlineata	ligase activity
EOG091200B5	YQE_10832	Dendroctonus ponderosae	ligase activity
EOG091200B8	OTAU001355	Onthophagus taurus	Putative uncharacterized protein
EOG091200B8	AGLA007610	Anoplophora glabripennis	Putative uncharacterized protein
EOG091200B8	APLA003686	Agrilus planipennis	Putative uncharacterized protein
EOG091200B8	TC001759	Tribolium castaneum	Putative uncharacterized protein
EOG091200B8	LDEC006806	Leptinotarsa decemlineata	Putative uncharacterized protein
EOG091200B8	YQE_09137	Dendroctonus ponderosae	Putative uncharacterized protein
EOG091200B9	OTAU003606	Onthophagus taurus	Putative uncharacterized protein
EOG091200B9	AGLA012572	Anoplophora glabripennis	Putative uncharacterized protein
EOG091200B9	APLA014649	Agrilus planipennis	Putative uncharacterized protein
EOG091200B9	TC000124	Tribolium castaneum	Putative uncharacterized protein
EOG091200B9	LDEC005037	Leptinotarsa decemlineata	Putative uncharacterized protein
EOG091200B9	YQE_03695	Dendroctonus ponderosae	Putative uncharacterized protein
EOG091200BB	OTAU001414	Onthophagus taurus	Putative uncharacterized protein
EOG091200BB	AGLA015675	Anoplophora glabripennis	Putative uncharacterized protein
EOG091200BB	APLA005630	Agrilus planipennis	Putative uncharacterized protein
EOG091200BB	TC001887	Tribolium castaneum	Putative uncharacterized protein
EOG091200BB	LDEC014390	Leptinotarsa decemlineata	Putative uncharacterized protein
EOG091200BB	YQE_09152	Dendroctonus ponderosae	Putative uncharacterized protein
EOG091200BD	OTAU005545	Onthophagus taurus	Putative uncharacterized protein
EOG091200BD	AGLA004495	Anoplophora glabripennis	Putative uncharacterized protein
EOG091200BD	APLA012545	Agrilus planipennis	Putative uncharacterized protein
EOG091200BD	TC007810	Tribolium castaneum	Putative uncharacterized protein
EOG091200BD	LDEC014763	Leptinotarsa decemlineata	Putative uncharacterized protein
EOG091200BD	YQE_04536	Dendroctonus ponderosae	Putative uncharacterized protein
EOG091200BE	OTAU004706	Onthophagus taurus	Putative uncharacterized protein
EOG091200BE	AGLA001294	Anoplophora glabripennis	Putative uncharacterized protein
EOG091200BE	APLA001764	Agrilus planipennis	Putative uncharacterized protein
EOG091200BE	TC010559	Tribolium castaneum	Putative uncharacterized protein
EOG091200BE	LDEC011349	Leptinotarsa decemlineata	Putative uncharacterized protein
EOG091200BE	YQE_03089	Dendroctonus ponderosae	Putative uncharacterized protein
EOG091200BF	OTAU008185	Onthophagus taurus	Putative uncharacterized protein
EOG091200BF	AGLA017557	Anoplophora glabripennis	Putative uncharacterized protein
EOG091200BF	APLA005945	Agrilus planipennis	Putative uncharacterized protein
EOG091200BF	TC014862	Tribolium castaneum	Putative uncharacterized protein
EOG091200BF	LDEC009955	Leptinotarsa decemlineata	Putative uncharacterized protein
EOG091200BF	YQE_10820	Dendroctonus ponderosae	Putative uncharacterized protein
EOG091200BH	OTAU010201	Onthophagus taurus	Putative uncharacterized protein
EOG091200BH	AGLA020194	Anoplophora glabripennis	Putative uncharacterized protein
EOG091200BH	APLA011704	Agrilus planipennis	Putative uncharacterized protein
EOG091200BH	TC030752	Tribolium castaneum	Putative uncharacterized protein
EOG091200BH	LDEC008940	Leptinotarsa decemlineata	Putative uncharacterized protein
EOG091200BH	YQE_06049	Dendroctonus ponderosae	Putative uncharacterized protein
EOG091200BI	OTAU003547	Onthophagus taurus	Putative uncharacterized protein
EOG091200BI	AGLA009780	Anoplophora glabripennis	Putative uncharacterized protein
EOG091200BI	APLA002656	Agrilus planipennis	Putative uncharacterized protein
EOG091200BI	TC010496	Tribolium castaneum	Putative uncharacterized protein

EOG091200BI	LDEC003831	Leptinotarsa decemlineata	Putative uncharacterized protein
EOG091200BI	YQE_12914	Dendroctonus ponderosae	Putative uncharacterized protein
EOG091200BJ	OTAU007192	Onthophagus taurus	Putative uncharacterized protein
EOG091200BJ	AGLA013541	Anoplophora glabripennis	Putative uncharacterized protein
EOG091200BJ	APLA010028	Agrilus planipennis	Putative uncharacterized protein
EOG091200BJ	TC014995	Tribolium castaneum	Putative uncharacterized protein
EOG091200BJ	LDEC022460	Leptinotarsa decemlineata	Putative uncharacterized protein
EOG091200BJ	YQE_10121	Dendroctonus ponderosae	Putative uncharacterized protein
EOG091200BL	OTAU010458	Onthophagus taurus	Putative uncharacterized protein
EOG091200BL	AGLA000773	Anoplophora glabripennis	Putative uncharacterized protein
EOG091200BL	APLA010315	Agrilus planipennis	Putative uncharacterized protein
EOG091200BL	TC007156	Tribolium castaneum	Putative uncharacterized protein
EOG091200BL	LDEC020645	Leptinotarsa decemlineata	Putative uncharacterized protein
EOG091200BL	YQE_10524	Dendroctonus ponderosae	Putative uncharacterized protein
EOG091200BN	OTAU000714	Onthophagus taurus	None
EOG091200BN	AGLA016556	Anoplophora glabripennis	None
EOG091200BN	APLA001773	Agrilus planipennis	None
EOG091200BN	TC031765	Tribolium castaneum	None
EOG091200BN	LDEC000507	Leptinotarsa decemlineata	None
EOG091200BN	YQE_07965	Dendroctonus ponderosae	None
EOG091200BO	OTAU002748	Onthophagus taurus	Putative uncharacterized protein
EOG091200BO	AGLA001151	Anoplophora glabripennis	Putative uncharacterized protein
EOG091200BO	APLA010000	Agrilus planipennis	Putative uncharacterized protein
EOG091200BO	TC005178	Tribolium castaneum	Putative uncharacterized protein
EOG091200BO	LDEC020951	Leptinotarsa decemlineata	Putative uncharacterized protein
EOG091200BO	YQE_06665	Dendroctonus ponderosae	Putative uncharacterized protein
EOG091200BS	OTAU003155	Onthophagus taurus	Putative uncharacterized protein
EOG091200BS	AGLA006735	Anoplophora glabripennis	Putative uncharacterized protein
EOG091200BS	APLA011891	Agrilus planipennis	Putative uncharacterized protein
EOG091200BS	TC014568	Tribolium castaneum	Putative uncharacterized protein
EOG091200BS	LDEC022355	Leptinotarsa decemlineata	Putative uncharacterized protein
EOG091200BS	YQE_07294	Dendroctonus ponderosae	Putative uncharacterized protein
EOG091200BT	OTAU001907	Onthophagus taurus	metal ion binding
EOG091200BT	AGLA015118	Anoplophora glabripennis	metal ion binding
EOG091200BT	APLA001211	Agrilus planipennis	metal ion binding
EOG091200BT	TC034845	Tribolium castaneum	metal ion binding
EOG091200BT	LDEC011699	Leptinotarsa decemlineata	metal ion binding
EOG091200BT	YQE_05547	Dendroctonus ponderosae	metal ion binding
EOG091200BU	OTAU002307	Onthophagus taurus	calcium ion binding
EOG091200BU	AGLA003101	Anoplophora glabripennis	calcium ion binding
EOG091200BU	APLA012536	Agrilus planipennis	calcium ion binding
EOG091200BU	TC032801	Tribolium castaneum	calcium ion binding
EOG091200BU	LDEC000029	Leptinotarsa decemlineata	calcium ion binding
EOG091200BU	YQE_09726	Dendroctonus ponderosae	calcium ion binding
EOG091200BV	OTAU016391	Onthophagus taurus	Putative uncharacterized protein
EOG091200BV	AGLA007054	Anoplophora glabripennis	Putative uncharacterized protein
EOG091200BV	APLA011921	Agrilus planipennis	Putative uncharacterized protein
EOG091200BV	TC000782	Tribolium castaneum	Putative uncharacterized protein
EOG091200BV	LDEC000818	Leptinotarsa decemlineata	Putative uncharacterized protein
EOG091200BV	YQE_08915	Dendroctonus ponderosae	Putative uncharacterized protein
EOG091200BW	OTAU013715	Onthophagus taurus	Argonaute 1
EOG091200BW	AGLA017309	Anoplophora glabripennis	Argonaute 1
EOG091200BW	APLA013701	Agrilus planipennis	Argonaute 1
EOG091200BW	TC005857	Tribolium castaneum	Argonaute 1
EOG091200BW	LDEC011109	Leptinotarsa decemlineata	Argonaute 1
EOG091200BW	YQE_02742	Dendroctonus ponderosae	Argonaute 1
EOG091200BX	OTAU010761	Onthophagus taurus	zinc ion binding
EOG091200BX	AGLA014246	Anoplophora glabripennis	zinc ion binding
EOG091200BX	APLA004965	Agrilus planipennis	zinc ion binding
EOG091200BX	TC034615	Tribolium castaneum	zinc ion binding
EOG091200BX	LDEC007365	Leptinotarsa decemlineata	zinc ion binding
EOG091200BX	YQE_11228	Dendroctonus ponderosae	zinc ion binding
EOG091200BY	OTAU000441	Onthophagus taurus	None
EOG091200BY	AGLA013937	Anoplophora glabripennis	None
EOG091200BY	APLA000393	Agrilus planipennis	None
EOG091200BY	TC032939	Tribolium castaneum	None
EOG091200BY	LDEC009310	Leptinotarsa decemlineata	None

EOG091200BY	YQE_06902	Dendroctonus ponderosae	None
EOG091200C0	OTAU003194	Onthophagus taurus	Spalt
EOG091200C0	AGLA007368	Anoplophora glabripennis	Spalt
EOG091200C0	APLA007347	Agrilus planipennis	Spalt
EOG091200C0	TC013501	Tribolium castaneum	Spalt
EOG091200C0	LDEC008341	Leptinotarsa decemlineata	Spalt
EOG091200C0	YQE_07172	Dendroctonus ponderosae	Spalt
EOG091200C1	OTAU003209	Onthophagus taurus	superoxide-generating NADPH oxidase activity
EOG091200C1	AGLA001743	Anoplophora glabripennis	superoxide-generating NADPH oxidase activity
EOG091200C1	APLA013444	Agrilus planipennis	superoxide-generating NADPH oxidase activity
EOG091200C1	TC033657	Tribolium castaneum	superoxide-generating NADPH oxidase activity
EOG091200C1	LDEC013091	Leptinotarsa decemlineata	superoxide-generating NADPH oxidase activity
EOG091200C1	YQE_12474	Dendroctonus ponderosae	superoxide-generating NADPH oxidase activity
EOG091200C3	OTAU008454	Onthophagus taurus	Putative uncharacterized protein
EOG091200C3	AGLA006637	Anoplophora glabripennis	Putative uncharacterized protein
EOG091200C3	APLA002004	Agrilus planipennis	Putative uncharacterized protein
EOG091200C3	TC002851	Tribolium castaneum	Putative uncharacterized protein
EOG091200C3	LDEC004974	Leptinotarsa decemlineata	Putative uncharacterized protein
EOG091200C3	YQE_12382	Dendroctonus ponderosae	Putative uncharacterized protein
EOG091200C7	OTAU000594	Onthophagus taurus	Putative uncharacterized protein
EOG091200C7	AGLA017459	Anoplophora glabripennis	Putative uncharacterized protein
EOG091200C7	APLA014333	Agrilus planipennis	Putative uncharacterized protein
EOG091200C7	TC012367	Tribolium castaneum	Putative uncharacterized protein
EOG091200C7	LDEC015595	Leptinotarsa decemlineata	Putative uncharacterized protein
EOG091200C7	YQE_10975	Dendroctonus ponderosae	Putative uncharacterized protein
EOG091200C8	OTAU014318	Onthophagus taurus	Putative uncharacterized protein
EOG091200C8	AGLA009398	Anoplophora glabripennis	Putative uncharacterized protein
EOG091200C8	APLA001679	Agrilus planipennis	Putative uncharacterized protein
EOG091200C8	TC010102	Tribolium castaneum	Putative uncharacterized protein
EOG091200C8	LDEC004432	Leptinotarsa decemlineata	Putative uncharacterized protein
EOG091200C8	YQE_05279	Dendroctonus ponderosae	Putative uncharacterized protein
EOG091200C9	OTAU007756	Onthophagus taurus	Putative uncharacterized protein
EOG091200C9	AGLA012663	Anoplophora glabripennis	Putative uncharacterized protein
EOG091200C9	APLA008801	Agrilus planipennis	Putative uncharacterized protein
EOG091200C9	TC009311	Tribolium castaneum	Putative uncharacterized protein
EOG091200C9	LDEC011011	Leptinotarsa decemlineata	Putative uncharacterized protein
EOG091200C9	YQE_05386	Dendroctonus ponderosae	Putative uncharacterized protein
EOG091200CA	OTAU012187	Onthophagus taurus	Putative uncharacterized protein
EOG091200CA	AGLA007671	Anoplophora glabripennis	Putative uncharacterized protein
EOG091200CA	APLA000027	Agrilus planipennis	Putative uncharacterized protein
EOG091200CA	TC007199	Tribolium castaneum	Putative uncharacterized protein
EOG091200CA	LDEC005088	Leptinotarsa decemlineata	Putative uncharacterized protein
EOG091200CA	YQE_02907	Dendroctonus ponderosae	Putative uncharacterized protein
EOG091200CC	OTAU006155	Onthophagus taurus	binding
EOG091200CC	AGLA002984	Anoplophora glabripennis	binding
EOG091200CC	APLA010742	Agrilus planipennis	binding
EOG091200CC	TC031089	Tribolium castaneum	binding
EOG091200CC	LDEC006237	Leptinotarsa decemlineata	binding
EOG091200CC	YQE_11690	Dendroctonus ponderosae	binding
EOG091200CD	OTAU008993	Onthophagus taurus	metal ion binding
EOG091200CD	AGLA010441	Anoplophora glabripennis	metal ion binding
EOG091200CD	APLA006453	Agrilus planipennis	metal ion binding
EOG091200CD	TC030914	Tribolium castaneum	metal ion binding
EOG091200CD	LDEC010476	Leptinotarsa decemlineata	metal ion binding
EOG091200CD	YQE_05902	Dendroctonus ponderosae	metal ion binding
EOG091200CE	OTAU013849	Onthophagus taurus	Putative uncharacterized protein
EOG091200CE	AGLA001934	Anoplophora glabripennis	Putative uncharacterized protein
EOG091200CE	APLA007996	Agrilus planipennis	Putative uncharacterized protein
EOG091200CE	TC005228	Tribolium castaneum	Putative uncharacterized protein
EOG091200CE	LDEC011196	Leptinotarsa decemlineata	Putative uncharacterized protein
EOG091200CE	YQE_04176	Dendroctonus ponderosae	Putative uncharacterized protein
EOG091200CJ	OTAU006839	Onthophagus taurus	Putative uncharacterized protein
EOG091200CJ	AGLA012778	Anoplophora glabripennis	Putative uncharacterized protein
EOG091200CJ	APLA004829	Agrilus planipennis	Putative uncharacterized protein
EOG091200CJ	TC014802	Tribolium castaneum	Putative uncharacterized protein
EOG091200CJ	LDEC009484	Leptinotarsa decemlineata	Putative uncharacterized protein
EOG091200CJ	YQE_07259	Dendroctonus ponderosae	Putative uncharacterized protein

EOG091200CL	OTAU013899	Onthophagus taurus	Putative uncharacterized protein
EOG091200CL	AGLA002278	Anoplophora glabripennis	Putative uncharacterized protein
EOG091200CL	APLA001061	Agrilus planipennis	Putative uncharacterized protein
EOG091200CL	TC011717	Tribolium castaneum	Putative uncharacterized protein
EOG091200CL	LDEC000583	Leptinotarsa decemlineata	Putative uncharacterized protein
EOG091200CL	YQE_07718	Dendroctonus ponderosae	Putative uncharacterized protein
EOG091200CP	OTAU003317	Onthophagus taurus	Aminopeptidase N-like protein
EOG091200CP	AGLA004579	Anoplophora glabripennis	Aminopeptidase N-like protein
EOG091200CP	APLA006360	Agrilus planipennis	Aminopeptidase N-like protein
EOG091200CP	TC004641	Tribolium castaneum	Aminopeptidase N-like protein
EOG091200CP	LDEC019715	Leptinotarsa decemlineata	Aminopeptidase N-like protein
EOG091200CP	YQE_09287	Dendroctonus ponderosae	Aminopeptidase N-like protein
EOG091200CQ	OTAU012814	Onthophagus taurus	DNA-directed RNA polymerase
EOG091200CQ	AGLA007095	Anoplophora glabripennis	DNA-directed RNA polymerase
EOG091200CQ	APLA014342	Agrilus planipennis	DNA-directed RNA polymerase
EOG091200CQ	TC015082	Tribolium castaneum	DNA-directed RNA polymerase
EOG091200CQ	LDEC002292	Leptinotarsa decemlineata	DNA-directed RNA polymerase
EOG091200CQ	YQE_11759	Dendroctonus ponderosae	DNA-directed RNA polymerase
EOG091200CS	OTAU010965	Onthophagus taurus	Putative uncharacterized protein
EOG091200CS	AGLA006908	Anoplophora glabripennis	Putative uncharacterized protein
EOG091200CS	APLA006685	Agrilus planipennis	Putative uncharacterized protein
EOG091200CS	TC010364	Tribolium castaneum	Putative uncharacterized protein
EOG091200CS	LDEC019429	Leptinotarsa decemlineata	Putative uncharacterized protein
EOG091200CS	YQE_12070	Dendroctonus ponderosae	Putative uncharacterized protein
EOG091200CT	OTAU002006	Onthophagus taurus	Putative uncharacterized protein
EOG091200CT	AGLA001315	Anoplophora glabripennis	Putative uncharacterized protein
EOG091200CT	APLA005311	Agrilus planipennis	Putative uncharacterized protein
EOG091200CT	TC000196	Tribolium castaneum	Putative uncharacterized protein
EOG091200CT	LDEC012367	Leptinotarsa decemlineata	Putative uncharacterized protein
EOG091200CT	YQE_10768	Dendroctonus ponderosae	Putative uncharacterized protein
EOG091200CV	OTAU016051	Onthophagus taurus	Putative uncharacterized protein
EOG091200CV	AGLA000131	Anoplophora glabripennis	Putative uncharacterized protein
EOG091200CV	APLA009355	Agrilus planipennis	Putative uncharacterized protein
EOG091200CV	TC001321	Tribolium castaneum	Putative uncharacterized protein
EOG091200CV	LDEC016501	Leptinotarsa decemlineata	Putative uncharacterized protein
EOG091200CV	YQE_08141	Dendroctonus ponderosae	Putative uncharacterized protein
EOG091200CX	OTAU001370	Onthophagus taurus	Putative uncharacterized protein
EOG091200CX	AGLA004539	Anoplophora glabripennis	Putative uncharacterized protein
EOG091200CX	APLA005614	Agrilus planipennis	Putative uncharacterized protein
EOG091200CX	TC004792	Tribolium castaneum	Putative uncharacterized protein
EOG091200CX	LDEC011475	Leptinotarsa decemlineata	Putative uncharacterized protein
EOG091200CX	YQE_02392	Dendroctonus ponderosae	Putative uncharacterized protein
EOG091200CY	OTAU000685	Onthophagus taurus	Putative uncharacterized protein
EOG091200CY	AGLA008125	Anoplophora glabripennis	Putative uncharacterized protein
EOG091200CY	APLA014953	Agrilus planipennis	Putative uncharacterized protein
EOG091200CY	TC011770	Tribolium castaneum	Putative uncharacterized protein
EOG091200CY	LDEC016253	Leptinotarsa decemlineata	Putative uncharacterized protein
EOG091200CY	YQE_04386	Dendroctonus ponderosae	Putative uncharacterized protein
EOG091200CZ	OTAU014738	Onthophagus taurus	Putative uncharacterized protein
EOG091200CZ	AGLA006341	Anoplophora glabripennis	Putative uncharacterized protein
EOG091200CZ	APLA000557	Agrilus planipennis	Putative uncharacterized protein
EOG091200CZ	TC015066	Tribolium castaneum	Putative uncharacterized protein
EOG091200CZ	LDEC007866	Leptinotarsa decemlineata	Putative uncharacterized protein
EOG091200CZ	YQE_05915	Dendroctonus ponderosae	Putative uncharacterized protein
EOG091200D0	OTAU003765	Onthophagus taurus	Putative uncharacterized protein
EOG091200D0	AGLA005756	Anoplophora glabripennis	Putative uncharacterized protein
EOG091200D0	APLA010176	Agrilus planipennis	Putative uncharacterized protein
EOG091200D0	TC009552	Tribolium castaneum	Putative uncharacterized protein
EOG091200D0	LDEC017903	Leptinotarsa decemlineata	Putative uncharacterized protein
EOG091200D0	YQE_11315	Dendroctonus ponderosae	Putative uncharacterized protein
EOG091200D2	OTAU006207	Onthophagus taurus	None
EOG091200D2	AGLA016914	Anoplophora glabripennis	None
EOG091200D2	APLA002519	Agrilus planipennis	None
EOG091200D2	TC034357	Tribolium castaneum	None
EOG091200D2	LDEC005125	Leptinotarsa decemlineata	None
EOG091200D2	YQE_12800	Dendroctonus ponderosae	None
EOG091200D3	OTAU005409	Onthophagus taurus	DE cadherin-like protein

EOG091200D3	AGLA000331	Anoplophora glabripennis	DE cadherin-like protein
EOG091200D3	APLA006412	Agrilus planipennis	DE cadherin-like protein
EOG091200D3	TC013570	Tribolium castaneum	DE cadherin-like protein
EOG091200D3	LDEC004521	Leptinotarsa decemlineata	DE cadherin-like protein
EOG091200D3	YQE_06698	Dendroctonus ponderosae	DE cadherin-like protein
EOG091200D4	OTAU008041	Onthophagus taurus	Putative uncharacterized protein
EOG091200D4	AGLA014094	Anoplophora glabripennis	Putative uncharacterized protein
EOG091200D4	APLA001425	Agrilus planipennis	Putative uncharacterized protein
EOG091200D4	TC015481	Tribolium castaneum	Putative uncharacterized protein
EOG091200D4	LDEC009837	Leptinotarsa decemlineata	Putative uncharacterized protein
EOG091200D4	YQE_06971	Dendroctonus ponderosae	Putative uncharacterized protein
EOG091200D5	OTAU009525	Onthophagus taurus	helicase activity
EOG091200D5	AGLA016978	Anoplophora glabripennis	helicase activity
EOG091200D5	APLA010851	Agrilus planipennis	helicase activity
EOG091200D5	TC033645	Tribolium castaneum	helicase activity
EOG091200D5	LDEC002198	Leptinotarsa decemlineata	helicase activity
EOG091200D5	YQE_11791	Dendroctonus ponderosae	helicase activity
EOG091200D6	OTAU006257	Onthophagus taurus	Delta-like protein
EOG091200D6	AGLA008757	Anoplophora glabripennis	Delta-like protein
EOG091200D6	APLA006117	Agrilus planipennis	Delta-like protein
EOG091200D6	TC010113	Tribolium castaneum	Delta-like protein
EOG091200D6	LDEC012442	Leptinotarsa decemlineata	Delta-like protein
EOG091200D6	YQE_02359	Dendroctonus ponderosae	Delta-like protein
EOG091200D7	OTAU003104	Onthophagus taurus	Putative uncharacterized protein
EOG091200D7	AGLA004796	Anoplophora glabripennis	Putative uncharacterized protein
EOG091200D7	APLA005212	Agrilus planipennis	Putative uncharacterized protein
EOG091200D7	TC030754	Tribolium castaneum	Putative uncharacterized protein
EOG091200D7	LDEC007082	Leptinotarsa decemlineata	Putative uncharacterized protein
EOG091200D7	YQE_07635	Dendroctonus ponderosae	Putative uncharacterized protein
EOG091200D8	OTAU007849	Onthophagus taurus	None
EOG091200D8	AGLA006451	Anoplophora glabripennis	None
EOG091200D8	APLA001818	Agrilus planipennis	None
EOG091200D8	TC031207	Tribolium castaneum	None
EOG091200D8	LDEC006169	Leptinotarsa decemlineata	None
EOG091200D8	YQE_11343	Dendroctonus ponderosae	None
EOG091200DA	OTAU008574	Onthophagus taurus	Putative uncharacterized protein
EOG091200DA	AGLA008662	Anoplophora glabripennis	Putative uncharacterized protein
EOG091200DA	APLA010276	Agrilus planipennis	Putative uncharacterized protein
EOG091200DA	TC010168	Tribolium castaneum	Putative uncharacterized protein
EOG091200DA	LDEC010005	Leptinotarsa decemlineata	Putative uncharacterized protein
EOG091200DA	YQE_11350	Dendroctonus ponderosae	Putative uncharacterized protein
EOG091200DB	OTAU011100	Onthophagus taurus	Putative uncharacterized protein
EOG091200DB	AGLA002407	Anoplophora glabripennis	Putative uncharacterized protein
EOG091200DB	APLA008955	Agrilus planipennis	Putative uncharacterized protein
EOG091200DB	TC004357	Tribolium castaneum	Putative uncharacterized protein
EOG091200DB	LDEC013402	Leptinotarsa decemlineata	Putative uncharacterized protein
EOG091200DB	YQE_10965	Dendroctonus ponderosae	Putative uncharacterized protein
EOG091200DD	OTAU012312	Onthophagus taurus	Coatomer subunit beta
EOG091200DD	AGLA006686	Anoplophora glabripennis	Coatomer subunit beta
EOG091200DD	APLA005501	Agrilus planipennis	Coatomer subunit beta
EOG091200DD	TC000641	Tribolium castaneum	Coatomer subunit beta
EOG091200DD	LDEC019587	Leptinotarsa decemlineata	Coatomer subunit beta
EOG091200DD	YQE_01620	Dendroctonus ponderosae	Coatomer subunit beta
EOG091200DE	OTAU011809	Onthophagus taurus	Putative uncharacterized protein
EOG091200DE	AGLA011032	Anoplophora glabripennis	Putative uncharacterized protein
EOG091200DE	APLA000482	Agrilus planipennis	Putative uncharacterized protein
EOG091200DE	TC005767	Tribolium castaneum	Putative uncharacterized protein
EOG091200DE	LDEC006954	Leptinotarsa decemlineata	Putative uncharacterized protein
EOG091200DE	YQE_01900	Dendroctonus ponderosae	Putative uncharacterized protein
EOG091200DF	OTAU000836	Onthophagus taurus	Putative uncharacterized protein
EOG091200DF	AGLA015851	Anoplophora glabripennis	Putative uncharacterized protein
EOG091200DF	APLA010601	Agrilus planipennis	Putative uncharacterized protein
EOG091200DF	TC004396	Tribolium castaneum	Putative uncharacterized protein
EOG091200DF	LDEC013041	Leptinotarsa decemlineata	Putative uncharacterized protein
EOG091200DF	YQE_08921	Dendroctonus ponderosae	Putative uncharacterized protein
EOG091200DH	OTAU013303	Onthophagus taurus	Putative uncharacterized protein
EOG091200DH	AGLA008860	Anoplophora glabripennis	Putative uncharacterized protein

EOG091200DH	APLA009378	Agrilus planipennis	Putative uncharacterized protein
EOG091200DH	TC014283	Tribolium castaneum	Putative uncharacterized protein
EOG091200DH	LDEC006184	Leptinotarsa decemlineata	Putative uncharacterized protein
EOG091200DH	YQE_01970	Dendroctonus ponderosae	Putative uncharacterized protein
EOG091200DJ	OTAU000841	Onthophagus taurus	Putative uncharacterized protein
EOG091200DJ	AGLA013148	Anoplophora glabripennis	Putative uncharacterized protein
EOG091200DJ	APLA015057	Agrilus planipennis	Putative uncharacterized protein
EOG091200DJ	TC016087	Tribolium castaneum	Putative uncharacterized protein
EOG091200DJ	LDEC005576	Leptinotarsa decemlineata	Putative uncharacterized protein
EOG091200DJ	YQE_07062	Dendroctonus ponderosae	Putative uncharacterized protein
EOG091200DL	OTAU013590	Onthophagus taurus	Putative uncharacterized protein
EOG091200DL	AGLA008463	Anoplophora glabripennis	Putative uncharacterized protein
EOG091200DL	APLA003048	Agrilus planipennis	Putative uncharacterized protein
EOG091200DL	TC014294	Tribolium castaneum	Putative uncharacterized protein
EOG091200DL	LDEC008327	Leptinotarsa decemlineata	Putative uncharacterized protein
EOG091200DL	YQE_06274	Dendroctonus ponderosae	Putative uncharacterized protein
EOG091200DM	OTAU004719	Onthophagus taurus	actin binding
EOG091200DM	AGLA007732	Anoplophora glabripennis	actin binding
EOG091200DM	APLA008393	Agrilus planipennis	actin binding
EOG091200DM	TC034589	Tribolium castaneum	actin binding
EOG091200DM	LDEC016193	Leptinotarsa decemlineata	actin binding
EOG091200DM	YQE_10221	Dendroctonus ponderosae	actin binding
EOG091200DN	OTAU004689	Onthophagus taurus	Putative uncharacterized protein
EOG091200DN	AGLA001255	Anoplophora glabripennis	Putative uncharacterized protein
EOG091200DN	APLA001064	Agrilus planipennis	Putative uncharacterized protein
EOG091200DN	TC012257	Tribolium castaneum	Putative uncharacterized protein
EOG091200DN	LDEC011035	Leptinotarsa decemlineata	Putative uncharacterized protein
EOG091200DN	YQE_08102	Dendroctonus ponderosae	Putative uncharacterized protein
EOG091200DO	OTAU003613	Onthophagus taurus	Putative uncharacterized protein
EOG091200DO	AGLA015162	Anoplophora glabripennis	Putative uncharacterized protein
EOG091200DO	APLA003740	Agrilus planipennis	Putative uncharacterized protein
EOG091200DO	TC003699	Tribolium castaneum	Putative uncharacterized protein
EOG091200DO	LDEC013128	Leptinotarsa decemlineata	Putative uncharacterized protein
EOG091200DO	YQE_07904	Dendroctonus ponderosae	Putative uncharacterized protein
EOG091200DP	OTAU009912	Onthophagus taurus	Putative uncharacterized protein
EOG091200DP	AGLA012213	Anoplophora glabripennis	Putative uncharacterized protein
EOG091200DP	APLA014542	Agrilus planipennis	Putative uncharacterized protein
EOG091200DP	TC013586	Tribolium castaneum	Putative uncharacterized protein
EOG091200DP	LDEC011073	Leptinotarsa decemlineata	Putative uncharacterized protein
EOG091200DP	YQE_09239	Dendroctonus ponderosae	Putative uncharacterized protein
EOG091200DQ	OTAU005231	Onthophagus taurus	Putative uncharacterized protein
EOG091200DQ	AGLA001691	Anoplophora glabripennis	Putative uncharacterized protein
EOG091200DQ	APLA010856	Agrilus planipennis	Putative uncharacterized protein
EOG091200DQ	TC009420	Tribolium castaneum	Putative uncharacterized protein
EOG091200DQ	LDEC006179	Leptinotarsa decemlineata	Putative uncharacterized protein
EOG091200DQ	YQE_11300	Dendroctonus ponderosae	Putative uncharacterized protein
EOG091200DR	OTAU004572	Onthophagus taurus	Putative uncharacterized protein
EOG091200DR	AGLA003702	Anoplophora glabripennis	Putative uncharacterized protein
EOG091200DR	APLA005403	Agrilus planipennis	Putative uncharacterized protein
EOG091200DR	TC008067	Tribolium castaneum	Putative uncharacterized protein
EOG091200DR	LDEC006028	Leptinotarsa decemlineata	Putative uncharacterized protein
EOG091200DR	YQE_09724	Dendroctonus ponderosae	Putative uncharacterized protein
EOG091200DS	OTAU000998	Onthophagus taurus	Putative uncharacterized protein
EOG091200DS	AGLA001897	Anoplophora glabripennis	Putative uncharacterized protein
EOG091200DS	APLA001325	Agrilus planipennis	Putative uncharacterized protein
EOG091200DS	TC000591	Tribolium castaneum	Putative uncharacterized protein
EOG091200DS	LDEC014072	Leptinotarsa decemlineata	Putative uncharacterized protein
EOG091200DS	YQE_02548	Dendroctonus ponderosae	Putative uncharacterized protein
EOG091200DT	OTAU007100	Onthophagus taurus	Putative uncharacterized protein
EOG091200DT	AGLA001096	Anoplophora glabripennis	Putative uncharacterized protein
EOG091200DT	APLA005962	Agrilus planipennis	Putative uncharacterized protein
EOG091200DT	TC015760	Tribolium castaneum	Putative uncharacterized protein
EOG091200DT	LDEC005193	Leptinotarsa decemlineata	Putative uncharacterized protein
EOG091200DT	YQE_11734	Dendroctonus ponderosae	Putative uncharacterized protein
EOG091200DW	OTAU001102	Onthophagus taurus	None
EOG091200DW	AGLA004512	Anoplophora glabripennis	None
EOG091200DW	APLA001926	Agrilus planipennis	None

EOG091200DW	TC032155	Tribolium castaneum	None
EOG091200DW	LDEC012285	Leptinotarsa decemlineata	None
EOG091200DW	YQE_06500	Dendroctonus ponderosae	None
EOG091200DY	OTAU007509	Onthophagus taurus	Putative uncharacterized protein
EOG091200DY	AGLA009461	Anoplophora glabripennis	Putative uncharacterized protein
EOG091200DY	APLA014385	Agrilus planipennis	Putative uncharacterized protein
EOG091200DY	TC015622	Tribolium castaneum	Putative uncharacterized protein
EOG091200DY	LDEC002238	Leptinotarsa decemlineata	Putative uncharacterized protein
EOG091200DY	YQE_11729	Dendroctonus ponderosae	Putative uncharacterized protein
EOG091200DZ	OTAU016027	Onthophagus taurus	None
EOG091200DZ	AGLA000031	Anoplophora glabripennis	None
EOG091200DZ	APLA008909	Agrilus planipennis	None
EOG091200DZ	TC031397	Tribolium castaneum	None
EOG091200DZ	LDEC012642	Leptinotarsa decemlineata	None
EOG091200DZ	YQE_10686	Dendroctonus ponderosae	None
EOG091200E0	OTAU007199	Onthophagus taurus	Putative uncharacterized protein
EOG091200E0	AGLA009969	Anoplophora glabripennis	Putative uncharacterized protein
EOG091200E0	APLA000547	Agrilus planipennis	Putative uncharacterized protein
EOG091200E0	TC015626	Tribolium castaneum	Putative uncharacterized protein
EOG091200E0	LDEC008819	Leptinotarsa decemlineata	Putative uncharacterized protein
EOG091200E0	YQE_11454	Dendroctonus ponderosae	Putative uncharacterized protein
EOG091200E4	OTAU000313	Onthophagus taurus	Putative uncharacterized protein
EOG091200E4	AGLA016576	Anoplophora glabripennis	Putative uncharacterized protein
EOG091200E4	APLA008107	Agrilus planipennis	Putative uncharacterized protein
EOG091200E4	TC013377	Tribolium castaneum	Putative uncharacterized protein
EOG091200E4	LDEC015115	Leptinotarsa decemlineata	Putative uncharacterized protein
EOG091200E4	YQE_06151	Dendroctonus ponderosae	Putative uncharacterized protein
EOG091200E6	OTAU002319	Onthophagus taurus	protein-hormone receptor activity
EOG091200E6	AGLA010148	Anoplophora glabripennis	protein-hormone receptor activity
EOG091200E6	APLA000229	Agrilus planipennis	protein-hormone receptor activity
EOG091200E6	TC008163	Tribolium castaneum	protein-hormone receptor activity
EOG091200E6	LDEC012905	Leptinotarsa decemlineata	protein-hormone receptor activity
EOG091200E6	YQE_00009	Dendroctonus ponderosae	protein-hormone receptor activity
EOG091200E7	OTAU006680	Onthophagus taurus	oxidoreductase activity
EOG091200E7	AGLA004224	Anoplophora glabripennis	oxidoreductase activity
EOG091200E7	APLA007224	Agrilus planipennis	oxidoreductase activity
EOG091200E7	TC032686	Tribolium castaneum	oxidoreductase activity
EOG091200E7	LDEC004944	Leptinotarsa decemlineata	oxidoreductase activity
EOG091200E7	YQE_10112	Dendroctonus ponderosae	oxidoreductase activity
EOG091200E8	OTAU007257	Onthophagus taurus	Putative uncharacterized protein
EOG091200E8	AGLA016406	Anoplophora glabripennis	Putative uncharacterized protein
EOG091200E8	APLA003259	Agrilus planipennis	Putative uncharacterized protein
EOG091200E8	TC011848	Tribolium castaneum	Putative uncharacterized protein
EOG091200E8	LDEC019894	Leptinotarsa decemlineata	Putative uncharacterized protein
EOG091200E8	YQE_06372	Dendroctonus ponderosae	Putative uncharacterized protein
EOG091200EA	OTAU000631	Onthophagus taurus	Nitric oxide synthase
EOG091200EA	AGLA008135	Anoplophora glabripennis	Nitric oxide synthase
EOG091200EA	APLA011551	Agrilus planipennis	Nitric oxide synthase
EOG091200EA	TC012639	Tribolium castaneum	Nitric oxide synthase
EOG091200EA	LDEC021084	Leptinotarsa decemlineata	Nitric oxide synthase
EOG091200EA	YQE_01996	Dendroctonus ponderosae	Nitric oxide synthase
EOG091200ED	OTAU012081	Onthophagus taurus	Putative uncharacterized protein
EOG091200ED	AGLA005856	Anoplophora glabripennis	Putative uncharacterized protein
EOG091200ED	APLA008835	Agrilus planipennis	Putative uncharacterized protein
EOG091200ED	TC008618	Tribolium castaneum	Putative uncharacterized protein
EOG091200ED	LDEC013270	Leptinotarsa decemlineata	Putative uncharacterized protein
EOG091200ED	YQE_03509	Dendroctonus ponderosae	Putative uncharacterized protein
EOG091200EH	OTAU015642	Onthophagus taurus	Putative uncharacterized protein
EOG091200EH	AGLA009294	Anoplophora glabripennis	Putative uncharacterized protein
EOG091200EH	APLA004496	Agrilus planipennis	Putative uncharacterized protein
EOG091200EH	TC005895	Tribolium castaneum	Putative uncharacterized protein
EOG091200EH	LDEC018339	Leptinotarsa decemlineata	Putative uncharacterized protein
EOG091200EH	YQE_12697	Dendroctonus ponderosae	Putative uncharacterized protein
EOG091200EL	OTAU014119	Onthophagus taurus	Putative uncharacterized protein
EOG091200EL	AGLA019363	Anoplophora glabripennis	Putative uncharacterized protein
EOG091200EL	APLA014013	Agrilus planipennis	Putative uncharacterized protein
EOG091200EL	TC009532	Tribolium castaneum	Putative uncharacterized protein

EOG091200EL	LDEC010648	Leptinotarsa decemlineata	Putative uncharacterized protein
EOG091200EL	YQE_11814	Dendroctonus ponderosae	Putative uncharacterized protein
EOG091200EM	OTAU001433	Onthophagus taurus	Putative uncharacterized protein
EOG091200EM	AGLA005400	Anoplophora glabripennis	Putative uncharacterized protein
EOG091200EM	APLA014537	Agrilus planipennis	Putative uncharacterized protein
EOG091200EM	TC004746	Tribolium castaneum	Putative uncharacterized protein
EOG091200EM	LDEC017481	Leptinotarsa decemlineata	Putative uncharacterized protein
EOG091200EM	YQE_02499	Dendroctonus ponderosae	Putative uncharacterized protein
EOG091200EN	OTAU000968	Onthophagus taurus	None
EOG091200EN	AGLA004163	Anoplophora glabripennis	None
EOG091200EN	APLA002910	Agrilus planipennis	None
EOG091200EN	TC032110	Tribolium castaneum	None
EOG091200EN	LDEC000903	Leptinotarsa decemlineata	None
EOG091200EN	YQE_09514	Dendroctonus ponderosae	None
EOG091200EO	OTAU010926	Onthophagus taurus	Translocase of outer membrane 34
EOG091200EO	AGLA003884	Anoplophora glabripennis	Translocase of outer membrane 34
EOG091200EO	APLA001874	Agrilus planipennis	Translocase of outer membrane 34
EOG091200EO	TC015213	Tribolium castaneum	Translocase of outer membrane 34
EOG091200EO	LDEC008805	Leptinotarsa decemlineata	Translocase of outer membrane 34
EOG091200EO	YQE_05895	Dendroctonus ponderosae	Translocase of outer membrane 34
EOG091200EQ	OTAU008165	Onthophagus taurus	Putative uncharacterized protein
EOG091200EQ	AGLA009474	Anoplophora glabripennis	Putative uncharacterized protein
EOG091200EQ	APLA014388	Agrilus planipennis	Putative uncharacterized protein
EOG091200EQ	TC015686	Tribolium castaneum	Putative uncharacterized protein
EOG091200EQ	LDEC012532	Leptinotarsa decemlineata	Putative uncharacterized protein
EOG091200EQ	YQE_10884	Dendroctonus ponderosae	Putative uncharacterized protein
EOG091200ER	OTAU013294	Onthophagus taurus	Putative uncharacterized protein
EOG091200ER	AGLA002641	Anoplophora glabripennis	Putative uncharacterized protein
EOG091200ER	APLA005155	Agrilus planipennis	Putative uncharacterized protein
EOG091200ER	TC030661	Tribolium castaneum	Putative uncharacterized protein
EOG091200ER	LDEC017379	Leptinotarsa decemlineata	Putative uncharacterized protein
EOG091200ER	YQE_10349	Dendroctonus ponderosae	Putative uncharacterized protein
EOG091200ES	OTAU000166	Onthophagus taurus	Putative uncharacterized protein
EOG091200ES	AGLA019849	Anoplophora glabripennis	Putative uncharacterized protein
EOG091200ES	APLA012126	Agrilus planipennis	Putative uncharacterized protein
EOG091200ES	TC014672	Tribolium castaneum	Putative uncharacterized protein
EOG091200ES	LDEC012481	Leptinotarsa decemlineata	Putative uncharacterized protein
EOG091200ES	YQE_08216	Dendroctonus ponderosae	Putative uncharacterized protein
EOG091200ET	OTAU004745	Onthophagus taurus	binding
EOG091200ET	AGLA005563	Anoplophora glabripennis	binding
EOG091200ET	APLA006297	Agrilus planipennis	binding
EOG091200ET	TC032667	Tribolium castaneum	binding
EOG091200ET	LDEC009712	Leptinotarsa decemlineata	binding
EOG091200ET	YQE_02273	Dendroctonus ponderosae	binding
EOG091200EV	OTAU012261	Onthophagus taurus	RecQ4
EOG091200EV	AGLA018293	Anoplophora glabripennis	RecQ4
EOG091200EV	APLA003757	Agrilus planipennis	RecQ4
EOG091200EV	TC003875	Tribolium castaneum	RecQ4
EOG091200EV	LDEC013588	Leptinotarsa decemlineata	RecQ4
EOG091200EV	YQE_03645	Dendroctonus ponderosae	RecQ4
EOG091200EX	OTAU001785	Onthophagus taurus	Putative uncharacterized protein
EOG091200EX	AGLA013678	Anoplophora glabripennis	Putative uncharacterized protein
EOG091200EX	APLA004454	Agrilus planipennis	Putative uncharacterized protein
EOG091200EX	TC005794	Tribolium castaneum	Putative uncharacterized protein
EOG091200EX	LDEC006329	Leptinotarsa decemlineata	Putative uncharacterized protein
EOG091200EX	YQE_12053	Dendroctonus ponderosae	Putative uncharacterized protein
EOG091200F2	OTAU008450	Onthophagus taurus	Putative uncharacterized protein
EOG091200F2	AGLA020296	Anoplophora glabripennis	Putative uncharacterized protein
EOG091200F2	APLA005247	Agrilus planipennis	Putative uncharacterized protein
EOG091200F2	TC030696	Tribolium castaneum	Putative uncharacterized protein
EOG091200F2	LDEC020283	Leptinotarsa decemlineata	Putative uncharacterized protein
EOG091200F2	YQE_08282	Dendroctonus ponderosae	Putative uncharacterized protein
EOG091200F4	OTAU015593	Onthophagus taurus	Putative uncharacterized protein
EOG091200F4	AGLA013981	Anoplophora glabripennis	Putative uncharacterized protein
EOG091200F4	APLA001344	Agrilus planipennis	Putative uncharacterized protein
EOG091200F4	TC011290	Tribolium castaneum	Putative uncharacterized protein
EOG091200F4	LDEC001716	Leptinotarsa decemlineata	Putative uncharacterized protein

EOG091200F4	YQE_08919	Dendroctonus ponderosae	Putative uncharacterized protein
EOG091200F5	OTAU006295	Onthophagus taurus	Putative uncharacterized protein
EOG091200F5	AGLA005270	Anoplophora glabripennis	Putative uncharacterized protein
EOG091200F5	APLA009043	Agrilus planipennis	Putative uncharacterized protein
EOG091200F5	TC007799	Tribolium castaneum	Putative uncharacterized protein
EOG091200F5	LDEC008479	Leptinotarsa decemlineata	Putative uncharacterized protein
EOG091200F5	YQE_10793	Dendroctonus ponderosae	Putative uncharacterized protein
EOG091200F7	OTAU013132	Onthophagus taurus	Putative uncharacterized protein
EOG091200F7	AGLA014732	Anoplophora glabripennis	Putative uncharacterized protein
EOG091200F7	APLA005073	Agrilus planipennis	Putative uncharacterized protein
EOG091200F7	TC005949	Tribolium castaneum	Putative uncharacterized protein
EOG091200F7	LDEC010112	Leptinotarsa decemlineata	Putative uncharacterized protein
EOG091200F7	YQE_05636	Dendroctonus ponderosae	Putative uncharacterized protein
EOG091200FC	OTAU008227	Onthophagus taurus	Putative uncharacterized protein
EOG091200FC	AGLA001384	Anoplophora glabripennis	Putative uncharacterized protein
EOG091200FC	APLA011916	Agrilus planipennis	Putative uncharacterized protein
EOG091200FC	TC000308	Tribolium castaneum	Putative uncharacterized protein
EOG091200FC	LDEC007311	Leptinotarsa decemlineata	Putative uncharacterized protein
EOG091200FC	YQE_11652	Dendroctonus ponderosae	Putative uncharacterized protein
EOG091200FE	OTAU005899	Onthophagus taurus	Putative uncharacterized protein
EOG091200FE	AGLA007885	Anoplophora glabripennis	Putative uncharacterized protein
EOG091200FE	APLA006858	Agrilus planipennis	Putative uncharacterized protein
EOG091200FE	TC014438	Tribolium castaneum	Putative uncharacterized protein
EOG091200FE	LDEC003064	Leptinotarsa decemlineata	Putative uncharacterized protein
EOG091200FE	YQE_06104	Dendroctonus ponderosae	Putative uncharacterized protein
EOG091200FF	OTAU014150	Onthophagus taurus	Putative uncharacterized protein
EOG091200FF	AGLA008981	Anoplophora glabripennis	Putative uncharacterized protein
EOG091200FF	APLA006444	Agrilus planipennis	Putative uncharacterized protein
EOG091200FF	TC015699	Tribolium castaneum	Putative uncharacterized protein
EOG091200FF	LDEC001901	Leptinotarsa decemlineata	Putative uncharacterized protein
EOG091200FF	YQE_05963	Dendroctonus ponderosae	Putative uncharacterized protein
EOG091200FG	OTAU011189	Onthophagus taurus	Putative uncharacterized protein
EOG091200FG	AGLA014185	Anoplophora glabripennis	Putative uncharacterized protein
EOG091200FG	APLA006609	Agrilus planipennis	Putative uncharacterized protein
EOG091200FG	TC007618	Tribolium castaneum	Putative uncharacterized protein
EOG091200FG	LDEC010944	Leptinotarsa decemlineata	Putative uncharacterized protein
EOG091200FG	YQE_05105	Dendroctonus ponderosae	Putative uncharacterized protein
EOG091200FH	OTAU000851	Onthophagus taurus	Putative uncharacterized protein
EOG091200FH	AGLA005644	Anoplophora glabripennis	Putative uncharacterized protein
EOG091200FH	APLA001363	Agrilus planipennis	Putative uncharacterized protein
EOG091200FH	TC011069	Tribolium castaneum	Putative uncharacterized protein
EOG091200FH	LDEC007515	Leptinotarsa decemlineata	Putative uncharacterized protein
EOG091200FH	YQE_07342	Dendroctonus ponderosae	Putative uncharacterized protein
EOG091200FK	OTAU000199	Onthophagus taurus	None
EOG091200FK	AGLA003638	Anoplophora glabripennis	None
EOG091200FK	APLA009013	Agrilus planipennis	None
EOG091200FK	TC033238	Tribolium castaneum	None
EOG091200FK	LDEC014386	Leptinotarsa decemlineata	None
EOG091200FK	YQE_02060	Dendroctonus ponderosae	None
EOG091200FL	OTAU006834	Onthophagus taurus	phospholipid binding
EOG091200FL	AGLA012777	Anoplophora glabripennis	phospholipid binding
EOG091200FL	APLA004831	Agrilus planipennis	phospholipid binding
EOG091200FL	TC033274	Tribolium castaneum	phospholipid binding
EOG091200FL	LDEC009483	Leptinotarsa decemlineata	phospholipid binding
EOG091200FL	YQE_07260	Dendroctonus ponderosae	phospholipid binding
EOG091200FM	OTAU011487	Onthophagus taurus	Putative uncharacterized protein
EOG091200FM	AGLA005252	Anoplophora glabripennis	Putative uncharacterized protein
EOG091200FM	APLA003887	Agrilus planipennis	Putative uncharacterized protein
EOG091200FM	TC007655	Tribolium castaneum	Putative uncharacterized protein
EOG091200FM	LDEC000073	Leptinotarsa decemlineata	Putative uncharacterized protein
EOG091200FM	YQE_03397	Dendroctonus ponderosae	Putative uncharacterized protein
EOG091200FN	OTAU015654	Onthophagus taurus	Putative uncharacterized protein
EOG091200FN	AGLA004339	Anoplophora glabripennis	Putative uncharacterized protein
EOG091200FN	APLA002478	Agrilus planipennis	Putative uncharacterized protein
EOG091200FN	TC013449	Tribolium castaneum	Putative uncharacterized protein
EOG091200FN	LDEC005760	Leptinotarsa decemlineata	Putative uncharacterized protein
EOG091200FN	YQE_04044	Dendroctonus ponderosae	Putative uncharacterized protein

EOG091200FP	OTAU004913	Onthophagus taurus	Putative uncharacterized protein
EOG091200FP	AGLA002408	Anoplophora glabripennis	Putative uncharacterized protein
EOG091200FP	APLA005508	Agrilus planipennis	Putative uncharacterized protein
EOG091200FP	TC000391	Tribolium castaneum	Putative uncharacterized protein
EOG091200FP	LDEC013396	Leptinotarsa decemlineata	Putative uncharacterized protein
EOG091200FP	YQE_08080	Dendroctonus ponderosae	Putative uncharacterized protein
EOG091200FR	OTAU013946	Onthophagus taurus	Putative uncharacterized protein
EOG091200FR	AGLA009936	Anoplophora glabripennis	Putative uncharacterized protein
EOG091200FR	APLA000651	Agrilus planipennis	Putative uncharacterized protein
EOG091200FR	TC014910	Tribolium castaneum	Putative uncharacterized protein
EOG091200FR	LDEC008023	Leptinotarsa decemlineata	Putative uncharacterized protein
EOG091200FR	YQE_10901	Dendroctonus ponderosae	Putative uncharacterized protein
EOG091200FT	OTAU002033	Onthophagus taurus	Putative uncharacterized protein
EOG091200FT	AGLA004408	Anoplophora glabripennis	Putative uncharacterized protein
EOG091200FT	APLA002511	Agrilus planipennis	Putative uncharacterized protein
EOG091200FT	TC012008	Tribolium castaneum	Putative uncharacterized protein
EOG091200FT	LDEC005115	Leptinotarsa decemlineata	Putative uncharacterized protein
EOG091200FT	YQE_07962	Dendroctonus ponderosae	Putative uncharacterized protein
EOG091200FU	OTAU002763	Onthophagus taurus	Putative uncharacterized protein
EOG091200FU	AGLA000255	Anoplophora glabripennis	Putative uncharacterized protein
EOG091200FU	APLA006414	Agrilus planipennis	Putative uncharacterized protein
EOG091200FU	TC001714	Tribolium castaneum	Putative uncharacterized protein
EOG091200FU	LDEC012035	Leptinotarsa decemlineata	Putative uncharacterized protein
EOG091200FU	YQE_02386	Dendroctonus ponderosae	Putative uncharacterized protein
EOG091200FV	OTAU005589	Onthophagus taurus	metallopeptidase activity
EOG091200FV	AGLA000862	Anoplophora glabripennis	metallopeptidase activity
EOG091200FV	APLA014311	Agrilus planipennis	metallopeptidase activity
EOG091200FV	TC033768	Tribolium castaneum	metallopeptidase activity
EOG091200FV	LDEC020601	Leptinotarsa decemlineata	metallopeptidase activity
EOG091200FV	YQE_08890	Dendroctonus ponderosae	metallopeptidase activity
EOG091200FW	OTAU006860	Onthophagus taurus	Putative uncharacterized protein
EOG091200FW	AGLA020813	Anoplophora glabripennis	Putative uncharacterized protein
EOG091200FW	APLA008532	Agrilus planipennis	Putative uncharacterized protein
EOG091200FW	TC013867	Tribolium castaneum	Putative uncharacterized protein
EOG091200FW	LDEC020806	Leptinotarsa decemlineata	Putative uncharacterized protein
EOG091200FW	YQE_11389	Dendroctonus ponderosae	Putative uncharacterized protein
EOG091200FY	OTAU016833	Onthophagus taurus	zinc ion binding
EOG091200FY	AGLA006133	Anoplophora glabripennis	zinc ion binding
EOG091200FY	APLA001309	Agrilus planipennis	zinc ion binding
EOG091200FY	TC032222	Tribolium castaneum	zinc ion binding
EOG091200FY	LDEC016729	Leptinotarsa decemlineata	zinc ion binding
EOG091200FY	YQE_08541	Dendroctonus ponderosae	zinc ion binding
EOG091200FZ	OTAU007592	Onthophagus taurus	Robo3
EOG091200FZ	AGLA010689	Anoplophora glabripennis	Robo3
EOG091200FZ	APLA004304	Agrilus planipennis	Robo3
EOG091200FZ	TC006743	Tribolium castaneum	Robo3
EOG091200FZ	LDEC017818	Leptinotarsa decemlineata	Robo3
EOG091200FZ	YQE_12039	Dendroctonus ponderosae	Robo3
EOG091200G0	OTAU006213	Onthophagus taurus	Putative uncharacterized protein
EOG091200G0	AGLA008667	Anoplophora glabripennis	Putative uncharacterized protein
EOG091200G0	APLA002528	Agrilus planipennis	Putative uncharacterized protein
EOG091200G0	TC011996	Tribolium castaneum	Putative uncharacterized protein
EOG091200G0	LDEC004139	Leptinotarsa decemlineata	Putative uncharacterized protein
EOG091200G0	YQE_07326	Dendroctonus ponderosae	Putative uncharacterized protein
EOG091200G5	OTAU013802	Onthophagus taurus	ribonuclease III activity
EOG091200G5	AGLA019222	Anoplophora glabripennis	ribonuclease III activity
EOG091200G5	APLA011610	Agrilus planipennis	ribonuclease III activity
EOG091200G5	TC031425	Tribolium castaneum	ribonuclease III activity
EOG091200G5	LDEC011163	Leptinotarsa decemlineata	ribonuclease III activity
EOG091200G5	YQE_04998	Dendroctonus ponderosae	ribonuclease III activity
EOG091200G6	OTAU013039	Onthophagus taurus	None
EOG091200G6	AGLA018371	Anoplophora glabripennis	None
EOG091200G6	APLA011359	Agrilus planipennis	None
EOG091200G6	TC034736	Tribolium castaneum	None
EOG091200G6	LDEC020704	Leptinotarsa decemlineata	None
EOG091200G6	YQE_10154	Dendroctonus ponderosae	None
EOG091200G7	OTAU007454	Onthophagus taurus	Anoctamin

EOG091200G7	AGLA013463	Anoplophora glabripennis	Anoctamin
EOG091200G7	APLA013500	Agrilus planipennis	Anoctamin
EOG091200G7	TC015391	Tribolium castaneum	Anoctamin
EOG091200G7	LDEC021414	Leptinotarsa decemlineata	Anoctamin
EOG091200G7	YQE_08383	Dendroctonus ponderosae	Anoctamin
EOG091200G8	OTAU014076	Onthophagus taurus	Cubitus interruptus
EOG091200G8	AGLA001544	Anoplophora glabripennis	Cubitus interruptus
EOG091200G8	APLA014575	Agrilus planipennis	Cubitus interruptus
EOG091200G8	TC003000	Tribolium castaneum	Cubitus interruptus
EOG091200G8	LDEC013937	Leptinotarsa decemlineata	Cubitus interruptus
EOG091200G8	YQE_04546	Dendroctonus ponderosae	Cubitus interruptus
EOG091200G9	OTAU003558	Onthophagus taurus	Putative uncharacterized protein
EOG091200G9	AGLA016099	Anoplophora glabripennis	Putative uncharacterized protein
EOG091200G9	APLA002644	Agrilus planipennis	Putative uncharacterized protein
EOG091200G9	TC002311	Tribolium castaneum	Putative uncharacterized protein
EOG091200G9	LDEC003847	Leptinotarsa decemlineata	Putative uncharacterized protein
EOG091200G9	YQE_08043	Dendroctonus ponderosae	Putative uncharacterized protein
EOG091200GE	OTAU010616	Onthophagus taurus	transferase activity
EOG091200GE	AGLA020925	Anoplophora glabripennis	transferase activity
EOG091200GE	APLA003060	Agrilus planipennis	transferase activity
EOG091200GE	TC033247	Tribolium castaneum	transferase activity
EOG091200GE	LDEC006516	Leptinotarsa decemlineata	transferase activity
EOG091200GE	YQE_03057	Dendroctonus ponderosae	transferase activity
EOG091200GG	OTAU008815	Onthophagus taurus	DNA binding
EOG091200GG	AGLA001746	Anoplophora glabripennis	DNA binding
EOG091200GG	APLA010749	Agrilus planipennis	DNA binding
EOG091200GG	TC031196	Tribolium castaneum	DNA binding
EOG091200GG	LDEC013672	Leptinotarsa decemlineata	DNA binding
EOG091200GG	YQE_12476	Dendroctonus ponderosae	DNA binding
EOG091200GI	OTAU011024	Onthophagus taurus	Putative uncharacterized protein
EOG091200GI	AGLA014140	Anoplophora glabripennis	Putative uncharacterized protein
EOG091200GI	APLA001524	Agrilus planipennis	Putative uncharacterized protein
EOG091200GI	TC002065	Tribolium castaneum	Putative uncharacterized protein
EOG091200GI	LDEC001695	Leptinotarsa decemlineata	Putative uncharacterized protein
EOG091200GI	YQE_11040	Dendroctonus ponderosae	Putative uncharacterized protein
EOG091200GO	OTAU011782	Onthophagus taurus	Putative uncharacterized protein
EOG091200GO	AGLA012516	Anoplophora glabripennis	Putative uncharacterized protein
EOG091200GO	APLA007154	Agrilus planipennis	Putative uncharacterized protein
EOG091200GO	TC009191	Tribolium castaneum	Putative uncharacterized protein
EOG091200GO	LDEC011997	Leptinotarsa decemlineata	Putative uncharacterized protein
EOG091200GO	YQE_05747	Dendroctonus ponderosae	Putative uncharacterized protein
EOG091200GR	OTAU006017	Onthophagus taurus	Putative uncharacterized protein
EOG091200GR	AGLA001701	Anoplophora glabripennis	Putative uncharacterized protein
EOG091200GR	APLA014035	Agrilus planipennis	Putative uncharacterized protein
EOG091200GR	TC009426	Tribolium castaneum	Putative uncharacterized protein
EOG091200GR	LDEC011860	Leptinotarsa decemlineata	Putative uncharacterized protein
EOG091200GR	YQE_10545	Dendroctonus ponderosae	Putative uncharacterized protein
EOG091200GS	OTAU013405	Onthophagus taurus	Putative uncharacterized protein
EOG091200GS	AGLA019668	Anoplophora glabripennis	Putative uncharacterized protein
EOG091200GS	APLA015092	Agrilus planipennis	Putative uncharacterized protein
EOG091200GS	TC003136	Tribolium castaneum	Putative uncharacterized protein
EOG091200GS	LDEC016777	Leptinotarsa decemlineata	Putative uncharacterized protein
EOG091200GS	YQE_03838	Dendroctonus ponderosae	Putative uncharacterized protein
EOG091200GT	OTAU000571	Onthophagus taurus	Kinesin heavy chain
EOG091200GT	AGLA005913	Anoplophora glabripennis	Kinesin heavy chain
EOG091200GT	APLA000849	Agrilus planipennis	Kinesin heavy chain
EOG091200GT	TC011608	Tribolium castaneum	Kinesin heavy chain
EOG091200GT	LDEC014475	Leptinotarsa decemlineata	Kinesin heavy chain
EOG091200GT	YQE_07950	Dendroctonus ponderosae	Kinesin heavy chain
EOG091200GU	OTAU000496	Onthophagus taurus	Putative uncharacterized protein
EOG091200GU	AGLA010520	Anoplophora glabripennis	Putative uncharacterized protein
EOG091200GU	APLA002320	Agrilus planipennis	Putative uncharacterized protein
EOG091200GU	TC011654	Tribolium castaneum	Putative uncharacterized protein
EOG091200GU	LDEC019622	Leptinotarsa decemlineata	Putative uncharacterized protein
EOG091200GU	YQE_04395	Dendroctonus ponderosae	Putative uncharacterized protein
EOG091200GV	OTAU004738	Onthophagus taurus	Putative uncharacterized protein
EOG091200GV	AGLA001461	Anoplophora glabripennis	Putative uncharacterized protein

EOG091200GV	APLA013270	Agrilus planipennis	Putative uncharacterized protein
EOG091200GV	TC010323	Tribolium castaneum	Putative uncharacterized protein
EOG091200GV	LDEC001490	Leptinotarsa decemlineata	Putative uncharacterized protein
EOG091200GV	YQE_05507	Dendroctonus ponderosae	Putative uncharacterized protein
EOG091200GY	OTAU009747	Onthophagus taurus	Putative uncharacterized protein
EOG091200GY	AGLA016277	Anoplophora glabripennis	Putative uncharacterized protein
EOG091200GY	APLA010884	Agrilus planipennis	Putative uncharacterized protein
EOG091200GY	TC007435	Tribolium castaneum	Putative uncharacterized protein
EOG091200GY	LDEC000714	Leptinotarsa decemlineata	Putative uncharacterized protein
EOG091200GY	YQE_03483	Dendroctonus ponderosae	Putative uncharacterized protein
EOG091200GZ	OTAU008923	Onthophagus taurus	Putative uncharacterized protein
EOG091200GZ	AGLA002147	Anoplophora glabripennis	Putative uncharacterized protein
EOG091200GZ	APLA010460	Agrilus planipennis	Putative uncharacterized protein
EOG091200GZ	TC004675	Tribolium castaneum	Putative uncharacterized protein
EOG091200GZ	LDEC022369	Leptinotarsa decemlineata	Putative uncharacterized protein
EOG091200GZ	YQE_09261	Dendroctonus ponderosae	Putative uncharacterized protein
EOG091200H2	OTAU000919	Onthophagus taurus	Putative uncharacterized protein
EOG091200H2	AGLA011357	Anoplophora glabripennis	Putative uncharacterized protein
EOG091200H2	APLA004138	Agrilus planipennis	Putative uncharacterized protein
EOG091200H2	TC011058	Tribolium castaneum	Putative uncharacterized protein
EOG091200H2	LDEC016479	Leptinotarsa decemlineata	Putative uncharacterized protein
EOG091200H2	YQE_09583	Dendroctonus ponderosae	Putative uncharacterized protein
EOG091200H4	OTAU006663	Onthophagus taurus	Putative uncharacterized protein
EOG091200H4	AGLA002014	Anoplophora glabripennis	Putative uncharacterized protein
EOG091200H4	APLA002824	Agrilus planipennis	Putative uncharacterized protein
EOG091200H4	TC002472	Tribolium castaneum	Putative uncharacterized protein
EOG091200H4	LDEC004584	Leptinotarsa decemlineata	Putative uncharacterized protein
EOG091200H4	YQE_12424	Dendroctonus ponderosae	Putative uncharacterized protein
EOG091200H5	OTAU010842	Onthophagus taurus	Putative uncharacterized protein
EOG091200H5	AGLA016310	Anoplophora glabripennis	Putative uncharacterized protein
EOG091200H5	APLA009492	Agrilus planipennis	Putative uncharacterized protein
EOG091200H5	TC016108	Tribolium castaneum	Putative uncharacterized protein
EOG091200H5	LDEC010330	Leptinotarsa decemlineata	Putative uncharacterized protein
EOG091200H5	YQE_08136	Dendroctonus ponderosae	Putative uncharacterized protein
EOG091200H7	OTAU015930	Onthophagus taurus	Putative uncharacterized protein
EOG091200H7	AGLA018949	Anoplophora glabripennis	Putative uncharacterized protein
EOG091200H7	APLA011749	Agrilus planipennis	Putative uncharacterized protein
EOG091200H7	TC012909	Tribolium castaneum	Putative uncharacterized protein
EOG091200H7	LDEC010547	Leptinotarsa decemlineata	Putative uncharacterized protein
EOG091200H7	YQE_08684	Dendroctonus ponderosae	Putative uncharacterized protein
EOG091200HA	OTAU002967	Onthophagus taurus	Putative uncharacterized protein
EOG091200HA	AGLA012344	Anoplophora glabripennis	Putative uncharacterized protein
EOG091200HA	APLA003482	Agrilus planipennis	Putative uncharacterized protein
EOG091200HA	TC008819	Tribolium castaneum	Putative uncharacterized protein
EOG091200HA	LDEC010665	Leptinotarsa decemlineata	Putative uncharacterized protein
EOG091200HA	YQE_09205	Dendroctonus ponderosae	Putative uncharacterized protein
EOG091200HC	OTAU009390	Onthophagus taurus	Putative uncharacterized protein
EOG091200HC	AGLA008671	Anoplophora glabripennis	Putative uncharacterized protein
EOG091200HC	APLA002531	Agrilus planipennis	Putative uncharacterized protein
EOG091200HC	TC012483	Tribolium castaneum	Putative uncharacterized protein
EOG091200HC	LDEC004135	Leptinotarsa decemlineata	Putative uncharacterized protein
EOG091200HC	YQE_07324	Dendroctonus ponderosae	Putative uncharacterized protein
EOG091200HD	OTAU001973	Onthophagus taurus	DNA-directed RNA polymerase
EOG091200HD	AGLA017626	Anoplophora glabripennis	DNA-directed RNA polymerase
EOG091200HD	APLA005772	Agrilus planipennis	DNA-directed RNA polymerase
EOG091200HD	TC006188	Tribolium castaneum	DNA-directed RNA polymerase
EOG091200HD	LDEC006770	Leptinotarsa decemlineata	DNA-directed RNA polymerase
EOG091200HD	YQE_10187	Dendroctonus ponderosae	DNA-directed RNA polymerase
EOG091200HH	OTAU004329	Onthophagus taurus	Putative uncharacterized protein
EOG091200HH	AGLA015680	Anoplophora glabripennis	Putative uncharacterized protein
EOG091200HH	APLA010439	Agrilus planipennis	Putative uncharacterized protein
EOG091200HH	TC005167	Tribolium castaneum	Putative uncharacterized protein
EOG091200HH	LDEC003526	Leptinotarsa decemlineata	Putative uncharacterized protein
EOG091200HH	YQE_09288	Dendroctonus ponderosae	Putative uncharacterized protein
EOG091200HI	OTAU007649	Onthophagus taurus	Putative uncharacterized protein
EOG091200HI	AGLA003850	Anoplophora glabripennis	Putative uncharacterized protein
EOG091200HI	APLA008815	Agrilus planipennis	Putative uncharacterized protein

EOG091200HI	TC008835	Tribolium castaneum	Putative uncharacterized protein
EOG091200HI	LDEC016664	Leptinotarsa decemlineata	Putative uncharacterized protein
EOG091200HI	YQE_11279	Dendroctonus ponderosae	Putative uncharacterized protein
EOG091200HL	OTAU001503	Onthophagus taurus	Putative uncharacterized protein
EOG091200HL	AGLA002171	Anoplophora glabripennis	Putative uncharacterized protein
EOG091200HL	APLA003663	Agrilus planipennis	Putative uncharacterized protein
EOG091200HL	TC001865	Tribolium castaneum	Putative uncharacterized protein
EOG091200HL	LDEC001516	Leptinotarsa decemlineata	Putative uncharacterized protein
EOG091200HL	YQE_06681	Dendroctonus ponderosae	Putative uncharacterized protein
EOG091200HN	OTAU011130	Onthophagus taurus	Putative uncharacterized protein
EOG091200HN	AGLA007021	Anoplophora glabripennis	Putative uncharacterized protein
EOG091200HN	APLA007550	Agrilus planipennis	Putative uncharacterized protein
EOG091200HN	TC013061	Tribolium castaneum	Putative uncharacterized protein
EOG091200HN	LDEC018787	Leptinotarsa decemlineata	Putative uncharacterized protein
EOG091200HN	YQE_06125	Dendroctonus ponderosae	Putative uncharacterized protein
EOG091200HO	OTAU015900	Onthophagus taurus	Putative uncharacterized protein
EOG091200HO	AGLA000211	Anoplophora glabripennis	Putative uncharacterized protein
EOG091200HO	APLA010011	Agrilus planipennis	Putative uncharacterized protein
EOG091200HO	TC004800	Tribolium castaneum	Putative uncharacterized protein
EOG091200HO	LDEC004516	Leptinotarsa decemlineata	Putative uncharacterized protein
EOG091200HO	YQE_06599	Dendroctonus ponderosae	Putative uncharacterized protein
EOG091200HP	OTAU004725	Onthophagus taurus	Putative uncharacterized protein
EOG091200HP	AGLA007730	Anoplophora glabripennis	Putative uncharacterized protein
EOG091200HP	APLA008396	Agrilus planipennis	Putative uncharacterized protein
EOG091200HP	TC003537	Tribolium castaneum	Putative uncharacterized protein
EOG091200HP	LDEC010296	Leptinotarsa decemlineata	Putative uncharacterized protein
EOG091200HP	YQE_10220	Dendroctonus ponderosae	Putative uncharacterized protein
EOG091200HR	OTAU002806	Onthophagus taurus	None
EOG091200HR	AGLA001175	Anoplophora glabripennis	None
EOG091200HR	APLA012821	Agrilus planipennis	None
EOG091200HR	TC031464	Tribolium castaneum	None
EOG091200HR	LDEC007817	Leptinotarsa decemlineata	None
EOG091200HR	YQE_02447	Dendroctonus ponderosae	None
EOG091200HS	OTAU002533	Onthophagus taurus	Putative uncharacterized protein
EOG091200HS	AGLA004920	Anoplophora glabripennis	Putative uncharacterized protein
EOG091200HS	APLA006952	Agrilus planipennis	Putative uncharacterized protein
EOG091200HS	TC008860	Tribolium castaneum	Putative uncharacterized protein
EOG091200HS	LDEC022408	Leptinotarsa decemlineata	Putative uncharacterized protein
EOG091200HS	YQE_03472	Dendroctonus ponderosae	Putative uncharacterized protein
EOG091200HT	OTAU002280	Onthophagus taurus	Putative uncharacterized protein
EOG091200HT	AGLA018159	Anoplophora glabripennis	Putative uncharacterized protein
EOG091200HT	APLA002501	Agrilus planipennis	Putative uncharacterized protein
EOG091200HT	TC007883	Tribolium castaneum	Putative uncharacterized protein
EOG091200HT	LDEC010935	Leptinotarsa decemlineata	Putative uncharacterized protein
EOG091200HT	YQE_07364	Dendroctonus ponderosae	Putative uncharacterized protein
EOG091200HU	OTAU001429	Onthophagus taurus	Putative uncharacterized protein
EOG091200HU	AGLA000378	Anoplophora glabripennis	Putative uncharacterized protein
EOG091200HU	APLA010456	Agrilus planipennis	Putative uncharacterized protein
EOG091200HU	TC013609	Tribolium castaneum	Putative uncharacterized protein
EOG091200HU	LDEC001562	Leptinotarsa decemlineata	Putative uncharacterized protein
EOG091200HU	YQE_05977	Dendroctonus ponderosae	Putative uncharacterized protein
EOG091200HW	OTAU008888	Onthophagus taurus	Putative uncharacterized protein
EOG091200HW	AGLA011156	Anoplophora glabripennis	Putative uncharacterized protein
EOG091200HW	APLA007893	Agrilus planipennis	Putative uncharacterized protein
EOG091200HW	TC011312	Tribolium castaneum	Putative uncharacterized protein
EOG091200HW	LDEC018095	Leptinotarsa decemlineata	Putative uncharacterized protein
EOG091200HW	YQE_08535	Dendroctonus ponderosae	Putative uncharacterized protein
EOG091200HX	OTAU000561	Onthophagus taurus	Putative uncharacterized protein
EOG091200HX	AGLA017195	Anoplophora glabripennis	Putative uncharacterized protein
EOG091200HX	APLA003245	Agrilus planipennis	Putative uncharacterized protein
EOG091200HX	TC012067	Tribolium castaneum	Putative uncharacterized protein
EOG091200HX	LDEC003864	Leptinotarsa decemlineata	Putative uncharacterized protein
EOG091200HX	YQE_07573	Dendroctonus ponderosae	Putative uncharacterized protein
EOG091200I3	OTAU001821	Onthophagus taurus	Putative uncharacterized protein
EOG091200I3	AGLA004993	Anoplophora glabripennis	Putative uncharacterized protein
EOG091200I3	APLA004508	Agrilus planipennis	Putative uncharacterized protein
EOG091200I3	TC006359	Tribolium castaneum	Putative uncharacterized protein

EOG091200I3	LDEC002993	Leptinotarsa decemlineata	Putative uncharacterized protein
EOG091200I3	YQE_07010	Dendroctonus ponderosae	Putative uncharacterized protein
EOG091200I6	OTAU001625	Onthophagus taurus	Putative uncharacterized protein
EOG091200I6	AGLA005936	Anoplophora glabripennis	Putative uncharacterized protein
EOG091200I6	APLA001179	Agrilus planipennis	Putative uncharacterized protein
EOG091200I6	TC006693	Tribolium castaneum	Putative uncharacterized protein
EOG091200I6	LDEC022078	Leptinotarsa decemlineata	Putative uncharacterized protein
EOG091200I6	YQE_05587	Dendroctonus ponderosae	Putative uncharacterized protein
EOG091200I8	OTAU016060	Onthophagus taurus	Tartan/capricious-like protein
EOG091200I8	AGLA015623	Anoplophora glabripennis	Tartan/capricious-like protein
EOG091200I8	APLA013366	Agrilus planipennis	Tartan/capricious-like protein
EOG091200I8	TC009455	Tribolium castaneum	Tartan/capricious-like protein
EOG091200I8	LDEC008957	Leptinotarsa decemlineata	Tartan/capricious-like protein
EOG091200I8	YQE_03534	Dendroctonus ponderosae	Tartan/capricious-like protein
EOG091200IB	OTAU001672	Onthophagus taurus	Putative uncharacterized protein
EOG091200IB	AGLA010195	Anoplophora glabripennis	Putative uncharacterized protein
EOG091200IB	APLA009856	Agrilus planipennis	Putative uncharacterized protein
EOG091200IB	TC006679	Tribolium castaneum	Putative uncharacterized protein
EOG091200IB	LDEC010122	Leptinotarsa decemlineata	Putative uncharacterized protein
EOG091200IB	YQE_12796	Dendroctonus ponderosae	Putative uncharacterized protein
EOG091200IC	OTAU000909	Onthophagus taurus	Putative uncharacterized protein
EOG091200IC	AGLA000635	Anoplophora glabripennis	Putative uncharacterized protein
EOG091200IC	APLA014588	Agrilus planipennis	Putative uncharacterized protein
EOG091200IC	TC000478	Tribolium castaneum	Putative uncharacterized protein
EOG091200IC	LDEC007055	Leptinotarsa decemlineata	Putative uncharacterized protein
EOG091200IC	YQE_07726	Dendroctonus ponderosae	Putative uncharacterized protein
EOG091200IG	OTAU001629	Onthophagus taurus	Putative uncharacterized protein
EOG091200IG	AGLA011080	Anoplophora glabripennis	Putative uncharacterized protein
EOG091200IG	APLA000960	Agrilus planipennis	Putative uncharacterized protein
EOG091200IG	TC006494	Tribolium castaneum	Putative uncharacterized protein
EOG091200IG	LDEC002920	Leptinotarsa decemlineata	Putative uncharacterized protein
EOG091200IG	YQE_02839	Dendroctonus ponderosae	Putative uncharacterized protein
EOG091200II	OTAU001532	Onthophagus taurus	Putative uncharacterized protein
EOG091200II	AGLA001246	Anoplophora glabripennis	Putative uncharacterized protein
EOG091200II	APLA005754	Agrilus planipennis	Putative uncharacterized protein
EOG091200II	TC006587	Tribolium castaneum	Putative uncharacterized protein
EOG091200II	LDEC011050	Leptinotarsa decemlineata	Putative uncharacterized protein
EOG091200II	YQE_12011	Dendroctonus ponderosae	Putative uncharacterized protein
EOG091200IK	OTAU008206	Onthophagus taurus	Putative uncharacterized protein
EOG091200IK	AGLA000645	Anoplophora glabripennis	Putative uncharacterized protein
EOG091200IK	APLA007970	Agrilus planipennis	Putative uncharacterized protein
EOG091200IK	TC001104	Tribolium castaneum	Putative uncharacterized protein
EOG091200IK	LDEC013068	Leptinotarsa decemlineata	Putative uncharacterized protein
EOG091200IK	YQE_01614	Dendroctonus ponderosae	Putative uncharacterized protein
EOG091200IL	OTAU013976	Onthophagus taurus	Putative uncharacterized protein
EOG091200IL	AGLA002972	Anoplophora glabripennis	Putative uncharacterized protein
EOG091200IL	APLA008598	Agrilus planipennis	Putative uncharacterized protein
EOG091200IL	TC000620	Tribolium castaneum	Putative uncharacterized protein
EOG091200IL	LDEC006249	Leptinotarsa decemlineata	Putative uncharacterized protein
EOG091200IL	YQE_01693	Dendroctonus ponderosae	Putative uncharacterized protein
EOG091200IN	OTAU010513	Onthophagus taurus	peptidase activity
EOG091200IN	AGLA003049	Anoplophora glabripennis	peptidase activity
EOG091200IN	APLA009585	Agrilus planipennis	peptidase activity
EOG091200IN	TC031093	Tribolium castaneum	peptidase activity
EOG091200IN	LDEC008861	Leptinotarsa decemlineata	peptidase activity
EOG091200IN	YQE_03371	Dendroctonus ponderosae	peptidase activity
EOG091200IO	OTAU013362	Onthophagus taurus	Putative uncharacterized protein
EOG091200IO	AGLA012458	Anoplophora glabripennis	Putative uncharacterized protein
EOG091200IO	APLA009441	Agrilus planipennis	Putative uncharacterized protein
EOG091200IO	TC030598	Tribolium castaneum	Putative uncharacterized protein
EOG091200IO	LDEC003195	Leptinotarsa decemlineata	Putative uncharacterized protein
EOG091200IO	YQE_09381	Dendroctonus ponderosae	Putative uncharacterized protein
EOG091200IP	OTAU000412	Onthophagus taurus	Putative uncharacterized protein
EOG091200IP	AGLA012358	Anoplophora glabripennis	Putative uncharacterized protein
EOG091200IP	APLA003192	Agrilus planipennis	Putative uncharacterized protein
EOG091200IP	TC011830	Tribolium castaneum	Putative uncharacterized protein
EOG091200IP	LDEC000531	Leptinotarsa decemlineata	Putative uncharacterized protein

EOG091200IP	YQE_00089	Dendroctonus ponderosae	Putative uncharacterized protein
EOG091200J2	OTAU000908	Onthophagus taurus	Putative uncharacterized protein
EOG091200J2	AGLA001314	Anoplophora glabripennis	Putative uncharacterized protein
EOG091200J2	APLA008581	Agrilus planipennis	Putative uncharacterized protein
EOG091200J2	TC001355	Tribolium castaneum	Putative uncharacterized protein
EOG091200J2	LDEC020605	Leptinotarsa decemlineata	Putative uncharacterized protein
EOG091200J2	YQE_11004	Dendroctonus ponderosae	Putative uncharacterized protein
EOG091200J3	OTAU003347	Onthophagus taurus	Putative uncharacterized protein
EOG091200J3	AGLA015684	Anoplophora glabripennis	Putative uncharacterized protein
EOG091200J3	APLA006371	Agrilus planipennis	Putative uncharacterized protein
EOG091200J3	TC005163	Tribolium castaneum	Putative uncharacterized protein
EOG091200J3	LDEC003522	Leptinotarsa decemlineata	Putative uncharacterized protein
EOG091200J3	YQE_09190	Dendroctonus ponderosae	Putative uncharacterized protein
EOG091200J4	OTAU001573	Onthophagus taurus	Putative uncharacterized protein
EOG091200J4	AGLA012675	Anoplophora glabripennis	Putative uncharacterized protein
EOG091200J4	APLA004477	Agrilus planipennis	Putative uncharacterized protein
EOG091200J4	TC008518	Tribolium castaneum	Putative uncharacterized protein
EOG091200J4	LDEC011065	Leptinotarsa decemlineata	Putative uncharacterized protein
EOG091200J4	YQE_02840	Dendroctonus ponderosae	Putative uncharacterized protein
EOG091200J6	OTAU001495	Onthophagus taurus	None
EOG091200J6	AGLA004547	Anoplophora glabripennis	None
EOG091200J6	APLA005593	Agrilus planipennis	None
EOG091200J6	TC034516	Tribolium castaneum	None
EOG091200J6	LDEC003507	Leptinotarsa decemlineata	None
EOG091200J6	YQE_03290	Dendroctonus ponderosae	None
EOG091200J7	OTAU001220	Onthophagus taurus	Putative uncharacterized protein
EOG091200J7	AGLA013514	Anoplophora glabripennis	Putative uncharacterized protein
EOG091200J7	APLA006385	Agrilus planipennis	Putative uncharacterized protein
EOG091200J7	TC001433	Tribolium castaneum	Putative uncharacterized protein
EOG091200J7	LDEC003538	Leptinotarsa decemlineata	Putative uncharacterized protein
EOG091200J7	YQE_06660	Dendroctonus ponderosae	Putative uncharacterized protein
EOG091200JC	OTAU001886	Onthophagus taurus	None
EOG091200JC	AGLA001658	Anoplophora glabripennis	None
EOG091200JC	APLA000390	Agrilus planipennis	None
EOG091200JC	TC033978	Tribolium castaneum	None
EOG091200JC	LDEC016787	Leptinotarsa decemlineata	None
EOG091200JC	YQE_12703	Dendroctonus ponderosae	None
EOG091200JD	OTAU001076	Onthophagus taurus	Tartan/capricious-like protein
EOG091200JD	AGLA017510	Anoplophora glabripennis	Tartan/capricious-like protein
EOG091200JD	APLA001250	Agrilus planipennis	Tartan/capricious-like protein
EOG091200JD	TC011379	Tribolium castaneum	Tartan/capricious-like protein
EOG091200JD	LDEC017956	Leptinotarsa decemlineata	Tartan/capricious-like protein
EOG091200JD	YQE_04693	Dendroctonus ponderosae	Tartan/capricious-like protein
EOG091200JE	OTAU001365	Onthophagus taurus	phosphatidylinositol 3-kinase regulator activity
EOG091200JE	AGLA000400	Anoplophora glabripennis	phosphatidylinositol 3-kinase regulator activity
EOG091200JE	APLA012421	Agrilus planipennis	phosphatidylinositol 3-kinase regulator activity
EOG091200JE	TC030884	Tribolium castaneum	phosphatidylinositol 3-kinase regulator activity
EOG091200JE	LDEC004700	Leptinotarsa decemlineata	phosphatidylinositol 3-kinase regulator activity
EOG091200JE	YQE_09232	Dendroctonus ponderosae	phosphatidylinositol 3-kinase regulator activity
EOG091200JG	OTAU006622	Onthophagus taurus	Putative uncharacterized protein
EOG091200JG	AGLA019067	Anoplophora glabripennis	Putative uncharacterized protein
EOG091200JG	APLA006672	Agrilus planipennis	Putative uncharacterized protein
EOG091200JG	TC003900	Tribolium castaneum	Putative uncharacterized protein
EOG091200JG	LDEC010291	Leptinotarsa decemlineata	Putative uncharacterized protein
EOG091200JG	YQE_10213	Dendroctonus ponderosae	Putative uncharacterized protein
EOG091200JH	OTAU001314	Onthophagus taurus	Putative uncharacterized protein
EOG091200JH	AGLA002166	Anoplophora glabripennis	Putative uncharacterized protein
EOG091200JH	APLA003681	Agrilus planipennis	Putative uncharacterized protein
EOG091200JH	TC004742	Tribolium castaneum	Putative uncharacterized protein
EOG091200JH	LDEC015950	Leptinotarsa decemlineata	Putative uncharacterized protein
EOG091200JH	YQE_03234	Dendroctonus ponderosae	Putative uncharacterized protein
EOG091200JJ	OTAU011092	Onthophagus taurus	None
EOG091200JJ	AGLA001390	Anoplophora glabripennis	None
EOG091200JJ	APLA007927	Agrilus planipennis	None
EOG091200JJ	TC032169	Tribolium castaneum	None
EOG091200JJ	LDEC013071	Leptinotarsa decemlineata	None
EOG091200JJ	YQE_08506	Dendroctonus ponderosae	None

EOG091200JL	OTAU005298	Onthophagus taurus	Putative uncharacterized protein
EOG091200JL	AGLA012010	Anoplophora glabripennis	Putative uncharacterized protein
EOG091200JL	APLA007608	Agrilus planipennis	Putative uncharacterized protein
EOG091200JL	TC003186	Tribolium castaneum	Putative uncharacterized protein
EOG091200JL	LDEC008763	Leptinotarsa decemlineata	Putative uncharacterized protein
EOG091200JL	YQE_06753	Dendroctonus ponderosae	Putative uncharacterized protein
EOG091200JQ	OTAU001177	Onthophagus taurus	Putative uncharacterized protein
EOG091200JQ	AGLA008108	Anoplophora glabripennis	Putative uncharacterized protein
EOG091200JQ	APLA010748	Agrilus planipennis	Putative uncharacterized protein
EOG091200JQ	TC004261	Tribolium castaneum	Putative uncharacterized protein
EOG091200JQ	LDEC007071	Leptinotarsa decemlineata	Putative uncharacterized protein
EOG091200JQ	YQE_08188	Dendroctonus ponderosae	Putative uncharacterized protein
EOG091200JS	OTAU006434	Onthophagus taurus	Putative uncharacterized protein
EOG091200JS	AGLA006941	Anoplophora glabripennis	Putative uncharacterized protein
EOG091200JS	APLA015316	Agrilus planipennis	Putative uncharacterized protein
EOG091200JS	TC002652	Tribolium castaneum	Putative uncharacterized protein
EOG091200JS	LDEC017765	Leptinotarsa decemlineata	Putative uncharacterized protein
EOG091200JS	YQE_03820	Dendroctonus ponderosae	Putative uncharacterized protein
EOG091200JU	OTAU008613	Onthophagus taurus	DNA topoisomerase
EOG091200JU	AGLA003693	Anoplophora glabripennis	DNA topoisomerase
EOG091200JU	APLA000224	Agrilus planipennis	DNA topoisomerase
EOG091200JU	TC007344	Tribolium castaneum	DNA topoisomerase
EOG091200JU	LDEC000611	Leptinotarsa decemlineata	DNA topoisomerase
EOG091200JU	YQE_11417	Dendroctonus ponderosae	DNA topoisomerase
EOG091200JW	OTAU003966	Onthophagus taurus	zinc ion binding
EOG091200JW	AGLA001034	Anoplophora glabripennis	zinc ion binding
EOG091200JW	APLA010543	Agrilus planipennis	zinc ion binding
EOG091200JW	TC034259	Tribolium castaneum	zinc ion binding
EOG091200JW	LDEC007508	Leptinotarsa decemlineata	zinc ion binding
EOG091200JW	YQE_11979	Dendroctonus ponderosae	zinc ion binding
EOG091200JX	OTAU007951	Onthophagus taurus	Putative uncharacterized protein
EOG091200JX	AGLA006816	Anoplophora glabripennis	Putative uncharacterized protein
EOG091200JX	APLA001576	Agrilus planipennis	Putative uncharacterized protein
EOG091200JX	TC009545	Tribolium castaneum	Putative uncharacterized protein
EOG091200JX	LDEC023241	Leptinotarsa decemlineata	Putative uncharacterized protein
EOG091200JX	YQE_10614	Dendroctonus ponderosae	Putative uncharacterized protein
EOG091200JY	OTAU001290	Onthophagus taurus	Putative uncharacterized protein
EOG091200JY	AGLA004300	Anoplophora glabripennis	Putative uncharacterized protein
EOG091200JY	APLA014548	Agrilus planipennis	Putative uncharacterized protein
EOG091200JY	TC001464	Tribolium castaneum	Putative uncharacterized protein
EOG091200JY	LDEC001116	Leptinotarsa decemlineata	Putative uncharacterized protein
EOG091200JY	YQE_09072	Dendroctonus ponderosae	Putative uncharacterized protein
EOG091200K0	OTAU007133	Onthophagus taurus	binding
EOG091200K0	AGLA009019	Anoplophora glabripennis	binding
EOG091200K0	APLA002220	Agrilus planipennis	binding
EOG091200K0	TC032790	Tribolium castaneum	binding
EOG091200K0	LDEC020644	Leptinotarsa decemlineata	binding
EOG091200K0	YQE_11990	Dendroctonus ponderosae	binding
EOG091200K2	OTAU006019	Onthophagus taurus	Putative uncharacterized protein
EOG091200K2	AGLA012283	Anoplophora glabripennis	Putative uncharacterized protein
EOG091200K2	APLA014775	Agrilus planipennis	Putative uncharacterized protein
EOG091200K2	TC009571	Tribolium castaneum	Putative uncharacterized protein
EOG091200K2	LDEC004070	Leptinotarsa decemlineata	Putative uncharacterized protein
EOG091200K2	YQE_03506	Dendroctonus ponderosae	Putative uncharacterized protein
EOG091200K4	OTAU014029	Onthophagus taurus	None
EOG091200K4	AGLA005446	Anoplophora glabripennis	None
EOG091200K4	APLA001077	Agrilus planipennis	None
EOG091200K4	TC034892	Tribolium castaneum	None
EOG091200K4	LDEC018187	Leptinotarsa decemlineata	None
EOG091200K4	YQE_07619	Dendroctonus ponderosae	None
EOG091200K5	OTAU007420	Onthophagus taurus	Putative uncharacterized protein
EOG091200K5	AGLA018081	Anoplophora glabripennis	Putative uncharacterized protein
EOG091200K5	APLA008012	Agrilus planipennis	Putative uncharacterized protein
EOG091200K5	TC000481	Tribolium castaneum	Putative uncharacterized protein
EOG091200K5	LDEC006869	Leptinotarsa decemlineata	Putative uncharacterized protein
EOG091200K5	YQE_04569	Dendroctonus ponderosae	Putative uncharacterized protein
EOG091200K8	OTAU016172	Onthophagus taurus	Putative uncharacterized protein

EOG091200K8	AGLA002580	Anoplophora glabripennis	Putative uncharacterized protein
EOG091200K8	APLA008951	Agrilus planipennis	Putative uncharacterized protein
EOG091200K8	TC004369	Tribolium castaneum	Putative uncharacterized protein
EOG091200K8	LDEC008090	Leptinotarsa decemlineata	Putative uncharacterized protein
EOG091200K8	YQE_03038	Dendroctonus ponderosae	Putative uncharacterized protein
EOG091200KA	OTAU002462	Onthophagus taurus	Putative uncharacterized protein
EOG091200KA	AGLA002085	Anoplophora glabripennis	Putative uncharacterized protein
EOG091200KA	APLA009703	Agrilus planipennis	Putative uncharacterized protein
EOG091200KA	TC002825	Tribolium castaneum	Putative uncharacterized protein
EOG091200KA	LDEC010197	Leptinotarsa decemlineata	Putative uncharacterized protein
EOG091200KA	YQE_07835	Dendroctonus ponderosae	Putative uncharacterized protein
EOG091200KC	OTAU000075	Onthophagus taurus	None
EOG091200KC	AGLA006982	Anoplophora glabripennis	None
EOG091200KC	APLA006161	Agrilus planipennis	None
EOG091200KC	TC033026	Tribolium castaneum	None
EOG091200KC	LDEC010816	Leptinotarsa decemlineata	None
EOG091200KC	YQE_06291	Dendroctonus ponderosae	None
EOG091200KD	OTAU002502	Onthophagus taurus	Putative uncharacterized protein
EOG091200KD	AGLA016069	Anoplophora glabripennis	Putative uncharacterized protein
EOG091200KD	APLA004751	Agrilus planipennis	Putative uncharacterized protein
EOG091200KD	TC009066	Tribolium castaneum	Putative uncharacterized protein
EOG091200KD	LDEC005816	Leptinotarsa decemlineata	Putative uncharacterized protein
EOG091200KD	YQE_04111	Dendroctonus ponderosae	Putative uncharacterized protein
EOG091200KF	OTAU013188	Onthophagus taurus	Putative uncharacterized protein
EOG091200KF	AGLA006351	Anoplophora glabripennis	Putative uncharacterized protein
EOG091200KF	APLA014379	Agrilus planipennis	Putative uncharacterized protein
EOG091200KF	TC015731	Tribolium castaneum	Putative uncharacterized protein
EOG091200KF	LDEC021621	Leptinotarsa decemlineata	Putative uncharacterized protein
EOG091200KF	YQE_05954	Dendroctonus ponderosae	Putative uncharacterized protein
EOG091200KH	OTAU000008	Onthophagus taurus	Putative uncharacterized protein
EOG091200KH	AGLA005486	Anoplophora glabripennis	Putative uncharacterized protein
EOG091200KH	APLA007269	Agrilus planipennis	Putative uncharacterized protein
EOG091200KH	TC013520	Tribolium castaneum	Putative uncharacterized protein
EOG091200KH	LDEC008333	Leptinotarsa decemlineata	Putative uncharacterized protein
EOG091200KH	YQE_06282	Dendroctonus ponderosae	Putative uncharacterized protein
EOG091200KI	OTAU015413	Onthophagus taurus	Putative uncharacterized protein
EOG091200KI	AGLA018025	Anoplophora glabripennis	Putative uncharacterized protein
EOG091200KI	APLA005950	Agrilus planipennis	Putative uncharacterized protein
EOG091200KI	TC015614	Tribolium castaneum	Putative uncharacterized protein
EOG091200KI	LDEC014931	Leptinotarsa decemlineata	Putative uncharacterized protein
EOG091200KI	YQE_11459	Dendroctonus ponderosae	Putative uncharacterized protein
EOG091200KK	OTAU015004	Onthophagus taurus	Putative uncharacterized protein
EOG091200KK	AGLA019158	Anoplophora glabripennis	Putative uncharacterized protein
EOG091200KK	APLA013801	Agrilus planipennis	Putative uncharacterized protein
EOG091200KK	TC002936	Tribolium castaneum	Putative uncharacterized protein
EOG091200KK	LDEC020371	Leptinotarsa decemlineata	Putative uncharacterized protein
EOG091200KK	YQE_11841	Dendroctonus ponderosae	Putative uncharacterized protein
EOG091200KM	OTAU001253	Onthophagus taurus	Putative uncharacterized protein
EOG091200KM	AGLA007622	Anoplophora glabripennis	Putative uncharacterized protein
EOG091200KM	APLA015441	Agrilus planipennis	Putative uncharacterized protein
EOG091200KM	TC001623	Tribolium castaneum	Putative uncharacterized protein
EOG091200KM	LDEC011168	Leptinotarsa decemlineata	Putative uncharacterized protein
EOG091200KM	YQE_06647	Dendroctonus ponderosae	Putative uncharacterized protein
EOG091200KO	OTAU009263	Onthophagus taurus	Putative uncharacterized protein
EOG091200KO	AGLA006460	Anoplophora glabripennis	Putative uncharacterized protein
EOG091200KO	APLA008823	Agrilus planipennis	Putative uncharacterized protein
EOG091200KO	TC009846	Tribolium castaneum	Putative uncharacterized protein
EOG091200KO	LDEC004799	Leptinotarsa decemlineata	Putative uncharacterized protein
EOG091200KO	YQE_02584	Dendroctonus ponderosae	Putative uncharacterized protein
EOG091200KP	OTAU004582	Onthophagus taurus	Putative uncharacterized protein
EOG091200KP	AGLA000782	Anoplophora glabripennis	Putative uncharacterized protein
EOG091200KP	APLA009056	Agrilus planipennis	Putative uncharacterized protein
EOG091200KP	TC007684	Tribolium castaneum	Putative uncharacterized protein
EOG091200KP	LDEC020650	Leptinotarsa decemlineata	Putative uncharacterized protein
EOG091200KP	YQE_09698	Dendroctonus ponderosae	Putative uncharacterized protein
EOG091200KR	OTAU011838	Onthophagus taurus	protein tyrosine phosphatase activity
EOG091200KR	AGLA012392	Anoplophora glabripennis	protein tyrosine phosphatase activity

EOG091200KR	APLA009889	Agrilus planipennis	protein tyrosine phosphatase activity
EOG091200KR	TC032131	Tribolium castaneum	protein tyrosine phosphatase activity
EOG091200KR	LDEC010167	Leptinotarsa decemlineata	protein tyrosine phosphatase activity
EOG091200KR	YQE_09597	Dendroctonus ponderosae	protein tyrosine phosphatase activity
EOG091200KS	OTAU009343	Onthophagus taurus	Putative uncharacterized protein
EOG091200KS	AGLA010981	Anoplophora glabripennis	Putative uncharacterized protein
EOG091200KS	APLA002863	Agrilus planipennis	Putative uncharacterized protein
EOG091200KS	TC013876	Tribolium castaneum	Putative uncharacterized protein
EOG091200KS	LDEC009388	Leptinotarsa decemlineata	Putative uncharacterized protein
EOG091200KS	YQE_07267	Dendroctonus ponderosae	Putative uncharacterized protein
EOG091200KU	OTAU004381	Onthophagus taurus	Putative uncharacterized protein
EOG091200KU	AGLA000429	Anoplophora glabripennis	Putative uncharacterized protein
EOG091200KU	APLA006741	Agrilus planipennis	Putative uncharacterized protein
EOG091200KU	TC001640	Tribolium castaneum	Putative uncharacterized protein
EOG091200KU	LDEC019095	Leptinotarsa decemlineata	Putative uncharacterized protein
EOG091200KU	YQE_06634	Dendroctonus ponderosae	Putative uncharacterized protein
EOG091200KW	OTAU005770	Onthophagus taurus	Putative uncharacterized protein
EOG091200KW	AGLA006226	Anoplophora glabripennis	Putative uncharacterized protein
EOG091200KW	APLA014846	Agrilus planipennis	Putative uncharacterized protein
EOG091200KW	TC004018	Tribolium castaneum	Putative uncharacterized protein
EOG091200KW	LDEC013127	Leptinotarsa decemlineata	Putative uncharacterized protein
EOG091200KW	YQE_05872	Dendroctonus ponderosae	Putative uncharacterized protein
EOG091200KY	OTAU005893	Onthophagus taurus	Putative uncharacterized protein
EOG091200KY	AGLA020114	Anoplophora glabripennis	Putative uncharacterized protein
EOG091200KY	APLA010842	Agrilus planipennis	Putative uncharacterized protein
EOG091200KY	TC013922	Tribolium castaneum	Putative uncharacterized protein
EOG091200KY	LDEC007759	Leptinotarsa decemlineata	Putative uncharacterized protein
EOG091200KY	YQE_07285	Dendroctonus ponderosae	Putative uncharacterized protein
EOG091200KZ	OTAU002744	Onthophagus taurus	Delta-like protein
EOG091200KZ	AGLA002187	Anoplophora glabripennis	Delta-like protein
EOG091200KZ	APLA009966	Agrilus planipennis	Delta-like protein
EOG091200KZ	TC004114	Tribolium castaneum	Delta-like protein
EOG091200KZ	LDEC011135	Leptinotarsa decemlineata	Delta-like protein
EOG091200KZ	YQE_06648	Dendroctonus ponderosae	Delta-like protein
EOG091200L1	OTAU000762	Onthophagus taurus	Tyrosine-protein kinase receptor
EOG091200L1	AGLA000591	Anoplophora glabripennis	Tyrosine-protein kinase receptor
EOG091200L1	APLA014318	Agrilus planipennis	Tyrosine-protein kinase receptor
EOG091200L1	TC002114	Tribolium castaneum	Tyrosine-protein kinase receptor
EOG091200L1	LDEC006995	Leptinotarsa decemlineata	Tyrosine-protein kinase receptor
EOG091200L1	YQE_03800	Dendroctonus ponderosae	Tyrosine-protein kinase receptor
EOG091200L3	OTAU010966	Onthophagus taurus	calcium ion binding
EOG091200L3	AGLA006910	Anoplophora glabripennis	calcium ion binding
EOG091200L3	APLA005448	Agrilus planipennis	calcium ion binding
EOG091200L3	TC032589	Tribolium castaneum	calcium ion binding
EOG091200L3	LDEC019427	Leptinotarsa decemlineata	calcium ion binding
EOG091200L3	YQE_10191	Dendroctonus ponderosae	calcium ion binding
EOG091200L4	OTAU016159	Onthophagus taurus	Putative uncharacterized protein
EOG091200L4	AGLA010333	Anoplophora glabripennis	Putative uncharacterized protein
EOG091200L4	APLA011532	Agrilus planipennis	Putative uncharacterized protein
EOG091200L4	TC011200	Tribolium castaneum	Putative uncharacterized protein
EOG091200L4	LDEC008263	Leptinotarsa decemlineata	Putative uncharacterized protein
EOG091200L4	YQE_08377	Dendroctonus ponderosae	Putative uncharacterized protein
EOG091200L8	OTAU002578	Onthophagus taurus	anion transmembrane transporter activity
EOG091200L8	AGLA011233	Anoplophora glabripennis	anion transmembrane transporter activity
EOG091200L8	APLA015314	Agrilus planipennis	anion transmembrane transporter activity
EOG091200L8	TC033053	Tribolium castaneum	anion transmembrane transporter activity
EOG091200L8	LDEC002626	Leptinotarsa decemlineata	anion transmembrane transporter activity
EOG091200L8	YQE_07552	Dendroctonus ponderosae	anion transmembrane transporter activity
EOG091200LA	OTAU002789	Onthophagus taurus	Disco-related
EOG091200LA	AGLA008327	Anoplophora glabripennis	Disco-related
EOG091200LA	APLA013966	Agrilus planipennis	Disco-related
EOG091200LA	TC001693	Tribolium castaneum	Disco-related
EOG091200LA	LDEC012715	Leptinotarsa decemlineata	Disco-related
EOG091200LA	YQE_09276	Dendroctonus ponderosae	Disco-related
EOG091200LD	OTAU005978	Onthophagus taurus	Putative uncharacterized protein
EOG091200LD	AGLA009169	Anoplophora glabripennis	Putative uncharacterized protein
EOG091200LD	APLA002049	Agrilus planipennis	Putative uncharacterized protein

EOG091200LD	TC011526	Tribolium castaneum	Putative uncharacterized protein
EOG091200LD	LDEC010801	Leptinotarsa decemlineata	Putative uncharacterized protein
EOG091200LD	YQE_11064	Dendroctonus ponderosae	Putative uncharacterized protein
EOG091200LE	OTAU004330	Onthophagus taurus	Putative uncharacterized protein
EOG091200LE	AGLA000206	Anoplophora glabripennis	Putative uncharacterized protein
EOG091200LE	APLA005609	Agrilus planipennis	Putative uncharacterized protein
EOG091200LE	TC010878	Tribolium castaneum	Putative uncharacterized protein
EOG091200LE	LDEC011427	Leptinotarsa decemlineata	Putative uncharacterized protein
EOG091200LE	YQE_06679	Dendroctonus ponderosae	Putative uncharacterized protein
EOG091200LF	OTAU003031	Onthophagus taurus	zinc ion binding
EOG091200LF	AGLA003655	Anoplophora glabripennis	zinc ion binding
EOG091200LF	APLA001000	Agrilus planipennis	zinc ion binding
EOG091200LF	TC034974	Tribolium castaneum	zinc ion binding
EOG091200LF	LDEC005329	Leptinotarsa decemlineata	zinc ion binding
EOG091200LF	YQE_05193	Dendroctonus ponderosae	zinc ion binding
EOG091200LI	OTAU001398	Onthophagus taurus	Putative uncharacterized protein
EOG091200LI	AGLA001080	Anoplophora glabripennis	Putative uncharacterized protein
EOG091200LI	APLA005586	Agrilus planipennis	Putative uncharacterized protein
EOG091200LI	TC004683	Tribolium castaneum	Putative uncharacterized protein
EOG091200LI	LDEC020746	Leptinotarsa decemlineata	Putative uncharacterized protein
EOG091200LI	YQE_11873	Dendroctonus ponderosae	Putative uncharacterized protein
EOG091200LJ	OTAU012817	Onthophagus taurus	Putative uncharacterized protein
EOG091200LJ	AGLA003946	Anoplophora glabripennis	Putative uncharacterized protein
EOG091200LJ	APLA003829	Agrilus planipennis	Putative uncharacterized protein
EOG091200LJ	TC015021	Tribolium castaneum	Putative uncharacterized protein
EOG091200LJ	LDEC012252	Leptinotarsa decemlineata	Putative uncharacterized protein
EOG091200LJ	YQE_05952	Dendroctonus ponderosae	Putative uncharacterized protein
EOG091200LL	OTAU012982	Onthophagus taurus	Putative uncharacterized protein
EOG091200LL	AGLA002029	Anoplophora glabripennis	Putative uncharacterized protein
EOG091200LL	APLA009710	Agrilus planipennis	Putative uncharacterized protein
EOG091200LL	TC005854	Tribolium castaneum	Putative uncharacterized protein
EOG091200LL	LDEC013419	Leptinotarsa decemlineata	Putative uncharacterized protein
EOG091200LL	YQE_02651	Dendroctonus ponderosae	Putative uncharacterized protein
EOG091200LM	OTAU013609	Onthophagus taurus	Putative uncharacterized protein
EOG091200LM	AGLA006852	Anoplophora glabripennis	Putative uncharacterized protein
EOG091200LM	APLA009530	Agrilus planipennis	Putative uncharacterized protein
EOG091200LM	TC012655	Tribolium castaneum	Putative uncharacterized protein
EOG091200LM	LDEC004174	Leptinotarsa decemlineata	Putative uncharacterized protein
EOG091200LM	YQE_04388	Dendroctonus ponderosae	Putative uncharacterized protein
EOG091200LN	OTAU002882	Onthophagus taurus	Putative uncharacterized protein
EOG091200LN	AGLA020571	Anoplophora glabripennis	Putative uncharacterized protein
EOG091200LN	APLA012586	Agrilus planipennis	Putative uncharacterized protein
EOG091200LN	TC030765	Tribolium castaneum	Putative uncharacterized protein
EOG091200LN	LDEC013876	Leptinotarsa decemlineata	Putative uncharacterized protein
EOG091200LN	YQE_10292	Dendroctonus ponderosae	Putative uncharacterized protein
EOG091200LO	OTAU016393	Onthophagus taurus	Putative uncharacterized protein
EOG091200LO	AGLA012357	Anoplophora glabripennis	Putative uncharacterized protein
EOG091200LO	APLA001388	Agrilus planipennis	Putative uncharacterized protein
EOG091200LO	TC011437	Tribolium castaneum	Putative uncharacterized protein
EOG091200LO	LDEC013920	Leptinotarsa decemlineata	Putative uncharacterized protein
EOG091200LO	YQE_07124	Dendroctonus ponderosae	Putative uncharacterized protein
EOG091200LP	OTAU003338	Onthophagus taurus	protein tyrosine phosphatase activity
EOG091200LP	AGLA015696	Anoplophora glabripennis	protein tyrosine phosphatase activity
EOG091200LP	APLA015278	Agrilus planipennis	protein tyrosine phosphatase activity
EOG091200LP	TC031516	Tribolium castaneum	protein tyrosine phosphatase activity
EOG091200LP	LDEC001082	Leptinotarsa decemlineata	protein tyrosine phosphatase activity
EOG091200LP	YQE_06606	Dendroctonus ponderosae	protein tyrosine phosphatase activity
EOG091200LQ	OTAU002942	Onthophagus taurus	Putative uncharacterized protein
EOG091200LQ	AGLA010055	Anoplophora glabripennis	Putative uncharacterized protein
EOG091200LQ	APLA001819	Agrilus planipennis	Putative uncharacterized protein
EOG091200LQ	TC009506	Tribolium castaneum	Putative uncharacterized protein
EOG091200LQ	LDEC012836	Leptinotarsa decemlineata	Putative uncharacterized protein
EOG091200LQ	YQE_12659	Dendroctonus ponderosae	Putative uncharacterized protein
EOG091200LS	OTAU002265	Onthophagus taurus	Putative uncharacterized protein
EOG091200LS	AGLA019519	Anoplophora glabripennis	Putative uncharacterized protein
EOG091200LS	APLA014656	Agrilus planipennis	Putative uncharacterized protein
EOG091200LS	TC001850	Tribolium castaneum	Putative uncharacterized protein

EOG091200LS	LDEC011919	Leptinotarsa decemlineata	Putative uncharacterized protein
EOG091200LS	YQE_04667	Dendroctonus ponderosae	Putative uncharacterized protein
EOG091200LY	OTAU002786	Onthophagus taurus	Capicua
EOG091200LY	AGLA000325	Anoplophora glabripennis	Capicua
EOG091200LY	APLA006356	Agrilus planipennis	Capicua
EOG091200LY	TC004697	Tribolium castaneum	Capicua
EOG091200LY	LDEC018997	Leptinotarsa decemlineata	Capicua
EOG091200LY	YQE_03282	Dendroctonus ponderosae	Capicua
EOG091200M0	OTAU002227	Onthophagus taurus	Putative uncharacterized protein
EOG091200M0	AGLA021096	Anoplophora glabripennis	Putative uncharacterized protein
EOG091200M0	APLA010553	Agrilus planipennis	Putative uncharacterized protein
EOG091200M0	TC012660	Tribolium castaneum	Putative uncharacterized protein
EOG091200M0	LDEC000523	Leptinotarsa decemlineata	Putative uncharacterized protein
EOG091200M0	YQE_07972	Dendroctonus ponderosae	Putative uncharacterized protein
EOG091200M1	OTAU007504	Onthophagus taurus	Putative uncharacterized protein
EOG091200M1	AGLA019229	Anoplophora glabripennis	Putative uncharacterized protein
EOG091200M1	APLA005942	Agrilus planipennis	Putative uncharacterized protein
EOG091200M1	TC015204	Tribolium castaneum	Putative uncharacterized protein
EOG091200M1	LDEC023008	Leptinotarsa decemlineata	Putative uncharacterized protein
EOG091200M1	YQE_02113	Dendroctonus ponderosae	Putative uncharacterized protein
EOG091200M3	OTAU011885	Onthophagus taurus	E3 ubiquitin-protein ligase
EOG091200M3	AGLA020244	Anoplophora glabripennis	E3 ubiquitin-protein ligase
EOG091200M3	APLA006851	Agrilus planipennis	E3 ubiquitin-protein ligase
EOG091200M3	TC014416	Tribolium castaneum	E3 ubiquitin-protein ligase
EOG091200M3	LDEC002628	Leptinotarsa decemlineata	E3 ubiquitin-protein ligase
EOG091200M3	YQE_07286	Dendroctonus ponderosae	E3 ubiquitin-protein ligase
EOG091200M4	OTAU012527	Onthophagus taurus	Putative uncharacterized protein
EOG091200M4	AGLA012880	Anoplophora glabripennis	Putative uncharacterized protein
EOG091200M4	APLA002989	Agrilus planipennis	Putative uncharacterized protein
EOG091200M4	TC000847	Tribolium castaneum	Putative uncharacterized protein
EOG091200M4	LDEC020712	Leptinotarsa decemlineata	Putative uncharacterized protein
EOG091200M4	YQE_10425	Dendroctonus ponderosae	Putative uncharacterized protein
EOG091200M7	OTAU004903	Onthophagus taurus	Putative uncharacterized protein
EOG091200M7	AGLA000124	Anoplophora glabripennis	Putative uncharacterized protein
EOG091200M7	APLA009898	Agrilus planipennis	Putative uncharacterized protein
EOG091200M7	TC004850	Tribolium castaneum	Putative uncharacterized protein
EOG091200M7	LDEC008041	Leptinotarsa decemlineata	Putative uncharacterized protein
EOG091200M7	YQE_08066	Dendroctonus ponderosae	Putative uncharacterized protein
EOG091200M8	OTAU001586	Onthophagus taurus	phosphoribosylformylglycinamidine cyclo-ligase activity
EOG091200M8	AGLA004972	Anoplophora glabripennis	phosphoribosylformylglycinamidine cyclo-ligase activity
EOG091200M8	APLA005811	Agrilus planipennis	phosphoribosylformylglycinamidine cyclo-ligase activity
EOG091200M8	TC034834	Tribolium castaneum	phosphoribosylformylglycinamidine cyclo-ligase activity
EOG091200M8	LDEC003012	Leptinotarsa decemlineata	phosphoribosylformylglycinamidine cyclo-ligase activity
EOG091200M8	YQE_07026	Dendroctonus ponderosae	phosphoribosylformylglycinamidine cyclo-ligase activity
EOG091200M9	OTAU005779	Onthophagus taurus	binding
EOG091200M9	AGLA020911	Anoplophora glabripennis	binding
EOG091200M9	APLA012471	Agrilus planipennis	binding
EOG091200M9	TC034427	Tribolium castaneum	binding
EOG091200M9	LDEC000480	Leptinotarsa decemlineata	binding
EOG091200M9	YQE_10164	Dendroctonus ponderosae	binding
EOG091200MB	OTAU007493	Onthophagus taurus	Putative uncharacterized protein
EOG091200MB	AGLA003331	Anoplophora glabripennis	Putative uncharacterized protein
EOG091200MB	APLA014380	Agrilus planipennis	Putative uncharacterized protein
EOG091200MB	TC014966	Tribolium castaneum	Putative uncharacterized protein
EOG091200MB	LDEC007888	Leptinotarsa decemlineata	Putative uncharacterized protein
EOG091200MB	YQE_02140	Dendroctonus ponderosae	Putative uncharacterized protein
EOG091200MC	OTAU009242	Onthophagus taurus	ligase activity
EOG091200MC	AGLA006377	Anoplophora glabripennis	ligase activity
EOG091200MC	APLA011325	Agrilus planipennis	ligase activity
EOG091200MC	TC033023	Tribolium castaneum	ligase activity
EOG091200MC	LDEC007271	Leptinotarsa decemlineata	ligase activity

EOG091200MC	YQE_02099	Dendroctonus ponderosae	ligase activity
EOG091200MD	OTAU013127	Onthophagus taurus	Putative uncharacterized protein
EOG091200MD	AGLA009874	Anoplophora glabripennis	Putative uncharacterized protein
EOG091200MD	APLA012298	Agrilus planipennis	Putative uncharacterized protein
EOG091200MD	TC005933	Tribolium castaneum	Putative uncharacterized protein
EOG091200MD	LDEC018632	Leptinotarsa decemlineata	Putative uncharacterized protein
EOG091200MD	YQE_12246	Dendroctonus ponderosae	Putative uncharacterized protein
EOG091200ME	OTAU003490	Onthophagus taurus	Putative uncharacterized protein
EOG091200ME	AGLA012506	Anoplophora glabripennis	Putative uncharacterized protein
EOG091200ME	APLA001589	Agrilus planipennis	Putative uncharacterized protein
EOG091200ME	TC008602	Tribolium castaneum	Putative uncharacterized protein
EOG091200ME	LDEC010012	Leptinotarsa decemlineata	Putative uncharacterized protein
EOG091200ME	YQE_02348	Dendroctonus ponderosae	Putative uncharacterized protein
EOG091200MH	OTAU010395	Onthophagus taurus	Putative uncharacterized protein
EOG091200MH	AGLA018166	Anoplophora glabripennis	Putative uncharacterized protein
EOG091200MH	APLA008102	Agrilus planipennis	Putative uncharacterized protein
EOG091200MH	TC010433	Tribolium castaneum	Putative uncharacterized protein
EOG091200MH	LDEC020713	Leptinotarsa decemlineata	Putative uncharacterized protein
EOG091200MH	YQE_04046	Dendroctonus ponderosae	Putative uncharacterized protein
EOG091200MI	OTAU002069	Onthophagus taurus	calcium ion binding
EOG091200MI	AGLA005554	Anoplophora glabripennis	calcium ion binding
EOG091200MI	APLA010563	Agrilus planipennis	calcium ion binding
EOG091200MI	TC034295	Tribolium castaneum	calcium ion binding
EOG091200MI	LDEC014703	Leptinotarsa decemlineata	calcium ion binding
EOG091200MI	YQE_07947	Dendroctonus ponderosae	calcium ion binding
EOG091200ML	OTAU004352	Onthophagus taurus	Gliotactin
EOG091200ML	AGLA012201	Anoplophora glabripennis	Gliotactin
EOG091200ML	APLA006781	Agrilus planipennis	Gliotactin
EOG091200ML	TC010824	Tribolium castaneum	Gliotactin
EOG091200ML	LDEC003533	Leptinotarsa decemlineata	Gliotactin
EOG091200ML	YQE_09110	Dendroctonus ponderosae	Gliotactin
EOG091200MM	OTAU006795	Onthophagus taurus	Rad50
EOG091200MM	AGLA008274	Anoplophora glabripennis	Rad50
EOG091200MM	APLA006643	Agrilus planipennis	Rad50
EOG091200MM	TC015093	Tribolium castaneum	Rad50
EOG091200MM	LDEC001276	Leptinotarsa decemlineata	Rad50
EOG091200MM	YQE_11919	Dendroctonus ponderosae	Rad50
EOG091200MN	OTAU005721	Onthophagus taurus	metallopeptidase activity
EOG091200MN	AGLA014904	Anoplophora glabripennis	metallopeptidase activity
EOG091200MN	APLA005178	Agrilus planipennis	metallopeptidase activity
EOG091200MN	TC031122	Tribolium castaneum	metallopeptidase activity
EOG091200MN	LDEC016711	Leptinotarsa decemlineata	metallopeptidase activity
EOG091200MN	YQE_06431	Dendroctonus ponderosae	metallopeptidase activity
EOG091200MP	OTAU003385	Onthophagus taurus	ATP binding
EOG091200MP	AGLA004401	Anoplophora glabripennis	ATP binding
EOG091200MP	APLA003267	Agrilus planipennis	ATP binding
EOG091200MP	TC034444	Tribolium castaneum	ATP binding
EOG091200MP	LDEC014876	Leptinotarsa decemlineata	ATP binding
EOG091200MP	YQE_12895	Dendroctonus ponderosae	ATP binding
EOG091200MQ	OTAU000284	Onthophagus taurus	None
EOG091200MQ	AGLA006073	Anoplophora glabripennis	None
EOG091200MQ	APLA007357	Agrilus planipennis	None
EOG091200MQ	TC033105	Tribolium castaneum	None
EOG091200MQ	LDEC013732	Leptinotarsa decemlineata	None
EOG091200MQ	YQE_10670	Dendroctonus ponderosae	None
EOG091200MR	OTAU008680	Onthophagus taurus	None
EOG091200MR	AGLA009498	Anoplophora glabripennis	None
EOG091200MR	APLA008478	Agrilus planipennis	None
EOG091200MR	TC034193	Tribolium castaneum	None
EOG091200MR	LDEC014346	Leptinotarsa decemlineata	None
EOG091200MR	YQE_12229	Dendroctonus ponderosae	None
EOG091200MT	OTAU000383	Onthophagus taurus	Putative uncharacterized protein
EOG091200MT	AGLA015996	Anoplophora glabripennis	Putative uncharacterized protein
EOG091200MT	APLA014327	Agrilus planipennis	Putative uncharacterized protein
EOG091200MT	TC011842	Tribolium castaneum	Putative uncharacterized protein
EOG091200MT	LDEC002079	Leptinotarsa decemlineata	Putative uncharacterized protein
EOG091200MT	YQE_07979	Dendroctonus ponderosae	Putative uncharacterized protein

EOG091200MZ	OTAU011713	Onthophagus taurus	Putative uncharacterized protein
EOG091200MZ	AGLA005896	Anoplophora glabripennis	Putative uncharacterized protein
EOG091200MZ	APLA000686	Agrilus planipennis	Putative uncharacterized protein
EOG091200MZ	TC015680	Tribolium castaneum	Putative uncharacterized protein
EOG091200MZ	LDEC008288	Leptinotarsa decemlineata	Putative uncharacterized protein
EOG091200MZ	YQE_08346	Dendroctonus ponderosae	Putative uncharacterized protein
EOG091200N0	OTAU004154	Onthophagus taurus	Prospero
EOG091200N0	AGLA010384	Anoplophora glabripennis	Prospero
EOG091200N0	APLA005692	Agrilus planipennis	Prospero
EOG091200N0	TC010596	Tribolium castaneum	Prospero
EOG091200N0	LDEC004085	Leptinotarsa decemlineata	Prospero
EOG091200N0	YQE_10516	Dendroctonus ponderosae	Prospero
EOG091200N1	OTAU006338	Onthophagus taurus	Putative uncharacterized protein
EOG091200N1	AGLA000138	Anoplophora glabripennis	Putative uncharacterized protein
EOG091200N1	APLA012674	Agrilus planipennis	Putative uncharacterized protein
EOG091200N1	TC000163	Tribolium castaneum	Putative uncharacterized protein
EOG091200N1	LDEC016697	Leptinotarsa decemlineata	Putative uncharacterized protein
EOG091200N1	YQE_01609	Dendroctonus ponderosae	Putative uncharacterized protein
EOG091200N2	OTAU014745	Onthophagus taurus	Ribonucleoside-diphosphate reductase
EOG091200N2	AGLA014831	Anoplophora glabripennis	Ribonucleoside-diphosphate reductase
EOG091200N2	APLA012574	Agrilus planipennis	Ribonucleoside-diphosphate reductase
EOG091200N2	TC007859	Tribolium castaneum	Ribonucleoside-diphosphate reductase
EOG091200N2	LDEC004606	Leptinotarsa decemlineata	Ribonucleoside-diphosphate reductase
EOG091200N2	YQE_04636	Dendroctonus ponderosae	Ribonucleoside-diphosphate reductase
EOG091200N3	OTAU015243	Onthophagus taurus	Putative uncharacterized protein
EOG091200N3	AGLA008069	Anoplophora glabripennis	Putative uncharacterized protein
EOG091200N3	APLA009462	Agrilus planipennis	Putative uncharacterized protein
EOG091200N3	TC015727	Tribolium castaneum	Putative uncharacterized protein
EOG091200N3	LDEC005185	Leptinotarsa decemlineata	Putative uncharacterized protein
EOG091200N3	YQE_11737	Dendroctonus ponderosae	Putative uncharacterized protein
EOG091200N9	OTAU001244	Onthophagus taurus	Putative uncharacterized protein
EOG091200N9	AGLA003890	Anoplophora glabripennis	Putative uncharacterized protein
EOG091200N9	APLA010643	Agrilus planipennis	Putative uncharacterized protein
EOG091200N9	TC013629	Tribolium castaneum	Putative uncharacterized protein
EOG091200N9	LDEC003630	Leptinotarsa decemlineata	Putative uncharacterized protein
EOG091200N9	YQE_02139	Dendroctonus ponderosae	Putative uncharacterized protein
EOG091200NA	OTAU006661	Onthophagus taurus	Putative uncharacterized protein
EOG091200NA	AGLA008916	Anoplophora glabripennis	Putative uncharacterized protein
EOG091200NA	APLA002032	Agrilus planipennis	Putative uncharacterized protein
EOG091200NA	TC010546	Tribolium castaneum	Putative uncharacterized protein
EOG091200NA	LDEC016390	Leptinotarsa decemlineata	Putative uncharacterized protein
EOG091200NA	YQE_04949	Dendroctonus ponderosae	Putative uncharacterized protein
EOG091200NB	OTAU003527	Onthophagus taurus	Putative uncharacterized protein
EOG091200NB	AGLA004919	Anoplophora glabripennis	Putative uncharacterized protein
EOG091200NB	APLA010094	Agrilus planipennis	Putative uncharacterized protein
EOG091200NB	TC008693	Tribolium castaneum	Putative uncharacterized protein
EOG091200NB	LDEC010382	Leptinotarsa decemlineata	Putative uncharacterized protein
EOG091200NB	YQE_05996	Dendroctonus ponderosae	Putative uncharacterized protein
EOG091200NC	OTAU013848	Onthophagus taurus	Putative uncharacterized protein
EOG091200NC	AGLA006931	Anoplophora glabripennis	Putative uncharacterized protein
EOG091200NC	APLA012391	Agrilus planipennis	Putative uncharacterized protein
EOG091200NC	TC003783	Tribolium castaneum	Putative uncharacterized protein
EOG091200NC	LDEC011461	Leptinotarsa decemlineata	Putative uncharacterized protein
EOG091200NC	YQE_03826	Dendroctonus ponderosae	Putative uncharacterized protein
EOG091200ND	OTAU006350	Onthophagus taurus	Putative uncharacterized protein
EOG091200ND	AGLA000013	Anoplophora glabripennis	Putative uncharacterized protein
EOG091200ND	APLA001301	Agrilus planipennis	Putative uncharacterized protein
EOG091200ND	TC000973	Tribolium castaneum	Putative uncharacterized protein
EOG091200ND	LDEC007047	Leptinotarsa decemlineata	Putative uncharacterized protein
EOG091200ND	YQE_10723	Dendroctonus ponderosae	Putative uncharacterized protein
EOG091200NE	OTAU013874	Onthophagus taurus	None
EOG091200NE	AGLA005888	Anoplophora glabripennis	None
EOG091200NE	APLA009421	Agrilus planipennis	None
EOG091200NE	TC033957	Tribolium castaneum	None
EOG091200NE	LDEC003676	Leptinotarsa decemlineata	None
EOG091200NE	YQE_11050	Dendroctonus ponderosae	None
EOG091200NF	OTAU000791	Onthophagus taurus	protein tyrosine phosphatase activity

EOG091200NF	AGLA011169	Anoplophora glabripennis	protein tyrosine phosphatase activity
EOG091200NF	APLA013599	Agrilus planipennis	protein tyrosine phosphatase activity
EOG091200NF	TC034442	Tribolium castaneum	protein tyrosine phosphatase activity
EOG091200NF	LDEC008550	Leptinotarsa decemlineata	protein tyrosine phosphatase activity
EOG091200NF	YQE_07986	Dendroctonus ponderosae	protein tyrosine phosphatase activity
EOG091200NI	OTAU014561	Onthophagus taurus	Putative uncharacterized protein
EOG091200NI	AGLA018224	Anoplophora glabripennis	Putative uncharacterized protein
EOG091200NI	APLA014879	Agrilus planipennis	Putative uncharacterized protein
EOG091200NI	TC015524	Tribolium castaneum	Putative uncharacterized protein
EOG091200NI	LDEC017391	Leptinotarsa decemlineata	Putative uncharacterized protein
EOG091200NI	YQE_10842	Dendroctonus ponderosae	Putative uncharacterized protein
EOG091200NJ	OTAU000056	Onthophagus taurus	Putative uncharacterized protein
EOG091200NJ	AGLA009542	Anoplophora glabripennis	Putative uncharacterized protein
EOG091200NJ	APLA012873	Agrilus planipennis	Putative uncharacterized protein
EOG091200NJ	TC013912	Tribolium castaneum	Putative uncharacterized protein
EOG091200NJ	LDEC003223	Leptinotarsa decemlineata	Putative uncharacterized protein
EOG091200NJ	YQE_07211	Dendroctonus ponderosae	Putative uncharacterized protein
EOG091200NL	OTAU008765	Onthophagus taurus	Eukaryotic translation initiation factor 3 subunit C
EOG091200NL	AGLA006335	Anoplophora glabripennis	Eukaryotic translation initiation factor 3 subunit C
EOG091200NL	APLA009446	Agrilus planipennis	Eukaryotic translation initiation factor 3 subunit C
EOG091200NL	TC015453	Tribolium castaneum	Eukaryotic translation initiation factor 3 subunit C
EOG091200NL	LDEC007874	Leptinotarsa decemlineata	Eukaryotic translation initiation factor 3 subunit C
EOG091200NL	YQE_04258	Dendroctonus ponderosae	Eukaryotic translation initiation factor 3 subunit C
EOG091200NM	OTAU012560	Onthophagus taurus	Putative uncharacterized protein
EOG091200NM	AGLA008218	Anoplophora glabripennis	Putative uncharacterized protein
EOG091200NM	APLA001931	Agrilus planipennis	Putative uncharacterized protein
EOG091200NM	TC000677	Tribolium castaneum	Putative uncharacterized protein
EOG091200NM	LDEC006624	Leptinotarsa decemlineata	Putative uncharacterized protein
EOG091200NM	YQE_11603	Dendroctonus ponderosae	Putative uncharacterized protein
EOG091200NQ	OTAU005975	Onthophagus taurus	6-phosphofructokinase
EOG091200NQ	AGLA003379	Anoplophora glabripennis	6-phosphofructokinase
EOG091200NQ	APLA013705	Agrilus planipennis	6-phosphofructokinase
EOG091200NQ	TC005855	Tribolium castaneum	6-phosphofructokinase
EOG091200NQ	LDEC004630	Leptinotarsa decemlineata	6-phosphofructokinase
EOG091200NQ	YQE_03694	Dendroctonus ponderosae	6-phosphofructokinase
EOG091200NU	OTAU014006	Onthophagus taurus	Putative uncharacterized protein
EOG091200NU	AGLA002610	Anoplophora glabripennis	Putative uncharacterized protein
EOG091200NU	APLA003924	Agrilus planipennis	Putative uncharacterized protein
EOG091200NU	TC010657	Tribolium castaneum	Putative uncharacterized protein
EOG091200NU	LDEC001985	Leptinotarsa decemlineata	Putative uncharacterized protein
EOG091200NU	YQE_05512	Dendroctonus ponderosae	Putative uncharacterized protein
EOG091200NX	OTAU004981	Onthophagus taurus	metallopeptidase activity
EOG091200NX	AGLA006663	Anoplophora glabripennis	metallopeptidase activity
EOG091200NX	APLA005011	Agrilus planipennis	metallopeptidase activity
EOG091200NX	TC032184	Tribolium castaneum	metallopeptidase activity
EOG091200NX	LDEC016021	Leptinotarsa decemlineata	metallopeptidase activity
EOG091200NX	YQE_01676	Dendroctonus ponderosae	metallopeptidase activity
EOG091200NY	OTAU007781	Onthophagus taurus	nucleic acid binding
EOG091200NY	AGLA015616	Anoplophora glabripennis	nucleic acid binding
EOG091200NY	APLA006969	Agrilus planipennis	nucleic acid binding
EOG091200NY	TC033816	Tribolium castaneum	nucleic acid binding
EOG091200NY	LDEC010376	Leptinotarsa decemlineata	nucleic acid binding
EOG091200NY	YQE_03387	Dendroctonus ponderosae	nucleic acid binding
EOG091200O1	OTAU003183	Onthophagus taurus	Putative uncharacterized protein
EOG091200O1	AGLA007361	Anoplophora glabripennis	Putative uncharacterized protein
EOG091200O1	APLA003597	Agrilus planipennis	Putative uncharacterized protein
EOG091200O1	TC014310	Tribolium castaneum	Putative uncharacterized protein
EOG091200O1	LDEC014818	Leptinotarsa decemlineata	Putative uncharacterized protein
EOG091200O1	YQE_07161	Dendroctonus ponderosae	Putative uncharacterized protein
EOG091200O2	OTAU002949	Onthophagus taurus	Putative uncharacterized protein
EOG091200O2	AGLA006818	Anoplophora glabripennis	Putative uncharacterized protein
EOG091200O2	APLA001838	Agrilus planipennis	Putative uncharacterized protein
EOG091200O2	TC009544	Tribolium castaneum	Putative uncharacterized protein
EOG091200O2	LDEC022301	Leptinotarsa decemlineata	Putative uncharacterized protein
EOG091200O2	YQE_10404	Dendroctonus ponderosae	Putative uncharacterized protein
EOG091200O3	OTAU009551	Onthophagus taurus	Putative uncharacterized protein
EOG091200O3	AGLA003783	Anoplophora glabripennis	Putative uncharacterized protein

EOG091200O3	APLA002235	Agrilus planipennis	Putative uncharacterized protein
EOG091200O3	TC007971	Tribolium castaneum	Putative uncharacterized protein
EOG091200O3	LDEC004391	Leptinotarsa decemlineata	Putative uncharacterized protein
EOG091200O3	YQE_04502	Dendroctonus ponderosae	Putative uncharacterized protein
EOG091200O4	OTAU016800	Onthophagus taurus	ion channel activity
EOG091200O4	AGLA010334	Anoplophora glabripennis	ion channel activity
EOG091200O4	APLA000689	Agrilus planipennis	ion channel activity
EOG091200O4	TC033354	Tribolium castaneum	ion channel activity
EOG091200O4	LDEC008277	Leptinotarsa decemlineata	ion channel activity
EOG091200O4	YQE_10149	Dendroctonus ponderosae	ion channel activity
EOG091200O5	OTAU009878	Onthophagus taurus	Putative uncharacterized protein
EOG091200O5	AGLA007438	Anoplophora glabripennis	Putative uncharacterized protein
EOG091200O5	APLA005857	Agrilus planipennis	Putative uncharacterized protein
EOG091200O5	TC001512	Tribolium castaneum	Putative uncharacterized protein
EOG091200O5	LDEC017527	Leptinotarsa decemlineata	Putative uncharacterized protein
EOG091200O5	YQE_11144	Dendroctonus ponderosae	Putative uncharacterized protein
EOG091200O6	OTAU009574	Onthophagus taurus	Putative uncharacterized protein
EOG091200O6	AGLA014415	Anoplophora glabripennis	Putative uncharacterized protein
EOG091200O6	APLA014763	Agrilus planipennis	Putative uncharacterized protein
EOG091200O6	TC007012	Tribolium castaneum	Putative uncharacterized protein
EOG091200O6	LDEC008314	Leptinotarsa decemlineata	Putative uncharacterized protein
EOG091200O6	YQE_03343	Dendroctonus ponderosae	Putative uncharacterized protein
EOG091200O8	OTAU010229	Onthophagus taurus	Putative uncharacterized protein
EOG091200O8	AGLA009546	Anoplophora glabripennis	Putative uncharacterized protein
EOG091200O8	APLA011581	Agrilus planipennis	Putative uncharacterized protein
EOG091200O8	TC013779	Tribolium castaneum	Putative uncharacterized protein
EOG091200O8	LDEC018029	Leptinotarsa decemlineata	Putative uncharacterized protein
EOG091200O8	YQE_07221	Dendroctonus ponderosae	Putative uncharacterized protein
EOG091200OA	OTAU009096	Onthophagus taurus	Putative uncharacterized protein
EOG091200OA	AGLA011077	Anoplophora glabripennis	Putative uncharacterized protein
EOG091200OA	APLA001192	Agrilus planipennis	Putative uncharacterized protein
EOG091200OA	TC005512	Tribolium castaneum	Putative uncharacterized protein
EOG091200OA	LDEC009779	Leptinotarsa decemlineata	Putative uncharacterized protein
EOG091200OA	YQE_07497	Dendroctonus ponderosae	Putative uncharacterized protein
EOG091200OD	OTAU002284	Onthophagus taurus	Putative uncharacterized protein
EOG091200OD	AGLA003724	Anoplophora glabripennis	Putative uncharacterized protein
EOG091200OD	APLA011019	Agrilus planipennis	Putative uncharacterized protein
EOG091200OD	TC008426	Tribolium castaneum	Putative uncharacterized protein
EOG091200OD	LDEC005084	Leptinotarsa decemlineata	Putative uncharacterized protein
EOG091200OD	YQE_06855	Dendroctonus ponderosae	Putative uncharacterized protein
EOG091200OE	OTAU009022	Onthophagus taurus	Putative uncharacterized protein
EOG091200OE	AGLA005773	Anoplophora glabripennis	Putative uncharacterized protein
EOG091200OE	APLA005989	Agrilus planipennis	Putative uncharacterized protein
EOG091200OE	TC014537	Tribolium castaneum	Putative uncharacterized protein
EOG091200OE	LDEC006418	Leptinotarsa decemlineata	Putative uncharacterized protein
EOG091200OE	YQE_05227	Dendroctonus ponderosae	Putative uncharacterized protein
EOG091200OF	OTAU016431	Onthophagus taurus	Putative uncharacterized protein
EOG091200OF	AGLA018077	Anoplophora glabripennis	Putative uncharacterized protein
EOG091200OF	APLA011608	Agrilus planipennis	Putative uncharacterized protein
EOG091200OF	TC002071	Tribolium castaneum	Putative uncharacterized protein
EOG091200OF	LDEC002773	Leptinotarsa decemlineata	Putative uncharacterized protein
EOG091200OF	YQE_02323	Dendroctonus ponderosae	Putative uncharacterized protein
EOG091200OG	OTAU000666	Onthophagus taurus	Putative uncharacterized protein
EOG091200OG	AGLA017465	Anoplophora glabripennis	Putative uncharacterized protein
EOG091200OG	APLA010399	Agrilus planipennis	Putative uncharacterized protein
EOG091200OG	TC012657	Tribolium castaneum	Putative uncharacterized protein
EOG091200OG	LDEC015588	Leptinotarsa decemlineata	Putative uncharacterized protein
EOG091200OG	YQE_01631	Dendroctonus ponderosae	Putative uncharacterized protein
EOG091200OK	OTAU005915	Onthophagus taurus	Blimp-1
EOG091200OK	AGLA005605	Anoplophora glabripennis	Blimp-1
EOG091200OK	APLA008066	Agrilus planipennis	Blimp-1
EOG091200OK	TC014741	Tribolium castaneum	Blimp-1
EOG091200OK	LDEC016433	Leptinotarsa decemlineata	Blimp-1
EOG091200OK	YQE_06336	Dendroctonus ponderosae	Blimp-1
EOG091200OL	OTAU009369	Onthophagus taurus	Putative uncharacterized protein
EOG091200OL	AGLA001244	Anoplophora glabripennis	Putative uncharacterized protein
EOG091200OL	APLA005762	Agrilus planipennis	Putative uncharacterized protein

EOG091200OL	TC006591	Tribolium castaneum	Putative uncharacterized protein
EOG091200OL	LDEC011054	Leptinotarsa decemlineata	Putative uncharacterized protein
EOG091200OL	YQE_06991	Dendroctonus ponderosae	Putative uncharacterized protein
EOG091200OO	OTAU004906	Onthophagus taurus	Aminopeptidase-like protein
EOG091200OO	AGLA006692	Anoplophora glabripennis	Aminopeptidase-like protein
EOG091200OO	APLA005506	Agrilus planipennis	Aminopeptidase-like protein
EOG091200OO	TC000165	Tribolium castaneum	Aminopeptidase-like protein
EOG091200OO	LDEC019591	Leptinotarsa decemlineata	Aminopeptidase-like protein
EOG091200OO	YQE_07722	Dendroctonus ponderosae	Aminopeptidase-like protein
EOG091200OP	OTAU000255	Onthophagus taurus	Putative uncharacterized protein
EOG091200OP	AGLA016812	Anoplophora glabripennis	Putative uncharacterized protein
EOG091200OP	APLA008495	Agrilus planipennis	Putative uncharacterized protein
EOG091200OP	TC013016	Tribolium castaneum	Putative uncharacterized protein
EOG091200OP	LDEC007852	Leptinotarsa decemlineata	Putative uncharacterized protein
EOG091200OP	YQE_08249	Dendroctonus ponderosae	Putative uncharacterized protein
EOG091200OQ	OTAU014396	Onthophagus taurus	Putative uncharacterized protein
EOG091200OQ	AGLA002475	Anoplophora glabripennis	Putative uncharacterized protein
EOG091200OQ	APLA005747	Agrilus planipennis	Putative uncharacterized protein
EOG091200OQ	TC005373	Tribolium castaneum	Putative uncharacterized protein
EOG091200OQ	LDEC012819	Leptinotarsa decemlineata	Putative uncharacterized protein
EOG091200OQ	YQE_07507	Dendroctonus ponderosae	Putative uncharacterized protein
EOG091200OS	OTAU005749	Onthophagus taurus	Putative uncharacterized protein
EOG091200OS	AGLA000557	Anoplophora glabripennis	Putative uncharacterized protein
EOG091200OS	APLA001018	Agrilus planipennis	Putative uncharacterized protein
EOG091200OS	TC003158	Tribolium castaneum	Putative uncharacterized protein
EOG091200OS	LDEC003728	Leptinotarsa decemlineata	Putative uncharacterized protein
EOG091200OS	YQE_07899	Dendroctonus ponderosae	Putative uncharacterized protein
EOG091200OT	OTAU003111	Onthophagus taurus	Putative uncharacterized protein
EOG091200OT	AGLA017223	Anoplophora glabripennis	Putative uncharacterized protein
EOG091200OT	APLA008693	Agrilus planipennis	Putative uncharacterized protein
EOG091200OT	TC012305	Tribolium castaneum	Putative uncharacterized protein
EOG091200OT	LDEC003856	Leptinotarsa decemlineata	Putative uncharacterized protein
EOG091200OT	YQE_04352	Dendroctonus ponderosae	Putative uncharacterized protein
EOG091200OW	OTAU016767	Onthophagus taurus	Putative uncharacterized protein
EOG091200OW	AGLA009733	Anoplophora glabripennis	Putative uncharacterized protein
EOG091200OW	APLA004459	Agrilus planipennis	Putative uncharacterized protein
EOG091200OW	TC006213	Tribolium castaneum	Putative uncharacterized protein
EOG091200OW	LDEC020228	Leptinotarsa decemlineata	Putative uncharacterized protein
EOG091200OW	YQE_12101	Dendroctonus ponderosae	Putative uncharacterized protein
EOG091200OY	OTAU012603	Onthophagus taurus	Putative uncharacterized protein
EOG091200OY	AGLA002259	Anoplophora glabripennis	Putative uncharacterized protein
EOG091200OY	APLA007905	Agrilus planipennis	Putative uncharacterized protein
EOG091200OY	TC001202	Tribolium castaneum	Putative uncharacterized protein
EOG091200OY	LDEC001578	Leptinotarsa decemlineata	Putative uncharacterized protein
EOG091200OY	YQE_07338	Dendroctonus ponderosae	Putative uncharacterized protein
EOG091200P0	OTAU001664	Onthophagus taurus	Putative uncharacterized protein nuoG
EOG091200P0	AGLA010666	Anoplophora glabripennis	Putative uncharacterized protein nuoG
EOG091200P0	APLA013661	Agrilus planipennis	Putative uncharacterized protein nuoG
EOG091200P0	TC006252	Tribolium castaneum	Putative uncharacterized protein nuoG
EOG091200P0	LDEC021639	Leptinotarsa decemlineata	Putative uncharacterized protein nuoG
EOG091200P0	YQE_05595	Dendroctonus ponderosae	Putative uncharacterized protein nuoG
EOG091200P1	OTAU001462	Onthophagus taurus	ubiquitin protein ligase binding
EOG091200P1	AGLA002200	Anoplophora glabripennis	ubiquitin protein ligase binding
EOG091200P1	APLA003561	Agrilus planipennis	ubiquitin protein ligase binding
EOG091200P1	TC031506	Tribolium castaneum	ubiquitin protein ligase binding
EOG091200P1	LDEC012581	Leptinotarsa decemlineata	ubiquitin protein ligase binding
EOG091200P1	YQE_03285	Dendroctonus ponderosae	ubiquitin protein ligase binding
EOG091200P5	OTAU001848	Onthophagus taurus	Putative uncharacterized protein
EOG091200P5	AGLA008885	Anoplophora glabripennis	Putative uncharacterized protein
EOG091200P5	APLA000414	Agrilus planipennis	Putative uncharacterized protein
EOG091200P5	TC006687	Tribolium castaneum	Putative uncharacterized protein
EOG091200P5	LDEC020058	Leptinotarsa decemlineata	Putative uncharacterized protein
EOG091200P5	YQE_06547	Dendroctonus ponderosae	Putative uncharacterized protein
EOG091200P6	OTAU003657	Onthophagus taurus	Putative uncharacterized protein
EOG091200P6	AGLA009516	Anoplophora glabripennis	Putative uncharacterized protein
EOG091200P6	APLA008318	Agrilus planipennis	Putative uncharacterized protein
EOG091200P6	TC007718	Tribolium castaneum	Putative uncharacterized protein

EOG091200P6	LDEC001383	Leptinotarsa decemlineata	Putative uncharacterized protein
EOG091200P6	YQE_10936	Dendroctonus ponderosae	Putative uncharacterized protein
EOG091200P8	OTAU004420	Onthophagus taurus	Putative uncharacterized protein
EOG091200P8	AGLA000738	Anoplophora glabripennis	Putative uncharacterized protein
EOG091200P8	APLA006558	Agrilus planipennis	Putative uncharacterized protein
EOG091200P8	TC008146	Tribolium castaneum	Putative uncharacterized protein
EOG091200P8	LDEC005097	Leptinotarsa decemlineata	Putative uncharacterized protein
EOG091200P8	YQE_11444	Dendroctonus ponderosae	Putative uncharacterized protein
EOG091200P9	OTAU002977	Onthophagus taurus	Putative uncharacterized protein
EOG091200P9	AGLA010307	Anoplophora glabripennis	Putative uncharacterized protein
EOG091200P9	APLA006017	Agrilus planipennis	Putative uncharacterized protein
EOG091200P9	TC009940	Tribolium castaneum	Putative uncharacterized protein
EOG091200P9	LDEC002217	Leptinotarsa decemlineata	Putative uncharacterized protein
EOG091200P9	YQE_10400	Dendroctonus ponderosae	Putative uncharacterized protein
EOG091200PD	OTAU013735	Onthophagus taurus	Putative uncharacterized protein
EOG091200PD	AGLA011138	Anoplophora glabripennis	Putative uncharacterized protein
EOG091200PD	APLA014278	Agrilus planipennis	Putative uncharacterized protein
EOG091200PD	TC013721	Tribolium castaneum	Putative uncharacterized protein
EOG091200PD	LDEC017547	Leptinotarsa decemlineata	Putative uncharacterized protein
EOG091200PD	YQE_08213	Dendroctonus ponderosae	Putative uncharacterized protein
EOG091200PF	OTAU000718	Onthophagus taurus	Putative uncharacterized protein
EOG091200PF	AGLA018569	Anoplophora glabripennis	Putative uncharacterized protein
EOG091200PF	APLA003286	Agrilus planipennis	Putative uncharacterized protein
EOG091200PF	TC012303	Tribolium castaneum	Putative uncharacterized protein
EOG091200PF	LDEC003875	Leptinotarsa decemlineata	Putative uncharacterized protein
EOG091200PF	YQE_01948	Dendroctonus ponderosae	Putative uncharacterized protein
EOG091200PI	OTAU000426	Onthophagus taurus	Putative uncharacterized protein
EOG091200PI	AGLA017350	Anoplophora glabripennis	Putative uncharacterized protein
EOG091200PI	APLA002379	Agrilus planipennis	Putative uncharacterized protein
EOG091200PI	TC011919	Tribolium castaneum	Putative uncharacterized protein
EOG091200PI	LDEC013103	Leptinotarsa decemlineata	Putative uncharacterized protein
EOG091200PI	YQE_05488	Dendroctonus ponderosae	Putative uncharacterized protein
EOG091200PJ	OTAU012923	Onthophagus taurus	Putative uncharacterized protein
EOG091200PJ	AGLA018746	Anoplophora glabripennis	Putative uncharacterized protein
EOG091200PJ	APLA000367	Agrilus planipennis	Putative uncharacterized protein
EOG091200PJ	TC006065	Tribolium castaneum	Putative uncharacterized protein
EOG091200PJ	LDEC004907	Leptinotarsa decemlineata	Putative uncharacterized protein
EOG091200PJ	YQE_06575	Dendroctonus ponderosae	Putative uncharacterized protein
EOG091200PP	OTAU008299	Onthophagus taurus	Roundabout
EOG091200PP	AGLA004269	Anoplophora glabripennis	Roundabout
EOG091200PP	APLA006804	Agrilus planipennis	Roundabout
EOG091200PP	TC002775	Tribolium castaneum	Roundabout
EOG091200PP	LDEC014340	Leptinotarsa decemlineata	Roundabout
EOG091200PP	YQE_07701	Dendroctonus ponderosae	Roundabout
EOG091200PQ	OTAU005774	Onthophagus taurus	Putative uncharacterized protein
EOG091200PQ	AGLA013038	Anoplophora glabripennis	Putative uncharacterized protein
EOG091200PQ	APLA010161	Agrilus planipennis	Putative uncharacterized protein
EOG091200PQ	TC002437	Tribolium castaneum	Putative uncharacterized protein
EOG091200PQ	LDEC009811	Leptinotarsa decemlineata	Putative uncharacterized protein
EOG091200PQ	YQE_05870	Dendroctonus ponderosae	Putative uncharacterized protein
EOG091200PR	OTAU003820	Onthophagus taurus	Putative uncharacterized protein
EOG091200PR	AGLA013879	Anoplophora glabripennis	Putative uncharacterized protein
EOG091200PR	APLA004612	Agrilus planipennis	Putative uncharacterized protein
EOG091200PR	TC009173	Tribolium castaneum	Putative uncharacterized protein
EOG091200PR	LDEC004469	Leptinotarsa decemlineata	Putative uncharacterized protein
EOG091200PR	YQE_10616	Dendroctonus ponderosae	Putative uncharacterized protein
EOG091200PS	OTAU007333	Onthophagus taurus	Putative uncharacterized protein
EOG091200PS	AGLA020010	Anoplophora glabripennis	Putative uncharacterized protein
EOG091200PS	APLA005259	Agrilus planipennis	Putative uncharacterized protein
EOG091200PS	TC015462	Tribolium castaneum	Putative uncharacterized protein
EOG091200PS	LDEC016536	Leptinotarsa decemlineata	Putative uncharacterized protein
EOG091200PS	YQE_11532	Dendroctonus ponderosae	Putative uncharacterized protein
EOG091200PT	OTAU012984	Onthophagus taurus	Putative uncharacterized protein
EOG091200PT	AGLA002028	Anoplophora glabripennis	Putative uncharacterized protein
EOG091200PT	APLA010335	Agrilus planipennis	Putative uncharacterized protein
EOG091200PT	TC002462	Tribolium castaneum	Putative uncharacterized protein
EOG091200PT	LDEC013418	Leptinotarsa decemlineata	Putative uncharacterized protein

EOG091200PT	YQE_02649	Dendroctonus ponderosae	Putative uncharacterized protein
EOG091200PV	OTAU000700	Onthophagus taurus	Putative uncharacterized protein
EOG091200PV	AGLA021358	Anoplophora glabripennis	Putative uncharacterized protein
EOG091200PV	APLA014337	Agrilus planipennis	Putative uncharacterized protein
EOG091200PV	TC002697	Tribolium castaneum	Putative uncharacterized protein
EOG091200PV	LDEC015601	Leptinotarsa decemlineata	Putative uncharacterized protein
EOG091200PV	YQE_11952	Dendroctonus ponderosae	Putative uncharacterized protein
EOG091200PW	OTAU009860	Onthophagus taurus	Putative uncharacterized protein
EOG091200PW	AGLA009071	Anoplophora glabripennis	Putative uncharacterized protein
EOG091200PW	APLA006321	Agrilus planipennis	Putative uncharacterized protein
EOG091200PW	TC004970	Tribolium castaneum	Putative uncharacterized protein
EOG091200PW	LDEC004866	Leptinotarsa decemlineata	Putative uncharacterized protein
EOG091200PW	YQE_10763	Dendroctonus ponderosae	Putative uncharacterized protein
EOG091200PX	OTAU009549	Onthophagus taurus	Anoctamin
EOG091200PX	AGLA000687	Anoplophora glabripennis	Anoctamin
EOG091200PX	APLA013379	Agrilus planipennis	Anoctamin
EOG091200PX	TC007305	Tribolium castaneum	Anoctamin
EOG091200PX	LDEC013965	Leptinotarsa decemlineata	Anoctamin
EOG091200PX	YQE_04763	Dendroctonus ponderosae	Anoctamin
EOG091200Q2	OTAU016382	Onthophagus taurus	Putative uncharacterized protein
EOG091200Q2	AGLA017658	Anoplophora glabripennis	Putative uncharacterized protein
EOG091200Q2	APLA007631	Agrilus planipennis	Putative uncharacterized protein
EOG091200Q2	TC000032	Tribolium castaneum	Putative uncharacterized protein
EOG091200Q2	LDEC010072	Leptinotarsa decemlineata	Putative uncharacterized protein
EOG091200Q2	YQE_08275	Dendroctonus ponderosae	Putative uncharacterized protein
EOG091200Q4	OTAU015878	Onthophagus taurus	Putative uncharacterized protein
EOG091200Q4	AGLA008048	Anoplophora glabripennis	Putative uncharacterized protein
EOG091200Q4	APLA011125	Agrilus planipennis	Putative uncharacterized protein
EOG091200Q4	TC007793	Tribolium castaneum	Putative uncharacterized protein
EOG091200Q4	LDEC016118	Leptinotarsa decemlineata	Putative uncharacterized protein
EOG091200Q4	YQE_02881	Dendroctonus ponderosae	Putative uncharacterized protein
EOG091200Q5	OTAU009087	Onthophagus taurus	Ubiquitin carboxyl-terminal hydrolase
EOG091200Q5	AGLA015120	Anoplophora glabripennis	Ubiquitin carboxyl-terminal hydrolase
EOG091200Q5	APLA011050	Agrilus planipennis	Ubiquitin carboxyl-terminal hydrolase
EOG091200Q5	TC005587	Tribolium castaneum	Ubiquitin carboxyl-terminal hydrolase
EOG091200Q5	LDEC011702	Leptinotarsa decemlineata	Ubiquitin carboxyl-terminal hydrolase
EOG091200Q5	YQE_09447	Dendroctonus ponderosae	Ubiquitin carboxyl-terminal hydrolase
EOG091200Q6	OTAU003101	Onthophagus taurus	Structural maintenance of chromosomes protein
EOG091200Q6	AGLA006900	Anoplophora glabripennis	Structural maintenance of chromosomes protein
EOG091200Q6	APLA005777	Agrilus planipennis	Structural maintenance of chromosomes protein
EOG091200Q6	TC000075	Tribolium castaneum	Structural maintenance of chromosomes protein
EOG091200Q6	LDEC009408	Leptinotarsa decemlineata	Structural maintenance of chromosomes protein
EOG091200Q6	YQE_11282	Dendroctonus ponderosae	Structural maintenance of chromosomes protein
EOG091200Q8	OTAU003375	Onthophagus taurus	Putative uncharacterized protein
EOG091200Q8	AGLA004836	Anoplophora glabripennis	Putative uncharacterized protein
EOG091200Q8	APLA014305	Agrilus planipennis	Putative uncharacterized protein
EOG091200Q8	TC010503	Tribolium castaneum	Putative uncharacterized protein
EOG091200Q8	LDEC004891	Leptinotarsa decemlineata	Putative uncharacterized protein
EOG091200Q8	YQE_10886	Dendroctonus ponderosae	Putative uncharacterized protein
EOG091200QA	OTAU004169	Onthophagus taurus	Putative uncharacterized protein
EOG091200QA	AGLA010111	Anoplophora glabripennis	Putative uncharacterized protein
EOG091200QA	APLA010837	Agrilus planipennis	Putative uncharacterized protein
EOG091200QA	TC014137	Tribolium castaneum	Putative uncharacterized protein
EOG091200QA	LDEC005453	Leptinotarsa decemlineata	Putative uncharacterized protein
EOG091200QA	YQE_11367	Dendroctonus ponderosae	Putative uncharacterized protein
EOG091200QB	OTAU007550	Onthophagus taurus	Putative uncharacterized protein
EOG091200QB	AGLA017851	Anoplophora glabripennis	Putative uncharacterized protein
EOG091200QB	APLA008272	Agrilus planipennis	Putative uncharacterized protein
EOG091200QB	TC014609	Tribolium castaneum	Putative uncharacterized protein
EOG091200QB	LDEC002153	Leptinotarsa decemlineata	Putative uncharacterized protein
EOG091200QB	YQE_04015	Dendroctonus ponderosae	Putative uncharacterized protein
EOG091200QH	OTAU011625	Onthophagus taurus	Putative uncharacterized protein
EOG091200QH	AGLA002436	Anoplophora glabripennis	Putative uncharacterized protein
EOG091200QH	APLA010598	Agrilus planipennis	Putative uncharacterized protein
EOG091200QH	TC002088	Tribolium castaneum	Putative uncharacterized protein
EOG091200QH	LDEC009634	Leptinotarsa decemlineata	Putative uncharacterized protein
EOG091200QH	YQE_05249	Dendroctonus ponderosae	Putative uncharacterized protein

EOG091200QI	OTAU008212	Onthophagus taurus	DNA ligase activity
EOG091200QI	AGLA001926	Anoplophora glabripennis	DNA ligase activity
EOG091200QI	APLA004368	Agrilus planipennis	DNA ligase activity
EOG091200QI	TC030983	Tribolium castaneum	DNA ligase activity
EOG091200QI	LDEC019023	Leptinotarsa decemlineata	DNA ligase activity
EOG091200QI	YQE_09498	Dendroctonus ponderosae	DNA ligase activity
EOG091200QK	OTAU006929	Onthophagus taurus	Putative uncharacterized protein
EOG091200QK	AGLA010746	Anoplophora glabripennis	Putative uncharacterized protein
EOG091200QK	APLA013399	Agrilus planipennis	Putative uncharacterized protein
EOG091200QK	TC003094	Tribolium castaneum	Putative uncharacterized protein
EOG091200QK	LDEC016953	Leptinotarsa decemlineata	Putative uncharacterized protein
EOG091200QK	YQE_05207	Dendroctonus ponderosae	Putative uncharacterized protein
EOG091200QL	OTAU009192	Onthophagus taurus	ATP binding
EOG091200QL	AGLA002527	Anoplophora glabripennis	ATP binding
EOG091200QL	APLA003827	Agrilus planipennis	ATP binding
EOG091200QL	TC030896	Tribolium castaneum	ATP binding
EOG091200QL	LDEC009764	Leptinotarsa decemlineata	ATP binding
EOG091200QL	YQE_06539	Dendroctonus ponderosae	ATP binding
EOG091200QO	OTAU005095	Onthophagus taurus	Putative uncharacterized protein
EOG091200QO	AGLA002380	Anoplophora glabripennis	Putative uncharacterized protein
EOG091200QO	APLA003508	Agrilus planipennis	Putative uncharacterized protein
EOG091200QO	TC013601	Tribolium castaneum	Putative uncharacterized protein
EOG091200QO	LDEC004112	Leptinotarsa decemlineata	Putative uncharacterized protein
EOG091200QO	YQE_11018	Dendroctonus ponderosae	Putative uncharacterized protein
EOG091200QQ	OTAU003672	Onthophagus taurus	ribonuclease T2 activity
EOG091200QQ	AGLA000681	Anoplophora glabripennis	ribonuclease T2 activity
EOG091200QQ	APLA005347	Agrilus planipennis	ribonuclease T2 activity
EOG091200QQ	TC032828	Tribolium castaneum	ribonuclease T2 activity
EOG091200QQ	LDEC000639	Leptinotarsa decemlineata	ribonuclease T2 activity
EOG091200QQ	YQE_03964	Dendroctonus ponderosae	ribonuclease T2 activity
EOG091200QR	OTAU002580	Onthophagus taurus	Putative uncharacterized protein
EOG091200QR	AGLA019994	Anoplophora glabripennis	Putative uncharacterized protein
EOG091200QR	APLA009740	Agrilus planipennis	Putative uncharacterized protein
EOG091200QR	TC030724	Tribolium castaneum	Putative uncharacterized protein
EOG091200QR	LDEC002622	Leptinotarsa decemlineata	Putative uncharacterized protein
EOG091200QR	YQE_06341	Dendroctonus ponderosae	Putative uncharacterized protein
EOG091200QT	OTAU000828	Onthophagus taurus	catalytic activity
EOG091200QT	AGLA018580	Anoplophora glabripennis	catalytic activity
EOG091200QT	APLA002968	Agrilus planipennis	catalytic activity
EOG091200QT	TC031384	Tribolium castaneum	catalytic activity
EOG091200QT	LDEC013485	Leptinotarsa decemlineata	catalytic activity
EOG091200QT	YQE_11232	Dendroctonus ponderosae	catalytic activity
EOG091200QV	OTAU012591	Onthophagus taurus	Putative uncharacterized protein
EOG091200QV	AGLA010652	Anoplophora glabripennis	Putative uncharacterized protein
EOG091200QV	APLA004593	Agrilus planipennis	Putative uncharacterized protein
EOG091200QV	TC009337	Tribolium castaneum	Putative uncharacterized protein
EOG091200QV	LDEC002333	Leptinotarsa decemlineata	Putative uncharacterized protein
EOG091200QV	YQE_03377	Dendroctonus ponderosae	Putative uncharacterized protein
EOG091200QX	OTAU003605	Onthophagus taurus	DNA polymerase
EOG091200QX	AGLA019829	Anoplophora glabripennis	DNA polymerase
EOG091200QX	APLA005002	Agrilus planipennis	DNA polymerase
EOG091200QX	TC004938	Tribolium castaneum	DNA polymerase
EOG091200QX	LDEC012115	Leptinotarsa decemlineata	DNA polymerase
EOG091200QX	YQE_08755	Dendroctonus ponderosae	DNA polymerase
EOG091200QY	OTAU011044	Onthophagus taurus	Putative uncharacterized protein
EOG091200QY	AGLA010624	Anoplophora glabripennis	Putative uncharacterized protein
EOG091200QY	APLA013114	Agrilus planipennis	Putative uncharacterized protein
EOG091200QY	TC014398	Tribolium castaneum	Putative uncharacterized protein
EOG091200QY	LDEC015139	Leptinotarsa decemlineata	Putative uncharacterized protein
EOG091200QY	YQE_11371	Dendroctonus ponderosae	Putative uncharacterized protein
EOG091200QZ	OTAU005829	Onthophagus taurus	Putative uncharacterized protein
EOG091200QZ	AGLA013891	Anoplophora glabripennis	Putative uncharacterized protein
EOG091200QZ	APLA000131	Agrilus planipennis	Putative uncharacterized protein
EOG091200QZ	TC000042	Tribolium castaneum	Putative uncharacterized protein
EOG091200QZ	LDEC002710	Leptinotarsa decemlineata	Putative uncharacterized protein
EOG091200QZ	YQE_06749	Dendroctonus ponderosae	Putative uncharacterized protein
EOG091200R0	OTAU002807	Onthophagus taurus	Putative uncharacterized protein

EOG091200R0	AGLA002175	Anoplophora glabripennis	Putative uncharacterized protein
EOG091200R0	APLA003483	Agrilus planipennis	Putative uncharacterized protein
EOG091200R0	TC013622	Tribolium castaneum	Putative uncharacterized protein
EOG091200R0	LDEC021384	Leptinotarsa decemlineata	Putative uncharacterized protein
EOG091200R0	YQE_09284	Dendroctonus ponderosae	Putative uncharacterized protein
EOG091200R2	OTAU011769	Onthophagus taurus	Putative uncharacterized protein
EOG091200R2	AGLA016309	Anoplophora glabripennis	Putative uncharacterized protein
EOG091200R2	APLA011278	Agrilus planipennis	Putative uncharacterized protein
EOG091200R2	TC009293	Tribolium castaneum	Putative uncharacterized protein
EOG091200R2	LDEC015094	Leptinotarsa decemlineata	Putative uncharacterized protein
EOG091200R2	YQE_10594	Dendroctonus ponderosae	Putative uncharacterized protein
EOG091200R4	OTAU006099	Onthophagus taurus	Putative uncharacterized protein
EOG091200R4	AGLA011941	Anoplophora glabripennis	Putative uncharacterized protein
EOG091200R4	APLA002879	Agrilus planipennis	Putative uncharacterized protein
EOG091200R4	TC013974	Tribolium castaneum	Putative uncharacterized protein
EOG091200R4	LDEC003250	Leptinotarsa decemlineata	Putative uncharacterized protein
EOG091200R4	YQE_03416	Dendroctonus ponderosae	Putative uncharacterized protein
EOG091200R7	OTAU003289	Onthophagus taurus	Putative uncharacterized protein
EOG091200R7	AGLA003828	Anoplophora glabripennis	Putative uncharacterized protein
EOG091200R7	APLA014477	Agrilus planipennis	Putative uncharacterized protein
EOG091200R7	TC008921	Tribolium castaneum	Putative uncharacterized protein
EOG091200R7	LDEC009333	Leptinotarsa decemlineata	Putative uncharacterized protein
EOG091200R7	YQE_05297	Dendroctonus ponderosae	Putative uncharacterized protein
EOG091200RB	OTAU009831	Onthophagus taurus	Putative uncharacterized protein
EOG091200RB	AGLA006305	Anoplophora glabripennis	Putative uncharacterized protein
EOG091200RB	APLA011270	Agrilus planipennis	Putative uncharacterized protein
EOG091200RB	TC008832	Tribolium castaneum	Putative uncharacterized protein
EOG091200RB	LDEC002102	Leptinotarsa decemlineata	Putative uncharacterized protein
EOG091200RB	YQE_11825	Dendroctonus ponderosae	Putative uncharacterized protein
EOG091200RC	OTAU004085	Onthophagus taurus	binding
EOG091200RC	AGLA008728	Anoplophora glabripennis	binding
EOG091200RC	APLA014523	Agrilus planipennis	binding
EOG091200RC	TC033813	Tribolium castaneum	binding
EOG091200RC	LDEC004845	Leptinotarsa decemlineata	binding
EOG091200RC	YQE_05739	Dendroctonus ponderosae	binding
EOG091200RD	OTAU000526	Onthophagus taurus	Putative uncharacterized protein
EOG091200RD	AGLA004243	Anoplophora glabripennis	Putative uncharacterized protein
EOG091200RD	APLA000162	Agrilus planipennis	Putative uncharacterized protein
EOG091200RD	TC003521	Tribolium castaneum	Putative uncharacterized protein
EOG091200RD	LDEC008219	Leptinotarsa decemlineata	Putative uncharacterized protein
EOG091200RD	YQE_07430	Dendroctonus ponderosae	Putative uncharacterized protein
EOG091200RF	OTAU000269	Onthophagus taurus	Putative uncharacterized protein
EOG091200RF	AGLA002652	Anoplophora glabripennis	Putative uncharacterized protein
EOG091200RF	APLA015132	Agrilus planipennis	Putative uncharacterized protein
EOG091200RF	TC014792	Tribolium castaneum	Putative uncharacterized protein
EOG091200RF	LDEC018026	Leptinotarsa decemlineata	Putative uncharacterized protein
EOG091200RF	YQE_12253	Dendroctonus ponderosae	Putative uncharacterized protein
EOG091200RG	OTAU007202	Onthophagus taurus	Putative uncharacterized protein
EOG091200RG	AGLA010329	Anoplophora glabripennis	Putative uncharacterized protein
EOG091200RG	APLA000556	Agrilus planipennis	Putative uncharacterized protein
EOG091200RG	TC015354	Tribolium castaneum	Putative uncharacterized protein
EOG091200RG	LDEC008534	Leptinotarsa decemlineata	Putative uncharacterized protein
EOG091200RG	YQE_05911	Dendroctonus ponderosae	Putative uncharacterized protein
EOG091200RH	OTAU002946	Onthophagus taurus	Putative uncharacterized protein
EOG091200RH	AGLA003834	Anoplophora glabripennis	Putative uncharacterized protein
EOG091200RH	APLA014481	Agrilus planipennis	Putative uncharacterized protein
EOG091200RH	TC009916	Tribolium castaneum	Putative uncharacterized protein
EOG091200RH	LDEC007777	Leptinotarsa decemlineata	Putative uncharacterized protein
EOG091200RH	YQE_05302	Dendroctonus ponderosae	Putative uncharacterized protein
EOG091200RI	OTAU002398	Onthophagus taurus	Putative uncharacterized protein
EOG091200RI	AGLA002036	Anoplophora glabripennis	Putative uncharacterized protein
EOG091200RI	APLA009154	Agrilus planipennis	Putative uncharacterized protein
EOG091200RI	TC002463	Tribolium castaneum	Putative uncharacterized protein
EOG091200RI	LDEC019751	Leptinotarsa decemlineata	Putative uncharacterized protein
EOG091200RI	YQE_03689	Dendroctonus ponderosae	Putative uncharacterized protein
EOG091200RJ	OTAU016129	Onthophagus taurus	Putative uncharacterized protein
EOG091200RJ	AGLA013304	Anoplophora glabripennis	Putative uncharacterized protein

EOG091200RJ	APLA006459	Agrilus planipennis	Putative uncharacterized protein
EOG091200RJ	TC030720	Tribolium castaneum	Putative uncharacterized protein
EOG091200RJ	LDEC020379	Leptinotarsa decemlineata	Putative uncharacterized protein
EOG091200RJ	YQE_10161	Dendroctonus ponderosae	Putative uncharacterized protein
EOG091200RK	OTAU003045	Onthophagus taurus	Ubiquitin carboxyl-terminal hydrolase
EOG091200RK	AGLA015040	Anoplophora glabripennis	Ubiquitin carboxyl-terminal hydrolase
EOG091200RK	APLA007962	Agrilus planipennis	Ubiquitin carboxyl-terminal hydrolase
EOG091200RK	TC003401	Tribolium castaneum	Ubiquitin carboxyl-terminal hydrolase
EOG091200RK	LDEC008727	Leptinotarsa decemlineata	Ubiquitin carboxyl-terminal hydrolase
EOG091200RK	YQE_02741	Dendroctonus ponderosae	Ubiquitin carboxyl-terminal hydrolase
EOG091200RL	OTAU001049	Onthophagus taurus	hydrolase activity
EOG091200RL	AGLA010907	Anoplophora glabripennis	hydrolase activity
EOG091200RL	APLA001977	Agrilus planipennis	hydrolase activity
EOG091200RL	TC031963	Tribolium castaneum	hydrolase activity
EOG091200RL	LDEC000813	Leptinotarsa decemlineata	hydrolase activity
EOG091200RL	YQE_04246	Dendroctonus ponderosae	hydrolase activity
EOG091200RM	OTAU000834	Onthophagus taurus	Putative uncharacterized protein
EOG091200RM	AGLA015848	Anoplophora glabripennis	Putative uncharacterized protein
EOG091200RM	APLA001963	Agrilus planipennis	Putative uncharacterized protein
EOG091200RM	TC004395	Tribolium castaneum	Putative uncharacterized protein
EOG091200RM	LDEC011351	Leptinotarsa decemlineata	Putative uncharacterized protein
EOG091200RM	YQE_08918	Dendroctonus ponderosae	Putative uncharacterized protein
EOG091200RN	OTAU009647	Onthophagus taurus	Putative uncharacterized protein
EOG091200RN	AGLA013700	Anoplophora glabripennis	Putative uncharacterized protein
EOG091200RN	APLA010579	Agrilus planipennis	Putative uncharacterized protein
EOG091200RN	TC004359	Tribolium castaneum	Putative uncharacterized protein
EOG091200RN	LDEC016543	Leptinotarsa decemlineata	Putative uncharacterized protein
EOG091200RN	YQE_06478	Dendroctonus ponderosae	Putative uncharacterized protein
EOG091200RO	OTAU002179	Onthophagus taurus	Putative uncharacterized protein
EOG091200RO	AGLA011381	Anoplophora glabripennis	Putative uncharacterized protein
EOG091200RO	APLA003235	Agrilus planipennis	Putative uncharacterized protein
EOG091200RO	TC012348	Tribolium castaneum	Putative uncharacterized protein
EOG091200RO	LDEC002071	Leptinotarsa decemlineata	Putative uncharacterized protein
EOG091200RO	YQE_08122	Dendroctonus ponderosae	Putative uncharacterized protein
EOG091200RP	OTAU004165	Onthophagus taurus	Putative uncharacterized protein
EOG091200RP	AGLA010687	Anoplophora glabripennis	Putative uncharacterized protein
EOG091200RP	APLA002444	Agrilus planipennis	Putative uncharacterized protein
EOG091200RP	TC013667	Tribolium castaneum	Putative uncharacterized protein
EOG091200RP	LDEC004843	Leptinotarsa decemlineata	Putative uncharacterized protein
EOG091200RP	YQE_10336	Dendroctonus ponderosae	Putative uncharacterized protein
EOG091200RQ	OTAU010929	Onthophagus taurus	Putative uncharacterized protein
EOG091200RQ	AGLA018374	Anoplophora glabripennis	Putative uncharacterized protein
EOG091200RQ	APLA011351	Agrilus planipennis	Putative uncharacterized protein
EOG091200RQ	TC015584	Tribolium castaneum	Putative uncharacterized protein
EOG091200RQ	LDEC002342	Leptinotarsa decemlineata	Putative uncharacterized protein
EOG091200RQ	YQE_05901	Dendroctonus ponderosae	Putative uncharacterized protein
EOG091200RS	OTAU003226	Onthophagus taurus	Putative uncharacterized protein
EOG091200RS	AGLA003508	Anoplophora glabripennis	Putative uncharacterized protein
EOG091200RS	APLA006630	Agrilus planipennis	Putative uncharacterized protein
EOG091200RS	TC003172	Tribolium castaneum	Putative uncharacterized protein
EOG091200RS	LDEC012701	Leptinotarsa decemlineata	Putative uncharacterized protein
EOG091200RS	YQE_11125	Dendroctonus ponderosae	Putative uncharacterized protein
EOG091200RU	OTAU014134	Onthophagus taurus	microtubule binding
EOG091200RU	AGLA011392	Anoplophora glabripennis	microtubule binding
EOG091200RU	APLA011575	Agrilus planipennis	microtubule binding
EOG091200RU	TC033048	Tribolium castaneum	microtubule binding
EOG091200RU	LDEC006496	Leptinotarsa decemlineata	microtubule binding
EOG091200RU	YQE_07219	Dendroctonus ponderosae	microtubule binding
EOG091200RV	OTAU005091	Onthophagus taurus	Putative uncharacterized protein
EOG091200RV	AGLA009742	Anoplophora glabripennis	Putative uncharacterized protein
EOG091200RV	APLA005785	Agrilus planipennis	Putative uncharacterized protein
EOG091200RV	TC005310	Tribolium castaneum	Putative uncharacterized protein
EOG091200RV	LDEC011343	Leptinotarsa decemlineata	Putative uncharacterized protein
EOG091200RV	YQE_05531	Dendroctonus ponderosae	Putative uncharacterized protein
EOG091200S0	OTAU010254	Onthophagus taurus	Putative uncharacterized protein
EOG091200S0	AGLA011848	Anoplophora glabripennis	Putative uncharacterized protein
EOG091200S0	APLA001845	Agrilus planipennis	Putative uncharacterized protein

EOG091200S0	TC009562	Tribolium castaneum	Putative uncharacterized protein
EOG091200S0	LDEC002751	Leptinotarsa decemlineata	Putative uncharacterized protein
EOG091200S0	YQE_11805	Dendroctonus ponderosae	Putative uncharacterized protein
EOG091200S1	OTAU004022	Onthophagus taurus	Putative uncharacterized protein
EOG091200S1	AGLA001442	Anoplophora glabripennis	Putative uncharacterized protein
EOG091200S1	APLA014003	Agrilus planipennis	Putative uncharacterized protein
EOG091200S1	TC002887	Tribolium castaneum	Putative uncharacterized protein
EOG091200S1	LDEC003349	Leptinotarsa decemlineata	Putative uncharacterized protein
EOG091200S1	YQE_05203	Dendroctonus ponderosae	Putative uncharacterized protein
EOG091200S2	OTAU004939	Onthophagus taurus	Putative uncharacterized protein
EOG091200S2	AGLA017147	Anoplophora glabripennis	Putative uncharacterized protein
EOG091200S2	APLA005297	Agrilus planipennis	Putative uncharacterized protein
EOG091200S2	TC001014	Tribolium castaneum	Putative uncharacterized protein
EOG091200S2	LDEC006629	Leptinotarsa decemlineata	Putative uncharacterized protein
EOG091200S2	YQE_10976	Dendroctonus ponderosae	Putative uncharacterized protein
EOG091200S4	OTAU012746	Onthophagus taurus	Putative uncharacterized protein
EOG091200S4	AGLA006064	Anoplophora glabripennis	Putative uncharacterized protein
EOG091200S4	APLA007350	Agrilus planipennis	Putative uncharacterized protein
EOG091200S4	TC014419	Tribolium castaneum	Putative uncharacterized protein
EOG091200S4	LDEC021712	Leptinotarsa decemlineata	Putative uncharacterized protein
EOG091200S4	YQE_01938	Dendroctonus ponderosae	Putative uncharacterized protein
EOG091200S6	OTAU001074	Onthophagus taurus	Putative uncharacterized protein
EOG091200S6	AGLA017511	Anoplophora glabripennis	Putative uncharacterized protein
EOG091200S6	APLA001345	Agrilus planipennis	Putative uncharacterized protein
EOG091200S6	TC001384	Tribolium castaneum	Putative uncharacterized protein
EOG091200S6	LDEC020925	Leptinotarsa decemlineata	Putative uncharacterized protein
EOG091200S6	YQE_09530	Dendroctonus ponderosae	Putative uncharacterized protein
EOG091200SA	OTAU003628	Onthophagus taurus	Putative uncharacterized protein
EOG091200SA	AGLA011236	Anoplophora glabripennis	Putative uncharacterized protein
EOG091200SA	APLA001484	Agrilus planipennis	Putative uncharacterized protein
EOG091200SA	TC002945	Tribolium castaneum	Putative uncharacterized protein
EOG091200SA	LDEC003268	Leptinotarsa decemlineata	Putative uncharacterized protein
EOG091200SA	YQE_12343	Dendroctonus ponderosae	Putative uncharacterized protein
EOG091200SB	OTAU000716	Onthophagus taurus	Putative uncharacterized protein
EOG091200SB	AGLA008127	Anoplophora glabripennis	Putative uncharacterized protein
EOG091200SB	APLA014957	Agrilus planipennis	Putative uncharacterized protein
EOG091200SB	TC012418	Tribolium castaneum	Putative uncharacterized protein
EOG091200SB	LDEC016251	Leptinotarsa decemlineata	Putative uncharacterized protein
EOG091200SB	YQE_12315	Dendroctonus ponderosae	Putative uncharacterized protein
EOG091200SC	OTAU000368	Onthophagus taurus	Putative uncharacterized protein
EOG091200SC	AGLA005097	Anoplophora glabripennis	Putative uncharacterized protein
EOG091200SC	APLA003318	Agrilus planipennis	Putative uncharacterized protein
EOG091200SC	TC012552	Tribolium castaneum	Putative uncharacterized protein
EOG091200SC	LDEC005501	Leptinotarsa decemlineata	Putative uncharacterized protein
EOG091200SC	YQE_01647	Dendroctonus ponderosae	Putative uncharacterized protein
EOG091200SD	OTAU003215	Onthophagus taurus	Putative uncharacterized protein
EOG091200SD	AGLA004903	Anoplophora glabripennis	Putative uncharacterized protein
EOG091200SD	APLA004737	Agrilus planipennis	Putative uncharacterized protein
EOG091200SD	TC014506	Tribolium castaneum	Putative uncharacterized protein
EOG091200SD	LDEC011327	Leptinotarsa decemlineata	Putative uncharacterized protein
EOG091200SD	YQE_09718	Dendroctonus ponderosae	Putative uncharacterized protein
EOG091200SF	OTAU001622	Onthophagus taurus	Eukaryotic translation initiation factor 3 subunit B
EOG091200SF	AGLA016925	Anoplophora glabripennis	Eukaryotic translation initiation factor 3 subunit B
EOG091200SF	APLA005476	Agrilus planipennis	Eukaryotic translation initiation factor 3 subunit B
EOG091200SF	TC006009	Tribolium castaneum	Eukaryotic translation initiation factor 3 subunit B
EOG091200SF	LDEC009783	Leptinotarsa decemlineata	Eukaryotic translation initiation factor 3 subunit B
EOG091200SF	YQE_12172	Dendroctonus ponderosae	Eukaryotic translation initiation factor 3 subunit B
EOG091200SG	OTAU000306	Onthophagus taurus	Putative uncharacterized protein
EOG091200SG	AGLA001754	Anoplophora glabripennis	Putative uncharacterized protein
EOG091200SG	APLA011635	Agrilus planipennis	Putative uncharacterized protein
EOG091200SG	TC014256	Tribolium castaneum	Putative uncharacterized protein
EOG091200SG	LDEC006188	Leptinotarsa decemlineata	Putative uncharacterized protein
EOG091200SG	YQE_11562	Dendroctonus ponderosae	Putative uncharacterized protein
EOG091200SI	OTAU003807	Onthophagus taurus	Semaphorin-1a-like protein
EOG091200SI	AGLA008397	Anoplophora glabripennis	Semaphorin-1a-like protein
EOG091200SI	APLA008176	Agrilus planipennis	Semaphorin-1a-like protein
EOG091200SI	TC010143	Tribolium castaneum	Semaphorin-1a-like protein

EOG091200SI	LDEC002132	Leptinotarsa decemlineata	Semaphorin-1a-like protein
EOG091200SI	YQE_05234	Dendroctonus ponderosae	Semaphorin-1a-like protein
EOG091200SJ	OTAU002592	Onthophagus taurus	Putative uncharacterized protein
EOG091200SJ	AGLA014399	Anoplophora glabripennis	Putative uncharacterized protein
EOG091200SJ	APLA008516	Agrilus planipennis	Putative uncharacterized protein
EOG091200SJ	TC014708	Tribolium castaneum	Putative uncharacterized protein
EOG091200SJ	LDEC006285	Leptinotarsa decemlineata	Putative uncharacterized protein
EOG091200SJ	YQE_08591	Dendroctonus ponderosae	Putative uncharacterized protein
EOG091200SK	OTAU008620	Onthophagus taurus	Putative uncharacterized protein
EOG091200SK	AGLA007827	Anoplophora glabripennis	Putative uncharacterized protein
EOG091200SK	APLA003218	Agrilus planipennis	Putative uncharacterized protein
EOG091200SK	TC001677	Tribolium castaneum	Putative uncharacterized protein
EOG091200SK	LDEC004273	Leptinotarsa decemlineata	Putative uncharacterized protein
EOG091200SK	YQE_10432	Dendroctonus ponderosae	Putative uncharacterized protein
EOG091200SL	OTAU013729	Onthophagus taurus	Putative uncharacterized protein
EOG091200SL	AGLA018539	Anoplophora glabripennis	Putative uncharacterized protein
EOG091200SL	APLA003655	Agrilus planipennis	Putative uncharacterized protein
EOG091200SL	TC004619	Tribolium castaneum	Putative uncharacterized protein
EOG091200SL	LDEC008797	Leptinotarsa decemlineata	Putative uncharacterized protein
EOG091200SL	YQE_03334	Dendroctonus ponderosae	Putative uncharacterized protein
EOG091200SN	OTAU001373	Onthophagus taurus	Trachealess
EOG091200SN	AGLA000355	Anoplophora glabripennis	Trachealess
EOG091200SN	APLA003635	Agrilus planipennis	Trachealess
EOG091200SN	TC001448	Tribolium castaneum	Trachealess
EOG091200SN	LDEC011476	Leptinotarsa decemlineata	Trachealess
EOG091200SN	YQE_03307	Dendroctonus ponderosae	Trachealess
EOG091200SO	OTAU005514	Onthophagus taurus	Putative uncharacterized protein
EOG091200SO	AGLA009851	Anoplophora glabripennis	Putative uncharacterized protein
EOG091200SO	APLA007415	Agrilus planipennis	Putative uncharacterized protein
EOG091200SO	TC012073	Tribolium castaneum	Putative uncharacterized protein
EOG091200SO	LDEC015695	Leptinotarsa decemlineata	Putative uncharacterized protein
EOG091200SO	YQE_07599	Dendroctonus ponderosae	Putative uncharacterized protein
EOG091200SP	OTAU006211	Onthophagus taurus	ABC transmembrane transporter
EOG091200SP	AGLA010510	Anoplophora glabripennis	ABC transmembrane transporter
EOG091200SP	APLA002526	Agrilus planipennis	ABC transmembrane transporter
EOG091200SP	TC011997	Tribolium castaneum	ABC transmembrane transporter
EOG091200SP	LDEC015760	Leptinotarsa decemlineata	ABC transmembrane transporter
EOG091200SP	YQE_01995	Dendroctonus ponderosae	ABC transmembrane transporter
EOG091200SR	OTAU004119	Onthophagus taurus	Prolyl oligopeptidase
EOG091200SR	AGLA000387	Anoplophora glabripennis	Prolyl oligopeptidase
EOG091200SR	APLA003578	Agrilus planipennis	Prolyl oligopeptidase
EOG091200SR	TC013574	Tribolium castaneum	Prolyl oligopeptidase
EOG091200SR	LDEC004682	Leptinotarsa decemlineata	Prolyl oligopeptidase
EOG091200SR	YQE_09142	Dendroctonus ponderosae	Prolyl oligopeptidase
EOG091200SS	OTAU013826	Onthophagus taurus	Putative uncharacterized protein
EOG091200SS	AGLA020961	Anoplophora glabripennis	Putative uncharacterized protein
EOG091200SS	APLA006682	Agrilus planipennis	Putative uncharacterized protein
EOG091200SS	TC003130	Tribolium castaneum	Putative uncharacterized protein
EOG091200SS	LDEC002674	Leptinotarsa decemlineata	Putative uncharacterized protein
EOG091200SS	YQE_02285	Dendroctonus ponderosae	Putative uncharacterized protein
EOG091200SU	OTAU000161	Onthophagus taurus	Putative uncharacterized protein
EOG091200SU	AGLA014799	Anoplophora glabripennis	Putative uncharacterized protein
EOG091200SU	APLA007202	Agrilus planipennis	Putative uncharacterized protein
EOG091200SU	TC014334	Tribolium castaneum	Putative uncharacterized protein
EOG091200SU	LDEC006699	Leptinotarsa decemlineata	Putative uncharacterized protein
EOG091200SU	YQE_02673	Dendroctonus ponderosae	Putative uncharacterized protein
EOG091200SX	OTAU007647	Onthophagus taurus	Putative uncharacterized protein
EOG091200SX	AGLA011812	Anoplophora glabripennis	Putative uncharacterized protein
EOG091200SX	APLA007146	Agrilus planipennis	Putative uncharacterized protein
EOG091200SX	TC009108	Tribolium castaneum	Putative uncharacterized protein
EOG091200SX	LDEC001281	Leptinotarsa decemlineata	Putative uncharacterized protein
EOG091200SX	YQE_05438	Dendroctonus ponderosae	Putative uncharacterized protein
EOG091200SY	OTAU008238	Onthophagus taurus	Putative uncharacterized protein
EOG091200SY	AGLA017332	Anoplophora glabripennis	Putative uncharacterized protein
EOG091200SY	APLA012183	Agrilus planipennis	Putative uncharacterized protein
EOG091200SY	TC001278	Tribolium castaneum	Putative uncharacterized protein
EOG091200SY	LDEC000769	Leptinotarsa decemlineata	Putative uncharacterized protein

EOG091200SY	YQE_09579	Dendroctonus ponderosae	Putative uncharacterized protein
EOG091200T0	OTAU009063	Onthophagus taurus	GTPase-activating protein 1
EOG091200T0	AGLA018031	Anoplophora glabripennis	GTPase-activating protein 1
EOG091200T0	APLA006202	Agrilus planipennis	GTPase-activating protein 1
EOG091200T0	TC014250	Tribolium castaneum	GTPase-activating protein 1
EOG091200T0	LDEC023327	Leptinotarsa decemlineata	GTPase-activating protein 1
EOG091200T0	YQE_06228	Dendroctonus ponderosae	GTPase-activating protein 1
EOG091200T1	OTAU013038	Onthophagus taurus	zinc ion binding
EOG091200T1	AGLA015578	Anoplophora glabripennis	zinc ion binding
EOG091200T1	APLA000603	Agrilus planipennis	zinc ion binding
EOG091200T1	TC031269	Tribolium castaneum	zinc ion binding
EOG091200T1	LDEC019417	Leptinotarsa decemlineata	zinc ion binding
EOG091200T1	YQE_11077	Dendroctonus ponderosae	zinc ion binding
EOG091200T2	OTAU008359	Onthophagus taurus	Putative uncharacterized protein
EOG091200T2	AGLA018768	Anoplophora glabripennis	Putative uncharacterized protein
EOG091200T2	APLA007945	Agrilus planipennis	Putative uncharacterized protein
EOG091200T2	TC001039	Tribolium castaneum	Putative uncharacterized protein
EOG091200T2	LDEC003147	Leptinotarsa decemlineata	Putative uncharacterized protein
EOG091200T2	YQE_06625	Dendroctonus ponderosae	Putative uncharacterized protein
EOG091200T4	OTAU011087	Onthophagus taurus	None
EOG091200T4	AGLA007821	Anoplophora glabripennis	None
EOG091200T4	APLA015076	Agrilus planipennis	None
EOG091200T4	TC034265	Tribolium castaneum	None
EOG091200T4	LDEC004265	Leptinotarsa decemlineata	None
EOG091200T4	YQE_07333	Dendroctonus ponderosae	None
EOG091200T5	OTAU000253	Onthophagus taurus	Putative uncharacterized protein
EOG091200T5	AGLA018194	Anoplophora glabripennis	Putative uncharacterized protein
EOG091200T5	APLA003622	Agrilus planipennis	Putative uncharacterized protein
EOG091200T5	TC013946	Tribolium castaneum	Putative uncharacterized protein
EOG091200T5	LDEC009513	Leptinotarsa decemlineata	Putative uncharacterized protein
EOG091200T5	YQE_06143	Dendroctonus ponderosae	Putative uncharacterized protein
EOG091200T7	OTAU007365	Onthophagus taurus	Putative uncharacterized protein
EOG091200T7	AGLA011578	Anoplophora glabripennis	Putative uncharacterized protein
EOG091200T7	APLA007878	Agrilus planipennis	Putative uncharacterized protein
EOG091200T7	TC010709	Tribolium castaneum	Putative uncharacterized protein
EOG091200T7	LDEC007786	Leptinotarsa decemlineata	Putative uncharacterized protein
EOG091200T7	YQE_12099	Dendroctonus ponderosae	Putative uncharacterized protein
EOG091200TA	OTAU001900	Onthophagus taurus	Ubiquitin carboxyl-terminal hydrolase
EOG091200TA	AGLA005319	Anoplophora glabripennis	Ubiquitin carboxyl-terminal hydrolase
EOG091200TA	APLA001235	Agrilus planipennis	Ubiquitin carboxyl-terminal hydrolase
EOG091200TA	TC006203	Tribolium castaneum	Ubiquitin carboxyl-terminal hydrolase
EOG091200TA	LDEC005876	Leptinotarsa decemlineata	Ubiquitin carboxyl-terminal hydrolase
EOG091200TA	YQE_12716	Dendroctonus ponderosae	Ubiquitin carboxyl-terminal hydrolase
EOG091200TB	OTAU009661	Onthophagus taurus	Putative uncharacterized protein
EOG091200TB	AGLA019308	Anoplophora glabripennis	Putative uncharacterized protein
EOG091200TB	APLA010151	Agrilus planipennis	Putative uncharacterized protein
EOG091200TB	TC007752	Tribolium castaneum	Putative uncharacterized protein
EOG091200TB	LDEC000101	Leptinotarsa decemlineata	Putative uncharacterized protein
EOG091200TB	YQE_08704	Dendroctonus ponderosae	Putative uncharacterized protein
EOG091200TC	OTAU001390	Onthophagus taurus	Putative uncharacterized protein
EOG091200TC	AGLA004538	Anoplophora glabripennis	Putative uncharacterized protein
EOG091200TC	APLA006370	Agrilus planipennis	Putative uncharacterized protein
EOG091200TC	TC005164	Tribolium castaneum	Putative uncharacterized protein
EOG091200TC	LDEC006826	Leptinotarsa decemlineata	Putative uncharacterized protein
EOG091200TC	YQE_06670	Dendroctonus ponderosae	Putative uncharacterized protein
EOG091200TE	OTAU004307	Onthophagus taurus	None
EOG091200TE	AGLA013114	Anoplophora glabripennis	None
EOG091200TE	APLA010014	Agrilus planipennis	None
EOG091200TE	TC031573	Tribolium castaneum	None
EOG091200TE	LDEC005950	Leptinotarsa decemlineata	None
EOG091200TE	YQE_09066	Dendroctonus ponderosae	None
EOG091200TF	OTAU005901	Onthophagus taurus	Putative uncharacterized protein
EOG091200TF	AGLA007884	Anoplophora glabripennis	Putative uncharacterized protein
EOG091200TF	APLA006857	Agrilus planipennis	Putative uncharacterized protein
EOG091200TF	TC014440	Tribolium castaneum	Putative uncharacterized protein
EOG091200TF	LDEC009033	Leptinotarsa decemlineata	Putative uncharacterized protein
EOG091200TF	YQE_06117	Dendroctonus ponderosae	Putative uncharacterized protein

EOG091200TG	OTAU014030	Onthophagus taurus	Putative uncharacterized protein
EOG091200TG	AGLA011180	Anoplophora glabripennis	Putative uncharacterized protein
EOG091200TG	APLA001076	Agrilus planipennis	Putative uncharacterized protein
EOG091200TG	TC011713	Tribolium castaneum	Putative uncharacterized protein
EOG091200TG	LDEC018636	Leptinotarsa decemlineata	Putative uncharacterized protein
EOG091200TG	YQE_08501	Dendroctonus ponderosae	Putative uncharacterized protein
EOG091200TK	OTAU007740	Onthophagus taurus	zinc ion binding
EOG091200TK	AGLA007881	Anoplophora glabripennis	zinc ion binding
EOG091200TK	APLA008509	Agrilus planipennis	zinc ion binding
EOG091200TK	TC033165	Tribolium castaneum	zinc ion binding
EOG091200TK	LDEC009030	Leptinotarsa decemlineata	zinc ion binding
EOG091200TK	YQE_06118	Dendroctonus ponderosae	zinc ion binding
EOG091200TN	OTAU006216	Onthophagus taurus	Putative uncharacterized protein
EOG091200TN	AGLA003487	Anoplophora glabripennis	Putative uncharacterized protein
EOG091200TN	APLA008135	Agrilus planipennis	Putative uncharacterized protein
EOG091200TN	TC010479	Tribolium castaneum	Putative uncharacterized protein
EOG091200TN	LDEC004034	Leptinotarsa decemlineata	Putative uncharacterized protein
EOG091200TN	YQE_09893	Dendroctonus ponderosae	Putative uncharacterized protein
EOG091200TP	OTAU004940	Onthophagus taurus	Putative uncharacterized protein
EOG091200TP	AGLA015347	Anoplophora glabripennis	Putative uncharacterized protein
EOG091200TP	APLA015383	Agrilus planipennis	Putative uncharacterized protein
EOG091200TP	TC000558	Tribolium castaneum	Putative uncharacterized protein
EOG091200TP	LDEC007120	Leptinotarsa decemlineata	Putative uncharacterized protein
EOG091200TP	YQE_10979	Dendroctonus ponderosae	Putative uncharacterized protein
EOG091200TQ	OTAU004292	Onthophagus taurus	Putative uncharacterized protein
EOG091200TQ	AGLA018930	Anoplophora glabripennis	Putative uncharacterized protein
EOG091200TQ	APLA010369	Agrilus planipennis	Putative uncharacterized protein
EOG091200TQ	TC004758	Tribolium castaneum	Putative uncharacterized protein
EOG091200TQ	LDEC011081	Leptinotarsa decemlineata	Putative uncharacterized protein
EOG091200TQ	YQE_06729	Dendroctonus ponderosae	Putative uncharacterized protein
EOG091200TR	OTAU006871	Onthophagus taurus	Putative uncharacterized protein
EOG091200TR	AGLA011389	Anoplophora glabripennis	Putative uncharacterized protein
EOG091200TR	APLA011748	Agrilus planipennis	Putative uncharacterized protein
EOG091200TR	TC013055	Tribolium castaneum	Putative uncharacterized protein
EOG091200TR	LDEC003228	Leptinotarsa decemlineata	Putative uncharacterized protein
EOG091200TR	YQE_07226	Dendroctonus ponderosae	Putative uncharacterized protein
EOG091200TV	OTAU009570	Onthophagus taurus	Argonaute 3
EOG091200TV	AGLA010388	Anoplophora glabripennis	Argonaute 3
EOG091200TV	APLA015264	Agrilus planipennis	Argonaute 3
EOG091200TV	TC008511	Tribolium castaneum	Argonaute 3
EOG091200TV	LDEC003980	Leptinotarsa decemlineata	Argonaute 3
EOG091200TV	YQE_10018	Dendroctonus ponderosae	Argonaute 3
EOG091200U0	OTAU003006	Onthophagus taurus	Putative uncharacterized protein
EOG091200U0	AGLA006610	Anoplophora glabripennis	Putative uncharacterized protein
EOG091200U0	APLA002331	Agrilus planipennis	Putative uncharacterized protein
EOG091200U0	TC012422	Tribolium castaneum	Putative uncharacterized protein
EOG091200U0	LDEC007674	Leptinotarsa decemlineata	Putative uncharacterized protein
EOG091200U0	YQE_07313	Dendroctonus ponderosae	Putative uncharacterized protein
EOG091200U2	OTAU000002	Onthophagus taurus	Putative uncharacterized protein
EOG091200U2	AGLA004345	Anoplophora glabripennis	Putative uncharacterized protein
EOG091200U2	APLA008083	Agrilus planipennis	Putative uncharacterized protein
EOG091200U2	TC013782	Tribolium castaneum	Putative uncharacterized protein
EOG091200U2	LDEC014577	Leptinotarsa decemlineata	Putative uncharacterized protein
EOG091200U2	YQE_06154	Dendroctonus ponderosae	Putative uncharacterized protein
EOG091200U4	OTAU004654	Onthophagus taurus	Putative uncharacterized protein
EOG091200U4	AGLA010751	Anoplophora glabripennis	Putative uncharacterized protein
EOG091200U4	APLA013404	Agrilus planipennis	Putative uncharacterized protein
EOG091200U4	TC002742	Tribolium castaneum	Putative uncharacterized protein
EOG091200U4	LDEC000449	Leptinotarsa decemlineata	Putative uncharacterized protein
EOG091200U4	YQE_07406	Dendroctonus ponderosae	Putative uncharacterized protein
EOG091200U5	OTAU000628	Onthophagus taurus	Putative uncharacterized protein
EOG091200U5	AGLA011963	Anoplophora glabripennis	Putative uncharacterized protein
EOG091200U5	APLA002122	Agrilus planipennis	Putative uncharacterized protein
EOG091200U5	TC012117	Tribolium castaneum	Putative uncharacterized protein
EOG091200U5	LDEC009815	Leptinotarsa decemlineata	Putative uncharacterized protein
EOG091200U5	YQE_08450	Dendroctonus ponderosae	Putative uncharacterized protein
EOG091200U7	OTAU012946	Onthophagus taurus	Putative uncharacterized protein

EOG091200U7	AGLA000394	Anoplophora glabripennis	Putative uncharacterized protein
EOG091200U7	APLA013631	Agrilus planipennis	Putative uncharacterized protein
EOG091200U7	TC013578	Tribolium castaneum	Putative uncharacterized protein
EOG091200U7	LDEC004688	Leptinotarsa decemlineata	Putative uncharacterized protein
EOG091200U7	YQE_06636	Dendroctonus ponderosae	Putative uncharacterized protein
EOG091200U9	OTAU002753	Onthophagus taurus	Serine protease P100
EOG091200U9	AGLA000241	Anoplophora glabripennis	Serine protease P100
EOG091200U9	APLA013954	Agrilus planipennis	Serine protease P100
EOG091200U9	TC010781	Tribolium castaneum	Serine protease P100
EOG091200U9	LDEC001041	Leptinotarsa decemlineata	Serine protease P100
EOG091200U9	YQE_09108	Dendroctonus ponderosae	Serine protease P100
EOG091200UB	OTAU005065	Onthophagus taurus	Putative uncharacterized protein
EOG091200UB	AGLA001646	Anoplophora glabripennis	Putative uncharacterized protein
EOG091200UB	APLA000382	Agrilus planipennis	Putative uncharacterized protein
EOG091200UB	TC005995	Tribolium castaneum	Putative uncharacterized protein
EOG091200UB	LDEC015040	Leptinotarsa decemlineata	Putative uncharacterized protein
EOG091200UB	YQE_08542	Dendroctonus ponderosae	Putative uncharacterized protein
EOG091200UC	OTAU016068	Onthophagus taurus	Putative uncharacterized protein
EOG091200UC	AGLA009863	Anoplophora glabripennis	Putative uncharacterized protein
EOG091200UC	APLA008079	Agrilus planipennis	Putative uncharacterized protein
EOG091200UC	TC013109	Tribolium castaneum	Putative uncharacterized protein
EOG091200UC	LDEC006271	Leptinotarsa decemlineata	Putative uncharacterized protein
EOG091200UC	YQE_08659	Dendroctonus ponderosae	Putative uncharacterized protein
EOG091200UF	OTAU010921	Onthophagus taurus	Putative uncharacterized protein
EOG091200UF	AGLA009472	Anoplophora glabripennis	Putative uncharacterized protein
EOG091200UF	APLA014386	Agrilus planipennis	Putative uncharacterized protein
EOG091200UF	TC015003	Tribolium castaneum	Putative uncharacterized protein
EOG091200UF	LDEC002240	Leptinotarsa decemlineata	Putative uncharacterized protein
EOG091200UF	YQE_11727	Dendroctonus ponderosae	Putative uncharacterized protein
EOG091200UG	OTAU003172	Onthophagus taurus	Putative uncharacterized protein
EOG091200UG	AGLA012670	Anoplophora glabripennis	Putative uncharacterized protein
EOG091200UG	APLA009094	Agrilus planipennis	Putative uncharacterized protein
EOG091200UG	TC006719	Tribolium castaneum	Putative uncharacterized protein
EOG091200UG	LDEC006749	Leptinotarsa decemlineata	Putative uncharacterized protein
EOG091200UG	YQE_06581	Dendroctonus ponderosae	Putative uncharacterized protein
EOG091200UH	OTAU005025	Onthophagus taurus	Hormone receptor in 39-like protein
EOG091200UH	AGLA014161	Anoplophora glabripennis	Hormone receptor in 39-like protein
EOG091200UH	APLA007971	Agrilus planipennis	Hormone receptor in 39-like protein
EOG091200UH	TC014986	Tribolium castaneum	Hormone receptor in 39-like protein
EOG091200UH	LDEC010317	Leptinotarsa decemlineata	Hormone receptor in 39-like protein
EOG091200UH	YQE_10672	Dendroctonus ponderosae	Hormone receptor in 39-like protein
EOG091200UJ	OTAU015332	Onthophagus taurus	Putative uncharacterized protein
EOG091200UJ	AGLA000016	Anoplophora glabripennis	Putative uncharacterized protein
EOG091200UJ	APLA008005	Agrilus planipennis	Putative uncharacterized protein
EOG091200UJ	TC000974	Tribolium castaneum	Putative uncharacterized protein
EOG091200UJ	LDEC013063	Leptinotarsa decemlineata	Putative uncharacterized protein
EOG091200UJ	YQE_10721	Dendroctonus ponderosae	Putative uncharacterized protein
EOG091200UK	OTAU001316	Onthophagus taurus	Putative uncharacterized protein
EOG091200UK	AGLA013820	Anoplophora glabripennis	Putative uncharacterized protein
EOG091200UK	APLA010657	Agrilus planipennis	Putative uncharacterized protein
EOG091200UK	TC012410	Tribolium castaneum	Putative uncharacterized protein
EOG091200UK	LDEC009543	Leptinotarsa decemlineata	Putative uncharacterized protein
EOG091200UK	YQE_04501	Dendroctonus ponderosae	Putative uncharacterized protein
EOG091200UL	OTAU010056	Onthophagus taurus	Putative uncharacterized protein
EOG091200UL	AGLA000847	Anoplophora glabripennis	Putative uncharacterized protein
EOG091200UL	APLA002262	Agrilus planipennis	Putative uncharacterized protein
EOG091200UL	TC007565	Tribolium castaneum	Putative uncharacterized protein
EOG091200UL	LDEC018657	Leptinotarsa decemlineata	Putative uncharacterized protein
EOG091200UL	YQE_04736	Dendroctonus ponderosae	Putative uncharacterized protein
EOG091200UM	OTAU010889	Onthophagus taurus	Putative uncharacterized protein
EOG091200UM	AGLA001519	Anoplophora glabripennis	Putative uncharacterized protein
EOG091200UM	APLA006669	Agrilus planipennis	Putative uncharacterized protein
EOG091200UM	TC002627	Tribolium castaneum	Putative uncharacterized protein
EOG091200UM	LDEC013431	Leptinotarsa decemlineata	Putative uncharacterized protein
EOG091200UM	YQE_05150	Dendroctonus ponderosae	Putative uncharacterized protein
EOG091200UP	OTAU015535	Onthophagus taurus	Putative uncharacterized protein
EOG091200UP	AGLA001086	Anoplophora glabripennis	Putative uncharacterized protein

EOG091200UP	APLA015213	Agrilus planipennis	Putative uncharacterized protein
EOG091200UP	TC015674	Tribolium castaneum	Putative uncharacterized protein
EOG091200UP	LDEC011751	Leptinotarsa decemlineata	Putative uncharacterized protein
EOG091200UP	YQE_11875	Dendroctonus ponderosae	Putative uncharacterized protein
EOG091200UR	OTAU001448	Onthophagus taurus	Semaphorin-1a-like protein
EOG091200UR	AGLA002366	Anoplophora glabripennis	Semaphorin-1a-like protein
EOG091200UR	APLA010634	Agrilus planipennis	Semaphorin-1a-like protein
EOG091200UR	TC014179	Tribolium castaneum	Semaphorin-1a-like protein
EOG091200UR	LDEC012575	Leptinotarsa decemlineata	Semaphorin-1a-like protein
EOG091200UR	YQE_02477	Dendroctonus ponderosae	Semaphorin-1a-like protein
EOG091200UU	OTAU002568	Onthophagus taurus	Putative uncharacterized protein
EOG091200UU	AGLA019783	Anoplophora glabripennis	Putative uncharacterized protein
EOG091200UU	APLA013123	Agrilus planipennis	Putative uncharacterized protein
EOG091200UU	TC014633	Tribolium castaneum	Putative uncharacterized protein
EOG091200UU	LDEC005967	Leptinotarsa decemlineata	Putative uncharacterized protein
EOG091200UU	YQE_06192	Dendroctonus ponderosae	Putative uncharacterized protein
EOG091200UV	OTAU016574	Onthophagus taurus	Putative uncharacterized protein
EOG091200UV	AGLA001833	Anoplophora glabripennis	Putative uncharacterized protein
EOG091200UV	APLA011981	Agrilus planipennis	Putative uncharacterized protein
EOG091200UV	TC011927	Tribolium castaneum	Putative uncharacterized protein
EOG091200UV	LDEC005773	Leptinotarsa decemlineata	Putative uncharacterized protein
EOG091200UV	YQE_10916	Dendroctonus ponderosae	Putative uncharacterized protein
EOG091200UX	OTAU008178	Onthophagus taurus	Putative uncharacterized protein
EOG091200UX	AGLA013711	Anoplophora glabripennis	Putative uncharacterized protein
EOG091200UX	APLA005943	Agrilus planipennis	Putative uncharacterized protein
EOG091200UX	TC015207	Tribolium castaneum	Putative uncharacterized protein
EOG091200UX	LDEC012241	Leptinotarsa decemlineata	Putative uncharacterized protein
EOG091200UX	YQE_11721	Dendroctonus ponderosae	Putative uncharacterized protein
EOG091200UY	OTAU006169	Onthophagus taurus	Putative uncharacterized protein
EOG091200UY	AGLA005781	Anoplophora glabripennis	Putative uncharacterized protein
EOG091200UY	APLA005987	Agrilus planipennis	Putative uncharacterized protein
EOG091200UY	TC014536	Tribolium castaneum	Putative uncharacterized protein
EOG091200UY	LDEC006415	Leptinotarsa decemlineata	Putative uncharacterized protein
EOG091200UY	YQE_05228	Dendroctonus ponderosae	Putative uncharacterized protein
EOG091200UZ	OTAU000137	Onthophagus taurus	Putative uncharacterized protein
EOG091200UZ	AGLA019787	Anoplophora glabripennis	Putative uncharacterized protein
EOG091200UZ	APLA003034	Agrilus planipennis	Putative uncharacterized protein
EOG091200UZ	TC013883	Tribolium castaneum	Putative uncharacterized protein
EOG091200UZ	LDEC007762	Leptinotarsa decemlineata	Putative uncharacterized protein
EOG091200UZ	YQE_06195	Dendroctonus ponderosae	Putative uncharacterized protein
EOG091200V1	OTAU006256	Onthophagus taurus	Putative uncharacterized protein
EOG091200V1	AGLA011809	Anoplophora glabripennis	Putative uncharacterized protein
EOG091200V1	APLA010852	Agrilus planipennis	Putative uncharacterized protein
EOG091200V1	TC009205	Tribolium castaneum	Putative uncharacterized protein
EOG091200V1	LDEC016651	Leptinotarsa decemlineata	Putative uncharacterized protein
EOG091200V1	YQE_05430	Dendroctonus ponderosae	Putative uncharacterized protein
EOG091200V3	OTAU007203	Onthophagus taurus	Putative uncharacterized protein
EOG091200V3	AGLA003474	Anoplophora glabripennis	Putative uncharacterized protein
EOG091200V3	APLA005952	Agrilus planipennis	Putative uncharacterized protein
EOG091200V3	TC006094	Tribolium castaneum	Putative uncharacterized protein
EOG091200V3	LDEC018109	Leptinotarsa decemlineata	Putative uncharacterized protein
EOG091200V3	YQE_02172	Dendroctonus ponderosae	Putative uncharacterized protein
EOG091200V4	OTAU006557	Onthophagus taurus	Putative uncharacterized protein
EOG091200V4	AGLA016219	Anoplophora glabripennis	Putative uncharacterized protein
EOG091200V4	APLA003748	Agrilus planipennis	Putative uncharacterized protein
EOG091200V4	TC002906	Tribolium castaneum	Putative uncharacterized protein
EOG091200V4	LDEC019783	Leptinotarsa decemlineata	Putative uncharacterized protein
EOG091200V4	YQE_09748	Dendroctonus ponderosae	Putative uncharacterized protein
EOG091200V5	OTAU007767	Onthophagus taurus	None
EOG091200V5	AGLA000920	Anoplophora glabripennis	None
EOG091200V5	APLA012216	Agrilus planipennis	None
EOG091200V5	TC033757	Tribolium castaneum	None
EOG091200V5	LDEC009339	Leptinotarsa decemlineata	None
EOG091200V5	YQE_05336	Dendroctonus ponderosae	None
EOG091200V6	OTAU004993	Onthophagus taurus	Putative uncharacterized protein
EOG091200V6	AGLA010161	Anoplophora glabripennis	Putative uncharacterized protein
EOG091200V6	APLA006330	Agrilus planipennis	Putative uncharacterized protein

EOG091200V6	TC011382	Tribolium castaneum	Putative uncharacterized protein
EOG091200V6	LDEC002776	Leptinotarsa decemlineata	Putative uncharacterized protein
EOG091200V6	YQE_09906	Dendroctonus ponderosae	Putative uncharacterized protein
EOG091200V9	OTAU008469	Onthophagus taurus	Spineless
EOG091200V9	AGLA000173	Anoplophora glabripennis	Spineless
EOG091200V9	APLA001941	Agrilus planipennis	Spineless
EOG091200V9	TC011105	Tribolium castaneum	Spineless
EOG091200V9	LDEC012674	Leptinotarsa decemlineata	Spineless
EOG091200V9	YQE_01597	Dendroctonus ponderosae	Spineless
EOG091200VC	OTAU009250	Onthophagus taurus	Putative uncharacterized protein
EOG091200VC	AGLA004440	Anoplophora glabripennis	Putative uncharacterized protein
EOG091200VC	APLA008270	Agrilus planipennis	Putative uncharacterized protein
EOG091200VC	TC014606	Tribolium castaneum	Putative uncharacterized protein
EOG091200VC	LDEC001198	Leptinotarsa decemlineata	Putative uncharacterized protein
EOG091200VC	YQE_06136	Dendroctonus ponderosae	Putative uncharacterized protein
EOG091200VD	OTAU003453	Onthophagus taurus	Putative uncharacterized protein
EOG091200VD	AGLA013864	Anoplophora glabripennis	Putative uncharacterized protein
EOG091200VD	APLA006195	Agrilus planipennis	Putative uncharacterized protein
EOG091200VD	TC009491	Tribolium castaneum	Putative uncharacterized protein
EOG091200VD	LDEC019906	Leptinotarsa decemlineata	Putative uncharacterized protein
EOG091200VD	YQE_05397	Dendroctonus ponderosae	Putative uncharacterized protein
EOG091200VE	OTAU009226	Onthophagus taurus	catalytic activity
EOG091200VE	AGLA010758	Anoplophora glabripennis	catalytic activity
EOG091200VE	APLA005444	Agrilus planipennis	catalytic activity
EOG091200VE	TC031030	Tribolium castaneum	catalytic activity
EOG091200VE	LDEC016713	Leptinotarsa decemlineata	catalytic activity
EOG091200VE	YQE_09888	Dendroctonus ponderosae	catalytic activity
EOG091200VG	OTAU012486	Onthophagus taurus	Putative uncharacterized protein
EOG091200VG	AGLA008515	Anoplophora glabripennis	Putative uncharacterized protein
EOG091200VG	APLA005774	Agrilus planipennis	Putative uncharacterized protein
EOG091200VG	TC006621	Tribolium castaneum	Putative uncharacterized protein
EOG091200VG	LDEC017113	Leptinotarsa decemlineata	Putative uncharacterized protein
EOG091200VG	YQE_12496	Dendroctonus ponderosae	Putative uncharacterized protein
EOG091200VH	OTAU002981	Onthophagus taurus	Putative uncharacterized protein
EOG091200VH	AGLA015214	Anoplophora glabripennis	Putative uncharacterized protein
EOG091200VH	APLA004611	Agrilus planipennis	Putative uncharacterized protein
EOG091200VH	TC009329	Tribolium castaneum	Putative uncharacterized protein
EOG091200VH	LDEC004448	Leptinotarsa decemlineata	Putative uncharacterized protein
EOG091200VH	YQE_08783	Dendroctonus ponderosae	Putative uncharacterized protein
EOG091200VI	OTAU003197	Onthophagus taurus	Putative uncharacterized protein
EOG091200VI	AGLA007371	Anoplophora glabripennis	Putative uncharacterized protein
EOG091200VI	APLA002756	Agrilus planipennis	Putative uncharacterized protein
EOG091200VI	TC014301	Tribolium castaneum	Putative uncharacterized protein
EOG091200VI	LDEC008337	Leptinotarsa decemlineata	Putative uncharacterized protein
EOG091200VI	YQE_06214	Dendroctonus ponderosae	Putative uncharacterized protein
EOG091200VK	OTAU011020	Onthophagus taurus	Putative uncharacterized protein
EOG091200VK	AGLA006562	Anoplophora glabripennis	Putative uncharacterized protein
EOG091200VK	APLA009518	Agrilus planipennis	Putative uncharacterized protein
EOG091200VK	TC000377	Tribolium castaneum	Putative uncharacterized protein
EOG091200VK	LDEC001657	Leptinotarsa decemlineata	Putative uncharacterized protein
EOG091200VK	YQE_10946	Dendroctonus ponderosae	Putative uncharacterized protein
EOG091200VL	OTAU005111	Onthophagus taurus	Putative uncharacterized protein
EOG091200VL	AGLA003398	Anoplophora glabripennis	Putative uncharacterized protein
EOG091200VL	APLA006661	Agrilus planipennis	Putative uncharacterized protein
EOG091200VL	TC002914	Tribolium castaneum	Putative uncharacterized protein
EOG091200VL	LDEC010041	Leptinotarsa decemlineata	Putative uncharacterized protein
EOG091200VL	YQE_01854	Dendroctonus ponderosae	Putative uncharacterized protein
EOG091200VN	OTAU011520	Onthophagus taurus	Putative uncharacterized protein
EOG091200VN	AGLA004694	Anoplophora glabripennis	Putative uncharacterized protein
EOG091200VN	APLA010306	Agrilus planipennis	Putative uncharacterized protein
EOG091200VN	TC007009	Tribolium castaneum	Putative uncharacterized protein
EOG091200VN	LDEC004381	Leptinotarsa decemlineata	Putative uncharacterized protein
EOG091200VN	YQE_01827	Dendroctonus ponderosae	Putative uncharacterized protein
EOG091200VO	OTAU000344	Onthophagus taurus	Putative uncharacterized protein
EOG091200VO	AGLA005458	Anoplophora glabripennis	Putative uncharacterized protein
EOG091200VO	APLA010431	Agrilus planipennis	Putative uncharacterized protein
EOG091200VO	TC011944	Tribolium castaneum	Putative uncharacterized protein

EOG091200VO	LDEC018185	Leptinotarsa decemlineata	Putative uncharacterized protein
EOG091200VO	YQE_04307	Dendroctonus ponderosae	Putative uncharacterized protein
EOG091200VT	OTAU011772	Onthophagus taurus	Putative uncharacterized protein
EOG091200VT	AGLA003851	Anoplophora glabripennis	Putative uncharacterized protein
EOG091200VT	APLA001604	Agrilus planipennis	Putative uncharacterized protein
EOG091200VT	TC008834	Tribolium castaneum	Putative uncharacterized protein
EOG091200VT	LDEC016662	Leptinotarsa decemlineata	Putative uncharacterized protein
EOG091200VT	YQE_05834	Dendroctonus ponderosae	Putative uncharacterized protein
EOG091200VV	OTAU008220	Onthophagus taurus	Transporter
EOG091200VV	AGLA001864	Anoplophora glabripennis	Transporter
EOG091200VV	APLA011685	Agrilus planipennis	Transporter
EOG091200VV	TC000366	Tribolium castaneum	Transporter
EOG091200VV	LDEC008883	Leptinotarsa decemlineata	Transporter
EOG091200VV	YQE_11214	Dendroctonus ponderosae	Transporter
EOG091200VY	OTAU004325	Onthophagus taurus	Putative uncharacterized protein
EOG091200VY	AGLA015678	Anoplophora glabripennis	Putative uncharacterized protein
EOG091200VY	APLA010450	Agrilus planipennis	Putative uncharacterized protein
EOG091200VY	TC005185	Tribolium castaneum	Putative uncharacterized protein
EOG091200VY	LDEC003536	Leptinotarsa decemlineata	Putative uncharacterized protein
EOG091200VY	YQE_09209	Dendroctonus ponderosae	Putative uncharacterized protein
EOG091200W0	OTAU004383	Onthophagus taurus	Transporter
EOG091200W0	AGLA004581	Anoplophora glabripennis	Transporter
EOG091200W0	APLA003499	Agrilus planipennis	Transporter
EOG091200W0	TC013646	Tribolium castaneum	Transporter
EOG091200W0	LDEC001527	Leptinotarsa decemlineata	Transporter
EOG091200W0	YQE_06674	Dendroctonus ponderosae	Transporter
EOG091200W2	OTAU005705	Onthophagus taurus	Putative uncharacterized protein
EOG091200W2	AGLA006186	Anoplophora glabripennis	Putative uncharacterized protein
EOG091200W2	APLA011485	Agrilus planipennis	Putative uncharacterized protein
EOG091200W2	TC003425	Tribolium castaneum	Putative uncharacterized protein
EOG091200W2	LDEC004489	Leptinotarsa decemlineata	Putative uncharacterized protein
EOG091200W2	YQE_07875	Dendroctonus ponderosae	Putative uncharacterized protein
EOG091200W3	OTAU000367	Onthophagus taurus	Putative uncharacterized protein
EOG091200W3	AGLA011372	Anoplophora glabripennis	Putative uncharacterized protein
EOG091200W3	APLA013809	Agrilus planipennis	Putative uncharacterized protein
EOG091200W3	TC012753	Tribolium castaneum	Putative uncharacterized protein
EOG091200W3	LDEC008604	Leptinotarsa decemlineata	Putative uncharacterized protein
EOG091200W3	YQE_10772	Dendroctonus ponderosae	Putative uncharacterized protein
EOG091200W4	OTAU007512	Onthophagus taurus	Putative uncharacterized protein
EOG091200W4	AGLA010439	Anoplophora glabripennis	Putative uncharacterized protein
EOG091200W4	APLA006455	Agrilus planipennis	Putative uncharacterized protein
EOG091200W4	TC015821	Tribolium castaneum	Putative uncharacterized protein
EOG091200W4	LDEC013173	Leptinotarsa decemlineata	Putative uncharacterized protein
EOG091200W4	YQE_10860	Dendroctonus ponderosae	Putative uncharacterized protein
EOG091200W6	OTAU015045	Onthophagus taurus	Putative uncharacterized protein
EOG091200W6	AGLA006484	Anoplophora glabripennis	Putative uncharacterized protein
EOG091200W6	APLA014913	Agrilus planipennis	Putative uncharacterized protein
EOG091200W6	TC009234	Tribolium castaneum	Putative uncharacterized protein
EOG091200W6	LDEC009360	Leptinotarsa decemlineata	Putative uncharacterized protein
EOG091200W6	YQE_03543	Dendroctonus ponderosae	Putative uncharacterized protein
EOG091200W8	OTAU000772	Onthophagus taurus	Putative uncharacterized protein
EOG091200W8	AGLA003406	Anoplophora glabripennis	Putative uncharacterized protein
EOG091200W8	APLA000768	Agrilus planipennis	Putative uncharacterized protein
EOG091200W8	TC010318	Tribolium castaneum	Putative uncharacterized protein
EOG091200W8	LDEC015834	Leptinotarsa decemlineata	Putative uncharacterized protein
EOG091200W8	YQE_12300	Dendroctonus ponderosae	Putative uncharacterized protein
EOG091200W9	OTAU011602	Onthophagus taurus	Putative uncharacterized protein
EOG091200W9	AGLA007187	Anoplophora glabripennis	Putative uncharacterized protein
EOG091200W9	APLA004893	Agrilus planipennis	Putative uncharacterized protein
EOG091200W9	TC009594	Tribolium castaneum	Putative uncharacterized protein
EOG091200W9	LDEC007698	Leptinotarsa decemlineata	Putative uncharacterized protein
EOG091200W9	YQE_11803	Dendroctonus ponderosae	Putative uncharacterized protein
EOG091200WB	OTAU016201	Onthophagus taurus	Putative uncharacterized protein
EOG091200WB	AGLA018257	Anoplophora glabripennis	Putative uncharacterized protein
EOG091200WB	APLA010272	Agrilus planipennis	Putative uncharacterized protein
EOG091200WB	TC009038	Tribolium castaneum	Putative uncharacterized protein
EOG091200WB	LDEC009969	Leptinotarsa decemlineata	Putative uncharacterized protein

EOG091200WB	YQE_08761	Dendroctonus ponderosae	Putative uncharacterized protein
EOG091200WC	OTAU002684	Onthophagus taurus	Putative uncharacterized protein
EOG091200WC	AGLA018462	Anoplophora glabripennis	Putative uncharacterized protein
EOG091200WC	APLA004856	Agrilus planipennis	Putative uncharacterized protein
EOG091200WC	TC013395	Tribolium castaneum	Putative uncharacterized protein
EOG091200WC	LDEC002596	Leptinotarsa decemlineata	Putative uncharacterized protein
EOG091200WC	YQE_06103	Dendroctonus ponderosae	Putative uncharacterized protein
EOG091200WG	OTAU007253	Onthophagus taurus	Putative uncharacterized protein
EOG091200WG	AGLA003278	Anoplophora glabripennis	Putative uncharacterized protein
EOG091200WG	APLA005550	Agrilus planipennis	Putative uncharacterized protein
EOG091200WG	TC011686	Tribolium castaneum	Putative uncharacterized protein
EOG091200WG	LDEC014406	Leptinotarsa decemlineata	Putative uncharacterized protein
EOG091200WG	YQE_07847	Dendroctonus ponderosae	Putative uncharacterized protein
EOG091200WI	OTAU000300	Onthophagus taurus	Putative uncharacterized protein
EOG091200WI	AGLA015394	Anoplophora glabripennis	Putative uncharacterized protein
EOG091200WI	APLA003107	Agrilus planipennis	Putative uncharacterized protein
EOG091200WI	TC014402	Tribolium castaneum	Putative uncharacterized protein
EOG091200WI	LDEC002655	Leptinotarsa decemlineata	Putative uncharacterized protein
EOG091200WI	YQE_03571	Dendroctonus ponderosae	Putative uncharacterized protein
EOG091200WJ	OTAU006093	Onthophagus taurus	Putative uncharacterized protein
EOG091200WJ	AGLA008141	Anoplophora glabripennis	Putative uncharacterized protein
EOG091200WJ	APLA002420	Agrilus planipennis	Putative uncharacterized protein
EOG091200WJ	TC030698	Tribolium castaneum	Putative uncharacterized protein
EOG091200WJ	LDEC007014	Leptinotarsa decemlineata	Putative uncharacterized protein
EOG091200WJ	YQE_07255	Dendroctonus ponderosae	Putative uncharacterized protein
EOG091200WK	OTAU001794	Onthophagus taurus	Putative uncharacterized protein
EOG091200WK	AGLA016591	Anoplophora glabripennis	Putative uncharacterized protein
EOG091200WK	APLA001159	Agrilus planipennis	Putative uncharacterized protein
EOG091200WK	TC005594	Tribolium castaneum	Putative uncharacterized protein
EOG091200WK	LDEC021317	Leptinotarsa decemlineata	Putative uncharacterized protein
EOG091200WK	YQE_07491	Dendroctonus ponderosae	Putative uncharacterized protein
EOG091200WL	OTAU009563	Onthophagus taurus	Putative uncharacterized protein
EOG091200WL	AGLA013804	Anoplophora glabripennis	Putative uncharacterized protein
EOG091200WL	APLA009215	Agrilus planipennis	Putative uncharacterized protein
EOG091200WL	TC007702	Tribolium castaneum	Putative uncharacterized protein
EOG091200WL	LDEC004396	Leptinotarsa decemlineata	Putative uncharacterized protein
EOG091200WL	YQE_04806	Dendroctonus ponderosae	Putative uncharacterized protein
EOG091200WM	OTAU004132	Onthophagus taurus	Putative uncharacterized protein
EOG091200WM	AGLA017802	Anoplophora glabripennis	Putative uncharacterized protein
EOG091200WM	APLA001592	Agrilus planipennis	Putative uncharacterized protein
EOG091200WM	TC009707	Tribolium castaneum	Putative uncharacterized protein
EOG091200WM	LDEC013024	Leptinotarsa decemlineata	Putative uncharacterized protein
EOG091200WM	YQE_08805	Dendroctonus ponderosae	Putative uncharacterized protein
EOG091200WN	OTAU014519	Onthophagus taurus	Putative uncharacterized protein
EOG091200WN	AGLA010586	Anoplophora glabripennis	Putative uncharacterized protein
EOG091200WN	APLA012846	Agrilus planipennis	Putative uncharacterized protein
EOG091200WN	TC015972	Tribolium castaneum	Putative uncharacterized protein
EOG091200WN	LDEC006797	Leptinotarsa decemlineata	Putative uncharacterized protein
EOG091200WN	YQE_06927	Dendroctonus ponderosae	Putative uncharacterized protein
EOG091200WR	OTAU010530	Onthophagus taurus	Putative uncharacterized protein
EOG091200WR	AGLA002057	Anoplophora glabripennis	Putative uncharacterized protein
EOG091200WR	APLA000806	Agrilus planipennis	Putative uncharacterized protein
EOG091200WR	TC001292	Tribolium castaneum	Putative uncharacterized protein
EOG091200WR	LDEC019707	Leptinotarsa decemlineata	Putative uncharacterized protein
EOG091200WR	YQE_03667	Dendroctonus ponderosae	Putative uncharacterized protein
EOG091200WT	OTAU011006	Onthophagus taurus	Putative uncharacterized protein
EOG091200WT	AGLA004564	Anoplophora glabripennis	Putative uncharacterized protein
EOG091200WT	APLA009988	Agrilus planipennis	Putative uncharacterized protein
EOG091200WT	TC010869	Tribolium castaneum	Putative uncharacterized protein
EOG091200WT	LDEC001530	Leptinotarsa decemlineata	Putative uncharacterized protein
EOG091200WT	YQE_09127	Dendroctonus ponderosae	Putative uncharacterized protein
EOG091200WX	OTAU006090	Onthophagus taurus	Putative uncharacterized protein
EOG091200WX	AGLA006389	Anoplophora glabripennis	Putative uncharacterized protein
EOG091200WX	APLA008278	Agrilus planipennis	Putative uncharacterized protein
EOG091200WX	TC014342	Tribolium castaneum	Putative uncharacterized protein
EOG091200WX	LDEC008750	Leptinotarsa decemlineata	Putative uncharacterized protein
EOG091200WX	YQE_04042	Dendroctonus ponderosae	Putative uncharacterized protein

EOG091200WY	OTAU015639	Onthophagus taurus	Putative uncharacterized protein
EOG091200WY	AGLA008003	Anoplophora glabripennis	Putative uncharacterized protein
EOG091200WY	APLA001702	Agrilus planipennis	Putative uncharacterized protein
EOG091200WY	TC030641	Tribolium castaneum	Putative uncharacterized protein
EOG091200WY	LDEC014303	Leptinotarsa decemlineata	Putative uncharacterized protein
EOG091200WY	YQE_01835	Dendroctonus ponderosae	Putative uncharacterized protein
EOG091200WZ	OTAU011460	Onthophagus taurus	Bicaudal D
EOG091200WZ	AGLA016972	Anoplophora glabripennis	Bicaudal D
EOG091200WZ	APLA001600	Agrilus planipennis	Bicaudal D
EOG091200WZ	TC009111	Tribolium castaneum	Bicaudal D
EOG091200WZ	LDEC002204	Leptinotarsa decemlineata	Bicaudal D
EOG091200WZ	YQE_02201	Dendroctonus ponderosae	Bicaudal D
EOG091200X0	OTAU010274	Onthophagus taurus	Putative uncharacterized protein
EOG091200X0	AGLA008940	Anoplophora glabripennis	Putative uncharacterized protein
EOG091200X0	APLA009772	Agrilus planipennis	Putative uncharacterized protein
EOG091200X0	TC006352	Tribolium castaneum	Putative uncharacterized protein
EOG091200X0	LDEC020259	Leptinotarsa decemlineata	Putative uncharacterized protein
EOG091200X0	YQE_05714	Dendroctonus ponderosae	Putative uncharacterized protein
EOG091200X3	OTAU003455	Onthophagus taurus	None
EOG091200X3	AGLA016895	Anoplophora glabripennis	None
EOG091200X3	APLA005105	Agrilus planipennis	None
EOG091200X3	TC031189	Tribolium castaneum	None
EOG091200X3	LDEC007733	Leptinotarsa decemlineata	None
EOG091200X3	YQE_10026	Dendroctonus ponderosae	None
EOG091200X4	OTAU008105	Onthophagus taurus	transferase activity
EOG091200X4	AGLA000637	Anoplophora glabripennis	transferase activity
EOG091200X4	APLA005339	Agrilus planipennis	transferase activity
EOG091200X4	TC030986	Tribolium castaneum	transferase activity
EOG091200X4	LDEC007059	Leptinotarsa decemlineata	transferase activity
EOG091200X4	YQE_08180	Dendroctonus ponderosae	transferase activity
EOG091200X5	OTAU009352	Onthophagus taurus	None
EOG091200X5	AGLA014696	Anoplophora glabripennis	None
EOG091200X5	APLA003080	Agrilus planipennis	None
EOG091200X5	TC031929	Tribolium castaneum	None
EOG091200X5	LDEC010560	Leptinotarsa decemlineata	None
EOG091200X5	YQE_10662	Dendroctonus ponderosae	None
EOG091200X8	OTAU006060	Onthophagus taurus	Ligase4-like protein
EOG091200X8	AGLA001028	Anoplophora glabripennis	Ligase4-like protein
EOG091200X8	APLA002908	Agrilus planipennis	Ligase4-like protein
EOG091200X8	TC012219	Tribolium castaneum	Ligase4-like protein
EOG091200X8	LDEC007503	Leptinotarsa decemlineata	Ligase4-like protein
EOG091200X8	YQE_12970	Dendroctonus ponderosae	Ligase4-like protein
EOG091200X9	OTAU000132	Onthophagus taurus	Pumilio
EOG091200X9	AGLA007044	Anoplophora glabripennis	Pumilio
EOG091200X9	APLA008093	Agrilus planipennis	Pumilio
EOG091200X9	TC005073	Tribolium castaneum	Pumilio
EOG091200X9	LDEC010405	Leptinotarsa decemlineata	Pumilio
EOG091200X9	YQE_10639	Dendroctonus ponderosae	Pumilio
EOG091200XA	OTAU003622	Onthophagus taurus	Putative uncharacterized protein
EOG091200XA	AGLA004249	Anoplophora glabripennis	Putative uncharacterized protein
EOG091200XA	APLA000824	Agrilus planipennis	Putative uncharacterized protein
EOG091200XA	TC003215	Tribolium castaneum	Putative uncharacterized protein
EOG091200XA	LDEC008210	Leptinotarsa decemlineata	Putative uncharacterized protein
EOG091200XA	YQE_07826	Dendroctonus ponderosae	Putative uncharacterized protein
EOG091200XC	OTAU005042	Onthophagus taurus	Putative uncharacterized protein
EOG091200XC	AGLA015045	Anoplophora glabripennis	Putative uncharacterized protein
EOG091200XC	APLA013213	Agrilus planipennis	Putative uncharacterized protein
EOG091200XC	TC009072	Tribolium castaneum	Putative uncharacterized protein
EOG091200XC	LDEC020670	Leptinotarsa decemlineata	Putative uncharacterized protein
EOG091200XC	YQE_03134	Dendroctonus ponderosae	Putative uncharacterized protein
EOG091200XE	OTAU013659	Onthophagus taurus	metal ion binding
EOG091200XE	AGLA003226	Anoplophora glabripennis	metal ion binding
EOG091200XE	APLA012274	Agrilus planipennis	metal ion binding
EOG091200XE	TC031364	Tribolium castaneum	metal ion binding
EOG091200XE	LDEC008077	Leptinotarsa decemlineata	metal ion binding
EOG091200XE	YQE_04790	Dendroctonus ponderosae	metal ion binding
EOG091200XF	OTAU011880	Onthophagus taurus	Putative uncharacterized protein

EOG091200XF	AGLA020153	Anoplophora glabripennis	Putative uncharacterized protein
EOG091200XF	APLA006850	Agrilus planipennis	Putative uncharacterized protein
EOG091200XF	TC013715	Tribolium castaneum	Putative uncharacterized protein
EOG091200XF	LDEC014501	Leptinotarsa decemlineata	Putative uncharacterized protein
EOG091200XF	YQE_06190	Dendroctonus ponderosae	Putative uncharacterized protein
EOG091200XK	OTAU015649	Onthophagus taurus	Non-lysosomal glucosylceramidase
EOG091200XK	AGLA015064	Anoplophora glabripennis	Non-lysosomal glucosylceramidase
EOG091200XK	APLA004579	Agrilus planipennis	Non-lysosomal glucosylceramidase
EOG091200XK	TC016367	Tribolium castaneum	Non-lysosomal glucosylceramidase
EOG091200XK	LDEC018925	Leptinotarsa decemlineata	Non-lysosomal glucosylceramidase
EOG091200XK	YQE_04294	Dendroctonus ponderosae	Non-lysosomal glucosylceramidase
EOG091200XN	OTAU004359	Onthophagus taurus	Putative uncharacterized protein
EOG091200XN	AGLA002372	Anoplophora glabripennis	Putative uncharacterized protein
EOG091200XN	APLA006786	Agrilus planipennis	Putative uncharacterized protein
EOG091200XN	TC010820	Tribolium castaneum	Putative uncharacterized protein
EOG091200XN	LDEC012572	Leptinotarsa decemlineata	Putative uncharacterized protein
EOG091200XN	YQE_06685	Dendroctonus ponderosae	Putative uncharacterized protein
EOG091200XO	OTAU001833	Onthophagus taurus	Putative uncharacterized protein
EOG091200XO	AGLA005414	Anoplophora glabripennis	Putative uncharacterized protein
EOG091200XO	APLA001151	Agrilus planipennis	Putative uncharacterized protein
EOG091200XO	TC005722	Tribolium castaneum	Putative uncharacterized protein
EOG091200XO	LDEC017604	Leptinotarsa decemlineata	Putative uncharacterized protein
EOG091200XO	YQE_06998	Dendroctonus ponderosae	Putative uncharacterized protein
EOG091200XQ	OTAU012276	Onthophagus taurus	Mitochondrial Rho GTPase
EOG091200XQ	AGLA011369	Anoplophora glabripennis	Mitochondrial Rho GTPase
EOG091200XQ	APLA003270	Agrilus planipennis	Mitochondrial Rho GTPase
EOG091200XQ	TC012343	Tribolium castaneum	Mitochondrial Rho GTPase
EOG091200XQ	LDEC008603	Leptinotarsa decemlineata	Mitochondrial Rho GTPase
EOG091200XQ	YQE_10079	Dendroctonus ponderosae	Mitochondrial Rho GTPase
EOG091200XV	OTAU009137	Onthophagus taurus	lyase activity
EOG091200XV	AGLA009008	Anoplophora glabripennis	lyase activity
EOG091200XV	APLA003036	Agrilus planipennis	lyase activity
EOG091200XV	TC033004	Tribolium castaneum	lyase activity
EOG091200XV	LDEC002138	Leptinotarsa decemlineata	lyase activity
EOG091200XV	YQE_08635	Dendroctonus ponderosae	lyase activity
EOG091200XX	OTAU000390	Onthophagus taurus	Putative uncharacterized protein
EOG091200XX	AGLA019743	Anoplophora glabripennis	Putative uncharacterized protein
EOG091200XX	APLA000294	Agrilus planipennis	Putative uncharacterized protein
EOG091200XX	TC000132	Tribolium castaneum	Putative uncharacterized protein
EOG091200XX	LDEC009326	Leptinotarsa decemlineata	Putative uncharacterized protein
EOG091200XX	YQE_11861	Dendroctonus ponderosae	Putative uncharacterized protein
EOG091200XY	OTAU000540	Onthophagus taurus	None
EOG091200XY	AGLA001467	Anoplophora glabripennis	None
EOG091200XY	APLA000329	Agrilus planipennis	None
EOG091200XY	TC030965	Tribolium castaneum	None
EOG091200XY	LDEC003309	Leptinotarsa decemlineata	None
EOG091200XY	YQE_01778	Dendroctonus ponderosae	None
EOG091200XZ	OTAU003390	Onthophagus taurus	Putative uncharacterized protein
EOG091200XZ	AGLA005131	Anoplophora glabripennis	Putative uncharacterized protein
EOG091200XZ	APLA002667	Agrilus planipennis	Putative uncharacterized protein
EOG091200XZ	TC012134	Tribolium castaneum	Putative uncharacterized protein
EOG091200XZ	LDEC019009	Leptinotarsa decemlineata	Putative uncharacterized protein
EOG091200XZ	YQE_12819	Dendroctonus ponderosae	Putative uncharacterized protein
EOG091200Y0	OTAU013953	Onthophagus taurus	Putative uncharacterized protein
EOG091200Y0	AGLA003546	Anoplophora glabripennis	Putative uncharacterized protein
EOG091200Y0	APLA007291	Agrilus planipennis	Putative uncharacterized protein
EOG091200Y0	TC002590	Tribolium castaneum	Putative uncharacterized protein
EOG091200Y0	LDEC016974	Leptinotarsa decemlineata	Putative uncharacterized protein
EOG091200Y0	YQE_07391	Dendroctonus ponderosae	Putative uncharacterized protein
EOG091200Y3	OTAU002083	Onthophagus taurus	Putative uncharacterized protein
EOG091200Y3	AGLA019317	Anoplophora glabripennis	Putative uncharacterized protein
EOG091200Y3	APLA001130	Agrilus planipennis	Putative uncharacterized protein
EOG091200Y3	TC012809	Tribolium castaneum	Putative uncharacterized protein
EOG091200Y3	LDEC022500	Leptinotarsa decemlineata	Putative uncharacterized protein
EOG091200Y3	YQE_04365	Dendroctonus ponderosae	Putative uncharacterized protein
EOG091200Y6	OTAU006203	Onthophagus taurus	Putative uncharacterized protein
EOG091200Y6	AGLA015595	Anoplophora glabripennis	Putative uncharacterized protein

EOG091200Y6	APLA015195	Agrilus planipennis	Putative uncharacterized protein
EOG091200Y6	TC004451	Tribolium castaneum	Putative uncharacterized protein
EOG091200Y6	LDEC019381	Leptinotarsa decemlineata	Putative uncharacterized protein
EOG091200Y6	YQE_07063	Dendroctonus ponderosae	Putative uncharacterized protein
EOG091200YB	OTAU000294	Onthophagus taurus	Putative uncharacterized protein
EOG091200YB	AGLA011922	Anoplophora glabripennis	Putative uncharacterized protein
EOG091200YB	APLA014459	Agrilus planipennis	Putative uncharacterized protein
EOG091200YB	TC012891	Tribolium castaneum	Putative uncharacterized protein
EOG091200YB	LDEC001177	Leptinotarsa decemlineata	Putative uncharacterized protein
EOG091200YB	YQE_02095	Dendroctonus ponderosae	Putative uncharacterized protein
EOG091200YG	OTAU007760	Onthophagus taurus	Ubiquitin carboxyl-terminal hydrolase
EOG091200YG	AGLA010632	Anoplophora glabripennis	Ubiquitin carboxyl-terminal hydrolase
EOG091200YG	APLA013323	Agrilus planipennis	Ubiquitin carboxyl-terminal hydrolase
EOG091200YG	TC030699	Tribolium castaneum	Ubiquitin carboxyl-terminal hydrolase
EOG091200YG	LDEC008998	Leptinotarsa decemlineata	Ubiquitin carboxyl-terminal hydrolase
EOG091200YG	YQE_12620	Dendroctonus ponderosae	Ubiquitin carboxyl-terminal hydrolase
EOG091200YH	OTAU000377	Onthophagus taurus	receptor signaling complex scaffold activity
EOG091200YH	AGLA005087	Anoplophora glabripennis	receptor signaling complex scaffold activity
EOG091200YH	APLA014058	Agrilus planipennis	receptor signaling complex scaffold activity
EOG091200YH	TC034397	Tribolium castaneum	receptor signaling complex scaffold activity
EOG091200YH	LDEC005508	Leptinotarsa decemlineata	receptor signaling complex scaffold activity
EOG091200YH	YQE_01641	Dendroctonus ponderosae	receptor signaling complex scaffold activity
EOG091200YI	OTAU009781	Onthophagus taurus	Putative uncharacterized protein
EOG091200YI	AGLA002124	Anoplophora glabripennis	Putative uncharacterized protein
EOG091200YI	APLA008128	Agrilus planipennis	Putative uncharacterized protein
EOG091200YI	TC002371	Tribolium castaneum	Putative uncharacterized protein
EOG091200YI	LDEC005440	Leptinotarsa decemlineata	Putative uncharacterized protein
EOG091200YI	YQE_09881	Dendroctonus ponderosae	Putative uncharacterized protein
EOG091200YK	OTAU001950	Onthophagus taurus	Putative uncharacterized protein
EOG091200YK	AGLA009989	Anoplophora glabripennis	Putative uncharacterized protein
EOG091200YK	APLA012302	Agrilus planipennis	Putative uncharacterized protein
EOG091200YK	TC006390	Tribolium castaneum	Putative uncharacterized protein
EOG091200YK	LDEC006308	Leptinotarsa decemlineata	Putative uncharacterized protein
EOG091200YK	YQE_05634	Dendroctonus ponderosae	Putative uncharacterized protein
EOG091200YM	OTAU016084	Onthophagus taurus	metal ion binding
EOG091200YM	AGLA012750	Anoplophora glabripennis	metal ion binding
EOG091200YM	APLA009124	Agrilus planipennis	metal ion binding
EOG091200YM	TC034819	Tribolium castaneum	metal ion binding
EOG091200YM	LDEC001860	Leptinotarsa decemlineata	metal ion binding
EOG091200YM	YQE_10139	Dendroctonus ponderosae	metal ion binding
EOG091200YN	OTAU001238	Onthophagus taurus	Guanylate cyclase
EOG091200YN	AGLA002137	Anoplophora glabripennis	Guanylate cyclase
EOG091200YN	APLA003698	Agrilus planipennis	Guanylate cyclase
EOG091200YN	TC004747	Tribolium castaneum	Guanylate cyclase
EOG091200YN	LDEC007179	Leptinotarsa decemlineata	Guanylate cyclase
EOG091200YN	YQE_09106	Dendroctonus ponderosae	Guanylate cyclase
EOG091200YP	OTAU013079	Onthophagus taurus	Putative uncharacterized protein
EOG091200YP	AGLA013735	Anoplophora glabripennis	Putative uncharacterized protein
EOG091200YP	APLA005909	Agrilus planipennis	Putative uncharacterized protein
EOG091200YP	TC015545	Tribolium castaneum	Putative uncharacterized protein
EOG091200YP	LDEC005630	Leptinotarsa decemlineata	Putative uncharacterized protein
EOG091200YP	YQE_03168	Dendroctonus ponderosae	Putative uncharacterized protein
EOG091200YR	OTAU001822	Onthophagus taurus	Putative uncharacterized protein
EOG091200YR	AGLA004990	Anoplophora glabripennis	Putative uncharacterized protein
EOG091200YR	APLA012075	Agrilus planipennis	Putative uncharacterized protein
EOG091200YR	TC006624	Tribolium castaneum	Putative uncharacterized protein
EOG091200YR	LDEC002995	Leptinotarsa decemlineata	Putative uncharacterized protein
EOG091200YR	YQE_07018	Dendroctonus ponderosae	Putative uncharacterized protein
EOG091200YS	OTAU005397	Onthophagus taurus	Putative uncharacterized protein
EOG091200YS	AGLA000414	Anoplophora glabripennis	Putative uncharacterized protein
EOG091200YS	APLA010002	Agrilus planipennis	Putative uncharacterized protein
EOG091200YS	TC001965	Tribolium castaneum	Putative uncharacterized protein
EOG091200YS	LDEC004224	Leptinotarsa decemlineata	Putative uncharacterized protein
EOG091200YS	YQE_09048	Dendroctonus ponderosae	Putative uncharacterized protein
EOG091200YT	OTAU003353	Onthophagus taurus	Putative uncharacterized protein
EOG091200YT	AGLA004578	Anoplophora glabripennis	Putative uncharacterized protein
EOG091200YT	APLA010448	Agrilus planipennis	Putative uncharacterized protein

EOG091200YT	TC014207	Tribolium castaneum	Putative uncharacterized protein
EOG091200YT	LDEC017482	Leptinotarsa decemlineata	Putative uncharacterized protein
EOG091200YT	YQE_09248	Dendroctonus ponderosae	Putative uncharacterized protein
EOG091200YU	OTAU015313	Onthophagus taurus	Putative uncharacterized protein
EOG091200YU	AGLA003872	Anoplophora glabripennis	Putative uncharacterized protein
EOG091200YU	APLA005890	Agrilus planipennis	Putative uncharacterized protein
EOG091200YU	TC015410	Tribolium castaneum	Putative uncharacterized protein
EOG091200YU	LDEC001821	Leptinotarsa decemlineata	Putative uncharacterized protein
EOG091200YU	YQE_08327	Dendroctonus ponderosae	Putative uncharacterized protein
EOG091200YW	OTAU008745	Onthophagus taurus	Putative uncharacterized protein
EOG091200YW	AGLA013825	Anoplophora glabripennis	Putative uncharacterized protein
EOG091200YW	APLA000618	Agrilus planipennis	Putative uncharacterized protein
EOG091200YW	TC015333	Tribolium castaneum	Putative uncharacterized protein
EOG091200YW	LDEC001807	Leptinotarsa decemlineata	Putative uncharacterized protein
EOG091200YW	YQE_01831	Dendroctonus ponderosae	Putative uncharacterized protein
EOG091200YX	OTAU005184	Onthophagus taurus	Putative uncharacterized protein
EOG091200YX	AGLA009162	Anoplophora glabripennis	Putative uncharacterized protein
EOG091200YX	APLA005445	Agrilus planipennis	Putative uncharacterized protein
EOG091200YX	TC003152	Tribolium castaneum	Putative uncharacterized protein
EOG091200YX	LDEC010260	Leptinotarsa decemlineata	Putative uncharacterized protein
EOG091200YX	YQE_07409	Dendroctonus ponderosae	Putative uncharacterized protein
EOG091200Z0	OTAU001009	Onthophagus taurus	Putative uncharacterized protein
EOG091200Z0	AGLA001322	Anoplophora glabripennis	Putative uncharacterized protein
EOG091200Z0	APLA013473	Agrilus planipennis	Putative uncharacterized protein
EOG091200Z0	TC000487	Tribolium castaneum	Putative uncharacterized protein
EOG091200Z0	LDEC012375	Leptinotarsa decemlineata	Putative uncharacterized protein
EOG091200Z0	YQE_11622	Dendroctonus ponderosae	Putative uncharacterized protein
EOG091200Z1	OTAU000369	Onthophagus taurus	Putative uncharacterized protein
EOG091200Z1	AGLA005109	Anoplophora glabripennis	Putative uncharacterized protein
EOG091200Z1	APLA003316	Agrilus planipennis	Putative uncharacterized protein
EOG091200Z1	TC011759	Tribolium castaneum	Putative uncharacterized protein
EOG091200Z1	LDEC003894	Leptinotarsa decemlineata	Putative uncharacterized protein
EOG091200Z1	YQE_11256	Dendroctonus ponderosae	Putative uncharacterized protein
EOG091200Z2	OTAU000145	Onthophagus taurus	Putative uncharacterized protein
EOG091200Z2	AGLA019789	Anoplophora glabripennis	Putative uncharacterized protein
EOG091200Z2	APLA008292	Agrilus planipennis	Putative uncharacterized protein
EOG091200Z2	TC013884	Tribolium castaneum	Putative uncharacterized protein
EOG091200Z2	LDEC005971	Leptinotarsa decemlineata	Putative uncharacterized protein
EOG091200Z2	YQE_06189	Dendroctonus ponderosae	Putative uncharacterized protein
EOG091200Z5	OTAU002236	Onthophagus taurus	Phosphatase
EOG091200Z5	AGLA020776	Anoplophora glabripennis	Phosphatase
EOG091200Z5	APLA010415	Agrilus planipennis	Phosphatase
EOG091200Z5	TC012508	Tribolium castaneum	Phosphatase
EOG091200Z5	LDEC000525	Leptinotarsa decemlineata	Phosphatase
EOG091200Z5	YQE_07974	Dendroctonus ponderosae	Phosphatase
EOG091200Z9	OTAU005529	Onthophagus taurus	Putative uncharacterized protein
EOG091200Z9	AGLA005617	Anoplophora glabripennis	Putative uncharacterized protein
EOG091200Z9	APLA007407	Agrilus planipennis	Putative uncharacterized protein
EOG091200Z9	TC012081	Tribolium castaneum	Putative uncharacterized protein
EOG091200Z9	LDEC019041	Leptinotarsa decemlineata	Putative uncharacterized protein
EOG091200Z9	YQE_01976	Dendroctonus ponderosae	Putative uncharacterized protein
EOG091200ZA	OTAU006840	Onthophagus taurus	Putative uncharacterized protein
EOG091200ZA	AGLA021063	Anoplophora glabripennis	Putative uncharacterized protein
EOG091200ZA	APLA005154	Agrilus planipennis	Putative uncharacterized protein
EOG091200ZA	TC013382	Tribolium castaneum	Putative uncharacterized protein
EOG091200ZA	LDEC002644	Leptinotarsa decemlineata	Putative uncharacterized protein
EOG091200ZA	YQE_04863	Dendroctonus ponderosae	Putative uncharacterized protein
EOG091200ZB	OTAU001871	Onthophagus taurus	insulin receptor binding
EOG091200ZB	AGLA014381	Anoplophora glabripennis	insulin receptor binding
EOG091200ZB	APLA000906	Agrilus planipennis	insulin receptor binding
EOG091200ZB	TC034081	Tribolium castaneum	insulin receptor binding
EOG091200ZB	LDEC011755	Leptinotarsa decemlineata	insulin receptor binding
EOG091200ZB	YQE_01891	Dendroctonus ponderosae	insulin receptor binding
EOG091200ZF	OTAU006925	Onthophagus taurus	Putative uncharacterized protein
EOG091200ZF	AGLA002081	Anoplophora glabripennis	Putative uncharacterized protein
EOG091200ZF	APLA013802	Agrilus planipennis	Putative uncharacterized protein
EOG091200ZF	TC002340	Tribolium castaneum	Putative uncharacterized protein

EOG091200ZF	LDEC009191	Leptinotarsa decemlineata	Putative uncharacterized protein
EOG091200ZF	YQE_06426	Dendroctonus ponderosae	Putative uncharacterized protein
EOG091200ZH	OTAU000041	Onthophagus taurus	U-shaped
EOG091200ZH	AGLA011933	Anoplophora glabripennis	U-shaped
EOG091200ZH	APLA002447	Agrilus planipennis	U-shaped
EOG091200ZH	TC013689	Tribolium castaneum	U-shaped
EOG091200ZH	LDEC016471	Leptinotarsa decemlineata	U-shaped
EOG091200ZH	YQE_03987	Dendroctonus ponderosae	U-shaped
EOG091200ZI	OTAU016491	Onthophagus taurus	Putative uncharacterized protein
EOG091200ZI	AGLA001561	Anoplophora glabripennis	Putative uncharacterized protein
EOG091200ZI	APLA008251	Agrilus planipennis	Putative uncharacterized protein
EOG091200ZI	TC011362	Tribolium castaneum	Putative uncharacterized protein
EOG091200ZI	LDEC009062	Leptinotarsa decemlineata	Putative uncharacterized protein
EOG091200ZI	YQE_02570	Dendroctonus ponderosae	Putative uncharacterized protein
EOG091200ZK	OTAU010321	Onthophagus taurus	Putative uncharacterized protein
EOG091200ZK	AGLA007283	Anoplophora glabripennis	Putative uncharacterized protein
EOG091200ZK	APLA003790	Agrilus planipennis	Putative uncharacterized protein
EOG091200ZK	TC008773	Tribolium castaneum	Putative uncharacterized protein
EOG091200ZK	LDEC019684	Leptinotarsa decemlineata	Putative uncharacterized protein
EOG091200ZK	YQE_07495	Dendroctonus ponderosae	Putative uncharacterized protein
EOG091200ZL	OTAU008503	Onthophagus taurus	Putative uncharacterized protein
EOG091200ZL	AGLA006696	Anoplophora glabripennis	Putative uncharacterized protein
EOG091200ZL	APLA012134	Agrilus planipennis	Putative uncharacterized protein
EOG091200ZL	TC013064	Tribolium castaneum	Putative uncharacterized protein
EOG091200ZL	LDEC014008	Leptinotarsa decemlineata	Putative uncharacterized protein
EOG091200ZL	YQE_03215	Dendroctonus ponderosae	Putative uncharacterized protein
EOG091200ZM	OTAU007809	Onthophagus taurus	Putative uncharacterized protein
EOG091200ZM	AGLA002888	Anoplophora glabripennis	Putative uncharacterized protein
EOG091200ZM	APLA012251	Agrilus planipennis	Putative uncharacterized protein
EOG091200ZM	TC007481	Tribolium castaneum	Putative uncharacterized protein
EOG091200ZM	LDEC000736	Leptinotarsa decemlineata	Putative uncharacterized protein
EOG091200ZM	YQE_12438	Dendroctonus ponderosae	Putative uncharacterized protein
EOG091200ZO	OTAU013063	Onthophagus taurus	Putative uncharacterized protein
EOG091200ZO	AGLA001344	Anoplophora glabripennis	Putative uncharacterized protein
EOG091200ZO	APLA007826	Agrilus planipennis	Putative uncharacterized protein
EOG091200ZO	TC000531	Tribolium castaneum	Putative uncharacterized protein
EOG091200ZO	LDEC003407	Leptinotarsa decemlineata	Putative uncharacterized protein
EOG091200ZO	YQE_10699	Dendroctonus ponderosae	Putative uncharacterized protein
EOG091200ZS	OTAU003922	Onthophagus taurus	Putative uncharacterized protein
EOG091200ZS	AGLA004396	Anoplophora glabripennis	Putative uncharacterized protein
EOG091200ZS	APLA003265	Agrilus planipennis	Putative uncharacterized protein
EOG091200ZS	TC011751	Tribolium castaneum	Putative uncharacterized protein
EOG091200ZS	LDEC014884	Leptinotarsa decemlineata	Putative uncharacterized protein
EOG091200ZS	YQE_12989	Dendroctonus ponderosae	Putative uncharacterized protein
EOG091200ZU	OTAU005962	Onthophagus taurus	Putative uncharacterized protein
EOG091200ZU	AGLA018724	Anoplophora glabripennis	Putative uncharacterized protein
EOG091200ZU	APLA007535	Agrilus planipennis	Putative uncharacterized protein
EOG091200ZU	TC009632	Tribolium castaneum	Putative uncharacterized protein
EOG091200ZU	LDEC004468	Leptinotarsa decemlineata	Putative uncharacterized protein
EOG091200ZU	YQE_13011	Dendroctonus ponderosae	Putative uncharacterized protein
EOG091200ZV	OTAU009530	Onthophagus taurus	Pyrexia
EOG091200ZV	AGLA001117	Anoplophora glabripennis	Pyrexia
EOG091200ZV	APLA004759	Agrilus planipennis	Pyrexia
EOG091200ZV	TC009731	Tribolium castaneum	Pyrexia
EOG091200ZV	LDEC005832	Leptinotarsa decemlineata	Pyrexia
EOG091200ZV	YQE_11341	Dendroctonus ponderosae	Pyrexia
EOG091200ZX	OTAU005014	Onthophagus taurus	Putative uncharacterized protein
EOG091200ZX	AGLA000783	Anoplophora glabripennis	Putative uncharacterized protein
EOG091200ZX	APLA002188	Agrilus planipennis	Putative uncharacterized protein
EOG091200ZX	TC007444	Tribolium castaneum	Putative uncharacterized protein
EOG091200ZX	LDEC000710	Leptinotarsa decemlineata	Putative uncharacterized protein
EOG091200ZX	YQE_04484	Dendroctonus ponderosae	Putative uncharacterized protein
EOG091200ZY	OTAU010150	Onthophagus taurus	Putative uncharacterized protein
EOG091200ZY	AGLA007185	Anoplophora glabripennis	Putative uncharacterized protein
EOG091200ZY	APLA010202	Agrilus planipennis	Putative uncharacterized protein
EOG091200ZY	TC009218	Tribolium castaneum	Putative uncharacterized protein
EOG091200ZY	LDEC014888	Leptinotarsa decemlineata	Putative uncharacterized protein

EOG091200ZY	YQE_11801	Dendroctonus ponderosae	Putative uncharacterized protein
EOG09120101	OTAU014055	Onthophagus taurus	Putative uncharacterized protein
EOG09120101	AGLA013313	Anoplophora glabripennis	Putative uncharacterized protein
EOG09120101	APLA005940	Agrilus planipennis	Putative uncharacterized protein
EOG09120101	TC015323	Tribolium castaneum	Putative uncharacterized protein
EOG09120101	LDEC009009	Leptinotarsa decemlineata	Putative uncharacterized protein
EOG09120101	YQE_02117	Dendroctonus ponderosae	Putative uncharacterized protein
EOG09120102	OTAU005638	Onthophagus taurus	Putative uncharacterized protein
EOG09120102	AGLA006459	Anoplophora glabripennis	Putative uncharacterized protein
EOG09120102	APLA001569	Agrilus planipennis	Putative uncharacterized protein
EOG09120102	TC008808	Tribolium castaneum	Putative uncharacterized protein
EOG09120102	LDEC006162	Leptinotarsa decemlineata	Putative uncharacterized protein
EOG09120102	YQE_11271	Dendroctonus ponderosae	Putative uncharacterized protein
EOG09120103	OTAU010635	Onthophagus taurus	Putative uncharacterized protein
EOG09120103	AGLA009270	Anoplophora glabripennis	Putative uncharacterized protein
EOG09120103	APLA003799	Agrilus planipennis	Putative uncharacterized protein
EOG09120103	TC003689	Tribolium castaneum	Putative uncharacterized protein
EOG09120103	LDEC009248	Leptinotarsa decemlineata	Putative uncharacterized protein
EOG09120103	YQE_02756	Dendroctonus ponderosae	Putative uncharacterized protein
EOG09120106	OTAU003185	Onthophagus taurus	Putative uncharacterized protein
EOG09120106	AGLA007365	Anoplophora glabripennis	Putative uncharacterized protein
EOG09120106	APLA002853	Agrilus planipennis	Putative uncharacterized protein
EOG09120106	TC013495	Tribolium castaneum	Putative uncharacterized protein
EOG09120106	LDEC015162	Leptinotarsa decemlineata	Putative uncharacterized protein
EOG09120106	YQE_07170	Dendroctonus ponderosae	Putative uncharacterized protein
EOG09120108	OTAU008229	Onthophagus taurus	Putative uncharacterized protein
EOG09120108	AGLA001895	Anoplophora glabripennis	Putative uncharacterized protein
EOG09120108	APLA001367	Agrilus planipennis	Putative uncharacterized protein
EOG09120108	TC000807	Tribolium castaneum	Putative uncharacterized protein
EOG09120108	LDEC006113	Leptinotarsa decemlineata	Putative uncharacterized protein
EOG09120108	YQE_09488	Dendroctonus ponderosae	Putative uncharacterized protein
EOG0912010A	OTAU000563	Onthophagus taurus	Putative uncharacterized protein
EOG0912010A	AGLA017233	Anoplophora glabripennis	Putative uncharacterized protein
EOG0912010A	APLA003244	Agrilus planipennis	Putative uncharacterized protein
EOG0912010A	TC012164	Tribolium castaneum	Putative uncharacterized protein
EOG0912010A	LDEC003863	Leptinotarsa decemlineata	Putative uncharacterized protein
EOG0912010A	YQE_07612	Dendroctonus ponderosae	Putative uncharacterized protein
EOG0912010B	OTAU013816	Onthophagus taurus	None
EOG0912010B	AGLA002933	Anoplophora glabripennis	None
EOG0912010B	APLA014069	Agrilus planipennis	None
EOG0912010B	TC032855	Tribolium castaneum	None
EOG0912010B	LDEC004369	Leptinotarsa decemlineata	None
EOG0912010B	YQE_04252	Dendroctonus ponderosae	None
EOG0912010D	OTAU005413	Onthophagus taurus	Putative uncharacterized protein
EOG0912010D	AGLA016425	Anoplophora glabripennis	Putative uncharacterized protein
EOG0912010D	APLA003680	Agrilus planipennis	Putative uncharacterized protein
EOG0912010D	TC004581	Tribolium castaneum	Putative uncharacterized protein
EOG0912010D	LDEC018157	Leptinotarsa decemlineata	Putative uncharacterized protein
EOG0912010D	YQE_09455	Dendroctonus ponderosae	Putative uncharacterized protein
EOG0912010F	OTAU016088	Onthophagus taurus	Putative uncharacterized protein
EOG0912010F	AGLA017502	Anoplophora glabripennis	Putative uncharacterized protein
EOG0912010F	APLA005306	Agrilus planipennis	Putative uncharacterized protein
EOG0912010F	TC011557	Tribolium castaneum	Putative uncharacterized protein
EOG0912010F	LDEC001659	Leptinotarsa decemlineata	Putative uncharacterized protein
EOG0912010F	YQE_02716	Dendroctonus ponderosae	Putative uncharacterized protein
EOG0912010H	OTAU008500	Onthophagus taurus	binding
EOG0912010H	AGLA011499	Anoplophora glabripennis	binding
EOG0912010H	APLA014454	Agrilus planipennis	binding
EOG0912010H	TC034612	Tribolium castaneum	binding
EOG0912010H	LDEC017781	Leptinotarsa decemlineata	binding
EOG0912010H	YQE_02089	Dendroctonus ponderosae	binding
EOG0912010L	OTAU009776	Onthophagus taurus	Minichromosome maintenance complex component 10
EOG0912010L	AGLA019728	Anoplophora glabripennis	Minichromosome maintenance complex component 10
EOG0912010L	APLA014006	Agrilus planipennis	Minichromosome maintenance complex component 10

EOG0912010L	TC011513	Tribolium castaneum	Minichromosome maintenance complex component 10
EOG0912010L	LDEC005910	Leptinotarsa decemlineata	Minichromosome maintenance complex component 10
EOG0912010L	YQE_11156	Dendroctonus ponderosae	Minichromosome maintenance complex component 10
EOG0912010M	OTAU008389	Onthophagus taurus	Putative uncharacterized protein
EOG0912010M	AGLA003554	Anoplophora glabripennis	Putative uncharacterized protein
EOG0912010M	APLA004108	Agrilus planipennis	Putative uncharacterized protein
EOG0912010M	TC001563	Tribolium castaneum	Putative uncharacterized protein
EOG0912010M	LDEC016958	Leptinotarsa decemlineata	Putative uncharacterized protein
EOG0912010M	YQE_10235	Dendroctonus ponderosae	Putative uncharacterized protein
EOG0912010N	OTAU009085	Onthophagus taurus	ATP binding
EOG0912010N	AGLA020541	Anoplophora glabripennis	ATP binding
EOG0912010N	APLA010350	Agrilus planipennis	ATP binding
EOG0912010N	TC034109	Tribolium castaneum	ATP binding
EOG0912010N	LDEC020170	Leptinotarsa decemlineata	ATP binding
EOG0912010N	YQE_05533	Dendroctonus ponderosae	ATP binding
EOG0912010P	OTAU001954	Onthophagus taurus	Putative uncharacterized protein
EOG0912010P	AGLA001238	Anoplophora glabripennis	Putative uncharacterized protein
EOG0912010P	APLA000401	Agrilus planipennis	Putative uncharacterized protein
EOG0912010P	TC006218	Tribolium castaneum	Putative uncharacterized protein
EOG0912010P	LDEC011060	Leptinotarsa decemlineata	Putative uncharacterized protein
EOG0912010P	YQE_12015	Dendroctonus ponderosae	Putative uncharacterized protein
EOG0912010Q	OTAU016065	Onthophagus taurus	Putative uncharacterized protein
EOG0912010Q	AGLA009859	Anoplophora glabripennis	Putative uncharacterized protein
EOG0912010Q	APLA008082	Agrilus planipennis	Putative uncharacterized protein
EOG0912010Q	TC013108	Tribolium castaneum	Putative uncharacterized protein
EOG0912010Q	LDEC006275	Leptinotarsa decemlineata	Putative uncharacterized protein
EOG0912010Q	YQE_04785	Dendroctonus ponderosae	Putative uncharacterized protein
EOG0912010R	OTAU004679	Onthophagus taurus	Putative uncharacterized protein
EOG0912010R	AGLA017206	Anoplophora glabripennis	Putative uncharacterized protein
EOG0912010R	APLA011804	Agrilus planipennis	Putative uncharacterized protein
EOG0912010R	TC011972	Tribolium castaneum	Putative uncharacterized protein
EOG0912010R	LDEC010805	Leptinotarsa decemlineata	Putative uncharacterized protein
EOG0912010R	YQE_09430	Dendroctonus ponderosae	Putative uncharacterized protein
EOG0912010T	OTAU008303	Onthophagus taurus	choline dehydrogenase activity
EOG0912010T	AGLA010314	Anoplophora glabripennis	choline dehydrogenase activity
EOG0912010T	APLA000638	Agrilus planipennis	choline dehydrogenase activity
EOG0912010T	TC033300	Tribolium castaneum	choline dehydrogenase activity
EOG0912010T	LDEC017154	Leptinotarsa decemlineata	choline dehydrogenase activity
EOG0912010T	YQE_10795	Dendroctonus ponderosae	choline dehydrogenase activity
EOG0912010U	OTAU002800	Onthophagus taurus	metal ion binding
EOG0912010U	AGLA002384	Anoplophora glabripennis	metal ion binding
EOG0912010U	APLA003535	Agrilus planipennis	metal ion binding
EOG0912010U	TC030879	Tribolium castaneum	metal ion binding
EOG0912010U	LDEC011955	Leptinotarsa decemlineata	metal ion binding
EOG0912010U	YQE_03275	Dendroctonus ponderosae	metal ion binding
EOG0912010V	OTAU007631	Onthophagus taurus	Putative uncharacterized protein
EOG0912010V	AGLA011845	Anoplophora glabripennis	Putative uncharacterized protein
EOG0912010V	APLA008828	Agrilus planipennis	Putative uncharacterized protein
EOG0912010V	TC008997	Tribolium castaneum	Putative uncharacterized protein
EOG0912010V	LDEC004813	Leptinotarsa decemlineata	Putative uncharacterized protein
EOG0912010V	YQE_02191	Dendroctonus ponderosae	Putative uncharacterized protein
EOG09120110	OTAU016979	Onthophagus taurus	isomerase activity
EOG09120110	AGLA003948	Anoplophora glabripennis	isomerase activity
EOG09120110	APLA009444	Agrilus planipennis	isomerase activity
EOG09120110	TC030909	Tribolium castaneum	isomerase activity
EOG09120110	LDEC012255	Leptinotarsa decemlineata	isomerase activity
EOG09120110	YQE_09869	Dendroctonus ponderosae	isomerase activity
EOG09120112	OTAU000142	Onthophagus taurus	Putative uncharacterized protein
EOG09120112	AGLA014701	Anoplophora glabripennis	Putative uncharacterized protein
EOG09120112	APLA012877	Agrilus planipennis	Putative uncharacterized protein
EOG09120112	TC004103	Tribolium castaneum	Putative uncharacterized protein
EOG09120112	LDEC010554	Leptinotarsa decemlineata	Putative uncharacterized protein
EOG09120112	YQE_01968	Dendroctonus ponderosae	Putative uncharacterized protein
EOG09120115	OTAU005710	Onthophagus taurus	Ionotropic receptor 8a

EOG09120115	AGLA006196	Anoplophora glabripennis	Ionotropic receptor 8a
EOG09120115	APLA001507	Agrilus planipennis	Ionotropic receptor 8a
EOG09120115	TC003572	Tribolium castaneum	Ionotropic receptor 8a
EOG09120115	LDEC016856	Leptinotarsa decemlineata	Ionotropic receptor 8a
EOG09120115	YQE_12359	Dendroctonus ponderosae	Ionotropic receptor 8a
EOG09120116	OTAU009507	Onthophagus taurus	Putative uncharacterized protein
EOG09120116	AGLA013501	Anoplophora glabripennis	Putative uncharacterized protein
EOG09120116	APLA009185	Agrilus planipennis	Putative uncharacterized protein
EOG09120116	TC003474	Tribolium castaneum	Putative uncharacterized protein
EOG09120116	LDEC016392	Leptinotarsa decemlineata	Putative uncharacterized protein
EOG09120116	YQE_02772	Dendroctonus ponderosae	Putative uncharacterized protein
EOG09120117	OTAU008210	Onthophagus taurus	Putative uncharacterized protein
EOG09120117	AGLA014071	Anoplophora glabripennis	Putative uncharacterized protein
EOG09120117	APLA012190	Agrilus planipennis	Putative uncharacterized protein
EOG09120117	TC000461	Tribolium castaneum	Putative uncharacterized protein
EOG09120117	LDEC000782	Leptinotarsa decemlineata	Putative uncharacterized protein
EOG09120117	YQE_02540	Dendroctonus ponderosae	Putative uncharacterized protein
EOG09120119	OTAU005599	Onthophagus taurus	Putative uncharacterized protein
EOG09120119	AGLA006310	Anoplophora glabripennis	Putative uncharacterized protein
EOG09120119	APLA004117	Agrilus planipennis	Putative uncharacterized protein
EOG09120119	TC010007	Tribolium castaneum	Putative uncharacterized protein
EOG09120119	LDEC002083	Leptinotarsa decemlineata	Putative uncharacterized protein
EOG09120119	YQE_10364	Dendroctonus ponderosae	Putative uncharacterized protein
EOG0912011A	OTAU014038	Onthophagus taurus	nucleoside-triphosphatase activity
EOG0912011A	AGLA003159	Anoplophora glabripennis	nucleoside-triphosphatase activity
EOG0912011A	APLA013048	Agrilus planipennis	nucleoside-triphosphatase activity
EOG0912011A	TC034861	Tribolium castaneum	nucleoside-triphosphatase activity
EOG0912011A	LDEC007616	Leptinotarsa decemlineata	nucleoside-triphosphatase activity
EOG0912011A	YQE_10052	Dendroctonus ponderosae	nucleoside-triphosphatase activity
EOG0912011C	OTAU002871	Onthophagus taurus	Putative uncharacterized protein
EOG0912011C	AGLA001135	Anoplophora glabripennis	Putative uncharacterized protein
EOG0912011C	APLA014984	Agrilus planipennis	Putative uncharacterized protein
EOG0912011C	TC004585	Tribolium castaneum	Putative uncharacterized protein
EOG0912011C	LDEC007789	Leptinotarsa decemlineata	Putative uncharacterized protein
EOG0912011C	YQE_09162	Dendroctonus ponderosae	Putative uncharacterized protein
EOG0912011D	OTAU005690	Onthophagus taurus	None
EOG0912011D	AGLA003462	Anoplophora glabripennis	None
EOG0912011D	APLA010536	Agrilus planipennis	None
EOG0912011D	TC033588	Tribolium castaneum	None
EOG0912011D	LDEC001322	Leptinotarsa decemlineata	None
EOG0912011D	YQE_11796	Dendroctonus ponderosae	None
EOG0912011E	OTAU001655	Onthophagus taurus	Putative uncharacterized protein
EOG0912011E	AGLA009880	Anoplophora glabripennis	Putative uncharacterized protein
EOG0912011E	APLA000966	Agrilus planipennis	Putative uncharacterized protein
EOG0912011E	TC005472	Tribolium castaneum	Putative uncharacterized protein
EOG0912011E	LDEC021547	Leptinotarsa decemlineata	Putative uncharacterized protein
EOG0912011E	YQE_05701	Dendroctonus ponderosae	Putative uncharacterized protein
EOG0912011I	OTAU007510	Onthophagus taurus	Putative uncharacterized protein
EOG0912011I	AGLA009962	Anoplophora glabripennis	Putative uncharacterized protein
EOG0912011I	APLA011501	Agrilus planipennis	Putative uncharacterized protein
EOG0912011I	TC015701	Tribolium castaneum	Putative uncharacterized protein
EOG0912011I	LDEC004745	Leptinotarsa decemlineata	Putative uncharacterized protein
EOG0912011I	YQE_10892	Dendroctonus ponderosae	Putative uncharacterized protein
EOG0912011J	OTAU006914	Onthophagus taurus	binding
EOG0912011J	AGLA021720	Anoplophora glabripennis	binding
EOG0912011J	APLA000302	Agrilus planipennis	binding
EOG0912011J	TC032583	Tribolium castaneum	binding
EOG0912011J	LDEC002697	Leptinotarsa decemlineata	binding
EOG0912011J	YQE_09972	Dendroctonus ponderosae	binding
EOG0912011L	OTAU006042	Onthophagus taurus	Putative uncharacterized protein
EOG0912011L	AGLA005159	Anoplophora glabripennis	Putative uncharacterized protein
EOG0912011L	APLA005669	Agrilus planipennis	Putative uncharacterized protein
EOG0912011L	TC008486	Tribolium castaneum	Putative uncharacterized protein
EOG0912011L	LDEC017052	Leptinotarsa decemlineata	Putative uncharacterized protein
EOG0912011L	YQE_12525	Dendroctonus ponderosae	Putative uncharacterized protein
EOG0912011M	OTAU000150	Onthophagus taurus	Putative uncharacterized protein
EOG0912011M	AGLA012788	Anoplophora glabripennis	Putative uncharacterized protein

EOG0912011M	APLA008534	Agrilus planipennis	Putative uncharacterized protein
EOG0912011M	TC014455	Tribolium castaneum	Putative uncharacterized protein
EOG0912011M	LDEC016469	Leptinotarsa decemlineata	Putative uncharacterized protein
EOG0912011M	YQE_02682	Dendroctonus ponderosae	Putative uncharacterized protein
EOG0912011Q	OTAU008375	Onthophagus taurus	Putative uncharacterized protein
EOG0912011Q	AGLA001632	Anoplophora glabripennis	Putative uncharacterized protein
EOG0912011Q	APLA002961	Agrilus planipennis	Putative uncharacterized protein
EOG0912011Q	TC000315	Tribolium castaneum	Putative uncharacterized protein
EOG0912011Q	LDEC016888	Leptinotarsa decemlineata	Putative uncharacterized protein
EOG0912011Q	YQE_06838	Dendroctonus ponderosae	Putative uncharacterized protein
EOG0912011R	OTAU009823	Onthophagus taurus	Putative uncharacterized protein
EOG0912011R	AGLA009911	Anoplophora glabripennis	Putative uncharacterized protein
EOG0912011R	APLA004523	Agrilus planipennis	Putative uncharacterized protein
EOG0912011R	TC005576	Tribolium castaneum	Putative uncharacterized protein
EOG0912011R	LDEC018967	Leptinotarsa decemlineata	Putative uncharacterized protein
EOG0912011R	YQE_06932	Dendroctonus ponderosae	Putative uncharacterized protein
EOG0912011T	OTAU001400	Onthophagus taurus	Putative uncharacterized protein
EOG0912011T	AGLA008326	Anoplophora glabripennis	Putative uncharacterized protein
EOG0912011T	APLA011839	Agrilus planipennis	Putative uncharacterized protein
EOG0912011T	TC001752	Tribolium castaneum	Putative uncharacterized protein
EOG0912011T	LDEC012714	Leptinotarsa decemlineata	Putative uncharacterized protein
EOG0912011T	YQE_06712	Dendroctonus ponderosae	Putative uncharacterized protein
EOG0912011V	OTAU001233	Onthophagus taurus	Putative uncharacterized protein
EOG0912011V	AGLA007630	Anoplophora glabripennis	Putative uncharacterized protein
EOG0912011V	APLA003549	Agrilus planipennis	Putative uncharacterized protein
EOG0912011V	TC001631	Tribolium castaneum	Putative uncharacterized protein
EOG0912011V	LDEC003513	Leptinotarsa decemlineata	Putative uncharacterized protein
EOG0912011V	YQE_09183	Dendroctonus ponderosae	Putative uncharacterized protein
EOG0912011W	OTAU008828	Onthophagus taurus	Aminopeptidase N-like protein
EOG0912011W	AGLA002946	Anoplophora glabripennis	Aminopeptidase N-like protein
EOG0912011W	APLA001279	Agrilus planipennis	Aminopeptidase N-like protein
EOG0912011W	TC004830	Tribolium castaneum	Aminopeptidase N-like protein
EOG0912011W	LDEC018555	Leptinotarsa decemlineata	Aminopeptidase N-like protein
EOG0912011W	YQE_10931	Dendroctonus ponderosae	Aminopeptidase N-like protein
EOG0912011Z	OTAU000856	Onthophagus taurus	Putative uncharacterized protein
EOG0912011Z	AGLA000929	Anoplophora glabripennis	Putative uncharacterized protein
EOG0912011Z	APLA009416	Agrilus planipennis	Putative uncharacterized protein
EOG0912011Z	TC004511	Tribolium castaneum	Putative uncharacterized protein
EOG0912011Z	LDEC005397	Leptinotarsa decemlineata	Putative uncharacterized protein
EOG0912011Z	YQE_02538	Dendroctonus ponderosae	Putative uncharacterized protein
EOG09120120	OTAU008666	Onthophagus taurus	Putative uncharacterized protein
EOG09120120	AGLA004997	Anoplophora glabripennis	Putative uncharacterized protein
EOG09120120	APLA001451	Agrilus planipennis	Putative uncharacterized protein
EOG09120120	TC005910	Tribolium castaneum	Putative uncharacterized protein
EOG09120120	LDEC002988	Leptinotarsa decemlineata	Putative uncharacterized protein
EOG09120120	YQE_07022	Dendroctonus ponderosae	Putative uncharacterized protein
EOG09120121	OTAU015817	Onthophagus taurus	Putative uncharacterized protein
EOG09120121	AGLA003003	Anoplophora glabripennis	Putative uncharacterized protein
EOG09120121	APLA011523	Agrilus planipennis	Putative uncharacterized protein
EOG09120121	TC000188	Tribolium castaneum	Putative uncharacterized protein
EOG09120121	LDEC009663	Leptinotarsa decemlineata	Putative uncharacterized protein
EOG09120121	YQE_01683	Dendroctonus ponderosae	Putative uncharacterized protein
EOG09120124	OTAU004103	Onthophagus taurus	Putative uncharacterized protein
EOG09120124	AGLA002809	Anoplophora glabripennis	Putative uncharacterized protein
EOG09120124	APLA006026	Agrilus planipennis	Putative uncharacterized protein
EOG09120124	TC009779	Tribolium castaneum	Putative uncharacterized protein
EOG09120124	LDEC004057	Leptinotarsa decemlineata	Putative uncharacterized protein
EOG09120124	YQE_05265	Dendroctonus ponderosae	Putative uncharacterized protein
EOG09120125	OTAU006031	Onthophagus taurus	Putative uncharacterized protein
EOG09120125	AGLA019155	Anoplophora glabripennis	Putative uncharacterized protein
EOG09120125	APLA002214	Agrilus planipennis	Putative uncharacterized protein
EOG09120125	TC008408	Tribolium castaneum	Putative uncharacterized protein
EOG09120125	LDEC006485	Leptinotarsa decemlineata	Putative uncharacterized protein
EOG09120125	YQE_04747	Dendroctonus ponderosae	Putative uncharacterized protein
EOG09120128	OTAU014061	Onthophagus taurus	Putative uncharacterized protein
EOG09120128	AGLA008309	Anoplophora glabripennis	Putative uncharacterized protein
EOG09120128	APLA002436	Agrilus planipennis	Putative uncharacterized protein

EOG09120128	TC013085	Tribolium castaneum	Putative uncharacterized protein
EOG09120128	LDEC012508	Leptinotarsa decemlineata	Putative uncharacterized protein
EOG09120128	YQE_13075	Dendroctonus ponderosae	Putative uncharacterized protein
EOG09120129	OTAU014701	Onthophagus taurus	Putative uncharacterized protein
EOG09120129	AGLA001371	Anoplophora glabripennis	Putative uncharacterized protein
EOG09120129	APLA005030	Agrilus planipennis	Putative uncharacterized protein
EOG09120129	TC000216	Tribolium castaneum	Putative uncharacterized protein
EOG09120129	LDEC007292	Leptinotarsa decemlineata	Putative uncharacterized protein
EOG09120129	YQE_01584	Dendroctonus ponderosae	Putative uncharacterized protein
EOG0912012A	OTAU010778	Onthophagus taurus	Putative uncharacterized protein
EOG0912012A	AGLA005973	Anoplophora glabripennis	Putative uncharacterized protein
EOG0912012A	APLA007500	Agrilus planipennis	Putative uncharacterized protein
EOG0912012A	TC006499	Tribolium castaneum	Putative uncharacterized protein
EOG0912012A	LDEC015395	Leptinotarsa decemlineata	Putative uncharacterized protein
EOG0912012A	YQE_06911	Dendroctonus ponderosae	Putative uncharacterized protein
EOG0912012B	OTAU004379	Onthophagus taurus	Putative uncharacterized protein
EOG0912012B	AGLA000270	Anoplophora glabripennis	Putative uncharacterized protein
EOG0912012B	APLA005581	Agrilus planipennis	Putative uncharacterized protein
EOG0912012B	TC004611	Tribolium castaneum	Putative uncharacterized protein
EOG0912012B	LDEC001081	Leptinotarsa decemlineata	Putative uncharacterized protein
EOG0912012B	YQE_09088	Dendroctonus ponderosae	Putative uncharacterized protein
EOG0912012D	OTAU007547	Onthophagus taurus	None
EOG0912012D	AGLA005595	Anoplophora glabripennis	None
EOG0912012D	APLA008059	Agrilus planipennis	None
EOG0912012D	TC033127	Tribolium castaneum	None
EOG0912012D	LDEC018991	Leptinotarsa decemlineata	None
EOG0912012D	YQE_02697	Dendroctonus ponderosae	None
EOG0912012E	OTAU015397	Onthophagus taurus	Putative uncharacterized protein
EOG0912012E	AGLA008091	Anoplophora glabripennis	Putative uncharacterized protein
EOG0912012E	APLA002943	Agrilus planipennis	Putative uncharacterized protein
EOG0912012E	TC001001	Tribolium castaneum	Putative uncharacterized protein
EOG0912012E	LDEC006603	Leptinotarsa decemlineata	Putative uncharacterized protein
EOG0912012E	YQE_06493	Dendroctonus ponderosae	Putative uncharacterized protein
EOG0912012F	OTAU008028	Onthophagus taurus	transferase activity
EOG0912012F	AGLA005970	Anoplophora glabripennis	transferase activity
EOG0912012F	APLA004483	Agrilus planipennis	transferase activity
EOG0912012F	TC031284	Tribolium castaneum	transferase activity
EOG0912012F	LDEC015390	Leptinotarsa decemlineata	transferase activity
EOG0912012F	YQE_06584	Dendroctonus ponderosae	transferase activity
EOG0912012J	OTAU012884	Onthophagus taurus	Putative uncharacterized protein
EOG0912012J	AGLA008092	Anoplophora glabripennis	Putative uncharacterized protein
EOG0912012J	APLA002941	Agrilus planipennis	Putative uncharacterized protein
EOG0912012J	TC000571	Tribolium castaneum	Putative uncharacterized protein
EOG0912012J	LDEC006600	Leptinotarsa decemlineata	Putative uncharacterized protein
EOG0912012J	YQE_08493	Dendroctonus ponderosae	Putative uncharacterized protein
EOG0912012K	OTAU007892	Onthophagus taurus	Putative uncharacterized protein
EOG0912012K	AGLA006725	Anoplophora glabripennis	Putative uncharacterized protein
EOG0912012K	APLA002673	Agrilus planipennis	Putative uncharacterized protein
EOG0912012K	TC013034	Tribolium castaneum	Putative uncharacterized protein
EOG0912012K	LDEC019553	Leptinotarsa decemlineata	Putative uncharacterized protein
EOG0912012K	YQE_06242	Dendroctonus ponderosae	Putative uncharacterized protein
EOG0912012L	OTAU016221	Onthophagus taurus	Ras opposite
EOG0912012L	AGLA011366	Anoplophora glabripennis	Ras opposite
EOG0912012L	APLA003019	Agrilus planipennis	Ras opposite
EOG0912012L	TC011120	Tribolium castaneum	Ras opposite
EOG0912012L	LDEC018675	Leptinotarsa decemlineata	Ras opposite
EOG0912012L	YQE_09572	Dendroctonus ponderosae	Ras opposite
EOG0912012N	OTAU014375	Onthophagus taurus	Putative uncharacterized protein
EOG0912012N	AGLA014964	Anoplophora glabripennis	Putative uncharacterized protein
EOG0912012N	APLA004086	Agrilus planipennis	Putative uncharacterized protein
EOG0912012N	TC002980	Tribolium castaneum	Putative uncharacterized protein
EOG0912012N	LDEC018283	Leptinotarsa decemlineata	Putative uncharacterized protein
EOG0912012N	YQE_09773	Dendroctonus ponderosae	Putative uncharacterized protein
EOG0912012O	OTAU003466	Onthophagus taurus	Putative uncharacterized protein
EOG0912012O	AGLA007985	Anoplophora glabripennis	Putative uncharacterized protein
EOG0912012O	APLA001636	Agrilus planipennis	Putative uncharacterized protein
EOG0912012O	TC008992	Tribolium castaneum	Putative uncharacterized protein

EOG0912012O	LDEC006156	Leptinotarsa decemlineata	Putative uncharacterized protein
EOG0912012O	YQE_03517	Dendroctonus ponderosae	Putative uncharacterized protein
EOG0912012R	OTAU003624	Onthophagus taurus	Putative uncharacterized protein
EOG0912012R	AGLA004255	Anoplophora glabripennis	Putative uncharacterized protein
EOG0912012R	APLA000827	Agrilus planipennis	Putative uncharacterized protein
EOG0912012R	TC002365	Tribolium castaneum	Putative uncharacterized protein
EOG0912012R	LDEC008206	Leptinotarsa decemlineata	Putative uncharacterized protein
EOG0912012R	YQE_07818	Dendroctonus ponderosae	Putative uncharacterized protein
EOG0912012S	OTAU005852	Onthophagus taurus	Putative uncharacterized protein
EOG0912012S	AGLA004447	Anoplophora glabripennis	Putative uncharacterized protein
EOG0912012S	APLA001503	Agrilus planipennis	Putative uncharacterized protein
EOG0912012S	TC002556	Tribolium castaneum	Putative uncharacterized protein
EOG0912012S	LDEC002700	Leptinotarsa decemlineata	Putative uncharacterized protein
EOG0912012S	YQE_09955	Dendroctonus ponderosae	Putative uncharacterized protein
EOG0912012V	OTAU005505	Onthophagus taurus	Putative uncharacterized protein
EOG0912012V	AGLA000794	Anoplophora glabripennis	Putative uncharacterized protein
EOG0912012V	APLA009058	Agrilus planipennis	Putative uncharacterized protein
EOG0912012V	TC007530	Tribolium castaneum	Putative uncharacterized protein
EOG0912012V	LDEC018608	Leptinotarsa decemlineata	Putative uncharacterized protein
EOG0912012V	YQE_04185	Dendroctonus ponderosae	Putative uncharacterized protein
EOG0912012Z	OTAU009832	Onthophagus taurus	Wishful thinking
EOG0912012Z	AGLA012661	Anoplophora glabripennis	Wishful thinking
EOG0912012Z	APLA008804	Agrilus planipennis	Wishful thinking
EOG0912012Z	TC009314	Tribolium castaneum	Wishful thinking
EOG0912012Z	LDEC011009	Leptinotarsa decemlineata	Wishful thinking
EOG0912012Z	YQE_05384	Dendroctonus ponderosae	Wishful thinking
EOG09120130	OTAU004066	Onthophagus taurus	Frizzled 2
EOG09120130	AGLA007531	Anoplophora glabripennis	Frizzled 2
EOG09120130	APLA002849	Agrilus planipennis	Frizzled 2
EOG09120130	TC003407	Tribolium castaneum	Frizzled 2
EOG09120130	LDEC003036	Leptinotarsa decemlineata	Frizzled 2
EOG09120130	YQE_08262	Dendroctonus ponderosae	Frizzled 2
EOG09120131	OTAU007404	Onthophagus taurus	Putative uncharacterized protein
EOG09120131	AGLA014058	Anoplophora glabripennis	Putative uncharacterized protein
EOG09120131	APLA000832	Agrilus planipennis	Putative uncharacterized protein
EOG09120131	TC013885	Tribolium castaneum	Putative uncharacterized protein
EOG09120131	LDEC005973	Leptinotarsa decemlineata	Putative uncharacterized protein
EOG09120131	YQE_06187	Dendroctonus ponderosae	Putative uncharacterized protein
EOG09120134	OTAU014359	Onthophagus taurus	Putative uncharacterized protein
EOG09120134	AGLA010415	Anoplophora glabripennis	Putative uncharacterized protein
EOG09120134	APLA002232	Agrilus planipennis	Putative uncharacterized protein
EOG09120134	TC008066	Tribolium castaneum	Putative uncharacterized protein
EOG09120134	LDEC000194	Leptinotarsa decemlineata	Putative uncharacterized protein
EOG09120134	YQE_08662	Dendroctonus ponderosae	Putative uncharacterized protein
EOG09120135	OTAU002818	Onthophagus taurus	Putative uncharacterized protein
EOG09120135	AGLA002176	Anoplophora glabripennis	Putative uncharacterized protein
EOG09120135	APLA006778	Agrilus planipennis	Putative uncharacterized protein
EOG09120135	TC014180	Tribolium castaneum	Putative uncharacterized protein
EOG09120135	LDEC001543	Leptinotarsa decemlineata	Putative uncharacterized protein
EOG09120135	YQE_09050	Dendroctonus ponderosae	Putative uncharacterized protein
EOG09120136	OTAU005370	Onthophagus taurus	Putative uncharacterized protein
EOG09120136	AGLA008927	Anoplophora glabripennis	Putative uncharacterized protein
EOG09120136	APLA000780	Agrilus planipennis	Putative uncharacterized protein
EOG09120136	TC002784	Tribolium castaneum	Putative uncharacterized protein
EOG09120136	LDEC001129	Leptinotarsa decemlineata	Putative uncharacterized protein
EOG09120136	YQE_09851	Dendroctonus ponderosae	Putative uncharacterized protein
EOG09120137	OTAU006488	Onthophagus taurus	3'-5' exonuclease activity
EOG09120137	AGLA003528	Anoplophora glabripennis	3'-5' exonuclease activity
EOG09120137	APLA013247	Agrilus planipennis	3'-5' exonuclease activity
EOG09120137	TC032570	Tribolium castaneum	3'-5' exonuclease activity
EOG09120137	LDEC012675	Leptinotarsa decemlineata	3'-5' exonuclease activity
EOG09120137	YQE_10262	Dendroctonus ponderosae	3'-5' exonuclease activity
EOG09120138	OTAU000760	Onthophagus taurus	calcium-dependent cysteine-type endopeptidase activity
EOG09120138	AGLA000589	Anoplophora glabripennis	calcium-dependent cysteine-type endopeptidase activity

EOG09120138	APLA012829	Agrilus planipennis	calcium-dependent cysteine-type endopeptidase activity
EOG09120138	TC032612	Tribolium castaneum	calcium-dependent cysteine-type endopeptidase activity
EOG09120138	LDEC006992	Leptinotarsa decemlineata	calcium-dependent cysteine-type endopeptidase activity
EOG09120138	YQE_12302	Dendroctonus ponderosae	calcium-dependent cysteine-type endopeptidase activity
EOG09120139	OTAU006645	Onthophagus taurus	Putative uncharacterized protein
EOG09120139	AGLA006893	Anoplophora glabripennis	Putative uncharacterized protein
EOG09120139	APLA003353	Agrilus planipennis	Putative uncharacterized protein
EOG09120139	TC000140	Tribolium castaneum	Putative uncharacterized protein
EOG09120139	LDEC004547	Leptinotarsa decemlineata	Putative uncharacterized protein
EOG09120139	YQE_08664	Dendroctonus ponderosae	Putative uncharacterized protein
EOG0912013A	OTAU007090	Onthophagus taurus	Putative uncharacterized protein
EOG0912013A	AGLA005337	Anoplophora glabripennis	Putative uncharacterized protein
EOG0912013A	APLA010716	Agrilus planipennis	Putative uncharacterized protein
EOG0912013A	TC014101	Tribolium castaneum	Putative uncharacterized protein
EOG0912013A	LDEC002760	Leptinotarsa decemlineata	Putative uncharacterized protein
EOG0912013A	YQE_08655	Dendroctonus ponderosae	Putative uncharacterized protein
EOG0912013B	OTAU008650	Onthophagus taurus	Putative uncharacterized protein
EOG0912013B	AGLA003705	Anoplophora glabripennis	Putative uncharacterized protein
EOG0912013B	APLA004193	Agrilus planipennis	Putative uncharacterized protein
EOG0912013B	TC007466	Tribolium castaneum	Putative uncharacterized protein
EOG0912013B	LDEC001393	Leptinotarsa decemlineata	Putative uncharacterized protein
EOG0912013B	YQE_09011	Dendroctonus ponderosae	Putative uncharacterized protein
EOG0912013C	OTAU009041	Onthophagus taurus	None
EOG0912013C	AGLA003212	Anoplophora glabripennis	None
EOG0912013C	APLA009415	Agrilus planipennis	None
EOG0912013C	TC031006	Tribolium castaneum	None
EOG0912013C	LDEC007389	Leptinotarsa decemlineata	None
EOG0912013C	YQE_10420	Dendroctonus ponderosae	None
EOG0912013D	OTAU007391	Onthophagus taurus	Putative uncharacterized protein
EOG0912013D	AGLA011521	Anoplophora glabripennis	Putative uncharacterized protein
EOG0912013D	APLA005123	Agrilus planipennis	Putative uncharacterized protein
EOG0912013D	TC013542	Tribolium castaneum	Putative uncharacterized protein
EOG0912013D	LDEC011416	Leptinotarsa decemlineata	Putative uncharacterized protein
EOG0912013D	YQE_04827	Dendroctonus ponderosae	Putative uncharacterized protein
EOG0912013E	OTAU014054	Onthophagus taurus	Putative uncharacterized protein
EOG0912013E	AGLA015574	Anoplophora glabripennis	Putative uncharacterized protein
EOG0912013E	APLA011970	Agrilus planipennis	Putative uncharacterized protein
EOG0912013E	TC015971	Tribolium castaneum	Putative uncharacterized protein
EOG0912013E	LDEC006935	Leptinotarsa decemlineata	Putative uncharacterized protein
EOG0912013E	YQE_10870	Dendroctonus ponderosae	Putative uncharacterized protein
EOG0912013G	OTAU012912	Onthophagus taurus	Putative uncharacterized protein
EOG0912013G	AGLA001912	Anoplophora glabripennis	Putative uncharacterized protein
EOG0912013G	APLA007593	Agrilus planipennis	Putative uncharacterized protein
EOG0912013G	TC001311	Tribolium castaneum	Putative uncharacterized protein
EOG0912013G	LDEC000843	Leptinotarsa decemlineata	Putative uncharacterized protein
EOG0912013G	YQE_09526	Dendroctonus ponderosae	Putative uncharacterized protein
EOG0912013H	OTAU012943	Onthophagus taurus	Putative uncharacterized protein
EOG0912013H	AGLA000214	Anoplophora glabripennis	Putative uncharacterized protein
EOG0912013H	APLA012408	Agrilus planipennis	Putative uncharacterized protein
EOG0912013H	TC004679	Tribolium castaneum	Putative uncharacterized protein
EOG0912013H	LDEC001071	Leptinotarsa decemlineata	Putative uncharacterized protein
EOG0912013H	YQE_03639	Dendroctonus ponderosae	Putative uncharacterized protein
EOG0912013J	OTAU002947	Onthophagus taurus	Putative uncharacterized protein
EOG0912013J	AGLA003456	Anoplophora glabripennis	Putative uncharacterized protein
EOG0912013J	APLA001670	Agrilus planipennis	Putative uncharacterized protein
EOG0912013J	TC009202	Tribolium castaneum	Putative uncharacterized protein
EOG0912013J	LDEC020309	Leptinotarsa decemlineata	Putative uncharacterized protein
EOG0912013J	YQE_13030	Dendroctonus ponderosae	Putative uncharacterized protein
EOG0912013L	OTAU008722	Onthophagus taurus	Ubiquitin carboxyl-terminal hydrolase
EOG0912013L	AGLA014941	Anoplophora glabripennis	Ubiquitin carboxyl-terminal hydrolase
EOG0912013L	APLA003768	Agrilus planipennis	Ubiquitin carboxyl-terminal hydrolase
EOG0912013L	TC002978	Tribolium castaneum	Ubiquitin carboxyl-terminal hydrolase
EOG0912013L	LDEC002050	Leptinotarsa decemlineata	Ubiquitin carboxyl-terminal hydrolase

EOG0912013L	YQE_11110	Dendroctonus ponderosae	Ubiquitin carboxyl-terminal hydrolase
EOG0912013M	OTAU007727	Onthophagus taurus	Putative uncharacterized protein
EOG0912013M	AGLA001757	Anoplophora glabripennis	Putative uncharacterized protein
EOG0912013M	APLA008113	Agrilus planipennis	Putative uncharacterized protein
EOG0912013M	TC014258	Tribolium castaneum	Putative uncharacterized protein
EOG0912013M	LDEC006192	Leptinotarsa decemlineata	Putative uncharacterized protein
EOG0912013M	YQE_03981	Dendroctonus ponderosae	Putative uncharacterized protein
EOG0912013O	OTAU001897	Onthophagus taurus	Putative uncharacterized protein
EOG0912013O	AGLA009096	Anoplophora glabripennis	Putative uncharacterized protein
EOG0912013O	APLA004460	Agrilus planipennis	Putative uncharacterized protein
EOG0912013O	TC006676	Tribolium castaneum	Putative uncharacterized protein
EOG0912013O	LDEC020225	Leptinotarsa decemlineata	Putative uncharacterized protein
EOG0912013O	YQE_07008	Dendroctonus ponderosae	Putative uncharacterized protein
EOG0912013Q	OTAU001406	Onthophagus taurus	Putative uncharacterized protein
EOG0912013Q	AGLA002206	Anoplophora glabripennis	Putative uncharacterized protein
EOG0912013Q	APLA003495	Agrilus planipennis	Putative uncharacterized protein
EOG0912013Q	TC004717	Tribolium castaneum	Putative uncharacterized protein
EOG0912013Q	LDEC011948	Leptinotarsa decemlineata	Putative uncharacterized protein
EOG0912013Q	YQE_09055	Dendroctonus ponderosae	Putative uncharacterized protein
EOG0912013R	OTAU016462	Onthophagus taurus	Putative uncharacterized protein
EOG0912013R	AGLA007402	Anoplophora glabripennis	Putative uncharacterized protein
EOG0912013R	APLA001186	Agrilus planipennis	Putative uncharacterized protein
EOG0912013R	TC005445	Tribolium castaneum	Putative uncharacterized protein
EOG0912013R	LDEC018142	Leptinotarsa decemlineata	Putative uncharacterized protein
EOG0912013R	YQE_05591	Dendroctonus ponderosae	Putative uncharacterized protein
EOG0912013S	OTAU003445	Onthophagus taurus	DNA binding
EOG0912013S	AGLA001965	Anoplophora glabripennis	DNA binding
EOG0912013S	APLA014161	Agrilus planipennis	DNA binding
EOG0912013S	TC031236	Tribolium castaneum	DNA binding
EOG0912013S	LDEC011767	Leptinotarsa decemlineata	DNA binding
EOG0912013S	YQE_08020	Dendroctonus ponderosae	DNA binding
EOG0912013V	OTAU006759	Onthophagus taurus	Putative uncharacterized protein
EOG0912013V	AGLA018155	Anoplophora glabripennis	Putative uncharacterized protein
EOG0912013V	APLA015412	Agrilus planipennis	Putative uncharacterized protein
EOG0912013V	TC010675	Tribolium castaneum	Putative uncharacterized protein
EOG0912013V	LDEC017340	Leptinotarsa decemlineata	Putative uncharacterized protein
EOG0912013V	YQE_04328	Dendroctonus ponderosae	Putative uncharacterized protein
EOG0912013W	OTAU012297	Onthophagus taurus	Putative uncharacterized protein
EOG0912013W	AGLA003843	Anoplophora glabripennis	Putative uncharacterized protein
EOG0912013W	APLA003481	Agrilus planipennis	Putative uncharacterized protein
EOG0912013W	TC010011	Tribolium castaneum	Putative uncharacterized protein
EOG0912013W	LDEC010662	Leptinotarsa decemlineata	Putative uncharacterized protein
EOG0912013W	YQE_13052	Dendroctonus ponderosae	Putative uncharacterized protein
EOG0912013X	OTAU000288	Onthophagus taurus	Putative uncharacterized protein
EOG0912013X	AGLA008457	Anoplophora glabripennis	Putative uncharacterized protein
EOG0912013X	APLA006220	Agrilus planipennis	Putative uncharacterized protein
EOG0912013X	TC013799	Tribolium castaneum	Putative uncharacterized protein
EOG0912013X	LDEC020093	Leptinotarsa decemlineata	Putative uncharacterized protein
EOG0912013X	YQE_04893	Dendroctonus ponderosae	Putative uncharacterized protein
EOG0912013Y	OTAU002741	Onthophagus taurus	Putative uncharacterized protein
EOG0912013Y	AGLA002360	Anoplophora glabripennis	Putative uncharacterized protein
EOG0912013Y	APLA003487	Agrilus planipennis	Putative uncharacterized protein
EOG0912013Y	TC004789	Tribolium castaneum	Putative uncharacterized protein
EOG0912013Y	LDEC001118	Leptinotarsa decemlineata	Putative uncharacterized protein
EOG0912013Y	YQE_09074	Dendroctonus ponderosae	Putative uncharacterized protein
EOG09120142	OTAU001917	Onthophagus taurus	Putative uncharacterized protein
EOG09120142	AGLA019506	Anoplophora glabripennis	Putative uncharacterized protein
EOG09120142	APLA005819	Agrilus planipennis	Putative uncharacterized protein
EOG09120142	TC005817	Tribolium castaneum	Putative uncharacterized protein
EOG09120142	LDEC011707	Leptinotarsa decemlineata	Putative uncharacterized protein
EOG09120142	YQE_05649	Dendroctonus ponderosae	Putative uncharacterized protein
EOG09120143	OTAU008833	Onthophagus taurus	Putative uncharacterized protein
EOG09120143	AGLA004174	Anoplophora glabripennis	Putative uncharacterized protein
EOG09120143	APLA004987	Agrilus planipennis	Putative uncharacterized protein
EOG09120143	TC000642	Tribolium castaneum	Putative uncharacterized protein
EOG09120143	LDEC006130	Leptinotarsa decemlineata	Putative uncharacterized protein
EOG09120143	YQE_09365	Dendroctonus ponderosae	Putative uncharacterized protein

EOG09120147	OTAU003366	Onthophagus taurus	Putative uncharacterized protein
EOG09120147	AGLA008372	Anoplophora glabripennis	Putative uncharacterized protein
EOG09120147	APLA001051	Agrilus planipennis	Putative uncharacterized protein
EOG09120147	TC011611	Tribolium castaneum	Putative uncharacterized protein
EOG09120147	LDEC019547	Leptinotarsa decemlineata	Putative uncharacterized protein
EOG09120147	YQE_12843	Dendroctonus ponderosae	Putative uncharacterized protein
EOG0912014A	OTAU013343	Onthophagus taurus	Pebbled
EOG0912014A	AGLA012486	Anoplophora glabripennis	Pebbled
EOG0912014A	APLA001552	Agrilus planipennis	Pebbled
EOG0912014A	TC009561	Tribolium castaneum	Pebbled
EOG0912014A	LDEC020625	Leptinotarsa decemlineata	Pebbled
EOG0912014A	YQE_06035	Dendroctonus ponderosae	Pebbled
EOG0912014B	OTAU006700	Onthophagus taurus	Putative uncharacterized protein
EOG0912014B	AGLA015459	Anoplophora glabripennis	Putative uncharacterized protein
EOG0912014B	APLA004976	Agrilus planipennis	Putative uncharacterized protein
EOG0912014B	TC004839	Tribolium castaneum	Putative uncharacterized protein
EOG0912014B	LDEC003573	Leptinotarsa decemlineata	Putative uncharacterized protein
EOG0912014B	YQE_05475	Dendroctonus ponderosae	Putative uncharacterized protein
EOG0912014D	OTAU005687	Onthophagus taurus	Putative uncharacterized protein
EOG0912014D	AGLA003467	Anoplophora glabripennis	Putative uncharacterized protein
EOG0912014D	APLA010540	Agrilus planipennis	Putative uncharacterized protein
EOG0912014D	TC009861	Tribolium castaneum	Putative uncharacterized protein
EOG0912014D	LDEC001337	Leptinotarsa decemlineata	Putative uncharacterized protein
EOG0912014D	YQE_00067	Dendroctonus ponderosae	Putative uncharacterized protein
EOG0912014E	OTAU000140	Onthophagus taurus	Putative uncharacterized protein
EOG0912014E	AGLA010974	Anoplophora glabripennis	Putative uncharacterized protein
EOG0912014E	APLA008289	Agrilus planipennis	Putative uncharacterized protein
EOG0912014E	TC013939	Tribolium castaneum	Putative uncharacterized protein
EOG0912014E	LDEC008911	Leptinotarsa decemlineata	Putative uncharacterized protein
EOG0912014E	YQE_07176	Dendroctonus ponderosae	Putative uncharacterized protein
EOG0912014F	OTAU013364	Onthophagus taurus	Putative uncharacterized protein
EOG0912014F	AGLA015071	Anoplophora glabripennis	Putative uncharacterized protein
EOG0912014F	APLA002559	Agrilus planipennis	Putative uncharacterized protein
EOG0912014F	TC015198	Tribolium castaneum	Putative uncharacterized protein
EOG0912014F	LDEC005615	Leptinotarsa decemlineata	Putative uncharacterized protein
EOG0912014F	YQE_08324	Dendroctonus ponderosae	Putative uncharacterized protein
EOG0912014G	OTAU001567	Onthophagus taurus	Smoothened
EOG0912014G	AGLA009541	Anoplophora glabripennis	Smoothened
EOG0912014G	APLA004452	Agrilus planipennis	Smoothened
EOG0912014G	TC005545	Tribolium castaneum	Smoothened
EOG0912014G	LDEC002966	Leptinotarsa decemlineata	Smoothened
EOG0912014G	YQE_12680	Dendroctonus ponderosae	Smoothened
EOG0912014H	OTAU007355	Onthophagus taurus	Putative uncharacterized protein
EOG0912014H	AGLA011901	Anoplophora glabripennis	Putative uncharacterized protein
EOG0912014H	APLA006265	Agrilus planipennis	Putative uncharacterized protein
EOG0912014H	TC000182	Tribolium castaneum	Putative uncharacterized protein
EOG0912014H	LDEC020326	Leptinotarsa decemlineata	Putative uncharacterized protein
EOG0912014H	YQE_05517	Dendroctonus ponderosae	Putative uncharacterized protein
EOG0912014I	OTAU005983	Onthophagus taurus	Putative uncharacterized protein
EOG0912014I	AGLA009167	Anoplophora glabripennis	Putative uncharacterized protein
EOG0912014I	APLA002053	Agrilus planipennis	Putative uncharacterized protein
EOG0912014I	TC011547	Tribolium castaneum	Putative uncharacterized protein
EOG0912014I	LDEC010803	Leptinotarsa decemlineata	Putative uncharacterized protein
EOG0912014I	YQE_09814	Dendroctonus ponderosae	Putative uncharacterized protein
EOG0912014K	OTAU003265	Onthophagus taurus	Putative uncharacterized protein
EOG0912014K	AGLA015635	Anoplophora glabripennis	Putative uncharacterized protein
EOG0912014K	APLA014620	Agrilus planipennis	Putative uncharacterized protein
EOG0912014K	TC008956	Tribolium castaneum	Putative uncharacterized protein
EOG0912014K	LDEC010688	Leptinotarsa decemlineata	Putative uncharacterized protein
EOG0912014K	YQE_13050	Dendroctonus ponderosae	Putative uncharacterized protein
EOG0912014L	OTAU012314	Onthophagus taurus	None
EOG0912014L	AGLA006685	Anoplophora glabripennis	None
EOG0912014L	APLA001917	Agrilus planipennis	None
EOG0912014L	TC032158	Tribolium castaneum	None
EOG0912014L	LDEC019878	Leptinotarsa decemlineata	None
EOG0912014L	YQE_08495	Dendroctonus ponderosae	None
EOG0912014M	OTAU005848	Onthophagus taurus	ionotropic glutamate receptor activity

EOG0912014M	AGLA004626	Anoplophora glabripennis	ionotropic glutamate receptor activity
EOG0912014M	APLA000095	Agrilus planipennis	ionotropic glutamate receptor activity
EOG0912014M	TC032553	Tribolium castaneum	ionotropic glutamate receptor activity
EOG0912014M	LDEC004053	Leptinotarsa decemlineata	ionotropic glutamate receptor activity
EOG0912014M	YQE_10229	Dendroctonus ponderosae	ionotropic glutamate receptor activity
EOG0912014N	OTAU004555	Onthophagus taurus	Putative uncharacterized protein
EOG0912014N	AGLA008287	Anoplophora glabripennis	Putative uncharacterized protein
EOG0912014N	APLA012587	Agrilus planipennis	Putative uncharacterized protein
EOG0912014N	TC009472	Tribolium castaneum	Putative uncharacterized protein
EOG0912014N	LDEC005212	Leptinotarsa decemlineata	Putative uncharacterized protein
EOG0912014N	YQE_04290	Dendroctonus ponderosae	Putative uncharacterized protein
EOG0912014S	OTAU015465	Onthophagus taurus	Putative uncharacterized protein
EOG0912014S	AGLA008103	Anoplophora glabripennis	Putative uncharacterized protein
EOG0912014S	APLA007706	Agrilus planipennis	Putative uncharacterized protein
EOG0912014S	TC000609	Tribolium castaneum	Putative uncharacterized protein
EOG0912014S	LDEC007068	Leptinotarsa decemlineata	Putative uncharacterized protein
EOG0912014S	YQE_08485	Dendroctonus ponderosae	Putative uncharacterized protein
EOG0912014T	OTAU000089	Onthophagus taurus	phospholipid binding
EOG0912014T	AGLA005289	Anoplophora glabripennis	phospholipid binding
EOG0912014T	APLA012899	Agrilus planipennis	phospholipid binding
EOG0912014T	TC033268	Tribolium castaneum	phospholipid binding
EOG0912014T	LDEC015814	Leptinotarsa decemlineata	phospholipid binding
EOG0912014T	YQE_07247	Dendroctonus ponderosae	phospholipid binding
EOG0912014U	OTAU011148	Onthophagus taurus	Putative uncharacterized protein
EOG0912014U	AGLA015874	Anoplophora glabripennis	Putative uncharacterized protein
EOG0912014U	APLA007987	Agrilus planipennis	Putative uncharacterized protein
EOG0912014U	TC013507	Tribolium castaneum	Putative uncharacterized protein
EOG0912014U	LDEC003102	Leptinotarsa decemlineata	Putative uncharacterized protein
EOG0912014U	YQE_11107	Dendroctonus ponderosae	Putative uncharacterized protein
EOG0912014V	OTAU003339	Onthophagus taurus	Domeless
EOG0912014V	AGLA004565	Anoplophora glabripennis	Domeless
EOG0912014V	APLA010642	Agrilus planipennis	Domeless
EOG0912014V	TC001874	Tribolium castaneum	Domeless
EOG0912014V	LDEC001534	Leptinotarsa decemlineata	Domeless
EOG0912014V	YQE_03312	Dendroctonus ponderosae	Domeless
EOG0912014W	OTAU001983	Onthophagus taurus	Putative uncharacterized protein
EOG0912014W	AGLA010949	Anoplophora glabripennis	Putative uncharacterized protein
EOG0912014W	APLA004545	Agrilus planipennis	Putative uncharacterized protein
EOG0912014W	TC005347	Tribolium castaneum	Putative uncharacterized protein
EOG0912014W	LDEC016299	Leptinotarsa decemlineata	Putative uncharacterized protein
EOG0912014W	YQE_09445	Dendroctonus ponderosae	Putative uncharacterized protein
EOG0912014Y	OTAU001257	Onthophagus taurus	nucleic acid binding
EOG0912014Y	AGLA002153	Anoplophora glabripennis	nucleic acid binding
EOG0912014Y	APLA001533	Agrilus planipennis	nucleic acid binding
EOG0912014Y	TC031468	Tribolium castaneum	nucleic acid binding
EOG0912014Y	LDEC022492	Leptinotarsa decemlineata	nucleic acid binding
EOG0912014Y	YQE_03263	Dendroctonus ponderosae	nucleic acid binding
EOG0912014Z	OTAU008033	Onthophagus taurus	Axin
EOG0912014Z	AGLA007306	Anoplophora glabripennis	Axin
EOG0912014Z	APLA001168	Agrilus planipennis	Axin
EOG0912014Z	TC006314	Tribolium castaneum	Axin
EOG0912014Z	LDEC011697	Leptinotarsa decemlineata	Axin
EOG0912014Z	YQE_07501	Dendroctonus ponderosae	Axin
EOG09120151	OTAU012620	Onthophagus taurus	Putative uncharacterized protein
EOG09120151	AGLA008391	Anoplophora glabripennis	Putative uncharacterized protein
EOG09120151	APLA001745	Agrilus planipennis	Putative uncharacterized protein
EOG09120151	TC008686	Tribolium castaneum	Putative uncharacterized protein
EOG09120151	LDEC013176	Leptinotarsa decemlineata	Putative uncharacterized protein
EOG09120151	YQE_07980	Dendroctonus ponderosae	Putative uncharacterized protein
EOG09120154	OTAU004213	Onthophagus taurus	microtubule binding
EOG09120154	AGLA009280	Anoplophora glabripennis	microtubule binding
EOG09120154	APLA008486	Agrilus planipennis	microtubule binding
EOG09120154	TC034689	Tribolium castaneum	microtubule binding
EOG09120154	LDEC009233	Leptinotarsa decemlineata	microtubule binding
EOG09120154	YQE_04259	Dendroctonus ponderosae	microtubule binding
EOG09120155	OTAU004920	Onthophagus taurus	Rho guanyl-nucleotide exchange factor activity
EOG09120155	AGLA001339	Anoplophora glabripennis	Rho guanyl-nucleotide exchange factor activity

EOG09120155	APLA004402	Agrilus planipennis	Rho guanyl-nucleotide exchange factor activity
EOG09120155	TC030997	Tribolium castaneum	Rho guanyl-nucleotide exchange factor activity
EOG09120155	LDEC003395	Leptinotarsa decemlineata	Rho guanyl-nucleotide exchange factor activity
EOG09120155	YQE_11640	Dendroctonus ponderosae	Rho guanyl-nucleotide exchange factor activity
EOG09120156	OTAU013358	Onthophagus taurus	Putative uncharacterized protein
EOG09120156	AGLA004921	Anoplophora glabripennis	Putative uncharacterized protein
EOG09120156	APLA014877	Agrilus planipennis	Putative uncharacterized protein
EOG09120156	TC006102	Tribolium castaneum	Putative uncharacterized protein
EOG09120156	LDEC010371	Leptinotarsa decemlineata	Putative uncharacterized protein
EOG09120156	YQE_11755	Dendroctonus ponderosae	Putative uncharacterized protein
EOG09120159	OTAU000650	Onthophagus taurus	Putative uncharacterized protein
EOG09120159	AGLA011056	Anoplophora glabripennis	Putative uncharacterized protein
EOG09120159	APLA002342	Agrilus planipennis	Putative uncharacterized protein
EOG09120159	TC012387	Tribolium castaneum	Putative uncharacterized protein
EOG09120159	LDEC011876	Leptinotarsa decemlineata	Putative uncharacterized protein
EOG09120159	YQE_11970	Dendroctonus ponderosae	Putative uncharacterized protein
EOG0912015C	OTAU009257	Onthophagus taurus	Putative uncharacterized protein
EOG0912015C	AGLA008629	Anoplophora glabripennis	Putative uncharacterized protein
EOG0912015C	APLA000903	Agrilus planipennis	Putative uncharacterized protein
EOG0912015C	TC015477	Tribolium castaneum	Putative uncharacterized protein
EOG0912015C	LDEC003029	Leptinotarsa decemlineata	Putative uncharacterized protein
EOG0912015C	YQE_01917	Dendroctonus ponderosae	Putative uncharacterized protein
EOG0912015D	OTAU008251	Onthophagus taurus	Putative uncharacterized protein
EOG0912015D	AGLA017219	Anoplophora glabripennis	Putative uncharacterized protein
EOG0912015D	APLA008550	Agrilus planipennis	Putative uncharacterized protein
EOG0912015D	TC003481	Tribolium castaneum	Putative uncharacterized protein
EOG0912015D	LDEC002033	Leptinotarsa decemlineata	Putative uncharacterized protein
EOG0912015D	YQE_10200	Dendroctonus ponderosae	Putative uncharacterized protein
EOG0912015E	OTAU004863	Onthophagus taurus	Rad21
EOG0912015E	AGLA000043	Anoplophora glabripennis	Rad21
EOG0912015E	APLA004381	Agrilus planipennis	Rad21
EOG0912015E	TC000968	Tribolium castaneum	Rad21
EOG0912015E	LDEC012647	Leptinotarsa decemlineata	Rad21
EOG0912015E	YQE_01674	Dendroctonus ponderosae	Rad21
EOG0912015F	OTAU013510	Onthophagus taurus	Putative uncharacterized protein
EOG0912015F	AGLA003761	Anoplophora glabripennis	Putative uncharacterized protein
EOG0912015F	APLA014071	Agrilus planipennis	Putative uncharacterized protein
EOG0912015F	TC007447	Tribolium castaneum	Putative uncharacterized protein
EOG0912015F	LDEC007964	Leptinotarsa decemlineata	Putative uncharacterized protein
EOG0912015F	YQE_08610	Dendroctonus ponderosae	Putative uncharacterized protein
EOG0912015J	OTAU010164	Onthophagus taurus	Nucleolar GTP-binding protein 1
EOG0912015J	AGLA008277	Anoplophora glabripennis	Nucleolar GTP-binding protein 1
EOG0912015J	APLA012580	Agrilus planipennis	Nucleolar GTP-binding protein 1
EOG0912015J	TC008833	Tribolium castaneum	Nucleolar GTP-binding protein 1
EOG0912015J	LDEC016650	Leptinotarsa decemlineata	Nucleolar GTP-binding protein 1
EOG0912015J	YQE_13008	Dendroctonus ponderosae	Nucleolar GTP-binding protein 1
EOG0912015N	OTAU012901	Onthophagus taurus	Putative uncharacterized protein
EOG0912015N	AGLA013699	Anoplophora glabripennis	Putative uncharacterized protein
EOG0912015N	APLA010577	Agrilus planipennis	Putative uncharacterized protein
EOG0912015N	TC030733	Tribolium castaneum	Putative uncharacterized protein
EOG0912015N	LDEC016544	Leptinotarsa decemlineata	Putative uncharacterized protein
EOG0912015N	YQE_05943	Dendroctonus ponderosae	Putative uncharacterized protein
EOG0912015O	OTAU006147	Onthophagus taurus	DEAD (Asp-Glu-Ala-Asp) box polypeptide 10
EOG0912015O	AGLA001387	Anoplophora glabripennis	DEAD (Asp-Glu-Ala-Asp) box polypeptide 10
EOG0912015O	APLA006624	Agrilus planipennis	DEAD (Asp-Glu-Ala-Asp) box polypeptide 10
EOG0912015O	TC001101	Tribolium castaneum	DEAD (Asp-Glu-Ala-Asp) box polypeptide 10
EOG0912015O	LDEC007314	Leptinotarsa decemlineata	DEAD (Asp-Glu-Ala-Asp) box polypeptide 10
EOG0912015O	YQE_11655	Dendroctonus ponderosae	DEAD (Asp-Glu-Ala-Asp) box polypeptide 10
EOG0912015P	OTAU008618	Onthophagus taurus	Putative uncharacterized protein
EOG0912015P	AGLA010037	Anoplophora glabripennis	Putative uncharacterized protein
EOG0912015P	APLA013393	Agrilus planipennis	Putative uncharacterized protein
EOG0912015P	TC008338	Tribolium castaneum	Putative uncharacterized protein
EOG0912015P	LDEC003812	Leptinotarsa decemlineata	Putative uncharacterized protein
EOG0912015P	YQE_02684	Dendroctonus ponderosae	Putative uncharacterized protein
EOG0912015Q	OTAU002176	Onthophagus taurus	Putative uncharacterized protein
EOG0912015Q	AGLA011415	Anoplophora glabripennis	Putative uncharacterized protein
EOG0912015Q	APLA014375	Agrilus planipennis	Putative uncharacterized protein

EOG0912015Q	TC011905	Tribolium castaneum	Putative uncharacterized protein
EOG0912015Q	LDEC017309	Leptinotarsa decemlineata	Putative uncharacterized protein
EOG0912015Q	YQE_10085	Dendroctonus ponderosae	Putative uncharacterized protein
EOG0912015S	OTAU003960	Onthophagus taurus	Putative uncharacterized protein
EOG0912015S	AGLA006037	Anoplophora glabripennis	Putative uncharacterized protein
EOG0912015S	APLA002620	Agrilus planipennis	Putative uncharacterized protein
EOG0912015S	TC001922	Tribolium castaneum	Putative uncharacterized protein
EOG0912015S	LDEC005146	Leptinotarsa decemlineata	Putative uncharacterized protein
EOG0912015S	YQE_12549	Dendroctonus ponderosae	Putative uncharacterized protein
EOG0912015U	OTAU006916	Onthophagus taurus	Putative uncharacterized protein
EOG0912015U	AGLA021721	Anoplophora glabripennis	Putative uncharacterized protein
EOG0912015U	APLA009165	Agrilus planipennis	Putative uncharacterized protein
EOG0912015U	TC002358	Tribolium castaneum	Putative uncharacterized protein
EOG0912015U	LDEC010256	Leptinotarsa decemlineata	Putative uncharacterized protein
EOG0912015U	YQE_09973	Dendroctonus ponderosae	Putative uncharacterized protein
EOG0912015W	OTAU014953	Onthophagus taurus	None
EOG0912015W	AGLA011064	Anoplophora glabripennis	None
EOG0912015W	APLA013609	Agrilus planipennis	None
EOG0912015W	TC031000	Tribolium castaneum	None
EOG0912015W	LDEC002571	Leptinotarsa decemlineata	None
EOG0912015W	YQE_01825	Dendroctonus ponderosae	None
EOG0912015Y	OTAU008164	Onthophagus taurus	Serine protease P153
EOG0912015Y	AGLA016189	Anoplophora glabripennis	Serine protease P153
EOG0912015Y	APLA001859	Agrilus planipennis	Serine protease P153
EOG0912015Y	TC015110	Tribolium castaneum	Serine protease P153
EOG0912015Y	LDEC001952	Leptinotarsa decemlineata	Serine protease P153
EOG0912015Y	YQE_11884	Dendroctonus ponderosae	Serine protease P153
EOG0912015Z	OTAU000157	Onthophagus taurus	Putative uncharacterized protein
EOG0912015Z	AGLA006272	Anoplophora glabripennis	Putative uncharacterized protein
EOG0912015Z	APLA002397	Agrilus planipennis	Putative uncharacterized protein
EOG0912015Z	TC014330	Tribolium castaneum	Putative uncharacterized protein
EOG0912015Z	LDEC005559	Leptinotarsa decemlineata	Putative uncharacterized protein
EOG0912015Z	YQE_02676	Dendroctonus ponderosae	Putative uncharacterized protein
EOG09120163	OTAU001636	Onthophagus taurus	Putative uncharacterized protein
EOG09120163	AGLA017561	Anoplophora glabripennis	Putative uncharacterized protein
EOG09120163	APLA012107	Agrilus planipennis	Putative uncharacterized protein
EOG09120163	TC006754	Tribolium castaneum	Putative uncharacterized protein
EOG09120163	LDEC019104	Leptinotarsa decemlineata	Putative uncharacterized protein
EOG09120163	YQE_06589	Dendroctonus ponderosae	Putative uncharacterized protein
EOG09120164	OTAU014707	Onthophagus taurus	Putative uncharacterized protein
EOG09120164	AGLA002593	Anoplophora glabripennis	Putative uncharacterized protein
EOG09120164	APLA004005	Agrilus planipennis	Putative uncharacterized protein
EOG09120164	TC016289	Tribolium castaneum	Putative uncharacterized protein
EOG09120164	LDEC003405	Leptinotarsa decemlineata	Putative uncharacterized protein
EOG09120164	YQE_08283	Dendroctonus ponderosae	Putative uncharacterized protein
EOG09120165	OTAU012919	Onthophagus taurus	Putative uncharacterized protein
EOG09120165	AGLA021443	Anoplophora glabripennis	Putative uncharacterized protein
EOG09120165	APLA000544	Agrilus planipennis	Putative uncharacterized protein
EOG09120165	TC006380	Tribolium castaneum	Putative uncharacterized protein
EOG09120165	LDEC002635	Leptinotarsa decemlineata	Putative uncharacterized protein
EOG09120165	YQE_11696	Dendroctonus ponderosae	Putative uncharacterized protein
EOG09120167	OTAU001432	Onthophagus taurus	None
EOG09120167	AGLA001139	Anoplophora glabripennis	None
EOG09120167	APLA014550	Agrilus planipennis	None
EOG09120167	TC034531	Tribolium castaneum	None
EOG09120167	LDEC013992	Leptinotarsa decemlineata	None
EOG09120167	YQE_09165	Dendroctonus ponderosae	None
EOG09120168	OTAU011403	Onthophagus taurus	Putative uncharacterized protein
EOG09120168	AGLA005485	Anoplophora glabripennis	Putative uncharacterized protein
EOG09120168	APLA015126	Agrilus planipennis	Putative uncharacterized protein
EOG09120168	TC013521	Tribolium castaneum	Putative uncharacterized protein
EOG09120168	LDEC008332	Leptinotarsa decemlineata	Putative uncharacterized protein
EOG09120168	YQE_06281	Dendroctonus ponderosae	Putative uncharacterized protein
EOG0912016A	OTAU013245	Onthophagus taurus	ATP binding
EOG0912016A	AGLA001349	Anoplophora glabripennis	ATP binding
EOG0912016A	APLA013122	Agrilus planipennis	ATP binding
EOG0912016A	TC032227	Tribolium castaneum	ATP binding

EOG0912016A	LDEC003557	Leptinotarsa decemlineata	ATP binding
EOG0912016A	YQE_08107	Dendroctonus ponderosae	ATP binding
EOG0912016D	OTAU007169	Onthophagus taurus	Putative uncharacterized protein
EOG0912016D	AGLA009235	Anoplophora glabripennis	Putative uncharacterized protein
EOG0912016D	APLA005917	Agrilus planipennis	Putative uncharacterized protein
EOG0912016D	TC015557	Tribolium castaneum	Putative uncharacterized protein
EOG0912016D	LDEC002539	Leptinotarsa decemlineata	Putative uncharacterized protein
EOG0912016D	YQE_11458	Dendroctonus ponderosae	Putative uncharacterized protein
EOG0912016E	OTAU012714	Onthophagus taurus	Putative uncharacterized protein
EOG0912016E	AGLA001548	Anoplophora glabripennis	Putative uncharacterized protein
EOG0912016E	APLA004101	Agrilus planipennis	Putative uncharacterized protein
EOG0912016E	TC002584	Tribolium castaneum	Putative uncharacterized protein
EOG0912016E	LDEC020512	Leptinotarsa decemlineata	Putative uncharacterized protein
EOG0912016E	YQE_03809	Dendroctonus ponderosae	Putative uncharacterized protein
EOG0912016G	OTAU004306	Onthophagus taurus	Putative uncharacterized protein
EOG0912016G	AGLA002223	Anoplophora glabripennis	Putative uncharacterized protein
EOG0912016G	APLA003571	Agrilus planipennis	Putative uncharacterized protein
EOG0912016G	TC004130	Tribolium castaneum	Putative uncharacterized protein
EOG0912016G	LDEC021386	Leptinotarsa decemlineata	Putative uncharacterized protein
EOG0912016G	YQE_09158	Dendroctonus ponderosae	Putative uncharacterized protein
EOG0912016H	OTAU004221	Onthophagus taurus	Putative uncharacterized protein
EOG0912016H	AGLA009267	Anoplophora glabripennis	Putative uncharacterized protein
EOG0912016H	APLA004861	Agrilus planipennis	Putative uncharacterized protein
EOG0912016H	TC014378	Tribolium castaneum	Putative uncharacterized protein
EOG0912016H	LDEC009038	Leptinotarsa decemlineata	Putative uncharacterized protein
EOG0912016H	YQE_08553	Dendroctonus ponderosae	Putative uncharacterized protein
EOG0912016I	OTAU001251	Onthophagus taurus	calcium ion binding
EOG0912016I	AGLA000220	Anoplophora glabripennis	calcium ion binding
EOG0912016I	APLA003553	Agrilus planipennis	calcium ion binding
EOG0912016I	TC031545	Tribolium castaneum	calcium ion binding
EOG0912016I	LDEC001072	Leptinotarsa decemlineata	calcium ion binding
EOG0912016I	YQE_09056	Dendroctonus ponderosae	calcium ion binding
EOG0912016L	OTAU001613	Onthophagus taurus	Putative uncharacterized protein
EOG0912016L	AGLA012680	Anoplophora glabripennis	Putative uncharacterized protein
EOG0912016L	APLA001197	Agrilus planipennis	Putative uncharacterized protein
EOG0912016L	TC006728	Tribolium castaneum	Putative uncharacterized protein
EOG0912016L	LDEC007979	Leptinotarsa decemlineata	Putative uncharacterized protein
EOG0912016L	YQE_12149	Dendroctonus ponderosae	Putative uncharacterized protein
EOG0912016O	OTAU009946	Onthophagus taurus	Putative uncharacterized protein
EOG0912016O	AGLA020704	Anoplophora glabripennis	Putative uncharacterized protein
EOG0912016O	APLA006014	Agrilus planipennis	Putative uncharacterized protein
EOG0912016O	TC008673	Tribolium castaneum	Putative uncharacterized protein
EOG0912016O	LDEC012324	Leptinotarsa decemlineata	Putative uncharacterized protein
EOG0912016O	YQE_03503	Dendroctonus ponderosae	Putative uncharacterized protein
EOG0912016Q	OTAU001623	Onthophagus taurus	Putative uncharacterized protein
EOG0912016Q	AGLA010217	Anoplophora glabripennis	Putative uncharacterized protein
EOG0912016Q	APLA012068	Agrilus planipennis	Putative uncharacterized protein
EOG0912016Q	TC005902	Tribolium castaneum	Putative uncharacterized protein
EOG0912016Q	LDEC002972	Leptinotarsa decemlineata	Putative uncharacterized protein
EOG0912016Q	YQE_12115	Dendroctonus ponderosae	Putative uncharacterized protein
EOG0912016S	OTAU015834	Onthophagus taurus	ion channel activity
EOG0912016S	AGLA002077	Anoplophora glabripennis	ion channel activity
EOG0912016S	APLA003775	Agrilus planipennis	ion channel activity
EOG0912016S	TC032339	Tribolium castaneum	ion channel activity
EOG0912016S	LDEC009195	Leptinotarsa decemlineata	ion channel activity
EOG0912016S	YQE_04943	Dendroctonus ponderosae	ion channel activity
EOG0912016T	OTAU008260	Onthophagus taurus	Putative uncharacterized protein
EOG0912016T	AGLA019274	Anoplophora glabripennis	Putative uncharacterized protein
EOG0912016T	APLA008555	Agrilus planipennis	Putative uncharacterized protein
EOG0912016T	TC030730	Tribolium castaneum	Putative uncharacterized protein
EOG0912016T	LDEC002029	Leptinotarsa decemlineata	Putative uncharacterized protein
EOG0912016T	YQE_06384	Dendroctonus ponderosae	Putative uncharacterized protein
EOG0912016U	OTAU010631	Onthophagus taurus	Putative uncharacterized protein
EOG0912016U	AGLA018749	Anoplophora glabripennis	Putative uncharacterized protein
EOG0912016U	APLA003049	Agrilus planipennis	Putative uncharacterized protein
EOG0912016U	TC013390	Tribolium castaneum	Putative uncharacterized protein
EOG0912016U	LDEC017191	Leptinotarsa decemlineata	Putative uncharacterized protein

EOG0912016U	YQE_06094	Dendroctonus ponderosae	Putative uncharacterized protein
EOG0912016V	OTAU012340	Onthophagus taurus	Putative uncharacterized protein
EOG0912016V	AGLA005279	Anoplophora glabripennis	Putative uncharacterized protein
EOG0912016V	APLA014672	Agrilus planipennis	Putative uncharacterized protein
EOG0912016V	TC008265	Tribolium castaneum	Putative uncharacterized protein
EOG0912016V	LDEC001356	Leptinotarsa decemlineata	Putative uncharacterized protein
EOG0912016V	YQE_05723	Dendroctonus ponderosae	Putative uncharacterized protein
EOG0912016X	OTAU003660	Onthophagus taurus	Putative uncharacterized protein
EOG0912016X	AGLA009514	Anoplophora glabripennis	Putative uncharacterized protein
EOG0912016X	APLA002270	Agrilus planipennis	Putative uncharacterized protein
EOG0912016X	TC008216	Tribolium castaneum	Putative uncharacterized protein
EOG0912016X	LDEC000091	Leptinotarsa decemlineata	Putative uncharacterized protein
EOG0912016X	YQE_10937	Dendroctonus ponderosae	Putative uncharacterized protein
EOG0912016Y	OTAU002615	Onthophagus taurus	Suppressor of hairless
EOG0912016Y	AGLA008854	Anoplophora glabripennis	Suppressor of hairless
EOG0912016Y	APLA002684	Agrilus planipennis	Suppressor of hairless
EOG0912016Y	TC014468	Tribolium castaneum	Suppressor of hairless
EOG0912016Y	LDEC018359	Leptinotarsa decemlineata	Suppressor of hairless
EOG0912016Y	YQE_03219	Dendroctonus ponderosae	Suppressor of hairless
EOG09120173	OTAU007506	Onthophagus taurus	acetylgalactosaminyltransferase activity
EOG09120173	AGLA010452	Anoplophora glabripennis	acetylgalactosaminyltransferase activity
EOG09120173	APLA009108	Agrilus planipennis	acetylgalactosaminyltransferase activity
EOG09120173	TC033453	Tribolium castaneum	acetylgalactosaminyltransferase activity
EOG09120173	LDEC010472	Leptinotarsa decemlineata	acetylgalactosaminyltransferase activity
EOG09120173	YQE_11763	Dendroctonus ponderosae	acetylgalactosaminyltransferase activity
EOG09120177	OTAU010048	Onthophagus taurus	Putative uncharacterized protein
EOG09120177	AGLA002941	Anoplophora glabripennis	Putative uncharacterized protein
EOG09120177	APLA005385	Agrilus planipennis	Putative uncharacterized protein
EOG09120177	TC007679	Tribolium castaneum	Putative uncharacterized protein
EOG09120177	LDEC002423	Leptinotarsa decemlineata	Putative uncharacterized protein
EOG09120177	YQE_11900	Dendroctonus ponderosae	Putative uncharacterized protein
EOG09120178	OTAU011542	Onthophagus taurus	Putative uncharacterized protein
EOG09120178	AGLA003413	Anoplophora glabripennis	Putative uncharacterized protein
EOG09120178	APLA002027	Agrilus planipennis	Putative uncharacterized protein
EOG09120178	TC002608	Tribolium castaneum	Putative uncharacterized protein
EOG09120178	LDEC020451	Leptinotarsa decemlineata	Putative uncharacterized protein
EOG09120178	YQE_02739	Dendroctonus ponderosae	Putative uncharacterized protein
EOG0912017C	OTAU015861	Onthophagus taurus	Putative uncharacterized protein
EOG0912017C	AGLA021000	Anoplophora glabripennis	Putative uncharacterized protein
EOG0912017C	APLA007223	Agrilus planipennis	Putative uncharacterized protein
EOG0912017C	TC002447	Tribolium castaneum	Putative uncharacterized protein
EOG0912017C	LDEC008584	Leptinotarsa decemlineata	Putative uncharacterized protein
EOG0912017C	YQE_02149	Dendroctonus ponderosae	Putative uncharacterized protein
EOG0912017D	OTAU000445	Onthophagus taurus	Putative uncharacterized protein
EOG0912017D	AGLA001276	Anoplophora glabripennis	Putative uncharacterized protein
EOG0912017D	APLA003299	Agrilus planipennis	Putative uncharacterized protein
EOG0912017D	TC012616	Tribolium castaneum	Putative uncharacterized protein
EOG0912017D	LDEC007712	Leptinotarsa decemlineata	Putative uncharacterized protein
EOG0912017D	YQE_10914	Dendroctonus ponderosae	Putative uncharacterized protein
EOG0912017E	OTAU007085	Onthophagus taurus	Putative uncharacterized protein
EOG0912017E	AGLA018722	Anoplophora glabripennis	Putative uncharacterized protein
EOG0912017E	APLA010720	Agrilus planipennis	Putative uncharacterized protein
EOG0912017E	TC014098	Tribolium castaneum	Putative uncharacterized protein
EOG0912017E	LDEC007356	Leptinotarsa decemlineata	Putative uncharacterized protein
EOG0912017E	YQE_08651	Dendroctonus ponderosae	Putative uncharacterized protein
EOG0912017F	OTAU001386	Onthophagus taurus	"transferase activity, transferring phosphorus-containing groups"
EOG0912017F	AGLA002154	Anoplophora glabripennis	"transferase activity, transferring phosphorus-containing groups"
EOG0912017F	APLA005627	Agrilus planipennis	"transferase activity, transferring phosphorus-containing groups"
EOG0912017F	TC034518	Tribolium castaneum	"transferase activity, transferring phosphorus-containing groups"
EOG0912017F	LDEC021763	Leptinotarsa decemlineata	"transferase activity, transferring phosphorus-containing groups"
EOG0912017F	YQE_09283	Dendroctonus ponderosae	"transferase activity, transferring phosphorus-containing groups"

EOG0912017G	OTAU012994	Onthophagus taurus	Putative uncharacterized protein
EOG0912017G	AGLA007740	Anoplophora glabripennis	Putative uncharacterized protein
EOG0912017G	APLA003754	Agrilus planipennis	Putative uncharacterized protein
EOG0912017G	TC002496	Tribolium castaneum	Putative uncharacterized protein
EOG0912017G	LDEC000226	Leptinotarsa decemlineata	Putative uncharacterized protein
EOG0912017G	YQE_09934	Dendroctonus ponderosae	Putative uncharacterized protein
EOG0912017L	OTAU017201	Onthophagus taurus	Putative uncharacterized protein
EOG0912017L	AGLA013466	Anoplophora glabripennis	Putative uncharacterized protein
EOG0912017L	APLA003252	Agrilus planipennis	Putative uncharacterized protein
EOG0912017L	TC010635	Tribolium castaneum	Putative uncharacterized protein
EOG0912017L	LDEC009329	Leptinotarsa decemlineata	Putative uncharacterized protein
EOG0912017L	YQE_12813	Dendroctonus ponderosae	Putative uncharacterized protein
EOG0912017N	OTAU001342	Onthophagus taurus	Putative uncharacterized protein
EOG0912017N	AGLA001181	Anoplophora glabripennis	Putative uncharacterized protein
EOG0912017N	APLA009677	Agrilus planipennis	Putative uncharacterized protein
EOG0912017N	TC006846	Tribolium castaneum	Putative uncharacterized protein
EOG0912017N	LDEC015737	Leptinotarsa decemlineata	Putative uncharacterized protein
EOG0912017N	YQE_02443	Dendroctonus ponderosae	Putative uncharacterized protein
EOG0912017P	OTAU002768	Onthophagus taurus	Putative uncharacterized protein
EOG0912017P	AGLA002178	Anoplophora glabripennis	Putative uncharacterized protein
EOG0912017P	APLA005623	Agrilus planipennis	Putative uncharacterized protein
EOG0912017P	TC001607	Tribolium castaneum	Putative uncharacterized protein
EOG0912017P	LDEC001521	Leptinotarsa decemlineata	Putative uncharacterized protein
EOG0912017P	YQE_03280	Dendroctonus ponderosae	Putative uncharacterized protein
EOG0912017Q	OTAU009007	Onthophagus taurus	None
EOG0912017Q	AGLA012410	Anoplophora glabripennis	None
EOG0912017Q	APLA008267	Agrilus planipennis	None
EOG0912017Q	TC033250	Tribolium castaneum	None
EOG0912017Q	LDEC001189	Leptinotarsa decemlineata	None
EOG0912017Q	YQE_04007	Dendroctonus ponderosae	None
EOG0912017S	OTAU016602	Onthophagus taurus	Putative uncharacterized protein
EOG0912017S	AGLA005226	Anoplophora glabripennis	Putative uncharacterized protein
EOG0912017S	APLA005229	Agrilus planipennis	Putative uncharacterized protein
EOG0912017S	TC011326	Tribolium castaneum	Putative uncharacterized protein
EOG0912017S	LDEC018928	Leptinotarsa decemlineata	Putative uncharacterized protein
EOG0912017S	YQE_10682	Dendroctonus ponderosae	Putative uncharacterized protein
EOG0912017T	OTAU007126	Onthophagus taurus	Putative uncharacterized protein
EOG0912017T	AGLA000456	Anoplophora glabripennis	Putative uncharacterized protein
EOG0912017T	APLA010882	Agrilus planipennis	Putative uncharacterized protein
EOG0912017T	TC007163	Tribolium castaneum	Putative uncharacterized protein
EOG0912017T	LDEC000976	Leptinotarsa decemlineata	Putative uncharacterized protein
EOG0912017T	YQE_07365	Dendroctonus ponderosae	Putative uncharacterized protein
EOG0912017V	OTAU011658	Onthophagus taurus	Putative uncharacterized protein
EOG0912017V	AGLA003455	Anoplophora glabripennis	Putative uncharacterized protein
EOG0912017V	APLA013324	Agrilus planipennis	Putative uncharacterized protein
EOG0912017V	TC009607	Tribolium castaneum	Putative uncharacterized protein
EOG0912017V	LDEC005235	Leptinotarsa decemlineata	Putative uncharacterized protein
EOG0912017V	YQE_13031	Dendroctonus ponderosae	Putative uncharacterized protein
EOG0912017X	OTAU000019	Onthophagus taurus	calcium ion binding
EOG0912017X	AGLA006405	Anoplophora glabripennis	calcium ion binding
EOG0912017X	APLA003099	Agrilus planipennis	calcium ion binding
EOG0912017X	TC031143	Tribolium castaneum	calcium ion binding
EOG0912017X	LDEC023349	Leptinotarsa decemlineata	calcium ion binding
EOG0912017X	YQE_06086	Dendroctonus ponderosae	calcium ion binding
EOG09120180	OTAU015401	Onthophagus taurus	Putative uncharacterized protein
EOG09120180	AGLA006031	Anoplophora glabripennis	Putative uncharacterized protein
EOG09120180	APLA009529	Agrilus planipennis	Putative uncharacterized protein
EOG09120180	TC011734	Tribolium castaneum	Putative uncharacterized protein
EOG09120180	LDEC011807	Leptinotarsa decemlineata	Putative uncharacterized protein
EOG09120180	YQE_07939	Dendroctonus ponderosae	Putative uncharacterized protein
EOG09120181	OTAU001249	Onthophagus taurus	None
EOG09120181	AGLA000223	Anoplophora glabripennis	None
EOG09120181	APLA006512	Agrilus planipennis	None
EOG09120181	TC031547	Tribolium castaneum	None
EOG09120181	LDEC001055	Leptinotarsa decemlineata	None
EOG09120181	YQE_07129	Dendroctonus ponderosae	None
EOG09120184	OTAU003686	Onthophagus taurus	DEAD/H (Asp-Glu-Ala-Asp/His) box polypeptide 11

EOG09120184	AGLA009026	Anoplophora glabripennis	DEAD/H (Asp-Glu-Ala-Asp/His) box polypeptide 11
EOG09120184	APLA005369	Agrilus planipennis	DEAD/H (Asp-Glu-Ala-Asp/His) box polypeptide 11
EOG09120184	TC008173	Tribolium castaneum	DEAD/H (Asp-Glu-Ala-Asp/His) box polypeptide 11
EOG09120184	LDEC006102	Leptinotarsa decemlineata	DEAD/H (Asp-Glu-Ala-Asp/His) box polypeptide 11
EOG09120184	YQE_01755	Dendroctonus ponderosae	DEAD/H (Asp-Glu-Ala-Asp/His) box polypeptide 11
EOG09120186	OTAU005887	Onthophagus taurus	Spire
EOG09120186	AGLA005493	Anoplophora glabripennis	Spire
EOG09120186	APLA014280	Agrilus planipennis	Spire
EOG09120186	TC014290	Tribolium castaneum	Spire
EOG09120186	LDEC003054	Leptinotarsa decemlineata	Spire
EOG09120186	YQE_06056	Dendroctonus ponderosae	Spire
EOG09120189	OTAU012539	Onthophagus taurus	Putative uncharacterized protein
EOG09120189	AGLA008419	Anoplophora glabripennis	Putative uncharacterized protein
EOG09120189	APLA008960	Agrilus planipennis	Putative uncharacterized protein
EOG09120189	TC004420	Tribolium castaneum	Putative uncharacterized protein
EOG09120189	LDEC001726	Leptinotarsa decemlineata	Putative uncharacterized protein
EOG09120189	YQE_03301	Dendroctonus ponderosae	Putative uncharacterized protein
EOG0912018A	OTAU004473	Onthophagus taurus	Putative uncharacterized protein
EOG0912018A	AGLA006094	Anoplophora glabripennis	Putative uncharacterized protein
EOG0912018A	APLA002084	Agrilus planipennis	Putative uncharacterized protein
EOG0912018A	TC003373	Tribolium castaneum	Putative uncharacterized protein
EOG0912018A	LDEC005477	Leptinotarsa decemlineata	Putative uncharacterized protein
EOG0912018A	YQE_10277	Dendroctonus ponderosae	Putative uncharacterized protein
EOG0912018D	OTAU001874	Onthophagus taurus	Putative uncharacterized protein
EOG0912018D	AGLA012276	Anoplophora glabripennis	Putative uncharacterized protein
EOG0912018D	APLA000910	Agrilus planipennis	Putative uncharacterized protein
EOG0912018D	TC005697	Tribolium castaneum	Putative uncharacterized protein
EOG0912018D	LDEC009254	Leptinotarsa decemlineata	Putative uncharacterized protein
EOG0912018D	YQE_12282	Dendroctonus ponderosae	Putative uncharacterized protein
EOG0912018E	OTAU011749	Onthophagus taurus	Putative uncharacterized protein
EOG0912018E	AGLA006344	Anoplophora glabripennis	Putative uncharacterized protein
EOG0912018E	APLA010705	Agrilus planipennis	Putative uncharacterized protein
EOG0912018E	TC006108	Tribolium castaneum	Putative uncharacterized protein
EOG0912018E	LDEC007863	Leptinotarsa decemlineata	Putative uncharacterized protein
EOG0912018E	YQE_04662	Dendroctonus ponderosae	Putative uncharacterized protein
EOG0912018F	OTAU003883	Onthophagus taurus	Putative uncharacterized protein
EOG0912018F	AGLA005766	Anoplophora glabripennis	Putative uncharacterized protein
EOG0912018F	APLA011253	Agrilus planipennis	Putative uncharacterized protein
EOG0912018F	TC008635	Tribolium castaneum	Putative uncharacterized protein
EOG0912018F	LDEC017979	Leptinotarsa decemlineata	Putative uncharacterized protein
EOG0912018F	YQE_11522	Dendroctonus ponderosae	Putative uncharacterized protein
EOG0912018K	OTAU002001	Onthophagus taurus	None
EOG0912018K	AGLA004051	Anoplophora glabripennis	None
EOG0912018K	APLA008190	Agrilus planipennis	None
EOG0912018K	TC033649	Tribolium castaneum	None
EOG0912018K	LDEC020392	Leptinotarsa decemlineata	None
EOG0912018K	YQE_10300	Dendroctonus ponderosae	None
EOG0912018M	OTAU008838	Onthophagus taurus	Putative uncharacterized protein
EOG0912018M	AGLA014084	Anoplophora glabripennis	Putative uncharacterized protein
EOG0912018M	APLA004743	Agrilus planipennis	Putative uncharacterized protein
EOG0912018M	TC010062	Tribolium castaneum	Putative uncharacterized protein
EOG0912018M	LDEC004565	Leptinotarsa decemlineata	Putative uncharacterized protein
EOG0912018M	YQE_05351	Dendroctonus ponderosae	Putative uncharacterized protein
EOG0912018O	OTAU002155	Onthophagus taurus	Putative uncharacterized protein
EOG0912018O	AGLA007500	Anoplophora glabripennis	Putative uncharacterized protein
EOG0912018O	APLA009694	Agrilus planipennis	Putative uncharacterized protein
EOG0912018O	TC002139	Tribolium castaneum	Putative uncharacterized protein
EOG0912018O	LDEC013826	Leptinotarsa decemlineata	Putative uncharacterized protein
EOG0912018O	YQE_08270	Dendroctonus ponderosae	Putative uncharacterized protein
EOG0912018Q	OTAU003127	Onthophagus taurus	Transporter
EOG0912018Q	AGLA016010	Anoplophora glabripennis	Transporter
EOG0912018Q	APLA014643	Agrilus planipennis	Transporter
EOG0912018Q	TC002347	Tribolium castaneum	Transporter
EOG0912018Q	LDEC005025	Leptinotarsa decemlineata	Transporter
EOG0912018Q	YQE_07922	Dendroctonus ponderosae	Transporter
EOG0912018U	OTAU006866	Onthophagus taurus	Putative uncharacterized protein
EOG0912018U	AGLA011722	Anoplophora glabripennis	Putative uncharacterized protein

EOG0912018U	APLA005790	Agrilus planipennis	Putative uncharacterized protein
EOG0912018U	TC014758	Tribolium castaneum	Putative uncharacterized protein
EOG0912018U	LDEC017505	Leptinotarsa decemlineata	Putative uncharacterized protein
EOG0912018U	YQE_07230	Dendroctonus ponderosae	Putative uncharacterized protein
EOG0912018V	OTAU008637	Onthophagus taurus	Putative uncharacterized protein
EOG0912018V	AGLA002604	Anoplophora glabripennis	Putative uncharacterized protein
EOG0912018V	APLA002325	Agrilus planipennis	Putative uncharacterized protein
EOG0912018V	TC010662	Tribolium castaneum	Putative uncharacterized protein
EOG0912018V	LDEC001975	Leptinotarsa decemlineata	Putative uncharacterized protein
EOG0912018V	YQE_11225	Dendroctonus ponderosae	Putative uncharacterized protein
EOG0912018W	OTAU003560	Onthophagus taurus	Putative uncharacterized protein
EOG0912018W	AGLA015975	Anoplophora glabripennis	Putative uncharacterized protein
EOG0912018W	APLA000201	Agrilus planipennis	Putative uncharacterized protein
EOG0912018W	TC030745	Tribolium castaneum	Putative uncharacterized protein
EOG0912018W	LDEC004400	Leptinotarsa decemlineata	Putative uncharacterized protein
EOG0912018W	YQE_04901	Dendroctonus ponderosae	Putative uncharacterized protein
EOG0912018Z	OTAU004039	Onthophagus taurus	Putative uncharacterized protein
EOG0912018Z	AGLA020660	Anoplophora glabripennis	Putative uncharacterized protein
EOG0912018Z	APLA006640	Agrilus planipennis	Putative uncharacterized protein
EOG0912018Z	TC010332	Tribolium castaneum	Putative uncharacterized protein
EOG0912018Z	LDEC004051	Leptinotarsa decemlineata	Putative uncharacterized protein
EOG0912018Z	YQE_11680	Dendroctonus ponderosae	Putative uncharacterized protein
EOG09120190	OTAU003835	Onthophagus taurus	Putative uncharacterized protein
EOG09120190	AGLA020334	Anoplophora glabripennis	Putative uncharacterized protein
EOG09120190	APLA010287	Agrilus planipennis	Putative uncharacterized protein
EOG09120190	TC009046	Tribolium castaneum	Putative uncharacterized protein
EOG09120190	LDEC005219	Leptinotarsa decemlineata	Putative uncharacterized protein
EOG09120190	YQE_08806	Dendroctonus ponderosae	Putative uncharacterized protein
EOG09120191	OTAU008088	Onthophagus taurus	Putative uncharacterized protein
EOG09120191	AGLA014977	Anoplophora glabripennis	Putative uncharacterized protein
EOG09120191	APLA008540	Agrilus planipennis	Putative uncharacterized protein
EOG09120191	TC002984	Tribolium castaneum	Putative uncharacterized protein
EOG09120191	LDEC012832	Leptinotarsa decemlineata	Putative uncharacterized protein
EOG09120191	YQE_09832	Dendroctonus ponderosae	Putative uncharacterized protein
EOG09120192	OTAU015977	Onthophagus taurus	Transporter
EOG09120192	AGLA015784	Anoplophora glabripennis	Transporter
EOG09120192	APLA003251	Agrilus planipennis	Transporter
EOG09120192	TC010681	Tribolium castaneum	Transporter
EOG09120192	LDEC021672	Leptinotarsa decemlineata	Transporter
EOG09120192	YQE_12803	Dendroctonus ponderosae	Transporter
EOG09120193	OTAU006620	Onthophagus taurus	DNA ligase
EOG09120193	AGLA009168	Anoplophora glabripennis	DNA ligase
EOG09120193	APLA006674	Agrilus planipennis	DNA ligase
EOG09120193	TC003800	Tribolium castaneum	DNA ligase
EOG09120193	LDEC010799	Leptinotarsa decemlineata	DNA ligase
EOG09120193	YQE_09817	Dendroctonus ponderosae	DNA ligase
EOG09120195	OTAU004193	Onthophagus taurus	Putative uncharacterized protein
EOG09120195	AGLA010789	Anoplophora glabripennis	Putative uncharacterized protein
EOG09120195	APLA005139	Agrilus planipennis	Putative uncharacterized protein
EOG09120195	TC030756	Tribolium castaneum	Putative uncharacterized protein
EOG09120195	LDEC006504	Leptinotarsa decemlineata	Putative uncharacterized protein
EOG09120195	YQE_06170	Dendroctonus ponderosae	Putative uncharacterized protein
EOG09120196	OTAU006370	Onthophagus taurus	Hydroxysteroid (17-beta) dehydrogenase 4
EOG09120196	AGLA020274	Anoplophora glabripennis	Hydroxysteroid (17-beta) dehydrogenase 4
EOG09120196	APLA008809	Agrilus planipennis	Hydroxysteroid (17-beta) dehydrogenase 4
EOG09120196	TC009495	Tribolium castaneum	Hydroxysteroid (17-beta) dehydrogenase 4
EOG09120196	LDEC011014	Leptinotarsa decemlineata	Hydroxysteroid (17-beta) dehydrogenase 4
EOG09120196	YQE_05388	Dendroctonus ponderosae	Hydroxysteroid (17-beta) dehydrogenase 4
EOG09120198	OTAU004554	Onthophagus taurus	Putative uncharacterized protein
EOG09120198	AGLA008285	Anoplophora glabripennis	Putative uncharacterized protein
EOG09120198	APLA012592	Agrilus planipennis	Putative uncharacterized protein
EOG09120198	TC009343	Tribolium castaneum	Putative uncharacterized protein
EOG09120198	LDEC016636	Leptinotarsa decemlineata	Putative uncharacterized protein
EOG09120198	YQE_08746	Dendroctonus ponderosae	Putative uncharacterized protein
EOG0912019A	OTAU000058	Onthophagus taurus	Cryptochrome 2
EOG0912019A	AGLA006406	Anoplophora glabripennis	Cryptochrome 2
EOG0912019A	APLA011443	Agrilus planipennis	Cryptochrome 2

EOG0912019A	TC010454	Tribolium castaneum	Cryptochrome 2
EOG0912019A	LDEC013805	Leptinotarsa decemlineata	Cryptochrome 2
EOG0912019A	YQE_06087	Dendroctonus ponderosae	Cryptochrome 2
EOG0912019C	OTAU008665	Onthophagus taurus	monooxygenase activity
EOG0912019C	AGLA007330	Anoplophora glabripennis	monooxygenase activity
EOG0912019C	APLA000941	Agrilus planipennis	monooxygenase activity
EOG0912019C	TC034197	Tribolium castaneum	monooxygenase activity
EOG0912019C	LDEC013545	Leptinotarsa decemlineata	monooxygenase activity
EOG0912019C	YQE_05776	Dendroctonus ponderosae	monooxygenase activity
EOG0912019D	OTAU000417	Onthophagus taurus	Short-gastrulation
EOG0912019D	AGLA009482	Anoplophora glabripennis	Short-gastrulation
EOG0912019D	APLA009543	Agrilus planipennis	Short-gastrulation
EOG0912019D	TC012650	Tribolium castaneum	Short-gastrulation
EOG0912019D	LDEC004182	Leptinotarsa decemlineata	Short-gastrulation
EOG0912019D	YQE_01998	Dendroctonus ponderosae	Short-gastrulation
EOG0912019E	OTAU009526	Onthophagus taurus	Putative uncharacterized protein
EOG0912019E	AGLA008284	Anoplophora glabripennis	Putative uncharacterized protein
EOG0912019E	APLA014519	Agrilus planipennis	Putative uncharacterized protein
EOG0912019E	TC009342	Tribolium castaneum	Putative uncharacterized protein
EOG0912019E	LDEC016638	Leptinotarsa decemlineata	Putative uncharacterized protein
EOG0912019E	YQE_03379	Dendroctonus ponderosae	Putative uncharacterized protein
EOG0912019F	OTAU012697	Onthophagus taurus	Putative uncharacterized protein
EOG0912019F	AGLA004685	Anoplophora glabripennis	Putative uncharacterized protein
EOG0912019F	APLA005402	Agrilus planipennis	Putative uncharacterized protein
EOG0912019F	TC007678	Tribolium castaneum	Putative uncharacterized protein
EOG0912019F	LDEC002430	Leptinotarsa decemlineata	Putative uncharacterized protein
EOG0912019F	YQE_09012	Dendroctonus ponderosae	Putative uncharacterized protein
EOG0912019G	OTAU003203	Onthophagus taurus	Putative uncharacterized protein
EOG0912019G	AGLA015869	Anoplophora glabripennis	Putative uncharacterized protein
EOG0912019G	APLA007983	Agrilus planipennis	Putative uncharacterized protein
EOG0912019G	TC014300	Tribolium castaneum	Putative uncharacterized protein
EOG0912019G	LDEC003093	Leptinotarsa decemlineata	Putative uncharacterized protein
EOG0912019G	YQE_12404	Dendroctonus ponderosae	Putative uncharacterized protein
EOG0912019I	OTAU007500	Onthophagus taurus	Putative uncharacterized protein
EOG0912019I	AGLA010449	Anoplophora glabripennis	Putative uncharacterized protein
EOG0912019I	APLA009106	Agrilus planipennis	Putative uncharacterized protein
EOG0912019I	TC014903	Tribolium castaneum	Putative uncharacterized protein
EOG0912019I	LDEC010464	Leptinotarsa decemlineata	Putative uncharacterized protein
EOG0912019I	YQE_08392	Dendroctonus ponderosae	Putative uncharacterized protein
EOG0912019J	OTAU000017	Onthophagus taurus	Putative uncharacterized protein
EOG0912019J	AGLA010716	Anoplophora glabripennis	Putative uncharacterized protein
EOG0912019J	APLA012979	Agrilus planipennis	Putative uncharacterized protein
EOG0912019J	TC014151	Tribolium castaneum	Putative uncharacterized protein
EOG0912019J	LDEC022108	Leptinotarsa decemlineata	Putative uncharacterized protein
EOG0912019J	YQE_02680	Dendroctonus ponderosae	Putative uncharacterized protein
EOG0912019K	OTAU011293	Onthophagus taurus	Putative uncharacterized protein
EOG0912019K	AGLA002637	Anoplophora glabripennis	Putative uncharacterized protein
EOG0912019K	APLA002725	Agrilus planipennis	Putative uncharacterized protein
EOG0912019K	TC012910	Tribolium castaneum	Putative uncharacterized protein
EOG0912019K	LDEC015023	Leptinotarsa decemlineata	Putative uncharacterized protein
EOG0912019K	YQE_05086	Dendroctonus ponderosae	Putative uncharacterized protein
EOG0912019M	OTAU014406	Onthophagus taurus	Putative uncharacterized protein
EOG0912019M	AGLA000345	Anoplophora glabripennis	Putative uncharacterized protein
EOG0912019M	APLA003504	Agrilus planipennis	Putative uncharacterized protein
EOG0912019M	TC001636	Tribolium castaneum	Putative uncharacterized protein
EOG0912019M	LDEC015743	Leptinotarsa decemlineata	Putative uncharacterized protein
EOG0912019M	YQE_09177	Dendroctonus ponderosae	Putative uncharacterized protein
EOG0912019N	OTAU005697	Onthophagus taurus	Putative uncharacterized protein
EOG0912019N	AGLA015143	Anoplophora glabripennis	Putative uncharacterized protein
EOG0912019N	APLA001749	Agrilus planipennis	Putative uncharacterized protein
EOG0912019N	TC008907	Tribolium castaneum	Putative uncharacterized protein
EOG0912019N	LDEC009346	Leptinotarsa decemlineata	Putative uncharacterized protein
EOG0912019N	YQE_05333	Dendroctonus ponderosae	Putative uncharacterized protein
EOG0912019Q	OTAU013711	Onthophagus taurus	Putative uncharacterized protein
EOG0912019Q	AGLA017444	Anoplophora glabripennis	Putative uncharacterized protein
EOG0912019Q	APLA011734	Agrilus planipennis	Putative uncharacterized protein
EOG0912019Q	TC003706	Tribolium castaneum	Putative uncharacterized protein

EOG0912019Q	LDEC005980	Leptinotarsa decemlineata	Putative uncharacterized protein
EOG0912019Q	YQE_06179	Dendroctonus ponderosae	Putative uncharacterized protein
EOG0912019R	OTAU000310	Onthophagus taurus	Putative uncharacterized protein
EOG0912019R	AGLA012601	Anoplophora glabripennis	Putative uncharacterized protein
EOG0912019R	APLA005124	Agrilus planipennis	Putative uncharacterized protein
EOG0912019R	TC014251	Tribolium castaneum	Putative uncharacterized protein
EOG0912019R	LDEC001144	Leptinotarsa decemlineata	Putative uncharacterized protein
EOG0912019R	YQE_06224	Dendroctonus ponderosae	Putative uncharacterized protein
EOG0912019S	OTAU011864	Onthophagus taurus	Belle
EOG0912019S	AGLA010706	Anoplophora glabripennis	Belle
EOG0912019S	APLA008616	Agrilus planipennis	Belle
EOG0912019S	TC013328	Tribolium castaneum	Belle
EOG0912019S	LDEC018194	Leptinotarsa decemlineata	Belle
EOG0912019S	YQE_06337	Dendroctonus ponderosae	Belle
EOG0912019X	OTAU002828	Onthophagus taurus	Putative uncharacterized protein
EOG0912019X	AGLA007618	Anoplophora glabripennis	Putative uncharacterized protein
EOG0912019X	APLA012400	Agrilus planipennis	Putative uncharacterized protein
EOG0912019X	TC004634	Tribolium castaneum	Putative uncharacterized protein
EOG0912019X	LDEC001525	Leptinotarsa decemlineata	Putative uncharacterized protein
EOG0912019X	YQE_09096	Dendroctonus ponderosae	Putative uncharacterized protein
EOG0912019Z	OTAU009877	Onthophagus taurus	T-complex protein 1 subunit alpha
EOG0912019Z	AGLA000042	Anoplophora glabripennis	T-complex protein 1 subunit alpha
EOG0912019Z	APLA005863	Agrilus planipennis	T-complex protein 1 subunit alpha
EOG0912019Z	TC000969	Tribolium castaneum	T-complex protein 1 subunit alpha
EOG0912019Z	LDEC019597	Leptinotarsa decemlineata	T-complex protein 1 subunit alpha
EOG0912019Z	YQE_10737	Dendroctonus ponderosae	T-complex protein 1 subunit alpha
EOG091201A1	OTAU009896	Onthophagus taurus	transferase activity
EOG091201A1	AGLA006670	Anoplophora glabripennis	transferase activity
EOG091201A1	APLA005521	Agrilus planipennis	transferase activity
EOG091201A1	TC032116	Tribolium castaneum	transferase activity
EOG091201A1	LDEC001606	Leptinotarsa decemlineata	transferase activity
EOG091201A1	YQE_10786	Dendroctonus ponderosae	transferase activity
EOG091201A2	OTAU000677	Onthophagus taurus	Putative uncharacterized protein
EOG091201A2	AGLA002597	Anoplophora glabripennis	Putative uncharacterized protein
EOG091201A2	APLA010420	Agrilus planipennis	Putative uncharacterized protein
EOG091201A2	TC010651	Tribolium castaneum	Putative uncharacterized protein
EOG091201A2	LDEC002009	Leptinotarsa decemlineata	Putative uncharacterized protein
EOG091201A2	YQE_08536	Dendroctonus ponderosae	Putative uncharacterized protein
EOG091201A4	OTAU009499	Onthophagus taurus	Putative uncharacterized protein
EOG091201A4	AGLA006753	Anoplophora glabripennis	Putative uncharacterized protein
EOG091201A4	APLA005891	Agrilus planipennis	Putative uncharacterized protein
EOG091201A4	TC014860	Tribolium castaneum	Putative uncharacterized protein
EOG091201A4	LDEC009957	Leptinotarsa decemlineata	Putative uncharacterized protein
EOG091201A4	YQE_10818	Dendroctonus ponderosae	Putative uncharacterized protein
EOG091201A5	OTAU004301	Onthophagus taurus	Putative uncharacterized protein
EOG091201A5	AGLA010378	Anoplophora glabripennis	Putative uncharacterized protein
EOG091201A5	APLA006777	Agrilus planipennis	Putative uncharacterized protein
EOG091201A5	TC014181	Tribolium castaneum	Putative uncharacterized protein
EOG091201A5	LDEC019791	Leptinotarsa decemlineata	Putative uncharacterized protein
EOG091201A5	YQE_11587	Dendroctonus ponderosae	Putative uncharacterized protein
EOG091201A6	OTAU001484	Onthophagus taurus	Putative uncharacterized protein
EOG091201A6	AGLA013539	Anoplophora glabripennis	Putative uncharacterized protein
EOG091201A6	APLA012012	Agrilus planipennis	Putative uncharacterized protein
EOG091201A6	TC006514	Tribolium castaneum	Putative uncharacterized protein
EOG091201A6	LDEC001544	Leptinotarsa decemlineata	Putative uncharacterized protein
EOG091201A6	YQE_09668	Dendroctonus ponderosae	Putative uncharacterized protein
EOG091201AC	OTAU009882	Onthophagus taurus	Putative uncharacterized protein
EOG091201AC	AGLA000143	Anoplophora glabripennis	Putative uncharacterized protein
EOG091201AC	APLA000142	Agrilus planipennis	Putative uncharacterized protein
EOG091201AC	TC000162	Tribolium castaneum	Putative uncharacterized protein
EOG091201AC	LDEC016692	Leptinotarsa decemlineata	Putative uncharacterized protein
EOG091201AC	YQE_10970	Dendroctonus ponderosae	Putative uncharacterized protein
EOG091201AD	OTAU007924	Onthophagus taurus	Putative uncharacterized protein
EOG091201AD	AGLA017235	Anoplophora glabripennis	Putative uncharacterized protein
EOG091201AD	APLA012606	Agrilus planipennis	Putative uncharacterized protein
EOG091201AD	TC012373	Tribolium castaneum	Putative uncharacterized protein
EOG091201AD	LDEC019832	Leptinotarsa decemlineata	Putative uncharacterized protein

EOG091201AD	YQE_01712	Dendroctonus ponderosae	Putative uncharacterized protein
EOG091201AE	OTAU016389	Onthophagus taurus	Putative uncharacterized protein
EOG091201AE	AGLA007344	Anoplophora glabripennis	Putative uncharacterized protein
EOG091201AE	APLA000646	Agrilus planipennis	Putative uncharacterized protein
EOG091201AE	TC015385	Tribolium castaneum	Putative uncharacterized protein
EOG091201AE	LDEC001934	Leptinotarsa decemlineata	Putative uncharacterized protein
EOG091201AE	YQE_05879	Dendroctonus ponderosae	Putative uncharacterized protein
EOG091201AG	OTAU000125	Onthophagus taurus	Putative uncharacterized protein
EOG091201AG	AGLA011120	Anoplophora glabripennis	Putative uncharacterized protein
EOG091201AG	APLA003085	Agrilus planipennis	Putative uncharacterized protein
EOG091201AG	TC014326	Tribolium castaneum	Putative uncharacterized protein
EOG091201AG	LDEC018086	Leptinotarsa decemlineata	Putative uncharacterized protein
EOG091201AG	YQE_07150	Dendroctonus ponderosae	Putative uncharacterized protein
EOG091201AH	OTAU012662	Onthophagus taurus	None
EOG091201AH	AGLA006869	Anoplophora glabripennis	None
EOG091201AH	APLA011340	Agrilus planipennis	None
EOG091201AH	TC032139	Tribolium castaneum	None
EOG091201AH	LDEC007434	Leptinotarsa decemlineata	None
EOG091201AH	YQE_08037	Dendroctonus ponderosae	None
EOG091201AK	OTAU014739	Onthophagus taurus	Putative uncharacterized protein
EOG091201AK	AGLA008563	Anoplophora glabripennis	Putative uncharacterized protein
EOG091201AK	APLA000537	Agrilus planipennis	Putative uncharacterized protein
EOG091201AK	TC014950	Tribolium castaneum	Putative uncharacterized protein
EOG091201AK	LDEC008022	Leptinotarsa decemlineata	Putative uncharacterized protein
EOG091201AK	YQE_10822	Dendroctonus ponderosae	Putative uncharacterized protein
EOG091201AL	OTAU005692	Onthophagus taurus	Putative uncharacterized protein
EOG091201AL	AGLA015882	Anoplophora glabripennis	Putative uncharacterized protein
EOG091201AL	APLA014250	Agrilus planipennis	Putative uncharacterized protein
EOG091201AL	TC008905	Tribolium castaneum	Putative uncharacterized protein
EOG091201AL	LDEC015989	Leptinotarsa decemlineata	Putative uncharacterized protein
EOG091201AL	YQE_00069	Dendroctonus ponderosae	Putative uncharacterized protein
EOG091201AN	OTAU002285	Onthophagus taurus	Putative uncharacterized protein
EOG091201AN	AGLA014408	Anoplophora glabripennis	Putative uncharacterized protein
EOG091201AN	APLA004189	Agrilus planipennis	Putative uncharacterized protein
EOG091201AN	TC008245	Tribolium castaneum	Putative uncharacterized protein
EOG091201AN	LDEC008312	Leptinotarsa decemlineata	Putative uncharacterized protein
EOG091201AN	YQE_05220	Dendroctonus ponderosae	Putative uncharacterized protein
EOG091201AO	OTAU004969	Onthophagus taurus	Putative uncharacterized protein
EOG091201AO	AGLA002534	Anoplophora glabripennis	Putative uncharacterized protein
EOG091201AO	APLA010862	Agrilus planipennis	Putative uncharacterized protein
EOG091201AO	TC011193	Tribolium castaneum	Putative uncharacterized protein
EOG091201AO	LDEC001796	Leptinotarsa decemlineata	Putative uncharacterized protein
EOG091201AO	YQE_04920	Dendroctonus ponderosae	Putative uncharacterized protein
EOG091201AP	OTAU012698	Onthophagus taurus	None
EOG091201AP	AGLA004689	Anoplophora glabripennis	None
EOG091201AP	APLA005391	Agrilus planipennis	None
EOG091201AP	TC031096	Tribolium castaneum	None
EOG091201AP	LDEC002428	Leptinotarsa decemlineata	None
EOG091201AP	YQE_06854	Dendroctonus ponderosae	None
EOG091201AQ	OTAU008309	Onthophagus taurus	helicase activity
EOG091201AQ	AGLA015579	Anoplophora glabripennis	helicase activity
EOG091201AQ	APLA000521	Agrilus planipennis	helicase activity
EOG091201AQ	TC033312	Tribolium castaneum	helicase activity
EOG091201AQ	LDEC008816	Leptinotarsa decemlineata	helicase activity
EOG091201AQ	YQE_02137	Dendroctonus ponderosae	helicase activity
EOG091201AS	OTAU008641	Onthophagus taurus	Knickkopf
EOG091201AS	AGLA002603	Anoplophora glabripennis	Knickkopf
EOG091201AS	APLA002326	Agrilus planipennis	Knickkopf
EOG091201AS	TC010653	Tribolium castaneum	Knickkopf
EOG091201AS	LDEC001976	Leptinotarsa decemlineata	Knickkopf
EOG091201AS	YQE_08297	Dendroctonus ponderosae	Knickkopf
EOG091201AT	OTAU002412	Onthophagus taurus	Putative uncharacterized protein
EOG091201AT	AGLA002054	Anoplophora glabripennis	Putative uncharacterized protein
EOG091201AT	APLA009142	Agrilus planipennis	Putative uncharacterized protein
EOG091201AT	TC004004	Tribolium castaneum	Putative uncharacterized protein
EOG091201AT	LDEC004961	Leptinotarsa decemlineata	Putative uncharacterized protein
EOG091201AT	YQE_03672	Dendroctonus ponderosae	Putative uncharacterized protein

EOG091201AV	OTAU011347	Onthophagus taurus	Putative uncharacterized protein
EOG091201AV	AGLA004248	Anoplophora glabripennis	Putative uncharacterized protein
EOG091201AV	APLA000823	Agrilus planipennis	Putative uncharacterized protein
EOG091201AV	TC003257	Tribolium castaneum	Putative uncharacterized protein
EOG091201AV	LDEC008223	Leptinotarsa decemlineata	Putative uncharacterized protein
EOG091201AV	YQE_03728	Dendroctonus ponderosae	Putative uncharacterized protein
EOG091201AW	OTAU013224	Onthophagus taurus	Putative uncharacterized protein
EOG091201AW	AGLA019351	Anoplophora glabripennis	Putative uncharacterized protein
EOG091201AW	APLA003191	Agrilus planipennis	Putative uncharacterized protein
EOG091201AW	TC016270	Tribolium castaneum	Putative uncharacterized protein
EOG091201AW	LDEC000567	Leptinotarsa decemlineata	Putative uncharacterized protein
EOG091201AW	YQE_12980	Dendroctonus ponderosae	Putative uncharacterized protein
EOG091201AZ	OTAU004253	Onthophagus taurus	Putative uncharacterized protein
EOG091201AZ	AGLA014961	Anoplophora glabripennis	Putative uncharacterized protein
EOG091201AZ	APLA011319	Agrilus planipennis	Putative uncharacterized protein
EOG091201AZ	TC012903	Tribolium castaneum	Putative uncharacterized protein
EOG091201AZ	LDEC009502	Leptinotarsa decemlineata	Putative uncharacterized protein
EOG091201AZ	YQE_11378	Dendroctonus ponderosae	Putative uncharacterized protein
EOG091201B2	OTAU001684	Onthophagus taurus	Putative uncharacterized protein
EOG091201B2	AGLA020596	Anoplophora glabripennis	Putative uncharacterized protein
EOG091201B2	APLA000373	Agrilus planipennis	Putative uncharacterized protein
EOG091201B2	TC005972	Tribolium castaneum	Putative uncharacterized protein
EOG091201B2	LDEC017312	Leptinotarsa decemlineata	Putative uncharacterized protein
EOG091201B2	YQE_06945	Dendroctonus ponderosae	Putative uncharacterized protein
EOG091201B6	OTAU013214	Onthophagus taurus	"Translation factor GUF1 homolog, mitochondrial "
EOG091201B6	AGLA010395	Anoplophora glabripennis	"Translation factor GUF1 homolog, mitochondrial "
EOG091201B6	APLA009236	Agrilus planipennis	"Translation factor GUF1 homolog, mitochondrial "
EOG091201B6	TC007205	Tribolium castaneum	"Translation factor GUF1 homolog, mitochondrial "
EOG091201B6	LDEC003965	Leptinotarsa decemlineata	"Translation factor GUF1 homolog, mitochondrial "
EOG091201B6	YQE_10395	Dendroctonus ponderosae	"Translation factor GUF1 homolog, mitochondrial "
EOG091201B8	OTAU010262	Onthophagus taurus	Putative uncharacterized protein
EOG091201B8	AGLA014716	Anoplophora glabripennis	Putative uncharacterized protein
EOG091201B8	APLA004562	Agrilus planipennis	Putative uncharacterized protein
EOG091201B8	TC009240	Tribolium castaneum	Putative uncharacterized protein
EOG091201B8	LDEC012001	Leptinotarsa decemlineata	Putative uncharacterized protein
EOG091201B8	YQE_12508	Dendroctonus ponderosae	Putative uncharacterized protein
EOG091201B9	OTAU008730	Onthophagus taurus	Putative uncharacterized protein
EOG091201B9	AGLA014948	Anoplophora glabripennis	Putative uncharacterized protein
EOG091201B9	APLA013360	Agrilus planipennis	Putative uncharacterized protein
EOG091201B9	TC002976	Tribolium castaneum	Putative uncharacterized protein
EOG091201B9	LDEC002041	Leptinotarsa decemlineata	Putative uncharacterized protein
EOG091201B9	YQE_10271	Dendroctonus ponderosae	Putative uncharacterized protein
EOG091201BA	OTAU005751	Onthophagus taurus	Putative uncharacterized protein
EOG091201BA	AGLA000550	Anoplophora glabripennis	Putative uncharacterized protein
EOG091201BA	APLA001478	Agrilus planipennis	Putative uncharacterized protein
EOG091201BA	TC000268	Tribolium castaneum	Putative uncharacterized protein
EOG091201BA	LDEC003720	Leptinotarsa decemlineata	Putative uncharacterized protein
EOG091201BA	YQE_09962	Dendroctonus ponderosae	Putative uncharacterized protein
EOG091201BB	OTAU003923	Onthophagus taurus	None
EOG091201BB	AGLA004397	Anoplophora glabripennis	None
EOG091201BB	APLA003266	Agrilus planipennis	None
EOG091201BB	TC031249	Tribolium castaneum	None
EOG091201BB	LDEC014883	Leptinotarsa decemlineata	None
EOG091201BB	YQE_12988	Dendroctonus ponderosae	None
EOG091201BC	OTAU000950	Onthophagus taurus	metallopeptidase activity
EOG091201BC	AGLA008478	Anoplophora glabripennis	metallopeptidase activity
EOG091201BC	APLA003155	Agrilus planipennis	metallopeptidase activity
EOG091201BC	TC030950	Tribolium castaneum	metallopeptidase activity
EOG091201BC	LDEC005528	Leptinotarsa decemlineata	metallopeptidase activity
EOG091201BC	YQE_09622	Dendroctonus ponderosae	metallopeptidase activity
EOG091201BF	OTAU010307	Onthophagus taurus	Putative uncharacterized protein
EOG091201BF	AGLA002777	Anoplophora glabripennis	Putative uncharacterized protein
EOG091201BF	APLA004620	Agrilus planipennis	Putative uncharacterized protein
EOG091201BF	TC009836	Tribolium castaneum	Putative uncharacterized protein
EOG091201BF	LDEC006449	Leptinotarsa decemlineata	Putative uncharacterized protein
EOG091201BF	YQE_08867	Dendroctonus ponderosae	Putative uncharacterized protein
EOG091201BI	OTAU012639	Onthophagus taurus	Putative uncharacterized protein

EOG091201BI	AGLA010027	Anoplophora glabripennis	Putative uncharacterized protein
EOG091201BI	APLA002291	Agrilus planipennis	Putative uncharacterized protein
EOG091201BI	TC008292	Tribolium castaneum	Putative uncharacterized protein
EOG091201BI	LDEC006848	Leptinotarsa decemlineata	Putative uncharacterized protein
EOG091201BI	YQE_11896	Dendroctonus ponderosae	Putative uncharacterized protein
EOG091201BJ	OTAU001150	Onthophagus taurus	Putative uncharacterized protein
EOG091201BJ	AGLA002569	Anoplophora glabripennis	Putative uncharacterized protein
EOG091201BJ	APLA001959	Agrilus planipennis	Putative uncharacterized protein
EOG091201BJ	TC002083	Tribolium castaneum	Putative uncharacterized protein
EOG091201BJ	LDEC008074	Leptinotarsa decemlineata	Putative uncharacterized protein
EOG091201BJ	YQE_04997	Dendroctonus ponderosae	Putative uncharacterized protein
EOG091201BN	OTAU001460	Onthophagus taurus	Putative uncharacterized protein
EOG091201BN	AGLA013536	Anoplophora glabripennis	Putative uncharacterized protein
EOG091201BN	APLA013963	Agrilus planipennis	Putative uncharacterized protein
EOG091201BN	TC015781	Tribolium castaneum	Putative uncharacterized protein
EOG091201BN	LDEC009970	Leptinotarsa decemlineata	Putative uncharacterized protein
EOG091201BN	YQE_09273	Dendroctonus ponderosae	Putative uncharacterized protein
EOG091201BT	OTAU011869	Onthophagus taurus	None
EOG091201BT	AGLA000023	Anoplophora glabripennis	None
EOG091201BT	APLA004393	Agrilus planipennis	None
EOG091201BT	TC034564	Tribolium castaneum	None
EOG091201BT	LDEC019430	Leptinotarsa decemlineata	None
EOG091201BT	YQE_07082	Dendroctonus ponderosae	None
EOG091201BU	OTAU001284	Onthophagus taurus	Putative uncharacterized protein
EOG091201BU	AGLA021640	Anoplophora glabripennis	Putative uncharacterized protein
EOG091201BU	APLA015312	Agrilus planipennis	Putative uncharacterized protein
EOG091201BU	TC004143	Tribolium castaneum	Putative uncharacterized protein
EOG091201BU	LDEC016070	Leptinotarsa decemlineata	Putative uncharacterized protein
EOG091201BU	YQE_02514	Dendroctonus ponderosae	Putative uncharacterized protein
EOG091201BV	OTAU005116	Onthophagus taurus	Putative uncharacterized protein
EOG091201BV	AGLA011993	Anoplophora glabripennis	Putative uncharacterized protein
EOG091201BV	APLA000270	Agrilus planipennis	Putative uncharacterized protein
EOG091201BV	TC002294	Tribolium castaneum	Putative uncharacterized protein
EOG091201BV	LDEC012936	Leptinotarsa decemlineata	Putative uncharacterized protein
EOG091201BV	YQE_11708	Dendroctonus ponderosae	Putative uncharacterized protein
EOG091201BW	OTAU015640	Onthophagus taurus	Putative uncharacterized protein
EOG091201BW	AGLA016351	Anoplophora glabripennis	Putative uncharacterized protein
EOG091201BW	APLA012304	Agrilus planipennis	Putative uncharacterized protein
EOG091201BW	TC005733	Tribolium castaneum	Putative uncharacterized protein
EOG091201BW	LDEC006319	Leptinotarsa decemlineata	Putative uncharacterized protein
EOG091201BW	YQE_06960	Dendroctonus ponderosae	Putative uncharacterized protein
EOG091201BY	OTAU000730	Onthophagus taurus	Putative uncharacterized protein
EOG091201BY	AGLA019320	Anoplophora glabripennis	Putative uncharacterized protein
EOG091201BY	APLA002638	Agrilus planipennis	Putative uncharacterized protein
EOG091201BY	TC012191	Tribolium castaneum	Putative uncharacterized protein
EOG091201BY	LDEC018077	Leptinotarsa decemlineata	Putative uncharacterized protein
EOG091201BY	YQE_12846	Dendroctonus ponderosae	Putative uncharacterized protein
EOG091201C1	OTAU008025	Onthophagus taurus	Putative uncharacterized protein
EOG091201C1	AGLA005966	Anoplophora glabripennis	Putative uncharacterized protein
EOG091201C1	APLA004486	Agrilus planipennis	Putative uncharacterized protein
EOG091201C1	TC006523	Tribolium castaneum	Putative uncharacterized protein
EOG091201C1	LDEC011213	Leptinotarsa decemlineata	Putative uncharacterized protein
EOG091201C1	YQE_07425	Dendroctonus ponderosae	Putative uncharacterized protein
EOG091201C2	OTAU013232	Onthophagus taurus	Putative uncharacterized protein
EOG091201C2	AGLA009781	Anoplophora glabripennis	Putative uncharacterized protein
EOG091201C2	APLA003234	Agrilus planipennis	Putative uncharacterized protein
EOG091201C2	TC010557	Tribolium castaneum	Putative uncharacterized protein
EOG091201C2	LDEC003829	Leptinotarsa decemlineata	Putative uncharacterized protein
EOG091201C2	YQE_12913	Dendroctonus ponderosae	Putative uncharacterized protein
EOG091201C5	OTAU001905	Onthophagus taurus	Putative uncharacterized protein
EOG091201C5	AGLA020951	Anoplophora glabripennis	Putative uncharacterized protein
EOG091201C5	APLA012088	Agrilus planipennis	Putative uncharacterized protein
EOG091201C5	TC006173	Tribolium castaneum	Putative uncharacterized protein
EOG091201C5	LDEC004910	Leptinotarsa decemlineata	Putative uncharacterized protein
EOG091201C5	YQE_12190	Dendroctonus ponderosae	Putative uncharacterized protein
EOG091201C6	OTAU008095	Onthophagus taurus	Putative uncharacterized protein
EOG091201C6	AGLA010747	Anoplophora glabripennis	Putative uncharacterized protein

EOG091201C6	APLA006623	Agrilus planipennis	Putative uncharacterized protein
EOG091201C6	TC030757	Tribolium castaneum	Putative uncharacterized protein
EOG091201C6	LDEC016954	Leptinotarsa decemlineata	Putative uncharacterized protein
EOG091201C6	YQE_11111	Dendroctonus ponderosae	Putative uncharacterized protein
EOG091201C7	OTAU009064	Onthophagus taurus	Putative uncharacterized protein
EOG091201C7	AGLA011520	Anoplophora glabripennis	Putative uncharacterized protein
EOG091201C7	APLA006201	Agrilus planipennis	Putative uncharacterized protein
EOG091201C7	TC013543	Tribolium castaneum	Putative uncharacterized protein
EOG091201C7	LDEC011415	Leptinotarsa decemlineata	Putative uncharacterized protein
EOG091201C7	YQE_02042	Dendroctonus ponderosae	Putative uncharacterized protein
EOG091201CA	OTAU014607	Onthophagus taurus	Putative uncharacterized protein
EOG091201CA	AGLA013440	Anoplophora glabripennis	Putative uncharacterized protein
EOG091201CA	APLA014592	Agrilus planipennis	Putative uncharacterized protein
EOG091201CA	TC011315	Tribolium castaneum	Putative uncharacterized protein
EOG091201CA	LDEC006039	Leptinotarsa decemlineata	Putative uncharacterized protein
EOG091201CA	YQE_11768	Dendroctonus ponderosae	Putative uncharacterized protein
EOG091201CE	OTAU009780	Onthophagus taurus	Putative uncharacterized protein
EOG091201CE	AGLA002082	Anoplophora glabripennis	Putative uncharacterized protein
EOG091201CE	APLA008130	Agrilus planipennis	Putative uncharacterized protein
EOG091201CE	TC002338	Tribolium castaneum	Putative uncharacterized protein
EOG091201CE	LDEC009192	Leptinotarsa decemlineata	Putative uncharacterized protein
EOG091201CE	YQE_06424	Dendroctonus ponderosae	Putative uncharacterized protein
EOG091201CF	OTAU000252	Onthophagus taurus	Polyhomeotic
EOG091201CF	AGLA010824	Anoplophora glabripennis	Polyhomeotic
EOG091201CF	APLA008078	Agrilus planipennis	Polyhomeotic
EOG091201CF	TC003752	Tribolium castaneum	Polyhomeotic
EOG091201CF	LDEC012135	Leptinotarsa decemlineata	Polyhomeotic
EOG091201CF	YQE_06072	Dendroctonus ponderosae	Polyhomeotic
EOG091201CH	OTAU001237	Onthophagus taurus	Putative uncharacterized protein
EOG091201CH	AGLA002174	Anoplophora glabripennis	Putative uncharacterized protein
EOG091201CH	APLA003697	Agrilus planipennis	Putative uncharacterized protein
EOG091201CH	TC002335	Tribolium castaneum	Putative uncharacterized protein
EOG091201CH	LDEC001522	Leptinotarsa decemlineata	Putative uncharacterized protein
EOG091201CH	YQE_09285	Dendroctonus ponderosae	Putative uncharacterized protein
EOG091201CJ	OTAU005182	Onthophagus taurus	Putative uncharacterized protein
EOG091201CJ	AGLA018202	Anoplophora glabripennis	Putative uncharacterized protein
EOG091201CJ	APLA000124	Agrilus planipennis	Putative uncharacterized protein
EOG091201CJ	TC003554	Tribolium castaneum	Putative uncharacterized protein
EOG091201CJ	LDEC016424	Leptinotarsa decemlineata	Putative uncharacterized protein
EOG091201CJ	YQE_04962	Dendroctonus ponderosae	Putative uncharacterized protein
EOG091201CK	OTAU002767	Onthophagus taurus	Putative uncharacterized protein
EOG091201CK	AGLA000303	Anoplophora glabripennis	Putative uncharacterized protein
EOG091201CK	APLA015139	Agrilus planipennis	Putative uncharacterized protein
EOG091201CK	TC001440	Tribolium castaneum	Putative uncharacterized protein
EOG091201CK	LDEC003757	Leptinotarsa decemlineata	Putative uncharacterized protein
EOG091201CK	YQE_02523	Dendroctonus ponderosae	Putative uncharacterized protein
EOG091201CL	OTAU007609	Onthophagus taurus	Putative uncharacterized protein
EOG091201CL	AGLA002019	Anoplophora glabripennis	Putative uncharacterized protein
EOG091201CL	APLA002828	Agrilus planipennis	Putative uncharacterized protein
EOG091201CL	TC006849	Tribolium castaneum	Putative uncharacterized protein
EOG091201CL	LDEC008563	Leptinotarsa decemlineata	Putative uncharacterized protein
EOG091201CL	YQE_10411	Dendroctonus ponderosae	Putative uncharacterized protein
EOG091201CN	OTAU000385	Onthophagus taurus	Putative uncharacterized protein
EOG091201CN	AGLA011669	Anoplophora glabripennis	Putative uncharacterized protein
EOG091201CN	APLA001097	Agrilus planipennis	Putative uncharacterized protein
EOG091201CN	TC012726	Tribolium castaneum	Putative uncharacterized protein
EOG091201CN	LDEC007750	Leptinotarsa decemlineata	Putative uncharacterized protein
EOG091201CN	YQE_07601	Dendroctonus ponderosae	Putative uncharacterized protein
EOG091201CP	OTAU002237	Onthophagus taurus	Serine protease H129
EOG091201CP	AGLA006856	Anoplophora glabripennis	Serine protease H129
EOG091201CP	APLA002344	Agrilus planipennis	Serine protease H129
EOG091201CP	TC012390	Tribolium castaneum	Serine protease H129
EOG091201CP	LDEC011869	Leptinotarsa decemlineata	Serine protease H129
EOG091201CP	YQE_02685	Dendroctonus ponderosae	Serine protease H129
EOG091201CQ	OTAU006187	Onthophagus taurus	None
EOG091201CQ	AGLA005806	Anoplophora glabripennis	None
EOG091201CQ	APLA014396	Agrilus planipennis	None

EOG091201CQ	TC031214	Tribolium castaneum	None
EOG091201CQ	LDEC010723	Leptinotarsa decemlineata	None
EOG091201CQ	YQE_12998	Dendroctonus ponderosae	None
EOG091201CV	OTAU002129	Onthophagus taurus	Putative uncharacterized protein
EOG091201CV	AGLA011729	Anoplophora glabripennis	Putative uncharacterized protein
EOG091201CV	APLA003274	Agrilus planipennis	Putative uncharacterized protein
EOG091201CV	TC012749	Tribolium castaneum	Putative uncharacterized protein
EOG091201CV	LDEC007630	Leptinotarsa decemlineata	Putative uncharacterized protein
EOG091201CV	YQE_10098	Dendroctonus ponderosae	Putative uncharacterized protein
EOG091201CW	OTAU001480	Onthophagus taurus	Putative uncharacterized protein
EOG091201CW	AGLA000312	Anoplophora glabripennis	Putative uncharacterized protein
EOG091201CW	APLA006355	Agrilus planipennis	Putative uncharacterized protein
EOG091201CW	TC004777	Tribolium castaneum	Putative uncharacterized protein
EOG091201CW	LDEC019936	Leptinotarsa decemlineata	Putative uncharacterized protein
EOG091201CW	YQE_06731	Dendroctonus ponderosae	Putative uncharacterized protein
EOG091201CX	OTAU007565	Onthophagus taurus	Putative uncharacterized protein
EOG091201CX	AGLA021892	Anoplophora glabripennis	Putative uncharacterized protein
EOG091201CX	APLA004851	Agrilus planipennis	Putative uncharacterized protein
EOG091201CX	TC005070	Tribolium castaneum	Putative uncharacterized protein
EOG091201CX	LDEC014003	Leptinotarsa decemlineata	Putative uncharacterized protein
EOG091201CX	YQE_12254	Dendroctonus ponderosae	Putative uncharacterized protein
EOG091201D1	OTAU014681	Onthophagus taurus	Putative uncharacterized protein
EOG091201D1	AGLA004049	Anoplophora glabripennis	Putative uncharacterized protein
EOG091201D1	APLA008103	Agrilus planipennis	Putative uncharacterized protein
EOG091201D1	TC009105	Tribolium castaneum	Putative uncharacterized protein
EOG091201D1	LDEC015191	Leptinotarsa decemlineata	Putative uncharacterized protein
EOG091201D1	YQE_10296	Dendroctonus ponderosae	Putative uncharacterized protein
EOG091201D4	OTAU005369	Onthophagus taurus	Putative uncharacterized protein
EOG091201D4	AGLA018406	Anoplophora glabripennis	Putative uncharacterized protein
EOG091201D4	APLA002090	Agrilus planipennis	Putative uncharacterized protein
EOG091201D4	TC003211	Tribolium castaneum	Putative uncharacterized protein
EOG091201D4	LDEC008608	Leptinotarsa decemlineata	Putative uncharacterized protein
EOG091201D4	YQE_05485	Dendroctonus ponderosae	Putative uncharacterized protein
EOG091201D6	OTAU009897	Onthophagus taurus	Putative uncharacterized protein
EOG091201D6	AGLA006669	Anoplophora glabripennis	Putative uncharacterized protein
EOG091201D6	APLA005523	Agrilus planipennis	Putative uncharacterized protein
EOG091201D6	TC000945	Tribolium castaneum	Putative uncharacterized protein
EOG091201D6	LDEC001602	Leptinotarsa decemlineata	Putative uncharacterized protein
EOG091201D6	YQE_10787	Dendroctonus ponderosae	Putative uncharacterized protein
EOG091201D7	OTAU005444	Onthophagus taurus	None
EOG091201D7	AGLA002605	Anoplophora glabripennis	None
EOG091201D7	APLA006365	Agrilus planipennis	None
EOG091201D7	TC031577	Tribolium castaneum	None
EOG091201D7	LDEC001979	Leptinotarsa decemlineata	None
EOG091201D7	YQE_10001	Dendroctonus ponderosae	None
EOG091201D8	OTAU015484	Onthophagus taurus	Putative uncharacterized protein
EOG091201D8	AGLA021003	Anoplophora glabripennis	Putative uncharacterized protein
EOG091201D8	APLA009663	Agrilus planipennis	Putative uncharacterized protein
EOG091201D8	TC006332	Tribolium castaneum	Putative uncharacterized protein
EOG091201D8	LDEC004982	Leptinotarsa decemlineata	Putative uncharacterized protein
EOG091201D8	YQE_12073	Dendroctonus ponderosae	Putative uncharacterized protein
EOG091201DA	OTAU016858	Onthophagus taurus	Putative uncharacterized protein
EOG091201DA	AGLA010434	Anoplophora glabripennis	Putative uncharacterized protein
EOG091201DA	APLA005574	Agrilus planipennis	Putative uncharacterized protein
EOG091201DA	TC015823	Tribolium castaneum	Putative uncharacterized protein
EOG091201DA	LDEC013168	Leptinotarsa decemlineata	Putative uncharacterized protein
EOG091201DA	YQE_02144	Dendroctonus ponderosae	Putative uncharacterized protein
EOG091201DB	OTAU011866	Onthophagus taurus	Putative uncharacterized protein
EOG091201DB	AGLA000125	Anoplophora glabripennis	Putative uncharacterized protein
EOG091201DB	APLA009349	Agrilus planipennis	Putative uncharacterized protein
EOG091201DB	TC000229	Tribolium castaneum	Putative uncharacterized protein
EOG091201DB	LDEC001622	Leptinotarsa decemlineata	Putative uncharacterized protein
EOG091201DB	YQE_07061	Dendroctonus ponderosae	Putative uncharacterized protein
EOG091201DC	OTAU010526	Onthophagus taurus	Uridine kinase
EOG091201DC	AGLA009164	Anoplophora glabripennis	Uridine kinase
EOG091201DC	APLA002052	Agrilus planipennis	Uridine kinase
EOG091201DC	TC011541	Tribolium castaneum	Uridine kinase

EOG091201DC	LDEC010264	Leptinotarsa decemlineata	Uridine kinase
EOG091201DC	YQE_10232	Dendroctonus ponderosae	Uridine kinase
EOG091201DD	OTAU006533	Onthophagus taurus	Putative uncharacterized protein
EOG091201DD	AGLA009125	Anoplophora glabripennis	Putative uncharacterized protein
EOG091201DD	APLA014638	Agrilus planipennis	Putative uncharacterized protein
EOG091201DD	TC002297	Tribolium castaneum	Putative uncharacterized protein
EOG091201DD	LDEC005435	Leptinotarsa decemlineata	Putative uncharacterized protein
EOG091201DD	YQE_11084	Dendroctonus ponderosae	Putative uncharacterized protein
EOG091201DE	OTAU005057	Onthophagus taurus	Putative uncharacterized protein
EOG091201DE	AGLA017732	Anoplophora glabripennis	Putative uncharacterized protein
EOG091201DE	APLA011047	Agrilus planipennis	Putative uncharacterized protein
EOG091201DE	TC030652	Tribolium castaneum	Putative uncharacterized protein
EOG091201DE	LDEC011693	Leptinotarsa decemlineata	Putative uncharacterized protein
EOG091201DE	YQE_12028	Dendroctonus ponderosae	Putative uncharacterized protein
EOG091201DF	OTAU013133	Onthophagus taurus	Putative uncharacterized protein
EOG091201DF	AGLA014729	Anoplophora glabripennis	Putative uncharacterized protein
EOG091201DF	APLA005069	Agrilus planipennis	Putative uncharacterized protein
EOG091201DF	TC006209	Tribolium castaneum	Putative uncharacterized protein
EOG091201DF	LDEC010114	Leptinotarsa decemlineata	Putative uncharacterized protein
EOG091201DF	YQE_05638	Dendroctonus ponderosae	Putative uncharacterized protein
EOG091201DG	OTAU003772	Onthophagus taurus	Putative uncharacterized protein
EOG091201DG	AGLA005995	Anoplophora glabripennis	Putative uncharacterized protein
EOG091201DG	APLA004877	Agrilus planipennis	Putative uncharacterized protein
EOG091201DG	TC014777	Tribolium castaneum	Putative uncharacterized protein
EOG091201DG	LDEC011667	Leptinotarsa decemlineata	Putative uncharacterized protein
EOG091201DG	YQE_08238	Dendroctonus ponderosae	Putative uncharacterized protein
EOG091201DI	OTAU006352	Onthophagus taurus	Glucose-6-phosphate isomerase
EOG091201DI	AGLA019362	Anoplophora glabripennis	Glucose-6-phosphate isomerase
EOG091201DI	APLA014015	Agrilus planipennis	Glucose-6-phosphate isomerase
EOG091201DI	TC030755	Tribolium castaneum	Glucose-6-phosphate isomerase
EOG091201DI	LDEC010650	Leptinotarsa decemlineata	Glucose-6-phosphate isomerase
EOG091201DI	YQE_11812	Dendroctonus ponderosae	Glucose-6-phosphate isomerase
EOG091201DK	OTAU011872	Onthophagus taurus	None
EOG091201DK	AGLA002421	Anoplophora glabripennis	None
EOG091201DK	APLA013380	Agrilus planipennis	None
EOG091201DK	TC031367	Tribolium castaneum	None
EOG091201DK	LDEC009283	Leptinotarsa decemlineata	None
EOG091201DK	YQE_05468	Dendroctonus ponderosae	None
EOG091201DN	OTAU001773	Onthophagus taurus	Putative uncharacterized protein
EOG091201DN	AGLA018472	Anoplophora glabripennis	Putative uncharacterized protein
EOG091201DN	APLA011058	Agrilus planipennis	Putative uncharacterized protein
EOG091201DN	TC005718	Tribolium castaneum	Putative uncharacterized protein
EOG091201DN	LDEC006765	Leptinotarsa decemlineata	Putative uncharacterized protein
EOG091201DN	YQE_12794	Dendroctonus ponderosae	Putative uncharacterized protein
EOG091201DO	OTAU007729	Onthophagus taurus	Putative uncharacterized protein
EOG091201DO	AGLA020734	Anoplophora glabripennis	Putative uncharacterized protein
EOG091201DO	APLA004806	Agrilus planipennis	Putative uncharacterized protein
EOG091201DO	TC013924	Tribolium castaneum	Putative uncharacterized protein
EOG091201DO	LDEC005959	Leptinotarsa decemlineata	Putative uncharacterized protein
EOG091201DO	YQE_10455	Dendroctonus ponderosae	Putative uncharacterized protein
EOG091201DR	OTAU006068	Onthophagus taurus	Putative uncharacterized protein
EOG091201DR	AGLA000785	Anoplophora glabripennis	Putative uncharacterized protein
EOG091201DR	APLA002191	Agrilus planipennis	Putative uncharacterized protein
EOG091201DR	TC008100	Tribolium castaneum	Putative uncharacterized protein
EOG091201DR	LDEC000713	Leptinotarsa decemlineata	Putative uncharacterized protein
EOG091201DR	YQE_04485	Dendroctonus ponderosae	Putative uncharacterized protein
EOG091201DU	OTAU002766	Onthophagus taurus	None
EOG091201DU	AGLA000304	Anoplophora glabripennis	None
EOG091201DU	APLA003520	Agrilus planipennis	None
EOG091201DU	TC031519	Tribolium castaneum	None
EOG091201DU	LDEC003756	Leptinotarsa decemlineata	None
EOG091201DU	YQE_02519	Dendroctonus ponderosae	None
EOG091201DX	OTAU013057	Onthophagus taurus	Supernumerary limbs
EOG091201DX	AGLA001321	Anoplophora glabripennis	Supernumerary limbs
EOG091201DX	APLA002934	Agrilus planipennis	Supernumerary limbs
EOG091201DX	TC001086	Tribolium castaneum	Supernumerary limbs
EOG091201DX	LDEC012373	Leptinotarsa decemlineata	Supernumerary limbs

EOG091201DX	YQE_02245	Dendroctonus ponderosae	Supernumerary limbs
EOG091201DZ	OTAU003444	Onthophagus taurus	Putative uncharacterized protein
EOG091201DZ	AGLA001963	Anoplophora glabripennis	Putative uncharacterized protein
EOG091201DZ	APLA013276	Agrilus planipennis	Putative uncharacterized protein
EOG091201DZ	TC012032	Tribolium castaneum	Putative uncharacterized protein
EOG091201DZ	LDEC015633	Leptinotarsa decemlineata	Putative uncharacterized protein
EOG091201DZ	YQE_11955	Dendroctonus ponderosae	Putative uncharacterized protein
EOG091201E0	OTAU002163	Onthophagus taurus	Putative uncharacterized protein
EOG091201E0	AGLA013778	Anoplophora glabripennis	Putative uncharacterized protein
EOG091201E0	APLA013192	Agrilus planipennis	Putative uncharacterized protein
EOG091201E0	TC003092	Tribolium castaneum	Putative uncharacterized protein
EOG091201E0	LDEC013031	Leptinotarsa decemlineata	Putative uncharacterized protein
EOG091201E0	YQE_12276	Dendroctonus ponderosae	Putative uncharacterized protein
EOG091201E6	OTAU009155	Onthophagus taurus	SNF1A/AMP-activated protein kinase
EOG091201E6	AGLA017927	Anoplophora glabripennis	SNF1A/AMP-activated protein kinase
EOG091201E6	APLA010535	Agrilus planipennis	SNF1A/AMP-activated protein kinase
EOG091201E6	TC008646	Tribolium castaneum	SNF1A/AMP-activated protein kinase
EOG091201E6	LDEC015368	Leptinotarsa decemlineata	SNF1A/AMP-activated protein kinase
EOG091201E6	YQE_12628	Dendroctonus ponderosae	SNF1A/AMP-activated protein kinase
EOG091201E8	OTAU002992	Onthophagus taurus	Putative uncharacterized protein
EOG091201E8	AGLA010356	Anoplophora glabripennis	Putative uncharacterized protein
EOG091201E8	APLA014483	Agrilus planipennis	Putative uncharacterized protein
EOG091201E8	TC010123	Tribolium castaneum	Putative uncharacterized protein
EOG091201E8	LDEC001327	Leptinotarsa decemlineata	Putative uncharacterized protein
EOG091201E8	YQE_01563	Dendroctonus ponderosae	Putative uncharacterized protein
EOG091201E9	OTAU004310	Onthophagus taurus	Putative uncharacterized protein
EOG091201E9	AGLA015676	Anoplophora glabripennis	Putative uncharacterized protein
EOG091201E9	APLA006395	Agrilus planipennis	Putative uncharacterized protein
EOG091201E9	TC004153	Tribolium castaneum	Putative uncharacterized protein
EOG091201E9	LDEC018072	Leptinotarsa decemlineata	Putative uncharacterized protein
EOG091201E9	YQE_02496	Dendroctonus ponderosae	Putative uncharacterized protein
EOG091201EA	OTAU002954	Onthophagus taurus	Putative uncharacterized protein
EOG091201EA	AGLA003844	Anoplophora glabripennis	Putative uncharacterized protein
EOG091201EA	APLA003480	Agrilus planipennis	Putative uncharacterized protein
EOG091201EA	TC008818	Tribolium castaneum	Putative uncharacterized protein
EOG091201EA	LDEC010660	Leptinotarsa decemlineata	Putative uncharacterized protein
EOG091201EA	YQE_05835	Dendroctonus ponderosae	Putative uncharacterized protein
EOG091201EB	OTAU006204	Onthophagus taurus	None
EOG091201EB	AGLA004414	Anoplophora glabripennis	None
EOG091201EB	APLA002517	Agrilus planipennis	None
EOG091201EB	TC034874	Tribolium castaneum	None
EOG091201EB	LDEC005120	Leptinotarsa decemlineata	None
EOG091201EB	YQE_07955	Dendroctonus ponderosae	None
EOG091201ED	OTAU006145	Onthophagus taurus	Putative uncharacterized protein
EOG091201ED	AGLA008609	Anoplophora glabripennis	Putative uncharacterized protein
EOG091201ED	APLA001985	Agrilus planipennis	Putative uncharacterized protein
EOG091201ED	TC011023	Tribolium castaneum	Putative uncharacterized protein
EOG091201ED	LDEC022379	Leptinotarsa decemlineata	Putative uncharacterized protein
EOG091201ED	YQE_06368	Dendroctonus ponderosae	Putative uncharacterized protein
EOG091201EG	OTAU004012	Onthophagus taurus	Putative uncharacterized protein
EOG091201EG	AGLA001459	Anoplophora glabripennis	Putative uncharacterized protein
EOG091201EG	APLA005200	Agrilus planipennis	Putative uncharacterized protein
EOG091201EG	TC002395	Tribolium castaneum	Putative uncharacterized protein
EOG091201EG	LDEC003369	Leptinotarsa decemlineata	Putative uncharacterized protein
EOG091201EG	YQE_07889	Dendroctonus ponderosae	Putative uncharacterized protein
EOG091201EI	OTAU004483	Onthophagus taurus	Putative uncharacterized protein
EOG091201EI	AGLA008592	Anoplophora glabripennis	Putative uncharacterized protein
EOG091201EI	APLA006694	Agrilus planipennis	Putative uncharacterized protein
EOG091201EI	TC010343	Tribolium castaneum	Putative uncharacterized protein
EOG091201EI	LDEC002689	Leptinotarsa decemlineata	Putative uncharacterized protein
EOG091201EI	YQE_09954	Dendroctonus ponderosae	Putative uncharacterized protein
EOG091201EJ	OTAU008944	Onthophagus taurus	Putative uncharacterized protein
EOG091201EJ	AGLA002090	Anoplophora glabripennis	Putative uncharacterized protein
EOG091201EJ	APLA013740	Agrilus planipennis	Putative uncharacterized protein
EOG091201EJ	TC003148	Tribolium castaneum	Putative uncharacterized protein
EOG091201EJ	LDEC010894	Leptinotarsa decemlineata	Putative uncharacterized protein
EOG091201EJ	YQE_09762	Dendroctonus ponderosae	Putative uncharacterized protein

EOG091201EK	OTAU012237	Onthophagus taurus	None
EOG091201EK	AGLA019275	Anoplophora glabripennis	None
EOG091201EK	APLA009151	Agrilus planipennis	None
EOG091201EK	TC032393	Tribolium castaneum	None
EOG091201EK	LDEC002028	Leptinotarsa decemlineata	None
EOG091201EK	YQE_06391	Dendroctonus ponderosae	None
EOG091201EN	OTAU011718	Onthophagus taurus	Putative uncharacterized protein
EOG091201EN	AGLA002513	Anoplophora glabripennis	Putative uncharacterized protein
EOG091201EN	APLA011969	Agrilus planipennis	Putative uncharacterized protein
EOG091201EN	TC006024	Tribolium castaneum	Putative uncharacterized protein
EOG091201EN	LDEC003611	Leptinotarsa decemlineata	Putative uncharacterized protein
EOG091201EN	YQE_01888	Dendroctonus ponderosae	Putative uncharacterized protein
EOG091201EU	OTAU003342	Onthophagus taurus	Elongation factor 1-alpha
EOG091201EU	AGLA004566	Anoplophora glabripennis	Elongation factor 1-alpha
EOG091201EU	APLA012401	Agrilus planipennis	Elongation factor 1-alpha
EOG091201EU	TC031771	Tribolium castaneum	Elongation factor 1-alpha
EOG091201EU	LDEC001536	Leptinotarsa decemlineata	Elongation factor 1-alpha
EOG091201EU	YQE_03313	Dendroctonus ponderosae	Elongation factor 1-alpha
EOG091201EV	OTAU009662	Onthophagus taurus	Putative uncharacterized protein
EOG091201EV	AGLA003054	Anoplophora glabripennis	Putative uncharacterized protein
EOG091201EV	APLA004200	Agrilus planipennis	Putative uncharacterized protein
EOG091201EV	TC001907	Tribolium castaneum	Putative uncharacterized protein
EOG091201EV	LDEC017230	Leptinotarsa decemlineata	Putative uncharacterized protein
EOG091201EV	YQE_00049	Dendroctonus ponderosae	Putative uncharacterized protein
EOG091201EX	OTAU013367	Onthophagus taurus	Putative uncharacterized protein
EOG091201EX	AGLA015117	Anoplophora glabripennis	Putative uncharacterized protein
EOG091201EX	APLA011046	Agrilus planipennis	Putative uncharacterized protein
EOG091201EX	TC005506	Tribolium castaneum	Putative uncharacterized protein
EOG091201EX	LDEC011599	Leptinotarsa decemlineata	Putative uncharacterized protein
EOG091201EX	YQE_12686	Dendroctonus ponderosae	Putative uncharacterized protein
EOG091201EY	OTAU015288	Onthophagus taurus	Coronin
EOG091201EY	AGLA009972	Anoplophora glabripennis	Coronin
EOG091201EY	APLA000665	Agrilus planipennis	Coronin
EOG091201EY	TC015441	Tribolium castaneum	Coronin
EOG091201EY	LDEC010965	Leptinotarsa decemlineata	Coronin
EOG091201EY	YQE_10120	Dendroctonus ponderosae	Coronin
EOG091201EZ	OTAU010883	Onthophagus taurus	Putative uncharacterized protein
EOG091201EZ	AGLA019738	Anoplophora glabripennis	Putative uncharacterized protein
EOG091201EZ	APLA000772	Agrilus planipennis	Putative uncharacterized protein
EOG091201EZ	TC000130	Tribolium castaneum	Putative uncharacterized protein
EOG091201EZ	LDEC005012	Leptinotarsa decemlineata	Putative uncharacterized protein
EOG091201EZ	YQE_11857	Dendroctonus ponderosae	Putative uncharacterized protein
EOG091201F1	OTAU013954	Onthophagus taurus	Putative uncharacterized protein
EOG091201F1	AGLA006265	Anoplophora glabripennis	Putative uncharacterized protein
EOG091201F1	APLA007292	Agrilus planipennis	Putative uncharacterized protein
EOG091201F1	TC003078	Tribolium castaneum	Putative uncharacterized protein
EOG091201F1	LDEC010612	Leptinotarsa decemlineata	Putative uncharacterized protein
EOG091201F1	YQE_12396	Dendroctonus ponderosae	Putative uncharacterized protein
EOG091201F3	OTAU002976	Onthophagus taurus	Putative uncharacterized protein
EOG091201F3	AGLA010308	Anoplophora glabripennis	Putative uncharacterized protein
EOG091201F3	APLA001682	Agrilus planipennis	Putative uncharacterized protein
EOG091201F3	TC009331	Tribolium castaneum	Putative uncharacterized protein
EOG091201F3	LDEC002218	Leptinotarsa decemlineata	Putative uncharacterized protein
EOG091201F3	YQE_11322	Dendroctonus ponderosae	Putative uncharacterized protein
EOG091201F4	OTAU001375	Onthophagus taurus	None
EOG091201F4	AGLA000357	Anoplophora glabripennis	None
EOG091201F4	APLA003629	Agrilus planipennis	None
EOG091201F4	TC034520	Tribolium castaneum	None
EOG091201F4	LDEC011485	Leptinotarsa decemlineata	None
EOG091201F4	YQE_03310	Dendroctonus ponderosae	None
EOG091201F5	OTAU010670	Onthophagus taurus	Putative uncharacterized protein
EOG091201F5	AGLA008272	Anoplophora glabripennis	Putative uncharacterized protein
EOG091201F5	APLA008789	Agrilus planipennis	Putative uncharacterized protein
EOG091201F5	TC009264	Tribolium castaneum	Putative uncharacterized protein
EOG091201F5	LDEC001270	Leptinotarsa decemlineata	Putative uncharacterized protein
EOG091201F5	YQE_11284	Dendroctonus ponderosae	Putative uncharacterized protein
EOG091201F6	OTAU001670	Onthophagus taurus	Signal recognition particle subunit SRP68

EOG091201F6	AGLA010209	Anoplophora glabripennis	Signal recognition particle subunit SRP68
EOG091201F6	APLA000516	Agrilus planipennis	Signal recognition particle subunit SRP68
EOG091201F6	TC034135	Tribolium castaneum	Signal recognition particle subunit SRP68
EOG091201F6	LDEC010125	Leptinotarsa decemlineata	Signal recognition particle subunit SRP68
EOG091201F6	YQE_08628	Dendroctonus ponderosae	Signal recognition particle subunit SRP68
EOG091201F7	OTAU011433	Onthophagus taurus	Putative uncharacterized protein
EOG091201F7	AGLA018570	Anoplophora glabripennis	Putative uncharacterized protein
EOG091201F7	APLA002127	Agrilus planipennis	Putative uncharacterized protein
EOG091201F7	TC012014	Tribolium castaneum	Putative uncharacterized protein
EOG091201F7	LDEC003878	Leptinotarsa decemlineata	Putative uncharacterized protein
EOG091201F7	YQE_04347	Dendroctonus ponderosae	Putative uncharacterized protein
EOG091201F9	OTAU013115	Onthophagus taurus	Putative uncharacterized protein
EOG091201F9	AGLA008315	Anoplophora glabripennis	Putative uncharacterized protein
EOG091201F9	APLA010387	Agrilus planipennis	Putative uncharacterized protein
EOG091201F9	TC013927	Tribolium castaneum	Putative uncharacterized protein
EOG091201F9	LDEC005359	Leptinotarsa decemlineata	Putative uncharacterized protein
EOG091201F9	YQE_08312	Dendroctonus ponderosae	Putative uncharacterized protein
EOG091201FC	OTAU014402	Onthophagus taurus	Putative uncharacterized protein
EOG091201FC	AGLA018530	Anoplophora glabripennis	Putative uncharacterized protein
EOG091201FC	APLA000368	Agrilus planipennis	Putative uncharacterized protein
EOG091201FC	TC006281	Tribolium castaneum	Putative uncharacterized protein
EOG091201FC	LDEC002447	Leptinotarsa decemlineata	Putative uncharacterized protein
EOG091201FC	YQE_05692	Dendroctonus ponderosae	Putative uncharacterized protein
EOG091201FE	OTAU007925	Onthophagus taurus	Putative uncharacterized protein
EOG091201FE	AGLA000201	Anoplophora glabripennis	Putative uncharacterized protein
EOG091201FE	APLA006679	Agrilus planipennis	Putative uncharacterized protein
EOG091201FE	TC002730	Tribolium castaneum	Putative uncharacterized protein
EOG091201FE	LDEC005419	Leptinotarsa decemlineata	Putative uncharacterized protein
EOG091201FE	YQE_07790	Dendroctonus ponderosae	Putative uncharacterized protein
EOG091201FG	OTAU010301	Onthophagus taurus	Putative uncharacterized protein
EOG091201FG	AGLA010237	Anoplophora glabripennis	Putative uncharacterized protein
EOG091201FG	APLA005700	Agrilus planipennis	Putative uncharacterized protein
EOG091201FG	TC009730	Tribolium castaneum	Putative uncharacterized protein
EOG091201FG	LDEC016006	Leptinotarsa decemlineata	Putative uncharacterized protein
EOG091201FG	YQE_11297	Dendroctonus ponderosae	Putative uncharacterized protein
EOG091201FJ	OTAU005237	Onthophagus taurus	Putative uncharacterized protein
EOG091201FJ	AGLA001697	Anoplophora glabripennis	Putative uncharacterized protein
EOG091201FJ	APLA014772	Agrilus planipennis	Putative uncharacterized protein
EOG091201FJ	TC009389	Tribolium castaneum	Putative uncharacterized protein
EOG091201FJ	LDEC012444	Leptinotarsa decemlineata	Putative uncharacterized protein
EOG091201FJ	YQE_02019	Dendroctonus ponderosae	Putative uncharacterized protein
EOG091201FK	OTAU002760	Onthophagus taurus	Putative uncharacterized protein
EOG091201FK	AGLA000250	Anoplophora glabripennis	Putative uncharacterized protein
EOG091201FK	APLA006417	Agrilus planipennis	Putative uncharacterized protein
EOG091201FK	TC001741	Tribolium castaneum	Putative uncharacterized protein
EOG091201FK	LDEC014136	Leptinotarsa decemlineata	Putative uncharacterized protein
EOG091201FK	YQE_09198	Dendroctonus ponderosae	Putative uncharacterized protein
EOG091201FL	OTAU011409	Onthophagus taurus	None
EOG091201FL	AGLA015824	Anoplophora glabripennis	None
EOG091201FL	APLA000377	Agrilus planipennis	None
EOG091201FL	TC034046	Tribolium castaneum	None
EOG091201FL	LDEC004924	Leptinotarsa decemlineata	None
EOG091201FL	YQE_12074	Dendroctonus ponderosae	None
EOG091201FM	OTAU015888	Onthophagus taurus	None
EOG091201FM	AGLA005720	Anoplophora glabripennis	None
EOG091201FM	APLA001474	Agrilus planipennis	None
EOG091201FM	TC032129	Tribolium castaneum	None
EOG091201FM	LDEC000023	Leptinotarsa decemlineata	None
EOG091201FM	YQE_09595	Dendroctonus ponderosae	None
EOG091201FN	OTAU004502	Onthophagus taurus	Putative uncharacterized protein
EOG091201FN	AGLA002121	Anoplophora glabripennis	Putative uncharacterized protein
EOG091201FN	APLA009145	Agrilus planipennis	Putative uncharacterized protein
EOG091201FN	TC003433	Tribolium castaneum	Putative uncharacterized protein
EOG091201FN	LDEC010230	Leptinotarsa decemlineata	Putative uncharacterized protein
EOG091201FN	YQE_10247	Dendroctonus ponderosae	Putative uncharacterized protein
EOG091201FO	OTAU005190	Onthophagus taurus	Putative uncharacterized protein
EOG091201FO	AGLA001506	Anoplophora glabripennis	Putative uncharacterized protein

EOG091201FO	APLA008428	Agrilus planipennis	Putative uncharacterized protein
EOG091201FO	TC000021	Tribolium castaneum	Putative uncharacterized protein
EOG091201FO	LDEC004637	Leptinotarsa decemlineata	Putative uncharacterized protein
EOG091201FO	YQE_01859	Dendroctonus ponderosae	Putative uncharacterized protein
EOG091201FQ	OTAU001219	Onthophagus taurus	Nicotinic acetylcholine receptor subunit beta1
EOG091201FQ	AGLA004530	Anoplophora glabripennis	Nicotinic acetylcholine receptor subunit beta1
EOG091201FQ	APLA006384	Agrilus planipennis	Nicotinic acetylcholine receptor subunit beta1
EOG091201FQ	TC001432	Tribolium castaneum	Nicotinic acetylcholine receptor subunit beta1
EOG091201FQ	LDEC020317	Leptinotarsa decemlineata	Nicotinic acetylcholine receptor subunit beta1
EOG091201FQ	YQE_06696	Dendroctonus ponderosae	Nicotinic acetylcholine receptor subunit beta1
EOG091201FR	OTAU006611	Onthophagus taurus	Putative uncharacterized protein
EOG091201FR	AGLA017919	Anoplophora glabripennis	Putative uncharacterized protein
EOG091201FR	APLA009814	Agrilus planipennis	Putative uncharacterized protein
EOG091201FR	TC008963	Tribolium castaneum	Putative uncharacterized protein
EOG091201FR	LDEC015376	Leptinotarsa decemlineata	Putative uncharacterized protein
EOG091201FR	YQE_02197	Dendroctonus ponderosae	Putative uncharacterized protein
EOG091201FS	OTAU000091	Onthophagus taurus	Importin subunit alpha
EOG091201FS	AGLA020127	Anoplophora glabripennis	Importin subunit alpha
EOG091201FS	APLA002485	Agrilus planipennis	Importin subunit alpha
EOG091201FS	TC014465	Tribolium castaneum	Importin subunit alpha
EOG091201FS	LDEC022806	Leptinotarsa decemlineata	Importin subunit alpha
EOG091201FS	YQE_10340	Dendroctonus ponderosae	Importin subunit alpha
EOG091201FU	OTAU004104	Onthophagus taurus	Putative uncharacterized protein
EOG091201FU	AGLA012281	Anoplophora glabripennis	Putative uncharacterized protein
EOG091201FU	APLA010086	Agrilus planipennis	Putative uncharacterized protein
EOG091201FU	TC009975	Tribolium castaneum	Putative uncharacterized protein
EOG091201FU	LDEC013676	Leptinotarsa decemlineata	Putative uncharacterized protein
EOG091201FU	YQE_05443	Dendroctonus ponderosae	Putative uncharacterized protein
EOG091201FV	OTAU005197	Onthophagus taurus	metal ion binding
EOG091201FV	AGLA000570	Anoplophora glabripennis	metal ion binding
EOG091201FV	APLA007022	Agrilus planipennis	metal ion binding
EOG091201FV	TC032308	Tribolium castaneum	metal ion binding
EOG091201FV	LDEC003742	Leptinotarsa decemlineata	metal ion binding
EOG091201FV	YQE_09970	Dendroctonus ponderosae	metal ion binding
EOG091201FX	OTAU006711	Onthophagus taurus	Putative uncharacterized protein
EOG091201FX	AGLA007770	Anoplophora glabripennis	Putative uncharacterized protein
EOG091201FX	APLA008586	Agrilus planipennis	Putative uncharacterized protein
EOG091201FX	TC001250	Tribolium castaneum	Putative uncharacterized protein
EOG091201FX	LDEC007633	Leptinotarsa decemlineata	Putative uncharacterized protein
EOG091201FX	YQE_02266	Dendroctonus ponderosae	Putative uncharacterized protein
EOG091201FY	OTAU001499	Onthophagus taurus	ATP binding
EOG091201FY	AGLA000272	Anoplophora glabripennis	ATP binding
EOG091201FY	APLA013948	Agrilus planipennis	ATP binding
EOG091201FY	TC034530	Tribolium castaneum	ATP binding
EOG091201FY	LDEC001076	Leptinotarsa decemlineata	ATP binding
EOG091201FY	YQE_06626	Dendroctonus ponderosae	ATP binding
EOG091201FZ	OTAU006016	Onthophagus taurus	Putative uncharacterized protein
EOG091201FZ	AGLA018702	Anoplophora glabripennis	Putative uncharacterized protein
EOG091201FZ	APLA013232	Agrilus planipennis	Putative uncharacterized protein
EOG091201FZ	TC009437	Tribolium castaneum	Putative uncharacterized protein
EOG091201FZ	LDEC014663	Leptinotarsa decemlineata	Putative uncharacterized protein
EOG091201FZ	YQE_06009	Dendroctonus ponderosae	Putative uncharacterized protein
EOG091201G0	OTAU003365	Onthophagus taurus	Putative uncharacterized protein
EOG091201G0	AGLA020612	Anoplophora glabripennis	Putative uncharacterized protein
EOG091201G0	APLA011795	Agrilus planipennis	Putative uncharacterized protein
EOG091201G0	TC012866	Tribolium castaneum	Putative uncharacterized protein
EOG091201G0	LDEC020995	Leptinotarsa decemlineata	Putative uncharacterized protein
EOG091201G0	YQE_02071	Dendroctonus ponderosae	Putative uncharacterized protein
EOG091201G4	OTAU009120	Onthophagus taurus	Putative uncharacterized protein
EOG091201G4	AGLA017836	Anoplophora glabripennis	Putative uncharacterized protein
EOG091201G4	APLA007580	Agrilus planipennis	Putative uncharacterized protein
EOG091201G4	TC013683	Tribolium castaneum	Putative uncharacterized protein
EOG091201G4	LDEC022336	Leptinotarsa decemlineata	Putative uncharacterized protein
EOG091201G4	YQE_04848	Dendroctonus ponderosae	Putative uncharacterized protein
EOG091201G7	OTAU007497	Onthophagus taurus	Putative uncharacterized protein
EOG091201G7	AGLA008573	Anoplophora glabripennis	Putative uncharacterized protein
EOG091201G7	APLA014873	Agrilus planipennis	Putative uncharacterized protein

EOG091201G7	TC015209	Tribolium castaneum	Putative uncharacterized protein
EOG091201G7	LDEC021060	Leptinotarsa decemlineata	Putative uncharacterized protein
EOG091201G7	YQE_11725	Dendroctonus ponderosae	Putative uncharacterized protein
EOG091201G8	OTAU001546	Onthophagus taurus	Putative uncharacterized protein
EOG091201G8	AGLA019185	Anoplophora glabripennis	Putative uncharacterized protein
EOG091201G8	APLA005826	Agrilus planipennis	Putative uncharacterized protein
EOG091201G8	TC006663	Tribolium castaneum	Putative uncharacterized protein
EOG091201G8	LDEC010094	Leptinotarsa decemlineata	Putative uncharacterized protein
EOG091201G8	YQE_05643	Dendroctonus ponderosae	Putative uncharacterized protein
EOG091201GD	OTAU003371	Onthophagus taurus	Cycle protein
EOG091201GD	AGLA007744	Anoplophora glabripennis	Cycle protein
EOG091201GD	APLA011372	Agrilus planipennis	Cycle protein
EOG091201GD	TC002494	Tribolium castaneum	Cycle protein
EOG091201GD	LDEC004019	Leptinotarsa decemlineata	Cycle protein
EOG091201GD	YQE_03758	Dendroctonus ponderosae	Cycle protein
EOG091201GE	OTAU002194	Onthophagus taurus	Putative uncharacterized protein
EOG091201GE	AGLA018299	Anoplophora glabripennis	Putative uncharacterized protein
EOG091201GE	APLA000132	Agrilus planipennis	Putative uncharacterized protein
EOG091201GE	TC002574	Tribolium castaneum	Putative uncharacterized protein
EOG091201GE	LDEC007903	Leptinotarsa decemlineata	Putative uncharacterized protein
EOG091201GE	YQE_05142	Dendroctonus ponderosae	Putative uncharacterized protein
EOG091201GF	OTAU000492	Onthophagus taurus	Putative uncharacterized protein
EOG091201GF	AGLA008178	Anoplophora glabripennis	Putative uncharacterized protein
EOG091201GF	APLA002317	Agrilus planipennis	Putative uncharacterized protein
EOG091201GF	TC011652	Tribolium castaneum	Putative uncharacterized protein
EOG091201GF	LDEC000537	Leptinotarsa decemlineata	Putative uncharacterized protein
EOG091201GF	YQE_12890	Dendroctonus ponderosae	Putative uncharacterized protein
EOG091201GI	OTAU010228	Onthophagus taurus	Putative uncharacterized protein
EOG091201GI	AGLA015561	Anoplophora glabripennis	Putative uncharacterized protein
EOG091201GI	APLA007254	Agrilus planipennis	Putative uncharacterized protein
EOG091201GI	TC010428	Tribolium castaneum	Putative uncharacterized protein
EOG091201GI	LDEC014808	Leptinotarsa decemlineata	Putative uncharacterized protein
EOG091201GI	YQE_00796	Dendroctonus ponderosae	Putative uncharacterized protein
EOG091201GK	OTAU002130	Onthophagus taurus	None
EOG091201GK	AGLA011730	Anoplophora glabripennis	None
EOG091201GK	APLA003275	Agrilus planipennis	None
EOG091201GK	TC034446	Tribolium castaneum	None
EOG091201GK	LDEC007629	Leptinotarsa decemlineata	None
EOG091201GK	YQE_10097	Dendroctonus ponderosae	None
EOG091201GL	OTAU009003	Onthophagus taurus	Transporter
EOG091201GL	AGLA001285	Anoplophora glabripennis	Transporter
EOG091201GL	APLA007743	Agrilus planipennis	Transporter
EOG091201GL	TC015882	Tribolium castaneum	Transporter
EOG091201GL	LDEC016043	Leptinotarsa decemlineata	Transporter
EOG091201GL	YQE_03083	Dendroctonus ponderosae	Transporter
EOG091201GM	OTAU003531	Onthophagus taurus	Putative uncharacterized protein
EOG091201GM	AGLA004915	Anoplophora glabripennis	Putative uncharacterized protein
EOG091201GM	APLA010091	Agrilus planipennis	Putative uncharacterized protein
EOG091201GM	TC008696	Tribolium castaneum	Putative uncharacterized protein
EOG091201GM	LDEC010390	Leptinotarsa decemlineata	Putative uncharacterized protein
EOG091201GM	YQE_05999	Dendroctonus ponderosae	Putative uncharacterized protein
EOG091201GN	OTAU006636	Onthophagus taurus	Putative uncharacterized protein
EOG091201GN	AGLA020214	Anoplophora glabripennis	Putative uncharacterized protein
EOG091201GN	APLA000770	Agrilus planipennis	Putative uncharacterized protein
EOG091201GN	TC002932	Tribolium castaneum	Putative uncharacterized protein
EOG091201GN	LDEC008568	Leptinotarsa decemlineata	Putative uncharacterized protein
EOG091201GN	YQE_10258	Dendroctonus ponderosae	Putative uncharacterized protein
EOG091201GO	OTAU003389	Onthophagus taurus	None
EOG091201GO	AGLA020772	Anoplophora glabripennis	None
EOG091201GO	APLA003196	Agrilus planipennis	None
EOG091201GO	TC034425	Tribolium castaneum	None
EOG091201GO	LDEC000527	Leptinotarsa decemlineata	None
EOG091201GO	YQE_08537	Dendroctonus ponderosae	None
EOG091201GP	OTAU001402	Onthophagus taurus	Putative uncharacterized protein
EOG091201GP	AGLA000374	Anoplophora glabripennis	Putative uncharacterized protein
EOG091201GP	APLA010646	Agrilus planipennis	Putative uncharacterized protein
EOG091201GP	TC013624	Tribolium castaneum	Putative uncharacterized protein

EOG091201GP	LDEC021649	Leptinotarsa decemlineata	Putative uncharacterized protein
EOG091201GP	YQE_02439	Dendroctonus ponderosae	Putative uncharacterized protein
EOG091201GR	OTAU006660	Onthophagus taurus	None
EOG091201GR	AGLA000578	Anoplophora glabripennis	None
EOG091201GR	APLA008132	Agrilus planipennis	None
EOG091201GR	TC032596	Tribolium castaneum	None
EOG091201GR	LDEC008402	Leptinotarsa decemlineata	None
EOG091201GR	YQE_08653	Dendroctonus ponderosae	None
EOG091201GS	OTAU015303	Onthophagus taurus	Putative uncharacterized protein
EOG091201GS	AGLA016963	Anoplophora glabripennis	Putative uncharacterized protein
EOG091201GS	APLA002409	Agrilus planipennis	Putative uncharacterized protein
EOG091201GS	TC013907	Tribolium castaneum	Putative uncharacterized protein
EOG091201GS	LDEC001149	Leptinotarsa decemlineata	Putative uncharacterized protein
EOG091201GS	YQE_10484	Dendroctonus ponderosae	Putative uncharacterized protein
EOG091201GT	OTAU013856	Onthophagus taurus	Putative uncharacterized protein
EOG091201GT	AGLA016298	Anoplophora glabripennis	Putative uncharacterized protein
EOG091201GT	APLA001827	Agrilus planipennis	Putative uncharacterized protein
EOG091201GT	TC014542	Tribolium castaneum	Putative uncharacterized protein
EOG091201GT	LDEC012321	Leptinotarsa decemlineata	Putative uncharacterized protein
EOG091201GT	YQE_03525	Dendroctonus ponderosae	Putative uncharacterized protein
EOG091201GX	OTAU012894	Onthophagus taurus	Amidophosphoribosyltransferase
EOG091201GX	AGLA019335	Anoplophora glabripennis	Amidophosphoribosyltransferase
EOG091201GX	APLA008936	Agrilus planipennis	Amidophosphoribosyltransferase
EOG091201GX	TC016203	Tribolium castaneum	Amidophosphoribosyltransferase
EOG091201GX	LDEC005599	Leptinotarsa decemlineata	Amidophosphoribosyltransferase
EOG091201GX	YQE_01606	Dendroctonus ponderosae	Amidophosphoribosyltransferase
EOG091201GY	OTAU001369	Onthophagus taurus	Putative uncharacterized protein
EOG091201GY	AGLA000408	Anoplophora glabripennis	Putative uncharacterized protein
EOG091201GY	APLA005584	Agrilus planipennis	Putative uncharacterized protein
EOG091201GY	TC013573	Tribolium castaneum	Putative uncharacterized protein
EOG091201GY	LDEC004676	Leptinotarsa decemlineata	Putative uncharacterized protein
EOG091201GY	YQE_06730	Dendroctonus ponderosae	Putative uncharacterized protein
EOG091201H0	OTAU010040	Onthophagus taurus	Putative uncharacterized protein
EOG091201H0	AGLA005255	Anoplophora glabripennis	Putative uncharacterized protein
EOG091201H0	APLA012795	Agrilus planipennis	Putative uncharacterized protein
EOG091201H0	TC008337	Tribolium castaneum	Putative uncharacterized protein
EOG091201H0	LDEC000081	Leptinotarsa decemlineata	Putative uncharacterized protein
EOG091201H0	YQE_02625	Dendroctonus ponderosae	Putative uncharacterized protein
EOG091201H4	OTAU001045	Onthophagus taurus	Putative uncharacterized protein
EOG091201H4	AGLA008058	Anoplophora glabripennis	Putative uncharacterized protein
EOG091201H4	APLA001255	Agrilus planipennis	Putative uncharacterized protein
EOG091201H4	TC000626	Tribolium castaneum	Putative uncharacterized protein
EOG091201H4	LDEC022271	Leptinotarsa decemlineata	Putative uncharacterized protein
EOG091201H4	YQE_02429	Dendroctonus ponderosae	Putative uncharacterized protein
EOG091201H7	OTAU011616	Onthophagus taurus	Putative uncharacterized protein
EOG091201H7	AGLA001308	Anoplophora glabripennis	Putative uncharacterized protein
EOG091201H7	APLA013923	Agrilus planipennis	Putative uncharacterized protein
EOG091201H7	TC004461	Tribolium castaneum	Putative uncharacterized protein
EOG091201H7	LDEC009638	Leptinotarsa decemlineata	Putative uncharacterized protein
EOG091201H7	YQE_01601	Dendroctonus ponderosae	Putative uncharacterized protein
EOG091201H8	OTAU005031	Onthophagus taurus	Putative uncharacterized protein
EOG091201H8	AGLA005819	Anoplophora glabripennis	Putative uncharacterized protein
EOG091201H8	APLA005006	Agrilus planipennis	Putative uncharacterized protein
EOG091201H8	TC005191	Tribolium castaneum	Putative uncharacterized protein
EOG091201H8	LDEC009109	Leptinotarsa decemlineata	Putative uncharacterized protein
EOG091201H8	YQE_09345	Dendroctonus ponderosae	Putative uncharacterized protein
EOG091201H9	OTAU001779	Onthophagus taurus	Putative uncharacterized protein
EOG091201H9	AGLA004989	Anoplophora glabripennis	Putative uncharacterized protein
EOG091201H9	APLA009647	Agrilus planipennis	Putative uncharacterized protein
EOG091201H9	TC005421	Tribolium castaneum	Putative uncharacterized protein
EOG091201H9	LDEC007982	Leptinotarsa decemlineata	Putative uncharacterized protein
EOG091201H9	YQE_05630	Dendroctonus ponderosae	Putative uncharacterized protein
EOG091201HE	OTAU005068	Onthophagus taurus	Putative uncharacterized protein
EOG091201HE	AGLA001231	Anoplophora glabripennis	Putative uncharacterized protein
EOG091201HE	APLA005739	Agrilus planipennis	Putative uncharacterized protein
EOG091201HE	TC005638	Tribolium castaneum	Putative uncharacterized protein
EOG091201HE	LDEC009677	Leptinotarsa decemlineata	Putative uncharacterized protein

EOG091201HE	YQE_07000	Dendroctonus ponderosae	Putative uncharacterized protein
EOG091201HG	OTAU005568	Onthophagus taurus	Putative uncharacterized protein
EOG091201HG	AGLA003759	Anoplophora glabripennis	Putative uncharacterized protein
EOG091201HG	APLA004700	Agrilus planipennis	Putative uncharacterized protein
EOG091201HG	TC007870	Tribolium castaneum	Putative uncharacterized protein
EOG091201HG	LDEC014780	Leptinotarsa decemlineata	Putative uncharacterized protein
EOG091201HG	YQE_12262	Dendroctonus ponderosae	Putative uncharacterized protein
EOG091201HI	OTAU001195	Onthophagus taurus	Putative uncharacterized protein
EOG091201HI	AGLA006102	Anoplophora glabripennis	Putative uncharacterized protein
EOG091201HI	APLA003137	Agrilus planipennis	Putative uncharacterized protein
EOG091201HI	TC003763	Tribolium castaneum	Putative uncharacterized protein
EOG091201HI	LDEC003389	Leptinotarsa decemlineata	Putative uncharacterized protein
EOG091201HI	YQE_10320	Dendroctonus ponderosae	Putative uncharacterized protein
EOG091201HJ	OTAU013897	Onthophagus taurus	Putative uncharacterized protein
EOG091201HJ	AGLA002277	Anoplophora glabripennis	Putative uncharacterized protein
EOG091201HJ	APLA014500	Agrilus planipennis	Putative uncharacterized protein
EOG091201HJ	TC001920	Tribolium castaneum	Putative uncharacterized protein
EOG091201HJ	LDEC000582	Leptinotarsa decemlineata	Putative uncharacterized protein
EOG091201HJ	YQE_08857	Dendroctonus ponderosae	Putative uncharacterized protein
EOG091201HK	OTAU002808	Onthophagus taurus	5-aminolevulinate synthase
EOG091201HK	AGLA000278	Anoplophora glabripennis	5-aminolevulinate synthase
EOG091201HK	APLA010645	Agrilus planipennis	5-aminolevulinate synthase
EOG091201HK	TC013340	Tribolium castaneum	5-aminolevulinate synthase
EOG091201HK	LDEC001114	Leptinotarsa decemlineata	5-aminolevulinate synthase
EOG091201HK	YQE_06632	Dendroctonus ponderosae	5-aminolevulinate synthase
EOG091201HL	OTAU007198	Onthophagus taurus	metal ion binding
EOG091201HL	AGLA009232	Anoplophora glabripennis	metal ion binding
EOG091201HL	APLA000647	Agrilus planipennis	metal ion binding
EOG091201HL	TC034738	Tribolium castaneum	metal ion binding
EOG091201HL	LDEC014925	Leptinotarsa decemlineata	metal ion binding
EOG091201HL	YQE_11490	Dendroctonus ponderosae	metal ion binding
EOG091201HN	OTAU009099	Onthophagus taurus	Putative uncharacterized protein
EOG091201HN	AGLA001227	Anoplophora glabripennis	Putative uncharacterized protein
EOG091201HN	APLA005734	Agrilus planipennis	Putative uncharacterized protein
EOG091201HN	TC006481	Tribolium castaneum	Putative uncharacterized protein
EOG091201HN	LDEC009669	Leptinotarsa decemlineata	Putative uncharacterized protein
EOG091201HN	YQE_12023	Dendroctonus ponderosae	Putative uncharacterized protein
EOG091201HP	OTAU002824	Onthophagus taurus	None
EOG091201HP	AGLA009444	Anoplophora glabripennis	None
EOG091201HP	APLA014041	Agrilus planipennis	None
EOG091201HP	TC034768	Tribolium castaneum	None
EOG091201HP	LDEC013017	Leptinotarsa decemlineata	None
EOG091201HP	YQE_03891	Dendroctonus ponderosae	None
EOG091201HU	OTAU011701	Onthophagus taurus	Putative uncharacterized protein
EOG091201HU	AGLA006383	Anoplophora glabripennis	Putative uncharacterized protein
EOG091201HU	APLA008956	Agrilus planipennis	Putative uncharacterized protein
EOG091201HU	TC004979	Tribolium castaneum	Putative uncharacterized protein
EOG091201HU	LDEC011742	Leptinotarsa decemlineata	Putative uncharacterized protein
EOG091201HU	YQE_07641	Dendroctonus ponderosae	Putative uncharacterized protein
EOG091201HW	OTAU002495	Onthophagus taurus	None
EOG091201HW	AGLA003129	Anoplophora glabripennis	None
EOG091201HW	APLA004762	Agrilus planipennis	None
EOG091201HW	TC033687	Tribolium castaneum	None
EOG091201HW	LDEC016672	Leptinotarsa decemlineata	None
EOG091201HW	YQE_08817	Dendroctonus ponderosae	None
EOG091201HY	OTAU011105	Onthophagus taurus	Putative uncharacterized protein
EOG091201HY	AGLA011576	Anoplophora glabripennis	Putative uncharacterized protein
EOG091201HY	APLA006260	Agrilus planipennis	Putative uncharacterized protein
EOG091201HY	TC000357	Tribolium castaneum	Putative uncharacterized protein
EOG091201HY	LDEC007780	Leptinotarsa decemlineata	Putative uncharacterized protein
EOG091201HY	YQE_04985	Dendroctonus ponderosae	Putative uncharacterized protein
EOG091201HZ	OTAU005897	Onthophagus taurus	Putative uncharacterized protein
EOG091201HZ	AGLA015339	Anoplophora glabripennis	Putative uncharacterized protein
EOG091201HZ	APLA006862	Agrilus planipennis	Putative uncharacterized protein
EOG091201HZ	TC014437	Tribolium castaneum	Putative uncharacterized protein
EOG091201HZ	LDEC020045	Leptinotarsa decemlineata	Putative uncharacterized protein
EOG091201HZ	YQE_08247	Dendroctonus ponderosae	Putative uncharacterized protein

EOG091201I6	OTAU005009	Onthophagus taurus	Putative uncharacterized protein
EOG091201I6	AGLA000733	Anoplophora glabripennis	Putative uncharacterized protein
EOG091201I6	APLA010986	Agrilus planipennis	Putative uncharacterized protein
EOG091201I6	TC007976	Tribolium castaneum	Putative uncharacterized protein
EOG091201I6	LDEC002407	Leptinotarsa decemlineata	Putative uncharacterized protein
EOG091201I6	YQE_04802	Dendroctonus ponderosae	Putative uncharacterized protein
EOG091201I9	OTAU009334	Onthophagus taurus	None
EOG091201I9	AGLA013800	Anoplophora glabripennis	None
EOG091201I9	APLA006854	Agrilus planipennis	None
EOG091201I9	TC033157	Tribolium castaneum	None
EOG091201I9	LDEC002630	Leptinotarsa decemlineata	None
EOG091201I9	YQE_07290	Dendroctonus ponderosae	None
EOG091201IA	OTAU005460	Onthophagus taurus	Putative uncharacterized protein
EOG091201IA	AGLA015343	Anoplophora glabripennis	Putative uncharacterized protein
EOG091201IA	APLA002688	Agrilus planipennis	Putative uncharacterized protein
EOG091201IA	TC012991	Tribolium castaneum	Putative uncharacterized protein
EOG091201IA	LDEC007753	Leptinotarsa decemlineata	Putative uncharacterized protein
EOG091201IA	YQE_07244	Dendroctonus ponderosae	Putative uncharacterized protein
EOG091201IC	OTAU012681	Onthophagus taurus	ATP binding
EOG091201IC	AGLA014761	Anoplophora glabripennis	ATP binding
EOG091201IC	APLA008924	Agrilus planipennis	ATP binding
EOG091201IC	TC031438	Tribolium castaneum	ATP binding
EOG091201IC	LDEC010146	Leptinotarsa decemlineata	ATP binding
EOG091201IC	YQE_06819	Dendroctonus ponderosae	ATP binding
EOG091201ID	OTAU013408	Onthophagus taurus	Putative uncharacterized protein
EOG091201ID	AGLA006059	Anoplophora glabripennis	Putative uncharacterized protein
EOG091201ID	APLA007349	Agrilus planipennis	Putative uncharacterized protein
EOG091201ID	TC014422	Tribolium castaneum	Putative uncharacterized protein
EOG091201ID	LDEC014787	Leptinotarsa decemlineata	Putative uncharacterized protein
EOG091201ID	YQE_10644	Dendroctonus ponderosae	Putative uncharacterized protein
EOG091201IF	OTAU011060	Onthophagus taurus	mannosyltransferase activity
EOG091201IF	AGLA012346	Anoplophora glabripennis	mannosyltransferase activity
EOG091201IF	APLA008830	Agrilus planipennis	mannosyltransferase activity
EOG091201IF	TC033711	Tribolium castaneum	mannosyltransferase activity
EOG091201IF	LDEC010658	Leptinotarsa decemlineata	mannosyltransferase activity
EOG091201IF	YQE_10621	Dendroctonus ponderosae	mannosyltransferase activity
EOG091201II	OTAU001825	Onthophagus taurus	Putative uncharacterized protein
EOG091201II	AGLA004983	Anoplophora glabripennis	Putative uncharacterized protein
EOG091201II	APLA001454	Agrilus planipennis	Putative uncharacterized protein
EOG091201II	TC006628	Tribolium castaneum	Putative uncharacterized protein
EOG091201II	LDEC003005	Leptinotarsa decemlineata	Putative uncharacterized protein
EOG091201II	YQE_07014	Dendroctonus ponderosae	Putative uncharacterized protein
EOG091201IM	OTAU008597	Onthophagus taurus	Putative uncharacterized protein
EOG091201IM	AGLA014416	Anoplophora glabripennis	Putative uncharacterized protein
EOG091201IM	APLA009229	Agrilus planipennis	Putative uncharacterized protein
EOG091201IM	TC007192	Tribolium castaneum	Putative uncharacterized protein
EOG091201IM	LDEC008315	Leptinotarsa decemlineata	Putative uncharacterized protein
EOG091201IM	YQE_02554	Dendroctonus ponderosae	Putative uncharacterized protein
EOG091201IN	OTAU010954	Onthophagus taurus	Putative uncharacterized protein
EOG091201IN	AGLA012730	Anoplophora glabripennis	Putative uncharacterized protein
EOG091201IN	APLA007319	Agrilus planipennis	Putative uncharacterized protein
EOG091201IN	TC003034	Tribolium castaneum	Putative uncharacterized protein
EOG091201IN	LDEC020107	Leptinotarsa decemlineata	Putative uncharacterized protein
EOG091201IN	YQE_07396	Dendroctonus ponderosae	Putative uncharacterized protein
EOG091201IO	OTAU013111	Onthophagus taurus	Putative uncharacterized protein
EOG091201IO	AGLA020211	Anoplophora glabripennis	Putative uncharacterized protein
EOG091201IO	APLA000749	Agrilus planipennis	Putative uncharacterized protein
EOG091201IO	TC000137	Tribolium castaneum	Putative uncharacterized protein
EOG091201IO	LDEC008570	Leptinotarsa decemlineata	Putative uncharacterized protein
EOG091201IO	YQE_07863	Dendroctonus ponderosae	Putative uncharacterized protein
EOG091201IP	OTAU008147	Onthophagus taurus	Putative uncharacterized protein
EOG091201IP	AGLA014664	Anoplophora glabripennis	Putative uncharacterized protein
EOG091201IP	APLA005928	Agrilus planipennis	Putative uncharacterized protein
EOG091201IP	TC015016	Tribolium castaneum	Putative uncharacterized protein
EOG091201IP	LDEC003687	Leptinotarsa decemlineata	Putative uncharacterized protein
EOG091201IP	YQE_08349	Dendroctonus ponderosae	Putative uncharacterized protein
EOG091201IQ	OTAU007040	Onthophagus taurus	Putative uncharacterized protein

EOG091201IQ	AGLA012467	Anoplophora glabripennis	Putative uncharacterized protein
EOG091201IQ	APLA001020	Agrilus planipennis	Putative uncharacterized protein
EOG091201IQ	TC003856	Tribolium castaneum	Putative uncharacterized protein
EOG091201IQ	LDEC011103	Leptinotarsa decemlineata	Putative uncharacterized protein
EOG091201IQ	YQE_03657	Dendroctonus ponderosae	Putative uncharacterized protein
EOG091201IR	OTAU002049	Onthophagus taurus	Dunce
EOG091201IR	AGLA011418	Anoplophora glabripennis	Dunce
EOG091201IR	APLA003228	Agrilus planipennis	Dunce
EOG091201IR	TC012593	Tribolium castaneum	Dunce
EOG091201IR	LDEC011302	Leptinotarsa decemlineata	Dunce
EOG091201IR	YQE_11958	Dendroctonus ponderosae	Dunce
EOG091201IS	OTAU008418	Onthophagus taurus	None
EOG091201IS	AGLA011659	Anoplophora glabripennis	None
EOG091201IS	APLA014242	Agrilus planipennis	None
EOG091201IS	TC033818	Tribolium castaneum	None
EOG091201IS	LDEC007814	Leptinotarsa decemlineata	None
EOG091201IS	YQE_11541	Dendroctonus ponderosae	None
EOG091201IT	OTAU001225	Onthophagus taurus	None
EOG091201IT	AGLA002368	Anoplophora glabripennis	None
EOG091201IT	APLA012403	Agrilus planipennis	None
EOG091201IT	TC034217	Tribolium castaneum	None
EOG091201IT	LDEC018865	Leptinotarsa decemlineata	None
EOG091201IT	YQE_02476	Dendroctonus ponderosae	None
EOG091201IU	OTAU014147	Onthophagus taurus	Putative uncharacterized protein
EOG091201IU	AGLA001774	Anoplophora glabripennis	Putative uncharacterized protein
EOG091201IU	APLA011706	Agrilus planipennis	Putative uncharacterized protein
EOG091201IU	TC014286	Tribolium castaneum	Putative uncharacterized protein
EOG091201IU	LDEC008934	Leptinotarsa decemlineata	Putative uncharacterized protein
EOG091201IU	YQE_06053	Dendroctonus ponderosae	Putative uncharacterized protein
EOG091201IW	OTAU008160	Onthophagus taurus	Putative uncharacterized protein
EOG091201IW	AGLA012494	Anoplophora glabripennis	Putative uncharacterized protein
EOG091201IW	APLA003546	Agrilus planipennis	Putative uncharacterized protein
EOG091201IW	TC001770	Tribolium castaneum	Putative uncharacterized protein
EOG091201IW	LDEC015018	Leptinotarsa decemlineata	Putative uncharacterized protein
EOG091201IW	YQE_04106	Dendroctonus ponderosae	Putative uncharacterized protein
EOG091201IX	OTAU000438	Onthophagus taurus	Putative uncharacterized protein
EOG091201IX	AGLA011667	Anoplophora glabripennis	Putative uncharacterized protein
EOG091201IX	APLA010526	Agrilus planipennis	Putative uncharacterized protein
EOG091201IX	TC012490	Tribolium castaneum	Putative uncharacterized protein
EOG091201IX	LDEC007747	Leptinotarsa decemlineata	Putative uncharacterized protein
EOG091201IX	YQE_07613	Dendroctonus ponderosae	Putative uncharacterized protein
EOG091201IY	OTAU008499	Onthophagus taurus	Putative uncharacterized protein
EOG091201IY	AGLA017161	Anoplophora glabripennis	Putative uncharacterized protein
EOG091201IY	APLA002864	Agrilus planipennis	Putative uncharacterized protein
EOG091201IY	TC013181	Tribolium castaneum	Putative uncharacterized protein
EOG091201IY	LDEC006756	Leptinotarsa decemlineata	Putative uncharacterized protein
EOG091201IY	YQE_06201	Dendroctonus ponderosae	Putative uncharacterized protein
EOG091201IZ	OTAU004125	Onthophagus taurus	catalytic activity
EOG091201IZ	AGLA017365	Anoplophora glabripennis	catalytic activity
EOG091201IZ	APLA004622	Agrilus planipennis	catalytic activity
EOG091201IZ	TC033610	Tribolium castaneum	catalytic activity
EOG091201IZ	LDEC020280	Leptinotarsa decemlineata	catalytic activity
EOG091201IZ	YQE_11275	Dendroctonus ponderosae	catalytic activity
EOG091201J4	OTAU009793	Onthophagus taurus	Putative uncharacterized protein
EOG091201J4	AGLA003507	Anoplophora glabripennis	Putative uncharacterized protein
EOG091201J4	APLA003384	Agrilus planipennis	Putative uncharacterized protein
EOG091201J4	TC003290	Tribolium castaneum	Putative uncharacterized protein
EOG091201J4	LDEC012703	Leptinotarsa decemlineata	Putative uncharacterized protein
EOG091201J4	YQE_07453	Dendroctonus ponderosae	Putative uncharacterized protein
EOG091201J5	OTAU007179	Onthophagus taurus	RNA binding
EOG091201J5	AGLA016195	Anoplophora glabripennis	RNA binding
EOG091201J5	APLA008780	Agrilus planipennis	RNA binding
EOG091201J5	TC033397	Tribolium castaneum	RNA binding
EOG091201J5	LDEC021992	Leptinotarsa decemlineata	RNA binding
EOG091201J5	YQE_02163	Dendroctonus ponderosae	RNA binding
EOG091201J8	OTAU014120	Onthophagus taurus	Putative uncharacterized protein
EOG091201J8	AGLA012694	Anoplophora glabripennis	Putative uncharacterized protein

EOG091201J8	APLA011313	Agrilus planipennis	Putative uncharacterized protein
EOG091201J8	TC012023	Tribolium castaneum	Putative uncharacterized protein
EOG091201J8	LDEC013632	Leptinotarsa decemlineata	Putative uncharacterized protein
EOG091201J8	YQE_02247	Dendroctonus ponderosae	Putative uncharacterized protein
EOG091201J9	OTAU004351	Onthophagus taurus	Putative uncharacterized protein
EOG091201J9	AGLA007616	Anoplophora glabripennis	Putative uncharacterized protein
EOG091201J9	APLA003488	Agrilus planipennis	Putative uncharacterized protein
EOG091201J9	TC004791	Tribolium castaneum	Putative uncharacterized protein
EOG091201J9	LDEC001117	Leptinotarsa decemlineata	Putative uncharacterized protein
EOG091201J9	YQE_09073	Dendroctonus ponderosae	Putative uncharacterized protein
EOG091201JA	OTAU006956	Onthophagus taurus	Putative uncharacterized protein
EOG091201JA	AGLA015053	Anoplophora glabripennis	Putative uncharacterized protein
EOG091201JA	APLA001656	Agrilus planipennis	Putative uncharacterized protein
EOG091201JA	TC030712	Tribolium castaneum	Putative uncharacterized protein
EOG091201JA	LDEC016379	Leptinotarsa decemlineata	Putative uncharacterized protein
EOG091201JA	YQE_12459	Dendroctonus ponderosae	Putative uncharacterized protein
EOG091201JC	OTAU008617	Onthophagus taurus	Putative uncharacterized protein
EOG091201JC	AGLA004507	Anoplophora glabripennis	Putative uncharacterized protein
EOG091201JC	APLA001308	Agrilus planipennis	Putative uncharacterized protein
EOG091201JC	TC001243	Tribolium castaneum	Putative uncharacterized protein
EOG091201JC	LDEC016730	Leptinotarsa decemlineata	Putative uncharacterized protein
EOG091201JC	YQE_08489	Dendroctonus ponderosae	Putative uncharacterized protein
EOG091201JE	OTAU000967	Onthophagus taurus	Putative uncharacterized protein
EOG091201JE	AGLA015901	Anoplophora glabripennis	Putative uncharacterized protein
EOG091201JE	APLA008229	Agrilus planipennis	Putative uncharacterized protein
EOG091201JE	TC001034	Tribolium castaneum	Putative uncharacterized protein
EOG091201JE	LDEC018502	Leptinotarsa decemlineata	Putative uncharacterized protein
EOG091201JE	YQE_01699	Dendroctonus ponderosae	Putative uncharacterized protein
EOG091201JF	OTAU001556	Onthophagus taurus	Putative uncharacterized protein
EOG091201JF	AGLA009098	Anoplophora glabripennis	Putative uncharacterized protein
EOG091201JF	APLA013154	Agrilus planipennis	Putative uncharacterized protein
EOG091201JF	TC005760	Tribolium castaneum	Putative uncharacterized protein
EOG091201JF	LDEC016210	Leptinotarsa decemlineata	Putative uncharacterized protein
EOG091201JF	YQE_12038	Dendroctonus ponderosae	Putative uncharacterized protein
EOG091201JG	OTAU008910	Onthophagus taurus	Putative uncharacterized protein
EOG091201JG	AGLA004595	Anoplophora glabripennis	Putative uncharacterized protein
EOG091201JG	APLA006748	Agrilus planipennis	Putative uncharacterized protein
EOG091201JG	TC004290	Tribolium castaneum	Putative uncharacterized protein
EOG091201JG	LDEC020931	Leptinotarsa decemlineata	Putative uncharacterized protein
EOG091201JG	YQE_09307	Dendroctonus ponderosae	Putative uncharacterized protein
EOG091201JJ	OTAU013298	Onthophagus taurus	None
EOG091201JJ	AGLA004214	Anoplophora glabripennis	None
EOG091201JJ	APLA004838	Agrilus planipennis	None
EOG091201JJ	TC033257	Tribolium castaneum	None
EOG091201JJ	LDEC010199	Leptinotarsa decemlineata	None
EOG091201JJ	YQE_06212	Dendroctonus ponderosae	None
EOG091201JK	OTAU003450	Onthophagus taurus	Putative uncharacterized protein
EOG091201JK	AGLA002799	Anoplophora glabripennis	Putative uncharacterized protein
EOG091201JK	APLA007157	Agrilus planipennis	Putative uncharacterized protein
EOG091201JK	TC009157	Tribolium castaneum	Putative uncharacterized protein
EOG091201JK	LDEC014717	Leptinotarsa decemlineata	Putative uncharacterized protein
EOG091201JK	YQE_11937	Dendroctonus ponderosae	Putative uncharacterized protein
EOG091201JL	OTAU010419	Onthophagus taurus	Putative uncharacterized protein
EOG091201JL	AGLA014163	Anoplophora glabripennis	Putative uncharacterized protein
EOG091201JL	APLA007972	Agrilus planipennis	Putative uncharacterized protein
EOG091201JL	TC011263	Tribolium castaneum	Putative uncharacterized protein
EOG091201JL	LDEC010314	Leptinotarsa decemlineata	Putative uncharacterized protein
EOG091201JL	YQE_07728	Dendroctonus ponderosae	Putative uncharacterized protein
EOG091201JN	OTAU006940	Onthophagus taurus	Putative uncharacterized protein
EOG091201JN	AGLA006257	Anoplophora glabripennis	Putative uncharacterized protein
EOG091201JN	APLA009177	Agrilus planipennis	Putative uncharacterized protein
EOG091201JN	TC003070	Tribolium castaneum	Putative uncharacterized protein
EOG091201JN	LDEC010602	Leptinotarsa decemlineata	Putative uncharacterized protein
EOG091201JN	YQE_03743	Dendroctonus ponderosae	Putative uncharacterized protein
EOG091201JO	OTAU009491	Onthophagus taurus	None
EOG091201JO	AGLA003883	Anoplophora glabripennis	None
EOG091201JO	APLA003840	Agrilus planipennis	None

EOG091201JO	TC034722	Tribolium castaneum	None
EOG091201JO	LDEC008804	Leptinotarsa decemlineata	None
EOG091201JO	YQE_11709	Dendroctonus ponderosae	None
EOG091201JQ	OTAU011717	Onthophagus taurus	Putative uncharacterized protein
EOG091201JQ	AGLA015910	Anoplophora glabripennis	Putative uncharacterized protein
EOG091201JQ	APLA000678	Agrilus planipennis	Putative uncharacterized protein
EOG091201JQ	TC015033	Tribolium castaneum	Putative uncharacterized protein
EOG091201JQ	LDEC009937	Leptinotarsa decemlineata	Putative uncharacterized protein
EOG091201JQ	YQE_05958	Dendroctonus ponderosae	Putative uncharacterized protein
EOG091201JU	OTAU000725	Onthophagus taurus	Putative uncharacterized protein
EOG091201JU	AGLA021602	Anoplophora glabripennis	Putative uncharacterized protein
EOG091201JU	APLA001793	Agrilus planipennis	Putative uncharacterized protein
EOG091201JU	TC011923	Tribolium castaneum	Putative uncharacterized protein
EOG091201JU	LDEC019902	Leptinotarsa decemlineata	Putative uncharacterized protein
EOG091201JU	YQE_02000	Dendroctonus ponderosae	Putative uncharacterized protein
EOG091201JX	OTAU005991	Onthophagus taurus	Putative uncharacterized protein
EOG091201JX	AGLA018216	Anoplophora glabripennis	Putative uncharacterized protein
EOG091201JX	APLA002073	Agrilus planipennis	Putative uncharacterized protein
EOG091201JX	TC011521	Tribolium castaneum	Putative uncharacterized protein
EOG091201JX	LDEC010286	Leptinotarsa decemlineata	Putative uncharacterized protein
EOG091201JX	YQE_11119	Dendroctonus ponderosae	Putative uncharacterized protein
EOG091201JZ	OTAU003316	Onthophagus taurus	DNA binding
EOG091201JZ	AGLA000417	Anoplophora glabripennis	DNA binding
EOG091201JZ	APLA006394	Agrilus planipennis	DNA binding
EOG091201JZ	TC031486	Tribolium castaneum	DNA binding
EOG091201JZ	LDEC004228	Leptinotarsa decemlineata	DNA binding
EOG091201JZ	YQE_09206	Dendroctonus ponderosae	DNA binding
EOG091201K0	OTAU008122	Onthophagus taurus	Putative uncharacterized protein
EOG091201K0	AGLA005829	Anoplophora glabripennis	Putative uncharacterized protein
EOG091201K0	APLA001245	Agrilus planipennis	Putative uncharacterized protein
EOG091201K0	TC000998	Tribolium castaneum	Putative uncharacterized protein
EOG091201K0	LDEC001686	Leptinotarsa decemlineata	Putative uncharacterized protein
EOG091201K0	YQE_09716	Dendroctonus ponderosae	Putative uncharacterized protein
EOG091201K2	OTAU010044	Onthophagus taurus	Regulator of telomere elongation helicase 1
EOG091201K2	AGLA016562	Anoplophora glabripennis	Regulator of telomere elongation helicase 1
EOG091201K2	APLA004209	Agrilus planipennis	Regulator of telomere elongation helicase 1
EOG091201K2	TC007666	Tribolium castaneum	Regulator of telomere elongation helicase 1
EOG091201K2	LDEC008497	Leptinotarsa decemlineata	Regulator of telomere elongation helicase 1
EOG091201K2	YQE_03016	Dendroctonus ponderosae	Regulator of telomere elongation helicase 1
EOG091201K3	OTAU007466	Onthophagus taurus	ATP synthase subunit beta
EOG091201K3	AGLA001082	Anoplophora glabripennis	ATP synthase subunit beta
EOG091201K3	APLA009120	Agrilus planipennis	ATP synthase subunit beta
EOG091201K3	TC015322	Tribolium castaneum	ATP synthase subunit beta
EOG091201K3	LDEC020740	Leptinotarsa decemlineata	ATP synthase subunit beta
EOG091201K3	YQE_08399	Dendroctonus ponderosae	ATP synthase subunit beta
EOG091201K4	OTAU002635	Onthophagus taurus	Putative uncharacterized protein
EOG091201K4	AGLA010325	Anoplophora glabripennis	Putative uncharacterized protein
EOG091201K4	APLA014716	Agrilus planipennis	Putative uncharacterized protein
EOG091201K4	TC030760	Tribolium castaneum	Putative uncharacterized protein
EOG091201K4	LDEC008538	Leptinotarsa decemlineata	Putative uncharacterized protein
EOG091201K4	YQE_11672	Dendroctonus ponderosae	Putative uncharacterized protein
EOG091201K5	OTAU012490	Onthophagus taurus	None
EOG091201K5	AGLA006680	Anoplophora glabripennis	None
EOG091201K5	APLA012245	Agrilus planipennis	None
EOG091201K5	TC034178	Tribolium castaneum	None
EOG091201K5	LDEC003554	Leptinotarsa decemlineata	None
EOG091201K5	YQE_04508	Dendroctonus ponderosae	None
EOG091201K6	OTAU009963	Onthophagus taurus	None
EOG091201K6	AGLA000827	Anoplophora glabripennis	None
EOG091201K6	APLA002147	Agrilus planipennis	None
EOG091201K6	TC032883	Tribolium castaneum	None
EOG091201K6	LDEC001391	Leptinotarsa decemlineata	None
EOG091201K6	YQE_08700	Dendroctonus ponderosae	None
EOG091201K7	OTAU002566	Onthophagus taurus	None
EOG091201K7	AGLA017931	Anoplophora glabripennis	None
EOG091201K7	APLA004888	Agrilus planipennis	None
EOG091201K7	TC033225	Tribolium castaneum	None

EOG091201K7	LDEC003236	Leptinotarsa decemlineata	None
EOG091201K7	YQE_03423	Dendroctonus ponderosae	None
EOG091201K9	OTAU012172	Onthophagus taurus	Putative uncharacterized protein
EOG091201K9	AGLA003055	Anoplophora glabripennis	Putative uncharacterized protein
EOG091201K9	APLA010135	Agrilus planipennis	Putative uncharacterized protein
EOG091201K9	TC007628	Tribolium castaneum	Putative uncharacterized protein
EOG091201K9	LDEC017229	Leptinotarsa decemlineata	Putative uncharacterized protein
EOG091201K9	YQE_03561	Dendroctonus ponderosae	Putative uncharacterized protein
EOG091201KC	OTAU013344	Onthophagus taurus	Putative uncharacterized protein
EOG091201KC	AGLA018471	Anoplophora glabripennis	Putative uncharacterized protein
EOG091201KC	APLA009086	Agrilus planipennis	Putative uncharacterized protein
EOG091201KC	TC000110	Tribolium castaneum	Putative uncharacterized protein
EOG091201KC	LDEC013675	Leptinotarsa decemlineata	Putative uncharacterized protein
EOG091201KC	YQE_04505	Dendroctonus ponderosae	Putative uncharacterized protein
EOG091201KF	OTAU015515	Onthophagus taurus	Putative uncharacterized protein
EOG091201KF	AGLA003454	Anoplophora glabripennis	Putative uncharacterized protein
EOG091201KF	APLA006929	Agrilus planipennis	Putative uncharacterized protein
EOG091201KF	TC009201	Tribolium castaneum	Putative uncharacterized protein
EOG091201KF	LDEC005237	Leptinotarsa decemlineata	Putative uncharacterized protein
EOG091201KF	YQE_11833	Dendroctonus ponderosae	Putative uncharacterized protein
EOG091201KI	OTAU003948	Onthophagus taurus	Putative uncharacterized protein
EOG091201KI	AGLA006870	Anoplophora glabripennis	Putative uncharacterized protein
EOG091201KI	APLA003224	Agrilus planipennis	Putative uncharacterized protein
EOG091201KI	TC012393	Tribolium castaneum	Putative uncharacterized protein
EOG091201KI	LDEC007432	Leptinotarsa decemlineata	Putative uncharacterized protein
EOG091201KI	YQE_08036	Dendroctonus ponderosae	Putative uncharacterized protein
EOG091201KK	OTAU005571	Onthophagus taurus	Putative uncharacterized protein
EOG091201KK	AGLA000845	Anoplophora glabripennis	Putative uncharacterized protein
EOG091201KK	APLA013021	Agrilus planipennis	Putative uncharacterized protein
EOG091201KK	TC007564	Tribolium castaneum	Putative uncharacterized protein
EOG091201KK	LDEC018654	Leptinotarsa decemlineata	Putative uncharacterized protein
EOG091201KK	YQE_04741	Dendroctonus ponderosae	Putative uncharacterized protein
EOG091201KL	OTAU000517	Onthophagus taurus	Putative uncharacterized protein
EOG091201KL	AGLA009214	Anoplophora glabripennis	Putative uncharacterized protein
EOG091201KL	APLA001053	Agrilus planipennis	Putative uncharacterized protein
EOG091201KL	TC011720	Tribolium castaneum	Putative uncharacterized protein
EOG091201KL	LDEC000578	Leptinotarsa decemlineata	Putative uncharacterized protein
EOG091201KL	YQE_08087	Dendroctonus ponderosae	Putative uncharacterized protein
EOG091201KN	OTAU007624	Onthophagus taurus	poly(ADP-ribose) glycohydrolase activity
EOG091201KN	AGLA005505	Anoplophora glabripennis	poly(ADP-ribose) glycohydrolase activity
EOG091201KN	APLA002403	Agrilus planipennis	poly(ADP-ribose) glycohydrolase activity
EOG091201KN	TC032986	Tribolium castaneum	poly(ADP-ribose) glycohydrolase activity
EOG091201KN	LDEC005351	Leptinotarsa decemlineata	poly(ADP-ribose) glycohydrolase activity
EOG091201KN	YQE_07274	Dendroctonus ponderosae	poly(ADP-ribose) glycohydrolase activity
EOG091201KQ	OTAU004313	Onthophagus taurus	protein dimerization activity
EOG091201KQ	AGLA014360	Anoplophora glabripennis	protein dimerization activity
EOG091201KQ	APLA010353	Agrilus planipennis	protein dimerization activity
EOG091201KQ	TC031479	Tribolium castaneum	protein dimerization activity
EOG091201KQ	LDEC004671	Leptinotarsa decemlineata	protein dimerization activity
EOG091201KQ	YQE_09240	Dendroctonus ponderosae	protein dimerization activity
EOG091201KR	OTAU007945	Onthophagus taurus	Putative uncharacterized protein
EOG091201KR	AGLA009698	Anoplophora glabripennis	Putative uncharacterized protein
EOG091201KR	APLA001843	Agrilus planipennis	Putative uncharacterized protein
EOG091201KR	TC009746	Tribolium castaneum	Putative uncharacterized protein
EOG091201KR	LDEC018918	Leptinotarsa decemlineata	Putative uncharacterized protein
EOG091201KR	YQE_12602	Dendroctonus ponderosae	Putative uncharacterized protein
EOG091201KS	OTAU008402	Onthophagus taurus	Putative uncharacterized protein
EOG091201KS	AGLA007736	Anoplophora glabripennis	Putative uncharacterized protein
EOG091201KS	APLA000320	Agrilus planipennis	Putative uncharacterized protein
EOG091201KS	TC003562	Tribolium castaneum	Putative uncharacterized protein
EOG091201KS	LDEC009411	Leptinotarsa decemlineata	Putative uncharacterized protein
EOG091201KS	YQE_07634	Dendroctonus ponderosae	Putative uncharacterized protein
EOG091201KV	OTAU014136	Onthophagus taurus	Putative uncharacterized protein
EOG091201KV	AGLA021072	Anoplophora glabripennis	Putative uncharacterized protein
EOG091201KV	APLA002422	Agrilus planipennis	Putative uncharacterized protein
EOG091201KV	TC014592	Tribolium castaneum	Putative uncharacterized protein
EOG091201KV	LDEC014494	Leptinotarsa decemlineata	Putative uncharacterized protein

EOG091201KV	YQE_06162	Dendroctonus ponderosae	Putative uncharacterized protein
EOG091201KW	OTAU016292	Onthophagus taurus	Putative uncharacterized protein
EOG091201KW	AGLA014193	Anoplophora glabripennis	Putative uncharacterized protein
EOG091201KW	APLA005256	Agrilus planipennis	Putative uncharacterized protein
EOG091201KW	TC015464	Tribolium castaneum	Putative uncharacterized protein
EOG091201KW	LDEC016531	Leptinotarsa decemlineata	Putative uncharacterized protein
EOG091201KW	YQE_02317	Dendroctonus ponderosae	Putative uncharacterized protein
EOG091201KX	OTAU012294	Onthophagus taurus	Putative uncharacterized protein
EOG091201KX	AGLA015218	Anoplophora glabripennis	Putative uncharacterized protein
EOG091201KX	APLA004610	Agrilus planipennis	Putative uncharacterized protein
EOG091201KX	TC009330	Tribolium castaneum	Putative uncharacterized protein
EOG091201KX	LDEC004452	Leptinotarsa decemlineata	Putative uncharacterized protein
EOG091201KX	YQE_05405	Dendroctonus ponderosae	Putative uncharacterized protein
EOG091201KZ	OTAU001010	Onthophagus taurus	Putative uncharacterized protein
EOG091201KZ	AGLA014468	Anoplophora glabripennis	Putative uncharacterized protein
EOG091201KZ	APLA005249	Agrilus planipennis	Putative uncharacterized protein
EOG091201KZ	TC004356	Tribolium castaneum	Putative uncharacterized protein
EOG091201KZ	LDEC007579	Leptinotarsa decemlineata	Putative uncharacterized protein
EOG091201KZ	YQE_10749	Dendroctonus ponderosae	Putative uncharacterized protein
EOG091201L0	OTAU001937	Onthophagus taurus	Putative uncharacterized protein
EOG091201L0	AGLA004787	Anoplophora glabripennis	Putative uncharacterized protein
EOG091201L0	APLA004275	Agrilus planipennis	Putative uncharacterized protein
EOG091201L0	TC006187	Tribolium castaneum	Putative uncharacterized protein
EOG091201L0	LDEC011588	Leptinotarsa decemlineata	Putative uncharacterized protein
EOG091201L0	YQE_05803	Dendroctonus ponderosae	Putative uncharacterized protein
EOG091201L2	OTAU012595	Onthophagus taurus	metal ion binding
EOG091201L2	AGLA006259	Anoplophora glabripennis	metal ion binding
EOG091201L2	APLA009180	Agrilus planipennis	metal ion binding
EOG091201L2	TC032343	Tribolium castaneum	metal ion binding
EOG091201L2	LDEC010605	Leptinotarsa decemlineata	metal ion binding
EOG091201L2	YQE_03742	Dendroctonus ponderosae	metal ion binding
EOG091201L5	OTAU002638	Onthophagus taurus	Putative uncharacterized protein nuoF
EOG091201L5	AGLA008437	Anoplophora glabripennis	Putative uncharacterized protein nuoF
EOG091201L5	APLA002461	Agrilus planipennis	Putative uncharacterized protein nuoF
EOG091201L5	TC014119	Tribolium castaneum	Putative uncharacterized protein nuoF
EOG091201L5	LDEC021246	Leptinotarsa decemlineata	Putative uncharacterized protein nuoF
EOG091201L5	YQE_11061	Dendroctonus ponderosae	Putative uncharacterized protein nuoF
EOG091201L7	OTAU014530	Onthophagus taurus	PNR-like protein
EOG091201L7	AGLA004378	Anoplophora glabripennis	PNR-like protein
EOG091201L7	APLA004823	Agrilus planipennis	PNR-like protein
EOG091201L7	TC013148	Tribolium castaneum	PNR-like protein
EOG091201L7	LDEC002295	Leptinotarsa decemlineata	PNR-like protein
EOG091201L7	YQE_08220	Dendroctonus ponderosae	PNR-like protein
EOG091201L8	OTAU013156	Onthophagus taurus	Citrate synthase
EOG091201L8	AGLA017284	Anoplophora glabripennis	Citrate synthase
EOG091201L8	APLA010376	Agrilus planipennis	Citrate synthase
EOG091201L8	TC013214	Tribolium castaneum	Citrate synthase
EOG091201L8	LDEC003087	Leptinotarsa decemlineata	Citrate synthase
EOG091201L8	YQE_08575	Dendroctonus ponderosae	Citrate synthase
EOG091201L9	OTAU000427	Onthophagus taurus	Putative uncharacterized protein
EOG091201L9	AGLA017377	Anoplophora glabripennis	Putative uncharacterized protein
EOG091201L9	APLA002378	Agrilus planipennis	Putative uncharacterized protein
EOG091201L9	TC011920	Tribolium castaneum	Putative uncharacterized protein
EOG091201L9	LDEC013095	Leptinotarsa decemlineata	Putative uncharacterized protein
EOG091201L9	YQE_06474	Dendroctonus ponderosae	Putative uncharacterized protein
EOG091201LB	OTAU012958	Onthophagus taurus	Putative uncharacterized protein
EOG091201LB	AGLA002165	Anoplophora glabripennis	Putative uncharacterized protein
EOG091201LB	APLA005949	Agrilus planipennis	Putative uncharacterized protein
EOG091201LB	TC014236	Tribolium castaneum	Putative uncharacterized protein
EOG091201LB	LDEC001514	Leptinotarsa decemlineata	Putative uncharacterized protein
EOG091201LB	YQE_05972	Dendroctonus ponderosae	Putative uncharacterized protein
EOG091201LC	OTAU004512	Onthophagus taurus	Putative uncharacterized protein
EOG091201LC	AGLA005924	Anoplophora glabripennis	Putative uncharacterized protein
EOG091201LC	APLA001121	Agrilus planipennis	Putative uncharacterized protein
EOG091201LC	TC012601	Tribolium castaneum	Putative uncharacterized protein
EOG091201LC	LDEC011445	Leptinotarsa decemlineata	Putative uncharacterized protein
EOG091201LC	YQE_10434	Dendroctonus ponderosae	Putative uncharacterized protein

EOG091201LD	OTAU006798	Onthophagus taurus	Putative uncharacterized protein
EOG091201LD	AGLA018416	Anoplophora glabripennis	Putative uncharacterized protein
EOG091201LD	APLA006067	Agrilus planipennis	Putative uncharacterized protein
EOG091201LD	TC003020	Tribolium castaneum	Putative uncharacterized protein
EOG091201LD	LDEC005928	Leptinotarsa decemlineata	Putative uncharacterized protein
EOG091201LD	YQE_05151	Dendroctonus ponderosae	Putative uncharacterized protein
EOG091201LF	OTAU011738	Onthophagus taurus	Putative uncharacterized protein
EOG091201LF	AGLA014905	Anoplophora glabripennis	Putative uncharacterized protein
EOG091201LF	APLA003764	Agrilus planipennis	Putative uncharacterized protein
EOG091201LF	TC002523	Tribolium castaneum	Putative uncharacterized protein
EOG091201LF	LDEC016705	Leptinotarsa decemlineata	Putative uncharacterized protein
EOG091201LF	YQE_09865	Dendroctonus ponderosae	Putative uncharacterized protein
EOG091201LG	OTAU000551	Onthophagus taurus	Archipelago
EOG091201LG	AGLA018129	Anoplophora glabripennis	Archipelago
EOG091201LG	APLA013799	Agrilus planipennis	Archipelago
EOG091201LG	TC006451	Tribolium castaneum	Archipelago
EOG091201LG	LDEC018004	Leptinotarsa decemlineata	Archipelago
EOG091201LG	YQE_02181	Dendroctonus ponderosae	Archipelago
EOG091201LH	OTAU014817	Onthophagus taurus	heme binding
EOG091201LH	AGLA006145	Anoplophora glabripennis	heme binding
EOG091201LH	APLA008030	Agrilus planipennis	heme binding
EOG091201LH	TC032054	Tribolium castaneum	heme binding
EOG091201LH	LDEC010358	Leptinotarsa decemlineata	heme binding
EOG091201LH	YQE_02985	Dendroctonus ponderosae	heme binding
EOG091201LI	OTAU006504	Onthophagus taurus	Putative uncharacterized protein
EOG091201LI	AGLA010932	Anoplophora glabripennis	Putative uncharacterized protein
EOG091201LI	APLA003064	Agrilus planipennis	Putative uncharacterized protein
EOG091201LI	TC014142	Tribolium castaneum	Putative uncharacterized protein
EOG091201LI	LDEC007284	Leptinotarsa decemlineata	Putative uncharacterized protein
EOG091201LI	YQE_06308	Dendroctonus ponderosae	Putative uncharacterized protein
EOG091201LJ	OTAU009909	Onthophagus taurus	Putative uncharacterized protein
EOG091201LJ	AGLA002148	Anoplophora glabripennis	Putative uncharacterized protein
EOG091201LJ	APLA010647	Agrilus planipennis	Putative uncharacterized protein
EOG091201LJ	TC004691	Tribolium castaneum	Putative uncharacterized protein
EOG091201LJ	LDEC019834	Leptinotarsa decemlineata	Putative uncharacterized protein
EOG091201LJ	YQE_09262	Dendroctonus ponderosae	Putative uncharacterized protein
EOG091201LM	OTAU006670	Onthophagus taurus	Putative uncharacterized protein
EOG091201LM	AGLA002009	Anoplophora glabripennis	Putative uncharacterized protein
EOG091201LM	APLA007622	Agrilus planipennis	Putative uncharacterized protein
EOG091201LM	TC002476	Tribolium castaneum	Putative uncharacterized protein
EOG091201LM	LDEC004578	Leptinotarsa decemlineata	Putative uncharacterized protein
EOG091201LM	YQE_12387	Dendroctonus ponderosae	Putative uncharacterized protein
EOG091201LN	OTAU004489	Onthophagus taurus	Putative uncharacterized protein
EOG091201LN	AGLA007513	Anoplophora glabripennis	Putative uncharacterized protein
EOG091201LN	APLA000140	Agrilus planipennis	Putative uncharacterized protein
EOG091201LN	TC002763	Tribolium castaneum	Putative uncharacterized protein
EOG091201LN	LDEC013853	Leptinotarsa decemlineata	Putative uncharacterized protein
EOG091201LN	YQE_06157	Dendroctonus ponderosae	Putative uncharacterized protein
EOG091201LO	OTAU002059	Onthophagus taurus	nucleic acid binding
EOG091201LO	AGLA005459	Anoplophora glabripennis	nucleic acid binding
EOG091201LO	APLA011769	Agrilus planipennis	nucleic acid binding
EOG091201LO	TC032508	Tribolium castaneum	nucleic acid binding
EOG091201LO	LDEC009835	Leptinotarsa decemlineata	nucleic acid binding
EOG091201LO	YQE_10031	Dendroctonus ponderosae	nucleic acid binding
EOG091201LZ	OTAU002362	Onthophagus taurus	Putative uncharacterized protein
EOG091201LZ	AGLA014409	Anoplophora glabripennis	Putative uncharacterized protein
EOG091201LZ	APLA010113	Agrilus planipennis	Putative uncharacterized protein
EOG091201LZ	TC007746	Tribolium castaneum	Putative uncharacterized protein
EOG091201LZ	LDEC008310	Leptinotarsa decemlineata	Putative uncharacterized protein
EOG091201LZ	YQE_01494	Dendroctonus ponderosae	Putative uncharacterized protein
EOG091201M1	OTAU004537	Onthophagus taurus	DNA binding
EOG091201M1	AGLA002620	Anoplophora glabripennis	DNA binding
EOG091201M1	APLA003921	Agrilus planipennis	DNA binding
EOG091201M1	TC034342	Tribolium castaneum	DNA binding
EOG091201M1	LDEC001996	Leptinotarsa decemlineata	DNA binding
EOG091201M1	YQE_12847	Dendroctonus ponderosae	DNA binding
EOG091201M3	OTAU000106	Onthophagus taurus	Putative uncharacterized protein

EOG091201M3	AGLA016079	Anoplophora glabripennis	Putative uncharacterized protein
EOG091201M3	APLA008072	Agrilus planipennis	Putative uncharacterized protein
EOG091201M3	TC014136	Tribolium castaneum	Putative uncharacterized protein
EOG091201M3	LDEC014010	Leptinotarsa decemlineata	Putative uncharacterized protein
EOG091201M3	YQE_07279	Dendroctonus ponderosae	Putative uncharacterized protein
EOG091201M4	OTAU006644	Onthophagus taurus	Putative uncharacterized protein
EOG091201M4	AGLA009131	Anoplophora glabripennis	Putative uncharacterized protein
EOG091201M4	APLA011392	Agrilus planipennis	Putative uncharacterized protein
EOG091201M4	TC002991	Tribolium castaneum	Putative uncharacterized protein
EOG091201M4	LDEC005483	Leptinotarsa decemlineata	Putative uncharacterized protein
EOG091201M4	YQE_09760	Dendroctonus ponderosae	Putative uncharacterized protein
EOG091201M5	OTAU008763	Onthophagus taurus	Putative uncharacterized protein
EOG091201M5	AGLA003342	Anoplophora glabripennis	Putative uncharacterized protein
EOG091201M5	APLA005946	Agrilus planipennis	Putative uncharacterized protein
EOG091201M5	TC030775	Tribolium castaneum	Putative uncharacterized protein
EOG091201M5	LDEC007879	Leptinotarsa decemlineata	Putative uncharacterized protein
EOG091201M5	YQE_11751	Dendroctonus ponderosae	Putative uncharacterized protein
EOG091201M8	OTAU014773	Onthophagus taurus	Putative uncharacterized protein
EOG091201M8	AGLA010922	Anoplophora glabripennis	Putative uncharacterized protein
EOG091201M8	APLA003089	Agrilus planipennis	Putative uncharacterized protein
EOG091201M8	TC013056	Tribolium castaneum	Putative uncharacterized protein
EOG091201M8	LDEC007240	Leptinotarsa decemlineata	Putative uncharacterized protein
EOG091201M8	YQE_02074	Dendroctonus ponderosae	Putative uncharacterized protein
EOG091201M9	OTAU002243	Onthophagus taurus	Putative uncharacterized protein
EOG091201M9	AGLA018574	Anoplophora glabripennis	Putative uncharacterized protein
EOG091201M9	APLA012600	Agrilus planipennis	Putative uncharacterized protein
EOG091201M9	TC012299	Tribolium castaneum	Putative uncharacterized protein
EOG091201M9	LDEC003882	Leptinotarsa decemlineata	Putative uncharacterized protein
EOG091201M9	YQE_12935	Dendroctonus ponderosae	Putative uncharacterized protein
EOG091201MA	OTAU012921	Onthophagus taurus	Putative uncharacterized protein
EOG091201MA	AGLA007299	Anoplophora glabripennis	Putative uncharacterized protein
EOG091201MA	APLA000959	Agrilus planipennis	Putative uncharacterized protein
EOG091201MA	TC005574	Tribolium castaneum	Putative uncharacterized protein
EOG091201MA	LDEC010515	Leptinotarsa decemlineata	Putative uncharacterized protein
EOG091201MA	YQE_02843	Dendroctonus ponderosae	Putative uncharacterized protein
EOG091201MB	OTAU003516	Onthophagus taurus	Putative uncharacterized protein
EOG091201MB	AGLA007798	Anoplophora glabripennis	Putative uncharacterized protein
EOG091201MB	APLA014048	Agrilus planipennis	Putative uncharacterized protein
EOG091201MB	TC009035	Tribolium castaneum	Putative uncharacterized protein
EOG091201MB	LDEC005803	Leptinotarsa decemlineata	Putative uncharacterized protein
EOG091201MB	YQE_08828	Dendroctonus ponderosae	Putative uncharacterized protein
EOG091201MC	OTAU001450	Onthophagus taurus	Inosine-5'-monophosphate dehydrogenase
EOG091201MC	AGLA011262	Anoplophora glabripennis	Inosine-5'-monophosphate dehydrogenase
EOG091201MC	APLA003519	Agrilus planipennis	Inosine-5'-monophosphate dehydrogenase
EOG091201MC	TC005099	Tribolium castaneum	Inosine-5'-monophosphate dehydrogenase
EOG091201MC	LDEC003754	Leptinotarsa decemlineata	Inosine-5'-monophosphate dehydrogenase
EOG091201MC	YQE_06695	Dendroctonus ponderosae	Inosine-5'-monophosphate dehydrogenase
EOG091201MD	OTAU000472	Onthophagus taurus	Putative uncharacterized protein
EOG091201MD	AGLA019099	Anoplophora glabripennis	Putative uncharacterized protein
EOG091201MD	APLA004004	Agrilus planipennis	Putative uncharacterized protein
EOG091201MD	TC012273	Tribolium castaneum	Putative uncharacterized protein
EOG091201MD	LDEC013104	Leptinotarsa decemlineata	Putative uncharacterized protein
EOG091201MD	YQE_12954	Dendroctonus ponderosae	Putative uncharacterized protein
EOG091201ME	OTAU003877	Onthophagus taurus	Putative uncharacterized protein
EOG091201ME	AGLA008289	Anoplophora glabripennis	Putative uncharacterized protein
EOG091201ME	APLA005723	Agrilus planipennis	Putative uncharacterized protein
EOG091201ME	TC009470	Tribolium castaneum	Putative uncharacterized protein
EOG091201ME	LDEC001263	Leptinotarsa decemlineata	Putative uncharacterized protein
EOG091201ME	YQE_06006	Dendroctonus ponderosae	Putative uncharacterized protein
EOG091201MH	OTAU004545	Onthophagus taurus	catalytic activity
EOG091201MH	AGLA010059	Anoplophora glabripennis	catalytic activity
EOG091201MH	APLA010072	Agrilus planipennis	catalytic activity
EOG091201MH	TC033870	Tribolium castaneum	catalytic activity
EOG091201MH	LDEC015176	Leptinotarsa decemlineata	catalytic activity
EOG091201MH	YQE_12655	Dendroctonus ponderosae	catalytic activity
EOG091201MI	OTAU013208	Onthophagus taurus	None
EOG091201MI	AGLA013504	Anoplophora glabripennis	None

EOG091201MI	APLA011469	Agrilus planipennis	None
EOG091201MI	TC032390	Tribolium castaneum	None
EOG091201MI	LDEC016391	Leptinotarsa decemlineata	None
EOG091201MI	YQE_04964	Dendroctonus ponderosae	None
EOG091201MJ	OTAU003849	Onthophagus taurus	Putative uncharacterized protein
EOG091201MJ	AGLA017747	Anoplophora glabripennis	Putative uncharacterized protein
EOG091201MJ	APLA013639	Agrilus planipennis	Putative uncharacterized protein
EOG091201MJ	TC015929	Tribolium castaneum	Putative uncharacterized protein
EOG091201MJ	LDEC001267	Leptinotarsa decemlineata	Putative uncharacterized protein
EOG091201MJ	YQE_04298	Dendroctonus ponderosae	Putative uncharacterized protein
EOG091201ML	OTAU005723	Onthophagus taurus	Histone deacetylase
EOG091201ML	AGLA014902	Anoplophora glabripennis	Histone deacetylase
EOG091201ML	APLA005175	Agrilus planipennis	Histone deacetylase
EOG091201ML	TC002540	Tribolium castaneum	Histone deacetylase
EOG091201ML	LDEC021033	Leptinotarsa decemlineata	Histone deacetylase
EOG091201ML	YQE_09818	Dendroctonus ponderosae	Histone deacetylase
EOG091201MN	OTAU001327	Onthophagus taurus	Putative uncharacterized protein
EOG091201MN	AGLA000215	Anoplophora glabripennis	Putative uncharacterized protein
EOG091201MN	APLA009959	Agrilus planipennis	Putative uncharacterized protein
EOG091201MN	TC010886	Tribolium castaneum	Putative uncharacterized protein
EOG091201MN	LDEC001062	Leptinotarsa decemlineata	Putative uncharacterized protein
EOG091201MN	YQE_09077	Dendroctonus ponderosae	Putative uncharacterized protein
EOG091201MQ	OTAU001132	Onthophagus taurus	Putative uncharacterized protein
EOG091201MQ	AGLA010488	Anoplophora glabripennis	Putative uncharacterized protein
EOG091201MQ	APLA004323	Agrilus planipennis	Putative uncharacterized protein
EOG091201MQ	TC001032	Tribolium castaneum	Putative uncharacterized protein
EOG091201MQ	LDEC006035	Leptinotarsa decemlineata	Putative uncharacterized protein
EOG091201MQ	YQE_09574	Dendroctonus ponderosae	Putative uncharacterized protein
EOG091201MR	OTAU004958	Onthophagus taurus	Putative uncharacterized protein
EOG091201MR	AGLA014470	Anoplophora glabripennis	Putative uncharacterized protein
EOG091201MR	APLA015467	Agrilus planipennis	Putative uncharacterized protein
EOG091201MR	TC004399	Tribolium castaneum	Putative uncharacterized protein
EOG091201MR	LDEC007574	Leptinotarsa decemlineata	Putative uncharacterized protein
EOG091201MR	YQE_08081	Dendroctonus ponderosae	Putative uncharacterized protein
EOG091201MS	OTAU007712	Onthophagus taurus	Putative uncharacterized protein
EOG091201MS	AGLA006197	Anoplophora glabripennis	Putative uncharacterized protein
EOG091201MS	APLA001506	Agrilus planipennis	Putative uncharacterized protein
EOG091201MS	TC003573	Tribolium castaneum	Putative uncharacterized protein
EOG091201MS	LDEC014125	Leptinotarsa decemlineata	Putative uncharacterized protein
EOG091201MS	YQE_04545	Dendroctonus ponderosae	Putative uncharacterized protein
EOG091201MT	OTAU007811	Onthophagus taurus	Putative uncharacterized protein
EOG091201MT	AGLA002889	Anoplophora glabripennis	Putative uncharacterized protein
EOG091201MT	APLA009561	Agrilus planipennis	Putative uncharacterized protein
EOG091201MT	TC008012	Tribolium castaneum	Putative uncharacterized protein
EOG091201MT	LDEC000738	Leptinotarsa decemlineata	Putative uncharacterized protein
EOG091201MT	YQE_04522	Dendroctonus ponderosae	Putative uncharacterized protein
EOG091201MU	OTAU005455	Onthophagus taurus	Putative uncharacterized protein
EOG091201MU	AGLA010553	Anoplophora glabripennis	Putative uncharacterized protein
EOG091201MU	APLA002693	Agrilus planipennis	Putative uncharacterized protein
EOG091201MU	TC010914	Tribolium castaneum	Putative uncharacterized protein
EOG091201MU	LDEC005947	Leptinotarsa decemlineata	Putative uncharacterized protein
EOG091201MU	YQE_06219	Dendroctonus ponderosae	Putative uncharacterized protein
EOG091201MV	OTAU007236	Onthophagus taurus	Putative uncharacterized protein
EOG091201MV	AGLA007950	Anoplophora glabripennis	Putative uncharacterized protein
EOG091201MV	APLA006561	Agrilus planipennis	Putative uncharacterized protein
EOG091201MV	TC007110	Tribolium castaneum	Putative uncharacterized protein
EOG091201MV	LDEC008670	Leptinotarsa decemlineata	Putative uncharacterized protein
EOG091201MV	YQE_09632	Dendroctonus ponderosae	Putative uncharacterized protein
EOG091201MZ	OTAU012567	Onthophagus taurus	binding
EOG091201MZ	AGLA012005	Anoplophora glabripennis	binding
EOG091201MZ	APLA006689	Agrilus planipennis	binding
EOG091201MZ	TC032293	Tribolium castaneum	binding
EOG091201MZ	LDEC008781	Leptinotarsa decemlineata	binding
EOG091201MZ	YQE_06752	Dendroctonus ponderosae	binding
EOG091201N0	OTAU001908	Onthophagus taurus	None
EOG091201N0	AGLA008353	Anoplophora glabripennis	None
EOG091201N0	APLA008460	Agrilus planipennis	None

EOG091201N0	TC034036	Tribolium castaneum	None
EOG091201N0	LDEC010084	Leptinotarsa decemlineata	None
EOG091201N0	YQE_12109	Dendroctonus ponderosae	None
EOG091201N3	OTAU012570	Onthophagus taurus	binding
EOG091201N3	AGLA012003	Anoplophora glabripennis	binding
EOG091201N3	APLA006688	Agrilus planipennis	binding
EOG091201N3	TC031020	Tribolium castaneum	binding
EOG091201N3	LDEC008777	Leptinotarsa decemlineata	binding
EOG091201N3	YQE_06751	Dendroctonus ponderosae	binding
EOG091201N4	OTAU001702	Onthophagus taurus	Putative uncharacterized protein
EOG091201N4	AGLA001189	Anoplophora glabripennis	Putative uncharacterized protein
EOG091201N4	APLA000448	Agrilus planipennis	Putative uncharacterized protein
EOG091201N4	TC005345	Tribolium castaneum	Putative uncharacterized protein
EOG091201N4	LDEC020673	Leptinotarsa decemlineata	Putative uncharacterized protein
EOG091201N4	YQE_12063	Dendroctonus ponderosae	Putative uncharacterized protein
EOG091201N5	OTAU005668	Onthophagus taurus	Putative uncharacterized protein
EOG091201N5	AGLA018362	Anoplophora glabripennis	Putative uncharacterized protein
EOG091201N5	APLA014142	Agrilus planipennis	Putative uncharacterized protein
EOG091201N5	TC030740	Tribolium castaneum	Putative uncharacterized protein
EOG091201N5	LDEC019350	Leptinotarsa decemlineata	Putative uncharacterized protein
EOG091201N5	YQE_05292	Dendroctonus ponderosae	Putative uncharacterized protein
EOG091201N6	OTAU005194	Onthophagus taurus	Putative uncharacterized protein
EOG091201N6	AGLA000600	Anoplophora glabripennis	Putative uncharacterized protein
EOG091201N6	APLA002017	Agrilus planipennis	Putative uncharacterized protein
EOG091201N6	TC003849	Tribolium castaneum	Putative uncharacterized protein
EOG091201N6	LDEC015098	Leptinotarsa decemlineata	Putative uncharacterized protein
EOG091201N6	YQE_05143	Dendroctonus ponderosae	Putative uncharacterized protein
EOG091201N7	OTAU011839	Onthophagus taurus	Putative uncharacterized protein
EOG091201N7	AGLA003229	Anoplophora glabripennis	Putative uncharacterized protein
EOG091201N7	APLA007906	Agrilus planipennis	Putative uncharacterized protein
EOG091201N7	TC011043	Tribolium castaneum	Putative uncharacterized protein
EOG091201N7	LDEC024257	Leptinotarsa decemlineata	Putative uncharacterized protein
EOG091201N7	YQE_03203	Dendroctonus ponderosae	Putative uncharacterized protein
EOG091201NA	OTAU010196	Onthophagus taurus	Anoctamin
EOG091201NA	AGLA011130	Anoplophora glabripennis	Anoctamin
EOG091201NA	APLA004889	Agrilus planipennis	Anoctamin
EOG091201NA	TC014620	Tribolium castaneum	Anoctamin
EOG091201NA	LDEC019228	Leptinotarsa decemlineata	Anoctamin
EOG091201NA	YQE_07144	Dendroctonus ponderosae	Anoctamin
EOG091201NB	OTAU016795	Onthophagus taurus	Putative uncharacterized protein
EOG091201NB	AGLA002816	Anoplophora glabripennis	Putative uncharacterized protein
EOG091201NB	APLA001624	Agrilus planipennis	Putative uncharacterized protein
EOG091201NB	TC015892	Tribolium castaneum	Putative uncharacterized protein
EOG091201NB	LDEC007299	Leptinotarsa decemlineata	Putative uncharacterized protein
EOG091201NB	YQE_02195	Dendroctonus ponderosae	Putative uncharacterized protein
EOG091201NC	OTAU010831	Onthophagus taurus	Putative uncharacterized protein nuoD
EOG091201NC	AGLA003882	Anoplophora glabripennis	Putative uncharacterized protein nuoD
EOG091201NC	APLA012855	Agrilus planipennis	Putative uncharacterized protein nuoD
EOG091201NC	TC015217	Tribolium castaneum	Putative uncharacterized protein nuoD
EOG091201NC	LDEC008803	Leptinotarsa decemlineata	Putative uncharacterized protein nuoD
EOG091201NC	YQE_10117	Dendroctonus ponderosae	Putative uncharacterized protein nuoD
EOG091201ND	OTAU002966	Onthophagus taurus	Putative uncharacterized protein
EOG091201ND	AGLA018135	Anoplophora glabripennis	Putative uncharacterized protein
EOG091201ND	APLA015480	Agrilus planipennis	Putative uncharacterized protein
EOG091201ND	TC008904	Tribolium castaneum	Putative uncharacterized protein
EOG091201ND	LDEC002212	Leptinotarsa decemlineata	Putative uncharacterized protein
EOG091201ND	YQE_03464	Dendroctonus ponderosae	Putative uncharacterized protein
EOG091201NF	OTAU009169	Onthophagus taurus	Putative uncharacterized protein
EOG091201NF	AGLA009194	Anoplophora glabripennis	Putative uncharacterized protein
EOG091201NF	APLA006009	Agrilus planipennis	Putative uncharacterized protein
EOG091201NF	TC009441	Tribolium castaneum	Putative uncharacterized protein
EOG091201NF	LDEC015728	Leptinotarsa decemlineata	Putative uncharacterized protein
EOG091201NF	YQE_06017	Dendroctonus ponderosae	Putative uncharacterized protein
EOG091201NG	OTAU009784	Onthophagus taurus	Putative uncharacterized protein
EOG091201NG	AGLA004859	Anoplophora glabripennis	Putative uncharacterized protein
EOG091201NG	APLA013250	Agrilus planipennis	Putative uncharacterized protein
EOG091201NG	TC003027	Tribolium castaneum	Putative uncharacterized protein

EOG091201NG	LDEC020111	Leptinotarsa decemlineata	Putative uncharacterized protein
EOG091201NG	YQE_04561	Dendroctonus ponderosae	Putative uncharacterized protein
EOG091201NJ	OTAU009561	Onthophagus taurus	Putative uncharacterized protein
EOG091201NJ	AGLA003098	Anoplophora glabripennis	Putative uncharacterized protein
EOG091201NJ	APLA012682	Agrilus planipennis	Putative uncharacterized protein
EOG091201NJ	TC007711	Tribolium castaneum	Putative uncharacterized protein
EOG091201NJ	LDEC000050	Leptinotarsa decemlineata	Putative uncharacterized protein
EOG091201NJ	YQE_11991	Dendroctonus ponderosae	Putative uncharacterized protein
EOG091201NK	OTAU014788	Onthophagus taurus	Putative uncharacterized protein
EOG091201NK	AGLA006550	Anoplophora glabripennis	Putative uncharacterized protein
EOG091201NK	APLA009932	Agrilus planipennis	Putative uncharacterized protein
EOG091201NK	TC016196	Tribolium castaneum	Putative uncharacterized protein
EOG091201NK	LDEC001644	Leptinotarsa decemlineata	Putative uncharacterized protein
EOG091201NK	YQE_09477	Dendroctonus ponderosae	Putative uncharacterized protein
EOG091201NO	OTAU002045	Onthophagus taurus	Putative uncharacterized protein
EOG091201NO	AGLA011190	Anoplophora glabripennis	Putative uncharacterized protein
EOG091201NO	APLA003222	Agrilus planipennis	Putative uncharacterized protein
EOG091201NO	TC001667	Tribolium castaneum	Putative uncharacterized protein
EOG091201NO	LDEC016607	Leptinotarsa decemlineata	Putative uncharacterized protein
EOG091201NO	YQE_08026	Dendroctonus ponderosae	Putative uncharacterized protein
EOG091201NP	OTAU007915	Onthophagus taurus	Putative uncharacterized protein
EOG091201NP	AGLA017237	Anoplophora glabripennis	Putative uncharacterized protein
EOG091201NP	APLA000668	Agrilus planipennis	Putative uncharacterized protein
EOG091201NP	TC012378	Tribolium castaneum	Putative uncharacterized protein
EOG091201NP	LDEC007737	Leptinotarsa decemlineata	Putative uncharacterized protein
EOG091201NP	YQE_01945	Dendroctonus ponderosae	Putative uncharacterized protein
EOG091201NS	OTAU012444	Onthophagus taurus	Putative uncharacterized protein
EOG091201NS	AGLA001937	Anoplophora glabripennis	Putative uncharacterized protein
EOG091201NS	APLA007122	Agrilus planipennis	Putative uncharacterized protein
EOG091201NS	TC012786	Tribolium castaneum	Putative uncharacterized protein
EOG091201NS	LDEC014097	Leptinotarsa decemlineata	Putative uncharacterized protein
EOG091201NS	YQE_04339	Dendroctonus ponderosae	Putative uncharacterized protein
EOG091201NU	OTAU006674	Onthophagus taurus	Putative uncharacterized protein
EOG091201NU	AGLA004238	Anoplophora glabripennis	Putative uncharacterized protein
EOG091201NU	APLA007229	Agrilus planipennis	Putative uncharacterized protein
EOG091201NU	TC004014	Tribolium castaneum	Putative uncharacterized protein
EOG091201NU	LDEC008574	Leptinotarsa decemlineata	Putative uncharacterized protein
EOG091201NU	YQE_10834	Dendroctonus ponderosae	Putative uncharacterized protein
EOG091201NY	OTAU001991	Onthophagus taurus	Putative uncharacterized protein
EOG091201NY	AGLA000534	Anoplophora glabripennis	Putative uncharacterized protein
EOG091201NY	APLA007458	Agrilus planipennis	Putative uncharacterized protein
EOG091201NY	TC007912	Tribolium castaneum	Putative uncharacterized protein
EOG091201NY	LDEC015144	Leptinotarsa decemlineata	Putative uncharacterized protein
EOG091201NY	YQE_12559	Dendroctonus ponderosae	Putative uncharacterized protein
EOG091201O1	OTAU007117	Onthophagus taurus	Putative uncharacterized protein
EOG091201O1	AGLA000629	Anoplophora glabripennis	Putative uncharacterized protein
EOG091201O1	APLA013503	Agrilus planipennis	Putative uncharacterized protein
EOG091201O1	TC000825	Tribolium castaneum	Putative uncharacterized protein
EOG091201O1	LDEC003160	Leptinotarsa decemlineata	Putative uncharacterized protein
EOG091201O1	YQE_01807	Dendroctonus ponderosae	Putative uncharacterized protein
EOG091201O7	OTAU000933	Onthophagus taurus	Putative uncharacterized protein
EOG091201O7	AGLA011670	Anoplophora glabripennis	Putative uncharacterized protein
EOG091201O7	APLA011531	Agrilus planipennis	Putative uncharacterized protein
EOG091201O7	TC004458	Tribolium castaneum	Putative uncharacterized protein
EOG091201O7	LDEC009646	Leptinotarsa decemlineata	Putative uncharacterized protein
EOG091201O7	YQE_12181	Dendroctonus ponderosae	Putative uncharacterized protein
EOG091201O8	OTAU000606	Onthophagus taurus	Putative uncharacterized protein
EOG091201O8	AGLA013245	Anoplophora glabripennis	Putative uncharacterized protein
EOG091201O8	APLA007536	Agrilus planipennis	Putative uncharacterized protein
EOG091201O8	TC008829	Tribolium castaneum	Putative uncharacterized protein
EOG091201O8	LDEC007561	Leptinotarsa decemlineata	Putative uncharacterized protein
EOG091201O8	YQE_04287	Dendroctonus ponderosae	Putative uncharacterized protein
EOG091201OA	OTAU003117	Onthophagus taurus	Putative uncharacterized protein
EOG091201OA	AGLA019278	Anoplophora glabripennis	Putative uncharacterized protein
EOG091201OA	APLA009150	Agrilus planipennis	Putative uncharacterized protein
EOG091201OA	TC002959	Tribolium castaneum	Putative uncharacterized protein
EOG091201OA	LDEC018280	Leptinotarsa decemlineata	Putative uncharacterized protein

EOG091201OA	YQE_07399	Dendroctonus ponderosae	Putative uncharacterized protein
EOG091201OB	OTAU004346	Onthophagus taurus	Putative uncharacterized protein
EOG091201OB	AGLA004598	Anoplophora glabripennis	Putative uncharacterized protein
EOG091201OB	APLA003517	Agrilus planipennis	Putative uncharacterized protein
EOG091201OB	TC004606	Tribolium castaneum	Putative uncharacterized protein
EOG091201OB	LDEC017473	Leptinotarsa decemlineata	Putative uncharacterized protein
EOG091201OB	YQE_09150	Dendroctonus ponderosae	Putative uncharacterized protein
EOG091201OC	OTAU014084	Onthophagus taurus	Putative uncharacterized protein
EOG091201OC	AGLA007746	Anoplophora glabripennis	Putative uncharacterized protein
EOG091201OC	APLA011374	Agrilus planipennis	Putative uncharacterized protein
EOG091201OC	TC003915	Tribolium castaneum	Putative uncharacterized protein
EOG091201OC	LDEC004017	Leptinotarsa decemlineata	Putative uncharacterized protein
EOG091201OC	YQE_07628	Dendroctonus ponderosae	Putative uncharacterized protein
EOG091201OD	OTAU009444	Onthophagus taurus	Putative uncharacterized protein
EOG091201OD	AGLA013211	Anoplophora glabripennis	Putative uncharacterized protein
EOG091201OD	APLA001946	Agrilus planipennis	Putative uncharacterized protein
EOG091201OD	TC008441	Tribolium castaneum	Putative uncharacterized protein
EOG091201OD	LDEC009605	Leptinotarsa decemlineata	Putative uncharacterized protein
EOG091201OD	YQE_02767	Dendroctonus ponderosae	Putative uncharacterized protein
EOG091201OE	OTAU004858	Onthophagus taurus	Putative uncharacterized protein
EOG091201OE	AGLA004807	Anoplophora glabripennis	Putative uncharacterized protein
EOG091201OE	APLA000303	Agrilus planipennis	Putative uncharacterized protein
EOG091201OE	TC003580	Tribolium castaneum	Putative uncharacterized protein
EOG091201OE	LDEC003304	Leptinotarsa decemlineata	Putative uncharacterized protein
EOG091201OE	YQE_07878	Dendroctonus ponderosae	Putative uncharacterized protein
EOG091201OG	OTAU014162	Onthophagus taurus	Putative uncharacterized protein
EOG091201OG	AGLA007556	Anoplophora glabripennis	Putative uncharacterized protein
EOG091201OG	APLA010940	Agrilus planipennis	Putative uncharacterized protein
EOG091201OG	TC006544	Tribolium castaneum	Putative uncharacterized protein
EOG091201OG	LDEC006641	Leptinotarsa decemlineata	Putative uncharacterized protein
EOG091201OG	YQE_12211	Dendroctonus ponderosae	Putative uncharacterized protein
EOG091201OJ	OTAU007178	Onthophagus taurus	Olfactory receptor
EOG091201OJ	AGLA010310	Anoplophora glabripennis	Olfactory receptor
EOG091201OJ	APLA005895	Agrilus planipennis	Olfactory receptor
EOG091201OJ	TC015127	Tribolium castaneum	Olfactory receptor
EOG091201OJ	LDEC018616	Leptinotarsa decemlineata	Olfactory receptor
EOG091201OJ	YQE_10845	Dendroctonus ponderosae	Olfactory receptor
EOG091201OL	OTAU010524	Onthophagus taurus	Putative uncharacterized protein
EOG091201OL	AGLA009165	Anoplophora glabripennis	Putative uncharacterized protein
EOG091201OL	APLA015344	Agrilus planipennis	Putative uncharacterized protein
EOG091201OL	TC011523	Tribolium castaneum	Putative uncharacterized protein
EOG091201OL	LDEC010265	Leptinotarsa decemlineata	Putative uncharacterized protein
EOG091201OL	YQE_05130	Dendroctonus ponderosae	Putative uncharacterized protein
EOG091201ON	OTAU001431	Onthophagus taurus	Staufen
EOG091201ON	AGLA018670	Anoplophora glabripennis	Staufen
EOG091201ON	APLA009962	Agrilus planipennis	Staufen
EOG091201ON	TC004615	Tribolium castaneum	Staufen
EOG091201ON	LDEC004679	Leptinotarsa decemlineata	Staufen
EOG091201ON	YQE_06727	Dendroctonus ponderosae	Staufen
EOG091201OP	OTAU006853	Onthophagus taurus	Budding uninhibited by benzimidazoles 1
EOG091201OP	AGLA018638	Anoplophora glabripennis	Budding uninhibited by benzimidazoles 1
EOG091201OP	APLA007552	Agrilus planipennis	Budding uninhibited by benzimidazoles 1
EOG091201OP	TC014636	Tribolium castaneum	Budding uninhibited by benzimidazoles 1
EOG091201OP	LDEC007756	Leptinotarsa decemlineata	Budding uninhibited by benzimidazoles 1
EOG091201OP	YQE_07280	Dendroctonus ponderosae	Budding uninhibited by benzimidazoles 1
EOG091201OR	OTAU007416	Onthophagus taurus	Putative uncharacterized protein
EOG091201OR	AGLA018381	Anoplophora glabripennis	Putative uncharacterized protein
EOG091201OR	APLA005877	Agrilus planipennis	Putative uncharacterized protein
EOG091201OR	TC001089	Tribolium castaneum	Putative uncharacterized protein
EOG091201OR	LDEC005284	Leptinotarsa decemlineata	Putative uncharacterized protein
EOG091201OR	YQE_02375	Dendroctonus ponderosae	Putative uncharacterized protein
EOG091201OT	OTAU008392	Onthophagus taurus	Putative uncharacterized protein
EOG091201OT	AGLA003552	Anoplophora glabripennis	Putative uncharacterized protein
EOG091201OT	APLA012677	Agrilus planipennis	Putative uncharacterized protein
EOG091201OT	TC001560	Tribolium castaneum	Putative uncharacterized protein
EOG091201OT	LDEC016965	Leptinotarsa decemlineata	Putative uncharacterized protein
EOG091201OT	YQE_07413	Dendroctonus ponderosae	Putative uncharacterized protein

EOG091201OU	OTAU011606	Onthophagus taurus	Putative uncharacterized protein
EOG091201OU	AGLA012513	Anoplophora glabripennis	Putative uncharacterized protein
EOG091201OU	APLA004896	Agrilus planipennis	Putative uncharacterized protein
EOG091201OU	TC009185	Tribolium castaneum	Putative uncharacterized protein
EOG091201OU	LDEC012024	Leptinotarsa decemlineata	Putative uncharacterized protein
EOG091201OU	YQE_12598	Dendroctonus ponderosae	Putative uncharacterized protein
EOG091201OY	OTAU007517	Onthophagus taurus	Putative uncharacterized protein
EOG091201OY	AGLA009520	Anoplophora glabripennis	Putative uncharacterized protein
EOG091201OY	APLA013707	Agrilus planipennis	Putative uncharacterized protein
EOG091201OY	TC002427	Tribolium castaneum	Putative uncharacterized protein
EOG091201OY	LDEC009402	Leptinotarsa decemlineata	Putative uncharacterized protein
EOG091201OY	YQE_09834	Dendroctonus ponderosae	Putative uncharacterized protein
EOG091201OZ	OTAU005570	Onthophagus taurus	Putative uncharacterized protein
EOG091201OZ	AGLA000844	Anoplophora glabripennis	Putative uncharacterized protein
EOG091201OZ	APLA006240	Agrilus planipennis	Putative uncharacterized protein
EOG091201OZ	TC007938	Tribolium castaneum	Putative uncharacterized protein
EOG091201OZ	LDEC018653	Leptinotarsa decemlineata	Putative uncharacterized protein
EOG091201OZ	YQE_04740	Dendroctonus ponderosae	Putative uncharacterized protein
EOG091201P2	OTAU007317	Onthophagus taurus	Piwi
EOG091201P2	AGLA006316	Anoplophora glabripennis	Piwi
EOG091201P2	APLA014130	Agrilus planipennis	Piwi
EOG091201P2	TC008711	Tribolium castaneum	Piwi
EOG091201P2	LDEC013903	Leptinotarsa decemlineata	Piwi
EOG091201P2	YQE_11330	Dendroctonus ponderosae	Piwi
EOG091201P3	OTAU010420	Onthophagus taurus	Putative uncharacterized protein
EOG091201P3	AGLA008842	Anoplophora glabripennis	Putative uncharacterized protein
EOG091201P3	APLA015389	Agrilus planipennis	Putative uncharacterized protein
EOG091201P3	TC016079	Tribolium castaneum	Putative uncharacterized protein
EOG091201P3	LDEC005570	Leptinotarsa decemlineata	Putative uncharacterized protein
EOG091201P3	YQE_10777	Dendroctonus ponderosae	Putative uncharacterized protein
EOG091201P4	OTAU007554	Onthophagus taurus	Putative uncharacterized protein
EOG091201P4	AGLA012042	Anoplophora glabripennis	Putative uncharacterized protein
EOG091201P4	APLA008521	Agrilus planipennis	Putative uncharacterized protein
EOG091201P4	TC014247	Tribolium castaneum	Putative uncharacterized protein
EOG091201P4	LDEC010528	Leptinotarsa decemlineata	Putative uncharacterized protein
EOG091201P4	YQE_10449	Dendroctonus ponderosae	Putative uncharacterized protein
EOG091201P5	OTAU001867	Onthophagus taurus	Putative uncharacterized protein
EOG091201P5	AGLA012541	Anoplophora glabripennis	Putative uncharacterized protein
EOG091201P5	APLA007506	Agrilus planipennis	Putative uncharacterized protein
EOG091201P5	TC005983	Tribolium castaneum	Putative uncharacterized protein
EOG091201P5	LDEC018979	Leptinotarsa decemlineata	Putative uncharacterized protein
EOG091201P5	YQE_01896	Dendroctonus ponderosae	Putative uncharacterized protein
EOG091201P7	OTAU003248	Onthophagus taurus	Putative uncharacterized protein
EOG091201P7	AGLA009216	Anoplophora glabripennis	Putative uncharacterized protein
EOG091201P7	APLA001058	Agrilus planipennis	Putative uncharacterized protein
EOG091201P7	TC012775	Tribolium castaneum	Putative uncharacterized protein
EOG091201P7	LDEC000576	Leptinotarsa decemlineata	Putative uncharacterized protein
EOG091201P7	YQE_08085	Dendroctonus ponderosae	Putative uncharacterized protein
EOG091201P8	OTAU014559	Onthophagus taurus	Putative uncharacterized protein
EOG091201P8	AGLA003935	Anoplophora glabripennis	Putative uncharacterized protein
EOG091201P8	APLA002544	Agrilus planipennis	Putative uncharacterized protein
EOG091201P8	TC015419	Tribolium castaneum	Putative uncharacterized protein
EOG091201P8	LDEC016761	Leptinotarsa decemlineata	Putative uncharacterized protein
EOG091201P8	YQE_09391	Dendroctonus ponderosae	Putative uncharacterized protein
EOG091201PD	OTAU001759	Onthophagus taurus	Putative uncharacterized protein
EOG091201PD	AGLA004957	Anoplophora glabripennis	Putative uncharacterized protein
EOG091201PD	APLA000511	Agrilus planipennis	Putative uncharacterized protein
EOG091201PD	TC006298	Tribolium castaneum	Putative uncharacterized protein
EOG091201PD	LDEC008168	Leptinotarsa decemlineata	Putative uncharacterized protein
EOG091201PD	YQE_05579	Dendroctonus ponderosae	Putative uncharacterized protein
EOG091201PE	OTAU001818	Onthophagus taurus	Putative uncharacterized protein
EOG091201PE	AGLA018763	Anoplophora glabripennis	Putative uncharacterized protein
EOG091201PE	APLA000424	Agrilus planipennis	Putative uncharacterized protein
EOG091201PE	TC005396	Tribolium castaneum	Putative uncharacterized protein
EOG091201PE	LDEC009319	Leptinotarsa decemlineata	Putative uncharacterized protein
EOG091201PE	YQE_06941	Dendroctonus ponderosae	Putative uncharacterized protein
EOG091201PF	OTAU012420	Onthophagus taurus	Nebbish

EOG091201PF	AGLA007362	Anoplophora glabripennis	Nebbish
EOG091201PF	APLA004844	Agrilus planipennis	Nebbish
EOG091201PF	TC013493	Tribolium castaneum	Nebbish
EOG091201PF	LDEC015169	Leptinotarsa decemlineata	Nebbish
EOG091201PF	YQE_07162	Dendroctonus ponderosae	Nebbish
EOG091201PH	OTAU001140	Onthophagus taurus	Putative uncharacterized protein
EOG091201PH	AGLA013267	Anoplophora glabripennis	Putative uncharacterized protein
EOG091201PH	APLA013300	Agrilus planipennis	Putative uncharacterized protein
EOG091201PH	TC011052	Tribolium castaneum	Putative uncharacterized protein
EOG091201PH	LDEC006148	Leptinotarsa decemlineata	Putative uncharacterized protein
EOG091201PH	YQE_09338	Dendroctonus ponderosae	Putative uncharacterized protein
EOG091201PI	OTAU003612	Onthophagus taurus	None
EOG091201PI	AGLA015160	Anoplophora glabripennis	None
EOG091201PI	APLA003737	Agrilus planipennis	None
EOG091201PI	TC032487	Tribolium castaneum	None
EOG091201PI	LDEC009798	Leptinotarsa decemlineata	None
EOG091201PI	YQE_07909	Dendroctonus ponderosae	None
EOG091201PK	OTAU010649	Onthophagus taurus	Putative uncharacterized protein
EOG091201PK	AGLA017388	Anoplophora glabripennis	Putative uncharacterized protein
EOG091201PK	APLA000146	Agrilus planipennis	Putative uncharacterized protein
EOG091201PK	TC003007	Tribolium castaneum	Putative uncharacterized protein
EOG091201PK	LDEC021164	Leptinotarsa decemlineata	Putative uncharacterized protein
EOG091201PK	YQE_11695	Dendroctonus ponderosae	Putative uncharacterized protein
EOG091201PL	OTAU000033	Onthophagus taurus	Putative uncharacterized protein
EOG091201PL	AGLA019377	Anoplophora glabripennis	Putative uncharacterized protein
EOG091201PL	APLA012037	Agrilus planipennis	Putative uncharacterized protein
EOG091201PL	TC013189	Tribolium castaneum	Putative uncharacterized protein
EOG091201PL	LDEC007228	Leptinotarsa decemlineata	Putative uncharacterized protein
EOG091201PL	YQE_08209	Dendroctonus ponderosae	Putative uncharacterized protein
EOG091201PM	OTAU008022	Onthophagus taurus	Putative uncharacterized protein
EOG091201PM	AGLA016926	Anoplophora glabripennis	Putative uncharacterized protein
EOG091201PM	APLA005798	Agrilus planipennis	Putative uncharacterized protein
EOG091201PM	TC006662	Tribolium castaneum	Putative uncharacterized protein
EOG091201PM	LDEC009784	Leptinotarsa decemlineata	Putative uncharacterized protein
EOG091201PM	YQE_12173	Dendroctonus ponderosae	Putative uncharacterized protein
EOG091201PP	OTAU009686	Onthophagus taurus	Putative uncharacterized protein
EOG091201PP	AGLA003093	Anoplophora glabripennis	Putative uncharacterized protein
EOG091201PP	APLA015026	Agrilus planipennis	Putative uncharacterized protein
EOG091201PP	TC007497	Tribolium castaneum	Putative uncharacterized protein
EOG091201PP	LDEC012724	Leptinotarsa decemlineata	Putative uncharacterized protein
EOG091201PP	YQE_02302	Dendroctonus ponderosae	Putative uncharacterized protein
EOG091201PR	OTAU004086	Onthophagus taurus	Putative uncharacterized protein
EOG091201PR	AGLA008729	Anoplophora glabripennis	Putative uncharacterized protein
EOG091201PR	APLA014522	Agrilus planipennis	Putative uncharacterized protein
EOG091201PR	TC010040	Tribolium castaneum	Putative uncharacterized protein
EOG091201PR	LDEC004840	Leptinotarsa decemlineata	Putative uncharacterized protein
EOG091201PR	YQE_05740	Dendroctonus ponderosae	Putative uncharacterized protein
EOG091201PT	OTAU005153	Onthophagus taurus	Putative uncharacterized protein
EOG091201PT	AGLA013169	Anoplophora glabripennis	Putative uncharacterized protein
EOG091201PT	APLA014637	Agrilus planipennis	Putative uncharacterized protein
EOG091201PT	TC030737	Tribolium castaneum	Putative uncharacterized protein
EOG091201PT	LDEC000805	Leptinotarsa decemlineata	Putative uncharacterized protein
EOG091201PT	YQE_08938	Dendroctonus ponderosae	Putative uncharacterized protein
EOG091201PU	OTAU001304	Onthophagus taurus	Putative uncharacterized protein
EOG091201PU	AGLA013538	Anoplophora glabripennis	Putative uncharacterized protein
EOG091201PU	APLA005632	Agrilus planipennis	Putative uncharacterized protein
EOG091201PU	TC001888	Tribolium castaneum	Putative uncharacterized protein
EOG091201PU	LDEC018071	Leptinotarsa decemlineata	Putative uncharacterized protein
EOG091201PU	YQE_09327	Dendroctonus ponderosae	Putative uncharacterized protein
EOG091201PV	OTAU002914	Onthophagus taurus	Putative uncharacterized protein
EOG091201PV	AGLA020485	Anoplophora glabripennis	Putative uncharacterized protein
EOG091201PV	APLA010075	Agrilus planipennis	Putative uncharacterized protein
EOG091201PV	TC009786	Tribolium castaneum	Putative uncharacterized protein
EOG091201PV	LDEC005806	Leptinotarsa decemlineata	Putative uncharacterized protein
EOG091201PV	YQE_03508	Dendroctonus ponderosae	Putative uncharacterized protein
EOG091201PY	OTAU009482	Onthophagus taurus	Putative uncharacterized protein
EOG091201PY	AGLA013824	Anoplophora glabripennis	Putative uncharacterized protein

EOG091201PY	APLA004047	Agrilus planipennis	Putative uncharacterized protein
EOG091201PY	TC015374	Tribolium castaneum	Putative uncharacterized protein
EOG091201PY	LDEC001806	Leptinotarsa decemlineata	Putative uncharacterized protein
EOG091201PY	YQE_11464	Dendroctonus ponderosae	Putative uncharacterized protein
EOG091201PZ	OTAU011263	Onthophagus taurus	Putative uncharacterized protein
EOG091201PZ	AGLA012002	Anoplophora glabripennis	Putative uncharacterized protein
EOG091201PZ	APLA000317	Agrilus planipennis	Putative uncharacterized protein
EOG091201PZ	TC000091	Tribolium castaneum	Putative uncharacterized protein
EOG091201PZ	LDEC005179	Leptinotarsa decemlineata	Putative uncharacterized protein
EOG091201PZ	YQE_06814	Dendroctonus ponderosae	Putative uncharacterized protein
EOG091201Q3	OTAU002838	Onthophagus taurus	Putative uncharacterized protein
EOG091201Q3	AGLA000212	Anoplophora glabripennis	Putative uncharacterized protein
EOG091201Q3	APLA011536	Agrilus planipennis	Putative uncharacterized protein
EOG091201Q3	TC004579	Tribolium castaneum	Putative uncharacterized protein
EOG091201Q3	LDEC004512	Leptinotarsa decemlineata	Putative uncharacterized protein
EOG091201Q3	YQE_09253	Dendroctonus ponderosae	Putative uncharacterized protein
EOG091201Q4	OTAU009163	Onthophagus taurus	Putative uncharacterized protein
EOG091201Q4	AGLA016789	Anoplophora glabripennis	Putative uncharacterized protein
EOG091201Q4	APLA013228	Agrilus planipennis	Putative uncharacterized protein
EOG091201Q4	TC009439	Tribolium castaneum	Putative uncharacterized protein
EOG091201Q4	LDEC021752	Leptinotarsa decemlineata	Putative uncharacterized protein
EOG091201Q4	YQE_00078	Dendroctonus ponderosae	Putative uncharacterized protein
EOG091201Q8	OTAU001627	Onthophagus taurus	Putative uncharacterized protein
EOG091201Q8	AGLA016355	Anoplophora glabripennis	Putative uncharacterized protein
EOG091201Q8	APLA012305	Agrilus planipennis	Putative uncharacterized protein
EOG091201Q8	TC006495	Tribolium castaneum	Putative uncharacterized protein
EOG091201Q8	LDEC006315	Leptinotarsa decemlineata	Putative uncharacterized protein
EOG091201Q8	YQE_05818	Dendroctonus ponderosae	Putative uncharacterized protein
EOG091201Q9	OTAU002595	Onthophagus taurus	Putative uncharacterized protein
EOG091201Q9	AGLA010081	Anoplophora glabripennis	Putative uncharacterized protein
EOG091201Q9	APLA008626	Agrilus planipennis	Putative uncharacterized protein
EOG091201Q9	TC013339	Tribolium castaneum	Putative uncharacterized protein
EOG091201Q9	LDEC008012	Leptinotarsa decemlineata	Putative uncharacterized protein
EOG091201Q9	YQE_11772	Dendroctonus ponderosae	Putative uncharacterized protein
EOG091201QA	OTAU002892	Onthophagus taurus	Putative uncharacterized protein
EOG091201QA	AGLA011589	Anoplophora glabripennis	Putative uncharacterized protein
EOG091201QA	APLA014036	Agrilus planipennis	Putative uncharacterized protein
EOG091201QA	TC009163	Tribolium castaneum	Putative uncharacterized protein
EOG091201QA	LDEC011983	Leptinotarsa decemlineata	Putative uncharacterized protein
EOG091201QA	YQE_05256	Dendroctonus ponderosae	Putative uncharacterized protein
EOG091201QB	OTAU003099	Onthophagus taurus	TRPL
EOG091201QB	AGLA009153	Anoplophora glabripennis	TRPL
EOG091201QB	APLA006648	Agrilus planipennis	TRPL
EOG091201QB	TC005682	Tribolium castaneum	TRPL
EOG091201QB	LDEC010791	Leptinotarsa decemlineata	TRPL
EOG091201QB	YQE_03138	Dendroctonus ponderosae	TRPL
EOG091201QC	OTAU004417	Onthophagus taurus	Plenty of SH3s
EOG091201QC	AGLA000735	Anoplophora glabripennis	Plenty of SH3s
EOG091201QC	APLA006562	Agrilus planipennis	Plenty of SH3s
EOG091201QC	TC007357	Tribolium castaneum	Plenty of SH3s
EOG091201QC	LDEC005095	Leptinotarsa decemlineata	Plenty of SH3s
EOG091201QC	YQE_11443	Dendroctonus ponderosae	Plenty of SH3s
EOG091201QE	OTAU004827	Onthophagus taurus	Tubulin gamma chain
EOG091201QE	AGLA003481	Anoplophora glabripennis	Tubulin gamma chain
EOG091201QE	APLA002551	Agrilus planipennis	Tubulin gamma chain
EOG091201QE	TC014883	Tribolium castaneum	Tubulin gamma chain
EOG091201QE	LDEC008363	Leptinotarsa decemlineata	Tubulin gamma chain
EOG091201QE	YQE_02176	Dendroctonus ponderosae	Tubulin gamma chain
EOG091201QF	OTAU000581	Onthophagus taurus	Putative uncharacterized protein
EOG091201QF	AGLA002271	Anoplophora glabripennis	Putative uncharacterized protein
EOG091201QF	APLA011304	Agrilus planipennis	Putative uncharacterized protein
EOG091201QF	TC012731	Tribolium castaneum	Putative uncharacterized protein
EOG091201QF	LDEC023280	Leptinotarsa decemlineata	Putative uncharacterized protein
EOG091201QF	YQE_12815	Dendroctonus ponderosae	Putative uncharacterized protein
EOG091201QJ	OTAU007553	Onthophagus taurus	Putative uncharacterized protein
EOG091201QJ	AGLA007575	Anoplophora glabripennis	Putative uncharacterized protein
EOG091201QJ	APLA012892	Agrilus planipennis	Putative uncharacterized protein

EOG091201QJ	TC013202	Tribolium castaneum	Putative uncharacterized protein
EOG091201QJ	LDEC001150	Leptinotarsa decemlineata	Putative uncharacterized protein
EOG091201QJ	YQE_11495	Dendroctonus ponderosae	Putative uncharacterized protein
EOG091201QL	OTAU001072	Onthophagus taurus	Putative uncharacterized protein
EOG091201QL	AGLA006108	Anoplophora glabripennis	Putative uncharacterized protein
EOG091201QL	APLA005276	Agrilus planipennis	Putative uncharacterized protein
EOG091201QL	TC030585	Tribolium castaneum	Putative uncharacterized protein
EOG091201QL	LDEC013256	Leptinotarsa decemlineata	Putative uncharacterized protein
EOG091201QL	YQE_01701	Dendroctonus ponderosae	Putative uncharacterized protein
EOG091201QM	OTAU013825	Onthophagus taurus	Putative uncharacterized protein
EOG091201QM	AGLA012543	Anoplophora glabripennis	Putative uncharacterized protein
EOG091201QM	APLA006698	Agrilus planipennis	Putative uncharacterized protein
EOG091201QM	TC003322	Tribolium castaneum	Putative uncharacterized protein
EOG091201QM	LDEC017756	Leptinotarsa decemlineata	Putative uncharacterized protein
EOG091201QM	YQE_10602	Dendroctonus ponderosae	Putative uncharacterized protein
EOG091201QN	OTAU009745	Onthophagus taurus	Putative uncharacterized protein
EOG091201QN	AGLA000713	Anoplophora glabripennis	Putative uncharacterized protein
EOG091201QN	APLA010312	Agrilus planipennis	Putative uncharacterized protein
EOG091201QN	TC008353	Tribolium castaneum	Putative uncharacterized protein
EOG091201QN	LDEC006098	Leptinotarsa decemlineata	Putative uncharacterized protein
EOG091201QN	YQE_07094	Dendroctonus ponderosae	Putative uncharacterized protein
EOG091201QQ	OTAU014037	Onthophagus taurus	Pontin
EOG091201QQ	AGLA003160	Anoplophora glabripennis	Pontin
EOG091201QQ	APLA012604	Agrilus planipennis	Pontin
EOG091201QQ	TC012184	Tribolium castaneum	Pontin
EOG091201QQ	LDEC007618	Leptinotarsa decemlineata	Pontin
EOG091201QQ	YQE_10056	Dendroctonus ponderosae	Pontin
EOG091201QR	OTAU003000	Onthophagus taurus	Aminopeptidase-like protein
EOG091201QR	AGLA013862	Anoplophora glabripennis	Aminopeptidase-like protein
EOG091201QR	APLA006196	Agrilus planipennis	Aminopeptidase-like protein
EOG091201QR	TC009901	Tribolium castaneum	Aminopeptidase-like protein
EOG091201QR	LDEC015178	Leptinotarsa decemlineata	Aminopeptidase-like protein
EOG091201QR	YQE_05399	Dendroctonus ponderosae	Aminopeptidase-like protein
EOG091201QT	OTAU016293	Onthophagus taurus	Putative uncharacterized protein
EOG091201QT	AGLA018766	Anoplophora glabripennis	Putative uncharacterized protein
EOG091201QT	APLA007941	Agrilus planipennis	Putative uncharacterized protein
EOG091201QT	TC001100	Tribolium castaneum	Putative uncharacterized protein
EOG091201QT	LDEC006628	Leptinotarsa decemlineata	Putative uncharacterized protein
EOG091201QT	YQE_07073	Dendroctonus ponderosae	Putative uncharacterized protein
EOG091201QU	OTAU000122	Onthophagus taurus	Putative uncharacterized protein
EOG091201QU	AGLA021432	Anoplophora glabripennis	Putative uncharacterized protein
EOG091201QU	APLA005142	Agrilus planipennis	Putative uncharacterized protein
EOG091201QU	TC014789	Tribolium castaneum	Putative uncharacterized protein
EOG091201QU	LDEC005951	Leptinotarsa decemlineata	Putative uncharacterized protein
EOG091201QU	YQE_08234	Dendroctonus ponderosae	Putative uncharacterized protein
EOG091201QV	OTAU009540	Onthophagus taurus	Putative uncharacterized protein
EOG091201QV	AGLA000464	Anoplophora glabripennis	Putative uncharacterized protein
EOG091201QV	APLA011595	Agrilus planipennis	Putative uncharacterized protein
EOG091201QV	TC008915	Tribolium castaneum	Putative uncharacterized protein
EOG091201QV	LDEC000962	Leptinotarsa decemlineata	Putative uncharacterized protein
EOG091201QV	YQE_10576	Dendroctonus ponderosae	Putative uncharacterized protein
EOG091201QX	OTAU011888	Onthophagus taurus	Putative uncharacterized protein
EOG091201QX	AGLA020733	Anoplophora glabripennis	Putative uncharacterized protein
EOG091201QX	APLA003052	Agrilus planipennis	Putative uncharacterized protein
EOG091201QX	TC014631	Tribolium castaneum	Putative uncharacterized protein
EOG091201QX	LDEC005960	Leptinotarsa decemlineata	Putative uncharacterized protein
EOG091201QX	YQE_10456	Dendroctonus ponderosae	Putative uncharacterized protein
EOG091201QZ	OTAU006185	Onthophagus taurus	Putative uncharacterized protein
EOG091201QZ	AGLA005805	Anoplophora glabripennis	Putative uncharacterized protein
EOG091201QZ	APLA014395	Agrilus planipennis	Putative uncharacterized protein
EOG091201QZ	TC010150	Tribolium castaneum	Putative uncharacterized protein
EOG091201QZ	LDEC013178	Leptinotarsa decemlineata	Putative uncharacterized protein
EOG091201QZ	YQE_08775	Dendroctonus ponderosae	Putative uncharacterized protein
EOG091201R0	OTAU011124	Onthophagus taurus	Putative uncharacterized protein
EOG091201R0	AGLA005484	Anoplophora glabripennis	Putative uncharacterized protein
EOG091201R0	APLA002483	Agrilus planipennis	Putative uncharacterized protein
EOG091201R0	TC013150	Tribolium castaneum	Putative uncharacterized protein

EOG091201R0	LDEC003067	Leptinotarsa decemlineata	Putative uncharacterized protein
EOG091201R0	YQE_04875	Dendroctonus ponderosae	Putative uncharacterized protein
EOG091201RB	OTAU002581	Onthophagus taurus	None
EOG091201RB	AGLA013617	Anoplophora glabripennis	None
EOG091201RB	APLA009739	Agrilus planipennis	None
EOG091201RB	TC034685	Tribolium castaneum	None
EOG091201RB	LDEC002590	Leptinotarsa decemlineata	None
EOG091201RB	YQE_06342	Dendroctonus ponderosae	None
EOG091201RF	OTAU001457	Onthophagus taurus	Putative uncharacterized protein
EOG091201RF	AGLA000317	Anoplophora glabripennis	Putative uncharacterized protein
EOG091201RF	APLA010650	Agrilus planipennis	Putative uncharacterized protein
EOG091201RF	TC004701	Tribolium castaneum	Putative uncharacterized protein
EOG091201RF	LDEC021922	Leptinotarsa decemlineata	Putative uncharacterized protein
EOG091201RF	YQE_03236	Dendroctonus ponderosae	Putative uncharacterized protein
EOG091201RH	OTAU003834	Onthophagus taurus	Putative uncharacterized protein
EOG091201RH	AGLA011181	Anoplophora glabripennis	Putative uncharacterized protein
EOG091201RH	APLA000851	Agrilus planipennis	Putative uncharacterized protein
EOG091201RH	TC012802	Tribolium castaneum	Putative uncharacterized protein
EOG091201RH	LDEC000093	Leptinotarsa decemlineata	Putative uncharacterized protein
EOG091201RH	YQE_11418	Dendroctonus ponderosae	Putative uncharacterized protein
EOG091201RI	OTAU013922	Onthophagus taurus	Hemipterous
EOG091201RI	AGLA014139	Anoplophora glabripennis	Hemipterous
EOG091201RI	APLA010571	Agrilus planipennis	Hemipterous
EOG091201RI	TC000385	Tribolium castaneum	Hemipterous
EOG091201RI	LDEC001697	Leptinotarsa decemlineata	Hemipterous
EOG091201RI	YQE_11042	Dendroctonus ponderosae	Hemipterous
EOG091201RL	OTAU001407	Onthophagus taurus	Putative uncharacterized protein
EOG091201RL	AGLA002207	Anoplophora glabripennis	Putative uncharacterized protein
EOG091201RL	APLA003496	Agrilus planipennis	Putative uncharacterized protein
EOG091201RL	TC004647	Tribolium castaneum	Putative uncharacterized protein
EOG091201RL	LDEC006821	Leptinotarsa decemlineata	Putative uncharacterized protein
EOG091201RL	YQE_09121	Dendroctonus ponderosae	Putative uncharacterized protein
EOG091201RN	OTAU008886	Onthophagus taurus	Putative uncharacterized protein
EOG091201RN	AGLA010541	Anoplophora glabripennis	Putative uncharacterized protein
EOG091201RN	APLA011679	Agrilus planipennis	Putative uncharacterized protein
EOG091201RN	TC001193	Tribolium castaneum	Putative uncharacterized protein
EOG091201RN	LDEC007790	Leptinotarsa decemlineata	Putative uncharacterized protein
EOG091201RN	YQE_02786	Dendroctonus ponderosae	Putative uncharacterized protein
EOG091201RO	OTAU007495	Onthophagus taurus	Putative uncharacterized protein
EOG091201RO	AGLA010450	Anoplophora glabripennis	Putative uncharacterized protein
EOG091201RO	APLA009117	Agrilus planipennis	Putative uncharacterized protein
EOG091201RO	TC014904	Tribolium castaneum	Putative uncharacterized protein
EOG091201RO	LDEC010470	Leptinotarsa decemlineata	Putative uncharacterized protein
EOG091201RO	YQE_10859	Dendroctonus ponderosae	Putative uncharacterized protein
EOG091201RR	OTAU009568	Onthophagus taurus	Putative uncharacterized protein
EOG091201RR	AGLA014398	Anoplophora glabripennis	Putative uncharacterized protein
EOG091201RR	APLA008517	Agrilus planipennis	Putative uncharacterized protein
EOG091201RR	TC014707	Tribolium castaneum	Putative uncharacterized protein
EOG091201RR	LDEC012542	Leptinotarsa decemlineata	Putative uncharacterized protein
EOG091201RR	YQE_08587	Dendroctonus ponderosae	Putative uncharacterized protein
EOG091201RU	OTAU013568	Onthophagus taurus	Putative uncharacterized protein
EOG091201RU	AGLA016641	Anoplophora glabripennis	Putative uncharacterized protein
EOG091201RU	APLA007376	Agrilus planipennis	Putative uncharacterized protein
EOG091201RU	TC002097	Tribolium castaneum	Putative uncharacterized protein
EOG091201RU	LDEC020802	Leptinotarsa decemlineata	Putative uncharacterized protein
EOG091201RU	YQE_09472	Dendroctonus ponderosae	Putative uncharacterized protein
EOG091201RV	OTAU001827	Onthophagus taurus	Carboxypeptidase A
EOG091201RV	AGLA004985	Anoplophora glabripennis	Carboxypeptidase A
EOG091201RV	APLA001460	Agrilus planipennis	Carboxypeptidase A
EOG091201RV	TC005500	Tribolium castaneum	Carboxypeptidase A
EOG091201RV	LDEC003002	Leptinotarsa decemlineata	Carboxypeptidase A
EOG091201RV	YQE_07016	Dendroctonus ponderosae	Carboxypeptidase A
EOG091201RW	OTAU011467	Onthophagus taurus	None
EOG091201RW	AGLA005363	Anoplophora glabripennis	None
EOG091201RW	APLA002439	Agrilus planipennis	None
EOG091201RW	TC033672	Tribolium castaneum	None
EOG091201RW	LDEC017007	Leptinotarsa decemlineata	None

EOG091201RW	YQE_10629	Dendroctonus ponderosae	None
EOG091201RX	OTAU004583	Onthophagus taurus	Putative uncharacterized protein
EOG091201RX	AGLA000445	Anoplophora glabripennis	Putative uncharacterized protein
EOG091201RX	APLA002227	Agrilus planipennis	Putative uncharacterized protein
EOG091201RX	TC007873	Tribolium castaneum	Putative uncharacterized protein
EOG091201RX	LDEC018660	Leptinotarsa decemlineata	Putative uncharacterized protein
EOG091201RX	YQE_06457	Dendroctonus ponderosae	Putative uncharacterized protein
EOG091201RY	OTAU010998	Onthophagus taurus	Putative uncharacterized protein
EOG091201RY	AGLA011211	Anoplophora glabripennis	Putative uncharacterized protein
EOG091201RY	APLA008071	Agrilus planipennis	Putative uncharacterized protein
EOG091201RY	TC014370	Tribolium castaneum	Putative uncharacterized protein
EOG091201RY	LDEC005448	Leptinotarsa decemlineata	Putative uncharacterized protein
EOG091201RY	YQE_02021	Dendroctonus ponderosae	Putative uncharacterized protein
EOG091201RZ	OTAU008633	Onthophagus taurus	Putative uncharacterized protein
EOG091201RZ	AGLA012688	Anoplophora glabripennis	Putative uncharacterized protein
EOG091201RZ	APLA014102	Agrilus planipennis	Putative uncharacterized protein
EOG091201RZ	TC011929	Tribolium castaneum	Putative uncharacterized protein
EOG091201RZ	LDEC013638	Leptinotarsa decemlineata	Putative uncharacterized protein
EOG091201RZ	YQE_07609	Dendroctonus ponderosae	Putative uncharacterized protein
EOG091201S1	OTAU015763	Onthophagus taurus	Putative uncharacterized protein
EOG091201S1	AGLA011069	Anoplophora glabripennis	Putative uncharacterized protein
EOG091201S1	APLA000501	Agrilus planipennis	Putative uncharacterized protein
EOG091201S1	TC015981	Tribolium castaneum	Putative uncharacterized protein
EOG091201S1	LDEC020211	Leptinotarsa decemlineata	Putative uncharacterized protein
EOG091201S1	YQE_06883	Dendroctonus ponderosae	Putative uncharacterized protein
EOG091201S3	OTAU002870	Onthophagus taurus	Putative uncharacterized protein
EOG091201S3	AGLA005399	Anoplophora glabripennis	Putative uncharacterized protein
EOG091201S3	APLA010447	Agrilus planipennis	Putative uncharacterized protein
EOG091201S3	TC001697	Tribolium castaneum	Putative uncharacterized protein
EOG091201S3	LDEC017480	Leptinotarsa decemlineata	Putative uncharacterized protein
EOG091201S3	YQE_03317	Dendroctonus ponderosae	Putative uncharacterized protein
EOG091201S4	OTAU003776	Onthophagus taurus	Putative uncharacterized protein
EOG091201S4	AGLA008976	Anoplophora glabripennis	Putative uncharacterized protein
EOG091201S4	APLA014344	Agrilus planipennis	Putative uncharacterized protein
EOG091201S4	TC006044	Tribolium castaneum	Putative uncharacterized protein
EOG091201S4	LDEC001895	Leptinotarsa decemlineata	Putative uncharacterized protein
EOG091201S4	YQE_05962	Dendroctonus ponderosae	Putative uncharacterized protein
EOG091201S8	OTAU002483	Onthophagus taurus	Putative uncharacterized protein
EOG091201S8	AGLA003119	Anoplophora glabripennis	Putative uncharacterized protein
EOG091201S8	APLA004658	Agrilus planipennis	Putative uncharacterized protein
EOG091201S8	TC009058	Tribolium castaneum	Putative uncharacterized protein
EOG091201S8	LDEC020720	Leptinotarsa decemlineata	Putative uncharacterized protein
EOG091201S8	YQE_08812	Dendroctonus ponderosae	Putative uncharacterized protein
EOG091201S9	OTAU012254	Onthophagus taurus	Putative uncharacterized protein
EOG091201S9	AGLA011009	Anoplophora glabripennis	Putative uncharacterized protein
EOG091201S9	APLA006580	Agrilus planipennis	Putative uncharacterized protein
EOG091201S9	TC007147	Tribolium castaneum	Putative uncharacterized protein
EOG091201S9	LDEC001380	Leptinotarsa decemlineata	Putative uncharacterized protein
EOG091201S9	YQE_04254	Dendroctonus ponderosae	Putative uncharacterized protein
EOG091201SB	OTAU001289	Onthophagus taurus	Putative uncharacterized protein
EOG091201SB	AGLA003059	Anoplophora glabripennis	Putative uncharacterized protein
EOG091201SB	APLA010677	Agrilus planipennis	Putative uncharacterized protein
EOG091201SB	TC001437	Tribolium castaneum	Putative uncharacterized protein
EOG091201SB	LDEC017223	Leptinotarsa decemlineata	Putative uncharacterized protein
EOG091201SB	YQE_03560	Dendroctonus ponderosae	Putative uncharacterized protein
EOG091201SE	OTAU013210	Onthophagus taurus	Putative uncharacterized protein
EOG091201SE	AGLA013502	Anoplophora glabripennis	Putative uncharacterized protein
EOG091201SE	APLA011470	Agrilus planipennis	Putative uncharacterized protein
EOG091201SE	TC002963	Tribolium castaneum	Putative uncharacterized protein
EOG091201SE	LDEC007825	Leptinotarsa decemlineata	Putative uncharacterized protein
EOG091201SE	YQE_08614	Dendroctonus ponderosae	Putative uncharacterized protein
EOG091201SG	OTAU001652	Onthophagus taurus	Putative uncharacterized protein
EOG091201SG	AGLA011022	Anoplophora glabripennis	Putative uncharacterized protein
EOG091201SG	APLA005789	Agrilus planipennis	Putative uncharacterized protein
EOG091201SG	TC005477	Tribolium castaneum	Putative uncharacterized protein
EOG091201SG	LDEC015058	Leptinotarsa decemlineata	Putative uncharacterized protein
EOG091201SG	YQE_07464	Dendroctonus ponderosae	Putative uncharacterized protein

EOG091201SI	OTAU008739	Onthophagus taurus	Histone deacetylase
EOG091201SI	AGLA003899	Anoplophora glabripennis	Histone deacetylase
EOG091201SI	APLA000606	Agrilus planipennis	Histone deacetylase
EOG091201SI	TC006104	Tribolium castaneum	Histone deacetylase
EOG091201SI	LDEC003619	Leptinotarsa decemlineata	Histone deacetylase
EOG091201SI	YQE_02132	Dendroctonus ponderosae	Histone deacetylase
EOG091201SK	OTAU010391	Onthophagus taurus	Importin subunit alpha
EOG091201SK	AGLA011678	Anoplophora glabripennis	Importin subunit alpha
EOG091201SK	APLA004186	Agrilus planipennis	Importin subunit alpha
EOG091201SK	TC000505	Tribolium castaneum	Importin subunit alpha
EOG091201SK	LDEC014164	Leptinotarsa decemlineata	Importin subunit alpha
EOG091201SK	YQE_01907	Dendroctonus ponderosae	Importin subunit alpha
EOG091201SL	OTAU001364	Onthophagus taurus	Signal recognition particle subunit SRP72
EOG091201SL	AGLA000377	Anoplophora glabripennis	Signal recognition particle subunit SRP72
EOG091201SL	APLA010016	Agrilus planipennis	Signal recognition particle subunit SRP72
EOG091201SL	TC012172	Tribolium castaneum	Signal recognition particle subunit SRP72
EOG091201SL	LDEC018476	Leptinotarsa decemlineata	Signal recognition particle subunit SRP72
EOG091201SL	YQE_06702	Dendroctonus ponderosae	Signal recognition particle subunit SRP72
EOG091201SM	OTAU003061	Onthophagus taurus	Thickveins
EOG091201SM	AGLA017897	Anoplophora glabripennis	Thickveins
EOG091201SM	APLA004775	Agrilus planipennis	Thickveins
EOG091201SM	TC006474	Tribolium castaneum	Thickveins
EOG091201SM	LDEC016551	Leptinotarsa decemlineata	Thickveins
EOG091201SM	YQE_05546	Dendroctonus ponderosae	Thickveins
EOG091201SN	OTAU016427	Onthophagus taurus	Putative uncharacterized protein
EOG091201SN	AGLA005734	Anoplophora glabripennis	Putative uncharacterized protein
EOG091201SN	APLA000546	Agrilus planipennis	Putative uncharacterized protein
EOG091201SN	TC015052	Tribolium castaneum	Putative uncharacterized protein
EOG091201SN	LDEC010946	Leptinotarsa decemlineata	Putative uncharacterized protein
EOG091201SN	YQE_10828	Dendroctonus ponderosae	Putative uncharacterized protein
EOG091201SO	OTAU007881	Onthophagus taurus	Putative uncharacterized protein
EOG091201SO	AGLA021589	Anoplophora glabripennis	Putative uncharacterized protein
EOG091201SO	APLA013915	Agrilus planipennis	Putative uncharacterized protein
EOG091201SO	TC013210	Tribolium castaneum	Putative uncharacterized protein
EOG091201SO	LDEC017552	Leptinotarsa decemlineata	Putative uncharacterized protein
EOG091201SO	YQE_04843	Dendroctonus ponderosae	Putative uncharacterized protein
EOG091201SQ	OTAU007489	Onthophagus taurus	Putative uncharacterized protein
EOG091201SQ	AGLA013315	Anoplophora glabripennis	Putative uncharacterized protein
EOG091201SQ	APLA005941	Agrilus planipennis	Putative uncharacterized protein
EOG091201SQ	TC015308	Tribolium castaneum	Putative uncharacterized protein
EOG091201SQ	LDEC012218	Leptinotarsa decemlineata	Putative uncharacterized protein
EOG091201SQ	YQE_02115	Dendroctonus ponderosae	Putative uncharacterized protein
EOG091201SR	OTAU000888	Onthophagus taurus	Putative uncharacterized protein
EOG091201SR	AGLA000045	Anoplophora glabripennis	Putative uncharacterized protein
EOG091201SR	APLA005202	Agrilus planipennis	Putative uncharacterized protein
EOG091201SR	TC000967	Tribolium castaneum	Putative uncharacterized protein
EOG091201SR	LDEC008056	Leptinotarsa decemlineata	Putative uncharacterized protein
EOG091201SR	YQE_08470	Dendroctonus ponderosae	Putative uncharacterized protein
EOG091201SY	OTAU001810	Onthophagus taurus	Putative uncharacterized protein
EOG091201SY	AGLA004988	Anoplophora glabripennis	Putative uncharacterized protein
EOG091201SY	APLA001464	Agrilus planipennis	Putative uncharacterized protein
EOG091201SY	TC006625	Tribolium castaneum	Putative uncharacterized protein
EOG091201SY	LDEC002998	Leptinotarsa decemlineata	Putative uncharacterized protein
EOG091201SY	YQE_07017	Dendroctonus ponderosae	Putative uncharacterized protein
EOG091201T0	OTAU001899	Onthophagus taurus	None
EOG091201T0	AGLA005320	Anoplophora glabripennis	None
EOG091201T0	APLA001234	Agrilus planipennis	None
EOG091201T0	TC033993	Tribolium castaneum	None
EOG091201T0	LDEC005878	Leptinotarsa decemlineata	None
EOG091201T0	YQE_12717	Dendroctonus ponderosae	None
EOG091201T1	OTAU010724	Onthophagus taurus	None
EOG091201T1	AGLA015062	Anoplophora glabripennis	None
EOG091201T1	APLA004526	Agrilus planipennis	None
EOG091201T1	TC034177	Tribolium castaneum	None
EOG091201T1	LDEC004937	Leptinotarsa decemlineata	None
EOG091201T1	YQE_12186	Dendroctonus ponderosae	None
EOG091201T6	OTAU011876	Onthophagus taurus	Serpin peptidase inhibitor 2

EOG091201T6	AGLA001571	Anoplophora glabripennis	Serpin peptidase inhibitor 2
EOG091201T6	APLA001264	Agrilus planipennis	Serpin peptidase inhibitor 2
EOG091201T6	TC002085	Tribolium castaneum	Serpin peptidase inhibitor 2
EOG091201T6	LDEC014481	Leptinotarsa decemlineata	Serpin peptidase inhibitor 2
EOG091201T6	YQE_09354	Dendroctonus ponderosae	Serpin peptidase inhibitor 2
EOG091201T8	OTAU004739	Onthophagus taurus	Putative uncharacterized protein
EOG091201T8	AGLA001462	Anoplophora glabripennis	Putative uncharacterized protein
EOG091201T8	APLA008407	Agrilus planipennis	Putative uncharacterized protein
EOG091201T8	TC010324	Tribolium castaneum	Putative uncharacterized protein
EOG091201T8	LDEC001492	Leptinotarsa decemlineata	Putative uncharacterized protein
EOG091201T8	YQE_10867	Dendroctonus ponderosae	Putative uncharacterized protein
EOG091201T9	OTAU012703	Onthophagus taurus	Putative uncharacterized protein
EOG091201T9	AGLA000478	Anoplophora glabripennis	Putative uncharacterized protein
EOG091201T9	APLA000001	Agrilus planipennis	Putative uncharacterized protein
EOG091201T9	TC007578	Tribolium castaneum	Putative uncharacterized protein
EOG091201T9	LDEC013462	Leptinotarsa decemlineata	Putative uncharacterized protein
EOG091201T9	YQE_05659	Dendroctonus ponderosae	Putative uncharacterized protein
EOG091201TA	OTAU007711	Onthophagus taurus	Putative uncharacterized protein
EOG091201TA	AGLA003859	Anoplophora glabripennis	Putative uncharacterized protein
EOG091201TA	APLA001661	Agrilus planipennis	Putative uncharacterized protein
EOG091201TA	TC008820	Tribolium castaneum	Putative uncharacterized protein
EOG091201TA	LDEC020031	Leptinotarsa decemlineata	Putative uncharacterized protein
EOG091201TA	YQE_10796	Dendroctonus ponderosae	Putative uncharacterized protein
EOG091201TB	OTAU001561	Onthophagus taurus	Putative uncharacterized protein
EOG091201TB	AGLA004785	Anoplophora glabripennis	Putative uncharacterized protein
EOG091201TB	APLA000924	Agrilus planipennis	Putative uncharacterized protein
EOG091201TB	TC005714	Tribolium castaneum	Putative uncharacterized protein
EOG091201TB	LDEC009253	Leptinotarsa decemlineata	Putative uncharacterized protein
EOG091201TB	YQE_05801	Dendroctonus ponderosae	Putative uncharacterized protein
EOG091201TC	OTAU000735	Onthophagus taurus	Putative uncharacterized protein
EOG091201TC	AGLA004608	Anoplophora glabripennis	Putative uncharacterized protein
EOG091201TC	APLA003925	Agrilus planipennis	Putative uncharacterized protein
EOG091201TC	TC012523	Tribolium castaneum	Putative uncharacterized protein
EOG091201TC	LDEC017426	Leptinotarsa decemlineata	Putative uncharacterized protein
EOG091201TC	YQE_12983	Dendroctonus ponderosae	Putative uncharacterized protein
EOG091201TD	OTAU009186	Onthophagus taurus	Putative uncharacterized protein
EOG091201TD	AGLA005736	Anoplophora glabripennis	Putative uncharacterized protein
EOG091201TD	APLA000626	Agrilus planipennis	Putative uncharacterized protein
EOG091201TD	TC015317	Tribolium castaneum	Putative uncharacterized protein
EOG091201TD	LDEC006933	Leptinotarsa decemlineata	Putative uncharacterized protein
EOG091201TD	YQE_09435	Dendroctonus ponderosae	Putative uncharacterized protein
EOG091201TE	OTAU012578	Onthophagus taurus	Coronin
EOG091201TE	AGLA002975	Anoplophora glabripennis	Coronin
EOG091201TE	APLA011614	Agrilus planipennis	Coronin
EOG091201TE	TC004245	Tribolium castaneum	Coronin
EOG091201TE	LDEC006241	Leptinotarsa decemlineata	Coronin
EOG091201TE	YQE_08185	Dendroctonus ponderosae	Coronin
EOG091201TF	OTAU000672	Onthophagus taurus	Putative uncharacterized protein
EOG091201TF	AGLA002599	Anoplophora glabripennis	Putative uncharacterized protein
EOG091201TF	APLA014943	Agrilus planipennis	Putative uncharacterized protein
EOG091201TF	TC010652	Tribolium castaneum	Putative uncharacterized protein
EOG091201TF	LDEC002006	Leptinotarsa decemlineata	Putative uncharacterized protein
EOG091201TF	YQE_07563	Dendroctonus ponderosae	Putative uncharacterized protein
EOG091201TH	OTAU001137	Onthophagus taurus	HR4
EOG091201TH	AGLA010495	Anoplophora glabripennis	HR4
EOG091201TH	APLA001973	Agrilus planipennis	HR4
EOG091201TH	TC000543	Tribolium castaneum	HR4
EOG091201TH	LDEC010173	Leptinotarsa decemlineata	HR4
EOG091201TH	YQE_08945	Dendroctonus ponderosae	HR4
EOG091201TN	OTAU009476	Onthophagus taurus	Putative uncharacterized protein
EOG091201TN	AGLA012746	Anoplophora glabripennis	Putative uncharacterized protein
EOG091201TN	APLA002563	Agrilus planipennis	Putative uncharacterized protein
EOG091201TN	TC006112	Tribolium castaneum	Putative uncharacterized protein
EOG091201TN	LDEC001865	Leptinotarsa decemlineata	Putative uncharacterized protein
EOG091201TN	YQE_08380	Dendroctonus ponderosae	Putative uncharacterized protein
EOG091201TO	OTAU010671	Onthophagus taurus	Putative uncharacterized protein
EOG091201TO	AGLA020530	Anoplophora glabripennis	Putative uncharacterized protein

EOG091201TO	APLA001641	Agrilus planipennis	Putative uncharacterized protein
EOG091201TO	TC010153	Tribolium castaneum	Putative uncharacterized protein
EOG091201TO	LDEC012323	Leptinotarsa decemlineata	Putative uncharacterized protein
EOG091201TO	YQE_03500	Dendroctonus ponderosae	Putative uncharacterized protein
EOG091201TS	OTAU000353	Onthophagus taurus	Putative uncharacterized protein
EOG091201TS	AGLA005909	Anoplophora glabripennis	Putative uncharacterized protein
EOG091201TS	APLA010437	Agrilus planipennis	Putative uncharacterized protein
EOG091201TS	TC011945	Tribolium castaneum	Putative uncharacterized protein
EOG091201TS	LDEC006219	Leptinotarsa decemlineata	Putative uncharacterized protein
EOG091201TS	YQE_02887	Dendroctonus ponderosae	Putative uncharacterized protein
EOG091201TT	OTAU014081	Onthophagus taurus	Putative uncharacterized protein
EOG091201TT	AGLA007945	Anoplophora glabripennis	Putative uncharacterized protein
EOG091201TT	APLA011797	Agrilus planipennis	Putative uncharacterized protein
EOG091201TT	TC002698	Tribolium castaneum	Putative uncharacterized protein
EOG091201TT	LDEC010207	Leptinotarsa decemlineata	Putative uncharacterized protein
EOG091201TT	YQE_12855	Dendroctonus ponderosae	Putative uncharacterized protein
EOG091201TU	OTAU007685	Onthophagus taurus	Putative uncharacterized protein
EOG091201TU	AGLA005915	Anoplophora glabripennis	Putative uncharacterized protein
EOG091201TU	APLA011309	Agrilus planipennis	Putative uncharacterized protein
EOG091201TU	TC012873	Tribolium castaneum	Putative uncharacterized protein
EOG091201TU	LDEC014477	Leptinotarsa decemlineata	Putative uncharacterized protein
EOG091201TU	YQE_07951	Dendroctonus ponderosae	Putative uncharacterized protein
EOG091201TV	OTAU001197	Onthophagus taurus	Putative uncharacterized protein
EOG091201TV	AGLA001418	Anoplophora glabripennis	Putative uncharacterized protein
EOG091201TV	APLA011621	Agrilus planipennis	Putative uncharacterized protein
EOG091201TV	TC002673	Tribolium castaneum	Putative uncharacterized protein
EOG091201TV	LDEC011822	Leptinotarsa decemlineata	Putative uncharacterized protein
EOG091201TV	YQE_01592	Dendroctonus ponderosae	Putative uncharacterized protein
EOG091201TW	OTAU007540	Onthophagus taurus	Putative uncharacterized protein
EOG091201TW	AGLA014567	Anoplophora glabripennis	Putative uncharacterized protein
EOG091201TW	APLA013918	Agrilus planipennis	Putative uncharacterized protein
EOG091201TW	TC013995	Tribolium castaneum	Putative uncharacterized protein
EOG091201TW	LDEC006521	Leptinotarsa decemlineata	Putative uncharacterized protein
EOG091201TW	YQE_01801	Dendroctonus ponderosae	Putative uncharacterized protein
EOG091201TY	OTAU010459	Onthophagus taurus	Putative uncharacterized protein
EOG091201TY	AGLA000775	Anoplophora glabripennis	Putative uncharacterized protein
EOG091201TY	APLA010314	Agrilus planipennis	Putative uncharacterized protein
EOG091201TY	TC007671	Tribolium castaneum	Putative uncharacterized protein
EOG091201TY	LDEC020646	Leptinotarsa decemlineata	Putative uncharacterized protein
EOG091201TY	YQE_10331	Dendroctonus ponderosae	Putative uncharacterized protein
EOG091201TZ	OTAU002722	Onthophagus taurus	Putative uncharacterized protein
EOG091201TZ	AGLA011726	Anoplophora glabripennis	Putative uncharacterized protein
EOG091201TZ	APLA010639	Agrilus planipennis	Putative uncharacterized protein
EOG091201TZ	TC004630	Tribolium castaneum	Putative uncharacterized protein
EOG091201TZ	LDEC017517	Leptinotarsa decemlineata	Putative uncharacterized protein
EOG091201TZ	YQE_04835	Dendroctonus ponderosae	Putative uncharacterized protein
EOG091201U1	OTAU000226	Onthophagus taurus	Putative uncharacterized protein
EOG091201U1	AGLA006428	Anoplophora glabripennis	Putative uncharacterized protein
EOG091201U1	APLA007220	Agrilus planipennis	Putative uncharacterized protein
EOG091201U1	TC014029	Tribolium castaneum	Putative uncharacterized protein
EOG091201U1	LDEC007257	Leptinotarsa decemlineata	Putative uncharacterized protein
EOG091201U1	YQE_04022	Dendroctonus ponderosae	Putative uncharacterized protein
EOG091201U3	OTAU013354	Onthophagus taurus	Putative uncharacterized protein
EOG091201U3	AGLA006745	Anoplophora glabripennis	Putative uncharacterized protein
EOG091201U3	APLA004055	Agrilus planipennis	Putative uncharacterized protein
EOG091201U3	TC015652	Tribolium castaneum	Putative uncharacterized protein
EOG091201U3	LDEC012952	Leptinotarsa decemlineata	Putative uncharacterized protein
EOG091201U3	YQE_10814	Dendroctonus ponderosae	Putative uncharacterized protein
EOG091201U4	OTAU008757	Onthophagus taurus	Putative uncharacterized protein
EOG091201U4	AGLA008566	Anoplophora glabripennis	Putative uncharacterized protein
EOG091201U4	APLA003855	Agrilus planipennis	Putative uncharacterized protein
EOG091201U4	TC014954	Tribolium castaneum	Putative uncharacterized protein
EOG091201U4	LDEC013744	Leptinotarsa decemlineata	Putative uncharacterized protein
EOG091201U4	YQE_10799	Dendroctonus ponderosae	Putative uncharacterized protein
EOG091201U6	OTAU002970	Onthophagus taurus	Putative uncharacterized protein
EOG091201U6	AGLA015467	Anoplophora glabripennis	Putative uncharacterized protein
EOG091201U6	APLA012593	Agrilus planipennis	Putative uncharacterized protein

EOG091201U6	TC009675	Tribolium castaneum	Putative uncharacterized protein
EOG091201U6	LDEC015181	Leptinotarsa decemlineata	Putative uncharacterized protein
EOG091201U6	YQE_10284	Dendroctonus ponderosae	Putative uncharacterized protein
EOG091201U7	OTAU005193	Onthophagus taurus	Putative uncharacterized protein
EOG091201U7	AGLA000566	Anoplophora glabripennis	Putative uncharacterized protein
EOG091201U7	APLA000296	Agrilus planipennis	Putative uncharacterized protein
EOG091201U7	TC003142	Tribolium castaneum	Putative uncharacterized protein
EOG091201U7	LDEC003736	Leptinotarsa decemlineata	Putative uncharacterized protein
EOG091201U7	YQE_02747	Dendroctonus ponderosae	Putative uncharacterized protein
EOG091201U8	OTAU005642	Onthophagus taurus	None
EOG091201U8	AGLA011775	Anoplophora glabripennis	None
EOG091201U8	APLA011245	Agrilus planipennis	None
EOG091201U8	TC033638	Tribolium castaneum	None
EOG091201U8	LDEC004456	Leptinotarsa decemlineata	None
EOG091201U8	YQE_12449	Dendroctonus ponderosae	None
EOG091201U9	OTAU007559	Onthophagus taurus	Putative uncharacterized protein
EOG091201U9	AGLA020718	Anoplophora glabripennis	Putative uncharacterized protein
EOG091201U9	APLA004846	Agrilus planipennis	Putative uncharacterized protein
EOG091201U9	TC013859	Tribolium castaneum	Putative uncharacterized protein
EOG091201U9	LDEC014816	Leptinotarsa decemlineata	Putative uncharacterized protein
EOG091201U9	YQE_07157	Dendroctonus ponderosae	Putative uncharacterized protein
EOG091201UA	OTAU014090	Onthophagus taurus	Putative uncharacterized protein
EOG091201UA	AGLA014691	Anoplophora glabripennis	Putative uncharacterized protein
EOG091201UA	APLA012143	Agrilus planipennis	Putative uncharacterized protein
EOG091201UA	TC010916	Tribolium castaneum	Putative uncharacterized protein
EOG091201UA	LDEC013713	Leptinotarsa decemlineata	Putative uncharacterized protein
EOG091201UA	YQE_06112	Dendroctonus ponderosae	Putative uncharacterized protein
EOG091201UD	OTAU000846	Onthophagus taurus	Putative uncharacterized protein
EOG091201UD	AGLA011355	Anoplophora glabripennis	Putative uncharacterized protein
EOG091201UD	APLA012182	Agrilus planipennis	Putative uncharacterized protein
EOG091201UD	TC011059	Tribolium castaneum	Putative uncharacterized protein
EOG091201UD	LDEC016482	Leptinotarsa decemlineata	Putative uncharacterized protein
EOG091201UD	YQE_11206	Dendroctonus ponderosae	Putative uncharacterized protein
EOG091201UE	OTAU001048	Onthophagus taurus	hydrolase activity
EOG091201UE	AGLA009423	Anoplophora glabripennis	hydrolase activity
EOG091201UE	APLA001975	Agrilus planipennis	hydrolase activity
EOG091201UE	TC032215	Tribolium castaneum	hydrolase activity
EOG091201UE	LDEC000791	Leptinotarsa decemlineata	hydrolase activity
EOG091201UE	YQE_04243	Dendroctonus ponderosae	hydrolase activity
EOG091201UF	OTAU002200	Onthophagus taurus	Putative uncharacterized protein
EOG091201UF	AGLA004810	Anoplophora glabripennis	Putative uncharacterized protein
EOG091201UF	APLA008703	Agrilus planipennis	Putative uncharacterized protein
EOG091201UF	TC002857	Tribolium castaneum	Putative uncharacterized protein
EOG091201UF	LDEC020639	Leptinotarsa decemlineata	Putative uncharacterized protein
EOG091201UF	YQE_07893	Dendroctonus ponderosae	Putative uncharacterized protein
EOG091201UI	OTAU009337	Onthophagus taurus	Putative uncharacterized protein
EOG091201UI	AGLA006183	Anoplophora glabripennis	Putative uncharacterized protein
EOG091201UI	APLA007043	Agrilus planipennis	Putative uncharacterized protein
EOG091201UI	TC003004	Tribolium castaneum	Putative uncharacterized protein
EOG091201UI	LDEC007583	Leptinotarsa decemlineata	Putative uncharacterized protein
EOG091201UI	YQE_03665	Dendroctonus ponderosae	Putative uncharacterized protein
EOG091201UJ	OTAU009980	Onthophagus taurus	Putative uncharacterized protein
EOG091201UJ	AGLA009527	Anoplophora glabripennis	Putative uncharacterized protein
EOG091201UJ	APLA008524	Agrilus planipennis	Putative uncharacterized protein
EOG091201UJ	TC013372	Tribolium castaneum	Putative uncharacterized protein
EOG091201UJ	LDEC014772	Leptinotarsa decemlineata	Putative uncharacterized protein
EOG091201UJ	YQE_08235	Dendroctonus ponderosae	Putative uncharacterized protein
EOG091201UN	OTAU006608	Onthophagus taurus	Putative uncharacterized protein
EOG091201UN	AGLA016359	Anoplophora glabripennis	Putative uncharacterized protein
EOG091201UN	APLA003021	Agrilus planipennis	Putative uncharacterized protein
EOG091201UN	TC009819	Tribolium castaneum	Putative uncharacterized protein
EOG091201UN	LDEC009874	Leptinotarsa decemlineata	Putative uncharacterized protein
EOG091201UN	YQE_10559	Dendroctonus ponderosae	Putative uncharacterized protein
EOG091201UO	OTAU000844	Onthophagus taurus	Putative uncharacterized protein
EOG091201UO	AGLA000046	Anoplophora glabripennis	Putative uncharacterized protein
EOG091201UO	APLA009890	Agrilus planipennis	Putative uncharacterized protein
EOG091201UO	TC004849	Tribolium castaneum	Putative uncharacterized protein

EOG091201UO	LDEC008051	Leptinotarsa decemlineata	Putative uncharacterized protein
EOG091201UO	YQE_11623	Dendroctonus ponderosae	Putative uncharacterized protein
EOG091201UQ	OTAU004163	Onthophagus taurus	None
EOG091201UQ	AGLA010686	Anoplophora glabripennis	None
EOG091201UQ	APLA008511	Agrilus planipennis	None
EOG091201UQ	TC033082	Tribolium castaneum	None
EOG091201UQ	LDEC007272	Leptinotarsa decemlineata	None
EOG091201UQ	YQE_10338	Dendroctonus ponderosae	None
EOG091201UT	OTAU003296	Onthophagus taurus	Adenosylhomocysteinase
EOG091201UT	AGLA020868	Anoplophora glabripennis	Adenosylhomocysteinase
EOG091201UT	APLA006390	Agrilus planipennis	Adenosylhomocysteinase
EOG091201UT	TC001438	Tribolium castaneum	Adenosylhomocysteinase
EOG091201UT	LDEC001035	Leptinotarsa decemlineata	Adenosylhomocysteinase
EOG091201UT	YQE_09166	Dendroctonus ponderosae	Adenosylhomocysteinase
EOG091201UU	OTAU006832	Onthophagus taurus	Putative uncharacterized protein
EOG091201UU	AGLA004212	Anoplophora glabripennis	Putative uncharacterized protein
EOG091201UU	APLA014799	Agrilus planipennis	Putative uncharacterized protein
EOG091201UU	TC014767	Tribolium castaneum	Putative uncharacterized protein
EOG091201UU	LDEC002621	Leptinotarsa decemlineata	Putative uncharacterized protein
EOG091201UU	YQE_04870	Dendroctonus ponderosae	Putative uncharacterized protein
EOG091201UW	OTAU006957	Onthophagus taurus	Putative uncharacterized protein
EOG091201UW	AGLA015054	Anoplophora glabripennis	Putative uncharacterized protein
EOG091201UW	APLA001601	Agrilus planipennis	Putative uncharacterized protein
EOG091201UW	TC010100	Tribolium castaneum	Putative uncharacterized protein
EOG091201UW	LDEC016378	Leptinotarsa decemlineata	Putative uncharacterized protein
EOG091201UW	YQE_12458	Dendroctonus ponderosae	Putative uncharacterized protein
EOG091201UX	OTAU000956	Onthophagus taurus	Putative uncharacterized protein
EOG091201UX	AGLA001593	Anoplophora glabripennis	Putative uncharacterized protein
EOG091201UX	APLA004343	Agrilus planipennis	Putative uncharacterized protein
EOG091201UX	TC011038	Tribolium castaneum	Putative uncharacterized protein
EOG091201UX	LDEC001630	Leptinotarsa decemlineata	Putative uncharacterized protein
EOG091201UX	YQE_04770	Dendroctonus ponderosae	Putative uncharacterized protein
EOG091201UY	OTAU000180	Onthophagus taurus	Putative uncharacterized protein
EOG091201UY	AGLA006374	Anoplophora glabripennis	Putative uncharacterized protein
EOG091201UY	APLA002669	Agrilus planipennis	Putative uncharacterized protein
EOG091201UY	TC013033	Tribolium castaneum	Putative uncharacterized protein
EOG091201UY	LDEC001882	Leptinotarsa decemlineata	Putative uncharacterized protein
EOG091201UY	YQE_06063	Dendroctonus ponderosae	Putative uncharacterized protein
EOG091201V1	OTAU013035	Onthophagus taurus	Putative uncharacterized protein
EOG091201V1	AGLA010324	Anoplophora glabripennis	Putative uncharacterized protein
EOG091201V1	APLA009443	Agrilus planipennis	Putative uncharacterized protein
EOG091201V1	TC013699	Tribolium castaneum	Putative uncharacterized protein
EOG091201V1	LDEC008540	Leptinotarsa decemlineata	Putative uncharacterized protein
EOG091201V1	YQE_11671	Dendroctonus ponderosae	Putative uncharacterized protein
EOG091201V3	OTAU005059	Onthophagus taurus	Putative uncharacterized protein
EOG091201V3	AGLA007252	Anoplophora glabripennis	Putative uncharacterized protein
EOG091201V3	APLA007509	Agrilus planipennis	Putative uncharacterized protein
EOG091201V3	TC005903	Tribolium castaneum	Putative uncharacterized protein
EOG091201V3	LDEC002456	Leptinotarsa decemlineata	Putative uncharacterized protein
EOG091201V3	YQE_12748	Dendroctonus ponderosae	Putative uncharacterized protein
EOG091201V4	OTAU010853	Onthophagus taurus	None
EOG091201V4	AGLA002677	Anoplophora glabripennis	None
EOG091201V4	APLA008925	Agrilus planipennis	None
EOG091201V4	TC032177	Tribolium castaneum	None
EOG091201V4	LDEC005664	Leptinotarsa decemlineata	None
EOG091201V4	YQE_11911	Dendroctonus ponderosae	None
EOG091201V5	OTAU000037	Onthophagus taurus	Putative uncharacterized protein
EOG091201V5	AGLA017888	Anoplophora glabripennis	Putative uncharacterized protein
EOG091201V5	APLA002450	Agrilus planipennis	Putative uncharacterized protein
EOG091201V5	TC013686	Tribolium castaneum	Putative uncharacterized protein
EOG091201V5	LDEC016286	Leptinotarsa decemlineata	Putative uncharacterized protein
EOG091201V5	YQE_10004	Dendroctonus ponderosae	Putative uncharacterized protein
EOG091201VB	OTAU004309	Onthophagus taurus	Putative uncharacterized protein
EOG091201VB	AGLA012209	Anoplophora glabripennis	Putative uncharacterized protein
EOG091201VB	APLA010356	Agrilus planipennis	Putative uncharacterized protein
EOG091201VB	TC004711	Tribolium castaneum	Putative uncharacterized protein
EOG091201VB	LDEC004673	Leptinotarsa decemlineata	Putative uncharacterized protein

EOG091201VB	YQE_09243	Dendroctonus ponderosae	Putative uncharacterized protein
EOG091201VC	OTAU004260	Onthophagus taurus	Putative uncharacterized protein
EOG091201VC	AGLA007191	Anoplophora glabripennis	Putative uncharacterized protein
EOG091201VC	APLA007207	Agrilus planipennis	Putative uncharacterized protein
EOG091201VC	TC003645	Tribolium castaneum	Putative uncharacterized protein
EOG091201VC	LDEC006534	Leptinotarsa decemlineata	Putative uncharacterized protein
EOG091201VC	YQE_09825	Dendroctonus ponderosae	Putative uncharacterized protein
EOG091201VE	OTAU001750	Onthophagus taurus	Putative uncharacterized protein
EOG091201VE	AGLA007253	Anoplophora glabripennis	Putative uncharacterized protein
EOG091201VE	APLA004504	Agrilus planipennis	Putative uncharacterized protein
EOG091201VE	TC006260	Tribolium castaneum	Putative uncharacterized protein
EOG091201VE	LDEC002457	Leptinotarsa decemlineata	Putative uncharacterized protein
EOG091201VE	YQE_12058	Dendroctonus ponderosae	Putative uncharacterized protein
EOG091201VF	OTAU008983	Onthophagus taurus	Putative uncharacterized protein
EOG091201VF	AGLA017194	Anoplophora glabripennis	Putative uncharacterized protein
EOG091201VF	APLA012608	Agrilus planipennis	Putative uncharacterized protein
EOG091201VF	TC012374	Tribolium castaneum	Putative uncharacterized protein
EOG091201VF	LDEC015686	Leptinotarsa decemlineata	Putative uncharacterized protein
EOG091201VF	YQE_11606	Dendroctonus ponderosae	Putative uncharacterized protein
EOG091201VG	OTAU005536	Onthophagus taurus	None
EOG091201VG	AGLA011664	Anoplophora glabripennis	None
EOG091201VG	APLA013160	Agrilus planipennis	None
EOG091201VG	TC034331	Tribolium castaneum	None
EOG091201VG	LDEC007739	Leptinotarsa decemlineata	None
EOG091201VG	YQE_02877	Dendroctonus ponderosae	None
EOG091201VI	OTAU003853	Onthophagus taurus	Putative uncharacterized protein
EOG091201VI	AGLA006478	Anoplophora glabripennis	Putative uncharacterized protein
EOG091201VI	APLA006944	Agrilus planipennis	Putative uncharacterized protein
EOG091201VI	TC008957	Tribolium castaneum	Putative uncharacterized protein
EOG091201VI	LDEC004415	Leptinotarsa decemlineata	Putative uncharacterized protein
EOG091201VI	YQE_02208	Dendroctonus ponderosae	Putative uncharacterized protein
EOG091201VJ	OTAU007225	Onthophagus taurus	Putative uncharacterized protein
EOG091201VJ	AGLA017212	Anoplophora glabripennis	Putative uncharacterized protein
EOG091201VJ	APLA000723	Agrilus planipennis	Putative uncharacterized protein
EOG091201VJ	TC002972	Tribolium castaneum	Putative uncharacterized protein
EOG091201VJ	LDEC002037	Leptinotarsa decemlineata	Putative uncharacterized protein
EOG091201VJ	YQE_10314	Dendroctonus ponderosae	Putative uncharacterized protein
EOG091201VK	OTAU002023	Onthophagus taurus	metal ion binding
EOG091201VK	AGLA004402	Anoplophora glabripennis	metal ion binding
EOG091201VK	APLA001784	Agrilus planipennis	metal ion binding
EOG091201VK	TC034344	Tribolium castaneum	metal ion binding
EOG091201VK	LDEC012707	Leptinotarsa decemlineata	metal ion binding
EOG091201VK	YQE_11258	Dendroctonus ponderosae	metal ion binding
EOG091201VM	OTAU015661	Onthophagus taurus	Cytochrome P450 301A1
EOG091201VM	AGLA004483	Anoplophora glabripennis	Cytochrome P450 301A1
EOG091201VM	APLA013387	Agrilus planipennis	Cytochrome P450 301A1
EOG091201VM	TC008302	Tribolium castaneum	Cytochrome P450 301A1
EOG091201VM	LDEC006354	Leptinotarsa decemlineata	Cytochrome P450 301A1
EOG091201VM	YQE_02973	Dendroctonus ponderosae	Cytochrome P450 301A1
EOG091201VP	OTAU005163	Onthophagus taurus	Putative uncharacterized protein
EOG091201VP	AGLA013932	Anoplophora glabripennis	Putative uncharacterized protein
EOG091201VP	APLA002770	Agrilus planipennis	Putative uncharacterized protein
EOG091201VP	TC003564	Tribolium castaneum	Putative uncharacterized protein
EOG091201VP	LDEC020599	Leptinotarsa decemlineata	Putative uncharacterized protein
EOG091201VP	YQE_11127	Dendroctonus ponderosae	Putative uncharacterized protein
EOG091201VQ	OTAU012329	Onthophagus taurus	Putative uncharacterized protein
EOG091201VQ	AGLA006338	Anoplophora glabripennis	Putative uncharacterized protein
EOG091201VQ	APLA003606	Agrilus planipennis	Putative uncharacterized protein
EOG091201VQ	TC013201	Tribolium castaneum	Putative uncharacterized protein
EOG091201VQ	LDEC007870	Leptinotarsa decemlineata	Putative uncharacterized protein
EOG091201VQ	YQE_08408	Dendroctonus ponderosae	Putative uncharacterized protein
EOG091201VU	OTAU000208	Onthophagus taurus	Putative uncharacterized protein
EOG091201VU	AGLA016392	Anoplophora glabripennis	Putative uncharacterized protein
EOG091201VU	APLA006215	Agrilus planipennis	Putative uncharacterized protein
EOG091201VU	TC013893	Tribolium castaneum	Putative uncharacterized protein
EOG091201VU	LDEC008711	Leptinotarsa decemlineata	Putative uncharacterized protein
EOG091201VU	YQE_10490	Dendroctonus ponderosae	Putative uncharacterized protein

EOG091201VV	OTAU005478	Onthophagus taurus	Putative uncharacterized protein
EOG091201VV	AGLA010977	Anoplophora glabripennis	Putative uncharacterized protein
EOG091201VV	APLA002858	Agrilus planipennis	Putative uncharacterized protein
EOG091201VV	TC013937	Tribolium castaneum	Putative uncharacterized protein
EOG091201VV	LDEC009382	Leptinotarsa decemlineata	Putative uncharacterized protein
EOG091201VV	YQE_07180	Dendroctonus ponderosae	Putative uncharacterized protein
EOG091201VX	OTAU000074	Onthophagus taurus	None
EOG091201VX	AGLA014129	Anoplophora glabripennis	None
EOG091201VX	APLA014654	Agrilus planipennis	None
EOG091201VX	TC033027	Tribolium castaneum	None
EOG091201VX	LDEC010822	Leptinotarsa decemlineata	None
EOG091201VX	YQE_06293	Dendroctonus ponderosae	None
EOG091201VY	OTAU009180	Onthophagus taurus	Putative uncharacterized protein
EOG091201VY	AGLA008095	Anoplophora glabripennis	Putative uncharacterized protein
EOG091201VY	APLA001307	Agrilus planipennis	Putative uncharacterized protein
EOG091201VY	TC000312	Tribolium castaneum	Putative uncharacterized protein
EOG091201VY	LDEC006598	Leptinotarsa decemlineata	Putative uncharacterized protein
EOG091201VY	YQE_12871	Dendroctonus ponderosae	Putative uncharacterized protein
EOG091201W0	OTAU001392	Onthophagus taurus	Putative uncharacterized protein
EOG091201W0	AGLA004573	Anoplophora glabripennis	Putative uncharacterized protein
EOG091201W0	APLA003649	Agrilus planipennis	Putative uncharacterized protein
EOG091201W0	TC004607	Tribolium castaneum	Putative uncharacterized protein
EOG091201W0	LDEC001566	Leptinotarsa decemlineata	Putative uncharacterized protein
EOG091201W0	YQE_09290	Dendroctonus ponderosae	Putative uncharacterized protein
EOG091201W1	OTAU013808	Onthophagus taurus	Putative uncharacterized protein
EOG091201W1	AGLA011152	Anoplophora glabripennis	Putative uncharacterized protein
EOG091201W1	APLA007889	Agrilus planipennis	Putative uncharacterized protein
EOG091201W1	TC030673	Tribolium castaneum	Putative uncharacterized protein
EOG091201W1	LDEC018094	Leptinotarsa decemlineata	Putative uncharacterized protein
EOG091201W1	YQE_08090	Dendroctonus ponderosae	Putative uncharacterized protein
EOG091201W3	OTAU015901	Onthophagus taurus	Putative uncharacterized protein
EOG091201W3	AGLA004541	Anoplophora glabripennis	Putative uncharacterized protein
EOG091201W3	APLA003559	Agrilus planipennis	Putative uncharacterized protein
EOG091201W3	TC001738	Tribolium castaneum	Putative uncharacterized protein
EOG091201W3	LDEC012320	Leptinotarsa decemlineata	Putative uncharacterized protein
EOG091201W3	YQE_02391	Dendroctonus ponderosae	Putative uncharacterized protein
EOG091201W5	OTAU008477	Onthophagus taurus	Putative uncharacterized protein
EOG091201W5	AGLA000020	Anoplophora glabripennis	Putative uncharacterized protein
EOG091201W5	APLA001944	Agrilus planipennis	Putative uncharacterized protein
EOG091201W5	TC011106	Tribolium castaneum	Putative uncharacterized protein
EOG091201W5	LDEC012668	Leptinotarsa decemlineata	Putative uncharacterized protein
EOG091201W5	YQE_08189	Dendroctonus ponderosae	Putative uncharacterized protein
EOG091201W9	OTAU000614	Onthophagus taurus	Putative uncharacterized protein
EOG091201W9	AGLA002625	Anoplophora glabripennis	Putative uncharacterized protein
EOG091201W9	APLA003190	Agrilus planipennis	Putative uncharacterized protein
EOG091201W9	TC011637	Tribolium castaneum	Putative uncharacterized protein
EOG091201W9	LDEC002002	Leptinotarsa decemlineata	Putative uncharacterized protein
EOG091201W9	YQE_03163	Dendroctonus ponderosae	Putative uncharacterized protein
EOG091201WA	OTAU016196	Onthophagus taurus	Putative uncharacterized protein
EOG091201WA	AGLA002976	Anoplophora glabripennis	Putative uncharacterized protein
EOG091201WA	APLA011613	Agrilus planipennis	Putative uncharacterized protein
EOG091201WA	TC004234	Tribolium castaneum	Putative uncharacterized protein
EOG091201WA	LDEC006244	Leptinotarsa decemlineata	Putative uncharacterized protein
EOG091201WA	YQE_11644	Dendroctonus ponderosae	Putative uncharacterized protein
EOG091201WE	OTAU014751	Onthophagus taurus	metallocarboxypeptidase activity
EOG091201WE	AGLA001357	Anoplophora glabripennis	metallocarboxypeptidase activity
EOG091201WE	APLA001528	Agrilus planipennis	metallocarboxypeptidase activity
EOG091201WE	TC031795	Tribolium castaneum	metallocarboxypeptidase activity
EOG091201WE	LDEC019883	Leptinotarsa decemlineata	metallocarboxypeptidase activity
EOG091201WE	YQE_07080	Dendroctonus ponderosae	metallocarboxypeptidase activity
EOG091201WI	OTAU003555	Onthophagus taurus	Bride of sevenless
EOG091201WI	AGLA016104	Anoplophora glabripennis	Bride of sevenless
EOG091201WI	APLA002648	Agrilus planipennis	Bride of sevenless
EOG091201WI	TC002308	Tribolium castaneum	Bride of sevenless
EOG091201WI	LDEC003843	Leptinotarsa decemlineata	Bride of sevenless
EOG091201WI	YQE_08041	Dendroctonus ponderosae	Bride of sevenless
EOG091201WJ	OTAU013123	Onthophagus taurus	Putative uncharacterized protein

EOG091201WJ	AGLA009912	Anoplophora glabripennis	Putative uncharacterized protein
EOG091201WJ	APLA004494	Agrilus planipennis	Putative uncharacterized protein
EOG091201WJ	TC006492	Tribolium castaneum	Putative uncharacterized protein
EOG091201WJ	LDEC018963	Leptinotarsa decemlineata	Putative uncharacterized protein
EOG091201WJ	YQE_06867	Dendroctonus ponderosae	Putative uncharacterized protein
EOG091201WL	OTAU013363	Onthophagus taurus	catalytic activity
EOG091201WL	AGLA005732	Anoplophora glabripennis	catalytic activity
EOG091201WL	APLA010024	Agrilus planipennis	catalytic activity
EOG091201WL	TC033415	Tribolium castaneum	catalytic activity
EOG091201WL	LDEC010949	Leptinotarsa decemlineata	catalytic activity
EOG091201WL	YQE_10831	Dendroctonus ponderosae	catalytic activity
EOG091201WM	OTAU001336	Onthophagus taurus	Putative uncharacterized protein
EOG091201WM	AGLA013532	Anoplophora glabripennis	Putative uncharacterized protein
EOG091201WM	APLA005598	Agrilus planipennis	Putative uncharacterized protein
EOG091201WM	TC013598	Tribolium castaneum	Putative uncharacterized protein
EOG091201WM	LDEC001030	Leptinotarsa decemlineata	Putative uncharacterized protein
EOG091201WM	YQE_09199	Dendroctonus ponderosae	Putative uncharacterized protein
EOG091201WN	OTAU008767	Onthophagus taurus	Putative uncharacterized protein
EOG091201WN	AGLA003335	Anoplophora glabripennis	Putative uncharacterized protein
EOG091201WN	APLA012854	Agrilus planipennis	Putative uncharacterized protein
EOG091201WN	TC014953	Tribolium castaneum	Putative uncharacterized protein
EOG091201WN	LDEC007884	Leptinotarsa decemlineata	Putative uncharacterized protein
EOG091201WN	YQE_05900	Dendroctonus ponderosae	Putative uncharacterized protein
EOG091201WO	OTAU003178	Onthophagus taurus	Putative uncharacterized protein
EOG091201WO	AGLA012334	Anoplophora glabripennis	Putative uncharacterized protein
EOG091201WO	APLA011756	Agrilus planipennis	Putative uncharacterized protein
EOG091201WO	TC014317	Tribolium castaneum	Putative uncharacterized protein
EOG091201WO	LDEC008951	Leptinotarsa decemlineata	Putative uncharacterized protein
EOG091201WO	YQE_07299	Dendroctonus ponderosae	Putative uncharacterized protein
EOG091201WP	OTAU007563	Onthophagus taurus	Putative uncharacterized protein
EOG091201WP	AGLA008323	Anoplophora glabripennis	Putative uncharacterized protein
EOG091201WP	APLA002467	Agrilus planipennis	Putative uncharacterized protein
EOG091201WP	TC005069	Tribolium castaneum	Putative uncharacterized protein
EOG091201WP	LDEC014001	Leptinotarsa decemlineata	Putative uncharacterized protein
EOG091201WP	YQE_02679	Dendroctonus ponderosae	Putative uncharacterized protein
EOG091201WS	OTAU000389	Onthophagus taurus	Putative uncharacterized protein
EOG091201WS	AGLA011666	Anoplophora glabripennis	Putative uncharacterized protein
EOG091201WS	APLA010501	Agrilus planipennis	Putative uncharacterized protein
EOG091201WS	TC011841	Tribolium castaneum	Putative uncharacterized protein
EOG091201WS	LDEC007745	Leptinotarsa decemlineata	Putative uncharacterized protein
EOG091201WS	YQE_10057	Dendroctonus ponderosae	Putative uncharacterized protein
EOG091201WT	OTAU005819	Onthophagus taurus	Putative uncharacterized protein
EOG091201WT	AGLA001517	Anoplophora glabripennis	Putative uncharacterized protein
EOG091201WT	APLA003794	Agrilus planipennis	Putative uncharacterized protein
EOG091201WT	TC002628	Tribolium castaneum	Putative uncharacterized protein
EOG091201WT	LDEC004656	Leptinotarsa decemlineata	Putative uncharacterized protein
EOG091201WT	YQE_01847	Dendroctonus ponderosae	Putative uncharacterized protein
EOG091201WU	OTAU004503	Onthophagus taurus	Putative uncharacterized protein
EOG091201WU	AGLA017213	Anoplophora glabripennis	Putative uncharacterized protein
EOG091201WU	APLA008542	Agrilus planipennis	Putative uncharacterized protein
EOG091201WU	TC002994	Tribolium castaneum	Putative uncharacterized protein
EOG091201WU	LDEC002036	Leptinotarsa decemlineata	Putative uncharacterized protein
EOG091201WU	YQE_07830	Dendroctonus ponderosae	Putative uncharacterized protein
EOG091201X1	OTAU004350	Onthophagus taurus	Putative uncharacterized protein
EOG091201X1	AGLA006007	Anoplophora glabripennis	Putative uncharacterized protein
EOG091201X1	APLA002736	Agrilus planipennis	Putative uncharacterized protein
EOG091201X1	TC013179	Tribolium castaneum	Putative uncharacterized protein
EOG091201X1	LDEC004097	Leptinotarsa decemlineata	Putative uncharacterized protein
EOG091201X1	YQE_03995	Dendroctonus ponderosae	Putative uncharacterized protein
EOG091201X3	OTAU011715	Onthophagus taurus	Reptin
EOG091201X3	AGLA010478	Anoplophora glabripennis	Reptin
EOG091201X3	APLA000688	Agrilus planipennis	Reptin
EOG091201X3	TC015193	Tribolium castaneum	Reptin
EOG091201X3	LDEC009005	Leptinotarsa decemlineata	Reptin
EOG091201X3	YQE_08364	Dendroctonus ponderosae	Reptin
EOG091201X9	OTAU006479	Onthophagus taurus	Putative uncharacterized protein
EOG091201X9	AGLA003569	Anoplophora glabripennis	Putative uncharacterized protein

EOG091201X9	APLA013405	Agrilus planipennis	Putative uncharacterized protein
EOG091201X9	TC003349	Tribolium castaneum	Putative uncharacterized protein
EOG091201X9	LDEC000447	Leptinotarsa decemlineata	Putative uncharacterized protein
EOG091201X9	YQE_09795	Dendroctonus ponderosae	Putative uncharacterized protein
EOG091201XA	OTAU000433	Onthophagus taurus	catalytic activity
EOG091201XA	AGLA005128	Anoplophora glabripennis	catalytic activity
EOG091201XA	APLA002664	Agrilus planipennis	catalytic activity
EOG091201XA	TC034329	Tribolium castaneum	catalytic activity
EOG091201XA	LDEC018238	Leptinotarsa decemlineata	catalytic activity
EOG091201XA	YQE_12817	Dendroctonus ponderosae	catalytic activity
EOG091201XB	OTAU005408	Onthophagus taurus	Ubiquitin carboxyl-terminal hydrolase
EOG091201XB	AGLA007403	Anoplophora glabripennis	Ubiquitin carboxyl-terminal hydrolase
EOG091201XB	APLA010451	Agrilus planipennis	Ubiquitin carboxyl-terminal hydrolase
EOG091201XB	TC006701	Tribolium castaneum	Ubiquitin carboxyl-terminal hydrolase
EOG091201XB	LDEC021437	Leptinotarsa decemlineata	Ubiquitin carboxyl-terminal hydrolase
EOG091201XB	YQE_08386	Dendroctonus ponderosae	Ubiquitin carboxyl-terminal hydrolase
EOG091201XC	OTAU011941	Onthophagus taurus	Putative uncharacterized protein
EOG091201XC	AGLA008454	Anoplophora glabripennis	Putative uncharacterized protein
EOG091201XC	APLA010144	Agrilus planipennis	Putative uncharacterized protein
EOG091201XC	TC005758	Tribolium castaneum	Putative uncharacterized protein
EOG091201XC	LDEC020314	Leptinotarsa decemlineata	Putative uncharacterized protein
EOG091201XC	YQE_04890	Dendroctonus ponderosae	Putative uncharacterized protein
EOG091201XD	OTAU003777	Onthophagus taurus	Putative uncharacterized protein
EOG091201XD	AGLA009603	Anoplophora glabripennis	Putative uncharacterized protein
EOG091201XD	APLA014024	Agrilus planipennis	Putative uncharacterized protein
EOG091201XD	TC010139	Tribolium castaneum	Putative uncharacterized protein
EOG091201XD	LDEC002124	Leptinotarsa decemlineata	Putative uncharacterized protein
EOG091201XD	YQE_05238	Dendroctonus ponderosae	Putative uncharacterized protein
EOG091201XF	OTAU016085	Onthophagus taurus	hydrolase activity
EOG091201XF	AGLA013479	Anoplophora glabripennis	hydrolase activity
EOG091201XF	APLA002573	Agrilus planipennis	hydrolase activity
EOG091201XF	TC034727	Tribolium castaneum	hydrolase activity
EOG091201XF	LDEC007956	Leptinotarsa decemlineata	hydrolase activity
EOG091201XF	YQE_08388	Dendroctonus ponderosae	hydrolase activity
EOG091201XG	OTAU004527	Onthophagus taurus	Putative uncharacterized protein
EOG091201XG	AGLA017110	Anoplophora glabripennis	Putative uncharacterized protein
EOG091201XG	APLA003246	Agrilus planipennis	Putative uncharacterized protein
EOG091201XG	TC012612	Tribolium castaneum	Putative uncharacterized protein
EOG091201XG	LDEC007607	Leptinotarsa decemlineata	Putative uncharacterized protein
EOG091201XG	YQE_07736	Dendroctonus ponderosae	Putative uncharacterized protein
EOG091201XK	OTAU001426	Onthophagus taurus	Oxysterol-binding protein
EOG091201XK	AGLA000393	Anoplophora glabripennis	Oxysterol-binding protein
EOG091201XK	APLA009977	Agrilus planipennis	Oxysterol-binding protein
EOG091201XK	TC014227	Tribolium castaneum	Oxysterol-binding protein
EOG091201XK	LDEC004690	Leptinotarsa decemlineata	Oxysterol-binding protein
EOG091201XK	YQE_06638	Dendroctonus ponderosae	Oxysterol-binding protein
EOG091201XL	OTAU008202	Onthophagus taurus	Putative uncharacterized protein
EOG091201XL	AGLA015507	Anoplophora glabripennis	Putative uncharacterized protein
EOG091201XL	APLA007839	Agrilus planipennis	Putative uncharacterized protein
EOG091201XL	TC002669	Tribolium castaneum	Putative uncharacterized protein
EOG091201XL	LDEC013384	Leptinotarsa decemlineata	Putative uncharacterized protein
EOG091201XL	YQE_08191	Dendroctonus ponderosae	Putative uncharacterized protein
EOG091201XM	OTAU000032	Onthophagus taurus	Putative uncharacterized protein
EOG091201XM	AGLA011936	Anoplophora glabripennis	Putative uncharacterized protein
EOG091201XM	APLA006225	Agrilus planipennis	Putative uncharacterized protein
EOG091201XM	TC013691	Tribolium castaneum	Putative uncharacterized protein
EOG091201XM	LDEC016280	Leptinotarsa decemlineata	Putative uncharacterized protein
EOG091201XM	YQE_03990	Dendroctonus ponderosae	Putative uncharacterized protein
EOG091201XN	OTAU011057	Onthophagus taurus	Putative uncharacterized protein
EOG091201XN	AGLA014818	Anoplophora glabripennis	Putative uncharacterized protein
EOG091201XN	APLA008826	Agrilus planipennis	Putative uncharacterized protein
EOG091201XN	TC008998	Tribolium castaneum	Putative uncharacterized protein
EOG091201XN	LDEC004820	Leptinotarsa decemlineata	Putative uncharacterized protein
EOG091201XN	YQE_05402	Dendroctonus ponderosae	Putative uncharacterized protein
EOG091201XP	OTAU000741	Onthophagus taurus	None
EOG091201XP	AGLA006028	Anoplophora glabripennis	None
EOG091201XP	APLA003953	Agrilus planipennis	None

EOG091201XP	TC031766	Tribolium castaneum	None
EOG091201XP	LDEC011808	Leptinotarsa decemlineata	None
EOG091201XP	YQE_12986	Dendroctonus ponderosae	None
EOG091201XS	OTAU002950	Onthophagus taurus	catalytic activity
EOG091201XS	AGLA006820	Anoplophora glabripennis	catalytic activity
EOG091201XS	APLA001839	Agrilus planipennis	catalytic activity
EOG091201XS	TC031215	Tribolium castaneum	catalytic activity
EOG091201XS	LDEC021222	Leptinotarsa decemlineata	catalytic activity
EOG091201XS	YQE_10036	Dendroctonus ponderosae	catalytic activity
EOG091201XX	OTAU009276	Onthophagus taurus	zinc ion binding
EOG091201XX	AGLA006472	Anoplophora glabripennis	zinc ion binding
EOG091201XX	APLA008814	Agrilus planipennis	zinc ion binding
EOG091201XX	TC033713	Tribolium castaneum	zinc ion binding
EOG091201XX	LDEC004806	Leptinotarsa decemlineata	zinc ion binding
EOG091201XX	YQE_12492	Dendroctonus ponderosae	zinc ion binding
EOG091201Y0	OTAU000732	Onthophagus taurus	Putative uncharacterized protein
EOG091201Y0	AGLA004610	Anoplophora glabripennis	Putative uncharacterized protein
EOG091201Y0	APLA015157	Agrilus planipennis	Putative uncharacterized protein
EOG091201Y0	TC011954	Tribolium castaneum	Putative uncharacterized protein
EOG091201Y0	LDEC018316	Leptinotarsa decemlineata	Putative uncharacterized protein
EOG091201Y0	YQE_04141	Dendroctonus ponderosae	Putative uncharacterized protein
EOG091201Y2	OTAU008391	Onthophagus taurus	Germ cell-less
EOG091201Y2	AGLA003553	Anoplophora glabripennis	Germ cell-less
EOG091201Y2	APLA004107	Agrilus planipennis	Germ cell-less
EOG091201Y2	TC001571	Tribolium castaneum	Germ cell-less
EOG091201Y2	LDEC016964	Leptinotarsa decemlineata	Germ cell-less
EOG091201Y2	YQE_10208	Dendroctonus ponderosae	Germ cell-less
EOG091201Y3	OTAU004534	Onthophagus taurus	phospholipid binding
EOG091201Y3	AGLA001274	Anoplophora glabripennis	phospholipid binding
EOG091201Y3	APLA008637	Agrilus planipennis	phospholipid binding
EOG091201Y3	TC034405	Tribolium castaneum	phospholipid binding
EOG091201Y3	LDEC007716	Leptinotarsa decemlineata	phospholipid binding
EOG091201Y3	YQE_07781	Dendroctonus ponderosae	phospholipid binding
EOG091201Y4	OTAU014570	Onthophagus taurus	None
EOG091201Y4	AGLA017067	Anoplophora glabripennis	None
EOG091201Y4	APLA005489	Agrilus planipennis	None
EOG091201Y4	TC034004	Tribolium castaneum	None
EOG091201Y4	LDEC022055	Leptinotarsa decemlineata	None
EOG091201Y4	YQE_07004	Dendroctonus ponderosae	None
EOG091201Y5	OTAU006218	Onthophagus taurus	GABA-gated anion channel splice variant 3b6b
EOG091201Y5	AGLA003489	Anoplophora glabripennis	GABA-gated anion channel splice variant 3b6b
EOG091201Y5	APLA003750	Agrilus planipennis	GABA-gated anion channel splice variant 3b6b
EOG091201Y5	TC010482	Tribolium castaneum	GABA-gated anion channel splice variant 3b6b
EOG091201Y5	LDEC004037	Leptinotarsa decemlineata	GABA-gated anion channel splice variant 3b6b
EOG091201Y5	YQE_10244	Dendroctonus ponderosae	GABA-gated anion channel splice variant 3b6b
EOG091201Y7	OTAU005474	Onthophagus taurus	nucleic acid binding
EOG091201Y7	AGLA010820	Anoplophora glabripennis	nucleic acid binding
EOG091201Y7	APLA003621	Agrilus planipennis	nucleic acid binding
EOG091201Y7	TC031140	Tribolium castaneum	nucleic acid binding
EOG091201Y7	LDEC007262	Leptinotarsa decemlineata	nucleic acid binding
EOG091201Y7	YQE_06326	Dendroctonus ponderosae	nucleic acid binding
EOG091201Y8	OTAU011594	Onthophagus taurus	Putative uncharacterized protein
EOG091201Y8	AGLA007157	Anoplophora glabripennis	Putative uncharacterized protein
EOG091201Y8	APLA004341	Agrilus planipennis	Putative uncharacterized protein
EOG091201Y8	TC000707	Tribolium castaneum	Putative uncharacterized protein
EOG091201Y8	LDEC013506	Leptinotarsa decemlineata	Putative uncharacterized protein
EOG091201Y8	YQE_10418	Dendroctonus ponderosae	Putative uncharacterized protein
EOG091201YA	OTAU014407	Onthophagus taurus	Serine protease H51
EOG091201YA	AGLA007617	Anoplophora glabripennis	Serine protease H51
EOG091201YA	APLA012414	Agrilus planipennis	Serine protease H51
EOG091201YA	TC004622	Tribolium castaneum	Serine protease H51
EOG091201YA	LDEC013124	Leptinotarsa decemlineata	Serine protease H51
EOG091201YA	YQE_09071	Dendroctonus ponderosae	Serine protease H51
EOG091201YF	OTAU017171	Onthophagus taurus	Cytochrome P450 307A1
EOG091201YF	AGLA001167	Anoplophora glabripennis	Cytochrome P450 307A1
EOG091201YF	APLA009964	Agrilus planipennis	Cytochrome P450 307A1
EOG091201YF	TC004159	Tribolium castaneum	Cytochrome P450 307A1

EOG091201YF	LDEC007473	Leptinotarsa decemlineata	Cytochrome P450 307A1
EOG091201YF	YQE_06604	Dendroctonus ponderosae	Cytochrome P450 307A1
EOG091201YJ	OTAU015641	Onthophagus taurus	Inositol-3-phosphate synthase
EOG091201YJ	AGLA009759	Anoplophora glabripennis	Inositol-3-phosphate synthase
EOG091201YJ	APLA012083	Agrilus planipennis	Inositol-3-phosphate synthase
EOG091201YJ	TC030051	Tribolium castaneum	Inositol-3-phosphate synthase
EOG091201YJ	LDEC012299	Leptinotarsa decemlineata	Inositol-3-phosphate synthase
EOG091201YJ	YQE_12767	Dendroctonus ponderosae	Inositol-3-phosphate synthase
EOG091201YK	OTAU010876	Onthophagus taurus	Eukaryotic translation initiation factor 3 subunit E
EOG091201YK	AGLA021603	Anoplophora glabripennis	Eukaryotic translation initiation factor 3 subunit E
EOG091201YK	APLA015197	Agrilus planipennis	Eukaryotic translation initiation factor 3 subunit E
EOG091201YK	TC002738	Tribolium castaneum	Eukaryotic translation initiation factor 3 subunit E
EOG091201YK	LDEC024057	Leptinotarsa decemlineata	Eukaryotic translation initiation factor 3 subunit E
EOG091201YK	YQE_12325	Dendroctonus ponderosae	Eukaryotic translation initiation factor 3 subunit E
EOG091201YL	OTAU008191	Onthophagus taurus	Putative uncharacterized protein
EOG091201YL	AGLA013829	Anoplophora glabripennis	Putative uncharacterized protein
EOG091201YL	APLA014720	Agrilus planipennis	Putative uncharacterized protein
EOG091201YL	TC015048	Tribolium castaneum	Putative uncharacterized protein
EOG091201YL	LDEC001810	Leptinotarsa decemlineata	Putative uncharacterized protein
EOG091201YL	YQE_08403	Dendroctonus ponderosae	Putative uncharacterized protein
EOG091201YN	OTAU007762	Onthophagus taurus	Putative uncharacterized protein
EOG091201YN	AGLA009711	Anoplophora glabripennis	Putative uncharacterized protein
EOG091201YN	APLA011247	Agrilus planipennis	Putative uncharacterized protein
EOG091201YN	TC009390	Tribolium castaneum	Putative uncharacterized protein
EOG091201YN	LDEC006429	Leptinotarsa decemlineata	Putative uncharacterized protein
EOG091201YN	YQE_11358	Dendroctonus ponderosae	Putative uncharacterized protein
EOG091201YO	OTAU015265	Onthophagus taurus	Putative uncharacterized protein
EOG091201YO	AGLA002416	Anoplophora glabripennis	Putative uncharacterized protein
EOG091201YO	APLA011609	Agrilus planipennis	Putative uncharacterized protein
EOG091201YO	TC011321	Tribolium castaneum	Putative uncharacterized protein
EOG091201YO	LDEC009290	Leptinotarsa decemlineata	Putative uncharacterized protein
EOG091201YO	YQE_08163	Dendroctonus ponderosae	Putative uncharacterized protein
EOG091201YQ	OTAU005276	Onthophagus taurus	Putative uncharacterized protein
EOG091201YQ	AGLA000537	Anoplophora glabripennis	Putative uncharacterized protein
EOG091201YQ	APLA015187	Agrilus planipennis	Putative uncharacterized protein
EOG091201YQ	TC007094	Tribolium castaneum	Putative uncharacterized protein
EOG091201YQ	LDEC015146	Leptinotarsa decemlineata	Putative uncharacterized protein
EOG091201YQ	YQE_01116	Dendroctonus ponderosae	Putative uncharacterized protein
EOG091201YR	OTAU002087	Onthophagus taurus	helicase activity
EOG091201YR	AGLA006830	Anoplophora glabripennis	helicase activity
EOG091201YR	APLA007130	Agrilus planipennis	helicase activity
EOG091201YR	TC033693	Tribolium castaneum	helicase activity
EOG091201YR	LDEC015373	Leptinotarsa decemlineata	helicase activity
EOG091201YR	YQE_02207	Dendroctonus ponderosae	helicase activity
EOG091201YT	OTAU001978	Onthophagus taurus	Putative uncharacterized protein
EOG091201YT	AGLA012278	Anoplophora glabripennis	Putative uncharacterized protein
EOG091201YT	APLA014771	Agrilus planipennis	Putative uncharacterized protein
EOG091201YT	TC009963	Tribolium castaneum	Putative uncharacterized protein
EOG091201YT	LDEC013674	Leptinotarsa decemlineata	Putative uncharacterized protein
EOG091201YT	YQE_02253	Dendroctonus ponderosae	Putative uncharacterized protein
EOG091201YU	OTAU005544	Onthophagus taurus	Putative uncharacterized protein
EOG091201YU	AGLA004476	Anoplophora glabripennis	Putative uncharacterized protein
EOG091201YU	APLA012544	Agrilus planipennis	Putative uncharacterized protein
EOG091201YU	TC007682	Tribolium castaneum	Putative uncharacterized protein
EOG091201YU	LDEC011630	Leptinotarsa decemlineata	Putative uncharacterized protein
EOG091201YU	YQE_10934	Dendroctonus ponderosae	Putative uncharacterized protein
EOG091201YW	OTAU003300	Onthophagus taurus	Putative uncharacterized protein
EOG091201YW	AGLA002226	Anoplophora glabripennis	Putative uncharacterized protein
EOG091201YW	APLA006388	Agrilus planipennis	Putative uncharacterized protein
EOG091201YW	TC016056	Tribolium castaneum	Putative uncharacterized protein
EOG091201YW	LDEC017470	Leptinotarsa decemlineata	Putative uncharacterized protein
EOG091201YW	YQE_09224	Dendroctonus ponderosae	Putative uncharacterized protein
EOG091201YX	OTAU005406	Onthophagus taurus	Putative uncharacterized protein
EOG091201YX	AGLA000280	Anoplophora glabripennis	Putative uncharacterized protein
EOG091201YX	APLA010462	Agrilus planipennis	Putative uncharacterized protein
EOG091201YX	TC001458	Tribolium castaneum	Putative uncharacterized protein
EOG091201YX	LDEC011128	Leptinotarsa decemlineata	Putative uncharacterized protein

EOG091201YX	YQE_06672	Dendroctonus ponderosae	Putative uncharacterized protein
EOG091201YY	OTAU002634	Onthophagus taurus	Putative uncharacterized protein
EOG091201YY	AGLA006001	Anoplophora glabripennis	Putative uncharacterized protein
EOG091201YY	APLA002454	Agrilus planipennis	Putative uncharacterized protein
EOG091201YY	TC014113	Tribolium castaneum	Putative uncharacterized protein
EOG091201YY	LDEC015353	Leptinotarsa decemlineata	Putative uncharacterized protein
EOG091201YY	YQE_06329	Dendroctonus ponderosae	Putative uncharacterized protein
EOG091201YZ	OTAU003393	Onthophagus taurus	Putative uncharacterized protein
EOG091201YZ	AGLA013887	Anoplophora glabripennis	Putative uncharacterized protein
EOG091201YZ	APLA015201	Agrilus planipennis	Putative uncharacterized protein
EOG091201YZ	TC000113	Tribolium castaneum	Putative uncharacterized protein
EOG091201YZ	LDEC002694	Leptinotarsa decemlineata	Putative uncharacterized protein
EOG091201YZ	YQE_03673	Dendroctonus ponderosae	Putative uncharacterized protein
EOG091201Z0	OTAU006551	Onthophagus taurus	Putative uncharacterized protein
EOG091201Z0	AGLA006775	Anoplophora glabripennis	Putative uncharacterized protein
EOG091201Z0	APLA006043	Agrilus planipennis	Putative uncharacterized protein
EOG091201Z0	TC003147	Tribolium castaneum	Putative uncharacterized protein
EOG091201Z0	LDEC011549	Leptinotarsa decemlineata	Putative uncharacterized protein
EOG091201Z0	YQE_07321	Dendroctonus ponderosae	Putative uncharacterized protein
EOG091201Z1	OTAU000448	Onthophagus taurus	None
EOG091201Z1	AGLA009788	Anoplophora glabripennis	None
EOG091201Z1	APLA014326	Agrilus planipennis	None
EOG091201Z1	TC034311	Tribolium castaneum	None
EOG091201Z1	LDEC007741	Leptinotarsa decemlineata	None
EOG091201Z1	YQE_07977	Dendroctonus ponderosae	None
EOG091201Z2	OTAU016851	Onthophagus taurus	Putative uncharacterized protein
EOG091201Z2	AGLA016610	Anoplophora glabripennis	Putative uncharacterized protein
EOG091201Z2	APLA000345	Agrilus planipennis	Putative uncharacterized protein
EOG091201Z2	TC006643	Tribolium castaneum	Putative uncharacterized protein
EOG091201Z2	LDEC009840	Leptinotarsa decemlineata	Putative uncharacterized protein
EOG091201Z2	YQE_06975	Dendroctonus ponderosae	Putative uncharacterized protein
EOG091201Z5	OTAU005672	Onthophagus taurus	Putative uncharacterized protein
EOG091201Z5	AGLA018358	Anoplophora glabripennis	Putative uncharacterized protein
EOG091201Z5	APLA014143	Agrilus planipennis	Putative uncharacterized protein
EOG091201Z5	TC009918	Tribolium castaneum	Putative uncharacterized protein
EOG091201Z5	LDEC011853	Leptinotarsa decemlineata	Putative uncharacterized protein
EOG091201Z5	YQE_05296	Dendroctonus ponderosae	Putative uncharacterized protein
EOG091201Z6	OTAU004467	Onthophagus taurus	Putative uncharacterized protein
EOG091201Z6	AGLA006089	Anoplophora glabripennis	Putative uncharacterized protein
EOG091201Z6	APLA006505	Agrilus planipennis	Putative uncharacterized protein
EOG091201Z6	TC001580	Tribolium castaneum	Putative uncharacterized protein
EOG091201Z6	LDEC000456	Leptinotarsa decemlineata	Putative uncharacterized protein
EOG091201Z6	YQE_06396	Dendroctonus ponderosae	Putative uncharacterized protein
EOG091201Z8	OTAU000336	Onthophagus taurus	Putative uncharacterized protein
EOG091201Z8	AGLA011339	Anoplophora glabripennis	Putative uncharacterized protein
EOG091201Z8	APLA007391	Agrilus planipennis	Putative uncharacterized protein
EOG091201Z8	TC012533	Tribolium castaneum	Putative uncharacterized protein
EOG091201Z8	LDEC005752	Leptinotarsa decemlineata	Putative uncharacterized protein
EOG091201Z8	YQE_08048	Dendroctonus ponderosae	Putative uncharacterized protein
EOG091201ZA	OTAU000661	Onthophagus taurus	Putative uncharacterized protein
EOG091201ZA	AGLA012953	Anoplophora glabripennis	Putative uncharacterized protein
EOG091201ZA	APLA006614	Agrilus planipennis	Putative uncharacterized protein
EOG091201ZA	TC000087	Tribolium castaneum	Putative uncharacterized protein
EOG091201ZA	LDEC003377	Leptinotarsa decemlineata	Putative uncharacterized protein
EOG091201ZA	YQE_07693	Dendroctonus ponderosae	Putative uncharacterized protein
EOG091201ZC	OTAU005050	Onthophagus taurus	metal ion binding
EOG091201ZC	AGLA014984	Anoplophora glabripennis	metal ion binding
EOG091201ZC	APLA007493	Agrilus planipennis	metal ion binding
EOG091201ZC	TC033971	Tribolium castaneum	metal ion binding
EOG091201ZC	LDEC009856	Leptinotarsa decemlineata	metal ion binding
EOG091201ZC	YQE_12235	Dendroctonus ponderosae	metal ion binding
EOG091201ZH	OTAU001206	Onthophagus taurus	Putative uncharacterized protein
EOG091201ZH	AGLA000081	Anoplophora glabripennis	Putative uncharacterized protein
EOG091201ZH	APLA007695	Agrilus planipennis	Putative uncharacterized protein
EOG091201ZH	TC002076	Tribolium castaneum	Putative uncharacterized protein
EOG091201ZH	LDEC014173	Leptinotarsa decemlineata	Putative uncharacterized protein
EOG091201ZH	YQE_01635	Dendroctonus ponderosae	Putative uncharacterized protein

EOG091201ZJ	OTAU004931	Onthophagus taurus	Putative uncharacterized protein
EOG091201ZJ	AGLA015663	Anoplophora glabripennis	Putative uncharacterized protein
EOG091201ZJ	APLA005007	Agrilus planipennis	Putative uncharacterized protein
EOG091201ZJ	TC004402	Tribolium castaneum	Putative uncharacterized protein
EOG091201ZJ	LDEC012662	Leptinotarsa decemlineata	Putative uncharacterized protein
EOG091201ZJ	YQE_11011	Dendroctonus ponderosae	Putative uncharacterized protein
EOG091201ZN	OTAU000801	Onthophagus taurus	None
EOG091201ZN	AGLA013983	Anoplophora glabripennis	None
EOG091201ZN	APLA007717	Agrilus planipennis	None
EOG091201ZN	TC032118	Tribolium castaneum	None
EOG091201ZN	LDEC001714	Leptinotarsa decemlineata	None
EOG091201ZN	YQE_09585	Dendroctonus ponderosae	None
EOG091201ZP	OTAU000643	Onthophagus taurus	Putative uncharacterized protein
EOG091201ZP	AGLA015983	Anoplophora glabripennis	Putative uncharacterized protein
EOG091201ZP	APLA003969	Agrilus planipennis	Putative uncharacterized protein
EOG091201ZP	TC011772	Tribolium castaneum	Putative uncharacterized protein
EOG091201ZP	LDEC017014	Leptinotarsa decemlineata	Putative uncharacterized protein
EOG091201ZP	YQE_01879	Dendroctonus ponderosae	Putative uncharacterized protein
EOG091201ZR	OTAU001831	Onthophagus taurus	Putative uncharacterized protein
EOG091201ZR	AGLA005025	Anoplophora glabripennis	Putative uncharacterized protein
EOG091201ZR	APLA004285	Agrilus planipennis	Putative uncharacterized protein
EOG091201ZR	TC006611	Tribolium castaneum	Putative uncharacterized protein
EOG091201ZR	LDEC006746	Leptinotarsa decemlineata	Putative uncharacterized protein
EOG091201ZR	YQE_07488	Dendroctonus ponderosae	Putative uncharacterized protein
EOG091201ZT	OTAU000172	Onthophagus taurus	Putative uncharacterized protein
EOG091201ZT	AGLA015537	Anoplophora glabripennis	Putative uncharacterized protein
EOG091201ZT	APLA004998	Agrilus planipennis	Putative uncharacterized protein
EOG091201ZT	TC001383	Tribolium castaneum	Putative uncharacterized protein
EOG091201ZT	LDEC001989	Leptinotarsa decemlineata	Putative uncharacterized protein
EOG091201ZT	YQE_09598	Dendroctonus ponderosae	Putative uncharacterized protein
EOG091201ZW	OTAU013084	Onthophagus taurus	Putative uncharacterized protein
EOG091201ZW	AGLA013163	Anoplophora glabripennis	Putative uncharacterized protein
EOG091201ZW	APLA004299	Agrilus planipennis	Putative uncharacterized protein
EOG091201ZW	TC002221	Tribolium castaneum	Putative uncharacterized protein
EOG091201ZW	LDEC011192	Leptinotarsa decemlineata	Putative uncharacterized protein
EOG091201ZW	YQE_07525	Dendroctonus ponderosae	Putative uncharacterized protein
EOG091201ZX	OTAU002288	Onthophagus taurus	Putative uncharacterized protein
EOG091201ZX	AGLA004671	Anoplophora glabripennis	Putative uncharacterized protein
EOG091201ZX	APLA011453	Agrilus planipennis	Putative uncharacterized protein
EOG091201ZX	TC008241	Tribolium castaneum	Putative uncharacterized protein
EOG091201ZX	LDEC018542	Leptinotarsa decemlineata	Putative uncharacterized protein
EOG091201ZX	YQE_04643	Dendroctonus ponderosae	Putative uncharacterized protein
EOG09120200	OTAU005236	Onthophagus taurus	Putative uncharacterized protein
EOG09120200	AGLA001696	Anoplophora glabripennis	Putative uncharacterized protein
EOG09120200	APLA014778	Agrilus planipennis	Putative uncharacterized protein
EOG09120200	TC009425	Tribolium castaneum	Putative uncharacterized protein
EOG09120200	LDEC012445	Leptinotarsa decemlineata	Putative uncharacterized protein
EOG09120200	YQE_11787	Dendroctonus ponderosae	Putative uncharacterized protein
EOG09120201	OTAU005750	Onthophagus taurus	Putative uncharacterized protein
EOG09120201	AGLA000549	Anoplophora glabripennis	Putative uncharacterized protein
EOG09120201	APLA001479	Agrilus planipennis	Putative uncharacterized protein
EOG09120201	TC000267	Tribolium castaneum	Putative uncharacterized protein
EOG09120201	LDEC003719	Leptinotarsa decemlineata	Putative uncharacterized protein
EOG09120201	YQE_03644	Dendroctonus ponderosae	Putative uncharacterized protein
EOG09120202	OTAU007329	Onthophagus taurus	Putative uncharacterized protein
EOG09120202	AGLA002970	Anoplophora glabripennis	Putative uncharacterized protein
EOG09120202	APLA005337	Agrilus planipennis	Putative uncharacterized protein
EOG09120202	TC001510	Tribolium castaneum	Putative uncharacterized protein
EOG09120202	LDEC006254	Leptinotarsa decemlineata	Putative uncharacterized protein
EOG09120202	YQE_10927	Dendroctonus ponderosae	Putative uncharacterized protein
EOG09120205	OTAU008116	Onthophagus taurus	Putative uncharacterized protein
EOG09120205	AGLA006118	Anoplophora glabripennis	Putative uncharacterized protein
EOG09120205	APLA010772	Agrilus planipennis	Putative uncharacterized protein
EOG09120205	TC011201	Tribolium castaneum	Putative uncharacterized protein
EOG09120205	LDEC008685	Leptinotarsa decemlineata	Putative uncharacterized protein
EOG09120205	YQE_01680	Dendroctonus ponderosae	Putative uncharacterized protein
EOG09120206	OTAU003110	Onthophagus taurus	Putative uncharacterized protein

EOG09120206	AGLA017224	Anoplophora glabripennis	Putative uncharacterized protein
EOG09120206	APLA002533	Agrilus planipennis	Putative uncharacterized protein
EOG09120206	TC012306	Tribolium castaneum	Putative uncharacterized protein
EOG09120206	LDEC003855	Leptinotarsa decemlineata	Putative uncharacterized protein
EOG09120206	YQE_10561	Dendroctonus ponderosae	Putative uncharacterized protein
EOG09120208	OTAU008653	Onthophagus taurus	Putative uncharacterized protein
EOG09120208	AGLA017672	Anoplophora glabripennis	Putative uncharacterized protein
EOG09120208	APLA011000	Agrilus planipennis	Putative uncharacterized protein
EOG09120208	TC007047	Tribolium castaneum	Putative uncharacterized protein
EOG09120208	LDEC007114	Leptinotarsa decemlineata	Putative uncharacterized protein
EOG09120208	YQE_10530	Dendroctonus ponderosae	Putative uncharacterized protein
EOG0912020D	OTAU004454	Onthophagus taurus	Putative uncharacterized protein
EOG0912020D	AGLA000610	Anoplophora glabripennis	Putative uncharacterized protein
EOG0912020D	APLA006498	Agrilus planipennis	Putative uncharacterized protein
EOG0912020D	TC001549	Tribolium castaneum	Putative uncharacterized protein
EOG0912020D	LDEC000455	Leptinotarsa decemlineata	Putative uncharacterized protein
EOG0912020D	YQE_11130	Dendroctonus ponderosae	Putative uncharacterized protein
EOG0912020G	OTAU007611	Onthophagus taurus	Putative uncharacterized protein
EOG0912020G	AGLA002017	Anoplophora glabripennis	Putative uncharacterized protein
EOG0912020G	APLA002827	Agrilus planipennis	Putative uncharacterized protein
EOG0912020G	TC002471	Tribolium castaneum	Putative uncharacterized protein
EOG0912020G	LDEC019756	Leptinotarsa decemlineata	Putative uncharacterized protein
EOG0912020G	YQE_09952	Dendroctonus ponderosae	Putative uncharacterized protein
EOG0912020I	OTAU009347	Onthophagus taurus	Putative uncharacterized protein
EOG0912020I	AGLA005993	Anoplophora glabripennis	Putative uncharacterized protein
EOG0912020I	APLA012148	Agrilus planipennis	Putative uncharacterized protein
EOG0912020I	TC013032	Tribolium castaneum	Putative uncharacterized protein
EOG0912020I	LDEC011663	Leptinotarsa decemlineata	Putative uncharacterized protein
EOG0912020I	YQE_06060	Dendroctonus ponderosae	Putative uncharacterized protein
EOG0912020J	OTAU008994	Onthophagus taurus	Putative uncharacterized protein
EOG0912020J	AGLA003350	Anoplophora glabripennis	Putative uncharacterized protein
EOG0912020J	APLA005953	Agrilus planipennis	Putative uncharacterized protein
EOG0912020J	TC015142	Tribolium castaneum	Putative uncharacterized protein
EOG0912020J	LDEC008620	Leptinotarsa decemlineata	Putative uncharacterized protein
EOG0912020J	YQE_10838	Dendroctonus ponderosae	Putative uncharacterized protein
EOG0912020M	OTAU006366	Onthophagus taurus	Putative uncharacterized protein
EOG0912020M	AGLA010000	Anoplophora glabripennis	Putative uncharacterized protein
EOG0912020M	APLA004908	Agrilus planipennis	Putative uncharacterized protein
EOG0912020M	TC009492	Tribolium castaneum	Putative uncharacterized protein
EOG0912020M	LDEC011001	Leptinotarsa decemlineata	Putative uncharacterized protein
EOG0912020M	YQE_05392	Dendroctonus ponderosae	Putative uncharacterized protein
EOG0912020N	OTAU005256	Onthophagus taurus	Putative uncharacterized protein
EOG0912020N	AGLA003028	Anoplophora glabripennis	Putative uncharacterized protein
EOG0912020N	APLA004692	Agrilus planipennis	Putative uncharacterized protein
EOG0912020N	TC007376	Tribolium castaneum	Putative uncharacterized protein
EOG0912020N	LDEC003952	Leptinotarsa decemlineata	Putative uncharacterized protein
EOG0912020N	YQE_02295	Dendroctonus ponderosae	Putative uncharacterized protein
EOG0912020P	OTAU001255	Onthophagus taurus	Putative uncharacterized protein
EOG0912020P	AGLA007620	Anoplophora glabripennis	Putative uncharacterized protein
EOG0912020P	APLA013960	Agrilus planipennis	Putative uncharacterized protein
EOG0912020P	TC001624	Tribolium castaneum	Putative uncharacterized protein
EOG0912020P	LDEC004312	Leptinotarsa decemlineata	Putative uncharacterized protein
EOG0912020P	YQE_06597	Dendroctonus ponderosae	Putative uncharacterized protein
EOG0912020S	OTAU001299	Onthophagus taurus	Putative uncharacterized protein
EOG0912020S	AGLA000351	Anoplophora glabripennis	Putative uncharacterized protein
EOG0912020S	APLA011267	Agrilus planipennis	Putative uncharacterized protein
EOG0912020S	TC004122	Tribolium castaneum	Putative uncharacterized protein
EOG0912020S	LDEC000051	Leptinotarsa decemlineata	Putative uncharacterized protein
EOG0912020S	YQE_06686	Dendroctonus ponderosae	Putative uncharacterized protein
EOG0912020V	OTAU001709	Onthophagus taurus	Putative uncharacterized protein
EOG0912020V	AGLA007332	Anoplophora glabripennis	Putative uncharacterized protein
EOG0912020V	APLA014792	Agrilus planipennis	Putative uncharacterized protein
EOG0912020V	TC005426	Tribolium castaneum	Putative uncharacterized protein
EOG0912020V	LDEC013535	Leptinotarsa decemlineata	Putative uncharacterized protein
EOG0912020V	YQE_12143	Dendroctonus ponderosae	Putative uncharacterized protein
EOG0912020W	OTAU008239	Onthophagus taurus	Putative uncharacterized protein
EOG0912020W	AGLA021558	Anoplophora glabripennis	Putative uncharacterized protein

EOG0912020W	APLA013926	Agrilus planipennis	Putative uncharacterized protein
EOG0912020W	TC004891	Tribolium castaneum	Putative uncharacterized protein
EOG0912020W	LDEC000774	Leptinotarsa decemlineata	Putative uncharacterized protein
EOG0912020W	YQE_09483	Dendroctonus ponderosae	Putative uncharacterized protein
EOG0912020X	OTAU015570	Onthophagus taurus	None
EOG0912020X	AGLA010466	Anoplophora glabripennis	None
EOG0912020X	APLA005927	Agrilus planipennis	None
EOG0912020X	TC030906	Tribolium castaneum	None
EOG0912020X	LDEC017448	Leptinotarsa decemlineata	None
EOG0912020X	YQE_06522	Dendroctonus ponderosae	None
EOG0912020Y	OTAU001418	Onthophagus taurus	Putative uncharacterized protein
EOG0912020Y	AGLA000347	Anoplophora glabripennis	Putative uncharacterized protein
EOG0912020Y	APLA005846	Agrilus planipennis	Putative uncharacterized protein
EOG0912020Y	TC010881	Tribolium castaneum	Putative uncharacterized protein
EOG0912020Y	LDEC006830	Leptinotarsa decemlineata	Putative uncharacterized protein
EOG0912020Y	YQE_09175	Dendroctonus ponderosae	Putative uncharacterized protein
EOG09120214	OTAU000194	Onthophagus taurus	None
EOG09120214	AGLA013793	Anoplophora glabripennis	None
EOG09120214	APLA009388	Agrilus planipennis	None
EOG09120214	TC033242	Tribolium castaneum	None
EOG09120214	LDEC000277	Leptinotarsa decemlineata	None
EOG09120214	YQE_02063	Dendroctonus ponderosae	None
EOG09120218	OTAU001955	Onthophagus taurus	Putative uncharacterized protein
EOG09120218	AGLA001237	Anoplophora glabripennis	Putative uncharacterized protein
EOG09120218	APLA000402	Agrilus planipennis	Putative uncharacterized protein
EOG09120218	TC005937	Tribolium castaneum	Putative uncharacterized protein
EOG09120218	LDEC011061	Leptinotarsa decemlineata	Putative uncharacterized protein
EOG09120218	YQE_12016	Dendroctonus ponderosae	Putative uncharacterized protein
EOG09120219	OTAU002655	Onthophagus taurus	None
EOG09120219	AGLA001127	Anoplophora glabripennis	None
EOG09120219	APLA007726	Agrilus planipennis	None
EOG09120219	TC034705	Tribolium castaneum	None
EOG09120219	LDEC001128	Leptinotarsa decemlineata	None
EOG09120219	YQE_06155	Dendroctonus ponderosae	None
EOG0912021A	OTAU002153	Onthophagus taurus	Putative uncharacterized protein
EOG0912021A	AGLA009161	Anoplophora glabripennis	Putative uncharacterized protein
EOG0912021A	APLA005446	Agrilus planipennis	Putative uncharacterized protein
EOG0912021A	TC003153	Tribolium castaneum	Putative uncharacterized protein
EOG0912021A	LDEC010796	Leptinotarsa decemlineata	Putative uncharacterized protein
EOG0912021A	YQE_09863	Dendroctonus ponderosae	Putative uncharacterized protein
EOG0912021B	OTAU001422	Onthophagus taurus	Putative uncharacterized protein
EOG0912021B	AGLA002201	Anoplophora glabripennis	Putative uncharacterized protein
EOG0912021B	APLA014677	Agrilus planipennis	Putative uncharacterized protein
EOG0912021B	TC004151	Tribolium castaneum	Putative uncharacterized protein
EOG0912021B	LDEC006828	Leptinotarsa decemlineata	Putative uncharacterized protein
EOG0912021B	YQE_07458	Dendroctonus ponderosae	Putative uncharacterized protein
EOG0912021E	OTAU005821	Onthophagus taurus	Putative uncharacterized protein
EOG0912021E	AGLA001516	Anoplophora glabripennis	Putative uncharacterized protein
EOG0912021E	APLA003791	Agrilus planipennis	Putative uncharacterized protein
EOG0912021E	TC002629	Tribolium castaneum	Putative uncharacterized protein
EOG0912021E	LDEC004655	Leptinotarsa decemlineata	Putative uncharacterized protein
EOG0912021E	YQE_06757	Dendroctonus ponderosae	Putative uncharacterized protein
EOG0912021G	OTAU000826	Onthophagus taurus	Putative uncharacterized protein
EOG0912021G	AGLA005232	Anoplophora glabripennis	Putative uncharacterized protein
EOG0912021G	APLA004985	Agrilus planipennis	Putative uncharacterized protein
EOG0912021G	TC001142	Tribolium castaneum	Putative uncharacterized protein
EOG0912021G	LDEC003562	Leptinotarsa decemlineata	Putative uncharacterized protein
EOG0912021G	YQE_11030	Dendroctonus ponderosae	Putative uncharacterized protein
EOG0912021I	OTAU002957	Onthophagus taurus	Putative uncharacterized protein
EOG0912021I	AGLA006306	Anoplophora glabripennis	Putative uncharacterized protein
EOG0912021I	APLA006019	Agrilus planipennis	Putative uncharacterized protein
EOG0912021I	TC009537	Tribolium castaneum	Putative uncharacterized protein
EOG0912021I	LDEC002100	Leptinotarsa decemlineata	Putative uncharacterized protein
EOG0912021I	YQE_10580	Dendroctonus ponderosae	Putative uncharacterized protein
EOG0912021J	OTAU012810	Onthophagus taurus	None
EOG0912021J	AGLA012968	Anoplophora glabripennis	None
EOG0912021J	APLA013602	Agrilus planipennis	None

EOG0912021J	TC034561	Tribolium castaneum	None
EOG0912021J	LDEC002743	Leptinotarsa decemlineata	None
EOG0912021J	YQE_07349	Dendroctonus ponderosae	None
EOG0912021L	OTAU009237	Onthophagus taurus	Putative uncharacterized protein
EOG0912021L	AGLA016394	Anoplophora glabripennis	Putative uncharacterized protein
EOG0912021L	APLA002471	Agrilus planipennis	Putative uncharacterized protein
EOG0912021L	TC013532	Tribolium castaneum	Putative uncharacterized protein
EOG0912021L	LDEC001142	Leptinotarsa decemlineata	Putative uncharacterized protein
EOG0912021L	YQE_08630	Dendroctonus ponderosae	Putative uncharacterized protein
EOG0912021N	OTAU002072	Onthophagus taurus	Putative uncharacterized protein
EOG0912021N	AGLA008375	Anoplophora glabripennis	Putative uncharacterized protein
EOG0912021N	APLA001117	Agrilus planipennis	Putative uncharacterized protein
EOG0912021N	TC016297	Tribolium castaneum	Putative uncharacterized protein
EOG0912021N	LDEC020788	Leptinotarsa decemlineata	Putative uncharacterized protein
EOG0912021N	YQE_04369	Dendroctonus ponderosae	Putative uncharacterized protein
EOG0912021P	OTAU002520	Onthophagus taurus	Putative uncharacterized protein
EOG0912021P	AGLA009411	Anoplophora glabripennis	Putative uncharacterized protein
EOG0912021P	APLA013876	Agrilus planipennis	Putative uncharacterized protein
EOG0912021P	TC016340	Tribolium castaneum	Putative uncharacterized protein
EOG0912021P	LDEC015182	Leptinotarsa decemlineata	Putative uncharacterized protein
EOG0912021P	YQE_13020	Dendroctonus ponderosae	Putative uncharacterized protein
EOG0912021Q	OTAU009585	Onthophagus taurus	None
EOG0912021Q	AGLA001997	Anoplophora glabripennis	None
EOG0912021Q	APLA000739	Agrilus planipennis	None
EOG0912021Q	TC032642	Tribolium castaneum	None
EOG0912021Q	LDEC003321	Leptinotarsa decemlineata	None
EOG0912021Q	YQE_12410	Dendroctonus ponderosae	None
EOG0912021R	OTAU005331	Onthophagus taurus	Ret oncogene
EOG0912021R	AGLA014351	Anoplophora glabripennis	Ret oncogene
EOG0912021R	APLA013531	Agrilus planipennis	Ret oncogene
EOG0912021R	TC012783	Tribolium castaneum	Ret oncogene
EOG0912021R	LDEC009746	Leptinotarsa decemlineata	Ret oncogene
EOG0912021R	YQE_12904	Dendroctonus ponderosae	Ret oncogene
EOG0912021S	OTAU001513	Onthophagus taurus	Putative uncharacterized protein
EOG0912021S	AGLA003823	Anoplophora glabripennis	Putative uncharacterized protein
EOG0912021S	APLA014043	Agrilus planipennis	Putative uncharacterized protein
EOG0912021S	TC030783	Tribolium castaneum	Putative uncharacterized protein
EOG0912021S	LDEC014307	Leptinotarsa decemlineata	Putative uncharacterized protein
EOG0912021S	YQE_12646	Dendroctonus ponderosae	Putative uncharacterized protein
EOG0912021U	OTAU013332	Onthophagus taurus	Elongation factor Tu
EOG0912021U	AGLA001948	Anoplophora glabripennis	Elongation factor Tu
EOG0912021U	APLA008652	Agrilus planipennis	Elongation factor Tu
EOG0912021U	TC011692	Tribolium castaneum	Elongation factor Tu
EOG0912021U	LDEC008350	Leptinotarsa decemlineata	Elongation factor Tu
EOG0912021U	YQE_08054	Dendroctonus ponderosae	Elongation factor Tu
EOG0912021X	OTAU007394	Onthophagus taurus	Putative uncharacterized protein
EOG0912021X	AGLA012037	Anoplophora glabripennis	Putative uncharacterized protein
EOG0912021X	APLA008635	Agrilus planipennis	Putative uncharacterized protein
EOG0912021X	TC014039	Tribolium castaneum	Putative uncharacterized protein
EOG0912021X	LDEC010523	Leptinotarsa decemlineata	Putative uncharacterized protein
EOG0912021X	YQE_05092	Dendroctonus ponderosae	Putative uncharacterized protein
EOG0912021Z	OTAU007598	Onthophagus taurus	Putative uncharacterized protein
EOG0912021Z	AGLA010754	Anoplophora glabripennis	Putative uncharacterized protein
EOG0912021Z	APLA006647	Agrilus planipennis	Putative uncharacterized protein
EOG0912021Z	TC003396	Tribolium castaneum	Putative uncharacterized protein
EOG0912021Z	LDEC016707	Leptinotarsa decemlineata	Putative uncharacterized protein
EOG0912021Z	YQE_09886	Dendroctonus ponderosae	Putative uncharacterized protein
EOG09120220	OTAU013888	Onthophagus taurus	Putative uncharacterized protein
EOG09120220	AGLA003100	Anoplophora glabripennis	Putative uncharacterized protein
EOG09120220	APLA003182	Agrilus planipennis	Putative uncharacterized protein
EOG09120220	TC007190	Tribolium castaneum	Putative uncharacterized protein
EOG09120220	LDEC020053	Leptinotarsa decemlineata	Putative uncharacterized protein
EOG09120220	YQE_09727	Dendroctonus ponderosae	Putative uncharacterized protein
EOG09120225	OTAU016532	Onthophagus taurus	Putative uncharacterized protein
EOG09120225	AGLA003526	Anoplophora glabripennis	Putative uncharacterized protein
EOG09120225	APLA000813	Agrilus planipennis	Putative uncharacterized protein
EOG09120225	TC003829	Tribolium castaneum	Putative uncharacterized protein

EOG09120225	LDEC012678	Leptinotarsa decemlineata	Putative uncharacterized protein
EOG09120225	YQE_11114	Dendroctonus ponderosae	Putative uncharacterized protein
EOG09120226	OTAU001634	Onthophagus taurus	Putative uncharacterized protein
EOG09120226	AGLA021188	Anoplophora glabripennis	Putative uncharacterized protein
EOG09120226	APLA012110	Agrilus planipennis	Putative uncharacterized protein
EOG09120226	TC005395	Tribolium castaneum	Putative uncharacterized protein
EOG09120226	LDEC009322	Leptinotarsa decemlineata	Putative uncharacterized protein
EOG09120226	YQE_09458	Dendroctonus ponderosae	Putative uncharacterized protein
EOG09120227	OTAU001489	Onthophagus taurus	Putative uncharacterized protein
EOG09120227	AGLA004546	Anoplophora glabripennis	Putative uncharacterized protein
EOG09120227	APLA003554	Agrilus planipennis	Putative uncharacterized protein
EOG09120227	TC004677	Tribolium castaneum	Putative uncharacterized protein
EOG09120227	LDEC003511	Leptinotarsa decemlineata	Putative uncharacterized protein
EOG09120227	YQE_03289	Dendroctonus ponderosae	Putative uncharacterized protein
EOG09120229	OTAU004220	Onthophagus taurus	Putative uncharacterized protein
EOG09120229	AGLA013066	Anoplophora glabripennis	Putative uncharacterized protein
EOG09120229	APLA008496	Agrilus planipennis	Putative uncharacterized protein
EOG09120229	TC014107	Tribolium castaneum	Putative uncharacterized protein
EOG09120229	LDEC009039	Leptinotarsa decemlineata	Putative uncharacterized protein
EOG09120229	YQE_03413	Dendroctonus ponderosae	Putative uncharacterized protein
EOG0912022B	OTAU003691	Onthophagus taurus	None
EOG0912022B	AGLA010422	Anoplophora glabripennis	None
EOG0912022B	APLA005379	Agrilus planipennis	None
EOG0912022B	TC031105	Tribolium castaneum	None
EOG0912022B	LDEC000158	Leptinotarsa decemlineata	None
EOG0912022B	YQE_04606	Dendroctonus ponderosae	None
EOG0912022D	OTAU014878	Onthophagus taurus	Putative uncharacterized protein
EOG0912022D	AGLA017509	Anoplophora glabripennis	Putative uncharacterized protein
EOG0912022D	APLA007902	Agrilus planipennis	Putative uncharacterized protein
EOG0912022D	TC004375	Tribolium castaneum	Putative uncharacterized protein
EOG0912022D	LDEC016875	Leptinotarsa decemlineata	Putative uncharacterized protein
EOG0912022D	YQE_08649	Dendroctonus ponderosae	Putative uncharacterized protein
EOG0912022E	OTAU000249	Onthophagus taurus	Putative uncharacterized protein
EOG0912022E	AGLA010979	Anoplophora glabripennis	Putative uncharacterized protein
EOG0912022E	APLA002860	Agrilus planipennis	Putative uncharacterized protein
EOG0912022E	TC013875	Tribolium castaneum	Putative uncharacterized protein
EOG0912022E	LDEC009384	Leptinotarsa decemlineata	Putative uncharacterized protein
EOG0912022E	YQE_07265	Dendroctonus ponderosae	Putative uncharacterized protein
EOG0912022F	OTAU003514	Onthophagus taurus	Estrogen-related receptor
EOG0912022F	AGLA016381	Anoplophora glabripennis	Estrogen-related receptor
EOG0912022F	APLA010074	Agrilus planipennis	Estrogen-related receptor
EOG0912022F	TC009140	Tribolium castaneum	Estrogen-related receptor
EOG0912022F	LDEC009878	Leptinotarsa decemlineata	Estrogen-related receptor
EOG0912022F	YQE_02252	Dendroctonus ponderosae	Estrogen-related receptor
EOG0912022I	OTAU011484	Onthophagus taurus	Putative uncharacterized protein
EOG0912022I	AGLA012698	Anoplophora glabripennis	Putative uncharacterized protein
EOG0912022I	APLA011311	Agrilus planipennis	Putative uncharacterized protein
EOG0912022I	TC012453	Tribolium castaneum	Putative uncharacterized protein
EOG0912022I	LDEC013628	Leptinotarsa decemlineata	Putative uncharacterized protein
EOG0912022I	YQE_08011	Dendroctonus ponderosae	Putative uncharacterized protein
EOG0912022J	OTAU005261	Onthophagus taurus	Putative uncharacterized protein
EOG0912022J	AGLA000767	Anoplophora glabripennis	Putative uncharacterized protein
EOG0912022J	APLA002277	Agrilus planipennis	Putative uncharacterized protein
EOG0912022J	TC008130	Tribolium castaneum	Putative uncharacterized protein
EOG0912022J	LDEC005102	Leptinotarsa decemlineata	Putative uncharacterized protein
EOG0912022J	YQE_03069	Dendroctonus ponderosae	Putative uncharacterized protein
EOG0912022K	OTAU008892	Onthophagus taurus	None
EOG0912022K	AGLA000987	Anoplophora glabripennis	None
EOG0912022K	APLA013474	Agrilus planipennis	None
EOG0912022K	TC032250	Tribolium castaneum	None
EOG0912022K	LDEC007402	Leptinotarsa decemlineata	None
EOG0912022K	YQE_04490	Dendroctonus ponderosae	None
EOG0912022M	OTAU004242	Onthophagus taurus	Putative uncharacterized protein
EOG0912022M	AGLA007140	Anoplophora glabripennis	Putative uncharacterized protein
EOG0912022M	APLA012265	Agrilus planipennis	Putative uncharacterized protein
EOG0912022M	TC014658	Tribolium castaneum	Putative uncharacterized protein
EOG0912022M	LDEC017652	Leptinotarsa decemlineata	Putative uncharacterized protein

EOG0912022M	YQE_08571	Dendroctonus ponderosae	Putative uncharacterized protein
EOG0912022N	OTAU006540	Onthophagus taurus	Putative uncharacterized protein
EOG0912022N	AGLA009121	Anoplophora glabripennis	Putative uncharacterized protein
EOG0912022N	APLA006680	Agrilus planipennis	Putative uncharacterized protein
EOG0912022N	TC003711	Tribolium castaneum	Putative uncharacterized protein
EOG0912022N	LDEC005429	Leptinotarsa decemlineata	Putative uncharacterized protein
EOG0912022N	YQE_07813	Dendroctonus ponderosae	Putative uncharacterized protein
EOG0912022O	OTAU014993	Onthophagus taurus	Putative uncharacterized protein
EOG0912022O	AGLA005079	Anoplophora glabripennis	Putative uncharacterized protein
EOG0912022O	APLA011516	Agrilus planipennis	Putative uncharacterized protein
EOG0912022O	TC001511	Tribolium castaneum	Putative uncharacterized protein
EOG0912022O	LDEC011378	Leptinotarsa decemlineata	Putative uncharacterized protein
EOG0912022O	YQE_09573	Dendroctonus ponderosae	Putative uncharacterized protein
EOG0912022P	OTAU001878	Onthophagus taurus	nucleic acid binding
EOG0912022P	AGLA004768	Anoplophora glabripennis	nucleic acid binding
EOG0912022P	APLA000917	Agrilus planipennis	nucleic acid binding
EOG0912022P	TC034076	Tribolium castaneum	nucleic acid binding
EOG0912022P	LDEC017829	Leptinotarsa decemlineata	nucleic acid binding
EOG0912022P	YQE_05797	Dendroctonus ponderosae	nucleic acid binding
EOG0912022Q	OTAU004968	Onthophagus taurus	Putative uncharacterized protein
EOG0912022Q	AGLA002533	Anoplophora glabripennis	Putative uncharacterized protein
EOG0912022Q	APLA010741	Agrilus planipennis	Putative uncharacterized protein
EOG0912022Q	TC011151	Tribolium castaneum	Putative uncharacterized protein
EOG0912022Q	LDEC001794	Leptinotarsa decemlineata	Putative uncharacterized protein
EOG0912022Q	YQE_07754	Dendroctonus ponderosae	Putative uncharacterized protein
EOG0912022R	OTAU000289	Onthophagus taurus	None
EOG0912022R	AGLA008456	Anoplophora glabripennis	None
EOG0912022R	APLA006221	Agrilus planipennis	None
EOG0912022R	TC033041	Tribolium castaneum	None
EOG0912022R	LDEC021508	Leptinotarsa decemlineata	None
EOG0912022R	YQE_04892	Dendroctonus ponderosae	None
EOG0912022S	OTAU003069	Onthophagus taurus	Isocitrate dehydrogenase
EOG0912022S	AGLA021356	Anoplophora glabripennis	Isocitrate dehydrogenase
EOG0912022S	APLA001137	Agrilus planipennis	Isocitrate dehydrogenase
EOG0912022S	TC011647	Tribolium castaneum	Isocitrate dehydrogenase
EOG0912022S	LDEC017891	Leptinotarsa decemlineata	Isocitrate dehydrogenase
EOG0912022S	YQE_04657	Dendroctonus ponderosae	Isocitrate dehydrogenase
EOG0912022T	OTAU015388	Onthophagus taurus	extracellular ligand-gated ion channel activity
EOG0912022T	AGLA001922	Anoplophora glabripennis	extracellular ligand-gated ion channel activity
EOG0912022T	APLA007855	Agrilus planipennis	extracellular ligand-gated ion channel activity
EOG0912022T	TC032251	Tribolium castaneum	extracellular ligand-gated ion channel activity
EOG0912022T	LDEC000863	Leptinotarsa decemlineata	extracellular ligand-gated ion channel activity
EOG0912022T	YQE_03400	Dendroctonus ponderosae	extracellular ligand-gated ion channel activity
EOG0912022X	OTAU000139	Onthophagus taurus	Aspartate aminotransferase
EOG0912022X	AGLA015173	Anoplophora glabripennis	Aspartate aminotransferase
EOG0912022X	APLA012145	Agrilus planipennis	Aspartate aminotransferase
EOG0912022X	TC013019	Tribolium castaneum	Aspartate aminotransferase
EOG0912022X	LDEC016361	Leptinotarsa decemlineata	Aspartate aminotransferase
EOG0912022X	YQE_02107	Dendroctonus ponderosae	Aspartate aminotransferase
EOG0912022Y	OTAU003767	Onthophagus taurus	catalytic activity
EOG0912022Y	AGLA005753	Anoplophora glabripennis	catalytic activity
EOG0912022Y	APLA010177	Agrilus planipennis	catalytic activity
EOG0912022Y	TC033566	Tribolium castaneum	catalytic activity
EOG0912022Y	LDEC021011	Leptinotarsa decemlineata	catalytic activity
EOG0912022Y	YQE_11316	Dendroctonus ponderosae	catalytic activity
EOG0912022Z	OTAU002557	Onthophagus taurus	Putative uncharacterized protein
EOG0912022Z	AGLA011927	Anoplophora glabripennis	Putative uncharacterized protein
EOG0912022Z	APLA014464	Agrilus planipennis	Putative uncharacterized protein
EOG0912022Z	TC016315	Tribolium castaneum	Putative uncharacterized protein
EOG0912022Z	LDEC011664	Leptinotarsa decemlineata	Putative uncharacterized protein
EOG0912022Z	YQE_06091	Dendroctonus ponderosae	Putative uncharacterized protein
EOG09120232	OTAU009490	Onthophagus taurus	None
EOG09120232	AGLA009941	Anoplophora glabripennis	None
EOG09120232	APLA014722	Agrilus planipennis	None
EOG09120232	TC033320	Tribolium castaneum	None
EOG09120232	LDEC013354	Leptinotarsa decemlineata	None
EOG09120232	YQE_09396	Dendroctonus ponderosae	None

EOG09120233	OTAU006861	Onthophagus taurus	None
EOG09120233	AGLA016081	Anoplophora glabripennis	None
EOG09120233	APLA003053	Agrilus planipennis	None
EOG09120233	TC034706	Tribolium castaneum	None
EOG09120233	LDEC014011	Leptinotarsa decemlineata	None
EOG09120233	YQE_02770	Dendroctonus ponderosae	None
EOG09120234	OTAU005195	Onthophagus taurus	Putative uncharacterized protein
EOG09120234	AGLA020517	Anoplophora glabripennis	Putative uncharacterized protein
EOG09120234	APLA009197	Agrilus planipennis	Putative uncharacterized protein
EOG09120234	TC003488	Tribolium castaneum	Putative uncharacterized protein
EOG09120234	LDEC016790	Leptinotarsa decemlineata	Putative uncharacterized protein
EOG09120234	YQE_12354	Dendroctonus ponderosae	Putative uncharacterized protein
EOG09120237	OTAU013083	Onthophagus taurus	Putative uncharacterized protein
EOG09120237	AGLA013161	Anoplophora glabripennis	Putative uncharacterized protein
EOG09120237	APLA009073	Agrilus planipennis	Putative uncharacterized protein
EOG09120237	TC002215	Tribolium castaneum	Putative uncharacterized protein
EOG09120237	LDEC013539	Leptinotarsa decemlineata	Putative uncharacterized protein
EOG09120237	YQE_07521	Dendroctonus ponderosae	Putative uncharacterized protein
EOG09120238	OTAU014741	Onthophagus taurus	Cytochrome P450 301B1
EOG09120238	AGLA004482	Anoplophora glabripennis	Cytochrome P450 301B1
EOG09120238	APLA013388	Agrilus planipennis	Cytochrome P450 301B1
EOG09120238	TC007167	Tribolium castaneum	Cytochrome P450 301B1
EOG09120238	LDEC006356	Leptinotarsa decemlineata	Cytochrome P450 301B1
EOG09120238	YQE_02974	Dendroctonus ponderosae	Cytochrome P450 301B1
EOG09120239	OTAU007713	Onthophagus taurus	Putative uncharacterized protein
EOG09120239	AGLA003562	Anoplophora glabripennis	Putative uncharacterized protein
EOG09120239	APLA002010	Agrilus planipennis	Putative uncharacterized protein
EOG09120239	TC000144	Tribolium castaneum	Putative uncharacterized protein
EOG09120239	LDEC020550	Leptinotarsa decemlineata	Putative uncharacterized protein
EOG09120239	YQE_11120	Dendroctonus ponderosae	Putative uncharacterized protein
EOG0912023C	OTAU002890	Onthophagus taurus	Putative uncharacterized protein
EOG0912023C	AGLA006526	Anoplophora glabripennis	Putative uncharacterized protein
EOG0912023C	APLA007166	Agrilus planipennis	Putative uncharacterized protein
EOG0912023C	TC009172	Tribolium castaneum	Putative uncharacterized protein
EOG0912023C	LDEC021367	Leptinotarsa decemlineata	Putative uncharacterized protein
EOG0912023C	YQE_13014	Dendroctonus ponderosae	Putative uncharacterized protein
EOG0912023D	OTAU001868	Onthophagus taurus	Putative uncharacterized protein
EOG0912023D	AGLA019337	Anoplophora glabripennis	Putative uncharacterized protein
EOG0912023D	APLA000517	Agrilus planipennis	Putative uncharacterized protein
EOG0912023D	TC005814	Tribolium castaneum	Putative uncharacterized protein
EOG0912023D	LDEC006327	Leptinotarsa decemlineata	Putative uncharacterized protein
EOG0912023D	YQE_05708	Dendroctonus ponderosae	Putative uncharacterized protein
EOG0912023F	OTAU009400	Onthophagus taurus	Putative uncharacterized protein
EOG0912023F	AGLA014976	Anoplophora glabripennis	Putative uncharacterized protein
EOG0912023F	APLA005231	Agrilus planipennis	Putative uncharacterized protein
EOG0912023F	TC003451	Tribolium castaneum	Putative uncharacterized protein
EOG0912023F	LDEC020095	Leptinotarsa decemlineata	Putative uncharacterized protein
EOG0912023F	YQE_09769	Dendroctonus ponderosae	Putative uncharacterized protein
EOG0912023H	OTAU009197	Onthophagus taurus	Putative uncharacterized protein
EOG0912023H	AGLA003902	Anoplophora glabripennis	Putative uncharacterized protein
EOG0912023H	APLA009448	Agrilus planipennis	Putative uncharacterized protein
EOG0912023H	TC015452	Tribolium castaneum	Putative uncharacterized protein
EOG0912023H	LDEC003615	Leptinotarsa decemlineata	Putative uncharacterized protein
EOG0912023H	YQE_11878	Dendroctonus ponderosae	Putative uncharacterized protein
EOG0912023K	OTAU002692	Onthophagus taurus	Integrin beta
EOG0912023K	AGLA018635	Anoplophora glabripennis	Integrin beta
EOG0912023K	APLA009721	Agrilus planipennis	Integrin beta
EOG0912023K	TC013706	Tribolium castaneum	Integrin beta
EOG0912023K	LDEC009492	Leptinotarsa decemlineata	Integrin beta
EOG0912023K	YQE_13074	Dendroctonus ponderosae	Integrin beta
EOG0912023L	OTAU008563	Onthophagus taurus	peptidase activity
EOG0912023L	AGLA011202	Anoplophora glabripennis	peptidase activity
EOG0912023L	APLA008195	Agrilus planipennis	peptidase activity
EOG0912023L	TC033856	Tribolium castaneum	peptidase activity
EOG0912023L	LDEC007689	Leptinotarsa decemlineata	peptidase activity
EOG0912023L	YQE_12585	Dendroctonus ponderosae	peptidase activity
EOG0912023S	OTAU007142	Onthophagus taurus	Putative uncharacterized protein

EOG0912023S	AGLA014828	Anoplophora glabripennis	Putative uncharacterized protein
EOG0912023S	APLA002979	Agrilus planipennis	Putative uncharacterized protein
EOG0912023S	TC000855	Tribolium castaneum	Putative uncharacterized protein
EOG0912023S	LDEC006060	Leptinotarsa decemlineata	Putative uncharacterized protein
EOG0912023S	YQE_03338	Dendroctonus ponderosae	Putative uncharacterized protein
EOG0912023T	OTAU001575	Onthophagus taurus	Serine hydroxymethyltransferase
EOG0912023T	AGLA011073	Anoplophora glabripennis	Serine hydroxymethyltransferase
EOG0912023T	APLA000499	Agrilus planipennis	Serine hydroxymethyltransferase
EOG0912023T	TC015993	Tribolium castaneum	Serine hydroxymethyltransferase
EOG0912023T	LDEC020216	Leptinotarsa decemlineata	Serine hydroxymethyltransferase
EOG0912023T	YQE_06917	Dendroctonus ponderosae	Serine hydroxymethyltransferase
EOG0912023U	OTAU011894	Onthophagus taurus	Putative uncharacterized protein
EOG0912023U	AGLA012047	Anoplophora glabripennis	Putative uncharacterized protein
EOG0912023U	APLA002479	Agrilus planipennis	Putative uncharacterized protein
EOG0912023U	TC010470	Tribolium castaneum	Putative uncharacterized protein
EOG0912023U	LDEC010541	Leptinotarsa decemlineata	Putative uncharacterized protein
EOG0912023U	YQE_04036	Dendroctonus ponderosae	Putative uncharacterized protein
EOG0912023V	OTAU008735	Onthophagus taurus	Putative uncharacterized protein
EOG0912023V	AGLA012705	Anoplophora glabripennis	Putative uncharacterized protein
EOG0912023V	APLA012630	Agrilus planipennis	Putative uncharacterized protein
EOG0912023V	TC003392	Tribolium castaneum	Putative uncharacterized protein
EOG0912023V	LDEC014223	Leptinotarsa decemlineata	Putative uncharacterized protein
EOG0912023V	YQE_07454	Dendroctonus ponderosae	Putative uncharacterized protein
EOG0912023X	OTAU001447	Onthophagus taurus	Putative uncharacterized protein
EOG0912023X	AGLA000126	Anoplophora glabripennis	Putative uncharacterized protein
EOG0912023X	APLA009351	Agrilus planipennis	Putative uncharacterized protein
EOG0912023X	TC000228	Tribolium castaneum	Putative uncharacterized protein
EOG0912023X	LDEC008086	Leptinotarsa decemlineata	Putative uncharacterized protein
EOG0912023X	YQE_08144	Dendroctonus ponderosae	Putative uncharacterized protein
EOG0912023Y	OTAU001482	Onthophagus taurus	Putative uncharacterized protein
EOG0912023Y	AGLA004522	Anoplophora glabripennis	Putative uncharacterized protein
EOG0912023Y	APLA003574	Agrilus planipennis	Putative uncharacterized protein
EOG0912023Y	TC001878	Tribolium castaneum	Putative uncharacterized protein
EOG0912023Y	LDEC019201	Leptinotarsa decemlineata	Putative uncharacterized protein
EOG0912023Y	YQE_06716	Dendroctonus ponderosae	Putative uncharacterized protein
EOG0912023Z	OTAU003362	Onthophagus taurus	Putative uncharacterized protein
EOG0912023Z	AGLA005911	Anoplophora glabripennis	Putative uncharacterized protein
EOG0912023Z	APLA000852	Agrilus planipennis	Putative uncharacterized protein
EOG0912023Z	TC012874	Tribolium castaneum	Putative uncharacterized protein
EOG0912023Z	LDEC018270	Leptinotarsa decemlineata	Putative uncharacterized protein
EOG0912023Z	YQE_08021	Dendroctonus ponderosae	Putative uncharacterized protein
EOG09120240	OTAU002015	Onthophagus taurus	Putative uncharacterized protein
EOG09120240	AGLA012676	Anoplophora glabripennis	Putative uncharacterized protein
EOG09120240	APLA000422	Agrilus planipennis	Putative uncharacterized protein
EOG09120240	TC006412	Tribolium castaneum	Putative uncharacterized protein
EOG09120240	LDEC011064	Leptinotarsa decemlineata	Putative uncharacterized protein
EOG09120240	YQE_08548	Dendroctonus ponderosae	Putative uncharacterized protein
EOG09120244	OTAU000225	Onthophagus taurus	Frizzled
EOG09120244	AGLA006427	Anoplophora glabripennis	Frizzled
EOG09120244	APLA001145	Agrilus planipennis	Frizzled
EOG09120244	TC014055	Tribolium castaneum	Frizzled
EOG09120244	LDEC007256	Leptinotarsa decemlineata	Frizzled
EOG09120244	YQE_05088	Dendroctonus ponderosae	Frizzled
EOG09120245	OTAU009637	Onthophagus taurus	Putative uncharacterized protein
EOG09120245	AGLA012936	Anoplophora glabripennis	Putative uncharacterized protein
EOG09120245	APLA004983	Agrilus planipennis	Putative uncharacterized protein
EOG09120245	TC001366	Tribolium castaneum	Putative uncharacterized protein
EOG09120245	LDEC012591	Leptinotarsa decemlineata	Putative uncharacterized protein
EOG09120245	YQE_11648	Dendroctonus ponderosae	Putative uncharacterized protein
EOG09120248	OTAU009501	Onthophagus taurus	Putative uncharacterized protein
EOG09120248	AGLA017277	Anoplophora glabripennis	Putative uncharacterized protein
EOG09120248	APLA005565	Agrilus planipennis	Putative uncharacterized protein
EOG09120248	TC014994	Tribolium castaneum	Putative uncharacterized protein
EOG09120248	LDEC015294	Leptinotarsa decemlineata	Putative uncharacterized protein
EOG09120248	YQE_11659	Dendroctonus ponderosae	Putative uncharacterized protein
EOG09120249	OTAU007248	Onthophagus taurus	Cappuccino
EOG09120249	AGLA001258	Anoplophora glabripennis	Cappuccino

EOG09120249	APLA001065	Agrilus planipennis	Cappuccino
EOG09120249	TC012258	Tribolium castaneum	Cappuccino
EOG09120249	LDEC011038	Leptinotarsa decemlineata	Cappuccino
EOG09120249	YQE_08101	Dendroctonus ponderosae	Cappuccino
EOG0912024A	OTAU005967	Onthophagus taurus	Putative uncharacterized protein
EOG0912024A	AGLA015234	Anoplophora glabripennis	Putative uncharacterized protein
EOG0912024A	APLA005226	Agrilus planipennis	Putative uncharacterized protein
EOG0912024A	TC000052	Tribolium castaneum	Putative uncharacterized protein
EOG0912024A	LDEC005010	Leptinotarsa decemlineata	Putative uncharacterized protein
EOG0912024A	YQE_05133	Dendroctonus ponderosae	Putative uncharacterized protein
EOG0912024B	OTAU009567	Onthophagus taurus	Putative uncharacterized protein
EOG0912024B	AGLA013801	Anoplophora glabripennis	Putative uncharacterized protein
EOG0912024B	APLA000206	Agrilus planipennis	Putative uncharacterized protein
EOG0912024B	TC007919	Tribolium castaneum	Putative uncharacterized protein
EOG0912024B	LDEC004399	Leptinotarsa decemlineata	Putative uncharacterized protein
EOG0912024B	YQE_04804	Dendroctonus ponderosae	Putative uncharacterized protein
EOG0912024C	OTAU005169	Onthophagus taurus	Pyrroline-5-carboxylate reductase
EOG0912024C	AGLA019780	Anoplophora glabripennis	Pyrroline-5-carboxylate reductase
EOG0912024C	APLA013778	Agrilus planipennis	Pyrroline-5-carboxylate reductase
EOG0912024C	TC002895	Tribolium castaneum	Pyrroline-5-carboxylate reductase
EOG0912024C	LDEC017644	Leptinotarsa decemlineata	Pyrroline-5-carboxylate reductase
EOG0912024C	YQE_07451	Dendroctonus ponderosae	Pyrroline-5-carboxylate reductase
EOG0912024G	OTAU006745	Onthophagus taurus	None
EOG0912024G	AGLA012885	Anoplophora glabripennis	None
EOG0912024G	APLA002977	Agrilus planipennis	None
EOG0912024G	TC030991	Tribolium castaneum	None
EOG0912024G	LDEC016219	Leptinotarsa decemlineata	None
EOG0912024G	YQE_02712	Dendroctonus ponderosae	None
EOG0912024K	OTAU004409	Onthophagus taurus	Putative uncharacterized protein
EOG0912024K	AGLA013198	Anoplophora glabripennis	Putative uncharacterized protein
EOG0912024K	APLA011437	Agrilus planipennis	Putative uncharacterized protein
EOG0912024K	TC007349	Tribolium castaneum	Putative uncharacterized protein
EOG0912024K	LDEC008303	Leptinotarsa decemlineata	Putative uncharacterized protein
EOG0912024K	YQE_11438	Dendroctonus ponderosae	Putative uncharacterized protein
EOG0912024L	OTAU004136	Onthophagus taurus	None
EOG0912024L	AGLA005851	Anoplophora glabripennis	None
EOG0912024L	APLA008871	Agrilus planipennis	None
EOG0912024L	TC033836	Tribolium castaneum	None
EOG0912024L	LDEC011904	Leptinotarsa decemlineata	None
EOG0912024L	YQE_03513	Dendroctonus ponderosae	None
EOG0912024M	OTAU012498	Onthophagus taurus	None
EOG0912024M	AGLA007911	Anoplophora glabripennis	None
EOG0912024M	APLA003730	Agrilus planipennis	None
EOG0912024M	TC032517	Tribolium castaneum	None
EOG0912024M	LDEC019668	Leptinotarsa decemlineata	None
EOG0912024M	YQE_01768	Dendroctonus ponderosae	None
EOG0912024Q	OTAU016634	Onthophagus taurus	None
EOG0912024Q	AGLA021124	Anoplophora glabripennis	None
EOG0912024Q	APLA014529	Agrilus planipennis	None
EOG0912024Q	TC034804	Tribolium castaneum	None
EOG0912024Q	LDEC014725	Leptinotarsa decemlineata	None
EOG0912024Q	YQE_08770	Dendroctonus ponderosae	None
EOG0912024V	OTAU000996	Onthophagus taurus	Timeout
EOG0912024V	AGLA000955	Anoplophora glabripennis	Timeout
EOG0912024V	APLA007864	Agrilus planipennis	Timeout
EOG0912024V	TC000593	Tribolium castaneum	Timeout
EOG0912024V	LDEC018588	Leptinotarsa decemlineata	Timeout
EOG0912024V	YQE_05056	Dendroctonus ponderosae	Timeout
EOG0912024W	OTAU008487	Onthophagus taurus	Putative uncharacterized protein
EOG0912024W	AGLA002093	Anoplophora glabripennis	Putative uncharacterized protein
EOG0912024W	APLA008171	Agrilus planipennis	Putative uncharacterized protein
EOG0912024W	TC003160	Tribolium castaneum	Putative uncharacterized protein
EOG0912024W	LDEC001338	Leptinotarsa decemlineata	Putative uncharacterized protein
EOG0912024W	YQE_07420	Dendroctonus ponderosae	Putative uncharacterized protein
EOG0912024Y	OTAU006989	Onthophagus taurus	Putative uncharacterized protein
EOG0912024Y	AGLA017126	Anoplophora glabripennis	Putative uncharacterized protein
EOG0912024Y	APLA000786	Agrilus planipennis	Putative uncharacterized protein

EOG0912024Y	TC006460	Tribolium castaneum	Putative uncharacterized protein
EOG0912024Y	LDEC007202	Leptinotarsa decemlineata	Putative uncharacterized protein
EOG0912024Y	YQE_01800	Dendroctonus ponderosae	Putative uncharacterized protein
EOG09120255	OTAU000549	Onthophagus taurus	None
EOG09120255	AGLA004817	Anoplophora glabripennis	None
EOG09120255	APLA000885	Agrilus planipennis	None
EOG09120255	TC031679	Tribolium castaneum	None
EOG09120255	LDEC015675	Leptinotarsa decemlineata	None
EOG09120255	YQE_05856	Dendroctonus ponderosae	None
EOG09120257	OTAU002893	Onthophagus taurus	Putative uncharacterized protein
EOG09120257	AGLA018204	Anoplophora glabripennis	Putative uncharacterized protein
EOG09120257	APLA010286	Agrilus planipennis	Putative uncharacterized protein
EOG09120257	TC009647	Tribolium castaneum	Putative uncharacterized protein
EOG09120257	LDEC011982	Leptinotarsa decemlineata	Putative uncharacterized protein
EOG09120257	YQE_06205	Dendroctonus ponderosae	Putative uncharacterized protein
EOG0912025C	OTAU010326	Onthophagus taurus	Saxophone
EOG0912025C	AGLA006512	Anoplophora glabripennis	Saxophone
EOG0912025C	APLA001599	Agrilus planipennis	Saxophone
EOG0912025C	TC015948	Tribolium castaneum	Saxophone
EOG0912025C	LDEC006428	Leptinotarsa decemlineata	Saxophone
EOG0912025C	YQE_02202	Dendroctonus ponderosae	Saxophone
EOG0912025G	OTAU013266	Onthophagus taurus	Putative uncharacterized protein
EOG0912025G	AGLA008019	Anoplophora glabripennis	Putative uncharacterized protein
EOG0912025G	APLA009132	Agrilus planipennis	Putative uncharacterized protein
EOG0912025G	TC015854	Tribolium castaneum	Putative uncharacterized protein
EOG0912025G	LDEC014923	Leptinotarsa decemlineata	Putative uncharacterized protein
EOG0912025G	YQE_09424	Dendroctonus ponderosae	Putative uncharacterized protein
EOG0912025J	OTAU000653	Onthophagus taurus	Putative uncharacterized protein
EOG0912025J	AGLA006612	Anoplophora glabripennis	Putative uncharacterized protein
EOG0912025J	APLA002336	Agrilus planipennis	Putative uncharacterized protein
EOG0912025J	TC011889	Tribolium castaneum	Putative uncharacterized protein
EOG0912025J	LDEC007264	Leptinotarsa decemlineata	Putative uncharacterized protein
EOG0912025J	YQE_00219	Dendroctonus ponderosae	Putative uncharacterized protein
EOG0912025L	OTAU002371	Onthophagus taurus	Putative uncharacterized protein
EOG0912025L	AGLA014893	Anoplophora glabripennis	Putative uncharacterized protein
EOG0912025L	APLA004240	Agrilus planipennis	Putative uncharacterized protein
EOG0912025L	TC007099	Tribolium castaneum	Putative uncharacterized protein
EOG0912025L	LDEC006468	Leptinotarsa decemlineata	Putative uncharacterized protein
EOG0912025L	YQE_07531	Dendroctonus ponderosae	Putative uncharacterized protein
EOG0912025Q	OTAU012345	Onthophagus taurus	Putative uncharacterized protein
EOG0912025Q	AGLA005483	Anoplophora glabripennis	Putative uncharacterized protein
EOG0912025Q	APLA011583	Agrilus planipennis	Putative uncharacterized protein
EOG0912025Q	TC013517	Tribolium castaneum	Putative uncharacterized protein
EOG0912025Q	LDEC008331	Leptinotarsa decemlineata	Putative uncharacterized protein
EOG0912025Q	YQE_02108	Dendroctonus ponderosae	Putative uncharacterized protein
EOG0912025S	OTAU000021	Onthophagus taurus	Putative uncharacterized protein
EOG0912025S	AGLA006390	Anoplophora glabripennis	Putative uncharacterized protein
EOG0912025S	APLA011177	Agrilus planipennis	Putative uncharacterized protein
EOG0912025S	TC014464	Tribolium castaneum	Putative uncharacterized protein
EOG0912025S	LDEC013327	Leptinotarsa decemlineata	Putative uncharacterized protein
EOG0912025S	YQE_02055	Dendroctonus ponderosae	Putative uncharacterized protein
EOG0912025U	OTAU000485	Onthophagus taurus	Putative uncharacterized protein
EOG0912025U	AGLA008174	Anoplophora glabripennis	Putative uncharacterized protein
EOG0912025U	APLA003992	Agrilus planipennis	Putative uncharacterized protein
EOG0912025U	TC011667	Tribolium castaneum	Putative uncharacterized protein
EOG0912025U	LDEC000559	Leptinotarsa decemlineata	Putative uncharacterized protein
EOG0912025U	YQE_12905	Dendroctonus ponderosae	Putative uncharacterized protein
EOG0912025V	OTAU001967	Onthophagus taurus	Putative uncharacterized protein
EOG0912025V	AGLA012484	Anoplophora glabripennis	Putative uncharacterized protein
EOG0912025V	APLA011059	Agrilus planipennis	Putative uncharacterized protein
EOG0912025V	TC006413	Tribolium castaneum	Putative uncharacterized protein
EOG0912025V	LDEC005866	Leptinotarsa decemlineata	Putative uncharacterized protein
EOG0912025V	YQE_12681	Dendroctonus ponderosae	Putative uncharacterized protein
EOG0912025Y	OTAU006783	Onthophagus taurus	Putative uncharacterized protein
EOG0912025Y	AGLA000511	Anoplophora glabripennis	Putative uncharacterized protein
EOG0912025Y	APLA013389	Agrilus planipennis	Putative uncharacterized protein
EOG0912025Y	TC007512	Tribolium castaneum	Putative uncharacterized protein

EOG0912025Y	LDEC002380	Leptinotarsa decemlineata	Putative uncharacterized protein
EOG0912025Y	YQE_06858	Dendroctonus ponderosae	Putative uncharacterized protein
EOG0912025Z	OTAU009727	Onthophagus taurus	Putative uncharacterized protein
EOG0912025Z	AGLA000689	Anoplophora glabripennis	Putative uncharacterized protein
EOG0912025Z	APLA012540	Agrilus planipennis	Putative uncharacterized protein
EOG0912025Z	TC008188	Tribolium castaneum	Putative uncharacterized protein
EOG0912025Z	LDEC000016	Leptinotarsa decemlineata	Putative uncharacterized protein
EOG0912025Z	YQE_09706	Dendroctonus ponderosae	Putative uncharacterized protein
EOG09120260	OTAU002435	Onthophagus taurus	Putative uncharacterized protein
EOG09120260	AGLA013113	Anoplophora glabripennis	Putative uncharacterized protein
EOG09120260	APLA008133	Agrilus planipennis	Putative uncharacterized protein
EOG09120260	TC010386	Tribolium castaneum	Putative uncharacterized protein
EOG09120260	LDEC023106	Leptinotarsa decemlineata	Putative uncharacterized protein
EOG09120260	YQE_09849	Dendroctonus ponderosae	Putative uncharacterized protein
EOG09120261	OTAU009701	Onthophagus taurus	Putative uncharacterized protein
EOG09120261	AGLA009032	Anoplophora glabripennis	Putative uncharacterized protein
EOG09120261	APLA006582	Agrilus planipennis	Putative uncharacterized protein
EOG09120261	TC007397	Tribolium castaneum	Putative uncharacterized protein
EOG09120261	LDEC018534	Leptinotarsa decemlineata	Putative uncharacterized protein
EOG09120261	YQE_08835	Dendroctonus ponderosae	Putative uncharacterized protein
EOG09120263	OTAU005355	Onthophagus taurus	"Glutamyl-tRNA(Gln) amidotransferase subunit B, mitochondrial "
EOG09120263	AGLA007517	Anoplophora glabripennis	"Glutamyl-tRNA(Gln) amidotransferase subunit B, mitochondrial "
EOG09120263	APLA006670	Agrilus planipennis	"Glutamyl-tRNA(Gln) amidotransferase subunit B, mitochondrial "
EOG09120263	TC030768	Tribolium castaneum	"Glutamyl-tRNA(Gln) amidotransferase subunit B, mitochondrial "
EOG09120263	LDEC013857	Leptinotarsa decemlineata	"Glutamyl-tRNA(Gln) amidotransferase subunit B, mitochondrial "
EOG09120263	YQE_09811	Dendroctonus ponderosae	"Glutamyl-tRNA(Gln) amidotransferase subunit B, mitochondrial "
EOG09120266	OTAU006530	Onthophagus taurus	Putative uncharacterized protein
EOG09120266	AGLA002102	Anoplophora glabripennis	Putative uncharacterized protein
EOG09120266	APLA014639	Agrilus planipennis	Putative uncharacterized protein
EOG09120266	TC002298	Tribolium castaneum	Putative uncharacterized protein
EOG09120266	LDEC009205	Leptinotarsa decemlineata	Putative uncharacterized protein
EOG09120266	YQE_07824	Dendroctonus ponderosae	Putative uncharacterized protein
EOG09120269	OTAU002047	Onthophagus taurus	Putative uncharacterized protein
EOG09120269	AGLA017463	Anoplophora glabripennis	Putative uncharacterized protein
EOG09120269	APLA003227	Agrilus planipennis	Putative uncharacterized protein
EOG09120269	TC008530	Tribolium castaneum	Putative uncharacterized protein
EOG09120269	LDEC020980	Leptinotarsa decemlineata	Putative uncharacterized protein
EOG09120269	YQE_06508	Dendroctonus ponderosae	Putative uncharacterized protein
EOG0912026B	OTAU011729	Onthophagus taurus	Putative uncharacterized protein
EOG0912026B	AGLA010411	Anoplophora glabripennis	Putative uncharacterized protein
EOG0912026B	APLA014517	Agrilus planipennis	Putative uncharacterized protein
EOG0912026B	TC007573	Tribolium castaneum	Putative uncharacterized protein
EOG0912026B	LDEC000191	Leptinotarsa decemlineata	Putative uncharacterized protein
EOG0912026B	YQE_02380	Dendroctonus ponderosae	Putative uncharacterized protein
EOG0912026E	OTAU006807	Onthophagus taurus	Putative uncharacterized protein
EOG0912026E	AGLA018419	Anoplophora glabripennis	Putative uncharacterized protein
EOG0912026E	APLA002012	Agrilus planipennis	Putative uncharacterized protein
EOG0912026E	TC003021	Tribolium castaneum	Putative uncharacterized protein
EOG0912026E	LDEC005929	Leptinotarsa decemlineata	Putative uncharacterized protein
EOG0912026E	YQE_02754	Dendroctonus ponderosae	Putative uncharacterized protein
EOG0912026F	OTAU010218	Onthophagus taurus	"Glutamyl-tRNA(Gln) amidotransferase subunit A, mitochondrial "
EOG0912026F	AGLA016080	Anoplophora glabripennis	"Glutamyl-tRNA(Gln) amidotransferase subunit A, mitochondrial "
EOG0912026F	APLA011722	Agrilus planipennis	"Glutamyl-tRNA(Gln) amidotransferase subunit A, mitochondrial "
EOG0912026F	TC013788	Tribolium castaneum	"Glutamyl-tRNA(Gln) amidotransferase subunit A, mitochondrial "
EOG0912026F	LDEC014012	Leptinotarsa decemlineata	"Glutamyl-tRNA(Gln) amidotransferase subunit A, mitochondrial "

EOG0912026F	YQE_02771	Dendroctonus ponderosae	"Glutamyl-tRNA(Gln) amidotransferase subunit A, mitochondrial "
EOG0912026G	OTAU012433	Onthophagus taurus	Putative uncharacterized protein
EOG0912026G	AGLA009228	Anoplophora glabripennis	Putative uncharacterized protein
EOG0912026G	APLA008770	Agrilus planipennis	Putative uncharacterized protein
EOG0912026G	TC015316	Tribolium castaneum	Putative uncharacterized protein
EOG0912026G	LDEC014927	Leptinotarsa decemlineata	Putative uncharacterized protein
EOG0912026G	YQE_10843	Dendroctonus ponderosae	Putative uncharacterized protein
EOG0912026N	OTAU003050	Onthophagus taurus	Putative uncharacterized protein
EOG0912026N	AGLA012766	Anoplophora glabripennis	Putative uncharacterized protein
EOG0912026N	APLA004788	Agrilus planipennis	Putative uncharacterized protein
EOG0912026N	TC006507	Tribolium castaneum	Putative uncharacterized protein
EOG0912026N	LDEC011711	Leptinotarsa decemlineata	Putative uncharacterized protein
EOG0912026N	YQE_05539	Dendroctonus ponderosae	Putative uncharacterized protein
EOG0912026O	OTAU009141	Onthophagus taurus	Putative uncharacterized protein
EOG0912026O	AGLA006646	Anoplophora glabripennis	Putative uncharacterized protein
EOG0912026O	APLA000829	Agrilus planipennis	Putative uncharacterized protein
EOG0912026O	TC003716	Tribolium castaneum	Putative uncharacterized protein
EOG0912026O	LDEC011097	Leptinotarsa decemlineata	Putative uncharacterized protein
EOG0912026O	YQE_05145	Dendroctonus ponderosae	Putative uncharacterized protein
EOG0912026P	OTAU010682	Onthophagus taurus	Encore
EOG0912026P	AGLA003441	Anoplophora glabripennis	Encore
EOG0912026P	APLA013225	Agrilus planipennis	Encore
EOG0912026P	TC009582	Tribolium castaneum	Encore
EOG0912026P	LDEC016670	Leptinotarsa decemlineata	Encore
EOG0912026P	YQE_04097	Dendroctonus ponderosae	Encore
EOG0912026R	OTAU010985	Onthophagus taurus	Putative uncharacterized protein
EOG0912026R	AGLA008112	Anoplophora glabripennis	Putative uncharacterized protein
EOG0912026R	APLA011138	Agrilus planipennis	Putative uncharacterized protein
EOG0912026R	TC000585	Tribolium castaneum	Putative uncharacterized protein
EOG0912026R	LDEC003171	Leptinotarsa decemlineata	Putative uncharacterized protein
EOG0912026R	YQE_07745	Dendroctonus ponderosae	Putative uncharacterized protein
EOG0912026U	OTAU005661	Onthophagus taurus	Putative uncharacterized protein
EOG0912026U	AGLA017320	Anoplophora glabripennis	Putative uncharacterized protein
EOG0912026U	APLA000728	Agrilus planipennis	Putative uncharacterized protein
EOG0912026U	TC002792	Tribolium castaneum	Putative uncharacterized protein
EOG0912026U	LDEC000216	Leptinotarsa decemlineata	Putative uncharacterized protein
EOG0912026U	YQE_07435	Dendroctonus ponderosae	Putative uncharacterized protein
EOG0912026X	OTAU009810	Onthophagus taurus	Putative uncharacterized protein
EOG0912026X	AGLA018742	Anoplophora glabripennis	Putative uncharacterized protein
EOG0912026X	APLA009657	Agrilus planipennis	Putative uncharacterized protein
EOG0912026X	TC005308	Tribolium castaneum	Putative uncharacterized protein
EOG0912026X	LDEC004903	Leptinotarsa decemlineata	Putative uncharacterized protein
EOG0912026X	YQE_05628	Dendroctonus ponderosae	Putative uncharacterized protein
EOG0912026Y	OTAU000063	Onthophagus taurus	Putative uncharacterized protein
EOG0912026Y	AGLA003996	Anoplophora glabripennis	Putative uncharacterized protein
EOG0912026Y	APLA009841	Agrilus planipennis	Putative uncharacterized protein
EOG0912026Y	TC013838	Tribolium castaneum	Putative uncharacterized protein
EOG0912026Y	LDEC020876	Leptinotarsa decemlineata	Putative uncharacterized protein
EOG0912026Y	YQE_03604	Dendroctonus ponderosae	Putative uncharacterized protein
EOG0912026Z	OTAU000090	Onthophagus taurus	Putative uncharacterized protein
EOG0912026Z	AGLA019745	Anoplophora glabripennis	Putative uncharacterized protein
EOG0912026Z	APLA002486	Agrilus planipennis	Putative uncharacterized protein
EOG0912026Z	TC013803	Tribolium castaneum	Putative uncharacterized protein
EOG0912026Z	LDEC022902	Leptinotarsa decemlineata	Putative uncharacterized protein
EOG0912026Z	YQE_10341	Dendroctonus ponderosae	Putative uncharacterized protein
EOG09120271	OTAU000229	Onthophagus taurus	None
EOG09120271	AGLA015186	Anoplophora glabripennis	None
EOG09120271	APLA011081	Agrilus planipennis	None
EOG09120271	TC033259	Tribolium castaneum	None
EOG09120271	LDEC006666	Leptinotarsa decemlineata	None
EOG09120271	YQE_03613	Dendroctonus ponderosae	None
EOG09120273	OTAU004597	Onthophagus taurus	acid phosphatase activity
EOG09120273	AGLA003070	Anoplophora glabripennis	acid phosphatase activity
EOG09120273	APLA000331	Agrilus planipennis	acid phosphatase activity
EOG09120273	TC032728	Tribolium castaneum	acid phosphatase activity
EOG09120273	LDEC008484	Leptinotarsa decemlineata	acid phosphatase activity

EOG09120273	YQE_11148	Dendroctonus ponderosae	acid phosphatase activity
EOG09120274	OTAU017057	Onthophagus taurus	Putative uncharacterized protein
EOG09120274	AGLA008271	Anoplophora glabripennis	Putative uncharacterized protein
EOG09120274	APLA005725	Agrilus planipennis	Putative uncharacterized protein
EOG09120274	TC009265	Tribolium castaneum	Putative uncharacterized protein
EOG09120274	LDEC001271	Leptinotarsa decemlineata	Putative uncharacterized protein
EOG09120274	YQE_13006	Dendroctonus ponderosae	Putative uncharacterized protein
EOG09120275	OTAU004357	Onthophagus taurus	DNA binding
EOG09120275	AGLA002374	Anoplophora glabripennis	DNA binding
EOG09120275	APLA003524	Agrilus planipennis	DNA binding
EOG09120275	TC031528	Tribolium castaneum	DNA binding
EOG09120275	LDEC018842	Leptinotarsa decemlineata	DNA binding
EOG09120275	YQE_06642	Dendroctonus ponderosae	DNA binding
EOG09120277	OTAU012613	Onthophagus taurus	Putative uncharacterized protein
EOG09120277	AGLA013121	Anoplophora glabripennis	Putative uncharacterized protein
EOG09120277	APLA001903	Agrilus planipennis	Putative uncharacterized protein
EOG09120277	TC000506	Tribolium castaneum	Putative uncharacterized protein
EOG09120277	LDEC003417	Leptinotarsa decemlineata	Putative uncharacterized protein
EOG09120277	YQE_07059	Dendroctonus ponderosae	Putative uncharacterized protein
EOG09120279	OTAU000101	Onthophagus taurus	Putative uncharacterized protein
EOG09120279	AGLA015345	Anoplophora glabripennis	Putative uncharacterized protein
EOG09120279	APLA008500	Agrilus planipennis	Putative uncharacterized protein
EOG09120279	TC014444	Tribolium castaneum	Putative uncharacterized protein
EOG09120279	LDEC005952	Leptinotarsa decemlineata	Putative uncharacterized protein
EOG09120279	YQE_07239	Dendroctonus ponderosae	Putative uncharacterized protein
EOG0912027F	OTAU001534	Onthophagus taurus	Putative uncharacterized protein
EOG0912027F	AGLA001242	Anoplophora glabripennis	Putative uncharacterized protein
EOG0912027F	APLA005757	Agrilus planipennis	Putative uncharacterized protein
EOG0912027F	TC005530	Tribolium castaneum	Putative uncharacterized protein
EOG0912027F	LDEC011056	Leptinotarsa decemlineata	Putative uncharacterized protein
EOG0912027F	YQE_06978	Dendroctonus ponderosae	Putative uncharacterized protein
EOG0912027H	OTAU003001	Onthophagus taurus	Putative uncharacterized protein
EOG0912027H	AGLA004059	Anoplophora glabripennis	Putative uncharacterized protein
EOG0912027H	APLA001607	Agrilus planipennis	Putative uncharacterized protein
EOG0912027H	TC009122	Tribolium castaneum	Putative uncharacterized protein
EOG0912027H	LDEC014456	Leptinotarsa decemlineata	Putative uncharacterized protein
EOG0912027H	YQE_10303	Dendroctonus ponderosae	Putative uncharacterized protein
EOG0912027I	OTAU015431	Onthophagus taurus	Putative uncharacterized protein
EOG0912027I	AGLA000646	Anoplophora glabripennis	Putative uncharacterized protein
EOG0912027I	APLA007965	Agrilus planipennis	Putative uncharacterized protein
EOG0912027I	TC001105	Tribolium castaneum	Putative uncharacterized protein
EOG0912027I	LDEC013065	Leptinotarsa decemlineata	Putative uncharacterized protein
EOG0912027I	YQE_04076	Dendroctonus ponderosae	Putative uncharacterized protein
EOG0912027J	OTAU010320	Onthophagus taurus	catalytic activity
EOG0912027J	AGLA001030	Anoplophora glabripennis	catalytic activity
EOG0912027J	APLA001794	Agrilus planipennis	catalytic activity
EOG0912027J	TC034287	Tribolium castaneum	catalytic activity
EOG0912027J	LDEC007506	Leptinotarsa decemlineata	catalytic activity
EOG0912027J	YQE_12974	Dendroctonus ponderosae	catalytic activity
EOG0912027L	OTAU003232	Onthophagus taurus	RAE1 RNA export 1 homolog
EOG0912027L	AGLA013782	Anoplophora glabripennis	RAE1 RNA export 1 homolog
EOG0912027L	APLA014840	Agrilus planipennis	RAE1 RNA export 1 homolog
EOG0912027L	TC009967	Tribolium castaneum	RAE1 RNA export 1 homolog
EOG0912027L	LDEC001214	Leptinotarsa decemlineata	RAE1 RNA export 1 homolog
EOG0912027L	YQE_03474	Dendroctonus ponderosae	RAE1 RNA export 1 homolog
EOG0912027M	OTAU002423	Onthophagus taurus	Putative uncharacterized protein
EOG0912027M	AGLA009150	Anoplophora glabripennis	Putative uncharacterized protein
EOG0912027M	APLA000149	Agrilus planipennis	Putative uncharacterized protein
EOG0912027M	TC005675	Tribolium castaneum	Putative uncharacterized protein
EOG0912027M	LDEC010786	Leptinotarsa decemlineata	Putative uncharacterized protein
EOG0912027M	YQE_04228	Dendroctonus ponderosae	Putative uncharacterized protein
EOG0912027N	OTAU000219	Onthophagus taurus	Polo
EOG0912027N	AGLA006421	Anoplophora glabripennis	Polo
EOG0912027N	APLA003101	Agrilus planipennis	Polo
EOG0912027N	TC014023	Tribolium castaneum	Polo
EOG0912027N	LDEC017189	Leptinotarsa decemlineata	Polo
EOG0912027N	YQE_05077	Dendroctonus ponderosae	Polo

EOG0912027Q	OTAU011539	Onthophagus taurus	Putative uncharacterized protein
EOG0912027Q	AGLA013500	Anoplophora glabripennis	Putative uncharacterized protein
EOG0912027Q	APLA009184	Agrilus planipennis	Putative uncharacterized protein
EOG0912027Q	TC002968	Tribolium castaneum	Putative uncharacterized protein
EOG0912027Q	LDEC007093	Leptinotarsa decemlineata	Putative uncharacterized protein
EOG0912027Q	YQE_11121	Dendroctonus ponderosae	Putative uncharacterized protein
EOG0912027U	OTAU012152	Onthophagus taurus	None
EOG0912027U	AGLA013162	Anoplophora glabripennis	None
EOG0912027U	APLA012079	Agrilus planipennis	None
EOG0912027U	TC033928	Tribolium castaneum	None
EOG0912027U	LDEC011190	Leptinotarsa decemlineata	None
EOG0912027U	YQE_06982	Dendroctonus ponderosae	None
EOG0912027V	OTAU015907	Onthophagus taurus	Putative uncharacterized protein
EOG0912027V	AGLA001013	Anoplophora glabripennis	Putative uncharacterized protein
EOG0912027V	APLA003189	Agrilus planipennis	Putative uncharacterized protein
EOG0912027V	TC012028	Tribolium castaneum	Putative uncharacterized protein
EOG0912027V	LDEC007486	Leptinotarsa decemlineata	Putative uncharacterized protein
EOG0912027V	YQE_04372	Dendroctonus ponderosae	Putative uncharacterized protein
EOG0912027W	OTAU001764	Onthophagus taurus	Putative uncharacterized protein
EOG0912027W	AGLA005305	Anoplophora glabripennis	Putative uncharacterized protein
EOG0912027W	APLA005792	Agrilus planipennis	Putative uncharacterized protein
EOG0912027W	TC006375	Tribolium castaneum	Putative uncharacterized protein
EOG0912027W	LDEC014093	Leptinotarsa decemlineata	Putative uncharacterized protein
EOG0912027W	YQE_07029	Dendroctonus ponderosae	Putative uncharacterized protein
EOG0912027X	OTAU000626	Onthophagus taurus	Putative uncharacterized protein
EOG0912027X	AGLA009787	Anoplophora glabripennis	Putative uncharacterized protein
EOG0912027X	APLA002323	Agrilus planipennis	Putative uncharacterized protein
EOG0912027X	TC012804	Tribolium castaneum	Putative uncharacterized protein
EOG0912027X	LDEC007743	Leptinotarsa decemlineata	Putative uncharacterized protein
EOG0912027X	YQE_10063	Dendroctonus ponderosae	Putative uncharacterized protein
EOG0912027Y	OTAU004526	Onthophagus taurus	Putative uncharacterized protein
EOG0912027Y	AGLA017109	Anoplophora glabripennis	Putative uncharacterized protein
EOG0912027Y	APLA007394	Agrilus planipennis	Putative uncharacterized protein
EOG0912027Y	TC011893	Tribolium castaneum	Putative uncharacterized protein
EOG0912027Y	LDEC007604	Leptinotarsa decemlineata	Putative uncharacterized protein
EOG0912027Y	YQE_07779	Dendroctonus ponderosae	Putative uncharacterized protein
EOG09120280	OTAU014962	Onthophagus taurus	hydrolase activity
EOG09120280	AGLA013741	Anoplophora glabripennis	hydrolase activity
EOG09120280	APLA000865	Agrilus planipennis	hydrolase activity
EOG09120280	TC031246	Tribolium castaneum	hydrolase activity
EOG09120280	LDEC003923	Leptinotarsa decemlineata	hydrolase activity
EOG09120280	YQE_07771	Dendroctonus ponderosae	hydrolase activity
EOG09120281	OTAU004510	Onthophagus taurus	Innexin
EOG09120281	AGLA003280	Anoplophora glabripennis	Innexin
EOG09120281	APLA001113	Agrilus planipennis	Innexin
EOG09120281	TC011709	Tribolium castaneum	Innexin
EOG09120281	LDEC014412	Leptinotarsa decemlineata	Innexin
EOG09120281	YQE_08519	Dendroctonus ponderosae	Innexin
EOG09120284	OTAU008062	Onthophagus taurus	Putative uncharacterized protein
EOG09120284	AGLA017521	Anoplophora glabripennis	Putative uncharacterized protein
EOG09120284	APLA002059	Agrilus planipennis	Putative uncharacterized protein
EOG09120284	TC001564	Tribolium castaneum	Putative uncharacterized protein
EOG09120284	LDEC010276	Leptinotarsa decemlineata	Putative uncharacterized protein
EOG09120284	YQE_11082	Dendroctonus ponderosae	Putative uncharacterized protein
EOG09120285	OTAU015359	Onthophagus taurus	Putative uncharacterized protein
EOG09120285	AGLA006312	Anoplophora glabripennis	Putative uncharacterized protein
EOG09120285	APLA013321	Agrilus planipennis	Putative uncharacterized protein
EOG09120285	TC030763	Tribolium castaneum	Putative uncharacterized protein
EOG09120285	LDEC013896	Leptinotarsa decemlineata	Putative uncharacterized protein
EOG09120285	YQE_13029	Dendroctonus ponderosae	Putative uncharacterized protein
EOG09120287	OTAU000278	Onthophagus taurus	Putative uncharacterized protein
EOG09120287	AGLA011400	Anoplophora glabripennis	Putative uncharacterized protein
EOG09120287	APLA012875	Agrilus planipennis	Putative uncharacterized protein
EOG09120287	TC014396	Tribolium castaneum	Putative uncharacterized protein
EOG09120287	LDEC006523	Leptinotarsa decemlineata	Putative uncharacterized protein
EOG09120287	YQE_07215	Dendroctonus ponderosae	Putative uncharacterized protein
EOG09120288	OTAU009483	Onthophagus taurus	Putative uncharacterized protein

EOG09120288	AGLA009973	Anoplophora glabripennis	Putative uncharacterized protein
EOG09120288	APLA010026	Agrilus planipennis	Putative uncharacterized protein
EOG09120288	TC015216	Tribolium castaneum	Putative uncharacterized protein
EOG09120288	LDEC010966	Leptinotarsa decemlineata	Putative uncharacterized protein
EOG09120288	YQE_10118	Dendroctonus ponderosae	Putative uncharacterized protein
EOG09120289	OTAU000942	Onthophagus taurus	Putative uncharacterized protein
EOG09120289	AGLA014757	Anoplophora glabripennis	Putative uncharacterized protein
EOG09120289	APLA001260	Agrilus planipennis	Putative uncharacterized protein
EOG09120289	TC004371	Tribolium castaneum	Putative uncharacterized protein
EOG09120289	LDEC010156	Leptinotarsa decemlineata	Putative uncharacterized protein
EOG09120289	YQE_06821	Dendroctonus ponderosae	Putative uncharacterized protein
EOG0912028A	OTAU000164	Onthophagus taurus	DNA binding
EOG0912028A	AGLA008828	Anoplophora glabripennis	DNA binding
EOG0912028A	APLA007205	Agrilus planipennis	DNA binding
EOG0912028A	TC031157	Tribolium castaneum	DNA binding
EOG0912028A	LDEC012480	Leptinotarsa decemlineata	DNA binding
EOG0912028A	YQE_08217	Dendroctonus ponderosae	DNA binding
EOG0912028B	OTAU011983	Onthophagus taurus	Putative uncharacterized protein
EOG0912028B	AGLA017286	Anoplophora glabripennis	Putative uncharacterized protein
EOG0912028B	APLA013895	Agrilus planipennis	Putative uncharacterized protein
EOG0912028B	TC014273	Tribolium castaneum	Putative uncharacterized protein
EOG0912028B	LDEC011419	Leptinotarsa decemlineata	Putative uncharacterized protein
EOG0912028B	YQE_04887	Dendroctonus ponderosae	Putative uncharacterized protein
EOG0912028E	OTAU005468	Onthophagus taurus	Putative uncharacterized protein
EOG0912028E	AGLA010807	Anoplophora glabripennis	Putative uncharacterized protein
EOG0912028E	APLA004842	Agrilus planipennis	Putative uncharacterized protein
EOG0912028E	TC013747	Tribolium castaneum	Putative uncharacterized protein
EOG0912028E	LDEC016367	Leptinotarsa decemlineata	Putative uncharacterized protein
EOG0912028E	YQE_09924	Dendroctonus ponderosae	Putative uncharacterized protein
EOG0912028F	OTAU005984	Onthophagus taurus	Putative uncharacterized protein
EOG0912028F	AGLA004650	Anoplophora glabripennis	Putative uncharacterized protein
EOG0912028F	APLA002054	Agrilus planipennis	Putative uncharacterized protein
EOG0912028F	TC011519	Tribolium castaneum	Putative uncharacterized protein
EOG0912028F	LDEC010292	Leptinotarsa decemlineata	Putative uncharacterized protein
EOG0912028F	YQE_08274	Dendroctonus ponderosae	Putative uncharacterized protein
EOG0912028H	OTAU001904	Onthophagus taurus	Putative uncharacterized protein
EOG0912028H	AGLA017729	Anoplophora glabripennis	Putative uncharacterized protein
EOG0912028H	APLA000457	Agrilus planipennis	Putative uncharacterized protein
EOG0912028H	TC005588	Tribolium castaneum	Putative uncharacterized protein
EOG0912028H	LDEC011700	Leptinotarsa decemlineata	Putative uncharacterized protein
EOG0912028H	YQE_05589	Dendroctonus ponderosae	Putative uncharacterized protein
EOG0912028I	OTAU006826	Onthophagus taurus	Putative uncharacterized protein
EOG0912028I	AGLA008580	Anoplophora glabripennis	Putative uncharacterized protein
EOG0912028I	APLA007323	Agrilus planipennis	Putative uncharacterized protein
EOG0912028I	TC010522	Tribolium castaneum	Putative uncharacterized protein
EOG0912028I	LDEC002681	Leptinotarsa decemlineata	Putative uncharacterized protein
EOG0912028I	YQE_04550	Dendroctonus ponderosae	Putative uncharacterized protein
EOG0912028K	OTAU001383	Onthophagus taurus	Putative uncharacterized protein
EOG0912028K	AGLA000391	Anoplophora glabripennis	Putative uncharacterized protein
EOG0912028K	APLA010444	Agrilus planipennis	Putative uncharacterized protein
EOG0912028K	TC013580	Tribolium castaneum	Putative uncharacterized protein
EOG0912028K	LDEC004692	Leptinotarsa decemlineata	Putative uncharacterized protein
EOG0912028K	YQE_06640	Dendroctonus ponderosae	Putative uncharacterized protein
EOG0912028M	OTAU008482	Onthophagus taurus	Putative uncharacterized protein
EOG0912028M	AGLA002091	Anoplophora glabripennis	Putative uncharacterized protein
EOG0912028M	APLA008161	Agrilus planipennis	Putative uncharacterized protein
EOG0912028M	TC003694	Tribolium castaneum	Putative uncharacterized protein
EOG0912028M	LDEC002023	Leptinotarsa decemlineata	Putative uncharacterized protein
EOG0912028M	YQE_06436	Dendroctonus ponderosae	Putative uncharacterized protein
EOG0912028N	OTAU006919	Onthophagus taurus	Putative uncharacterized protein
EOG0912028N	AGLA012001	Anoplophora glabripennis	Putative uncharacterized protein
EOG0912028N	APLA006917	Agrilus planipennis	Putative uncharacterized protein
EOG0912028N	TC010568	Tribolium castaneum	Putative uncharacterized protein
EOG0912028N	LDEC005181	Leptinotarsa decemlineata	Putative uncharacterized protein
EOG0912028N	YQE_06810	Dendroctonus ponderosae	Putative uncharacterized protein
EOG0912028Q	OTAU011076	Onthophagus taurus	Putative uncharacterized protein
EOG0912028Q	AGLA001614	Anoplophora glabripennis	Putative uncharacterized protein

EOG0912028Q	APLA002987	Agrilus planipennis	Putative uncharacterized protein
EOG0912028Q	TC000849	Tribolium castaneum	Putative uncharacterized protein
EOG0912028Q	LDEC014548	Leptinotarsa decemlineata	Putative uncharacterized protein
EOG0912028Q	YQE_03863	Dendroctonus ponderosae	Putative uncharacterized protein
EOG0912028R	OTAU015905	Onthophagus taurus	Putative uncharacterized protein
EOG0912028R	AGLA015003	Anoplophora glabripennis	Putative uncharacterized protein
EOG0912028R	APLA003187	Agrilus planipennis	Putative uncharacterized protein
EOG0912028R	TC012492	Tribolium castaneum	Putative uncharacterized protein
EOG0912028R	LDEC011813	Leptinotarsa decemlineata	Putative uncharacterized protein
EOG0912028R	YQE_08177	Dendroctonus ponderosae	Putative uncharacterized protein
EOG0912028T	OTAU012815	Onthophagus taurus	Putative uncharacterized protein
EOG0912028T	AGLA015073	Anoplophora glabripennis	Putative uncharacterized protein
EOG0912028T	APLA002556	Agrilus planipennis	Putative uncharacterized protein
EOG0912028T	TC015509	Tribolium castaneum	Putative uncharacterized protein
EOG0912028T	LDEC005611	Leptinotarsa decemlineata	Putative uncharacterized protein
EOG0912028T	YQE_05918	Dendroctonus ponderosae	Putative uncharacterized protein
EOG0912028U	OTAU004105	Onthophagus taurus	Putative uncharacterized protein
EOG0912028U	AGLA001709	Anoplophora glabripennis	Putative uncharacterized protein
EOG0912028U	APLA001847	Agrilus planipennis	Putative uncharacterized protein
EOG0912028U	TC009236	Tribolium castaneum	Putative uncharacterized protein
EOG0912028U	LDEC013991	Leptinotarsa decemlineata	Putative uncharacterized protein
EOG0912028U	YQE_04128	Dendroctonus ponderosae	Putative uncharacterized protein
EOG0912028V	OTAU001816	Onthophagus taurus	Basket
EOG0912028V	AGLA007304	Anoplophora glabripennis	Basket
EOG0912028V	APLA004432	Agrilus planipennis	Basket
EOG0912028V	TC006810	Tribolium castaneum	Basket
EOG0912028V	LDEC004935	Leptinotarsa decemlineata	Basket
EOG0912028V	YQE_10181	Dendroctonus ponderosae	Basket
EOG0912028W	OTAU002114	Onthophagus taurus	Putative uncharacterized protein
EOG0912028W	AGLA017197	Anoplophora glabripennis	Putative uncharacterized protein
EOG0912028W	APLA003243	Agrilus planipennis	Putative uncharacterized protein
EOG0912028W	TC012068	Tribolium castaneum	Putative uncharacterized protein
EOG0912028W	LDEC003865	Leptinotarsa decemlineata	Putative uncharacterized protein
EOG0912028W	YQE_10070	Dendroctonus ponderosae	Putative uncharacterized protein
EOG0912028X	OTAU000842	Onthophagus taurus	Putative uncharacterized protein
EOG0912028X	AGLA001334	Anoplophora glabripennis	Putative uncharacterized protein
EOG0912028X	APLA013032	Agrilus planipennis	Putative uncharacterized protein
EOG0912028X	TC004855	Tribolium castaneum	Putative uncharacterized protein
EOG0912028X	LDEC008037	Leptinotarsa decemlineata	Putative uncharacterized protein
EOG0912028X	YQE_07684	Dendroctonus ponderosae	Putative uncharacterized protein
EOG0912028Y	OTAU001970	Onthophagus taurus	Putative uncharacterized protein
EOG0912028Y	AGLA018475	Anoplophora glabripennis	Putative uncharacterized protein
EOG0912028Y	APLA011056	Agrilus planipennis	Putative uncharacterized protein
EOG0912028Y	TC005717	Tribolium castaneum	Putative uncharacterized protein
EOG0912028Y	LDEC006764	Leptinotarsa decemlineata	Putative uncharacterized protein
EOG0912028Y	YQE_12792	Dendroctonus ponderosae	Putative uncharacterized protein
EOG09120291	OTAU005222	Onthophagus taurus	protein kinase binding
EOG09120291	AGLA004073	Anoplophora glabripennis	protein kinase binding
EOG09120291	APLA006005	Agrilus planipennis	protein kinase binding
EOG09120291	TC031193	Tribolium castaneum	protein kinase binding
EOG09120291	LDEC012801	Leptinotarsa decemlineata	protein kinase binding
EOG09120291	YQE_09719	Dendroctonus ponderosae	protein kinase binding
EOG09120298	OTAU011037	Onthophagus taurus	Putative uncharacterized protein
EOG09120298	AGLA006382	Anoplophora glabripennis	Putative uncharacterized protein
EOG09120298	APLA008286	Agrilus planipennis	Putative uncharacterized protein
EOG09120298	TC013451	Tribolium castaneum	Putative uncharacterized protein
EOG09120298	LDEC011740	Leptinotarsa decemlineata	Putative uncharacterized protein
EOG09120298	YQE_08999	Dendroctonus ponderosae	Putative uncharacterized protein
EOG09120299	OTAU007473	Onthophagus taurus	Putative uncharacterized protein
EOG09120299	AGLA010437	Anoplophora glabripennis	Putative uncharacterized protein
EOG09120299	APLA014426	Agrilus planipennis	Putative uncharacterized protein
EOG09120299	TC030744	Tribolium castaneum	Putative uncharacterized protein
EOG09120299	LDEC013161	Leptinotarsa decemlineata	Putative uncharacterized protein
EOG09120299	YQE_11748	Dendroctonus ponderosae	Putative uncharacterized protein
EOG0912029C	OTAU000724	Onthophagus taurus	Putative uncharacterized protein
EOG0912029C	AGLA014655	Anoplophora glabripennis	Putative uncharacterized protein
EOG0912029C	APLA003282	Agrilus planipennis	Putative uncharacterized protein

EOG0912029C	TC012175	Tribolium castaneum	Putative uncharacterized protein
EOG0912029C	LDEC010259	Leptinotarsa decemlineata	Putative uncharacterized protein
EOG0912029C	YQE_09437	Dendroctonus ponderosae	Putative uncharacterized protein
EOG0912029D	OTAU004097	Onthophagus taurus	Putative uncharacterized protein
EOG0912029D	AGLA014888	Anoplophora glabripennis	Putative uncharacterized protein
EOG0912029D	APLA011268	Agrilus planipennis	Putative uncharacterized protein
EOG0912029D	TC008899	Tribolium castaneum	Putative uncharacterized protein
EOG0912029D	LDEC011847	Leptinotarsa decemlineata	Putative uncharacterized protein
EOG0912029D	YQE_05416	Dendroctonus ponderosae	Putative uncharacterized protein
EOG0912029E	OTAU015230	Onthophagus taurus	Putative uncharacterized protein
EOG0912029E	AGLA005729	Anoplophora glabripennis	Putative uncharacterized protein
EOG0912029E	APLA000559	Agrilus planipennis	Putative uncharacterized protein
EOG0912029E	TC015690	Tribolium castaneum	Putative uncharacterized protein
EOG0912029E	LDEC010954	Leptinotarsa decemlineata	Putative uncharacterized protein
EOG0912029E	YQE_11482	Dendroctonus ponderosae	Putative uncharacterized protein
EOG0912029I	OTAU010996	Onthophagus taurus	Putative uncharacterized protein
EOG0912029I	AGLA005607	Anoplophora glabripennis	Putative uncharacterized protein
EOG0912029I	APLA008067	Agrilus planipennis	Putative uncharacterized protein
EOG0912029I	TC013076	Tribolium castaneum	Putative uncharacterized protein
EOG0912029I	LDEC022290	Leptinotarsa decemlineata	Putative uncharacterized protein
EOG0912029I	YQE_12255	Dendroctonus ponderosae	Putative uncharacterized protein
EOG0912029K	OTAU000261	Onthophagus taurus	Putative uncharacterized protein
EOG0912029K	AGLA020613	Anoplophora glabripennis	Putative uncharacterized protein
EOG0912029K	APLA011091	Agrilus planipennis	Putative uncharacterized protein
EOG0912029K	TC014434	Tribolium castaneum	Putative uncharacterized protein
EOG0912029K	LDEC012551	Leptinotarsa decemlineata	Putative uncharacterized protein
EOG0912029K	YQE_01698	Dendroctonus ponderosae	Putative uncharacterized protein
EOG0912029L	OTAU010756	Onthophagus taurus	Putative uncharacterized protein
EOG0912029L	AGLA000599	Anoplophora glabripennis	Putative uncharacterized protein
EOG0912029L	APLA002025	Agrilus planipennis	Putative uncharacterized protein
EOG0912029L	TC003848	Tribolium castaneum	Putative uncharacterized protein
EOG0912029L	LDEC007908	Leptinotarsa decemlineata	Putative uncharacterized protein
EOG0912029L	YQE_05138	Dendroctonus ponderosae	Putative uncharacterized protein
EOG0912029M	OTAU004664	Onthophagus taurus	Putative uncharacterized protein
EOG0912029M	AGLA015772	Anoplophora glabripennis	Putative uncharacterized protein
EOG0912029M	APLA003722	Agrilus planipennis	Putative uncharacterized protein
EOG0912029M	TC010516	Tribolium castaneum	Putative uncharacterized protein
EOG0912029M	LDEC002665	Leptinotarsa decemlineata	Putative uncharacterized protein
EOG0912029M	YQE_02290	Dendroctonus ponderosae	Putative uncharacterized protein
EOG0912029N	OTAU001571	Onthophagus taurus	None
EOG0912029N	AGLA010184	Anoplophora glabripennis	None
EOG0912029N	APLA005771	Agrilus planipennis	None
EOG0912029N	TC031275	Tribolium castaneum	None
EOG0912029N	LDEC023590	Leptinotarsa decemlineata	None
EOG0912029N	YQE_06920	Dendroctonus ponderosae	None
EOG0912029P	OTAU006735	Onthophagus taurus	Putative uncharacterized protein
EOG0912029P	AGLA000649	Anoplophora glabripennis	Putative uncharacterized protein
EOG0912029P	APLA002990	Agrilus planipennis	Putative uncharacterized protein
EOG0912029P	TC001370	Tribolium castaneum	Putative uncharacterized protein
EOG0912029P	LDEC010508	Leptinotarsa decemlineata	Putative uncharacterized protein
EOG0912029P	YQE_03923	Dendroctonus ponderosae	Putative uncharacterized protein
EOG0912029R	OTAU015525	Onthophagus taurus	"mannosyl-oligosaccharide 1,2-alpha-mannosidase activity"
EOG0912029R	AGLA010149	Anoplophora glabripennis	"mannosyl-oligosaccharide 1,2-alpha-mannosidase activity"
EOG0912029R	APLA006340	Agrilus planipennis	"mannosyl-oligosaccharide 1,2-alpha-mannosidase activity"
EOG0912029R	TC031392	Tribolium castaneum	"mannosyl-oligosaccharide 1,2-alpha-mannosidase activity"
EOG0912029R	LDEC019990	Leptinotarsa decemlineata	"mannosyl-oligosaccharide 1,2-alpha-mannosidase activity"
EOG0912029R	YQE_03890	Dendroctonus ponderosae	"mannosyl-oligosaccharide 1,2-alpha-mannosidase activity"
EOG0912029T	OTAU001241	Onthophagus taurus	Putative uncharacterized protein
EOG0912029T	AGLA021444	Anoplophora glabripennis	Putative uncharacterized protein
EOG0912029T	APLA008717	Agrilus planipennis	Putative uncharacterized protein
EOG0912029T	TC004676	Tribolium castaneum	Putative uncharacterized protein

EOG0912029T	LDEC003545	Leptinotarsa decemlineata	Putative uncharacterized protein
EOG0912029T	YQE_03720	Dendroctonus ponderosae	Putative uncharacterized protein
EOG0912029W	OTAU007392	Onthophagus taurus	Putative uncharacterized protein
EOG0912029W	AGLA017289	Anoplophora glabripennis	Putative uncharacterized protein
EOG0912029W	APLA005121	Agrilus planipennis	Putative uncharacterized protein
EOG0912029W	TC013541	Tribolium castaneum	Putative uncharacterized protein
EOG0912029W	LDEC011418	Leptinotarsa decemlineata	Putative uncharacterized protein
EOG0912029W	YQE_04886	Dendroctonus ponderosae	Putative uncharacterized protein
EOG0912029X	OTAU002974	Onthophagus taurus	Putative uncharacterized protein
EOG0912029X	AGLA021401	Anoplophora glabripennis	Putative uncharacterized protein
EOG0912029X	APLA008795	Agrilus planipennis	Putative uncharacterized protein
EOG0912029X	TC009182	Tribolium castaneum	Putative uncharacterized protein
EOG0912029X	LDEC004437	Leptinotarsa decemlineata	Putative uncharacterized protein
EOG0912029X	YQE_04295	Dendroctonus ponderosae	Putative uncharacterized protein
EOG0912029Y	OTAU003812	Onthophagus taurus	None
EOG0912029Y	AGLA008395	Anoplophora glabripennis	None
EOG0912029Y	APLA008174	Agrilus planipennis	None
EOG0912029Y	TC033184	Tribolium castaneum	None
EOG0912029Y	LDEC020681	Leptinotarsa decemlineata	None
EOG0912029Y	YQE_11539	Dendroctonus ponderosae	None
EOG0912029Z	OTAU010658	Onthophagus taurus	Putative uncharacterized protein
EOG0912029Z	AGLA010223	Anoplophora glabripennis	Putative uncharacterized protein
EOG0912029Z	APLA004512	Agrilus planipennis	Putative uncharacterized protein
EOG0912029Z	TC006501	Tribolium castaneum	Putative uncharacterized protein
EOG0912029Z	LDEC002962	Leptinotarsa decemlineata	Putative uncharacterized protein
EOG0912029Z	YQE_07479	Dendroctonus ponderosae	Putative uncharacterized protein
EOG091202A2	OTAU012019	Onthophagus taurus	Putative uncharacterized protein
EOG091202A2	AGLA015919	Anoplophora glabripennis	Putative uncharacterized protein
EOG091202A2	APLA015425	Agrilus planipennis	Putative uncharacterized protein
EOG091202A2	TC015544	Tribolium castaneum	Putative uncharacterized protein
EOG091202A2	LDEC001943	Leptinotarsa decemlineata	Putative uncharacterized protein
EOG091202A2	YQE_05503	Dendroctonus ponderosae	Putative uncharacterized protein
EOG091202A6	OTAU014461	Onthophagus taurus	Putative uncharacterized protein
EOG091202A6	AGLA003269	Anoplophora glabripennis	Putative uncharacterized protein
EOG091202A6	APLA002372	Agrilus planipennis	Putative uncharacterized protein
EOG091202A6	TC011603	Tribolium castaneum	Putative uncharacterized protein
EOG091202A6	LDEC015998	Leptinotarsa decemlineata	Putative uncharacterized protein
EOG091202A6	YQE_12920	Dendroctonus ponderosae	Putative uncharacterized protein
EOG091202A7	OTAU002935	Onthophagus taurus	substrate-specific transmembrane transporter activity
EOG091202A7	AGLA015816	Anoplophora glabripennis	substrate-specific transmembrane transporter activity
EOG091202A7	APLA007523	Agrilus planipennis	substrate-specific transmembrane transporter activity
EOG091202A7	TC033857	Tribolium castaneum	substrate-specific transmembrane transporter activity
EOG091202A7	LDEC019999	Leptinotarsa decemlineata	substrate-specific transmembrane transporter activity
EOG091202A7	YQE_08842	Dendroctonus ponderosae	substrate-specific transmembrane transporter activity
EOG091202A8	OTAU006550	Onthophagus taurus	Putative uncharacterized protein
EOG091202A8	AGLA006776	Anoplophora glabripennis	Putative uncharacterized protein
EOG091202A8	APLA008143	Agrilus planipennis	Putative uncharacterized protein
EOG091202A8	TC003316	Tribolium castaneum	Putative uncharacterized protein
EOG091202A8	LDEC011548	Leptinotarsa decemlineata	Putative uncharacterized protein
EOG091202A8	YQE_10871	Dendroctonus ponderosae	Putative uncharacterized protein
EOG091202A9	OTAU000413	Onthophagus taurus	Methionine aminopeptidase
EOG091202A9	AGLA011334	Anoplophora glabripennis	Methionine aminopeptidase
EOG091202A9	APLA010400	Agrilus planipennis	Methionine aminopeptidase
EOG091202A9	TC006844	Tribolium castaneum	Methionine aminopeptidase
EOG091202A9	LDEC005746	Leptinotarsa decemlineata	Methionine aminopeptidase
EOG091202A9	YQE_12858	Dendroctonus ponderosae	Methionine aminopeptidase
EOG091202AA	OTAU002679	Onthophagus taurus	Putative uncharacterized protein
EOG091202AA	AGLA007029	Anoplophora glabripennis	Putative uncharacterized protein
EOG091202AA	APLA012978	Agrilus planipennis	Putative uncharacterized protein
EOG091202AA	TC010426	Tribolium castaneum	Putative uncharacterized protein
EOG091202AA	LDEC012345	Leptinotarsa decemlineata	Putative uncharacterized protein
EOG091202AA	YQE_11390	Dendroctonus ponderosae	Putative uncharacterized protein
EOG091202AB	OTAU000414	Onthophagus taurus	Putative uncharacterized protein
EOG091202AB	AGLA007816	Anoplophora glabripennis	Putative uncharacterized protein
EOG091202AB	APLA003206	Agrilus planipennis	Putative uncharacterized protein
EOG091202AB	TC012645	Tribolium castaneum	Putative uncharacterized protein
EOG091202AB	LDEC004247	Leptinotarsa decemlineata	Putative uncharacterized protein

EOG091202AB	YQE_08479	Dendroctonus ponderosae	Putative uncharacterized protein
EOG091202AC	OTAU011451	Onthophagus taurus	Putative uncharacterized protein
EOG091202AC	AGLA000639	Anoplophora glabripennis	Putative uncharacterized protein
EOG091202AC	APLA005341	Agrilus planipennis	Putative uncharacterized protein
EOG091202AC	TC001356	Tribolium castaneum	Putative uncharacterized protein
EOG091202AC	LDEC007056	Leptinotarsa decemlineata	Putative uncharacterized protein
EOG091202AC	YQE_07733	Dendroctonus ponderosae	Putative uncharacterized protein
EOG091202AE	OTAU010166	Onthophagus taurus	Putative uncharacterized protein
EOG091202AE	AGLA008270	Anoplophora glabripennis	Putative uncharacterized protein
EOG091202AE	APLA012581	Agrilus planipennis	Putative uncharacterized protein
EOG091202AE	TC009540	Tribolium castaneum	Putative uncharacterized protein
EOG091202AE	LDEC016646	Leptinotarsa decemlineata	Putative uncharacterized protein
EOG091202AE	YQE_13007	Dendroctonus ponderosae	Putative uncharacterized protein
EOG091202AG	OTAU007567	Onthophagus taurus	nucleotide binding
EOG091202AG	AGLA012522	Anoplophora glabripennis	nucleotide binding
EOG091202AG	APLA000504	Agrilus planipennis	nucleotide binding
EOG091202AG	TC034052	Tribolium castaneum	nucleotide binding
EOG091202AG	LDEC010081	Leptinotarsa decemlineata	nucleotide binding
EOG091202AG	YQE_05765	Dendroctonus ponderosae	nucleotide binding
EOG091202AH	OTAU007219	Onthophagus taurus	Putative uncharacterized protein
EOG091202AH	AGLA004277	Anoplophora glabripennis	Putative uncharacterized protein
EOG091202AH	APLA002041	Agrilus planipennis	Putative uncharacterized protein
EOG091202AH	TC011537	Tribolium castaneum	Putative uncharacterized protein
EOG091202AH	LDEC011317	Leptinotarsa decemlineata	Putative uncharacterized protein
EOG091202AH	YQE_04939	Dendroctonus ponderosae	Putative uncharacterized protein
EOG091202AI	OTAU005080	Onthophagus taurus	Putative uncharacterized protein
EOG091202AI	AGLA007281	Anoplophora glabripennis	Putative uncharacterized protein
EOG091202AI	APLA005759	Agrilus planipennis	Putative uncharacterized protein
EOG091202AI	TC006630	Tribolium castaneum	Putative uncharacterized protein
EOG091202AI	LDEC020674	Leptinotarsa decemlineata	Putative uncharacterized protein
EOG091202AI	YQE_09464	Dendroctonus ponderosae	Putative uncharacterized protein
EOG091202AK	OTAU002700	Onthophagus taurus	Putative uncharacterized protein
EOG091202AK	AGLA017885	Anoplophora glabripennis	Putative uncharacterized protein
EOG091202AK	APLA008298	Agrilus planipennis	Putative uncharacterized protein
EOG091202AK	TC014024	Tribolium castaneum	Putative uncharacterized protein
EOG091202AK	LDEC010763	Leptinotarsa decemlineata	Putative uncharacterized protein
EOG091202AK	YQE_09006	Dendroctonus ponderosae	Putative uncharacterized protein
EOG091202AL	OTAU002682	Onthophagus taurus	Putative uncharacterized protein
EOG091202AL	AGLA016744	Anoplophora glabripennis	Putative uncharacterized protein
EOG091202AL	APLA013126	Agrilus planipennis	Putative uncharacterized protein
EOG091202AL	TC013186	Tribolium castaneum	Putative uncharacterized protein
EOG091202AL	LDEC005966	Leptinotarsa decemlineata	Putative uncharacterized protein
EOG091202AL	YQE_06196	Dendroctonus ponderosae	Putative uncharacterized protein
EOG091202AO	OTAU001063	Onthophagus taurus	None
EOG091202AO	AGLA014159	Anoplophora glabripennis	None
EOG091202AO	APLA007947	Agrilus planipennis	None
EOG091202AO	TC034900	Tribolium castaneum	None
EOG091202AO	LDEC010324	Leptinotarsa decemlineata	None
EOG091202AO	YQE_10754	Dendroctonus ponderosae	None
EOG091202AP	OTAU002849	Onthophagus taurus	Putative uncharacterized protein
EOG091202AP	AGLA002156	Anoplophora glabripennis	Putative uncharacterized protein
EOG091202AP	APLA006758	Agrilus planipennis	Putative uncharacterized protein
EOG091202AP	TC010850	Tribolium castaneum	Putative uncharacterized protein
EOG091202AP	LDEC004212	Leptinotarsa decemlineata	Putative uncharacterized protein
EOG091202AP	YQE_09060	Dendroctonus ponderosae	Putative uncharacterized protein
EOG091202AS	OTAU012544	Onthophagus taurus	Putative uncharacterized protein
EOG091202AS	AGLA000831	Anoplophora glabripennis	Putative uncharacterized protein
EOG091202AS	APLA002172	Agrilus planipennis	Putative uncharacterized protein
EOG091202AS	TC008324	Tribolium castaneum	Putative uncharacterized protein
EOG091202AS	LDEC002092	Leptinotarsa decemlineata	Putative uncharacterized protein
EOG091202AS	YQE_04184	Dendroctonus ponderosae	Putative uncharacterized protein
EOG091202AT	OTAU009174	Onthophagus taurus	Putative uncharacterized protein
EOG091202AT	AGLA002518	Anoplophora glabripennis	Putative uncharacterized protein
EOG091202AT	APLA000698	Agrilus planipennis	Putative uncharacterized protein
EOG091202AT	TC004246	Tribolium castaneum	Putative uncharacterized protein
EOG091202AT	LDEC003596	Leptinotarsa decemlineata	Putative uncharacterized protein
EOG091202AT	YQE_06532	Dendroctonus ponderosae	Putative uncharacterized protein

EOG091202AU	OTAU012325	Onthophagus taurus	Putative uncharacterized protein
EOG091202AU	AGLA018297	Anoplophora glabripennis	Putative uncharacterized protein
EOG091202AU	APLA015217	Agrilus planipennis	Putative uncharacterized protein
EOG091202AU	TC013357	Tribolium castaneum	Putative uncharacterized protein
EOG091202AU	LDEC013030	Leptinotarsa decemlineata	Putative uncharacterized protein
EOG091202AU	YQE_01858	Dendroctonus ponderosae	Putative uncharacterized protein
EOG091202AY	OTAU016157	Onthophagus taurus	Putative uncharacterized protein
EOG091202AY	AGLA012734	Anoplophora glabripennis	Putative uncharacterized protein
EOG091202AY	APLA001705	Agrilus planipennis	Putative uncharacterized protein
EOG091202AY	TC014863	Tribolium castaneum	Putative uncharacterized protein
EOG091202AY	LDEC009948	Leptinotarsa decemlineata	Putative uncharacterized protein
EOG091202AY	YQE_11477	Dendroctonus ponderosae	Putative uncharacterized protein
EOG091202B0	OTAU011699	Onthophagus taurus	Putative uncharacterized protein
EOG091202B0	AGLA009245	Anoplophora glabripennis	Putative uncharacterized protein
EOG091202B0	APLA014872	Agrilus planipennis	Putative uncharacterized protein
EOG091202B0	TC006048	Tribolium castaneum	Putative uncharacterized protein
EOG091202B0	LDEC019174	Leptinotarsa decemlineata	Putative uncharacterized protein
EOG091202B0	YQE_08354	Dendroctonus ponderosae	Putative uncharacterized protein
EOG091202B1	OTAU016150	Onthophagus taurus	Putative uncharacterized protein
EOG091202B1	AGLA010988	Anoplophora glabripennis	Putative uncharacterized protein
EOG091202B1	APLA008506	Agrilus planipennis	Putative uncharacterized protein
EOG091202B1	TC014452	Tribolium castaneum	Putative uncharacterized protein
EOG091202B1	LDEC015610	Leptinotarsa decemlineata	Putative uncharacterized protein
EOG091202B1	YQE_06120	Dendroctonus ponderosae	Putative uncharacterized protein
EOG091202B6	OTAU017150	Onthophagus taurus	Putative uncharacterized protein
EOG091202B6	AGLA003322	Anoplophora glabripennis	Putative uncharacterized protein
EOG091202B6	APLA000578	Agrilus planipennis	Putative uncharacterized protein
EOG091202B6	TC015057	Tribolium castaneum	Putative uncharacterized protein
EOG091202B6	LDEC007896	Leptinotarsa decemlineata	Putative uncharacterized protein
EOG091202B6	YQE_08366	Dendroctonus ponderosae	Putative uncharacterized protein
EOG091202B8	OTAU006381	Onthophagus taurus	Putative uncharacterized protein
EOG091202B8	AGLA019314	Anoplophora glabripennis	Putative uncharacterized protein
EOG091202B8	APLA013328	Agrilus planipennis	Putative uncharacterized protein
EOG091202B8	TC030595	Tribolium castaneum	Putative uncharacterized protein
EOG091202B8	LDEC006157	Leptinotarsa decemlineata	Putative uncharacterized protein
EOG091202B8	YQE_08777	Dendroctonus ponderosae	Putative uncharacterized protein
EOG091202BB	OTAU009966	Onthophagus taurus	Putative uncharacterized protein
EOG091202BB	AGLA000832	Anoplophora glabripennis	Putative uncharacterized protein
EOG091202BB	APLA008316	Agrilus planipennis	Putative uncharacterized protein
EOG091202BB	TC008273	Tribolium castaneum	Putative uncharacterized protein
EOG091202BB	LDEC006029	Leptinotarsa decemlineata	Putative uncharacterized protein
EOG091202BB	YQE_04523	Dendroctonus ponderosae	Putative uncharacterized protein
EOG091202BC	OTAU000495	Onthophagus taurus	Putative uncharacterized protein
EOG091202BC	AGLA008177	Anoplophora glabripennis	Putative uncharacterized protein
EOG091202BC	APLA002318	Agrilus planipennis	Putative uncharacterized protein
EOG091202BC	TC011653	Tribolium castaneum	Putative uncharacterized protein
EOG091202BC	LDEC000535	Leptinotarsa decemlineata	Putative uncharacterized protein
EOG091202BC	YQE_12892	Dendroctonus ponderosae	Putative uncharacterized protein
EOG091202BF	OTAU004496	Onthophagus taurus	Putative uncharacterized protein
EOG091202BF	AGLA012232	Anoplophora glabripennis	Putative uncharacterized protein
EOG091202BF	APLA011472	Agrilus planipennis	Putative uncharacterized protein
EOG091202BF	TC003476	Tribolium castaneum	Putative uncharacterized protein
EOG091202BF	LDEC007834	Leptinotarsa decemlineata	Putative uncharacterized protein
EOG091202BF	YQE_12320	Dendroctonus ponderosae	Putative uncharacterized protein
EOG091202BI	OTAU001692	Onthophagus taurus	Putative uncharacterized protein
EOG091202BI	AGLA019330	Anoplophora glabripennis	Putative uncharacterized protein
EOG091202BI	APLA005066	Agrilus planipennis	Putative uncharacterized protein
EOG091202BI	TC006210	Tribolium castaneum	Putative uncharacterized protein
EOG091202BI	LDEC010096	Leptinotarsa decemlineata	Putative uncharacterized protein
EOG091202BI	YQE_05641	Dendroctonus ponderosae	Putative uncharacterized protein
EOG091202BK	OTAU006843	Onthophagus taurus	Putative uncharacterized protein
EOG091202BK	AGLA021476	Anoplophora glabripennis	Putative uncharacterized protein
EOG091202BK	APLA006141	Agrilus planipennis	Putative uncharacterized protein
EOG091202BK	TC014040	Tribolium castaneum	Putative uncharacterized protein
EOG091202BK	LDEC014503	Leptinotarsa decemlineata	Putative uncharacterized protein
EOG091202BK	YQE_07264	Dendroctonus ponderosae	Putative uncharacterized protein
EOG091202BL	OTAU003693	Onthophagus taurus	Putative uncharacterized protein

EOG091202BL	AGLA010423	Anoplophora glabripennis	Putative uncharacterized protein
EOG091202BL	APLA005381	Agrilus planipennis	Putative uncharacterized protein
EOG091202BL	TC007326	Tribolium castaneum	Putative uncharacterized protein
EOG091202BL	LDEC000159	Leptinotarsa decemlineata	Putative uncharacterized protein
EOG091202BL	YQE_04605	Dendroctonus ponderosae	Putative uncharacterized protein
EOG091202BN	OTAU004191	Onthophagus taurus	Putative uncharacterized protein
EOG091202BN	AGLA010788	Anoplophora glabripennis	Putative uncharacterized protein
EOG091202BN	APLA005138	Agrilus planipennis	Putative uncharacterized protein
EOG091202BN	TC013676	Tribolium castaneum	Putative uncharacterized protein
EOG091202BN	LDEC006506	Leptinotarsa decemlineata	Putative uncharacterized protein
EOG091202BN	YQE_06171	Dendroctonus ponderosae	Putative uncharacterized protein
EOG091202BP	OTAU000662	Onthophagus taurus	CLOCK
EOG091202BP	AGLA012952	Anoplophora glabripennis	CLOCK
EOG091202BP	APLA006615	Agrilus planipennis	CLOCK
EOG091202BP	TC000088	Tribolium castaneum	CLOCK
EOG091202BP	LDEC003378	Leptinotarsa decemlineata	CLOCK
EOG091202BP	YQE_03736	Dendroctonus ponderosae	CLOCK
EOG091202BR	OTAU016770	Onthophagus taurus	Putative uncharacterized protein
EOG091202BR	AGLA000501	Anoplophora glabripennis	Putative uncharacterized protein
EOG091202BR	APLA000073	Agrilus planipennis	Putative uncharacterized protein
EOG091202BR	TC007615	Tribolium castaneum	Putative uncharacterized protein
EOG091202BR	LDEC002390	Leptinotarsa decemlineata	Putative uncharacterized protein
EOG091202BR	YQE_12427	Dendroctonus ponderosae	Putative uncharacterized protein
EOG091202BS	OTAU008171	Onthophagus taurus	Putative uncharacterized protein
EOG091202BS	AGLA021376	Anoplophora glabripennis	Putative uncharacterized protein
EOG091202BS	APLA006474	Agrilus planipennis	Putative uncharacterized protein
EOG091202BS	TC015634	Tribolium castaneum	Putative uncharacterized protein
EOG091202BS	LDEC003704	Leptinotarsa decemlineata	Putative uncharacterized protein
EOG091202BS	YQE_11047	Dendroctonus ponderosae	Putative uncharacterized protein
EOG091202BU	OTAU012956	Onthophagus taurus	Putative uncharacterized protein
EOG091202BU	AGLA002383	Anoplophora glabripennis	Putative uncharacterized protein
EOG091202BU	APLA003536	Agrilus planipennis	Putative uncharacterized protein
EOG091202BU	TC001612	Tribolium castaneum	Putative uncharacterized protein
EOG091202BU	LDEC011952	Leptinotarsa decemlineata	Putative uncharacterized protein
EOG091202BU	YQE_05976	Dendroctonus ponderosae	Putative uncharacterized protein
EOG091202BX	OTAU010909	Onthophagus taurus	Putative uncharacterized protein
EOG091202BX	AGLA009980	Anoplophora glabripennis	Putative uncharacterized protein
EOG091202BX	APLA002548	Agrilus planipennis	Putative uncharacterized protein
EOG091202BX	TC014882	Tribolium castaneum	Putative uncharacterized protein
EOG091202BX	LDEC010980	Leptinotarsa decemlineata	Putative uncharacterized protein
EOG091202BX	YQE_10132	Dendroctonus ponderosae	Putative uncharacterized protein
EOG091202BY	OTAU000077	Onthophagus taurus	Putative uncharacterized protein
EOG091202BY	AGLA007589	Anoplophora glabripennis	Putative uncharacterized protein
EOG091202BY	APLA006154	Agrilus planipennis	Putative uncharacterized protein
EOG091202BY	TC013857	Tribolium castaneum	Putative uncharacterized protein
EOG091202BY	LDEC003260	Leptinotarsa decemlineata	Putative uncharacterized protein
EOG091202BY	YQE_03596	Dendroctonus ponderosae	Putative uncharacterized protein
EOG091202BZ	OTAU013033	Onthophagus taurus	Putative uncharacterized protein
EOG091202BZ	AGLA003937	Anoplophora glabripennis	Putative uncharacterized protein
EOG091202BZ	APLA010701	Agrilus planipennis	Putative uncharacterized protein
EOG091202BZ	TC015831	Tribolium castaneum	Putative uncharacterized protein
EOG091202BZ	LDEC016765	Leptinotarsa decemlineata	Putative uncharacterized protein
EOG091202BZ	YQE_09397	Dendroctonus ponderosae	Putative uncharacterized protein
EOG091202C2	OTAU004637	Onthophagus taurus	None
EOG091202C2	AGLA004095	Anoplophora glabripennis	None
EOG091202C2	APLA006935	Agrilus planipennis	None
EOG091202C2	TC033797	Tribolium castaneum	None
EOG091202C2	LDEC018797	Leptinotarsa decemlineata	None
EOG091202C2	YQE_06016	Dendroctonus ponderosae	None
EOG091202C6	OTAU004299	Onthophagus taurus	Putative uncharacterized protein
EOG091202C6	AGLA013526	Anoplophora glabripennis	Putative uncharacterized protein
EOG091202C6	APLA010458	Agrilus planipennis	Putative uncharacterized protein
EOG091202C6	TC014215	Tribolium castaneum	Putative uncharacterized protein
EOG091202C6	LDEC001012	Leptinotarsa decemlineata	Putative uncharacterized protein
EOG091202C6	YQE_06733	Dendroctonus ponderosae	Putative uncharacterized protein
EOG091202C7	OTAU008553	Onthophagus taurus	Putative uncharacterized protein
EOG091202C7	AGLA017762	Anoplophora glabripennis	Putative uncharacterized protein

EOG091202C7	APLA000394	Agrilus planipennis	Putative uncharacterized protein
EOG091202C7	TC006428	Tribolium castaneum	Putative uncharacterized protein
EOG091202C7	LDEC018337	Leptinotarsa decemlineata	Putative uncharacterized protein
EOG091202C7	YQE_06903	Dendroctonus ponderosae	Putative uncharacterized protein
EOG091202C8	OTAU002962	Onthophagus taurus	Putative uncharacterized protein
EOG091202C8	AGLA011863	Anoplophora glabripennis	Putative uncharacterized protein
EOG091202C8	APLA003416	Agrilus planipennis	Putative uncharacterized protein
EOG091202C8	TC009830	Tribolium castaneum	Putative uncharacterized protein
EOG091202C8	LDEC002368	Leptinotarsa decemlineata	Putative uncharacterized protein
EOG091202C8	YQE_05050	Dendroctonus ponderosae	Putative uncharacterized protein
EOG091202CA	OTAU001388	Onthophagus taurus	transferase activity
EOG091202CA	AGLA013531	Anoplophora glabripennis	transferase activity
EOG091202CA	APLA005600	Agrilus planipennis	transferase activity
EOG091202CA	TC030882	Tribolium castaneum	transferase activity
EOG091202CA	LDEC017475	Leptinotarsa decemlineata	transferase activity
EOG091202CA	YQE_09200	Dendroctonus ponderosae	transferase activity
EOG091202CB	OTAU000463	Onthophagus taurus	Putative uncharacterized protein
EOG091202CB	AGLA006076	Anoplophora glabripennis	Putative uncharacterized protein
EOG091202CB	APLA012159	Agrilus planipennis	Putative uncharacterized protein
EOG091202CB	TC012597	Tribolium castaneum	Putative uncharacterized protein
EOG091202CB	LDEC009432	Leptinotarsa decemlineata	Putative uncharacterized protein
EOG091202CB	YQE_12851	Dendroctonus ponderosae	Putative uncharacterized protein
EOG091202CE	OTAU003866	Onthophagus taurus	Putative uncharacterized protein
EOG091202CE	AGLA014820	Anoplophora glabripennis	Putative uncharacterized protein
EOG091202CE	APLA005716	Agrilus planipennis	Putative uncharacterized protein
EOG091202CE	TC009299	Tribolium castaneum	Putative uncharacterized protein
EOG091202CE	LDEC004782	Leptinotarsa decemlineata	Putative uncharacterized protein
EOG091202CE	YQE_11929	Dendroctonus ponderosae	Putative uncharacterized protein
EOG091202CF	OTAU001319	Onthophagus taurus	Putative uncharacterized protein
EOG091202CF	AGLA000412	Anoplophora glabripennis	Putative uncharacterized protein
EOG091202CF	APLA003569	Agrilus planipennis	Putative uncharacterized protein
EOG091202CF	TC014173	Tribolium castaneum	Putative uncharacterized protein
EOG091202CF	LDEC012582	Leptinotarsa decemlineata	Putative uncharacterized protein
EOG091202CF	YQE_05971	Dendroctonus ponderosae	Putative uncharacterized protein
EOG091202CH	OTAU009196	Onthophagus taurus	Putative uncharacterized protein
EOG091202CH	AGLA016197	Anoplophora glabripennis	Putative uncharacterized protein
EOG091202CH	APLA000545	Agrilus planipennis	Putative uncharacterized protein
EOG091202CH	TC015051	Tribolium castaneum	Putative uncharacterized protein
EOG091202CH	LDEC008020	Leptinotarsa decemlineata	Putative uncharacterized protein
EOG091202CH	YQE_08332	Dendroctonus ponderosae	Putative uncharacterized protein
EOG091202CI	OTAU003548	Onthophagus taurus	Putative uncharacterized protein
EOG091202CI	AGLA009778	Anoplophora glabripennis	Putative uncharacterized protein
EOG091202CI	APLA002655	Agrilus planipennis	Putative uncharacterized protein
EOG091202CI	TC002265	Tribolium castaneum	Putative uncharacterized protein
EOG091202CI	LDEC003832	Leptinotarsa decemlineata	Putative uncharacterized protein
EOG091202CI	YQE_12915	Dendroctonus ponderosae	Putative uncharacterized protein
EOG091202CP	OTAU012926	Onthophagus taurus	Putative uncharacterized protein
EOG091202CP	AGLA010261	Anoplophora glabripennis	Putative uncharacterized protein
EOG091202CP	APLA000464	Agrilus planipennis	Putative uncharacterized protein
EOG091202CP	TC005482	Tribolium castaneum	Putative uncharacterized protein
EOG091202CP	LDEC009844	Leptinotarsa decemlineata	Putative uncharacterized protein
EOG091202CP	YQE_12737	Dendroctonus ponderosae	Putative uncharacterized protein
EOG091202CQ	OTAU012903	Onthophagus taurus	Putative uncharacterized protein
EOG091202CQ	AGLA013698	Anoplophora glabripennis	Putative uncharacterized protein
EOG091202CQ	APLA001400	Agrilus planipennis	Putative uncharacterized protein
EOG091202CQ	TC001237	Tribolium castaneum	Putative uncharacterized protein
EOG091202CQ	LDEC016546	Leptinotarsa decemlineata	Putative uncharacterized protein
EOG091202CQ	YQE_01697	Dendroctonus ponderosae	Putative uncharacterized protein
EOG091202CS	OTAU008197	Onthophagus taurus	nucleic acid binding
EOG091202CS	AGLA019638	Anoplophora glabripennis	nucleic acid binding
EOG091202CS	APLA010569	Agrilus planipennis	nucleic acid binding
EOG091202CS	TC032198	Tribolium castaneum	nucleic acid binding
EOG091202CS	LDEC014031	Leptinotarsa decemlineata	nucleic acid binding
EOG091202CS	YQE_11209	Dendroctonus ponderosae	nucleic acid binding
EOG091202CU	OTAU005286	Onthophagus taurus	Putative uncharacterized protein
EOG091202CU	AGLA003775	Anoplophora glabripennis	Putative uncharacterized protein
EOG091202CU	APLA011445	Agrilus planipennis	Putative uncharacterized protein

EOG091202CU	TC007382	Tribolium castaneum	Putative uncharacterized protein
EOG091202CU	LDEC007966	Leptinotarsa decemlineata	Putative uncharacterized protein
EOG091202CU	YQE_02568	Dendroctonus ponderosae	Putative uncharacterized protein
EOG091202CV	OTAU000346	Onthophagus taurus	Putative uncharacterized protein
EOG091202CV	AGLA005457	Anoplophora glabripennis	Putative uncharacterized protein
EOG091202CV	APLA010430	Agrilus planipennis	Putative uncharacterized protein
EOG091202CV	TC012544	Tribolium castaneum	Putative uncharacterized protein
EOG091202CV	LDEC018184	Leptinotarsa decemlineata	Putative uncharacterized protein
EOG091202CV	YQE_04306	Dendroctonus ponderosae	Putative uncharacterized protein
EOG091202CY	OTAU010422	Onthophagus taurus	Putative uncharacterized protein
EOG091202CY	AGLA015457	Anoplophora glabripennis	Putative uncharacterized protein
EOG091202CY	APLA005509	Agrilus planipennis	Putative uncharacterized protein
EOG091202CY	TC001165	Tribolium castaneum	Putative uncharacterized protein
EOG091202CY	LDEC003576	Leptinotarsa decemlineata	Putative uncharacterized protein
EOG091202CY	YQE_07721	Dendroctonus ponderosae	Putative uncharacterized protein
EOG091202CZ	OTAU014409	Onthophagus taurus	Putative uncharacterized protein
EOG091202CZ	AGLA015672	Anoplophora glabripennis	Putative uncharacterized protein
EOG091202CZ	APLA003484	Agrilus planipennis	Putative uncharacterized protein
EOG091202CZ	TC004803	Tribolium castaneum	Putative uncharacterized protein
EOG091202CZ	LDEC009162	Leptinotarsa decemlineata	Putative uncharacterized protein
EOG091202CZ	YQE_09280	Dendroctonus ponderosae	Putative uncharacterized protein
EOG091202D0	OTAU017269	Onthophagus taurus	Putative uncharacterized protein
EOG091202D0	AGLA018771	Anoplophora glabripennis	Putative uncharacterized protein
EOG091202D0	APLA011919	Agrilus planipennis	Putative uncharacterized protein
EOG091202D0	TC004416	Tribolium castaneum	Putative uncharacterized protein
EOG091202D0	LDEC003151	Leptinotarsa decemlineata	Putative uncharacterized protein
EOG091202D0	YQE_10987	Dendroctonus ponderosae	Putative uncharacterized protein
EOG091202D1	OTAU016361	Onthophagus taurus	None
EOG091202D1	AGLA003644	Anoplophora glabripennis	None
EOG091202D1	APLA009004	Agrilus planipennis	None
EOG091202D1	TC033237	Tribolium castaneum	None
EOG091202D1	LDEC006715	Leptinotarsa decemlineata	None
EOG091202D1	YQE_08598	Dendroctonus ponderosae	None
EOG091202D2	OTAU006485	Onthophagus taurus	metal ion binding
EOG091202D2	AGLA003527	Anoplophora glabripennis	metal ion binding
EOG091202D2	APLA012635	Agrilus planipennis	metal ion binding
EOG091202D2	TC032568	Tribolium castaneum	metal ion binding
EOG091202D2	LDEC012676	Leptinotarsa decemlineata	metal ion binding
EOG091202D2	YQE_11112	Dendroctonus ponderosae	metal ion binding
EOG091202D7	OTAU011926	Onthophagus taurus	Putative uncharacterized protein
EOG091202D7	AGLA007779	Anoplophora glabripennis	Putative uncharacterized protein
EOG091202D7	APLA001290	Agrilus planipennis	Putative uncharacterized protein
EOG091202D7	TC001231	Tribolium castaneum	Putative uncharacterized protein
EOG091202D7	LDEC019813	Leptinotarsa decemlineata	Putative uncharacterized protein
EOG091202D7	YQE_11207	Dendroctonus ponderosae	Putative uncharacterized protein
EOG091202D9	OTAU008338	Onthophagus taurus	Putative uncharacterized protein
EOG091202D9	AGLA006761	Anoplophora glabripennis	Putative uncharacterized protein
EOG091202D9	APLA006345	Agrilus planipennis	Putative uncharacterized protein
EOG091202D9	TC011388	Tribolium castaneum	Putative uncharacterized protein
EOG091202D9	LDEC007535	Leptinotarsa decemlineata	Putative uncharacterized protein
EOG091202D9	YQE_10954	Dendroctonus ponderosae	Putative uncharacterized protein
EOG091202DA	OTAU004416	Onthophagus taurus	None
EOG091202DA	AGLA013205	Anoplophora glabripennis	None
EOG091202DA	APLA010610	Agrilus planipennis	None
EOG091202DA	TC032825	Tribolium castaneum	None
EOG091202DA	LDEC004351	Leptinotarsa decemlineata	None
EOG091202DA	YQE_06843	Dendroctonus ponderosae	None
EOG091202DB	OTAU007364	Onthophagus taurus	Eukaryotic translation initiation factor 3 subunit M
EOG091202DB	AGLA008610	Anoplophora glabripennis	Eukaryotic translation initiation factor 3 subunit M
EOG091202DB	APLA001987	Agrilus planipennis	Eukaryotic translation initiation factor 3 subunit M
EOG091202DB	TC011022	Tribolium castaneum	Eukaryotic translation initiation factor 3 subunit M
EOG091202DB	LDEC022378	Leptinotarsa decemlineata	Eukaryotic translation initiation factor 3 subunit M
EOG091202DB	YQE_06367	Dendroctonus ponderosae	Eukaryotic translation initiation factor 3 subunit M
EOG091202DC	OTAU013333	Onthophagus taurus	Putative uncharacterized protein
EOG091202DC	AGLA003262	Anoplophora glabripennis	Putative uncharacterized protein
EOG091202DC	APLA015074	Agrilus planipennis	Putative uncharacterized protein
EOG091202DC	TC001643	Tribolium castaneum	Putative uncharacterized protein

EOG091202DC	LDEC016901	Leptinotarsa decemlineata	Putative uncharacterized protein
EOG091202DC	YQE_08046	Dendroctonus ponderosae	Putative uncharacterized protein
EOG091202DE	OTAU004578	Onthophagus taurus	Putative uncharacterized protein
EOG091202DE	AGLA003735	Anoplophora glabripennis	Putative uncharacterized protein
EOG091202DE	APLA009050	Agrilus planipennis	Putative uncharacterized protein
EOG091202DE	TC007685	Tribolium castaneum	Putative uncharacterized protein
EOG091202DE	LDEC022938	Leptinotarsa decemlineata	Putative uncharacterized protein
EOG091202DE	YQE_11430	Dendroctonus ponderosae	Putative uncharacterized protein
EOG091202DG	OTAU008226	Onthophagus taurus	None
EOG091202DG	AGLA005065	Anoplophora glabripennis	None
EOG091202DG	APLA015287	Agrilus planipennis	None
EOG091202DG	TC032219	Tribolium castaneum	None
EOG091202DG	LDEC013314	Leptinotarsa decemlineata	None
EOG091202DG	YQE_09489	Dendroctonus ponderosae	None
EOG091202DH	OTAU006662	Onthophagus taurus	Putative uncharacterized protein
EOG091202DH	AGLA009175	Anoplophora glabripennis	Putative uncharacterized protein
EOG091202DH	APLA002057	Agrilus planipennis	Putative uncharacterized protein
EOG091202DH	TC012980	Tribolium castaneum	Putative uncharacterized protein
EOG091202DH	LDEC010269	Leptinotarsa decemlineata	Putative uncharacterized protein
EOG091202DH	YQE_04947	Dendroctonus ponderosae	Putative uncharacterized protein
EOG091202DJ	OTAU012612	Onthophagus taurus	Punt
EOG091202DJ	AGLA008618	Anoplophora glabripennis	Punt
EOG091202DJ	APLA004443	Agrilus planipennis	Punt
EOG091202DJ	TC011357	Tribolium castaneum	Punt
EOG091202DJ	LDEC008502	Leptinotarsa decemlineata	Punt
EOG091202DJ	YQE_02369	Dendroctonus ponderosae	Punt
EOG091202DM	OTAU002530	Onthophagus taurus	Putative uncharacterized protein
EOG091202DM	AGLA005370	Anoplophora glabripennis	Putative uncharacterized protein
EOG091202DM	APLA013060	Agrilus planipennis	Putative uncharacterized protein
EOG091202DM	TC016342	Tribolium castaneum	Putative uncharacterized protein
EOG091202DM	LDEC014891	Leptinotarsa decemlineata	Putative uncharacterized protein
EOG091202DM	YQE_12502	Dendroctonus ponderosae	Putative uncharacterized protein
EOG091202DN	OTAU006379	Onthophagus taurus	Putative uncharacterized protein
EOG091202DN	AGLA019315	Anoplophora glabripennis	Putative uncharacterized protein
EOG091202DN	APLA004558	Agrilus planipennis	Putative uncharacterized protein
EOG091202DN	TC009450	Tribolium castaneum	Putative uncharacterized protein
EOG091202DN	LDEC006160	Leptinotarsa decemlineata	Putative uncharacterized protein
EOG091202DN	YQE_05725	Dendroctonus ponderosae	Putative uncharacterized protein
EOG091202DO	OTAU005995	Onthophagus taurus	Putative uncharacterized protein
EOG091202DO	AGLA018210	Anoplophora glabripennis	Putative uncharacterized protein
EOG091202DO	APLA002069	Agrilus planipennis	Putative uncharacterized protein
EOG091202DO	TC011548	Tribolium castaneum	Putative uncharacterized protein
EOG091202DO	LDEC010282	Leptinotarsa decemlineata	Putative uncharacterized protein
EOG091202DO	YQE_06449	Dendroctonus ponderosae	Putative uncharacterized protein
EOG091202DP	OTAU001642	Onthophagus taurus	Putative uncharacterized protein
EOG091202DP	AGLA018303	Anoplophora glabripennis	Putative uncharacterized protein
EOG091202DP	APLA001203	Agrilus planipennis	Putative uncharacterized protein
EOG091202DP	TC006307	Tribolium castaneum	Putative uncharacterized protein
EOG091202DP	LDEC017817	Leptinotarsa decemlineata	Putative uncharacterized protein
EOG091202DP	YQE_12072	Dendroctonus ponderosae	Putative uncharacterized protein
EOG091202DR	OTAU005060	Onthophagus taurus	None
EOG091202DR	AGLA020239	Anoplophora glabripennis	None
EOG091202DR	APLA000907	Agrilus planipennis	None
EOG091202DR	TC034184	Tribolium castaneum	None
EOG091202DR	LDEC019109	Leptinotarsa decemlineata	None
EOG091202DR	YQE_12162	Dendroctonus ponderosae	None
EOG091202DS	OTAU011122	Onthophagus taurus	Putative uncharacterized protein
EOG091202DS	AGLA021292	Anoplophora glabripennis	Putative uncharacterized protein
EOG091202DS	APLA011489	Agrilus planipennis	Putative uncharacterized protein
EOG091202DS	TC000117	Tribolium castaneum	Putative uncharacterized protein
EOG091202DS	LDEC005032	Leptinotarsa decemlineata	Putative uncharacterized protein
EOG091202DS	YQE_07920	Dendroctonus ponderosae	Putative uncharacterized protein
EOG091202DU	OTAU001208	Onthophagus taurus	Putative uncharacterized protein
EOG091202DU	AGLA000083	Anoplophora glabripennis	Putative uncharacterized protein
EOG091202DU	APLA007697	Agrilus planipennis	Putative uncharacterized protein
EOG091202DU	TC002075	Tribolium castaneum	Putative uncharacterized protein
EOG091202DU	LDEC014175	Leptinotarsa decemlineata	Putative uncharacterized protein

EOG091202DU	YQE_08178	Dendroctonus ponderosae	Putative uncharacterized protein
EOG091202DZ	OTAU015311	Onthophagus taurus	Serine/threonine-protein phosphatase
EOG091202DZ	AGLA003873	Anoplophora glabripennis	Serine/threonine-protein phosphatase
EOG091202DZ	APLA005888	Agrilus planipennis	Serine/threonine-protein phosphatase
EOG091202DZ	TC015321	Tribolium castaneum	Serine/threonine-protein phosphatase
EOG091202DZ	LDEC001822	Leptinotarsa decemlineata	Serine/threonine-protein phosphatase
EOG091202DZ	YQE_08326	Dendroctonus ponderosae	Serine/threonine-protein phosphatase
EOG091202E0	OTAU016218	Onthophagus taurus	Putative uncharacterized protein
EOG091202E0	AGLA000926	Anoplophora glabripennis	Putative uncharacterized protein
EOG091202E0	APLA010775	Agrilus planipennis	Putative uncharacterized protein
EOG091202E0	TC004512	Tribolium castaneum	Putative uncharacterized protein
EOG091202E0	LDEC024200	Leptinotarsa decemlineata	Putative uncharacterized protein
EOG091202E0	YQE_11203	Dendroctonus ponderosae	Putative uncharacterized protein
EOG091202E2	OTAU004115	Onthophagus taurus	Putative uncharacterized protein
EOG091202E2	AGLA007232	Anoplophora glabripennis	Putative uncharacterized protein
EOG091202E2	APLA001544	Agrilus planipennis	Putative uncharacterized protein
EOG091202E2	TC009788	Tribolium castaneum	Putative uncharacterized protein
EOG091202E2	LDEC012225	Leptinotarsa decemlineata	Putative uncharacterized protein
EOG091202E2	YQE_08785	Dendroctonus ponderosae	Putative uncharacterized protein
EOG091202E6	OTAU001754	Onthophagus taurus	Putative uncharacterized protein
EOG091202E6	AGLA007256	Anoplophora glabripennis	Putative uncharacterized protein
EOG091202E6	APLA005824	Agrilus planipennis	Putative uncharacterized protein
EOG091202E6	TC006261	Tribolium castaneum	Putative uncharacterized protein
EOG091202E6	LDEC002460	Leptinotarsa decemlineata	Putative uncharacterized protein
EOG091202E6	YQE_12066	Dendroctonus ponderosae	Putative uncharacterized protein
EOG091202E7	OTAU007618	Onthophagus taurus	Scabrous
EOG091202E7	AGLA008920	Anoplophora glabripennis	Scabrous
EOG091202E7	APLA014558	Agrilus planipennis	Scabrous
EOG091202E7	TC003194	Tribolium castaneum	Scabrous
EOG091202E7	LDEC014150	Leptinotarsa decemlineata	Scabrous
EOG091202E7	YQE_09837	Dendroctonus ponderosae	Scabrous
EOG091202EB	OTAU010378	Onthophagus taurus	Putative uncharacterized protein
EOG091202EB	AGLA009937	Anoplophora glabripennis	Putative uncharacterized protein
EOG091202EB	APLA000599	Agrilus planipennis	Putative uncharacterized protein
EOG091202EB	TC015628	Tribolium castaneum	Putative uncharacterized protein
EOG091202EB	LDEC022327	Leptinotarsa decemlineata	Putative uncharacterized protein
EOG091202EB	YQE_03439	Dendroctonus ponderosae	Putative uncharacterized protein
EOG091202EE	OTAU003166	Onthophagus taurus	Putative uncharacterized protein
EOG091202EE	AGLA007586	Anoplophora glabripennis	Putative uncharacterized protein
EOG091202EE	APLA012889	Agrilus planipennis	Putative uncharacterized protein
EOG091202EE	TC014601	Tribolium castaneum	Putative uncharacterized protein
EOG091202EE	LDEC010751	Leptinotarsa decemlineata	Putative uncharacterized protein
EOG091202EE	YQE_04853	Dendroctonus ponderosae	Putative uncharacterized protein
EOG091202EI	OTAU006374	Onthophagus taurus	Putative uncharacterized protein
EOG091202EI	AGLA020905	Anoplophora glabripennis	Putative uncharacterized protein
EOG091202EI	APLA011299	Agrilus planipennis	Putative uncharacterized protein
EOG091202EI	TC008821	Tribolium castaneum	Putative uncharacterized protein
EOG091202EI	LDEC016850	Leptinotarsa decemlineata	Putative uncharacterized protein
EOG091202EI	YQE_11298	Dendroctonus ponderosae	Putative uncharacterized protein
EOG091202EJ	OTAU008746	Onthophagus taurus	Pleiohomeotic
EOG091202EJ	AGLA009938	Anoplophora glabripennis	Pleiohomeotic
EOG091202EJ	APLA000683	Agrilus planipennis	Pleiohomeotic
EOG091202EJ	TC015577	Tribolium castaneum	Pleiohomeotic
EOG091202EJ	LDEC020445	Leptinotarsa decemlineata	Pleiohomeotic
EOG091202EJ	YQE_09404	Dendroctonus ponderosae	Pleiohomeotic
EOG091202EK	OTAU013911	Onthophagus taurus	Putative uncharacterized protein
EOG091202EK	AGLA002020	Anoplophora glabripennis	Putative uncharacterized protein
EOG091202EK	APLA002829	Agrilus planipennis	Putative uncharacterized protein
EOG091202EK	TC006850	Tribolium castaneum	Putative uncharacterized protein
EOG091202EK	LDEC008564	Leptinotarsa decemlineata	Putative uncharacterized protein
EOG091202EK	YQE_10412	Dendroctonus ponderosae	Putative uncharacterized protein
EOG091202EM	OTAU012575	Onthophagus taurus	Putative uncharacterized protein
EOG091202EM	AGLA000983	Anoplophora glabripennis	Putative uncharacterized protein
EOG091202EM	APLA014267	Agrilus planipennis	Putative uncharacterized protein
EOG091202EM	TC000244	Tribolium castaneum	Putative uncharacterized protein
EOG091202EM	LDEC008138	Leptinotarsa decemlineata	Putative uncharacterized protein
EOG091202EM	YQE_03361	Dendroctonus ponderosae	Putative uncharacterized protein

EOG091202EN	OTAU000245	Onthophagus taurus	Putative uncharacterized protein
EOG091202EN	AGLA011215	Anoplophora glabripennis	Putative uncharacterized protein
EOG091202EN	APLA002868	Agrilus planipennis	Putative uncharacterized protein
EOG091202EN	TC013982	Tribolium castaneum	Putative uncharacterized protein
EOG091202EN	LDEC017708	Leptinotarsa decemlineata	Putative uncharacterized protein
EOG091202EN	YQE_02066	Dendroctonus ponderosae	Putative uncharacterized protein
EOG091202ET	OTAU000796	Onthophagus taurus	Putative uncharacterized protein
EOG091202ET	AGLA001975	Anoplophora glabripennis	Putative uncharacterized protein
EOG091202ET	APLA012987	Agrilus planipennis	Putative uncharacterized protein
EOG091202ET	TC011784	Tribolium castaneum	Putative uncharacterized protein
EOG091202ET	LDEC008543	Leptinotarsa decemlineata	Putative uncharacterized protein
EOG091202ET	YQE_07984	Dendroctonus ponderosae	Putative uncharacterized protein
EOG091202EW	OTAU002846	Onthophagus taurus	Putative uncharacterized protein
EOG091202EW	AGLA002167	Anoplophora glabripennis	Putative uncharacterized protein
EOG091202EW	APLA000905	Agrilus planipennis	Putative uncharacterized protein
EOG091202EW	TC014166	Tribolium castaneum	Putative uncharacterized protein
EOG091202EW	LDEC004198	Leptinotarsa decemlineata	Putative uncharacterized protein
EOG091202EW	YQE_03286	Dendroctonus ponderosae	Putative uncharacterized protein
EOG091202EX	OTAU003383	Onthophagus taurus	Putative uncharacterized protein
EOG091202EX	AGLA006600	Anoplophora glabripennis	Putative uncharacterized protein
EOG091202EX	APLA013341	Agrilus planipennis	Putative uncharacterized protein
EOG091202EX	TC012055	Tribolium castaneum	Putative uncharacterized protein
EOG091202EX	LDEC007664	Leptinotarsa decemlineata	Putative uncharacterized protein
EOG091202EX	YQE_07844	Dendroctonus ponderosae	Putative uncharacterized protein
EOG091202F1	OTAU005179	Onthophagus taurus	Putative uncharacterized protein
EOG091202F1	AGLA020746	Anoplophora glabripennis	Putative uncharacterized protein
EOG091202F1	APLA012202	Agrilus planipennis	Putative uncharacterized protein
EOG091202F1	TC003553	Tribolium castaneum	Putative uncharacterized protein
EOG091202F1	LDEC016408	Leptinotarsa decemlineata	Putative uncharacterized protein
EOG091202F1	YQE_07448	Dendroctonus ponderosae	Putative uncharacterized protein
EOG091202F2	OTAU009130	Onthophagus taurus	Putative uncharacterized protein
EOG091202F2	AGLA010802	Anoplophora glabripennis	Putative uncharacterized protein
EOG091202F2	APLA008503	Agrilus planipennis	Putative uncharacterized protein
EOG091202F2	TC013702	Tribolium castaneum	Putative uncharacterized protein
EOG091202F2	LDEC016364	Leptinotarsa decemlineata	Putative uncharacterized protein
EOG091202F2	YQE_04823	Dendroctonus ponderosae	Putative uncharacterized protein
EOG091202F5	OTAU007816	Onthophagus taurus	Putative uncharacterized protein
EOG091202F5	AGLA002896	Anoplophora glabripennis	Putative uncharacterized protein
EOG091202F5	APLA009554	Agrilus planipennis	Putative uncharacterized protein
EOG091202F5	TC008009	Tribolium castaneum	Putative uncharacterized protein
EOG091202F5	LDEC000745	Leptinotarsa decemlineata	Putative uncharacterized protein
EOG091202F5	YQE_05456	Dendroctonus ponderosae	Putative uncharacterized protein
EOG091202FC	OTAU003184	Onthophagus taurus	Putative uncharacterized protein
EOG091202FC	AGLA007363	Anoplophora glabripennis	Putative uncharacterized protein
EOG091202FC	APLA002854	Agrilus planipennis	Putative uncharacterized protein
EOG091202FC	TC014309	Tribolium castaneum	Putative uncharacterized protein
EOG091202FC	LDEC007610	Leptinotarsa decemlineata	Putative uncharacterized protein
EOG091202FC	YQE_07167	Dendroctonus ponderosae	Putative uncharacterized protein
EOG091202FJ	OTAU002749	Onthophagus taurus	Putative uncharacterized protein
EOG091202FJ	AGLA000236	Anoplophora glabripennis	Putative uncharacterized protein
EOG091202FJ	APLA010008	Agrilus planipennis	Putative uncharacterized protein
EOG091202FJ	TC001594	Tribolium castaneum	Putative uncharacterized protein
EOG091202FJ	LDEC001045	Leptinotarsa decemlineata	Putative uncharacterized protein
EOG091202FJ	YQE_09191	Dendroctonus ponderosae	Putative uncharacterized protein
EOG091202FK	OTAU005531	Onthophagus taurus	Putative uncharacterized protein
EOG091202FK	AGLA021737	Anoplophora glabripennis	Putative uncharacterized protein
EOG091202FK	APLA013968	Agrilus planipennis	Putative uncharacterized protein
EOG091202FK	TC011620	Tribolium castaneum	Putative uncharacterized protein
EOG091202FK	LDEC007244	Leptinotarsa decemlineata	Putative uncharacterized protein
EOG091202FK	YQE_10005	Dendroctonus ponderosae	Putative uncharacterized protein
EOG091202FM	OTAU006386	Onthophagus taurus	Putative uncharacterized protein
EOG091202FM	AGLA004485	Anoplophora glabripennis	Putative uncharacterized protein
EOG091202FM	APLA002276	Agrilus planipennis	Putative uncharacterized protein
EOG091202FM	TC007515	Tribolium castaneum	Putative uncharacterized protein
EOG091202FM	LDEC006353	Leptinotarsa decemlineata	Putative uncharacterized protein
EOG091202FM	YQE_02972	Dendroctonus ponderosae	Putative uncharacterized protein
EOG091202FN	OTAU004118	Onthophagus taurus	Putative uncharacterized protein

EOG091202FN	AGLA009181	Anoplophora glabripennis	Putative uncharacterized protein
EOG091202FN	APLA001540	Agrilus planipennis	Putative uncharacterized protein
EOG091202FN	TC009994	Tribolium castaneum	Putative uncharacterized protein
EOG091202FN	LDEC015715	Leptinotarsa decemlineata	Putative uncharacterized protein
EOG091202FN	YQE_05997	Dendroctonus ponderosae	Putative uncharacterized protein
EOG091202FP	OTAU003610	Onthophagus taurus	Putative uncharacterized protein
EOG091202FP	AGLA015164	Anoplophora glabripennis	Putative uncharacterized protein
EOG091202FP	APLA003736	Agrilus planipennis	Putative uncharacterized protein
EOG091202FP	TC002732	Tribolium castaneum	Putative uncharacterized protein
EOG091202FP	LDEC018218	Leptinotarsa decemlineata	Putative uncharacterized protein
EOG091202FP	YQE_07907	Dendroctonus ponderosae	Putative uncharacterized protein
EOG091202FS	OTAU009304	Onthophagus taurus	Putative uncharacterized protein
EOG091202FS	AGLA013125	Anoplophora glabripennis	Putative uncharacterized protein
EOG091202FS	APLA009805	Agrilus planipennis	Putative uncharacterized protein
EOG091202FS	TC010059	Tribolium castaneum	Putative uncharacterized protein
EOG091202FS	LDEC010677	Leptinotarsa decemlineata	Putative uncharacterized protein
EOG091202FS	YQE_06030	Dendroctonus ponderosae	Putative uncharacterized protein
EOG091202FV	OTAU003052	Onthophagus taurus	Putative uncharacterized protein
EOG091202FV	AGLA009283	Anoplophora glabripennis	Putative uncharacterized protein
EOG091202FV	APLA004787	Agrilus planipennis	Putative uncharacterized protein
EOG091202FV	TC005603	Tribolium castaneum	Putative uncharacterized protein
EOG091202FV	LDEC011709	Leptinotarsa decemlineata	Putative uncharacterized protein
EOG091202FV	YQE_05540	Dendroctonus ponderosae	Putative uncharacterized protein
EOG091202FW	OTAU000029	Onthophagus taurus	Putative uncharacterized protein
EOG091202FW	AGLA011939	Anoplophora glabripennis	Putative uncharacterized protein
EOG091202FW	APLA006229	Agrilus planipennis	Putative uncharacterized protein
EOG091202FW	TC013432	Tribolium castaneum	Putative uncharacterized protein
EOG091202FW	LDEC021755	Leptinotarsa decemlineata	Putative uncharacterized protein
EOG091202FW	YQE_03993	Dendroctonus ponderosae	Putative uncharacterized protein
EOG091202FY	OTAU007452	Onthophagus taurus	Putative uncharacterized protein
EOG091202FY	AGLA006676	Anoplophora glabripennis	Putative uncharacterized protein
EOG091202FY	APLA004338	Agrilus planipennis	Putative uncharacterized protein
EOG091202FY	TC000949	Tribolium castaneum	Putative uncharacterized protein
EOG091202FY	LDEC001616	Leptinotarsa decemlineata	Putative uncharacterized protein
EOG091202FY	YQE_07690	Dendroctonus ponderosae	Putative uncharacterized protein
EOG091202FZ	OTAU003586	Onthophagus taurus	Putative uncharacterized protein
EOG091202FZ	AGLA002308	Anoplophora glabripennis	Putative uncharacterized protein
EOG091202FZ	APLA003983	Agrilus planipennis	Putative uncharacterized protein
EOG091202FZ	TC012528	Tribolium castaneum	Putative uncharacterized protein
EOG091202FZ	LDEC004159	Leptinotarsa decemlineata	Putative uncharacterized protein
EOG091202FZ	YQE_01987	Dendroctonus ponderosae	Putative uncharacterized protein
EOG091202G0	OTAU007908	Onthophagus taurus	Putative uncharacterized protein
EOG091202G0	AGLA018016	Anoplophora glabripennis	Putative uncharacterized protein
EOG091202G0	APLA011982	Agrilus planipennis	Putative uncharacterized protein
EOG091202G0	TC030743	Tribolium castaneum	Putative uncharacterized protein
EOG091202G0	LDEC005770	Leptinotarsa decemlineata	Putative uncharacterized protein
EOG091202G0	YQE_08478	Dendroctonus ponderosae	Putative uncharacterized protein
EOG091202G1	OTAU010227	Onthophagus taurus	Putative uncharacterized protein
EOG091202G1	AGLA008300	Anoplophora glabripennis	Putative uncharacterized protein
EOG091202G1	APLA012566	Agrilus planipennis	Putative uncharacterized protein
EOG091202G1	TC009349	Tribolium castaneum	Putative uncharacterized protein
EOG091202G1	LDEC001255	Leptinotarsa decemlineata	Putative uncharacterized protein
EOG091202G1	YQE_13054	Dendroctonus ponderosae	Putative uncharacterized protein
EOG091202G2	OTAU000811	Onthophagus taurus	Putative uncharacterized protein
EOG091202G2	AGLA013449	Anoplophora glabripennis	Putative uncharacterized protein
EOG091202G2	APLA014917	Agrilus planipennis	Putative uncharacterized protein
EOG091202G2	TC011050	Tribolium castaneum	Putative uncharacterized protein
EOG091202G2	LDEC011565	Leptinotarsa decemlineata	Putative uncharacterized protein
EOG091202G2	YQE_04438	Dendroctonus ponderosae	Putative uncharacterized protein
EOG091202G4	OTAU009864	Onthophagus taurus	Putative uncharacterized protein
EOG091202G4	AGLA007005	Anoplophora glabripennis	Putative uncharacterized protein
EOG091202G4	APLA015051	Agrilus planipennis	Putative uncharacterized protein
EOG091202G4	TC004865	Tribolium castaneum	Putative uncharacterized protein
EOG091202G4	LDEC019182	Leptinotarsa decemlineata	Putative uncharacterized protein
EOG091202G4	YQE_02373	Dendroctonus ponderosae	Putative uncharacterized protein
EOG091202G7	OTAU005450	Onthophagus taurus	Putative uncharacterized protein
EOG091202G7	AGLA004338	Anoplophora glabripennis	Putative uncharacterized protein

EOG091202G7	APLA002480	Agrilus planipennis	Putative uncharacterized protein
EOG091202G7	TC014343	Tribolium castaneum	Putative uncharacterized protein
EOG091202G7	LDEC014573	Leptinotarsa decemlineata	Putative uncharacterized protein
EOG091202G7	YQE_04043	Dendroctonus ponderosae	Putative uncharacterized protein
EOG091202G8	OTAU001994	Onthophagus taurus	Putative uncharacterized protein
EOG091202G8	AGLA007162	Anoplophora glabripennis	Putative uncharacterized protein
EOG091202G8	APLA008185	Agrilus planipennis	Putative uncharacterized protein
EOG091202G8	TC009034	Tribolium castaneum	Putative uncharacterized protein
EOG091202G8	LDEC020002	Leptinotarsa decemlineata	Putative uncharacterized protein
EOG091202G8	YQE_05371	Dendroctonus ponderosae	Putative uncharacterized protein
EOG091202G9	OTAU004585	Onthophagus taurus	Putative uncharacterized protein
EOG091202G9	AGLA014411	Anoplophora glabripennis	Putative uncharacterized protein
EOG091202G9	APLA013382	Agrilus planipennis	Putative uncharacterized protein
EOG091202G9	TC007191	Tribolium castaneum	Putative uncharacterized protein
EOG091202G9	LDEC008308	Leptinotarsa decemlineata	Putative uncharacterized protein
EOG091202G9	YQE_02301	Dendroctonus ponderosae	Putative uncharacterized protein
EOG091202GA	OTAU012177	Onthophagus taurus	Putative uncharacterized protein
EOG091202GA	AGLA009532	Anoplophora glabripennis	Putative uncharacterized protein
EOG091202GA	APLA007186	Agrilus planipennis	Putative uncharacterized protein
EOG091202GA	TC007735	Tribolium castaneum	Putative uncharacterized protein
EOG091202GA	LDEC018730	Leptinotarsa decemlineata	Putative uncharacterized protein
EOG091202GA	YQE_07109	Dendroctonus ponderosae	Putative uncharacterized protein
EOG091202GB	OTAU007054	Onthophagus taurus	Putative uncharacterized protein
EOG091202GB	AGLA000618	Anoplophora glabripennis	Putative uncharacterized protein
EOG091202GB	APLA014195	Agrilus planipennis	Putative uncharacterized protein
EOG091202GB	TC001555	Tribolium castaneum	Putative uncharacterized protein
EOG091202GB	LDEC005487	Leptinotarsa decemlineata	Putative uncharacterized protein
EOG091202GB	YQE_06423	Dendroctonus ponderosae	Putative uncharacterized protein
EOG091202GD	OTAU011917	Onthophagus taurus	ligase activity
EOG091202GD	AGLA015013	Anoplophora glabripennis	ligase activity
EOG091202GD	APLA004281	Agrilus planipennis	ligase activity
EOG091202GD	TC031264	Tribolium castaneum	ligase activity
EOG091202GD	LDEC015054	Leptinotarsa decemlineata	ligase activity
EOG091202GD	YQE_06562	Dendroctonus ponderosae	ligase activity
EOG091202GF	OTAU005000	Onthophagus taurus	Putative uncharacterized protein
EOG091202GF	AGLA010153	Anoplophora glabripennis	Putative uncharacterized protein
EOG091202GF	APLA006338	Agrilus planipennis	Putative uncharacterized protein
EOG091202GF	TC011092	Tribolium castaneum	Putative uncharacterized protein
EOG091202GF	LDEC019000	Leptinotarsa decemlineata	Putative uncharacterized protein
EOG091202GF	YQE_08941	Dendroctonus ponderosae	Putative uncharacterized protein
EOG091202GH	OTAU001243	Onthophagus taurus	Seven up
EOG091202GH	AGLA004542	Anoplophora glabripennis	Seven up
EOG091202GH	APLA003558	Agrilus planipennis	Seven up
EOG091202GH	TC001722	Tribolium castaneum	Seven up
EOG091202GH	LDEC012182	Leptinotarsa decemlineata	Seven up
EOG091202GH	YQE_06714	Dendroctonus ponderosae	Seven up
EOG091202GJ	OTAU003996	Onthophagus taurus	Netrin
EOG091202GJ	AGLA011992	Anoplophora glabripennis	Netrin
EOG091202GJ	APLA000269	Agrilus planipennis	Netrin
EOG091202GJ	TC002285	Tribolium castaneum	Netrin
EOG091202GJ	LDEC012930	Leptinotarsa decemlineata	Netrin
EOG091202GJ	YQE_11707	Dendroctonus ponderosae	Netrin
EOG091202GK	OTAU006640	Onthophagus taurus	Ribose-phosphate pyrophosphokinase
EOG091202GK	AGLA009129	Anoplophora glabripennis	Ribose-phosphate pyrophosphokinase
EOG091202GK	APLA013710	Agrilus planipennis	Ribose-phosphate pyrophosphokinase
EOG091202GK	TC002990	Tribolium castaneum	Ribose-phosphate pyrophosphokinase
EOG091202GK	LDEC005491	Leptinotarsa decemlineata	Ribose-phosphate pyrophosphokinase
EOG091202GK	YQE_09829	Dendroctonus ponderosae	Ribose-phosphate pyrophosphokinase
EOG091202GL	OTAU013071	Onthophagus taurus	Putative uncharacterized protein
EOG091202GL	AGLA003886	Anoplophora glabripennis	Putative uncharacterized protein
EOG091202GL	APLA000529	Agrilus planipennis	Putative uncharacterized protein
EOG091202GL	TC015492	Tribolium castaneum	Putative uncharacterized protein
EOG091202GL	LDEC008810	Leptinotarsa decemlineata	Putative uncharacterized protein
EOG091202GL	YQE_02136	Dendroctonus ponderosae	Putative uncharacterized protein
EOG091202GM	OTAU006365	Onthophagus taurus	Putative uncharacterized protein
EOG091202GM	AGLA009805	Anoplophora glabripennis	Putative uncharacterized protein
EOG091202GM	APLA010102	Agrilus planipennis	Putative uncharacterized protein

EOG091202GM	TC013256	Tribolium castaneum	Putative uncharacterized protein
EOG091202GM	LDEC013874	Leptinotarsa decemlineata	Putative uncharacterized protein
EOG091202GM	YQE_11293	Dendroctonus ponderosae	Putative uncharacterized protein
EOG091202GO	OTAU003225	Onthophagus taurus	Putative uncharacterized protein
EOG091202GO	AGLA003453	Anoplophora glabripennis	Putative uncharacterized protein
EOG091202GO	APLA001671	Agrilus planipennis	Putative uncharacterized protein
EOG091202GO	TC009001	Tribolium castaneum	Putative uncharacterized protein
EOG091202GO	LDEC013020	Leptinotarsa decemlineata	Putative uncharacterized protein
EOG091202GO	YQE_08821	Dendroctonus ponderosae	Putative uncharacterized protein
EOG091202GU	OTAU008566	Onthophagus taurus	Putative uncharacterized protein
EOG091202GU	AGLA011206	Anoplophora glabripennis	Putative uncharacterized protein
EOG091202GU	APLA011101	Agrilus planipennis	Putative uncharacterized protein
EOG091202GU	TC030609	Tribolium castaneum	Putative uncharacterized protein
EOG091202GU	LDEC007693	Leptinotarsa decemlineata	Putative uncharacterized protein
EOG091202GU	YQE_12587	Dendroctonus ponderosae	Putative uncharacterized protein
EOG091202GV	OTAU005592	Onthophagus taurus	oxidoreductase activity
EOG091202GV	AGLA003419	Anoplophora glabripennis	oxidoreductase activity
EOG091202GV	APLA003023	Agrilus planipennis	oxidoreductase activity
EOG091202GV	TC033558	Tribolium castaneum	oxidoreductase activity
EOG091202GV	LDEC013028	Leptinotarsa decemlineata	oxidoreductase activity
EOG091202GV	YQE_06852	Dendroctonus ponderosae	oxidoreductase activity
EOG091202GX	OTAU002955	Onthophagus taurus	Putative uncharacterized protein
EOG091202GX	AGLA003847	Anoplophora glabripennis	Putative uncharacterized protein
EOG091202GX	APLA003477	Agrilus planipennis	Putative uncharacterized protein
EOG091202GX	TC008817	Tribolium castaneum	Putative uncharacterized protein
EOG091202GX	LDEC020745	Leptinotarsa decemlineata	Putative uncharacterized protein
EOG091202GX	YQE_05837	Dendroctonus ponderosae	Putative uncharacterized protein
EOG091202GY	OTAU009002	Onthophagus taurus	Putative uncharacterized protein
EOG091202GY	AGLA001284	Anoplophora glabripennis	Putative uncharacterized protein
EOG091202GY	APLA007741	Agrilus planipennis	Putative uncharacterized protein
EOG091202GY	TC015880	Tribolium castaneum	Putative uncharacterized protein
EOG091202GY	LDEC016042	Leptinotarsa decemlineata	Putative uncharacterized protein
EOG091202GY	YQE_03082	Dendroctonus ponderosae	Putative uncharacterized protein
EOG091202GZ	OTAU005351	Onthophagus taurus	Putative uncharacterized protein
EOG091202GZ	AGLA008297	Anoplophora glabripennis	Putative uncharacterized protein
EOG091202GZ	APLA012569	Agrilus planipennis	Putative uncharacterized protein
EOG091202GZ	TC009468	Tribolium castaneum	Putative uncharacterized protein
EOG091202GZ	LDEC001258	Leptinotarsa decemlineata	Putative uncharacterized protein
EOG091202GZ	YQE_11307	Dendroctonus ponderosae	Putative uncharacterized protein
EOG091202H0	OTAU015860	Onthophagus taurus	Putative uncharacterized protein
EOG091202H0	AGLA021001	Anoplophora glabripennis	Putative uncharacterized protein
EOG091202H0	APLA007222	Agrilus planipennis	Putative uncharacterized protein
EOG091202H0	TC003977	Tribolium castaneum	Putative uncharacterized protein
EOG091202H0	LDEC008585	Leptinotarsa decemlineata	Putative uncharacterized protein
EOG091202H0	YQE_02148	Dendroctonus ponderosae	Putative uncharacterized protein
EOG091202H1	OTAU012479	Onthophagus taurus	Putative uncharacterized protein
EOG091202H1	AGLA005326	Anoplophora glabripennis	Putative uncharacterized protein
EOG091202H1	APLA007491	Agrilus planipennis	Putative uncharacterized protein
EOG091202H1	TC006466	Tribolium castaneum	Putative uncharacterized protein
EOG091202H1	LDEC005887	Leptinotarsa decemlineata	Putative uncharacterized protein
EOG091202H1	YQE_12710	Dendroctonus ponderosae	Putative uncharacterized protein
EOG091202H5	OTAU004791	Onthophagus taurus	Putative uncharacterized protein
EOG091202H5	AGLA011571	Anoplophora glabripennis	Putative uncharacterized protein
EOG091202H5	APLA006256	Agrilus planipennis	Putative uncharacterized protein
EOG091202H5	TC000838	Tribolium castaneum	Putative uncharacterized protein
EOG091202H5	LDEC007776	Leptinotarsa decemlineata	Putative uncharacterized protein
EOG091202H5	YQE_01761	Dendroctonus ponderosae	Putative uncharacterized protein
EOG091202HA	OTAU005403	Onthophagus taurus	Putative uncharacterized protein
EOG091202HA	AGLA014972	Anoplophora glabripennis	Putative uncharacterized protein
EOG091202HA	APLA014547	Agrilus planipennis	Putative uncharacterized protein
EOG091202HA	TC004584	Tribolium castaneum	Putative uncharacterized protein
EOG091202HA	LDEC020102	Leptinotarsa decemlineata	Putative uncharacterized protein
EOG091202HA	YQE_10266	Dendroctonus ponderosae	Putative uncharacterized protein
EOG091202HB	OTAU003598	Onthophagus taurus	Putative uncharacterized protein
EOG091202HB	AGLA000425	Anoplophora glabripennis	Putative uncharacterized protein
EOG091202HB	APLA012528	Agrilus planipennis	Putative uncharacterized protein
EOG091202HB	TC003203	Tribolium castaneum	Putative uncharacterized protein

EOG091202HB	LDEC009185	Leptinotarsa decemlineata	Putative uncharacterized protein
EOG091202HB	YQE_10210	Dendroctonus ponderosae	Putative uncharacterized protein
EOG091202HC	OTAU006922	Onthophagus taurus	Putative uncharacterized protein
EOG091202HC	AGLA003411	Anoplophora glabripennis	Putative uncharacterized protein
EOG091202HC	APLA010330	Agrilus planipennis	Putative uncharacterized protein
EOG091202HC	TC003821	Tribolium castaneum	Putative uncharacterized protein
EOG091202HC	LDEC015832	Leptinotarsa decemlineata	Putative uncharacterized protein
EOG091202HC	YQE_11150	Dendroctonus ponderosae	Putative uncharacterized protein
EOG091202HD	OTAU001824	Onthophagus taurus	Putative uncharacterized protein
EOG091202HD	AGLA004984	Anoplophora glabripennis	Putative uncharacterized protein
EOG091202HD	APLA001462	Agrilus planipennis	Putative uncharacterized protein
EOG091202HD	TC006627	Tribolium castaneum	Putative uncharacterized protein
EOG091202HD	LDEC003004	Leptinotarsa decemlineata	Putative uncharacterized protein
EOG091202HD	YQE_07015	Dendroctonus ponderosae	Putative uncharacterized protein
EOG091202HE	OTAU001344	Onthophagus taurus	Putative uncharacterized protein
EOG091202HE	AGLA000279	Anoplophora glabripennis	Putative uncharacterized protein
EOG091202HE	APLA003690	Agrilus planipennis	Putative uncharacterized protein
EOG091202HE	TC013565	Tribolium castaneum	Putative uncharacterized protein
EOG091202HE	LDEC011131	Leptinotarsa decemlineata	Putative uncharacterized protein
EOG091202HE	YQE_06633	Dendroctonus ponderosae	Putative uncharacterized protein
EOG091202HF	OTAU012079	Onthophagus taurus	Putative uncharacterized protein
EOG091202HF	AGLA017803	Anoplophora glabripennis	Putative uncharacterized protein
EOG091202HF	APLA008837	Agrilus planipennis	Putative uncharacterized protein
EOG091202HF	TC009000	Tribolium castaneum	Putative uncharacterized protein
EOG091202HF	LDEC013023	Leptinotarsa decemlineata	Putative uncharacterized protein
EOG091202HF	YQE_08819	Dendroctonus ponderosae	Putative uncharacterized protein
EOG091202HG	OTAU001807	Onthophagus taurus	Putative uncharacterized protein
EOG091202HG	AGLA004999	Anoplophora glabripennis	Putative uncharacterized protein
EOG091202HG	APLA005766	Agrilus planipennis	Putative uncharacterized protein
EOG091202HG	TC006709	Tribolium castaneum	Putative uncharacterized protein
EOG091202HG	LDEC018881	Leptinotarsa decemlineata	Putative uncharacterized protein
EOG091202HG	YQE_07502	Dendroctonus ponderosae	Putative uncharacterized protein
EOG091202HH	OTAU002416	Onthophagus taurus	neuropeptide Y receptor activity
EOG091202HH	AGLA008917	Anoplophora glabripennis	neuropeptide Y receptor activity
EOG091202HH	APLA015067	Agrilus planipennis	neuropeptide Y receptor activity
EOG091202HH	TC034574	Tribolium castaneum	neuropeptide Y receptor activity
EOG091202HH	LDEC020362	Leptinotarsa decemlineata	neuropeptide Y receptor activity
EOG091202HH	YQE_07443	Dendroctonus ponderosae	neuropeptide Y receptor activity
EOG091202HJ	OTAU003103	Onthophagus taurus	Putative uncharacterized protein
EOG091202HJ	AGLA004798	Anoplophora glabripennis	Putative uncharacterized protein
EOG091202HJ	APLA005216	Agrilus planipennis	Putative uncharacterized protein
EOG091202HJ	TC002812	Tribolium castaneum	Putative uncharacterized protein
EOG091202HJ	LDEC020231	Leptinotarsa decemlineata	Putative uncharacterized protein
EOG091202HJ	YQE_07638	Dendroctonus ponderosae	Putative uncharacterized protein
EOG091202HK	OTAU001445	Onthophagus taurus	Putative uncharacterized protein
EOG091202HK	AGLA011270	Anoplophora glabripennis	Putative uncharacterized protein
EOG091202HK	APLA005615	Agrilus planipennis	Putative uncharacterized protein
EOG091202HK	TC001975	Tribolium castaneum	Putative uncharacterized protein
EOG091202HK	LDEC016842	Leptinotarsa decemlineata	Putative uncharacterized protein
EOG091202HK	YQE_02482	Dendroctonus ponderosae	Putative uncharacterized protein
EOG091202HL	OTAU003285	Onthophagus taurus	Putative uncharacterized protein
EOG091202HL	AGLA008520	Anoplophora glabripennis	Putative uncharacterized protein
EOG091202HL	APLA004628	Agrilus planipennis	Putative uncharacterized protein
EOG091202HL	TC009322	Tribolium castaneum	Putative uncharacterized protein
EOG091202HL	LDEC017111	Leptinotarsa decemlineata	Putative uncharacterized protein
EOG091202HL	YQE_02344	Dendroctonus ponderosae	Putative uncharacterized protein
EOG091202HN	OTAU015398	Onthophagus taurus	RNA binding
EOG091202HN	AGLA011058	Anoplophora glabripennis	RNA binding
EOG091202HN	APLA009528	Agrilus planipennis	RNA binding
EOG091202HN	TC034449	Tribolium castaneum	RNA binding
EOG091202HN	LDEC011802	Leptinotarsa decemlineata	RNA binding
EOG091202HN	YQE_07937	Dendroctonus ponderosae	RNA binding
EOG091202HO	OTAU004145	Onthophagus taurus	None
EOG091202HO	AGLA017799	Anoplophora glabripennis	None
EOG091202HO	APLA001590	Agrilus planipennis	None
EOG091202HO	TC034790	Tribolium castaneum	None
EOG091202HO	LDEC013015	Leptinotarsa decemlineata	None

EOG091202HO	YQE_08807	Dendroctonus ponderosae	None
EOG091202HP	OTAU007369	Onthophagus taurus	Putative uncharacterized protein
EOG091202HP	AGLA001379	Anoplophora glabripennis	Putative uncharacterized protein
EOG091202HP	APLA007963	Agrilus planipennis	Putative uncharacterized protein
EOG091202HP	TC000219	Tribolium castaneum	Putative uncharacterized protein
EOG091202HP	LDEC007307	Leptinotarsa decemlineata	Putative uncharacterized protein
EOG091202HP	YQE_10694	Dendroctonus ponderosae	Putative uncharacterized protein
EOG091202HR	OTAU004735	Onthophagus taurus	Putative uncharacterized protein
EOG091202HR	AGLA017655	Anoplophora glabripennis	Putative uncharacterized protein
EOG091202HR	APLA008405	Agrilus planipennis	Putative uncharacterized protein
EOG091202HR	TC000026	Tribolium castaneum	Putative uncharacterized protein
EOG091202HR	LDEC010069	Leptinotarsa decemlineata	Putative uncharacterized protein
EOG091202HR	YQE_07791	Dendroctonus ponderosae	Putative uncharacterized protein
EOG091202HS	OTAU006921	Onthophagus taurus	Putative uncharacterized protein
EOG091202HS	AGLA009136	Anoplophora glabripennis	Putative uncharacterized protein
EOG091202HS	APLA014002	Agrilus planipennis	Putative uncharacterized protein
EOG091202HS	TC010569	Tribolium castaneum	Putative uncharacterized protein
EOG091202HS	LDEC014500	Leptinotarsa decemlineata	Putative uncharacterized protein
EOG091202HS	YQE_10259	Dendroctonus ponderosae	Putative uncharacterized protein
EOG091202HT	OTAU002607	Onthophagus taurus	O-acyltransferase
EOG091202HT	AGLA014687	Anoplophora glabripennis	O-acyltransferase
EOG091202HT	APLA002680	Agrilus planipennis	O-acyltransferase
EOG091202HT	TC014471	Tribolium castaneum	O-acyltransferase
EOG091202HT	LDEC001148	Leptinotarsa decemlineata	O-acyltransferase
EOG091202HT	YQE_03152	Dendroctonus ponderosae	O-acyltransferase
EOG091202HW	OTAU003139	Onthophagus taurus	Putative uncharacterized protein
EOG091202HW	AGLA017700	Anoplophora glabripennis	Putative uncharacterized protein
EOG091202HW	APLA013808	Agrilus planipennis	Putative uncharacterized protein
EOG091202HW	TC000059	Tribolium castaneum	Putative uncharacterized protein
EOG091202HW	LDEC005047	Leptinotarsa decemlineata	Putative uncharacterized protein
EOG091202HW	YQE_03678	Dendroctonus ponderosae	Putative uncharacterized protein
EOG091202HX	OTAU005086	Onthophagus taurus	Putative uncharacterized protein
EOG091202HX	AGLA007307	Anoplophora glabripennis	Putative uncharacterized protein
EOG091202HX	APLA000465	Agrilus planipennis	Putative uncharacterized protein
EOG091202HX	TC005736	Tribolium castaneum	Putative uncharacterized protein
EOG091202HX	LDEC021323	Leptinotarsa decemlineata	Putative uncharacterized protein
EOG091202HX	YQE_11997	Dendroctonus ponderosae	Putative uncharacterized protein
EOG091202HY	OTAU006543	Onthophagus taurus	Putative uncharacterized protein
EOG091202HY	AGLA006642	Anoplophora glabripennis	Putative uncharacterized protein
EOG091202HY	APLA009147	Agrilus planipennis	Putative uncharacterized protein
EOG091202HY	TC002426	Tribolium castaneum	Putative uncharacterized protein
EOG091202HY	LDEC004972	Leptinotarsa decemlineata	Putative uncharacterized protein
EOG091202HY	YQE_03643	Dendroctonus ponderosae	Putative uncharacterized protein
EOG091202HZ	OTAU008474	Onthophagus taurus	acetylgalactosaminyltransferase activity
EOG091202HZ	AGLA000620	Anoplophora glabripennis	acetylgalactosaminyltransferase activity
EOG091202HZ	APLA001943	Agrilus planipennis	acetylgalactosaminyltransferase activity
EOG091202HZ	TC031391	Tribolium castaneum	acetylgalactosaminyltransferase activity
EOG091202HZ	LDEC012670	Leptinotarsa decemlineata	acetylgalactosaminyltransferase activity
EOG091202HZ	YQE_08126	Dendroctonus ponderosae	acetylgalactosaminyltransferase activity
EOG091202I2	OTAU006831	Onthophagus taurus	None
EOG091202I2	AGLA004216	Anoplophora glabripennis	None
EOG091202I2	APLA004837	Agrilus planipennis	None
EOG091202I2	TC033256	Tribolium castaneum	None
EOG091202I2	LDEC010196	Leptinotarsa decemlineata	None
EOG091202I2	YQE_07175	Dendroctonus ponderosae	None
EOG091202I3	OTAU006613	Onthophagus taurus	Putative uncharacterized protein
EOG091202I3	AGLA021707	Anoplophora glabripennis	Putative uncharacterized protein
EOG091202I3	APLA009816	Agrilus planipennis	Putative uncharacterized protein
EOG091202I3	TC008962	Tribolium castaneum	Putative uncharacterized protein
EOG091202I3	LDEC015382	Leptinotarsa decemlineata	Putative uncharacterized protein
EOG091202I3	YQE_11560	Dendroctonus ponderosae	Putative uncharacterized protein
EOG091202I4	OTAU000466	Onthophagus taurus	Putative uncharacterized protein
EOG091202I4	AGLA006083	Anoplophora glabripennis	Putative uncharacterized protein
EOG091202I4	APLA013846	Agrilus planipennis	Putative uncharacterized protein
EOG091202I4	TC012600	Tribolium castaneum	Putative uncharacterized protein
EOG091202I4	LDEC010212	Leptinotarsa decemlineata	Putative uncharacterized protein
EOG091202I4	YQE_12979	Dendroctonus ponderosae	Putative uncharacterized protein

EOG091202I6	OTAU006346	Onthophagus taurus	Putative uncharacterized protein
EOG091202I6	AGLA009624	Anoplophora glabripennis	Putative uncharacterized protein
EOG091202I6	APLA011280	Agrilus planipennis	Putative uncharacterized protein
EOG091202I6	TC000109	Tribolium castaneum	Putative uncharacterized protein
EOG091202I6	LDEC019905	Leptinotarsa decemlineata	Putative uncharacterized protein
EOG091202I6	YQE_12657	Dendroctonus ponderosae	Putative uncharacterized protein
EOG091202I8	OTAU017255	Onthophagus taurus	Aspartate aminotransferase
EOG091202I8	AGLA006417	Anoplophora glabripennis	Aspartate aminotransferase
EOG091202I8	APLA012983	Agrilus planipennis	Aspartate aminotransferase
EOG091202I8	TC014364	Tribolium castaneum	Aspartate aminotransferase
EOG091202I8	LDEC006675	Leptinotarsa decemlineata	Aspartate aminotransferase
EOG091202I8	YQE_04016	Dendroctonus ponderosae	Aspartate aminotransferase
EOG091202I9	OTAU011892	Onthophagus taurus	Putative uncharacterized protein
EOG091202I9	AGLA012835	Anoplophora glabripennis	Putative uncharacterized protein
EOG091202I9	APLA002695	Agrilus planipennis	Putative uncharacterized protein
EOG091202I9	TC014815	Tribolium castaneum	Putative uncharacterized protein
EOG091202I9	LDEC005945	Leptinotarsa decemlineata	Putative uncharacterized protein
EOG091202I9	YQE_06221	Dendroctonus ponderosae	Putative uncharacterized protein
EOG091202IA	OTAU007330	Onthophagus taurus	Putative uncharacterized protein
EOG091202IA	AGLA007052	Anoplophora glabripennis	Putative uncharacterized protein
EOG091202IA	APLA011922	Agrilus planipennis	Putative uncharacterized protein
EOG091202IA	TC000780	Tribolium castaneum	Putative uncharacterized protein
EOG091202IA	LDEC000817	Leptinotarsa decemlineata	Putative uncharacterized protein
EOG091202IA	YQE_08935	Dendroctonus ponderosae	Putative uncharacterized protein
EOG091202IB	OTAU014399	Onthophagus taurus	Putative uncharacterized protein
EOG091202IB	AGLA006307	Anoplophora glabripennis	Putative uncharacterized protein
EOG091202IB	APLA006018	Agrilus planipennis	Putative uncharacterized protein
EOG091202IB	TC009266	Tribolium castaneum	Putative uncharacterized protein
EOG091202IB	LDEC002099	Leptinotarsa decemlineata	Putative uncharacterized protein
EOG091202IB	YQE_08678	Dendroctonus ponderosae	Putative uncharacterized protein
EOG091202IC	OTAU016107	Onthophagus taurus	Putative uncharacterized protein
EOG091202IC	AGLA008682	Anoplophora glabripennis	Putative uncharacterized protein
EOG091202IC	APLA002360	Agrilus planipennis	Putative uncharacterized protein
EOG091202IC	TC010678	Tribolium castaneum	Putative uncharacterized protein
EOG091202IC	LDEC022183	Leptinotarsa decemlineata	Putative uncharacterized protein
EOG091202IC	YQE_04325	Dendroctonus ponderosae	Putative uncharacterized protein
EOG091202ID	OTAU005875	Onthophagus taurus	DN cadherin-like protein
EOG091202ID	AGLA001778	Anoplophora glabripennis	DN cadherin-like protein
EOG091202ID	APLA003582	Agrilus planipennis	DN cadherin-like protein
EOG091202ID	TC013220	Tribolium castaneum	DN cadherin-like protein
EOG091202ID	LDEC008928	Leptinotarsa decemlineata	DN cadherin-like protein
EOG091202ID	YQE_03878	Dendroctonus ponderosae	DN cadherin-like protein
EOG091202IE	OTAU001509	Onthophagus taurus	Putative uncharacterized protein
EOG091202IE	AGLA011740	Anoplophora glabripennis	Putative uncharacterized protein
EOG091202IE	APLA004121	Agrilus planipennis	Putative uncharacterized protein
EOG091202IE	TC008927	Tribolium castaneum	Putative uncharacterized protein
EOG091202IE	LDEC016586	Leptinotarsa decemlineata	Putative uncharacterized protein
EOG091202IE	YQE_03175	Dendroctonus ponderosae	Putative uncharacterized protein
EOG091202IF	OTAU003418	Onthophagus taurus	Putative uncharacterized protein
EOG091202IF	AGLA007733	Anoplophora glabripennis	Putative uncharacterized protein
EOG091202IF	APLA013547	Agrilus planipennis	Putative uncharacterized protein
EOG091202IF	TC002993	Tribolium castaneum	Putative uncharacterized protein
EOG091202IF	LDEC010227	Leptinotarsa decemlineata	Putative uncharacterized protein
EOG091202IF	YQE_09800	Dendroctonus ponderosae	Putative uncharacterized protein
EOG091202IG	OTAU012513	Onthophagus taurus	Putative uncharacterized protein
EOG091202IG	AGLA014080	Anoplophora glabripennis	Putative uncharacterized protein
EOG091202IG	APLA012194	Agrilus planipennis	Putative uncharacterized protein
EOG091202IG	TC000458	Tribolium castaneum	Putative uncharacterized protein
EOG091202IG	LDEC000786	Leptinotarsa decemlineata	Putative uncharacterized protein
EOG091202IG	YQE_09485	Dendroctonus ponderosae	Putative uncharacterized protein
EOG091202IH	OTAU013567	Onthophagus taurus	Putative uncharacterized protein
EOG091202IH	AGLA021137	Anoplophora glabripennis	Putative uncharacterized protein
EOG091202IH	APLA007377	Agrilus planipennis	Putative uncharacterized protein
EOG091202IH	TC002096	Tribolium castaneum	Putative uncharacterized protein
EOG091202IH	LDEC022634	Leptinotarsa decemlineata	Putative uncharacterized protein
EOG091202IH	YQE_06146	Dendroctonus ponderosae	Putative uncharacterized protein
EOG091202II	OTAU005490	Onthophagus taurus	Putative uncharacterized protein

EOG091202II	AGLA008307	Anoplophora glabripennis	Putative uncharacterized protein
EOG091202II	APLA002434	Agrilus planipennis	Putative uncharacterized protein
EOG091202II	TC014730	Tribolium castaneum	Putative uncharacterized protein
EOG091202II	LDEC012509	Leptinotarsa decemlineata	Putative uncharacterized protein
EOG091202II	YQE_03062	Dendroctonus ponderosae	Putative uncharacterized protein
EOG091202IJ	OTAU001600	Onthophagus taurus	Putative uncharacterized protein
EOG091202IJ	AGLA005040	Anoplophora glabripennis	Putative uncharacterized protein
EOG091202IJ	APLA005483	Agrilus planipennis	Putative uncharacterized protein
EOG091202IJ	TC005867	Tribolium castaneum	Putative uncharacterized protein
EOG091202IJ	LDEC015502	Leptinotarsa decemlineata	Putative uncharacterized protein
EOG091202IJ	YQE_07002	Dendroctonus ponderosae	Putative uncharacterized protein
EOG091202IK	OTAU011837	Onthophagus taurus	Putative uncharacterized protein
EOG091202IK	AGLA008793	Anoplophora glabripennis	Putative uncharacterized protein
EOG091202IK	APLA009907	Agrilus planipennis	Putative uncharacterized protein
EOG091202IK	TC000986	Tribolium castaneum	Putative uncharacterized protein
EOG091202IK	LDEC001669	Leptinotarsa decemlineata	Putative uncharacterized protein
EOG091202IK	YQE_10618	Dendroctonus ponderosae	Putative uncharacterized protein
EOG091202IL	OTAU007483	Onthophagus taurus	Putative uncharacterized protein
EOG091202IL	AGLA004877	Anoplophora glabripennis	Putative uncharacterized protein
EOG091202IL	APLA015485	Agrilus planipennis	Putative uncharacterized protein
EOG091202IL	TC014856	Tribolium castaneum	Putative uncharacterized protein
EOG091202IL	LDEC005381	Leptinotarsa decemlineata	Putative uncharacterized protein
EOG091202IL	YQE_11584	Dendroctonus ponderosae	Putative uncharacterized protein
EOG091202IN	OTAU009133	Onthophagus taurus	Putative uncharacterized protein
EOG091202IN	AGLA015176	Anoplophora glabripennis	Putative uncharacterized protein
EOG091202IN	APLA015050	Agrilus planipennis	Putative uncharacterized protein
EOG091202IN	TC013701	Tribolium castaneum	Putative uncharacterized protein
EOG091202IN	LDEC016358	Leptinotarsa decemlineata	Putative uncharacterized protein
EOG091202IN	YQE_04839	Dendroctonus ponderosae	Putative uncharacterized protein
EOG091202IO	OTAU000788	Onthophagus taurus	Putative uncharacterized protein
EOG091202IO	AGLA011167	Anoplophora glabripennis	Putative uncharacterized protein
EOG091202IO	APLA013597	Agrilus planipennis	Putative uncharacterized protein
EOG091202IO	TC012712	Tribolium castaneum	Putative uncharacterized protein
EOG091202IO	LDEC008556	Leptinotarsa decemlineata	Putative uncharacterized protein
EOG091202IO	YQE_07990	Dendroctonus ponderosae	Putative uncharacterized protein
EOG091202IP	OTAU010995	Onthophagus taurus	Putative uncharacterized protein
EOG091202IP	AGLA008089	Anoplophora glabripennis	Putative uncharacterized protein
EOG091202IP	APLA007600	Agrilus planipennis	Putative uncharacterized protein
EOG091202IP	TC000574	Tribolium castaneum	Putative uncharacterized protein
EOG091202IP	LDEC003587	Leptinotarsa decemlineata	Putative uncharacterized protein
EOG091202IP	YQE_10958	Dendroctonus ponderosae	Putative uncharacterized protein
EOG091202IQ	OTAU013380	Onthophagus taurus	Putative uncharacterized protein
EOG091202IQ	AGLA008997	Anoplophora glabripennis	Putative uncharacterized protein
EOG091202IQ	APLA011333	Agrilus planipennis	Putative uncharacterized protein
EOG091202IQ	TC013168	Tribolium castaneum	Putative uncharacterized protein
EOG091202IQ	LDEC017886	Leptinotarsa decemlineata	Putative uncharacterized protein
EOG091202IQ	YQE_10569	Dendroctonus ponderosae	Putative uncharacterized protein
EOG091202IR	OTAU007686	Onthophagus taurus	DNA binding
EOG091202IR	AGLA001048	Anoplophora glabripennis	DNA binding
EOG091202IR	APLA000859	Agrilus planipennis	DNA binding
EOG091202IR	TC034305	Tribolium castaneum	DNA binding
EOG091202IR	LDEC009466	Leptinotarsa decemlineata	DNA binding
EOG091202IR	YQE_12837	Dendroctonus ponderosae	DNA binding
EOG091202IS	OTAU003850	Onthophagus taurus	Putative uncharacterized protein
EOG091202IS	AGLA014808	Anoplophora glabripennis	Putative uncharacterized protein
EOG091202IS	APLA013640	Agrilus planipennis	Putative uncharacterized protein
EOG091202IS	TC015920	Tribolium castaneum	Putative uncharacterized protein
EOG091202IS	LDEC001266	Leptinotarsa decemlineata	Putative uncharacterized protein
EOG091202IS	YQE_04299	Dendroctonus ponderosae	Putative uncharacterized protein
EOG091202IV	OTAU016601	Onthophagus taurus	Putative uncharacterized protein
EOG091202IV	AGLA007479	Anoplophora glabripennis	Putative uncharacterized protein
EOG091202IV	APLA008322	Agrilus planipennis	Putative uncharacterized protein
EOG091202IV	TC007804	Tribolium castaneum	Putative uncharacterized protein
EOG091202IV	LDEC018529	Leptinotarsa decemlineata	Putative uncharacterized protein
EOG091202IV	YQE_08395	Dendroctonus ponderosae	Putative uncharacterized protein
EOG091202IY	OTAU000057	Onthophagus taurus	Chaperone protein DnaJ 3
EOG091202IY	AGLA010923	Anoplophora glabripennis	Chaperone protein DnaJ 3

EOG091202IY	APLA014691	Agrilus planipennis	Chaperone protein DnaJ 3
EOG091202IY	TC013913	Tribolium castaneum	Chaperone protein DnaJ 3
EOG091202IY	LDEC007239	Leptinotarsa decemlineata	Chaperone protein DnaJ 3
EOG091202IY	YQE_02072	Dendroctonus ponderosae	Chaperone protein DnaJ 3
EOG091202J4	OTAU007714	Onthophagus taurus	Putative uncharacterized protein
EOG091202J4	AGLA003561	Anoplophora glabripennis	Putative uncharacterized protein
EOG091202J4	APLA002002	Agrilus planipennis	Putative uncharacterized protein
EOG091202J4	TC002432	Tribolium castaneum	Putative uncharacterized protein
EOG091202J4	LDEC016975	Leptinotarsa decemlineata	Putative uncharacterized protein
EOG091202J4	YQE_11115	Dendroctonus ponderosae	Putative uncharacterized protein
EOG091202J5	OTAU001850	Onthophagus taurus	Putative uncharacterized protein
EOG091202J5	AGLA010198	Anoplophora glabripennis	Putative uncharacterized protein
EOG091202J5	APLA009863	Agrilus planipennis	Putative uncharacterized protein
EOG091202J5	TC005330	Tribolium castaneum	Putative uncharacterized protein
EOG091202J5	LDEC006762	Leptinotarsa decemlineata	Putative uncharacterized protein
EOG091202J5	YQE_12797	Dendroctonus ponderosae	Putative uncharacterized protein
EOG091202J6	OTAU007797	Onthophagus taurus	Putative uncharacterized protein
EOG091202J6	AGLA000817	Anoplophora glabripennis	Putative uncharacterized protein
EOG091202J6	APLA012804	Agrilus planipennis	Putative uncharacterized protein
EOG091202J6	TC007966	Tribolium castaneum	Putative uncharacterized protein
EOG091202J6	LDEC001387	Leptinotarsa decemlineata	Putative uncharacterized protein
EOG091202J6	YQE_09608	Dendroctonus ponderosae	Putative uncharacterized protein
EOG091202J7	OTAU007977	Onthophagus taurus	Putative uncharacterized protein
EOG091202J7	AGLA011242	Anoplophora glabripennis	Putative uncharacterized protein
EOG091202J7	APLA008420	Agrilus planipennis	Putative uncharacterized protein
EOG091202J7	TC003493	Tribolium castaneum	Putative uncharacterized protein
EOG091202J7	LDEC003266	Leptinotarsa decemlineata	Putative uncharacterized protein
EOG091202J7	YQE_12344	Dendroctonus ponderosae	Putative uncharacterized protein
EOG091202J8	OTAU008193	Onthophagus taurus	Putative uncharacterized protein
EOG091202J8	AGLA009971	Anoplophora glabripennis	Putative uncharacterized protein
EOG091202J8	APLA010025	Agrilus planipennis	Putative uncharacterized protein
EOG091202J8	TC015215	Tribolium castaneum	Putative uncharacterized protein
EOG091202J8	LDEC010963	Leptinotarsa decemlineata	Putative uncharacterized protein
EOG091202J8	YQE_02007	Dendroctonus ponderosae	Putative uncharacterized protein
EOG091202J9	OTAU005402	Onthophagus taurus	Putative uncharacterized protein
EOG091202J9	AGLA000327	Anoplophora glabripennis	Putative uncharacterized protein
EOG091202J9	APLA009972	Agrilus planipennis	Putative uncharacterized protein
EOG091202J9	TC004116	Tribolium castaneum	Putative uncharacterized protein
EOG091202J9	LDEC004325	Leptinotarsa decemlineata	Putative uncharacterized protein
EOG091202J9	YQE_09058	Dendroctonus ponderosae	Putative uncharacterized protein
EOG091202JA	OTAU000203	Onthophagus taurus	zinc ion binding
EOG091202JA	AGLA004434	Anoplophora glabripennis	zinc ion binding
EOG091202JA	APLA002468	Agrilus planipennis	zinc ion binding
EOG091202JA	TC033149	Tribolium castaneum	zinc ion binding
EOG091202JA	LDEC001200	Leptinotarsa decemlineata	zinc ion binding
EOG091202JA	YQE_06132	Dendroctonus ponderosae	zinc ion binding
EOG091202JB	OTAU003219	Onthophagus taurus	Putative uncharacterized protein
EOG091202JB	AGLA004907	Anoplophora glabripennis	Putative uncharacterized protein
EOG091202JB	APLA010110	Agrilus planipennis	Putative uncharacterized protein
EOG091202JB	TC014513	Tribolium castaneum	Putative uncharacterized protein
EOG091202JB	LDEC011333	Leptinotarsa decemlineata	Putative uncharacterized protein
EOG091202JB	YQE_12497	Dendroctonus ponderosae	Putative uncharacterized protein
EOG091202JC	OTAU005796	Onthophagus taurus	Putative uncharacterized protein
EOG091202JC	AGLA001511	Anoplophora glabripennis	Putative uncharacterized protein
EOG091202JC	APLA003787	Agrilus planipennis	Putative uncharacterized protein
EOG091202JC	TC003806	Tribolium castaneum	Putative uncharacterized protein
EOG091202JC	LDEC004646	Leptinotarsa decemlineata	Putative uncharacterized protein
EOG091202JC	YQE_04557	Dendroctonus ponderosae	Putative uncharacterized protein
EOG091202JD	OTAU009184	Onthophagus taurus	Putative uncharacterized protein
EOG091202JD	AGLA005735	Anoplophora glabripennis	Putative uncharacterized protein
EOG091202JD	APLA000623	Agrilus planipennis	Putative uncharacterized protein
EOG091202JD	TC015318	Tribolium castaneum	Putative uncharacterized protein
EOG091202JD	LDEC006934	Leptinotarsa decemlineata	Putative uncharacterized protein
EOG091202JD	YQE_09433	Dendroctonus ponderosae	Putative uncharacterized protein
EOG091202JE	OTAU001989	Onthophagus taurus	Putative uncharacterized protein
EOG091202JE	AGLA002491	Anoplophora glabripennis	Putative uncharacterized protein
EOG091202JE	APLA012077	Agrilus planipennis	Putative uncharacterized protein

EOG091202JE	TC005357	Tribolium castaneum	Putative uncharacterized protein
EOG091202JE	LDEC006333	Leptinotarsa decemlineata	Putative uncharacterized protein
EOG091202JE	YQE_07516	Dendroctonus ponderosae	Putative uncharacterized protein
EOG091202JH	OTAU001226	Onthophagus taurus	Putative uncharacterized protein
EOG091202JH	AGLA002227	Anoplophora glabripennis	Putative uncharacterized protein
EOG091202JH	APLA012402	Agrilus planipennis	Putative uncharacterized protein
EOG091202JH	TC001465	Tribolium castaneum	Putative uncharacterized protein
EOG091202JH	LDEC021748	Leptinotarsa decemlineata	Putative uncharacterized protein
EOG091202JH	YQE_09225	Dendroctonus ponderosae	Putative uncharacterized protein
EOG091202JJ	OTAU011276	Onthophagus taurus	None
EOG091202JJ	AGLA003677	Anoplophora glabripennis	None
EOG091202JJ	APLA001994	Agrilus planipennis	None
EOG091202JJ	TC031034	Tribolium castaneum	None
EOG091202JJ	LDEC012170	Leptinotarsa decemlineata	None
EOG091202JJ	YQE_00014	Dendroctonus ponderosae	None
EOG091202JL	OTAU003762	Onthophagus taurus	Arrestin 2
EOG091202JL	AGLA005762	Anoplophora glabripennis	Arrestin 2
EOG091202JL	APLA015254	Agrilus planipennis	Arrestin 2
EOG091202JL	TC009551	Tribolium castaneum	Arrestin 2
EOG091202JL	LDEC017898	Leptinotarsa decemlineata	Arrestin 2
EOG091202JL	YQE_11312	Dendroctonus ponderosae	Arrestin 2
EOG091202JM	OTAU000428	Onthophagus taurus	Putative uncharacterized protein
EOG091202JM	AGLA007644	Anoplophora glabripennis	Putative uncharacterized protein
EOG091202JM	APLA000861	Agrilus planipennis	Putative uncharacterized protein
EOG091202JM	TC012553	Tribolium castaneum	Putative uncharacterized protein
EOG091202JM	LDEC003899	Leptinotarsa decemlineata	Putative uncharacterized protein
EOG091202JM	YQE_05496	Dendroctonus ponderosae	Putative uncharacterized protein
EOG091202JO	OTAU013097	Onthophagus taurus	Putative uncharacterized protein
EOG091202JO	AGLA019294	Anoplophora glabripennis	Putative uncharacterized protein
EOG091202JO	APLA008227	Agrilus planipennis	Putative uncharacterized protein
EOG091202JO	TC011256	Tribolium castaneum	Putative uncharacterized protein
EOG091202JO	LDEC015463	Leptinotarsa decemlineata	Putative uncharacterized protein
EOG091202JO	YQE_11226	Dendroctonus ponderosae	Putative uncharacterized protein
EOG091202JQ	OTAU000547	Onthophagus taurus	Putative uncharacterized protein
EOG091202JQ	AGLA004824	Anoplophora glabripennis	Putative uncharacterized protein
EOG091202JQ	APLA000888	Agrilus planipennis	Putative uncharacterized protein
EOG091202JQ	TC002268	Tribolium castaneum	Putative uncharacterized protein
EOG091202JQ	LDEC016048	Leptinotarsa decemlineata	Putative uncharacterized protein
EOG091202JQ	YQE_05858	Dendroctonus ponderosae	Putative uncharacterized protein
EOG091202JS	OTAU001780	Onthophagus taurus	Putative uncharacterized protein
EOG091202JS	AGLA019001	Anoplophora glabripennis	Putative uncharacterized protein
EOG091202JS	APLA005426	Agrilus planipennis	Putative uncharacterized protein
EOG091202JS	TC004314	Tribolium castaneum	Putative uncharacterized protein
EOG091202JS	LDEC024522	Leptinotarsa decemlineata	Putative uncharacterized protein
EOG091202JS	YQE_12700	Dendroctonus ponderosae	Putative uncharacterized protein
EOG091202JT	OTAU005417	Onthophagus taurus	Putative uncharacterized protein
EOG091202JT	AGLA004570	Anoplophora glabripennis	Putative uncharacterized protein
EOG091202JT	APLA010638	Agrilus planipennis	Putative uncharacterized protein
EOG091202JT	TC001964	Tribolium castaneum	Putative uncharacterized protein
EOG091202JT	LDEC001531	Leptinotarsa decemlineata	Putative uncharacterized protein
EOG091202JT	YQE_09254	Dendroctonus ponderosae	Putative uncharacterized protein
EOG091202JU	OTAU003974	Onthophagus taurus	Putative uncharacterized protein
EOG091202JU	AGLA016403	Anoplophora glabripennis	Putative uncharacterized protein
EOG091202JU	APLA011994	Agrilus planipennis	Putative uncharacterized protein
EOG091202JU	TC012625	Tribolium castaneum	Putative uncharacterized protein
EOG091202JU	LDEC017311	Leptinotarsa decemlineata	Putative uncharacterized protein
EOG091202JU	YQE_12959	Dendroctonus ponderosae	Putative uncharacterized protein
EOG091202JV	OTAU007648	Onthophagus taurus	Putative uncharacterized protein
EOG091202JV	AGLA011813	Anoplophora glabripennis	Putative uncharacterized protein
EOG091202JV	APLA007147	Agrilus planipennis	Putative uncharacterized protein
EOG091202JV	TC009722	Tribolium castaneum	Putative uncharacterized protein
EOG091202JV	LDEC001282	Leptinotarsa decemlineata	Putative uncharacterized protein
EOG091202JV	YQE_05284	Dendroctonus ponderosae	Putative uncharacterized protein
EOG091202JW	OTAU003368	Onthophagus taurus	Putative uncharacterized protein
EOG091202JW	AGLA008370	Anoplophora glabripennis	Putative uncharacterized protein
EOG091202JW	APLA001049	Agrilus planipennis	Putative uncharacterized protein
EOG091202JW	TC012869	Tribolium castaneum	Putative uncharacterized protein

EOG091202JW	LDEC015890	Leptinotarsa decemlineata	Putative uncharacterized protein
EOG091202JW	YQE_12842	Dendroctonus ponderosae	Putative uncharacterized protein
EOG091202K2	OTAU008695	Onthophagus taurus	None
EOG091202K2	AGLA020647	Anoplophora glabripennis	None
EOG091202K2	APLA001396	Agrilus planipennis	None
EOG091202K2	TC032221	Tribolium castaneum	None
EOG091202K2	LDEC015079	Leptinotarsa decemlineata	None
EOG091202K2	YQE_02347	Dendroctonus ponderosae	None
EOG091202K4	OTAU000560	Onthophagus taurus	Putative uncharacterized protein
EOG091202K4	AGLA020758	Anoplophora glabripennis	Putative uncharacterized protein
EOG091202K4	APLA013284	Agrilus planipennis	Putative uncharacterized protein
EOG091202K4	TC012506	Tribolium castaneum	Putative uncharacterized protein
EOG091202K4	LDEC011921	Leptinotarsa decemlineata	Putative uncharacterized protein
EOG091202K4	YQE_03100	Dendroctonus ponderosae	Putative uncharacterized protein
EOG091202K7	OTAU006635	Onthophagus taurus	Putative uncharacterized protein
EOG091202K7	AGLA020215	Anoplophora glabripennis	Putative uncharacterized protein
EOG091202K7	APLA000769	Agrilus planipennis	Putative uncharacterized protein
EOG091202K7	TC003502	Tribolium castaneum	Putative uncharacterized protein
EOG091202K7	LDEC008569	Leptinotarsa decemlineata	Putative uncharacterized protein
EOG091202K7	YQE_03719	Dendroctonus ponderosae	Putative uncharacterized protein
EOG091202K9	OTAU007032	Onthophagus taurus	None
EOG091202K9	AGLA003386	Anoplophora glabripennis	None
EOG091202K9	APLA001012	Agrilus planipennis	None
EOG091202K9	TC032584	Tribolium castaneum	None
EOG091202K9	LDEC010050	Leptinotarsa decemlineata	None
EOG091202K9	YQE_03638	Dendroctonus ponderosae	None
EOG091202KA	OTAU001691	Onthophagus taurus	Putative uncharacterized protein
EOG091202KA	AGLA010944	Anoplophora glabripennis	Putative uncharacterized protein
EOG091202KA	APLA004542	Agrilus planipennis	Putative uncharacterized protein
EOG091202KA	TC005756	Tribolium castaneum	Putative uncharacterized protein
EOG091202KA	LDEC016295	Leptinotarsa decemlineata	Putative uncharacterized protein
EOG091202KA	YQE_09441	Dendroctonus ponderosae	Putative uncharacterized protein
EOG091202KB	OTAU005970	Onthophagus taurus	Putative uncharacterized protein
EOG091202KB	AGLA006209	Anoplophora glabripennis	Putative uncharacterized protein
EOG091202KB	APLA014440	Agrilus planipennis	Putative uncharacterized protein
EOG091202KB	TC002396	Tribolium castaneum	Putative uncharacterized protein
EOG091202KB	LDEC017203	Leptinotarsa decemlineata	Putative uncharacterized protein
EOG091202KB	YQE_05883	Dendroctonus ponderosae	Putative uncharacterized protein
EOG091202KC	OTAU002546	Onthophagus taurus	Putative uncharacterized protein
EOG091202KC	AGLA011082	Anoplophora glabripennis	Putative uncharacterized protein
EOG091202KC	APLA015391	Agrilus planipennis	Putative uncharacterized protein
EOG091202KC	TC005458	Tribolium castaneum	Putative uncharacterized protein
EOG091202KC	LDEC002919	Leptinotarsa decemlineata	Putative uncharacterized protein
EOG091202KC	YQE_07477	Dendroctonus ponderosae	Putative uncharacterized protein
EOG091202KD	OTAU003905	Onthophagus taurus	Putative uncharacterized protein
EOG091202KD	AGLA015639	Anoplophora glabripennis	Putative uncharacterized protein
EOG091202KD	APLA011973	Agrilus planipennis	Putative uncharacterized protein
EOG091202KD	TC010176	Tribolium castaneum	Putative uncharacterized protein
EOG091202KD	LDEC010193	Leptinotarsa decemlineata	Putative uncharacterized protein
EOG091202KD	YQE_12642	Dendroctonus ponderosae	Putative uncharacterized protein
EOG091202KF	OTAU003868	Onthophagus taurus	damaged DNA binding
EOG091202KF	AGLA011843	Anoplophora glabripennis	damaged DNA binding
EOG091202KF	APLA004572	Agrilus planipennis	damaged DNA binding
EOG091202KF	TC031208	Tribolium castaneum	damaged DNA binding
EOG091202KF	LDEC009370	Leptinotarsa decemlineata	damaged DNA binding
EOG091202KF	YQE_09722	Dendroctonus ponderosae	damaged DNA binding
EOG091202KH	OTAU011767	Onthophagus taurus	None
EOG091202KH	AGLA016994	Anoplophora glabripennis	None
EOG091202KH	APLA005904	Agrilus planipennis	None
EOG091202KH	TC034748	Tribolium castaneum	None
EOG091202KH	LDEC003634	Leptinotarsa decemlineata	None
EOG091202KH	YQE_02745	Dendroctonus ponderosae	None
EOG091202KI	OTAU006747	Onthophagus taurus	Putative uncharacterized protein
EOG091202KI	AGLA010205	Anoplophora glabripennis	Putative uncharacterized protein
EOG091202KI	APLA001780	Agrilus planipennis	Putative uncharacterized protein
EOG091202KI	TC002258	Tribolium castaneum	Putative uncharacterized protein
EOG091202KI	LDEC000521	Leptinotarsa decemlineata	Putative uncharacterized protein

EOG091202KI	YQE_07970	Dendroctonus ponderosae	Putative uncharacterized protein
EOG091202KJ	OTAU006725	Onthophagus taurus	Putative uncharacterized protein
EOG091202KJ	AGLA001404	Anoplophora glabripennis	Putative uncharacterized protein
EOG091202KJ	APLA003710	Agrilus planipennis	Putative uncharacterized protein
EOG091202KJ	TC004962	Tribolium castaneum	Putative uncharacterized protein
EOG091202KJ	LDEC014750	Leptinotarsa decemlineata	Putative uncharacterized protein
EOG091202KJ	YQE_10759	Dendroctonus ponderosae	Putative uncharacterized protein
EOG091202KM	OTAU007496	Onthophagus taurus	Putative uncharacterized protein
EOG091202KM	AGLA010454	Anoplophora glabripennis	Putative uncharacterized protein
EOG091202KM	APLA005970	Agrilus planipennis	Putative uncharacterized protein
EOG091202KM	TC015811	Tribolium castaneum	Putative uncharacterized protein
EOG091202KM	LDEC015298	Leptinotarsa decemlineata	Putative uncharacterized protein
EOG091202KM	YQE_11758	Dendroctonus ponderosae	Putative uncharacterized protein
EOG091202KS	OTAU000043	Onthophagus taurus	Putative uncharacterized protein
EOG091202KS	AGLA017890	Anoplophora glabripennis	Putative uncharacterized protein
EOG091202KS	APLA002451	Agrilus planipennis	Putative uncharacterized protein
EOG091202KS	TC013685	Tribolium castaneum	Putative uncharacterized protein
EOG091202KS	LDEC016283	Leptinotarsa decemlineata	Putative uncharacterized protein
EOG091202KS	YQE_04520	Dendroctonus ponderosae	Putative uncharacterized protein
EOG091202KV	OTAU004984	Onthophagus taurus	Putative uncharacterized protein
EOG091202KV	AGLA006665	Anoplophora glabripennis	Putative uncharacterized protein
EOG091202KV	APLA005013	Agrilus planipennis	Putative uncharacterized protein
EOG091202KV	TC000412	Tribolium castaneum	Putative uncharacterized protein
EOG091202KV	LDEC001597	Leptinotarsa decemlineata	Putative uncharacterized protein
EOG091202KV	YQE_07699	Dendroctonus ponderosae	Putative uncharacterized protein
EOG091202KX	OTAU011131	Onthophagus taurus	Putative uncharacterized protein
EOG091202KX	AGLA021070	Anoplophora glabripennis	Putative uncharacterized protein
EOG091202KX	APLA005146	Agrilus planipennis	Putative uncharacterized protein
EOG091202KX	TC014595	Tribolium castaneum	Putative uncharacterized protein
EOG091202KX	LDEC014496	Leptinotarsa decemlineata	Putative uncharacterized protein
EOG091202KX	YQE_06209	Dendroctonus ponderosae	Putative uncharacterized protein
EOG091202L1	OTAU015040	Onthophagus taurus	Putative uncharacterized protein
EOG091202L1	AGLA006301	Anoplophora glabripennis	Putative uncharacterized protein
EOG091202L1	APLA011275	Agrilus planipennis	Putative uncharacterized protein
EOG091202L1	TC009295	Tribolium castaneum	Putative uncharacterized protein
EOG091202L1	LDEC002109	Leptinotarsa decemlineata	Putative uncharacterized protein
EOG091202L1	YQE_01566	Dendroctonus ponderosae	Putative uncharacterized protein
EOG091202L3	OTAU008125	Onthophagus taurus	Putative uncharacterized protein
EOG091202L3	AGLA005831	Anoplophora glabripennis	Putative uncharacterized protein
EOG091202L3	APLA001248	Agrilus planipennis	Putative uncharacterized protein
EOG091202L3	TC000575	Tribolium castaneum	Putative uncharacterized protein
EOG091202L3	LDEC001688	Leptinotarsa decemlineata	Putative uncharacterized protein
EOG091202L3	YQE_09714	Dendroctonus ponderosae	Putative uncharacterized protein
EOG091202L4	OTAU005049	Onthophagus taurus	Putative uncharacterized protein
EOG091202L4	AGLA014986	Anoplophora glabripennis	Putative uncharacterized protein
EOG091202L4	APLA007489	Agrilus planipennis	Putative uncharacterized protein
EOG091202L4	TC006147	Tribolium castaneum	Putative uncharacterized protein
EOG091202L4	LDEC009858	Leptinotarsa decemlineata	Putative uncharacterized protein
EOG091202L4	YQE_07859	Dendroctonus ponderosae	Putative uncharacterized protein
EOG091202L6	OTAU017051	Onthophagus taurus	Putative uncharacterized protein
EOG091202L6	AGLA010019	Anoplophora glabripennis	Putative uncharacterized protein
EOG091202L6	APLA002300	Agrilus planipennis	Putative uncharacterized protein
EOG091202L6	TC008298	Tribolium castaneum	Putative uncharacterized protein
EOG091202L6	LDEC000668	Leptinotarsa decemlineata	Putative uncharacterized protein
EOG091202L6	YQE_05217	Dendroctonus ponderosae	Putative uncharacterized protein
EOG091202L9	OTAU007718	Onthophagus taurus	Putative uncharacterized protein
EOG091202L9	AGLA021246	Anoplophora glabripennis	Putative uncharacterized protein
EOG091202L9	APLA002022	Agrilus planipennis	Putative uncharacterized protein
EOG091202L9	TC011515	Tribolium castaneum	Putative uncharacterized protein
EOG091202L9	LDEC005932	Leptinotarsa decemlineata	Putative uncharacterized protein
EOG091202L9	YQE_02177	Dendroctonus ponderosae	Putative uncharacterized protein
EOG091202LA	OTAU015510	Onthophagus taurus	Putative uncharacterized protein
EOG091202LA	AGLA008866	Anoplophora glabripennis	Putative uncharacterized protein
EOG091202LA	APLA004437	Agrilus planipennis	Putative uncharacterized protein
EOG091202LA	TC006811	Tribolium castaneum	Putative uncharacterized protein
EOG091202LA	LDEC004934	Leptinotarsa decemlineata	Putative uncharacterized protein
EOG091202LA	YQE_10186	Dendroctonus ponderosae	Putative uncharacterized protein

EOG091202LB	OTAU008276	Onthophagus taurus	Putative uncharacterized protein
EOG091202LB	AGLA005260	Anoplophora glabripennis	Putative uncharacterized protein
EOG091202LB	APLA002222	Agrilus planipennis	Putative uncharacterized protein
EOG091202LB	TC008297	Tribolium castaneum	Putative uncharacterized protein
EOG091202LB	LDEC008458	Leptinotarsa decemlineata	Putative uncharacterized protein
EOG091202LB	YQE_04207	Dendroctonus ponderosae	Putative uncharacterized protein
EOG091202LD	OTAU016047	Onthophagus taurus	Putative uncharacterized protein
EOG091202LD	AGLA000641	Anoplophora glabripennis	Putative uncharacterized protein
EOG091202LD	APLA007929	Agrilus planipennis	Putative uncharacterized protein
EOG091202LD	TC000463	Tribolium castaneum	Putative uncharacterized protein
EOG091202LD	LDEC013066	Leptinotarsa decemlineata	Putative uncharacterized protein
EOG091202LD	YQE_01616	Dendroctonus ponderosae	Putative uncharacterized protein
EOG091202LE	OTAU002837	Onthophagus taurus	Putative uncharacterized protein
EOG091202LE	AGLA000209	Anoplophora glabripennis	Putative uncharacterized protein
EOG091202LE	APLA014546	Agrilus planipennis	Putative uncharacterized protein
EOG091202LE	TC004801	Tribolium castaneum	Putative uncharacterized protein
EOG091202LE	LDEC004519	Leptinotarsa decemlineata	Putative uncharacterized protein
EOG091202LE	YQE_06601	Dendroctonus ponderosae	Putative uncharacterized protein
EOG091202LF	OTAU008938	Onthophagus taurus	Putative uncharacterized protein
EOG091202LF	AGLA018373	Anoplophora glabripennis	Putative uncharacterized protein
EOG091202LF	APLA006809	Agrilus planipennis	Putative uncharacterized protein
EOG091202LF	TC003655	Tribolium castaneum	Putative uncharacterized protein
EOG091202LF	LDEC009007	Leptinotarsa decemlineata	Putative uncharacterized protein
EOG091202LF	YQE_08371	Dendroctonus ponderosae	Putative uncharacterized protein
EOG091202LN	OTAU008158	Onthophagus taurus	Putative uncharacterized protein
EOG091202LN	AGLA003353	Anoplophora glabripennis	Putative uncharacterized protein
EOG091202LN	APLA014361	Agrilus planipennis	Putative uncharacterized protein
EOG091202LN	TC015755	Tribolium castaneum	Putative uncharacterized protein
EOG091202LN	LDEC008630	Leptinotarsa decemlineata	Putative uncharacterized protein
EOG091202LN	YQE_08424	Dendroctonus ponderosae	Putative uncharacterized protein
EOG091202LT	OTAU001103	Onthophagus taurus	Putative uncharacterized protein
EOG091202LT	AGLA002965	Anoplophora glabripennis	Putative uncharacterized protein
EOG091202LT	APLA009341	Agrilus planipennis	Putative uncharacterized protein
EOG091202LT	TC004846	Tribolium castaneum	Putative uncharacterized protein
EOG091202LT	LDEC010987	Leptinotarsa decemlineata	Putative uncharacterized protein
EOG091202LT	YQE_08157	Dendroctonus ponderosae	Putative uncharacterized protein
EOG091202LW	OTAU000304	Onthophagus taurus	Queuine tRNA-ribosyltransferase
EOG091202LW	AGLA014199	Anoplophora glabripennis	Queuine tRNA-ribosyltransferase
EOG091202LW	APLA009733	Agrilus planipennis	Queuine tRNA-ribosyltransferase
EOG091202LW	TC010418	Tribolium castaneum	Queuine tRNA-ribosyltransferase
EOG091202LW	LDEC015114	Leptinotarsa decemlineata	Queuine tRNA-ribosyltransferase
EOG091202LW	YQE_08300	Dendroctonus ponderosae	Queuine tRNA-ribosyltransferase
EOG091202LZ	OTAU010328	Onthophagus taurus	nucleotidyltransferase activity
EOG091202LZ	AGLA006508	Anoplophora glabripennis	nucleotidyltransferase activity
EOG091202LZ	APLA011110	Agrilus planipennis	nucleotidyltransferase activity
EOG091202LZ	TC033614	Tribolium castaneum	nucleotidyltransferase activity
EOG091202LZ	LDEC014617	Leptinotarsa decemlineata	nucleotidyltransferase activity
EOG091202LZ	YQE_03060	Dendroctonus ponderosae	nucleotidyltransferase activity
EOG091202M1	OTAU009467	Onthophagus taurus	hydrolase activity
EOG091202M1	AGLA016555	Anoplophora glabripennis	hydrolase activity
EOG091202M1	APLA000847	Agrilus planipennis	hydrolase activity
EOG091202M1	TC031691	Tribolium castaneum	hydrolase activity
EOG091202M1	LDEC000508	Leptinotarsa decemlineata	hydrolase activity
EOG091202M1	YQE_07967	Dendroctonus ponderosae	hydrolase activity
EOG091202M5	OTAU008733	Onthophagus taurus	Putative uncharacterized protein
EOG091202M5	AGLA014949	Anoplophora glabripennis	Putative uncharacterized protein
EOG091202M5	APLA013745	Agrilus planipennis	Putative uncharacterized protein
EOG091202M5	TC002973	Tribolium castaneum	Putative uncharacterized protein
EOG091202M5	LDEC002042	Leptinotarsa decemlineata	Putative uncharacterized protein
EOG091202M5	YQE_10270	Dendroctonus ponderosae	Putative uncharacterized protein
EOG091202M6	OTAU001938	Onthophagus taurus	Putative uncharacterized protein
EOG091202M6	AGLA007273	Anoplophora glabripennis	Putative uncharacterized protein
EOG091202M6	APLA004478	Agrilus planipennis	Putative uncharacterized protein
EOG091202M6	TC006364	Tribolium castaneum	Putative uncharacterized protein
EOG091202M6	LDEC009693	Leptinotarsa decemlineata	Putative uncharacterized protein
EOG091202M6	YQE_01910	Dendroctonus ponderosae	Putative uncharacterized protein
EOG091202M7	OTAU007451	Onthophagus taurus	Putative uncharacterized protein

EOG091202M7	AGLA004183	Anoplophora glabripennis	Putative uncharacterized protein
EOG091202M7	APLA011957	Agrilus planipennis	Putative uncharacterized protein
EOG091202M7	TC015505	Tribolium castaneum	Putative uncharacterized protein
EOG091202M7	LDEC005619	Leptinotarsa decemlineata	Putative uncharacterized protein
EOG091202M7	YQE_11717	Dendroctonus ponderosae	Putative uncharacterized protein
EOG091202M9	OTAU009230	Onthophagus taurus	Putative uncharacterized protein
EOG091202M9	AGLA013904	Anoplophora glabripennis	Putative uncharacterized protein
EOG091202M9	APLA003780	Agrilus planipennis	Putative uncharacterized protein
EOG091202M9	TC002132	Tribolium castaneum	Putative uncharacterized protein
EOG091202M9	LDEC002739	Leptinotarsa decemlineata	Putative uncharacterized protein
EOG091202M9	YQE_02654	Dendroctonus ponderosae	Putative uncharacterized protein
EOG091202MC	OTAU000031	Onthophagus taurus	Putative uncharacterized protein
EOG091202MC	AGLA011937	Anoplophora glabripennis	Putative uncharacterized protein
EOG091202MC	APLA006226	Agrilus planipennis	Putative uncharacterized protein
EOG091202MC	TC013430	Tribolium castaneum	Putative uncharacterized protein
EOG091202MC	LDEC015561	Leptinotarsa decemlineata	Putative uncharacterized protein
EOG091202MC	YQE_03991	Dendroctonus ponderosae	Putative uncharacterized protein
EOG091202MD	OTAU009027	Onthophagus taurus	Putative uncharacterized protein
EOG091202MD	AGLA015436	Anoplophora glabripennis	Putative uncharacterized protein
EOG091202MD	APLA005096	Agrilus planipennis	Putative uncharacterized protein
EOG091202MD	TC009268	Tribolium castaneum	Putative uncharacterized protein
EOG091202MD	LDEC006434	Leptinotarsa decemlineata	Putative uncharacterized protein
EOG091202MD	YQE_10592	Dendroctonus ponderosae	Putative uncharacterized protein
EOG091202MG	OTAU013622	Onthophagus taurus	Putative uncharacterized protein
EOG091202MG	AGLA007173	Anoplophora glabripennis	Putative uncharacterized protein
EOG091202MG	APLA001846	Agrilus planipennis	Putative uncharacterized protein
EOG091202MG	TC015943	Tribolium castaneum	Putative uncharacterized protein
EOG091202MG	LDEC010856	Leptinotarsa decemlineata	Putative uncharacterized protein
EOG091202MG	YQE_05242	Dendroctonus ponderosae	Putative uncharacterized protein
EOG091202MI	OTAU002017	Onthophagus taurus	Putative uncharacterized protein
EOG091202MI	AGLA003145	Anoplophora glabripennis	Putative uncharacterized protein
EOG091202MI	APLA002140	Agrilus planipennis	Putative uncharacterized protein
EOG091202MI	TC011967	Tribolium castaneum	Putative uncharacterized protein
EOG091202MI	LDEC014553	Leptinotarsa decemlineata	Putative uncharacterized protein
EOG091202MI	YQE_04316	Dendroctonus ponderosae	Putative uncharacterized protein
EOG091202MP	OTAU006519	Onthophagus taurus	Putative uncharacterized protein
EOG091202MP	AGLA016453	Anoplophora glabripennis	Putative uncharacterized protein
EOG091202MP	APLA011378	Agrilus planipennis	Putative uncharacterized protein
EOG091202MP	TC003222	Tribolium castaneum	Putative uncharacterized protein
EOG091202MP	LDEC013263	Leptinotarsa decemlineata	Putative uncharacterized protein
EOG091202MP	YQE_09853	Dendroctonus ponderosae	Putative uncharacterized protein
EOG091202MQ	OTAU014510	Onthophagus taurus	None
EOG091202MQ	AGLA017924	Anoplophora glabripennis	None
EOG091202MQ	APLA013209	Agrilus planipennis	None
EOG091202MQ	TC033615	Tribolium castaneum	None
EOG091202MQ	LDEC015379	Leptinotarsa decemlineata	None
EOG091202MQ	YQE_05321	Dendroctonus ponderosae	None
EOG091202MU	OTAU013015	Onthophagus taurus	Putative uncharacterized protein
EOG091202MU	AGLA010696	Anoplophora glabripennis	Putative uncharacterized protein
EOG091202MU	APLA011067	Agrilus planipennis	Putative uncharacterized protein
EOG091202MU	TC006345	Tribolium castaneum	Putative uncharacterized protein
EOG091202MU	LDEC013900	Leptinotarsa decemlineata	Putative uncharacterized protein
EOG091202MU	YQE_05621	Dendroctonus ponderosae	Putative uncharacterized protein
EOG091202MW	OTAU007475	Onthophagus taurus	Putative uncharacterized protein
EOG091202MW	AGLA006340	Anoplophora glabripennis	Putative uncharacterized protein
EOG091202MW	APLA003823	Agrilus planipennis	Putative uncharacterized protein
EOG091202MW	TC015728	Tribolium castaneum	Putative uncharacterized protein
EOG091202MW	LDEC007867	Leptinotarsa decemlineata	Putative uncharacterized protein
EOG091202MW	YQE_08348	Dendroctonus ponderosae	Putative uncharacterized protein
EOG091202MY	OTAU003268	Onthophagus taurus	oxidoreductase activity
EOG091202MY	AGLA003348	Anoplophora glabripennis	oxidoreductase activity
EOG091202MY	APLA013504	Agrilus planipennis	oxidoreductase activity
EOG091202MY	TC034016	Tribolium castaneum	oxidoreductase activity
EOG091202MY	LDEC008615	Leptinotarsa decemlineata	oxidoreductase activity
EOG091202MY	YQE_11747	Dendroctonus ponderosae	oxidoreductase activity
EOG091202N2	OTAU007968	Onthophagus taurus	substrate-specific transmembrane transporter activity
EOG091202N2	AGLA012888	Anoplophora glabripennis	substrate-specific transmembrane transporter activity

EOG091202N2	APLA013203	Agrilus planipennis	substrate-specific transmembrane transporter activity
EOG091202N2	TC032400	Tribolium castaneum	substrate-specific transmembrane transporter activity
EOG091202N2	LDEC014444	Leptinotarsa decemlineata	substrate-specific transmembrane transporter activity
EOG091202N2	YQE_02180	Dendroctonus ponderosae	substrate-specific transmembrane transporter activity
EOG091202N3	OTAU006496	Onthophagus taurus	None
EOG091202N3	AGLA010887	Anoplophora glabripennis	None
EOG091202N3	APLA008995	Agrilus planipennis	None
EOG091202N3	TC033086	Tribolium castaneum	None
EOG091202N3	LDEC021543	Leptinotarsa decemlineata	None
EOG091202N3	YQE_06313	Dendroctonus ponderosae	None
EOG091202N5	OTAU001283	Onthophagus taurus	Putative uncharacterized protein
EOG091202N5	AGLA000323	Anoplophora glabripennis	Putative uncharacterized protein
EOG091202N5	APLA011852	Agrilus planipennis	Putative uncharacterized protein
EOG091202N5	TC004142	Tribolium castaneum	Putative uncharacterized protein
EOG091202N5	LDEC020548	Leptinotarsa decemlineata	Putative uncharacterized protein
EOG091202N5	YQE_01961	Dendroctonus ponderosae	Putative uncharacterized protein
EOG091202N7	OTAU008403	Onthophagus taurus	Putative uncharacterized protein
EOG091202N7	AGLA001473	Anoplophora glabripennis	Putative uncharacterized protein
EOG091202N7	APLA008732	Agrilus planipennis	Putative uncharacterized protein
EOG091202N7	TC002777	Tribolium castaneum	Putative uncharacterized protein
EOG091202N7	LDEC019639	Leptinotarsa decemlineata	Putative uncharacterized protein
EOG091202N7	YQE_05179	Dendroctonus ponderosae	Putative uncharacterized protein
EOG091202N8	OTAU013607	Onthophagus taurus	Putative uncharacterized protein
EOG091202N8	AGLA019581	Anoplophora glabripennis	Putative uncharacterized protein
EOG091202N8	APLA003963	Agrilus planipennis	Putative uncharacterized protein
EOG091202N8	TC012406	Tribolium castaneum	Putative uncharacterized protein
EOG091202N8	LDEC000533	Leptinotarsa decemlineata	Putative uncharacterized protein
EOG091202N8	YQE_10315	Dendroctonus ponderosae	Putative uncharacterized protein
EOG091202N9	OTAU013136	Onthophagus taurus	Putative uncharacterized protein
EOG091202N9	AGLA017453	Anoplophora glabripennis	Putative uncharacterized protein
EOG091202N9	APLA012089	Agrilus planipennis	Putative uncharacterized protein
EOG091202N9	TC005889	Tribolium castaneum	Putative uncharacterized protein
EOG091202N9	LDEC015896	Leptinotarsa decemlineata	Putative uncharacterized protein
EOG091202N9	YQE_12089	Dendroctonus ponderosae	Putative uncharacterized protein
EOG091202NA	OTAU004926	Onthophagus taurus	Synaptotagmin IV
EOG091202NA	AGLA017431	Anoplophora glabripennis	Synaptotagmin IV
EOG091202NA	APLA005273	Agrilus planipennis	Synaptotagmin IV
EOG091202NA	TC011462	Tribolium castaneum	Synaptotagmin IV
EOG091202NA	LDEC003390	Leptinotarsa decemlineata	Synaptotagmin IV
EOG091202NA	YQE_09729	Dendroctonus ponderosae	Synaptotagmin IV
EOG091202NC	OTAU003932	Onthophagus taurus	hydrolase activity
EOG091202NC	AGLA018971	Anoplophora glabripennis	hydrolase activity
EOG091202NC	APLA011993	Agrilus planipennis	hydrolase activity
EOG091202NC	TC034322	Tribolium castaneum	hydrolase activity
EOG091202NC	LDEC015605	Leptinotarsa decemlineata	hydrolase activity
EOG091202NC	YQE_07994	Dendroctonus ponderosae	hydrolase activity
EOG091202NH	OTAU005964	Onthophagus taurus	Putative uncharacterized protein
EOG091202NH	AGLA003366	Anoplophora glabripennis	Putative uncharacterized protein
EOG091202NH	APLA003804	Agrilus planipennis	Putative uncharacterized protein
EOG091202NH	TC002836	Tribolium castaneum	Putative uncharacterized protein
EOG091202NH	LDEC018412	Leptinotarsa decemlineata	Putative uncharacterized protein
EOG091202NH	YQE_05160	Dendroctonus ponderosae	Putative uncharacterized protein
EOG091202NJ	OTAU002594	Onthophagus taurus	Putative uncharacterized protein
EOG091202NJ	AGLA015967	Anoplophora glabripennis	Putative uncharacterized protein
EOG091202NJ	APLA008631	Agrilus planipennis	Putative uncharacterized protein
EOG091202NJ	TC014478	Tribolium castaneum	Putative uncharacterized protein
EOG091202NJ	LDEC001155	Leptinotarsa decemlineata	Putative uncharacterized protein
EOG091202NJ	YQE_12256	Dendroctonus ponderosae	Putative uncharacterized protein
EOG091202NK	OTAU014898	Onthophagus taurus	Serpin peptidase inhibitor 28
EOG091202NK	AGLA003994	Anoplophora glabripennis	Serpin peptidase inhibitor 28
EOG091202NK	APLA009838	Agrilus planipennis	Serpin peptidase inhibitor 28
EOG091202NK	TC013310	Tribolium castaneum	Serpin peptidase inhibitor 28
EOG091202NK	LDEC020562	Leptinotarsa decemlineata	Serpin peptidase inhibitor 28
EOG091202NK	YQE_10373	Dendroctonus ponderosae	Serpin peptidase inhibitor 28
EOG091202NR	OTAU010163	Onthophagus taurus	Putative uncharacterized protein
EOG091202NR	AGLA008294	Anoplophora glabripennis	Putative uncharacterized protein
EOG091202NR	APLA012572	Agrilus planipennis	Putative uncharacterized protein

EOG091202NR	TC009345	Tribolium castaneum	Putative uncharacterized protein
EOG091202NR	LDEC001261	Leptinotarsa decemlineata	Putative uncharacterized protein
EOG091202NR	YQE_06004	Dendroctonus ponderosae	Putative uncharacterized protein
EOG091202NV	OTAU006718	Onthophagus taurus	Putative uncharacterized protein
EOG091202NV	AGLA000014	Anoplophora glabripennis	Putative uncharacterized protein
EOG091202NV	APLA008006	Agrilus planipennis	Putative uncharacterized protein
EOG091202NV	TC000610	Tribolium castaneum	Putative uncharacterized protein
EOG091202NV	LDEC007044	Leptinotarsa decemlineata	Putative uncharacterized protein
EOG091202NV	YQE_10722	Dendroctonus ponderosae	Putative uncharacterized protein
EOG091202NW	OTAU003854	Onthophagus taurus	Putative uncharacterized protein
EOG091202NW	AGLA007989	Anoplophora glabripennis	Putative uncharacterized protein
EOG091202NW	APLA006946	Agrilus planipennis	Putative uncharacterized protein
EOG091202NW	TC009024	Tribolium castaneum	Putative uncharacterized protein
EOG091202NW	LDEC010704	Leptinotarsa decemlineata	Putative uncharacterized protein
EOG091202NW	YQE_02213	Dendroctonus ponderosae	Putative uncharacterized protein
EOG091202NX	OTAU012239	Onthophagus taurus	hydrolase activity
EOG091202NX	AGLA018520	Anoplophora glabripennis	hydrolase activity
EOG091202NX	APLA006974	Agrilus planipennis	hydrolase activity
EOG091202NX	TC033621	Tribolium castaneum	hydrolase activity
EOG091202NX	LDEC012896	Leptinotarsa decemlineata	hydrolase activity
EOG091202NX	YQE_03522	Dendroctonus ponderosae	hydrolase activity
EOG091202NY	OTAU014204	Onthophagus taurus	Putative uncharacterized protein
EOG091202NY	AGLA000558	Anoplophora glabripennis	Putative uncharacterized protein
EOG091202NY	APLA001483	Agrilus planipennis	Putative uncharacterized protein
EOG091202NY	TC003159	Tribolium castaneum	Putative uncharacterized protein
EOG091202NY	LDEC003729	Leptinotarsa decemlineata	Putative uncharacterized protein
EOG091202NY	YQE_07898	Dendroctonus ponderosae	Putative uncharacterized protein
EOG091202O0	OTAU001536	Onthophagus taurus	Putative uncharacterized protein
EOG091202O0	AGLA001236	Anoplophora glabripennis	Putative uncharacterized protein
EOG091202O0	APLA000404	Agrilus planipennis	Putative uncharacterized protein
EOG091202O0	TC006219	Tribolium castaneum	Putative uncharacterized protein
EOG091202O0	LDEC011063	Leptinotarsa decemlineata	Putative uncharacterized protein
EOG091202O0	YQE_12017	Dendroctonus ponderosae	Putative uncharacterized protein
EOG091202O1	OTAU005591	Onthophagus taurus	Putative uncharacterized protein
EOG091202O1	AGLA003831	Anoplophora glabripennis	Putative uncharacterized protein
EOG091202O1	APLA007206	Agrilus planipennis	Putative uncharacterized protein
EOG091202O1	TC008922	Tribolium castaneum	Putative uncharacterized protein
EOG091202O1	LDEC016809	Leptinotarsa decemlineata	Putative uncharacterized protein
EOG091202O1	YQE_03434	Dendroctonus ponderosae	Putative uncharacterized protein
EOG091202O3	OTAU012137	Onthophagus taurus	Putative uncharacterized protein
EOG091202O3	AGLA014975	Anoplophora glabripennis	Putative uncharacterized protein
EOG091202O3	APLA004076	Agrilus planipennis	Putative uncharacterized protein
EOG091202O3	TC002983	Tribolium castaneum	Putative uncharacterized protein
EOG091202O3	LDEC014857	Leptinotarsa decemlineata	Putative uncharacterized protein
EOG091202O3	YQE_07395	Dendroctonus ponderosae	Putative uncharacterized protein
EOG091202O4	OTAU004525	Onthophagus taurus	Putative uncharacterized protein
EOG091202O4	AGLA016062	Anoplophora glabripennis	Putative uncharacterized protein
EOG091202O4	APLA002364	Agrilus planipennis	Putative uncharacterized protein
EOG091202O4	TC011896	Tribolium castaneum	Putative uncharacterized protein
EOG091202O4	LDEC007602	Leptinotarsa decemlineata	Putative uncharacterized protein
EOG091202O4	YQE_08602	Dendroctonus ponderosae	Putative uncharacterized protein
EOG091202O5	OTAU007603	Onthophagus taurus	None
EOG091202O5	AGLA020241	Anoplophora glabripennis	None
EOG091202O5	APLA008285	Agrilus planipennis	None
EOG091202O5	TC032604	Tribolium castaneum	None
EOG091202O5	LDEC001489	Leptinotarsa decemlineata	None
EOG091202O5	YQE_08373	Dendroctonus ponderosae	None
EOG091202O8	OTAU011812	Onthophagus taurus	Putative uncharacterized protein
EOG091202O8	AGLA016423	Anoplophora glabripennis	Putative uncharacterized protein
EOG091202O8	APLA004501	Agrilus planipennis	Putative uncharacterized protein
EOG091202O8	TC007436	Tribolium castaneum	Putative uncharacterized protein
EOG091202O8	LDEC024059	Leptinotarsa decemlineata	Putative uncharacterized protein
EOG091202O8	YQE_12299	Dendroctonus ponderosae	Putative uncharacterized protein
EOG091202O9	OTAU000632	Onthophagus taurus	Putative uncharacterized protein
EOG091202O9	AGLA020408	Anoplophora glabripennis	Putative uncharacterized protein
EOG091202O9	APLA010058	Agrilus planipennis	Putative uncharacterized protein
EOG091202O9	TC012118	Tribolium castaneum	Putative uncharacterized protein

EOG091202O9	LDEC021627	Leptinotarsa decemlineata	Putative uncharacterized protein
EOG091202O9	YQE_12955	Dendroctonus ponderosae	Putative uncharacterized protein
EOG091202OD	OTAU010154	Onthophagus taurus	Putative uncharacterized protein
EOG091202OD	AGLA001388	Anoplophora glabripennis	Putative uncharacterized protein
EOG091202OD	APLA007924	Agrilus planipennis	Putative uncharacterized protein
EOG091202OD	TC000466	Tribolium castaneum	Putative uncharacterized protein
EOG091202OD	LDEC007315	Leptinotarsa decemlineata	Putative uncharacterized protein
EOG091202OD	YQE_08504	Dendroctonus ponderosae	Putative uncharacterized protein
EOG091202OE	OTAU002016	Onthophagus taurus	Putative uncharacterized protein
EOG091202OE	AGLA007309	Anoplophora glabripennis	Putative uncharacterized protein
EOG091202OE	APLA000466	Agrilus planipennis	Putative uncharacterized protein
EOG091202OE	TC005738	Tribolium castaneum	Putative uncharacterized protein
EOG091202OE	LDEC004936	Leptinotarsa decemlineata	Putative uncharacterized protein
EOG091202OE	YQE_01720	Dendroctonus ponderosae	Putative uncharacterized protein
EOG091202OJ	OTAU002463	Onthophagus taurus	Putative uncharacterized protein
EOG091202OJ	AGLA017141	Anoplophora glabripennis	Putative uncharacterized protein
EOG091202OJ	APLA003387	Agrilus planipennis	Putative uncharacterized protein
EOG091202OJ	TC002647	Tribolium castaneum	Putative uncharacterized protein
EOG091202OJ	LDEC014537	Leptinotarsa decemlineata	Putative uncharacterized protein
EOG091202OJ	YQE_04539	Dendroctonus ponderosae	Putative uncharacterized protein
EOG091202OL	OTAU009480	Onthophagus taurus	Putative uncharacterized protein
EOG091202OL	AGLA015075	Anoplophora glabripennis	Putative uncharacterized protein
EOG091202OL	APLA010236	Agrilus planipennis	Putative uncharacterized protein
EOG091202OL	TC015650	Tribolium castaneum	Putative uncharacterized protein
EOG091202OL	LDEC005618	Leptinotarsa decemlineata	Putative uncharacterized protein
EOG091202OL	YQE_09380	Dendroctonus ponderosae	Putative uncharacterized protein
EOG091202OM	OTAU001242	Onthophagus taurus	Putative uncharacterized protein
EOG091202OM	AGLA007602	Anoplophora glabripennis	Putative uncharacterized protein
EOG091202OM	APLA003670	Agrilus planipennis	Putative uncharacterized protein
EOG091202OM	TC001754	Tribolium castaneum	Putative uncharacterized protein
EOG091202OM	LDEC015735	Leptinotarsa decemlineata	Putative uncharacterized protein
EOG091202OM	YQE_09130	Dendroctonus ponderosae	Putative uncharacterized protein
EOG091202OO	OTAU007494	Onthophagus taurus	Putative uncharacterized protein
EOG091202OO	AGLA012745	Anoplophora glabripennis	Putative uncharacterized protein
EOG091202OO	APLA002564	Agrilus planipennis	Putative uncharacterized protein
EOG091202OO	TC006113	Tribolium castaneum	Putative uncharacterized protein
EOG091202OO	LDEC001866	Leptinotarsa decemlineata	Putative uncharacterized protein
EOG091202OO	YQE_08381	Dendroctonus ponderosae	Putative uncharacterized protein
EOG091202OP	OTAU004324	Onthophagus taurus	Putative uncharacterized protein
EOG091202OP	AGLA015681	Anoplophora glabripennis	Putative uncharacterized protein
EOG091202OP	APLA010449	Agrilus planipennis	Putative uncharacterized protein
EOG091202OP	TC005166	Tribolium castaneum	Putative uncharacterized protein
EOG091202OP	LDEC003523	Leptinotarsa decemlineata	Putative uncharacterized protein
EOG091202OP	YQE_09301	Dendroctonus ponderosae	Putative uncharacterized protein
EOG091202OQ	OTAU001698	Onthophagus taurus	Putative uncharacterized protein
EOG091202OQ	AGLA010219	Anoplophora glabripennis	Putative uncharacterized protein
EOG091202OQ	APLA012070	Agrilus planipennis	Putative uncharacterized protein
EOG091202OQ	TC005306	Tribolium castaneum	Putative uncharacterized protein
EOG091202OQ	LDEC002970	Leptinotarsa decemlineata	Putative uncharacterized protein
EOG091202OQ	YQE_12050	Dendroctonus ponderosae	Putative uncharacterized protein
EOG091202OR	OTAU000516	Onthophagus taurus	Putative uncharacterized protein
EOG091202OR	AGLA020873	Anoplophora glabripennis	Putative uncharacterized protein
EOG091202OR	APLA010418	Agrilus planipennis	Putative uncharacterized protein
EOG091202OR	TC012468	Tribolium castaneum	Putative uncharacterized protein
EOG091202OR	LDEC020998	Leptinotarsa decemlineata	Putative uncharacterized protein
EOG091202OR	YQE_01685	Dendroctonus ponderosae	Putative uncharacterized protein
EOG091202OS	OTAU004914	Onthophagus taurus	Branched-chain-amino-acid aminotransferase
EOG091202OS	AGLA012813	Anoplophora glabripennis	Branched-chain-amino-acid aminotransferase
EOG091202OS	APLA006327	Agrilus planipennis	Branched-chain-amino-acid aminotransferase
EOG091202OS	TC000403	Tribolium castaneum	Branched-chain-amino-acid aminotransferase
EOG091202OS	LDEC019699	Leptinotarsa decemlineata	Branched-chain-amino-acid aminotransferase
EOG091202OS	YQE_01599	Dendroctonus ponderosae	Branched-chain-amino-acid aminotransferase
EOG091202OU	OTAU003884	Onthophagus taurus	Putative uncharacterized protein
EOG091202OU	AGLA011818	Anoplophora glabripennis	Putative uncharacterized protein
EOG091202OU	APLA014832	Agrilus planipennis	Putative uncharacterized protein
EOG091202OU	TC009816	Tribolium castaneum	Putative uncharacterized protein
EOG091202OU	LDEC016657	Leptinotarsa decemlineata	Putative uncharacterized protein

EOG091202OU	YQE_04093	Dendroctonus ponderosae	Putative uncharacterized protein
EOG091202OW	OTAU016814	Onthophagus taurus	Putative uncharacterized protein
EOG091202OW	AGLA018407	Anoplophora glabripennis	Putative uncharacterized protein
EOG091202OW	APLA012998	Agrilus planipennis	Putative uncharacterized protein
EOG091202OW	TC011228	Tribolium castaneum	Putative uncharacterized protein
EOG091202OW	LDEC012525	Leptinotarsa decemlineata	Putative uncharacterized protein
EOG091202OW	YQE_08467	Dendroctonus ponderosae	Putative uncharacterized protein
EOG091202OX	OTAU011394	Onthophagus taurus	Putative uncharacterized protein
EOG091202OX	AGLA004843	Anoplophora glabripennis	Putative uncharacterized protein
EOG091202OX	APLA014306	Agrilus planipennis	Putative uncharacterized protein
EOG091202OX	TC002912	Tribolium castaneum	Putative uncharacterized protein
EOG091202OX	LDEC020641	Leptinotarsa decemlineata	Putative uncharacterized protein
EOG091202OX	YQE_11497	Dendroctonus ponderosae	Putative uncharacterized protein
EOG091202OZ	OTAU004428	Onthophagus taurus	None
EOG091202OZ	AGLA000741	Anoplophora glabripennis	None
EOG091202OZ	APLA006560	Agrilus planipennis	None
EOG091202OZ	TC032824	Tribolium castaneum	None
EOG091202OZ	LDEC005099	Leptinotarsa decemlineata	None
EOG091202OZ	YQE_11446	Dendroctonus ponderosae	None
EOG091202P3	OTAU013031	Onthophagus taurus	Putative uncharacterized protein
EOG091202P3	AGLA003889	Anoplophora glabripennis	Putative uncharacterized protein
EOG091202P3	APLA000591	Agrilus planipennis	Putative uncharacterized protein
EOG091202P3	TC006055	Tribolium castaneum	Putative uncharacterized protein
EOG091202P3	LDEC003627	Leptinotarsa decemlineata	Putative uncharacterized protein
EOG091202P3	YQE_06369	Dendroctonus ponderosae	Putative uncharacterized protein
EOG091202P4	OTAU002880	Onthophagus taurus	Putative uncharacterized protein
EOG091202P4	AGLA021975	Anoplophora glabripennis	Putative uncharacterized protein
EOG091202P4	APLA014833	Agrilus planipennis	Putative uncharacterized protein
EOG091202P4	TC009147	Tribolium castaneum	Putative uncharacterized protein
EOG091202P4	LDEC002582	Leptinotarsa decemlineata	Putative uncharacterized protein
EOG091202P4	YQE_03544	Dendroctonus ponderosae	Putative uncharacterized protein
EOG091202P5	OTAU014516	Onthophagus taurus	Putative uncharacterized protein
EOG091202P5	AGLA010178	Anoplophora glabripennis	Putative uncharacterized protein
EOG091202P5	APLA012844	Agrilus planipennis	Putative uncharacterized protein
EOG091202P5	TC015996	Tribolium castaneum	Putative uncharacterized protein
EOG091202P5	LDEC006790	Leptinotarsa decemlineata	Putative uncharacterized protein
EOG091202P5	YQE_05767	Dendroctonus ponderosae	Putative uncharacterized protein
EOG091202P7	OTAU014260	Onthophagus taurus	Diphthamide biosynthesis protein 1
EOG091202P7	AGLA015237	Anoplophora glabripennis	Diphthamide biosynthesis protein 1
EOG091202P7	APLA012901	Agrilus planipennis	Diphthamide biosynthesis protein 1
EOG091202P7	TC000051	Tribolium castaneum	Diphthamide biosynthesis protein 1
EOG091202P7	LDEC005009	Leptinotarsa decemlineata	Diphthamide biosynthesis protein 1
EOG091202P7	YQE_11855	Dendroctonus ponderosae	Diphthamide biosynthesis protein 1
EOG091202P9	OTAU010914	Onthophagus taurus	Serine/threonine-protein phosphatase
EOG091202P9	AGLA013734	Anoplophora glabripennis	Serine/threonine-protein phosphatase
EOG091202P9	APLA013081	Agrilus planipennis	Serine/threonine-protein phosphatase
EOG091202P9	TC015726	Tribolium castaneum	Serine/threonine-protein phosphatase
EOG091202P9	LDEC005634	Leptinotarsa decemlineata	Serine/threonine-protein phosphatase
EOG091202P9	YQE_11869	Dendroctonus ponderosae	Serine/threonine-protein phosphatase
EOG091202PD	OTAU009129	Onthophagus taurus	Putative uncharacterized protein
EOG091202PD	AGLA011124	Anoplophora glabripennis	Putative uncharacterized protein
EOG091202PD	APLA003093	Agrilus planipennis	Putative uncharacterized protein
EOG091202PD	TC014327	Tribolium castaneum	Putative uncharacterized protein
EOG091202PD	LDEC018083	Leptinotarsa decemlineata	Putative uncharacterized protein
EOG091202PD	YQE_07146	Dendroctonus ponderosae	Putative uncharacterized protein
EOG091202PG	OTAU012889	Onthophagus taurus	Fructose-bisphosphate aldolase
EOG091202PG	AGLA005724	Anoplophora glabripennis	Fructose-bisphosphate aldolase
EOG091202PG	APLA000565	Agrilus planipennis	Fructose-bisphosphate aldolase
EOG091202PG	TC014998	Tribolium castaneum	Fructose-bisphosphate aldolase
EOG091202PG	LDEC010962	Leptinotarsa decemlineata	Fructose-bisphosphate aldolase
EOG091202PG	YQE_11479	Dendroctonus ponderosae	Fructose-bisphosphate aldolase
EOG091202PH	OTAU006129	Onthophagus taurus	Putative uncharacterized protein
EOG091202PH	AGLA020902	Anoplophora glabripennis	Putative uncharacterized protein
EOG091202PH	APLA011615	Agrilus planipennis	Putative uncharacterized protein
EOG091202PH	TC011295	Tribolium castaneum	Putative uncharacterized protein
EOG091202PH	LDEC007384	Leptinotarsa decemlineata	Putative uncharacterized protein
EOG091202PH	YQE_11205	Dendroctonus ponderosae	Putative uncharacterized protein

EOG091202PI	OTAU010953	Onthophagus taurus	Putative uncharacterized protein
EOG091202PI	AGLA012729	Anoplophora glabripennis	Putative uncharacterized protein
EOG091202PI	APLA007317	Agrilus planipennis	Putative uncharacterized protein
EOG091202PI	TC003389	Tribolium castaneum	Putative uncharacterized protein
EOG091202PI	LDEC020106	Leptinotarsa decemlineata	Putative uncharacterized protein
EOG091202PI	YQE_07394	Dendroctonus ponderosae	Putative uncharacterized protein
EOG091202PK	OTAU003773	Onthophagus taurus	Putative uncharacterized protein
EOG091202PK	AGLA011815	Anoplophora glabripennis	Putative uncharacterized protein
EOG091202PK	APLA004586	Agrilus planipennis	Putative uncharacterized protein
EOG091202PK	TC009340	Tribolium castaneum	Putative uncharacterized protein
EOG091202PK	LDEC016654	Leptinotarsa decemlineata	Putative uncharacterized protein
EOG091202PK	YQE_05440	Dendroctonus ponderosae	Putative uncharacterized protein
EOG091202PM	OTAU004987	Onthophagus taurus	Putative uncharacterized protein
EOG091202PM	AGLA013174	Anoplophora glabripennis	Putative uncharacterized protein
EOG091202PM	APLA004332	Agrilus planipennis	Putative uncharacterized protein
EOG091202PM	TC011240	Tribolium castaneum	Putative uncharacterized protein
EOG091202PM	LDEC006888	Leptinotarsa decemlineata	Putative uncharacterized protein
EOG091202PM	YQE_12057	Dendroctonus ponderosae	Putative uncharacterized protein
EOG091202PN	OTAU005027	Onthophagus taurus	"NADPH:adrenodoxin oxidoreductase, mitochondrial "
EOG091202PN	AGLA008844	Anoplophora glabripennis	"NADPH:adrenodoxin oxidoreductase, mitochondrial "
EOG091202PN	APLA007957	Agrilus planipennis	"NADPH:adrenodoxin oxidoreductase, mitochondrial "
EOG091202PN	TC014987	Tribolium castaneum	"NADPH:adrenodoxin oxidoreductase, mitochondrial "
EOG091202PN	LDEC019238	Leptinotarsa decemlineata	"NADPH:adrenodoxin oxidoreductase, mitochondrial "
EOG091202PN	YQE_11610	Dendroctonus ponderosae	"NADPH:adrenodoxin oxidoreductase, mitochondrial "
EOG091202PR	OTAU016865	Onthophagus taurus	Putative uncharacterized protein
EOG091202PR	AGLA001924	Anoplophora glabripennis	Putative uncharacterized protein
EOG091202PR	APLA007859	Agrilus planipennis	Putative uncharacterized protein
EOG091202PR	TC004861	Tribolium castaneum	Putative uncharacterized protein
EOG091202PR	LDEC000869	Leptinotarsa decemlineata	Putative uncharacterized protein
EOG091202PR	YQE_06828	Dendroctonus ponderosae	Putative uncharacterized protein
EOG091202PT	OTAU005864	Onthophagus taurus	Putative uncharacterized protein
EOG091202PT	AGLA010920	Anoplophora glabripennis	Putative uncharacterized protein
EOG091202PT	APLA003593	Agrilus planipennis	Putative uncharacterized protein
EOG091202PT	TC014106	Tribolium castaneum	Putative uncharacterized protein
EOG091202PT	LDEC006501	Leptinotarsa decemlineata	Putative uncharacterized protein
EOG091202PT	YQE_02073	Dendroctonus ponderosae	Putative uncharacterized protein
EOG091202PU	OTAU003838	Onthophagus taurus	Putative uncharacterized protein
EOG091202PU	AGLA017744	Anoplophora glabripennis	Putative uncharacterized protein
EOG091202PU	APLA010172	Agrilus planipennis	Putative uncharacterized protein
EOG091202PU	TC008879	Tribolium castaneum	Putative uncharacterized protein
EOG091202PU	LDEC001216	Leptinotarsa decemlineata	Putative uncharacterized protein
EOG091202PU	YQE_04081	Dendroctonus ponderosae	Putative uncharacterized protein
EOG091202PV	OTAU001360	Onthophagus taurus	Putative uncharacterized protein
EOG091202PV	AGLA000384	Anoplophora glabripennis	Putative uncharacterized protein
EOG091202PV	APLA010010	Agrilus planipennis	Putative uncharacterized protein
EOG091202PV	TC004723	Tribolium castaneum	Putative uncharacterized protein
EOG091202PV	LDEC004210	Leptinotarsa decemlineata	Putative uncharacterized protein
EOG091202PV	YQE_06669	Dendroctonus ponderosae	Putative uncharacterized protein
EOG091202Q1	OTAU002868	Onthophagus taurus	Putative uncharacterized protein
EOG091202Q1	AGLA013528	Anoplophora glabripennis	Putative uncharacterized protein
EOG091202Q1	APLA005606	Agrilus planipennis	Putative uncharacterized protein
EOG091202Q1	TC013594	Tribolium castaneum	Putative uncharacterized protein
EOG091202Q1	LDEC017479	Leptinotarsa decemlineata	Putative uncharacterized protein
EOG091202Q1	YQE_09204	Dendroctonus ponderosae	Putative uncharacterized protein
EOG091202Q2	OTAU004392	Onthophagus taurus	Shifted
EOG091202Q2	AGLA002183	Anoplophora glabripennis	Shifted
EOG091202Q2	APLA013928	Agrilus planipennis	Shifted
EOG091202Q2	TC001979	Tribolium castaneum	Shifted
EOG091202Q2	LDEC011928	Leptinotarsa decemlineata	Shifted
EOG091202Q2	YQE_06618	Dendroctonus ponderosae	Shifted
EOG091202Q8	OTAU004321	Onthophagus taurus	Putative uncharacterized protein

EOG091202Q8	AGLA004529	Anoplophora glabripennis	Putative uncharacterized protein
EOG091202Q8	APLA006789	Agrilus planipennis	Putative uncharacterized protein
EOG091202Q8	TC001461	Tribolium castaneum	Putative uncharacterized protein
EOG091202Q8	LDEC001542	Leptinotarsa decemlineata	Putative uncharacterized protein
EOG091202Q8	YQE_07236	Dendroctonus ponderosae	Putative uncharacterized protein
EOG091202Q9	OTAU007785	Onthophagus taurus	transferase activity
EOG091202Q9	AGLA006304	Anoplophora glabripennis	transferase activity
EOG091202Q9	APLA001649	Agrilus planipennis	transferase activity
EOG091202Q9	TC031204	Tribolium castaneum	transferase activity
EOG091202Q9	LDEC002089	Leptinotarsa decemlineata	transferase activity
EOG091202Q9	YQE_11925	Dendroctonus ponderosae	transferase activity
EOG091202QB	OTAU012244	Onthophagus taurus	Vasa
EOG091202QB	AGLA020799	Anoplophora glabripennis	Vasa
EOG091202QB	APLA004643	Agrilus planipennis	Vasa
EOG091202QB	TC010103	Tribolium castaneum	Vasa
EOG091202QB	LDEC018398	Leptinotarsa decemlineata	Vasa
EOG091202QB	YQE_04121	Dendroctonus ponderosae	Vasa
EOG091202QC	OTAU009339	Onthophagus taurus	Putative uncharacterized protein
EOG091202QC	AGLA012607	Anoplophora glabripennis	Putative uncharacterized protein
EOG091202QC	APLA012893	Agrilus planipennis	Putative uncharacterized protein
EOG091202QC	TC013486	Tribolium castaneum	Putative uncharacterized protein
EOG091202QC	LDEC011393	Leptinotarsa decemlineata	Putative uncharacterized protein
EOG091202QC	YQE_08252	Dendroctonus ponderosae	Putative uncharacterized protein
EOG091202QD	OTAU010314	Onthophagus taurus	None
EOG091202QD	AGLA004070	Anoplophora glabripennis	None
EOG091202QD	APLA011230	Agrilus planipennis	None
EOG091202QD	TC033642	Tribolium castaneum	None
EOG091202QD	LDEC012803	Leptinotarsa decemlineata	None
EOG091202QD	YQE_09920	Dendroctonus ponderosae	None
EOG091202QF	OTAU005476	Onthophagus taurus	Putative uncharacterized protein
EOG091202QF	AGLA010817	Anoplophora glabripennis	Putative uncharacterized protein
EOG091202QF	APLA003602	Agrilus planipennis	Putative uncharacterized protein
EOG091202QF	TC014048	Tribolium castaneum	Putative uncharacterized protein
EOG091202QF	LDEC004425	Leptinotarsa decemlineata	Putative uncharacterized protein
EOG091202QF	YQE_06328	Dendroctonus ponderosae	Putative uncharacterized protein
EOG091202QG	OTAU000387	Onthophagus taurus	Putative uncharacterized protein
EOG091202QG	AGLA015999	Anoplophora glabripennis	Putative uncharacterized protein
EOG091202QG	APLA000184	Agrilus planipennis	Putative uncharacterized protein
EOG091202QG	TC012448	Tribolium castaneum	Putative uncharacterized protein
EOG091202QG	LDEC021833	Leptinotarsa decemlineata	Putative uncharacterized protein
EOG091202QG	YQE_01658	Dendroctonus ponderosae	Putative uncharacterized protein
EOG091202QL	OTAU000979	Onthophagus taurus	Putative uncharacterized protein
EOG091202QL	AGLA002977	Anoplophora glabripennis	Putative uncharacterized protein
EOG091202QL	APLA007688	Agrilus planipennis	Putative uncharacterized protein
EOG091202QL	TC004235	Tribolium castaneum	Putative uncharacterized protein
EOG091202QL	LDEC006238	Leptinotarsa decemlineata	Putative uncharacterized protein
EOG091202QL	YQE_07074	Dendroctonus ponderosae	Putative uncharacterized protein
EOG091202QP	OTAU001415	Onthophagus taurus	Putative uncharacterized protein
EOG091202QP	AGLA015674	Anoplophora glabripennis	Putative uncharacterized protein
EOG091202QP	APLA005631	Agrilus planipennis	Putative uncharacterized protein
EOG091202QP	TC001864	Tribolium castaneum	Putative uncharacterized protein
EOG091202QP	LDEC012587	Leptinotarsa decemlineata	Putative uncharacterized protein
EOG091202QP	YQE_09153	Dendroctonus ponderosae	Putative uncharacterized protein
EOG091202QQ	OTAU000625	Onthophagus taurus	Putative uncharacterized protein
EOG091202QQ	AGLA009784	Anoplophora glabripennis	Putative uncharacterized protein
EOG091202QQ	APLA002124	Agrilus planipennis	Putative uncharacterized protein
EOG091202QQ	TC011816	Tribolium castaneum	Putative uncharacterized protein
EOG091202QQ	LDEC003827	Leptinotarsa decemlineata	Putative uncharacterized protein
EOG091202QQ	YQE_12863	Dendroctonus ponderosae	Putative uncharacterized protein
EOG091202QR	OTAU000739	Onthophagus taurus	Putative uncharacterized protein
EOG091202QR	AGLA017379	Anoplophora glabripennis	Putative uncharacterized protein
EOG091202QR	APLA002375	Agrilus planipennis	Putative uncharacterized protein
EOG091202QR	TC012587	Tribolium castaneum	Putative uncharacterized protein
EOG091202QR	LDEC004087	Leptinotarsa decemlineata	Putative uncharacterized protein
EOG091202QR	YQE_12872	Dendroctonus ponderosae	Putative uncharacterized protein
EOG091202QT	OTAU010311	Onthophagus taurus	Regucalcin
EOG091202QT	AGLA006488	Anoplophora glabripennis	Regucalcin

EOG091202QT	APLA011225	Agrilus planipennis	Regucalcin
EOG091202QT	TC009114	Tribolium castaneum	Regucalcin
EOG091202QT	LDEC002196	Leptinotarsa decemlineata	Regucalcin
EOG091202QT	YQE_10571	Dendroctonus ponderosae	Regucalcin
EOG091202QV	OTAU005807	Onthophagus taurus	4-hydroxyphenylpyruvate dioxygenase
EOG091202QV	AGLA002298	Anoplophora glabripennis	4-hydroxyphenylpyruvate dioxygenase
EOG091202QV	APLA003951	Agrilus planipennis	4-hydroxyphenylpyruvate dioxygenase
EOG091202QV	TC012702	Tribolium castaneum	4-hydroxyphenylpyruvate dioxygenase
EOG091202QV	LDEC004144	Leptinotarsa decemlineata	4-hydroxyphenylpyruvate dioxygenase
EOG091202QV	YQE_05504	Dendroctonus ponderosae	4-hydroxyphenylpyruvate dioxygenase
EOG091202QW	OTAU004523	Onthophagus taurus	Putative uncharacterized protein
EOG091202QW	AGLA016061	Anoplophora glabripennis	Putative uncharacterized protein
EOG091202QW	APLA004041	Agrilus planipennis	Putative uncharacterized protein
EOG091202QW	TC012611	Tribolium castaneum	Putative uncharacterized protein
EOG091202QW	LDEC007601	Leptinotarsa decemlineata	Putative uncharacterized protein
EOG091202QW	YQE_07319	Dendroctonus ponderosae	Putative uncharacterized protein
EOG091202QY	OTAU004487	Onthophagus taurus	Putative uncharacterized protein
EOG091202QY	AGLA021668	Anoplophora glabripennis	Putative uncharacterized protein
EOG091202QY	APLA013645	Agrilus planipennis	Putative uncharacterized protein
EOG091202QY	TC008898	Tribolium castaneum	Putative uncharacterized protein
EOG091202QY	LDEC019354	Leptinotarsa decemlineata	Putative uncharacterized protein
EOG091202QY	YQE_05409	Dendroctonus ponderosae	Putative uncharacterized protein
EOG091202R2	OTAU000184	Onthophagus taurus	ATP binding
EOG091202R2	AGLA006361	Anoplophora glabripennis	ATP binding
EOG091202R2	APLA003031	Agrilus planipennis	ATP binding
EOG091202R2	TC033152	Tribolium castaneum	ATP binding
EOG091202R2	LDEC002616	Leptinotarsa decemlineata	ATP binding
EOG091202R2	YQE_08321	Dendroctonus ponderosae	ATP binding
EOG091202R7	OTAU008766	Onthophagus taurus	None
EOG091202R7	AGLA015571	Anoplophora glabripennis	None
EOG091202R7	APLA002589	Agrilus planipennis	None
EOG091202R7	TC030911	Tribolium castaneum	None
EOG091202R7	LDEC005620	Leptinotarsa decemlineata	None
EOG091202R7	YQE_11753	Dendroctonus ponderosae	None
EOG091202R9	OTAU009498	Onthophagus taurus	Putative uncharacterized protein
EOG091202R9	AGLA010435	Anoplophora glabripennis	Putative uncharacterized protein
EOG091202R9	APLA005575	Agrilus planipennis	Putative uncharacterized protein
EOG091202R9	TC015824	Tribolium castaneum	Putative uncharacterized protein
EOG091202R9	LDEC013163	Leptinotarsa decemlineata	Putative uncharacterized protein
EOG091202R9	YQE_02145	Dendroctonus ponderosae	Putative uncharacterized protein
EOG091202RA	OTAU011296	Onthophagus taurus	None
EOG091202RA	AGLA020870	Anoplophora glabripennis	None
EOG091202RA	APLA004869	Agrilus planipennis	None
EOG091202RA	TC033264	Tribolium castaneum	None
EOG091202RA	LDEC001124	Leptinotarsa decemlineata	None
EOG091202RA	YQE_08237	Dendroctonus ponderosae	None
EOG091202RB	OTAU000282	Onthophagus taurus	None
EOG091202RB	AGLA009766	Anoplophora glabripennis	None
EOG091202RB	APLA007360	Agrilus planipennis	None
EOG091202RB	TC033104	Tribolium castaneum	None
EOG091202RB	LDEC016370	Leptinotarsa decemlineata	None
EOG091202RB	YQE_04429	Dendroctonus ponderosae	None
EOG091202RC	OTAU010532	Onthophagus taurus	"LIM homeobox transcription factor 1, beta "
EOG091202RC	AGLA002059	Anoplophora glabripennis	"LIM homeobox transcription factor 1, beta "
EOG091202RC	APLA000805	Agrilus planipennis	"LIM homeobox transcription factor 1, beta "
EOG091202RC	TC001291	Tribolium castaneum	"LIM homeobox transcription factor 1, beta "
EOG091202RC	LDEC019709	Leptinotarsa decemlineata	"LIM homeobox transcription factor 1, beta "
EOG091202RC	YQE_03691	Dendroctonus ponderosae	"LIM homeobox transcription factor 1, beta "
EOG091202RG	OTAU015219	Onthophagus taurus	Putative uncharacterized protein
EOG091202RG	AGLA003052	Anoplophora glabripennis	Putative uncharacterized protein
EOG091202RG	APLA004203	Agrilus planipennis	Putative uncharacterized protein
EOG091202RG	TC008381	Tribolium castaneum	Putative uncharacterized protein
EOG091202RG	LDEC006462	Leptinotarsa decemlineata	Putative uncharacterized protein
EOG091202RG	YQE_07533	Dendroctonus ponderosae	Putative uncharacterized protein
EOG091202RI	OTAU003137	Onthophagus taurus	Putative uncharacterized protein
EOG091202RI	AGLA017698	Anoplophora glabripennis	Putative uncharacterized protein
EOG091202RI	APLA013806	Agrilus planipennis	Putative uncharacterized protein

EOG091202RI	TC000125	Tribolium castaneum	Putative uncharacterized protein
EOG091202RI	LDEC005022	Leptinotarsa decemlineata	Putative uncharacterized protein
EOG091202RI	YQE_05190	Dendroctonus ponderosae	Putative uncharacterized protein
EOG091202RJ	OTAU004901	Onthophagus taurus	None
EOG091202RJ	AGLA007933	Anoplophora glabripennis	None
EOG091202RJ	APLA013536	Agrilus planipennis	None
EOG091202RJ	TC034367	Tribolium castaneum	None
EOG091202RJ	LDEC010236	Leptinotarsa decemlineata	None
EOG091202RJ	YQE_12861	Dendroctonus ponderosae	None
EOG091202RL	OTAU001391	Onthophagus taurus	Putative uncharacterized protein
EOG091202RL	AGLA004571	Anoplophora glabripennis	Putative uncharacterized protein
EOG091202RL	APLA010658	Agrilus planipennis	Putative uncharacterized protein
EOG091202RL	TC001773	Tribolium castaneum	Putative uncharacterized protein
EOG091202RL	LDEC001565	Leptinotarsa decemlineata	Putative uncharacterized protein
EOG091202RL	YQE_02387	Dendroctonus ponderosae	Putative uncharacterized protein
EOG091202RQ	OTAU000876	Onthophagus taurus	Putative uncharacterized protein
EOG091202RQ	AGLA000157	Anoplophora glabripennis	Putative uncharacterized protein
EOG091202RQ	APLA001266	Agrilus planipennis	Putative uncharacterized protein
EOG091202RQ	TC004824	Tribolium castaneum	Putative uncharacterized protein
EOG091202RQ	LDEC018558	Leptinotarsa decemlineata	Putative uncharacterized protein
EOG091202RQ	YQE_08482	Dendroctonus ponderosae	Putative uncharacterized protein
EOG091202RR	OTAU011854	Onthophagus taurus	Putative uncharacterized protein
EOG091202RR	AGLA003157	Anoplophora glabripennis	Putative uncharacterized protein
EOG091202RR	APLA013049	Agrilus planipennis	Putative uncharacterized protein
EOG091202RR	TC012278	Tribolium castaneum	Putative uncharacterized protein
EOG091202RR	LDEC007614	Leptinotarsa decemlineata	Putative uncharacterized protein
EOG091202RR	YQE_10054	Dendroctonus ponderosae	Putative uncharacterized protein
EOG091202RS	OTAU004861	Onthophagus taurus	Putative uncharacterized protein
EOG091202RS	AGLA001670	Anoplophora glabripennis	Putative uncharacterized protein
EOG091202RS	APLA013151	Agrilus planipennis	Putative uncharacterized protein
EOG091202RS	TC005539	Tribolium castaneum	Putative uncharacterized protein
EOG091202RS	LDEC004901	Leptinotarsa decemlineata	Putative uncharacterized protein
EOG091202RS	YQE_12033	Dendroctonus ponderosae	Putative uncharacterized protein
EOG091202RU	OTAU001033	Onthophagus taurus	Putative uncharacterized protein
EOG091202RU	AGLA018003	Anoplophora glabripennis	Putative uncharacterized protein
EOG091202RU	APLA008024	Agrilus planipennis	Putative uncharacterized protein
EOG091202RU	TC000688	Tribolium castaneum	Putative uncharacterized protein
EOG091202RU	LDEC006133	Leptinotarsa decemlineata	Putative uncharacterized protein
EOG091202RU	YQE_09604	Dendroctonus ponderosae	Putative uncharacterized protein
EOG091202RV	OTAU006821	Onthophagus taurus	Putative uncharacterized protein
EOG091202RV	AGLA019023	Anoplophora glabripennis	Putative uncharacterized protein
EOG091202RV	APLA006667	Agrilus planipennis	Putative uncharacterized protein
EOG091202RV	TC002137	Tribolium castaneum	Putative uncharacterized protein
EOG091202RV	LDEC014911	Leptinotarsa decemlineata	Putative uncharacterized protein
EOG091202RV	YQE_12313	Dendroctonus ponderosae	Putative uncharacterized protein
EOG091202RW	OTAU003351	Onthophagus taurus	Putative uncharacterized protein
EOG091202RW	AGLA004540	Anoplophora glabripennis	Putative uncharacterized protein
EOG091202RW	APLA009990	Agrilus planipennis	Putative uncharacterized protein
EOG091202RW	TC010778	Tribolium castaneum	Putative uncharacterized protein
EOG091202RW	LDEC003164	Leptinotarsa decemlineata	Putative uncharacterized protein
EOG091202RW	YQE_09291	Dendroctonus ponderosae	Putative uncharacterized protein
EOG091202RY	OTAU010496	Onthophagus taurus	Putative uncharacterized protein
EOG091202RY	AGLA010778	Anoplophora glabripennis	Putative uncharacterized protein
EOG091202RY	APLA012002	Agrilus planipennis	Putative uncharacterized protein
EOG091202RY	TC010372	Tribolium castaneum	Putative uncharacterized protein
EOG091202RY	LDEC016716	Leptinotarsa decemlineata	Putative uncharacterized protein
EOG091202RY	YQE_07834	Dendroctonus ponderosae	Putative uncharacterized protein
EOG091202RZ	OTAU003098	Onthophagus taurus	substrate-specific transmembrane transporter activity
EOG091202RZ	AGLA009154	Anoplophora glabripennis	substrate-specific transmembrane transporter activity
EOG091202RZ	APLA006649	Agrilus planipennis	substrate-specific transmembrane transporter activity
EOG091202RZ	TC034087	Tribolium castaneum	substrate-specific transmembrane transporter activity
EOG091202RZ	LDEC010792	Leptinotarsa decemlineata	substrate-specific transmembrane transporter activity
EOG091202RZ	YQE_09309	Dendroctonus ponderosae	substrate-specific transmembrane transporter activity
EOG091202S0	OTAU007919	Onthophagus taurus	None
EOG091202S0	AGLA003131	Anoplophora glabripennis	None
EOG091202S0	APLA002353	Agrilus planipennis	None
EOG091202S0	TC034435	Tribolium castaneum	None

EOG091202S0	LDEC013692	Leptinotarsa decemlineata	None
EOG091202S0	YQE_04393	Dendroctonus ponderosae	None
EOG091202S3	OTAU007646	Onthophagus taurus	Putative uncharacterized protein
EOG091202S3	AGLA015619	Anoplophora glabripennis	Putative uncharacterized protein
EOG091202S3	APLA003429	Agrilus planipennis	Putative uncharacterized protein
EOG091202S3	TC009832	Tribolium castaneum	Putative uncharacterized protein
EOG091202S3	LDEC008965	Leptinotarsa decemlineata	Putative uncharacterized protein
EOG091202S3	YQE_03536	Dendroctonus ponderosae	Putative uncharacterized protein
EOG091202S4	OTAU002193	Onthophagus taurus	Putative uncharacterized protein
EOG091202S4	AGLA020514	Anoplophora glabripennis	Putative uncharacterized protein
EOG091202S4	APLA005998	Agrilus planipennis	Putative uncharacterized protein
EOG091202S4	TC002948	Tribolium castaneum	Putative uncharacterized protein
EOG091202S4	LDEC005049	Leptinotarsa decemlineata	Putative uncharacterized protein
EOG091202S4	YQE_04553	Dendroctonus ponderosae	Putative uncharacterized protein
EOG091202S5	OTAU000663	Onthophagus taurus	Putative uncharacterized protein
EOG091202S5	AGLA012956	Anoplophora glabripennis	Putative uncharacterized protein
EOG091202S5	APLA006613	Agrilus planipennis	Putative uncharacterized protein
EOG091202S5	TC000081	Tribolium castaneum	Putative uncharacterized protein
EOG091202S5	LDEC003374	Leptinotarsa decemlineata	Putative uncharacterized protein
EOG091202S5	YQE_03734	Dendroctonus ponderosae	Putative uncharacterized protein
EOG091202S6	OTAU009544	Onthophagus taurus	AP-2
EOG091202S6	AGLA008267	Anoplophora glabripennis	AP-2
EOG091202S6	APLA001648	Agrilus planipennis	AP-2
EOG091202S6	TC009922	Tribolium castaneum	AP-2
EOG091202S6	LDEC004787	Leptinotarsa decemlineata	AP-2
EOG091202S6	YQE_10574	Dendroctonus ponderosae	AP-2
EOG091202S7	OTAU000133	Onthophagus taurus	Putative uncharacterized protein
EOG091202S7	AGLA007043	Anoplophora glabripennis	Putative uncharacterized protein
EOG091202S7	APLA008095	Agrilus planipennis	Putative uncharacterized protein
EOG091202S7	TC005074	Tribolium castaneum	Putative uncharacterized protein
EOG091202S7	LDEC010406	Leptinotarsa decemlineata	Putative uncharacterized protein
EOG091202S7	YQE_10638	Dendroctonus ponderosae	Putative uncharacterized protein
EOG091202S8	OTAU007841	Onthophagus taurus	Putative uncharacterized protein
EOG091202S8	AGLA017753	Anoplophora glabripennis	Putative uncharacterized protein
EOG091202S8	APLA011107	Agrilus planipennis	Putative uncharacterized protein
EOG091202S8	TC015912	Tribolium castaneum	Putative uncharacterized protein
EOG091202S8	LDEC001225	Leptinotarsa decemlineata	Putative uncharacterized protein
EOG091202S8	YQE_11820	Dendroctonus ponderosae	Putative uncharacterized protein
EOG091202S9	OTAU012706	Onthophagus taurus	Lethal Sp8
EOG091202S9	AGLA001943	Anoplophora glabripennis	Lethal Sp8
EOG091202S9	APLA007115	Agrilus planipennis	Lethal Sp8
EOG091202S9	TC011697	Tribolium castaneum	Lethal Sp8
EOG091202S9	LDEC008343	Leptinotarsa decemlineata	Lethal Sp8
EOG091202S9	YQE_04342	Dendroctonus ponderosae	Lethal Sp8
EOG091202SA	OTAU010628	Onthophagus taurus	Putative uncharacterized protein
EOG091202SA	AGLA019474	Anoplophora glabripennis	Putative uncharacterized protein
EOG091202SA	APLA012885	Agrilus planipennis	Putative uncharacterized protein
EOG091202SA	TC014410	Tribolium castaneum	Putative uncharacterized protein
EOG091202SA	LDEC017195	Leptinotarsa decemlineata	Putative uncharacterized protein
EOG091202SA	YQE_04865	Dendroctonus ponderosae	Putative uncharacterized protein
EOG091202SC	OTAU015736	Onthophagus taurus	nucleic acid binding
EOG091202SC	AGLA002522	Anoplophora glabripennis	nucleic acid binding
EOG091202SC	APLA000691	Agrilus planipennis	nucleic acid binding
EOG091202SC	TC031853	Tribolium castaneum	nucleic acid binding
EOG091202SC	LDEC009758	Leptinotarsa decemlineata	nucleic acid binding
EOG091202SC	YQE_06536	Dendroctonus ponderosae	nucleic acid binding
EOG091202SD	OTAU005349	Onthophagus taurus	Putative uncharacterized protein
EOG091202SD	AGLA008299	Anoplophora glabripennis	Putative uncharacterized protein
EOG091202SD	APLA012568	Agrilus planipennis	Putative uncharacterized protein
EOG091202SD	TC009347	Tribolium castaneum	Putative uncharacterized protein
EOG091202SD	LDEC001251	Leptinotarsa decemlineata	Putative uncharacterized protein
EOG091202SD	YQE_11304	Dendroctonus ponderosae	Putative uncharacterized protein
EOG091202SI	OTAU000667	Onthophagus taurus	Putative uncharacterized protein
EOG091202SI	AGLA017239	Anoplophora glabripennis	Putative uncharacterized protein
EOG091202SI	APLA001094	Agrilus planipennis	Putative uncharacterized protein
EOG091202SI	TC012725	Tribolium castaneum	Putative uncharacterized protein
EOG091202SI	LDEC014363	Leptinotarsa decemlineata	Putative uncharacterized protein

EOG091202SI	YQE_07603	Dendroctonus ponderosae	Putative uncharacterized protein
EOG091202SJ	OTAU012800	Onthophagus taurus	tropomyosin binding
EOG091202SJ	AGLA016268	Anoplophora glabripennis	tropomyosin binding
EOG091202SJ	APLA012021	Agrilus planipennis	tropomyosin binding
EOG091202SJ	TC031361	Tribolium castaneum	tropomyosin binding
EOG091202SJ	LDEC010351	Leptinotarsa decemlineata	tropomyosin binding
EOG091202SJ	YQE_06833	Dendroctonus ponderosae	tropomyosin binding
EOG091202SK	OTAU010911	Onthophagus taurus	Putative uncharacterized protein
EOG091202SK	AGLA017558	Anoplophora glabripennis	Putative uncharacterized protein
EOG091202SK	APLA001711	Agrilus planipennis	Putative uncharacterized protein
EOG091202SK	TC015856	Tribolium castaneum	Putative uncharacterized protein
EOG091202SK	LDEC009956	Leptinotarsa decemlineata	Putative uncharacterized protein
EOG091202SK	YQE_10817	Dendroctonus ponderosae	Putative uncharacterized protein
EOG091202SM	OTAU010925	Onthophagus taurus	Putative uncharacterized protein
EOG091202SM	AGLA014569	Anoplophora glabripennis	Putative uncharacterized protein
EOG091202SM	APLA003772	Agrilus planipennis	Putative uncharacterized protein
EOG091202SM	TC013994	Tribolium castaneum	Putative uncharacterized protein
EOG091202SM	LDEC003218	Leptinotarsa decemlineata	Putative uncharacterized protein
EOG091202SM	YQE_02024	Dendroctonus ponderosae	Putative uncharacterized protein
EOG091202SN	OTAU016127	Onthophagus taurus	Putative uncharacterized protein
EOG091202SN	AGLA013305	Anoplophora glabripennis	Putative uncharacterized protein
EOG091202SN	APLA006458	Agrilus planipennis	Putative uncharacterized protein
EOG091202SN	TC015607	Tribolium castaneum	Putative uncharacterized protein
EOG091202SN	LDEC019638	Leptinotarsa decemlineata	Putative uncharacterized protein
EOG091202SN	YQE_10162	Dendroctonus ponderosae	Putative uncharacterized protein
EOG091202SP	OTAU000324	Onthophagus taurus	None
EOG091202SP	AGLA004358	Anoplophora glabripennis	None
EOG091202SP	APLA002703	Agrilus planipennis	None
EOG091202SP	TC033275	Tribolium castaneum	None
EOG091202SP	LDEC002187	Leptinotarsa decemlineata	None
EOG091202SP	YQE_11363	Dendroctonus ponderosae	None
EOG091202SS	OTAU003585	Onthophagus taurus	Putative uncharacterized protein
EOG091202SS	AGLA002310	Anoplophora glabripennis	Putative uncharacterized protein
EOG091202SS	APLA003985	Agrilus planipennis	Putative uncharacterized protein
EOG091202SS	TC011953	Tribolium castaneum	Putative uncharacterized protein
EOG091202SS	LDEC004131	Leptinotarsa decemlineata	Putative uncharacterized protein
EOG091202SS	YQE_01992	Dendroctonus ponderosae	Putative uncharacterized protein
EOG091202SU	OTAU008431	Onthophagus taurus	TNF-receptor-associated factor 2
EOG091202SU	AGLA013816	Anoplophora glabripennis	TNF-receptor-associated factor 2
EOG091202SU	APLA003179	Agrilus planipennis	TNF-receptor-associated factor 2
EOG091202SU	TC007706	Tribolium castaneum	TNF-receptor-associated factor 2
EOG091202SU	LDEC009544	Leptinotarsa decemlineata	TNF-receptor-associated factor 2
EOG091202SU	YQE_09471	Dendroctonus ponderosae	TNF-receptor-associated factor 2
EOG091202SV	OTAU008396	Onthophagus taurus	Putative uncharacterized protein
EOG091202SV	AGLA001520	Anoplophora glabripennis	Putative uncharacterized protein
EOG091202SV	APLA007021	Agrilus planipennis	Putative uncharacterized protein
EOG091202SV	TC003812	Tribolium castaneum	Putative uncharacterized protein
EOG091202SV	LDEC013432	Leptinotarsa decemlineata	Putative uncharacterized protein
EOG091202SV	YQE_12366	Dendroctonus ponderosae	Putative uncharacterized protein
EOG091202SW	OTAU004726	Onthophagus taurus	Putative uncharacterized protein
EOG091202SW	AGLA007729	Anoplophora glabripennis	Putative uncharacterized protein
EOG091202SW	APLA008397	Agrilus planipennis	Putative uncharacterized protein
EOG091202SW	TC002911	Tribolium castaneum	Putative uncharacterized protein
EOG091202SW	LDEC010295	Leptinotarsa decemlineata	Putative uncharacterized protein
EOG091202SW	YQE_10219	Dendroctonus ponderosae	Putative uncharacterized protein
EOG091202SZ	OTAU001287	Onthophagus taurus	Putative uncharacterized protein
EOG091202SZ	AGLA002194	Anoplophora glabripennis	Putative uncharacterized protein
EOG091202SZ	APLA003560	Agrilus planipennis	Putative uncharacterized protein
EOG091202SZ	TC004145	Tribolium castaneum	Putative uncharacterized protein
EOG091202SZ	LDEC015873	Leptinotarsa decemlineata	Putative uncharacterized protein
EOG091202SZ	YQE_09097	Dendroctonus ponderosae	Putative uncharacterized protein
EOG091202T2	OTAU006580	Onthophagus taurus	Putative uncharacterized protein
EOG091202T2	AGLA011703	Anoplophora glabripennis	Putative uncharacterized protein
EOG091202T2	APLA005204	Agrilus planipennis	Putative uncharacterized protein
EOG091202T2	TC003627	Tribolium castaneum	Putative uncharacterized protein
EOG091202T2	LDEC013196	Leptinotarsa decemlineata	Putative uncharacterized protein
EOG091202T2	YQE_07459	Dendroctonus ponderosae	Putative uncharacterized protein

EOG091202T4	OTAU013794	Onthophagus taurus	Putative uncharacterized protein
EOG091202T4	AGLA001764	Anoplophora glabripennis	Putative uncharacterized protein
EOG091202T4	APLA011593	Agrilus planipennis	Putative uncharacterized protein
EOG091202T4	TC013552	Tribolium castaneum	Putative uncharacterized protein
EOG091202T4	LDEC006201	Leptinotarsa decemlineata	Putative uncharacterized protein
EOG091202T4	YQE_03976	Dendroctonus ponderosae	Putative uncharacterized protein
EOG091202T5	OTAU001784	Onthophagus taurus	Putative uncharacterized protein
EOG091202T5	AGLA019338	Anoplophora glabripennis	Putative uncharacterized protein
EOG091202T5	APLA004455	Agrilus planipennis	Putative uncharacterized protein
EOG091202T5	TC006344	Tribolium castaneum	Putative uncharacterized protein
EOG091202T5	LDEC006328	Leptinotarsa decemlineata	Putative uncharacterized protein
EOG091202T5	YQE_12068	Dendroctonus ponderosae	Putative uncharacterized protein
EOG091202T7	OTAU007129	Onthophagus taurus	Putative uncharacterized protein
EOG091202T7	AGLA000454	Anoplophora glabripennis	Putative uncharacterized protein
EOG091202T7	APLA010465	Agrilus planipennis	Putative uncharacterized protein
EOG091202T7	TC007162	Tribolium castaneum	Putative uncharacterized protein
EOG091202T7	LDEC000978	Leptinotarsa decemlineata	Putative uncharacterized protein
EOG091202T7	YQE_07366	Dendroctonus ponderosae	Putative uncharacterized protein
EOG091202T8	OTAU016558	Onthophagus taurus	Putative uncharacterized protein
EOG091202T8	AGLA017853	Anoplophora glabripennis	Putative uncharacterized protein
EOG091202T8	APLA008273	Agrilus planipennis	Putative uncharacterized protein
EOG091202T8	TC013007	Tribolium castaneum	Putative uncharacterized protein
EOG091202T8	LDEC014014	Leptinotarsa decemlineata	Putative uncharacterized protein
EOG091202T8	YQE_12291	Dendroctonus ponderosae	Putative uncharacterized protein
EOG091202T9	OTAU000704	Onthophagus taurus	Putative uncharacterized protein
EOG091202T9	AGLA006883	Anoplophora glabripennis	Putative uncharacterized protein
EOG091202T9	APLA008386	Agrilus planipennis	Putative uncharacterized protein
EOG091202T9	TC002495	Tribolium castaneum	Putative uncharacterized protein
EOG091202T9	LDEC015350	Leptinotarsa decemlineata	Putative uncharacterized protein
EOG091202T9	YQE_11075	Dendroctonus ponderosae	Putative uncharacterized protein
EOG091202TA	OTAU000055	Onthophagus taurus	Putative uncharacterized protein
EOG091202TA	AGLA009543	Anoplophora glabripennis	Putative uncharacterized protein
EOG091202TA	APLA012872	Agrilus planipennis	Putative uncharacterized protein
EOG091202TA	TC013890	Tribolium castaneum	Putative uncharacterized protein
EOG091202TA	LDEC003224	Leptinotarsa decemlineata	Putative uncharacterized protein
EOG091202TA	YQE_07212	Dendroctonus ponderosae	Putative uncharacterized protein
EOG091202TB	OTAU011017	Onthophagus taurus	Putative uncharacterized protein
EOG091202TB	AGLA012929	Anoplophora glabripennis	Putative uncharacterized protein
EOG091202TB	APLA007873	Agrilus planipennis	Putative uncharacterized protein
EOG091202TB	TC011081	Tribolium castaneum	Putative uncharacterized protein
EOG091202TB	LDEC014283	Leptinotarsa decemlineata	Putative uncharacterized protein
EOG091202TB	YQE_10928	Dendroctonus ponderosae	Putative uncharacterized protein
EOG091202TC	OTAU015168	Onthophagus taurus	Putative uncharacterized protein
EOG091202TC	AGLA015485	Anoplophora glabripennis	Putative uncharacterized protein
EOG091202TC	APLA003821	Agrilus planipennis	Putative uncharacterized protein
EOG091202TC	TC014911	Tribolium castaneum	Putative uncharacterized protein
EOG091202TC	LDEC008033	Leptinotarsa decemlineata	Putative uncharacterized protein
EOG091202TC	YQE_10903	Dendroctonus ponderosae	Putative uncharacterized protein
EOG091202TE	OTAU007710	Onthophagus taurus	Putative uncharacterized protein
EOG091202TE	AGLA003564	Anoplophora glabripennis	Putative uncharacterized protein
EOG091202TE	APLA002009	Agrilus planipennis	Putative uncharacterized protein
EOG091202TE	TC001573	Tribolium castaneum	Putative uncharacterized protein
EOG091202TE	LDEC020551	Leptinotarsa decemlineata	Putative uncharacterized protein
EOG091202TE	YQE_06441	Dendroctonus ponderosae	Putative uncharacterized protein
EOG091202TG	OTAU005862	Onthophagus taurus	Putative uncharacterized protein
EOG091202TG	AGLA020715	Anoplophora glabripennis	Putative uncharacterized protein
EOG091202TG	APLA003596	Agrilus planipennis	Putative uncharacterized protein
EOG091202TG	TC013491	Tribolium castaneum	Putative uncharacterized protein
EOG091202TG	LDEC016520	Leptinotarsa decemlineata	Putative uncharacterized protein
EOG091202TG	YQE_07165	Dendroctonus ponderosae	Putative uncharacterized protein
EOG091202TH	OTAU001877	Onthophagus taurus	Putative uncharacterized protein
EOG091202TH	AGLA006629	Anoplophora glabripennis	Putative uncharacterized protein
EOG091202TH	APLA000914	Agrilus planipennis	Putative uncharacterized protein
EOG091202TH	TC006419	Tribolium castaneum	Putative uncharacterized protein
EOG091202TH	LDEC009263	Leptinotarsa decemlineata	Putative uncharacterized protein
EOG091202TH	YQE_05795	Dendroctonus ponderosae	Putative uncharacterized protein
EOG091202TJ	OTAU007539	Onthophagus taurus	Putative uncharacterized protein

EOG091202TJ	AGLA010986	Anoplophora glabripennis	Putative uncharacterized protein
EOG091202TJ	APLA011332	Agrilus planipennis	Putative uncharacterized protein
EOG091202TJ	TC014503	Tribolium castaneum	Putative uncharacterized protein
EOG091202TJ	LDEC021216	Leptinotarsa decemlineata	Putative uncharacterized protein
EOG091202TJ	YQE_06129	Dendroctonus ponderosae	Putative uncharacterized protein
EOG091202TK	OTAU007176	Onthophagus taurus	Ubiquitin carboxyl-terminal hydrolase
EOG091202TK	AGLA005892	Anoplophora glabripennis	Ubiquitin carboxyl-terminal hydrolase
EOG091202TK	APLA000680	Agrilus planipennis	Ubiquitin carboxyl-terminal hydrolase
EOG091202TK	TC033362	Tribolium castaneum	Ubiquitin carboxyl-terminal hydrolase
EOG091202TK	LDEC000519	Leptinotarsa decemlineata	Ubiquitin carboxyl-terminal hydrolase
EOG091202TK	YQE_11057	Dendroctonus ponderosae	Ubiquitin carboxyl-terminal hydrolase
EOG091202TM	OTAU005882	Onthophagus taurus	mRNA cap guanine-N7 methyltransferase
EOG091202TM	AGLA015562	Anoplophora glabripennis	mRNA cap guanine-N7 methyltransferase
EOG091202TM	APLA012981	Agrilus planipennis	mRNA cap guanine-N7 methyltransferase
EOG091202TM	TC010427	Tribolium castaneum	mRNA cap guanine-N7 methyltransferase
EOG091202TM	LDEC002620	Leptinotarsa decemlineata	mRNA cap guanine-N7 methyltransferase
EOG091202TM	YQE_11399	Dendroctonus ponderosae	mRNA cap guanine-N7 methyltransferase
EOG091202TN	OTAU007363	Onthophagus taurus	Putative uncharacterized protein
EOG091202TN	AGLA018770	Anoplophora glabripennis	Putative uncharacterized protein
EOG091202TN	APLA007934	Agrilus planipennis	Putative uncharacterized protein
EOG091202TN	TC004980	Tribolium castaneum	Putative uncharacterized protein
EOG091202TN	LDEC003149	Leptinotarsa decemlineata	Putative uncharacterized protein
EOG091202TN	YQE_10985	Dendroctonus ponderosae	Putative uncharacterized protein
EOG091202TO	OTAU007342	Onthophagus taurus	Yellow-y
EOG091202TO	AGLA005222	Anoplophora glabripennis	Yellow-y
EOG091202TO	APLA014263	Agrilus planipennis	Yellow-y
EOG091202TO	TC000802	Tribolium castaneum	Yellow-y
EOG091202TO	LDEC018932	Leptinotarsa decemlineata	Yellow-y
EOG091202TO	YQE_10774	Dendroctonus ponderosae	Yellow-y
EOG091202TP	OTAU004114	Onthophagus taurus	Putative uncharacterized protein
EOG091202TP	AGLA007234	Anoplophora glabripennis	Putative uncharacterized protein
EOG091202TP	APLA001545	Agrilus planipennis	Putative uncharacterized protein
EOG091202TP	TC009045	Tribolium castaneum	Putative uncharacterized protein
EOG091202TP	LDEC009010	Leptinotarsa decemlineata	Putative uncharacterized protein
EOG091202TP	YQE_08798	Dendroctonus ponderosae	Putative uncharacterized protein
EOG091202TQ	OTAU001260	Onthophagus taurus	nucleotide binding
EOG091202TQ	AGLA000375	Anoplophora glabripennis	nucleotide binding
EOG091202TQ	APLA010005	Agrilus planipennis	nucleotide binding
EOG091202TQ	TC031499	Tribolium castaneum	nucleotide binding
EOG091202TQ	LDEC001561	Leptinotarsa decemlineata	nucleotide binding
EOG091202TQ	YQE_06700	Dendroctonus ponderosae	nucleotide binding
EOG091202TS	OTAU014056	Onthophagus taurus	Putative uncharacterized protein
EOG091202TS	AGLA012464	Anoplophora glabripennis	Putative uncharacterized protein
EOG091202TS	APLA010703	Agrilus planipennis	Putative uncharacterized protein
EOG091202TS	TC003565	Tribolium castaneum	Putative uncharacterized protein
EOG091202TS	LDEC003189	Leptinotarsa decemlineata	Putative uncharacterized protein
EOG091202TS	YQE_05896	Dendroctonus ponderosae	Putative uncharacterized protein
EOG091202TU	OTAU006760	Onthophagus taurus	Putative uncharacterized protein
EOG091202TU	AGLA002601	Anoplophora glabripennis	Putative uncharacterized protein
EOG091202TU	APLA008672	Agrilus planipennis	Putative uncharacterized protein
EOG091202TU	TC010660	Tribolium castaneum	Putative uncharacterized protein
EOG091202TU	LDEC002003	Leptinotarsa decemlineata	Putative uncharacterized protein
EOG091202TU	YQE_04968	Dendroctonus ponderosae	Putative uncharacterized protein
EOG091202TX	OTAU000748	Onthophagus taurus	Putative uncharacterized protein
EOG091202TX	AGLA002595	Anoplophora glabripennis	Putative uncharacterized protein
EOG091202TX	APLA010423	Agrilus planipennis	Putative uncharacterized protein
EOG091202TX	TC010650	Tribolium castaneum	Putative uncharacterized protein
EOG091202TX	LDEC002010	Leptinotarsa decemlineata	Putative uncharacterized protein
EOG091202TX	YQE_07976	Dendroctonus ponderosae	Putative uncharacterized protein
EOG091202TY	OTAU005217	Onthophagus taurus	Fork head
EOG091202TY	AGLA002850	Anoplophora glabripennis	Fork head
EOG091202TY	APLA014247	Agrilus planipennis	Fork head
EOG091202TY	TC013245	Tribolium castaneum	Fork head
EOG091202TY	LDEC012456	Leptinotarsa decemlineata	Fork head
EOG091202TY	YQE_04282	Dendroctonus ponderosae	Fork head
EOG091202TZ	OTAU011386	Onthophagus taurus	Putative uncharacterized protein
EOG091202TZ	AGLA014066	Anoplophora glabripennis	Putative uncharacterized protein

EOG091202TZ	APLA011745	Agrilus planipennis	Putative uncharacterized protein
EOG091202TZ	TC013887	Tribolium castaneum	Putative uncharacterized protein
EOG091202TZ	LDEC005978	Leptinotarsa decemlineata	Putative uncharacterized protein
EOG091202TZ	YQE_06183	Dendroctonus ponderosae	Putative uncharacterized protein
EOG091202U0	OTAU006659	Onthophagus taurus	Putative uncharacterized protein
EOG091202U0	AGLA021653	Anoplophora glabripennis	Putative uncharacterized protein
EOG091202U0	APLA004089	Agrilus planipennis	Putative uncharacterized protein
EOG091202U0	TC002979	Tribolium castaneum	Putative uncharacterized protein
EOG091202U0	LDEC016320	Leptinotarsa decemlineata	Putative uncharacterized protein
EOG091202U0	YQE_06430	Dendroctonus ponderosae	Putative uncharacterized protein
EOG091202U1	OTAU005458	Onthophagus taurus	Putative uncharacterized protein
EOG091202U1	AGLA015344	Anoplophora glabripennis	Putative uncharacterized protein
EOG091202U1	APLA002691	Agrilus planipennis	Putative uncharacterized protein
EOG091202U1	TC012992	Tribolium castaneum	Putative uncharacterized protein
EOG091202U1	LDEC005953	Leptinotarsa decemlineata	Putative uncharacterized protein
EOG091202U1	YQE_00065	Dendroctonus ponderosae	Putative uncharacterized protein
EOG091202U2	OTAU003903	Onthophagus taurus	Putative uncharacterized protein
EOG091202U2	AGLA017928	Anoplophora glabripennis	Putative uncharacterized protein
EOG091202U2	APLA007127	Agrilus planipennis	Putative uncharacterized protein
EOG091202U2	TC009040	Tribolium castaneum	Putative uncharacterized protein
EOG091202U2	LDEC015369	Leptinotarsa decemlineata	Putative uncharacterized protein
EOG091202U2	YQE_12629	Dendroctonus ponderosae	Putative uncharacterized protein
EOG091202U4	OTAU002021	Onthophagus taurus	Pelota
EOG091202U4	AGLA003165	Anoplophora glabripennis	Pelota
EOG091202U4	APLA002128	Agrilus planipennis	Pelota
EOG091202U4	TC001682	Tribolium castaneum	Pelota
EOG091202U4	LDEC007623	Leptinotarsa decemlineata	Pelota
EOG091202U4	YQE_09743	Dendroctonus ponderosae	Pelota
EOG091202U6	OTAU010131	Onthophagus taurus	Putative uncharacterized protein
EOG091202U6	AGLA013437	Anoplophora glabripennis	Putative uncharacterized protein
EOG091202U6	APLA002119	Agrilus planipennis	Putative uncharacterized protein
EOG091202U6	TC004467	Tribolium castaneum	Putative uncharacterized protein
EOG091202U6	LDEC006056	Leptinotarsa decemlineata	Putative uncharacterized protein
EOG091202U6	YQE_09481	Dendroctonus ponderosae	Putative uncharacterized protein
EOG091202U7	OTAU008447	Onthophagus taurus	Putative uncharacterized protein
EOG091202U7	AGLA004801	Anoplophora glabripennis	Putative uncharacterized protein
EOG091202U7	APLA013613	Agrilus planipennis	Putative uncharacterized protein
EOG091202U7	TC002813	Tribolium castaneum	Putative uncharacterized protein
EOG091202U7	LDEC021908	Leptinotarsa decemlineata	Putative uncharacterized protein
EOG091202U7	YQE_07639	Dendroctonus ponderosae	Putative uncharacterized protein
EOG091202U8	OTAU002299	Onthophagus taurus	Putative uncharacterized protein
EOG091202U8	AGLA004663	Anoplophora glabripennis	Putative uncharacterized protein
EOG091202U8	APLA011457	Agrilus planipennis	Putative uncharacterized protein
EOG091202U8	TC008243	Tribolium castaneum	Putative uncharacterized protein
EOG091202U8	LDEC018537	Leptinotarsa decemlineata	Putative uncharacterized protein
EOG091202U8	YQE_03066	Dendroctonus ponderosae	Putative uncharacterized protein
EOG091202U9	OTAU001712	Onthophagus taurus	Putative uncharacterized protein
EOG091202U9	AGLA012271	Anoplophora glabripennis	Putative uncharacterized protein
EOG091202U9	APLA005743	Agrilus planipennis	Putative uncharacterized protein
EOG091202U9	TC005369	Tribolium castaneum	Putative uncharacterized protein
EOG091202U9	LDEC022047	Leptinotarsa decemlineata	Putative uncharacterized protein
EOG091202U9	YQE_05785	Dendroctonus ponderosae	Putative uncharacterized protein
EOG091202UB	OTAU004009	Onthophagus taurus	Eukaryotic translation initiation factor 3 subunit I
EOG091202UB	AGLA001449	Anoplophora glabripennis	Eukaryotic translation initiation factor 3 subunit I
EOG091202UB	APLA005196	Agrilus planipennis	Eukaryotic translation initiation factor 3 subunit I
EOG091202UB	TC002391	Tribolium castaneum	Eukaryotic translation initiation factor 3 subunit I
EOG091202UB	LDEC013421	Leptinotarsa decemlineata	Eukaryotic translation initiation factor 3 subunit I
EOG091202UB	YQE_01850	Dendroctonus ponderosae	Eukaryotic translation initiation factor 3 subunit I
EOG091202UC	OTAU005602	Onthophagus taurus	Putative uncharacterized protein
EOG091202UC	AGLA013241	Anoplophora glabripennis	Putative uncharacterized protein
EOG091202UC	APLA010173	Agrilus planipennis	Putative uncharacterized protein
EOG091202UC	TC009258	Tribolium castaneum	Putative uncharacterized protein
EOG091202UC	LDEC012856	Leptinotarsa decemlineata	Putative uncharacterized protein
EOG091202UC	YQE_11311	Dendroctonus ponderosae	Putative uncharacterized protein
EOG091202UD	OTAU008908	Onthophagus taurus	Dawdle
EOG091202UD	AGLA004588	Anoplophora glabripennis	Dawdle
EOG091202UD	APLA003545	Agrilus planipennis	Dawdle

EOG091202UD	TC004297	Tribolium castaneum	Dawdle
EOG091202UD	LDEC018122	Leptinotarsa decemlineata	Dawdle
EOG091202UD	YQE_09294	Dendroctonus ponderosae	Dawdle
EOG091202UH	OTAU013131	Onthophagus taurus	Putative uncharacterized protein
EOG091202UH	AGLA012542	Anoplophora glabripennis	Putative uncharacterized protein
EOG091202UH	APLA007482	Agrilus planipennis	Putative uncharacterized protein
EOG091202UH	TC006166	Tribolium castaneum	Putative uncharacterized protein
EOG091202UH	LDEC018974	Leptinotarsa decemlineata	Putative uncharacterized protein
EOG091202UH	YQE_01897	Dendroctonus ponderosae	Putative uncharacterized protein
EOG091202UI	OTAU015897	Onthophagus taurus	G-protein coupled receptor binding
EOG091202UI	AGLA017187	Anoplophora glabripennis	G-protein coupled receptor binding
EOG091202UI	APLA003300	Agrilus planipennis	G-protein coupled receptor binding
EOG091202UI	TC034430	Tribolium castaneum	G-protein coupled receptor binding
EOG091202UI	LDEC009478	Leptinotarsa decemlineata	G-protein coupled receptor binding
EOG091202UI	YQE_10007	Dendroctonus ponderosae	G-protein coupled receptor binding
EOG091202UJ	OTAU013613	Onthophagus taurus	Oxysterol-binding protein
EOG091202UJ	AGLA015625	Anoplophora glabripennis	Oxysterol-binding protein
EOG091202UJ	APLA010170	Agrilus planipennis	Oxysterol-binding protein
EOG091202UJ	TC033806	Tribolium castaneum	Oxysterol-binding protein
EOG091202UJ	LDEC013913	Leptinotarsa decemlineata	Oxysterol-binding protein
EOG091202UJ	YQE_08718	Dendroctonus ponderosae	Oxysterol-binding protein
EOG091202UM	OTAU001408	Onthophagus taurus	nucleotide binding
EOG091202UM	AGLA002208	Anoplophora glabripennis	nucleotide binding
EOG091202UM	APLA015261	Agrilus planipennis	nucleotide binding
EOG091202UM	TC034524	Tribolium castaneum	nucleotide binding
EOG091202UM	LDEC006817	Leptinotarsa decemlineata	nucleotide binding
EOG091202UM	YQE_09122	Dendroctonus ponderosae	nucleotide binding
EOG091202UN	OTAU005647	Onthophagus taurus	DNA binding
EOG091202UN	AGLA007540	Anoplophora glabripennis	DNA binding
EOG091202UN	APLA007800	Agrilus planipennis	DNA binding
EOG091202UN	TC032451	Tribolium castaneum	DNA binding
EOG091202UN	LDEC000201	Leptinotarsa decemlineata	DNA binding
EOG091202UN	YQE_07794	Dendroctonus ponderosae	DNA binding
EOG091202UO	OTAU010566	Onthophagus taurus	Putative uncharacterized protein
EOG091202UO	AGLA001304	Anoplophora glabripennis	Putative uncharacterized protein
EOG091202UO	APLA005267	Agrilus planipennis	Putative uncharacterized protein
EOG091202UO	TC004932	Tribolium castaneum	Putative uncharacterized protein
EOG091202UO	LDEC009642	Leptinotarsa decemlineata	Putative uncharacterized protein
EOG091202UO	YQE_11027	Dendroctonus ponderosae	Putative uncharacterized protein
EOG091202US	OTAU003554	Onthophagus taurus	Putative uncharacterized protein
EOG091202US	AGLA016106	Anoplophora glabripennis	Putative uncharacterized protein
EOG091202US	APLA002649	Agrilus planipennis	Putative uncharacterized protein
EOG091202US	TC002263	Tribolium castaneum	Putative uncharacterized protein
EOG091202US	LDEC003840	Leptinotarsa decemlineata	Putative uncharacterized protein
EOG091202US	YQE_08039	Dendroctonus ponderosae	Putative uncharacterized protein
EOG091202UV	OTAU009524	Onthophagus taurus	Putative uncharacterized protein
EOG091202UV	AGLA006321	Anoplophora glabripennis	Putative uncharacterized protein
EOG091202UV	APLA010854	Agrilus planipennis	Putative uncharacterized protein
EOG091202UV	TC009207	Tribolium castaneum	Putative uncharacterized protein
EOG091202UV	LDEC007567	Leptinotarsa decemlineata	Putative uncharacterized protein
EOG091202UV	YQE_05286	Dendroctonus ponderosae	Putative uncharacterized protein
EOG091202UY	OTAU006248	Onthophagus taurus	Putative uncharacterized protein
EOG091202UY	AGLA004089	Anoplophora glabripennis	Putative uncharacterized protein
EOG091202UY	APLA001820	Agrilus planipennis	Putative uncharacterized protein
EOG091202UY	TC001316	Tribolium castaneum	Putative uncharacterized protein
EOG091202UY	LDEC014685	Leptinotarsa decemlineata	Putative uncharacterized protein
EOG091202UY	YQE_05248	Dendroctonus ponderosae	Putative uncharacterized protein
EOG091202UZ	OTAU011785	Onthophagus taurus	Cysteine synthase
EOG091202UZ	AGLA006458	Anoplophora glabripennis	Cysteine synthase
EOG091202UZ	APLA014127	Agrilus planipennis	Cysteine synthase
EOG091202UZ	TC033633	Tribolium castaneum	Cysteine synthase
EOG091202UZ	LDEC000759	Leptinotarsa decemlineata	Cysteine synthase
EOG091202UZ	YQE_11852	Dendroctonus ponderosae	Cysteine synthase
EOG091202V0	OTAU002250	Onthophagus taurus	Putative uncharacterized protein
EOG091202V0	AGLA020814	Anoplophora glabripennis	Putative uncharacterized protein
EOG091202V0	APLA013912	Agrilus planipennis	Putative uncharacterized protein
EOG091202V0	TC013868	Tribolium castaneum	Putative uncharacterized protein

EOG091202V0	LDEC000263	Leptinotarsa decemlineata	Putative uncharacterized protein
EOG091202V0	YQE_08210	Dendroctonus ponderosae	Putative uncharacterized protein
EOG091202V1	OTAU010752	Onthophagus taurus	Putative uncharacterized protein
EOG091202V1	AGLA000490	Anoplophora glabripennis	Putative uncharacterized protein
EOG091202V1	APLA002303	Agrilus planipennis	Putative uncharacterized protein
EOG091202V1	TC007036	Tribolium castaneum	Putative uncharacterized protein
EOG091202V1	LDEC002403	Leptinotarsa decemlineata	Putative uncharacterized protein
EOG091202V1	YQE_11449	Dendroctonus ponderosae	Putative uncharacterized protein
EOG091202V2	OTAU011183	Onthophagus taurus	Putative uncharacterized protein
EOG091202V2	AGLA002937	Anoplophora glabripennis	Putative uncharacterized protein
EOG091202V2	APLA013458	Agrilus planipennis	Putative uncharacterized protein
EOG091202V2	TC007006	Tribolium castaneum	Putative uncharacterized protein
EOG091202V2	LDEC004373	Leptinotarsa decemlineata	Putative uncharacterized protein
EOG091202V2	YQE_10019	Dendroctonus ponderosae	Putative uncharacterized protein
EOG091202V3	OTAU004042	Onthophagus taurus	Putative uncharacterized protein
EOG091202V3	AGLA002118	Anoplophora glabripennis	Putative uncharacterized protein
EOG091202V3	APLA004510	Agrilus planipennis	Putative uncharacterized protein
EOG091202V3	TC005457	Tribolium castaneum	Putative uncharacterized protein
EOG091202V3	LDEC010241	Leptinotarsa decemlineata	Putative uncharacterized protein
EOG091202V3	YQE_05556	Dendroctonus ponderosae	Putative uncharacterized protein
EOG091202V6	OTAU009266	Onthophagus taurus	Putative uncharacterized protein
EOG091202V6	AGLA006464	Anoplophora glabripennis	Putative uncharacterized protein
EOG091202V6	APLA008819	Agrilus planipennis	Putative uncharacterized protein
EOG091202V6	TC009847	Tribolium castaneum	Putative uncharacterized protein
EOG091202V6	LDEC016635	Leptinotarsa decemlineata	Putative uncharacterized protein
EOG091202V6	YQE_03528	Dendroctonus ponderosae	Putative uncharacterized protein
EOG091202V8	OTAU007615	Onthophagus taurus	Putative uncharacterized protein
EOG091202V8	AGLA019261	Anoplophora glabripennis	Putative uncharacterized protein
EOG091202V8	APLA008300	Agrilus planipennis	Putative uncharacterized protein
EOG091202V8	TC003201	Tribolium castaneum	Putative uncharacterized protein
EOG091202V8	LDEC004991	Leptinotarsa decemlineata	Putative uncharacterized protein
EOG091202V8	YQE_04541	Dendroctonus ponderosae	Putative uncharacterized protein
EOG091202VD	OTAU003935	Onthophagus taurus	Crossveinless 2
EOG091202VD	AGLA008237	Anoplophora glabripennis	Crossveinless 2
EOG091202VD	APLA011414	Agrilus planipennis	Crossveinless 2
EOG091202VD	TC012674	Tribolium castaneum	Crossveinless 2
EOG091202VD	LDEC000488	Leptinotarsa decemlineata	Crossveinless 2
EOG091202VD	YQE_02892	Dendroctonus ponderosae	Crossveinless 2
EOG091202VG	OTAU002864	Onthophagus taurus	Putative uncharacterized protein
EOG091202VG	AGLA000301	Anoplophora glabripennis	Putative uncharacterized protein
EOG091202VG	APLA005611	Agrilus planipennis	Putative uncharacterized protein
EOG091202VG	TC010814	Tribolium castaneum	Putative uncharacterized protein
EOG091202VG	LDEC003758	Leptinotarsa decemlineata	Putative uncharacterized protein
EOG091202VG	YQE_02524	Dendroctonus ponderosae	Putative uncharacterized protein
EOG091202VJ	OTAU000102	Onthophagus taurus	None
EOG091202VJ	AGLA014432	Anoplophora glabripennis	None
EOG091202VJ	APLA012880	Agrilus planipennis	None
EOG091202VJ	TC033009	Tribolium castaneum	None
EOG091202VJ	LDEC008702	Leptinotarsa decemlineata	None
EOG091202VJ	YQE_04005	Dendroctonus ponderosae	None
EOG091202VK	OTAU007938	Onthophagus taurus	Putative uncharacterized protein
EOG091202VK	AGLA011822	Anoplophora glabripennis	Putative uncharacterized protein
EOG091202VK	APLA003440	Agrilus planipennis	Putative uncharacterized protein
EOG091202VK	TC009872	Tribolium castaneum	Putative uncharacterized protein
EOG091202VK	LDEC001284	Leptinotarsa decemlineata	Putative uncharacterized protein
EOG091202VK	YQE_10405	Dendroctonus ponderosae	Putative uncharacterized protein
EOG091202VL	OTAU009303	Onthophagus taurus	None
EOG091202VL	AGLA007174	Anoplophora glabripennis	None
EOG091202VL	APLA010195	Agrilus planipennis	None
EOG091202VL	TC033575	Tribolium castaneum	None
EOG091202VL	LDEC010854	Leptinotarsa decemlineata	None
EOG091202VL	YQE_05243	Dendroctonus ponderosae	None
EOG091202VN	OTAU007975	Onthophagus taurus	Putative uncharacterized protein
EOG091202VN	AGLA006901	Anoplophora glabripennis	Putative uncharacterized protein
EOG091202VN	APLA000128	Agrilus planipennis	Putative uncharacterized protein
EOG091202VN	TC000095	Tribolium castaneum	Putative uncharacterized protein
EOG091202VN	LDEC020981	Leptinotarsa decemlineata	Putative uncharacterized protein

EOG091202VN	YQE_09855	Dendroctonus ponderosae	Putative uncharacterized protein
EOG091202VO	OTAU014259	Onthophagus taurus	Putative uncharacterized protein
EOG091202VO	AGLA015236	Anoplophora glabripennis	Putative uncharacterized protein
EOG091202VO	APLA000767	Agrilus planipennis	Putative uncharacterized protein
EOG091202VO	TC000050	Tribolium castaneum	Putative uncharacterized protein
EOG091202VO	LDEC005008	Leptinotarsa decemlineata	Putative uncharacterized protein
EOG091202VO	YQE_11856	Dendroctonus ponderosae	Putative uncharacterized protein
EOG091202VR	OTAU001830	Onthophagus taurus	Putative uncharacterized protein
EOG091202VR	AGLA005026	Anoplophora glabripennis	Putative uncharacterized protein
EOG091202VR	APLA004286	Agrilus planipennis	Putative uncharacterized protein
EOG091202VR	TC006608	Tribolium castaneum	Putative uncharacterized protein
EOG091202VR	LDEC006743	Leptinotarsa decemlineata	Putative uncharacterized protein
EOG091202VR	YQE_07485	Dendroctonus ponderosae	Putative uncharacterized protein
EOG091202VS	OTAU001291	Onthophagus taurus	None
EOG091202VS	AGLA001143	Anoplophora glabripennis	None
EOG091202VS	APLA006375	Agrilus planipennis	None
EOG091202VS	TC031525	Tribolium castaneum	None
EOG091202VS	LDEC010415	Leptinotarsa decemlineata	None
EOG091202VS	YQE_03328	Dendroctonus ponderosae	None
EOG091202VV	OTAU002688	Onthophagus taurus	Putative uncharacterized protein
EOG091202VV	AGLA010972	Anoplophora glabripennis	Putative uncharacterized protein
EOG091202VV	APLA008290	Agrilus planipennis	Putative uncharacterized protein
EOG091202VV	TC013870	Tribolium castaneum	Putative uncharacterized protein
EOG091202VV	LDEC008913	Leptinotarsa decemlineata	Putative uncharacterized protein
EOG091202VV	YQE_07439	Dendroctonus ponderosae	Putative uncharacterized protein
EOG091202VW	OTAU016174	Onthophagus taurus	Putative uncharacterized protein
EOG091202VW	AGLA021572	Anoplophora glabripennis	Putative uncharacterized protein
EOG091202VW	APLA008950	Agrilus planipennis	Putative uncharacterized protein
EOG091202VW	TC004386	Tribolium castaneum	Putative uncharacterized protein
EOG091202VW	LDEC006111	Leptinotarsa decemlineata	Putative uncharacterized protein
EOG091202VW	YQE_09474	Dendroctonus ponderosae	Putative uncharacterized protein
EOG091202W0	OTAU011771	Onthophagus taurus	Putative uncharacterized protein
EOG091202W0	AGLA008264	Anoplophora glabripennis	Putative uncharacterized protein
EOG091202W0	APLA001654	Agrilus planipennis	Putative uncharacterized protein
EOG091202W0	TC009927	Tribolium castaneum	Putative uncharacterized protein
EOG091202W0	LDEC001301	Leptinotarsa decemlineata	Putative uncharacterized protein
EOG091202W0	YQE_00082	Dendroctonus ponderosae	Putative uncharacterized protein
EOG091202W2	OTAU007249	Onthophagus taurus	3-oxoacyl-
EOG091202W2	AGLA011184	Anoplophora glabripennis	3-oxoacyl-
EOG091202W2	APLA010548	Agrilus planipennis	3-oxoacyl-
EOG091202W2	TC011607	Tribolium castaneum	3-oxoacyl-
EOG091202W2	LDEC009935	Leptinotarsa decemlineata	3-oxoacyl-
EOG091202W2	YQE_11080	Dendroctonus ponderosae	3-oxoacyl-
EOG091202W3	OTAU014996	Onthophagus taurus	None
EOG091202W3	AGLA021659	Anoplophora glabripennis	None
EOG091202W3	APLA011623	Agrilus planipennis	None
EOG091202W3	TC030999	Tribolium castaneum	None
EOG091202W3	LDEC008153	Leptinotarsa decemlineata	None
EOG091202W3	YQE_09669	Dendroctonus ponderosae	None
EOG091202W7	OTAU001582	Onthophagus taurus	Putative uncharacterized protein
EOG091202W7	AGLA011021	Anoplophora glabripennis	Putative uncharacterized protein
EOG091202W7	APLA005058	Agrilus planipennis	Putative uncharacterized protein
EOG091202W7	TC002177	Tribolium castaneum	Putative uncharacterized protein
EOG091202W7	LDEC015059	Leptinotarsa decemlineata	Putative uncharacterized protein
EOG091202W7	YQE_12001	Dendroctonus ponderosae	Putative uncharacterized protein
EOG091202W9	OTAU001979	Onthophagus taurus	Putative uncharacterized protein
EOG091202W9	AGLA010222	Anoplophora glabripennis	Putative uncharacterized protein
EOG091202W9	APLA014770	Agrilus planipennis	Putative uncharacterized protein
EOG091202W9	TC005597	Tribolium castaneum	Putative uncharacterized protein
EOG091202W9	LDEC002964	Leptinotarsa decemlineata	Putative uncharacterized protein
EOG091202W9	YQE_12056	Dendroctonus ponderosae	Putative uncharacterized protein
EOG091202WA	OTAU001343	Onthophagus taurus	None
EOG091202WA	AGLA001179	Anoplophora glabripennis	None
EOG091202WA	APLA009678	Agrilus planipennis	None
EOG091202WA	TC031556	Tribolium castaneum	None
EOG091202WA	LDEC015742	Leptinotarsa decemlineata	None
EOG091202WA	YQE_02444	Dendroctonus ponderosae	None

EOG091202WB	OTAU015859	Onthophagus taurus	Putative uncharacterized protein
EOG091202WB	AGLA021002	Anoplophora glabripennis	Putative uncharacterized protein
EOG091202WB	APLA007221	Agrilus planipennis	Putative uncharacterized protein
EOG091202WB	TC002446	Tribolium castaneum	Putative uncharacterized protein
EOG091202WB	LDEC008586	Leptinotarsa decemlineata	Putative uncharacterized protein
EOG091202WB	YQE_02150	Dendroctonus ponderosae	Putative uncharacterized protein
EOG091202WC	OTAU005061	Onthophagus taurus	Putative uncharacterized protein
EOG091202WC	AGLA020108	Anoplophora glabripennis	Putative uncharacterized protein
EOG091202WC	APLA000473	Agrilus planipennis	Putative uncharacterized protein
EOG091202WC	TC006774	Tribolium castaneum	Putative uncharacterized protein
EOG091202WC	LDEC010564	Leptinotarsa decemlineata	Putative uncharacterized protein
EOG091202WC	YQE_05788	Dendroctonus ponderosae	Putative uncharacterized protein
EOG091202WF	OTAU000671	Onthophagus taurus	Putative uncharacterized protein
EOG091202WF	AGLA013350	Anoplophora glabripennis	Putative uncharacterized protein
EOG091202WF	APLA002665	Agrilus planipennis	Putative uncharacterized protein
EOG091202WF	TC012046	Tribolium castaneum	Putative uncharacterized protein
EOG091202WF	LDEC018237	Leptinotarsa decemlineata	Putative uncharacterized protein
EOG091202WF	YQE_12814	Dendroctonus ponderosae	Putative uncharacterized protein
EOG091202WG	OTAU002550	Onthophagus taurus	Putative uncharacterized protein
EOG091202WG	AGLA018491	Anoplophora glabripennis	Putative uncharacterized protein
EOG091202WG	APLA014471	Agrilus planipennis	Putative uncharacterized protein
EOG091202WG	TC016320	Tribolium castaneum	Putative uncharacterized protein
EOG091202WG	LDEC020891	Leptinotarsa decemlineata	Putative uncharacterized protein
EOG091202WG	YQE_02046	Dendroctonus ponderosae	Putative uncharacterized protein
EOG091202WI	OTAU017397	Onthophagus taurus	Putative uncharacterized protein
EOG091202WI	AGLA008967	Anoplophora glabripennis	Putative uncharacterized protein
EOG091202WI	APLA001713	Agrilus planipennis	Putative uncharacterized protein
EOG091202WI	TC015163	Tribolium castaneum	Putative uncharacterized protein
EOG091202WI	LDEC001880	Leptinotarsa decemlineata	Putative uncharacterized protein
EOG091202WI	YQE_05949	Dendroctonus ponderosae	Putative uncharacterized protein
EOG091202WJ	OTAU008887	Onthophagus taurus	Putative uncharacterized protein
EOG091202WJ	AGLA012920	Anoplophora glabripennis	Putative uncharacterized protein
EOG091202WJ	APLA007936	Agrilus planipennis	Putative uncharacterized protein
EOG091202WJ	TC011311	Tribolium castaneum	Putative uncharacterized protein
EOG091202WJ	LDEC006593	Leptinotarsa decemlineata	Putative uncharacterized protein
EOG091202WJ	YQE_01649	Dendroctonus ponderosae	Putative uncharacterized protein
EOG091202WO	OTAU011808	Onthophagus taurus	Putative uncharacterized protein
EOG091202WO	AGLA011028	Anoplophora glabripennis	Putative uncharacterized protein
EOG091202WO	APLA012295	Agrilus planipennis	Putative uncharacterized protein
EOG091202WO	TC005759	Tribolium castaneum	Putative uncharacterized protein
EOG091202WO	LDEC016202	Leptinotarsa decemlineata	Putative uncharacterized protein
EOG091202WO	YQE_07855	Dendroctonus ponderosae	Putative uncharacterized protein
EOG091202WR	OTAU012996	Onthophagus taurus	Putative uncharacterized protein
EOG091202WR	AGLA007741	Anoplophora glabripennis	Putative uncharacterized protein
EOG091202WR	APLA003756	Agrilus planipennis	Putative uncharacterized protein
EOG091202WR	TC003908	Tribolium castaneum	Putative uncharacterized protein
EOG091202WR	LDEC000228	Leptinotarsa decemlineata	Putative uncharacterized protein
EOG091202WR	YQE_09894	Dendroctonus ponderosae	Putative uncharacterized protein
EOG091202WS	OTAU013352	Onthophagus taurus	Putative uncharacterized protein
EOG091202WS	AGLA012400	Anoplophora glabripennis	Putative uncharacterized protein
EOG091202WS	APLA010678	Agrilus planipennis	Putative uncharacterized protein
EOG091202WS	TC011137	Tribolium castaneum	Putative uncharacterized protein
EOG091202WS	LDEC019160	Leptinotarsa decemlineata	Putative uncharacterized protein
EOG091202WS	YQE_03144	Dendroctonus ponderosae	Putative uncharacterized protein
EOG091202WT	OTAU011032	Onthophagus taurus	Putative uncharacterized protein
EOG091202WT	AGLA016529	Anoplophora glabripennis	Putative uncharacterized protein
EOG091202WT	APLA003708	Agrilus planipennis	Putative uncharacterized protein
EOG091202WT	TC004956	Tribolium castaneum	Putative uncharacterized protein
EOG091202WT	LDEC021663	Leptinotarsa decemlineata	Putative uncharacterized protein
EOG091202WT	YQE_08511	Dendroctonus ponderosae	Putative uncharacterized protein
EOG091202WV	OTAU002762	Onthophagus taurus	Putative uncharacterized protein
EOG091202WV	AGLA000253	Anoplophora glabripennis	Putative uncharacterized protein
EOG091202WV	APLA006415	Agrilus planipennis	Putative uncharacterized protein
EOG091202WV	TC001743	Tribolium castaneum	Putative uncharacterized protein
EOG091202WV	LDEC012033	Leptinotarsa decemlineata	Putative uncharacterized protein
EOG091202WV	YQE_02385	Dendroctonus ponderosae	Putative uncharacterized protein
EOG091202WY	OTAU003861	Onthophagus taurus	Putative uncharacterized protein

EOG091202WY	AGLA013015	Anoplophora glabripennis	Putative uncharacterized protein
EOG091202WY	APLA011189	Agrilus planipennis	Putative uncharacterized protein
EOG091202WY	TC009598	Tribolium castaneum	Putative uncharacterized protein
EOG091202WY	LDEC004837	Leptinotarsa decemlineata	Putative uncharacterized protein
EOG091202WY	YQE_03527	Dendroctonus ponderosae	Putative uncharacterized protein
EOG091202WZ	OTAU014202	Onthophagus taurus	Shaven
EOG091202WZ	AGLA006193	Anoplophora glabripennis	Shaven
EOG091202WZ	APLA011475	Agrilus planipennis	Shaven
EOG091202WZ	TC003570	Tribolium castaneum	Shaven
EOG091202WZ	LDEC012765	Leptinotarsa decemlineata	Shaven
EOG091202WZ	YQE_12360	Dendroctonus ponderosae	Shaven
EOG091202X0	OTAU003108	Onthophagus taurus	Polarization-related protein LKB1
EOG091202X0	AGLA017231	Anoplophora glabripennis	Polarization-related protein LKB1
EOG091202X0	APLA004012	Agrilus planipennis	Polarization-related protein LKB1
EOG091202X0	TC012166	Tribolium castaneum	Polarization-related protein LKB1
EOG091202X0	LDEC003861	Leptinotarsa decemlineata	Polarization-related protein LKB1
EOG091202X0	YQE_07575	Dendroctonus ponderosae	Polarization-related protein LKB1
EOG091202X1	OTAU012027	Onthophagus taurus	Serine protease H164
EOG091202X1	AGLA004870	Anoplophora glabripennis	Serine protease H164
EOG091202X1	APLA008762	Agrilus planipennis	Serine protease H164
EOG091202X1	TC015670	Tribolium castaneum	Serine protease H164
EOG091202X1	LDEC005371	Leptinotarsa decemlineata	Serine protease H164
EOG091202X1	YQE_10812	Dendroctonus ponderosae	Serine protease H164
EOG091202X6	OTAU006390	Onthophagus taurus	Putative uncharacterized protein
EOG091202X6	AGLA000512	Anoplophora glabripennis	Putative uncharacterized protein
EOG091202X6	APLA002171	Agrilus planipennis	Putative uncharacterized protein
EOG091202X6	TC007514	Tribolium castaneum	Putative uncharacterized protein
EOG091202X6	LDEC002379	Leptinotarsa decemlineata	Putative uncharacterized protein
EOG091202X6	YQE_06856	Dendroctonus ponderosae	Putative uncharacterized protein
EOG091202X8	OTAU013599	Onthophagus taurus	Putative uncharacterized protein
EOG091202X8	AGLA008850	Anoplophora glabripennis	Putative uncharacterized protein
EOG091202X8	APLA007731	Agrilus planipennis	Putative uncharacterized protein
EOG091202X8	TC014612	Tribolium castaneum	Putative uncharacterized protein
EOG091202X8	LDEC009045	Leptinotarsa decemlineata	Putative uncharacterized protein
EOG091202X8	YQE_03217	Dendroctonus ponderosae	Putative uncharacterized protein
EOG091202X9	OTAU012493	Onthophagus taurus	Putative uncharacterized protein
EOG091202X9	AGLA020909	Anoplophora glabripennis	Putative uncharacterized protein
EOG091202X9	APLA009701	Agrilus planipennis	Putative uncharacterized protein
EOG091202X9	TC003328	Tribolium castaneum	Putative uncharacterized protein
EOG091202X9	LDEC002669	Leptinotarsa decemlineata	Putative uncharacterized protein
EOG091202X9	YQE_02286	Dendroctonus ponderosae	Putative uncharacterized protein
EOG091202XB	OTAU015647	Onthophagus taurus	Putative uncharacterized protein
EOG091202XB	AGLA009705	Anoplophora glabripennis	Putative uncharacterized protein
EOG091202XB	APLA010869	Agrilus planipennis	Putative uncharacterized protein
EOG091202XB	TC009740	Tribolium castaneum	Putative uncharacterized protein
EOG091202XB	LDEC018926	Leptinotarsa decemlineata	Putative uncharacterized protein
EOG091202XB	YQE_11349	Dendroctonus ponderosae	Putative uncharacterized protein
EOG091202XH	OTAU010538	Onthophagus taurus	metal ion binding
EOG091202XH	AGLA017280	Anoplophora glabripennis	metal ion binding
EOG091202XH	APLA007390	Agrilus planipennis	metal ion binding
EOG091202XH	TC033042	Tribolium castaneum	metal ion binding
EOG091202XH	LDEC003069	Leptinotarsa decemlineata	metal ion binding
EOG091202XH	YQE_06249	Dendroctonus ponderosae	metal ion binding
EOG091202XJ	OTAU001893	Onthophagus taurus	Putative uncharacterized protein
EOG091202XJ	AGLA002493	Anoplophora glabripennis	Putative uncharacterized protein
EOG091202XJ	APLA012076	Agrilus planipennis	Putative uncharacterized protein
EOG091202XJ	TC005356	Tribolium castaneum	Putative uncharacterized protein
EOG091202XJ	LDEC005860	Leptinotarsa decemlineata	Putative uncharacterized protein
EOG091202XJ	YQE_07517	Dendroctonus ponderosae	Putative uncharacterized protein
EOG091202XL	OTAU001551	Onthophagus taurus	Putative uncharacterized protein
EOG091202XL	AGLA010580	Anoplophora glabripennis	Putative uncharacterized protein
EOG091202XL	APLA004524	Agrilus planipennis	Putative uncharacterized protein
EOG091202XL	TC005649	Tribolium castaneum	Putative uncharacterized protein
EOG091202XL	LDEC017275	Leptinotarsa decemlineata	Putative uncharacterized protein
EOG091202XL	YQE_05713	Dendroctonus ponderosae	Putative uncharacterized protein
EOG091202XP	OTAU014011	Onthophagus taurus	Putative uncharacterized protein
EOG091202XP	AGLA002692	Anoplophora glabripennis	Putative uncharacterized protein

EOG091202XP	APLA004067	Agrilus planipennis	Putative uncharacterized protein
EOG091202XP	TC004918	Tribolium castaneum	Putative uncharacterized protein
EOG091202XP	LDEC021702	Leptinotarsa decemlineata	Putative uncharacterized protein
EOG091202XP	YQE_11420	Dendroctonus ponderosae	Putative uncharacterized protein
EOG091202XQ	OTAU005812	Onthophagus taurus	Putative uncharacterized protein
EOG091202XQ	AGLA001512	Anoplophora glabripennis	Putative uncharacterized protein
EOG091202XQ	APLA003788	Agrilus planipennis	Putative uncharacterized protein
EOG091202XQ	TC002635	Tribolium castaneum	Putative uncharacterized protein
EOG091202XQ	LDEC004650	Leptinotarsa decemlineata	Putative uncharacterized protein
EOG091202XQ	YQE_04555	Dendroctonus ponderosae	Putative uncharacterized protein
EOG091202XS	OTAU013639	Onthophagus taurus	Putative uncharacterized protein
EOG091202XS	AGLA003060	Anoplophora glabripennis	Putative uncharacterized protein
EOG091202XS	APLA002236	Agrilus planipennis	Putative uncharacterized protein
EOG091202XS	TC007925	Tribolium castaneum	Putative uncharacterized protein
EOG091202XS	LDEC017222	Leptinotarsa decemlineata	Putative uncharacterized protein
EOG091202XS	YQE_01757	Dendroctonus ponderosae	Putative uncharacterized protein
EOG091202XT	OTAU004702	Onthophagus taurus	Putative uncharacterized protein
EOG091202XT	AGLA012322	Anoplophora glabripennis	Putative uncharacterized protein
EOG091202XT	APLA001761	Agrilus planipennis	Putative uncharacterized protein
EOG091202XT	TC012252	Tribolium castaneum	Putative uncharacterized protein
EOG091202XT	LDEC020790	Leptinotarsa decemlineata	Putative uncharacterized protein
EOG091202XT	YQE_07607	Dendroctonus ponderosae	Putative uncharacterized protein
EOG091202XU	OTAU015746	Onthophagus taurus	Putative uncharacterized protein
EOG091202XU	AGLA001063	Anoplophora glabripennis	Putative uncharacterized protein
EOG091202XU	APLA013083	Agrilus planipennis	Putative uncharacterized protein
EOG091202XU	TC015411	Tribolium castaneum	Putative uncharacterized protein
EOG091202XU	LDEC014186	Leptinotarsa decemlineata	Putative uncharacterized protein
EOG091202XU	YQE_03663	Dendroctonus ponderosae	Putative uncharacterized protein
EOG091202XV	OTAU006268	Onthophagus taurus	Gustatory receptor 2
EOG091202XV	AGLA003085	Anoplophora glabripennis	Gustatory receptor 2
EOG091202XV	APLA013145	Agrilus planipennis	Gustatory receptor 2
EOG091202XV	TC030103	Tribolium castaneum	Gustatory receptor 2
EOG091202XV	LDEC014761	Leptinotarsa decemlineata	Gustatory receptor 2
EOG091202XV	YQE_06884	Dendroctonus ponderosae	Gustatory receptor 2
EOG091202XY	OTAU003933	Onthophagus taurus	Putative uncharacterized protein
EOG091202XY	AGLA008232	Anoplophora glabripennis	Putative uncharacterized protein
EOG091202XY	APLA007404	Agrilus planipennis	Putative uncharacterized protein
EOG091202XY	TC011833	Tribolium castaneum	Putative uncharacterized protein
EOG091202XY	LDEC000489	Leptinotarsa decemlineata	Putative uncharacterized protein
EOG091202XY	YQE_02894	Dendroctonus ponderosae	Putative uncharacterized protein
EOG091202XZ	OTAU001065	Onthophagus taurus	Putative uncharacterized protein
EOG091202XZ	AGLA016315	Anoplophora glabripennis	Putative uncharacterized protein
EOG091202XZ	APLA007946	Agrilus planipennis	Putative uncharacterized protein
EOG091202XZ	TC011562	Tribolium castaneum	Putative uncharacterized protein
EOG091202XZ	LDEC010325	Leptinotarsa decemlineata	Putative uncharacterized protein
EOG091202XZ	YQE_07668	Dendroctonus ponderosae	Putative uncharacterized protein
EOG091202Y0	OTAU010997	Onthophagus taurus	Brachyenteron
EOG091202Y0	AGLA011137	Anoplophora glabripennis	Brachyenteron
EOG091202Y0	APLA008068	Agrilus planipennis	Brachyenteron
EOG091202Y0	TC014076	Tribolium castaneum	Brachyenteron
EOG091202Y0	LDEC017551	Leptinotarsa decemlineata	Brachyenteron
EOG091202Y0	YQE_08215	Dendroctonus ponderosae	Brachyenteron
EOG091202Y1	OTAU007769	Onthophagus taurus	None
EOG091202Y1	AGLA014649	Anoplophora glabripennis	None
EOG091202Y1	APLA007054	Agrilus planipennis	None
EOG091202Y1	TC033756	Tribolium castaneum	None
EOG091202Y1	LDEC023560	Leptinotarsa decemlineata	None
EOG091202Y1	YQE_05342	Dendroctonus ponderosae	None
EOG091202Y4	OTAU001752	Onthophagus taurus	None
EOG091202Y4	AGLA008637	Anoplophora glabripennis	None
EOG091202Y4	APLA012322	Agrilus planipennis	None
EOG091202Y4	TC034049	Tribolium castaneum	None
EOG091202Y4	LDEC003025	Leptinotarsa decemlineata	None
EOG091202Y4	YQE_08487	Dendroctonus ponderosae	None
EOG091202Y8	OTAU008451	Onthophagus taurus	Putative uncharacterized protein
EOG091202Y8	AGLA002313	Anoplophora glabripennis	Putative uncharacterized protein
EOG091202Y8	APLA002532	Agrilus planipennis	Putative uncharacterized protein

EOG091202Y8	TC011992	Tribolium castaneum	Putative uncharacterized protein
EOG091202Y8	LDEC004134	Leptinotarsa decemlineata	Putative uncharacterized protein
EOG091202Y8	YQE_01990	Dendroctonus ponderosae	Putative uncharacterized protein
EOG091202YC	OTAU001585	Onthophagus taurus	Putative uncharacterized protein
EOG091202YC	AGLA004970	Anoplophora glabripennis	Putative uncharacterized protein
EOG091202YC	APLA004499	Agrilus planipennis	Putative uncharacterized protein
EOG091202YC	TC015223	Tribolium castaneum	Putative uncharacterized protein
EOG091202YC	LDEC011215	Leptinotarsa decemlineata	Putative uncharacterized protein
EOG091202YC	YQE_05648	Dendroctonus ponderosae	Putative uncharacterized protein
EOG091202YE	OTAU008837	Onthophagus taurus	Putative uncharacterized protein
EOG091202YE	AGLA009838	Anoplophora glabripennis	Putative uncharacterized protein
EOG091202YE	APLA007428	Agrilus planipennis	Putative uncharacterized protein
EOG091202YE	TC008758	Tribolium castaneum	Putative uncharacterized protein
EOG091202YE	LDEC009357	Leptinotarsa decemlineata	Putative uncharacterized protein
EOG091202YE	YQE_05352	Dendroctonus ponderosae	Putative uncharacterized protein
EOG091202YG	OTAU013051	Onthophagus taurus	catalytic activity
EOG091202YG	AGLA007243	Anoplophora glabripennis	catalytic activity
EOG091202YG	APLA007080	Agrilus planipennis	catalytic activity
EOG091202YG	TC033681	Tribolium castaneum	catalytic activity
EOG091202YG	LDEC009021	Leptinotarsa decemlineata	catalytic activity
EOG091202YG	YQE_08791	Dendroctonus ponderosae	catalytic activity
EOG091202YH	OTAU011756	Onthophagus taurus	Putative uncharacterized protein
EOG091202YH	AGLA002030	Anoplophora glabripennis	Putative uncharacterized protein
EOG091202YH	APLA005241	Agrilus planipennis	Putative uncharacterized protein
EOG091202YH	TC005853	Tribolium castaneum	Putative uncharacterized protein
EOG091202YH	LDEC013422	Leptinotarsa decemlineata	Putative uncharacterized protein
EOG091202YH	YQE_12383	Dendroctonus ponderosae	Putative uncharacterized protein
EOG091202YI	OTAU000296	Onthophagus taurus	Putative uncharacterized protein
EOG091202YI	AGLA011918	Anoplophora glabripennis	Putative uncharacterized protein
EOG091202YI	APLA014457	Agrilus planipennis	Putative uncharacterized protein
EOG091202YI	TC012889	Tribolium castaneum	Putative uncharacterized protein
EOG091202YI	LDEC014431	Leptinotarsa decemlineata	Putative uncharacterized protein
EOG091202YI	YQE_02093	Dendroctonus ponderosae	Putative uncharacterized protein
EOG091202YK	OTAU004372	Onthophagus taurus	Putative uncharacterized protein
EOG091202YK	AGLA011323	Anoplophora glabripennis	Putative uncharacterized protein
EOG091202YK	APLA006425	Agrilus planipennis	Putative uncharacterized protein
EOG091202YK	TC010864	Tribolium castaneum	Putative uncharacterized protein
EOG091202YK	LDEC005730	Leptinotarsa decemlineata	Putative uncharacterized protein
EOG091202YK	YQE_00059	Dendroctonus ponderosae	Putative uncharacterized protein
EOG091202YL	OTAU013823	Onthophagus taurus	Putative uncharacterized protein
EOG091202YL	AGLA017693	Anoplophora glabripennis	Putative uncharacterized protein
EOG091202YL	APLA006696	Agrilus planipennis	Putative uncharacterized protein
EOG091202YL	TC003528	Tribolium castaneum	Putative uncharacterized protein
EOG091202YL	LDEC002670	Leptinotarsa decemlineata	Putative uncharacterized protein
EOG091202YL	YQE_02279	Dendroctonus ponderosae	Putative uncharacterized protein
EOG091202YP	OTAU002825	Onthophagus taurus	Putative uncharacterized protein
EOG091202YP	AGLA000335	Anoplophora glabripennis	Putative uncharacterized protein
EOG091202YP	APLA009993	Agrilus planipennis	Putative uncharacterized protein
EOG091202YP	TC014817	Tribolium castaneum	Putative uncharacterized protein
EOG091202YP	LDEC004707	Leptinotarsa decemlineata	Putative uncharacterized protein
EOG091202YP	YQE_07860	Dendroctonus ponderosae	Putative uncharacterized protein
EOG091202YR	OTAU004016	Onthophagus taurus	Putative uncharacterized protein
EOG091202YR	AGLA001448	Anoplophora glabripennis	Putative uncharacterized protein
EOG091202YR	APLA005197	Agrilus planipennis	Putative uncharacterized protein
EOG091202YR	TC003871	Tribolium castaneum	Putative uncharacterized protein
EOG091202YR	LDEC013420	Leptinotarsa decemlineata	Putative uncharacterized protein
EOG091202YR	YQE_01849	Dendroctonus ponderosae	Putative uncharacterized protein
EOG091202YS	OTAU006799	Onthophagus taurus	Putative uncharacterized protein
EOG091202YS	AGLA004381	Anoplophora glabripennis	Putative uncharacterized protein
EOG091202YS	APLA002552	Agrilus planipennis	Putative uncharacterized protein
EOG091202YS	TC001418	Tribolium castaneum	Putative uncharacterized protein
EOG091202YS	LDEC005451	Leptinotarsa decemlineata	Putative uncharacterized protein
EOG091202YS	YQE_11397	Dendroctonus ponderosae	Putative uncharacterized protein
EOG091202YT	OTAU008360	Onthophagus taurus	Putative uncharacterized protein
EOG091202YT	AGLA018769	Anoplophora glabripennis	Putative uncharacterized protein
EOG091202YT	APLA007932	Agrilus planipennis	Putative uncharacterized protein
EOG091202YT	TC000537	Tribolium castaneum	Putative uncharacterized protein

EOG091202YT	LDEC003148	Leptinotarsa decemlineata	Putative uncharacterized protein
EOG091202YT	YQE_07689	Dendroctonus ponderosae	Putative uncharacterized protein
EOG091202YU	OTAU001923	Onthophagus taurus	Putative uncharacterized protein
EOG091202YU	AGLA010660	Anoplophora glabripennis	Putative uncharacterized protein
EOG091202YU	APLA001239	Agrilus planipennis	Putative uncharacterized protein
EOG091202YU	TC006226	Tribolium castaneum	Putative uncharacterized protein
EOG091202YU	LDEC017461	Leptinotarsa decemlineata	Putative uncharacterized protein
EOG091202YU	YQE_12079	Dendroctonus ponderosae	Putative uncharacterized protein
EOG091202YV	OTAU003199	Onthophagus taurus	Putative uncharacterized protein
EOG091202YV	AGLA015866	Anoplophora glabripennis	Putative uncharacterized protein
EOG091202YV	APLA002757	Agrilus planipennis	Putative uncharacterized protein
EOG091202YV	TC013503	Tribolium castaneum	Putative uncharacterized protein
EOG091202YV	LDEC008336	Leptinotarsa decemlineata	Putative uncharacterized protein
EOG091202YV	YQE_06215	Dendroctonus ponderosae	Putative uncharacterized protein
EOG091202YW	OTAU004211	Onthophagus taurus	Putative uncharacterized protein
EOG091202YW	AGLA016711	Anoplophora glabripennis	Putative uncharacterized protein
EOG091202YW	APLA006865	Agrilus planipennis	Putative uncharacterized protein
EOG091202YW	TC014726	Tribolium castaneum	Putative uncharacterized protein
EOG091202YW	LDEC015569	Leptinotarsa decemlineata	Putative uncharacterized protein
EOG091202YW	YQE_13078	Dendroctonus ponderosae	Putative uncharacterized protein
EOG091202YX	OTAU015362	Onthophagus taurus	Adenylosuccinate synthetase
EOG091202YX	AGLA008262	Anoplophora glabripennis	Adenylosuccinate synthetase
EOG091202YX	APLA007847	Agrilus planipennis	Adenylosuccinate synthetase
EOG091202YX	TC034777	Tribolium castaneum	Adenylosuccinate synthetase
EOG091202YX	LDEC004780	Leptinotarsa decemlineata	Adenylosuccinate synthetase
EOG091202YX	YQE_12607	Dendroctonus ponderosae	Adenylosuccinate synthetase
EOG091202YZ	OTAU008806	Onthophagus taurus	Putative uncharacterized protein
EOG091202YZ	AGLA001744	Anoplophora glabripennis	Putative uncharacterized protein
EOG091202YZ	APLA013446	Agrilus planipennis	Putative uncharacterized protein
EOG091202YZ	TC013284	Tribolium castaneum	Putative uncharacterized protein
EOG091202YZ	LDEC013673	Leptinotarsa decemlineata	Putative uncharacterized protein
EOG091202YZ	YQE_12475	Dendroctonus ponderosae	Putative uncharacterized protein
EOG091202Z2	OTAU003070	Onthophagus taurus	Putative uncharacterized protein
EOG091202Z2	AGLA017112	Anoplophora glabripennis	Putative uncharacterized protein
EOG091202Z2	APLA001138	Agrilus planipennis	Putative uncharacterized protein
EOG091202Z2	TC016291	Tribolium castaneum	Putative uncharacterized protein
EOG091202Z2	LDEC020895	Leptinotarsa decemlineata	Putative uncharacterized protein
EOG091202Z2	YQE_04670	Dendroctonus ponderosae	Putative uncharacterized protein
EOG091202Z3	OTAU012020	Onthophagus taurus	Putative uncharacterized protein
EOG091202Z3	AGLA006747	Anoplophora glabripennis	Putative uncharacterized protein
EOG091202Z3	APLA004056	Agrilus planipennis	Putative uncharacterized protein
EOG091202Z3	TC014886	Tribolium castaneum	Putative uncharacterized protein
EOG091202Z3	LDEC012953	Leptinotarsa decemlineata	Putative uncharacterized protein
EOG091202Z3	YQE_00141	Dendroctonus ponderosae	Putative uncharacterized protein
EOG091202Z4	OTAU010932	Onthophagus taurus	Putative uncharacterized protein
EOG091202Z4	AGLA001061	Anoplophora glabripennis	Putative uncharacterized protein
EOG091202Z4	APLA008041	Agrilus planipennis	Putative uncharacterized protein
EOG091202Z4	TC015511	Tribolium castaneum	Putative uncharacterized protein
EOG091202Z4	LDEC007996	Leptinotarsa decemlineata	Putative uncharacterized protein
EOG091202Z4	YQE_08460	Dendroctonus ponderosae	Putative uncharacterized protein
EOG091202Z5	OTAU006647	Onthophagus taurus	Putative uncharacterized protein
EOG091202Z5	AGLA006891	Anoplophora glabripennis	Putative uncharacterized protein
EOG091202Z5	APLA003348	Agrilus planipennis	Putative uncharacterized protein
EOG091202Z5	TC000040	Tribolium castaneum	Putative uncharacterized protein
EOG091202Z5	LDEC004542	Leptinotarsa decemlineata	Putative uncharacterized protein
EOG091202Z5	YQE_04955	Dendroctonus ponderosae	Putative uncharacterized protein
EOG091202Z7	OTAU001933	Onthophagus taurus	None
EOG091202Z7	AGLA009761	Anoplophora glabripennis	None
EOG091202Z7	APLA004505	Agrilus planipennis	None
EOG091202Z7	TC033984	Tribolium castaneum	None
EOG091202Z7	LDEC011595	Leptinotarsa decemlineata	None
EOG091202Z7	YQE_12082	Dendroctonus ponderosae	None
EOG091202Z8	OTAU000114	Onthophagus taurus	Putative uncharacterized protein
EOG091202Z8	AGLA021705	Anoplophora glabripennis	Putative uncharacterized protein
EOG091202Z8	APLA010567	Agrilus planipennis	Putative uncharacterized protein
EOG091202Z8	TC000380	Tribolium castaneum	Putative uncharacterized protein
EOG091202Z8	LDEC014032	Leptinotarsa decemlineata	Putative uncharacterized protein

EOG091202Z8	YQE_01741	Dendroctonus ponderosae	Putative uncharacterized protein
EOG091202ZB	OTAU015074	Onthophagus taurus	copper ion binding
EOG091202ZB	AGLA003368	Anoplophora glabripennis	copper ion binding
EOG091202ZB	APLA001521	Agrilus planipennis	copper ion binding
EOG091202ZB	TC034624	Tribolium castaneum	copper ion binding
EOG091202ZB	LDEC000040	Leptinotarsa decemlineata	copper ion binding
EOG091202ZB	YQE_03787	Dendroctonus ponderosae	copper ion binding
EOG091202ZC	OTAU001906	Onthophagus taurus	Putative uncharacterized protein
EOG091202ZC	AGLA015121	Anoplophora glabripennis	Putative uncharacterized protein
EOG091202ZC	APLA001191	Agrilus planipennis	Putative uncharacterized protein
EOG091202ZC	TC005589	Tribolium castaneum	Putative uncharacterized protein
EOG091202ZC	LDEC011701	Leptinotarsa decemlineata	Putative uncharacterized protein
EOG091202ZC	YQE_07009	Dendroctonus ponderosae	Putative uncharacterized protein
EOG091202ZD	OTAU007326	Onthophagus taurus	Putative uncharacterized protein
EOG091202ZD	AGLA007773	Anoplophora glabripennis	Putative uncharacterized protein
EOG091202ZD	APLA008584	Agrilus planipennis	Putative uncharacterized protein
EOG091202ZD	TC001252	Tribolium castaneum	Putative uncharacterized protein
EOG091202ZD	LDEC017871	Leptinotarsa decemlineata	Putative uncharacterized protein
EOG091202ZD	YQE_02268	Dendroctonus ponderosae	Putative uncharacterized protein
EOG091202ZF	OTAU001947	Onthophagus taurus	Putative uncharacterized protein
EOG091202ZF	AGLA001240	Anoplophora glabripennis	Putative uncharacterized protein
EOG091202ZF	APLA000399	Agrilus planipennis	Putative uncharacterized protein
EOG091202ZF	TC006217	Tribolium castaneum	Putative uncharacterized protein
EOG091202ZF	LDEC011058	Leptinotarsa decemlineata	Putative uncharacterized protein
EOG091202ZF	YQE_12014	Dendroctonus ponderosae	Putative uncharacterized protein
EOG091202ZG	OTAU002174	Onthophagus taurus	Putative uncharacterized protein
EOG091202ZG	AGLA011332	Anoplophora glabripennis	Putative uncharacterized protein
EOG091202ZG	APLA012625	Agrilus planipennis	Putative uncharacterized protein
EOG091202ZG	TC004088	Tribolium castaneum	Putative uncharacterized protein
EOG091202ZG	LDEC005745	Leptinotarsa decemlineata	Putative uncharacterized protein
EOG091202ZG	YQE_10008	Dendroctonus ponderosae	Putative uncharacterized protein
EOG091202ZI	OTAU007879	Onthophagus taurus	Putative uncharacterized protein
EOG091202ZI	AGLA005479	Anoplophora glabripennis	Putative uncharacterized protein
EOG091202ZI	APLA010385	Agrilus planipennis	Putative uncharacterized protein
EOG091202ZI	TC014488	Tribolium castaneum	Putative uncharacterized protein
EOG091202ZI	LDEC003086	Leptinotarsa decemlineata	Putative uncharacterized protein
EOG091202ZI	YQE_02076	Dendroctonus ponderosae	Putative uncharacterized protein
EOG091202ZJ	OTAU002978	Onthophagus taurus	Putative uncharacterized protein
EOG091202ZJ	AGLA015433	Anoplophora glabripennis	Putative uncharacterized protein
EOG091202ZJ	APLA001019	Agrilus planipennis	Putative uncharacterized protein
EOG091202ZJ	TC009267	Tribolium castaneum	Putative uncharacterized protein
EOG091202ZJ	LDEC014616	Leptinotarsa decemlineata	Putative uncharacterized protein
EOG091202ZJ	YQE_11281	Dendroctonus ponderosae	Putative uncharacterized protein
EOG091202ZM	OTAU001617	Onthophagus taurus	Putative uncharacterized protein
EOG091202ZM	AGLA016356	Anoplophora glabripennis	Putative uncharacterized protein
EOG091202ZM	APLA005484	Agrilus planipennis	Putative uncharacterized protein
EOG091202ZM	TC005516	Tribolium castaneum	Putative uncharacterized protein
EOG091202ZM	LDEC006313	Leptinotarsa decemlineata	Putative uncharacterized protein
EOG091202ZM	YQE_07426	Dendroctonus ponderosae	Putative uncharacterized protein
EOG091202ZR	OTAU011779	Onthophagus taurus	Putative uncharacterized protein
EOG091202ZR	AGLA017715	Anoplophora glabripennis	Putative uncharacterized protein
EOG091202ZR	APLA001629	Agrilus planipennis	Putative uncharacterized protein
EOG091202ZR	TC009613	Tribolium castaneum	Putative uncharacterized protein
EOG091202ZR	LDEC009366	Leptinotarsa decemlineata	Putative uncharacterized protein
EOG091202ZR	YQE_05750	Dendroctonus ponderosae	Putative uncharacterized protein
EOG091202ZT	OTAU010366	Onthophagus taurus	Putative uncharacterized protein
EOG091202ZT	AGLA002968	Anoplophora glabripennis	Putative uncharacterized protein
EOG091202ZT	APLA004626	Agrilus planipennis	Putative uncharacterized protein
EOG091202ZT	TC000823	Tribolium castaneum	Putative uncharacterized protein
EOG091202ZT	LDEC006257	Leptinotarsa decemlineata	Putative uncharacterized protein
EOG091202ZT	YQE_11642	Dendroctonus ponderosae	Putative uncharacterized protein
EOG091202ZY	OTAU001293	Onthophagus taurus	Putative uncharacterized protein
EOG091202ZY	AGLA001145	Anoplophora glabripennis	Putative uncharacterized protein
EOG091202ZY	APLA006376	Agrilus planipennis	Putative uncharacterized protein
EOG091202ZY	TC005170	Tribolium castaneum	Putative uncharacterized protein
EOG091202ZY	LDEC015534	Leptinotarsa decemlineata	Putative uncharacterized protein
EOG091202ZY	YQE_03330	Dendroctonus ponderosae	Putative uncharacterized protein

EOG09120304	OTAU015650	Onthophagus taurus	Invected
EOG09120304	AGLA016154	Anoplophora glabripennis	Invected
EOG09120304	APLA004581	Agrilus planipennis	Invected
EOG09120304	TC016368	Tribolium castaneum	Invected
EOG09120304	LDEC016184	Leptinotarsa decemlineata	Invected
EOG09120304	YQE_10617	Dendroctonus ponderosae	Invected
EOG09120306	OTAU001540	Onthophagus taurus	Putative uncharacterized protein
EOG09120306	AGLA018510	Anoplophora glabripennis	Putative uncharacterized protein
EOG09120306	APLA012054	Agrilus planipennis	Putative uncharacterized protein
EOG09120306	TC005087	Tribolium castaneum	Putative uncharacterized protein
EOG09120306	LDEC005872	Leptinotarsa decemlineata	Putative uncharacterized protein
EOG09120306	YQE_12673	Dendroctonus ponderosae	Putative uncharacterized protein
EOG09120307	OTAU004270	Onthophagus taurus	Putative uncharacterized protein
EOG09120307	AGLA007198	Anoplophora glabripennis	Putative uncharacterized protein
EOG09120307	APLA001030	Agrilus planipennis	Putative uncharacterized protein
EOG09120307	TC002783	Tribolium castaneum	Putative uncharacterized protein
EOG09120307	LDEC006538	Leptinotarsa decemlineata	Putative uncharacterized protein
EOG09120307	YQE_09824	Dendroctonus ponderosae	Putative uncharacterized protein
EOG09120308	OTAU001478	Onthophagus taurus	Hormone receptor in 78-like protein
EOG09120308	AGLA001129	Anoplophora glabripennis	Hormone receptor in 78-like protein
EOG09120308	APLA006359	Agrilus planipennis	Hormone receptor in 78-like protein
EOG09120308	TC004598	Tribolium castaneum	Hormone receptor in 78-like protein
EOG09120308	LDEC014581	Leptinotarsa decemlineata	Hormone receptor in 78-like protein
EOG09120308	YQE_02452	Dendroctonus ponderosae	Hormone receptor in 78-like protein
EOG09120309	OTAU008749	Onthophagus taurus	Putative uncharacterized protein
EOG09120309	AGLA008560	Anoplophora glabripennis	Putative uncharacterized protein
EOG09120309	APLA005939	Agrilus planipennis	Putative uncharacterized protein
EOG09120309	TC006040	Tribolium castaneum	Putative uncharacterized protein
EOG09120309	LDEC008003	Leptinotarsa decemlineata	Putative uncharacterized protein
EOG09120309	YQE_11947	Dendroctonus ponderosae	Putative uncharacterized protein
EOG0912030D	OTAU004617	Onthophagus taurus	hydrolase activity
EOG0912030D	AGLA000525	Anoplophora glabripennis	hydrolase activity
EOG0912030D	APLA000217	Agrilus planipennis	hydrolase activity
EOG0912030D	TC032734	Tribolium castaneum	hydrolase activity
EOG0912030D	LDEC006840	Leptinotarsa decemlineata	hydrolase activity
EOG0912030D	YQE_10409	Dendroctonus ponderosae	hydrolase activity
EOG0912030E	OTAU013137	Onthophagus taurus	Putative uncharacterized protein
EOG0912030E	AGLA019002	Anoplophora glabripennis	Putative uncharacterized protein
EOG0912030E	APLA012090	Agrilus planipennis	Putative uncharacterized protein
EOG0912030E	TC005305	Tribolium castaneum	Putative uncharacterized protein
EOG0912030E	LDEC013279	Leptinotarsa decemlineata	Putative uncharacterized protein
EOG0912030E	YQE_12699	Dendroctonus ponderosae	Putative uncharacterized protein
EOG0912030G	OTAU003105	Onthophagus taurus	Putative uncharacterized protein
EOG0912030G	AGLA017226	Anoplophora glabripennis	Putative uncharacterized protein
EOG0912030G	APLA002639	Agrilus planipennis	Putative uncharacterized protein
EOG0912030G	TC012661	Tribolium castaneum	Putative uncharacterized protein
EOG0912030G	LDEC003857	Leptinotarsa decemlineata	Putative uncharacterized protein
EOG0912030G	YQE_04313	Dendroctonus ponderosae	Putative uncharacterized protein
EOG0912030H	OTAU007610	Onthophagus taurus	Putative uncharacterized protein
EOG0912030H	AGLA002016	Anoplophora glabripennis	Putative uncharacterized protein
EOG0912030H	APLA002826	Agrilus planipennis	Putative uncharacterized protein
EOG0912030H	TC006290	Tribolium castaneum	Putative uncharacterized protein
EOG0912030H	LDEC019757	Leptinotarsa decemlineata	Putative uncharacterized protein
EOG0912030H	YQE_09951	Dendroctonus ponderosae	Putative uncharacterized protein
EOG0912030K	OTAU007490	Onthophagus taurus	Putative uncharacterized protein
EOG0912030K	AGLA015576	Anoplophora glabripennis	Putative uncharacterized protein
EOG0912030K	APLA014870	Agrilus planipennis	Putative uncharacterized protein
EOG0912030K	TC015184	Tribolium castaneum	Putative uncharacterized protein
EOG0912030K	LDEC006937	Leptinotarsa decemlineata	Putative uncharacterized protein
EOG0912030K	YQE_10836	Dendroctonus ponderosae	Putative uncharacterized protein
EOG0912030M	OTAU007487	Onthophagus taurus	Replication factor C 40kD subunit
EOG0912030M	AGLA016289	Anoplophora glabripennis	Replication factor C 40kD subunit
EOG0912030M	APLA000884	Agrilus planipennis	Replication factor C 40kD subunit
EOG0912030M	TC010228	Tribolium castaneum	Replication factor C 40kD subunit
EOG0912030M	LDEC005050	Leptinotarsa decemlineata	Replication factor C 40kD subunit
EOG0912030M	YQE_02120	Dendroctonus ponderosae	Replication factor C 40kD subunit
EOG0912030N	OTAU011382	Onthophagus taurus	Putative uncharacterized protein

EOG0912030N	AGLA020073	Anoplophora glabripennis	Putative uncharacterized protein
EOG0912030N	APLA006844	Agrilus planipennis	Putative uncharacterized protein
EOG0912030N	TC013385	Tribolium castaneum	Putative uncharacterized protein
EOG0912030N	LDEC002637	Leptinotarsa decemlineata	Putative uncharacterized protein
EOG0912030N	YQE_10470	Dendroctonus ponderosae	Putative uncharacterized protein
EOG0912030O	OTAU013081	Onthophagus taurus	Putative uncharacterized protein
EOG0912030O	AGLA009293	Anoplophora glabripennis	Putative uncharacterized protein
EOG0912030O	APLA004507	Agrilus planipennis	Putative uncharacterized protein
EOG0912030O	TC006259	Tribolium castaneum	Putative uncharacterized protein
EOG0912030O	LDEC018341	Leptinotarsa decemlineata	Putative uncharacterized protein
EOG0912030O	YQE_12696	Dendroctonus ponderosae	Putative uncharacterized protein
EOG0912030P	OTAU000274	Onthophagus taurus	Putative uncharacterized protein
EOG0912030P	AGLA009865	Anoplophora glabripennis	Putative uncharacterized protein
EOG0912030P	APLA011718	Agrilus planipennis	Putative uncharacterized protein
EOG0912030P	TC014704	Tribolium castaneum	Putative uncharacterized protein
EOG0912030P	LDEC006269	Leptinotarsa decemlineata	Putative uncharacterized protein
EOG0912030P	YQE_03599	Dendroctonus ponderosae	Putative uncharacterized protein
EOG0912030R	OTAU004904	Onthophagus taurus	Putative uncharacterized protein
EOG0912030R	AGLA006693	Anoplophora glabripennis	Putative uncharacterized protein
EOG0912030R	APLA005504	Agrilus planipennis	Putative uncharacterized protein
EOG0912030R	TC001375	Tribolium castaneum	Putative uncharacterized protein
EOG0912030R	LDEC019593	Leptinotarsa decemlineata	Putative uncharacterized protein
EOG0912030R	YQE_07723	Dendroctonus ponderosae	Putative uncharacterized protein
EOG0912030W	OTAU000536	Onthophagus taurus	None
EOG0912030W	AGLA001470	Anoplophora glabripennis	None
EOG0912030W	APLA008734	Agrilus planipennis	None
EOG0912030W	TC034995	Tribolium castaneum	None
EOG0912030W	LDEC016174	Leptinotarsa decemlineata	None
EOG0912030W	YQE_01774	Dendroctonus ponderosae	None
EOG0912030Y	OTAU005487	Onthophagus taurus	Putative uncharacterized protein
EOG0912030Y	AGLA009553	Anoplophora glabripennis	Putative uncharacterized protein
EOG0912030Y	APLA004811	Agrilus planipennis	Putative uncharacterized protein
EOG0912030Y	TC014461	Tribolium castaneum	Putative uncharacterized protein
EOG0912030Y	LDEC002648	Leptinotarsa decemlineata	Putative uncharacterized protein
EOG0912030Y	YQE_10460	Dendroctonus ponderosae	Putative uncharacterized protein
EOG09120312	OTAU002738	Onthophagus taurus	Putative uncharacterized protein
EOG09120312	AGLA002185	Anoplophora glabripennis	Putative uncharacterized protein
EOG09120312	APLA003563	Agrilus planipennis	Putative uncharacterized protein
EOG09120312	TC010401	Tribolium castaneum	Putative uncharacterized protein
EOG09120312	LDEC019217	Leptinotarsa decemlineata	Putative uncharacterized protein
EOG09120312	YQE_06651	Dendroctonus ponderosae	Putative uncharacterized protein
EOG09120313	OTAU006648	Onthophagus taurus	Putative uncharacterized protein
EOG09120313	AGLA006889	Anoplophora glabripennis	Putative uncharacterized protein
EOG09120313	APLA003347	Agrilus planipennis	Putative uncharacterized protein
EOG09120313	TC000041	Tribolium castaneum	Putative uncharacterized protein
EOG09120313	LDEC004537	Leptinotarsa decemlineata	Putative uncharacterized protein
EOG09120313	YQE_10239	Dendroctonus ponderosae	Putative uncharacterized protein
EOG09120315	OTAU006814	Onthophagus taurus	Putative uncharacterized protein
EOG09120315	AGLA001154	Anoplophora glabripennis	Putative uncharacterized protein
EOG09120315	APLA012406	Agrilus planipennis	Putative uncharacterized protein
EOG09120315	TC001970	Tribolium castaneum	Putative uncharacterized protein
EOG09120315	LDEC001545	Leptinotarsa decemlineata	Putative uncharacterized protein
EOG09120315	YQE_02486	Dendroctonus ponderosae	Putative uncharacterized protein
EOG09120316	OTAU014970	Onthophagus taurus	Putative uncharacterized protein
EOG09120316	AGLA008679	Anoplophora glabripennis	Putative uncharacterized protein
EOG09120316	APLA002357	Agrilus planipennis	Putative uncharacterized protein
EOG09120316	TC010679	Tribolium castaneum	Putative uncharacterized protein
EOG09120316	LDEC019275	Leptinotarsa decemlineata	Putative uncharacterized protein
EOG09120316	YQE_08014	Dendroctonus ponderosae	Putative uncharacterized protein
EOG09120317	OTAU016030	Onthophagus taurus	Putative uncharacterized protein
EOG09120317	AGLA014967	Anoplophora glabripennis	Putative uncharacterized protein
EOG09120317	APLA004083	Agrilus planipennis	Putative uncharacterized protein
EOG09120317	TC003458	Tribolium castaneum	Putative uncharacterized protein
EOG09120317	LDEC001481	Leptinotarsa decemlineata	Putative uncharacterized protein
EOG09120317	YQE_09874	Dendroctonus ponderosae	Putative uncharacterized protein
EOG09120319	OTAU004121	Onthophagus taurus	Tamo
EOG09120319	AGLA002756	Anoplophora glabripennis	Tamo

EOG09120319	APLA001583	Agrilus planipennis	Tamo
EOG09120319	TC015946	Tribolium castaneum	Tamo
EOG09120319	LDEC015708	Leptinotarsa decemlineata	Tamo
EOG09120319	YQE_10625	Dendroctonus ponderosae	Tamo
EOG0912031B	OTAU016003	Onthophagus taurus	Putative uncharacterized protein
EOG0912031B	AGLA002811	Anoplophora glabripennis	Putative uncharacterized protein
EOG0912031B	APLA006029	Agrilus planipennis	Putative uncharacterized protein
EOG0912031B	TC009780	Tribolium castaneum	Putative uncharacterized protein
EOG0912031B	LDEC004068	Leptinotarsa decemlineata	Putative uncharacterized protein
EOG0912031B	YQE_11550	Dendroctonus ponderosae	Putative uncharacterized protein
EOG0912031C	OTAU001726	Onthophagus taurus	Putative uncharacterized protein
EOG0912031C	AGLA009306	Anoplophora glabripennis	Putative uncharacterized protein
EOG0912031C	APLA012289	Agrilus planipennis	Putative uncharacterized protein
EOG0912031C	TC006736	Tribolium castaneum	Putative uncharacterized protein
EOG0912031C	LDEC003279	Leptinotarsa decemlineata	Putative uncharacterized protein
EOG0912031C	YQE_12093	Dendroctonus ponderosae	Putative uncharacterized protein
EOG0912031D	OTAU000804	Onthophagus taurus	Putative uncharacterized protein
EOG0912031D	AGLA002443	Anoplophora glabripennis	Putative uncharacterized protein
EOG0912031D	APLA001970	Agrilus planipennis	Putative uncharacterized protein
EOG0912031D	TC004574	Tribolium castaneum	Putative uncharacterized protein
EOG0912031D	LDEC001762	Leptinotarsa decemlineata	Putative uncharacterized protein
EOG0912031D	YQE_06511	Dendroctonus ponderosae	Putative uncharacterized protein
EOG0912031F	OTAU000275	Onthophagus taurus	Putative uncharacterized protein
EOG0912031F	AGLA011401	Anoplophora glabripennis	Putative uncharacterized protein
EOG0912031F	APLA012874	Agrilus planipennis	Putative uncharacterized protein
EOG0912031F	TC013405	Tribolium castaneum	Putative uncharacterized protein
EOG0912031F	LDEC006522	Leptinotarsa decemlineata	Putative uncharacterized protein
EOG0912031F	YQE_07216	Dendroctonus ponderosae	Putative uncharacterized protein
EOG0912031H	OTAU008217	Onthophagus taurus	Inhibitor of apoptosis 2 protein
EOG0912031H	AGLA001860	Anoplophora glabripennis	Inhibitor of apoptosis 2 protein
EOG0912031H	APLA011680	Agrilus planipennis	Inhibitor of apoptosis 2 protein
EOG0912031H	TC001189	Tribolium castaneum	Inhibitor of apoptosis 2 protein
EOG0912031H	LDEC008879	Leptinotarsa decemlineata	Inhibitor of apoptosis 2 protein
EOG0912031H	YQE_03892	Dendroctonus ponderosae	Inhibitor of apoptosis 2 protein
EOG0912031K	OTAU000048	Onthophagus taurus	Putative uncharacterized protein
EOG0912031K	AGLA007902	Anoplophora glabripennis	Putative uncharacterized protein
EOG0912031K	APLA000839	Agrilus planipennis	Putative uncharacterized protein
EOG0912031K	TC014642	Tribolium castaneum	Putative uncharacterized protein
EOG0912031K	LDEC012327	Leptinotarsa decemlineata	Putative uncharacterized protein
EOG0912031K	YQE_07199	Dendroctonus ponderosae	Putative uncharacterized protein
EOG0912031O	OTAU003120	Onthophagus taurus	Putative uncharacterized protein
EOG0912031O	AGLA021666	Anoplophora glabripennis	Putative uncharacterized protein
EOG0912031O	APLA014645	Agrilus planipennis	Putative uncharacterized protein
EOG0912031O	TC000044	Tribolium castaneum	Putative uncharacterized protein
EOG0912031O	LDEC005031	Leptinotarsa decemlineata	Putative uncharacterized protein
EOG0912031O	YQE_07919	Dendroctonus ponderosae	Putative uncharacterized protein
EOG0912031Q	OTAU001565	Onthophagus taurus	None
EOG0912031Q	AGLA004782	Anoplophora glabripennis	None
EOG0912031Q	APLA000921	Agrilus planipennis	None
EOG0912031Q	TC034075	Tribolium castaneum	None
EOG0912031Q	LDEC008651	Leptinotarsa decemlineata	None
EOG0912031Q	YQE_05799	Dendroctonus ponderosae	None
EOG0912031T	OTAU004662	Onthophagus taurus	Putative uncharacterized protein
EOG0912031T	AGLA007919	Anoplophora glabripennis	Putative uncharacterized protein
EOG0912031T	APLA003735	Agrilus planipennis	Putative uncharacterized protein
EOG0912031T	TC010543	Tribolium castaneum	Putative uncharacterized protein
EOG0912031T	LDEC011641	Leptinotarsa decemlineata	Putative uncharacterized protein
EOG0912031T	YQE_07912	Dendroctonus ponderosae	Putative uncharacterized protein
EOG0912031W	OTAU003119	Onthophagus taurus	Ubiquitin-specific protease
EOG0912031W	AGLA021682	Anoplophora glabripennis	Ubiquitin-specific protease
EOG0912031W	APLA011490	Agrilus planipennis	Ubiquitin-specific protease
EOG0912031W	TC000045	Tribolium castaneum	Ubiquitin-specific protease
EOG0912031W	LDEC005018	Leptinotarsa decemlineata	Ubiquitin-specific protease
EOG0912031W	YQE_07918	Dendroctonus ponderosae	Ubiquitin-specific protease
EOG0912031X	OTAU015034	Onthophagus taurus	Putative uncharacterized protein
EOG0912031X	AGLA018336	Anoplophora glabripennis	Putative uncharacterized protein
EOG0912031X	APLA000370	Agrilus planipennis	Putative uncharacterized protein

EOG0912031X	TC005869	Tribolium castaneum	Putative uncharacterized protein
EOG0912031X	LDEC002449	Leptinotarsa decemlineata	Putative uncharacterized protein
EOG0912031X	YQE_05689	Dendroctonus ponderosae	Putative uncharacterized protein
EOG0912031Y	OTAU004197	Onthophagus taurus	Putative uncharacterized protein
EOG0912031Y	AGLA009659	Anoplophora glabripennis	Putative uncharacterized protein
EOG0912031Y	APLA012883	Agrilus planipennis	Putative uncharacterized protein
EOG0912031Y	TC010900	Tribolium castaneum	Putative uncharacterized protein
EOG0912031Y	LDEC008704	Leptinotarsa decemlineata	Putative uncharacterized protein
EOG0912031Y	YQE_09536	Dendroctonus ponderosae	Putative uncharacterized protein
EOG0912031Z	OTAU005078	Onthophagus taurus	Putative uncharacterized protein
EOG0912031Z	AGLA008868	Anoplophora glabripennis	Putative uncharacterized protein
EOG0912031Z	APLA004441	Agrilus planipennis	Putative uncharacterized protein
EOG0912031Z	TC005313	Tribolium castaneum	Putative uncharacterized protein
EOG0912031Z	LDEC004927	Leptinotarsa decemlineata	Putative uncharacterized protein
EOG0912031Z	YQE_10184	Dendroctonus ponderosae	Putative uncharacterized protein
EOG09120320	OTAU001922	Onthophagus taurus	Putative uncharacterized protein
EOG09120320	AGLA010661	Anoplophora glabripennis	Putative uncharacterized protein
EOG09120320	APLA001238	Agrilus planipennis	Putative uncharacterized protein
EOG09120320	TC005927	Tribolium castaneum	Putative uncharacterized protein
EOG09120320	LDEC022573	Leptinotarsa decemlineata	Putative uncharacterized protein
EOG09120320	YQE_12078	Dendroctonus ponderosae	Putative uncharacterized protein
EOG09120324	OTAU011774	Onthophagus taurus	Putative uncharacterized protein
EOG09120324	AGLA017708	Anoplophora glabripennis	Putative uncharacterized protein
EOG09120324	APLA001567	Agrilus planipennis	Putative uncharacterized protein
EOG09120324	TC009195	Tribolium castaneum	Putative uncharacterized protein
EOG09120324	LDEC009350	Leptinotarsa decemlineata	Putative uncharacterized protein
EOG09120324	YQE_05755	Dendroctonus ponderosae	Putative uncharacterized protein
EOG09120325	OTAU014575	Onthophagus taurus	Putative uncharacterized protein
EOG09120325	AGLA005137	Anoplophora glabripennis	Putative uncharacterized protein
EOG09120325	APLA007464	Agrilus planipennis	Putative uncharacterized protein
EOG09120325	TC007421	Tribolium castaneum	Putative uncharacterized protein
EOG09120325	LDEC003815	Leptinotarsa decemlineata	Putative uncharacterized protein
EOG09120325	YQE_10922	Dendroctonus ponderosae	Putative uncharacterized protein
EOG09120328	OTAU015042	Onthophagus taurus	GPI-anchor transamidase activity
EOG09120328	AGLA015710	Anoplophora glabripennis	GPI-anchor transamidase activity
EOG09120328	APLA003428	Agrilus planipennis	GPI-anchor transamidase activity
EOG09120328	TC033585	Tribolium castaneum	GPI-anchor transamidase activity
EOG09120328	LDEC004434	Leptinotarsa decemlineata	GPI-anchor transamidase activity
EOG09120328	YQE_12452	Dendroctonus ponderosae	GPI-anchor transamidase activity
EOG0912032C	OTAU003196	Onthophagus taurus	Putative uncharacterized protein
EOG0912032C	AGLA007370	Anoplophora glabripennis	Putative uncharacterized protein
EOG0912032C	APLA002755	Agrilus planipennis	Putative uncharacterized protein
EOG0912032C	TC013502	Tribolium castaneum	Putative uncharacterized protein
EOG0912032C	LDEC008338	Leptinotarsa decemlineata	Putative uncharacterized protein
EOG0912032C	YQE_06213	Dendroctonus ponderosae	Putative uncharacterized protein
EOG0912032E	OTAU006879	Onthophagus taurus	Putative uncharacterized protein
EOG0912032E	AGLA011594	Anoplophora glabripennis	Putative uncharacterized protein
EOG0912032E	APLA006002	Agrilus planipennis	Putative uncharacterized protein
EOG0912032E	TC009637	Tribolium castaneum	Putative uncharacterized protein
EOG0912032E	LDEC011987	Leptinotarsa decemlineata	Putative uncharacterized protein
EOG0912032E	YQE_10556	Dendroctonus ponderosae	Putative uncharacterized protein
EOG0912032I	OTAU011806	Onthophagus taurus	Putative uncharacterized protein
EOG0912032I	AGLA013755	Anoplophora glabripennis	Putative uncharacterized protein
EOG0912032I	APLA011244	Agrilus planipennis	Putative uncharacterized protein
EOG0912032I	TC015632	Tribolium castaneum	Putative uncharacterized protein
EOG0912032I	LDEC012235	Leptinotarsa decemlineata	Putative uncharacterized protein
EOG0912032I	YQE_00023	Dendroctonus ponderosae	Putative uncharacterized protein
EOG0912032K	OTAU004371	Onthophagus taurus	Putative uncharacterized protein
EOG0912032K	AGLA000339	Anoplophora glabripennis	Putative uncharacterized protein
EOG0912032K	APLA006426	Agrilus planipennis	Putative uncharacterized protein
EOG0912032K	TC010863	Tribolium castaneum	Putative uncharacterized protein
EOG0912032K	LDEC003749	Leptinotarsa decemlineata	Putative uncharacterized protein
EOG0912032K	YQE_02495	Dendroctonus ponderosae	Putative uncharacterized protein
EOG0912032L	OTAU005603	Onthophagus taurus	Putative uncharacterized protein
EOG0912032L	AGLA013240	Anoplophora glabripennis	Putative uncharacterized protein
EOG0912032L	APLA010182	Agrilus planipennis	Putative uncharacterized protein
EOG0912032L	TC009259	Tribolium castaneum	Putative uncharacterized protein

EOG0912032L	LDEC012857	Leptinotarsa decemlineata	Putative uncharacterized protein
EOG0912032L	YQE_11310	Dendroctonus ponderosae	Putative uncharacterized protein
EOG0912032O	OTAU005079	Onthophagus taurus	Putative uncharacterized protein
EOG0912032O	AGLA008867	Anoplophora glabripennis	Putative uncharacterized protein
EOG0912032O	APLA004438	Agrilus planipennis	Putative uncharacterized protein
EOG0912032O	TC005314	Tribolium castaneum	Putative uncharacterized protein
EOG0912032O	LDEC004929	Leptinotarsa decemlineata	Putative uncharacterized protein
EOG0912032O	YQE_10185	Dendroctonus ponderosae	Putative uncharacterized protein
EOG0912032Q	OTAU008890	Onthophagus taurus	Putative uncharacterized protein
EOG0912032Q	AGLA011155	Anoplophora glabripennis	Putative uncharacterized protein
EOG0912032Q	APLA007890	Agrilus planipennis	Putative uncharacterized protein
EOG0912032Q	TC011313	Tribolium castaneum	Putative uncharacterized protein
EOG0912032Q	LDEC018096	Leptinotarsa decemlineata	Putative uncharacterized protein
EOG0912032Q	YQE_11605	Dendroctonus ponderosae	Putative uncharacterized protein
EOG0912032W	OTAU000629	Onthophagus taurus	Putative uncharacterized protein
EOG0912032W	AGLA009786	Anoplophora glabripennis	Putative uncharacterized protein
EOG0912032W	APLA002322	Agrilus planipennis	Putative uncharacterized protein
EOG0912032W	TC012209	Tribolium castaneum	Putative uncharacterized protein
EOG0912032W	LDEC003824	Leptinotarsa decemlineata	Putative uncharacterized protein
EOG0912032W	YQE_12896	Dendroctonus ponderosae	Putative uncharacterized protein
EOG0912032X	OTAU010463	Onthophagus taurus	Putative uncharacterized protein
EOG0912032X	AGLA005165	Anoplophora glabripennis	Putative uncharacterized protein
EOG0912032X	APLA004197	Agrilus planipennis	Putative uncharacterized protein
EOG0912032X	TC007103	Tribolium castaneum	Putative uncharacterized protein
EOG0912032X	LDEC004026	Leptinotarsa decemlineata	Putative uncharacterized protein
EOG0912032X	YQE_09032	Dendroctonus ponderosae	Putative uncharacterized protein
EOG0912032Z	OTAU009375	Onthophagus taurus	Putative uncharacterized protein
EOG0912032Z	AGLA010930	Anoplophora glabripennis	Putative uncharacterized protein
EOG0912032Z	APLA013902	Agrilus planipennis	Putative uncharacterized protein
EOG0912032Z	TC013003	Tribolium castaneum	Putative uncharacterized protein
EOG0912032Z	LDEC007222	Leptinotarsa decemlineata	Putative uncharacterized protein
EOG0912032Z	YQE_02069	Dendroctonus ponderosae	Putative uncharacterized protein
EOG09120330	OTAU000481	Onthophagus taurus	Putative uncharacterized protein
EOG09120330	AGLA020051	Anoplophora glabripennis	Putative uncharacterized protein
EOG09120330	APLA003311	Agrilus planipennis	Putative uncharacterized protein
EOG09120330	TC010961	Tribolium castaneum	Putative uncharacterized protein
EOG09120330	LDEC004024	Leptinotarsa decemlineata	Putative uncharacterized protein
EOG09120330	YQE_10027	Dendroctonus ponderosae	Putative uncharacterized protein
EOG09120333	OTAU002644	Onthophagus taurus	Putative uncharacterized protein
EOG09120333	AGLA010799	Anoplophora glabripennis	Putative uncharacterized protein
EOG09120333	APLA013129	Agrilus planipennis	Putative uncharacterized protein
EOG09120333	TC014393	Tribolium castaneum	Putative uncharacterized protein
EOG09120333	LDEC011732	Leptinotarsa decemlineata	Putative uncharacterized protein
EOG09120333	YQE_06169	Dendroctonus ponderosae	Putative uncharacterized protein
EOG09120334	OTAU011344	Onthophagus taurus	Putative uncharacterized protein
EOG09120334	AGLA004604	Anoplophora glabripennis	Putative uncharacterized protein
EOG09120334	APLA008694	Agrilus planipennis	Putative uncharacterized protein
EOG09120334	TC011991	Tribolium castaneum	Putative uncharacterized protein
EOG09120334	LDEC017428	Leptinotarsa decemlineata	Putative uncharacterized protein
EOG09120334	YQE_10083	Dendroctonus ponderosae	Putative uncharacterized protein
EOG09120337	OTAU007162	Onthophagus taurus	Knot
EOG09120337	AGLA002392	Anoplophora glabripennis	Knot
EOG09120337	APLA007445	Agrilus planipennis	Knot
EOG09120337	TC001270	Tribolium castaneum	Knot
EOG09120337	LDEC007542	Leptinotarsa decemlineata	Knot
EOG09120337	YQE_08127	Dendroctonus ponderosae	Knot
EOG09120339	OTAU004718	Onthophagus taurus	Putative uncharacterized protein
EOG09120339	AGLA018969	Anoplophora glabripennis	Putative uncharacterized protein
EOG09120339	APLA013139	Agrilus planipennis	Putative uncharacterized protein
EOG09120339	TC012411	Tribolium castaneum	Putative uncharacterized protein
EOG09120339	LDEC018368	Leptinotarsa decemlineata	Putative uncharacterized protein
EOG09120339	YQE_12877	Dendroctonus ponderosae	Putative uncharacterized protein
EOG0912033A	OTAU014840	Onthophagus taurus	ATP binding
EOG0912033A	AGLA007454	Anoplophora glabripennis	ATP binding
EOG0912033A	APLA007948	Agrilus planipennis	ATP binding
EOG0912033A	TC032012	Tribolium castaneum	ATP binding
EOG0912033A	LDEC010338	Leptinotarsa decemlineata	ATP binding

EOG0912033A	YQE_02361	Dendroctonus ponderosae	ATP binding
EOG0912033G	OTAU011348	Onthophagus taurus	Putative uncharacterized protein
EOG0912033G	AGLA020659	Anoplophora glabripennis	Putative uncharacterized protein
EOG0912033G	APLA006619	Agrilus planipennis	Putative uncharacterized protein
EOG0912033G	TC010335	Tribolium castaneum	Putative uncharacterized protein
EOG0912033G	LDEC007079	Leptinotarsa decemlineata	Putative uncharacterized protein
EOG0912033G	YQE_02154	Dendroctonus ponderosae	Putative uncharacterized protein
EOG0912033J	OTAU004160	Onthophagus taurus	Putative uncharacterized protein
EOG0912033J	AGLA011888	Anoplophora glabripennis	Putative uncharacterized protein
EOG0912033J	APLA005732	Agrilus planipennis	Putative uncharacterized protein
EOG0912033J	TC010595	Tribolium castaneum	Putative uncharacterized protein
EOG0912033J	LDEC014659	Leptinotarsa decemlineata	Putative uncharacterized protein
EOG0912033J	YQE_10514	Dendroctonus ponderosae	Putative uncharacterized protein
EOG0912033K	OTAU008339	Onthophagus taurus	Putative uncharacterized protein
EOG0912033K	AGLA011192	Anoplophora glabripennis	Putative uncharacterized protein
EOG0912033K	APLA007675	Agrilus planipennis	Putative uncharacterized protein
EOG0912033K	TC011650	Tribolium castaneum	Putative uncharacterized protein
EOG0912033K	LDEC023114	Leptinotarsa decemlineata	Putative uncharacterized protein
EOG0912033K	YQE_07954	Dendroctonus ponderosae	Putative uncharacterized protein
EOG0912033L	OTAU001004	Onthophagus taurus	Putative uncharacterized protein
EOG0912033L	AGLA002447	Anoplophora glabripennis	Putative uncharacterized protein
EOG0912033L	APLA004316	Agrilus planipennis	Putative uncharacterized protein
EOG0912033L	TC000408	Tribolium castaneum	Putative uncharacterized protein
EOG0912033L	LDEC019601	Leptinotarsa decemlineata	Putative uncharacterized protein
EOG0912033L	YQE_10785	Dendroctonus ponderosae	Putative uncharacterized protein
EOG0912033N	OTAU001332	Onthophagus taurus	Serine protease P53
EOG0912033N	AGLA011263	Anoplophora glabripennis	Serine protease P53
EOG0912033N	APLA003523	Agrilus planipennis	Serine protease P53
EOG0912033N	TC004635	Tribolium castaneum	Serine protease P53
EOG0912033N	LDEC003783	Leptinotarsa decemlineata	Serine protease P53
EOG0912033N	YQE_02518	Dendroctonus ponderosae	Serine protease P53
EOG0912033Q	OTAU005456	Onthophagus taurus	Putative uncharacterized protein
EOG0912033Q	AGLA010554	Anoplophora glabripennis	Putative uncharacterized protein
EOG0912033Q	APLA002692	Agrilus planipennis	Putative uncharacterized protein
EOG0912033Q	TC010924	Tribolium castaneum	Putative uncharacterized protein
EOG0912033Q	LDEC005948	Leptinotarsa decemlineata	Putative uncharacterized protein
EOG0912033Q	YQE_08232	Dendroctonus ponderosae	Putative uncharacterized protein
EOG0912033T	OTAU004361	Onthophagus taurus	Putative uncharacterized protein
EOG0912033T	AGLA000410	Anoplophora glabripennis	Putative uncharacterized protein
EOG0912033T	APLA010591	Agrilus planipennis	Putative uncharacterized protein
EOG0912033T	TC010825	Tribolium castaneum	Putative uncharacterized protein
EOG0912033T	LDEC018936	Leptinotarsa decemlineata	Putative uncharacterized protein
EOG0912033T	YQE_09111	Dendroctonus ponderosae	Putative uncharacterized protein
EOG0912033U	OTAU001118	Onthophagus taurus	Putative uncharacterized protein
EOG0912033U	AGLA001393	Anoplophora glabripennis	Putative uncharacterized protein
EOG0912033U	APLA006737	Agrilus planipennis	Putative uncharacterized protein
EOG0912033U	TC000395	Tribolium castaneum	Putative uncharacterized protein
EOG0912033U	LDEC018942	Leptinotarsa decemlineata	Putative uncharacterized protein
EOG0912033U	YQE_10707	Dendroctonus ponderosae	Putative uncharacterized protein
EOG0912033V	OTAU001099	Onthophagus taurus	None
EOG0912033V	AGLA008101	Anoplophora glabripennis	None
EOG0912033V	APLA001929	Agrilus planipennis	None
EOG0912033V	TC034557	Tribolium castaneum	None
EOG0912033V	LDEC014285	Leptinotarsa decemlineata	None
EOG0912033V	YQE_06496	Dendroctonus ponderosae	None
EOG0912033W	OTAU017120	Onthophagus taurus	Putative uncharacterized protein
EOG0912033W	AGLA006677	Anoplophora glabripennis	Putative uncharacterized protein
EOG0912033W	APLA004337	Agrilus planipennis	Putative uncharacterized protein
EOG0912033W	TC000317	Tribolium castaneum	Putative uncharacterized protein
EOG0912033W	LDEC001614	Leptinotarsa decemlineata	Putative uncharacterized protein
EOG0912033W	YQE_07068	Dendroctonus ponderosae	Putative uncharacterized protein
EOG09120341	OTAU010912	Onthophagus taurus	Putative uncharacterized protein
EOG09120341	AGLA013715	Anoplophora glabripennis	Putative uncharacterized protein
EOG09120341	APLA008750	Agrilus planipennis	Putative uncharacterized protein
EOG09120341	TC014971	Tribolium castaneum	Putative uncharacterized protein
EOG09120341	LDEC004742	Leptinotarsa decemlineata	Putative uncharacterized protein
EOG09120341	YQE_11863	Dendroctonus ponderosae	Putative uncharacterized protein

EOG09120345	OTAU010902	Onthophagus taurus	Putative uncharacterized protein
EOG09120345	AGLA003480	Anoplophora glabripennis	Putative uncharacterized protein
EOG09120345	APLA002550	Agrilus planipennis	Putative uncharacterized protein
EOG09120345	TC015835	Tribolium castaneum	Putative uncharacterized protein
EOG09120345	LDEC008360	Leptinotarsa decemlineata	Putative uncharacterized protein
EOG09120345	YQE_02175	Dendroctonus ponderosae	Putative uncharacterized protein
EOG09120346	OTAU006822	Onthophagus taurus	Annexin
EOG09120346	AGLA008584	Anoplophora glabripennis	Annexin
EOG09120346	APLA000108	Agrilus planipennis	Annexin
EOG09120346	TC010520	Tribolium castaneum	Annexin
EOG09120346	LDEC002683	Leptinotarsa decemlineata	Annexin
EOG09120346	YQE_04549	Dendroctonus ponderosae	Annexin
EOG09120347	OTAU015334	Onthophagus taurus	DNA primase
EOG09120347	AGLA001955	Anoplophora glabripennis	DNA primase
EOG09120347	APLA008659	Agrilus planipennis	DNA primase
EOG09120347	TC011690	Tribolium castaneum	DNA primase
EOG09120347	LDEC015643	Leptinotarsa decemlineata	DNA primase
EOG09120347	YQE_02897	Dendroctonus ponderosae	DNA primase
EOG0912034A	OTAU000776	Onthophagus taurus	Putative uncharacterized protein
EOG0912034A	AGLA008594	Anoplophora glabripennis	Putative uncharacterized protein
EOG0912034A	APLA014414	Agrilus planipennis	Putative uncharacterized protein
EOG0912034A	TC010267	Tribolium castaneum	Putative uncharacterized protein
EOG0912034A	LDEC022147	Leptinotarsa decemlineata	Putative uncharacterized protein
EOG0912034A	YQE_01787	Dendroctonus ponderosae	Putative uncharacterized protein
EOG0912034B	OTAU011530	Onthophagus taurus	Putative uncharacterized protein
EOG0912034B	AGLA000469	Anoplophora glabripennis	Putative uncharacterized protein
EOG0912034B	APLA008615	Agrilus planipennis	Putative uncharacterized protein
EOG0912034B	TC007967	Tribolium castaneum	Putative uncharacterized protein
EOG0912034B	LDEC000955	Leptinotarsa decemlineata	Putative uncharacterized protein
EOG0912034B	YQE_10807	Dendroctonus ponderosae	Putative uncharacterized protein
EOG0912034D	OTAU008734	Onthophagus taurus	Putative uncharacterized protein
EOG0912034D	AGLA016658	Anoplophora glabripennis	Putative uncharacterized protein
EOG0912034D	APLA000147	Agrilus planipennis	Putative uncharacterized protein
EOG0912034D	TC003323	Tribolium castaneum	Putative uncharacterized protein
EOG0912034D	LDEC017757	Leptinotarsa decemlineata	Putative uncharacterized protein
EOG0912034D	YQE_03716	Dendroctonus ponderosae	Putative uncharacterized protein
EOG0912034F	OTAU011939	Onthophagus taurus	None
EOG0912034F	AGLA004340	Anoplophora glabripennis	None
EOG0912034F	APLA011720	Agrilus planipennis	None
EOG0912034F	TC033135	Tribolium castaneum	None
EOG0912034F	LDEC008747	Leptinotarsa decemlineata	None
EOG0912034F	YQE_07559	Dendroctonus ponderosae	None
EOG0912034G	OTAU013583	Onthophagus taurus	Putative uncharacterized protein
EOG0912034G	AGLA008012	Anoplophora glabripennis	Putative uncharacterized protein
EOG0912034G	APLA009138	Agrilus planipennis	Putative uncharacterized protein
EOG0912034G	TC015310	Tribolium castaneum	Putative uncharacterized protein
EOG0912034G	LDEC008794	Leptinotarsa decemlineata	Putative uncharacterized protein
EOG0912034G	YQE_04671	Dendroctonus ponderosae	Putative uncharacterized protein
EOG0912034M	OTAU006836	Onthophagus taurus	Putative uncharacterized protein
EOG0912034M	AGLA012767	Anoplophora glabripennis	Putative uncharacterized protein
EOG0912034M	APLA004830	Agrilus planipennis	Putative uncharacterized protein
EOG0912034M	TC014608	Tribolium castaneum	Putative uncharacterized protein
EOG0912034M	LDEC014507	Leptinotarsa decemlineata	Putative uncharacterized protein
EOG0912034M	YQE_07263	Dendroctonus ponderosae	Putative uncharacterized protein
EOG0912034O	OTAU004024	Onthophagus taurus	Putative uncharacterized protein
EOG0912034O	AGLA001440	Anoplophora glabripennis	Putative uncharacterized protein
EOG0912034O	APLA015473	Agrilus planipennis	Putative uncharacterized protein
EOG0912034O	TC002490	Tribolium castaneum	Putative uncharacterized protein
EOG0912034O	LDEC003347	Leptinotarsa decemlineata	Putative uncharacterized protein
EOG0912034O	YQE_02645	Dendroctonus ponderosae	Putative uncharacterized protein
EOG0912034R	OTAU008036	Onthophagus taurus	Putative uncharacterized protein
EOG0912034R	AGLA016611	Anoplophora glabripennis	Putative uncharacterized protein
EOG0912034R	APLA001423	Agrilus planipennis	Putative uncharacterized protein
EOG0912034R	TC006564	Tribolium castaneum	Putative uncharacterized protein
EOG0912034R	LDEC009838	Leptinotarsa decemlineata	Putative uncharacterized protein
EOG0912034R	YQE_06974	Dendroctonus ponderosae	Putative uncharacterized protein
EOG0912034S	OTAU005472	Onthophagus taurus	Putative uncharacterized protein

EOG0912034S	AGLA010822	Anoplophora glabripennis	Putative uncharacterized protein
EOG0912034S	APLA003618	Agrilus planipennis	Putative uncharacterized protein
EOG0912034S	TC013743	Tribolium castaneum	Putative uncharacterized protein
EOG0912034S	LDEC007263	Leptinotarsa decemlineata	Putative uncharacterized protein
EOG0912034S	YQE_06325	Dendroctonus ponderosae	Putative uncharacterized protein
EOG0912034V	OTAU005910	Onthophagus taurus	Putative uncharacterized protein
EOG0912034V	AGLA017325	Anoplophora glabripennis	Putative uncharacterized protein
EOG0912034V	APLA004864	Agrilus planipennis	Putative uncharacterized protein
EOG0912034V	TC030591	Tribolium castaneum	Putative uncharacterized protein
EOG0912034V	LDEC003465	Leptinotarsa decemlineata	Putative uncharacterized protein
EOG0912034V	YQE_08992	Dendroctonus ponderosae	Putative uncharacterized protein
EOG0912034Z	OTAU011831	Onthophagus taurus	None
EOG0912034Z	AGLA012829	Anoplophora glabripennis	None
EOG0912034Z	APLA006140	Agrilus planipennis	None
EOG0912034Z	TC033019	Tribolium castaneum	None
EOG0912034Z	LDEC005985	Leptinotarsa decemlineata	None
EOG0912034Z	YQE_06223	Dendroctonus ponderosae	None
EOG09120350	OTAU010659	Onthophagus taurus	Putative uncharacterized protein
EOG09120350	AGLA005423	Anoplophora glabripennis	Putative uncharacterized protein
EOG09120350	APLA004415	Agrilus planipennis	Putative uncharacterized protein
EOG09120350	TC005570	Tribolium castaneum	Putative uncharacterized protein
EOG09120350	LDEC018048	Leptinotarsa decemlineata	Putative uncharacterized protein
EOG09120350	YQE_12219	Dendroctonus ponderosae	Putative uncharacterized protein
EOG09120353	OTAU002726	Onthophagus taurus	Putative uncharacterized protein
EOG09120353	AGLA000048	Anoplophora glabripennis	Putative uncharacterized protein
EOG09120353	APLA011442	Agrilus planipennis	Putative uncharacterized protein
EOG09120353	TC014174	Tribolium castaneum	Putative uncharacterized protein
EOG09120353	LDEC001623	Leptinotarsa decemlineata	Putative uncharacterized protein
EOG09120353	YQE_01651	Dendroctonus ponderosae	Putative uncharacterized protein
EOG09120355	OTAU006244	Onthophagus taurus	Putative uncharacterized protein
EOG09120355	AGLA011642	Anoplophora glabripennis	Putative uncharacterized protein
EOG09120355	APLA009332	Agrilus planipennis	Putative uncharacterized protein
EOG09120355	TC009284	Tribolium castaneum	Putative uncharacterized protein
EOG09120355	LDEC007446	Leptinotarsa decemlineata	Putative uncharacterized protein
EOG09120355	YQE_08724	Dendroctonus ponderosae	Putative uncharacterized protein
EOG09120356	OTAU000442	Onthophagus taurus	Putative uncharacterized protein
EOG09120356	AGLA004855	Anoplophora glabripennis	Putative uncharacterized protein
EOG09120356	APLA006510	Agrilus planipennis	Putative uncharacterized protein
EOG09120356	TC002529	Tribolium castaneum	Putative uncharacterized protein
EOG09120356	LDEC017755	Leptinotarsa decemlineata	Putative uncharacterized protein
EOG09120356	YQE_09997	Dendroctonus ponderosae	Putative uncharacterized protein
EOG09120357	OTAU015236	Onthophagus taurus	tRNA pseudouridine synthase A 2
EOG09120357	AGLA002942	Anoplophora glabripennis	tRNA pseudouridine synthase A 2
EOG09120357	APLA004551	Agrilus planipennis	tRNA pseudouridine synthase A 2
EOG09120357	TC004829	Tribolium castaneum	tRNA pseudouridine synthase A 2
EOG09120357	LDEC002422	Leptinotarsa decemlineata	tRNA pseudouridine synthase A 2
EOG09120357	YQE_11899	Dendroctonus ponderosae	tRNA pseudouridine synthase A 2
EOG0912035E	OTAU007991	Onthophagus taurus	Putative uncharacterized protein
EOG0912035E	AGLA006263	Anoplophora glabripennis	Putative uncharacterized protein
EOG0912035E	APLA000760	Agrilus planipennis	Putative uncharacterized protein
EOG0912035E	TC003370	Tribolium castaneum	Putative uncharacterized protein
EOG0912035E	LDEC010610	Leptinotarsa decemlineata	Putative uncharacterized protein
EOG0912035E	YQE_12371	Dendroctonus ponderosae	Putative uncharacterized protein
EOG0912035F	OTAU006634	Onthophagus taurus	Putative uncharacterized protein
EOG0912035F	AGLA002027	Anoplophora glabripennis	Putative uncharacterized protein
EOG0912035F	APLA010333	Agrilus planipennis	Putative uncharacterized protein
EOG0912035F	TC003964	Tribolium castaneum	Putative uncharacterized protein
EOG0912035F	LDEC013415	Leptinotarsa decemlineata	Putative uncharacterized protein
EOG0912035F	YQE_02648	Dendroctonus ponderosae	Putative uncharacterized protein
EOG0912035H	OTAU007191	Onthophagus taurus	Putative uncharacterized protein
EOG0912035H	AGLA006336	Anoplophora glabripennis	Putative uncharacterized protein
EOG0912035H	APLA014717	Agrilus planipennis	Putative uncharacterized protein
EOG0912035H	TC015675	Tribolium castaneum	Putative uncharacterized protein
EOG0912035H	LDEC007872	Leptinotarsa decemlineata	Putative uncharacterized protein
EOG0912035H	YQE_08412	Dendroctonus ponderosae	Putative uncharacterized protein
EOG0912035I	OTAU001700	Onthophagus taurus	TGF-beta activated kinase 1
EOG0912035I	AGLA005418	Anoplophora glabripennis	TGF-beta activated kinase 1

EOG0912035I	APLA000942	Agrilus planipennis	TGF-beta activated kinase 1
EOG0912035I	TC005572	Tribolium castaneum	TGF-beta activated kinase 1
EOG0912035I	LDEC018053	Leptinotarsa decemlineata	TGF-beta activated kinase 1
EOG0912035I	YQE_06860	Dendroctonus ponderosae	TGF-beta activated kinase 1
EOG0912035N	OTAU002829	Onthophagus taurus	Putative uncharacterized protein
EOG0912035N	AGLA001174	Anoplophora glabripennis	Putative uncharacterized protein
EOG0912035N	APLA006761	Agrilus planipennis	Putative uncharacterized protein
EOG0912035N	TC004603	Tribolium castaneum	Putative uncharacterized protein
EOG0912035N	LDEC002038	Leptinotarsa decemlineata	Putative uncharacterized protein
EOG0912035N	YQE_02448	Dendroctonus ponderosae	Putative uncharacterized protein
EOG0912035Q	OTAU007080	Onthophagus taurus	adenyl nucleotide binding
EOG0912035Q	AGLA020897	Anoplophora glabripennis	adenyl nucleotide binding
EOG0912035Q	APLA004826	Agrilus planipennis	adenyl nucleotide binding
EOG0912035Q	TC033217	Tribolium castaneum	adenyl nucleotide binding
EOG0912035Q	LDEC019446	Leptinotarsa decemlineata	adenyl nucleotide binding
EOG0912035Q	YQE_08222	Dendroctonus ponderosae	adenyl nucleotide binding
EOG0912035S	OTAU002253	Onthophagus taurus	Putative uncharacterized protein
EOG0912035S	AGLA003523	Anoplophora glabripennis	Putative uncharacterized protein
EOG0912035S	APLA000121	Agrilus planipennis	Putative uncharacterized protein
EOG0912035S	TC004410	Tribolium castaneum	Putative uncharacterized protein
EOG0912035S	LDEC012684	Leptinotarsa decemlineata	Putative uncharacterized protein
EOG0912035S	YQE_10261	Dendroctonus ponderosae	Putative uncharacterized protein
EOG0912035V	OTAU015030	Onthophagus taurus	Putative uncharacterized protein
EOG0912035V	AGLA006141	Anoplophora glabripennis	Putative uncharacterized protein
EOG0912035V	APLA001387	Agrilus planipennis	Putative uncharacterized protein
EOG0912035V	TC000811	Tribolium castaneum	Putative uncharacterized protein
EOG0912035V	LDEC010359	Leptinotarsa decemlineata	Putative uncharacterized protein
EOG0912035V	YQE_05025	Dendroctonus ponderosae	Putative uncharacterized protein
EOG0912035W	OTAU000217	Onthophagus taurus	Putative uncharacterized protein
EOG0912035W	AGLA006418	Anoplophora glabripennis	Putative uncharacterized protein
EOG0912035W	APLA003095	Agrilus planipennis	Putative uncharacterized protein
EOG0912035W	TC030621	Tribolium castaneum	Putative uncharacterized protein
EOG0912035W	LDEC017186	Leptinotarsa decemlineata	Putative uncharacterized protein
EOG0912035W	YQE_06058	Dendroctonus ponderosae	Putative uncharacterized protein
EOG09120362	OTAU003880	Onthophagus taurus	Putative uncharacterized protein
EOG09120362	AGLA005769	Anoplophora glabripennis	Putative uncharacterized protein
EOG09120362	APLA007136	Agrilus planipennis	Putative uncharacterized protein
EOG09120362	TC010178	Tribolium castaneum	Putative uncharacterized protein
EOG09120362	LDEC017976	Leptinotarsa decemlineata	Putative uncharacterized protein
EOG09120362	YQE_11525	Dendroctonus ponderosae	Putative uncharacterized protein
EOG09120363	OTAU016191	Onthophagus taurus	None
EOG09120363	AGLA009433	Anoplophora glabripennis	None
EOG09120363	APLA014788	Agrilus planipennis	None
EOG09120363	TC031362	Tribolium castaneum	None
EOG09120363	LDEC010348	Leptinotarsa decemlineata	None
EOG09120363	YQE_06835	Dendroctonus ponderosae	None
EOG09120364	OTAU007637	Onthophagus taurus	kinase activity
EOG09120364	AGLA009510	Anoplophora glabripennis	kinase activity
EOG09120364	APLA011004	Agrilus planipennis	kinase activity
EOG09120364	TC034641	Tribolium castaneum	kinase activity
EOG09120364	LDEC000041	Leptinotarsa decemlineata	kinase activity
EOG09120364	YQE_00043	Dendroctonus ponderosae	kinase activity
EOG09120368	OTAU007556	Onthophagus taurus	Putative uncharacterized protein
EOG09120368	AGLA002670	Anoplophora glabripennis	Putative uncharacterized protein
EOG09120368	APLA002710	Agrilus planipennis	Putative uncharacterized protein
EOG09120368	TC013861	Tribolium castaneum	Putative uncharacterized protein
EOG09120368	LDEC008104	Leptinotarsa decemlineata	Putative uncharacterized protein
EOG09120368	YQE_06066	Dendroctonus ponderosae	Putative uncharacterized protein
EOG0912036A	OTAU002187	Onthophagus taurus	Putative uncharacterized protein
EOG0912036A	AGLA011612	Anoplophora glabripennis	Putative uncharacterized protein
EOG0912036A	APLA000783	Agrilus planipennis	Putative uncharacterized protein
EOG0912036A	TC006457	Tribolium castaneum	Putative uncharacterized protein
EOG0912036A	LDEC007214	Leptinotarsa decemlineata	Putative uncharacterized protein
EOG0912036A	YQE_02179	Dendroctonus ponderosae	Putative uncharacterized protein
EOG0912036C	OTAU002843	Onthophagus taurus	Putative uncharacterized protein
EOG0912036C	AGLA001170	Anoplophora glabripennis	Putative uncharacterized protein
EOG0912036C	APLA009991	Agrilus planipennis	Putative uncharacterized protein

EOG0912036C	TC004121	Tribolium castaneum	Putative uncharacterized protein
EOG0912036C	LDEC007477	Leptinotarsa decemlineata	Putative uncharacterized protein
EOG0912036C	YQE_06602	Dendroctonus ponderosae	Putative uncharacterized protein
EOG0912036D	OTAU011387	Onthophagus taurus	Putative uncharacterized protein
EOG0912036D	AGLA014065	Anoplophora glabripennis	Putative uncharacterized protein
EOG0912036D	APLA011746	Agrilus planipennis	Putative uncharacterized protein
EOG0912036D	TC013916	Tribolium castaneum	Putative uncharacterized protein
EOG0912036D	LDEC005977	Leptinotarsa decemlineata	Putative uncharacterized protein
EOG0912036D	YQE_06184	Dendroctonus ponderosae	Putative uncharacterized protein
EOG0912036F	OTAU002948	Onthophagus taurus	Flap endonuclease 1
EOG0912036F	AGLA006817	Anoplophora glabripennis	Flap endonuclease 1
EOG0912036F	APLA001840	Agrilus planipennis	Flap endonuclease 1
EOG0912036F	TC009261	Tribolium castaneum	Flap endonuclease 1
EOG0912036F	LDEC022300	Leptinotarsa decemlineata	Flap endonuclease 1
EOG0912036F	YQE_10615	Dendroctonus ponderosae	Flap endonuclease 1
EOG0912036G	OTAU006546	Onthophagus taurus	Putative uncharacterized protein
EOG0912036G	AGLA004643	Anoplophora glabripennis	Putative uncharacterized protein
EOG0912036G	APLA008137	Agrilus planipennis	Putative uncharacterized protein
EOG0912036G	TC002499	Tribolium castaneum	Putative uncharacterized protein
EOG0912036G	LDEC001509	Leptinotarsa decemlineata	Putative uncharacterized protein
EOG0912036G	YQE_07431	Dendroctonus ponderosae	Putative uncharacterized protein
EOG0912036L	OTAU005564	Onthophagus taurus	Serpin peptidase inhibitor 26
EOG0912036L	AGLA014445	Anoplophora glabripennis	Serpin peptidase inhibitor 26
EOG0912036L	APLA008327	Agrilus planipennis	Serpin peptidase inhibitor 26
EOG0912036L	TC007869	Tribolium castaneum	Serpin peptidase inhibitor 26
EOG0912036L	LDEC004591	Leptinotarsa decemlineata	Serpin peptidase inhibitor 26
EOG0912036L	YQE_03007	Dendroctonus ponderosae	Serpin peptidase inhibitor 26
EOG0912036O	OTAU011979	Onthophagus taurus	Putative uncharacterized protein
EOG0912036O	AGLA022023	Anoplophora glabripennis	Putative uncharacterized protein
EOG0912036O	APLA002491	Agrilus planipennis	Putative uncharacterized protein
EOG0912036O	TC013806	Tribolium castaneum	Putative uncharacterized protein
EOG0912036O	LDEC023256	Leptinotarsa decemlineata	Putative uncharacterized protein
EOG0912036O	YQE_03471	Dendroctonus ponderosae	Putative uncharacterized protein
EOG0912036P	OTAU008748	Onthophagus taurus	Putative uncharacterized protein
EOG0912036P	AGLA012755	Anoplophora glabripennis	Putative uncharacterized protein
EOG0912036P	APLA005567	Agrilus planipennis	Putative uncharacterized protein
EOG0912036P	TC014868	Tribolium castaneum	Putative uncharacterized protein
EOG0912036P	LDEC001851	Leptinotarsa decemlineata	Putative uncharacterized protein
EOG0912036P	YQE_08343	Dendroctonus ponderosae	Putative uncharacterized protein
EOG0912036R	OTAU000966	Onthophagus taurus	Putative uncharacterized protein
EOG0912036R	AGLA017916	Anoplophora glabripennis	Putative uncharacterized protein
EOG0912036R	APLA007954	Agrilus planipennis	Putative uncharacterized protein
EOG0912036R	TC011563	Tribolium castaneum	Putative uncharacterized protein
EOG0912036R	LDEC002192	Leptinotarsa decemlineata	Putative uncharacterized protein
EOG0912036R	YQE_11052	Dendroctonus ponderosae	Putative uncharacterized protein
EOG0912036S	OTAU001974	Onthophagus taurus	Putative uncharacterized protein
EOG0912036S	AGLA018480	Anoplophora glabripennis	Putative uncharacterized protein
EOG0912036S	APLA005775	Agrilus planipennis	Putative uncharacterized protein
EOG0912036S	TC006189	Tribolium castaneum	Putative uncharacterized protein
EOG0912036S	LDEC006768	Leptinotarsa decemlineata	Putative uncharacterized protein
EOG0912036S	YQE_12788	Dendroctonus ponderosae	Putative uncharacterized protein
EOG0912036V	OTAU008459	Onthophagus taurus	Putative uncharacterized protein
EOG0912036V	AGLA003492	Anoplophora glabripennis	Putative uncharacterized protein
EOG0912036V	APLA000189	Agrilus planipennis	Putative uncharacterized protein
EOG0912036V	TC030877	Tribolium castaneum	Putative uncharacterized protein
EOG0912036V	LDEC009418	Leptinotarsa decemlineata	Putative uncharacterized protein
EOG0912036V	YQE_06401	Dendroctonus ponderosae	Putative uncharacterized protein
EOG0912036W	OTAU000686	Onthophagus taurus	Putative uncharacterized protein
EOG0912036W	AGLA001016	Anoplophora glabripennis	Putative uncharacterized protein
EOG0912036W	APLA007816	Agrilus planipennis	Putative uncharacterized protein
EOG0912036W	TC008549	Tribolium castaneum	Putative uncharacterized protein
EOG0912036W	LDEC007490	Leptinotarsa decemlineata	Putative uncharacterized protein
EOG0912036W	YQE_07615	Dendroctonus ponderosae	Putative uncharacterized protein
EOG0912036X	OTAU009546	Onthophagus taurus	Putative uncharacterized protein
EOG0912036X	AGLA014437	Anoplophora glabripennis	Putative uncharacterized protein
EOG0912036X	APLA010915	Agrilus planipennis	Putative uncharacterized protein
EOG0912036X	TC007940	Tribolium castaneum	Putative uncharacterized protein

EOG0912036X	LDEC019977	Leptinotarsa decemlineata	Putative uncharacterized protein
EOG0912036X	YQE_02552	Dendroctonus ponderosae	Putative uncharacterized protein
EOG0912036Y	OTAU011792	Onthophagus taurus	Putative uncharacterized protein
EOG0912036Y	AGLA005975	Anoplophora glabripennis	Putative uncharacterized protein
EOG0912036Y	APLA007497	Agrilus planipennis	Putative uncharacterized protein
EOG0912036Y	TC005610	Tribolium castaneum	Putative uncharacterized protein
EOG0912036Y	LDEC015398	Leptinotarsa decemlineata	Putative uncharacterized protein
EOG0912036Y	YQE_06882	Dendroctonus ponderosae	Putative uncharacterized protein
EOG09120371	OTAU006151	Onthophagus taurus	Transaldolase
EOG09120371	AGLA013244	Anoplophora glabripennis	Transaldolase
EOG09120371	APLA008988	Agrilus planipennis	Transaldolase
EOG09120371	TC000616	Tribolium castaneum	Transaldolase
EOG09120371	LDEC001758	Leptinotarsa decemlineata	Transaldolase
EOG09120371	YQE_04225	Dendroctonus ponderosae	Transaldolase
EOG09120374	OTAU005077	Onthophagus taurus	Putative uncharacterized protein
EOG09120374	AGLA011033	Anoplophora glabripennis	Putative uncharacterized protein
EOG09120374	APLA000483	Agrilus planipennis	Putative uncharacterized protein
EOG09120374	TC006361	Tribolium castaneum	Putative uncharacterized protein
EOG09120374	LDEC006959	Leptinotarsa decemlineata	Putative uncharacterized protein
EOG09120374	YQE_01901	Dendroctonus ponderosae	Putative uncharacterized protein
EOG09120375	OTAU005405	Onthophagus taurus	Putative uncharacterized protein
EOG09120375	AGLA007605	Anoplophora glabripennis	Putative uncharacterized protein
EOG09120375	APLA003672	Agrilus planipennis	Putative uncharacterized protein
EOG09120375	TC001620	Tribolium castaneum	Putative uncharacterized protein
EOG09120375	LDEC015739	Leptinotarsa decemlineata	Putative uncharacterized protein
EOG09120375	YQE_09133	Dendroctonus ponderosae	Putative uncharacterized protein
EOG09120376	OTAU004764	Onthophagus taurus	Putative uncharacterized protein
EOG09120376	AGLA015852	Anoplophora glabripennis	Putative uncharacterized protein
EOG09120376	APLA010574	Agrilus planipennis	Putative uncharacterized protein
EOG09120376	TC030776	Tribolium castaneum	Putative uncharacterized protein
EOG09120376	LDEC013039	Leptinotarsa decemlineata	Putative uncharacterized protein
EOG09120376	YQE_11835	Dendroctonus ponderosae	Putative uncharacterized protein
EOG0912037A	OTAU011612	Onthophagus taurus	Putative uncharacterized protein
EOG0912037A	AGLA018705	Anoplophora glabripennis	Putative uncharacterized protein
EOG0912037A	APLA013235	Agrilus planipennis	Putative uncharacterized protein
EOG0912037A	TC009435	Tribolium castaneum	Putative uncharacterized protein
EOG0912037A	LDEC014670	Leptinotarsa decemlineata	Putative uncharacterized protein
EOG0912037A	YQE_13025	Dendroctonus ponderosae	Putative uncharacterized protein
EOG0912037B	OTAU012859	Onthophagus taurus	Putative uncharacterized protein
EOG0912037B	AGLA005891	Anoplophora glabripennis	Putative uncharacterized protein
EOG0912037B	APLA015434	Agrilus planipennis	Putative uncharacterized protein
EOG0912037B	TC014963	Tribolium castaneum	Putative uncharacterized protein
EOG0912037B	LDEC008292	Leptinotarsa decemlineata	Putative uncharacterized protein
EOG0912037B	YQE_05501	Dendroctonus ponderosae	Putative uncharacterized protein
EOG0912037F	OTAU013455	Onthophagus taurus	Putative uncharacterized protein
EOG0912037F	AGLA000466	Anoplophora glabripennis	Putative uncharacterized protein
EOG0912037F	APLA010131	Agrilus planipennis	Putative uncharacterized protein
EOG0912037F	TC008303	Tribolium castaneum	Putative uncharacterized protein
EOG0912037F	LDEC000958	Leptinotarsa decemlineata	Putative uncharacterized protein
EOG0912037F	YQE_08665	Dendroctonus ponderosae	Putative uncharacterized protein
EOG0912037G	OTAU003741	Onthophagus taurus	Putative uncharacterized protein
EOG0912037G	AGLA006453	Anoplophora glabripennis	Putative uncharacterized protein
EOG0912037G	APLA014787	Agrilus planipennis	Putative uncharacterized protein
EOG0912037G	TC008848	Tribolium castaneum	Putative uncharacterized protein
EOG0912037G	LDEC005802	Leptinotarsa decemlineata	Putative uncharacterized protein
EOG0912037G	YQE_11346	Dendroctonus ponderosae	Putative uncharacterized protein
EOG0912037I	OTAU010772	Onthophagus taurus	Putative uncharacterized protein
EOG0912037I	AGLA018782	Anoplophora glabripennis	Putative uncharacterized protein
EOG0912037I	APLA007217	Agrilus planipennis	Putative uncharacterized protein
EOG0912037I	TC002403	Tribolium castaneum	Putative uncharacterized protein
EOG0912037I	LDEC006193	Leptinotarsa decemlineata	Putative uncharacterized protein
EOG0912037I	YQE_09996	Dendroctonus ponderosae	Putative uncharacterized protein
EOG0912037N	OTAU004113	Onthophagus taurus	Putative uncharacterized protein
EOG0912037N	AGLA010956	Anoplophora glabripennis	Putative uncharacterized protein
EOG0912037N	APLA001546	Agrilus planipennis	Putative uncharacterized protein
EOG0912037N	TC009787	Tribolium castaneum	Putative uncharacterized protein
EOG0912037N	LDEC017658	Leptinotarsa decemlineata	Putative uncharacterized protein

EOG0912037N	YQE_10285	Dendroctonus ponderosae	Putative uncharacterized protein
EOG0912037O	OTAU007110	Onthophagus taurus	Putative uncharacterized protein
EOG0912037O	AGLA002502	Anoplophora glabripennis	Putative uncharacterized protein
EOG0912037O	APLA004923	Agrilus planipennis	Putative uncharacterized protein
EOG0912037O	TC005288	Tribolium castaneum	Putative uncharacterized protein
EOG0912037O	LDEC003605	Leptinotarsa decemlineata	Putative uncharacterized protein
EOG0912037O	YQE_07757	Dendroctonus ponderosae	Putative uncharacterized protein
EOG0912037P	OTAU015122	Onthophagus taurus	Putative uncharacterized protein
EOG0912037P	AGLA000495	Anoplophora glabripennis	Putative uncharacterized protein
EOG0912037P	APLA006602	Agrilus planipennis	Putative uncharacterized protein
EOG0912037P	TC007511	Tribolium castaneum	Putative uncharacterized protein
EOG0912037P	LDEC002396	Leptinotarsa decemlineata	Putative uncharacterized protein
EOG0912037P	YQE_02550	Dendroctonus ponderosae	Putative uncharacterized protein
EOG0912037Q	OTAU009983	Onthophagus taurus	Putative uncharacterized protein
EOG0912037Q	AGLA014242	Anoplophora glabripennis	Putative uncharacterized protein
EOG0912037Q	APLA012140	Agrilus planipennis	Putative uncharacterized protein
EOG0912037Q	TC013062	Tribolium castaneum	Putative uncharacterized protein
EOG0912037Q	LDEC014005	Leptinotarsa decemlineata	Putative uncharacterized protein
EOG0912037Q	YQE_07554	Dendroctonus ponderosae	Putative uncharacterized protein
EOG0912037R	OTAU013566	Onthophagus taurus	Putative uncharacterized protein
EOG0912037R	AGLA019101	Anoplophora glabripennis	Putative uncharacterized protein
EOG0912037R	APLA007378	Agrilus planipennis	Putative uncharacterized protein
EOG0912037R	TC002100	Tribolium castaneum	Putative uncharacterized protein
EOG0912037R	LDEC022633	Leptinotarsa decemlineata	Putative uncharacterized protein
EOG0912037R	YQE_04852	Dendroctonus ponderosae	Putative uncharacterized protein
EOG0912037S	OTAU001149	Onthophagus taurus	Putative uncharacterized protein
EOG0912037S	AGLA002565	Anoplophora glabripennis	Putative uncharacterized protein
EOG0912037S	APLA001964	Agrilus planipennis	Putative uncharacterized protein
EOG0912037S	TC002070	Tribolium castaneum	Putative uncharacterized protein
EOG0912037S	LDEC008073	Leptinotarsa decemlineata	Putative uncharacterized protein
EOG0912037S	YQE_11898	Dendroctonus ponderosae	Putative uncharacterized protein
EOG0912037V	OTAU006264	Onthophagus taurus	Putative uncharacterized protein
EOG0912037V	AGLA003090	Anoplophora glabripennis	Putative uncharacterized protein
EOG0912037V	APLA014759	Agrilus planipennis	Putative uncharacterized protein
EOG0912037V	TC007151	Tribolium castaneum	Putative uncharacterized protein
EOG0912037V	LDEC012189	Leptinotarsa decemlineata	Putative uncharacterized protein
EOG0912037V	YQE_02855	Dendroctonus ponderosae	Putative uncharacterized protein
EOG0912037W	OTAU008737	Onthophagus taurus	None
EOG0912037W	AGLA009460	Anoplophora glabripennis	None
EOG0912037W	APLA014389	Agrilus planipennis	None
EOG0912037W	TC030908	Tribolium castaneum	None
EOG0912037W	LDEC002227	Leptinotarsa decemlineata	None
EOG0912037W	YQE_02122	Dendroctonus ponderosae	None
EOG0912037Z	OTAU001834	Onthophagus taurus	Putative uncharacterized protein
EOG0912037Z	AGLA020028	Anoplophora glabripennis	Putative uncharacterized protein
EOG0912037Z	APLA004464	Agrilus planipennis	Putative uncharacterized protein
EOG0912037Z	TC005724	Tribolium castaneum	Putative uncharacterized protein
EOG0912037Z	LDEC017594	Leptinotarsa decemlineata	Putative uncharacterized protein
EOG0912037Z	YQE_12019	Dendroctonus ponderosae	Putative uncharacterized protein
EOG09120380	OTAU014967	Onthophagus taurus	Putative uncharacterized protein
EOG09120380	AGLA013754	Anoplophora glabripennis	Putative uncharacterized protein
EOG09120380	APLA000870	Agrilus planipennis	Putative uncharacterized protein
EOG09120380	TC012561	Tribolium castaneum	Putative uncharacterized protein
EOG09120380	LDEC003913	Leptinotarsa decemlineata	Putative uncharacterized protein
EOG09120380	YQE_05508	Dendroctonus ponderosae	Putative uncharacterized protein
EOG09120381	OTAU000929	Onthophagus taurus	Putative uncharacterized protein
EOG09120381	AGLA004319	Anoplophora glabripennis	Putative uncharacterized protein
EOG09120381	APLA005314	Agrilus planipennis	Putative uncharacterized protein
EOG09120381	TC011159	Tribolium castaneum	Putative uncharacterized protein
EOG09120381	LDEC008067	Leptinotarsa decemlineata	Putative uncharacterized protein
EOG09120381	YQE_11589	Dendroctonus ponderosae	Putative uncharacterized protein
EOG09120389	OTAU003524	Onthophagus taurus	Putative uncharacterized protein
EOG09120389	AGLA010326	Anoplophora glabripennis	Putative uncharacterized protein
EOG09120389	APLA004046	Agrilus planipennis	Putative uncharacterized protein
EOG09120389	TC030613	Tribolium castaneum	Putative uncharacterized protein
EOG09120389	LDEC008537	Leptinotarsa decemlineata	Putative uncharacterized protein
EOG09120389	YQE_11731	Dendroctonus ponderosae	Putative uncharacterized protein

EOG0912038A	OTAU002562	Onthophagus taurus	Putative uncharacterized protein
EOG0912038A	AGLA011924	Anoplophora glabripennis	Putative uncharacterized protein
EOG0912038A	APLA014461	Agrilus planipennis	Putative uncharacterized protein
EOG0912038A	TC012896	Tribolium castaneum	Putative uncharacterized protein
EOG0912038A	LDEC019853	Leptinotarsa decemlineata	Putative uncharacterized protein
EOG0912038A	YQE_02097	Dendroctonus ponderosae	Putative uncharacterized protein
EOG0912038D	OTAU000673	Onthophagus taurus	Putative uncharacterized protein
EOG0912038D	AGLA009844	Anoplophora glabripennis	Putative uncharacterized protein
EOG0912038D	APLA001092	Agrilus planipennis	Putative uncharacterized protein
EOG0912038D	TC030603	Tribolium castaneum	Putative uncharacterized protein
EOG0912038D	LDEC014350	Leptinotarsa decemlineata	Putative uncharacterized protein
EOG0912038D	YQE_01664	Dendroctonus ponderosae	Putative uncharacterized protein
EOG0912038H	OTAU015514	Onthophagus taurus	Putative uncharacterized protein
EOG0912038H	AGLA017805	Anoplophora glabripennis	Putative uncharacterized protein
EOG0912038H	APLA006934	Agrilus planipennis	Putative uncharacterized protein
EOG0912038H	TC009608	Tribolium castaneum	Putative uncharacterized protein
EOG0912038H	LDEC012869	Leptinotarsa decemlineata	Putative uncharacterized protein
EOG0912038H	YQE_11831	Dendroctonus ponderosae	Putative uncharacterized protein
EOG0912038K	OTAU010476	Onthophagus taurus	Putative uncharacterized protein
EOG0912038K	AGLA014181	Anoplophora glabripennis	Putative uncharacterized protein
EOG0912038K	APLA013271	Agrilus planipennis	Putative uncharacterized protein
EOG0912038K	TC002803	Tribolium castaneum	Putative uncharacterized protein
EOG0912038K	LDEC013204	Leptinotarsa decemlineata	Putative uncharacterized protein
EOG0912038K	YQE_07798	Dendroctonus ponderosae	Putative uncharacterized protein
EOG0912038L	OTAU001544	Onthophagus taurus	None
EOG0912038L	AGLA018643	Anoplophora glabripennis	None
EOG0912038L	APLA005829	Agrilus planipennis	None
EOG0912038L	TC031289	Tribolium castaneum	None
EOG0912038L	LDEC016561	Leptinotarsa decemlineata	None
EOG0912038L	YQE_05646	Dendroctonus ponderosae	None
EOG0912038N	OTAU010836	Onthophagus taurus	None
EOG0912038N	AGLA016198	Anoplophora glabripennis	None
EOG0912038N	APLA014362	Agrilus planipennis	None
EOG0912038N	TC033316	Tribolium castaneum	None
EOG0912038N	LDEC007998	Leptinotarsa decemlineata	None
EOG0912038N	YQE_09409	Dendroctonus ponderosae	None
EOG0912038O	OTAU007508	Onthophagus taurus	Putative uncharacterized protein
EOG0912038O	AGLA017912	Anoplophora glabripennis	Putative uncharacterized protein
EOG0912038O	APLA000684	Agrilus planipennis	Putative uncharacterized protein
EOG0912038O	TC015050	Tribolium castaneum	Putative uncharacterized protein
EOG0912038O	LDEC012533	Leptinotarsa decemlineata	Putative uncharacterized protein
EOG0912038O	YQE_02126	Dendroctonus ponderosae	Putative uncharacterized protein
EOG0912038P	OTAU006277	Onthophagus taurus	Putative uncharacterized protein
EOG0912038P	AGLA003756	Anoplophora glabripennis	Putative uncharacterized protein
EOG0912038P	APLA004213	Agrilus planipennis	Putative uncharacterized protein
EOG0912038P	TC007211	Tribolium castaneum	Putative uncharacterized protein
EOG0912038P	LDEC015005	Leptinotarsa decemlineata	Putative uncharacterized protein
EOG0912038P	YQE_04627	Dendroctonus ponderosae	Putative uncharacterized protein
EOG0912038S	OTAU006736	Onthophagus taurus	Phosphoserine aminotransferase
EOG0912038S	AGLA006117	Anoplophora glabripennis	Phosphoserine aminotransferase
EOG0912038S	APLA005307	Agrilus planipennis	Phosphoserine aminotransferase
EOG0912038S	TC000542	Tribolium castaneum	Phosphoserine aminotransferase
EOG0912038S	LDEC008684	Leptinotarsa decemlineata	Phosphoserine aminotransferase
EOG0912038S	YQE_01679	Dendroctonus ponderosae	Phosphoserine aminotransferase
EOG0912038T	OTAU004081	Onthophagus taurus	Putative uncharacterized protein
EOG0912038T	AGLA007520	Anoplophora glabripennis	Putative uncharacterized protein
EOG0912038T	APLA002831	Agrilus planipennis	Putative uncharacterized protein
EOG0912038T	TC003010	Tribolium castaneum	Putative uncharacterized protein
EOG0912038T	LDEC013858	Leptinotarsa decemlineata	Putative uncharacterized protein
EOG0912038T	YQE_07422	Dendroctonus ponderosae	Putative uncharacterized protein
EOG0912038Y	OTAU007761	Onthophagus taurus	Putative uncharacterized protein
EOG0912038Y	AGLA017427	Anoplophora glabripennis	Putative uncharacterized protein
EOG0912038Y	APLA008179	Agrilus planipennis	Putative uncharacterized protein
EOG0912038Y	TC010140	Tribolium castaneum	Putative uncharacterized protein
EOG0912038Y	LDEC013061	Leptinotarsa decemlineata	Putative uncharacterized protein
EOG0912038Y	YQE_06869	Dendroctonus ponderosae	Putative uncharacterized protein
EOG09120392	OTAU003452	Onthophagus taurus	Putative uncharacterized protein

EOG09120392	AGLA013865	Anoplophora glabripennis	Putative uncharacterized protein
EOG09120392	APLA006193	Agrilus planipennis	Putative uncharacterized protein
EOG09120392	TC009319	Tribolium castaneum	Putative uncharacterized protein
EOG09120392	LDEC019907	Leptinotarsa decemlineata	Putative uncharacterized protein
EOG09120392	YQE_05396	Dendroctonus ponderosae	Putative uncharacterized protein
EOG09120396	OTAU016184	Onthophagus taurus	Optomotor blind related gene 1 protein
EOG09120396	AGLA010322	Anoplophora glabripennis	Optomotor blind related gene 1 protein
EOG09120396	APLA009455	Agrilus planipennis	Optomotor blind related gene 1 protein
EOG09120396	TC015327	Tribolium castaneum	Optomotor blind related gene 1 protein
EOG09120396	LDEC008541	Leptinotarsa decemlineata	Optomotor blind related gene 1 protein
EOG09120396	YQE_10853	Dendroctonus ponderosae	Optomotor blind related gene 1 protein
EOG0912039A	OTAU001202	Onthophagus taurus	Putative uncharacterized protein
EOG0912039A	AGLA001425	Anoplophora glabripennis	Putative uncharacterized protein
EOG0912039A	APLA005279	Agrilus planipennis	Putative uncharacterized protein
EOG0912039A	TC002671	Tribolium castaneum	Putative uncharacterized protein
EOG0912039A	LDEC009573	Leptinotarsa decemlineata	Putative uncharacterized protein
EOG0912039A	YQE_07671	Dendroctonus ponderosae	Putative uncharacterized protein
EOG0912039C	OTAU008308	Onthophagus taurus	Putative uncharacterized protein
EOG0912039C	AGLA015484	Anoplophora glabripennis	Putative uncharacterized protein
EOG0912039C	APLA003822	Agrilus planipennis	Putative uncharacterized protein
EOG0912039C	TC015802	Tribolium castaneum	Putative uncharacterized protein
EOG0912039C	LDEC008034	Leptinotarsa decemlineata	Putative uncharacterized protein
EOG0912039C	YQE_10904	Dendroctonus ponderosae	Putative uncharacterized protein
EOG0912039D	OTAU015210	Onthophagus taurus	Putative uncharacterized protein
EOG0912039D	AGLA004677	Anoplophora glabripennis	Putative uncharacterized protein
EOG0912039D	APLA002199	Agrilus planipennis	Putative uncharacterized protein
EOG0912039D	TC008474	Tribolium castaneum	Putative uncharacterized protein
EOG0912039D	LDEC008304	Leptinotarsa decemlineata	Putative uncharacterized protein
EOG0912039D	YQE_06463	Dendroctonus ponderosae	Putative uncharacterized protein
EOG0912039F	OTAU005171	Onthophagus taurus	Putative uncharacterized protein
EOG0912039F	AGLA010124	Anoplophora glabripennis	Putative uncharacterized protein
EOG0912039F	APLA011287	Agrilus planipennis	Putative uncharacterized protein
EOG0912039F	TC003548	Tribolium castaneum	Putative uncharacterized protein
EOG0912039F	LDEC017642	Leptinotarsa decemlineata	Putative uncharacterized protein
EOG0912039F	YQE_11128	Dendroctonus ponderosae	Putative uncharacterized protein
EOG0912039G	OTAU011994	Onthophagus taurus	tRNA pseudouridine synthase A 1
EOG0912039G	AGLA009027	Anoplophora glabripennis	tRNA pseudouridine synthase A 1
EOG0912039G	APLA011406	Agrilus planipennis	tRNA pseudouridine synthase A 1
EOG0912039G	TC030541	Tribolium castaneum	tRNA pseudouridine synthase A 1
EOG0912039G	LDEC006103	Leptinotarsa decemlineata	tRNA pseudouridine synthase A 1
EOG0912039G	YQE_01756	Dendroctonus ponderosae	tRNA pseudouridine synthase A 1
EOG0912039H	OTAU001587	Onthophagus taurus	Putative uncharacterized protein
EOG0912039H	AGLA004971	Anoplophora glabripennis	Putative uncharacterized protein
EOG0912039H	APLA004498	Agrilus planipennis	Putative uncharacterized protein
EOG0912039H	TC015484	Tribolium castaneum	Putative uncharacterized protein
EOG0912039H	LDEC003014	Leptinotarsa decemlineata	Putative uncharacterized protein
EOG0912039H	YQE_07027	Dendroctonus ponderosae	Putative uncharacterized protein
EOG0912039J	OTAU012555	Onthophagus taurus	Putative uncharacterized protein
EOG0912039J	AGLA000130	Anoplophora glabripennis	Putative uncharacterized protein
EOG0912039J	APLA009352	Agrilus planipennis	Putative uncharacterized protein
EOG0912039J	TC001320	Tribolium castaneum	Putative uncharacterized protein
EOG0912039J	LDEC008081	Leptinotarsa decemlineata	Putative uncharacterized protein
EOG0912039J	YQE_08142	Dendroctonus ponderosae	Putative uncharacterized protein
EOG0912039L	OTAU006963	Onthophagus taurus	Putative uncharacterized protein
EOG0912039L	AGLA007838	Anoplophora glabripennis	Putative uncharacterized protein
EOG0912039L	APLA003220	Agrilus planipennis	Putative uncharacterized protein
EOG0912039L	TC001676	Tribolium castaneum	Putative uncharacterized protein
EOG0912039L	LDEC004287	Leptinotarsa decemlineata	Putative uncharacterized protein
EOG0912039L	YQE_07318	Dendroctonus ponderosae	Putative uncharacterized protein
EOG0912039N	OTAU003378	Onthophagus taurus	Putative uncharacterized protein
EOG0912039N	AGLA006583	Anoplophora glabripennis	Putative uncharacterized protein
EOG0912039N	APLA007677	Agrilus planipennis	Putative uncharacterized protein
EOG0912039N	TC012060	Tribolium castaneum	Putative uncharacterized protein
EOG0912039N	LDEC007659	Leptinotarsa decemlineata	Putative uncharacterized protein
EOG0912039N	YQE_12964	Dendroctonus ponderosae	Putative uncharacterized protein
EOG0912039V	OTAU007370	Onthophagus taurus	Putative uncharacterized protein
EOG0912039V	AGLA001375	Anoplophora glabripennis	Putative uncharacterized protein

EOG0912039V	APLA005033	Agrilus planipennis	Putative uncharacterized protein
EOG0912039V	TC001328	Tribolium castaneum	Putative uncharacterized protein
EOG0912039V	LDEC007301	Leptinotarsa decemlineata	Putative uncharacterized protein
EOG0912039V	YQE_01586	Dendroctonus ponderosae	Putative uncharacterized protein
EOG0912039W	OTAU000395	Onthophagus taurus	None
EOG0912039W	AGLA011370	Anoplophora glabripennis	None
EOG0912039W	APLA003279	Agrilus planipennis	None
EOG0912039W	TC034891	Tribolium castaneum	None
EOG0912039W	LDEC008600	Leptinotarsa decemlineata	None
EOG0912039W	YQE_10080	Dendroctonus ponderosae	None
EOG0912039X	OTAU002088	Onthophagus taurus	Putative uncharacterized protein
EOG0912039X	AGLA003298	Anoplophora glabripennis	Putative uncharacterized protein
EOG0912039X	APLA001122	Agrilus planipennis	Putative uncharacterized protein
EOG0912039X	TC011666	Tribolium castaneum	Putative uncharacterized protein
EOG0912039X	LDEC017634	Leptinotarsa decemlineata	Putative uncharacterized protein
EOG0912039X	YQE_10909	Dendroctonus ponderosae	Putative uncharacterized protein
EOG0912039Z	OTAU006369	Onthophagus taurus	Exuperantia
EOG0912039Z	AGLA009995	Anoplophora glabripennis	Exuperantia
EOG0912039Z	APLA008818	Agrilus planipennis	Exuperantia
EOG0912039Z	TC009494	Tribolium castaneum	Exuperantia
EOG0912039Z	LDEC011015	Leptinotarsa decemlineata	Exuperantia
EOG0912039Z	YQE_05389	Dendroctonus ponderosae	Exuperantia
EOG091203A0	OTAU005808	Onthophagus taurus	Putative uncharacterized protein
EOG091203A0	AGLA002281	Anoplophora glabripennis	Putative uncharacterized protein
EOG091203A0	APLA003974	Agrilus planipennis	Putative uncharacterized protein
EOG091203A0	TC012758	Tribolium castaneum	Putative uncharacterized protein
EOG091203A0	LDEC000587	Leptinotarsa decemlineata	Putative uncharacterized protein
EOG091203A0	YQE_08064	Dendroctonus ponderosae	Putative uncharacterized protein
EOG091203A6	OTAU010012	Onthophagus taurus	"NADH dehydrogenase (Ubiquinone) 1 alpha subcomplex, 10 "
EOG091203A6	AGLA021200	Anoplophora glabripennis	"NADH dehydrogenase (Ubiquinone) 1 alpha subcomplex, 10 "
EOG091203A6	APLA009245	Agrilus planipennis	"NADH dehydrogenase (Ubiquinone) 1 alpha subcomplex, 10 "
EOG091203A6	TC008030	Tribolium castaneum	"NADH dehydrogenase (Ubiquinone) 1 alpha subcomplex, 10 "
EOG091203A6	LDEC018600	Leptinotarsa decemlineata	"NADH dehydrogenase (Ubiquinone) 1 alpha subcomplex, 10 "
EOG091203A6	YQE_05459	Dendroctonus ponderosae	"NADH dehydrogenase (Ubiquinone) 1 alpha subcomplex, 10 "
EOG091203A7	OTAU009335	Onthophagus taurus	Putative uncharacterized protein
EOG091203A7	AGLA013798	Anoplophora glabripennis	Putative uncharacterized protein
EOG091203A7	APLA006852	Agrilus planipennis	Putative uncharacterized protein
EOG091203A7	TC013384	Tribolium castaneum	Putative uncharacterized protein
EOG091203A7	LDEC002629	Leptinotarsa decemlineata	Putative uncharacterized protein
EOG091203A7	YQE_07288	Dendroctonus ponderosae	Putative uncharacterized protein
EOG091203A8	OTAU004385	Onthophagus taurus	Serine protease P144
EOG091203A8	AGLA012200	Anoplophora glabripennis	Serine protease P144
EOG091203A8	APLA003640	Agrilus planipennis	Serine protease P144
EOG091203A8	TC013613	Tribolium castaneum	Serine protease P144
EOG091203A8	LDEC020610	Leptinotarsa decemlineata	Serine protease P144
EOG091203A8	YQE_06663	Dendroctonus ponderosae	Serine protease P144
EOG091203A9	OTAU009094	Onthophagus taurus	Putative uncharacterized protein
EOG091203A9	AGLA009649	Anoplophora glabripennis	Putative uncharacterized protein
EOG091203A9	APLA000454	Agrilus planipennis	Putative uncharacterized protein
EOG091203A9	TC015976	Tribolium castaneum	Putative uncharacterized protein
EOG091203A9	LDEC022144	Leptinotarsa decemlineata	Putative uncharacterized protein
EOG091203A9	YQE_12102	Dendroctonus ponderosae	Putative uncharacterized protein
EOG091203AB	OTAU012991	Onthophagus taurus	Putative uncharacterized protein
EOG091203AB	AGLA006682	Anoplophora glabripennis	Putative uncharacterized protein
EOG091203AB	APLA001914	Agrilus planipennis	Putative uncharacterized protein
EOG091203AB	TC000508	Tribolium castaneum	Putative uncharacterized protein
EOG091203AB	LDEC003553	Leptinotarsa decemlineata	Putative uncharacterized protein
EOG091203AB	YQE_08490	Dendroctonus ponderosae	Putative uncharacterized protein
EOG091203AH	OTAU010084	Onthophagus taurus	Putative uncharacterized protein
EOG091203AH	AGLA003349	Anoplophora glabripennis	Putative uncharacterized protein
EOG091203AH	APLA013502	Agrilus planipennis	Putative uncharacterized protein

EOG091203AH	TC015315	Tribolium castaneum	Putative uncharacterized protein
EOG091203AH	LDEC011752	Leptinotarsa decemlineata	Putative uncharacterized protein
EOG091203AH	YQE_11745	Dendroctonus ponderosae	Putative uncharacterized protein
EOG091203AI	OTAU001666	Onthophagus taurus	Putative uncharacterized protein
EOG091203AI	AGLA011081	Anoplophora glabripennis	Putative uncharacterized protein
EOG091203AI	APLA000954	Agrilus planipennis	Putative uncharacterized protein
EOG091203AI	TC005618	Tribolium castaneum	Putative uncharacterized protein
EOG091203AI	LDEC002921	Leptinotarsa decemlineata	Putative uncharacterized protein
EOG091203AI	YQE_05817	Dendroctonus ponderosae	Putative uncharacterized protein
EOG091203AJ	OTAU004792	Onthophagus taurus	Putative uncharacterized protein
EOG091203AJ	AGLA011572	Anoplophora glabripennis	Putative uncharacterized protein
EOG091203AJ	APLA006255	Agrilus planipennis	Putative uncharacterized protein
EOG091203AJ	TC000737	Tribolium castaneum	Putative uncharacterized protein
EOG091203AJ	LDEC007775	Leptinotarsa decemlineata	Putative uncharacterized protein
EOG091203AJ	YQE_01760	Dendroctonus ponderosae	Putative uncharacterized protein
EOG091203AL	OTAU016838	Onthophagus taurus	Putative uncharacterized protein
EOG091203AL	AGLA010702	Anoplophora glabripennis	Putative uncharacterized protein
EOG091203AL	APLA011065	Agrilus planipennis	Putative uncharacterized protein
EOG091203AL	TC006355	Tribolium castaneum	Putative uncharacterized protein
EOG091203AL	LDEC004913	Leptinotarsa decemlineata	Putative uncharacterized protein
EOG091203AL	YQE_05654	Dendroctonus ponderosae	Putative uncharacterized protein
EOG091203AN	OTAU012902	Onthophagus taurus	nucleotide binding
EOG091203AN	AGLA008772	Anoplophora glabripennis	nucleotide binding
EOG091203AN	APLA003811	Agrilus planipennis	nucleotide binding
EOG091203AN	TC031430	Tribolium castaneum	nucleotide binding
EOG091203AN	LDEC015790	Leptinotarsa decemlineata	nucleotide binding
EOG091203AN	YQE_08523	Dendroctonus ponderosae	nucleotide binding
EOG091203AO	OTAU016386	Onthophagus taurus	Putative uncharacterized protein
EOG091203AO	AGLA008727	Anoplophora glabripennis	Putative uncharacterized protein
EOG091203AO	APLA004905	Agrilus planipennis	Putative uncharacterized protein
EOG091203AO	TC008754	Tribolium castaneum	Putative uncharacterized protein
EOG091203AO	LDEC004847	Leptinotarsa decemlineata	Putative uncharacterized protein
EOG091203AO	YQE_05738	Dendroctonus ponderosae	Putative uncharacterized protein
EOG091203AP	OTAU007328	Onthophagus taurus	None
EOG091203AP	AGLA000105	Anoplophora glabripennis	None
EOG091203AP	APLA011140	Agrilus planipennis	None
EOG091203AP	TC031365	Tribolium castaneum	None
EOG091203AP	LDEC016552	Leptinotarsa decemlineata	None
EOG091203AP	YQE_10761	Dendroctonus ponderosae	None
EOG091203AR	OTAU002848	Onthophagus taurus	Putative uncharacterized protein
EOG091203AR	AGLA002163	Anoplophora glabripennis	Putative uncharacterized protein
EOG091203AR	APLA006757	Agrilus planipennis	Putative uncharacterized protein
EOG091203AR	TC007292	Tribolium castaneum	Putative uncharacterized protein
EOG091203AR	LDEC020320	Leptinotarsa decemlineata	Putative uncharacterized protein
EOG091203AR	YQE_03318	Dendroctonus ponderosae	Putative uncharacterized protein
EOG091203AX	OTAU013066	Onthophagus taurus	Putative uncharacterized protein
EOG091203AX	AGLA011147	Anoplophora glabripennis	Putative uncharacterized protein
EOG091203AX	APLA008348	Agrilus planipennis	Putative uncharacterized protein
EOG091203AX	TC030629	Tribolium castaneum	Putative uncharacterized protein
EOG091203AX	LDEC013717	Leptinotarsa decemlineata	Putative uncharacterized protein
EOG091203AX	YQE_08198	Dendroctonus ponderosae	Putative uncharacterized protein
EOG091203AY	OTAU008374	Onthophagus taurus	Putative uncharacterized protein
EOG091203AY	AGLA001633	Anoplophora glabripennis	Putative uncharacterized protein
EOG091203AY	APLA002962	Agrilus planipennis	Putative uncharacterized protein
EOG091203AY	TC000835	Tribolium castaneum	Putative uncharacterized protein
EOG091203AY	LDEC018896	Leptinotarsa decemlineata	Putative uncharacterized protein
EOG091203AY	YQE_06839	Dendroctonus ponderosae	Putative uncharacterized protein
EOG091203B0	OTAU010553	Onthophagus taurus	None
EOG091203B0	AGLA003011	Anoplophora glabripennis	None
EOG091203B0	APLA004161	Agrilus planipennis	None
EOG091203B0	TC034542	Tribolium castaneum	None
EOG091203B0	LDEC009526	Leptinotarsa decemlineata	None
EOG091203B0	YQE_10732	Dendroctonus ponderosae	None
EOG091203B1	OTAU008313	Onthophagus taurus	DNA binding
EOG091203B1	AGLA006328	Anoplophora glabripennis	DNA binding
EOG091203B1	APLA003836	Agrilus planipennis	DNA binding
EOG091203B1	TC033450	Tribolium castaneum	DNA binding

EOG091203B1	LDEC010813	Leptinotarsa decemlineata	DNA binding
EOG091203B1	YQE_10906	Dendroctonus ponderosae	DNA binding
EOG091203B3	OTAU008445	Onthophagus taurus	Putative uncharacterized protein
EOG091203B3	AGLA004450	Anoplophora glabripennis	Putative uncharacterized protein
EOG091203B3	APLA006683	Agrilus planipennis	Putative uncharacterized protein
EOG091203B3	TC002366	Tribolium castaneum	Putative uncharacterized protein
EOG091203B3	LDEC002703	Leptinotarsa decemlineata	Putative uncharacterized protein
EOG091203B3	YQE_12419	Dendroctonus ponderosae	Putative uncharacterized protein
EOG091203B8	OTAU001612	Onthophagus taurus	None
EOG091203B8	AGLA007559	Anoplophora glabripennis	None
EOG091203B8	APLA012291	Agrilus planipennis	None
EOG091203B8	TC034118	Tribolium castaneum	None
EOG091203B8	LDEC006637	Leptinotarsa decemlineata	None
EOG091203B8	YQE_12210	Dendroctonus ponderosae	None
EOG091203BA	OTAU012270	Onthophagus taurus	Putative uncharacterized protein
EOG091203BA	AGLA018576	Anoplophora glabripennis	Putative uncharacterized protein
EOG091203BA	APLA013845	Agrilus planipennis	Putative uncharacterized protein
EOG091203BA	TC011817	Tribolium castaneum	Putative uncharacterized protein
EOG091203BA	LDEC015900	Leptinotarsa decemlineata	Putative uncharacterized protein
EOG091203BA	YQE_12575	Dendroctonus ponderosae	Putative uncharacterized protein
EOG091203BB	OTAU007625	Onthophagus taurus	Putative uncharacterized protein
EOG091203BB	AGLA005506	Anoplophora glabripennis	Putative uncharacterized protein
EOG091203BB	APLA002404	Agrilus planipennis	Putative uncharacterized protein
EOG091203BB	TC002246	Tribolium castaneum	Putative uncharacterized protein
EOG091203BB	LDEC005349	Leptinotarsa decemlineata	Putative uncharacterized protein
EOG091203BB	YQE_07275	Dendroctonus ponderosae	Putative uncharacterized protein
EOG091203BC	OTAU006650	Onthophagus taurus	Putative uncharacterized protein
EOG091203BC	AGLA021290	Anoplophora glabripennis	Putative uncharacterized protein
EOG091203BC	APLA014581	Agrilus planipennis	Putative uncharacterized protein
EOG091203BC	TC000118	Tribolium castaneum	Putative uncharacterized protein
EOG091203BC	LDEC005035	Leptinotarsa decemlineata	Putative uncharacterized protein
EOG091203BC	YQE_03697	Dendroctonus ponderosae	Putative uncharacterized protein
EOG091203BD	OTAU004296	Onthophagus taurus	GTP binding
EOG091203BD	AGLA006281	Anoplophora glabripennis	GTP binding
EOG091203BD	APLA009445	Agrilus planipennis	GTP binding
EOG091203BD	TC034529	Tribolium castaneum	GTP binding
EOG091203BD	LDEC017597	Leptinotarsa decemlineata	GTP binding
EOG091203BD	YQE_09101	Dendroctonus ponderosae	GTP binding
EOG091203BE	OTAU004772	Onthophagus taurus	Putative uncharacterized protein
EOG091203BE	AGLA011908	Anoplophora glabripennis	Putative uncharacterized protein
EOG091203BE	APLA001402	Agrilus planipennis	Putative uncharacterized protein
EOG091203BE	TC000388	Tribolium castaneum	Putative uncharacterized protein
EOG091203BE	LDEC001421	Leptinotarsa decemlineata	Putative uncharacterized protein
EOG091203BE	YQE_04924	Dendroctonus ponderosae	Putative uncharacterized protein
EOG091203BF	OTAU014241	Onthophagus taurus	Putative uncharacterized protein
EOG091203BF	AGLA015000	Anoplophora glabripennis	Putative uncharacterized protein
EOG091203BF	APLA010560	Agrilus planipennis	Putative uncharacterized protein
EOG091203BF	TC016299	Tribolium castaneum	Putative uncharacterized protein
EOG091203BF	LDEC006339	Leptinotarsa decemlineata	Putative uncharacterized protein
EOG091203BF	YQE_12829	Dendroctonus ponderosae	Putative uncharacterized protein
EOG091203BG	OTAU007939	Onthophagus taurus	Putative uncharacterized protein
EOG091203BG	AGLA020484	Anoplophora glabripennis	Putative uncharacterized protein
EOG091203BG	APLA001663	Agrilus planipennis	Putative uncharacterized protein
EOG091203BG	TC009879	Tribolium castaneum	Putative uncharacterized protein
EOG091203BG	LDEC005811	Leptinotarsa decemlineata	Putative uncharacterized protein
EOG091203BG	YQE_10401	Dendroctonus ponderosae	Putative uncharacterized protein
EOG091203BH	OTAU013026	Onthophagus taurus	None
EOG091203BH	AGLA003895	Anoplophora glabripennis	None
EOG091203BH	APLA000593	Agrilus planipennis	None
EOG091203BH	TC033407	Tribolium castaneum	None
EOG091203BH	LDEC003625	Leptinotarsa decemlineata	None
EOG091203BH	YQE_11877	Dendroctonus ponderosae	None
EOG091203BL	OTAU000531	Onthophagus taurus	Putative uncharacterized protein
EOG091203BL	AGLA008913	Anoplophora glabripennis	Putative uncharacterized protein
EOG091203BL	APLA000166	Agrilus planipennis	Putative uncharacterized protein
EOG091203BL	TC003525	Tribolium castaneum	Putative uncharacterized protein
EOG091203BL	LDEC007077	Leptinotarsa decemlineata	Putative uncharacterized protein

EOG091203BL	YQE_10257	Dendroctonus ponderosae	Putative uncharacterized protein
EOG091203BM	OTAU011035	Onthophagus taurus	Putative uncharacterized protein
EOG091203BM	AGLA006385	Anoplophora glabripennis	Putative uncharacterized protein
EOG091203BM	APLA011584	Agrilus planipennis	Putative uncharacterized protein
EOG091203BM	TC030750	Tribolium castaneum	Putative uncharacterized protein
EOG091203BM	LDEC011743	Leptinotarsa decemlineata	Putative uncharacterized protein
EOG091203BM	YQE_04041	Dendroctonus ponderosae	Putative uncharacterized protein
EOG091203BR	OTAU002180	Onthophagus taurus	Putative uncharacterized protein
EOG091203BR	AGLA011380	Anoplophora glabripennis	Putative uncharacterized protein
EOG091203BR	APLA003968	Agrilus planipennis	Putative uncharacterized protein
EOG091203BR	TC012123	Tribolium castaneum	Putative uncharacterized protein
EOG091203BR	LDEC002072	Leptinotarsa decemlineata	Putative uncharacterized protein
EOG091203BR	YQE_08125	Dendroctonus ponderosae	Putative uncharacterized protein
EOG091203BS	OTAU009841	Onthophagus taurus	Putative uncharacterized protein
EOG091203BS	AGLA002793	Anoplophora glabripennis	Putative uncharacterized protein
EOG091203BS	APLA007161	Agrilus planipennis	Putative uncharacterized protein
EOG091203BS	TC009151	Tribolium castaneum	Putative uncharacterized protein
EOG091203BS	LDEC013706	Leptinotarsa decemlineata	Putative uncharacterized protein
EOG091203BS	YQE_11934	Dendroctonus ponderosae	Putative uncharacterized protein
EOG091203BV	OTAU002617	Onthophagus taurus	Putative uncharacterized protein
EOG091203BV	AGLA016520	Anoplophora glabripennis	Putative uncharacterized protein
EOG091203BV	APLA008380	Agrilus planipennis	Putative uncharacterized protein
EOG091203BV	TC003757	Tribolium castaneum	Putative uncharacterized protein
EOG091203BV	LDEC002488	Leptinotarsa decemlineata	Putative uncharacterized protein
EOG091203BV	YQE_07222	Dendroctonus ponderosae	Putative uncharacterized protein
EOG091203BY	OTAU008564	Onthophagus taurus	peptidase activity
EOG091203BY	AGLA011203	Anoplophora glabripennis	peptidase activity
EOG091203BY	APLA011098	Agrilus planipennis	peptidase activity
EOG091203BY	TC033855	Tribolium castaneum	peptidase activity
EOG091203BY	LDEC007690	Leptinotarsa decemlineata	peptidase activity
EOG091203BY	YQE_12586	Dendroctonus ponderosae	peptidase activity
EOG091203C1	OTAU016275	Onthophagus taurus	Putative uncharacterized protein
EOG091203C1	AGLA021651	Anoplophora glabripennis	Putative uncharacterized protein
EOG091203C1	APLA001826	Agrilus planipennis	Putative uncharacterized protein
EOG091203C1	TC014541	Tribolium castaneum	Putative uncharacterized protein
EOG091203C1	LDEC011854	Leptinotarsa decemlineata	Putative uncharacterized protein
EOG091203C1	YQE_05448	Dendroctonus ponderosae	Putative uncharacterized protein
EOG091203C5	OTAU016454	Onthophagus taurus	hydrolase activity
EOG091203C5	AGLA001201	Anoplophora glabripennis	hydrolase activity
EOG091203C5	APLA000440	Agrilus planipennis	hydrolase activity
EOG091203C5	TC034199	Tribolium castaneum	hydrolase activity
EOG091203C5	LDEC008269	Leptinotarsa decemlineata	hydrolase activity
EOG091203C5	YQE_01728	Dendroctonus ponderosae	hydrolase activity
EOG091203C6	OTAU009910	Onthophagus taurus	Putative uncharacterized protein
EOG091203C6	AGLA002150	Anoplophora glabripennis	Putative uncharacterized protein
EOG091203C6	APLA009961	Agrilus planipennis	Putative uncharacterized protein
EOG091203C6	TC004673	Tribolium castaneum	Putative uncharacterized protein
EOG091203C6	LDEC014963	Leptinotarsa decemlineata	Putative uncharacterized protein
EOG091203C6	YQE_09264	Dendroctonus ponderosae	Putative uncharacterized protein
EOG091203C7	OTAU000457	Onthophagus taurus	Putative uncharacterized protein
EOG091203C7	AGLA001492	Anoplophora glabripennis	Putative uncharacterized protein
EOG091203C7	APLA010331	Agrilus planipennis	Putative uncharacterized protein
EOG091203C7	TC003826	Tribolium castaneum	Putative uncharacterized protein
EOG091203C7	LDEC017563	Leptinotarsa decemlineata	Putative uncharacterized protein
EOG091203C7	YQE_01785	Dendroctonus ponderosae	Putative uncharacterized protein
EOG091203C8	OTAU011340	Onthophagus taurus	Putative uncharacterized protein
EOG091203C8	AGLA012503	Anoplophora glabripennis	Putative uncharacterized protein
EOG091203C8	APLA005718	Agrilus planipennis	Putative uncharacterized protein
EOG091203C8	TC030583	Tribolium castaneum	Putative uncharacterized protein
EOG091203C8	LDEC010013	Leptinotarsa decemlineata	Putative uncharacterized protein
EOG091203C8	YQE_02346	Dendroctonus ponderosae	Putative uncharacterized protein
EOG091203CF	OTAU004878	Onthophagus taurus	Putative uncharacterized protein
EOG091203CF	AGLA001953	Anoplophora glabripennis	Putative uncharacterized protein
EOG091203CF	APLA008656	Agrilus planipennis	Putative uncharacterized protein
EOG091203CF	TC011691	Tribolium castaneum	Putative uncharacterized protein
EOG091203CF	LDEC015644	Leptinotarsa decemlineata	Putative uncharacterized protein
EOG091203CF	YQE_08033	Dendroctonus ponderosae	Putative uncharacterized protein

EOG091203CI	OTAU005047	Onthophagus taurus	Glyceraldehyde-3-phosphate dehydrogenase
EOG091203CI	AGLA018100	Anoplophora glabripennis	Glyceraldehyde-3-phosphate dehydrogenase
EOG091203CI	APLA007488	Agrilus planipennis	Glyceraldehyde-3-phosphate dehydrogenase
EOG091203CI	TC006170	Tribolium castaneum	Glyceraldehyde-3-phosphate dehydrogenase
EOG091203CI	LDEC009853	Leptinotarsa decemlineata	Glyceraldehyde-3-phosphate dehydrogenase
EOG091203CI	YQE_12029	Dendroctonus ponderosae	Glyceraldehyde-3-phosphate dehydrogenase
EOG091203CJ	OTAU007962	Onthophagus taurus	Putative uncharacterized protein
EOG091203CJ	AGLA019576	Anoplophora glabripennis	Putative uncharacterized protein
EOG091203CJ	APLA013197	Agrilus planipennis	Putative uncharacterized protein
EOG091203CJ	TC002931	Tribolium castaneum	Putative uncharacterized protein
EOG091203CJ	LDEC020413	Leptinotarsa decemlineata	Putative uncharacterized protein
EOG091203CJ	YQE_11840	Dendroctonus ponderosae	Putative uncharacterized protein
EOG091203CL	OTAU012858	Onthophagus taurus	Putative uncharacterized protein
EOG091203CL	AGLA001089	Anoplophora glabripennis	Putative uncharacterized protein
EOG091203CL	APLA005959	Agrilus planipennis	Putative uncharacterized protein
EOG091203CL	TC014947	Tribolium castaneum	Putative uncharacterized protein
EOG091203CL	LDEC008757	Leptinotarsa decemlineata	Putative uncharacterized protein
EOG091203CL	YQE_11677	Dendroctonus ponderosae	Putative uncharacterized protein
EOG091203CO	OTAU002136	Onthophagus taurus	Putative uncharacterized protein
EOG091203CO	AGLA020048	Anoplophora glabripennis	Putative uncharacterized protein
EOG091203CO	APLA000287	Agrilus planipennis	Putative uncharacterized protein
EOG091203CO	TC003561	Tribolium castaneum	Putative uncharacterized protein
EOG091203CO	LDEC019724	Leptinotarsa decemlineata	Putative uncharacterized protein
EOG091203CO	YQE_07838	Dendroctonus ponderosae	Putative uncharacterized protein
EOG091203CP	OTAU003441	Onthophagus taurus	Putative uncharacterized protein
EOG091203CP	AGLA007498	Anoplophora glabripennis	Putative uncharacterized protein
EOG091203CP	APLA006714	Agrilus planipennis	Putative uncharacterized protein
EOG091203CP	TC003270	Tribolium castaneum	Putative uncharacterized protein
EOG091203CP	LDEC013828	Leptinotarsa decemlineata	Putative uncharacterized protein
EOG091203CP	YQE_07417	Dendroctonus ponderosae	Putative uncharacterized protein
EOG091203CQ	OTAU004340	Onthophagus taurus	Putative uncharacterized protein
EOG091203CQ	AGLA000282	Anoplophora glabripennis	Putative uncharacterized protein
EOG091203CQ	APLA006362	Agrilus planipennis	Putative uncharacterized protein
EOG091203CQ	TC001460	Tribolium castaneum	Putative uncharacterized protein
EOG091203CQ	LDEC001016	Leptinotarsa decemlineata	Putative uncharacterized protein
EOG091203CQ	YQE_06658	Dendroctonus ponderosae	Putative uncharacterized protein
EOG091203CR	OTAU001084	Onthophagus taurus	phosphatidylserine decarboxylase activity
EOG091203CR	AGLA014756	Anoplophora glabripennis	phosphatidylserine decarboxylase activity
EOG091203CR	APLA009513	Agrilus planipennis	phosphatidylserine decarboxylase activity
EOG091203CR	TC032021	Tribolium castaneum	phosphatidylserine decarboxylase activity
EOG091203CR	LDEC021225	Leptinotarsa decemlineata	phosphatidylserine decarboxylase activity
EOG091203CR	YQE_06822	Dendroctonus ponderosae	phosphatidylserine decarboxylase activity
EOG091203CU	OTAU000559	Onthophagus taurus	None
EOG091203CU	AGLA003273	Anoplophora glabripennis	None
EOG091203CU	APLA014324	Agrilus planipennis	None
EOG091203CU	TC031252	Tribolium castaneum	None
EOG091203CU	LDEC022904	Leptinotarsa decemlineata	None
EOG091203CU	YQE_12926	Dendroctonus ponderosae	None
EOG091203CW	OTAU007484	Onthophagus taurus	Putative uncharacterized protein
EOG091203CW	AGLA012738	Anoplophora glabripennis	Putative uncharacterized protein
EOG091203CW	APLA008751	Agrilus planipennis	Putative uncharacterized protein
EOG091203CW	TC014893	Tribolium castaneum	Putative uncharacterized protein
EOG091203CW	LDEC009951	Leptinotarsa decemlineata	Putative uncharacterized protein
EOG091203CW	YQE_10821	Dendroctonus ponderosae	Putative uncharacterized protein
EOG091203CX	OTAU004370	Onthophagus taurus	Putative uncharacterized protein
EOG091203CX	AGLA000338	Anoplophora glabripennis	Putative uncharacterized protein
EOG091203CX	APLA006427	Agrilus planipennis	Putative uncharacterized protein
EOG091203CX	TC010786	Tribolium castaneum	Putative uncharacterized protein
EOG091203CX	LDEC004712	Leptinotarsa decemlineata	Putative uncharacterized protein
EOG091203CX	YQE_02494	Dendroctonus ponderosae	Putative uncharacterized protein
EOG091203CY	OTAU006571	Onthophagus taurus	Putative uncharacterized protein
EOG091203CY	AGLA011694	Anoplophora glabripennis	Putative uncharacterized protein
EOG091203CY	APLA003744	Agrilus planipennis	Putative uncharacterized protein
EOG091203CY	TC002805	Tribolium castaneum	Putative uncharacterized protein
EOG091203CY	LDEC013201	Leptinotarsa decemlineata	Putative uncharacterized protein
EOG091203CY	YQE_07787	Dendroctonus ponderosae	Putative uncharacterized protein
EOG091203D0	OTAU014859	Onthophagus taurus	Putative uncharacterized protein

EOG091203D0	AGLA007814	Anoplophora glabripennis	Putative uncharacterized protein
EOG091203D0	APLA003210	Agrilus planipennis	Putative uncharacterized protein
EOG091203D0	TC001942	Tribolium castaneum	Putative uncharacterized protein
EOG091203D0	LDEC004245	Leptinotarsa decemlineata	Putative uncharacterized protein
EOG091203D0	YQE_12539	Dendroctonus ponderosae	Putative uncharacterized protein
EOG091203D1	OTAU005617	Onthophagus taurus	Putative uncharacterized protein
EOG091203D1	AGLA001323	Anoplophora glabripennis	Putative uncharacterized protein
EOG091203D1	APLA013463	Agrilus planipennis	Putative uncharacterized protein
EOG091203D1	TC001084	Tribolium castaneum	Putative uncharacterized protein
EOG091203D1	LDEC012376	Leptinotarsa decemlineata	Putative uncharacterized protein
EOG091203D1	YQE_01574	Dendroctonus ponderosae	Putative uncharacterized protein
EOG091203D3	OTAU003841	Onthophagus taurus	None
EOG091203D3	AGLA009442	Anoplophora glabripennis	None
EOG091203D3	APLA009318	Agrilus planipennis	None
EOG091203D3	TC033720	Tribolium castaneum	None
EOG091203D3	LDEC007550	Leptinotarsa decemlineata	None
EOG091203D3	YQE_13041	Dendroctonus ponderosae	None
EOG091203D6	OTAU010401	Onthophagus taurus	Putative uncharacterized protein
EOG091203D6	AGLA013173	Anoplophora glabripennis	Putative uncharacterized protein
EOG091203D6	APLA012197	Agrilus planipennis	Putative uncharacterized protein
EOG091203D6	TC016198	Tribolium castaneum	Putative uncharacterized protein
EOG091203D6	LDEC006392	Leptinotarsa decemlineata	Putative uncharacterized protein
EOG091203D6	YQE_05518	Dendroctonus ponderosae	Putative uncharacterized protein
EOG091203DB	OTAU005418	Onthophagus taurus	Putative uncharacterized protein
EOG091203DB	AGLA004569	Anoplophora glabripennis	Putative uncharacterized protein
EOG091203DB	APLA006413	Agrilus planipennis	Putative uncharacterized protein
EOG091203DB	TC001983	Tribolium castaneum	Putative uncharacterized protein
EOG091203DB	LDEC001532	Leptinotarsa decemlineata	Putative uncharacterized protein
EOG091203DB	YQE_09256	Dendroctonus ponderosae	Putative uncharacterized protein
EOG091203DC	OTAU000470	Onthophagus taurus	Putative uncharacterized protein
EOG091203DC	AGLA019098	Anoplophora glabripennis	Putative uncharacterized protein
EOG091203DC	APLA004000	Agrilus planipennis	Putative uncharacterized protein
EOG091203DC	TC030602	Tribolium castaneum	Putative uncharacterized protein
EOG091203DC	LDEC013101	Leptinotarsa decemlineata	Putative uncharacterized protein
EOG091203DC	YQE_01660	Dendroctonus ponderosae	Putative uncharacterized protein
EOG091203DD	OTAU008039	Onthophagus taurus	Putative uncharacterized protein
EOG091203DD	AGLA014099	Anoplophora glabripennis	Putative uncharacterized protein
EOG091203DD	APLA001435	Agrilus planipennis	Putative uncharacterized protein
EOG091203DD	TC015235	Tribolium castaneum	Putative uncharacterized protein
EOG091203DD	LDEC010567	Leptinotarsa decemlineata	Putative uncharacterized protein
EOG091203DD	YQE_06968	Dendroctonus ponderosae	Putative uncharacterized protein
EOG091203DF	OTAU006908	Onthophagus taurus	Putative uncharacterized protein
EOG091203DF	AGLA003858	Anoplophora glabripennis	Putative uncharacterized protein
EOG091203DF	APLA008834	Agrilus planipennis	Putative uncharacterized protein
EOG091203DF	TC009705	Tribolium castaneum	Putative uncharacterized protein
EOG091203DF	LDEC020033	Leptinotarsa decemlineata	Putative uncharacterized protein
EOG091203DF	YQE_04077	Dendroctonus ponderosae	Putative uncharacterized protein
EOG091203DL	OTAU001952	Onthophagus taurus	Putative uncharacterized protein
EOG091203DL	AGLA011051	Anoplophora glabripennis	Putative uncharacterized protein
EOG091203DL	APLA012836	Agrilus planipennis	Putative uncharacterized protein
EOG091203DL	TC005825	Tribolium castaneum	Putative uncharacterized protein
EOG091203DL	LDEC009304	Leptinotarsa decemlineata	Putative uncharacterized protein
EOG091203DL	YQE_12114	Dendroctonus ponderosae	Putative uncharacterized protein
EOG091203DM	OTAU002134	Onthophagus taurus	Serine/threonine-protein phosphatase
EOG091203DM	AGLA001482	Anoplophora glabripennis	Serine/threonine-protein phosphatase
EOG091203DM	APLA000289	Agrilus planipennis	Serine/threonine-protein phosphatase
EOG091203DM	TC000103	Tribolium castaneum	Serine/threonine-protein phosphatase
EOG091203DM	LDEC003293	Leptinotarsa decemlineata	Serine/threonine-protein phosphatase
EOG091203DM	YQE_09950	Dendroctonus ponderosae	Serine/threonine-protein phosphatase
EOG091203DN	OTAU005383	Onthophagus taurus	None
EOG091203DN	AGLA004555	Anoplophora glabripennis	None
EOG091203DN	APLA010659	Agrilus planipennis	None
EOG091203DN	TC031558	Tribolium castaneum	None
EOG091203DN	LDEC003517	Leptinotarsa decemlineata	None
EOG091203DN	YQE_09213	Dendroctonus ponderosae	None
EOG091203DO	OTAU004109	Onthophagus taurus	Putative uncharacterized protein
EOG091203DO	AGLA001700	Anoplophora glabripennis	Putative uncharacterized protein

EOG091203DO	APLA010087	Agrilus planipennis	Putative uncharacterized protein
EOG091203DO	TC009977	Tribolium castaneum	Putative uncharacterized protein
EOG091203DO	LDEC024498	Leptinotarsa decemlineata	Putative uncharacterized protein
EOG091203DO	YQE_03502	Dendroctonus ponderosae	Putative uncharacterized protein
EOG091203DQ	OTAU005147	Onthophagus taurus	Putative uncharacterized protein
EOG091203DQ	AGLA005181	Anoplophora glabripennis	Putative uncharacterized protein
EOG091203DQ	APLA000063	Agrilus planipennis	Putative uncharacterized protein
EOG091203DQ	TC007896	Tribolium castaneum	Putative uncharacterized protein
EOG091203DQ	LDEC009962	Leptinotarsa decemlineata	Putative uncharacterized protein
EOG091203DQ	YQE_07654	Dendroctonus ponderosae	Putative uncharacterized protein
EOG091203DR	OTAU016007	Onthophagus taurus	Putative uncharacterized protein
EOG091203DR	AGLA008005	Anoplophora glabripennis	Putative uncharacterized protein
EOG091203DR	APLA001701	Agrilus planipennis	Putative uncharacterized protein
EOG091203DR	TC030588	Tribolium castaneum	Putative uncharacterized protein
EOG091203DR	LDEC014297	Leptinotarsa decemlineata	Putative uncharacterized protein
EOG091203DR	YQE_01832	Dendroctonus ponderosae	Putative uncharacterized protein
EOG091203DS	OTAU003556	Onthophagus taurus	Putative uncharacterized protein
EOG091203DS	AGLA016103	Anoplophora glabripennis	Putative uncharacterized protein
EOG091203DS	APLA002647	Agrilus planipennis	Putative uncharacterized protein
EOG091203DS	TC002309	Tribolium castaneum	Putative uncharacterized protein
EOG091203DS	LDEC003844	Leptinotarsa decemlineata	Putative uncharacterized protein
EOG091203DS	YQE_08042	Dendroctonus ponderosae	Putative uncharacterized protein
EOG091203DU	OTAU011846	Onthophagus taurus	ionotropic glutamate receptor activity
EOG091203DU	AGLA007073	Anoplophora glabripennis	ionotropic glutamate receptor activity
EOG091203DU	APLA009903	Agrilus planipennis	ionotropic glutamate receptor activity
EOG091203DU	TC032065	Tribolium castaneum	ionotropic glutamate receptor activity
EOG091203DU	LDEC001661	Leptinotarsa decemlineata	ionotropic glutamate receptor activity
EOG091203DU	YQE_11187	Dendroctonus ponderosae	ionotropic glutamate receptor activity
EOG091203DV	OTAU003094	Onthophagus taurus	Putative uncharacterized protein
EOG091203DV	AGLA002086	Anoplophora glabripennis	Putative uncharacterized protein
EOG091203DV	APLA012858	Agrilus planipennis	Putative uncharacterized protein
EOG091203DV	TC030612	Tribolium castaneum	Putative uncharacterized protein
EOG091203DV	LDEC009189	Leptinotarsa decemlineata	Putative uncharacterized protein
EOG091203DV	YQE_06434	Dendroctonus ponderosae	Putative uncharacterized protein
EOG091203DX	OTAU008485	Onthophagus taurus	Putative uncharacterized protein
EOG091203DX	AGLA002094	Anoplophora glabripennis	Putative uncharacterized protein
EOG091203DX	APLA008150	Agrilus planipennis	Putative uncharacterized protein
EOG091203DX	TC003307	Tribolium castaneum	Putative uncharacterized protein
EOG091203DX	LDEC011999	Leptinotarsa decemlineata	Putative uncharacterized protein
EOG091203DX	YQE_07401	Dendroctonus ponderosae	Putative uncharacterized protein
EOG091203DY	OTAU009528	Onthophagus taurus	Putative uncharacterized protein
EOG091203DY	AGLA010630	Anoplophora glabripennis	Putative uncharacterized protein
EOG091203DY	APLA013879	Agrilus planipennis	Putative uncharacterized protein
EOG091203DY	TC009252	Tribolium castaneum	Putative uncharacterized protein
EOG091203DY	LDEC002364	Leptinotarsa decemlineata	Putative uncharacterized protein
EOG091203DY	YQE_05830	Dendroctonus ponderosae	Putative uncharacterized protein
EOG091203DZ	OTAU003239	Onthophagus taurus	Putative uncharacterized protein
EOG091203DZ	AGLA011816	Anoplophora glabripennis	Putative uncharacterized protein
EOG091203DZ	APLA001688	Agrilus planipennis	Putative uncharacterized protein
EOG091203DZ	TC009549	Tribolium castaneum	Putative uncharacterized protein
EOG091203DZ	LDEC024024	Leptinotarsa decemlineata	Putative uncharacterized protein
EOG091203DZ	YQE_11321	Dendroctonus ponderosae	Putative uncharacterized protein
EOG091203E1	OTAU009872	Onthophagus taurus	Methylthioribose-1-phosphate isomerase
EOG091203E1	AGLA016267	Anoplophora glabripennis	Methylthioribose-1-phosphate isomerase
EOG091203E1	APLA013006	Agrilus planipennis	Methylthioribose-1-phosphate isomerase
EOG091203E1	TC002054	Tribolium castaneum	Methylthioribose-1-phosphate isomerase
EOG091203E1	LDEC010352	Leptinotarsa decemlineata	Methylthioribose-1-phosphate isomerase
EOG091203E1	YQE_06832	Dendroctonus ponderosae	Methylthioribose-1-phosphate isomerase
EOG091203E2	OTAU007715	Onthophagus taurus	Sprouty-related protein with EVH-1 domain
EOG091203E2	AGLA003558	Anoplophora glabripennis	Sprouty-related protein with EVH-1 domain
EOG091203E2	APLA002001	Agrilus planipennis	Sprouty-related protein with EVH-1 domain
EOG091203E2	TC001559	Tribolium castaneum	Sprouty-related protein with EVH-1 domain
EOG091203E2	LDEC020556	Leptinotarsa decemlineata	Sprouty-related protein with EVH-1 domain
EOG091203E2	YQE_11117	Dendroctonus ponderosae	Sprouty-related protein with EVH-1 domain
EOG091203E3	OTAU002925	Onthophagus taurus	Putative uncharacterized protein
EOG091203E3	AGLA019631	Anoplophora glabripennis	Putative uncharacterized protein
EOG091203E3	APLA014527	Agrilus planipennis	Putative uncharacterized protein

EOG091203E3	TC009503	Tribolium castaneum	Putative uncharacterized protein
EOG091203E3	LDEC011029	Leptinotarsa decemlineata	Putative uncharacterized protein
EOG091203E3	YQE_02350	Dendroctonus ponderosae	Putative uncharacterized protein
EOG091203E5	OTAU000838	Onthophagus taurus	Putative uncharacterized protein
EOG091203E5	AGLA008432	Anoplophora glabripennis	Putative uncharacterized protein
EOG091203E5	APLA014978	Agrilus planipennis	Putative uncharacterized protein
EOG091203E5	TC011377	Tribolium castaneum	Putative uncharacterized protein
EOG091203E5	LDEC009148	Leptinotarsa decemlineata	Putative uncharacterized protein
EOG091203E5	YQE_04598	Dendroctonus ponderosae	Putative uncharacterized protein
EOG091203E6	OTAU003696	Onthophagus taurus	Putative uncharacterized protein
EOG091203E6	AGLA010135	Anoplophora glabripennis	Putative uncharacterized protein
EOG091203E6	APLA005405	Agrilus planipennis	Putative uncharacterized protein
EOG091203E6	TC007325	Tribolium castaneum	Putative uncharacterized protein
EOG091203E6	LDEC006010	Leptinotarsa decemlineata	Putative uncharacterized protein
EOG091203E6	YQE_04601	Dendroctonus ponderosae	Putative uncharacterized protein
EOG091203E7	OTAU009652	Onthophagus taurus	Putative uncharacterized protein
EOG091203E7	AGLA015667	Anoplophora glabripennis	Putative uncharacterized protein
EOG091203E7	APLA013593	Agrilus planipennis	Putative uncharacterized protein
EOG091203E7	TC000274	Tribolium castaneum	Putative uncharacterized protein
EOG091203E7	LDEC013060	Leptinotarsa decemlineata	Putative uncharacterized protein
EOG091203E7	YQE_07079	Dendroctonus ponderosae	Putative uncharacterized protein
EOG091203E9	OTAU001029	Onthophagus taurus	Putative uncharacterized protein
EOG091203E9	AGLA005231	Anoplophora glabripennis	Putative uncharacterized protein
EOG091203E9	APLA013002	Agrilus planipennis	Putative uncharacterized protein
EOG091203E9	TC004426	Tribolium castaneum	Putative uncharacterized protein
EOG091203E9	LDEC003561	Leptinotarsa decemlineata	Putative uncharacterized protein
EOG091203E9	YQE_11650	Dendroctonus ponderosae	Putative uncharacterized protein
EOG091203EA	OTAU007704	Onthophagus taurus	Putative uncharacterized protein
EOG091203EA	AGLA006184	Anoplophora glabripennis	Putative uncharacterized protein
EOG091203EA	APLA011484	Agrilus planipennis	Putative uncharacterized protein
EOG091203EA	TC003426	Tribolium castaneum	Putative uncharacterized protein
EOG091203EA	LDEC004487	Leptinotarsa decemlineata	Putative uncharacterized protein
EOG091203EA	YQE_03666	Dendroctonus ponderosae	Putative uncharacterized protein
EOG091203EC	OTAU000094	Onthophagus taurus	Putative uncharacterized protein
EOG091203EC	AGLA010620	Anoplophora glabripennis	Putative uncharacterized protein
EOG091203EC	APLA010722	Agrilus planipennis	Putative uncharacterized protein
EOG091203EC	TC014329	Tribolium castaneum	Putative uncharacterized protein
EOG091203EC	LDEC015134	Leptinotarsa decemlineata	Putative uncharacterized protein
EOG091203EC	YQE_06263	Dendroctonus ponderosae	Putative uncharacterized protein
EOG091203EF	OTAU016134	Onthophagus taurus	DNA binding
EOG091203EF	AGLA008562	Anoplophora glabripennis	DNA binding
EOG091203EF	APLA012856	Agrilus planipennis	DNA binding
EOG091203EF	TC033444	Tribolium castaneum	DNA binding
EOG091203EF	LDEC013736	Leptinotarsa decemlineata	DNA binding
EOG091203EF	YQE_10826	Dendroctonus ponderosae	DNA binding
EOG091203EG	OTAU000827	Onthophagus taurus	Putative uncharacterized protein
EOG091203EG	AGLA013984	Anoplophora glabripennis	Putative uncharacterized protein
EOG091203EG	APLA008957	Agrilus planipennis	Putative uncharacterized protein
EOG091203EG	TC011004	Tribolium castaneum	Putative uncharacterized protein
EOG091203EG	LDEC001713	Leptinotarsa decemlineata	Putative uncharacterized protein
EOG091203EG	YQE_10414	Dendroctonus ponderosae	Putative uncharacterized protein
EOG091203EI	OTAU000530	Onthophagus taurus	Putative uncharacterized protein
EOG091203EI	AGLA005969	Anoplophora glabripennis	Putative uncharacterized protein
EOG091203EI	APLA010032	Agrilus planipennis	Putative uncharacterized protein
EOG091203EI	TC002920	Tribolium castaneum	Putative uncharacterized protein
EOG091203EI	LDEC015389	Leptinotarsa decemlineata	Putative uncharacterized protein
EOG091203EI	YQE_06585	Dendroctonus ponderosae	Putative uncharacterized protein
EOG091203EN	OTAU008630	Onthophagus taurus	Putative uncharacterized protein
EOG091203EN	AGLA017529	Anoplophora glabripennis	Putative uncharacterized protein
EOG091203EN	APLA014098	Agrilus planipennis	Putative uncharacterized protein
EOG091203EN	TC001412	Tribolium castaneum	Putative uncharacterized protein
EOG091203EN	LDEC013647	Leptinotarsa decemlineata	Putative uncharacterized protein
EOG091203EN	YQE_12809	Dendroctonus ponderosae	Putative uncharacterized protein
EOG091203ET	OTAU005883	Onthophagus taurus	Putative uncharacterized protein
EOG091203ET	AGLA007028	Anoplophora glabripennis	Putative uncharacterized protein
EOG091203ET	APLA012129	Agrilus planipennis	Putative uncharacterized protein
EOG091203ET	TC010441	Tribolium castaneum	Putative uncharacterized protein

EOG091203ET	LDEC014802	Leptinotarsa decemlineata	Putative uncharacterized protein
EOG091203ET	YQE_07118	Dendroctonus ponderosae	Putative uncharacterized protein
EOG091203EU	OTAU001003	Onthophagus taurus	Putative uncharacterized protein
EOG091203EU	AGLA001901	Anoplophora glabripennis	Putative uncharacterized protein
EOG091203EU	APLA001331	Agrilus planipennis	Putative uncharacterized protein
EOG091203EU	TC000584	Tribolium castaneum	Putative uncharacterized protein
EOG091203EU	LDEC008143	Leptinotarsa decemlineata	Putative uncharacterized protein
EOG091203EU	YQE_08693	Dendroctonus ponderosae	Putative uncharacterized protein
EOG091203EV	OTAU004252	Onthophagus taurus	Putative uncharacterized protein
EOG091203EV	AGLA018198	Anoplophora glabripennis	Putative uncharacterized protein
EOG091203EV	APLA015276	Agrilus planipennis	Putative uncharacterized protein
EOG091203EV	TC013878	Tribolium castaneum	Putative uncharacterized protein
EOG091203EV	LDEC009508	Leptinotarsa decemlineata	Putative uncharacterized protein
EOG091203EV	YQE_00007	Dendroctonus ponderosae	Putative uncharacterized protein
EOG091203EY	OTAU007734	Onthophagus taurus	Putative uncharacterized protein
EOG091203EY	AGLA006069	Anoplophora glabripennis	Putative uncharacterized protein
EOG091203EY	APLA007354	Agrilus planipennis	Putative uncharacterized protein
EOG091203EY	TC013313	Tribolium castaneum	Putative uncharacterized protein
EOG091203EY	LDEC006676	Leptinotarsa decemlineata	Putative uncharacterized protein
EOG091203EY	YQE_04872	Dendroctonus ponderosae	Putative uncharacterized protein
EOG091203F1	OTAU008504	Onthophagus taurus	Putative uncharacterized protein
EOG091203F1	AGLA009560	Anoplophora glabripennis	Putative uncharacterized protein
EOG091203F1	APLA012971	Agrilus planipennis	Putative uncharacterized protein
EOG091203F1	TC010438	Tribolium castaneum	Putative uncharacterized protein
EOG091203F1	LDEC002603	Leptinotarsa decemlineata	Putative uncharacterized protein
EOG091203F1	YQE_10466	Dendroctonus ponderosae	Putative uncharacterized protein
EOG091203F3	OTAU006094	Onthophagus taurus	Putative uncharacterized protein
EOG091203F3	AGLA018398	Anoplophora glabripennis	Putative uncharacterized protein
EOG091203F3	APLA002685	Agrilus planipennis	Putative uncharacterized protein
EOG091203F3	TC013082	Tribolium castaneum	Putative uncharacterized protein
EOG091203F3	LDEC006511	Leptinotarsa decemlineata	Putative uncharacterized protein
EOG091203F3	YQE_05073	Dendroctonus ponderosae	Putative uncharacterized protein
EOG091203F4	OTAU003415	Onthophagus taurus	"transferase activity, transferring phosphorus-containing groups"
EOG091203F4	AGLA020439	Anoplophora glabripennis	"transferase activity, transferring phosphorus-containing groups"
EOG091203F4	APLA002509	Agrilus planipennis	"transferase activity, transferring phosphorus-containing groups"
EOG091203F4	TC031247	Tribolium castaneum	"transferase activity, transferring phosphorus-containing groups"
EOG091203F4	LDEC012288	Leptinotarsa decemlineata	"transferase activity, transferring phosphorus-containing groups"
EOG091203F4	YQE_12571	Dendroctonus ponderosae	"transferase activity, transferring phosphorus-containing groups"
EOG091203F6	OTAU003819	Onthophagus taurus	Protein Wnt
EOG091203F6	AGLA013044	Anoplophora glabripennis	Protein Wnt
EOG091203F6	APLA004752	Agrilus planipennis	Protein Wnt
EOG091203F6	TC033668	Tribolium castaneum	Protein Wnt
EOG091203F6	LDEC015458	Leptinotarsa decemlineata	Protein Wnt
EOG091203F6	YQE_06033	Dendroctonus ponderosae	Protein Wnt
EOG091203F9	OTAU000823	Onthophagus taurus	Putative uncharacterized protein
EOG091203F9	AGLA013485	Anoplophora glabripennis	Putative uncharacterized protein
EOG091203F9	APLA001349	Agrilus planipennis	Putative uncharacterized protein
EOG091203F9	TC004566	Tribolium castaneum	Putative uncharacterized protein
EOG091203F9	LDEC016755	Leptinotarsa decemlineata	Putative uncharacterized protein
EOG091203F9	YQE_10951	Dendroctonus ponderosae	Putative uncharacterized protein
EOG091203FD	OTAU014555	Onthophagus taurus	None
EOG091203FD	AGLA008556	Anoplophora glabripennis	None
EOG091203FD	APLA002554	Agrilus planipennis	None
EOG091203FD	TC033958	Tribolium castaneum	None
EOG091203FD	LDEC008014	Leptinotarsa decemlineata	None
EOG091203FD	YQE_11474	Dendroctonus ponderosae	None
EOG091203FH	OTAU014754	Onthophagus taurus	Putative uncharacterized protein
EOG091203FH	AGLA008425	Anoplophora glabripennis	Putative uncharacterized protein
EOG091203FH	APLA001529	Agrilus planipennis	Putative uncharacterized protein
EOG091203FH	TC016125	Tribolium castaneum	Putative uncharacterized protein
EOG091203FH	LDEC009149	Leptinotarsa decemlineata	Putative uncharacterized protein

EOG091203FH	YQE_08937	Dendroctonus ponderosae	Putative uncharacterized protein
EOG091203FI	OTAU012168	Onthophagus taurus	Putative uncharacterized protein
EOG091203FI	AGLA003741	Anoplophora glabripennis	Putative uncharacterized protein
EOG091203FI	APLA010137	Agrilus planipennis	Putative uncharacterized protein
EOG091203FI	TC007872	Tribolium castaneum	Putative uncharacterized protein
EOG091203FI	LDEC016677	Leptinotarsa decemlineata	Putative uncharacterized protein
EOG091203FI	YQE_07304	Dendroctonus ponderosae	Putative uncharacterized protein
EOG091203FL	OTAU002810	Onthophagus taurus	Putative uncharacterized protein
EOG091203FL	AGLA000277	Anoplophora glabripennis	Putative uncharacterized protein
EOG091203FL	APLA003666	Agrilus planipennis	Putative uncharacterized protein
EOG091203FL	TC014241	Tribolium castaneum	Putative uncharacterized protein
EOG091203FL	LDEC001115	Leptinotarsa decemlineata	Putative uncharacterized protein
EOG091203FL	YQE_06631	Dendroctonus ponderosae	Putative uncharacterized protein
EOG091203FS	OTAU006855	Onthophagus taurus	Putative uncharacterized protein
EOG091203FS	AGLA020116	Anoplophora glabripennis	Putative uncharacterized protein
EOG091203FS	APLA012136	Agrilus planipennis	Putative uncharacterized protein
EOG091203FS	TC014630	Tribolium castaneum	Putative uncharacterized protein
EOG091203FS	LDEC007758	Leptinotarsa decemlineata	Putative uncharacterized protein
EOG091203FS	YQE_07282	Dendroctonus ponderosae	Putative uncharacterized protein
EOG091203FT	OTAU003278	Onthophagus taurus	Putative uncharacterized protein
EOG091203FT	AGLA015709	Anoplophora glabripennis	Putative uncharacterized protein
EOG091203FT	APLA004633	Agrilus planipennis	Putative uncharacterized protein
EOG091203FT	TC008948	Tribolium castaneum	Putative uncharacterized protein
EOG091203FT	LDEC020883	Leptinotarsa decemlineata	Putative uncharacterized protein
EOG091203FT	YQE_11504	Dendroctonus ponderosae	Putative uncharacterized protein
EOG091203FU	OTAU013865	Onthophagus taurus	Putative uncharacterized protein
EOG091203FU	AGLA010428	Anoplophora glabripennis	Putative uncharacterized protein
EOG091203FU	APLA004045	Agrilus planipennis	Putative uncharacterized protein
EOG091203FU	TC015393	Tribolium castaneum	Putative uncharacterized protein
EOG091203FU	LDEC010455	Leptinotarsa decemlineata	Putative uncharacterized protein
EOG091203FU	YQE_10143	Dendroctonus ponderosae	Putative uncharacterized protein
EOG091203FV	OTAU008976	Onthophagus taurus	metal ion binding
EOG091203FV	AGLA005918	Anoplophora glabripennis	metal ion binding
EOG091203FV	APLA005553	Agrilus planipennis	metal ion binding
EOG091203FV	TC031254	Tribolium castaneum	metal ion binding
EOG091203FV	LDEC013208	Leptinotarsa decemlineata	metal ion binding
EOG091203FV	YQE_12873	Dendroctonus ponderosae	metal ion binding
EOG091203FX	OTAU000259	Onthophagus taurus	Putative uncharacterized protein
EOG091203FX	AGLA001504	Anoplophora glabripennis	Putative uncharacterized protein
EOG091203FX	APLA008492	Agrilus planipennis	Putative uncharacterized protein
EOG091203FX	TC014435	Tribolium castaneum	Putative uncharacterized protein
EOG091203FX	LDEC007012	Leptinotarsa decemlineata	Putative uncharacterized protein
EOG091203FX	YQE_05076	Dendroctonus ponderosae	Putative uncharacterized protein
EOG091203FY	OTAU001030	Onthophagus taurus	Putative uncharacterized protein
EOG091203FY	AGLA001573	Anoplophora glabripennis	Putative uncharacterized protein
EOG091203FY	APLA001346	Agrilus planipennis	Putative uncharacterized protein
EOG091203FY	TC016216	Tribolium castaneum	Putative uncharacterized protein
EOG091203FY	LDEC008931	Leptinotarsa decemlineata	Putative uncharacterized protein
EOG091203FY	YQE_07931	Dendroctonus ponderosae	Putative uncharacterized protein
EOG091203G0	OTAU002951	Onthophagus taurus	Putative uncharacterized protein
EOG091203G0	AGLA006821	Anoplophora glabripennis	Putative uncharacterized protein
EOG091203G0	APLA001842	Agrilus planipennis	Putative uncharacterized protein
EOG091203G0	TC009742	Tribolium castaneum	Putative uncharacterized protein
EOG091203G0	LDEC021221	Leptinotarsa decemlineata	Putative uncharacterized protein
EOG091203G0	YQE_10033	Dendroctonus ponderosae	Putative uncharacterized protein
EOG091203G1	OTAU006371	Onthophagus taurus	Spindle A-like protein
EOG091203G1	AGLA020278	Anoplophora glabripennis	Spindle A-like protein
EOG091203G1	APLA008808	Agrilus planipennis	Spindle A-like protein
EOG091203G1	TC007805	Tribolium castaneum	Spindle A-like protein
EOG091203G1	LDEC011013	Leptinotarsa decemlineata	Spindle A-like protein
EOG091203G1	YQE_05308	Dendroctonus ponderosae	Spindle A-like protein
EOG091203G2	OTAU015077	Onthophagus taurus	Galactose-1-phosphate uridylyltransferase
EOG091203G2	AGLA003371	Anoplophora glabripennis	Galactose-1-phosphate uridylyltransferase
EOG091203G2	APLA001518	Agrilus planipennis	Galactose-1-phosphate uridylyltransferase
EOG091203G2	TC032578	Tribolium castaneum	Galactose-1-phosphate uridylyltransferase
EOG091203G2	LDEC010961	Leptinotarsa decemlineata	Galactose-1-phosphate uridylyltransferase
EOG091203G2	YQE_03790	Dendroctonus ponderosae	Galactose-1-phosphate uridylyltransferase

EOG091203G3	OTAU008879	Onthophagus taurus	cAMP-dependent protein kinase regulator activity
EOG091203G3	AGLA004504	Anoplophora glabripennis	cAMP-dependent protein kinase regulator activity
EOG091203G3	APLA009906	Agrilus planipennis	cAMP-dependent protein kinase regulator activity
EOG091203G3	TC032066	Tribolium castaneum	cAMP-dependent protein kinase regulator activity
EOG091203G3	LDEC014769	Leptinotarsa decemlineata	cAMP-dependent protein kinase regulator activity
EOG091203G3	YQE_06428	Dendroctonus ponderosae	cAMP-dependent protein kinase regulator activity
EOG091203G4	OTAU000376	Onthophagus taurus	Putative uncharacterized protein
EOG091203G4	AGLA005088	Anoplophora glabripennis	Putative uncharacterized protein
EOG091203G4	APLA014057	Agrilus planipennis	Putative uncharacterized protein
EOG091203G4	TC011911	Tribolium castaneum	Putative uncharacterized protein
EOG091203G4	LDEC005506	Leptinotarsa decemlineata	Putative uncharacterized protein
EOG091203G4	YQE_01642	Dendroctonus ponderosae	Putative uncharacterized protein
EOG091203G6	OTAU016178	Onthophagus taurus	Putative uncharacterized protein
EOG091203G6	AGLA020371	Anoplophora glabripennis	Putative uncharacterized protein
EOG091203G6	APLA008465	Agrilus planipennis	Putative uncharacterized protein
EOG091203G6	TC006629	Tribolium castaneum	Putative uncharacterized protein
EOG091203G6	LDEC016557	Leptinotarsa decemlineata	Putative uncharacterized protein
EOG091203G6	YQE_06357	Dendroctonus ponderosae	Putative uncharacterized protein
EOG091203G8	OTAU014753	Onthophagus taurus	Putative uncharacterized protein
EOG091203G8	AGLA001354	Anoplophora glabripennis	Putative uncharacterized protein
EOG091203G8	APLA001526	Agrilus planipennis	Putative uncharacterized protein
EOG091203G8	TC011005	Tribolium castaneum	Putative uncharacterized protein
EOG091203G8	LDEC003551	Leptinotarsa decemlineata	Putative uncharacterized protein
EOG091203G8	YQE_06142	Dendroctonus ponderosae	Putative uncharacterized protein
EOG091203G9	OTAU003572	Onthophagus taurus	Putative uncharacterized protein
EOG091203G9	AGLA005681	Anoplophora glabripennis	Putative uncharacterized protein
EOG091203G9	APLA002390	Agrilus planipennis	Putative uncharacterized protein
EOG091203G9	TC012706	Tribolium castaneum	Putative uncharacterized protein
EOG091203G9	LDEC012145	Leptinotarsa decemlineata	Putative uncharacterized protein
EOG091203G9	YQE_08429	Dendroctonus ponderosae	Putative uncharacterized protein
EOG091203GC	OTAU014926	Onthophagus taurus	Putative uncharacterized protein
EOG091203GC	AGLA012398	Anoplophora glabripennis	Putative uncharacterized protein
EOG091203GC	APLA010679	Agrilus planipennis	Putative uncharacterized protein
EOG091203GC	TC011138	Tribolium castaneum	Putative uncharacterized protein
EOG091203GC	LDEC013488	Leptinotarsa decemlineata	Putative uncharacterized protein
EOG091203GC	YQE_01325	Dendroctonus ponderosae	Putative uncharacterized protein
EOG091203GD	OTAU003614	Onthophagus taurus	Putative uncharacterized protein
EOG091203GD	AGLA015158	Anoplophora glabripennis	Putative uncharacterized protein
EOG091203GD	APLA003741	Agrilus planipennis	Putative uncharacterized protein
EOG091203GD	TC002734	Tribolium castaneum	Putative uncharacterized protein
EOG091203GD	LDEC013131	Leptinotarsa decemlineata	Putative uncharacterized protein
EOG091203GD	YQE_07908	Dendroctonus ponderosae	Putative uncharacterized protein
EOG091203GH	OTAU001866	Onthophagus taurus	Putative uncharacterized protein
EOG091203GH	AGLA015012	Anoplophora glabripennis	Putative uncharacterized protein
EOG091203GH	APLA011060	Agrilus planipennis	Putative uncharacterized protein
EOG091203GH	TC005719	Tribolium castaneum	Putative uncharacterized protein
EOG091203GH	LDEC005870	Leptinotarsa decemlineata	Putative uncharacterized protein
EOG091203GH	YQE_05585	Dendroctonus ponderosae	Putative uncharacterized protein
EOG091203GI	OTAU005239	Onthophagus taurus	Putative uncharacterized protein
EOG091203GI	AGLA004091	Anoplophora glabripennis	Putative uncharacterized protein
EOG091203GI	APLA013306	Agrilus planipennis	Putative uncharacterized protein
EOG091203GI	TC015954	Tribolium castaneum	Putative uncharacterized protein
EOG091203GI	LDEC004414	Leptinotarsa decemlineata	Putative uncharacterized protein
EOG091203GI	YQE_03911	Dendroctonus ponderosae	Putative uncharacterized protein
EOG091203GJ	OTAU008995	Onthophagus taurus	Putative uncharacterized protein
EOG091203GJ	AGLA008010	Anoplophora glabripennis	Putative uncharacterized protein
EOG091203GJ	APLA012866	Agrilus planipennis	Putative uncharacterized protein
EOG091203GJ	TC015120	Tribolium castaneum	Putative uncharacterized protein
EOG091203GJ	LDEC017783	Leptinotarsa decemlineata	Putative uncharacterized protein
EOG091203GJ	YQE_11465	Dendroctonus ponderosae	Putative uncharacterized protein
EOG091203GL	OTAU007632	Onthophagus taurus	Putative uncharacterized protein
EOG091203GL	AGLA011844	Anoplophora glabripennis	Putative uncharacterized protein
EOG091203GL	APLA008825	Agrilus planipennis	Putative uncharacterized protein
EOG091203GL	TC009845	Tribolium castaneum	Putative uncharacterized protein
EOG091203GL	LDEC004803	Leptinotarsa decemlineata	Putative uncharacterized protein
EOG091203GL	YQE_02190	Dendroctonus ponderosae	Putative uncharacterized protein
EOG091203GM	OTAU001745	Onthophagus taurus	Putative uncharacterized protein

EOG091203GM	AGLA018312	Anoplophora glabripennis	Putative uncharacterized protein
EOG091203GM	APLA012293	Agrilus planipennis	Putative uncharacterized protein
EOG091203GM	TC005820	Tribolium castaneum	Putative uncharacterized protein
EOG091203GM	LDEC004922	Leptinotarsa decemlineata	Putative uncharacterized protein
EOG091203GM	YQE_06938	Dendroctonus ponderosae	Putative uncharacterized protein
EOG091203GN	OTAU011703	Onthophagus taurus	Aminomethyltransferase
EOG091203GN	AGLA018222	Anoplophora glabripennis	Aminomethyltransferase
EOG091203GN	APLA005967	Agrilus planipennis	Aminomethyltransferase
EOG091203GN	TC015522	Tribolium castaneum	Aminomethyltransferase
EOG091203GN	LDEC003678	Leptinotarsa decemlineata	Aminomethyltransferase
EOG091203GN	YQE_05934	Dendroctonus ponderosae	Aminomethyltransferase
EOG091203GR	OTAU002822	Onthophagus taurus	Putative uncharacterized protein
EOG091203GR	AGLA007614	Anoplophora glabripennis	Putative uncharacterized protein
EOG091203GR	APLA003683	Agrilus planipennis	Putative uncharacterized protein
EOG091203GR	TC010871	Tribolium castaneum	Putative uncharacterized protein
EOG091203GR	LDEC015734	Leptinotarsa decemlineata	Putative uncharacterized protein
EOG091203GR	YQE_06691	Dendroctonus ponderosae	Putative uncharacterized protein
EOG091203GS	OTAU002775	Onthophagus taurus	Putative uncharacterized protein
EOG091203GS	AGLA000430	Anoplophora glabripennis	Putative uncharacterized protein
EOG091203GS	APLA006770	Agrilus planipennis	Putative uncharacterized protein
EOG091203GS	TC004703	Tribolium castaneum	Putative uncharacterized protein
EOG091203GS	LDEC008365	Leptinotarsa decemlineata	Putative uncharacterized protein
EOG091203GS	YQE_03298	Dendroctonus ponderosae	Putative uncharacterized protein
EOG091203GT	OTAU008240	Onthophagus taurus	Putative uncharacterized protein
EOG091203GT	AGLA021689	Anoplophora glabripennis	Putative uncharacterized protein
EOG091203GT	APLA002949	Agrilus planipennis	Putative uncharacterized protein
EOG091203GT	TC000771	Tribolium castaneum	Putative uncharacterized protein
EOG091203GT	LDEC005670	Leptinotarsa decemlineata	Putative uncharacterized protein
EOG091203GT	YQE_07343	Dendroctonus ponderosae	Putative uncharacterized protein
EOG091203GX	OTAU011415	Onthophagus taurus	Putative uncharacterized protein
EOG091203GX	AGLA021257	Anoplophora glabripennis	Putative uncharacterized protein
EOG091203GX	APLA009760	Agrilus planipennis	Putative uncharacterized protein
EOG091203GX	TC005393	Tribolium castaneum	Putative uncharacterized protein
EOG091203GX	LDEC005007	Leptinotarsa decemlineata	Putative uncharacterized protein
EOG091203GX	YQE_05770	Dendroctonus ponderosae	Putative uncharacterized protein
EOG091203GY	OTAU012257	Onthophagus taurus	Putative uncharacterized protein
EOG091203GY	AGLA019742	Anoplophora glabripennis	Putative uncharacterized protein
EOG091203GY	APLA000771	Agrilus planipennis	Putative uncharacterized protein
EOG091203GY	TC000049	Tribolium castaneum	Putative uncharacterized protein
EOG091203GY	LDEC005014	Leptinotarsa decemlineata	Putative uncharacterized protein
EOG091203GY	YQE_11858	Dendroctonus ponderosae	Putative uncharacterized protein
EOG091203H0	OTAU008248	Onthophagus taurus	Putative uncharacterized protein
EOG091203H0	AGLA013771	Anoplophora glabripennis	Putative uncharacterized protein
EOG091203H0	APLA000148	Agrilus planipennis	Putative uncharacterized protein
EOG091203H0	TC003874	Tribolium castaneum	Putative uncharacterized protein
EOG091203H0	LDEC013595	Leptinotarsa decemlineata	Putative uncharacterized protein
EOG091203H0	YQE_12353	Dendroctonus ponderosae	Putative uncharacterized protein
EOG091203H2	OTAU002240	Onthophagus taurus	Putative uncharacterized protein
EOG091203H2	AGLA004451	Anoplophora glabripennis	Putative uncharacterized protein
EOG091203H2	APLA001489	Agrilus planipennis	Putative uncharacterized protein
EOG091203H2	TC003998	Tribolium castaneum	Putative uncharacterized protein
EOG091203H2	LDEC022146	Leptinotarsa decemlineata	Putative uncharacterized protein
EOG091203H2	YQE_01786	Dendroctonus ponderosae	Putative uncharacterized protein
EOG091203H4	OTAU013909	Onthophagus taurus	Cuticular protein analogous to peritrophins 1-J
EOG091203H4	AGLA000177	Anoplophora glabripennis	Cuticular protein analogous to peritrophins 1-J
EOG091203H4	APLA001937	Agrilus planipennis	Cuticular protein analogous to peritrophins 1-J
EOG091203H4	TC011101	Tribolium castaneum	Cuticular protein analogous to peritrophins 1-J
EOG091203H4	LDEC015701	Leptinotarsa decemlineata	Cuticular protein analogous to peritrophins 1-J
EOG091203H4	YQE_01590	Dendroctonus ponderosae	Cuticular protein analogous to peritrophins 1-J
EOG091203H6	OTAU013373	Onthophagus taurus	Putative uncharacterized protein
EOG091203H6	AGLA001216	Anoplophora glabripennis	Putative uncharacterized protein
EOG091203H6	APLA009855	Agrilus planipennis	Putative uncharacterized protein
EOG091203H6	TC005644	Tribolium castaneum	Putative uncharacterized protein
EOG091203H6	LDEC019265	Leptinotarsa decemlineata	Putative uncharacterized protein
EOG091203H6	YQE_12024	Dendroctonus ponderosae	Putative uncharacterized protein
EOG091203H8	OTAU016655	Onthophagus taurus	Gram-negative bacteria binding protein 3
EOG091203H8	AGLA004274	Anoplophora glabripennis	Gram-negative bacteria binding protein 3

EOG091203H8	APLA000762	Agrilus planipennis	Gram-negative bacteria binding protein 3
EOG091203H8	TC003991	Tribolium castaneum	Gram-negative bacteria binding protein 3
EOG091203H8	LDEC011305	Leptinotarsa decemlineata	Gram-negative bacteria binding protein 3
EOG091203H8	YQE_08530	Dendroctonus ponderosae	Gram-negative bacteria binding protein 3
EOG091203HA	OTAU001882	Onthophagus taurus	Arginyl-tRNA--protein transferase 1
EOG091203HA	AGLA010180	Anoplophora glabripennis	Arginyl-tRNA--protein transferase 1
EOG091203HA	APLA000926	Agrilus planipennis	Arginyl-tRNA--protein transferase 1
EOG091203HA	TC005959	Tribolium castaneum	Arginyl-tRNA--protein transferase 1
EOG091203HA	LDEC006789	Leptinotarsa decemlineata	Arginyl-tRNA--protein transferase 1
EOG091203HA	YQE_06923	Dendroctonus ponderosae	Arginyl-tRNA--protein transferase 1
EOG091203HB	OTAU001516	Onthophagus taurus	Putative uncharacterized protein
EOG091203HB	AGLA003821	Anoplophora glabripennis	Putative uncharacterized protein
EOG091203HB	APLA013240	Agrilus planipennis	Putative uncharacterized protein
EOG091203HB	TC009913	Tribolium castaneum	Putative uncharacterized protein
EOG091203HB	LDEC011994	Leptinotarsa decemlineata	Putative uncharacterized protein
EOG091203HB	YQE_07682	Dendroctonus ponderosae	Putative uncharacterized protein
EOG091203HC	OTAU005676	Onthophagus taurus	Putative uncharacterized protein
EOG091203HC	AGLA014815	Anoplophora glabripennis	Putative uncharacterized protein
EOG091203HC	APLA014022	Agrilus planipennis	Putative uncharacterized protein
EOG091203HC	TC030543	Tribolium castaneum	Putative uncharacterized protein
EOG091203HC	LDEC004789	Leptinotarsa decemlineata	Putative uncharacterized protein
EOG091203HC	YQE_08854	Dendroctonus ponderosae	Putative uncharacterized protein
EOG091203HJ	OTAU007808	Onthophagus taurus	Putative uncharacterized protein
EOG091203HJ	AGLA002885	Anoplophora glabripennis	Putative uncharacterized protein
EOG091203HJ	APLA012253	Agrilus planipennis	Putative uncharacterized protein
EOG091203HJ	TC007480	Tribolium castaneum	Putative uncharacterized protein
EOG091203HJ	LDEC000747	Leptinotarsa decemlineata	Putative uncharacterized protein
EOG091203HJ	YQE_03017	Dendroctonus ponderosae	Putative uncharacterized protein
EOG091203HK	OTAU010151	Onthophagus taurus	polysaccharide binding
EOG091203HK	AGLA007186	Anoplophora glabripennis	polysaccharide binding
EOG091203HK	APLA004892	Agrilus planipennis	polysaccharide binding
EOG091203HK	TC033581	Tribolium castaneum	polysaccharide binding
EOG091203HK	LDEC014889	Leptinotarsa decemlineata	polysaccharide binding
EOG091203HK	YQE_11802	Dendroctonus ponderosae	polysaccharide binding
EOG091203HM	OTAU014896	Onthophagus taurus	Putative uncharacterized protein
EOG091203HM	AGLA003992	Anoplophora glabripennis	Putative uncharacterized protein
EOG091203HM	APLA009837	Agrilus planipennis	Putative uncharacterized protein
EOG091203HM	TC013312	Tribolium castaneum	Putative uncharacterized protein
EOG091203HM	LDEC020566	Leptinotarsa decemlineata	Putative uncharacterized protein
EOG091203HM	YQE_10374	Dendroctonus ponderosae	Putative uncharacterized protein
EOG091203HO	OTAU002986	Onthophagus taurus	DNA binding
EOG091203HO	AGLA013007	Anoplophora glabripennis	DNA binding
EOG091203HO	APLA014136	Agrilus planipennis	DNA binding
EOG091203HO	TC033576	Tribolium castaneum	DNA binding
EOG091203HO	LDEC004804	Leptinotarsa decemlineata	DNA binding
EOG091203HO	YQE_03520	Dendroctonus ponderosae	DNA binding
EOG091203HP	OTAU006873	Onthophagus taurus	Putative uncharacterized protein
EOG091203HP	AGLA016077	Anoplophora glabripennis	Putative uncharacterized protein
EOG091203HP	APLA002689	Agrilus planipennis	Putative uncharacterized protein
EOG091203HP	TC013083	Tribolium castaneum	Putative uncharacterized protein
EOG091203HP	LDEC006508	Leptinotarsa decemlineata	Putative uncharacterized protein
EOG091203HP	YQE_06130	Dendroctonus ponderosae	Putative uncharacterized protein
EOG091203HQ	OTAU001619	Onthophagus taurus	Putative uncharacterized protein
EOG091203HQ	AGLA016923	Anoplophora glabripennis	Putative uncharacterized protein
EOG091203HQ	APLA005481	Agrilus planipennis	Putative uncharacterized protein
EOG091203HQ	TC006139	Tribolium castaneum	Putative uncharacterized protein
EOG091203HQ	LDEC015047	Leptinotarsa decemlineata	Putative uncharacterized protein
EOG091203HQ	YQE_01915	Dendroctonus ponderosae	Putative uncharacterized protein
EOG091203HT	OTAU001250	Onthophagus taurus	Putative uncharacterized protein
EOG091203HT	AGLA000219	Anoplophora glabripennis	Putative uncharacterized protein
EOG091203HT	APLA009998	Agrilus planipennis	Putative uncharacterized protein
EOG091203HT	TC010766	Tribolium castaneum	Putative uncharacterized protein
EOG091203HT	LDEC001068	Leptinotarsa decemlineata	Putative uncharacterized protein
EOG091203HT	YQE_09080	Dendroctonus ponderosae	Putative uncharacterized protein
EOG091203HU	OTAU003999	Onthophagus taurus	Putative uncharacterized protein
EOG091203HU	AGLA013017	Anoplophora glabripennis	Putative uncharacterized protein
EOG091203HU	APLA013781	Agrilus planipennis	Putative uncharacterized protein

EOG091203HU	TC002332	Tribolium castaneum	Putative uncharacterized protein
EOG091203HU	LDEC004834	Leptinotarsa decemlineata	Putative uncharacterized protein
EOG091203HU	YQE_08774	Dendroctonus ponderosae	Putative uncharacterized protein
EOG091203HY	OTAU012033	Onthophagus taurus	ATP binding
EOG091203HY	AGLA001295	Anoplophora glabripennis	ATP binding
EOG091203HY	APLA011790	Agrilus planipennis	ATP binding
EOG091203HY	TC035002	Tribolium castaneum	ATP binding
EOG091203HY	LDEC011350	Leptinotarsa decemlineata	ATP binding
EOG091203HY	YQE_08446	Dendroctonus ponderosae	ATP binding
EOG091203HZ	OTAU012098	Onthophagus taurus	Putative uncharacterized protein
EOG091203HZ	AGLA010997	Anoplophora glabripennis	Putative uncharacterized protein
EOG091203HZ	APLA010948	Agrilus planipennis	Putative uncharacterized protein
EOG091203HZ	TC008343	Tribolium castaneum	Putative uncharacterized protein
EOG091203HZ	LDEC009538	Leptinotarsa decemlineata	Putative uncharacterized protein
EOG091203HZ	YQE_04797	Dendroctonus ponderosae	Putative uncharacterized protein
EOG091203I3	OTAU002750	Onthophagus taurus	Putative uncharacterized protein
EOG091203I3	AGLA020826	Anoplophora glabripennis	Putative uncharacterized protein
EOG091203I3	APLA014538	Agrilus planipennis	Putative uncharacterized protein
EOG091203I3	TC001774	Tribolium castaneum	Putative uncharacterized protein
EOG091203I3	LDEC001038	Leptinotarsa decemlineata	Putative uncharacterized protein
EOG091203I3	YQE_09147	Dendroctonus ponderosae	Putative uncharacterized protein
EOG091203I4	OTAU001112	Onthophagus taurus	Putative uncharacterized protein
EOG091203I4	AGLA006103	Anoplophora glabripennis	Putative uncharacterized protein
EOG091203I4	APLA010417	Agrilus planipennis	Putative uncharacterized protein
EOG091203I4	TC000297	Tribolium castaneum	Putative uncharacterized protein
EOG091203I4	LDEC013258	Leptinotarsa decemlineata	Putative uncharacterized protein
EOG091203I4	YQE_08134	Dendroctonus ponderosae	Putative uncharacterized protein
EOG091203I7	OTAU007499	Onthophagus taurus	nucleic acid binding
EOG091203I7	AGLA013710	Anoplophora glabripennis	nucleic acid binding
EOG091203I7	APLA015191	Agrilus planipennis	nucleic acid binding
EOG091203I7	TC031865	Tribolium castaneum	nucleic acid binding
EOG091203I7	LDEC012243	Leptinotarsa decemlineata	nucleic acid binding
EOG091203I7	YQE_05938	Dendroctonus ponderosae	nucleic acid binding
EOG091203IB	OTAU000266	Onthophagus taurus	Putative uncharacterized protein
EOG091203IB	AGLA002655	Anoplophora glabripennis	Putative uncharacterized protein
EOG091203IB	APLA002737	Agrilus planipennis	Putative uncharacterized protein
EOG091203IB	TC013376	Tribolium castaneum	Putative uncharacterized protein
EOG091203IB	LDEC018017	Leptinotarsa decemlineata	Putative uncharacterized protein
EOG091203IB	YQE_10659	Dendroctonus ponderosae	Putative uncharacterized protein
EOG091203IC	OTAU003423	Onthophagus taurus	Ftz transcription factor 1
EOG091203IC	AGLA013765	Anoplophora glabripennis	Ftz transcription factor 1
EOG091203IC	APLA006720	Agrilus planipennis	Ftz transcription factor 1
EOG091203IC	TC002550	Tribolium castaneum	Ftz transcription factor 1
EOG091203IC	LDEC014512	Leptinotarsa decemlineata	Ftz transcription factor 1
EOG091203IC	YQE_06806	Dendroctonus ponderosae	Ftz transcription factor 1
EOG091203ID	OTAU005713	Onthophagus taurus	zinc ion binding
EOG091203ID	AGLA006897	Anoplophora glabripennis	zinc ion binding
EOG091203ID	APLA000297	Agrilus planipennis	zinc ion binding
EOG091203ID	TC032347	Tribolium castaneum	zinc ion binding
EOG091203ID	LDEC000233	Leptinotarsa decemlineata	zinc ion binding
EOG091203ID	YQE_06443	Dendroctonus ponderosae	zinc ion binding
EOG091203IE	OTAU003961	Onthophagus taurus	Putative uncharacterized protein
EOG091203IE	AGLA006038	Anoplophora glabripennis	Putative uncharacterized protein
EOG091203IE	APLA002617	Agrilus planipennis	Putative uncharacterized protein
EOG091203IE	TC001944	Tribolium castaneum	Putative uncharacterized protein
EOG091203IE	LDEC005145	Leptinotarsa decemlineata	Putative uncharacterized protein
EOG091203IE	YQE_11982	Dendroctonus ponderosae	Putative uncharacterized protein
EOG091203IF	OTAU000711	Onthophagus taurus	Putative uncharacterized protein
EOG091203IF	AGLA004617	Anoplophora glabripennis	Putative uncharacterized protein
EOG091203IF	APLA001765	Agrilus planipennis	Putative uncharacterized protein
EOG091203IF	TC011960	Tribolium castaneum	Putative uncharacterized protein
EOG091203IF	LDEC022104	Leptinotarsa decemlineata	Putative uncharacterized protein
EOG091203IF	YQE_03435	Dendroctonus ponderosae	Putative uncharacterized protein
EOG091203IH	OTAU012924	Onthophagus taurus	Putative uncharacterized protein
EOG091203IH	AGLA016434	Anoplophora glabripennis	Putative uncharacterized protein
EOG091203IH	APLA007478	Agrilus planipennis	Putative uncharacterized protein
EOG091203IH	TC005664	Tribolium castaneum	Putative uncharacterized protein

EOG091203IH	LDEC019086	Leptinotarsa decemlineata	Putative uncharacterized protein
EOG091203IH	YQE_12705	Dendroctonus ponderosae	Putative uncharacterized protein
EOG091203IP	OTAU013737	Onthophagus taurus	Putative uncharacterized protein
EOG091203IP	AGLA020987	Anoplophora glabripennis	Putative uncharacterized protein
EOG091203IP	APLA005558	Agrilus planipennis	Putative uncharacterized protein
EOG091203IP	TC012698	Tribolium castaneum	Putative uncharacterized protein
EOG091203IP	LDEC004128	Leptinotarsa decemlineata	Putative uncharacterized protein
EOG091203IP	YQE_10167	Dendroctonus ponderosae	Putative uncharacterized protein
EOG091203IR	OTAU015005	Onthophagus taurus	Putative uncharacterized protein
EOG091203IR	AGLA003376	Anoplophora glabripennis	Putative uncharacterized protein
EOG091203IR	APLA002088	Agrilus planipennis	Putative uncharacterized protein
EOG091203IR	TC011516	Tribolium castaneum	Putative uncharacterized protein
EOG091203IR	LDEC023580	Leptinotarsa decemlineata	Putative uncharacterized protein
EOG091203IR	YQE_03795	Dendroctonus ponderosae	Putative uncharacterized protein
EOG091203IX	OTAU005204	Onthophagus taurus	Putative uncharacterized protein
EOG091203IX	AGLA015540	Anoplophora glabripennis	Putative uncharacterized protein
EOG091203IX	APLA005728	Agrilus planipennis	Putative uncharacterized protein
EOG091203IX	TC010592	Tribolium castaneum	Putative uncharacterized protein
EOG091203IX	LDEC014656	Leptinotarsa decemlineata	Putative uncharacterized protein
EOG091203IX	YQE_10510	Dendroctonus ponderosae	Putative uncharacterized protein
EOG091203IY	OTAU000099	Onthophagus taurus	Putative uncharacterized protein
EOG091203IY	AGLA015340	Anoplophora glabripennis	Putative uncharacterized protein
EOG091203IY	APLA009379	Agrilus planipennis	Putative uncharacterized protein
EOG091203IY	TC013369	Tribolium castaneum	Putative uncharacterized protein
EOG091203IY	LDEC005956	Leptinotarsa decemlineata	Putative uncharacterized protein
EOG091203IY	YQE_08239	Dendroctonus ponderosae	Putative uncharacterized protein
EOG091203J2	OTAU006376	Onthophagus taurus	Putative uncharacterized protein
EOG091203J2	AGLA006493	Anoplophora glabripennis	Putative uncharacterized protein
EOG091203J2	APLA014029	Agrilus planipennis	Putative uncharacterized protein
EOG091203J2	TC010161	Tribolium castaneum	Putative uncharacterized protein
EOG091203J2	LDEC002247	Leptinotarsa decemlineata	Putative uncharacterized protein
EOG091203J2	YQE_10619	Dendroctonus ponderosae	Putative uncharacterized protein
EOG091203J3	OTAU009704	Onthophagus taurus	Putative uncharacterized protein
EOG091203J3	AGLA000791	Anoplophora glabripennis	Putative uncharacterized protein
EOG091203J3	APLA006586	Agrilus planipennis	Putative uncharacterized protein
EOG091203J3	TC008220	Tribolium castaneum	Putative uncharacterized protein
EOG091203J3	LDEC004392	Leptinotarsa decemlineata	Putative uncharacterized protein
EOG091203J3	YQE_12129	Dendroctonus ponderosae	Putative uncharacterized protein
EOG091203J4	OTAU014674	Onthophagus taurus	NAD+ kinase activity
EOG091203J4	AGLA013005	Anoplophora glabripennis	NAD+ kinase activity
EOG091203J4	APLA013222	Agrilus planipennis	NAD+ kinase activity
EOG091203J4	TC034764	Tribolium castaneum	NAD+ kinase activity
EOG091203J4	LDEC008966	Leptinotarsa decemlineata	NAD+ kinase activity
EOG091203J4	YQE_01928	Dendroctonus ponderosae	NAD+ kinase activity
EOG091203J5	OTAU001901	Onthophagus taurus	Putative uncharacterized protein
EOG091203J5	AGLA009302	Anoplophora glabripennis	Putative uncharacterized protein
EOG091203J5	APLA000396	Agrilus planipennis	Putative uncharacterized protein
EOG091203J5	TC005424	Tribolium castaneum	Putative uncharacterized protein
EOG091203J5	LDEC021830	Leptinotarsa decemlineata	Putative uncharacterized protein
EOG091203J5	YQE_12755	Dendroctonus ponderosae	Putative uncharacterized protein
EOG091203J6	OTAU005113	Onthophagus taurus	protein kinase binding
EOG091203J6	AGLA003407	Anoplophora glabripennis	protein kinase binding
EOG091203J6	APLA014045	Agrilus planipennis	protein kinase binding
EOG091203J6	TC034617	Tribolium castaneum	protein kinase binding
EOG091203J6	LDEC015828	Leptinotarsa decemlineata	protein kinase binding
EOG091203J6	YQE_09981	Dendroctonus ponderosae	protein kinase binding
EOG091203J8	OTAU002799	Onthophagus taurus	sequence-specific DNA binding
EOG091203J8	AGLA002387	Anoplophora glabripennis	sequence-specific DNA binding
EOG091203J8	APLA003534	Agrilus planipennis	sequence-specific DNA binding
EOG091203J8	TC031481	Tribolium castaneum	sequence-specific DNA binding
EOG091203J8	LDEC011960	Leptinotarsa decemlineata	sequence-specific DNA binding
EOG091203J8	YQE_03277	Dendroctonus ponderosae	sequence-specific DNA binding
EOG091203J9	OTAU017047	Onthophagus taurus	Putative uncharacterized protein
EOG091203J9	AGLA006853	Anoplophora glabripennis	Putative uncharacterized protein
EOG091203J9	APLA003945	Agrilus planipennis	Putative uncharacterized protein
EOG091203J9	TC012656	Tribolium castaneum	Putative uncharacterized protein
EOG091203J9	LDEC004172	Leptinotarsa decemlineata	Putative uncharacterized protein

EOG091203J9	YQE_04390	Dendroctonus ponderosae	Putative uncharacterized protein
EOG091203JA	OTAU012034	Onthophagus taurus	Grapes
EOG091203JA	AGLA019366	Anoplophora glabripennis	Grapes
EOG091203JA	APLA011794	Agrilus planipennis	Grapes
EOG091203JA	TC001409	Tribolium castaneum	Grapes
EOG091203JA	LDEC018517	Leptinotarsa decemlineata	Grapes
EOG091203JA	YQE_11060	Dendroctonus ponderosae	Grapes
EOG091203JB	OTAU001041	Onthophagus taurus	Spatzle 5
EOG091203JB	AGLA013548	Anoplophora glabripennis	Spatzle 5
EOG091203JB	APLA003134	Agrilus planipennis	Spatzle 5
EOG091203JB	TC011304	Tribolium castaneum	Spatzle 5
EOG091203JB	LDEC009132	Leptinotarsa decemlineata	Spatzle 5
EOG091203JB	YQE_01750	Dendroctonus ponderosae	Spatzle 5
EOG091203JD	OTAU006624	Onthophagus taurus	Putative uncharacterized protein
EOG091203JD	AGLA004620	Anoplophora glabripennis	Putative uncharacterized protein
EOG091203JD	APLA013279	Agrilus planipennis	Putative uncharacterized protein
EOG091203JD	TC003901	Tribolium castaneum	Putative uncharacterized protein
EOG091203JD	LDEC018775	Leptinotarsa decemlineata	Putative uncharacterized protein
EOG091203JD	YQE_10214	Dendroctonus ponderosae	Putative uncharacterized protein
EOG091203JE	OTAU015840	Onthophagus taurus	Putative uncharacterized protein
EOG091203JE	AGLA015669	Anoplophora glabripennis	Putative uncharacterized protein
EOG091203JE	APLA013594	Agrilus planipennis	Putative uncharacterized protein
EOG091203JE	TC000275	Tribolium castaneum	Putative uncharacterized protein
EOG091203JE	LDEC013255	Leptinotarsa decemlineata	Putative uncharacterized protein
EOG091203JE	YQE_10681	Dendroctonus ponderosae	Putative uncharacterized protein
EOG091203JH	OTAU014304	Onthophagus taurus	zinc ion binding
EOG091203JH	AGLA009807	Anoplophora glabripennis	zinc ion binding
EOG091203JH	APLA010101	Agrilus planipennis	zinc ion binding
EOG091203JH	TC033186	Tribolium castaneum	zinc ion binding
EOG091203JH	LDEC013868	Leptinotarsa decemlineata	zinc ion binding
EOG091203JH	YQE_11288	Dendroctonus ponderosae	zinc ion binding
EOG091203JI	OTAU000977	Onthophagus taurus	Putative uncharacterized protein
EOG091203JI	AGLA008487	Anoplophora glabripennis	Putative uncharacterized protein
EOG091203JI	APLA010587	Agrilus planipennis	Putative uncharacterized protein
EOG091203JI	TC014819	Tribolium castaneum	Putative uncharacterized protein
EOG091203JI	LDEC016034	Leptinotarsa decemlineata	Putative uncharacterized protein
EOG091203JI	YQE_09624	Dendroctonus ponderosae	Putative uncharacterized protein
EOG091203JN	OTAU000627	Onthophagus taurus	Putative uncharacterized protein
EOG091203JN	AGLA017462	Anoplophora glabripennis	Putative uncharacterized protein
EOG091203JN	APLA002324	Agrilus planipennis	Putative uncharacterized protein
EOG091203JN	TC012803	Tribolium castaneum	Putative uncharacterized protein
EOG091203JN	LDEC015590	Leptinotarsa decemlineata	Putative uncharacterized protein
EOG091203JN	YQE_06473	Dendroctonus ponderosae	Putative uncharacterized protein
EOG091203JP	OTAU000802	Onthophagus taurus	Putative uncharacterized protein
EOG091203JP	AGLA001325	Anoplophora glabripennis	Putative uncharacterized protein
EOG091203JP	APLA007703	Agrilus planipennis	Putative uncharacterized protein
EOG091203JP	TC001083	Tribolium castaneum	Putative uncharacterized protein
EOG091203JP	LDEC012379	Leptinotarsa decemlineata	Putative uncharacterized protein
EOG091203JP	YQE_01805	Dendroctonus ponderosae	Putative uncharacterized protein
EOG091203JQ	OTAU004619	Onthophagus taurus	Putative uncharacterized protein
EOG091203JQ	AGLA000474	Anoplophora glabripennis	Putative uncharacterized protein
EOG091203JQ	APLA000218	Agrilus planipennis	Putative uncharacterized protein
EOG091203JQ	TC007802	Tribolium castaneum	Putative uncharacterized protein
EOG091203JQ	LDEC000954	Leptinotarsa decemlineata	Putative uncharacterized protein
EOG091203JQ	YQE_07052	Dendroctonus ponderosae	Putative uncharacterized protein
EOG091203JR	OTAU006021	Onthophagus taurus	None
EOG091203JR	AGLA000690	Anoplophora glabripennis	None
EOG091203JR	APLA012538	Agrilus planipennis	None
EOG091203JR	TC032835	Tribolium castaneum	None
EOG091203JR	LDEC018307	Leptinotarsa decemlineata	None
EOG091203JR	YQE_09705	Dendroctonus ponderosae	None
EOG091203JS	OTAU015435	Onthophagus taurus	Putative uncharacterized protein
EOG091203JS	AGLA007257	Anoplophora glabripennis	Putative uncharacterized protein
EOG091203JS	APLA000348	Agrilus planipennis	Putative uncharacterized protein
EOG091203JS	TC005893	Tribolium castaneum	Putative uncharacterized protein
EOG091203JS	LDEC004890	Leptinotarsa decemlineata	Putative uncharacterized protein
EOG091203JS	YQE_12067	Dendroctonus ponderosae	Putative uncharacterized protein

EOG091203JT	OTAU014704	Onthophagus taurus	Putative uncharacterized protein
EOG091203JT	AGLA013628	Anoplophora glabripennis	Putative uncharacterized protein
EOG091203JT	APLA006165	Agrilus planipennis	Putative uncharacterized protein
EOG091203JT	TC011346	Tribolium castaneum	Putative uncharacterized protein
EOG091203JT	LDEC010436	Leptinotarsa decemlineata	Putative uncharacterized protein
EOG091203JT	YQE_11037	Dendroctonus ponderosae	Putative uncharacterized protein
EOG091203JV	OTAU006800	Onthophagus taurus	Putative uncharacterized protein
EOG091203JV	AGLA003410	Anoplophora glabripennis	Putative uncharacterized protein
EOG091203JV	APLA010329	Agrilus planipennis	Putative uncharacterized protein
EOG091203JV	TC003819	Tribolium castaneum	Putative uncharacterized protein
EOG091203JV	LDEC015831	Leptinotarsa decemlineata	Putative uncharacterized protein
EOG091203JV	YQE_12310	Dendroctonus ponderosae	Putative uncharacterized protein
EOG091203JX	OTAU001240	Onthophagus taurus	Putative uncharacterized protein
EOG091203JX	AGLA000422	Anoplophora glabripennis	Putative uncharacterized protein
EOG091203JX	APLA010438	Agrilus planipennis	Putative uncharacterized protein
EOG091203JX	TC004616	Tribolium castaneum	Putative uncharacterized protein
EOG091203JX	LDEC011083	Leptinotarsa decemlineata	Putative uncharacterized protein
EOG091203JX	YQE_06725	Dendroctonus ponderosae	Putative uncharacterized protein
EOG091203K1	OTAU011495	Onthophagus taurus	Putative uncharacterized protein
EOG091203K1	AGLA009755	Anoplophora glabripennis	Putative uncharacterized protein
EOG091203K1	APLA005059	Agrilus planipennis	Putative uncharacterized protein
EOG091203K1	TC005968	Tribolium castaneum	Putative uncharacterized protein
EOG091203K1	LDEC012304	Leptinotarsa decemlineata	Putative uncharacterized protein
EOG091203K1	YQE_12766	Dendroctonus ponderosae	Putative uncharacterized protein
EOG091203K6	OTAU005873	Onthophagus taurus	Putative uncharacterized protein
EOG091203K6	AGLA001787	Anoplophora glabripennis	Putative uncharacterized protein
EOG091203K6	APLA003585	Agrilus planipennis	Putative uncharacterized protein
EOG091203K6	TC014580	Tribolium castaneum	Putative uncharacterized protein
EOG091203K6	LDEC015567	Leptinotarsa decemlineata	Putative uncharacterized protein
EOG091203K6	YQE_08640	Dendroctonus ponderosae	Putative uncharacterized protein
EOG091203K7	OTAU016031	Onthophagus taurus	Putative uncharacterized protein
EOG091203K7	AGLA007008	Anoplophora glabripennis	Putative uncharacterized protein
EOG091203K7	APLA003761	Agrilus planipennis	Putative uncharacterized protein
EOG091203K7	TC003883	Tribolium castaneum	Putative uncharacterized protein
EOG091203K7	LDEC016708	Leptinotarsa decemlineata	Putative uncharacterized protein
EOG091203K7	YQE_09866	Dendroctonus ponderosae	Putative uncharacterized protein
EOG091203K8	OTAU008361	Onthophagus taurus	Putative uncharacterized protein
EOG091203K8	AGLA019435	Anoplophora glabripennis	Putative uncharacterized protein
EOG091203K8	APLA008944	Agrilus planipennis	Putative uncharacterized protein
EOG091203K8	TC011160	Tribolium castaneum	Putative uncharacterized protein
EOG091203K8	LDEC000008	Leptinotarsa decemlineata	Putative uncharacterized protein
EOG091203K8	YQE_09666	Dendroctonus ponderosae	Putative uncharacterized protein
EOG091203KA	OTAU002710	Onthophagus taurus	Putative uncharacterized protein
EOG091203KA	AGLA018062	Anoplophora glabripennis	Putative uncharacterized protein
EOG091203KA	APLA007363	Agrilus planipennis	Putative uncharacterized protein
EOG091203KA	TC010464	Tribolium castaneum	Putative uncharacterized protein
EOG091203KA	LDEC005661	Leptinotarsa decemlineata	Putative uncharacterized protein
EOG091203KA	YQE_01726	Dendroctonus ponderosae	Putative uncharacterized protein
EOG091203KD	OTAU017160	Onthophagus taurus	ionotropic glutamate receptor activity
EOG091203KD	AGLA006579	Anoplophora glabripennis	ionotropic glutamate receptor activity
EOG091203KD	APLA010559	Agrilus planipennis	ionotropic glutamate receptor activity
EOG091203KD	TC032853	Tribolium castaneum	ionotropic glutamate receptor activity
EOG091203KD	LDEC017662	Leptinotarsa decemlineata	ionotropic glutamate receptor activity
EOG091203KD	YQE_08083	Dendroctonus ponderosae	ionotropic glutamate receptor activity
EOG091203KE	OTAU011760	Onthophagus taurus	Putative uncharacterized protein
EOG091203KE	AGLA002813	Anoplophora glabripennis	Putative uncharacterized protein
EOG091203KE	APLA013656	Agrilus planipennis	Putative uncharacterized protein
EOG091203KE	TC009781	Tribolium castaneum	Putative uncharacterized protein
EOG091203KE	LDEC015012	Leptinotarsa decemlineata	Putative uncharacterized protein
EOG091203KE	YQE_11548	Dendroctonus ponderosae	Putative uncharacterized protein
EOG091203KF	OTAU001962	Onthophagus taurus	Putative uncharacterized protein
EOG091203KF	AGLA009308	Anoplophora glabripennis	Putative uncharacterized protein
EOG091203KF	APLA012292	Agrilus planipennis	Putative uncharacterized protein
EOG091203KF	TC030618	Tribolium castaneum	Putative uncharacterized protein
EOG091203KF	LDEC003273	Leptinotarsa decemlineata	Putative uncharacterized protein
EOG091203KF	YQE_12095	Dendroctonus ponderosae	Putative uncharacterized protein
EOG091203KJ	OTAU011660	Onthophagus taurus	Putative uncharacterized protein

EOG091203KJ	AGLA017804	Anoplophora glabripennis	Putative uncharacterized protein
EOG091203KJ	APLA001555	Agrilus planipennis	Putative uncharacterized protein
EOG091203KJ	TC009674	Tribolium castaneum	Putative uncharacterized protein
EOG091203KJ	LDEC012868	Leptinotarsa decemlineata	Putative uncharacterized protein
EOG091203KJ	YQE_08822	Dendroctonus ponderosae	Putative uncharacterized protein
EOG091203KM	OTAU015299	Onthophagus taurus	Putative uncharacterized protein
EOG091203KM	AGLA009011	Anoplophora glabripennis	Putative uncharacterized protein
EOG091203KM	APLA002410	Agrilus planipennis	Putative uncharacterized protein
EOG091203KM	TC016306	Tribolium castaneum	Putative uncharacterized protein
EOG091203KM	LDEC005337	Leptinotarsa decemlineata	Putative uncharacterized protein
EOG091203KM	YQE_10477	Dendroctonus ponderosae	Putative uncharacterized protein
EOG091203KO	OTAU001903	Onthophagus taurus	S-adenosylmethionine decarboxylase proenzyme
EOG091203KO	AGLA017733	Anoplophora glabripennis	S-adenosylmethionine decarboxylase proenzyme
EOG091203KO	APLA001210	Agrilus planipennis	S-adenosylmethionine decarboxylase proenzyme
EOG091203KO	TC005583	Tribolium castaneum	S-adenosylmethionine decarboxylase proenzyme
EOG091203KO	LDEC011694	Leptinotarsa decemlineata	S-adenosylmethionine decarboxylase proenzyme
EOG091203KO	YQE_07471	Dendroctonus ponderosae	S-adenosylmethionine decarboxylase proenzyme
EOG091203KQ	OTAU003294	Onthophagus taurus	Putative uncharacterized protein
EOG091203KQ	AGLA000088	Anoplophora glabripennis	Putative uncharacterized protein
EOG091203KQ	APLA004881	Agrilus planipennis	Putative uncharacterized protein
EOG091203KQ	TC014209	Tribolium castaneum	Putative uncharacterized protein
EOG091203KQ	LDEC007429	Leptinotarsa decemlineata	Putative uncharacterized protein
EOG091203KQ	YQE_06710	Dendroctonus ponderosae	Putative uncharacterized protein
EOG091203KV	OTAU007783	Onthophagus taurus	None
EOG091203KV	AGLA008266	Anoplophora glabripennis	None
EOG091203KV	APLA001651	Agrilus planipennis	None
EOG091203KV	TC033755	Tribolium castaneum	None
EOG091203KV	LDEC004788	Leptinotarsa decemlineata	None
EOG091203KV	YQE_11930	Dendroctonus ponderosae	None
EOG091203KW	OTAU012030	Onthophagus taurus	Putative uncharacterized protein
EOG091203KW	AGLA006067	Anoplophora glabripennis	Putative uncharacterized protein
EOG091203KW	APLA007352	Agrilus planipennis	Putative uncharacterized protein
EOG091203KW	TC013314	Tribolium castaneum	Putative uncharacterized protein
EOG091203KW	LDEC022614	Leptinotarsa decemlineata	Putative uncharacterized protein
EOG091203KW	YQE_04871	Dendroctonus ponderosae	Putative uncharacterized protein
EOG091203KZ	OTAU011802	Onthophagus taurus	nucleotide binding
EOG091203KZ	AGLA001668	Anoplophora glabripennis	nucleotide binding
EOG091203KZ	APLA000487	Agrilus planipennis	nucleotide binding
EOG091203KZ	TC033943	Tribolium castaneum	nucleotide binding
EOG091203KZ	LDEC011201	Leptinotarsa decemlineata	nucleotide binding
EOG091203KZ	YQE_12770	Dendroctonus ponderosae	nucleotide binding
EOG091203L0	OTAU001972	Onthophagus taurus	Putative uncharacterized protein
EOG091203L0	AGLA018476	Anoplophora glabripennis	Putative uncharacterized protein
EOG091203L0	APLA011055	Agrilus planipennis	Putative uncharacterized protein
EOG091203L0	TC006415	Tribolium castaneum	Putative uncharacterized protein
EOG091203L0	LDEC006766	Leptinotarsa decemlineata	Putative uncharacterized protein
EOG091203L0	YQE_12791	Dendroctonus ponderosae	Putative uncharacterized protein
EOG091203L1	OTAU000814	Onthophagus taurus	Putative uncharacterized protein
EOG091203L1	AGLA001408	Anoplophora glabripennis	Putative uncharacterized protein
EOG091203L1	APLA004382	Agrilus planipennis	Putative uncharacterized protein
EOG091203L1	TC004435	Tribolium castaneum	Putative uncharacterized protein
EOG091203L1	LDEC013381	Leptinotarsa decemlineata	Putative uncharacterized protein
EOG091203L1	YQE_07738	Dendroctonus ponderosae	Putative uncharacterized protein
EOG091203L5	OTAU011710	Onthophagus taurus	Putative uncharacterized protein
EOG091203L5	AGLA014126	Anoplophora glabripennis	Putative uncharacterized protein
EOG091203L5	APLA011502	Agrilus planipennis	Putative uncharacterized protein
EOG091203L5	TC002107	Tribolium castaneum	Putative uncharacterized protein
EOG091203L5	LDEC002271	Leptinotarsa decemlineata	Putative uncharacterized protein
EOG091203L5	YQE_02933	Dendroctonus ponderosae	Putative uncharacterized protein
EOG091203L6	OTAU002202	Onthophagus taurus	None
EOG091203L6	AGLA003664	Anoplophora glabripennis	None
EOG091203L6	APLA008707	Agrilus planipennis	None
EOG091203L6	TC034593	Tribolium castaneum	None
EOG091203L6	LDEC003319	Leptinotarsa decemlineata	None
EOG091203L6	YQE_05197	Dendroctonus ponderosae	None
EOG091203L7	OTAU008764	Onthophagus taurus	Putative uncharacterized protein
EOG091203L7	AGLA008564	Anoplophora glabripennis	Putative uncharacterized protein

EOG091203L7	APLA003859	Agrilus planipennis	Putative uncharacterized protein
EOG091203L7	TC015756	Tribolium castaneum	Putative uncharacterized protein
EOG091203L7	LDEC013739	Leptinotarsa decemlineata	Putative uncharacterized protein
EOG091203L7	YQE_10798	Dendroctonus ponderosae	Putative uncharacterized protein
EOG091203LF	OTAU000439	Onthophagus taurus	Putative uncharacterized protein
EOG091203LF	AGLA011191	Anoplophora glabripennis	Putative uncharacterized protein
EOG091203LF	APLA015194	Agrilus planipennis	Putative uncharacterized protein
EOG091203LF	TC011621	Tribolium castaneum	Putative uncharacterized protein
EOG091203LF	LDEC016610	Leptinotarsa decemlineata	Putative uncharacterized protein
EOG091203LF	YQE_08024	Dendroctonus ponderosae	Putative uncharacterized protein
EOG091203LH	OTAU002694	Onthophagus taurus	Putative uncharacterized protein
EOG091203LH	AGLA016712	Anoplophora glabripennis	Putative uncharacterized protein
EOG091203LH	APLA002428	Agrilus planipennis	Putative uncharacterized protein
EOG091203LH	TC013318	Tribolium castaneum	Putative uncharacterized protein
EOG091203LH	LDEC009380	Leptinotarsa decemlineata	Putative uncharacterized protein
EOG091203LH	YQE_07278	Dendroctonus ponderosae	Putative uncharacterized protein
EOG091203LI	OTAU010828	Onthophagus taurus	Putative uncharacterized protein
EOG091203LI	AGLA003690	Anoplophora glabripennis	Putative uncharacterized protein
EOG091203LI	APLA009237	Agrilus planipennis	Putative uncharacterized protein
EOG091203LI	TC007206	Tribolium castaneum	Putative uncharacterized protein
EOG091203LI	LDEC012728	Leptinotarsa decemlineata	Putative uncharacterized protein
EOG091203LI	YQE_04206	Dendroctonus ponderosae	Putative uncharacterized protein
EOG091203LJ	OTAU001788	Onthophagus taurus	Putative uncharacterized protein
EOG091203LJ	AGLA001204	Anoplophora glabripennis	Putative uncharacterized protein
EOG091203LJ	APLA000443	Agrilus planipennis	Putative uncharacterized protein
EOG091203LJ	TC006794	Tribolium castaneum	Putative uncharacterized protein
EOG091203LJ	LDEC018497	Leptinotarsa decemlineata	Putative uncharacterized protein
EOG091203LJ	YQE_12118	Dendroctonus ponderosae	Putative uncharacterized protein
EOG091203LM	OTAU012808	Onthophagus taurus	Putative uncharacterized protein
EOG091203LM	AGLA010485	Anoplophora glabripennis	Putative uncharacterized protein
EOG091203LM	APLA011915	Agrilus planipennis	Putative uncharacterized protein
EOG091203LM	TC001120	Tribolium castaneum	Putative uncharacterized protein
EOG091203LM	LDEC002745	Leptinotarsa decemlineata	Putative uncharacterized protein
EOG091203LM	YQE_07347	Dendroctonus ponderosae	Putative uncharacterized protein
EOG091203LO	OTAU000948	Onthophagus taurus	Oo18 RNA-binding protein
EOG091203LO	AGLA013170	Anoplophora glabripennis	Oo18 RNA-binding protein
EOG091203LO	APLA009868	Agrilus planipennis	Oo18 RNA-binding protein
EOG091203LO	TC011262	Tribolium castaneum	Oo18 RNA-binding protein
EOG091203LO	LDEC000806	Leptinotarsa decemlineata	Oo18 RNA-binding protein
EOG091203LO	YQE_10950	Dendroctonus ponderosae	Oo18 RNA-binding protein
EOG091203LQ	OTAU016640	Onthophagus taurus	E78 nuclear receptor
EOG091203LQ	AGLA002007	Anoplophora glabripennis	E78 nuclear receptor
EOG091203LQ	APLA007620	Agrilus planipennis	E78 nuclear receptor
EOG091203LQ	TC003935	Tribolium castaneum	E78 nuclear receptor
EOG091203LQ	LDEC004576	Leptinotarsa decemlineata	E78 nuclear receptor
EOG091203LQ	YQE_12388	Dendroctonus ponderosae	E78 nuclear receptor
EOG091203LS	OTAU007994	Onthophagus taurus	Putative uncharacterized protein
EOG091203LS	AGLA006182	Anoplophora glabripennis	Putative uncharacterized protein
EOG091203LS	APLA006057	Agrilus planipennis	Putative uncharacterized protein
EOG091203LS	TC003427	Tribolium castaneum	Putative uncharacterized protein
EOG091203LS	LDEC004484	Leptinotarsa decemlineata	Putative uncharacterized protein
EOG091203LS	YQE_03650	Dendroctonus ponderosae	Putative uncharacterized protein
EOG091203LU	OTAU002989	Onthophagus taurus	Putative uncharacterized protein
EOG091203LU	AGLA010351	Anoplophora glabripennis	Putative uncharacterized protein
EOG091203LU	APLA001848	Agrilus planipennis	Putative uncharacterized protein
EOG091203LU	TC010122	Tribolium castaneum	Putative uncharacterized protein
EOG091203LU	LDEC001329	Leptinotarsa decemlineata	Putative uncharacterized protein
EOG091203LU	YQE_01565	Dendroctonus ponderosae	Putative uncharacterized protein
EOG091203LV	OTAU011611	Onthophagus taurus	Putative uncharacterized protein
EOG091203LV	AGLA018703	Anoplophora glabripennis	Putative uncharacterized protein
EOG091203LV	APLA013234	Agrilus planipennis	Putative uncharacterized protein
EOG091203LV	TC009436	Tribolium castaneum	Putative uncharacterized protein
EOG091203LV	LDEC002642	Leptinotarsa decemlineata	Putative uncharacterized protein
EOG091203LV	YQE_13028	Dendroctonus ponderosae	Putative uncharacterized protein
EOG091203LY	OTAU005604	Onthophagus taurus	None
EOG091203LY	AGLA011819	Anoplophora glabripennis	None
EOG091203LY	APLA010184	Agrilus planipennis	None

EOG091203LY	TC033702	Tribolium castaneum	None
EOG091203LY	LDEC001211	Leptinotarsa decemlineata	None
EOG091203LY	YQE_11326	Dendroctonus ponderosae	None
EOG091203LZ	OTAU002743	Onthophagus taurus	Putative uncharacterized protein
EOG091203LZ	AGLA001164	Anoplophora glabripennis	Putative uncharacterized protein
EOG091203LZ	APLA012809	Agrilus planipennis	Putative uncharacterized protein
EOG091203LZ	TC004633	Tribolium castaneum	Putative uncharacterized protein
EOG091203LZ	LDEC019564	Leptinotarsa decemlineata	Putative uncharacterized protein
EOG091203LZ	YQE_06713	Dendroctonus ponderosae	Putative uncharacterized protein
EOG091203M3	OTAU001228	Onthophagus taurus	Putative uncharacterized protein
EOG091203M3	AGLA002169	Anoplophora glabripennis	Putative uncharacterized protein
EOG091203M3	APLA013634	Agrilus planipennis	Putative uncharacterized protein
EOG091203M3	TC010804	Tribolium castaneum	Putative uncharacterized protein
EOG091203M3	LDEC001518	Leptinotarsa decemlineata	Putative uncharacterized protein
EOG091203M3	YQE_02472	Dendroctonus ponderosae	Putative uncharacterized protein
EOG091203M4	OTAU012333	Onthophagus taurus	Putative uncharacterized protein
EOG091203M4	AGLA016451	Anoplophora glabripennis	Putative uncharacterized protein
EOG091203M4	APLA003185	Agrilus planipennis	Putative uncharacterized protein
EOG091203M4	TC008489	Tribolium castaneum	Putative uncharacterized protein
EOG091203M4	LDEC020948	Leptinotarsa decemlineata	Putative uncharacterized protein
EOG091203M4	YQE_05707	Dendroctonus ponderosae	Putative uncharacterized protein
EOG091203M5	OTAU003142	Onthophagus taurus	Putative uncharacterized protein
EOG091203M5	AGLA012573	Anoplophora glabripennis	Putative uncharacterized protein
EOG091203M5	APLA013811	Agrilus planipennis	Putative uncharacterized protein
EOG091203M5	TC000060	Tribolium castaneum	Putative uncharacterized protein
EOG091203M5	LDEC005038	Leptinotarsa decemlineata	Putative uncharacterized protein
EOG091203M5	YQE_12286	Dendroctonus ponderosae	Putative uncharacterized protein
EOG091203M7	OTAU003852	Onthophagus taurus	Putative uncharacterized protein
EOG091203M7	AGLA014882	Anoplophora glabripennis	Putative uncharacterized protein
EOG091203M7	APLA010865	Agrilus planipennis	Putative uncharacterized protein
EOG091203M7	TC008844	Tribolium castaneum	Putative uncharacterized protein
EOG091203M7	LDEC007807	Leptinotarsa decemlineata	Putative uncharacterized protein
EOG091203M7	YQE_08749	Dendroctonus ponderosae	Putative uncharacterized protein
EOG091203M8	OTAU008567	Onthophagus taurus	Serine protease P84
EOG091203M8	AGLA011208	Anoplophora glabripennis	Serine protease P84
EOG091203M8	APLA011102	Agrilus planipennis	Serine protease P84
EOG091203M8	TC008657	Tribolium castaneum	Serine protease P84
EOG091203M8	LDEC007695	Leptinotarsa decemlineata	Serine protease P84
EOG091203M8	YQE_12588	Dendroctonus ponderosae	Serine protease P84
EOG091203MD	OTAU011583	Onthophagus taurus	Putative uncharacterized protein
EOG091203MD	AGLA020388	Anoplophora glabripennis	Putative uncharacterized protein
EOG091203MD	APLA000273	Agrilus planipennis	Putative uncharacterized protein
EOG091203MD	TC002288	Tribolium castaneum	Putative uncharacterized protein
EOG091203MD	LDEC004949	Leptinotarsa decemlineata	Putative uncharacterized protein
EOG091203MD	YQE_03725	Dendroctonus ponderosae	Putative uncharacterized protein
EOG091203MG	OTAU011491	Onthophagus taurus	Putative uncharacterized protein
EOG091203MG	AGLA008869	Anoplophora glabripennis	Putative uncharacterized protein
EOG091203MG	APLA000462	Agrilus planipennis	Putative uncharacterized protein
EOG091203MG	TC005312	Tribolium castaneum	Putative uncharacterized protein
EOG091203MG	LDEC016166	Leptinotarsa decemlineata	Putative uncharacterized protein
EOG091203MG	YQE_12049	Dendroctonus ponderosae	Putative uncharacterized protein
EOG091203MH	OTAU007717	Onthophagus taurus	Putative uncharacterized protein
EOG091203MH	AGLA021247	Anoplophora glabripennis	Putative uncharacterized protein
EOG091203MH	APLA002016	Agrilus planipennis	Putative uncharacterized protein
EOG091203MH	TC011549	Tribolium castaneum	Putative uncharacterized protein
EOG091203MH	LDEC005931	Leptinotarsa decemlineata	Putative uncharacterized protein
EOG091203MH	YQE_05164	Dendroctonus ponderosae	Putative uncharacterized protein
EOG091203MJ	OTAU002697	Onthophagus taurus	"translation release factor activity, codon specific"
EOG091203MJ	AGLA008314	Anoplophora glabripennis	"translation release factor activity, codon specific"
EOG091203MJ	APLA003069	Agrilus planipennis	"translation release factor activity, codon specific"
EOG091203MJ	TC033022	Tribolium castaneum	"translation release factor activity, codon specific"
EOG091203MJ	LDEC005358	Leptinotarsa decemlineata	"translation release factor activity, codon specific"
EOG091203MJ	YQE_11372	Dendroctonus ponderosae	"translation release factor activity, codon specific"
EOG091203ML	OTAU008759	Onthophagus taurus	Putative uncharacterized protein
EOG091203ML	AGLA003339	Anoplophora glabripennis	Putative uncharacterized protein
EOG091203ML	APLA009467	Agrilus planipennis	Putative uncharacterized protein
EOG091203ML	TC015749	Tribolium castaneum	Putative uncharacterized protein

EOG091203ML	LDEC007880	Leptinotarsa decemlineata	Putative uncharacterized protein
EOG091203ML	YQE_11675	Dendroctonus ponderosae	Putative uncharacterized protein
EOG091203MO	OTAU005537	Onthophagus taurus	Star
EOG091203MO	AGLA017375	Anoplophora glabripennis	Star
EOG091203MO	APLA010402	Agrilus planipennis	Star
EOG091203MO	TC012408	Tribolium castaneum	Star
EOG091203MO	LDEC013098	Leptinotarsa decemlineata	Star
EOG091203MO	YQE_11599	Dendroctonus ponderosae	Star
EOG091203MP	OTAU004368	Onthophagus taurus	None
EOG091203MP	AGLA004562	Anoplophora glabripennis	None
EOG091203MP	APLA006429	Agrilus planipennis	None
EOG091203MP	TC031541	Tribolium castaneum	None
EOG091203MP	LDEC004725	Leptinotarsa decemlineata	None
EOG091203MP	YQE_05966	Dendroctonus ponderosae	None
EOG091203MR	OTAU004387	Onthophagus taurus	None
EOG091203MR	AGLA002152	Anoplophora glabripennis	None
EOG091203MR	APLA015275	Agrilus planipennis	None
EOG091203MR	TC031466	Tribolium castaneum	None
EOG091203MR	LDEC016403	Leptinotarsa decemlineata	None
EOG091203MR	YQE_09267	Dendroctonus ponderosae	None
EOG091203MT	OTAU009699	Onthophagus taurus	Putative uncharacterized protein
EOG091203MT	AGLA000718	Anoplophora glabripennis	Putative uncharacterized protein
EOG091203MT	APLA015125	Agrilus planipennis	Putative uncharacterized protein
EOG091203MT	TC007398	Tribolium castaneum	Putative uncharacterized protein
EOG091203MT	LDEC008424	Leptinotarsa decemlineata	Putative uncharacterized protein
EOG091203MT	YQE_04974	Dendroctonus ponderosae	Putative uncharacterized protein
EOG091203MV	OTAU012263	Onthophagus taurus	Putative uncharacterized protein
EOG091203MV	AGLA017876	Anoplophora glabripennis	Putative uncharacterized protein
EOG091203MV	APLA006633	Agrilus planipennis	Putative uncharacterized protein
EOG091203MV	TC010336	Tribolium castaneum	Putative uncharacterized protein
EOG091203MV	LDEC007080	Leptinotarsa decemlineata	Putative uncharacterized protein
EOG091203MV	YQE_08374	Dendroctonus ponderosae	Putative uncharacterized protein
EOG091203MX	OTAU002561	Onthophagus taurus	Putative uncharacterized protein
EOG091203MX	AGLA011925	Anoplophora glabripennis	Putative uncharacterized protein
EOG091203MX	APLA014463	Agrilus planipennis	Putative uncharacterized protein
EOG091203MX	TC012894	Tribolium castaneum	Putative uncharacterized protein
EOG091203MX	LDEC001174	Leptinotarsa decemlineata	Putative uncharacterized protein
EOG091203MX	YQE_06089	Dendroctonus ponderosae	Putative uncharacterized protein
EOG091203MZ	OTAU007957	Onthophagus taurus	Putative uncharacterized protein
EOG091203MZ	AGLA011825	Anoplophora glabripennis	Putative uncharacterized protein
EOG091203MZ	APLA003444	Agrilus planipennis	Putative uncharacterized protein
EOG091203MZ	TC009874	Tribolium castaneum	Putative uncharacterized protein
EOG091203MZ	LDEC001295	Leptinotarsa decemlineata	Putative uncharacterized protein
EOG091203MZ	YQE_10611	Dendroctonus ponderosae	Putative uncharacterized protein
EOG091203N2	OTAU000904	Onthophagus taurus	Putative uncharacterized protein
EOG091203N2	AGLA000120	Anoplophora glabripennis	Putative uncharacterized protein
EOG091203N2	APLA007702	Agrilus planipennis	Putative uncharacterized protein
EOG091203N2	TC011468	Tribolium castaneum	Putative uncharacterized protein
EOG091203N2	LDEC003122	Leptinotarsa decemlineata	Putative uncharacterized protein
EOG091203N2	YQE_08146	Dendroctonus ponderosae	Putative uncharacterized protein
EOG091203N3	OTAU001518	Onthophagus taurus	Putative uncharacterized protein
EOG091203N3	AGLA008521	Anoplophora glabripennis	Putative uncharacterized protein
EOG091203N3	APLA004627	Agrilus planipennis	Putative uncharacterized protein
EOG091203N3	TC009487	Tribolium castaneum	Putative uncharacterized protein
EOG091203N3	LDEC017112	Leptinotarsa decemlineata	Putative uncharacterized protein
EOG091203N3	YQE_02343	Dendroctonus ponderosae	Putative uncharacterized protein
EOG091203N4	OTAU001366	Onthophagus taurus	Cathepsin K
EOG091203N4	AGLA000399	Anoplophora glabripennis	Cathepsin K
EOG091203N4	APLA009501	Agrilus planipennis	Cathepsin K
EOG091203N4	TC013582	Tribolium castaneum	Cathepsin K
EOG091203N4	LDEC004699	Leptinotarsa decemlineata	Cathepsin K
EOG091203N4	YQE_09230	Dendroctonus ponderosae	Cathepsin K
EOG091203N6	OTAU013293	Onthophagus taurus	Putative uncharacterized protein
EOG091203N6	AGLA002640	Anoplophora glabripennis	Putative uncharacterized protein
EOG091203N6	APLA002730	Agrilus planipennis	Putative uncharacterized protein
EOG091203N6	TC014639	Tribolium castaneum	Putative uncharacterized protein
EOG091203N6	LDEC017381	Leptinotarsa decemlineata	Putative uncharacterized protein

EOG091203N6	YQE_10348	Dendroctonus ponderosae	Putative uncharacterized protein
EOG091203N7	OTAU004979	Onthophagus taurus	Putative uncharacterized protein
EOG091203N7	AGLA000159	Anoplophora glabripennis	Putative uncharacterized protein
EOG091203N7	APLA001270	Agrilus planipennis	Putative uncharacterized protein
EOG091203N7	TC004825	Tribolium castaneum	Putative uncharacterized protein
EOG091203N7	LDEC018557	Leptinotarsa decemlineata	Putative uncharacterized protein
EOG091203N7	YQE_08481	Dendroctonus ponderosae	Putative uncharacterized protein
EOG091203N8	OTAU009042	Onthophagus taurus	Putative uncharacterized protein
EOG091203N8	AGLA021132	Anoplophora glabripennis	Putative uncharacterized protein
EOG091203N8	APLA012513	Agrilus planipennis	Putative uncharacterized protein
EOG091203N8	TC004889	Tribolium castaneum	Putative uncharacterized protein
EOG091203N8	LDEC010340	Leptinotarsa decemlineata	Putative uncharacterized protein
EOG091203N8	YQE_10423	Dendroctonus ponderosae	Putative uncharacterized protein
EOG091203N9	OTAU002761	Onthophagus taurus	Putative uncharacterized protein
EOG091203N9	AGLA000251	Anoplophora glabripennis	Putative uncharacterized protein
EOG091203N9	APLA006416	Agrilus planipennis	Putative uncharacterized protein
EOG091203N9	TC001742	Tribolium castaneum	Putative uncharacterized protein
EOG091203N9	LDEC012032	Leptinotarsa decemlineata	Putative uncharacterized protein
EOG091203N9	YQE_06737	Dendroctonus ponderosae	Putative uncharacterized protein
EOG091203NB	OTAU003559	Onthophagus taurus	Putative uncharacterized protein
EOG091203NB	AGLA017227	Anoplophora glabripennis	Putative uncharacterized protein
EOG091203NB	APLA002641	Agrilus planipennis	Putative uncharacterized protein
EOG091203NB	TC011843	Tribolium castaneum	Putative uncharacterized protein
EOG091203NB	LDEC003858	Leptinotarsa decemlineata	Putative uncharacterized protein
EOG091203NB	YQE_04312	Dendroctonus ponderosae	Putative uncharacterized protein
EOG091203NF	OTAU004961	Onthophagus taurus	Putative uncharacterized protein
EOG091203NF	AGLA021366	Anoplophora glabripennis	Putative uncharacterized protein
EOG091203NF	APLA009413	Agrilus planipennis	Putative uncharacterized protein
EOG091203NF	TC000953	Tribolium castaneum	Putative uncharacterized protein
EOG091203NF	LDEC014018	Leptinotarsa decemlineata	Putative uncharacterized protein
EOG091203NF	YQE_11208	Dendroctonus ponderosae	Putative uncharacterized protein
EOG091203NG	OTAU007645	Onthophagus taurus	ATP synthase gamma chain
EOG091203NG	AGLA015620	Anoplophora glabripennis	ATP synthase gamma chain
EOG091203NG	APLA003430	Agrilus planipennis	ATP synthase gamma chain
EOG091203NG	TC009010	Tribolium castaneum	ATP synthase gamma chain
EOG091203NG	LDEC004838	Leptinotarsa decemlineata	ATP synthase gamma chain
EOG091203NG	YQE_03537	Dendroctonus ponderosae	ATP synthase gamma chain
EOG091203NH	OTAU012322	Onthophagus taurus	Putative uncharacterized protein
EOG091203NH	AGLA010987	Anoplophora glabripennis	Putative uncharacterized protein
EOG091203NH	APLA008505	Agrilus planipennis	Putative uncharacterized protein
EOG091203NH	TC014453	Tribolium castaneum	Putative uncharacterized protein
EOG091203NH	LDEC021211	Leptinotarsa decemlineata	Putative uncharacterized protein
EOG091203NH	YQE_06123	Dendroctonus ponderosae	Putative uncharacterized protein
EOG091203NL	OTAU010375	Onthophagus taurus	Putative uncharacterized protein
EOG091203NL	AGLA009252	Anoplophora glabripennis	Putative uncharacterized protein
EOG091203NL	APLA006447	Agrilus planipennis	Putative uncharacterized protein
EOG091203NL	TC015502	Tribolium castaneum	Putative uncharacterized protein
EOG091203NL	LDEC002354	Leptinotarsa decemlineata	Putative uncharacterized protein
EOG091203NL	YQE_05929	Dendroctonus ponderosae	Putative uncharacterized protein
EOG091203NQ	OTAU002940	Onthophagus taurus	Putative uncharacterized protein
EOG091203NQ	AGLA002803	Anoplophora glabripennis	Putative uncharacterized protein
EOG091203NQ	APLA007139	Agrilus planipennis	Putative uncharacterized protein
EOG091203NQ	TC009660	Tribolium castaneum	Putative uncharacterized protein
EOG091203NQ	LDEC014722	Leptinotarsa decemlineata	Putative uncharacterized protein
EOG091203NQ	YQE_10543	Dendroctonus ponderosae	Putative uncharacterized protein
EOG091203NR	OTAU005611	Onthophagus taurus	Putative uncharacterized protein
EOG091203NR	AGLA006482	Anoplophora glabripennis	Putative uncharacterized protein
EOG091203NR	APLA014536	Agrilus planipennis	Putative uncharacterized protein
EOG091203NR	TC009328	Tribolium castaneum	Putative uncharacterized protein
EOG091203NR	LDEC004416	Leptinotarsa decemlineata	Putative uncharacterized protein
EOG091203NR	YQE_04292	Dendroctonus ponderosae	Putative uncharacterized protein
EOG091203NS	OTAU011626	Onthophagus taurus	None
EOG091203NS	AGLA002435	Anoplophora glabripennis	None
EOG091203NS	APLA001968	Agrilus planipennis	None
EOG091203NS	TC031075	Tribolium castaneum	None
EOG091203NS	LDEC009629	Leptinotarsa decemlineata	None
EOG091203NS	YQE_08117	Dendroctonus ponderosae	None

EOG091203NT	OTAU008194	Onthophagus taurus	Putative uncharacterized protein
EOG091203NT	AGLA013830	Anoplophora glabripennis	Putative uncharacterized protein
EOG091203NT	APLA014721	Agrilus planipennis	Putative uncharacterized protein
EOG091203NT	TC015655	Tribolium castaneum	Putative uncharacterized protein
EOG091203NT	LDEC001811	Leptinotarsa decemlineata	Putative uncharacterized protein
EOG091203NT	YQE_08404	Dendroctonus ponderosae	Putative uncharacterized protein
EOG091203NU	OTAU001351	Onthophagus taurus	metal ion binding
EOG091203NU	AGLA000231	Anoplophora glabripennis	metal ion binding
EOG091203NU	APLA005604	Agrilus planipennis	metal ion binding
EOG091203NU	TC031549	Tribolium castaneum	metal ion binding
EOG091203NU	LDEC001025	Leptinotarsa decemlineata	metal ion binding
EOG091203NU	YQE_05974	Dendroctonus ponderosae	metal ion binding
EOG091203NZ	OTAU000703	Onthophagus taurus	Putative uncharacterized protein
EOG091203NZ	AGLA018757	Anoplophora glabripennis	Putative uncharacterized protein
EOG091203NZ	APLA014680	Agrilus planipennis	Putative uncharacterized protein
EOG091203NZ	TC011614	Tribolium castaneum	Putative uncharacterized protein
EOG091203NZ	LDEC000472	Leptinotarsa decemlineata	Putative uncharacterized protein
EOG091203NZ	YQE_05787	Dendroctonus ponderosae	Putative uncharacterized protein
EOG091203O1	OTAU004294	Onthophagus taurus	Putative uncharacterized protein
EOG091203O1	AGLA000386	Anoplophora glabripennis	Putative uncharacterized protein
EOG091203O1	APLA006785	Agrilus planipennis	Putative uncharacterized protein
EOG091203O1	TC010832	Tribolium castaneum	Putative uncharacterized protein
EOG091203O1	LDEC003534	Leptinotarsa decemlineata	Putative uncharacterized protein
EOG091203O1	YQE_09109	Dendroctonus ponderosae	Putative uncharacterized protein
EOG091203O2	OTAU004971	Onthophagus taurus	None
EOG091203O2	AGLA000097	Anoplophora glabripennis	None
EOG091203O2	APLA012555	Agrilus planipennis	None
EOG091203O2	TC035020	Tribolium castaneum	None
EOG091203O2	LDEC006611	Leptinotarsa decemlineata	None
EOG091203O2	YQE_10727	Dendroctonus ponderosae	None
EOG091203O6	OTAU002223	Onthophagus taurus	Putative uncharacterized protein
EOG091203O6	AGLA003265	Anoplophora glabripennis	Putative uncharacterized protein
EOG091203O6	APLA002369	Agrilus planipennis	Putative uncharacterized protein
EOG091203O6	TC011605	Tribolium castaneum	Putative uncharacterized protein
EOG091203O6	LDEC016898	Leptinotarsa decemlineata	Putative uncharacterized protein
EOG091203O6	YQE_12975	Dendroctonus ponderosae	Putative uncharacterized protein
EOG091203O9	OTAU011385	Onthophagus taurus	Putative uncharacterized protein
EOG091203O9	AGLA007019	Anoplophora glabripennis	Putative uncharacterized protein
EOG091203O9	APLA007549	Agrilus planipennis	Putative uncharacterized protein
EOG091203O9	TC014754	Tribolium castaneum	Putative uncharacterized protein
EOG091203O9	LDEC018790	Leptinotarsa decemlineata	Putative uncharacterized protein
EOG091203O9	YQE_06124	Dendroctonus ponderosae	Putative uncharacterized protein
EOG091203OA	OTAU001234	Onthophagus taurus	Putative uncharacterized protein
EOG091203OA	AGLA000314	Anoplophora glabripennis	Putative uncharacterized protein
EOG091203OA	APLA000615	Agrilus planipennis	Putative uncharacterized protein
EOG091203OA	TC001753	Tribolium castaneum	Putative uncharacterized protein
EOG091203OA	LDEC020932	Leptinotarsa decemlineata	Putative uncharacterized protein
EOG091203OA	YQE_09065	Dendroctonus ponderosae	Putative uncharacterized protein
EOG091203OC	OTAU008756	Onthophagus taurus	Putative uncharacterized protein
EOG091203OC	AGLA008569	Anoplophora glabripennis	Putative uncharacterized protein
EOG091203OC	APLA009109	Agrilus planipennis	Putative uncharacterized protein
EOG091203OC	TC014951	Tribolium castaneum	Putative uncharacterized protein
EOG091203OC	LDEC013749	Leptinotarsa decemlineata	Putative uncharacterized protein
EOG091203OC	YQE_10801	Dendroctonus ponderosae	Putative uncharacterized protein
EOG091203OD	OTAU006476	Onthophagus taurus	metal ion binding
EOG091203OD	AGLA003572	Anoplophora glabripennis	metal ion binding
EOG091203OD	APLA013406	Agrilus planipennis	metal ion binding
EOG091203OD	TC032328	Tribolium castaneum	metal ion binding
EOG091203OD	LDEC000445	Leptinotarsa decemlineata	metal ion binding
EOG091203OD	YQE_09794	Dendroctonus ponderosae	metal ion binding
EOG091203OG	OTAU007012	Onthophagus taurus	Putative uncharacterized protein
EOG091203OG	AGLA009737	Anoplophora glabripennis	Putative uncharacterized protein
EOG091203OG	APLA001201	Agrilus planipennis	Putative uncharacterized protein
EOG091203OG	TC006211	Tribolium castaneum	Putative uncharacterized protein
EOG091203OG	LDEC009683	Leptinotarsa decemlineata	Putative uncharacterized protein
EOG091203OG	YQE_07469	Dendroctonus ponderosae	Putative uncharacterized protein
EOG091203OK	OTAU002554	Onthophagus taurus	Putative uncharacterized protein

EOG091203OK	AGLA011930	Anoplophora glabripennis	Putative uncharacterized protein
EOG091203OK	APLA014467	Agrilus planipennis	Putative uncharacterized protein
EOG091203OK	TC016314	Tribolium castaneum	Putative uncharacterized protein
EOG091203OK	LDEC007248	Leptinotarsa decemlineata	Putative uncharacterized protein
EOG091203OK	YQE_06075	Dendroctonus ponderosae	Putative uncharacterized protein
EOG091203OL	OTAU005213	Onthophagus taurus	"Hypoxia inducible factor 1, alpha subunit "
EOG091203OL	AGLA002861	Anoplophora glabripennis	"Hypoxia inducible factor 1, alpha subunit "
EOG091203OL	APLA012168	Agrilus planipennis	"Hypoxia inducible factor 1, alpha subunit "
EOG091203OL	TC013241	Tribolium castaneum	"Hypoxia inducible factor 1, alpha subunit "
EOG091203OL	LDEC012449	Leptinotarsa decemlineata	"Hypoxia inducible factor 1, alpha subunit "
EOG091203OL	YQE_05314	Dendroctonus ponderosae	"Hypoxia inducible factor 1, alpha subunit "
EOG091203OO	OTAU000435	Onthophagus taurus	Putative uncharacterized protein
EOG091203OO	AGLA011668	Anoplophora glabripennis	Putative uncharacterized protein
EOG091203OO	APLA010525	Agrilus planipennis	Putative uncharacterized protein
EOG091203OO	TC011978	Tribolium castaneum	Putative uncharacterized protein
EOG091203OO	LDEC007748	Leptinotarsa decemlineata	Putative uncharacterized protein
EOG091203OO	YQE_12037	Dendroctonus ponderosae	Putative uncharacterized protein
EOG091203OS	OTAU002652	Onthophagus taurus	Putative uncharacterized protein
EOG091203OS	AGLA021318	Anoplophora glabripennis	Putative uncharacterized protein
EOG091203OS	APLA007720	Agrilus planipennis	Putative uncharacterized protein
EOG091203OS	TC014015	Tribolium castaneum	Putative uncharacterized protein
EOG091203OS	LDEC001151	Leptinotarsa decemlineata	Putative uncharacterized protein
EOG091203OS	YQE_04001	Dendroctonus ponderosae	Putative uncharacterized protein
EOG091203OV	OTAU005982	Onthophagus taurus	Putative uncharacterized protein
EOG091203OV	AGLA004285	Anoplophora glabripennis	Putative uncharacterized protein
EOG091203OV	APLA002046	Agrilus planipennis	Putative uncharacterized protein
EOG091203OV	TC011527	Tribolium castaneum	Putative uncharacterized protein
EOG091203OV	LDEC010782	Leptinotarsa decemlineata	Putative uncharacterized protein
EOG091203OV	YQE_03541	Dendroctonus ponderosae	Putative uncharacterized protein
EOG091203OW	OTAU007108	Onthophagus taurus	Putative uncharacterized protein
EOG091203OW	AGLA010430	Anoplophora glabripennis	Putative uncharacterized protein
EOG091203OW	APLA000666	Agrilus planipennis	Putative uncharacterized protein
EOG091203OW	TC014957	Tribolium castaneum	Putative uncharacterized protein
EOG091203OW	LDEC010458	Leptinotarsa decemlineata	Putative uncharacterized protein
EOG091203OW	YQE_10863	Dendroctonus ponderosae	Putative uncharacterized protein
EOG091203OY	OTAU013435	Onthophagus taurus	Putative uncharacterized protein
EOG091203OY	AGLA000139	Anoplophora glabripennis	Putative uncharacterized protein
EOG091203OY	APLA012675	Agrilus planipennis	Putative uncharacterized protein
EOG091203OY	TC001377	Tribolium castaneum	Putative uncharacterized protein
EOG091203OY	LDEC016696	Leptinotarsa decemlineata	Putative uncharacterized protein
EOG091203OY	YQE_01608	Dendroctonus ponderosae	Putative uncharacterized protein
EOG091203OZ	OTAU002211	Onthophagus taurus	Putative uncharacterized protein
EOG091203OZ	AGLA021566	Anoplophora glabripennis	Putative uncharacterized protein
EOG091203OZ	APLA002366	Agrilus planipennis	Putative uncharacterized protein
EOG091203OZ	TC011606	Tribolium castaneum	Putative uncharacterized protein
EOG091203OZ	LDEC011355	Leptinotarsa decemlineata	Putative uncharacterized protein
EOG091203OZ	YQE_07316	Dendroctonus ponderosae	Putative uncharacterized protein
EOG091203P3	OTAU005827	Onthophagus taurus	Putative uncharacterized protein
EOG091203P3	AGLA003396	Anoplophora glabripennis	Putative uncharacterized protein
EOG091203P3	APLA001498	Agrilus planipennis	Putative uncharacterized protein
EOG091203P3	TC002915	Tribolium castaneum	Putative uncharacterized protein
EOG091203P3	LDEC010049	Leptinotarsa decemlineata	Putative uncharacterized protein
EOG091203P3	YQE_12273	Dendroctonus ponderosae	Putative uncharacterized protein
EOG091203P4	OTAU007116	Onthophagus taurus	Putative uncharacterized protein
EOG091203P4	AGLA005893	Anoplophora glabripennis	Putative uncharacterized protein
EOG091203P4	APLA009449	Agrilus planipennis	Putative uncharacterized protein
EOG091203P4	TC006041	Tribolium castaneum	Putative uncharacterized protein
EOG091203P4	LDEC008291	Leptinotarsa decemlineata	Putative uncharacterized protein
EOG091203P4	YQE_11058	Dendroctonus ponderosae	Putative uncharacterized protein
EOG091203P6	OTAU012472	Onthophagus taurus	Putative uncharacterized protein
EOG091203P6	AGLA000491	Anoplophora glabripennis	Putative uncharacterized protein
EOG091203P6	APLA002302	Agrilus planipennis	Putative uncharacterized protein
EOG091203P6	TC008478	Tribolium castaneum	Putative uncharacterized protein
EOG091203P6	LDEC002404	Leptinotarsa decemlineata	Putative uncharacterized protein
EOG091203P6	YQE_11448	Dendroctonus ponderosae	Putative uncharacterized protein
EOG091203PA	OTAU006351	Onthophagus taurus	Putative uncharacterized protein
EOG091203PA	AGLA012501	Anoplophora glabripennis	Putative uncharacterized protein

EOG091203PA	APLA014017	Agrilus planipennis	Putative uncharacterized protein
EOG091203PA	TC010209	Tribolium castaneum	Putative uncharacterized protein
EOG091203PA	LDEC010016	Leptinotarsa decemlineata	Putative uncharacterized protein
EOG091203PA	YQE_03521	Dendroctonus ponderosae	Putative uncharacterized protein
EOG091203PF	OTAU001566	Onthophagus taurus	Putative uncharacterized protein
EOG091203PF	AGLA013679	Anoplophora glabripennis	Putative uncharacterized protein
EOG091203PF	APLA004453	Agrilus planipennis	Putative uncharacterized protein
EOG091203PF	TC030614	Tribolium castaneum	Putative uncharacterized protein
EOG091203PF	LDEC005862	Leptinotarsa decemlineata	Putative uncharacterized protein
EOG091203PF	YQE_12052	Dendroctonus ponderosae	Putative uncharacterized protein
EOG091203PG	OTAU006722	Onthophagus taurus	Held out wings
EOG091203PG	AGLA000626	Anoplophora glabripennis	Held out wings
EOG091203PG	APLA009878	Agrilus planipennis	Held out wings
EOG091203PG	TC000827	Tribolium castaneum	Held out wings
EOG091203PG	LDEC003159	Leptinotarsa decemlineata	Held out wings
EOG091203PG	YQE_07739	Dendroctonus ponderosae	Held out wings
EOG091203PH	OTAU007331	Onthophagus taurus	Putative uncharacterized protein
EOG091203PH	AGLA005061	Anoplophora glabripennis	Putative uncharacterized protein
EOG091203PH	APLA005334	Agrilus planipennis	Putative uncharacterized protein
EOG091203PH	TC000767	Tribolium castaneum	Putative uncharacterized protein
EOG091203PH	LDEC006657	Leptinotarsa decemlineata	Putative uncharacterized protein
EOG091203PH	YQE_09493	Dendroctonus ponderosae	Putative uncharacterized protein
EOG091203PK	OTAU013835	Onthophagus taurus	Putative uncharacterized protein
EOG091203PK	AGLA013677	Anoplophora glabripennis	Putative uncharacterized protein
EOG091203PK	APLA012300	Agrilus planipennis	Putative uncharacterized protein
EOG091203PK	TC006665	Tribolium castaneum	Putative uncharacterized protein
EOG091203PK	LDEC016560	Leptinotarsa decemlineata	Putative uncharacterized protein
EOG091203PK	YQE_05650	Dendroctonus ponderosae	Putative uncharacterized protein
EOG091203PL	OTAU000774	Onthophagus taurus	Putative uncharacterized protein
EOG091203PL	AGLA008598	Anoplophora glabripennis	Putative uncharacterized protein
EOG091203PL	APLA008566	Agrilus planipennis	Putative uncharacterized protein
EOG091203PL	TC010260	Tribolium castaneum	Putative uncharacterized protein
EOG091203PL	LDEC017613	Leptinotarsa decemlineata	Putative uncharacterized protein
EOG091203PL	YQE_01790	Dendroctonus ponderosae	Putative uncharacterized protein
EOG091203PM	OTAU007449	Onthophagus taurus	Putative uncharacterized protein
EOG091203PM	AGLA006345	Anoplophora glabripennis	Putative uncharacterized protein
EOG091203PM	APLA011959	Agrilus planipennis	Putative uncharacterized protein
EOG091203PM	TC006109	Tribolium castaneum	Putative uncharacterized protein
EOG091203PM	LDEC007862	Leptinotarsa decemlineata	Putative uncharacterized protein
EOG091203PM	YQE_04664	Dendroctonus ponderosae	Putative uncharacterized protein
EOG091203PN	OTAU000399	Onthophagus taurus	Putative uncharacterized protein
EOG091203PN	AGLA006635	Anoplophora glabripennis	Putative uncharacterized protein
EOG091203PN	APLA011010	Agrilus planipennis	Putative uncharacterized protein
EOG091203PN	TC002855	Tribolium castaneum	Putative uncharacterized protein
EOG091203PN	LDEC012484	Leptinotarsa decemlineata	Putative uncharacterized protein
EOG091203PN	YQE_07895	Dendroctonus ponderosae	Putative uncharacterized protein
EOG091203PP	OTAU008755	Onthophagus taurus	None
EOG091203PP	AGLA019573	Anoplophora glabripennis	None
EOG091203PP	APLA002566	Agrilus planipennis	None
EOG091203PP	TC033954	Tribolium castaneum	None
EOG091203PP	LDEC001868	Leptinotarsa decemlineata	None
EOG091203PP	YQE_08390	Dendroctonus ponderosae	None
EOG091203PU	OTAU014135	Onthophagus taurus	Glass bottom boat
EOG091203PU	AGLA011397	Anoplophora glabripennis	Glass bottom boat
EOG091203PU	APLA011576	Agrilus planipennis	Glass bottom boat
EOG091203PU	TC014017	Tribolium castaneum	Glass bottom boat
EOG091203PU	LDEC006498	Leptinotarsa decemlineata	Glass bottom boat
EOG091203PU	YQE_07218	Dendroctonus ponderosae	Glass bottom boat
EOG091203PZ	OTAU000773	Onthophagus taurus	Putative uncharacterized protein
EOG091203PZ	AGLA003417	Anoplophora glabripennis	Putative uncharacterized protein
EOG091203PZ	APLA000766	Agrilus planipennis	Putative uncharacterized protein
EOG091203PZ	TC010317	Tribolium castaneum	Putative uncharacterized protein
EOG091203PZ	LDEC020454	Leptinotarsa decemlineata	Putative uncharacterized protein
EOG091203PZ	YQE_11149	Dendroctonus ponderosae	Putative uncharacterized protein
EOG091203Q0	OTAU006632	Onthophagus taurus	Putative uncharacterized protein
EOG091203Q0	AGLA006784	Anoplophora glabripennis	Putative uncharacterized protein
EOG091203Q0	APLA001476	Agrilus planipennis	Putative uncharacterized protein

EOG091203Q0	TC003966	Tribolium castaneum	Putative uncharacterized protein
EOG091203Q0	LDEC011541	Leptinotarsa decemlineata	Putative uncharacterized protein
EOG091203Q0	YQE_11159	Dendroctonus ponderosae	Putative uncharacterized protein
EOG091203Q3	OTAU001135	Onthophagus taurus	Mannose-6-phosphate isomerase
EOG091203Q3	AGLA009028	Anoplophora glabripennis	Mannose-6-phosphate isomerase
EOG091203Q3	APLA004325	Agrilus planipennis	Mannose-6-phosphate isomerase
EOG091203Q3	TC007395	Tribolium castaneum	Mannose-6-phosphate isomerase
EOG091203Q3	LDEC010280	Leptinotarsa decemlineata	Mannose-6-phosphate isomerase
EOG091203Q3	YQE_08833	Dendroctonus ponderosae	Mannose-6-phosphate isomerase
EOG091203Q9	OTAU006862	Onthophagus taurus	Putative uncharacterized protein
EOG091203Q9	AGLA008461	Anoplophora glabripennis	Putative uncharacterized protein
EOG091203Q9	APLA008279	Agrilus planipennis	Putative uncharacterized protein
EOG091203Q9	TC013516	Tribolium castaneum	Putative uncharacterized protein
EOG091203Q9	LDEC008329	Leptinotarsa decemlineata	Putative uncharacterized protein
EOG091203Q9	YQE_06275	Dendroctonus ponderosae	Putative uncharacterized protein
EOG091203QE	OTAU001723	Onthophagus taurus	Putative uncharacterized protein
EOG091203QE	AGLA002490	Anoplophora glabripennis	Putative uncharacterized protein
EOG091203QE	APLA005466	Agrilus planipennis	Putative uncharacterized protein
EOG091203QE	TC005359	Tribolium castaneum	Putative uncharacterized protein
EOG091203QE	LDEC006336	Leptinotarsa decemlineata	Putative uncharacterized protein
EOG091203QE	YQE_07513	Dendroctonus ponderosae	Putative uncharacterized protein
EOG091203QG	OTAU002669	Onthophagus taurus	Putative uncharacterized protein
EOG091203QG	AGLA009661	Anoplophora glabripennis	Putative uncharacterized protein
EOG091203QG	APLA008485	Agrilus planipennis	Putative uncharacterized protein
EOG091203QG	TC013727	Tribolium castaneum	Putative uncharacterized protein
EOG091203QG	LDEC008701	Leptinotarsa decemlineata	Putative uncharacterized protein
EOG091203QG	YQE_06509	Dendroctonus ponderosae	Putative uncharacterized protein
EOG091203QH	OTAU003662	Onthophagus taurus	Putative uncharacterized protein
EOG091203QH	AGLA009512	Anoplophora glabripennis	Putative uncharacterized protein
EOG091203QH	APLA008319	Agrilus planipennis	Putative uncharacterized protein
EOG091203QH	TC007558	Tribolium castaneum	Putative uncharacterized protein
EOG091203QH	LDEC000036	Leptinotarsa decemlineata	Putative uncharacterized protein
EOG091203QH	YQE_12264	Dendroctonus ponderosae	Putative uncharacterized protein
EOG091203QJ	OTAU003013	Onthophagus taurus	None
EOG091203QJ	AGLA008911	Anoplophora glabripennis	None
EOG091203QJ	APLA015208	Agrilus planipennis	None
EOG091203QJ	TC032404	Tribolium castaneum	None
EOG091203QJ	LDEC007076	Leptinotarsa decemlineata	None
EOG091203QJ	YQE_10256	Dendroctonus ponderosae	None
EOG091203QK	OTAU013029	Onthophagus taurus	binding
EOG091203QK	AGLA016038	Anoplophora glabripennis	binding
EOG091203QK	APLA000595	Agrilus planipennis	binding
EOG091203QK	TC033460	Tribolium castaneum	binding
EOG091203QK	LDEC008267	Leptinotarsa decemlineata	binding
EOG091203QK	YQE_11880	Dendroctonus ponderosae	binding
EOG091203QL	OTAU008947	Onthophagus taurus	Putative uncharacterized protein
EOG091203QL	AGLA006645	Anoplophora glabripennis	Putative uncharacterized protein
EOG091203QL	APLA009146	Agrilus planipennis	Putative uncharacterized protein
EOG091203QL	TC002404	Tribolium castaneum	Putative uncharacterized protein
EOG091203QL	LDEC004971	Leptinotarsa decemlineata	Putative uncharacterized protein
EOG091203QL	YQE_04554	Dendroctonus ponderosae	Putative uncharacterized protein
EOG091203QM	OTAU009763	Onthophagus taurus	Putative uncharacterized protein
EOG091203QM	AGLA005524	Anoplophora glabripennis	Putative uncharacterized protein
EOG091203QM	APLA011775	Agrilus planipennis	Putative uncharacterized protein
EOG091203QM	TC001918	Tribolium castaneum	Putative uncharacterized protein
EOG091203QM	LDEC011151	Leptinotarsa decemlineata	Putative uncharacterized protein
EOG091203QM	YQE_08159	Dendroctonus ponderosae	Putative uncharacterized protein
EOG091203QN	OTAU012463	Onthophagus taurus	None
EOG091203QN	AGLA011351	Anoplophora glabripennis	None
EOG091203QN	APLA014499	Agrilus planipennis	None
EOG091203QN	TC034362	Tribolium castaneum	None
EOG091203QN	LDEC019231	Leptinotarsa decemlineata	None
EOG091203QN	YQE_11596	Dendroctonus ponderosae	None
EOG091203QO	OTAU002305	Onthophagus taurus	Putative uncharacterized protein
EOG091203QO	AGLA009465	Anoplophora glabripennis	Putative uncharacterized protein
EOG091203QO	APLA005825	Agrilus planipennis	Putative uncharacterized protein
EOG091203QO	TC007438	Tribolium castaneum	Putative uncharacterized protein

EOG091203QO	LDEC002230	Leptinotarsa decemlineata	Putative uncharacterized protein
EOG091203QO	YQE_10900	Dendroctonus ponderosae	Putative uncharacterized protein
EOG091203QR	OTAU005230	Onthophagus taurus	Putative uncharacterized protein
EOG091203QR	AGLA001690	Anoplophora glabripennis	Putative uncharacterized protein
EOG091203QR	APLA010857	Agrilus planipennis	Putative uncharacterized protein
EOG091203QR	TC030545	Tribolium castaneum	Putative uncharacterized protein
EOG091203QR	LDEC006181	Leptinotarsa decemlineata	Putative uncharacterized protein
EOG091203QR	YQE_11299	Dendroctonus ponderosae	Putative uncharacterized protein
EOG091203QU	OTAU002029	Onthophagus taurus	None
EOG091203QU	AGLA004405	Anoplophora glabripennis	None
EOG091203QU	APLA013339	Agrilus planipennis	None
EOG091203QU	TC034347	Tribolium castaneum	None
EOG091203QU	LDEC005110	Leptinotarsa decemlineata	None
EOG091203QU	YQE_07964	Dendroctonus ponderosae	None
EOG091203QW	OTAU002279	Onthophagus taurus	None
EOG091203QW	AGLA015609	Anoplophora glabripennis	None
EOG091203QW	APLA007196	Agrilus planipennis	None
EOG091203QW	TC032761	Tribolium castaneum	None
EOG091203QW	LDEC008784	Leptinotarsa decemlineata	None
EOG091203QW	YQE_00142	Dendroctonus ponderosae	None
EOG091203QY	OTAU009500	Onthophagus taurus	Putative uncharacterized protein
EOG091203QY	AGLA012461	Anoplophora glabripennis	Putative uncharacterized protein
EOG091203QY	APLA009439	Agrilus planipennis	Putative uncharacterized protein
EOG091203QY	TC015094	Tribolium castaneum	Putative uncharacterized protein
EOG091203QY	LDEC003192	Leptinotarsa decemlineata	Putative uncharacterized protein
EOG091203QY	YQE_09384	Dendroctonus ponderosae	Putative uncharacterized protein
EOG091203R0	OTAU009164	Onthophagus taurus	Hormone receptor 51
EOG091203R0	AGLA009198	Anoplophora glabripennis	Hormone receptor 51
EOG091203R0	APLA008839	Agrilus planipennis	Hormone receptor 51
EOG091203R0	TC009378	Tribolium castaneum	Hormone receptor 51
EOG091203R0	LDEC021751	Leptinotarsa decemlineata	Hormone receptor 51
EOG091203R0	YQE_03137	Dendroctonus ponderosae	Hormone receptor 51
EOG091203R3	OTAU002602	Onthophagus taurus	Escargot/snail protein homolog
EOG091203R3	AGLA014675	Anoplophora glabripennis	Escargot/snail protein homolog
EOG091203R3	APLA002676	Agrilus planipennis	Escargot/snail protein homolog
EOG091203R3	TC014474	Tribolium castaneum	Escargot/snail protein homolog
EOG091203R3	LDEC001157	Leptinotarsa decemlineata	Escargot/snail protein homolog
EOG091203R3	YQE_07640	Dendroctonus ponderosae	Escargot/snail protein homolog
EOG091203R4	OTAU001631	Onthophagus taurus	Defective in cullin neddylation protein
EOG091203R4	AGLA007267	Anoplophora glabripennis	Defective in cullin neddylation protein
EOG091203R4	APLA006275	Agrilus planipennis	Defective in cullin neddylation protein
EOG091203R4	TC031278	Tribolium castaneum	Defective in cullin neddylation protein
EOG091203R4	LDEC019370	Leptinotarsa decemlineata	Defective in cullin neddylation protein
EOG091203R4	YQE_12670	Dendroctonus ponderosae	Defective in cullin neddylation protein
EOG091203R7	OTAU004203	Onthophagus taurus	Putative uncharacterized protein
EOG091203R7	AGLA002243	Anoplophora glabripennis	Putative uncharacterized protein
EOG091203R7	APLA012043	Agrilus planipennis	Putative uncharacterized protein
EOG091203R7	TC011352	Tribolium castaneum	Putative uncharacterized protein
EOG091203R7	LDEC020420	Leptinotarsa decemlineata	Putative uncharacterized protein
EOG091203R7	YQE_04006	Dendroctonus ponderosae	Putative uncharacterized protein
EOG091203R9	OTAU008813	Onthophagus taurus	pre-mRNA intronic binding
EOG091203R9	AGLA002770	Anoplophora glabripennis	pre-mRNA intronic binding
EOG091203R9	APLA008788	Agrilus planipennis	pre-mRNA intronic binding
EOG091203R9	TC033519	Tribolium castaneum	pre-mRNA intronic binding
EOG091203R9	LDEC016622	Leptinotarsa decemlineata	pre-mRNA intronic binding
EOG091203R9	YQE_12464	Dendroctonus ponderosae	pre-mRNA intronic binding
EOG091203RB	OTAU001053	Onthophagus taurus	None
EOG091203RB	AGLA010900	Anoplophora glabripennis	None
EOG091203RB	APLA001982	Agrilus planipennis	None
EOG091203RB	TC031964	Tribolium castaneum	None
EOG091203RB	LDEC000810	Leptinotarsa decemlineata	None
EOG091203RB	YQE_02718	Dendroctonus ponderosae	None
EOG091203RK	OTAU010332	Onthophagus taurus	None
EOG091203RK	AGLA007161	Anoplophora glabripennis	None
EOG091203RK	APLA010270	Agrilus planipennis	None
EOG091203RK	TC033696	Tribolium castaneum	None
EOG091203RK	LDEC020003	Leptinotarsa decemlineata	None

EOG091203RK	YQE_02352	Dendroctonus ponderosae	None
EOG091203RM	OTAU012178	Onthophagus taurus	Putative uncharacterized protein
EOG091203RM	AGLA014412	Anoplophora glabripennis	Putative uncharacterized protein
EOG091203RM	APLA000082	Agrilus planipennis	Putative uncharacterized protein
EOG091203RM	TC008021	Tribolium castaneum	Putative uncharacterized protein
EOG091203RM	LDEC008306	Leptinotarsa decemlineata	Putative uncharacterized protein
EOG091203RM	YQE_02300	Dendroctonus ponderosae	Putative uncharacterized protein
EOG091203RP	OTAU005620	Onthophagus taurus	Putative uncharacterized protein
EOG091203RP	AGLA006814	Anoplophora glabripennis	Putative uncharacterized protein
EOG091203RP	APLA006119	Agrilus planipennis	Putative uncharacterized protein
EOG091203RP	TC009300	Tribolium castaneum	Putative uncharacterized protein
EOG091203RP	LDEC022464	Leptinotarsa decemlineata	Putative uncharacterized protein
EOG091203RP	YQE_10403	Dendroctonus ponderosae	Putative uncharacterized protein
EOG091203RS	OTAU006568	Onthophagus taurus	Putative uncharacterized protein
EOG091203RS	AGLA011692	Anoplophora glabripennis	Putative uncharacterized protein
EOG091203RS	APLA013269	Agrilus planipennis	Putative uncharacterized protein
EOG091203RS	TC002804	Tribolium castaneum	Putative uncharacterized protein
EOG091203RS	LDEC013203	Leptinotarsa decemlineata	Putative uncharacterized protein
EOG091203RS	YQE_07799	Dendroctonus ponderosae	Putative uncharacterized protein
EOG091203RT	OTAU002699	Onthophagus taurus	Ultraspiracle
EOG091203RT	AGLA008320	Anoplophora glabripennis	Ultraspiracle
EOG091203RT	APLA008297	Agrilus planipennis	Ultraspiracle
EOG091203RT	TC014027	Tribolium castaneum	Ultraspiracle
EOG091203RT	LDEC013998	Leptinotarsa decemlineata	Ultraspiracle
EOG091203RT	YQE_09004	Dendroctonus ponderosae	Ultraspiracle
EOG091203RU	OTAU003865	Onthophagus taurus	Putative uncharacterized protein
EOG091203RU	AGLA009696	Anoplophora glabripennis	Putative uncharacterized protein
EOG091203RU	APLA005717	Agrilus planipennis	Putative uncharacterized protein
EOG091203RU	TC009297	Tribolium castaneum	Putative uncharacterized protein
EOG091203RU	LDEC016306	Leptinotarsa decemlineata	Putative uncharacterized protein
EOG091203RU	YQE_02018	Dendroctonus ponderosae	Putative uncharacterized protein
EOG091203RW	OTAU015620	Onthophagus taurus	None
EOG091203RW	AGLA004233	Anoplophora glabripennis	None
EOG091203RW	APLA010956	Agrilus planipennis	None
EOG091203RW	TC032683	Tribolium castaneum	None
EOG091203RW	LDEC008582	Leptinotarsa decemlineata	None
EOG091203RW	YQE_02153	Dendroctonus ponderosae	None
EOG091203S0	OTAU010172	Onthophagus taurus	catalytic activity
EOG091203S0	AGLA018364	Anoplophora glabripennis	catalytic activity
EOG091203S0	APLA009621	Agrilus planipennis	catalytic activity
EOG091203S0	TC033830	Tribolium castaneum	catalytic activity
EOG091203S0	LDEC009355	Leptinotarsa decemlineata	catalytic activity
EOG091203S0	YQE_05366	Dendroctonus ponderosae	catalytic activity
EOG091203S1	OTAU002547	Onthophagus taurus	Putative uncharacterized protein
EOG091203S1	AGLA011044	Anoplophora glabripennis	Putative uncharacterized protein
EOG091203S1	APLA000479	Agrilus planipennis	Putative uncharacterized protein
EOG091203S1	TC006388	Tribolium castaneum	Putative uncharacterized protein
EOG091203S1	LDEC006973	Leptinotarsa decemlineata	Putative uncharacterized protein
EOG091203S1	YQE_05681	Dendroctonus ponderosae	Putative uncharacterized protein
EOG091203S5	OTAU012224	Onthophagus taurus	Putative uncharacterized protein
EOG091203S5	AGLA007785	Anoplophora glabripennis	Putative uncharacterized protein
EOG091203S5	APLA011131	Agrilus planipennis	Putative uncharacterized protein
EOG091203S5	TC001228	Tribolium castaneum	Putative uncharacterized protein
EOG091203S5	LDEC010157	Leptinotarsa decemlineata	Putative uncharacterized protein
EOG091203S5	YQE_08929	Dendroctonus ponderosae	Putative uncharacterized protein
EOG091203S6	OTAU000291	Onthophagus taurus	Putative uncharacterized protein
EOG091203S6	AGLA010982	Anoplophora glabripennis	Putative uncharacterized protein
EOG091203S6	APLA013282	Agrilus planipennis	Putative uncharacterized protein
EOG091203S6	TC013075	Tribolium castaneum	Putative uncharacterized protein
EOG091203S6	LDEC021212	Leptinotarsa decemlineata	Putative uncharacterized protein
EOG091203S6	YQE_08583	Dendroctonus ponderosae	Putative uncharacterized protein
EOG091203S7	OTAU000050	Onthophagus taurus	Putative uncharacterized protein
EOG091203S7	AGLA006401	Anoplophora glabripennis	Putative uncharacterized protein
EOG091203S7	APLA009825	Agrilus planipennis	Putative uncharacterized protein
EOG091203S7	TC010409	Tribolium castaneum	Putative uncharacterized protein
EOG091203S7	LDEC013326	Leptinotarsa decemlineata	Putative uncharacterized protein
EOG091203S7	YQE_02081	Dendroctonus ponderosae	Putative uncharacterized protein

EOG091203S8	OTAU006319	Onthophagus taurus	Putative uncharacterized protein
EOG091203S8	AGLA002248	Anoplophora glabripennis	Putative uncharacterized protein
EOG091203S8	APLA007426	Agrilus planipennis	Putative uncharacterized protein
EOG091203S8	TC004464	Tribolium castaneum	Putative uncharacterized protein
EOG091203S8	LDEC022668	Leptinotarsa decemlineata	Putative uncharacterized protein
EOG091203S8	YQE_02926	Dendroctonus ponderosae	Putative uncharacterized protein
EOG091203SC	OTAU006962	Onthophagus taurus	Mitochondrial GTPase 1
EOG091203SC	AGLA015046	Anoplophora glabripennis	Mitochondrial GTPase 1
EOG091203SC	APLA004613	Agrilus planipennis	Mitochondrial GTPase 1
EOG091203SC	TC009617	Tribolium castaneum	Mitochondrial GTPase 1
EOG091203SC	LDEC021270	Leptinotarsa decemlineata	Mitochondrial GTPase 1
EOG091203SC	YQE_08859	Dendroctonus ponderosae	Mitochondrial GTPase 1
EOG091203SE	OTAU017186	Onthophagus taurus	Putative uncharacterized protein
EOG091203SE	AGLA018974	Anoplophora glabripennis	Putative uncharacterized protein
EOG091203SE	APLA008962	Agrilus planipennis	Putative uncharacterized protein
EOG091203SE	TC000504	Tribolium castaneum	Putative uncharacterized protein
EOG091203SE	LDEC013928	Leptinotarsa decemlineata	Putative uncharacterized protein
EOG091203SE	YQE_02307	Dendroctonus ponderosae	Putative uncharacterized protein
EOG091203SH	OTAU005411	Onthophagus taurus	Putative uncharacterized protein
EOG091203SH	AGLA004582	Anoplophora glabripennis	Putative uncharacterized protein
EOG091203SH	APLA003541	Agrilus planipennis	Putative uncharacterized protein
EOG091203SH	TC004761	Tribolium castaneum	Putative uncharacterized protein
EOG091203SH	LDEC001528	Leptinotarsa decemlineata	Putative uncharacterized protein
EOG091203SH	YQE_09099	Dendroctonus ponderosae	Putative uncharacterized protein
EOG091203SI	OTAU000980	Onthophagus taurus	Obstractor D
EOG091203SI	AGLA002997	Anoplophora glabripennis	Obstractor D
EOG091203SI	APLA011526	Agrilus planipennis	Obstractor D
EOG091203SI	TC001350	Tribolium castaneum	Obstractor D
EOG091203SI	LDEC009655	Leptinotarsa decemlineata	Obstractor D
EOG091203SI	YQE_01682	Dendroctonus ponderosae	Obstractor D
EOG091203SL	OTAU004782	Onthophagus taurus	Fruitless
EOG091203SL	AGLA000007	Anoplophora glabripennis	Fruitless
EOG091203SL	APLA007848	Agrilus planipennis	Fruitless
EOG091203SL	TC000589	Tribolium castaneum	Fruitless
EOG091203SL	LDEC003177	Leptinotarsa decemlineata	Fruitless
EOG091203SL	YQE_07054	Dendroctonus ponderosae	Fruitless
EOG091203SM	OTAU005662	Onthophagus taurus	Putative uncharacterized protein
EOG091203SM	AGLA016913	Anoplophora glabripennis	Putative uncharacterized protein
EOG091203SM	APLA013592	Agrilus planipennis	Putative uncharacterized protein
EOG091203SM	TC003642	Tribolium castaneum	Putative uncharacterized protein
EOG091203SM	LDEC000222	Leptinotarsa decemlineata	Putative uncharacterized protein
EOG091203SM	YQE_11093	Dendroctonus ponderosae	Putative uncharacterized protein
EOG091203SN	OTAU002746	Onthophagus taurus	Putative uncharacterized protein
EOG091203SN	AGLA000233	Anoplophora glabripennis	Putative uncharacterized protein
EOG091203SN	APLA010533	Agrilus planipennis	Putative uncharacterized protein
EOG091203SN	TC005174	Tribolium castaneum	Putative uncharacterized protein
EOG091203SN	LDEC001026	Leptinotarsa decemlineata	Putative uncharacterized protein
EOG091203SN	YQE_05973	Dendroctonus ponderosae	Putative uncharacterized protein
EOG091203SO	OTAU015391	Onthophagus taurus	Putative uncharacterized protein
EOG091203SO	AGLA002235	Anoplophora glabripennis	Putative uncharacterized protein
EOG091203SO	APLA010389	Agrilus planipennis	Putative uncharacterized protein
EOG091203SO	TC014373	Tribolium castaneum	Putative uncharacterized protein
EOG091203SO	LDEC014797	Leptinotarsa decemlineata	Putative uncharacterized protein
EOG091203SO	YQE_11778	Dendroctonus ponderosae	Putative uncharacterized protein
EOG091203SQ	OTAU014658	Onthophagus taurus	None
EOG091203SQ	AGLA012713	Anoplophora glabripennis	None
EOG091203SQ	APLA012003	Agrilus planipennis	None
EOG091203SQ	TC031756	Tribolium castaneum	None
EOG091203SQ	LDEC019921	Leptinotarsa decemlineata	None
EOG091203SQ	YQE_10218	Dendroctonus ponderosae	None
EOG091203SS	OTAU009800	Onthophagus taurus	Putative uncharacterized protein
EOG091203SS	AGLA011877	Anoplophora glabripennis	Putative uncharacterized protein
EOG091203SS	APLA004424	Agrilus planipennis	Putative uncharacterized protein
EOG091203SS	TC005408	Tribolium castaneum	Putative uncharacterized protein
EOG091203SS	LDEC018739	Leptinotarsa decemlineata	Putative uncharacterized protein
EOG091203SS	YQE_05651	Dendroctonus ponderosae	Putative uncharacterized protein
EOG091203ST	OTAU011498	Onthophagus taurus	Putative uncharacterized protein

EOG091203ST	AGLA002492	Anoplophora glabripennis	Putative uncharacterized protein
EOG091203ST	APLA005465	Agrilus planipennis	Putative uncharacterized protein
EOG091203ST	TC005358	Tribolium castaneum	Putative uncharacterized protein
EOG091203ST	LDEC005861	Leptinotarsa decemlineata	Putative uncharacterized protein
EOG091203ST	YQE_07514	Dendroctonus ponderosae	Putative uncharacterized protein
EOG091203SU	OTAU006296	Onthophagus taurus	Putative uncharacterized protein
EOG091203SU	AGLA005271	Anoplophora glabripennis	Putative uncharacterized protein
EOG091203SU	APLA006563	Agrilus planipennis	Putative uncharacterized protein
EOG091203SU	TC007695	Tribolium castaneum	Putative uncharacterized protein
EOG091203SU	LDEC008481	Leptinotarsa decemlineata	Putative uncharacterized protein
EOG091203SU	YQE_04777	Dendroctonus ponderosae	Putative uncharacterized protein
EOG091203SV	OTAU004222	Onthophagus taurus	Putative uncharacterized protein
EOG091203SV	AGLA009266	Anoplophora glabripennis	Putative uncharacterized protein
EOG091203SV	APLA004860	Agrilus planipennis	Putative uncharacterized protein
EOG091203SV	TC013167	Tribolium castaneum	Putative uncharacterized protein
EOG091203SV	LDEC009048	Leptinotarsa decemlineata	Putative uncharacterized protein
EOG091203SV	YQE_08554	Dendroctonus ponderosae	Putative uncharacterized protein
EOG091203SW	OTAU008188	Onthophagus taurus	cytoskeletal adaptor activity
EOG091203SW	AGLA003916	Anoplophora glabripennis	cytoskeletal adaptor activity
EOG091203SW	APLA008752	Agrilus planipennis	cytoskeletal adaptor activity
EOG091203SW	TC033382	Tribolium castaneum	cytoskeletal adaptor activity
EOG091203SW	LDEC005626	Leptinotarsa decemlineata	cytoskeletal adaptor activity
EOG091203SW	YQE_06526	Dendroctonus ponderosae	cytoskeletal adaptor activity
EOG091203SY	OTAU010147	Onthophagus taurus	Putative uncharacterized protein
EOG091203SY	AGLA007183	Anoplophora glabripennis	Putative uncharacterized protein
EOG091203SY	APLA010200	Agrilus planipennis	Putative uncharacterized protein
EOG091203SY	TC009220	Tribolium castaneum	Putative uncharacterized protein
EOG091203SY	LDEC017703	Leptinotarsa decemlineata	Putative uncharacterized protein
EOG091203SY	YQE_11799	Dendroctonus ponderosae	Putative uncharacterized protein
EOG091203SZ	OTAU002943	Onthophagus taurus	Putative uncharacterized protein
EOG091203SZ	AGLA002802	Anoplophora glabripennis	Putative uncharacterized protein
EOG091203SZ	APLA007138	Agrilus planipennis	Putative uncharacterized protein
EOG091203SZ	TC009661	Tribolium castaneum	Putative uncharacterized protein
EOG091203SZ	LDEC014720	Leptinotarsa decemlineata	Putative uncharacterized protein
EOG091203SZ	YQE_10544	Dendroctonus ponderosae	Putative uncharacterized protein
EOG091203T0	OTAU007612	Onthophagus taurus	Putative uncharacterized protein
EOG091203T0	AGLA007515	Anoplophora glabripennis	Putative uncharacterized protein
EOG091203T0	APLA012294	Agrilus planipennis	Putative uncharacterized protein
EOG091203T0	TC003669	Tribolium castaneum	Putative uncharacterized protein
EOG091203T0	LDEC013851	Leptinotarsa decemlineata	Putative uncharacterized protein
EOG091203T0	YQE_10264	Dendroctonus ponderosae	Putative uncharacterized protein
EOG091203T3	OTAU009489	Onthophagus taurus	Putative uncharacterized protein
EOG091203T3	AGLA010327	Anoplophora glabripennis	Putative uncharacterized protein
EOG091203T3	APLA014350	Agrilus planipennis	Putative uncharacterized protein
EOG091203T3	TC015334	Tribolium castaneum	Putative uncharacterized protein
EOG091203T3	LDEC008536	Leptinotarsa decemlineata	Putative uncharacterized protein
EOG091203T3	YQE_11719	Dendroctonus ponderosae	Putative uncharacterized protein
EOG091203T6	OTAU015302	Onthophagus taurus	Putative uncharacterized protein
EOG091203T6	AGLA009010	Anoplophora glabripennis	Putative uncharacterized protein
EOG091203T6	APLA002408	Agrilus planipennis	Putative uncharacterized protein
EOG091203T6	TC013906	Tribolium castaneum	Putative uncharacterized protein
EOG091203T6	LDEC002141	Leptinotarsa decemlineata	Putative uncharacterized protein
EOG091203T6	YQE_10483	Dendroctonus ponderosae	Putative uncharacterized protein
EOG091203T8	OTAU001008	Onthophagus taurus	None
EOG091203T8	AGLA002442	Anoplophora glabripennis	None
EOG091203T8	APLA004311	Agrilus planipennis	None
EOG091203T8	TC031969	Tribolium castaneum	None
EOG091203T8	LDEC001763	Leptinotarsa decemlineata	None
EOG091203T8	YQE_01633	Dendroctonus ponderosae	None
EOG091203TC	OTAU009068	Onthophagus taurus	Protein Wnt
EOG091203TC	AGLA001771	Anoplophora glabripennis	Protein Wnt
EOG091203TC	APLA011586	Agrilus planipennis	Protein Wnt
EOG091203TC	TC014270	Tribolium castaneum	Protein Wnt
EOG091203TC	LDEC011407	Leptinotarsa decemlineata	Protein Wnt
EOG091203TC	YQE_04018	Dendroctonus ponderosae	Protein Wnt
EOG091203TE	OTAU005225	Onthophagus taurus	metal ion transmembrane transporter activity
EOG091203TE	AGLA004079	Anoplophora glabripennis	metal ion transmembrane transporter activity

EOG091203TE	APLA001822	Agrilus planipennis	metal ion transmembrane transporter activity
EOG091203TE	TC033699	Tribolium castaneum	metal ion transmembrane transporter activity
EOG091203TE	LDEC014678	Leptinotarsa decemlineata	metal ion transmembrane transporter activity
EOG091203TE	YQE_11511	Dendroctonus ponderosae	metal ion transmembrane transporter activity
EOG091203TG	OTAU013732	Onthophagus taurus	Putative uncharacterized protein
EOG091203TG	AGLA010705	Anoplophora glabripennis	Putative uncharacterized protein
EOG091203TG	APLA008614	Agrilus planipennis	Putative uncharacterized protein
EOG091203TG	TC013327	Tribolium castaneum	Putative uncharacterized protein
EOG091203TG	LDEC018191	Leptinotarsa decemlineata	Putative uncharacterized protein
EOG091203TG	YQE_06321	Dendroctonus ponderosae	Putative uncharacterized protein
EOG091203TQ	OTAU002755	Onthophagus taurus	Putative uncharacterized protein
EOG091203TQ	AGLA000243	Anoplophora glabripennis	Putative uncharacterized protein
EOG091203TQ	APLA006424	Agrilus planipennis	Putative uncharacterized protein
EOG091203TQ	TC010779	Tribolium castaneum	Putative uncharacterized protein
EOG091203TQ	LDEC020161	Leptinotarsa decemlineata	Putative uncharacterized protein
EOG091203TQ	YQE_01939	Dendroctonus ponderosae	Putative uncharacterized protein
EOG091203TS	OTAU001945	Onthophagus taurus	Putative uncharacterized protein
EOG091203TS	AGLA005933	Anoplophora glabripennis	Putative uncharacterized protein
EOG091203TS	APLA001190	Agrilus planipennis	Putative uncharacterized protein
EOG091203TS	TC005444	Tribolium castaneum	Putative uncharacterized protein
EOG091203TS	LDEC009300	Leptinotarsa decemlineata	Putative uncharacterized protein
EOG091203TS	YQE_05588	Dendroctonus ponderosae	Putative uncharacterized protein
EOG091203TT	OTAU005584	Onthophagus taurus	Putative uncharacterized protein
EOG091203TT	AGLA000755	Anoplophora glabripennis	Putative uncharacterized protein
EOG091203TT	APLA009260	Agrilus planipennis	Putative uncharacterized protein
EOG091203TT	TC008136	Tribolium castaneum	Putative uncharacterized protein
EOG091203TT	LDEC000993	Leptinotarsa decemlineata	Putative uncharacterized protein
EOG091203TT	YQE_03856	Dendroctonus ponderosae	Putative uncharacterized protein
EOG091203TV	OTAU009393	Onthophagus taurus	Putative uncharacterized protein
EOG091203TV	AGLA008669	Anoplophora glabripennis	Putative uncharacterized protein
EOG091203TV	APLA002529	Agrilus planipennis	Putative uncharacterized protein
EOG091203TV	TC012481	Tribolium castaneum	Putative uncharacterized protein
EOG091203TV	LDEC004137	Leptinotarsa decemlineata	Putative uncharacterized protein
EOG091203TV	YQE_01994	Dendroctonus ponderosae	Putative uncharacterized protein
EOG091203TW	OTAU004902	Onthophagus taurus	Putative uncharacterized protein
EOG091203TW	AGLA000123	Anoplophora glabripennis	Putative uncharacterized protein
EOG091203TW	APLA009895	Agrilus planipennis	Putative uncharacterized protein
EOG091203TW	TC004530	Tribolium castaneum	Putative uncharacterized protein
EOG091203TW	LDEC008042	Leptinotarsa decemlineata	Putative uncharacterized protein
EOG091203TW	YQE_08067	Dendroctonus ponderosae	Putative uncharacterized protein
EOG091203U0	OTAU001361	Onthophagus taurus	Putative uncharacterized protein
EOG091203U0	AGLA007613	Anoplophora glabripennis	Putative uncharacterized protein
EOG091203U0	APLA011482	Agrilus planipennis	Putative uncharacterized protein
EOG091203U0	TC003817	Tribolium castaneum	Putative uncharacterized protein
EOG091203U0	LDEC006813	Leptinotarsa decemlineata	Putative uncharacterized protein
EOG091203U0	YQE_02925	Dendroctonus ponderosae	Putative uncharacterized protein
EOG091203U1	OTAU006154	Onthophagus taurus	Putative uncharacterized protein
EOG091203U1	AGLA005060	Anoplophora glabripennis	Putative uncharacterized protein
EOG091203U1	APLA005338	Agrilus planipennis	Putative uncharacterized protein
EOG091203U1	TC000793	Tribolium castaneum	Putative uncharacterized protein
EOG091203U1	LDEC006658	Leptinotarsa decemlineata	Putative uncharacterized protein
EOG091203U1	YQE_07120	Dendroctonus ponderosae	Putative uncharacterized protein
EOG091203U2	OTAU010283	Onthophagus taurus	Pannier
EOG091203U2	AGLA003977	Anoplophora glabripennis	Pannier
EOG091203U2	APLA009834	Agrilus planipennis	Pannier
EOG091203U2	TC010407	Tribolium castaneum	Pannier
EOG091203U2	LDEC012262	Leptinotarsa decemlineata	Pannier
EOG091203U2	YQE_01723	Dendroctonus ponderosae	Pannier
EOG091203U3	OTAU005816	Onthophagus taurus	Putative uncharacterized protein
EOG091203U3	AGLA003134	Anoplophora glabripennis	Putative uncharacterized protein
EOG091203U3	APLA007673	Agrilus planipennis	Putative uncharacterized protein
EOG091203U3	TC012736	Tribolium castaneum	Putative uncharacterized protein
EOG091203U3	LDEC014564	Leptinotarsa decemlineata	Putative uncharacterized protein
EOG091203U3	YQE_10769	Dendroctonus ponderosae	Putative uncharacterized protein
EOG091203U4	OTAU001170	Onthophagus taurus	Putative uncharacterized protein
EOG091203U4	AGLA002439	Anoplophora glabripennis	Putative uncharacterized protein
EOG091203U4	APLA013927	Agrilus planipennis	Putative uncharacterized protein

EOG091203U4	TC011435	Tribolium castaneum	Putative uncharacterized protein
EOG091203U4	LDEC009635	Leptinotarsa decemlineata	Putative uncharacterized protein
EOG091203U4	YQE_08182	Dendroctonus ponderosae	Putative uncharacterized protein
EOG091203U6	OTAU007139	Onthophagus taurus	Putative uncharacterized protein
EOG091203U6	AGLA009016	Anoplophora glabripennis	Putative uncharacterized protein
EOG091203U6	APLA009224	Agrilus planipennis	Putative uncharacterized protein
EOG091203U6	TC007985	Tribolium castaneum	Putative uncharacterized protein
EOG091203U6	LDEC020631	Leptinotarsa decemlineata	Putative uncharacterized protein
EOG091203U6	YQE_11988	Dendroctonus ponderosae	Putative uncharacterized protein
EOG091203U9	OTAU015632	Onthophagus taurus	Putative uncharacterized protein
EOG091203U9	AGLA006357	Anoplophora glabripennis	Putative uncharacterized protein
EOG091203U9	APLA011367	Agrilus planipennis	Putative uncharacterized protein
EOG091203U9	TC006132	Tribolium castaneum	Putative uncharacterized protein
EOG091203U9	LDEC009767	Leptinotarsa decemlineata	Putative uncharacterized protein
EOG091203U9	YQE_01885	Dendroctonus ponderosae	Putative uncharacterized protein
EOG091203UB	OTAU013425	Onthophagus taurus	Putative uncharacterized protein
EOG091203UB	AGLA009246	Anoplophora glabripennis	Putative uncharacterized protein
EOG091203UB	APLA014876	Agrilus planipennis	Putative uncharacterized protein
EOG091203UB	TC006049	Tribolium castaneum	Putative uncharacterized protein
EOG091203UB	LDEC016754	Leptinotarsa decemlineata	Putative uncharacterized protein
EOG091203UB	YQE_11757	Dendroctonus ponderosae	Putative uncharacterized protein
EOG091203UE	OTAU012820	Onthophagus taurus	Putative uncharacterized protein
EOG091203UE	AGLA004182	Anoplophora glabripennis	Putative uncharacterized protein
EOG091203UE	APLA010029	Agrilus planipennis	Putative uncharacterized protein
EOG091203UE	TC015199	Tribolium castaneum	Putative uncharacterized protein
EOG091203UE	LDEC019901	Leptinotarsa decemlineata	Putative uncharacterized protein
EOG091203UE	YQE_05953	Dendroctonus ponderosae	Putative uncharacterized protein
EOG091203UG	OTAU001514	Onthophagus taurus	Putative uncharacterized protein
EOG091203UG	AGLA003822	Anoplophora glabripennis	Putative uncharacterized protein
EOG091203UG	APLA013239	Agrilus planipennis	Putative uncharacterized protein
EOG091203UG	TC009914	Tribolium castaneum	Putative uncharacterized protein
EOG091203UG	LDEC014308	Leptinotarsa decemlineata	Putative uncharacterized protein
EOG091203UG	YQE_07680	Dendroctonus ponderosae	Putative uncharacterized protein
EOG091203UK	OTAU011595	Onthophagus taurus	Putative uncharacterized protein
EOG091203UK	AGLA007156	Anoplophora glabripennis	Putative uncharacterized protein
EOG091203UK	APLA004959	Agrilus planipennis	Putative uncharacterized protein
EOG091203UK	TC000706	Tribolium castaneum	Putative uncharacterized protein
EOG091203UK	LDEC004502	Leptinotarsa decemlineata	Putative uncharacterized protein
EOG091203UK	YQE_06390	Dendroctonus ponderosae	Putative uncharacterized protein
EOG091203UM	OTAU002687	Onthophagus taurus	Putative uncharacterized protein
EOG091203UM	AGLA006391	Anoplophora glabripennis	Putative uncharacterized protein
EOG091203UM	APLA004817	Agrilus planipennis	Putative uncharacterized protein
EOG091203UM	TC013350	Tribolium castaneum	Putative uncharacterized protein
EOG091203UM	LDEC013329	Leptinotarsa decemlineata	Putative uncharacterized protein
EOG091203UM	YQE_02054	Dendroctonus ponderosae	Putative uncharacterized protein
EOG091203UN	OTAU001073	Onthophagus taurus	Putative uncharacterized protein
EOG091203UN	AGLA006104	Anoplophora glabripennis	Putative uncharacterized protein
EOG091203UN	APLA007701	Agrilus planipennis	Putative uncharacterized protein
EOG091203UN	TC011012	Tribolium castaneum	Putative uncharacterized protein
EOG091203UN	LDEC003125	Leptinotarsa decemlineata	Putative uncharacterized protein
EOG091203UN	YQE_06868	Dendroctonus ponderosae	Putative uncharacterized protein
EOG091203UO	OTAU005580	Onthophagus taurus	None
EOG091203UO	AGLA000747	Anoplophora glabripennis	None
EOG091203UO	APLA009253	Agrilus planipennis	None
EOG091203UO	TC032823	Tribolium castaneum	None
EOG091203UO	LDEC000984	Leptinotarsa decemlineata	None
EOG091203UO	YQE_00959	Dendroctonus ponderosae	None
EOG091203UR	OTAU008534	Onthophagus taurus	Putative uncharacterized protein
EOG091203UR	AGLA010912	Anoplophora glabripennis	Putative uncharacterized protein
EOG091203UR	APLA007419	Agrilus planipennis	Putative uncharacterized protein
EOG091203UR	TC009354	Tribolium castaneum	Putative uncharacterized protein
EOG091203UR	LDEC001244	Leptinotarsa decemlineata	Putative uncharacterized protein
EOG091203UR	YQE_10541	Dendroctonus ponderosae	Putative uncharacterized protein
EOG091203UU	OTAU008960	Onthophagus taurus	Aurora kinase A
EOG091203UU	AGLA006781	Anoplophora glabripennis	Aurora kinase A
EOG091203UU	APLA006288	Agrilus planipennis	Aurora kinase A
EOG091203UU	TC001817	Tribolium castaneum	Aurora kinase A

EOG091203UU	LDEC011543	Leptinotarsa decemlineata	Aurora kinase A
EOG091203UU	YQE_11155	Dendroctonus ponderosae	Aurora kinase A
EOG091203UW	OTAU003692	Onthophagus taurus	Malate dehydrogenase
EOG091203UW	AGLA000725	Anoplophora glabripennis	Malate dehydrogenase
EOG091203UW	APLA005378	Agrilus planipennis	Malate dehydrogenase
EOG091203UW	TC008177	Tribolium castaneum	Malate dehydrogenase
EOG091203UW	LDEC002421	Leptinotarsa decemlineata	Malate dehydrogenase
EOG091203UW	YQE_11902	Dendroctonus ponderosae	Malate dehydrogenase
EOG091203V1	OTAU009145	Onthophagus taurus	Putative uncharacterized protein
EOG091203V1	AGLA004462	Anoplophora glabripennis	Putative uncharacterized protein
EOG091203V1	APLA009187	Agrilus planipennis	Putative uncharacterized protein
EOG091203V1	TC002710	Tribolium castaneum	Putative uncharacterized protein
EOG091203V1	LDEC013369	Leptinotarsa decemlineata	Putative uncharacterized protein
EOG091203V1	YQE_12334	Dendroctonus ponderosae	Putative uncharacterized protein
EOG091203V2	OTAU005104	Onthophagus taurus	Putative uncharacterized protein
EOG091203V2	AGLA016093	Anoplophora glabripennis	Putative uncharacterized protein
EOG091203V2	APLA012828	Agrilus planipennis	Putative uncharacterized protein
EOG091203V2	TC006323	Tribolium castaneum	Putative uncharacterized protein
EOG091203V2	LDEC006300	Leptinotarsa decemlineata	Putative uncharacterized protein
EOG091203V2	YQE_06908	Dendroctonus ponderosae	Putative uncharacterized protein
EOG091203V4	OTAU008874	Onthophagus taurus	Putative uncharacterized protein
EOG091203V4	AGLA021874	Anoplophora glabripennis	Putative uncharacterized protein
EOG091203V4	APLA009888	Agrilus planipennis	Putative uncharacterized protein
EOG091203V4	TC000577	Tribolium castaneum	Putative uncharacterized protein
EOG091203V4	LDEC010163	Leptinotarsa decemlineata	Putative uncharacterized protein
EOG091203V4	YQE_00618	Dendroctonus ponderosae	Putative uncharacterized protein
EOG091203V5	OTAU002268	Onthophagus taurus	Putative uncharacterized protein
EOG091203V5	AGLA019322	Anoplophora glabripennis	Putative uncharacterized protein
EOG091203V5	APLA002315	Agrilus planipennis	Putative uncharacterized protein
EOG091203V5	TC012192	Tribolium castaneum	Putative uncharacterized protein
EOG091203V5	LDEC018079	Leptinotarsa decemlineata	Putative uncharacterized protein
EOG091203V5	YQE_12844	Dendroctonus ponderosae	Putative uncharacterized protein
EOG091203V7	OTAU010922	Onthophagus taurus	Putative uncharacterized protein
EOG091203V7	AGLA018375	Anoplophora glabripennis	Putative uncharacterized protein
EOG091203V7	APLA011857	Agrilus planipennis	Putative uncharacterized protein
EOG091203V7	TC015126	Tribolium castaneum	Putative uncharacterized protein
EOG091203V7	LDEC002343	Leptinotarsa decemlineata	Putative uncharacterized protein
EOG091203V7	YQE_02170	Dendroctonus ponderosae	Putative uncharacterized protein
EOG091203V8	OTAU006896	Onthophagus taurus	Putative uncharacterized protein
EOG091203V8	AGLA019760	Anoplophora glabripennis	Putative uncharacterized protein
EOG091203V8	APLA005703	Agrilus planipennis	Putative uncharacterized protein
EOG091203V8	TC014519	Tribolium castaneum	Putative uncharacterized protein
EOG091203V8	LDEC016592	Leptinotarsa decemlineata	Putative uncharacterized protein
EOG091203V8	YQE_10542	Dendroctonus ponderosae	Putative uncharacterized protein
EOG091203VA	OTAU004840	Onthophagus taurus	Putative uncharacterized protein
EOG091203VA	AGLA008932	Anoplophora glabripennis	Putative uncharacterized protein
EOG091203VA	APLA009590	Agrilus planipennis	Putative uncharacterized protein
EOG091203VA	TC003258	Tribolium castaneum	Putative uncharacterized protein
EOG091203VA	LDEC003370	Leptinotarsa decemlineata	Putative uncharacterized protein
EOG091203VA	YQE_01846	Dendroctonus ponderosae	Putative uncharacterized protein
EOG091203VB	OTAU014089	Onthophagus taurus	Autophagy-related protein 3
EOG091203VB	AGLA007891	Anoplophora glabripennis	Autophagy-related protein 3
EOG091203VB	APLA004875	Agrilus planipennis	Autophagy-related protein 3
EOG091203VB	TC010917	Tribolium castaneum	Autophagy-related protein 3
EOG091203VB	LDEC003062	Leptinotarsa decemlineata	Autophagy-related protein 3
EOG091203VB	YQE_06109	Dendroctonus ponderosae	Autophagy-related protein 3
EOG091203VF	OTAU012242	Onthophagus taurus	Proteasome subunit alpha type
EOG091203VF	AGLA018515	Anoplophora glabripennis	Proteasome subunit alpha type
EOG091203VF	APLA006975	Agrilus planipennis	Proteasome subunit alpha type
EOG091203VF	TC008727	Tribolium castaneum	Proteasome subunit alpha type
EOG091203VF	LDEC017099	Leptinotarsa decemlineata	Proteasome subunit alpha type
EOG091203VF	YQE_04120	Dendroctonus ponderosae	Proteasome subunit alpha type
EOG091203VG	OTAU003667	Onthophagus taurus	Putative uncharacterized protein
EOG091203VG	AGLA000684	Anoplophora glabripennis	Putative uncharacterized protein
EOG091203VG	APLA005353	Agrilus planipennis	Putative uncharacterized protein
EOG091203VG	TC008186	Tribolium castaneum	Putative uncharacterized protein
EOG091203VG	LDEC006005	Leptinotarsa decemlineata	Putative uncharacterized protein

EOG091203VG	YQE_04725	Dendroctonus ponderosae	Putative uncharacterized protein
EOG091203VH	OTAU007227	Onthophagus taurus	Putative uncharacterized protein
EOG091203VH	AGLA002115	Anoplophora glabripennis	Putative uncharacterized protein
EOG091203VH	APLA000722	Agrilus planipennis	Putative uncharacterized protein
EOG091203VH	TC003468	Tribolium castaneum	Putative uncharacterized protein
EOG091203VH	LDEC010245	Leptinotarsa decemlineata	Putative uncharacterized protein
EOG091203VH	YQE_08256	Dendroctonus ponderosae	Putative uncharacterized protein
EOG091203VK	OTAU000171	Onthophagus taurus	Putative uncharacterized protein
EOG091203VK	AGLA003642	Anoplophora glabripennis	Putative uncharacterized protein
EOG091203VK	APLA009007	Agrilus planipennis	Putative uncharacterized protein
EOG091203VK	TC014715	Tribolium castaneum	Putative uncharacterized protein
EOG091203VK	LDEC003458	Leptinotarsa decemlineata	Putative uncharacterized protein
EOG091203VK	YQE_08600	Dendroctonus ponderosae	Putative uncharacterized protein
EOG091203VO	OTAU007930	Onthophagus taurus	Putative uncharacterized protein
EOG091203VO	AGLA011821	Anoplophora glabripennis	Putative uncharacterized protein
EOG091203VO	APLA003438	Agrilus planipennis	Putative uncharacterized protein
EOG091203VO	TC009871	Tribolium castaneum	Putative uncharacterized protein
EOG091203VO	LDEC001285	Leptinotarsa decemlineata	Putative uncharacterized protein
EOG091203VO	YQE_10032	Dendroctonus ponderosae	Putative uncharacterized protein
EOG091203VP	OTAU001667	Onthophagus taurus	Putative uncharacterized protein
EOG091203VP	AGLA007302	Anoplophora glabripennis	Putative uncharacterized protein
EOG091203VP	APLA009755	Agrilus planipennis	Putative uncharacterized protein
EOG091203VP	TC005562	Tribolium castaneum	Putative uncharacterized protein
EOG091203VP	LDEC013373	Leptinotarsa decemlineata	Putative uncharacterized protein
EOG091203VP	YQE_06873	Dendroctonus ponderosae	Putative uncharacterized protein
EOG091203VQ	OTAU003860	Onthophagus taurus	Putative uncharacterized protein
EOG091203VQ	AGLA008249	Anoplophora glabripennis	Putative uncharacterized protein
EOG091203VQ	APLA013308	Agrilus planipennis	Putative uncharacterized protein
EOG091203VQ	TC009214	Tribolium castaneum	Putative uncharacterized protein
EOG091203VQ	LDEC004826	Leptinotarsa decemlineata	Putative uncharacterized protein
EOG091203VQ	YQE_03475	Dendroctonus ponderosae	Putative uncharacterized protein
EOG091203VU	OTAU004548	Onthophagus taurus	Putative uncharacterized protein
EOG091203VU	AGLA019635	Anoplophora glabripennis	Putative uncharacterized protein
EOG091203VU	APLA014525	Agrilus planipennis	Putative uncharacterized protein
EOG091203VU	TC009504	Tribolium castaneum	Putative uncharacterized protein
EOG091203VU	LDEC011020	Leptinotarsa decemlineata	Putative uncharacterized protein
EOG091203VU	YQE_10568	Dendroctonus ponderosae	Putative uncharacterized protein
EOG091203VV	OTAU008507	Onthophagus taurus	None
EOG091203VV	AGLA011718	Anoplophora glabripennis	None
EOG091203VV	APLA005119	Agrilus planipennis	None
EOG091203VV	TC033263	Tribolium castaneum	None
EOG091203VV	LDEC018985	Leptinotarsa decemlineata	None
EOG091203VV	YQE_07233	Dendroctonus ponderosae	None
EOG091203VX	OTAU007457	Onthophagus taurus	Putative uncharacterized protein
EOG091203VX	AGLA001098	Anoplophora glabripennis	Putative uncharacterized protein
EOG091203VX	APLA005965	Agrilus planipennis	Putative uncharacterized protein
EOG091203VX	TC014942	Tribolium castaneum	Putative uncharacterized protein
EOG091203VX	LDEC005190	Leptinotarsa decemlineata	Putative uncharacterized protein
EOG091203VX	YQE_11735	Dendroctonus ponderosae	Putative uncharacterized protein
EOG091203VZ	OTAU001163	Onthophagus taurus	SIFamide receptor
EOG091203VZ	AGLA004317	Anoplophora glabripennis	SIFamide receptor
EOG091203VZ	APLA005529	Agrilus planipennis	SIFamide receptor
EOG091203VZ	TC011156	Tribolium castaneum	SIFamide receptor
EOG091203VZ	LDEC001777	Leptinotarsa decemlineata	SIFamide receptor
EOG091203VZ	YQE_09537	Dendroctonus ponderosae	SIFamide receptor
EOG091203W0	OTAU009246	Onthophagus taurus	Putative uncharacterized protein
EOG091203W0	AGLA017932	Anoplophora glabripennis	Putative uncharacterized protein
EOG091203W0	APLA011328	Agrilus planipennis	Putative uncharacterized protein
EOG091203W0	TC013308	Tribolium castaneum	Putative uncharacterized protein
EOG091203W0	LDEC003233	Leptinotarsa decemlineata	Putative uncharacterized protein
EOG091203W0	YQE_03421	Dendroctonus ponderosae	Putative uncharacterized protein
EOG091203W2	OTAU000386	Onthophagus taurus	Putative uncharacterized protein
EOG091203W2	AGLA005105	Anoplophora glabripennis	Putative uncharacterized protein
EOG091203W2	APLA007665	Agrilus planipennis	Putative uncharacterized protein
EOG091203W2	TC011917	Tribolium castaneum	Putative uncharacterized protein
EOG091203W2	LDEC003891	Leptinotarsa decemlineata	Putative uncharacterized protein
EOG091203W2	YQE_06371	Dendroctonus ponderosae	Putative uncharacterized protein

EOG091203W6	OTAU003182	Onthophagus taurus	Putative uncharacterized protein
EOG091203W6	AGLA007360	Anoplophora glabripennis	Putative uncharacterized protein
EOG091203W6	APLA003598	Agrilus planipennis	Putative uncharacterized protein
EOG091203W6	TC014311	Tribolium castaneum	Putative uncharacterized protein
EOG091203W6	LDEC014814	Leptinotarsa decemlineata	Putative uncharacterized protein
EOG091203W6	YQE_07160	Dendroctonus ponderosae	Putative uncharacterized protein
EOG091203W7	OTAU005443	Onthophagus taurus	Putative uncharacterized protein
EOG091203W7	AGLA000318	Anoplophora glabripennis	Putative uncharacterized protein
EOG091203W7	APLA005583	Agrilus planipennis	Putative uncharacterized protein
EOG091203W7	TC001762	Tribolium castaneum	Putative uncharacterized protein
EOG091203W7	LDEC020388	Leptinotarsa decemlineata	Putative uncharacterized protein
EOG091203W7	YQE_03242	Dendroctonus ponderosae	Putative uncharacterized protein
EOG091203W8	OTAU015301	Onthophagus taurus	Heparan-sulfate-2-sulfotransferase
EOG091203W8	AGLA020869	Anoplophora glabripennis	Heparan-sulfate-2-sulfotransferase
EOG091203W8	APLA008353	Agrilus planipennis	Heparan-sulfate-2-sulfotransferase
EOG091203W8	TC013753	Tribolium castaneum	Heparan-sulfate-2-sulfotransferase
EOG091203W8	LDEC001127	Leptinotarsa decemlineata	Heparan-sulfate-2-sulfotransferase
EOG091203W8	YQE_08248	Dendroctonus ponderosae	Heparan-sulfate-2-sulfotransferase
EOG091203WH	OTAU005689	Onthophagus taurus	Putative uncharacterized protein
EOG091203WH	AGLA003464	Anoplophora glabripennis	Putative uncharacterized protein
EOG091203WH	APLA010537	Agrilus planipennis	Putative uncharacterized protein
EOG091203WH	TC010201	Tribolium castaneum	Putative uncharacterized protein
EOG091203WH	LDEC001336	Leptinotarsa decemlineata	Putative uncharacterized protein
EOG091203WH	YQE_00036	Dendroctonus ponderosae	Putative uncharacterized protein
EOG091203WI	OTAU010046	Onthophagus taurus	None
EOG091203WI	AGLA010797	Anoplophora glabripennis	None
EOG091203WI	APLA005141	Agrilus planipennis	None
EOG091203WI	TC034711	Tribolium castaneum	None
EOG091203WI	LDEC011734	Leptinotarsa decemlineata	None
EOG091203WI	YQE_06164	Dendroctonus ponderosae	None
EOG091203WK	OTAU004000	Onthophagus taurus	Putative uncharacterized protein
EOG091203WK	AGLA006790	Anoplophora glabripennis	Putative uncharacterized protein
EOG091203WK	APLA005195	Agrilus planipennis	Putative uncharacterized protein
EOG091203WK	TC002425	Tribolium castaneum	Putative uncharacterized protein
EOG091203WK	LDEC009800	Leptinotarsa decemlineata	Putative uncharacterized protein
EOG091203WK	YQE_10890	Dendroctonus ponderosae	Putative uncharacterized protein
EOG091203WL	OTAU009885	Onthophagus taurus	Putative uncharacterized protein
EOG091203WL	AGLA006135	Anoplophora glabripennis	Putative uncharacterized protein
EOG091203WL	APLA002965	Agrilus planipennis	Putative uncharacterized protein
EOG091203WL	TC009997	Tribolium castaneum	Putative uncharacterized protein
EOG091203WL	LDEC018937	Leptinotarsa decemlineata	Putative uncharacterized protein
EOG091203WL	YQE_09862	Dendroctonus ponderosae	Putative uncharacterized protein
EOG091203WM	OTAU003244	Onthophagus taurus	Putative uncharacterized protein
EOG091203WM	AGLA005767	Anoplophora glabripennis	Putative uncharacterized protein
EOG091203WM	APLA011252	Agrilus planipennis	Putative uncharacterized protein
EOG091203WM	TC030769	Tribolium castaneum	Putative uncharacterized protein
EOG091203WM	LDEC017978	Leptinotarsa decemlineata	Putative uncharacterized protein
EOG091203WM	YQE_11523	Dendroctonus ponderosae	Putative uncharacterized protein
EOG091203WQ	OTAU002903	Onthophagus taurus	Putative uncharacterized protein
EOG091203WQ	AGLA005846	Anoplophora glabripennis	Putative uncharacterized protein
EOG091203WQ	APLA010076	Agrilus planipennis	Putative uncharacterized protein
EOG091203WQ	TC009047	Tribolium castaneum	Putative uncharacterized protein
EOG091203WQ	LDEC017974	Leptinotarsa decemlineata	Putative uncharacterized protein
EOG091203WQ	YQE_03515	Dendroctonus ponderosae	Putative uncharacterized protein
EOG091203WT	OTAU004685	Onthophagus taurus	Cytochrome P450 306A1
EOG091203WT	AGLA005521	Anoplophora glabripennis	Cytochrome P450 306A1
EOG091203WT	APLA011773	Agrilus planipennis	Cytochrome P450 306A1
EOG091203WT	TC001916	Tribolium castaneum	Cytochrome P450 306A1
EOG091203WT	LDEC011153	Leptinotarsa decemlineata	Cytochrome P450 306A1
EOG091203WT	YQE_10911	Dendroctonus ponderosae	Cytochrome P450 306A1
EOG091203WW	OTAU001387	Onthophagus taurus	Putative uncharacterized protein
EOG091203WW	AGLA004343	Anoplophora glabripennis	Putative uncharacterized protein
EOG091203WW	APLA007555	Agrilus planipennis	Putative uncharacterized protein
EOG091203WW	TC013781	Tribolium castaneum	Putative uncharacterized protein
EOG091203WW	LDEC014572	Leptinotarsa decemlineata	Putative uncharacterized protein
EOG091203WW	YQE_07560	Dendroctonus ponderosae	Putative uncharacterized protein
EOG091203WY	OTAU001781	Onthophagus taurus	Putative uncharacterized protein

EOG091203WY	AGLA016349	Anoplophora glabripennis	Putative uncharacterized protein
EOG091203WY	APLA004456	Agrilus planipennis	Putative uncharacterized protein
EOG091203WY	TC005793	Tribolium castaneum	Putative uncharacterized protein
EOG091203WY	LDEC006321	Leptinotarsa decemlineata	Putative uncharacterized protein
EOG091203WY	YQE_12069	Dendroctonus ponderosae	Putative uncharacterized protein
EOG091203X0	OTAU008145	Onthophagus taurus	Putative uncharacterized protein
EOG091203X0	AGLA006352	Anoplophora glabripennis	Putative uncharacterized protein
EOG091203X0	APLA011364	Agrilus planipennis	Putative uncharacterized protein
EOG091203X0	TC006020	Tribolium castaneum	Putative uncharacterized protein
EOG091203X0	LDEC021622	Leptinotarsa decemlineata	Putative uncharacterized protein
EOG091203X0	YQE_01883	Dendroctonus ponderosae	Putative uncharacterized protein
EOG091203X1	OTAU003864	Onthophagus taurus	Proteasome subunit beta type
EOG091203X1	AGLA012504	Anoplophora glabripennis	Proteasome subunit beta type
EOG091203X1	APLA014016	Agrilus planipennis	Proteasome subunit beta type
EOG091203X1	TC030625	Tribolium castaneum	Proteasome subunit beta type
EOG091203X1	LDEC010014	Leptinotarsa decemlineata	Proteasome subunit beta type
EOG091203X1	YQE_11784	Dendroctonus ponderosae	Proteasome subunit beta type
EOG091203X2	OTAU011574	Onthophagus taurus	Putative uncharacterized protein
EOG091203X2	AGLA015191	Anoplophora glabripennis	Putative uncharacterized protein
EOG091203X2	APLA013738	Agrilus planipennis	Putative uncharacterized protein
EOG091203X2	TC003914	Tribolium castaneum	Putative uncharacterized protein
EOG091203X2	LDEC009330	Leptinotarsa decemlineata	Putative uncharacterized protein
EOG091203X2	YQE_11147	Dendroctonus ponderosae	Putative uncharacterized protein
EOG091203X4	OTAU000474	Onthophagus taurus	Putative uncharacterized protein
EOG091203X4	AGLA013890	Anoplophora glabripennis	Putative uncharacterized protein
EOG091203X4	APLA001502	Agrilus planipennis	Putative uncharacterized protein
EOG091203X4	TC000138	Tribolium castaneum	Putative uncharacterized protein
EOG091203X4	LDEC002708	Leptinotarsa decemlineata	Putative uncharacterized protein
EOG091203X4	YQE_02184	Dendroctonus ponderosae	Putative uncharacterized protein
EOG091203X7	OTAU006012	Onthophagus taurus	Putative uncharacterized protein
EOG091203X7	AGLA006937	Anoplophora glabripennis	Putative uncharacterized protein
EOG091203X7	APLA005173	Agrilus planipennis	Putative uncharacterized protein
EOG091203X7	TC003789	Tribolium castaneum	Putative uncharacterized protein
EOG091203X7	LDEC017761	Leptinotarsa decemlineata	Putative uncharacterized protein
EOG091203X7	YQE_03821	Dendroctonus ponderosae	Putative uncharacterized protein
EOG091203X8	OTAU007362	Onthophagus taurus	Putative uncharacterized protein
EOG091203X8	AGLA011672	Anoplophora glabripennis	Putative uncharacterized protein
EOG091203X8	APLA011911	Agrilus planipennis	Putative uncharacterized protein
EOG091203X8	TC001040	Tribolium castaneum	Putative uncharacterized protein
EOG091203X8	LDEC012657	Leptinotarsa decemlineata	Putative uncharacterized protein
EOG091203X8	YQE_07731	Dendroctonus ponderosae	Putative uncharacterized protein
EOG091203X9	OTAU010712	Onthophagus taurus	Putative uncharacterized protein
EOG091203X9	AGLA004655	Anoplophora glabripennis	Putative uncharacterized protein
EOG091203X9	APLA003180	Agrilus planipennis	Putative uncharacterized protein
EOG091203X9	TC007773	Tribolium castaneum	Putative uncharacterized protein
EOG091203X9	LDEC000938	Leptinotarsa decemlineata	Putative uncharacterized protein
EOG091203X9	YQE_12439	Dendroctonus ponderosae	Putative uncharacterized protein
EOG091203XC	OTAU007351	Onthophagus taurus	Putative uncharacterized protein
EOG091203XC	AGLA018382	Anoplophora glabripennis	Putative uncharacterized protein
EOG091203XC	APLA007979	Agrilus planipennis	Putative uncharacterized protein
EOG091203XC	TC005007	Tribolium castaneum	Putative uncharacterized protein
EOG091203XC	LDEC005283	Leptinotarsa decemlineata	Putative uncharacterized protein
EOG091203XC	YQE_01740	Dendroctonus ponderosae	Putative uncharacterized protein
EOG091203XD	OTAU013610	Onthophagus taurus	Putative uncharacterized protein
EOG091203XD	AGLA012983	Anoplophora glabripennis	Putative uncharacterized protein
EOG091203XD	APLA010403	Agrilus planipennis	Putative uncharacterized protein
EOG091203XD	TC012071	Tribolium castaneum	Putative uncharacterized protein
EOG091203XD	LDEC020589	Leptinotarsa decemlineata	Putative uncharacterized protein
EOG091203XD	YQE_12577	Dendroctonus ponderosae	Putative uncharacterized protein
EOG091203XE	OTAU000396	Onthophagus taurus	Putative uncharacterized protein
EOG091203XE	AGLA004811	Anoplophora glabripennis	Putative uncharacterized protein
EOG091203XE	APLA004111	Agrilus planipennis	Putative uncharacterized protein
EOG091203XE	TC003582	Tribolium castaneum	Putative uncharacterized protein
EOG091203XE	LDEC023279	Leptinotarsa decemlineata	Putative uncharacterized protein
EOG091203XE	YQE_07880	Dendroctonus ponderosae	Putative uncharacterized protein
EOG091203XG	OTAU002290	Onthophagus taurus	RNA binding
EOG091203XG	AGLA009036	Anoplophora glabripennis	RNA binding

EOG091203XG	APLA011026	Agrilus planipennis	RNA binding
EOG091203XG	TC034663	Tribolium castaneum	RNA binding
EOG091203XG	LDEC012322	Leptinotarsa decemlineata	RNA binding
EOG091203XG	YQE_11591	Dendroctonus ponderosae	RNA binding
EOG091203XI	OTAU008388	Onthophagus taurus	Putative uncharacterized protein
EOG091203XI	AGLA008538	Anoplophora glabripennis	Putative uncharacterized protein
EOG091203XI	APLA004745	Agrilus planipennis	Putative uncharacterized protein
EOG091203XI	TC009456	Tribolium castaneum	Putative uncharacterized protein
EOG091203XI	LDEC014275	Leptinotarsa decemlineata	Putative uncharacterized protein
EOG091203XI	YQE_03531	Dendroctonus ponderosae	Putative uncharacterized protein
EOG091203XJ	OTAU012998	Onthophagus taurus	Putative uncharacterized protein
EOG091203XJ	AGLA016566	Anoplophora glabripennis	Putative uncharacterized protein
EOG091203XJ	APLA008321	Agrilus planipennis	Putative uncharacterized protein
EOG091203XJ	TC007817	Tribolium castaneum	Putative uncharacterized protein
EOG091203XJ	LDEC014389	Leptinotarsa decemlineata	Putative uncharacterized protein
EOG091203XJ	YQE_02231	Dendroctonus ponderosae	Putative uncharacterized protein
EOG091203XK	OTAU010900	Onthophagus taurus	Putative uncharacterized protein
EOG091203XK	AGLA006752	Anoplophora glabripennis	Putative uncharacterized protein
EOG091203XK	APLA000628	Agrilus planipennis	Putative uncharacterized protein
EOG091203XK	TC015324	Tribolium castaneum	Putative uncharacterized protein
EOG091203XK	LDEC009958	Leptinotarsa decemlineata	Putative uncharacterized protein
EOG091203XK	YQE_10816	Dendroctonus ponderosae	Putative uncharacterized protein
EOG091203XL	OTAU004341	Onthophagus taurus	Putative uncharacterized protein
EOG091203XL	AGLA000271	Anoplophora glabripennis	Putative uncharacterized protein
EOG091203XL	APLA009983	Agrilus planipennis	Putative uncharacterized protein
EOG091203XL	TC004765	Tribolium castaneum	Putative uncharacterized protein
EOG091203XL	LDEC001078	Leptinotarsa decemlineata	Putative uncharacterized protein
EOG091203XL	YQE_09087	Dendroctonus ponderosae	Putative uncharacterized protein
EOG091203XM	OTAU004927	Onthophagus taurus	Putative uncharacterized protein
EOG091203XM	AGLA002456	Anoplophora glabripennis	Putative uncharacterized protein
EOG091203XM	APLA005272	Agrilus planipennis	Putative uncharacterized protein
EOG091203XM	TC011461	Tribolium castaneum	Putative uncharacterized protein
EOG091203XM	LDEC011583	Leptinotarsa decemlineata	Putative uncharacterized protein
EOG091203XM	YQE_05478	Dendroctonus ponderosae	Putative uncharacterized protein
EOG091203XN	OTAU005973	Onthophagus taurus	Putative uncharacterized protein
EOG091203XN	AGLA003550	Anoplophora glabripennis	Putative uncharacterized protein
EOG091203XN	APLA013708	Agrilus planipennis	Putative uncharacterized protein
EOG091203XN	TC001570	Tribolium castaneum	Putative uncharacterized protein
EOG091203XN	LDEC016969	Leptinotarsa decemlineata	Putative uncharacterized protein
EOG091203XN	YQE_07398	Dendroctonus ponderosae	Putative uncharacterized protein
EOG091203XP	OTAU002719	Onthophagus taurus	Protein Wnt
EOG091203XP	AGLA011222	Anoplophora glabripennis	Protein Wnt
EOG091203XP	APLA009727	Agrilus planipennis	Protein Wnt
EOG091203XP	TC013707	Tribolium castaneum	Protein Wnt
EOG091203XP	LDEC012982	Leptinotarsa decemlineata	Protein Wnt
EOG091203XP	YQE_13083	Dendroctonus ponderosae	Protein Wnt
EOG091203XQ	OTAU000613	Onthophagus taurus	Putative uncharacterized protein
EOG091203XQ	AGLA005116	Anoplophora glabripennis	Putative uncharacterized protein
EOG091203XQ	APLA006700	Agrilus planipennis	Putative uncharacterized protein
EOG091203XQ	TC012179	Tribolium castaneum	Putative uncharacterized protein
EOG091203XQ	LDEC003881	Leptinotarsa decemlineata	Putative uncharacterized protein
EOG091203XQ	YQE_05790	Dendroctonus ponderosae	Putative uncharacterized protein
EOG091203XR	OTAU008489	Onthophagus taurus	Putative uncharacterized protein
EOG091203XR	AGLA002095	Anoplophora glabripennis	Putative uncharacterized protein
EOG091203XR	APLA008157	Agrilus planipennis	Putative uncharacterized protein
EOG091203XR	TC003306	Tribolium castaneum	Putative uncharacterized protein
EOG091203XR	LDEC019635	Leptinotarsa decemlineata	Putative uncharacterized protein
EOG091203XR	YQE_07419	Dendroctonus ponderosae	Putative uncharacterized protein
EOG091203XS	OTAU009891	Onthophagus taurus	Serine protease P44
EOG091203XS	AGLA000326	Anoplophora glabripennis	Serine protease P44
EOG091203XS	APLA009970	Agrilus planipennis	Serine protease P44
EOG091203XS	TC004160	Tribolium castaneum	Serine protease P44
EOG091203XS	LDEC007479	Leptinotarsa decemlineata	Serine protease P44
EOG091203XS	YQE_06643	Dendroctonus ponderosae	Serine protease P44
EOG091203XV	OTAU004096	Onthophagus taurus	Putative uncharacterized protein
EOG091203XV	AGLA014887	Anoplophora glabripennis	Putative uncharacterized protein
EOG091203XV	APLA008187	Agrilus planipennis	Putative uncharacterized protein

EOG091203XV	TC009947	Tribolium castaneum	Putative uncharacterized protein
EOG091203XV	LDEC011848	Leptinotarsa decemlineata	Putative uncharacterized protein
EOG091203XV	YQE_05415	Dendroctonus ponderosae	Putative uncharacterized protein
EOG091203XY	OTAU003299	Onthophagus taurus	Putative uncharacterized protein
EOG091203XY	AGLA002381	Anoplophora glabripennis	Putative uncharacterized protein
EOG091203XY	APLA006389	Agrilus planipennis	Putative uncharacterized protein
EOG091203XY	TC016055	Tribolium castaneum	Putative uncharacterized protein
EOG091203XY	LDEC003543	Leptinotarsa decemlineata	Putative uncharacterized protein
EOG091203XY	YQE_09141	Dendroctonus ponderosae	Putative uncharacterized protein
EOG091203Y0	OTAU011404	Onthophagus taurus	Putative uncharacterized protein
EOG091203Y0	AGLA020993	Anoplophora glabripennis	Putative uncharacterized protein
EOG091203Y0	APLA011578	Agrilus planipennis	Putative uncharacterized protein
EOG091203Y0	TC014037	Tribolium castaneum	Putative uncharacterized protein
EOG091203Y0	LDEC003484	Leptinotarsa decemlineata	Putative uncharacterized protein
EOG091203Y0	YQE_05094	Dendroctonus ponderosae	Putative uncharacterized protein
EOG091203Y2	OTAU017178	Onthophagus taurus	Putative uncharacterized protein
EOG091203Y2	AGLA000502	Anoplophora glabripennis	Putative uncharacterized protein
EOG091203Y2	APLA000075	Agrilus planipennis	Putative uncharacterized protein
EOG091203Y2	TC007891	Tribolium castaneum	Putative uncharacterized protein
EOG091203Y2	LDEC002389	Leptinotarsa decemlineata	Putative uncharacterized protein
EOG091203Y2	YQE_11897	Dendroctonus ponderosae	Putative uncharacterized protein
EOG091203Y3	OTAU005607	Onthophagus taurus	Putative uncharacterized protein
EOG091203Y3	AGLA011820	Anoplophora glabripennis	Putative uncharacterized protein
EOG091203Y3	APLA010183	Agrilus planipennis	Putative uncharacterized protein
EOG091203Y3	TC009550	Tribolium castaneum	Putative uncharacterized protein
EOG091203Y3	LDEC001286	Leptinotarsa decemlineata	Putative uncharacterized protein
EOG091203Y3	YQE_04105	Dendroctonus ponderosae	Putative uncharacterized protein
EOG091203Y6	OTAU015344	Onthophagus taurus	Putative uncharacterized protein
EOG091203Y6	AGLA006772	Anoplophora glabripennis	Putative uncharacterized protein
EOG091203Y6	APLA012228	Agrilus planipennis	Putative uncharacterized protein
EOG091203Y6	TC012928	Tribolium castaneum	Putative uncharacterized protein
EOG091203Y6	LDEC000763	Leptinotarsa decemlineata	Putative uncharacterized protein
EOG091203Y6	YQE_01822	Dendroctonus ponderosae	Putative uncharacterized protein
EOG091203YC	OTAU003980	Onthophagus taurus	None
EOG091203YC	AGLA008960	Anoplophora glabripennis	None
EOG091203YC	APLA010064	Agrilus planipennis	None
EOG091203YC	TC034275	Tribolium castaneum	None
EOG091203YC	LDEC014103	Leptinotarsa decemlineata	None
EOG091203YC	YQE_11598	Dendroctonus ponderosae	None
EOG091203YD	OTAU009311	Onthophagus taurus	Putative uncharacterized protein
EOG091203YD	AGLA011836	Anoplophora glabripennis	Putative uncharacterized protein
EOG091203YD	APLA009811	Agrilus planipennis	Putative uncharacterized protein
EOG091203YD	TC009870	Tribolium castaneum	Putative uncharacterized protein
EOG091203YD	LDEC001289	Leptinotarsa decemlineata	Putative uncharacterized protein
EOG091203YD	YQE_13059	Dendroctonus ponderosae	Putative uncharacterized protein
EOG091203YF	OTAU000498	Onthophagus taurus	neuropeptide Y receptor activity
EOG091203YF	AGLA008360	Anoplophora glabripennis	neuropeptide Y receptor activity
EOG091203YF	APLA013182	Agrilus planipennis	neuropeptide Y receptor activity
EOG091203YF	TC034469	Tribolium castaneum	neuropeptide Y receptor activity
EOG091203YF	LDEC011839	Leptinotarsa decemlineata	neuropeptide Y receptor activity
EOG091203YF	YQE_04397	Dendroctonus ponderosae	neuropeptide Y receptor activity
EOG091203YH	OTAU009254	Onthophagus taurus	Putative uncharacterized protein
EOG091203YH	AGLA004439	Anoplophora glabripennis	Putative uncharacterized protein
EOG091203YH	APLA008271	Agrilus planipennis	Putative uncharacterized protein
EOG091203YH	TC014607	Tribolium castaneum	Putative uncharacterized protein
EOG091203YH	LDEC001197	Leptinotarsa decemlineata	Putative uncharacterized protein
EOG091203YH	YQE_06135	Dendroctonus ponderosae	Putative uncharacterized protein
EOG091203YJ	OTAU000425	Onthophagus taurus	None
EOG091203YJ	AGLA012985	Anoplophora glabripennis	None
EOG091203YJ	APLA007667	Agrilus planipennis	None
EOG091203YJ	TC031235	Tribolium castaneum	None
EOG091203YJ	LDEC020590	Leptinotarsa decemlineata	None
EOG091203YJ	YQE_05486	Dendroctonus ponderosae	None
EOG091203YL	OTAU013010	Onthophagus taurus	Putative uncharacterized protein
EOG091203YL	AGLA011070	Anoplophora glabripennis	Putative uncharacterized protein
EOG091203YL	APLA000500	Agrilus planipennis	Putative uncharacterized protein
EOG091203YL	TC015990	Tribolium castaneum	Putative uncharacterized protein

EOG091203YL	LDEC020212	Leptinotarsa decemlineata	Putative uncharacterized protein
EOG091203YL	YQE_12729	Dendroctonus ponderosae	Putative uncharacterized protein
EOG091203YP	OTAU000648	Onthophagus taurus	Putative uncharacterized protein
EOG091203YP	AGLA013854	Anoplophora glabripennis	Putative uncharacterized protein
EOG091203YP	APLA002340	Agrilus planipennis	Putative uncharacterized protein
EOG091203YP	TC012096	Tribolium castaneum	Putative uncharacterized protein
EOG091203YP	LDEC007654	Leptinotarsa decemlineata	Putative uncharacterized protein
EOG091203YP	YQE_11971	Dendroctonus ponderosae	Putative uncharacterized protein
EOG091203YQ	OTAU005560	Onthophagus taurus	Putative uncharacterized protein
EOG091203YQ	AGLA014180	Anoplophora glabripennis	Putative uncharacterized protein
EOG091203YQ	APLA001414	Agrilus planipennis	Putative uncharacterized protein
EOG091203YQ	TC008017	Tribolium castaneum	Putative uncharacterized protein
EOG091203YQ	LDEC015747	Leptinotarsa decemlineata	Putative uncharacterized protein
EOG091203YQ	YQE_04654	Dendroctonus ponderosae	Putative uncharacterized protein
EOG091203YS	OTAU012948	Onthophagus taurus	None
EOG091203YS	AGLA000396	Anoplophora glabripennis	None
EOG091203YS	APLA013629	Agrilus planipennis	None
EOG091203YS	TC031509	Tribolium castaneum	None
EOG091203YS	LDEC004684	Leptinotarsa decemlineata	None
EOG091203YS	YQE_03270	Dendroctonus ponderosae	None
EOG091203YT	OTAU009205	Onthophagus taurus	Boule
EOG091203YT	AGLA015066	Anoplophora glabripennis	Boule
EOG091203YT	APLA000650	Agrilus planipennis	Boule
EOG091203YT	TC015063	Tribolium castaneum	Boule
EOG091203YT	LDEC005610	Leptinotarsa decemlineata	Boule
EOG091203YT	YQE_11580	Dendroctonus ponderosae	Boule
EOG091203YX	OTAU002709	Onthophagus taurus	Putative uncharacterized protein
EOG091203YX	AGLA018060	Anoplophora glabripennis	Putative uncharacterized protein
EOG091203YX	APLA013905	Agrilus planipennis	Putative uncharacterized protein
EOG091203YX	TC010400	Tribolium castaneum	Putative uncharacterized protein
EOG091203YX	LDEC002748	Leptinotarsa decemlineata	Putative uncharacterized protein
EOG091203YX	YQE_01725	Dendroctonus ponderosae	Putative uncharacterized protein
EOG091203YZ	OTAU011954	Onthophagus taurus	Putative uncharacterized protein
EOG091203YZ	AGLA001712	Anoplophora glabripennis	Putative uncharacterized protein
EOG091203YZ	APLA001675	Agrilus planipennis	Putative uncharacterized protein
EOG091203YZ	TC008731	Tribolium castaneum	Putative uncharacterized protein
EOG091203YZ	LDEC016007	Leptinotarsa decemlineata	Putative uncharacterized protein
EOG091203YZ	YQE_12611	Dendroctonus ponderosae	Putative uncharacterized protein
EOG091203Z2	OTAU006864	Onthophagus taurus	None
EOG091203Z2	AGLA008464	Anoplophora glabripennis	None
EOG091203Z2	APLA008282	Agrilus planipennis	None
EOG091203Z2	TC031144	Tribolium castaneum	None
EOG091203Z2	LDEC008325	Leptinotarsa decemlineata	None
EOG091203Z2	YQE_06272	Dendroctonus ponderosae	None
EOG091203Z4	OTAU011114	Onthophagus taurus	Putative uncharacterized protein
EOG091203Z4	AGLA002232	Anoplophora glabripennis	Putative uncharacterized protein
EOG091203Z4	APLA010394	Agrilus planipennis	Putative uncharacterized protein
EOG091203Z4	TC014371	Tribolium castaneum	Putative uncharacterized protein
EOG091203Z4	LDEC002154	Leptinotarsa decemlineata	Putative uncharacterized protein
EOG091203Z4	YQE_05069	Dendroctonus ponderosae	Putative uncharacterized protein
EOG091203Z5	OTAU002167	Onthophagus taurus	Putative uncharacterized protein
EOG091203Z5	AGLA011325	Anoplophora glabripennis	Putative uncharacterized protein
EOG091203Z5	APLA003997	Agrilus planipennis	Putative uncharacterized protein
EOG091203Z5	TC002162	Tribolium castaneum	Putative uncharacterized protein
EOG091203Z5	LDEC005732	Leptinotarsa decemlineata	Putative uncharacterized protein
EOG091203Z5	YQE_06378	Dendroctonus ponderosae	Putative uncharacterized protein
EOG091203Z6	OTAU000719	Onthophagus taurus	Putative uncharacterized protein
EOG091203Z6	AGLA014848	Anoplophora glabripennis	Putative uncharacterized protein
EOG091203Z6	APLA001772	Agrilus planipennis	Putative uncharacterized protein
EOG091203Z6	TC012205	Tribolium castaneum	Putative uncharacterized protein
EOG091203Z6	LDEC000502	Leptinotarsa decemlineata	Putative uncharacterized protein
EOG091203Z6	YQE_11081	Dendroctonus ponderosae	Putative uncharacterized protein
EOG091203Z7	OTAU001168	Onthophagus taurus	Putative uncharacterized protein
EOG091203Z7	AGLA018383	Anoplophora glabripennis	Putative uncharacterized protein
EOG091203Z7	APLA011701	Agrilus planipennis	Putative uncharacterized protein
EOG091203Z7	TC004385	Tribolium castaneum	Putative uncharacterized protein
EOG091203Z7	LDEC005281	Leptinotarsa decemlineata	Putative uncharacterized protein

EOG091203Z7	YQE_04608	Dendroctonus ponderosae	Putative uncharacterized protein
EOG091203Z8	OTAU015931	Onthophagus taurus	Putative uncharacterized protein
EOG091203Z8	AGLA012793	Anoplophora glabripennis	Putative uncharacterized protein
EOG091203Z8	APLA014881	Agrilus planipennis	Putative uncharacterized protein
EOG091203Z8	TC012908	Tribolium castaneum	Putative uncharacterized protein
EOG091203Z8	LDEC016465	Leptinotarsa decemlineata	Putative uncharacterized protein
EOG091203Z8	YQE_02678	Dendroctonus ponderosae	Putative uncharacterized protein
EOG091203Z9	OTAU013723	Onthophagus taurus	Annexin
EOG091203Z9	AGLA001996	Anoplophora glabripennis	Annexin
EOG091203Z9	APLA002393	Agrilus planipennis	Annexin
EOG091203Z9	TC011788	Tribolium castaneum	Annexin
EOG091203Z9	LDEC022622	Leptinotarsa decemlineata	Annexin
EOG091203Z9	YQE_08432	Dendroctonus ponderosae	Annexin
EOG091203ZB	OTAU005628	Onthophagus taurus	Putative uncharacterized protein
EOG091203ZB	AGLA009683	Anoplophora glabripennis	Putative uncharacterized protein
EOG091203ZB	APLA011239	Agrilus planipennis	Putative uncharacterized protein
EOG091203ZB	TC008606	Tribolium castaneum	Putative uncharacterized protein
EOG091203ZB	LDEC019994	Leptinotarsa decemlineata	Putative uncharacterized protein
EOG091203ZB	YQE_03453	Dendroctonus ponderosae	Putative uncharacterized protein
EOG091203ZD	OTAU001782	Onthophagus taurus	Putative uncharacterized protein
EOG091203ZD	AGLA004783	Anoplophora glabripennis	Putative uncharacterized protein
EOG091203ZD	APLA000925	Agrilus planipennis	Putative uncharacterized protein
EOG091203ZD	TC005715	Tribolium castaneum	Putative uncharacterized protein
EOG091203ZD	LDEC008650	Leptinotarsa decemlineata	Putative uncharacterized protein
EOG091203ZD	YQE_05800	Dendroctonus ponderosae	Putative uncharacterized protein
EOG091203ZF	OTAU002552	Onthophagus taurus	Putative uncharacterized protein
EOG091203ZF	AGLA018489	Anoplophora glabripennis	Putative uncharacterized protein
EOG091203ZF	APLA015046	Agrilus planipennis	Putative uncharacterized protein
EOG091203ZF	TC016311	Tribolium castaneum	Putative uncharacterized protein
EOG091203ZF	LDEC020893	Leptinotarsa decemlineata	Putative uncharacterized protein
EOG091203ZF	YQE_02048	Dendroctonus ponderosae	Putative uncharacterized protein
EOG091203ZG	OTAU007722	Onthophagus taurus	Putative uncharacterized protein
EOG091203ZG	AGLA001760	Anoplophora glabripennis	Putative uncharacterized protein
EOG091203ZG	APLA008108	Agrilus planipennis	Putative uncharacterized protein
EOG091203ZG	TC013555	Tribolium castaneum	Putative uncharacterized protein
EOG091203ZG	LDEC006197	Leptinotarsa decemlineata	Putative uncharacterized protein
EOG091203ZG	YQE_03980	Dendroctonus ponderosae	Putative uncharacterized protein
EOG091203ZH	OTAU003599	Onthophagus taurus	Diphosphomevalonate decarboxylase
EOG091203ZH	AGLA004253	Anoplophora glabripennis	Diphosphomevalonate decarboxylase
EOG091203ZH	APLA000828	Agrilus planipennis	Diphosphomevalonate decarboxylase
EOG091203ZH	TC003224	Tribolium castaneum	Diphosphomevalonate decarboxylase
EOG091203ZH	LDEC008208	Leptinotarsa decemlineata	Diphosphomevalonate decarboxylase
EOG091203ZH	YQE_07816	Dendroctonus ponderosae	Diphosphomevalonate decarboxylase
EOG091203ZI	OTAU000567	Onthophagus taurus	Putative uncharacterized protein
EOG091203ZI	AGLA017198	Anoplophora glabripennis	Putative uncharacterized protein
EOG091203ZI	APLA004008	Agrilus planipennis	Putative uncharacterized protein
EOG091203ZI	TC012308	Tribolium castaneum	Putative uncharacterized protein
EOG091203ZI	LDEC003866	Leptinotarsa decemlineata	Putative uncharacterized protein
EOG091203ZI	YQE_10072	Dendroctonus ponderosae	Putative uncharacterized protein
EOG091203ZK	OTAU013368	Onthophagus taurus	Putative uncharacterized protein
EOG091203ZK	AGLA014733	Anoplophora glabripennis	Putative uncharacterized protein
EOG091203ZK	APLA004774	Agrilus planipennis	Putative uncharacterized protein
EOG091203ZK	TC006476	Tribolium castaneum	Putative uncharacterized protein
EOG091203ZK	LDEC010111	Leptinotarsa decemlineata	Putative uncharacterized protein
EOG091203ZK	YQE_12027	Dendroctonus ponderosae	Putative uncharacterized protein
EOG091203ZL	OTAU011944	Onthophagus taurus	Putative uncharacterized protein
EOG091203ZL	AGLA021554	Anoplophora glabripennis	Putative uncharacterized protein
EOG091203ZL	APLA007386	Agrilus planipennis	Putative uncharacterized protein
EOG091203ZL	TC013795	Tribolium castaneum	Putative uncharacterized protein
EOG091203ZL	LDEC020311	Leptinotarsa decemlineata	Putative uncharacterized protein
EOG091203ZL	YQE_02041	Dendroctonus ponderosae	Putative uncharacterized protein
EOG091203ZN	OTAU012624	Onthophagus taurus	Putative uncharacterized protein
EOG091203ZN	AGLA011591	Anoplophora glabripennis	Putative uncharacterized protein
EOG091203ZN	APLA014034	Agrilus planipennis	Putative uncharacterized protein
EOG091203ZN	TC009164	Tribolium castaneum	Putative uncharacterized protein
EOG091203ZN	LDEC011985	Leptinotarsa decemlineata	Putative uncharacterized protein
EOG091203ZN	YQE_10554	Dendroctonus ponderosae	Putative uncharacterized protein

EOG091203ZQ	OTAU003836	Onthophagus taurus	Putative uncharacterized protein
EOG091203ZQ	AGLA020335	Anoplophora glabripennis	Putative uncharacterized protein
EOG091203ZQ	APLA007056	Agrilus planipennis	Putative uncharacterized protein
EOG091203ZQ	TC009789	Tribolium castaneum	Putative uncharacterized protein
EOG091203ZQ	LDEC005218	Leptinotarsa decemlineata	Putative uncharacterized protein
EOG091203ZQ	YQE_08803	Dendroctonus ponderosae	Putative uncharacterized protein
EOG091203ZR	OTAU006863	Onthophagus taurus	Putative uncharacterized protein
EOG091203ZR	AGLA008460	Anoplophora glabripennis	Putative uncharacterized protein
EOG091203ZR	APLA008280	Agrilus planipennis	Putative uncharacterized protein
EOG091203ZR	TC014293	Tribolium castaneum	Putative uncharacterized protein
EOG091203ZR	LDEC008330	Leptinotarsa decemlineata	Putative uncharacterized protein
EOG091203ZR	YQE_06276	Dendroctonus ponderosae	Putative uncharacterized protein
EOG091203ZS	OTAU000118	Onthophagus taurus	Gustatory receptor
EOG091203ZS	AGLA014951	Anoplophora glabripennis	Gustatory receptor
EOG091203ZS	APLA006867	Agrilus planipennis	Gustatory receptor
EOG091203ZS	TC014690	Tribolium castaneum	Gustatory receptor
EOG091203ZS	LDEC002040	Leptinotarsa decemlineata	Gustatory receptor
EOG091203ZS	YQE_10245	Dendroctonus ponderosae	Gustatory receptor
EOG091203ZT	OTAU010851	Onthophagus taurus	Putative uncharacterized protein
EOG091203ZT	AGLA000631	Anoplophora glabripennis	Putative uncharacterized protein
EOG091203ZT	APLA014264	Agrilus planipennis	Putative uncharacterized protein
EOG091203ZT	TC000747	Tribolium castaneum	Putative uncharacterized protein
EOG091203ZT	LDEC003162	Leptinotarsa decemlineata	Putative uncharacterized protein
EOG091203ZT	YQE_01806	Dendroctonus ponderosae	Putative uncharacterized protein
EOG091203ZU	OTAU011615	Onthophagus taurus	Putative uncharacterized protein
EOG091203ZU	AGLA018699	Anoplophora glabripennis	Putative uncharacterized protein
EOG091203ZU	APLA007048	Agrilus planipennis	Putative uncharacterized protein
EOG091203ZU	TC009383	Tribolium castaneum	Putative uncharacterized protein
EOG091203ZU	LDEC014668	Leptinotarsa decemlineata	Putative uncharacterized protein
EOG091203ZU	YQE_12993	Dendroctonus ponderosae	Putative uncharacterized protein
EOG091203ZW	OTAU013712	Onthophagus taurus	None
EOG091203ZW	AGLA017445	Anoplophora glabripennis	None
EOG091203ZW	APLA011735	Agrilus planipennis	None
EOG091203ZW	TC032492	Tribolium castaneum	None
EOG091203ZW	LDEC005969	Leptinotarsa decemlineata	None
EOG091203ZW	YQE_06178	Dendroctonus ponderosae	None
EOG09120408	OTAU009557	Onthophagus taurus	Putative uncharacterized protein
EOG09120408	AGLA004656	Anoplophora glabripennis	Putative uncharacterized protein
EOG09120408	APLA008182	Agrilus planipennis	Putative uncharacterized protein
EOG09120408	TC007722	Tribolium castaneum	Putative uncharacterized protein
EOG09120408	LDEC000939	Leptinotarsa decemlineata	Putative uncharacterized protein
EOG09120408	YQE_05463	Dendroctonus ponderosae	Putative uncharacterized protein
EOG09120409	OTAU003873	Onthophagus taurus	Putative uncharacterized protein
EOG09120409	AGLA011838	Anoplophora glabripennis	Putative uncharacterized protein
EOG09120409	APLA013650	Agrilus planipennis	Putative uncharacterized protein
EOG09120409	TC009964	Tribolium castaneum	Putative uncharacterized protein
EOG09120409	LDEC009358	Leptinotarsa decemlineata	Putative uncharacterized protein
EOG09120409	YQE_05254	Dendroctonus ponderosae	Putative uncharacterized protein
EOG0912040A	OTAU014334	Onthophagus taurus	Obstractor B
EOG0912040A	AGLA009081	Anoplophora glabripennis	Obstractor B
EOG0912040A	APLA010776	Agrilus planipennis	Obstractor B
EOG0912040A	TC011139	Tribolium castaneum	Obstractor B
EOG0912040A	LDEC010572	Leptinotarsa decemlineata	Obstractor B
EOG0912040A	YQE_11656	Dendroctonus ponderosae	Obstractor B
EOG0912040B	OTAU000568	Onthophagus taurus	Putative uncharacterized protein
EOG0912040B	AGLA017199	Anoplophora glabripennis	Putative uncharacterized protein
EOG0912040B	APLA002535	Agrilus planipennis	Putative uncharacterized protein
EOG0912040B	TC012167	Tribolium castaneum	Putative uncharacterized protein
EOG0912040B	LDEC003867	Leptinotarsa decemlineata	Putative uncharacterized protein
EOG0912040B	YQE_10071	Dendroctonus ponderosae	Putative uncharacterized protein
EOG0912040D	OTAU007683	Onthophagus taurus	Putative uncharacterized protein
EOG0912040D	AGLA005916	Anoplophora glabripennis	Putative uncharacterized protein
EOG0912040D	APLA005551	Agrilus planipennis	Putative uncharacterized protein
EOG0912040D	TC011609	Tribolium castaneum	Putative uncharacterized protein
EOG0912040D	LDEC014478	Leptinotarsa decemlineata	Putative uncharacterized protein
EOG0912040D	YQE_12876	Dendroctonus ponderosae	Putative uncharacterized protein
EOG0912040G	OTAU012899	Onthophagus taurus	Serine protease P12

EOG0912040G	AGLA003242	Anoplophora glabripennis	Serine protease P12
EOG0912040G	APLA008919	Agrilus planipennis	Serine protease P12
EOG0912040G	TC000546	Tribolium castaneum	Serine protease P12
EOG0912040G	LDEC008091	Leptinotarsa decemlineata	Serine protease P12
EOG0912040G	YQE_07858	Dendroctonus ponderosae	Serine protease P12
EOG0912040H	OTAU014178	Onthophagus taurus	DNA binding
EOG0912040H	AGLA005431	Anoplophora glabripennis	DNA binding
EOG0912040H	APLA010935	Agrilus planipennis	DNA binding
EOG0912040H	TC034116	Tribolium castaneum	DNA binding
EOG0912040H	LDEC006652	Leptinotarsa decemlineata	DNA binding
EOG0912040H	YQE_12216	Dendroctonus ponderosae	DNA binding
EOG0912040M	OTAU011860	Onthophagus taurus	Putative uncharacterized protein
EOG0912040M	AGLA010600	Anoplophora glabripennis	Putative uncharacterized protein
EOG0912040M	APLA002886	Agrilus planipennis	Putative uncharacterized protein
EOG0912040M	TC014760	Tribolium castaneum	Putative uncharacterized protein
EOG0912040M	LDEC015118	Leptinotarsa decemlineata	Putative uncharacterized protein
EOG0912040M	YQE_10372	Dendroctonus ponderosae	Putative uncharacterized protein
EOG0912040O	OTAU000752	Onthophagus taurus	Putative uncharacterized protein
EOG0912040O	AGLA018572	Anoplophora glabripennis	Putative uncharacterized protein
EOG0912040O	APLA003948	Agrilus planipennis	Putative uncharacterized protein
EOG0912040O	TC011922	Tribolium castaneum	Putative uncharacterized protein
EOG0912040O	LDEC003876	Leptinotarsa decemlineata	Putative uncharacterized protein
EOG0912040O	YQE_01951	Dendroctonus ponderosae	Putative uncharacterized protein
EOG0912040P	OTAU003218	Onthophagus taurus	DNA binding
EOG0912040P	AGLA004906	Anoplophora glabripennis	DNA binding
EOG0912040P	APLA004734	Agrilus planipennis	DNA binding
EOG0912040P	TC034783	Tribolium castaneum	DNA binding
EOG0912040P	LDEC011332	Leptinotarsa decemlineata	DNA binding
EOG0912040P	YQE_05307	Dendroctonus ponderosae	DNA binding
EOG0912040Q	OTAU002705	Onthophagus taurus	None
EOG0912040Q	AGLA020491	Anoplophora glabripennis	None
EOG0912040Q	APLA013913	Agrilus planipennis	None
EOG0912040Q	TC033052	Tribolium castaneum	None
EOG0912040Q	LDEC002764	Leptinotarsa decemlineata	None
EOG0912040Q	YQE_10242	Dendroctonus ponderosae	None
EOG0912040T	OTAU006131	Onthophagus taurus	Putative uncharacterized protein
EOG0912040T	AGLA008619	Anoplophora glabripennis	Putative uncharacterized protein
EOG0912040T	APLA007904	Agrilus planipennis	Putative uncharacterized protein
EOG0912040T	TC011042	Tribolium castaneum	Putative uncharacterized protein
EOG0912040T	LDEC009129	Leptinotarsa decemlineata	Putative uncharacterized protein
EOG0912040T	YQE_08648	Dendroctonus ponderosae	Putative uncharacterized protein
EOG0912040X	OTAU005888	Onthophagus taurus	Proteasome subunit beta type
EOG0912040X	AGLA015564	Anoplophora glabripennis	Proteasome subunit beta type
EOG0912040X	APLA012977	Agrilus planipennis	Proteasome subunit beta type
EOG0912040X	TC010442	Tribolium castaneum	Proteasome subunit beta type
EOG0912040X	LDEC020234	Leptinotarsa decemlineata	Proteasome subunit beta type
EOG0912040X	YQE_11398	Dendroctonus ponderosae	Proteasome subunit beta type
EOG0912040Y	OTAU003512	Onthophagus taurus	Putative uncharacterized protein
EOG0912040Y	AGLA016379	Anoplophora glabripennis	Putative uncharacterized protein
EOG0912040Y	APLA010073	Agrilus planipennis	Putative uncharacterized protein
EOG0912040Y	TC009139	Tribolium castaneum	Putative uncharacterized protein
EOG0912040Y	LDEC009876	Leptinotarsa decemlineata	Putative uncharacterized protein
EOG0912040Y	YQE_13066	Dendroctonus ponderosae	Putative uncharacterized protein
EOG09120410	OTAU017018	Onthophagus taurus	None
EOG09120410	AGLA000508	Anoplophora glabripennis	None
EOG09120410	APLA013395	Agrilus planipennis	None
EOG09120410	TC032798	Tribolium castaneum	None
EOG09120410	LDEC002382	Leptinotarsa decemlineata	None
EOG09120410	YQE_11918	Dendroctonus ponderosae	None
EOG09120411	OTAU005997	Onthophagus taurus	Putative uncharacterized protein
EOG09120411	AGLA021722	Anoplophora glabripennis	Putative uncharacterized protein
EOG09120411	APLA009166	Agrilus planipennis	Putative uncharacterized protein
EOG09120411	TC002350	Tribolium castaneum	Putative uncharacterized protein
EOG09120411	LDEC003343	Leptinotarsa decemlineata	Putative uncharacterized protein
EOG09120411	YQE_12432	Dendroctonus ponderosae	Putative uncharacterized protein
EOG09120412	OTAU004855	Onthophagus taurus	Putative uncharacterized protein
EOG09120412	AGLA006198	Anoplophora glabripennis	Putative uncharacterized protein

EOG09120412	APLA000276	Agrilus planipennis	Putative uncharacterized protein
EOG09120412	TC003574	Tribolium castaneum	Putative uncharacterized protein
EOG09120412	LDEC010893	Leptinotarsa decemlineata	Putative uncharacterized protein
EOG09120412	YQE_07891	Dendroctonus ponderosae	Putative uncharacterized protein
EOG09120414	OTAU013196	Onthophagus taurus	Putative uncharacterized protein
EOG09120414	AGLA001660	Anoplophora glabripennis	Putative uncharacterized protein
EOG09120414	APLA000491	Agrilus planipennis	Putative uncharacterized protein
EOG09120414	TC005104	Tribolium castaneum	Putative uncharacterized protein
EOG09120414	LDEC011193	Leptinotarsa decemlineata	Putative uncharacterized protein
EOG09120414	YQE_12773	Dendroctonus ponderosae	Putative uncharacterized protein
EOG09120418	OTAU003570	Onthophagus taurus	Putative uncharacterized protein
EOG09120418	AGLA002596	Anoplophora glabripennis	Putative uncharacterized protein
EOG09120418	APLA010419	Agrilus planipennis	Putative uncharacterized protein
EOG09120418	TC010664	Tribolium castaneum	Putative uncharacterized protein
EOG09120418	LDEC002011	Leptinotarsa decemlineata	Putative uncharacterized protein
EOG09120418	YQE_10061	Dendroctonus ponderosae	Putative uncharacterized protein
EOG0912041B	OTAU009532	Onthophagus taurus	Putative uncharacterized protein
EOG0912041B	AGLA001114	Anoplophora glabripennis	Putative uncharacterized protein
EOG0912041B	APLA004757	Agrilus planipennis	Putative uncharacterized protein
EOG0912041B	TC009732	Tribolium castaneum	Putative uncharacterized protein
EOG0912041B	LDEC005834	Leptinotarsa decemlineata	Putative uncharacterized protein
EOG0912041B	YQE_06031	Dendroctonus ponderosae	Putative uncharacterized protein
EOG0912041C	OTAU017200	Onthophagus taurus	Putative uncharacterized protein
EOG0912041C	AGLA016408	Anoplophora glabripennis	Putative uncharacterized protein
EOG0912041C	APLA002662	Agrilus planipennis	Putative uncharacterized protein
EOG0912041C	TC010609	Tribolium castaneum	Putative uncharacterized protein
EOG0912041C	LDEC016525	Leptinotarsa decemlineata	Putative uncharacterized protein
EOG0912041C	YQE_11239	Dendroctonus ponderosae	Putative uncharacterized protein
EOG0912041D	OTAU016459	Onthophagus taurus	Putative uncharacterized protein
EOG0912041D	AGLA003367	Anoplophora glabripennis	Putative uncharacterized protein
EOG0912041D	APLA001523	Agrilus planipennis	Putative uncharacterized protein
EOG0912041D	TC002837	Tribolium castaneum	Putative uncharacterized protein
EOG0912041D	LDEC024133	Leptinotarsa decemlineata	Putative uncharacterized protein
EOG0912041D	YQE_05161	Dendroctonus ponderosae	Putative uncharacterized protein
EOG0912041E	OTAU006149	Onthophagus taurus	Putative uncharacterized protein
EOG0912041E	AGLA012928	Anoplophora glabripennis	Putative uncharacterized protein
EOG0912041E	APLA007874	Agrilus planipennis	Putative uncharacterized protein
EOG0912041E	TC004364	Tribolium castaneum	Putative uncharacterized protein
EOG0912041E	LDEC003564	Leptinotarsa decemlineata	Putative uncharacterized protein
EOG0912041E	YQE_00040	Dendroctonus ponderosae	Putative uncharacterized protein
EOG0912041F	OTAU002877	Onthophagus taurus	Putative uncharacterized protein
EOG0912041F	AGLA010961	Anoplophora glabripennis	Putative uncharacterized protein
EOG0912041F	APLA012589	Agrilus planipennis	Putative uncharacterized protein
EOG0912041F	TC015957	Tribolium castaneum	Putative uncharacterized protein
EOG0912041F	LDEC020077	Leptinotarsa decemlineata	Putative uncharacterized protein
EOG0912041F	YQE_10287	Dendroctonus ponderosae	Putative uncharacterized protein
EOG0912041G	OTAU000312	Onthophagus taurus	Putative uncharacterized protein
EOG0912041G	AGLA008318	Anoplophora glabripennis	Putative uncharacterized protein
EOG0912041G	APLA003074	Agrilus planipennis	Putative uncharacterized protein
EOG0912041G	TC013770	Tribolium castaneum	Putative uncharacterized protein
EOG0912041G	LDEC005362	Leptinotarsa decemlineata	Putative uncharacterized protein
EOG0912041G	YQE_11375	Dendroctonus ponderosae	Putative uncharacterized protein
EOG0912041I	OTAU011752	Onthophagus taurus	Putative uncharacterized protein
EOG0912041I	AGLA003871	Anoplophora glabripennis	Putative uncharacterized protein
EOG0912041I	APLA002574	Agrilus planipennis	Putative uncharacterized protein
EOG0912041I	TC030628	Tribolium castaneum	Putative uncharacterized protein
EOG0912041I	LDEC001820	Leptinotarsa decemlineata	Putative uncharacterized protein
EOG0912041I	YQE_03115	Dendroctonus ponderosae	Putative uncharacterized protein
EOG0912041M	OTAU011762	Onthophagus taurus	Putative uncharacterized protein
EOG0912041M	AGLA002814	Anoplophora glabripennis	Putative uncharacterized protein
EOG0912041M	APLA005702	Agrilus planipennis	Putative uncharacterized protein
EOG0912041M	TC009782	Tribolium castaneum	Putative uncharacterized protein
EOG0912041M	LDEC015013	Leptinotarsa decemlineata	Putative uncharacterized protein
EOG0912041M	YQE_11546	Dendroctonus ponderosae	Putative uncharacterized protein
EOG0912041Q	OTAU012323	Onthophagus taurus	Putative uncharacterized protein
EOG0912041Q	AGLA000178	Anoplophora glabripennis	Putative uncharacterized protein
EOG0912041Q	APLA008504	Agrilus planipennis	Putative uncharacterized protein

EOG0912041Q	TC014454	Tribolium castaneum	Putative uncharacterized protein
EOG0912041Q	LDEC004902	Leptinotarsa decemlineata	Putative uncharacterized protein
EOG0912041Q	YQE_08138	Dendroctonus ponderosae	Putative uncharacterized protein
EOG0912041U	OTAU005794	Onthophagus taurus	Putative uncharacterized protein
EOG0912041U	AGLA017743	Anoplophora glabripennis	Putative uncharacterized protein
EOG0912041U	APLA000178	Agrilus planipennis	Putative uncharacterized protein
EOG0912041U	TC003400	Tribolium castaneum	Putative uncharacterized protein
EOG0912041U	LDEC005902	Leptinotarsa decemlineata	Putative uncharacterized protein
EOG0912041U	YQE_09991	Dendroctonus ponderosae	Putative uncharacterized protein
EOG0912041V	OTAU000382	Onthophagus taurus	Eukaryotic translation initiation factor 3 subunit G
EOG0912041V	AGLA021771	Anoplophora glabripennis	Eukaryotic translation initiation factor 3 subunit G
EOG0912041V	APLA001093	Agrilus planipennis	Eukaryotic translation initiation factor 3 subunit G
EOG0912041V	TC012449	Tribolium castaneum	Eukaryotic translation initiation factor 3 subunit G
EOG0912041V	LDEC021834	Leptinotarsa decemlineata	Eukaryotic translation initiation factor 3 subunit G
EOG0912041V	YQE_01659	Dendroctonus ponderosae	Eukaryotic translation initiation factor 3 subunit G
EOG0912041W	OTAU000117	Onthophagus taurus	Putative uncharacterized protein
EOG0912041W	AGLA016965	Anoplophora glabripennis	Putative uncharacterized protein
EOG0912041W	APLA014805	Agrilus planipennis	Putative uncharacterized protein
EOG0912041W	TC013117	Tribolium castaneum	Putative uncharacterized protein
EOG0912041W	LDEC005907	Leptinotarsa decemlineata	Putative uncharacterized protein
EOG0912041W	YQE_03679	Dendroctonus ponderosae	Putative uncharacterized protein
EOG0912041Y	OTAU016091	Onthophagus taurus	Putative uncharacterized protein
EOG0912041Y	AGLA002995	Anoplophora glabripennis	Putative uncharacterized protein
EOG0912041Y	APLA006352	Agrilus planipennis	Putative uncharacterized protein
EOG0912041Y	TC000094	Tribolium castaneum	Putative uncharacterized protein
EOG0912041Y	LDEC009653	Leptinotarsa decemlineata	Putative uncharacterized protein
EOG0912041Y	YQE_09163	Dendroctonus ponderosae	Putative uncharacterized protein
EOG0912041Z	OTAU001988	Onthophagus taurus	Putative uncharacterized protein
EOG0912041Z	AGLA002494	Anoplophora glabripennis	Putative uncharacterized protein
EOG0912041Z	APLA001463	Agrilus planipennis	Putative uncharacterized protein
EOG0912041Z	TC006779	Tribolium castaneum	Putative uncharacterized protein
EOG0912041Z	LDEC005859	Leptinotarsa decemlineata	Putative uncharacterized protein
EOG0912041Z	YQE_12006	Dendroctonus ponderosae	Putative uncharacterized protein
EOG09120422	OTAU006122	Onthophagus taurus	Putative uncharacterized protein
EOG09120422	AGLA008057	Anoplophora glabripennis	Putative uncharacterized protein
EOG09120422	APLA001243	Agrilus planipennis	Putative uncharacterized protein
EOG09120422	TC000627	Tribolium castaneum	Putative uncharacterized protein
EOG09120422	LDEC022707	Leptinotarsa decemlineata	Putative uncharacterized protein
EOG09120422	YQE_11133	Dendroctonus ponderosae	Putative uncharacterized protein
EOG09120423	OTAU001311	Onthophagus taurus	Putative uncharacterized protein
EOG09120423	AGLA000404	Anoplophora glabripennis	Putative uncharacterized protein
EOG09120423	APLA010459	Agrilus planipennis	Putative uncharacterized protein
EOG09120423	TC014222	Tribolium castaneum	Putative uncharacterized protein
EOG09120423	LDEC004702	Leptinotarsa decemlineata	Putative uncharacterized protein
EOG09120423	YQE_09233	Dendroctonus ponderosae	Putative uncharacterized protein
EOG09120426	OTAU016489	Onthophagus taurus	Runt
EOG09120426	AGLA005437	Anoplophora glabripennis	Runt
EOG09120426	APLA010938	Agrilus planipennis	Runt
EOG09120426	TC006542	Tribolium castaneum	Runt
EOG09120426	LDEC006645	Leptinotarsa decemlineata	Runt
EOG09120426	YQE_12214	Dendroctonus ponderosae	Runt
EOG09120427	OTAU008600	Onthophagus taurus	Putative uncharacterized protein
EOG09120427	AGLA000769	Anoplophora glabripennis	Putative uncharacterized protein
EOG09120427	APLA009231	Agrilus planipennis	Putative uncharacterized protein
EOG09120427	TC007193	Tribolium castaneum	Putative uncharacterized protein
EOG09120427	LDEC000612	Leptinotarsa decemlineata	Putative uncharacterized protein
EOG09120427	YQE_04060	Dendroctonus ponderosae	Putative uncharacterized protein
EOG09120428	OTAU002171	Onthophagus taurus	Putative uncharacterized protein
EOG09120428	AGLA011329	Anoplophora glabripennis	Putative uncharacterized protein
EOG09120428	APLA003200	Agrilus planipennis	Putative uncharacterized protein
EOG09120428	TC014291	Tribolium castaneum	Putative uncharacterized protein
EOG09120428	LDEC005739	Leptinotarsa decemlineata	Putative uncharacterized protein
EOG09120428	YQE_10011	Dendroctonus ponderosae	Putative uncharacterized protein
EOG0912042A	OTAU008448	Onthophagus taurus	Putative uncharacterized protein
EOG0912042A	AGLA004806	Anoplophora glabripennis	Putative uncharacterized protein
EOG0912042A	APLA005248	Agrilus planipennis	Putative uncharacterized protein
EOG0912042A	TC002814	Tribolium castaneum	Putative uncharacterized protein

EOG0912042A	LDEC015330	Leptinotarsa decemlineata	Putative uncharacterized protein
EOG0912042A	YQE_08285	Dendroctonus ponderosae	Putative uncharacterized protein
EOG0912042F	OTAU006923	Onthophagus taurus	Putative uncharacterized protein
EOG0912042F	AGLA000602	Anoplophora glabripennis	Putative uncharacterized protein
EOG0912042F	APLA002011	Agrilus planipennis	Putative uncharacterized protein
EOG0912042F	TC002569	Tribolium castaneum	Putative uncharacterized protein
EOG0912042F	LDEC015100	Leptinotarsa decemlineata	Putative uncharacterized protein
EOG0912042F	YQE_03706	Dendroctonus ponderosae	Putative uncharacterized protein
EOG0912042G	OTAU001690	Onthophagus taurus	Putative uncharacterized protein
EOG0912042G	AGLA010943	Anoplophora glabripennis	Putative uncharacterized protein
EOG0912042G	APLA004537	Agrilus planipennis	Putative uncharacterized protein
EOG0912042G	TC006236	Tribolium castaneum	Putative uncharacterized protein
EOG0912042G	LDEC016294	Leptinotarsa decemlineata	Putative uncharacterized protein
EOG0912042G	YQE_09440	Dendroctonus ponderosae	Putative uncharacterized protein
EOG0912042I	OTAU002858	Onthophagus taurus	Putative uncharacterized protein
EOG0912042I	AGLA000382	Anoplophora glabripennis	Putative uncharacterized protein
EOG0912042I	APLA006369	Agrilus planipennis	Putative uncharacterized protein
EOG0912042I	TC013604	Tribolium castaneum	Putative uncharacterized protein
EOG0912042I	LDEC004202	Leptinotarsa decemlineata	Putative uncharacterized protein
EOG0912042I	YQE_06711	Dendroctonus ponderosae	Putative uncharacterized protein
EOG0912042J	OTAU014184	Onthophagus taurus	Putative uncharacterized protein
EOG0912042J	AGLA012749	Anoplophora glabripennis	Putative uncharacterized protein
EOG0912042J	APLA009126	Agrilus planipennis	Putative uncharacterized protein
EOG0912042J	TC006114	Tribolium castaneum	Putative uncharacterized protein
EOG0912042J	LDEC001861	Leptinotarsa decemlineata	Putative uncharacterized protein
EOG0912042J	YQE_10138	Dendroctonus ponderosae	Putative uncharacterized protein
EOG0912042K	OTAU011185	Onthophagus taurus	Putative uncharacterized protein
EOG0912042K	AGLA014191	Anoplophora glabripennis	Putative uncharacterized protein
EOG0912042K	APLA006608	Agrilus planipennis	Putative uncharacterized protein
EOG0912042K	TC008092	Tribolium castaneum	Putative uncharacterized protein
EOG0912042K	LDEC007116	Leptinotarsa decemlineata	Putative uncharacterized protein
EOG0912042K	YQE_03190	Dendroctonus ponderosae	Putative uncharacterized protein
EOG0912042Q	OTAU016537	Onthophagus taurus	Putative uncharacterized protein
EOG0912042Q	AGLA013470	Anoplophora glabripennis	Putative uncharacterized protein
EOG0912042Q	APLA010030	Agrilus planipennis	Putative uncharacterized protein
EOG0912042Q	TC015364	Tribolium castaneum	Putative uncharacterized protein
EOG0912042Q	LDEC007935	Leptinotarsa decemlineata	Putative uncharacterized protein
EOG0912042Q	YQE_08387	Dendroctonus ponderosae	Putative uncharacterized protein
EOG0912042R	OTAU013517	Onthophagus taurus	Putative uncharacterized protein
EOG0912042R	AGLA017472	Anoplophora glabripennis	Putative uncharacterized protein
EOG0912042R	APLA000874	Agrilus planipennis	Putative uncharacterized protein
EOG0912042R	TC016287	Tribolium castaneum	Putative uncharacterized protein
EOG0912042R	LDEC018372	Leptinotarsa decemlineata	Putative uncharacterized protein
EOG0912042R	YQE_08155	Dendroctonus ponderosae	Putative uncharacterized protein
EOG0912042W	OTAU002647	Onthophagus taurus	Putative uncharacterized protein
EOG0912042W	AGLA010794	Anoplophora glabripennis	Putative uncharacterized protein
EOG0912042W	APLA010729	Agrilus planipennis	Putative uncharacterized protein
EOG0912042W	TC013413	Tribolium castaneum	Putative uncharacterized protein
EOG0912042W	LDEC003482	Leptinotarsa decemlineata	Putative uncharacterized protein
EOG0912042W	YQE_06166	Dendroctonus ponderosae	Putative uncharacterized protein
EOG0912042Y	OTAU005407	Onthophagus taurus	Putative uncharacterized protein
EOG0912042Y	AGLA000281	Anoplophora glabripennis	Putative uncharacterized protein
EOG0912042Y	APLA006366	Agrilus planipennis	Putative uncharacterized protein
EOG0912042Y	TC001443	Tribolium castaneum	Putative uncharacterized protein
EOG0912042Y	LDEC001015	Leptinotarsa decemlineata	Putative uncharacterized protein
EOG0912042Y	YQE_06677	Dendroctonus ponderosae	Putative uncharacterized protein
EOG0912042Z	OTAU002879	Onthophagus taurus	Putative uncharacterized protein
EOG0912042Z	AGLA009184	Anoplophora glabripennis	Putative uncharacterized protein
EOG0912042Z	APLA014830	Agrilus planipennis	Putative uncharacterized protein
EOG0912042Z	TC009145	Tribolium castaneum	Putative uncharacterized protein
EOG0912042Z	LDEC015719	Leptinotarsa decemlineata	Putative uncharacterized protein
EOG0912042Z	YQE_03904	Dendroctonus ponderosae	Putative uncharacterized protein
EOG09120430	OTAU003661	Onthophagus taurus	Croquemort
EOG09120430	AGLA000789	Anoplophora glabripennis	Croquemort
EOG09120430	APLA006566	Agrilus planipennis	Croquemort
EOG09120430	TC007247	Tribolium castaneum	Croquemort
EOG09120430	LDEC004394	Leptinotarsa decemlineata	Croquemort

EOG09120430	YQE_04900	Dendroctonus ponderosae	Croquemort
EOG09120431	OTAU009696	Onthophagus taurus	Putative uncharacterized protein
EOG09120431	AGLA000470	Anoplophora glabripennis	Putative uncharacterized protein
EOG09120431	APLA000219	Agrilus planipennis	Putative uncharacterized protein
EOG09120431	TC010229	Tribolium castaneum	Putative uncharacterized protein
EOG09120431	LDEC000956	Leptinotarsa decemlineata	Putative uncharacterized protein
EOG09120431	YQE_07975	Dendroctonus ponderosae	Putative uncharacterized protein
EOG09120435	OTAU000097	Onthophagus taurus	Putative uncharacterized protein
EOG09120435	AGLA014429	Anoplophora glabripennis	Putative uncharacterized protein
EOG09120435	APLA012882	Agrilus planipennis	Putative uncharacterized protein
EOG09120435	TC010901	Tribolium castaneum	Putative uncharacterized protein
EOG09120435	LDEC008707	Leptinotarsa decemlineata	Putative uncharacterized protein
EOG09120435	YQE_04004	Dendroctonus ponderosae	Putative uncharacterized protein
EOG0912043A	OTAU009728	Onthophagus taurus	None
EOG0912043A	AGLA003109	Anoplophora glabripennis	None
EOG0912043A	APLA014075	Agrilus planipennis	None
EOG0912043A	TC033166	Tribolium castaneum	None
EOG0912043A	LDEC010320	Leptinotarsa decemlineata	None
EOG0912043A	YQE_11551	Dendroctonus ponderosae	None
EOG0912043C	OTAU009341	Onthophagus taurus	Putative uncharacterized protein
EOG0912043C	AGLA012609	Anoplophora glabripennis	Putative uncharacterized protein
EOG0912043C	APLA012895	Agrilus planipennis	Putative uncharacterized protein
EOG0912043C	TC006385	Tribolium castaneum	Putative uncharacterized protein
EOG0912043C	LDEC013809	Leptinotarsa decemlineata	Putative uncharacterized protein
EOG0912043C	YQE_08254	Dendroctonus ponderosae	Putative uncharacterized protein
EOG0912043E	OTAU005101	Onthophagus taurus	Putative uncharacterized protein
EOG0912043E	AGLA020401	Anoplophora glabripennis	Putative uncharacterized protein
EOG0912043E	APLA005741	Agrilus planipennis	Putative uncharacterized protein
EOG0912043E	TC005370	Tribolium castaneum	Putative uncharacterized protein
EOG0912043E	LDEC019100	Leptinotarsa decemlineata	Putative uncharacterized protein
EOG0912043E	YQE_07478	Dendroctonus ponderosae	Putative uncharacterized protein
EOG0912043F	OTAU005740	Onthophagus taurus	Putative uncharacterized protein
EOG0912043F	AGLA016530	Anoplophora glabripennis	Putative uncharacterized protein
EOG0912043F	APLA006088	Agrilus planipennis	Putative uncharacterized protein
EOG0912043F	TC002951	Tribolium castaneum	Putative uncharacterized protein
EOG0912043F	LDEC005914	Leptinotarsa decemlineata	Putative uncharacterized protein
EOG0912043F	YQE_12309	Dendroctonus ponderosae	Putative uncharacterized protein
EOG0912043H	OTAU008329	Onthophagus taurus	Putative uncharacterized protein
EOG0912043H	AGLA006156	Anoplophora glabripennis	Putative uncharacterized protein
EOG0912043H	APLA006175	Agrilus planipennis	Putative uncharacterized protein
EOG0912043H	TC000750	Tribolium castaneum	Putative uncharacterized protein
EOG0912043H	LDEC007364	Leptinotarsa decemlineata	Putative uncharacterized protein
EOG0912043H	YQE_03396	Dendroctonus ponderosae	Putative uncharacterized protein
EOG0912043J	OTAU015146	Onthophagus taurus	Putative uncharacterized protein
EOG0912043J	AGLA001579	Anoplophora glabripennis	Putative uncharacterized protein
EOG0912043J	APLA008226	Agrilus planipennis	Putative uncharacterized protein
EOG0912043J	TC001525	Tribolium castaneum	Putative uncharacterized protein
EOG0912043J	LDEC007763	Leptinotarsa decemlineata	Putative uncharacterized protein
EOG0912043J	YQE_11916	Dendroctonus ponderosae	Putative uncharacterized protein
EOG0912043M	OTAU002933	Onthophagus taurus	Proteasome subunit alpha type
EOG0912043M	AGLA016485	Anoplophora glabripennis	Proteasome subunit alpha type
EOG0912043M	APLA010791	Agrilus planipennis	Proteasome subunit alpha type
EOG0912043M	TC008671	Tribolium castaneum	Proteasome subunit alpha type
EOG0912043M	LDEC010714	Leptinotarsa decemlineata	Proteasome subunit alpha type
EOG0912043M	YQE_05420	Dendroctonus ponderosae	Proteasome subunit alpha type
EOG0912043Q	OTAU001246	Onthophagus taurus	Putative uncharacterized protein
EOG0912043Q	AGLA002230	Anoplophora glabripennis	Putative uncharacterized protein
EOG0912043Q	APLA003664	Agrilus planipennis	Putative uncharacterized protein
EOG0912043Q	TC014243	Tribolium castaneum	Putative uncharacterized protein
EOG0912043Q	LDEC018596	Leptinotarsa decemlineata	Putative uncharacterized protein
EOG0912043Q	YQE_09149	Dendroctonus ponderosae	Putative uncharacterized protein
EOG0912043T	OTAU009314	Onthophagus taurus	Putative uncharacterized protein
EOG0912043T	AGLA013785	Anoplophora glabripennis	Putative uncharacterized protein
EOG0912043T	APLA011260	Agrilus planipennis	Putative uncharacterized protein
EOG0912043T	TC009968	Tribolium castaneum	Putative uncharacterized protein
EOG0912043T	LDEC014263	Leptinotarsa decemlineata	Putative uncharacterized protein
EOG0912043T	YQE_05712	Dendroctonus ponderosae	Putative uncharacterized protein

EOG0912043V	OTAU015119	Onthophagus taurus	Putative uncharacterized protein
EOG0912043V	AGLA003667	Anoplophora glabripennis	Putative uncharacterized protein
EOG0912043V	APLA008704	Agrilus planipennis	Putative uncharacterized protein
EOG0912043V	TC030605	Tribolium castaneum	Putative uncharacterized protein
EOG0912043V	LDEC009795	Leptinotarsa decemlineata	Putative uncharacterized protein
EOG0912043V	YQE_05195	Dendroctonus ponderosae	Putative uncharacterized protein
EOG0912043Z	OTAU016006	Onthophagus taurus	Putative uncharacterized protein
EOG0912043Z	AGLA015580	Anoplophora glabripennis	Putative uncharacterized protein
EOG0912043Z	APLA005902	Agrilus planipennis	Putative uncharacterized protein
EOG0912043Z	TC015635	Tribolium castaneum	Putative uncharacterized protein
EOG0912043Z	LDEC003632	Leptinotarsa decemlineata	Putative uncharacterized protein
EOG0912043Z	YQE_02134	Dendroctonus ponderosae	Putative uncharacterized protein
EOG09120441	OTAU003689	Onthophagus taurus	DNA binding
EOG09120441	AGLA013209	Anoplophora glabripennis	DNA binding
EOG09120441	APLA005374	Agrilus planipennis	DNA binding
EOG09120441	TC032832	Tribolium castaneum	DNA binding
EOG09120441	LDEC009603	Leptinotarsa decemlineata	DNA binding
EOG09120441	YQE_06850	Dendroctonus ponderosae	DNA binding
EOG09120442	OTAU006320	Onthophagus taurus	calcium ion binding
EOG09120442	AGLA013293	Anoplophora glabripennis	calcium ion binding
EOG09120442	APLA008958	Agrilus planipennis	calcium ion binding
EOG09120442	TC032018	Tribolium castaneum	calcium ion binding
EOG09120442	LDEC022515	Leptinotarsa decemlineata	calcium ion binding
EOG09120442	YQE_09043	Dendroctonus ponderosae	calcium ion binding
EOG09120444	OTAU002340	Onthophagus taurus	Putative uncharacterized protein
EOG09120444	AGLA003114	Anoplophora glabripennis	Putative uncharacterized protein
EOG09120444	APLA005654	Agrilus planipennis	Putative uncharacterized protein
EOG09120444	TC008460	Tribolium castaneum	Putative uncharacterized protein
EOG09120444	LDEC011553	Leptinotarsa decemlineata	Putative uncharacterized protein
EOG09120444	YQE_12568	Dendroctonus ponderosae	Putative uncharacterized protein
EOG09120448	OTAU001491	Onthophagus taurus	Putative uncharacterized protein
EOG09120448	AGLA004545	Anoplophora glabripennis	Putative uncharacterized protein
EOG09120448	APLA010048	Agrilus planipennis	Putative uncharacterized protein
EOG09120448	TC004690	Tribolium castaneum	Putative uncharacterized protein
EOG09120448	LDEC023984	Leptinotarsa decemlineata	Putative uncharacterized protein
EOG09120448	YQE_03288	Dendroctonus ponderosae	Putative uncharacterized protein
EOG09120449	OTAU001732	Onthophagus taurus	Putative uncharacterized protein
EOG09120449	AGLA008639	Anoplophora glabripennis	Putative uncharacterized protein
EOG09120449	APLA012311	Agrilus planipennis	Putative uncharacterized protein
EOG09120449	TC030611	Tribolium castaneum	Putative uncharacterized protein
EOG09120449	LDEC003020	Leptinotarsa decemlineata	Putative uncharacterized protein
EOG09120449	YQE_05551	Dendroctonus ponderosae	Putative uncharacterized protein
EOG0912044B	OTAU002197	Onthophagus taurus	Putative uncharacterized protein
EOG0912044B	AGLA020399	Anoplophora glabripennis	Putative uncharacterized protein
EOG0912044B	APLA000127	Agrilus planipennis	Putative uncharacterized protein
EOG0912044B	TC003487	Tribolium castaneum	Putative uncharacterized protein
EOG0912044B	LDEC005913	Leptinotarsa decemlineata	Putative uncharacterized protein
EOG0912044B	YQE_03773	Dendroctonus ponderosae	Putative uncharacterized protein
EOG0912044G	OTAU014659	Onthophagus taurus	Putative uncharacterized protein
EOG0912044G	AGLA012710	Anoplophora glabripennis	Putative uncharacterized protein
EOG0912044G	APLA012004	Agrilus planipennis	Putative uncharacterized protein
EOG0912044G	TC002341	Tribolium castaneum	Putative uncharacterized protein
EOG0912044G	LDEC019917	Leptinotarsa decemlineata	Putative uncharacterized protein
EOG0912044G	YQE_07833	Dendroctonus ponderosae	Putative uncharacterized protein
EOG0912044J	OTAU000080	Onthophagus taurus	Elongation factor Ts
EOG0912044J	AGLA014401	Anoplophora glabripennis	Elongation factor Ts
EOG0912044J	APLA009000	Agrilus planipennis	Elongation factor Ts
EOG0912044J	TC013107	Tribolium castaneum	Elongation factor Ts
EOG0912044J	LDEC006288	Leptinotarsa decemlineata	Elongation factor Ts
EOG0912044J	YQE_08593	Dendroctonus ponderosae	Elongation factor Ts
EOG0912044K	OTAU000532	Onthophagus taurus	Putative uncharacterized protein
EOG0912044K	AGLA009471	Anoplophora glabripennis	Putative uncharacterized protein
EOG0912044K	APLA002029	Agrilus planipennis	Putative uncharacterized protein
EOG0912044K	TC003526	Tribolium castaneum	Putative uncharacterized protein
EOG0912044K	LDEC002241	Leptinotarsa decemlineata	Putative uncharacterized protein
EOG0912044K	YQE_11728	Dendroctonus ponderosae	Putative uncharacterized protein
EOG0912044N	OTAU008205	Onthophagus taurus	Putative uncharacterized protein

EOG0912044N	AGLA008421	Anoplophora glabripennis	Putative uncharacterized protein
EOG0912044N	APLA006178	Agrilus planipennis	Putative uncharacterized protein
EOG0912044N	TC015170	Tribolium castaneum	Putative uncharacterized protein
EOG0912044N	LDEC001732	Leptinotarsa decemlineata	Putative uncharacterized protein
EOG0912044N	YQE_09310	Dendroctonus ponderosae	Putative uncharacterized protein
EOG0912044P	OTAU009873	Onthophagus taurus	Putative uncharacterized protein
EOG0912044P	AGLA000145	Anoplophora glabripennis	Putative uncharacterized protein
EOG0912044P	APLA014593	Agrilus planipennis	Putative uncharacterized protein
EOG0912044P	TC000161	Tribolium castaneum	Putative uncharacterized protein
EOG0912044P	LDEC016690	Leptinotarsa decemlineata	Putative uncharacterized protein
EOG0912044P	YQE_07056	Dendroctonus ponderosae	Putative uncharacterized protein
EOG0912044Q	OTAU005911	Onthophagus taurus	Putative uncharacterized protein
EOG0912044Q	AGLA017324	Anoplophora glabripennis	Putative uncharacterized protein
EOG0912044Q	APLA004863	Agrilus planipennis	Putative uncharacterized protein
EOG0912044Q	TC014336	Tribolium castaneum	Putative uncharacterized protein
EOG0912044Q	LDEC003464	Leptinotarsa decemlineata	Putative uncharacterized protein
EOG0912044Q	YQE_08993	Dendroctonus ponderosae	Putative uncharacterized protein
EOG0912044T	OTAU000527	Onthophagus taurus	Putative uncharacterized protein
EOG0912044T	AGLA004242	Anoplophora glabripennis	Putative uncharacterized protein
EOG0912044T	APLA000161	Agrilus planipennis	Putative uncharacterized protein
EOG0912044T	TC003520	Tribolium castaneum	Putative uncharacterized protein
EOG0912044T	LDEC008218	Leptinotarsa decemlineata	Putative uncharacterized protein
EOG0912044T	YQE_11089	Dendroctonus ponderosae	Putative uncharacterized protein
EOG0912044U	OTAU007353	Onthophagus taurus	Putative uncharacterized protein
EOG0912044U	AGLA011904	Anoplophora glabripennis	Putative uncharacterized protein
EOG0912044U	APLA001404	Agrilus planipennis	Putative uncharacterized protein
EOG0912044U	TC000167	Tribolium castaneum	Putative uncharacterized protein
EOG0912044U	LDEC011568	Leptinotarsa decemlineata	Putative uncharacterized protein
EOG0912044U	YQE_12503	Dendroctonus ponderosae	Putative uncharacterized protein
EOG0912044X	OTAU000633	Onthophagus taurus	Putative uncharacterized protein
EOG0912044X	AGLA020568	Anoplophora glabripennis	Putative uncharacterized protein
EOG0912044X	APLA014336	Agrilus planipennis	Putative uncharacterized protein
EOG0912044X	TC012325	Tribolium castaneum	Putative uncharacterized protein
EOG0912044X	LDEC018233	Leptinotarsa decemlineata	Putative uncharacterized protein
EOG0912044X	YQE_12811	Dendroctonus ponderosae	Putative uncharacterized protein
EOG09120450	OTAU008124	Onthophagus taurus	Putative uncharacterized protein
EOG09120450	AGLA005078	Anoplophora glabripennis	Putative uncharacterized protein
EOG09120450	APLA005500	Agrilus planipennis	Putative uncharacterized protein
EOG09120450	TC011317	Tribolium castaneum	Putative uncharacterized protein
EOG09120450	LDEC011377	Leptinotarsa decemlineata	Putative uncharacterized protein
EOG09120450	YQE_09599	Dendroctonus ponderosae	Putative uncharacterized protein
EOG09120452	OTAU006878	Onthophagus taurus	Putative uncharacterized protein
EOG09120452	AGLA011593	Anoplophora glabripennis	Putative uncharacterized protein
EOG09120452	APLA006001	Agrilus planipennis	Putative uncharacterized protein
EOG09120452	TC009171	Tribolium castaneum	Putative uncharacterized protein
EOG09120452	LDEC004465	Leptinotarsa decemlineata	Putative uncharacterized protein
EOG09120452	YQE_10557	Dendroctonus ponderosae	Putative uncharacterized protein
EOG09120457	OTAU007731	Onthophagus taurus	Putative uncharacterized protein
EOG09120457	AGLA016073	Anoplophora glabripennis	Putative uncharacterized protein
EOG09120457	APLA009732	Agrilus planipennis	Putative uncharacterized protein
EOG09120457	TC010419	Tribolium castaneum	Putative uncharacterized protein
EOG09120457	LDEC015112	Leptinotarsa decemlineata	Putative uncharacterized protein
EOG09120457	YQE_08302	Dendroctonus ponderosae	Putative uncharacterized protein
EOG09120458	OTAU013337	Onthophagus taurus	Putative uncharacterized protein
EOG09120458	AGLA017923	Anoplophora glabripennis	Putative uncharacterized protein
EOG09120458	APLA012284	Agrilus planipennis	Putative uncharacterized protein
EOG09120458	TC015914	Tribolium castaneum	Putative uncharacterized protein
EOG09120458	LDEC015378	Leptinotarsa decemlineata	Putative uncharacterized protein
EOG09120458	YQE_05319	Dendroctonus ponderosae	Putative uncharacterized protein
EOG09120459	OTAU006373	Onthophagus taurus	Putative uncharacterized protein
EOG09120459	AGLA006638	Anoplophora glabripennis	Putative uncharacterized protein
EOG09120459	APLA007699	Agrilus planipennis	Putative uncharacterized protein
EOG09120459	TC002852	Tribolium castaneum	Putative uncharacterized protein
EOG09120459	LDEC004976	Leptinotarsa decemlineata	Putative uncharacterized protein
EOG09120459	YQE_07897	Dendroctonus ponderosae	Putative uncharacterized protein
EOG0912045A	OTAU000155	Onthophagus taurus	Putative uncharacterized protein
EOG0912045A	AGLA006017	Anoplophora glabripennis	Putative uncharacterized protein

EOG0912045A	APLA002733	Agrilus planipennis	Putative uncharacterized protein
EOG0912045A	TC014627	Tribolium castaneum	Putative uncharacterized protein
EOG0912045A	LDEC004108	Leptinotarsa decemlineata	Putative uncharacterized protein
EOG0912045A	YQE_04000	Dendroctonus ponderosae	Putative uncharacterized protein
EOG0912045B	OTAU011714	Onthophagus taurus	Putative uncharacterized protein
EOG0912045B	AGLA005895	Anoplophora glabripennis	Putative uncharacterized protein
EOG0912045B	APLA000687	Agrilus planipennis	Putative uncharacterized protein
EOG0912045B	TC015679	Tribolium castaneum	Putative uncharacterized protein
EOG0912045B	LDEC008289	Leptinotarsa decemlineata	Putative uncharacterized protein
EOG0912045B	YQE_09408	Dendroctonus ponderosae	Putative uncharacterized protein
EOG0912045D	OTAU012611	Onthophagus taurus	Putative uncharacterized protein
EOG0912045D	AGLA020461	Anoplophora glabripennis	Putative uncharacterized protein
EOG0912045D	APLA007854	Agrilus planipennis	Putative uncharacterized protein
EOG0912045D	TC011351	Tribolium castaneum	Putative uncharacterized protein
EOG0912045D	LDEC005274	Leptinotarsa decemlineata	Putative uncharacterized protein
EOG0912045D	YQE_04612	Dendroctonus ponderosae	Putative uncharacterized protein
EOG0912045E	OTAU001393	Onthophagus taurus	Putative uncharacterized protein
EOG0912045E	AGLA012993	Anoplophora glabripennis	Putative uncharacterized protein
EOG0912045E	APLA003567	Agrilus planipennis	Putative uncharacterized protein
EOG0912045E	TC014172	Tribolium castaneum	Putative uncharacterized protein
EOG0912045E	LDEC017387	Leptinotarsa decemlineata	Putative uncharacterized protein
EOG0912045E	YQE_05970	Dendroctonus ponderosae	Putative uncharacterized protein
EOG0912045I	OTAU011357	Onthophagus taurus	Abnormal chemosensory jump 6
EOG0912045I	AGLA008924	Anoplophora glabripennis	Abnormal chemosensory jump 6
EOG0912045I	APLA013757	Agrilus planipennis	Abnormal chemosensory jump 6
EOG0912045I	TC003196	Tribolium castaneum	Abnormal chemosensory jump 6
EOG0912045I	LDEC019440	Leptinotarsa decemlineata	Abnormal chemosensory jump 6
EOG0912045I	YQE_09839	Dendroctonus ponderosae	Abnormal chemosensory jump 6
EOG0912045J	OTAU000789	Onthophagus taurus	Putative uncharacterized protein
EOG0912045J	AGLA011168	Anoplophora glabripennis	Putative uncharacterized protein
EOG0912045J	APLA013598	Agrilus planipennis	Putative uncharacterized protein
EOG0912045J	TC011780	Tribolium castaneum	Putative uncharacterized protein
EOG0912045J	LDEC008553	Leptinotarsa decemlineata	Putative uncharacterized protein
EOG0912045J	YQE_07987	Dendroctonus ponderosae	Putative uncharacterized protein
EOG0912045T	OTAU001352	Onthophagus taurus	Putative uncharacterized protein
EOG0912045T	AGLA007611	Anoplophora glabripennis	Putative uncharacterized protein
EOG0912045T	APLA003688	Agrilus planipennis	Putative uncharacterized protein
EOG0912045T	TC001758	Tribolium castaneum	Putative uncharacterized protein
EOG0912045T	LDEC006808	Leptinotarsa decemlineata	Putative uncharacterized protein
EOG0912045T	YQE_09135	Dendroctonus ponderosae	Putative uncharacterized protein
EOG0912045U	OTAU002374	Onthophagus taurus	Putative uncharacterized protein
EOG0912045U	AGLA000849	Anoplophora glabripennis	Putative uncharacterized protein
EOG0912045U	APLA004239	Agrilus planipennis	Putative uncharacterized protein
EOG0912045U	TC008355	Tribolium castaneum	Putative uncharacterized protein
EOG0912045U	LDEC016682	Leptinotarsa decemlineata	Putative uncharacterized protein
EOG0912045U	YQE_04738	Dendroctonus ponderosae	Putative uncharacterized protein
EOG0912045V	OTAU002583	Onthophagus taurus	Putative uncharacterized protein
EOG0912045V	AGLA009548	Anoplophora glabripennis	Putative uncharacterized protein
EOG0912045V	APLA011086	Agrilus planipennis	Putative uncharacterized protein
EOG0912045V	TC013355	Tribolium castaneum	Putative uncharacterized protein
EOG0912045V	LDEC002633	Leptinotarsa decemlineata	Putative uncharacterized protein
EOG0912045V	YQE_10450	Dendroctonus ponderosae	Putative uncharacterized protein
EOG0912045W	OTAU004997	Onthophagus taurus	Putative uncharacterized protein
EOG0912045W	AGLA010154	Anoplophora glabripennis	Putative uncharacterized protein
EOG0912045W	APLA006337	Agrilus planipennis	Putative uncharacterized protein
EOG0912045W	TC011383	Tribolium castaneum	Putative uncharacterized protein
EOG0912045W	LDEC019003	Leptinotarsa decemlineata	Putative uncharacterized protein
EOG0912045W	YQE_03579	Dendroctonus ponderosae	Putative uncharacterized protein
EOG09120460	OTAU011804	Onthophagus taurus	Putative uncharacterized protein
EOG09120460	AGLA006509	Anoplophora glabripennis	Putative uncharacterized protein
EOG09120460	APLA011114	Agrilus planipennis	Putative uncharacterized protein
EOG09120460	TC015933	Tribolium castaneum	Putative uncharacterized protein
EOG09120460	LDEC020596	Leptinotarsa decemlineata	Putative uncharacterized protein
EOG09120460	YQE_12460	Dendroctonus ponderosae	Putative uncharacterized protein
EOG09120463	OTAU012072	Onthophagus taurus	Putative uncharacterized protein
EOG09120463	AGLA003547	Anoplophora glabripennis	Putative uncharacterized protein
EOG09120463	APLA004103	Agrilus planipennis	Putative uncharacterized protein

EOG09120463	TC003836	Tribolium castaneum	Putative uncharacterized protein
EOG09120463	LDEC016972	Leptinotarsa decemlineata	Putative uncharacterized protein
EOG09120463	YQE_10206	Dendroctonus ponderosae	Putative uncharacterized protein
EOG09120467	OTAU000825	Onthophagus taurus	Putative uncharacterized protein
EOG09120467	AGLA013448	Anoplophora glabripennis	Putative uncharacterized protein
EOG09120467	APLA003156	Agrilus planipennis	Putative uncharacterized protein
EOG09120467	TC013754	Tribolium castaneum	Putative uncharacterized protein
EOG09120467	LDEC012185	Leptinotarsa decemlineata	Putative uncharacterized protein
EOG09120467	YQE_04441	Dendroctonus ponderosae	Putative uncharacterized protein
EOG09120468	OTAU001647	Onthophagus taurus	Putative uncharacterized protein
EOG09120468	AGLA007318	Anoplophora glabripennis	Putative uncharacterized protein
EOG09120468	APLA001204	Agrilus planipennis	Putative uncharacterized protein
EOG09120468	TC006308	Tribolium castaneum	Putative uncharacterized protein
EOG09120468	LDEC023691	Leptinotarsa decemlineata	Putative uncharacterized protein
EOG09120468	YQE_12199	Dendroctonus ponderosae	Putative uncharacterized protein
EOG0912046B	OTAU012771	Onthophagus taurus	Putative uncharacterized protein
EOG0912046B	AGLA001373	Anoplophora glabripennis	Putative uncharacterized protein
EOG0912046B	APLA005032	Agrilus planipennis	Putative uncharacterized protein
EOG0912046B	TC001329	Tribolium castaneum	Putative uncharacterized protein
EOG0912046B	LDEC007298	Leptinotarsa decemlineata	Putative uncharacterized protein
EOG0912046B	YQE_03770	Dendroctonus ponderosae	Putative uncharacterized protein
EOG0912046K	OTAU002788	Onthophagus taurus	zinc ion binding
EOG0912046K	AGLA000424	Anoplophora glabripennis	zinc ion binding
EOG0912046K	APLA010665	Agrilus planipennis	zinc ion binding
EOG0912046K	TC031557	Tribolium castaneum	zinc ion binding
EOG0912046K	LDEC018119	Leptinotarsa decemlineata	zinc ion binding
EOG0912046K	YQE_02395	Dendroctonus ponderosae	zinc ion binding
EOG0912046L	OTAU002928	Onthophagus taurus	Protein Wnt
EOG0912046L	AGLA016179	Anoplophora glabripennis	Protein Wnt
EOG0912046L	APLA007530	Agrilus planipennis	Protein Wnt
EOG0912046L	TC010155	Tribolium castaneum	Protein Wnt
EOG0912046L	LDEC010710	Leptinotarsa decemlineata	Protein Wnt
EOG0912046L	YQE_05427	Dendroctonus ponderosae	Protein Wnt
EOG0912046M	OTAU005828	Onthophagus taurus	"Cytochrome b-c1 complex subunit Rieske, mitochondrial "
EOG0912046M	AGLA013892	Anoplophora glabripennis	"Cytochrome b-c1 complex subunit Rieske, mitochondrial "
EOG0912046M	APLA000133	Agrilus planipennis	"Cytochrome b-c1 complex subunit Rieske, mitochondrial "
EOG0912046M	TC000043	Tribolium castaneum	"Cytochrome b-c1 complex subunit Rieske, mitochondrial "
EOG0912046M	LDEC002711	Leptinotarsa decemlineata	"Cytochrome b-c1 complex subunit Rieske, mitochondrial "
EOG0912046M	YQE_06758	Dendroctonus ponderosae	"Cytochrome b-c1 complex subunit Rieske, mitochondrial "
EOG0912046Q	OTAU009676	Onthophagus taurus	Putative uncharacterized protein
EOG0912046Q	AGLA001947	Anoplophora glabripennis	Putative uncharacterized protein
EOG0912046Q	APLA008660	Agrilus planipennis	Putative uncharacterized protein
EOG0912046Q	TC012793	Tribolium castaneum	Putative uncharacterized protein
EOG0912046Q	LDEC008349	Leptinotarsa decemlineata	Putative uncharacterized protein
EOG0912046Q	YQE_04346	Dendroctonus ponderosae	Putative uncharacterized protein
EOG0912046S	OTAU012422	Onthophagus taurus	Putative uncharacterized protein
EOG0912046S	AGLA015044	Anoplophora glabripennis	Putative uncharacterized protein
EOG0912046S	APLA007071	Agrilus planipennis	Putative uncharacterized protein
EOG0912046S	TC009754	Tribolium castaneum	Putative uncharacterized protein
EOG0912046S	LDEC020669	Leptinotarsa decemlineata	Putative uncharacterized protein
EOG0912046S	YQE_02587	Dendroctonus ponderosae	Putative uncharacterized protein
EOG0912046T	OTAU004598	Onthophagus taurus	Putative uncharacterized protein
EOG0912046T	AGLA003069	Anoplophora glabripennis	Putative uncharacterized protein
EOG0912046T	APLA004493	Agrilus planipennis	Putative uncharacterized protein
EOG0912046T	TC008023	Tribolium castaneum	Putative uncharacterized protein
EOG0912046T	LDEC008483	Leptinotarsa decemlineata	Putative uncharacterized protein
EOG0912046T	YQE_08970	Dendroctonus ponderosae	Putative uncharacterized protein
EOG0912046U	OTAU007492	Onthophagus taurus	Putative uncharacterized protein
EOG0912046U	AGLA003333	Anoplophora glabripennis	Putative uncharacterized protein
EOG0912046U	APLA014377	Agrilus planipennis	Putative uncharacterized protein
EOG0912046U	TC015001	Tribolium castaneum	Putative uncharacterized protein

EOG0912046U	LDEC007886	Leptinotarsa decemlineata	Putative uncharacterized protein
EOG0912046U	YQE_12944	Dendroctonus ponderosae	Putative uncharacterized protein
EOG0912046V	OTAU005029	Onthophagus taurus	Putative uncharacterized protein
EOG0912046V	AGLA009065	Anoplophora glabripennis	Putative uncharacterized protein
EOG0912046V	APLA011144	Agrilus planipennis	Putative uncharacterized protein
EOG0912046V	TC004969	Tribolium castaneum	Putative uncharacterized protein
EOG0912046V	LDEC018939	Leptinotarsa decemlineata	Putative uncharacterized protein
EOG0912046V	YQE_11619	Dendroctonus ponderosae	Putative uncharacterized protein
EOG0912046Y	OTAU006391	Onthophagus taurus	Putative uncharacterized protein
EOG0912046Y	AGLA016560	Anoplophora glabripennis	Putative uncharacterized protein
EOG0912046Y	APLA004210	Agrilus planipennis	Putative uncharacterized protein
EOG0912046Y	TC007833	Tribolium castaneum	Putative uncharacterized protein
EOG0912046Y	LDEC008496	Leptinotarsa decemlineata	Putative uncharacterized protein
EOG0912046Y	YQE_00957	Dendroctonus ponderosae	Putative uncharacterized protein
EOG09120472	OTAU001768	Onthophagus taurus	Putative uncharacterized protein
EOG09120472	AGLA010191	Anoplophora glabripennis	Putative uncharacterized protein
EOG09120472	APLA009861	Agrilus planipennis	Putative uncharacterized protein
EOG09120472	TC006792	Tribolium castaneum	Putative uncharacterized protein
EOG09120472	LDEC006776	Leptinotarsa decemlineata	Putative uncharacterized protein
EOG09120472	YQE_05657	Dendroctonus ponderosae	Putative uncharacterized protein
EOG09120474	OTAU005871	Onthophagus taurus	Putative uncharacterized protein
EOG09120474	AGLA018548	Anoplophora glabripennis	Putative uncharacterized protein
EOG09120474	APLA003588	Agrilus planipennis	Putative uncharacterized protein
EOG09120474	TC013217	Tribolium castaneum	Putative uncharacterized protein
EOG09120474	LDEC015560	Leptinotarsa decemlineata	Putative uncharacterized protein
EOG09120474	YQE_11387	Dendroctonus ponderosae	Putative uncharacterized protein
EOG09120478	OTAU013945	Onthophagus taurus	Myoglianin
EOG09120478	AGLA016043	Anoplophora glabripennis	Myoglianin
EOG09120478	APLA000652	Agrilus planipennis	Myoglianin
EOG09120478	TC015805	Tribolium castaneum	Myoglianin
EOG09120478	LDEC008520	Leptinotarsa decemlineata	Myoglianin
EOG09120478	YQE_07329	Dendroctonus ponderosae	Myoglianin
EOG0912047A	OTAU015173	Onthophagus taurus	Putative uncharacterized protein
EOG0912047A	AGLA013469	Anoplophora glabripennis	Putative uncharacterized protein
EOG0912047A	APLA002576	Agrilus planipennis	Putative uncharacterized protein
EOG0912047A	TC015384	Tribolium castaneum	Putative uncharacterized protein
EOG0912047A	LDEC007934	Leptinotarsa decemlineata	Putative uncharacterized protein
EOG0912047A	YQE_08345	Dendroctonus ponderosae	Putative uncharacterized protein
EOG0912047B	OTAU002555	Onthophagus taurus	Putative uncharacterized protein
EOG0912047B	AGLA011929	Anoplophora glabripennis	Putative uncharacterized protein
EOG0912047B	APLA014466	Agrilus planipennis	Putative uncharacterized protein
EOG0912047B	TC016318	Tribolium castaneum	Putative uncharacterized protein
EOG0912047B	LDEC007247	Leptinotarsa decemlineata	Putative uncharacterized protein
EOG0912047B	YQE_06076	Dendroctonus ponderosae	Putative uncharacterized protein
EOG0912047C	OTAU000809	Onthophagus taurus	Putative uncharacterized protein
EOG0912047C	AGLA013636	Anoplophora glabripennis	Putative uncharacterized protein
EOG0912047C	APLA012177	Agrilus planipennis	Putative uncharacterized protein
EOG0912047C	TC004978	Tribolium castaneum	Putative uncharacterized protein
EOG0912047C	LDEC010448	Leptinotarsa decemlineata	Putative uncharacterized protein
EOG0912047C	YQE_09494	Dendroctonus ponderosae	Putative uncharacterized protein
EOG0912047D	OTAU007401	Onthophagus taurus	Putative uncharacterized protein
EOG0912047D	AGLA016647	Anoplophora glabripennis	Putative uncharacterized protein
EOG0912047D	APLA007373	Agrilus planipennis	Putative uncharacterized protein
EOG0912047D	TC002099	Tribolium castaneum	Putative uncharacterized protein
EOG0912047D	LDEC020801	Leptinotarsa decemlineata	Putative uncharacterized protein
EOG0912047D	YQE_02701	Dendroctonus ponderosae	Putative uncharacterized protein
EOG0912047G	OTAU005853	Onthophagus taurus	Eukaryotic translation initiation factor 6
EOG0912047G	AGLA000581	Anoplophora glabripennis	Eukaryotic translation initiation factor 6
EOG0912047G	APLA001504	Agrilus planipennis	Eukaryotic translation initiation factor 6
EOG0912047G	TC002553	Tribolium castaneum	Eukaryotic translation initiation factor 6
EOG0912047G	LDEC008396	Leptinotarsa decemlineata	Eukaryotic translation initiation factor 6
EOG0912047G	YQE_03671	Dendroctonus ponderosae	Eukaryotic translation initiation factor 6
EOG0912047H	OTAU004493	Onthophagus taurus	Putative uncharacterized protein
EOG0912047H	AGLA017524	Anoplophora glabripennis	Putative uncharacterized protein
EOG0912047H	APLA002063	Agrilus planipennis	Putative uncharacterized protein
EOG0912047H	TC003713	Tribolium castaneum	Putative uncharacterized protein
EOG0912047H	LDEC010277	Leptinotarsa decemlineata	Putative uncharacterized protein

EOG0912047H	YQE_07810	Dendroctonus ponderosae	Putative uncharacterized protein
EOG0912047I	OTAU002941	Onthophagus taurus	Putative uncharacterized protein
EOG0912047I	AGLA002805	Anoplophora glabripennis	Putative uncharacterized protein
EOG0912047I	APLA015479	Agrilus planipennis	Putative uncharacterized protein
EOG0912047I	TC009778	Tribolium castaneum	Putative uncharacterized protein
EOG0912047I	LDEC004066	Leptinotarsa decemlineata	Putative uncharacterized protein
EOG0912047I	YQE_11272	Dendroctonus ponderosae	Putative uncharacterized protein
EOG0912047K	OTAU003397	Onthophagus taurus	Putative uncharacterized protein
EOG0912047K	AGLA021608	Anoplophora glabripennis	Putative uncharacterized protein
EOG0912047K	APLA000721	Agrilus planipennis	Putative uncharacterized protein
EOG0912047K	TC002949	Tribolium castaneum	Putative uncharacterized protein
EOG0912047K	LDEC005051	Leptinotarsa decemlineata	Putative uncharacterized protein
EOG0912047K	YQE_03659	Dendroctonus ponderosae	Putative uncharacterized protein
EOG0912047R	OTAU011228	Onthophagus taurus	Putative uncharacterized protein
EOG0912047R	AGLA000761	Anoplophora glabripennis	Putative uncharacterized protein
EOG0912047R	APLA013179	Agrilus planipennis	Putative uncharacterized protein
EOG0912047R	TC008135	Tribolium castaneum	Putative uncharacterized protein
EOG0912047R	LDEC001000	Leptinotarsa decemlineata	Putative uncharacterized protein
EOG0912047R	YQE_01864	Dendroctonus ponderosae	Putative uncharacterized protein
EOG0912047T	OTAU009834	Onthophagus taurus	Putative uncharacterized protein
EOG0912047T	AGLA012660	Anoplophora glabripennis	Putative uncharacterized protein
EOG0912047T	APLA008805	Agrilus planipennis	Putative uncharacterized protein
EOG0912047T	TC009496	Tribolium castaneum	Putative uncharacterized protein
EOG0912047T	LDEC011008	Leptinotarsa decemlineata	Putative uncharacterized protein
EOG0912047T	YQE_05381	Dendroctonus ponderosae	Putative uncharacterized protein
EOG0912047V	OTAU002801	Onthophagus taurus	Putative uncharacterized protein
EOG0912047V	AGLA001163	Anoplophora glabripennis	Putative uncharacterized protein
EOG0912047V	APLA013950	Agrilus planipennis	Putative uncharacterized protein
EOG0912047V	TC013625	Tribolium castaneum	Putative uncharacterized protein
EOG0912047V	LDEC022723	Leptinotarsa decemlineata	Putative uncharacterized protein
EOG0912047V	YQE_03305	Dendroctonus ponderosae	Putative uncharacterized protein
EOG0912047W	OTAU007976	Onthophagus taurus	Putative uncharacterized protein
EOG0912047W	AGLA011239	Anoplophora glabripennis	Putative uncharacterized protein
EOG0912047W	APLA008419	Agrilus planipennis	Putative uncharacterized protein
EOG0912047W	TC003492	Tribolium castaneum	Putative uncharacterized protein
EOG0912047W	LDEC003267	Leptinotarsa decemlineata	Putative uncharacterized protein
EOG0912047W	YQE_12342	Dendroctonus ponderosae	Putative uncharacterized protein
EOG0912047Y	OTAU003440	Onthophagus taurus	Putative uncharacterized protein
EOG0912047Y	AGLA004259	Anoplophora glabripennis	Putative uncharacterized protein
EOG0912047Y	APLA000092	Agrilus planipennis	Putative uncharacterized protein
EOG0912047Y	TC003668	Tribolium castaneum	Putative uncharacterized protein
EOG0912047Y	LDEC008200	Leptinotarsa decemlineata	Putative uncharacterized protein
EOG0912047Y	YQE_11105	Dendroctonus ponderosae	Putative uncharacterized protein
EOG09120480	OTAU012227	Onthophagus taurus	Putative uncharacterized protein
EOG09120480	AGLA007791	Anoplophora glabripennis	Putative uncharacterized protein
EOG09120480	APLA011128	Agrilus planipennis	Putative uncharacterized protein
EOG09120480	TC000334	Tribolium castaneum	Putative uncharacterized protein
EOG09120480	LDEC021186	Leptinotarsa decemlineata	Putative uncharacterized protein
EOG09120480	YQE_07220	Dendroctonus ponderosae	Putative uncharacterized protein
EOG09120481	OTAU004481	Onthophagus taurus	Putative uncharacterized protein
EOG09120481	AGLA007510	Anoplophora glabripennis	Putative uncharacterized protein
EOG09120481	APLA012733	Agrilus planipennis	Putative uncharacterized protein
EOG09120481	TC002762	Tribolium castaneum	Putative uncharacterized protein
EOG09120481	LDEC013849	Leptinotarsa decemlineata	Putative uncharacterized protein
EOG09120481	YQE_11096	Dendroctonus ponderosae	Putative uncharacterized protein
EOG09120482	OTAU009134	Onthophagus taurus	Putative uncharacterized protein
EOG09120482	AGLA011126	Anoplophora glabripennis	Putative uncharacterized protein
EOG09120482	APLA003086	Agrilus planipennis	Putative uncharacterized protein
EOG09120482	TC013469	Tribolium castaneum	Putative uncharacterized protein
EOG09120482	LDEC015903	Leptinotarsa decemlineata	Putative uncharacterized protein
EOG09120482	YQE_07148	Dendroctonus ponderosae	Putative uncharacterized protein
EOG09120484	OTAU005515	Onthophagus taurus	Putative uncharacterized protein
EOG09120484	AGLA009852	Anoplophora glabripennis	Putative uncharacterized protein
EOG09120484	APLA007414	Agrilus planipennis	Putative uncharacterized protein
EOG09120484	TC012405	Tribolium castaneum	Putative uncharacterized protein
EOG09120484	LDEC015696	Leptinotarsa decemlineata	Putative uncharacterized protein
EOG09120484	YQE_01985	Dendroctonus ponderosae	Putative uncharacterized protein

EOG09120486	OTAU012957	Onthophagus taurus	Putative uncharacterized protein
EOG09120486	AGLA000258	Anoplophora glabripennis	Putative uncharacterized protein
EOG09120486	APLA003493	Agrilus planipennis	Putative uncharacterized protein
EOG09120486	TC001746	Tribolium castaneum	Putative uncharacterized protein
EOG09120486	LDEC012623	Leptinotarsa decemlineata	Putative uncharacterized protein
EOG09120486	YQE_09325	Dendroctonus ponderosae	Putative uncharacterized protein
EOG09120488	OTAU005669	Onthophagus taurus	Putative uncharacterized protein
EOG09120488	AGLA018812	Anoplophora glabripennis	Putative uncharacterized protein
EOG09120488	APLA003419	Agrilus planipennis	Putative uncharacterized protein
EOG09120488	TC008717	Tribolium castaneum	Putative uncharacterized protein
EOG09120488	LDEC000267	Leptinotarsa decemlineata	Putative uncharacterized protein
EOG09120488	YQE_01473	Dendroctonus ponderosae	Putative uncharacterized protein
EOG0912048D	OTAU008479	Onthophagus taurus	Putative uncharacterized protein
EOG0912048D	AGLA007796	Anoplophora glabripennis	Putative uncharacterized protein
EOG0912048D	APLA014274	Agrilus planipennis	Putative uncharacterized protein
EOG0912048D	TC001226	Tribolium castaneum	Putative uncharacterized protein
EOG0912048D	LDEC010158	Leptinotarsa decemlineata	Putative uncharacterized protein
EOG0912048D	YQE_02805	Dendroctonus ponderosae	Putative uncharacterized protein
EOG0912048E	OTAU008751	Onthophagus taurus	Putative uncharacterized protein
EOG0912048E	AGLA008013	Anoplophora glabripennis	Putative uncharacterized protein
EOG0912048E	APLA009137	Agrilus planipennis	Putative uncharacterized protein
EOG0912048E	TC006025	Tribolium castaneum	Putative uncharacterized protein
EOG0912048E	LDEC021569	Leptinotarsa decemlineata	Putative uncharacterized protein
EOG0912048E	YQE_04672	Dendroctonus ponderosae	Putative uncharacterized protein
EOG0912048F	OTAU010535	Onthophagus taurus	DNA binding
EOG0912048F	AGLA002060	Anoplophora glabripennis	DNA binding
EOG0912048F	APLA000809	Agrilus planipennis	DNA binding
EOG0912048F	TC034566	Tribolium castaneum	DNA binding
EOG0912048F	LDEC022429	Leptinotarsa decemlineata	DNA binding
EOG0912048F	YQE_03690	Dendroctonus ponderosae	DNA binding
EOG0912048I	OTAU003940	Onthophagus taurus	Putative uncharacterized protein
EOG0912048I	AGLA009222	Anoplophora glabripennis	Putative uncharacterized protein
EOG0912048I	APLA003197	Agrilus planipennis	Putative uncharacterized protein
EOG0912048I	TC016258	Tribolium castaneum	Putative uncharacterized protein
EOG0912048I	LDEC013112	Leptinotarsa decemlineata	Putative uncharacterized protein
EOG0912048I	YQE_04045	Dendroctonus ponderosae	Putative uncharacterized protein
EOG0912048J	OTAU013803	Onthophagus taurus	metal ion binding
EOG0912048J	AGLA006337	Anoplophora glabripennis	metal ion binding
EOG0912048J	APLA010023	Agrilus planipennis	metal ion binding
EOG0912048J	TC033321	Tribolium castaneum	metal ion binding
EOG0912048J	LDEC007871	Leptinotarsa decemlineata	metal ion binding
EOG0912048J	YQE_08409	Dendroctonus ponderosae	metal ion binding
EOG0912048K	OTAU003360	Onthophagus taurus	Putative uncharacterized protein
EOG0912048K	AGLA008891	Anoplophora glabripennis	Putative uncharacterized protein
EOG0912048K	APLA001085	Agrilus planipennis	Putative uncharacterized protein
EOG0912048K	TC010990	Tribolium castaneum	Putative uncharacterized protein
EOG0912048K	LDEC021472	Leptinotarsa decemlineata	Putative uncharacterized protein
EOG0912048K	YQE_11837	Dendroctonus ponderosae	Putative uncharacterized protein
EOG0912048O	OTAU011475	Onthophagus taurus	Ash
EOG0912048O	AGLA008221	Anoplophora glabripennis	Ash
EOG0912048O	APLA012679	Agrilus planipennis	Ash
EOG0912048O	TC008433	Tribolium castaneum	Ash
EOG0912048O	LDEC003645	Leptinotarsa decemlineata	Ash
EOG0912048O	YQE_00101	Dendroctonus ponderosae	Ash
EOG0912048R	OTAU000858	Onthophagus taurus	Proteasome subunit alpha type
EOG0912048R	AGLA016024	Anoplophora glabripennis	Proteasome subunit alpha type
EOG0912048R	APLA010347	Agrilus planipennis	Proteasome subunit alpha type
EOG0912048R	TC004868	Tribolium castaneum	Proteasome subunit alpha type
EOG0912048R	LDEC024487	Leptinotarsa decemlineata	Proteasome subunit alpha type
EOG0912048R	YQE_00218	Dendroctonus ponderosae	Proteasome subunit alpha type
EOG0912048W	OTAU008016	Onthophagus taurus	Putative uncharacterized protein
EOG0912048W	AGLA019712	Anoplophora glabripennis	Putative uncharacterized protein
EOG0912048W	APLA005780	Agrilus planipennis	Putative uncharacterized protein
EOG0912048W	TC005987	Tribolium castaneum	Putative uncharacterized protein
EOG0912048W	LDEC010110	Leptinotarsa decemlineata	Putative uncharacterized protein
EOG0912048W	YQE_12688	Dendroctonus ponderosae	Putative uncharacterized protein
EOG0912048X	OTAU010917	Onthophagus taurus	Phosphoglycerate kinase

EOG0912048X	AGLA015916	Anoplophora glabripennis	Phosphoglycerate kinase
EOG0912048X	APLA003817	Agrilus planipennis	Phosphoglycerate kinase
EOG0912048X	TC015540	Tribolium castaneum	Phosphoglycerate kinase
EOG0912048X	LDEC009940	Leptinotarsa decemlineata	Phosphoglycerate kinase
EOG0912048X	YQE_11461	Dendroctonus ponderosae	Phosphoglycerate kinase
EOG0912048Z	OTAU011273	Onthophagus taurus	Proteasome subunit alpha type
EOG0912048Z	AGLA012330	Anoplophora glabripennis	Proteasome subunit alpha type
EOG0912048Z	APLA011713	Agrilus planipennis	Proteasome subunit alpha type
EOG0912048Z	TC014574	Tribolium castaneum	Proteasome subunit alpha type
EOG0912048Z	LDEC021657	Leptinotarsa decemlineata	Proteasome subunit alpha type
EOG0912048Z	YQE_08552	Dendroctonus ponderosae	Proteasome subunit alpha type
EOG09120491	OTAU002959	Onthophagus taurus	Putative uncharacterized protein
EOG09120491	AGLA007228	Anoplophora glabripennis	Putative uncharacterized protein
EOG09120491	APLA003393	Agrilus planipennis	Putative uncharacterized protein
EOG09120491	TC009822	Tribolium castaneum	Putative uncharacterized protein
EOG09120491	LDEC012222	Leptinotarsa decemlineata	Putative uncharacterized protein
EOG09120491	YQE_12457	Dendroctonus ponderosae	Putative uncharacterized protein
EOG09120494	OTAU003645	Onthophagus taurus	Putative uncharacterized protein
EOG09120494	AGLA003757	Anoplophora glabripennis	Putative uncharacterized protein
EOG09120494	APLA012542	Agrilus planipennis	Putative uncharacterized protein
EOG09120494	TC008262	Tribolium castaneum	Putative uncharacterized protein
EOG09120494	LDEC020054	Leptinotarsa decemlineata	Putative uncharacterized protein
EOG09120494	YQE_12910	Dendroctonus ponderosae	Putative uncharacterized protein
EOG09120495	OTAU007081	Onthophagus taurus	Putative uncharacterized protein
EOG09120495	AGLA010619	Anoplophora glabripennis	Putative uncharacterized protein
EOG09120495	APLA015411	Agrilus planipennis	Putative uncharacterized protein
EOG09120495	TC014093	Tribolium castaneum	Putative uncharacterized protein
EOG09120495	LDEC015133	Leptinotarsa decemlineata	Putative uncharacterized protein
EOG09120495	YQE_06262	Dendroctonus ponderosae	Putative uncharacterized protein
EOG09120499	OTAU015591	Onthophagus taurus	Putative uncharacterized protein
EOG09120499	AGLA020483	Anoplophora glabripennis	Putative uncharacterized protein
EOG09120499	APLA005869	Agrilus planipennis	Putative uncharacterized protein
EOG09120499	TC000962	Tribolium castaneum	Putative uncharacterized protein
EOG09120499	LDEC005814	Leptinotarsa decemlineata	Putative uncharacterized protein
EOG09120499	YQE_10402	Dendroctonus ponderosae	Putative uncharacterized protein
EOG0912049B	OTAU011182	Onthophagus taurus	Putative uncharacterized protein
EOG0912049B	AGLA004690	Anoplophora glabripennis	Putative uncharacterized protein
EOG0912049B	APLA010322	Agrilus planipennis	Putative uncharacterized protein
EOG0912049B	TC007008	Tribolium castaneum	Putative uncharacterized protein
EOG0912049B	LDEC004378	Leptinotarsa decemlineata	Putative uncharacterized protein
EOG0912049B	YQE_05462	Dendroctonus ponderosae	Putative uncharacterized protein
EOG0912049D	OTAU000054	Onthophagus taurus	Putative uncharacterized protein
EOG0912049D	AGLA009540	Anoplophora glabripennis	Putative uncharacterized protein
EOG0912049D	APLA011573	Agrilus planipennis	Putative uncharacterized protein
EOG0912049D	TC013911	Tribolium castaneum	Putative uncharacterized protein
EOG0912049D	LDEC003222	Leptinotarsa decemlineata	Putative uncharacterized protein
EOG0912049D	YQE_07213	Dendroctonus ponderosae	Putative uncharacterized protein
EOG0912049E	OTAU000890	Onthophagus taurus	Cuticular protein analogous to peritrophins 3-D1
EOG0912049E	AGLA009078	Anoplophora glabripennis	Cuticular protein analogous to peritrophins 3-D1
EOG0912049E	APLA010778	Agrilus planipennis	Cuticular protein analogous to peritrophins 3-D1
EOG0912049E	TC011142	Tribolium castaneum	Cuticular protein analogous to peritrophins 3-D1
EOG0912049E	LDEC004876	Leptinotarsa decemlineata	Cuticular protein analogous to peritrophins 3-D1
EOG0912049E	YQE_04720	Dendroctonus ponderosae	Cuticular protein analogous to peritrophins 3-D1
EOG0912049G	OTAU002905	Onthophagus taurus	Putative uncharacterized protein
EOG0912049G	AGLA006525	Anoplophora glabripennis	Putative uncharacterized protein
EOG0912049G	APLA007167	Agrilus planipennis	Putative uncharacterized protein
EOG0912049G	TC009165	Tribolium castaneum	Putative uncharacterized protein
EOG0912049G	LDEC017854	Leptinotarsa decemlineata	Putative uncharacterized protein
EOG0912049G	YQE_11931	Dendroctonus ponderosae	Putative uncharacterized protein
EOG0912049L	OTAU011437	Onthophagus taurus	Putative uncharacterized protein
EOG0912049L	AGLA001892	Anoplophora glabripennis	Putative uncharacterized protein
EOG0912049L	APLA011694	Agrilus planipennis	Putative uncharacterized protein
EOG0912049L	TC000372	Tribolium castaneum	Putative uncharacterized protein
EOG0912049L	LDEC005265	Leptinotarsa decemlineata	Putative uncharacterized protein
EOG0912049L	YQE_09057	Dendroctonus ponderosae	Putative uncharacterized protein
EOG0912049N	OTAU002322	Onthophagus taurus	None
EOG0912049N	AGLA013206	Anoplophora glabripennis	None

EOG0912049N	APLA012685	Agrilus planipennis	None
EOG0912049N	TC032880	Tribolium castaneum	None
EOG0912049N	LDEC006104	Leptinotarsa decemlineata	None
EOG0912049N	YQE_01754	Dendroctonus ponderosae	None
EOG0912049O	OTAU005492	Onthophagus taurus	Putative uncharacterized protein
EOG0912049O	AGLA008306	Anoplophora glabripennis	Putative uncharacterized protein
EOG0912049O	APLA002432	Agrilus planipennis	Putative uncharacterized protein
EOG0912049O	TC013087	Tribolium castaneum	Putative uncharacterized protein
EOG0912049O	LDEC012506	Leptinotarsa decemlineata	Putative uncharacterized protein
EOG0912049O	YQE_03063	Dendroctonus ponderosae	Putative uncharacterized protein
EOG0912049R	OTAU016826	Onthophagus taurus	Death related ced-3/Nedd2-like protein
EOG0912049R	AGLA006429	Anoplophora glabripennis	Death related ced-3/Nedd2-like protein
EOG0912049R	APLA003617	Agrilus planipennis	Death related ced-3/Nedd2-like protein
EOG0912049R	TC014026	Tribolium castaneum	Death related ced-3/Nedd2-like protein
EOG0912049R	LDEC022924	Leptinotarsa decemlineata	Death related ced-3/Nedd2-like protein
EOG0912049R	YQE_04021	Dendroctonus ponderosae	Death related ced-3/Nedd2-like protein
EOG0912049T	OTAU003493	Onthophagus taurus	Putative uncharacterized protein
EOG0912049T	AGLA018686	Anoplophora glabripennis	Putative uncharacterized protein
EOG0912049T	APLA014453	Agrilus planipennis	Putative uncharacterized protein
EOG0912049T	TC008880	Tribolium castaneum	Putative uncharacterized protein
EOG0912049T	LDEC004447	Leptinotarsa decemlineata	Putative uncharacterized protein
EOG0912049T	YQE_04293	Dendroctonus ponderosae	Putative uncharacterized protein
EOG0912049U	OTAU000634	Onthophagus taurus	Putative uncharacterized protein
EOG0912049U	AGLA020567	Anoplophora glabripennis	Putative uncharacterized protein
EOG0912049U	APLA014329	Agrilus planipennis	Putative uncharacterized protein
EOG0912049U	TC012326	Tribolium castaneum	Putative uncharacterized protein
EOG0912049U	LDEC018232	Leptinotarsa decemlineata	Putative uncharacterized protein
EOG0912049U	YQE_12825	Dendroctonus ponderosae	Putative uncharacterized protein
EOG0912049V	OTAU009985	Onthophagus taurus	Putative uncharacterized protein
EOG0912049V	AGLA001932	Anoplophora glabripennis	Putative uncharacterized protein
EOG0912049V	APLA007861	Agrilus planipennis	Putative uncharacterized protein
EOG0912049V	TC005229	Tribolium castaneum	Putative uncharacterized protein
EOG0912049V	LDEC000878	Leptinotarsa decemlineata	Putative uncharacterized protein
EOG0912049V	YQE_02407	Dendroctonus ponderosae	Putative uncharacterized protein
EOG0912049X	OTAU007062	Onthophagus taurus	Putative uncharacterized protein
EOG0912049X	AGLA009146	Anoplophora glabripennis	Putative uncharacterized protein
EOG0912049X	APLA012011	Agrilus planipennis	Putative uncharacterized protein
EOG0912049X	TC010485	Tribolium castaneum	Putative uncharacterized protein
EOG0912049X	LDEC010574	Leptinotarsa decemlineata	Putative uncharacterized protein
EOG0912049X	YQE_10231	Dendroctonus ponderosae	Putative uncharacterized protein
EOG0912049Z	OTAU005464	Onthophagus taurus	Putative uncharacterized protein
EOG0912049Z	AGLA005991	Anoplophora glabripennis	Putative uncharacterized protein
EOG0912049Z	APLA009848	Agrilus planipennis	Putative uncharacterized protein
EOG0912049Z	TC013118	Tribolium castaneum	Putative uncharacterized protein
EOG0912049Z	LDEC011671	Leptinotarsa decemlineata	Putative uncharacterized protein
EOG0912049Z	YQE_06062	Dendroctonus ponderosae	Putative uncharacterized protein
EOG091204A1	OTAU009775	Onthophagus taurus	Putative uncharacterized protein
EOG091204A1	AGLA017518	Anoplophora glabripennis	Putative uncharacterized protein
EOG091204A1	APLA006368	Agrilus planipennis	Putative uncharacterized protein
EOG091204A1	TC004755	Tribolium castaneum	Putative uncharacterized protein
EOG091204A1	LDEC010272	Leptinotarsa decemlineata	Putative uncharacterized protein
EOG091204A1	YQE_06719	Dendroctonus ponderosae	Putative uncharacterized protein
EOG091204A6	OTAU000590	Onthophagus taurus	Putative uncharacterized protein
EOG091204A6	AGLA007742	Anoplophora glabripennis	Putative uncharacterized protein
EOG091204A6	APLA011369	Agrilus planipennis	Putative uncharacterized protein
EOG091204A6	TC003909	Tribolium castaneum	Putative uncharacterized protein
EOG091204A6	LDEC000231	Leptinotarsa decemlineata	Putative uncharacterized protein
EOG091204A6	YQE_09796	Dendroctonus ponderosae	Putative uncharacterized protein
EOG091204A8	OTAU003206	Onthophagus taurus	Putative uncharacterized protein
EOG091204A8	AGLA015871	Anoplophora glabripennis	Putative uncharacterized protein
EOG091204A8	APLA007985	Agrilus planipennis	Putative uncharacterized protein
EOG091204A8	TC014297	Tribolium castaneum	Putative uncharacterized protein
EOG091204A8	LDEC003096	Leptinotarsa decemlineata	Putative uncharacterized protein
EOG091204A8	YQE_08621	Dendroctonus ponderosae	Putative uncharacterized protein
EOG091204A9	OTAU017363	Onthophagus taurus	Putative uncharacterized protein
EOG091204A9	AGLA000198	Anoplophora glabripennis	Putative uncharacterized protein
EOG091204A9	APLA008678	Agrilus planipennis	Putative uncharacterized protein

EOG091204A9	TC005896	Tribolium castaneum	Putative uncharacterized protein
EOG091204A9	LDEC005413	Leptinotarsa decemlineata	Putative uncharacterized protein
EOG091204A9	YQE_11518	Dendroctonus ponderosae	Putative uncharacterized protein
EOG091204AC	OTAU003485	Onthophagus taurus	Putative uncharacterized protein
EOG091204AC	AGLA008528	Anoplophora glabripennis	Putative uncharacterized protein
EOG091204AC	APLA012229	Agrilus planipennis	Putative uncharacterized protein
EOG091204AC	TC009327	Tribolium castaneum	Putative uncharacterized protein
EOG091204AC	LDEC017107	Leptinotarsa decemlineata	Putative uncharacterized protein
EOG091204AC	YQE_02340	Dendroctonus ponderosae	Putative uncharacterized protein
EOG091204AF	OTAU015022	Onthophagus taurus	Putative uncharacterized protein
EOG091204AF	AGLA012018	Anoplophora glabripennis	Putative uncharacterized protein
EOG091204AF	APLA008313	Agrilus planipennis	Putative uncharacterized protein
EOG091204AF	TC007170	Tribolium castaneum	Putative uncharacterized protein
EOG091204AF	LDEC007113	Leptinotarsa decemlineata	Putative uncharacterized protein
EOG091204AF	YQE_04580	Dendroctonus ponderosae	Putative uncharacterized protein
EOG091204AG	OTAU002751	Onthophagus taurus	Putative uncharacterized protein
EOG091204AG	AGLA000237	Anoplophora glabripennis	Putative uncharacterized protein
EOG091204AG	APLA010007	Agrilus planipennis	Putative uncharacterized protein
EOG091204AG	TC001593	Tribolium castaneum	Putative uncharacterized protein
EOG091204AG	LDEC001043	Leptinotarsa decemlineata	Putative uncharacterized protein
EOG091204AG	YQE_09192	Dendroctonus ponderosae	Putative uncharacterized protein
EOG091204AH	OTAU003909	Onthophagus taurus	Ribonuclease
EOG091204AH	AGLA013664	Anoplophora glabripennis	Ribonuclease
EOG091204AH	APLA003017	Agrilus planipennis	Ribonuclease
EOG091204AH	TC011359	Tribolium castaneum	Ribonuclease
EOG091204AH	LDEC004460	Leptinotarsa decemlineata	Ribonuclease
EOG091204AH	YQE_09571	Dendroctonus ponderosae	Ribonuclease
EOG091204AJ	OTAU005451	Onthophagus taurus	Putative uncharacterized protein
EOG091204AJ	AGLA016747	Anoplophora glabripennis	Putative uncharacterized protein
EOG091204AJ	APLA012138	Agrilus planipennis	Putative uncharacterized protein
EOG091204AJ	TC013192	Tribolium castaneum	Putative uncharacterized protein
EOG091204AJ	LDEC005963	Leptinotarsa decemlineata	Putative uncharacterized protein
EOG091204AJ	YQE_06198	Dendroctonus ponderosae	Putative uncharacterized protein
EOG091204AL	OTAU007917	Onthophagus taurus	Putative uncharacterized protein
EOG091204AL	AGLA017193	Anoplophora glabripennis	Putative uncharacterized protein
EOG091204AL	APLA012609	Agrilus planipennis	Putative uncharacterized protein
EOG091204AL	TC012375	Tribolium castaneum	Putative uncharacterized protein
EOG091204AL	LDEC015684	Leptinotarsa decemlineata	Putative uncharacterized protein
EOG091204AL	YQE_06505	Dendroctonus ponderosae	Putative uncharacterized protein
EOG091204AN	OTAU011778	Onthophagus taurus	Putative uncharacterized protein
EOG091204AN	AGLA017714	Anoplophora glabripennis	Putative uncharacterized protein
EOG091204AN	APLA001631	Agrilus planipennis	Putative uncharacterized protein
EOG091204AN	TC009612	Tribolium castaneum	Putative uncharacterized protein
EOG091204AN	LDEC009362	Leptinotarsa decemlineata	Putative uncharacterized protein
EOG091204AN	YQE_05751	Dendroctonus ponderosae	Putative uncharacterized protein
EOG091204AO	OTAU013077	Onthophagus taurus	Putative uncharacterized protein
EOG091204AO	AGLA013738	Anoplophora glabripennis	Putative uncharacterized protein
EOG091204AO	APLA005910	Agrilus planipennis	Putative uncharacterized protein
EOG091204AO	TC015211	Tribolium castaneum	Putative uncharacterized protein
EOG091204AO	LDEC005617	Leptinotarsa decemlineata	Putative uncharacterized protein
EOG091204AO	YQE_02938	Dendroctonus ponderosae	Putative uncharacterized protein
EOG091204AQ	OTAU000975	Onthophagus taurus	Putative uncharacterized protein
EOG091204AQ	AGLA004120	Anoplophora glabripennis	Putative uncharacterized protein
EOG091204AQ	APLA004098	Agrilus planipennis	Putative uncharacterized protein
EOG091204AQ	TC011318	Tribolium castaneum	Putative uncharacterized protein
EOG091204AQ	LDEC007053	Leptinotarsa decemlineata	Putative uncharacterized protein
EOG091204AQ	YQE_07715	Dendroctonus ponderosae	Putative uncharacterized protein
EOG091204AU	OTAU000276	Onthophagus taurus	Putative uncharacterized protein
EOG091204AU	AGLA009864	Anoplophora glabripennis	Putative uncharacterized protein
EOG091204AU	APLA011719	Agrilus planipennis	Putative uncharacterized protein
EOG091204AU	TC013110	Tribolium castaneum	Putative uncharacterized protein
EOG091204AU	LDEC006270	Leptinotarsa decemlineata	Putative uncharacterized protein
EOG091204AU	YQE_03973	Dendroctonus ponderosae	Putative uncharacterized protein
EOG091204AV	OTAU000059	Onthophagus taurus	Putative uncharacterized protein
EOG091204AV	AGLA009544	Anoplophora glabripennis	Putative uncharacterized protein
EOG091204AV	APLA011572	Agrilus planipennis	Putative uncharacterized protein
EOG091204AV	TC013891	Tribolium castaneum	Putative uncharacterized protein

EOG091204AV	LDEC003225	Leptinotarsa decemlineata	Putative uncharacterized protein
EOG091204AV	YQE_07225	Dendroctonus ponderosae	Putative uncharacterized protein
EOG091204AW	OTAU005787	Onthophagus taurus	Putative uncharacterized protein
EOG091204AW	AGLA001479	Anoplophora glabripennis	Putative uncharacterized protein
EOG091204AW	APLA000126	Agrilus planipennis	Putative uncharacterized protein
EOG091204AW	TC000069	Tribolium castaneum	Putative uncharacterized protein
EOG091204AW	LDEC003296	Leptinotarsa decemlineata	Putative uncharacterized protein
EOG091204AW	YQE_05185	Dendroctonus ponderosae	Putative uncharacterized protein
EOG091204AY	OTAU002115	Onthophagus taurus	Putative uncharacterized protein
EOG091204AY	AGLA017229	Anoplophora glabripennis	Putative uncharacterized protein
EOG091204AY	APLA003242	Agrilus planipennis	Putative uncharacterized protein
EOG091204AY	TC015985	Tribolium castaneum	Putative uncharacterized protein
EOG091204AY	LDEC003860	Leptinotarsa decemlineata	Putative uncharacterized protein
EOG091204AY	YQE_07576	Dendroctonus ponderosae	Putative uncharacterized protein
EOG091204B0	OTAU001836	Onthophagus taurus	Putative uncharacterized protein
EOG091204B0	AGLA005408	Anoplophora glabripennis	Putative uncharacterized protein
EOG091204B0	APLA001712	Agrilus planipennis	Putative uncharacterized protein
EOG091204B0	TC006409	Tribolium castaneum	Putative uncharacterized protein
EOG091204B0	LDEC017602	Leptinotarsa decemlineata	Putative uncharacterized protein
EOG091204B0	YQE_06995	Dendroctonus ponderosae	Putative uncharacterized protein
EOG091204B3	OTAU005583	Onthophagus taurus	Putative uncharacterized protein
EOG091204B3	AGLA000754	Anoplophora glabripennis	Putative uncharacterized protein
EOG091204B3	APLA009259	Agrilus planipennis	Putative uncharacterized protein
EOG091204B3	TC007369	Tribolium castaneum	Putative uncharacterized protein
EOG091204B3	LDEC000992	Leptinotarsa decemlineata	Putative uncharacterized protein
EOG091204B3	YQE_03857	Dendroctonus ponderosae	Putative uncharacterized protein
EOG091204B4	OTAU002183	Onthophagus taurus	Putative uncharacterized protein
EOG091204B4	AGLA001956	Anoplophora glabripennis	Putative uncharacterized protein
EOG091204B4	APLA008651	Agrilus planipennis	Putative uncharacterized protein
EOG091204B4	TC011815	Tribolium castaneum	Putative uncharacterized protein
EOG091204B4	LDEC015642	Leptinotarsa decemlineata	Putative uncharacterized protein
EOG091204B4	YQE_08439	Dendroctonus ponderosae	Putative uncharacterized protein
EOG091204B5	OTAU014018	Onthophagus taurus	Putative uncharacterized protein
EOG091204B5	AGLA003727	Anoplophora glabripennis	Putative uncharacterized protein
EOG091204B5	APLA001407	Agrilus planipennis	Putative uncharacterized protein
EOG091204B5	TC007473	Tribolium castaneum	Putative uncharacterized protein
EOG091204B5	LDEC016687	Leptinotarsa decemlineata	Putative uncharacterized protein
EOG091204B5	YQE_12663	Dendroctonus ponderosae	Putative uncharacterized protein
EOG091204B9	OTAU007172	Onthophagus taurus	Putative uncharacterized protein
EOG091204B9	AGLA015907	Anoplophora glabripennis	Putative uncharacterized protein
EOG091204B9	APLA000682	Agrilus planipennis	Putative uncharacterized protein
EOG091204B9	TC015539	Tribolium castaneum	Putative uncharacterized protein
EOG091204B9	LDEC009934	Leptinotarsa decemlineata	Putative uncharacterized protein
EOG091204B9	YQE_11710	Dendroctonus ponderosae	Putative uncharacterized protein
EOG091204BA	OTAU004041	Onthophagus taurus	Putative uncharacterized protein
EOG091204BA	AGLA020655	Anoplophora glabripennis	Putative uncharacterized protein
EOG091204BA	APLA006637	Agrilus planipennis	Putative uncharacterized protein
EOG091204BA	TC010381	Tribolium castaneum	Putative uncharacterized protein
EOG091204BA	LDEC002019	Leptinotarsa decemlineata	Putative uncharacterized protein
EOG091204BA	YQE_02151	Dendroctonus ponderosae	Putative uncharacterized protein
EOG091204BB	OTAU001178	Onthophagus taurus	Putative uncharacterized protein
EOG091204BB	AGLA001417	Anoplophora glabripennis	Putative uncharacterized protein
EOG091204BB	APLA008577	Agrilus planipennis	Putative uncharacterized protein
EOG091204BB	TC001960	Tribolium castaneum	Putative uncharacterized protein
EOG091204BB	LDEC017043	Leptinotarsa decemlineata	Putative uncharacterized protein
EOG091204BB	YQE_06359	Dendroctonus ponderosae	Putative uncharacterized protein
EOG091204BD	OTAU007634	Onthophagus taurus	Putative uncharacterized protein
EOG091204BD	AGLA014143	Anoplophora glabripennis	Putative uncharacterized protein
EOG091204BD	APLA004380	Agrilus planipennis	Putative uncharacterized protein
EOG091204BD	TC010925	Tribolium castaneum	Putative uncharacterized protein
EOG091204BD	LDEC008152	Leptinotarsa decemlineata	Putative uncharacterized protein
EOG091204BD	YQE_00577	Dendroctonus ponderosae	Putative uncharacterized protein
EOG091204BG	OTAU002891	Onthophagus taurus	Putative uncharacterized protein
EOG091204BG	AGLA021530	Anoplophora glabripennis	Putative uncharacterized protein
EOG091204BG	APLA007537	Agrilus planipennis	Putative uncharacterized protein
EOG091204BG	TC009633	Tribolium castaneum	Putative uncharacterized protein
EOG091204BG	LDEC004467	Leptinotarsa decemlineata	Putative uncharacterized protein

EOG091204BG	YQE_13012	Dendroctonus ponderosae	Putative uncharacterized protein
EOG091204BH	OTAU008346	Onthophagus taurus	Putative uncharacterized protein
EOG091204BH	AGLA003144	Anoplophora glabripennis	Putative uncharacterized protein
EOG091204BH	APLA014472	Agrilus planipennis	Putative uncharacterized protein
EOG091204BH	TC011968	Tribolium castaneum	Putative uncharacterized protein
EOG091204BH	LDEC014554	Leptinotarsa decemlineata	Putative uncharacterized protein
EOG091204BH	YQE_08008	Dendroctonus ponderosae	Putative uncharacterized protein
EOG091204BI	OTAU012502	Onthophagus taurus	Putative uncharacterized protein
EOG091204BI	AGLA008502	Anoplophora glabripennis	Putative uncharacterized protein
EOG091204BI	APLA003728	Agrilus planipennis	Putative uncharacterized protein
EOG091204BI	TC010534	Tribolium castaneum	Putative uncharacterized protein
EOG091204BI	LDEC002662	Leptinotarsa decemlineata	Putative uncharacterized protein
EOG091204BI	YQE_01764	Dendroctonus ponderosae	Putative uncharacterized protein
EOG091204BK	OTAU010684	Onthophagus taurus	Putative uncharacterized protein
EOG091204BK	AGLA005765	Anoplophora glabripennis	Putative uncharacterized protein
EOG091204BK	APLA013226	Agrilus planipennis	Putative uncharacterized protein
EOG091204BK	TC009230	Tribolium castaneum	Putative uncharacterized protein
EOG091204BK	LDEC005234	Leptinotarsa decemlineata	Putative uncharacterized protein
EOG091204BK	YQE_04289	Dendroctonus ponderosae	Putative uncharacterized protein
EOG091204BM	OTAU012959	Onthophagus taurus	Putative uncharacterized protein
EOG091204BM	AGLA000080	Anoplophora glabripennis	Putative uncharacterized protein
EOG091204BM	APLA004543	Agrilus planipennis	Putative uncharacterized protein
EOG091204BM	TC002077	Tribolium castaneum	Putative uncharacterized protein
EOG091204BM	LDEC017314	Leptinotarsa decemlineata	Putative uncharacterized protein
EOG091204BM	YQE_10841	Dendroctonus ponderosae	Putative uncharacterized protein
EOG091204BN	OTAU002440	Onthophagus taurus	Putative uncharacterized protein
EOG091204BN	AGLA019020	Anoplophora glabripennis	Putative uncharacterized protein
EOG091204BN	APLA008422	Agrilus planipennis	Putative uncharacterized protein
EOG091204BN	TC002125	Tribolium castaneum	Putative uncharacterized protein
EOG091204BN	LDEC002017	Leptinotarsa decemlineata	Putative uncharacterized protein
EOG091204BN	YQE_03836	Dendroctonus ponderosae	Putative uncharacterized protein
EOG091204BO	OTAU010250	Onthophagus taurus	Abdominal-B
EOG091204BO	AGLA001835	Anoplophora glabripennis	Abdominal-B
EOG091204BO	APLA002105	Agrilus planipennis	Abdominal-B
EOG091204BO	TC000889	Tribolium castaneum	Abdominal-B
EOG091204BO	LDEC005307	Leptinotarsa decemlineata	Abdominal-B
EOG091204BO	YQE_04570	Dendroctonus ponderosae	Abdominal-B
EOG091204BP	OTAU001502	Onthophagus taurus	Putative uncharacterized protein
EOG091204BP	AGLA014040	Anoplophora glabripennis	Putative uncharacterized protein
EOG091204BP	APLA000014	Agrilus planipennis	Putative uncharacterized protein
EOG091204BP	TC001866	Tribolium castaneum	Putative uncharacterized protein
EOG091204BP	LDEC012522	Leptinotarsa decemlineata	Putative uncharacterized protein
EOG091204BP	YQE_06682	Dendroctonus ponderosae	Putative uncharacterized protein
EOG091204BQ	OTAU001368	Onthophagus taurus	Putative uncharacterized protein
EOG091204BQ	AGLA000397	Anoplophora glabripennis	Putative uncharacterized protein
EOG091204BQ	APLA012420	Agrilus planipennis	Putative uncharacterized protein
EOG091204BQ	TC014225	Tribolium castaneum	Putative uncharacterized protein
EOG091204BQ	LDEC004697	Leptinotarsa decemlineata	Putative uncharacterized protein
EOG091204BQ	YQE_09228	Dendroctonus ponderosae	Putative uncharacterized protein
EOG091204BR	OTAU013424	Onthophagus taurus	Putative uncharacterized protein
EOG091204BR	AGLA006349	Anoplophora glabripennis	Putative uncharacterized protein
EOG091204BR	APLA000667	Agrilus planipennis	Putative uncharacterized protein
EOG091204BR	TC014905	Tribolium castaneum	Putative uncharacterized protein
EOG091204BR	LDEC007858	Leptinotarsa decemlineata	Putative uncharacterized protein
EOG091204BR	YQE_11054	Dendroctonus ponderosae	Putative uncharacterized protein
EOG091204BU	OTAU009883	Onthophagus taurus	Putative uncharacterized protein
EOG091204BU	AGLA000142	Anoplophora glabripennis	Putative uncharacterized protein
EOG091204BU	APLA012840	Agrilus planipennis	Putative uncharacterized protein
EOG091204BU	TC000224	Tribolium castaneum	Putative uncharacterized protein
EOG091204BU	LDEC016693	Leptinotarsa decemlineata	Putative uncharacterized protein
EOG091204BU	YQE_08060	Dendroctonus ponderosae	Putative uncharacterized protein
EOG091204BV	OTAU010313	Onthophagus taurus	Putative uncharacterized protein
EOG091204BV	AGLA004071	Anoplophora glabripennis	Putative uncharacterized protein
EOG091204BV	APLA011229	Agrilus planipennis	Putative uncharacterized protein
EOG091204BV	TC009712	Tribolium castaneum	Putative uncharacterized protein
EOG091204BV	LDEC012802	Leptinotarsa decemlineata	Putative uncharacterized protein
EOG091204BV	YQE_05251	Dendroctonus ponderosae	Putative uncharacterized protein

EOG091204BY	OTAU002091	Onthophagus taurus	Putative uncharacterized protein
EOG091204BY	AGLA003297	Anoplophora glabripennis	Putative uncharacterized protein
EOG091204BY	APLA001124	Agrilus planipennis	Putative uncharacterized protein
EOG091204BY	TC012844	Tribolium castaneum	Putative uncharacterized protein
EOG091204BY	LDEC017641	Leptinotarsa decemlineata	Putative uncharacterized protein
EOG091204BY	YQE_04319	Dendroctonus ponderosae	Putative uncharacterized protein
EOG091204C3	OTAU011731	Onthophagus taurus	Putative uncharacterized protein
EOG091204C3	AGLA020712	Anoplophora glabripennis	Putative uncharacterized protein
EOG091204C3	APLA000209	Agrilus planipennis	Putative uncharacterized protein
EOG091204C3	TC007574	Tribolium castaneum	Putative uncharacterized protein
EOG091204C3	LDEC006472	Leptinotarsa decemlineata	Putative uncharacterized protein
EOG091204C3	YQE_04481	Dendroctonus ponderosae	Putative uncharacterized protein
EOG091204C7	OTAU004992	Onthophagus taurus	Putative uncharacterized protein
EOG091204C7	AGLA010162	Anoplophora glabripennis	Putative uncharacterized protein
EOG091204C7	APLA006329	Agrilus planipennis	Putative uncharacterized protein
EOG091204C7	TC011093	Tribolium castaneum	Putative uncharacterized protein
EOG091204C7	LDEC006859	Leptinotarsa decemlineata	Putative uncharacterized protein
EOG091204C7	YQE_09901	Dendroctonus ponderosae	Putative uncharacterized protein
EOG091204C8	OTAU011816	Onthophagus taurus	Putative uncharacterized protein
EOG091204C8	AGLA010339	Anoplophora glabripennis	Putative uncharacterized protein
EOG091204C8	APLA001549	Agrilus planipennis	Putative uncharacterized protein
EOG091204C8	TC008707	Tribolium castaneum	Putative uncharacterized protein
EOG091204C8	LDEC001300	Leptinotarsa decemlineata	Putative uncharacterized protein
EOG091204C8	YQE_10601	Dendroctonus ponderosae	Putative uncharacterized protein
EOG091204CC	OTAU004499	Onthophagus taurus	Putative uncharacterized protein
EOG091204CC	AGLA003310	Anoplophora glabripennis	Putative uncharacterized protein
EOG091204CC	APLA004191	Agrilus planipennis	Putative uncharacterized protein
EOG091204CC	TC003731	Tribolium castaneum	Putative uncharacterized protein
EOG091204CC	LDEC014838	Leptinotarsa decemlineata	Putative uncharacterized protein
EOG091204CC	YQE_10060	Dendroctonus ponderosae	Putative uncharacterized protein
EOG091204CE	OTAU010083	Onthophagus taurus	Putative uncharacterized protein
EOG091204CE	AGLA009965	Anoplophora glabripennis	Putative uncharacterized protein
EOG091204CE	APLA000589	Agrilus planipennis	Putative uncharacterized protein
EOG091204CE	TC015665	Tribolium castaneum	Putative uncharacterized protein
EOG091204CE	LDEC006921	Leptinotarsa decemlineata	Putative uncharacterized protein
EOG091204CE	YQE_11713	Dendroctonus ponderosae	Putative uncharacterized protein
EOG091204CJ	OTAU011613	Onthophagus taurus	Putative uncharacterized protein
EOG091204CJ	AGLA018696	Anoplophora glabripennis	Putative uncharacterized protein
EOG091204CJ	APLA013236	Agrilus planipennis	Putative uncharacterized protein
EOG091204CJ	TC009432	Tribolium castaneum	Putative uncharacterized protein
EOG091204CJ	LDEC014661	Leptinotarsa decemlineata	Putative uncharacterized protein
EOG091204CJ	YQE_13024	Dendroctonus ponderosae	Putative uncharacterized protein
EOG091204CL	OTAU006092	Onthophagus taurus	Putative uncharacterized protein
EOG091204CL	AGLA012771	Anoplophora glabripennis	Putative uncharacterized protein
EOG091204CL	APLA005152	Agrilus planipennis	Putative uncharacterized protein
EOG091204CL	TC013008	Tribolium castaneum	Putative uncharacterized protein
EOG091204CL	LDEC014508	Leptinotarsa decemlineata	Putative uncharacterized protein
EOG091204CL	YQE_07262	Dendroctonus ponderosae	Putative uncharacterized protein
EOG091204CP	OTAU008543	Onthophagus taurus	Triosephosphate isomerase
EOG091204CP	AGLA000848	Anoplophora glabripennis	Triosephosphate isomerase
EOG091204CP	APLA006547	Agrilus planipennis	Triosephosphate isomerase
EOG091204CP	TC007346	Tribolium castaneum	Triosephosphate isomerase
EOG091204CP	LDEC016681	Leptinotarsa decemlineata	Triosephosphate isomerase
EOG091204CP	YQE_04739	Dendroctonus ponderosae	Triosephosphate isomerase
EOG091204CQ	OTAU013793	Onthophagus taurus	None
EOG091204CQ	AGLA016368	Anoplophora glabripennis	None
EOG091204CQ	APLA004646	Agrilus planipennis	None
EOG091204CQ	TC033703	Tribolium castaneum	None
EOG091204CQ	LDEC020153	Leptinotarsa decemlineata	None
EOG091204CQ	YQE_10623	Dendroctonus ponderosae	None
EOG091204CR	OTAU012117	Onthophagus taurus	Putative uncharacterized protein
EOG091204CR	AGLA016541	Anoplophora glabripennis	Putative uncharacterized protein
EOG091204CR	APLA008027	Agrilus planipennis	Putative uncharacterized protein
EOG091204CR	TC016200	Tribolium castaneum	Putative uncharacterized protein
EOG091204CR	LDEC018507	Leptinotarsa decemlineata	Putative uncharacterized protein
EOG091204CR	YQE_12182	Dendroctonus ponderosae	Putative uncharacterized protein
EOG091204CV	OTAU009348	Onthophagus taurus	Putative uncharacterized protein

EOG091204CV	AGLA005992	Anoplophora glabripennis	Putative uncharacterized protein
EOG091204CV	APLA012147	Agrilus planipennis	Putative uncharacterized protein
EOG091204CV	TC014793	Tribolium castaneum	Putative uncharacterized protein
EOG091204CV	LDEC024404	Leptinotarsa decemlineata	Putative uncharacterized protein
EOG091204CV	YQE_06061	Dendroctonus ponderosae	Putative uncharacterized protein
EOG091204CW	OTAU014005	Onthophagus taurus	Putative uncharacterized protein
EOG091204CW	AGLA002611	Anoplophora glabripennis	Putative uncharacterized protein
EOG091204CW	APLA003923	Agrilus planipennis	Putative uncharacterized protein
EOG091204CW	TC012458	Tribolium castaneum	Putative uncharacterized protein
EOG091204CW	LDEC001986	Leptinotarsa decemlineata	Putative uncharacterized protein
EOG091204CW	YQE_03162	Dendroctonus ponderosae	Putative uncharacterized protein
EOG091204CX	OTAU007635	Onthophagus taurus	None
EOG091204CX	AGLA010051	Anoplophora glabripennis	None
EOG091204CX	APLA004396	Agrilus planipennis	None
EOG091204CX	TC033553	Tribolium castaneum	None
EOG091204CX	LDEC011031	Leptinotarsa decemlineata	None
EOG091204CX	YQE_03451	Dendroctonus ponderosae	None
EOG091204D3	OTAU015815	Onthophagus taurus	Putative uncharacterized protein
EOG091204D3	AGLA018776	Anoplophora glabripennis	Putative uncharacterized protein
EOG091204D3	APLA001904	Agrilus planipennis	Putative uncharacterized protein
EOG091204D3	TC001071	Tribolium castaneum	Putative uncharacterized protein
EOG091204D3	LDEC018561	Leptinotarsa decemlineata	Putative uncharacterized protein
EOG091204D3	YQE_07067	Dendroctonus ponderosae	Putative uncharacterized protein
EOG091204D4	OTAU005753	Onthophagus taurus	Putative uncharacterized protein
EOG091204D4	AGLA000556	Anoplophora glabripennis	Putative uncharacterized protein
EOG091204D4	APLA001480	Agrilus planipennis	Putative uncharacterized protein
EOG091204D4	TC000269	Tribolium castaneum	Putative uncharacterized protein
EOG091204D4	LDEC023486	Leptinotarsa decemlineata	Putative uncharacterized protein
EOG091204D4	YQE_07900	Dendroctonus ponderosae	Putative uncharacterized protein
EOG091204D8	OTAU007771	Onthophagus taurus	Putative uncharacterized protein
EOG091204D8	AGLA014651	Anoplophora glabripennis	Putative uncharacterized protein
EOG091204D8	APLA007053	Agrilus planipennis	Putative uncharacterized protein
EOG091204D8	TC009931	Tribolium castaneum	Putative uncharacterized protein
EOG091204D8	LDEC009336	Leptinotarsa decemlineata	Putative uncharacterized protein
EOG091204D8	YQE_05343	Dendroctonus ponderosae	Putative uncharacterized protein
EOG091204DF	OTAU015536	Onthophagus taurus	Putative uncharacterized protein
EOG091204DF	AGLA016194	Anoplophora glabripennis	Putative uncharacterized protein
EOG091204DF	APLA000532	Agrilus planipennis	Putative uncharacterized protein
EOG091204DF	TC015668	Tribolium castaneum	Putative uncharacterized protein
EOG091204DF	LDEC019453	Leptinotarsa decemlineata	Putative uncharacterized protein
EOG091204DF	YQE_02162	Dendroctonus ponderosae	Putative uncharacterized protein
EOG091204DG	OTAU006750	Onthophagus taurus	Putative uncharacterized protein
EOG091204DG	AGLA001808	Anoplophora glabripennis	Putative uncharacterized protein
EOG091204DG	APLA001040	Agrilus planipennis	Putative uncharacterized protein
EOG091204DG	TC010671	Tribolium castaneum	Putative uncharacterized protein
EOG091204DG	LDEC005796	Leptinotarsa decemlineata	Putative uncharacterized protein
EOG091204DG	YQE_04332	Dendroctonus ponderosae	Putative uncharacterized protein
EOG091204DI	OTAU005558	Onthophagus taurus	Putative uncharacterized protein
EOG091204DI	AGLA003754	Anoplophora glabripennis	Putative uncharacterized protein
EOG091204DI	APLA013141	Agrilus planipennis	Putative uncharacterized protein
EOG091204DI	TC007476	Tribolium castaneum	Putative uncharacterized protein
EOG091204DI	LDEC000105	Leptinotarsa decemlineata	Putative uncharacterized protein
EOG091204DI	YQE_09535	Dendroctonus ponderosae	Putative uncharacterized protein
EOG091204DL	OTAU006360	Onthophagus taurus	Diphthine synthase
EOG091204DL	AGLA019755	Anoplophora glabripennis	Diphthine synthase
EOG091204DL	APLA010096	Agrilus planipennis	Diphthine synthase
EOG091204DL	TC014546	Tribolium castaneum	Diphthine synthase
EOG091204DL	LDEC018201	Leptinotarsa decemlineata	Diphthine synthase
EOG091204DL	YQE_06001	Dendroctonus ponderosae	Diphthine synthase
EOG091204DR	OTAU012080	Onthophagus taurus	Putative uncharacterized protein
EOG091204DR	AGLA003452	Anoplophora glabripennis	Putative uncharacterized protein
EOG091204DR	APLA008186	Agrilus planipennis	Putative uncharacterized protein
EOG091204DR	TC009839	Tribolium castaneum	Putative uncharacterized protein
EOG091204DR	LDEC013022	Leptinotarsa decemlineata	Putative uncharacterized protein
EOG091204DR	YQE_08820	Dendroctonus ponderosae	Putative uncharacterized protein
EOG091204DT	OTAU001171	Onthophagus taurus	Ribulose-phosphate 3-epimerase
EOG091204DT	AGLA002438	Anoplophora glabripennis	Ribulose-phosphate 3-epimerase

EOG091204DT	APLA015453	Agrilus planipennis	Ribulose-phosphate 3-epimerase
EOG091204DT	TC011438	Tribolium castaneum	Ribulose-phosphate 3-epimerase
EOG091204DT	LDEC009636	Leptinotarsa decemlineata	Ribulose-phosphate 3-epimerase
EOG091204DT	YQE_08183	Dendroctonus ponderosae	Ribulose-phosphate 3-epimerase
EOG091204DX	OTAU000944	Onthophagus taurus	Eukaryotic initiation factor 4E
EOG091204DX	AGLA002981	Anoplophora glabripennis	Eukaryotic initiation factor 4E
EOG091204DX	APLA012173	Agrilus planipennis	Eukaryotic initiation factor 4E
EOG091204DX	TC012945	Tribolium castaneum	Eukaryotic initiation factor 4E
EOG091204DX	LDEC006233	Leptinotarsa decemlineata	Eukaryotic initiation factor 4E
EOG091204DX	YQE_07070	Dendroctonus ponderosae	Eukaryotic initiation factor 4E
EOG091204DZ	OTAU002969	Onthophagus taurus	Putative uncharacterized protein
EOG091204DZ	AGLA020570	Anoplophora glabripennis	Putative uncharacterized protein
EOG091204DZ	APLA012594	Agrilus planipennis	Putative uncharacterized protein
EOG091204DZ	TC030601	Tribolium castaneum	Putative uncharacterized protein
EOG091204DZ	LDEC013872	Leptinotarsa decemlineata	Putative uncharacterized protein
EOG091204DZ	YQE_12945	Dendroctonus ponderosae	Putative uncharacterized protein
EOG091204E0	OTAU010923	Onthophagus taurus	Putative uncharacterized protein
EOG091204E0	AGLA003334	Anoplophora glabripennis	Putative uncharacterized protein
EOG091204E0	APLA014381	Agrilus planipennis	Putative uncharacterized protein
EOG091204E0	TC015732	Tribolium castaneum	Putative uncharacterized protein
EOG091204E0	LDEC007885	Leptinotarsa decemlineata	Putative uncharacterized protein
EOG091204E0	YQE_08459	Dendroctonus ponderosae	Putative uncharacterized protein
EOG091204E1	OTAU003858	Onthophagus taurus	Putative uncharacterized protein
EOG091204E1	AGLA008251	Anoplophora glabripennis	Putative uncharacterized protein
EOG091204E1	APLA013309	Agrilus planipennis	Putative uncharacterized protein
EOG091204E1	TC008865	Tribolium castaneum	Putative uncharacterized protein
EOG091204E1	LDEC014260	Leptinotarsa decemlineata	Putative uncharacterized protein
EOG091204E1	YQE_13001	Dendroctonus ponderosae	Putative uncharacterized protein
EOG091204E2	OTAU010365	Onthophagus taurus	Putative uncharacterized protein
EOG091204E2	AGLA007050	Anoplophora glabripennis	Putative uncharacterized protein
EOG091204E2	APLA011924	Agrilus planipennis	Putative uncharacterized protein
EOG091204E2	TC000779	Tribolium castaneum	Putative uncharacterized protein
EOG091204E2	LDEC002574	Leptinotarsa decemlineata	Putative uncharacterized protein
EOG091204E2	YQE_07131	Dendroctonus ponderosae	Putative uncharacterized protein
EOG091204E6	OTAU012418	Onthophagus taurus	Putative uncharacterized protein
EOG091204E6	AGLA020710	Anoplophora glabripennis	Putative uncharacterized protein
EOG091204E6	APLA013212	Agrilus planipennis	Putative uncharacterized protein
EOG091204E6	TC009073	Tribolium castaneum	Putative uncharacterized protein
EOG091204E6	LDEC004785	Leptinotarsa decemlineata	Putative uncharacterized protein
EOG091204E6	YQE_02585	Dendroctonus ponderosae	Putative uncharacterized protein
EOG091204E8	OTAU006664	Onthophagus taurus	Putative uncharacterized protein
EOG091204E8	AGLA002015	Anoplophora glabripennis	Putative uncharacterized protein
EOG091204E8	APLA002825	Agrilus planipennis	Putative uncharacterized protein
EOG091204E8	TC003940	Tribolium castaneum	Putative uncharacterized protein
EOG091204E8	LDEC004585	Leptinotarsa decemlineata	Putative uncharacterized protein
EOG091204E8	YQE_12423	Dendroctonus ponderosae	Putative uncharacterized protein
EOG091204EB	OTAU016797	Onthophagus taurus	Putative uncharacterized protein
EOG091204EB	AGLA002749	Anoplophora glabripennis	Putative uncharacterized protein
EOG091204EB	APLA013881	Agrilus planipennis	Putative uncharacterized protein
EOG091204EB	TC009067	Tribolium castaneum	Putative uncharacterized protein
EOG091204EB	LDEC012808	Leptinotarsa decemlineata	Putative uncharacterized protein
EOG091204EB	YQE_12595	Dendroctonus ponderosae	Putative uncharacterized protein
EOG091204EC	OTAU011797	Onthophagus taurus	Frizzled 4
EOG091204EC	AGLA016594	Anoplophora glabripennis	Frizzled 4
EOG091204EC	APLA001158	Agrilus planipennis	Frizzled 4
EOG091204EC	TC006527	Tribolium castaneum	Frizzled 4
EOG091204EC	LDEC014936	Leptinotarsa decemlineata	Frizzled 4
EOG091204EC	YQE_12239	Dendroctonus ponderosae	Frizzled 4
EOG091204EE	OTAU008736	Onthophagus taurus	Putative uncharacterized protein
EOG091204EE	AGLA003351	Anoplophora glabripennis	Putative uncharacterized protein
EOG091204EE	APLA001853	Agrilus planipennis	Putative uncharacterized protein
EOG091204EE	TC015566	Tribolium castaneum	Putative uncharacterized protein
EOG091204EE	LDEC008622	Leptinotarsa decemlineata	Putative uncharacterized protein
EOG091204EE	YQE_11742	Dendroctonus ponderosae	Putative uncharacterized protein
EOG091204EH	OTAU003256	Onthophagus taurus	Cuticular protein analogous to peritrophins 1-H
EOG091204EH	AGLA009202	Anoplophora glabripennis	Cuticular protein analogous to peritrophins 1-H
EOG091204EH	APLA008873	Agrilus planipennis	Cuticular protein analogous to peritrophins 1-H

EOG091204EH	TC009894	Tribolium castaneum	Cuticular protein analogous to peritrophins 1-H
EOG091204EH	LDEC016579	Leptinotarsa decemlineata	Cuticular protein analogous to peritrophins 1-H
EOG091204EH	YQE_01930	Dendroctonus ponderosae	Cuticular protein analogous to peritrophins 1-H
EOG091204EI	OTAU001728	Onthophagus taurus	Putative uncharacterized protein
EOG091204EI	AGLA009725	Anoplophora glabripennis	Putative uncharacterized protein
EOG091204EI	APLA012288	Agrilus planipennis	Putative uncharacterized protein
EOG091204EI	TC006738	Tribolium castaneum	Putative uncharacterized protein
EOG091204EI	LDEC018486	Leptinotarsa decemlineata	Putative uncharacterized protein
EOG091204EI	YQE_12157	Dendroctonus ponderosae	Putative uncharacterized protein
EOG091204EK	OTAU009172	Onthophagus taurus	None
EOG091204EK	AGLA004050	Anoplophora glabripennis	None
EOG091204EK	APLA005993	Agrilus planipennis	None
EOG091204EK	TC034779	Tribolium castaneum	None
EOG091204EK	LDEC020396	Leptinotarsa decemlineata	None
EOG091204EK	YQE_10298	Dendroctonus ponderosae	None
EOG091204EN	OTAU002548	Onthophagus taurus	Putative uncharacterized protein
EOG091204EN	AGLA019570	Anoplophora glabripennis	Putative uncharacterized protein
EOG091204EN	APLA009762	Agrilus planipennis	Putative uncharacterized protein
EOG091204EN	TC006746	Tribolium castaneum	Putative uncharacterized protein
EOG091204EN	LDEC009325	Leptinotarsa decemlineata	Putative uncharacterized protein
EOG091204EN	YQE_05772	Dendroctonus ponderosae	Putative uncharacterized protein
EOG091204ER	OTAU002813	Onthophagus taurus	Putative uncharacterized protein
EOG091204ER	AGLA002146	Anoplophora glabripennis	Putative uncharacterized protein
EOG091204ER	APLA006738	Agrilus planipennis	Putative uncharacterized protein
EOG091204ER	TC014238	Tribolium castaneum	Putative uncharacterized protein
EOG091204ER	LDEC019097	Leptinotarsa decemlineata	Putative uncharacterized protein
EOG091204ER	YQE_02498	Dendroctonus ponderosae	Putative uncharacterized protein
EOG091204ET	OTAU000247	Onthophagus taurus	Putative uncharacterized protein
EOG091204ET	AGLA010610	Anoplophora glabripennis	Putative uncharacterized protein
EOG091204ET	APLA002866	Agrilus planipennis	Putative uncharacterized protein
EOG091204ET	TC013984	Tribolium castaneum	Putative uncharacterized protein
EOG091204ET	LDEC015123	Leptinotarsa decemlineata	Putative uncharacterized protein
EOG091204ET	YQE_03608	Dendroctonus ponderosae	Putative uncharacterized protein
EOG091204EW	OTAU003828	Onthophagus taurus	"phosphotransferase activity, for other substituted phosphate groups"
EOG091204EW	AGLA003860	Anoplophora glabripennis	"phosphotransferase activity, for other substituted phosphate groups"
EOG091204EW	APLA001659	Agrilus planipennis	"phosphotransferase activity, for other substituted phosphate groups"
EOG091204EW	TC033796	Tribolium castaneum	"phosphotransferase activity, for other substituted phosphate groups"
EOG091204EW	LDEC020032	Leptinotarsa decemlineata	"phosphotransferase activity, for other substituted phosphate groups"
EOG091204EW	YQE_13053	Dendroctonus ponderosae	"phosphotransferase activity, for other substituted phosphate groups"
EOG091204EX	OTAU007981	Onthophagus taurus	Putative uncharacterized protein
EOG091204EX	AGLA011247	Anoplophora glabripennis	Putative uncharacterized protein
EOG091204EX	APLA011181	Agrilus planipennis	Putative uncharacterized protein
EOG091204EX	TC002943	Tribolium castaneum	Putative uncharacterized protein
EOG091204EX	LDEC005002	Leptinotarsa decemlineata	Putative uncharacterized protein
EOG091204EX	YQE_12348	Dendroctonus ponderosae	Putative uncharacterized protein
EOG091204EY	OTAU002203	Onthophagus taurus	Putative uncharacterized protein
EOG091204EY	AGLA003665	Anoplophora glabripennis	Putative uncharacterized protein
EOG091204EY	APLA008708	Agrilus planipennis	Putative uncharacterized protein
EOG091204EY	TC002842	Tribolium castaneum	Putative uncharacterized protein
EOG091204EY	LDEC009797	Leptinotarsa decemlineata	Putative uncharacterized protein
EOG091204EY	YQE_05196	Dendroctonus ponderosae	Putative uncharacterized protein
EOG091204F2	OTAU000151	Onthophagus taurus	Putative uncharacterized protein
EOG091204F2	AGLA012787	Anoplophora glabripennis	Putative uncharacterized protein
EOG091204F2	APLA008535	Agrilus planipennis	Putative uncharacterized protein
EOG091204F2	TC014322	Tribolium castaneum	Putative uncharacterized protein
EOG091204F2	LDEC016470	Leptinotarsa decemlineata	Putative uncharacterized protein
EOG091204F2	YQE_02681	Dendroctonus ponderosae	Putative uncharacterized protein
EOG091204F3	OTAU003867	Onthophagus taurus	metal ion binding
EOG091204F3	AGLA005770	Anoplophora glabripennis	metal ion binding
EOG091204F3	APLA009328	Agrilus planipennis	metal ion binding
EOG091204F3	TC034798	Tribolium castaneum	metal ion binding

EOG091204F3	LDEC011890	Leptinotarsa decemlineata	metal ion binding
EOG091204F3	YQE_02337	Dendroctonus ponderosae	metal ion binding
EOG091204F4	OTAU001770	Onthophagus taurus	Putative uncharacterized protein
EOG091204F4	AGLA010193	Anoplophora glabripennis	Putative uncharacterized protein
EOG091204F4	APLA009862	Agrilus planipennis	Putative uncharacterized protein
EOG091204F4	TC005341	Tribolium castaneum	Putative uncharacterized protein
EOG091204F4	LDEC006774	Leptinotarsa decemlineata	Putative uncharacterized protein
EOG091204F4	YQE_12148	Dendroctonus ponderosae	Putative uncharacterized protein
EOG091204F5	OTAU002044	Onthophagus taurus	Putative uncharacterized protein
EOG091204F5	AGLA017464	Anoplophora glabripennis	Putative uncharacterized protein
EOG091204F5	APLA003223	Agrilus planipennis	Putative uncharacterized protein
EOG091204F5	TC001674	Tribolium castaneum	Putative uncharacterized protein
EOG091204F5	LDEC015589	Leptinotarsa decemlineata	Putative uncharacterized protein
EOG091204F5	YQE_06510	Dendroctonus ponderosae	Putative uncharacterized protein
EOG091204F7	OTAU014451	Onthophagus taurus	Putative uncharacterized protein
EOG091204F7	AGLA016710	Anoplophora glabripennis	Putative uncharacterized protein
EOG091204F7	APLA006864	Agrilus planipennis	Putative uncharacterized protein
EOG091204F7	TC013090	Tribolium castaneum	Putative uncharacterized protein
EOG091204F7	LDEC015564	Leptinotarsa decemlineata	Putative uncharacterized protein
EOG091204F7	YQE_08310	Dendroctonus ponderosae	Putative uncharacterized protein
EOG091204F8	OTAU015902	Onthophagus taurus	Putative uncharacterized protein
EOG091204F8	AGLA002186	Anoplophora glabripennis	Putative uncharacterized protein
EOG091204F8	APLA010368	Agrilus planipennis	Putative uncharacterized protein
EOG091204F8	TC001699	Tribolium castaneum	Putative uncharacterized protein
EOG091204F8	LDEC001564	Leptinotarsa decemlineata	Putative uncharacterized protein
EOG091204F8	YQE_06653	Dendroctonus ponderosae	Putative uncharacterized protein
EOG091204FB	OTAU001535	Onthophagus taurus	Putative uncharacterized protein
EOG091204FB	AGLA018806	Anoplophora glabripennis	Putative uncharacterized protein
EOG091204FB	APLA008461	Agrilus planipennis	Putative uncharacterized protein
EOG091204FB	TC005551	Tribolium castaneum	Putative uncharacterized protein
EOG091204FB	LDEC010099	Leptinotarsa decemlineata	Putative uncharacterized protein
EOG091204FB	YQE_10172	Dendroctonus ponderosae	Putative uncharacterized protein
EOG091204FG	OTAU007896	Onthophagus taurus	Rab-protein 5
EOG091204FG	AGLA011715	Anoplophora glabripennis	Rab-protein 5
EOG091204FG	APLA013899	Agrilus planipennis	Rab-protein 5
EOG091204FG	TC014786	Tribolium castaneum	Rab-protein 5
EOG091204FG	LDEC007224	Leptinotarsa decemlineata	Rab-protein 5
EOG091204FG	YQE_07235	Dendroctonus ponderosae	Rab-protein 5
EOG091204FH	OTAU002089	Onthophagus taurus	Putative uncharacterized protein
EOG091204FH	AGLA003302	Anoplophora glabripennis	Putative uncharacterized protein
EOG091204FH	APLA013844	Agrilus planipennis	Putative uncharacterized protein
EOG091204FH	TC011626	Tribolium castaneum	Putative uncharacterized protein
EOG091204FH	LDEC017533	Leptinotarsa decemlineata	Putative uncharacterized protein
EOG091204FH	YQE_07584	Dendroctonus ponderosae	Putative uncharacterized protein
EOG091204FI	OTAU003503	Onthophagus taurus	None
EOG091204FI	AGLA015207	Anoplophora glabripennis	None
EOG091204FI	APLA010071	Agrilus planipennis	None
EOG091204FI	TC033624	Tribolium castaneum	None
EOG091204FI	LDEC014451	Leptinotarsa decemlineata	None
EOG091204FI	YQE_13068	Dendroctonus ponderosae	None
EOG091204FJ	OTAU007171	Onthophagus taurus	Putative uncharacterized protein
EOG091204FJ	AGLA018376	Anoplophora glabripennis	Putative uncharacterized protein
EOG091204FJ	APLA011858	Agrilus planipennis	Putative uncharacterized protein
EOG091204FJ	TC012099	Tribolium castaneum	Putative uncharacterized protein
EOG091204FJ	LDEC002344	Leptinotarsa decemlineata	Putative uncharacterized protein
EOG091204FJ	YQE_02171	Dendroctonus ponderosae	Putative uncharacterized protein
EOG091204FP	OTAU009933	Onthophagus taurus	Putative uncharacterized protein
EOG091204FP	AGLA005081	Anoplophora glabripennis	Putative uncharacterized protein
EOG091204FP	APLA012178	Agrilus planipennis	Putative uncharacterized protein
EOG091204FP	TC002667	Tribolium castaneum	Putative uncharacterized protein
EOG091204FP	LDEC011376	Leptinotarsa decemlineata	Putative uncharacterized protein
EOG091204FP	YQE_11196	Dendroctonus ponderosae	Putative uncharacterized protein
EOG091204FS	OTAU001963	Onthophagus taurus	Putative uncharacterized protein
EOG091204FS	AGLA019713	Anoplophora glabripennis	Putative uncharacterized protein
EOG091204FS	APLA005802	Agrilus planipennis	Putative uncharacterized protein
EOG091204FS	TC030596	Tribolium castaneum	Putative uncharacterized protein
EOG091204FS	LDEC010108	Leptinotarsa decemlineata	Putative uncharacterized protein

EOG091204FS	YQE_12171	Dendroctonus ponderosae	Putative uncharacterized protein
EOG091204FU	OTAU015963	Onthophagus taurus	Putative uncharacterized protein
EOG091204FU	AGLA005726	Anoplophora glabripennis	Putative uncharacterized protein
EOG091204FU	APLA000562	Agrilus planipennis	Putative uncharacterized protein
EOG091204FU	TC015688	Tribolium castaneum	Putative uncharacterized protein
EOG091204FU	LDEC010959	Leptinotarsa decemlineata	Putative uncharacterized protein
EOG091204FU	YQE_11480	Dendroctonus ponderosae	Putative uncharacterized protein
EOG091204FX	OTAU011412	Onthophagus taurus	Putative uncharacterized protein
EOG091204FX	AGLA007300	Anoplophora glabripennis	Putative uncharacterized protein
EOG091204FX	APLA009757	Agrilus planipennis	Putative uncharacterized protein
EOG091204FX	TC006549	Tribolium castaneum	Putative uncharacterized protein
EOG091204FX	LDEC010512	Leptinotarsa decemlineata	Putative uncharacterized protein
EOG091204FX	YQE_06876	Dendroctonus ponderosae	Putative uncharacterized protein
EOG091204FY	OTAU003410	Onthophagus taurus	Putative uncharacterized protein
EOG091204FY	AGLA020985	Anoplophora glabripennis	Putative uncharacterized protein
EOG091204FY	APLA002356	Agrilus planipennis	Putative uncharacterized protein
EOG091204FY	TC012697	Tribolium castaneum	Putative uncharacterized protein
EOG091204FY	LDEC004126	Leptinotarsa decemlineata	Putative uncharacterized protein
EOG091204FY	YQE_10165	Dendroctonus ponderosae	Putative uncharacterized protein
EOG091204FZ	OTAU009475	Onthophagus taurus	Putative uncharacterized protein
EOG091204FZ	AGLA005727	Anoplophora glabripennis	Putative uncharacterized protein
EOG091204FZ	APLA000561	Agrilus planipennis	Putative uncharacterized protein
EOG091204FZ	TC014996	Tribolium castaneum	Putative uncharacterized protein
EOG091204FZ	LDEC010958	Leptinotarsa decemlineata	Putative uncharacterized protein
EOG091204FZ	YQE_11481	Dendroctonus ponderosae	Putative uncharacterized protein
EOG091204G7	OTAU000889	Onthophagus taurus	Putative uncharacterized protein
EOG091204G7	AGLA002162	Anoplophora glabripennis	Putative uncharacterized protein
EOG091204G7	APLA005203	Agrilus planipennis	Putative uncharacterized protein
EOG091204G7	TC000613	Tribolium castaneum	Putative uncharacterized protein
EOG091204G7	LDEC008055	Leptinotarsa decemlineata	Putative uncharacterized protein
EOG091204G7	YQE_06645	Dendroctonus ponderosae	Putative uncharacterized protein
EOG091204GD	OTAU002164	Onthophagus taurus	Putative uncharacterized protein
EOG091204GD	AGLA021488	Anoplophora glabripennis	Putative uncharacterized protein
EOG091204GD	APLA013195	Agrilus planipennis	Putative uncharacterized protein
EOG091204GD	TC003351	Tribolium castaneum	Putative uncharacterized protein
EOG091204GD	LDEC011106	Leptinotarsa decemlineata	Putative uncharacterized protein
EOG091204GD	YQE_02186	Dendroctonus ponderosae	Putative uncharacterized protein
EOG091204GF	OTAU009875	Onthophagus taurus	Uridine kinase
EOG091204GF	AGLA000147	Anoplophora glabripennis	Uridine kinase
EOG091204GF	APLA008439	Agrilus planipennis	Uridine kinase
EOG091204GF	TC000160	Tribolium castaneum	Uridine kinase
EOG091204GF	LDEC013483	Leptinotarsa decemlineata	Uridine kinase
EOG091204GF	YQE_10998	Dendroctonus ponderosae	Uridine kinase
EOG091204GG	OTAU001533	Onthophagus taurus	Putative uncharacterized protein
EOG091204GG	AGLA009099	Anoplophora glabripennis	Putative uncharacterized protein
EOG091204GG	APLA013155	Agrilus planipennis	Putative uncharacterized protein
EOG091204GG	TC005831	Tribolium castaneum	Putative uncharacterized protein
EOG091204GG	LDEC016208	Leptinotarsa decemlineata	Putative uncharacterized protein
EOG091204GG	YQE_12007	Dendroctonus ponderosae	Putative uncharacterized protein
EOG091204GJ	OTAU003596	Onthophagus taurus	Putative uncharacterized protein
EOG091204GJ	AGLA008545	Anoplophora glabripennis	Putative uncharacterized protein
EOG091204GJ	APLA012525	Agrilus planipennis	Putative uncharacterized protein
EOG091204GJ	TC003246	Tribolium castaneum	Putative uncharacterized protein
EOG091204GJ	LDEC002016	Leptinotarsa decemlineata	Putative uncharacterized protein
EOG091204GJ	YQE_04957	Dendroctonus ponderosae	Putative uncharacterized protein
EOG091204GK	OTAU013239	Onthophagus taurus	Putative uncharacterized protein
EOG091204GK	AGLA011153	Anoplophora glabripennis	Putative uncharacterized protein
EOG091204GK	APLA007891	Agrilus planipennis	Putative uncharacterized protein
EOG091204GK	TC009996	Tribolium castaneum	Putative uncharacterized protein
EOG091204GK	LDEC013398	Leptinotarsa decemlineata	Putative uncharacterized protein
EOG091204GK	YQE_08093	Dendroctonus ponderosae	Putative uncharacterized protein
EOG091204GL	OTAU000747	Onthophagus taurus	Putative uncharacterized protein
EOG091204GL	AGLA017188	Anoplophora glabripennis	Putative uncharacterized protein
EOG091204GL	APLA002352	Agrilus planipennis	Putative uncharacterized protein
EOG091204GL	TC012682	Tribolium castaneum	Putative uncharacterized protein
EOG091204GL	LDEC015688	Leptinotarsa decemlineata	Putative uncharacterized protein
EOG091204GL	YQE_11240	Dendroctonus ponderosae	Putative uncharacterized protein

EOG091204GN	OTAU009514	Onthophagus taurus	Rho1
EOG091204GN	AGLA002801	Anoplophora glabripennis	Rho1
EOG091204GN	APLA007155	Agrilus planipennis	Rho1
EOG091204GN	TC009158	Tribolium castaneum	Rho1
EOG091204GN	LDEC014721	Leptinotarsa decemlineata	Rho1
EOG091204GN	YQE_11938	Dendroctonus ponderosae	Rho1
EOG091204GP	OTAU002639	Onthophagus taurus	Putative uncharacterized protein
EOG091204GP	AGLA008438	Anoplophora glabripennis	Putative uncharacterized protein
EOG091204GP	APLA002460	Agrilus planipennis	Putative uncharacterized protein
EOG091204GP	TC013692	Tribolium castaneum	Putative uncharacterized protein
EOG091204GP	LDEC021242	Leptinotarsa decemlineata	Putative uncharacterized protein
EOG091204GP	YQE_03797	Dendroctonus ponderosae	Putative uncharacterized protein
EOG091204GU	OTAU003752	Onthophagus taurus	Putative uncharacterized protein
EOG091204GU	AGLA006471	Anoplophora glabripennis	Putative uncharacterized protein
EOG091204GU	APLA008813	Agrilus planipennis	Putative uncharacterized protein
EOG091204GU	TC005498	Tribolium castaneum	Putative uncharacterized protein
EOG091204GU	LDEC004805	Leptinotarsa decemlineata	Putative uncharacterized protein
EOG091204GU	YQE_12491	Dendroctonus ponderosae	Putative uncharacterized protein
EOG091204GY	OTAU000958	Onthophagus taurus	Elbow B
EOG091204GY	AGLA021634	Anoplophora glabripennis	Elbow B
EOG091204GY	APLA004363	Agrilus planipennis	Elbow B
EOG091204GY	TC000868	Tribolium castaneum	Elbow B
EOG091204GY	LDEC002879	Leptinotarsa decemlineata	Elbow B
EOG091204GY	YQE_02795	Dendroctonus ponderosae	Elbow B
EOG091204GZ	OTAU001756	Onthophagus taurus	Putative uncharacterized protein
EOG091204GZ	AGLA004958	Anoplophora glabripennis	Putative uncharacterized protein
EOG091204GZ	APLA000510	Agrilus planipennis	Putative uncharacterized protein
EOG091204GZ	TC005845	Tribolium castaneum	Putative uncharacterized protein
EOG091204GZ	LDEC008165	Leptinotarsa decemlineata	Putative uncharacterized protein
EOG091204GZ	YQE_05578	Dendroctonus ponderosae	Putative uncharacterized protein
EOG091204H0	OTAU010221	Onthophagus taurus	DNA binding
EOG091204H0	AGLA007546	Anoplophora glabripennis	DNA binding
EOG091204H0	APLA007802	Agrilus planipennis	DNA binding
EOG091204H0	TC031040	Tribolium castaneum	DNA binding
EOG091204H0	LDEC015753	Leptinotarsa decemlineata	DNA binding
EOG091204H0	YQE_07795	Dendroctonus ponderosae	DNA binding
EOG091204H1	OTAU004143	Onthophagus taurus	Putative uncharacterized protein
EOG091204H1	AGLA009453	Anoplophora glabripennis	Putative uncharacterized protein
EOG091204H1	APLA007052	Agrilus planipennis	Putative uncharacterized protein
EOG091204H1	TC009851	Tribolium castaneum	Putative uncharacterized protein
EOG091204H1	LDEC024607	Leptinotarsa decemlineata	Putative uncharacterized protein
EOG091204H1	YQE_12448	Dendroctonus ponderosae	Putative uncharacterized protein
EOG091204H2	OTAU000338	Onthophagus taurus	Putative uncharacterized protein
EOG091204H2	AGLA011336	Anoplophora glabripennis	Putative uncharacterized protein
EOG091204H2	APLA007392	Agrilus planipennis	Putative uncharacterized protein
EOG091204H2	TC012534	Tribolium castaneum	Putative uncharacterized protein
EOG091204H2	LDEC005750	Leptinotarsa decemlineata	Putative uncharacterized protein
EOG091204H2	YQE_12884	Dendroctonus ponderosae	Putative uncharacterized protein
EOG091204H6	OTAU016337	Onthophagus taurus	Putative uncharacterized protein
EOG091204H6	AGLA003008	Anoplophora glabripennis	Putative uncharacterized protein
EOG091204H6	APLA013465	Agrilus planipennis	Putative uncharacterized protein
EOG091204H6	TC001143	Tribolium castaneum	Putative uncharacterized protein
EOG091204H6	LDEC014166	Leptinotarsa decemlineata	Putative uncharacterized protein
EOG091204H6	YQE_01654	Dendroctonus ponderosae	Putative uncharacterized protein
EOG091204H8	OTAU015622	Onthophagus taurus	Putative uncharacterized protein
EOG091204H8	AGLA012028	Anoplophora glabripennis	Putative uncharacterized protein
EOG091204H8	APLA008536	Agrilus planipennis	Putative uncharacterized protein
EOG091204H8	TC014689	Tribolium castaneum	Putative uncharacterized protein
EOG091204H8	LDEC020597	Leptinotarsa decemlineata	Putative uncharacterized protein
EOG091204H8	YQE_07534	Dendroctonus ponderosae	Putative uncharacterized protein
EOG091204HA	OTAU009488	Onthophagus taurus	nucleotide binding
EOG091204HA	AGLA012748	Anoplophora glabripennis	nucleotide binding
EOG091204HA	APLA002567	Agrilus planipennis	nucleotide binding
EOG091204HA	TC034818	Tribolium castaneum	nucleotide binding
EOG091204HA	LDEC024050	Leptinotarsa decemlineata	nucleotide binding
EOG091204HA	YQE_08397	Dendroctonus ponderosae	nucleotide binding
EOG091204HC	OTAU009340	Onthophagus taurus	Putative uncharacterized protein

EOG091204HC	AGLA014563	Anoplophora glabripennis	Putative uncharacterized protein
EOG091204HC	APLA012891	Agrilus planipennis	Putative uncharacterized protein
EOG091204HC	TC013489	Tribolium castaneum	Putative uncharacterized protein
EOG091204HC	LDEC021620	Leptinotarsa decemlineata	Putative uncharacterized protein
EOG091204HC	YQE_02695	Dendroctonus ponderosae	Putative uncharacterized protein
EOG091204HD	OTAU000564	Onthophagus taurus	Putative uncharacterized protein
EOG091204HD	AGLA017200	Anoplophora glabripennis	Putative uncharacterized protein
EOG091204HD	APLA012508	Agrilus planipennis	Putative uncharacterized protein
EOG091204HD	TC012307	Tribolium castaneum	Putative uncharacterized protein
EOG091204HD	LDEC003868	Leptinotarsa decemlineata	Putative uncharacterized protein
EOG091204HD	YQE_04768	Dendroctonus ponderosae	Putative uncharacterized protein
EOG091204HE	OTAU002833	Onthophagus taurus	Putative uncharacterized protein
EOG091204HE	AGLA004551	Anoplophora glabripennis	Putative uncharacterized protein
EOG091204HE	APLA006361	Agrilus planipennis	Putative uncharacterized protein
EOG091204HE	TC014165	Tribolium castaneum	Putative uncharacterized protein
EOG091204HE	LDEC004114	Leptinotarsa decemlineata	Putative uncharacterized protein
EOG091204HE	YQE_02502	Dendroctonus ponderosae	Putative uncharacterized protein
EOG091204HF	OTAU011610	Onthophagus taurus	Putative uncharacterized protein
EOG091204HF	AGLA018704	Anoplophora glabripennis	Putative uncharacterized protein
EOG091204HF	APLA013233	Agrilus planipennis	Putative uncharacterized protein
EOG091204HF	TC009380	Tribolium castaneum	Putative uncharacterized protein
EOG091204HF	LDEC022821	Leptinotarsa decemlineata	Putative uncharacterized protein
EOG091204HF	YQE_13027	Dendroctonus ponderosae	Putative uncharacterized protein
EOG091204HG	OTAU004140	Onthophagus taurus	Putative uncharacterized protein
EOG091204HG	AGLA008647	Anoplophora glabripennis	Putative uncharacterized protein
EOG091204HG	APLA014037	Agrilus planipennis	Putative uncharacterized protein
EOG091204HG	TC010003	Tribolium castaneum	Putative uncharacterized protein
EOG091204HG	LDEC001347	Leptinotarsa decemlineata	Putative uncharacterized protein
EOG091204HG	YQE_04088	Dendroctonus ponderosae	Putative uncharacterized protein
EOG091204HJ	OTAU005643	Onthophagus taurus	Putative uncharacterized protein
EOG091204HJ	AGLA011774	Anoplophora glabripennis	Putative uncharacterized protein
EOG091204HJ	APLA015161	Agrilus planipennis	Putative uncharacterized protein
EOG091204HJ	TC009771	Tribolium castaneum	Putative uncharacterized protein
EOG091204HJ	LDEC004446	Leptinotarsa decemlineata	Putative uncharacterized protein
EOG091204HJ	YQE_04296	Dendroctonus ponderosae	Putative uncharacterized protein
EOG091204HK	OTAU004962	Onthophagus taurus	hydrolase activity
EOG091204HK	AGLA016801	Anoplophora glabripennis	hydrolase activity
EOG091204HK	APLA009411	Agrilus planipennis	hydrolase activity
EOG091204HK	TC032269	Tribolium castaneum	hydrolase activity
EOG091204HK	LDEC001755	Leptinotarsa decemlineata	hydrolase activity
EOG091204HK	YQE_03572	Dendroctonus ponderosae	hydrolase activity
EOG091204HL	OTAU012540	Onthophagus taurus	Putative uncharacterized protein
EOG091204HL	AGLA017228	Anoplophora glabripennis	Putative uncharacterized protein
EOG091204HL	APLA002640	Agrilus planipennis	Putative uncharacterized protein
EOG091204HL	TC002259	Tribolium castaneum	Putative uncharacterized protein
EOG091204HL	LDEC003859	Leptinotarsa decemlineata	Putative uncharacterized protein
EOG091204HL	YQE_04311	Dendroctonus ponderosae	Putative uncharacterized protein
EOG091204HM	OTAU006048	Onthophagus taurus	Putative uncharacterized protein
EOG091204HM	AGLA005153	Anoplophora glabripennis	Putative uncharacterized protein
EOG091204HM	APLA005662	Agrilus planipennis	Putative uncharacterized protein
EOG091204HM	TC007377	Tribolium castaneum	Putative uncharacterized protein
EOG091204HM	LDEC012408	Leptinotarsa decemlineata	Putative uncharacterized protein
EOG091204HM	YQE_00070	Dendroctonus ponderosae	Putative uncharacterized protein
EOG091204HW	OTAU004703	Onthophagus taurus	Putative uncharacterized protein
EOG091204HW	AGLA012321	Anoplophora glabripennis	Putative uncharacterized protein
EOG091204HW	APLA001763	Agrilus planipennis	Putative uncharacterized protein
EOG091204HW	TC012213	Tribolium castaneum	Putative uncharacterized protein
EOG091204HW	LDEC020791	Leptinotarsa decemlineata	Putative uncharacterized protein
EOG091204HW	YQE_07606	Dendroctonus ponderosae	Putative uncharacterized protein
EOG091204HY	OTAU007931	Onthophagus taurus	Proteasome subunit beta type
EOG091204HY	AGLA005854	Anoplophora glabripennis	Proteasome subunit beta type
EOG091204HY	APLA008836	Agrilus planipennis	Proteasome subunit beta type
EOG091204HY	TC008617	Tribolium castaneum	Proteasome subunit beta type
EOG091204HY	LDEC011907	Leptinotarsa decemlineata	Proteasome subunit beta type
EOG091204HY	YQE_03511	Dendroctonus ponderosae	Proteasome subunit beta type
EOG091204HZ	OTAU006685	Onthophagus taurus	Putative uncharacterized protein
EOG091204HZ	AGLA004219	Anoplophora glabripennis	Putative uncharacterized protein

EOG091204HZ	APLA007228	Agrilus planipennis	Putative uncharacterized protein
EOG091204HZ	TC002405	Tribolium castaneum	Putative uncharacterized protein
EOG091204HZ	LDEC004894	Leptinotarsa decemlineata	Putative uncharacterized protein
EOG091204HZ	YQE_09999	Dendroctonus ponderosae	Putative uncharacterized protein
EOG091204I0	OTAU011858	Onthophagus taurus	Putative uncharacterized protein
EOG091204I0	AGLA013427	Anoplophora glabripennis	Putative uncharacterized protein
EOG091204I0	APLA002890	Agrilus planipennis	Putative uncharacterized protein
EOG091204I0	TC013041	Tribolium castaneum	Putative uncharacterized protein
EOG091204I0	LDEC001173	Leptinotarsa decemlineata	Putative uncharacterized protein
EOG091204I0	YQE_04012	Dendroctonus ponderosae	Putative uncharacterized protein
EOG091204I1	OTAU000642	Onthophagus taurus	Putative uncharacterized protein
EOG091204I1	AGLA020410	Anoplophora glabripennis	Putative uncharacterized protein
EOG091204I1	APLA010057	Agrilus planipennis	Putative uncharacterized protein
EOG091204I1	TC012119	Tribolium castaneum	Putative uncharacterized protein
EOG091204I1	LDEC021181	Leptinotarsa decemlineata	Putative uncharacterized protein
EOG091204I1	YQE_10078	Dendroctonus ponderosae	Putative uncharacterized protein
EOG091204I2	OTAU013082	Onthophagus taurus	Putative uncharacterized protein
EOG091204I2	AGLA013160	Anoplophora glabripennis	Putative uncharacterized protein
EOG091204I2	APLA009072	Agrilus planipennis	Putative uncharacterized protein
EOG091204I2	TC002174	Tribolium castaneum	Putative uncharacterized protein
EOG091204I2	LDEC013537	Leptinotarsa decemlineata	Putative uncharacterized protein
EOG091204I2	YQE_07522	Dendroctonus ponderosae	Putative uncharacterized protein
EOG091204I3	OTAU009756	Onthophagus taurus	Putative uncharacterized protein
EOG091204I3	AGLA017991	Anoplophora glabripennis	Putative uncharacterized protein
EOG091204I3	APLA011778	Agrilus planipennis	Putative uncharacterized protein
EOG091204I3	TC011518	Tribolium castaneum	Putative uncharacterized protein
EOG091204I3	LDEC003902	Leptinotarsa decemlineata	Putative uncharacterized protein
EOG091204I3	YQE_01296	Dendroctonus ponderosae	Putative uncharacterized protein
EOG091204I5	OTAU009642	Onthophagus taurus	Putative uncharacterized protein
EOG091204I5	AGLA007778	Anoplophora glabripennis	Putative uncharacterized protein
EOG091204I5	APLA001291	Agrilus planipennis	Putative uncharacterized protein
EOG091204I5	TC000326	Tribolium castaneum	Putative uncharacterized protein
EOG091204I5	LDEC020921	Leptinotarsa decemlineata	Putative uncharacterized protein
EOG091204I5	YQE_09589	Dendroctonus ponderosae	Putative uncharacterized protein
EOG091204I8	OTAU008968	Onthophagus taurus	Putative uncharacterized protein
EOG091204I8	AGLA006208	Anoplophora glabripennis	Putative uncharacterized protein
EOG091204I8	APLA014441	Agrilus planipennis	Putative uncharacterized protein
EOG091204I8	TC004029	Tribolium castaneum	Putative uncharacterized protein
EOG091204I8	LDEC017202	Leptinotarsa decemlineata	Putative uncharacterized protein
EOG091204I8	YQE_05882	Dendroctonus ponderosae	Putative uncharacterized protein
EOG091204IA	OTAU001562	Onthophagus taurus	Putative uncharacterized protein
EOG091204IA	AGLA010680	Anoplophora glabripennis	Putative uncharacterized protein
EOG091204IA	APLA013666	Agrilus planipennis	Putative uncharacterized protein
EOG091204IA	TC005503	Tribolium castaneum	Putative uncharacterized protein
EOG091204IA	LDEC021858	Leptinotarsa decemlineata	Putative uncharacterized protein
EOG091204IA	YQE_12136	Dendroctonus ponderosae	Putative uncharacterized protein
EOG091204IC	OTAU010933	Onthophagus taurus	Putative uncharacterized protein
EOG091204IC	AGLA013832	Anoplophora glabripennis	Putative uncharacterized protein
EOG091204IC	APLA015235	Agrilus planipennis	Putative uncharacterized protein
EOG091204IC	TC015302	Tribolium castaneum	Putative uncharacterized protein
EOG091204IC	LDEC001812	Leptinotarsa decemlineata	Putative uncharacterized protein
EOG091204IC	YQE_05923	Dendroctonus ponderosae	Putative uncharacterized protein
EOG091204IG	OTAU004244	Onthophagus taurus	Putative uncharacterized protein
EOG091204IG	AGLA013067	Anoplophora glabripennis	Putative uncharacterized protein
EOG091204IG	APLA001143	Agrilus planipennis	Putative uncharacterized protein
EOG091204IG	TC013156	Tribolium castaneum	Putative uncharacterized protein
EOG091204IG	LDEC017297	Leptinotarsa decemlineata	Putative uncharacterized protein
EOG091204IG	YQE_08573	Dendroctonus ponderosae	Putative uncharacterized protein
EOG091204IK	OTAU012893	Onthophagus taurus	Putative uncharacterized protein
EOG091204IK	AGLA012551	Anoplophora glabripennis	Putative uncharacterized protein
EOG091204IK	APLA007635	Agrilus planipennis	Putative uncharacterized protein
EOG091204IK	TC016337	Tribolium castaneum	Putative uncharacterized protein
EOG091204IK	LDEC012355	Leptinotarsa decemlineata	Putative uncharacterized protein
EOG091204IK	YQE_03831	Dendroctonus ponderosae	Putative uncharacterized protein
EOG091204IL	OTAU009128	Onthophagus taurus	Putative uncharacterized protein
EOG091204IL	AGLA012043	Anoplophora glabripennis	Putative uncharacterized protein
EOG091204IL	APLA008519	Agrilus planipennis	Putative uncharacterized protein

EOG091204IL	TC013564	Tribolium castaneum	Putative uncharacterized protein
EOG091204IL	LDEC010527	Leptinotarsa decemlineata	Putative uncharacterized protein
EOG091204IL	YQE_10353	Dendroctonus ponderosae	Putative uncharacterized protein
EOG091204IN	OTAU012896	Onthophagus taurus	Putative uncharacterized protein
EOG091204IN	AGLA016539	Anoplophora glabripennis	Putative uncharacterized protein
EOG091204IN	APLA015352	Agrilus planipennis	Putative uncharacterized protein
EOG091204IN	TC016226	Tribolium castaneum	Putative uncharacterized protein
EOG091204IN	LDEC005594	Leptinotarsa decemlineata	Putative uncharacterized protein
EOG091204IN	YQE_10765	Dendroctonus ponderosae	Putative uncharacterized protein
EOG091204IO	OTAU006377	Onthophagus taurus	Putative uncharacterized protein
EOG091204IO	AGLA006494	Anoplophora glabripennis	Putative uncharacterized protein
EOG091204IO	APLA014044	Agrilus planipennis	Putative uncharacterized protein
EOG091204IO	TC008666	Tribolium castaneum	Putative uncharacterized protein
EOG091204IO	LDEC012447	Leptinotarsa decemlineata	Putative uncharacterized protein
EOG091204IO	YQE_00083	Dendroctonus ponderosae	Putative uncharacterized protein
EOG091204IT	OTAU011599	Onthophagus taurus	None
EOG091204IT	AGLA013512	Anoplophora glabripennis	None
EOG091204IT	APLA000569	Agrilus planipennis	None
EOG091204IT	TC030900	Tribolium castaneum	None
EOG091204IT	LDEC003697	Leptinotarsa decemlineata	None
EOG091204IT	YQE_02939	Dendroctonus ponderosae	None
EOG091204IZ	OTAU011870	Onthophagus taurus	Putative uncharacterized protein
EOG091204IZ	AGLA004121	Anoplophora glabripennis	Putative uncharacterized protein
EOG091204IZ	APLA007614	Agrilus planipennis	Putative uncharacterized protein
EOG091204IZ	TC030670	Tribolium castaneum	Putative uncharacterized protein
EOG091204IZ	LDEC009630	Leptinotarsa decemlineata	Putative uncharacterized protein
EOG091204IZ	YQE_10925	Dendroctonus ponderosae	Putative uncharacterized protein
EOG091204J0	OTAU015813	Onthophagus taurus	Dissatisfaction
EOG091204J0	AGLA006678	Anoplophora glabripennis	Dissatisfaction
EOG091204J0	APLA001905	Agrilus planipennis	Dissatisfaction
EOG091204J0	TC001069	Tribolium castaneum	Dissatisfaction
EOG091204J0	LDEC003556	Leptinotarsa decemlineata	Dissatisfaction
EOG091204J0	YQE_10678	Dendroctonus ponderosae	Dissatisfaction
EOG091204J6	OTAU000683	Onthophagus taurus	Putative uncharacterized protein
EOG091204J6	AGLA008122	Anoplophora glabripennis	Putative uncharacterized protein
EOG091204J6	APLA014952	Agrilus planipennis	Putative uncharacterized protein
EOG091204J6	TC011768	Tribolium castaneum	Putative uncharacterized protein
EOG091204J6	LDEC016256	Leptinotarsa decemlineata	Putative uncharacterized protein
EOG091204J6	YQE_06504	Dendroctonus ponderosae	Putative uncharacterized protein
EOG091204J7	OTAU002683	Onthophagus taurus	Putative uncharacterized protein
EOG091204J7	AGLA010823	Anoplophora glabripennis	Putative uncharacterized protein
EOG091204J7	APLA003611	Agrilus planipennis	Putative uncharacterized protein
EOG091204J7	TC013737	Tribolium castaneum	Putative uncharacterized protein
EOG091204J7	LDEC007265	Leptinotarsa decemlineata	Putative uncharacterized protein
EOG091204J7	YQE_06323	Dendroctonus ponderosae	Putative uncharacterized protein
EOG091204JA	OTAU000555	Onthophagus taurus	lipid binding
EOG091204JA	AGLA005533	Anoplophora glabripennis	lipid binding
EOG091204JA	APLA011782	Agrilus planipennis	lipid binding
EOG091204JA	TC034248	Tribolium castaneum	lipid binding
EOG091204JA	LDEC011141	Leptinotarsa decemlineata	lipid binding
EOG091204JA	YQE_07941	Dendroctonus ponderosae	lipid binding
EOG091204JC	OTAU005100	Onthophagus taurus	Putative uncharacterized protein
EOG091204JC	AGLA010699	Anoplophora glabripennis	Putative uncharacterized protein
EOG091204JC	APLA011064	Agrilus planipennis	Putative uncharacterized protein
EOG091204JC	TC005781	Tribolium castaneum	Putative uncharacterized protein
EOG091204JC	LDEC004912	Leptinotarsa decemlineata	Putative uncharacterized protein
EOG091204JC	YQE_05653	Dendroctonus ponderosae	Putative uncharacterized protein
EOG091204JD	OTAU005292	Onthophagus taurus	Putative uncharacterized protein
EOG091204JD	AGLA000522	Anoplophora glabripennis	Putative uncharacterized protein
EOG091204JD	APLA002282	Agrilus planipennis	Putative uncharacterized protein
EOG091204JD	TC007393	Tribolium castaneum	Putative uncharacterized protein
EOG091204JD	LDEC006835	Leptinotarsa decemlineata	Putative uncharacterized protein
EOG091204JD	YQE_04750	Dendroctonus ponderosae	Putative uncharacterized protein
EOG091204JE	OTAU005019	Onthophagus taurus	Putative uncharacterized protein
EOG091204JE	AGLA005980	Anoplophora glabripennis	Putative uncharacterized protein
EOG091204JE	APLA000902	Agrilus planipennis	Putative uncharacterized protein
EOG091204JE	TC015243	Tribolium castaneum	Putative uncharacterized protein

EOG091204JE	LDEC015404	Leptinotarsa decemlineata	Putative uncharacterized protein
EOG091204JE	YQE_05781	Dendroctonus ponderosae	Putative uncharacterized protein
EOG091204JJ	OTAU005588	Onthophagus taurus	Putative uncharacterized protein
EOG091204JJ	AGLA000907	Anoplophora glabripennis	Putative uncharacterized protein
EOG091204JJ	APLA014309	Agrilus planipennis	Putative uncharacterized protein
EOG091204JJ	TC009949	Tribolium castaneum	Putative uncharacterized protein
EOG091204JJ	LDEC015193	Leptinotarsa decemlineata	Putative uncharacterized protein
EOG091204JJ	YQE_08710	Dendroctonus ponderosae	Putative uncharacterized protein
EOG091204JM	OTAU002778	Onthophagus taurus	Putative uncharacterized protein
EOG091204JM	AGLA019770	Anoplophora glabripennis	Putative uncharacterized protein
EOG091204JM	APLA006772	Agrilus planipennis	Putative uncharacterized protein
EOG091204JM	TC014185	Tribolium castaneum	Putative uncharacterized protein
EOG091204JM	LDEC021031	Leptinotarsa decemlineata	Putative uncharacterized protein
EOG091204JM	YQE_02832	Dendroctonus ponderosae	Putative uncharacterized protein
EOG091204JR	OTAU002116	Onthophagus taurus	Putative uncharacterized protein
EOG091204JR	AGLA001761	Anoplophora glabripennis	Putative uncharacterized protein
EOG091204JR	APLA003288	Agrilus planipennis	Putative uncharacterized protein
EOG091204JR	TC012624	Tribolium castaneum	Putative uncharacterized protein
EOG091204JR	LDEC006196	Leptinotarsa decemlineata	Putative uncharacterized protein
EOG091204JR	YQE_12956	Dendroctonus ponderosae	Putative uncharacterized protein
EOG091204JS	OTAU003460	Onthophagus taurus	Putative uncharacterized protein
EOG091204JS	AGLA016362	Anoplophora glabripennis	Putative uncharacterized protein
EOG091204JS	APLA011251	Agrilus planipennis	Putative uncharacterized protein
EOG091204JS	TC009023	Tribolium castaneum	Putative uncharacterized protein
EOG091204JS	LDEC009872	Leptinotarsa decemlineata	Putative uncharacterized protein
EOG091204JS	YQE_11809	Dendroctonus ponderosae	Putative uncharacterized protein
EOG091204JW	OTAU005868	Onthophagus taurus	DNA binding
EOG091204JW	AGLA018551	Anoplophora glabripennis	DNA binding
EOG091204JW	APLA003590	Agrilus planipennis	DNA binding
EOG091204JW	TC033194	Tribolium castaneum	DNA binding
EOG091204JW	LDEC014812	Leptinotarsa decemlineata	DNA binding
EOG091204JW	YQE_11392	Dendroctonus ponderosae	DNA binding
EOG091204JY	OTAU005940	Onthophagus taurus	Putative uncharacterized protein
EOG091204JY	AGLA000439	Anoplophora glabripennis	Putative uncharacterized protein
EOG091204JY	APLA009206	Agrilus planipennis	Putative uncharacterized protein
EOG091204JY	TC008456	Tribolium castaneum	Putative uncharacterized protein
EOG091204JY	LDEC008665	Leptinotarsa decemlineata	Putative uncharacterized protein
EOG091204JY	YQE_04391	Dendroctonus ponderosae	Putative uncharacterized protein
EOG091204JZ	OTAU014200	Onthophagus taurus	Putative uncharacterized protein
EOG091204JZ	AGLA018529	Anoplophora glabripennis	Putative uncharacterized protein
EOG091204JZ	APLA000371	Agrilus planipennis	Putative uncharacterized protein
EOG091204JZ	TC006239	Tribolium castaneum	Putative uncharacterized protein
EOG091204JZ	LDEC002446	Leptinotarsa decemlineata	Putative uncharacterized protein
EOG091204JZ	YQE_05691	Dendroctonus ponderosae	Putative uncharacterized protein
EOG091204K1	OTAU004044	Onthophagus taurus	Putative uncharacterized protein
EOG091204K1	AGLA017875	Anoplophora glabripennis	Putative uncharacterized protein
EOG091204K1	APLA006622	Agrilus planipennis	Putative uncharacterized protein
EOG091204K1	TC010377	Tribolium castaneum	Putative uncharacterized protein
EOG091204K1	LDEC020285	Leptinotarsa decemlineata	Putative uncharacterized protein
EOG091204K1	YQE_02751	Dendroctonus ponderosae	Putative uncharacterized protein
EOG091204K2	OTAU015518	Onthophagus taurus	Putative uncharacterized protein
EOG091204K2	AGLA010804	Anoplophora glabripennis	Putative uncharacterized protein
EOG091204K2	APLA013253	Agrilus planipennis	Putative uncharacterized protein
EOG091204K2	TC013703	Tribolium castaneum	Putative uncharacterized protein
EOG091204K2	LDEC016363	Leptinotarsa decemlineata	Putative uncharacterized protein
EOG091204K2	YQE_05080	Dendroctonus ponderosae	Putative uncharacterized protein
EOG091204K4	OTAU006874	Onthophagus taurus	Putative uncharacterized protein
EOG091204K4	AGLA008852	Anoplophora glabripennis	Putative uncharacterized protein
EOG091204K4	APLA007730	Agrilus planipennis	Putative uncharacterized protein
EOG091204K4	TC014467	Tribolium castaneum	Putative uncharacterized protein
EOG091204K4	LDEC018355	Leptinotarsa decemlineata	Putative uncharacterized protein
EOG091204K4	YQE_07555	Dendroctonus ponderosae	Putative uncharacterized protein
EOG091204K6	OTAU000162	Onthophagus taurus	Putative uncharacterized protein
EOG091204K6	AGLA014800	Anoplophora glabripennis	Putative uncharacterized protein
EOG091204K6	APLA007203	Agrilus planipennis	Putative uncharacterized protein
EOG091204K6	TC030751	Tribolium castaneum	Putative uncharacterized protein
EOG091204K6	LDEC006707	Leptinotarsa decemlineata	Putative uncharacterized protein

EOG091204K6	YQE_08986	Dendroctonus ponderosae	Putative uncharacterized protein
EOG091204KB	OTAU010506	Onthophagus taurus	NADH dehydrogenase (Ubiquinone) flavoprotein 2
EOG091204KB	AGLA003566	Anoplophora glabripennis	NADH dehydrogenase (Ubiquinone) flavoprotein 2
EOG091204KB	APLA008155	Agrilus planipennis	NADH dehydrogenase (Ubiquinone) flavoprotein 2
EOG091204KB	TC003690	Tribolium castaneum	NADH dehydrogenase (Ubiquinone) flavoprotein 2
EOG091204KB	LDEC016959	Leptinotarsa decemlineata	NADH dehydrogenase (Ubiquinone) flavoprotein 2
EOG091204KB	YQE_07392	Dendroctonus ponderosae	NADH dehydrogenase (Ubiquinone) flavoprotein 2
EOG091204KC	OTAU011551	Onthophagus taurus	Putative uncharacterized protein
EOG091204KC	AGLA004168	Anoplophora glabripennis	Putative uncharacterized protein
EOG091204KC	APLA002906	Agrilus planipennis	Putative uncharacterized protein
EOG091204KC	TC000930	Tribolium castaneum	Putative uncharacterized protein
EOG091204KC	LDEC000772	Leptinotarsa decemlineata	Putative uncharacterized protein
EOG091204KC	YQE_09511	Dendroctonus ponderosae	Putative uncharacterized protein
EOG091204KD	OTAU010310	Onthophagus taurus	Putative uncharacterized protein
EOG091204KD	AGLA006489	Anoplophora glabripennis	Putative uncharacterized protein
EOG091204KD	APLA011226	Agrilus planipennis	Putative uncharacterized protein
EOG091204KD	TC009716	Tribolium castaneum	Putative uncharacterized protein
EOG091204KD	LDEC002194	Leptinotarsa decemlineata	Putative uncharacterized protein
EOG091204KD	YQE_11789	Dendroctonus ponderosae	Putative uncharacterized protein
EOG091204KE	OTAU002716	Onthophagus taurus	Protein Wnt
EOG091204KE	AGLA011224	Anoplophora glabripennis	Protein Wnt
EOG091204KE	APLA009728	Agrilus planipennis	Protein Wnt
EOG091204KE	TC014084	Tribolium castaneum	Protein Wnt
EOG091204KE	LDEC012980	Leptinotarsa decemlineata	Protein Wnt
EOG091204KE	YQE_13084	Dendroctonus ponderosae	Protein Wnt
EOG091204KI	OTAU010542	Onthophagus taurus	Putative uncharacterized protein
EOG091204KI	AGLA021409	Anoplophora glabripennis	Putative uncharacterized protein
EOG091204KI	APLA004859	Agrilus planipennis	Putative uncharacterized protein
EOG091204KI	TC013393	Tribolium castaneum	Putative uncharacterized protein
EOG091204KI	LDEC002597	Leptinotarsa decemlineata	Putative uncharacterized protein
EOG091204KI	YQE_06101	Dendroctonus ponderosae	Putative uncharacterized protein
EOG091204KK	OTAU004052	Onthophagus taurus	Putative uncharacterized protein
EOG091204KK	AGLA006656	Anoplophora glabripennis	Putative uncharacterized protein
EOG091204KK	APLA006905	Agrilus planipennis	Putative uncharacterized protein
EOG091204KK	TC002504	Tribolium castaneum	Putative uncharacterized protein
EOG091204KK	LDEC004662	Leptinotarsa decemlineata	Putative uncharacterized protein
EOG091204KK	YQE_12368	Dendroctonus ponderosae	Putative uncharacterized protein
EOG091204KN	OTAU015470	Onthophagus taurus	dolichol kinase activity
EOG091204KN	AGLA008104	Anoplophora glabripennis	dolichol kinase activity
EOG091204KN	APLA007712	Agrilus planipennis	dolichol kinase activity
EOG091204KN	TC032121	Tribolium castaneum	dolichol kinase activity
EOG091204KN	LDEC007069	Leptinotarsa decemlineata	dolichol kinase activity
EOG091204KN	YQE_11617	Dendroctonus ponderosae	dolichol kinase activity
EOG091204KO	OTAU015253	Onthophagus taurus	zinc ion binding
EOG091204KO	AGLA012795	Anoplophora glabripennis	zinc ion binding
EOG091204KO	APLA003079	Agrilus planipennis	zinc ion binding
EOG091204KO	TC031695	Tribolium castaneum	zinc ion binding
EOG091204KO	LDEC016463	Leptinotarsa decemlineata	zinc ion binding
EOG091204KO	YQE_04049	Dendroctonus ponderosae	zinc ion binding
EOG091204KP	OTAU010497	Onthophagus taurus	None
EOG091204KP	AGLA010777	Anoplophora glabripennis	None
EOG091204KP	APLA012001	Agrilus planipennis	None
EOG091204KP	TC032592	Tribolium castaneum	None
EOG091204KP	LDEC016709	Leptinotarsa decemlineata	None
EOG091204KP	YQE_10236	Dendroctonus ponderosae	None
EOG091204KQ	OTAU001146	Onthophagus taurus	Nimrod B
EOG091204KQ	AGLA015417	Anoplophora glabripennis	Nimrod B
EOG091204KQ	APLA012025	Agrilus planipennis	Nimrod B
EOG091204KQ	TC011428	Tribolium castaneum	Nimrod B
EOG091204KQ	LDEC010916	Leptinotarsa decemlineata	Nimrod B
EOG091204KQ	YQE_04232	Dendroctonus ponderosae	Nimrod B
EOG091204KR	OTAU012431	Onthophagus taurus	Putative uncharacterized protein
EOG091204KR	AGLA005495	Anoplophora glabripennis	Putative uncharacterized protein
EOG091204KR	APLA011582	Agrilus planipennis	Putative uncharacterized protein
EOG091204KR	TC014292	Tribolium castaneum	Putative uncharacterized protein
EOG091204KR	LDEC003056	Leptinotarsa decemlineata	Putative uncharacterized protein
EOG091204KR	YQE_06115	Dendroctonus ponderosae	Putative uncharacterized protein

EOG091204KS	OTAU009445	Onthophagus taurus	Putative uncharacterized protein
EOG091204KS	AGLA010022	Anoplophora glabripennis	Putative uncharacterized protein
EOG091204KS	APLA002165	Agrilus planipennis	Putative uncharacterized protein
EOG091204KS	TC007935	Tribolium castaneum	Putative uncharacterized protein
EOG091204KS	LDEC000679	Leptinotarsa decemlineata	Putative uncharacterized protein
EOG091204KS	YQE_09034	Dendroctonus ponderosae	Putative uncharacterized protein
EOG091204KV	OTAU006614	Onthophagus taurus	Putative uncharacterized protein
EOG091204KV	AGLA017921	Anoplophora glabripennis	Putative uncharacterized protein
EOG091204KV	APLA009815	Agrilus planipennis	Putative uncharacterized protein
EOG091204KV	TC009965	Tribolium castaneum	Putative uncharacterized protein
EOG091204KV	LDEC015374	Leptinotarsa decemlineata	Putative uncharacterized protein
EOG091204KV	YQE_05318	Dendroctonus ponderosae	Putative uncharacterized protein
EOG091204KX	OTAU003475	Onthophagus taurus	Putative uncharacterized protein
EOG091204KX	AGLA013130	Anoplophora glabripennis	Putative uncharacterized protein
EOG091204KX	APLA001596	Agrilus planipennis	Putative uncharacterized protein
EOG091204KX	TC010030	Tribolium castaneum	Putative uncharacterized protein
EOG091204KX	LDEC010673	Leptinotarsa decemlineata	Putative uncharacterized protein
EOG091204KX	YQE_02332	Dendroctonus ponderosae	Putative uncharacterized protein
EOG091204L1	OTAU011215	Onthophagus taurus	Putative uncharacterized protein
EOG091204L1	AGLA004455	Anoplophora glabripennis	Putative uncharacterized protein
EOG091204L1	APLA007887	Agrilus planipennis	Putative uncharacterized protein
EOG091204L1	TC010359	Tribolium castaneum	Putative uncharacterized protein
EOG091204L1	LDEC002698	Leptinotarsa decemlineata	Putative uncharacterized protein
EOG091204L1	YQE_09957	Dendroctonus ponderosae	Putative uncharacterized protein
EOG091204L2	OTAU012891	Onthophagus taurus	Putative uncharacterized protein
EOG091204L2	AGLA017660	Anoplophora glabripennis	Putative uncharacterized protein
EOG091204L2	APLA007633	Agrilus planipennis	Putative uncharacterized protein
EOG091204L2	TC000028	Tribolium castaneum	Putative uncharacterized protein
EOG091204L2	LDEC021628	Leptinotarsa decemlineata	Putative uncharacterized protein
EOG091204L2	YQE_08271	Dendroctonus ponderosae	Putative uncharacterized protein
EOG091204L3	OTAU015262	Onthophagus taurus	Putative uncharacterized protein
EOG091204L3	AGLA002434	Anoplophora glabripennis	Putative uncharacterized protein
EOG091204L3	APLA001303	Agrilus planipennis	Putative uncharacterized protein
EOG091204L3	TC011026	Tribolium castaneum	Putative uncharacterized protein
EOG091204L3	LDEC009628	Leptinotarsa decemlineata	Putative uncharacterized protein
EOG091204L3	YQE_07698	Dendroctonus ponderosae	Putative uncharacterized protein
EOG091204L4	OTAU001898	Onthophagus taurus	Putative uncharacterized protein
EOG091204L4	AGLA009732	Anoplophora glabripennis	Putative uncharacterized protein
EOG091204L4	APLA004462	Agrilus planipennis	Putative uncharacterized protein
EOG091204L4	TC005943	Tribolium castaneum	Putative uncharacterized protein
EOG091204L4	LDEC020226	Leptinotarsa decemlineata	Putative uncharacterized protein
EOG091204L4	YQE_12100	Dendroctonus ponderosae	Putative uncharacterized protein
EOG091204L5	OTAU006030	Onthophagus taurus	Putative uncharacterized protein
EOG091204L5	AGLA009526	Anoplophora glabripennis	Putative uncharacterized protein
EOG091204L5	APLA004215	Agrilus planipennis	Putative uncharacterized protein
EOG091204L5	TC007970	Tribolium castaneum	Putative uncharacterized protein
EOG091204L5	LDEC014777	Leptinotarsa decemlineata	Putative uncharacterized protein
EOG091204L5	YQE_04948	Dendroctonus ponderosae	Putative uncharacterized protein
EOG091204L8	OTAU004491	Onthophagus taurus	Putative uncharacterized protein
EOG091204L8	AGLA008929	Anoplophora glabripennis	Putative uncharacterized protein
EOG091204L8	APLA006924	Agrilus planipennis	Putative uncharacterized protein
EOG091204L8	TC003247	Tribolium castaneum	Putative uncharacterized protein
EOG091204L8	LDEC012358	Leptinotarsa decemlineata	Putative uncharacterized protein
EOG091204L8	YQE_03419	Dendroctonus ponderosae	Putative uncharacterized protein
EOG091204L9	OTAU000240	Onthophagus taurus	Putative uncharacterized protein
EOG091204L9	AGLA011944	Anoplophora glabripennis	Putative uncharacterized protein
EOG091204L9	APLA002877	Agrilus planipennis	Putative uncharacterized protein
EOG091204L9	TC013835	Tribolium castaneum	Putative uncharacterized protein
EOG091204L9	LDEC003253	Leptinotarsa decemlineata	Putative uncharacterized protein
EOG091204L9	YQE_06139	Dendroctonus ponderosae	Putative uncharacterized protein
EOG091204LC	OTAU002953	Onthophagus taurus	Putative uncharacterized protein
EOG091204LC	AGLA011862	Anoplophora glabripennis	Putative uncharacterized protein
EOG091204LC	APLA003431	Agrilus planipennis	Putative uncharacterized protein
EOG091204LC	TC009011	Tribolium castaneum	Putative uncharacterized protein
EOG091204LC	LDEC002367	Leptinotarsa decemlineata	Putative uncharacterized protein
EOG091204LC	YQE_12613	Dendroctonus ponderosae	Putative uncharacterized protein
EOG091204LF	OTAU000521	Onthophagus taurus	Putative uncharacterized protein

EOG091204LF	AGLA019446	Anoplophora glabripennis	Putative uncharacterized protein
EOG091204LF	APLA012468	Agrilus planipennis	Putative uncharacterized protein
EOG091204LF	TC011836	Tribolium castaneum	Putative uncharacterized protein
EOG091204LF	LDEC000482	Leptinotarsa decemlineata	Putative uncharacterized protein
EOG091204LF	YQE_02889	Dendroctonus ponderosae	Putative uncharacterized protein
EOG091204LK	OTAU003956	Onthophagus taurus	Putative uncharacterized protein
EOG091204LK	AGLA006036	Anoplophora glabripennis	Putative uncharacterized protein
EOG091204LK	APLA002628	Agrilus planipennis	Putative uncharacterized protein
EOG091204LK	TC004311	Tribolium castaneum	Putative uncharacterized protein
EOG091204LK	LDEC005148	Leptinotarsa decemlineata	Putative uncharacterized protein
EOG091204LK	YQE_12550	Dendroctonus ponderosae	Putative uncharacterized protein
EOG091204LL	OTAU002827	Onthophagus taurus	Putative uncharacterized protein
EOG091204LL	AGLA000334	Anoplophora glabripennis	Putative uncharacterized protein
EOG091204LL	APLA009992	Agrilus planipennis	Putative uncharacterized protein
EOG091204LL	TC004767	Tribolium castaneum	Putative uncharacterized protein
EOG091204LL	LDEC004706	Leptinotarsa decemlineata	Putative uncharacterized protein
EOG091204LL	YQE_02492	Dendroctonus ponderosae	Putative uncharacterized protein
EOG091204LM	OTAU001778	Onthophagus taurus	Putative uncharacterized protein
EOG091204LM	AGLA007288	Anoplophora glabripennis	Putative uncharacterized protein
EOG091204LM	APLA009743	Agrilus planipennis	Putative uncharacterized protein
EOG091204LM	TC005557	Tribolium castaneum	Putative uncharacterized protein
EOG091204LM	LDEC015004	Leptinotarsa decemlineata	Putative uncharacterized protein
EOG091204LM	YQE_09460	Dendroctonus ponderosae	Putative uncharacterized protein
EOG091204LN	OTAU000183	Onthophagus taurus	Putative uncharacterized protein
EOG091204LN	AGLA006365	Anoplophora glabripennis	Putative uncharacterized protein
EOG091204LN	APLA003029	Agrilus planipennis	Putative uncharacterized protein
EOG091204LN	TC014772	Tribolium castaneum	Putative uncharacterized protein
EOG091204LN	LDEC002614	Leptinotarsa decemlineata	Putative uncharacterized protein
EOG091204LN	YQE_02837	Dendroctonus ponderosae	Putative uncharacterized protein
EOG091204LO	OTAU003978	Onthophagus taurus	Putative uncharacterized protein
EOG091204LO	AGLA008958	Anoplophora glabripennis	Putative uncharacterized protein
EOG091204LO	APLA010066	Agrilus planipennis	Putative uncharacterized protein
EOG091204LO	TC016280	Tribolium castaneum	Putative uncharacterized protein
EOG091204LO	LDEC014104	Leptinotarsa decemlineata	Putative uncharacterized protein
EOG091204LO	YQE_12924	Dendroctonus ponderosae	Putative uncharacterized protein
EOG091204LQ	OTAU006055	Onthophagus taurus	None
EOG091204LQ	AGLA003779	Anoplophora glabripennis	None
EOG091204LQ	APLA015265	Agrilus planipennis	None
EOG091204LQ	TC032817	Tribolium castaneum	None
EOG091204LQ	LDEC018756	Leptinotarsa decemlineata	None
EOG091204LQ	YQE_04728	Dendroctonus ponderosae	None
EOG091204LT	OTAU012310	Onthophagus taurus	Putative uncharacterized protein
EOG091204LT	AGLA006689	Anoplophora glabripennis	Putative uncharacterized protein
EOG091204LT	APLA005502	Agrilus planipennis	Putative uncharacterized protein
EOG091204LT	TC000933	Tribolium castaneum	Putative uncharacterized protein
EOG091204LT	LDEC019589	Leptinotarsa decemlineata	Putative uncharacterized protein
EOG091204LT	YQE_01622	Dendroctonus ponderosae	Putative uncharacterized protein
EOG091204LU	OTAU010244	Onthophagus taurus	Putative uncharacterized protein
EOG091204LU	AGLA001499	Anoplophora glabripennis	Putative uncharacterized protein
EOG091204LU	APLA008727	Agrilus planipennis	Putative uncharacterized protein
EOG091204LU	TC000100	Tribolium castaneum	Putative uncharacterized protein
EOG091204LU	LDEC002461	Leptinotarsa decemlineata	Putative uncharacterized protein
EOG091204LU	YQE_05851	Dendroctonus ponderosae	Putative uncharacterized protein
EOG091204LW	OTAU001412	Onthophagus taurus	Putative uncharacterized protein
EOG091204LW	AGLA008329	Anoplophora glabripennis	Putative uncharacterized protein
EOG091204LW	APLA009941	Agrilus planipennis	Putative uncharacterized protein
EOG091204LW	TC004670	Tribolium castaneum	Putative uncharacterized protein
EOG091204LW	LDEC011944	Leptinotarsa decemlineata	Putative uncharacterized protein
EOG091204LW	YQE_09278	Dendroctonus ponderosae	Putative uncharacterized protein
EOG091204LX	OTAU010428	Onthophagus taurus	Putative uncharacterized protein
EOG091204LX	AGLA020438	Anoplophora glabripennis	Putative uncharacterized protein
EOG091204LX	APLA005517	Agrilus planipennis	Putative uncharacterized protein
EOG091204LX	TC001162	Tribolium castaneum	Putative uncharacterized protein
EOG091204LX	LDEC021558	Leptinotarsa decemlineata	Putative uncharacterized protein
EOG091204LX	YQE_03926	Dendroctonus ponderosae	Putative uncharacterized protein
EOG091204LY	OTAU006361	Onthophagus taurus	Putative uncharacterized protein
EOG091204LY	AGLA004912	Anoplophora glabripennis	Putative uncharacterized protein

EOG091204LY	APLA015466	Agrilus planipennis	Putative uncharacterized protein
EOG091204LY	TC014518	Tribolium castaneum	Putative uncharacterized protein
EOG091204LY	LDEC010387	Leptinotarsa decemlineata	Putative uncharacterized protein
EOG091204LY	YQE_12623	Dendroctonus ponderosae	Putative uncharacterized protein
EOG091204LZ	OTAU005820	Onthophagus taurus	Putative uncharacterized protein
EOG091204LZ	AGLA001515	Anoplophora glabripennis	Putative uncharacterized protein
EOG091204LZ	APLA003792	Agrilus planipennis	Putative uncharacterized protein
EOG091204LZ	TC003810	Tribolium castaneum	Putative uncharacterized protein
EOG091204LZ	LDEC004654	Leptinotarsa decemlineata	Putative uncharacterized protein
EOG091204LZ	YQE_06756	Dendroctonus ponderosae	Putative uncharacterized protein
EOG091204M3	OTAU008470	Onthophagus taurus	Putative uncharacterized protein
EOG091204M3	AGLA000172	Anoplophora glabripennis	Putative uncharacterized protein
EOG091204M3	APLA001942	Agrilus planipennis	Putative uncharacterized protein
EOG091204M3	TC011372	Tribolium castaneum	Putative uncharacterized protein
EOG091204M3	LDEC012673	Leptinotarsa decemlineata	Putative uncharacterized protein
EOG091204M3	YQE_01596	Dendroctonus ponderosae	Putative uncharacterized protein
EOG091204M7	OTAU009071	Onthophagus taurus	Putative uncharacterized protein
EOG091204M7	AGLA000442	Anoplophora glabripennis	Putative uncharacterized protein
EOG091204M7	APLA006597	Agrilus planipennis	Putative uncharacterized protein
EOG091204M7	TC007153	Tribolium castaneum	Putative uncharacterized protein
EOG091204M7	LDEC008659	Leptinotarsa decemlineata	Putative uncharacterized protein
EOG091204M7	YQE_02068	Dendroctonus ponderosae	Putative uncharacterized protein
EOG091204M8	OTAU008677	Onthophagus taurus	Putative uncharacterized protein
EOG091204M8	AGLA016353	Anoplophora glabripennis	Putative uncharacterized protein
EOG091204M8	APLA012307	Agrilus planipennis	Putative uncharacterized protein
EOG091204M8	TC005615	Tribolium castaneum	Putative uncharacterized protein
EOG091204M8	LDEC006317	Leptinotarsa decemlineata	Putative uncharacterized protein
EOG091204M8	YQE_06582	Dendroctonus ponderosae	Putative uncharacterized protein
EOG091204MC	OTAU012507	Onthophagus taurus	Mutagen-sensitive 312
EOG091204MC	AGLA015618	Anoplophora glabripennis	Mutagen-sensitive 312
EOG091204MC	APLA005996	Agrilus planipennis	Mutagen-sensitive 312
EOG091204MC	TC008786	Tribolium castaneum	Mutagen-sensitive 312
EOG091204MC	LDEC010373	Leptinotarsa decemlineata	Mutagen-sensitive 312
EOG091204MC	YQE_03385	Dendroctonus ponderosae	Mutagen-sensitive 312
EOG091204MF	OTAU006739	Onthophagus taurus	Putative uncharacterized protein
EOG091204MF	AGLA008774	Anoplophora glabripennis	Putative uncharacterized protein
EOG091204MF	APLA005321	Agrilus planipennis	Putative uncharacterized protein
EOG091204MF	TC001477	Tribolium castaneum	Putative uncharacterized protein
EOG091204MF	LDEC014242	Leptinotarsa decemlineata	Putative uncharacterized protein
EOG091204MF	YQE_07077	Dendroctonus ponderosae	Putative uncharacterized protein
EOG091204MG	OTAU015489	Onthophagus taurus	Putative uncharacterized protein
EOG091204MG	AGLA008676	Anoplophora glabripennis	Putative uncharacterized protein
EOG091204MG	APLA003898	Agrilus planipennis	Putative uncharacterized protein
EOG091204MG	TC010637	Tribolium castaneum	Putative uncharacterized protein
EOG091204MG	LDEC019276	Leptinotarsa decemlineata	Putative uncharacterized protein
EOG091204MG	YQE_07566	Dendroctonus ponderosae	Putative uncharacterized protein
EOG091204MH	OTAU006649	Onthophagus taurus	Putative uncharacterized protein
EOG091204MH	AGLA013770	Anoplophora glabripennis	Putative uncharacterized protein
EOG091204MH	APLA015204	Agrilus planipennis	Putative uncharacterized protein
EOG091204MH	TC010563	Tribolium castaneum	Putative uncharacterized protein
EOG091204MH	LDEC005180	Leptinotarsa decemlineata	Putative uncharacterized protein
EOG091204MH	YQE_06809	Dendroctonus ponderosae	Putative uncharacterized protein
EOG091204MK	OTAU008249	Onthophagus taurus	Putative uncharacterized protein
EOG091204MK	AGLA020661	Anoplophora glabripennis	Putative uncharacterized protein
EOG091204MK	APLA006641	Agrilus planipennis	Putative uncharacterized protein
EOG091204MK	TC010379	Tribolium castaneum	Putative uncharacterized protein
EOG091204MK	LDEC004052	Leptinotarsa decemlineata	Putative uncharacterized protein
EOG091204MK	YQE_11681	Dendroctonus ponderosae	Putative uncharacterized protein
EOG091204ML	OTAU000121	Onthophagus taurus	structural constituent of cuticle
EOG091204ML	AGLA016445	Anoplophora glabripennis	structural constituent of cuticle
EOG091204ML	APLA004799	Agrilus planipennis	structural constituent of cuticle
EOG091204ML	TC031150	Tribolium castaneum	structural constituent of cuticle
EOG091204ML	LDEC020132	Leptinotarsa decemlineata	structural constituent of cuticle
EOG091204ML	YQE_04821	Dendroctonus ponderosae	structural constituent of cuticle
EOG091204MP	OTAU004137	Onthophagus taurus	Putative uncharacterized protein
EOG091204MP	AGLA005850	Anoplophora glabripennis	Putative uncharacterized protein
EOG091204MP	APLA008872	Agrilus planipennis	Putative uncharacterized protein

EOG091204MP	TC010199	Tribolium castaneum	Putative uncharacterized protein
EOG091204MP	LDEC011903	Leptinotarsa decemlineata	Putative uncharacterized protein
EOG091204MP	YQE_03514	Dendroctonus ponderosae	Putative uncharacterized protein
EOG091204MS	OTAU003251	Onthophagus taurus	None
EOG091204MS	AGLA006831	Anoplophora glabripennis	None
EOG091204MS	APLA010530	Agrilus planipennis	None
EOG091204MS	TC033845	Tribolium castaneum	None
EOG091204MS	LDEC015515	Leptinotarsa decemlineata	None
EOG091204MS	YQE_12632	Dendroctonus ponderosae	None
EOG091204MU	OTAU014497	Onthophagus taurus	Putative uncharacterized protein
EOG091204MU	AGLA003390	Anoplophora glabripennis	Putative uncharacterized protein
EOG091204MU	APLA000298	Agrilus planipennis	Putative uncharacterized protein
EOG091204MU	TC002618	Tribolium castaneum	Putative uncharacterized protein
EOG091204MU	LDEC004618	Leptinotarsa decemlineata	Putative uncharacterized protein
EOG091204MU	YQE_12279	Dendroctonus ponderosae	Putative uncharacterized protein
EOG091204MW	OTAU005994	Onthophagus taurus	Putative uncharacterized protein
EOG091204MW	AGLA018212	Anoplophora glabripennis	Putative uncharacterized protein
EOG091204MW	APLA002071	Agrilus planipennis	Putative uncharacterized protein
EOG091204MW	TC011543	Tribolium castaneum	Putative uncharacterized protein
EOG091204MW	LDEC010284	Leptinotarsa decemlineata	Putative uncharacterized protein
EOG091204MW	YQE_09778	Dendroctonus ponderosae	Putative uncharacterized protein
EOG091204N1	OTAU010309	Onthophagus taurus	Putative uncharacterized protein
EOG091204N1	AGLA006491	Anoplophora glabripennis	Putative uncharacterized protein
EOG091204N1	APLA011227	Agrilus planipennis	Putative uncharacterized protein
EOG091204N1	TC009113	Tribolium castaneum	Putative uncharacterized protein
EOG091204N1	LDEC002193	Leptinotarsa decemlineata	Putative uncharacterized protein
EOG091204N1	YQE_11790	Dendroctonus ponderosae	Putative uncharacterized protein
EOG091204N6	OTAU014131	Onthophagus taurus	Putative uncharacterized protein
EOG091204N6	AGLA020429	Anoplophora glabripennis	Putative uncharacterized protein
EOG091204N6	APLA000616	Agrilus planipennis	Putative uncharacterized protein
EOG091204N6	TC016023	Tribolium castaneum	Putative uncharacterized protein
EOG091204N6	LDEC022803	Leptinotarsa decemlineata	Putative uncharacterized protein
EOG091204N6	YQE_11494	Dendroctonus ponderosae	Putative uncharacterized protein
EOG091204N7	OTAU002757	Onthophagus taurus	Putative uncharacterized protein
EOG091204N7	AGLA000247	Anoplophora glabripennis	Putative uncharacterized protein
EOG091204N7	APLA006421	Agrilus planipennis	Putative uncharacterized protein
EOG091204N7	TC001740	Tribolium castaneum	Putative uncharacterized protein
EOG091204N7	LDEC014127	Leptinotarsa decemlineata	Putative uncharacterized protein
EOG091204N7	YQE_09197	Dendroctonus ponderosae	Putative uncharacterized protein
EOG091204NB	OTAU008170	Onthophagus taurus	Putative uncharacterized protein
EOG091204NB	AGLA021377	Anoplophora glabripennis	Putative uncharacterized protein
EOG091204NB	APLA000627	Agrilus planipennis	Putative uncharacterized protein
EOG091204NB	TC015415	Tribolium castaneum	Putative uncharacterized protein
EOG091204NB	LDEC003701	Leptinotarsa decemlineata	Putative uncharacterized protein
EOG091204NB	YQE_11456	Dendroctonus ponderosae	Putative uncharacterized protein
EOG091204ND	OTAU005612	Onthophagus taurus	ATP binding
EOG091204ND	AGLA006481	Anoplophora glabripennis	ATP binding
EOG091204ND	APLA010871	Agrilus planipennis	ATP binding
EOG091204ND	TC031176	Tribolium castaneum	ATP binding
EOG091204ND	LDEC004417	Leptinotarsa decemlineata	ATP binding
EOG091204ND	YQE_05278	Dendroctonus ponderosae	ATP binding
EOG091204NI	OTAU011287	Onthophagus taurus	NAD+ binding
EOG091204NI	AGLA008020	Anoplophora glabripennis	NAD+ binding
EOG091204NI	APLA013082	Agrilus planipennis	NAD+ binding
EOG091204NI	TC033432	Tribolium castaneum	NAD+ binding
EOG091204NI	LDEC014924	Leptinotarsa decemlineata	NAD+ binding
EOG091204NI	YQE_11882	Dendroctonus ponderosae	NAD+ binding
EOG091204NL	OTAU000010	Onthophagus taurus	Putative uncharacterized protein
EOG091204NL	AGLA005491	Anoplophora glabripennis	Putative uncharacterized protein
EOG091204NL	APLA012139	Agrilus planipennis	Putative uncharacterized protein
EOG091204NL	TC013519	Tribolium castaneum	Putative uncharacterized protein
EOG091204NL	LDEC003052	Leptinotarsa decemlineata	Putative uncharacterized protein
EOG091204NL	YQE_04265	Dendroctonus ponderosae	Putative uncharacterized protein
EOG091204NS	OTAU014145	Onthophagus taurus	Putative uncharacterized protein
EOG091204NS	AGLA001775	Anoplophora glabripennis	Putative uncharacterized protein
EOG091204NS	APLA011707	Agrilus planipennis	Putative uncharacterized protein
EOG091204NS	TC014287	Tribolium castaneum	Putative uncharacterized protein

EOG091204NS	LDEC008932	Leptinotarsa decemlineata	Putative uncharacterized protein
EOG091204NS	YQE_06055	Dendroctonus ponderosae	Putative uncharacterized protein
EOG091204NX	OTAU004949	Onthophagus taurus	Putative uncharacterized protein
EOG091204NX	AGLA014871	Anoplophora glabripennis	Putative uncharacterized protein
EOG091204NX	APLA005293	Agrilus planipennis	Putative uncharacterized protein
EOG091204NX	TC000560	Tribolium castaneum	Putative uncharacterized protein
EOG091204NX	LDEC017177	Leptinotarsa decemlineata	Putative uncharacterized protein
EOG091204NX	YQE_10984	Dendroctonus ponderosae	Putative uncharacterized protein
EOG091204NY	OTAU005471	Onthophagus taurus	Putative uncharacterized protein
EOG091204NY	AGLA010809	Anoplophora glabripennis	Putative uncharacterized protein
EOG091204NY	APLA004843	Agrilus planipennis	Putative uncharacterized protein
EOG091204NY	TC014045	Tribolium castaneum	Putative uncharacterized protein
EOG091204NY	LDEC016368	Leptinotarsa decemlineata	Putative uncharacterized protein
EOG091204NY	YQE_11775	Dendroctonus ponderosae	Putative uncharacterized protein
EOG091204O1	OTAU000609	Onthophagus taurus	Putative uncharacterized protein
EOG091204O1	AGLA018153	Anoplophora glabripennis	Putative uncharacterized protein
EOG091204O1	APLA002362	Agrilus planipennis	Putative uncharacterized protein
EOG091204O1	TC010676	Tribolium castaneum	Putative uncharacterized protein
EOG091204O1	LDEC021273	Leptinotarsa decemlineata	Putative uncharacterized protein
EOG091204O1	YQE_04327	Dendroctonus ponderosae	Putative uncharacterized protein
EOG091204O2	OTAU012541	Onthophagus taurus	Putative uncharacterized protein
EOG091204O2	AGLA006766	Anoplophora glabripennis	Putative uncharacterized protein
EOG091204O2	APLA015387	Agrilus planipennis	Putative uncharacterized protein
EOG091204O2	TC005008	Tribolium castaneum	Putative uncharacterized protein
EOG091204O2	LDEC008154	Leptinotarsa decemlineata	Putative uncharacterized protein
EOG091204O2	YQE_01743	Dendroctonus ponderosae	Putative uncharacterized protein
EOG091204O3	OTAU001828	Onthophagus taurus	Putative uncharacterized protein
EOG091204O3	AGLA005029	Anoplophora glabripennis	Putative uncharacterized protein
EOG091204O3	APLA004290	Agrilus planipennis	Putative uncharacterized protein
EOG091204O3	TC005864	Tribolium castaneum	Putative uncharacterized protein
EOG091204O3	LDEC006736	Leptinotarsa decemlineata	Putative uncharacterized protein
EOG091204O3	YQE_05554	Dendroctonus ponderosae	Putative uncharacterized protein
EOG091204O6	OTAU002972	Onthophagus taurus	Putative uncharacterized protein
EOG091204O6	AGLA021398	Anoplophora glabripennis	Putative uncharacterized protein
EOG091204O6	APLA008794	Agrilus planipennis	Putative uncharacterized protein
EOG091204O6	TC009623	Tribolium castaneum	Putative uncharacterized protein
EOG091204O6	LDEC004436	Leptinotarsa decemlineata	Putative uncharacterized protein
EOG091204O6	YQE_11283	Dendroctonus ponderosae	Putative uncharacterized protein
EOG091204O7	OTAU015637	Onthophagus taurus	Putative uncharacterized protein
EOG091204O7	AGLA009744	Anoplophora glabripennis	Putative uncharacterized protein
EOG091204O7	APLA009660	Agrilus planipennis	Putative uncharacterized protein
EOG091204O7	TC005309	Tribolium castaneum	Putative uncharacterized protein
EOG091204O7	LDEC011347	Leptinotarsa decemlineata	Putative uncharacterized protein
EOG091204O7	YQE_02845	Dendroctonus ponderosae	Putative uncharacterized protein
EOG091204O8	OTAU007730	Onthophagus taurus	Putative uncharacterized protein
EOG091204O8	AGLA021684	Anoplophora glabripennis	Putative uncharacterized protein
EOG091204O8	APLA004854	Agrilus planipennis	Putative uncharacterized protein
EOG091204O8	TC013923	Tribolium castaneum	Putative uncharacterized protein
EOG091204O8	LDEC007755	Leptinotarsa decemlineata	Putative uncharacterized protein
EOG091204O8	YQE_07292	Dendroctonus ponderosae	Putative uncharacterized protein
EOG091204O9	OTAU013673	Onthophagus taurus	Putative uncharacterized protein
EOG091204O9	AGLA003340	Anoplophora glabripennis	Putative uncharacterized protein
EOG091204O9	APLA005947	Agrilus planipennis	Putative uncharacterized protein
EOG091204O9	TC015141	Tribolium castaneum	Putative uncharacterized protein
EOG091204O9	LDEC007881	Leptinotarsa decemlineata	Putative uncharacterized protein
EOG091204O9	YQE_11752	Dendroctonus ponderosae	Putative uncharacterized protein
EOG091204OA	OTAU011115	Onthophagus taurus	Putative uncharacterized protein
EOG091204OA	AGLA002234	Anoplophora glabripennis	Putative uncharacterized protein
EOG091204OA	APLA010393	Agrilus planipennis	Putative uncharacterized protein
EOG091204OA	TC014372	Tribolium castaneum	Putative uncharacterized protein
EOG091204OA	LDEC014786	Leptinotarsa decemlineata	Putative uncharacterized protein
EOG091204OA	YQE_08111	Dendroctonus ponderosae	Putative uncharacterized protein
EOG091204OD	OTAU001606	Onthophagus taurus	Putative uncharacterized protein
EOG091204OD	AGLA005298	Anoplophora glabripennis	Putative uncharacterized protein
EOG091204OD	APLA005832	Agrilus planipennis	Putative uncharacterized protein
EOG091204OD	TC006500	Tribolium castaneum	Putative uncharacterized protein
EOG091204OD	LDEC018803	Leptinotarsa decemlineata	Putative uncharacterized protein

EOG091204OD	YQE_07034	Dendroctonus ponderosae	Putative uncharacterized protein
EOG091204OF	OTAU004762	Onthophagus taurus	Putative uncharacterized protein
EOG091204OF	AGLA019920	Anoplophora glabripennis	Putative uncharacterized protein
EOG091204OF	APLA003705	Agrilus planipennis	Putative uncharacterized protein
EOG091204OF	TC004960	Tribolium castaneum	Putative uncharacterized protein
EOG091204OF	LDEC014746	Leptinotarsa decemlineata	Putative uncharacterized protein
EOG091204OF	YQE_10757	Dendroctonus ponderosae	Putative uncharacterized protein
EOG091204OH	OTAU000534	Onthophagus taurus	Putative uncharacterized protein
EOG091204OH	AGLA006024	Anoplophora glabripennis	Putative uncharacterized protein
EOG091204OH	APLA002030	Agrilus planipennis	Putative uncharacterized protein
EOG091204OH	TC002919	Tribolium castaneum	Putative uncharacterized protein
EOG091204OH	LDEC017927	Leptinotarsa decemlineata	Putative uncharacterized protein
EOG091204OH	YQE_07788	Dendroctonus ponderosae	Putative uncharacterized protein
EOG091204OI	OTAU003395	Onthophagus taurus	Putative uncharacterized protein
EOG091204OI	AGLA018417	Anoplophora glabripennis	Putative uncharacterized protein
EOG091204OI	APLA006066	Agrilus planipennis	Putative uncharacterized protein
EOG091204OI	TC003403	Tribolium castaneum	Putative uncharacterized protein
EOG091204OI	LDEC005927	Leptinotarsa decemlineata	Putative uncharacterized protein
EOG091204OI	YQE_02753	Dendroctonus ponderosae	Putative uncharacterized protein
EOG091204OJ	OTAU009929	Onthophagus taurus	None
EOG091204OJ	AGLA001599	Anoplophora glabripennis	None
EOG091204OJ	APLA011558	Agrilus planipennis	None
EOG091204OJ	TC032543	Tribolium castaneum	None
EOG091204OJ	LDEC012604	Leptinotarsa decemlineata	None
EOG091204OJ	YQE_11192	Dendroctonus ponderosae	None
EOG091204OM	OTAU007144	Onthophagus taurus	None
EOG091204OM	AGLA014829	Anoplophora glabripennis	None
EOG091204OM	APLA002980	Agrilus planipennis	None
EOG091204OM	TC032079	Tribolium castaneum	None
EOG091204OM	LDEC006061	Leptinotarsa decemlineata	None
EOG091204OM	YQE_11780	Dendroctonus ponderosae	None
EOG091204OO	OTAU014410	Onthophagus taurus	Putative uncharacterized protein
EOG091204OO	AGLA000307	Anoplophora glabripennis	Putative uncharacterized protein
EOG091204OO	APLA008966	Agrilus planipennis	Putative uncharacterized protein
EOG091204OO	TC004724	Tribolium castaneum	Putative uncharacterized protein
EOG091204OO	LDEC022719	Leptinotarsa decemlineata	Putative uncharacterized protein
EOG091204OO	YQE_06687	Dendroctonus ponderosae	Putative uncharacterized protein
EOG091204OW	OTAU013577	Onthophagus taurus	Putative uncharacterized protein
EOG091204OW	AGLA003959	Anoplophora glabripennis	Putative uncharacterized protein
EOG091204OW	APLA005954	Agrilus planipennis	Putative uncharacterized protein
EOG091204OW	TC015403	Tribolium castaneum	Putative uncharacterized protein
EOG091204OW	LDEC012247	Leptinotarsa decemlineata	Putative uncharacterized protein
EOG091204OW	YQE_11470	Dendroctonus ponderosae	Putative uncharacterized protein
EOG091204OY	OTAU006380	Onthophagus taurus	Putative uncharacterized protein
EOG091204OY	AGLA010964	Anoplophora glabripennis	Putative uncharacterized protein
EOG091204OY	APLA012583	Agrilus planipennis	Putative uncharacterized protein
EOG091204OY	TC015890	Tribolium castaneum	Putative uncharacterized protein
EOG091204OY	LDEC013878	Leptinotarsa decemlineata	Putative uncharacterized protein
EOG091204OY	YQE_10289	Dendroctonus ponderosae	Putative uncharacterized protein
EOG091204P4	OTAU006555	Onthophagus taurus	Putative uncharacterized protein
EOG091204P4	AGLA000199	Anoplophora glabripennis	Putative uncharacterized protein
EOG091204P4	APLA014640	Agrilus planipennis	Putative uncharacterized protein
EOG091204P4	TC002731	Tribolium castaneum	Putative uncharacterized protein
EOG091204P4	LDEC005417	Leptinotarsa decemlineata	Putative uncharacterized protein
EOG091204P4	YQE_07403	Dendroctonus ponderosae	Putative uncharacterized protein
EOG091204P5	OTAU009202	Onthophagus taurus	Putative uncharacterized protein
EOG091204P5	AGLA009978	Anoplophora glabripennis	Putative uncharacterized protein
EOG091204P5	APLA005933	Agrilus planipennis	Putative uncharacterized protein
EOG091204P5	TC015026	Tribolium castaneum	Putative uncharacterized protein
EOG091204P5	LDEC010975	Leptinotarsa decemlineata	Putative uncharacterized protein
EOG091204P5	YQE_11248	Dendroctonus ponderosae	Putative uncharacterized protein
EOG091204P6	OTAU015374	Onthophagus taurus	DNA binding
EOG091204P6	AGLA006235	Anoplophora glabripennis	DNA binding
EOG091204P6	APLA002998	Agrilus planipennis	DNA binding
EOG091204P6	TC032174	Tribolium castaneum	DNA binding
EOG091204P6	LDEC021567	Leptinotarsa decemlineata	DNA binding
EOG091204P6	YQE_02425	Dendroctonus ponderosae	DNA binding

EOG091204P7	OTAU002673	Onthophagus taurus	Putative uncharacterized protein
EOG091204P7	AGLA006359	Anoplophora glabripennis	Putative uncharacterized protein
EOG091204P7	APLA012976	Agrilus planipennis	Putative uncharacterized protein
EOG091204P7	TC010444	Tribolium castaneum	Putative uncharacterized protein
EOG091204P7	LDEC002619	Leptinotarsa decemlineata	Putative uncharacterized protein
EOG091204P7	YQE_11391	Dendroctonus ponderosae	Putative uncharacterized protein
EOG091204P9	OTAU009093	Onthophagus taurus	DNA methyltransferase 2
EOG091204P9	AGLA011078	Anoplophora glabripennis	DNA methyltransferase 2
EOG091204P9	APLA001231	Agrilus planipennis	DNA methyltransferase 2
EOG091204P9	TC005511	Tribolium castaneum	DNA methyltransferase 2
EOG091204P9	LDEC009781	Leptinotarsa decemlineata	DNA methyltransferase 2
EOG091204P9	YQE_07503	Dendroctonus ponderosae	DNA methyltransferase 2
EOG091204PB	OTAU004068	Onthophagus taurus	Putative uncharacterized protein
EOG091204PB	AGLA007530	Anoplophora glabripennis	Putative uncharacterized protein
EOG091204PB	APLA002843	Agrilus planipennis	Putative uncharacterized protein
EOG091204PB	TC003408	Tribolium castaneum	Putative uncharacterized protein
EOG091204PB	LDEC003037	Leptinotarsa decemlineata	Putative uncharacterized protein
EOG091204PB	YQE_08261	Dendroctonus ponderosae	Putative uncharacterized protein
EOG091204PC	OTAU002110	Onthophagus taurus	Putative uncharacterized protein
EOG091204PC	AGLA014236	Anoplophora glabripennis	Putative uncharacterized protein
EOG091204PC	APLA011412	Agrilus planipennis	Putative uncharacterized protein
EOG091204PC	TC004326	Tribolium castaneum	Putative uncharacterized protein
EOG091204PC	LDEC023696	Leptinotarsa decemlineata	Putative uncharacterized protein
EOG091204PC	YQE_12883	Dendroctonus ponderosae	Putative uncharacterized protein
EOG091204PE	OTAU005452	Onthophagus taurus	Putative uncharacterized protein
EOG091204PE	AGLA018397	Anoplophora glabripennis	Putative uncharacterized protein
EOG091204PE	APLA002686	Agrilus planipennis	Putative uncharacterized protein
EOG091204PE	TC014733	Tribolium castaneum	Putative uncharacterized protein
EOG091204PE	LDEC006510	Leptinotarsa decemlineata	Putative uncharacterized protein
EOG091204PE	YQE_05074	Dendroctonus ponderosae	Putative uncharacterized protein
EOG091204PF	OTAU005138	Onthophagus taurus	Putative uncharacterized protein
EOG091204PF	AGLA005178	Anoplophora glabripennis	Putative uncharacterized protein
EOG091204PF	APLA000056	Agrilus planipennis	Putative uncharacterized protein
EOG091204PF	TC007903	Tribolium castaneum	Putative uncharacterized protein
EOG091204PF	LDEC001409	Leptinotarsa decemlineata	Putative uncharacterized protein
EOG091204PF	YQE_02100	Dendroctonus ponderosae	Putative uncharacterized protein
EOG091204PG	OTAU010827	Onthophagus taurus	Putative uncharacterized protein
EOG091204PG	AGLA007957	Anoplophora glabripennis	Putative uncharacterized protein
EOG091204PG	APLA012699	Agrilus planipennis	Putative uncharacterized protein
EOG091204PG	TC008271	Tribolium castaneum	Putative uncharacterized protein
EOG091204PG	LDEC004588	Leptinotarsa decemlineata	Putative uncharacterized protein
EOG091204PG	YQE_04186	Dendroctonus ponderosae	Putative uncharacterized protein
EOG091204PN	OTAU010462	Onthophagus taurus	catalytic activity
EOG091204PN	AGLA012170	Anoplophora glabripennis	catalytic activity
EOG091204PN	APLA004198	Agrilus planipennis	catalytic activity
EOG091204PN	TC032710	Tribolium castaneum	catalytic activity
EOG091204PN	LDEC017928	Leptinotarsa decemlineata	catalytic activity
EOG091204PN	YQE_08698	Dendroctonus ponderosae	catalytic activity
EOG091204PO	OTAU005024	Onthophagus taurus	Putative uncharacterized protein
EOG091204PO	AGLA014160	Anoplophora glabripennis	Putative uncharacterized protein
EOG091204PO	APLA007973	Agrilus planipennis	Putative uncharacterized protein
EOG091204PO	TC015698	Tribolium castaneum	Putative uncharacterized protein
EOG091204PO	LDEC010322	Leptinotarsa decemlineata	Putative uncharacterized protein
EOG091204PO	YQE_10750	Dendroctonus ponderosae	Putative uncharacterized protein
EOG091204PP	OTAU000953	Onthophagus taurus	Putative uncharacterized protein
EOG091204PP	AGLA000156	Anoplophora glabripennis	Putative uncharacterized protein
EOG091204PP	APLA008245	Agrilus planipennis	Putative uncharacterized protein
EOG091204PP	TC004823	Tribolium castaneum	Putative uncharacterized protein
EOG091204PP	LDEC018559	Leptinotarsa decemlineata	Putative uncharacterized protein
EOG091204PP	YQE_04130	Dendroctonus ponderosae	Putative uncharacterized protein
EOG091204PS	OTAU015628	Onthophagus taurus	Putative uncharacterized protein
EOG091204PS	AGLA014810	Anoplophora glabripennis	Putative uncharacterized protein
EOG091204PS	APLA014835	Agrilus planipennis	Putative uncharacterized protein
EOG091204PS	TC011867	Tribolium castaneum	Putative uncharacterized protein
EOG091204PS	LDEC004817	Leptinotarsa decemlineata	Putative uncharacterized protein
EOG091204PS	YQE_05044	Dendroctonus ponderosae	Putative uncharacterized protein
EOG091204PV	OTAU000624	Onthophagus taurus	Putative uncharacterized protein

EOG091204PV	AGLA009782	Anoplophora glabripennis	Putative uncharacterized protein
EOG091204PV	APLA003917	Agrilus planipennis	Putative uncharacterized protein
EOG091204PV	TC011682	Tribolium castaneum	Putative uncharacterized protein
EOG091204PV	LDEC003828	Leptinotarsa decemlineata	Putative uncharacterized protein
EOG091204PV	YQE_12912	Dendroctonus ponderosae	Putative uncharacterized protein
EOG091204PW	OTAU007843	Onthophagus taurus	Hydroxypyruvate isomerase
EOG091204PW	AGLA001172	Anoplophora glabripennis	Hydroxypyruvate isomerase
EOG091204PW	APLA001176	Agrilus planipennis	Hydroxypyruvate isomerase
EOG091204PW	TC030659	Tribolium castaneum	Hydroxypyruvate isomerase
EOG091204PW	LDEC007465	Leptinotarsa decemlineata	Hydroxypyruvate isomerase
EOG091204PW	YQE_02471	Dendroctonus ponderosae	Hydroxypyruvate isomerase
EOG091204PX	OTAU003533	Onthophagus taurus	Putative uncharacterized protein
EOG091204PX	AGLA020443	Anoplophora glabripennis	Putative uncharacterized protein
EOG091204PX	APLA010088	Agrilus planipennis	Putative uncharacterized protein
EOG091204PX	TC008697	Tribolium castaneum	Putative uncharacterized protein
EOG091204PX	LDEC000246	Leptinotarsa decemlineata	Putative uncharacterized protein
EOG091204PX	YQE_05400	Dendroctonus ponderosae	Putative uncharacterized protein
EOG091204PZ	OTAU003102	Onthophagus taurus	Putative uncharacterized protein
EOG091204PZ	AGLA009151	Anoplophora glabripennis	Putative uncharacterized protein
EOG091204PZ	APLA006650	Agrilus planipennis	Putative uncharacterized protein
EOG091204PZ	TC005676	Tribolium castaneum	Putative uncharacterized protein
EOG091204PZ	LDEC010788	Leptinotarsa decemlineata	Putative uncharacterized protein
EOG091204PZ	YQE_08531	Dendroctonus ponderosae	Putative uncharacterized protein
EOG091204Q0	OTAU006829	Onthophagus taurus	Putative uncharacterized protein
EOG091204Q0	AGLA012709	Anoplophora glabripennis	Putative uncharacterized protein
EOG091204Q0	APLA007322	Agrilus planipennis	Putative uncharacterized protein
EOG091204Q0	TC003033	Tribolium castaneum	Putative uncharacterized protein
EOG091204Q0	LDEC020110	Leptinotarsa decemlineata	Putative uncharacterized protein
EOG091204Q0	YQE_10273	Dendroctonus ponderosae	Putative uncharacterized protein
EOG091204Q2	OTAU006768	Onthophagus taurus	Putative uncharacterized protein
EOG091204Q2	AGLA007012	Anoplophora glabripennis	Putative uncharacterized protein
EOG091204Q2	APLA009707	Agrilus planipennis	Putative uncharacterized protein
EOG091204Q2	TC003894	Tribolium castaneum	Putative uncharacterized protein
EOG091204Q2	LDEC006505	Leptinotarsa decemlineata	Putative uncharacterized protein
EOG091204Q2	YQE_10227	Dendroctonus ponderosae	Putative uncharacterized protein
EOG091204Q5	OTAU005484	Onthophagus taurus	Putative uncharacterized protein
EOG091204Q5	AGLA009557	Anoplophora glabripennis	Putative uncharacterized protein
EOG091204Q5	APLA004815	Agrilus planipennis	Putative uncharacterized protein
EOG091204Q5	TC014463	Tribolium castaneum	Putative uncharacterized protein
EOG091204Q5	LDEC002652	Leptinotarsa decemlineata	Putative uncharacterized protein
EOG091204Q5	YQE_10461	Dendroctonus ponderosae	Putative uncharacterized protein
EOG091204Q8	OTAU007068	Onthophagus taurus	Putative uncharacterized protein
EOG091204Q8	AGLA009141	Anoplophora glabripennis	Putative uncharacterized protein
EOG091204Q8	APLA012016	Agrilus planipennis	Putative uncharacterized protein
EOG091204Q8	TC010475	Tribolium castaneum	Putative uncharacterized protein
EOG091204Q8	LDEC016327	Leptinotarsa decemlineata	Putative uncharacterized protein
EOG091204Q8	YQE_09891	Dendroctonus ponderosae	Putative uncharacterized protein
EOG091204Q9	OTAU002046	Onthophagus taurus	Putative uncharacterized protein
EOG091204Q9	AGLA007096	Anoplophora glabripennis	Putative uncharacterized protein
EOG091204Q9	APLA003225	Agrilus planipennis	Putative uncharacterized protein
EOG091204Q9	TC004316	Tribolium castaneum	Putative uncharacterized protein
EOG091204Q9	LDEC008801	Leptinotarsa decemlineata	Putative uncharacterized protein
EOG091204Q9	YQE_02160	Dendroctonus ponderosae	Putative uncharacterized protein
EOG091204QI	OTAU009478	Onthophagus taurus	Putative uncharacterized protein
EOG091204QI	AGLA013474	Anoplophora glabripennis	Putative uncharacterized protein
EOG091204QI	APLA010033	Agrilus planipennis	Putative uncharacterized protein
EOG091204QI	TC015176	Tribolium castaneum	Putative uncharacterized protein
EOG091204QI	LDEC007939	Leptinotarsa decemlineata	Putative uncharacterized protein
EOG091204QI	YQE_09386	Dendroctonus ponderosae	Putative uncharacterized protein
EOG091204QJ	OTAU003942	Onthophagus taurus	Putative uncharacterized protein
EOG091204QJ	AGLA021097	Anoplophora glabripennis	Putative uncharacterized protein
EOG091204QJ	APLA010515	Agrilus planipennis	Putative uncharacterized protein
EOG091204QJ	TC011971	Tribolium castaneum	Putative uncharacterized protein
EOG091204QJ	LDEC000524	Leptinotarsa decemlineata	Putative uncharacterized protein
EOG091204QJ	YQE_07973	Dendroctonus ponderosae	Putative uncharacterized protein
EOG091204QK	OTAU013621	Onthophagus taurus	Peptidyl-prolyl cis-trans isomerase
EOG091204QK	AGLA018141	Anoplophora glabripennis	Peptidyl-prolyl cis-trans isomerase

EOG091204QK	APLA004583	Agrilus planipennis	Peptidyl-prolyl cis-trans isomerase
EOG091204QK	TC009394	Tribolium castaneum	Peptidyl-prolyl cis-trans isomerase
EOG091204QK	LDEC020301	Leptinotarsa decemlineata	Peptidyl-prolyl cis-trans isomerase
EOG091204QK	YQE_13036	Dendroctonus ponderosae	Peptidyl-prolyl cis-trans isomerase
EOG091204QN	OTAU000757	Onthophagus taurus	metal ion binding
EOG091204QN	AGLA000584	Anoplophora glabripennis	metal ion binding
EOG091204QN	APLA007032	Agrilus planipennis	metal ion binding
EOG091204QN	TC032606	Tribolium castaneum	metal ion binding
EOG091204QN	LDEC008394	Leptinotarsa decemlineata	metal ion binding
EOG091204QN	YQE_12304	Dendroctonus ponderosae	metal ion binding
EOG091204QO	OTAU005562	Onthophagus taurus	Putative uncharacterized protein
EOG091204QO	AGLA004874	Anoplophora glabripennis	Putative uncharacterized protein
EOG091204QO	APLA000622	Agrilus planipennis	Putative uncharacterized protein
EOG091204QO	TC015490	Tribolium castaneum	Putative uncharacterized protein
EOG091204QO	LDEC005375	Leptinotarsa decemlineata	Putative uncharacterized protein
EOG091204QO	YQE_01731	Dendroctonus ponderosae	Putative uncharacterized protein
EOG091204QQ	OTAU009248	Onthophagus taurus	Putative uncharacterized protein
EOG091204QQ	AGLA002635	Anoplophora glabripennis	Putative uncharacterized protein
EOG091204QQ	APLA004850	Agrilus planipennis	Putative uncharacterized protein
EOG091204QQ	TC013187	Tribolium castaneum	Putative uncharacterized protein
EOG091204QQ	LDEC015029	Leptinotarsa decemlineata	Putative uncharacterized protein
EOG091204QQ	YQE_10346	Dendroctonus ponderosae	Putative uncharacterized protein
EOG091204QS	OTAU000308	Onthophagus taurus	Putative uncharacterized protein
EOG091204QS	AGLA012604	Anoplophora glabripennis	Putative uncharacterized protein
EOG091204QS	APLA005128	Agrilus planipennis	Putative uncharacterized protein
EOG091204QS	TC014254	Tribolium castaneum	Putative uncharacterized protein
EOG091204QS	LDEC011396	Leptinotarsa decemlineata	Putative uncharacterized protein
EOG091204QS	YQE_06227	Dendroctonus ponderosae	Putative uncharacterized protein
EOG091204QT	OTAU008223	Onthophagus taurus	None
EOG091204QT	AGLA002557	Anoplophora glabripennis	None
EOG091204QT	APLA008989	Agrilus planipennis	None
EOG091204QT	TC032220	Tribolium castaneum	None
EOG091204QT	LDEC001759	Leptinotarsa decemlineata	None
EOG091204QT	YQE_04224	Dendroctonus ponderosae	None
EOG091204QU	OTAU008619	Onthophagus taurus	ATP-dependent (S)-NAD(P)H-hydrate dehydratase
EOG091204QU	AGLA010040	Anoplophora glabripennis	ATP-dependent (S)-NAD(P)H-hydrate dehydratase
EOG091204QU	APLA007456	Agrilus planipennis	ATP-dependent (S)-NAD(P)H-hydrate dehydratase
EOG091204QU	TC008339	Tribolium castaneum	ATP-dependent (S)-NAD(P)H-hydrate dehydratase
EOG091204QU	LDEC005089	Leptinotarsa decemlineata	ATP-dependent (S)-NAD(P)H-hydrate dehydratase
EOG091204QU	YQE_05854	Dendroctonus ponderosae	ATP-dependent (S)-NAD(P)H-hydrate dehydratase
EOG091204R0	OTAU009517	Onthophagus taurus	Putative uncharacterized protein
EOG091204R0	AGLA012285	Anoplophora glabripennis	Putative uncharacterized protein
EOG091204R0	APLA006006	Agrilus planipennis	Putative uncharacterized protein
EOG091204R0	TC009385	Tribolium castaneum	Putative uncharacterized protein
EOG091204R0	LDEC004073	Leptinotarsa decemlineata	Putative uncharacterized protein
EOG091204R0	YQE_05288	Dendroctonus ponderosae	Putative uncharacterized protein
EOG091204R4	OTAU008599	Onthophagus taurus	Putative uncharacterized protein
EOG091204R4	AGLA000770	Anoplophora glabripennis	Putative uncharacterized protein
EOG091204R4	APLA009230	Agrilus planipennis	Putative uncharacterized protein
EOG091204R4	TC008282	Tribolium castaneum	Putative uncharacterized protein
EOG091204R4	LDEC000614	Leptinotarsa decemlineata	Putative uncharacterized protein
EOG091204R4	YQE_02557	Dendroctonus ponderosae	Putative uncharacterized protein
EOG091204R7	OTAU001589	Onthophagus taurus	Putative uncharacterized protein
EOG091204R7	AGLA004975	Anoplophora glabripennis	Putative uncharacterized protein
EOG091204R7	APLA005813	Agrilus planipennis	Putative uncharacterized protein
EOG091204R7	TC006360	Tribolium castaneum	Putative uncharacterized protein
EOG091204R7	LDEC003010	Leptinotarsa decemlineata	Putative uncharacterized protein
EOG091204R7	YQE_07024	Dendroctonus ponderosae	Putative uncharacterized protein
EOG091204R9	OTAU003202	Onthophagus taurus	Putative uncharacterized protein
EOG091204R9	AGLA015868	Anoplophora glabripennis	Putative uncharacterized protein
EOG091204R9	APLA007982	Agrilus planipennis	Putative uncharacterized protein
EOG091204R9	TC013505	Tribolium castaneum	Putative uncharacterized protein
EOG091204R9	LDEC003092	Leptinotarsa decemlineata	Putative uncharacterized protein
EOG091204R9	YQE_07203	Dendroctonus ponderosae	Putative uncharacterized protein
EOG091204RA	OTAU004043	Onthophagus taurus	Putative uncharacterized protein
EOG091204RA	AGLA020662	Anoplophora glabripennis	Putative uncharacterized protein
EOG091204RA	APLA008125	Agrilus planipennis	Putative uncharacterized protein

EOG091204RA	TC010333	Tribolium castaneum	Putative uncharacterized protein
EOG091204RA	LDEC007090	Leptinotarsa decemlineata	Putative uncharacterized protein
EOG091204RA	YQE_08376	Dendroctonus ponderosae	Putative uncharacterized protein
EOG091204RB	OTAU013378	Onthophagus taurus	Putative uncharacterized protein
EOG091204RB	AGLA010969	Anoplophora glabripennis	Putative uncharacterized protein
EOG091204RB	APLA003608	Agrilus planipennis	Putative uncharacterized protein
EOG091204RB	TC013872	Tribolium castaneum	Putative uncharacterized protein
EOG091204RB	LDEC008918	Leptinotarsa decemlineata	Putative uncharacterized protein
EOG091204RB	YQE_02696	Dendroctonus ponderosae	Putative uncharacterized protein
EOG091204RD	OTAU012059	Onthophagus taurus	None
EOG091204RD	AGLA001576	Anoplophora glabripennis	None
EOG091204RD	APLA009525	Agrilus planipennis	None
EOG091204RD	TC031939	Tribolium castaneum	None
EOG091204RD	LDEC021754	Leptinotarsa decemlineata	None
EOG091204RD	YQE_11915	Dendroctonus ponderosae	None
EOG091204RM	OTAU013024	Onthophagus taurus	Putative uncharacterized protein
EOG091204RM	AGLA000259	Anoplophora glabripennis	Putative uncharacterized protein
EOG091204RM	APLA012424	Agrilus planipennis	Putative uncharacterized protein
EOG091204RM	TC013652	Tribolium castaneum	Putative uncharacterized protein
EOG091204RM	LDEC017737	Leptinotarsa decemlineata	Putative uncharacterized protein
EOG091204RM	YQE_02504	Dendroctonus ponderosae	Putative uncharacterized protein
EOG091204RN	OTAU006362	Onthophagus taurus	Putative uncharacterized protein
EOG091204RN	AGLA009404	Anoplophora glabripennis	Putative uncharacterized protein
EOG091204RN	APLA010106	Agrilus planipennis	Putative uncharacterized protein
EOG091204RN	TC013254	Tribolium castaneum	Putative uncharacterized protein
EOG091204RN	LDEC011537	Leptinotarsa decemlineata	Putative uncharacterized protein
EOG091204RN	YQE_08766	Dendroctonus ponderosae	Putative uncharacterized protein
EOG091204RO	OTAU009881	Onthophagus taurus	Putative uncharacterized protein
EOG091204RO	AGLA000144	Anoplophora glabripennis	Putative uncharacterized protein
EOG091204RO	APLA008440	Agrilus planipennis	Putative uncharacterized protein
EOG091204RO	TC001379	Tribolium castaneum	Putative uncharacterized protein
EOG091204RO	LDEC016691	Leptinotarsa decemlineata	Putative uncharacterized protein
EOG091204RO	YQE_07057	Dendroctonus ponderosae	Putative uncharacterized protein
EOG091204RP	OTAU005013	Onthophagus taurus	Putative uncharacterized protein
EOG091204RP	AGLA013345	Anoplophora glabripennis	Putative uncharacterized protein
EOG091204RP	APLA002193	Agrilus planipennis	Putative uncharacterized protein
EOG091204RP	TC007443	Tribolium castaneum	Putative uncharacterized protein
EOG091204RP	LDEC018602	Leptinotarsa decemlineata	Putative uncharacterized protein
EOG091204RP	YQE_07044	Dendroctonus ponderosae	Putative uncharacterized protein
EOG091204RQ	OTAU009553	Onthophagus taurus	Hyaluronidase
EOG091204RQ	AGLA020430	Anoplophora glabripennis	Hyaluronidase
EOG091204RQ	APLA009426	Agrilus planipennis	Hyaluronidase
EOG091204RQ	TC015488	Tribolium castaneum	Hyaluronidase
EOG091204RQ	LDEC013356	Leptinotarsa decemlineata	Hyaluronidase
EOG091204RQ	YQE_05875	Dendroctonus ponderosae	Hyaluronidase
EOG091204RR	OTAU016924	Onthophagus taurus	None
EOG091204RR	AGLA006945	Anoplophora glabripennis	None
EOG091204RR	APLA000257	Agrilus planipennis	None
EOG091204RR	TC032549	Tribolium castaneum	None
EOG091204RR	LDEC018263	Leptinotarsa decemlineata	None
EOG091204RR	YQE_03817	Dendroctonus ponderosae	None
EOG091204RW	OTAU006278	Onthophagus taurus	Putative uncharacterized protein
EOG091204RW	AGLA003764	Anoplophora glabripennis	Putative uncharacterized protein
EOG091204RW	APLA012541	Agrilus planipennis	Putative uncharacterized protein
EOG091204RW	TC007212	Tribolium castaneum	Putative uncharacterized protein
EOG091204RW	LDEC020055	Leptinotarsa decemlineata	Putative uncharacterized protein
EOG091204RW	YQE_06335	Dendroctonus ponderosae	Putative uncharacterized protein
EOG091204RX	OTAU004422	Onthophagus taurus	Putative uncharacterized protein
EOG091204RX	AGLA000739	Anoplophora glabripennis	Putative uncharacterized protein
EOG091204RX	APLA006559	Agrilus planipennis	Putative uncharacterized protein
EOG091204RX	TC007359	Tribolium castaneum	Putative uncharacterized protein
EOG091204RX	LDEC005098	Leptinotarsa decemlineata	Putative uncharacterized protein
EOG091204RX	YQE_11445	Dendroctonus ponderosae	Putative uncharacterized protein
EOG091204S0	OTAU009373	Onthophagus taurus	Putative uncharacterized protein
EOG091204S0	AGLA015388	Anoplophora glabripennis	Putative uncharacterized protein
EOG091204S0	APLA009812	Agrilus planipennis	Putative uncharacterized protein
EOG091204S0	TC008966	Tribolium castaneum	Putative uncharacterized protein

EOG091204S0	LDEC022016	Leptinotarsa decemlineata	Putative uncharacterized protein
EOG091204S0	YQE_11552	Dendroctonus ponderosae	Putative uncharacterized protein
EOG091204S1	OTAU009837	Onthophagus taurus	Methionine aminopeptidase
EOG091204S1	AGLA012662	Anoplophora glabripennis	Methionine aminopeptidase
EOG091204S1	APLA008803	Agrilus planipennis	Methionine aminopeptidase
EOG091204S1	TC033550	Tribolium castaneum	Methionine aminopeptidase
EOG091204S1	LDEC011010	Leptinotarsa decemlineata	Methionine aminopeptidase
EOG091204S1	YQE_05385	Dendroctonus ponderosae	Methionine aminopeptidase
EOG091204S9	OTAU010567	Onthophagus taurus	Putative uncharacterized protein
EOG091204S9	AGLA001305	Anoplophora glabripennis	Putative uncharacterized protein
EOG091204S9	APLA005268	Agrilus planipennis	Putative uncharacterized protein
EOG091204S9	TC004931	Tribolium castaneum	Putative uncharacterized protein
EOG091204S9	LDEC009641	Leptinotarsa decemlineata	Putative uncharacterized protein
EOG091204S9	YQE_10747	Dendroctonus ponderosae	Putative uncharacterized protein
EOG091204SB	OTAU012225	Onthophagus taurus	Putative uncharacterized protein
EOG091204SB	AGLA008769	Anoplophora glabripennis	Putative uncharacterized protein
EOG091204SB	APLA008442	Agrilus planipennis	Putative uncharacterized protein
EOG091204SB	TC011475	Tribolium castaneum	Putative uncharacterized protein
EOG091204SB	LDEC019736	Leptinotarsa decemlineata	Putative uncharacterized protein
EOG091204SB	YQE_08094	Dendroctonus ponderosae	Putative uncharacterized protein
EOG091204SC	OTAU014007	Onthophagus taurus	Putative uncharacterized protein
EOG091204SC	AGLA002623	Anoplophora glabripennis	Putative uncharacterized protein
EOG091204SC	APLA010395	Agrilus planipennis	Putative uncharacterized protein
EOG091204SC	TC012466	Tribolium castaneum	Putative uncharacterized protein
EOG091204SC	LDEC001999	Leptinotarsa decemlineata	Putative uncharacterized protein
EOG091204SC	YQE_03158	Dendroctonus ponderosae	Putative uncharacterized protein
EOG091204SD	OTAU006825	Onthophagus taurus	Putative uncharacterized protein
EOG091204SD	AGLA008582	Anoplophora glabripennis	Putative uncharacterized protein
EOG091204SD	APLA007326	Agrilus planipennis	Putative uncharacterized protein
EOG091204SD	TC010521	Tribolium castaneum	Putative uncharacterized protein
EOG091204SD	LDEC002682	Leptinotarsa decemlineata	Putative uncharacterized protein
EOG091204SD	YQE_04548	Dendroctonus ponderosae	Putative uncharacterized protein
EOG091204SH	OTAU016045	Onthophagus taurus	Putative uncharacterized protein
EOG091204SH	AGLA006552	Anoplophora glabripennis	Putative uncharacterized protein
EOG091204SH	APLA004329	Agrilus planipennis	Putative uncharacterized protein
EOG091204SH	TC007954	Tribolium castaneum	Putative uncharacterized protein
EOG091204SH	LDEC001648	Leptinotarsa decemlineata	Putative uncharacterized protein
EOG091204SH	YQE_09478	Dendroctonus ponderosae	Putative uncharacterized protein
EOG091204SI	OTAU014961	Onthophagus taurus	Putative uncharacterized protein
EOG091204SI	AGLA013739	Anoplophora glabripennis	Putative uncharacterized protein
EOG091204SI	APLA000864	Agrilus planipennis	Putative uncharacterized protein
EOG091204SI	TC012554	Tribolium castaneum	Putative uncharacterized protein
EOG091204SI	LDEC003925	Leptinotarsa decemlineata	Putative uncharacterized protein
EOG091204SI	YQE_07773	Dendroctonus ponderosae	Putative uncharacterized protein
EOG091204SM	OTAU008140	Onthophagus taurus	Putative uncharacterized protein
EOG091204SM	AGLA021458	Anoplophora glabripennis	Putative uncharacterized protein
EOG091204SM	APLA005283	Agrilus planipennis	Putative uncharacterized protein
EOG091204SM	TC011556	Tribolium castaneum	Putative uncharacterized protein
EOG091204SM	LDEC010346	Leptinotarsa decemlineata	Putative uncharacterized protein
EOG091204SM	YQE_11218	Dendroctonus ponderosae	Putative uncharacterized protein
EOG091204SO	OTAU012930	Onthophagus taurus	Putative uncharacterized protein
EOG091204SO	AGLA011049	Anoplophora glabripennis	Putative uncharacterized protein
EOG091204SO	APLA012838	Agrilus planipennis	Putative uncharacterized protein
EOG091204SO	TC006318	Tribolium castaneum	Putative uncharacterized protein
EOG091204SO	LDEC009301	Leptinotarsa decemlineata	Putative uncharacterized protein
EOG091204SO	YQE_06922	Dendroctonus ponderosae	Putative uncharacterized protein
EOG091204SR	OTAU006132	Onthophagus taurus	Putative uncharacterized protein
EOG091204SR	AGLA001566	Anoplophora glabripennis	Putative uncharacterized protein
EOG091204SR	APLA009477	Agrilus planipennis	Putative uncharacterized protein
EOG091204SR	TC000605	Tribolium castaneum	Putative uncharacterized protein
EOG091204SR	LDEC013220	Leptinotarsa decemlineata	Putative uncharacterized protein
EOG091204SR	YQE_08927	Dendroctonus ponderosae	Putative uncharacterized protein
EOG091204SU	OTAU003032	Onthophagus taurus	Putative uncharacterized protein
EOG091204SU	AGLA003656	Anoplophora glabripennis	Putative uncharacterized protein
EOG091204SU	APLA014804	Agrilus planipennis	Putative uncharacterized protein
EOG091204SU	TC006987	Tribolium castaneum	Putative uncharacterized protein
EOG091204SU	LDEC005328	Leptinotarsa decemlineata	Putative uncharacterized protein

EOG091204SU	YQE_04862	Dendroctonus ponderosae	Putative uncharacterized protein
EOG091204SY	OTAU007181	Onthophagus taurus	Putative uncharacterized protein
EOG091204SY	AGLA013823	Anoplophora glabripennis	Putative uncharacterized protein
EOG091204SY	APLA009428	Agrilus planipennis	Putative uncharacterized protein
EOG091204SY	TC015129	Tribolium castaneum	Putative uncharacterized protein
EOG091204SY	LDEC001805	Leptinotarsa decemlineata	Putative uncharacterized protein
EOG091204SY	YQE_10844	Dendroctonus ponderosae	Putative uncharacterized protein
EOG091204T2	OTAU002127	Onthophagus taurus	Putative uncharacterized protein
EOG091204T2	AGLA015367	Anoplophora glabripennis	Putative uncharacterized protein
EOG091204T2	APLA013099	Agrilus planipennis	Putative uncharacterized protein
EOG091204T2	TC011744	Tribolium castaneum	Putative uncharacterized protein
EOG091204T2	LDEC018805	Leptinotarsa decemlineata	Putative uncharacterized protein
EOG091204T2	YQE_10100	Dendroctonus ponderosae	Putative uncharacterized protein
EOG091204T3	OTAU008118	Onthophagus taurus	Putative uncharacterized protein
EOG091204T3	AGLA018377	Anoplophora glabripennis	Putative uncharacterized protein
EOG091204T3	APLA007897	Agrilus planipennis	Putative uncharacterized protein
EOG091204T3	TC000477	Tribolium castaneum	Putative uncharacterized protein
EOG091204T3	LDEC014068	Leptinotarsa decemlineata	Putative uncharacterized protein
EOG091204T3	YQE_10137	Dendroctonus ponderosae	Putative uncharacterized protein
EOG091204T6	OTAU011895	Onthophagus taurus	Putative uncharacterized protein
EOG091204T6	AGLA012834	Anoplophora glabripennis	Putative uncharacterized protein
EOG091204T6	APLA002694	Agrilus planipennis	Putative uncharacterized protein
EOG091204T6	TC012993	Tribolium castaneum	Putative uncharacterized protein
EOG091204T6	LDEC005944	Leptinotarsa decemlineata	Putative uncharacterized protein
EOG091204T6	YQE_06222	Dendroctonus ponderosae	Putative uncharacterized protein
EOG091204TE	OTAU011034	Onthophagus taurus	Putative uncharacterized protein
EOG091204TE	AGLA007070	Anoplophora glabripennis	Putative uncharacterized protein
EOG091204TE	APLA009881	Agrilus planipennis	Putative uncharacterized protein
EOG091204TE	TC000830	Tribolium castaneum	Putative uncharacterized protein
EOG091204TE	LDEC002554	Leptinotarsa decemlineata	Putative uncharacterized protein
EOG091204TE	YQE_02410	Dendroctonus ponderosae	Putative uncharacterized protein
EOG091204TF	OTAU004278	Onthophagus taurus	None
EOG091204TF	AGLA009058	Anoplophora glabripennis	None
EOG091204TF	APLA001022	Agrilus planipennis	None
EOG091204TF	TC034595	Tribolium castaneum	None
EOG091204TF	LDEC017727	Leptinotarsa decemlineata	None
EOG091204TF	YQE_04966	Dendroctonus ponderosae	None
EOG091204TQ	OTAU009569	Onthophagus taurus	Putative uncharacterized protein
EOG091204TQ	AGLA009049	Anoplophora glabripennis	Putative uncharacterized protein
EOG091204TQ	APLA009049	Agrilus planipennis	Putative uncharacterized protein
EOG091204TQ	TC007228	Tribolium castaneum	Putative uncharacterized protein
EOG091204TQ	LDEC000156	Leptinotarsa decemlineata	Putative uncharacterized protein
EOG091204TQ	YQE_09674	Dendroctonus ponderosae	Putative uncharacterized protein
EOG091204TS	OTAU000431	Onthophagus taurus	Putative uncharacterized protein
EOG091204TS	AGLA012359	Anoplophora glabripennis	Putative uncharacterized protein
EOG091204TS	APLA001039	Agrilus planipennis	Putative uncharacterized protein
EOG091204TS	TC012677	Tribolium castaneum	Putative uncharacterized protein
EOG091204TS	LDEC000572	Leptinotarsa decemlineata	Putative uncharacterized protein
EOG091204TS	YQE_12903	Dendroctonus ponderosae	Putative uncharacterized protein
EOG091204TV	OTAU003122	Onthophagus taurus	Putative uncharacterized protein
EOG091204TV	AGLA015232	Anoplophora glabripennis	Putative uncharacterized protein
EOG091204TV	APLA008215	Agrilus planipennis	Putative uncharacterized protein
EOG091204TV	TC000127	Tribolium castaneum	Putative uncharacterized protein
EOG091204TV	LDEC005011	Leptinotarsa decemlineata	Putative uncharacterized protein
EOG091204TV	YQE_05132	Dendroctonus ponderosae	Putative uncharacterized protein
EOG091204TW	OTAU007501	Onthophagus taurus	Proteasome subunit beta type
EOG091204TW	AGLA019227	Anoplophora glabripennis	Proteasome subunit beta type
EOG091204TW	APLA015189	Agrilus planipennis	Proteasome subunit beta type
EOG091204TW	TC015205	Tribolium castaneum	Proteasome subunit beta type
EOG091204TW	LDEC012245	Leptinotarsa decemlineata	Proteasome subunit beta type
EOG091204TW	YQE_02114	Dendroctonus ponderosae	Proteasome subunit beta type
EOG091204TX	OTAU002922	Onthophagus taurus	Putative uncharacterized protein
EOG091204TX	AGLA018239	Anoplophora glabripennis	Putative uncharacterized protein
EOG091204TX	APLA014528	Agrilus planipennis	Putative uncharacterized protein
EOG091204TX	TC009501	Tribolium castaneum	Putative uncharacterized protein
EOG091204TX	LDEC011021	Leptinotarsa decemlineata	Putative uncharacterized protein
EOG091204TX	YQE_05372	Dendroctonus ponderosae	Putative uncharacterized protein

EOG091204U1	OTAU006215	Onthophagus taurus	Putative uncharacterized protein
EOG091204U1	AGLA020052	Anoplophora glabripennis	Putative uncharacterized protein
EOG091204U1	APLA002096	Agrilus planipennis	Putative uncharacterized protein
EOG091204U1	TC010483	Tribolium castaneum	Putative uncharacterized protein
EOG091204U1	LDEC004025	Leptinotarsa decemlineata	Putative uncharacterized protein
EOG091204U1	YQE_06439	Dendroctonus ponderosae	Putative uncharacterized protein
EOG091204U2	OTAU006625	Onthophagus taurus	Nbs
EOG091204U2	AGLA004207	Anoplophora glabripennis	Nbs
EOG091204U2	APLA014443	Agrilus planipennis	Nbs
EOG091204U2	TC013036	Tribolium castaneum	Nbs
EOG091204U2	LDEC013801	Leptinotarsa decemlineata	Nbs
EOG091204U2	YQE_07208	Dendroctonus ponderosae	Nbs
EOG091204U3	OTAU003474	Onthophagus taurus	Putative uncharacterized protein
EOG091204U3	AGLA013129	Anoplophora glabripennis	Putative uncharacterized protein
EOG091204U3	APLA001597	Agrilus planipennis	Putative uncharacterized protein
EOG091204U3	TC008799	Tribolium castaneum	Putative uncharacterized protein
EOG091204U3	LDEC010674	Leptinotarsa decemlineata	Putative uncharacterized protein
EOG091204U3	YQE_02333	Dendroctonus ponderosae	Putative uncharacterized protein
EOG091204U4	OTAU008883	Onthophagus taurus	Putative uncharacterized protein
EOG091204U4	AGLA006558	Anoplophora glabripennis	Putative uncharacterized protein
EOG091204U4	APLA009516	Agrilus planipennis	Putative uncharacterized protein
EOG091204U4	TC000379	Tribolium castaneum	Putative uncharacterized protein
EOG091204U4	LDEC021172	Leptinotarsa decemlineata	Putative uncharacterized protein
EOG091204U4	YQE_10947	Dendroctonus ponderosae	Putative uncharacterized protein
EOG091204UA	OTAU007053	Onthophagus taurus	Putative uncharacterized protein
EOG091204UA	AGLA000617	Anoplophora glabripennis	Putative uncharacterized protein
EOG091204UA	APLA014194	Agrilus planipennis	Putative uncharacterized protein
EOG091204UA	TC001577	Tribolium castaneum	Putative uncharacterized protein
EOG091204UA	LDEC005482	Leptinotarsa decemlineata	Putative uncharacterized protein
EOG091204UA	YQE_06420	Dendroctonus ponderosae	Putative uncharacterized protein
EOG091204UB	OTAU003181	Onthophagus taurus	None
EOG091204UB	AGLA012332	Anoplophora glabripennis	None
EOG091204UB	APLA011715	Agrilus planipennis	None
EOG091204UB	TC033128	Tribolium castaneum	None
EOG091204UB	LDEC021658	Leptinotarsa decemlineata	None
EOG091204UB	YQE_08623	Dendroctonus ponderosae	None
EOG091204UC	OTAU000066	Onthophagus taurus	Putative uncharacterized protein
EOG091204UC	AGLA004002	Anoplophora glabripennis	Putative uncharacterized protein
EOG091204UC	APLA010799	Agrilus planipennis	Putative uncharacterized protein
EOG091204UC	TC013839	Tribolium castaneum	Putative uncharacterized protein
EOG091204UC	LDEC020882	Leptinotarsa decemlineata	Putative uncharacterized protein
EOG091204UC	YQE_06304	Dendroctonus ponderosae	Putative uncharacterized protein
EOG091204UE	OTAU002899	Onthophagus taurus	Nicotinic acetylcholine receptor a9 subunit
EOG091204UE	AGLA006522	Anoplophora glabripennis	Nicotinic acetylcholine receptor a9 subunit
EOG091204UE	APLA007165	Agrilus planipennis	Nicotinic acetylcholine receptor a9 subunit
EOG091204UE	TC009170	Tribolium castaneum	Nicotinic acetylcholine receptor a9 subunit
EOG091204UE	LDEC016302	Leptinotarsa decemlineata	Nicotinic acetylcholine receptor a9 subunit
EOG091204UE	YQE_05258	Dendroctonus ponderosae	Nicotinic acetylcholine receptor a9 subunit
EOG091204UF	OTAU001261	Onthophagus taurus	Brain peptide IDL-like protein
EOG091204UF	AGLA000266	Anoplophora glabripennis	Brain peptide IDL-like protein
EOG091204UF	APLA010674	Agrilus planipennis	Brain peptide IDL-like protein
EOG091204UF	TC004766	Tribolium castaneum	Brain peptide IDL-like protein
EOG091204UF	LDEC009918	Leptinotarsa decemlineata	Brain peptide IDL-like protein
EOG091204UF	YQE_02491	Dendroctonus ponderosae	Brain peptide IDL-like protein
EOG091204UG	OTAU015984	Onthophagus taurus	Putative uncharacterized protein
EOG091204UG	AGLA001386	Anoplophora glabripennis	Putative uncharacterized protein
EOG091204UG	APLA007944	Agrilus planipennis	Putative uncharacterized protein
EOG091204UG	TC000467	Tribolium castaneum	Putative uncharacterized protein
EOG091204UG	LDEC007313	Leptinotarsa decemlineata	Putative uncharacterized protein
EOG091204UG	YQE_11654	Dendroctonus ponderosae	Putative uncharacterized protein
EOG091204UO	OTAU010668	Onthophagus taurus	Putative uncharacterized protein
EOG091204UO	AGLA021760	Anoplophora glabripennis	Putative uncharacterized protein
EOG091204UO	APLA014838	Agrilus planipennis	Putative uncharacterized protein
EOG091204UO	TC009595	Tribolium castaneum	Putative uncharacterized protein
EOG091204UO	LDEC004824	Leptinotarsa decemlineata	Putative uncharacterized protein
EOG091204UO	YQE_07642	Dendroctonus ponderosae	Putative uncharacterized protein
EOG091204UT	OTAU013821	Onthophagus taurus	Putative uncharacterized protein

EOG091204UT	AGLA016654	Anoplophora glabripennis	Putative uncharacterized protein
EOG091204UT	APLA006658	Agrilus planipennis	Putative uncharacterized protein
EOG091204UT	TC003331	Tribolium castaneum	Putative uncharacterized protein
[truncated: 872,698 more chars]
